# A unified strategy for α-alkoxyalkyl radical generation *via* pinacol boronic ester precursors

**DOI:** 10.1039/d6qo01025c

**Published:** 2026-09-15

**Authors:** Emy André-Joyaux, Lise Benoist, Agathe Fayolle, Fabrice Dénès, Philippe Renaud

**Affiliations:** a University of Bern, Department of Chemistry, Biochemistry and Pharmaceutical Sciences Freiestrasse 3 CH-3012 Bern Switzerland philippe.renaud@unibe.ch fabrice.denes@univ-nantes.fr; b Nantes Université, CNRS, CEISAM, UMR 6230 F-44000 Nantes France

## Abstract

A general method to generate α-alkoxyalkyl radicals from pinacol boronic ester precursors (R–Bpin) has been developed. These radical precursors are stable in air, easy to prepare, and readily accessible *via* Matteson homologation followed by a 1,2-metallate shift with a broad range of metal alkoxides. The key step is a transesterification with catechol methyl borate (MeOBcat), converting the inert Bpin species into a radical-active Bcat intermediate, which then undergoes nucleohomolytic substitution at boron to release the desired radical. This approach is practical, scalable (demonstrated at 4 mmol scale), and broadly applicable. It avoids toxic reagents and expensive photocatalysts, offering a straightforward route to ether-containing molecules relevant to the synthesis of natural product and bioactive compounds.

## Abstract 2

α-Alkoxyalkyl radicals are generated from easily available and air stable pinacol boronic esters. This approach is practical, scalable, and broadly applicable. It offers a straightforward route to ether-containing molecules.

## Graphical abstract

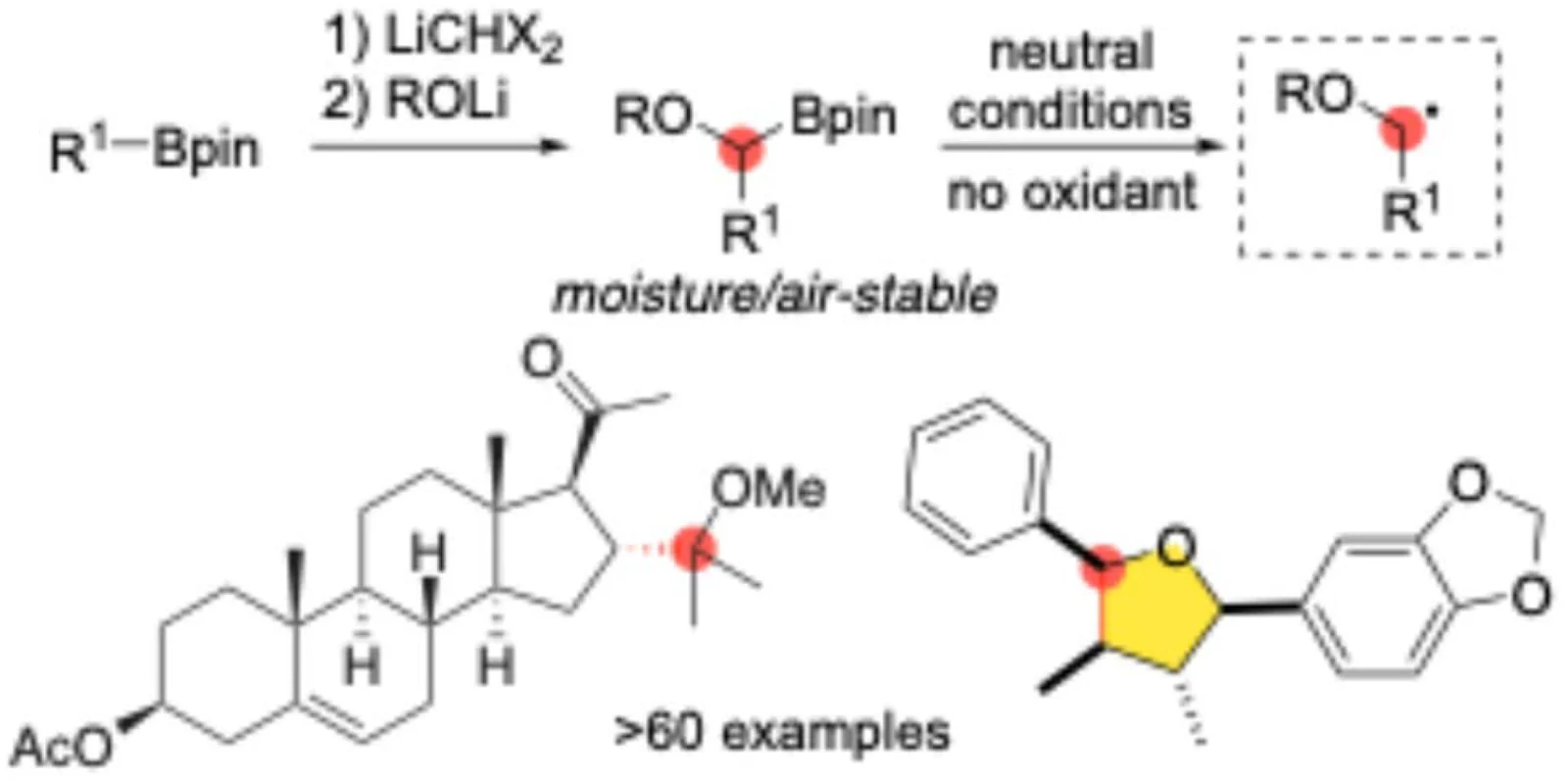

## Introduction

**Fig. 1:**
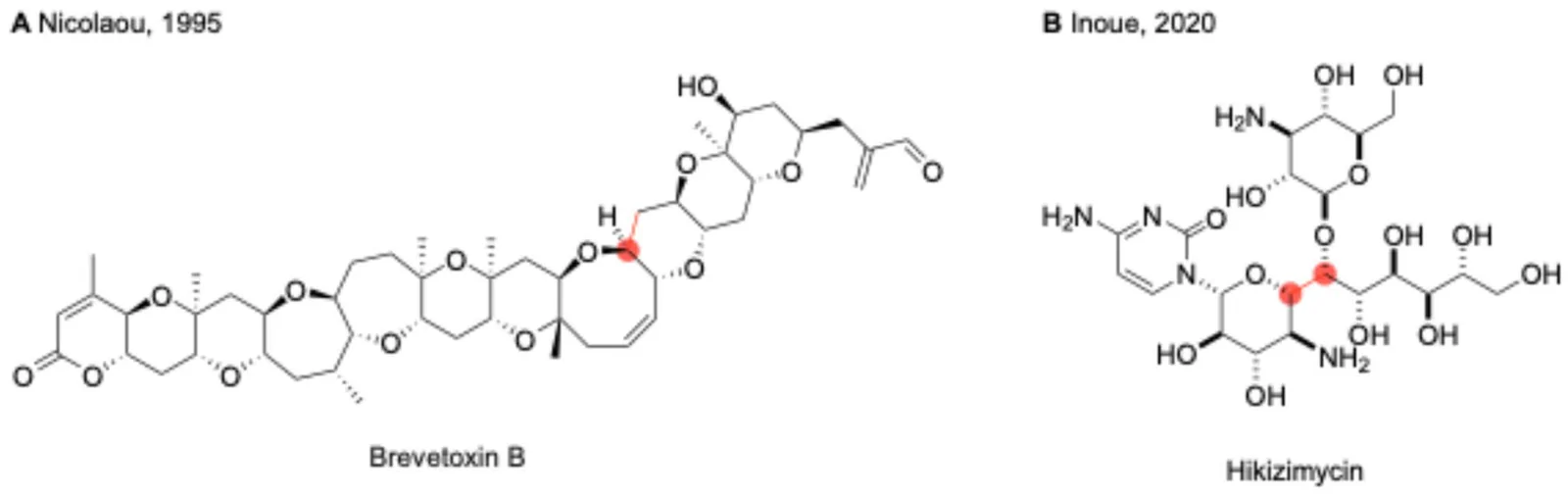
Selected examples of natural products synthetized *via* α-alkoxy radicals (red dot). The bond formed *via* an α-alkoxy radical is highlighted in red.

The dialkyl ether moiety is present in a wide range of biologically active compounds. These include both acyclic and cyclic congeners, which are isolated from natural sources^1–3^ or produced by the pharmaceutical industry.^4^ Dialkyl ethers can be accessed by a variety of synthetic methods,^5,6^ with the well-known Williamson ether synthesis still being the most popular,^7^ but the access to sterically hindered ethers still remains a challenge. As a complementary approach, the generation of α-alkoxy radicals has emerged as a straightforward strategy to access complex ether-containing compounds and has been used for decades in the total synthesis of natural products. For instance, this approach was employed in 1995 by Nicolaou and coworkers for the synthesis of Brevetoxin B (Fig. 1A),^8^ and more recently by Inoue and coworkers for the synthesis of Hikizimycin (Fig. 1B) and other highly oxygenated compounds.^9,10^

**Scheme 1:**
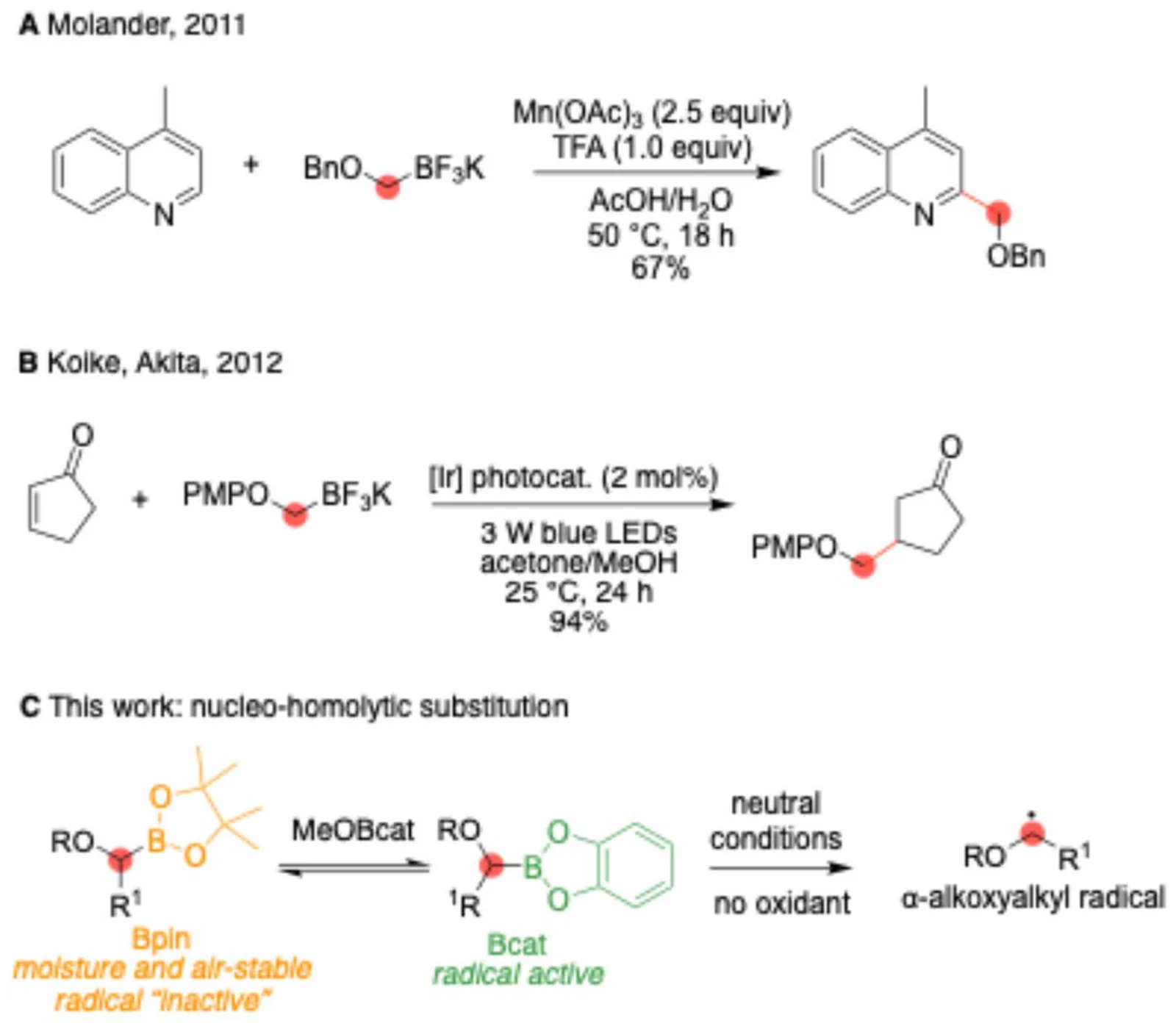
Selected examples of existing methods for the generation of α-alkoxy radicals from boronic acid derivatives.

Numerous methods have been developed over the years for the generation of α-alkoxy radicals, from classical tin-mediated radical chain reaction conditions to promote C–X bond abstraction (X = Br, SPh, SePh, TePh, NO_2_)^11–22^ to direct hydrogen atom transfer (HAT) processes.^23^ Since the pioneering work on the addition of alkyl radicals to enones and related compounds using trialkylboranes as radical precursors,^24,25^ organoboron derivatives have emerged as useful precursors for carbon-centred radicals and these have been employed to achieved a wide range of synthetic transformations.^26–28^ In this context, oxidative methods have been used to produce α-alkoxyalkyl radicals from the corresponding potassium trifluoroborate derivatives.^29^ Molander and coworkers reported the generation of alkoxyalkyl radicals from air- and moisture-stable potassium trifluoroborates using Mn(OAc)_3_ as an oxidant and their use in Minisci reactions (Scheme 1A).^30^ Shortly later, Koike, Akita and co-workers showed that Giese-type addition could be achieved using potassium alkyl- and alkoxyalkyltrifluoroborates or (triol)borates as radical precursors using an iridium-based photocatalyst [Ir(dF(CF_3_)ppy)_2_(bpy)](PF_6_) under irradiation with visible-light (Scheme 1B).^31,32^ Since then, efficient processes based upon similar oxidative conditions have been developed to allow for the preparation of primary and secondary benzylic ethers *via* photoredox-cross-coupling using Ir/Ni dual catalysis.^33–37^ Minisci reactions have also been carried out using potassium α-alkoxyalkyltrifluoroborates in the presence of acridinium photo-catalysts and potassium persulfate as co-oxidant.^38^

Despite the development of various efficient strategies for generating α-alkoxyalkyl radicals, including those described in Scheme 1, each method has its inherent limitations, such as reagent toxicity, the site-selectivity in HAT reactions involving non-symmetrical dialkyl ethers, the use of costly, rare-metal based catalysts, or the need for stoichiometric oxidants. Our group developed efficient chain reactions based on the use of *B*-alkylcatecholboranes (R–Bcat) as alkyl radical precursors using a chain reaction involving a nucleohomolytic substitution process.^39–43^ These air-sensitive radical precursors can be easily obtained *in situ* by hydroboration of alkenes with catecholborane or, alternatively, from the corresponding isolable, air-stable pinacol boronic esters (R–Bpin) using an acid-catalysed boron-transesterification with 4-*tert*-butylcatechol (TBC). Efficient radical protodeboronation could be achieved following the second strategy,^44^ and further developments with sub-stoichiometric amounts of catechol methyl borate (MeOBcat) to perform the boron-transesterification allowed for efficient C–C and C–heteroatom bonds formation.^45^ Our long-standing interest for the generation of carbon-centered radicals from organoboron species prompted us to investigate the generation of α-alkoxyalkyl radicals. Herein, we report a general protocol for the generation of α-alkoxyalkyl radicals starting from isolable, air-stable, and readily available α-alkoxyalkyl pinacol boronic ester precursors. The attractiveness of the method relies in the easy preparation of the desired α-alkoxyalkylBpin precursors from readily available alkyl pinacol boronic esters *via* a robust sequence involving Matteson homologation and subsequent 1,2-metallate shift with diverse metal alkoxides (Scheme 1C).^46–49^ The chain reaction proceeds under neutral conditions, involves an efficient nucleohomolytic substitution reaction and does not require the use of oxidative conditions.

## Results

### Addition to enones

**Scheme 2:**
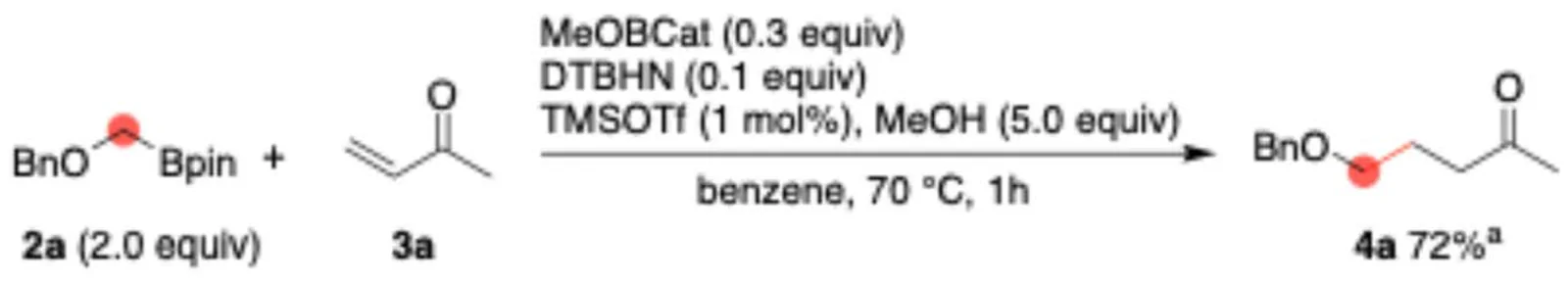
Initial reaction conditions for the addition of 2a to metylvinylketone. ^*a*^Yield determined by NMR using 1,4-dimethoxybenzene an internal standard.

Considering the nucleophilic character of the α-alkoxyalkyl radicals, we decided to explore their generation from α-alkoxyalkylBpin precursors 2 in the presence of electron deficient alkenes **3** (see SI, Scheme S1 for the structure of the precursors). The reaction was tested first with 2a and methylvinylketone 3a using reaction conditions previously developed for the generation of alkyl radicals from R–Bpin precursors, namely di-*tert*-butylhyponitrite (DTBHN) as a radical initiator, MeOBcat (0.3 equiv.) to achieve the boronic ester transesterification and a catalytic amount of triflic acid generated from TMSOTf and MeOH. The reaction was run in benzene at 70 °C. Under these conditions, the desired addition product 4a was obtained in 72% yield (Scheme 2).

**Scheme 3:**
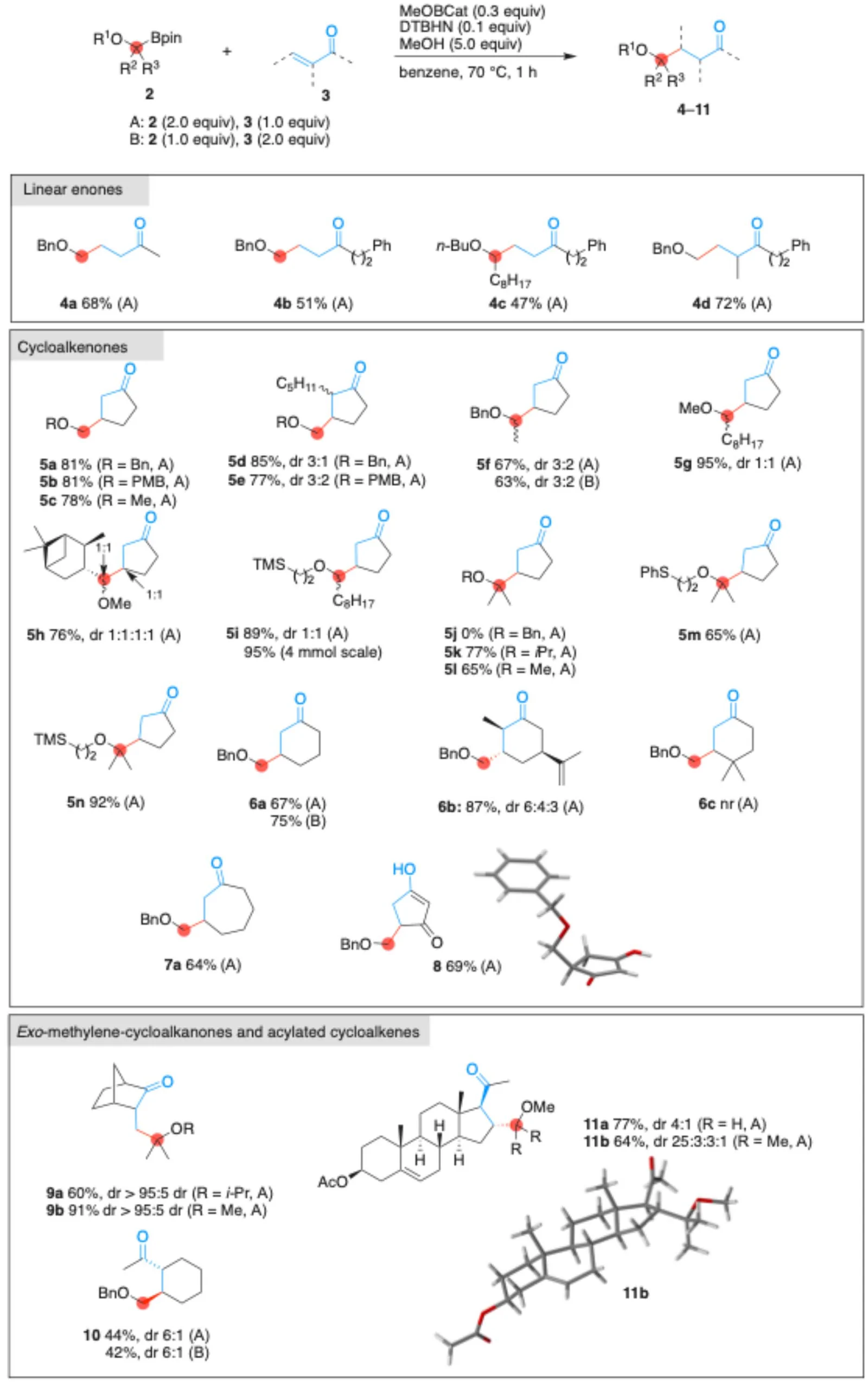
Hydro-α-alkoxyalkylation of enones (yields of isolated products).

A rapid screening of the reaction conditions (see SI for details) showed that, unlike simple alkyl-Bpin precursors, α-alkoxyalkyl analogues efficiently underwent boron-transesterification without the need for an acid catalysis. The isolated yields for the preparation of 4a were very similar with or without MeOH. Based on a previous observation showing that accumulation of boron enolates could lead to side reactions,^50^ all reactions were performed using systematically five equivalents of MeOH to protonate the boron enolate intermediate. Two complementary sets of reaction conditions were then developed using either Bpin precursor (conditions A) or enone (conditions B) as limiting reagent. The scope of the reaction was investigated on a series of representative enones (Scheme 3). Under the optimized reaction conditions, the addition of a two-fold excess of radical precursor 2a (conditions A) to methylvinylketone 3a, a radical trap very sensitive to polymerization, afforded the desired adduct 4a in 68% isolated yield. In the addition to 5-phenylpent-1-en-3-one 3b, primary and secondary alkoxyalkyl radical precursors 2a and 2h afforded the desired adducts 4b and 4c in 51% and 47% yields, respectively. The addition of 2a to 2-methyl-5-phenylpent-1-en-3-one 3c gave 4d in 72% yield. The addition to cycloalkenones was investigated next. The addition of primary α-alkoxyalkyl radicals derived from 2a–c to cyclopent-2-enone 3d delivered the expected adducts 5a–c in high yields (78–81%) using an excess of R–Bpin precursor derivative (conditions A). The addition of primary α-alkoxyalkyl radicals 2a and 2b to 2-*n*-pentylcyclopent-2-enone 3e also proceeded in high yields, delivering 5d and 5e in 85% and 77% yields, respectively. With precursors of secondary α-alkoxyalkyl radicals, the choice of the substituent on the oxygen atom proved to have an influence on the yield of the reaction, with the *O*-benzyl protected precursor giving the lowest yields. For instance, while the use of *O*-benzylated precursor 2e in the addition to cyclopentenone 3d led to adduct 5f in only 67% yield (conditions A) or 63% yield (conditions B, excess of the radical trap), significantly higher yields (76–95%) were obtained for the preparation of 5g–i with α-alkoxyalkyl radical precursors 2f, 2g and 2i presenting an alkyl substituent on the oxygen atom. The Bpin precursor 2g used for the preparation of **5h** was obtained *via* a sequence hydroboration/Matteson homologation, illustrating the versatility of the approach to access precursors of elaborated α-alkoxyalkyl radicals. Worthy of note is the use of an *O*-trimethylsilylethyl protected precursor for the preparation of 5i, which allows for the deprotection of the alkoxy group.^51^ The reaction could be scaled up and gave 5i in 95% yield (4 mmol scale). A more dramatic effect of the protecting group was observed with precursors of tertiary α-alkoxyalkyl radicals. No traces of desired adduct 5j was formed between cyclopentenone 3d and tertiary 1-benzyloxyalkyl radical derived from 2k. In this case, we suspected that a competing β-fragmentation process was taking place, the tertiary radical leading to acetone and a stabilized benzylic radical.^52^ Indeed, Steenken and coworkers demonstrated by EPR study that the substitution at the radical centre of α-alkoxyalkyl radicals increases the rate of formation of a carbonyl compound and a new carbon-centred radical species *via* β-fragmentation,^53^ with the latter becoming faster when the carbon-centred radical released is more stable. When the benzyl substituent on the oxygen atom was replaced by a less stabilizing primary or secondary alkyl group, the deboronative radical conjugate addition to cyclopent-2-enone 3d proceeded smoothly, delivering adducts 5k–n in moderate to excellent yields (65–98%) from precursors 2l–o. Less strained cyclohex-2-enones 3f and 3g, as well as cyclohept-2-enone 3i, also proved suitable partners for the addition of benzylated primary precursor 2a, and good yields were obtained for the preparation of 6a (67% with conditions A, 75% with conditions B), 6b (87%) and 7a (64%). In contrast, the more hindered 4,4-dimethylcyclohex-2-enone 3h proved totally unreactive and no traces of adduct 6c were observed under these reaction conditions, most likely due to the steric bulk of the enone preventing radical addition. The alkoxyalkylation was then carried out on 4-cyclopentene-1,3-dione 3j, which delivered the hydroalkoxymethylated compound 8 in 69% yield. The structure of 8 was unambiguously confirmed by single-crystal X-ray diffraction analysis. The addition of the precursors 2l and 2m on the *exo*-methylene cyclolalkanone 3k gave the conjugated products 9a and 9b in good to excellent yields (60% and 91%, respectively). These results corroborate the hypothesis of β-fragmentation outlined above. In these two cases, high levels of *endo*-stereoselectivity were achieved for the control of the new stereogenic centers (dr > 95 : 5). The *endo*-selectivity of those products, also established in the literature for similar compounds, was determined for 9b by NMR NOESY experiments (see the SI for details).^54^ Finally 1-acylcyclohexene and 1-acylcyclopentene derivatives also proved to be suitable substrates. Addition of primary precursor 2a to 1-acetylcyclohexene 3l and 16-dehydropregnenolone acetate 3m, a key synthon for natural steroids and drugs, gave the products 10 and 11a in 44% and 77% yields, respectively.^55^ The addition of tertiary precursor 2m to 16-dehydropregnenolone acetate 3m delivered 11b in 64% yield. In these three last cases, good levels of stereoselectivity were observed for the control of the two new stereogenic centers.

### Reaction mechanism for the conjugated addition to enones

**Scheme 4:**
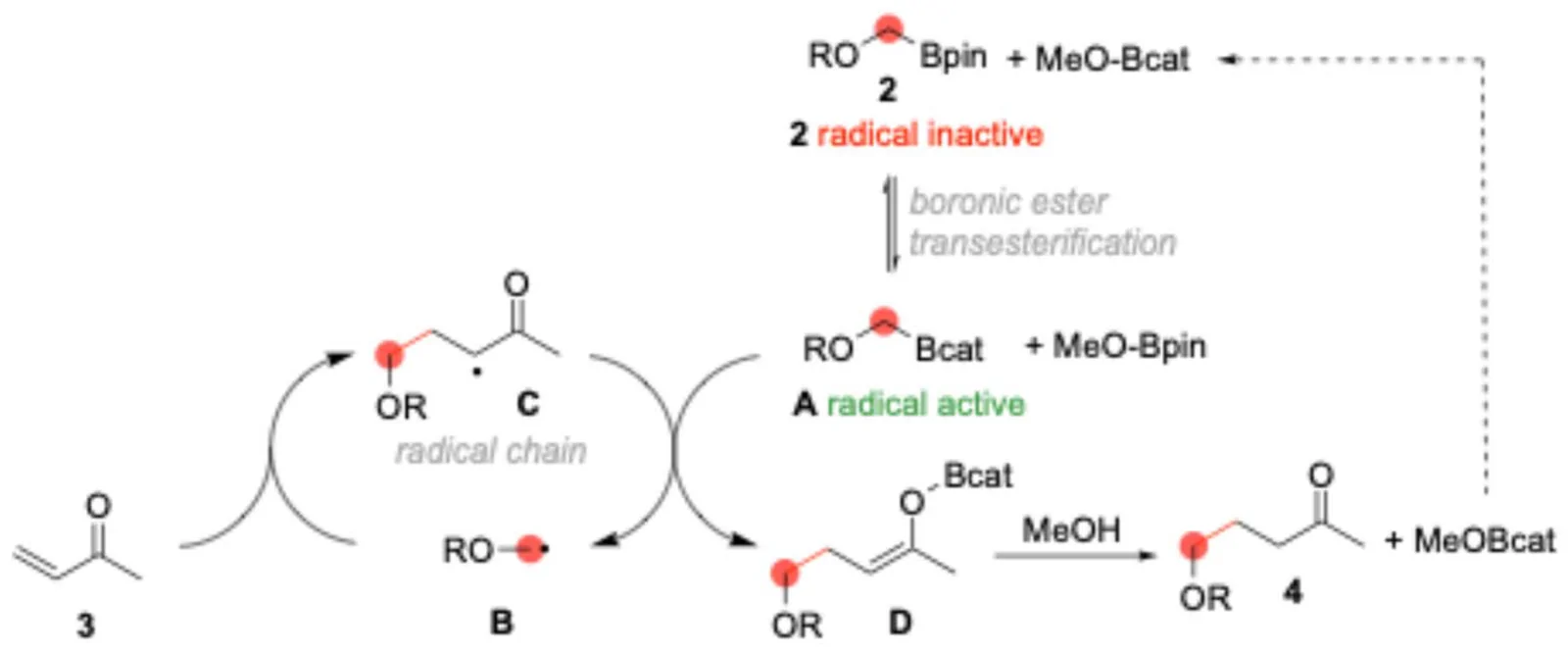
Proposed reaction mechanism for the deboronative radical chain conjugate addition to enones.

A plausible reaction mechanism for the radical addition to enones is depicted in Scheme 4. Transesterification of the boronic acid pinacol ester precursor 2 to the corresponding α-alkoxalkylBcat species A is achieved by reaction with MeOBcat.^56^ Initiation then involves the nucleo-homolytic substitution at the boron atom of the α-alkoxalkylBcat intermediate by a *tert*-butoxyl radical resulting from the cleavage of DTBHN upon heating (not shown).^57^ The resulting α-alkoxyalkyl radical B adds to the enone 3 to give enolyl radical C. The enolyl radical C, which possesses sufficient spin density at the oxygen atom to propagate the radical chain by reacting with α-alkoxalkylBcat species A, leads to boron enolate D and regenerates the reactive species B. The boron-enolate D is then protonated by MeOH, delivering the corresponding adduct 4 and regenerating the MeOBcat species. In absence of MeOH, the catecholboron-enolate D itself is likely to be involved in the boron-transesterification with the R–Bpin precursor. Attempts at trapping the final boron-enolate (for reaction carried out under aprotic conditions) using radical-polar crossover reaction with aldehydes proved capricious and, in our hands, low-yielding.

### Functionalisation with sulfonyl-based radical traps

**Scheme 5:**
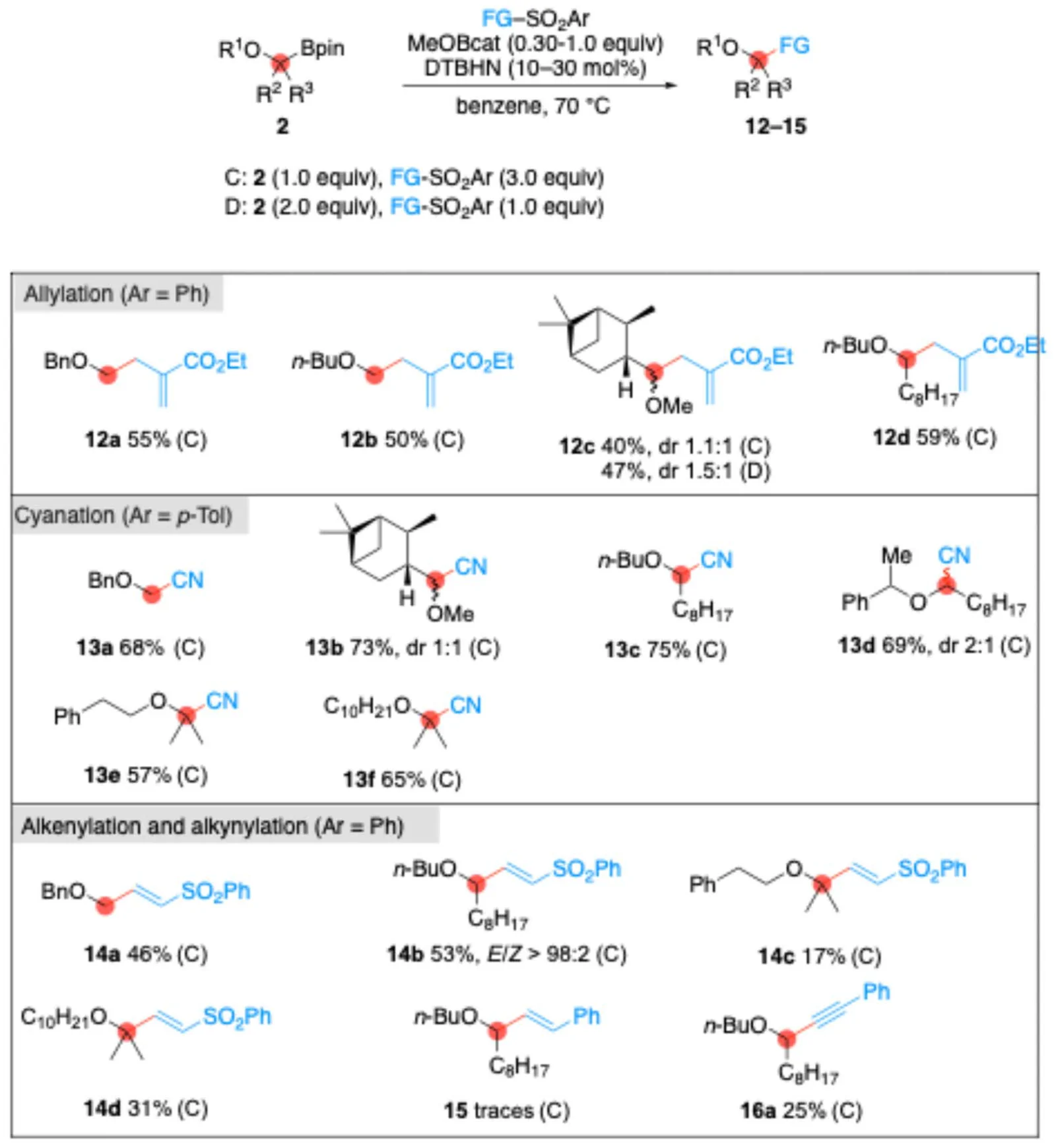
Functionalization of α-alkoxyalkyl boronic esters (yields of isolated products).

We chose to extend the scope of the alkoxyalkylation reaction with the use of easily available benzenesulfonyl-based radical traps (PhSO_2_–FG), which have been long-known to proceed *via* a chain reaction mechanism involving a transient benzenesulfonyl radical (PhSO_2_˙).^58^ Using reaction conditions C [Bpin precursor (1.0 equivalent), sulfone (3.0 equivalents), MeOBcat (0.3 equivalent) and DTBHN (10 mol%) in benzene at 70 °C], the reaction between precursor 2a and allyl sulfone 5a delivered the expected allylation product 12a in 55% yield. In this case, the best results were obtained in the absence of any additives (MeOH and/or TMSOTf) as their presence proved to be detrimental for the reaction (Scheme 5). Primary and secondary α-alkoxalkyl radical precursors 2a, 2d, 2g and 2h engaged in reaction with the electron-poor allylsulfone 5a to give the corresponding allylated adducts 12a–d in moderate yields (40–59%) using a three-fold excess of the radical trap (conditions C). Interestingly, the reaction carried out with an excess of Bpin precursor (2 equivalents) (conditions D) also provided satisfying yield for the allylated product 12c. The functionalization of α-alkoxalkyl radical precursors was then extended to other sulfonyl-based radical traps. The cyanation of primary Bpin precursor 2a with tosylcyanide delivered *O*-benzylated cyanhydrine 13a in 68% yield. Secondary α-alkoxalkyl radical precursors 2g, 2h and 2j were cyanated in good yields (69–75% for 13b–d). Tertiary α-alkoxalkyl radical precursors 2p and 2q gave the cyanated products 13e, and 13f in 57 and 65% yields, respectively. Primary and secondary α-alkoxalkyl radical precursors 2a and 2h could be alkenylated to give the products 14a and 14b in moderate yields (46 and 53%, respectively) and good levels of diastereoselectivity in favour of the *E*-isomer using (*E*)-1,2-bis(phenylsulfonyl)ethene. Tertiary α-alkoxalkyl radicals proved poorly reactive in the alkylation reaction with (*E*)-1,2-bis(phenylsulfonyl)ethene, with 14c and 14d being obtained in only 17% and 31% yields, respectively, from radical precursors 2p and 2q. In these cases, 2-phenylethanol and 1-decanol were isolated in up to 40% yield, showing that those products are rather unstable and prone to fragmentation. Alkenylation using less reactive (2-(phenylsulfonyl)vinyl)benzene gave only traces of alkene 15. Alkynylation of secondary α-alkoxalkyl radical precursors 2h with ((phenylethynyl)sulfonyl)benzene gave the corresponding alkynylated product 16a in only 25% yield. In this case, a radical chain mechanism involves the sulfonyl radical released during the attack of the α-alkoxyalkyl radical to the radical trap. The sulfonyl radical sustains the radical chain by reacting with α-alkoxyalkylBcat species generated by transesterification at the boron atom.^56,57^

**Scheme 6:**
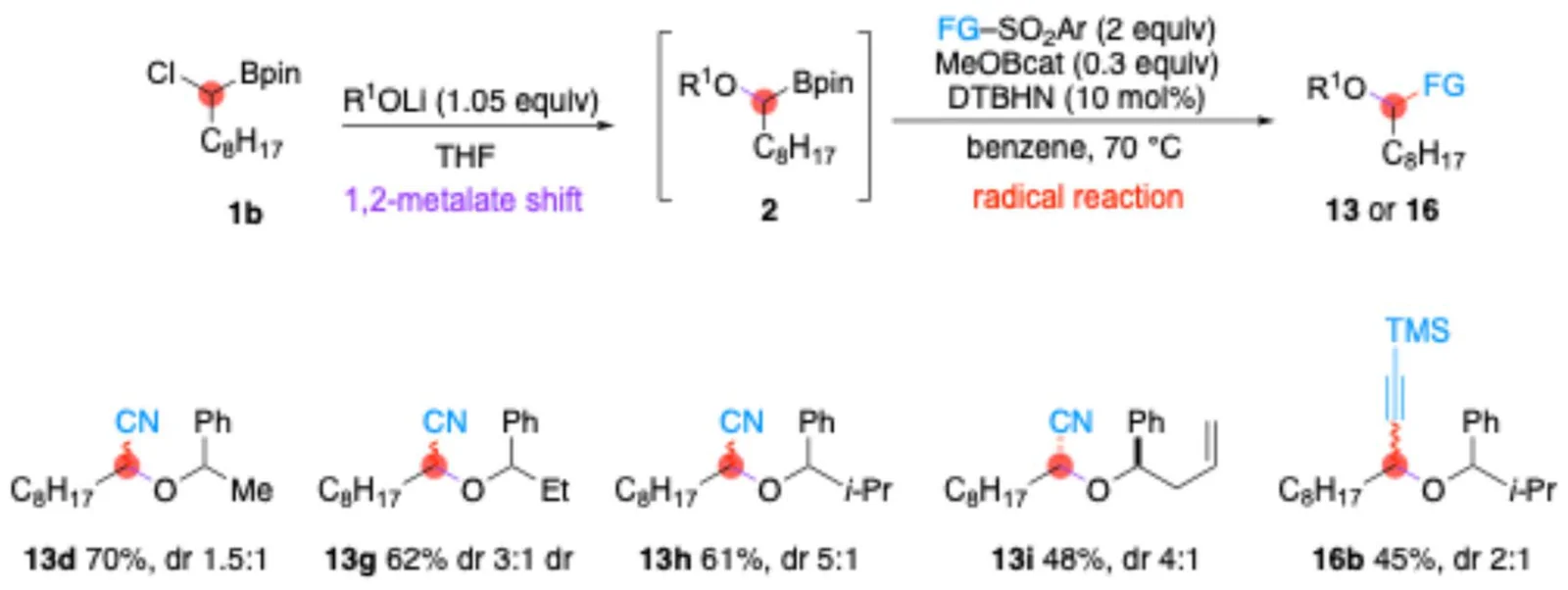
One-pot sequence for the diastereoselective cyanation and alkynylation of α-alkoxyalkyl boronic esters (yields of isolated products).

The possibility of performing inter- and intramolecular C–C bond formation using a one-pot protocol directly from α-chloroalkylBpin precursors was investigated next. Conducting the first step in benzene resulted in moderate yields over the two steps, with side-products arising from the *O*-1,2-migration of the pinacol moiety. Eventually, it was found that the best results were obtained by preparing the α-alkoxyalkyl pinacolboronic esters in THF and by running the radical reaction in benzene (see the SI for details). Under these optimized reaction conditions, the one-pot sequence involving a deboronative radical cyanation gave functionalized *O*-alkylated cyanohydrins 13d, and 13g–i in satisfying yields (48–70%) and diastereoselectivity levels ranging from 1.5 : 1 to 5 : 1 (Scheme 6). The 70% yield obtained for 13d using the one-pot protocol compares favourably with that previously obtained using the two-step approach with isolation of the Bpin precursor intermediate. Interestingly, no product arising from the 5-*exo-trig* cyclisation was observed in the reaction leading to 13i, suggesting that the cyanation of the α-alkoxyalkyl radical intermediate occurred faster than cyclisation. Finally, the alkynylation leading to 16b proceeded in 45% (dr 2 : 1), competing favorably with the yields obtained for alkynylation in the two-step process (see Scheme 5).

### Cyclization reactions

**Scheme 7:**
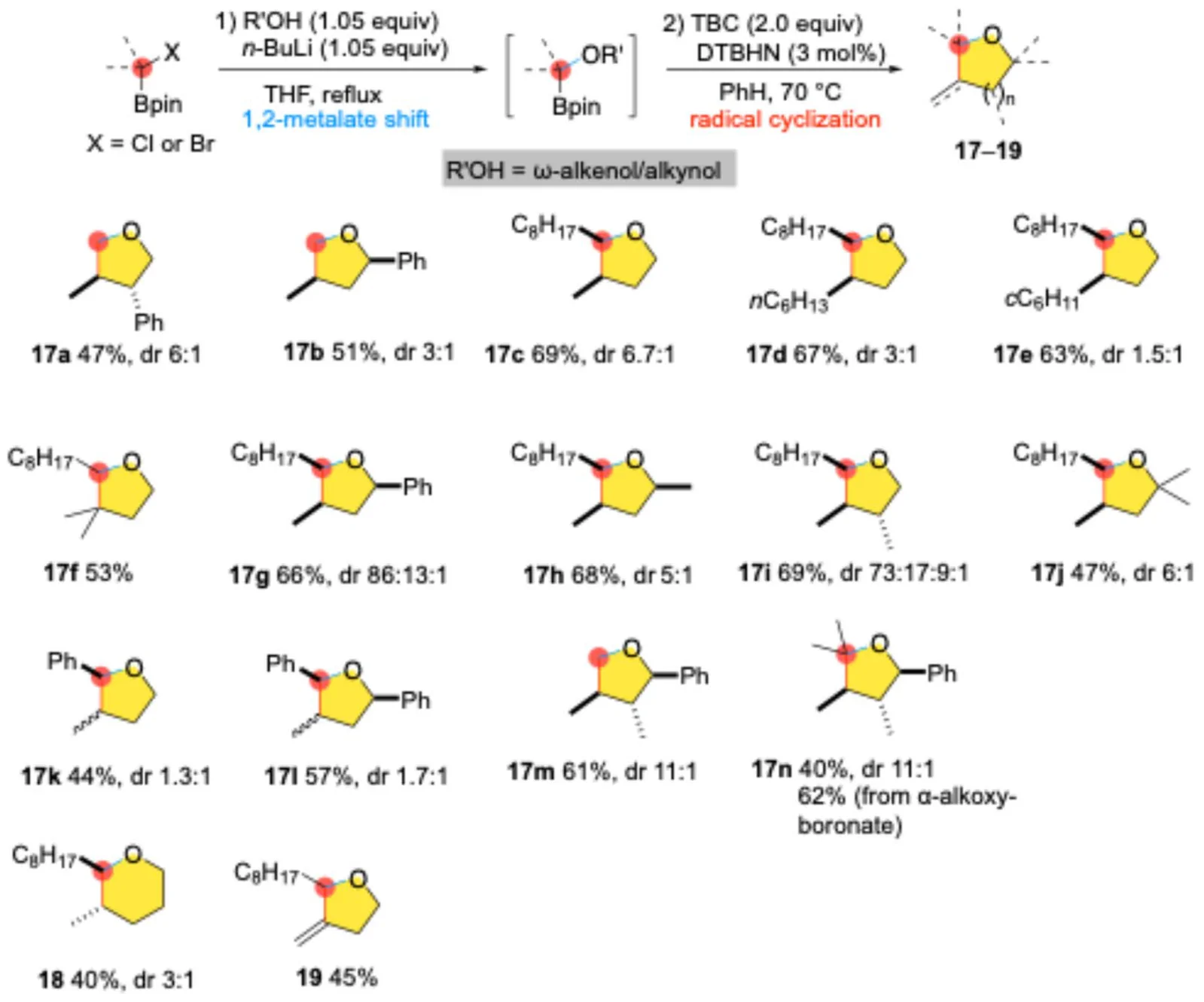
One-pot procedure for the preparation oxygen containing heterocycles. Relative configurations (major shown) tentatively assigned based on the Beckwith–Houk transition-state model for 5-*exo-trig* and 6-*exo-trig* cyclization.

**Scheme 8:**
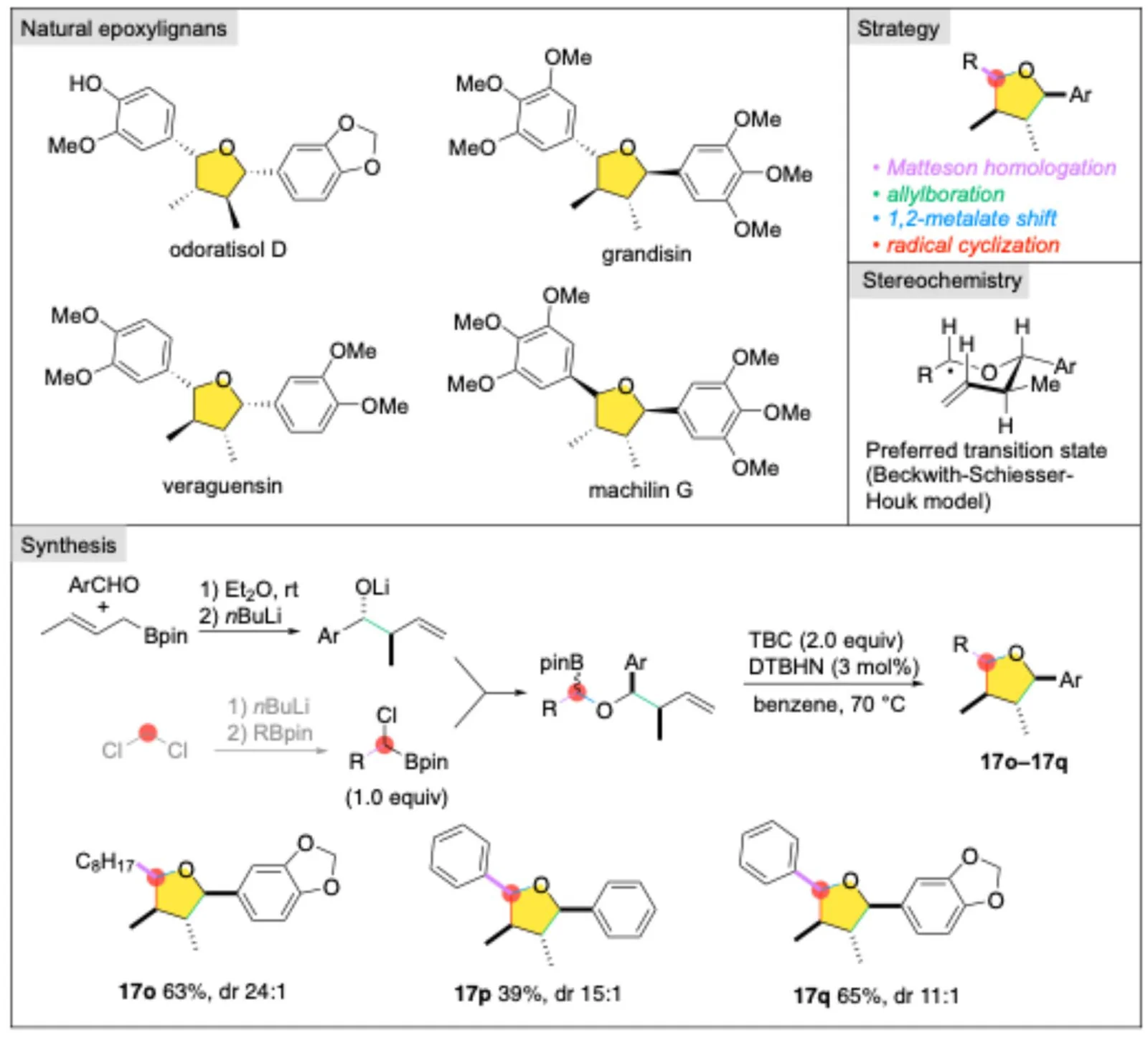
Stereoselective preparation of tetrasubstituted tetrahydrofurans related to neolignans.

The cyclization of 1-(alkenyloxy)alkyl radicals is an attractive method for the preparation of tetrahydrofurans. However, in contrast to 3-oxahex-5-enyl radicals, which undergo 1,5-*exo-trig* ring closure with high absolute rate constants (9 × 10^6^ s^−1^ at 25 °C),^59^ the cyclization of 2-oxa-5-hexenyl radicals has been reported to be about two order of magnitude slower (5 × 10^4^ s^−1^ at 25 °C).^60,61^ Beckwith and Glover reasoned that the activation energy “*reflects the energy required to bring the unpaired electron out of conjugation with the adjacent oxygen lone pair*” to adopt the optimum chair-like transition state for the ring closure.^59^ To achieve a deboronative radical cyclization with α-(alkenyloxy)alkyl radicals, the cyclization must compete with the intermolecular trapping of the radical prior to cyclization. This competition between cyclization and intermolecular trapping was emphasized by the afore-mentioned example of cyanation leading to 13i (Scheme 6). We already demonstrated that *tert*-butylcatechol (TBC) was a suitable source of hydrogen donor to achieve efficient tin-free hydroalkylation reaction. The rate constants for the reduction of secondary and primary alkyl radicals by TBC (1.3 × 10^6^ M^−1^ s^−1^ at 80 °C and 6.2 × 10^5^ M^−1^ s^−1^ at 50 °C, respectively) allows for its use as a source of hydrogen donor in radical rearrangements.^44,62,63^ We decided then to explore the use of TBC both to promote the transesterification of the air-stable Bpin precursors to the radical-active Bcat species intermediates and serve as a source of hydrogen atom to complete the radical chain reaction. Keeping in mind this possible competition with the direct reduction of α-(alkenyloxy)alkyl radicals by TBC, we started to investigate the 5-*exo-trig* ring closure of alkoxymethyl radicals. For our initial studies, α-bromomethyl pinacol boronic ester was used. The latter underwent a 1,2-metallate shift in THF as solvent with an *in situ* prepared lithium 2-phenylbut-3-en-1-olate to generate the corresponding α-(alkenyloxy)alkyl pinacoboronic ester intermediate. After switching to benzene, the 5-*exo-trig* ring closure to 17a was achieved in 47% yield (dr 6 : 1) with TBC and DTBHN (Scheme 7). Similarly, the one-pot sequence carried out with 1-phenylbut-3-en-1-ol as the homoallylic alcohol led to 2,4-disubstituted THF 17b in a moderate 51% yield (dr 3 : 1). Encouraged by these promising results, a series of pinacol α-(alkenyloxy)alkyl boronic esters were synthetized *via* the 1,2-metallate rearrangement of α-haloalkyl boronic ester ate-complexes with lithium alcoholate and engaged *in situ* in the cyclization process under the optimized reaction conditions. The resulting substituted α-alkoxyalkyl radicals proved slightly more reactive, delivering 2,3-disubstituted THFs 17c–e in good yields (63–69%). The levels of diastereoselectivity depend on the substitution pattern at the olefinic partner, with the best results in terms of selectivity being obtained with terminal, monosubstituted alkenes. For instance, 17c (dr 6.7 : 1) was obtained with a significant higher level of diastereoselectivity than that observed with internal alkene leading to 17d (dr 3 : 1), or trisubstituted alkene giving 17e (dr 1.5 : 1), in agreement with the steric repulsion generated by the two alkyl chains in *pseudo*-equatorial positions (see Scheme 8). The radical cyclization on a 1,1-disubstituted alkene gave the 2,3,3-trisubstituted THF 17f in 53% yield, with no traces of product resulting from an *endo*-cyclization mode. This contrasts with the all carbon system, for which *endo*-cyclization is usually observed.^64^ Likewise, using this one-pot sequence 2,3,5- and 2,3,4-trisubstituted THFs could be obtained from readily available substituted homoallyl alcohols. For instance, 2,3,5-trisubstituted THFs 17g and 17h were prepared in 66% and 68% yields, respectively, while 2,3,5-trisubstituted THFs 17i was isolated 69% yield as a mixture of four diastereomers (dr 73 : 17 : 9 : 1). Tetrasubstituted THFs 17j presenting a quaternary center at the α-position to the oxygen atom were prepared from a tertiary homoallyl alcohol and isolated in 47% and with a good level of diastereoselectivity (dr 6 : 1). To further study the influence of substitution pattern on the cyclization process, we examined the cyclization of α-alkoxyaryl radicals. With these less reactive benzylic radical intermediates, 2,3-disubstituted THF 17k and 2,3,5-trisubstituted THF 17l were then isolated in 44% and 57% yields, respectively. Finally, we tested the influence of the presence of an additional substituent at the allylic position. As expected, it allows for a better control of the diastereoselectivity of the radical cyclization step, with levels of stereoselectivity ranging from dr 11 : 1 up to 24 : 1. For instance, 1-phenyl-THF derivative 17m was isolated in 61% yield, with a high level of stereoselectivity (dr 11 : 1). The use of fully substituted haloalkylBpin derivatives such as Br(Me)_2_CBpin allowed for the preparation pentasubstituted THFs, as illustrated by the preparation of 17n, isolated in a moderate 40% yield using the one-pot protocol. In this case, the reaction carried out from the isolated α-(alkenyloxy)alkyl pinacoboronic ester intermediate delivered 17n in 62% yield (dr 92 : 8), demonstrating that the radical cyclization step took place smoothly in this case. A 6-*exo-trig* ring closure was evaluated and provided tetrahydropyran 18 in 40% yield as a 3 : 1 mixture of diastereomers. In this example, the reduced 1-(alkenyloxy)alkyl radical was obtained in 40% yield without any traces of alkene isomerization, suggesting that no competing HAT has occurred. Finally, monosubstituted 3-methylenetetrahydrofuran 19 was isolated in 45% yield through cyclization onto an alkyne substrate.

Tetrahydrofuran neolignans derived from natural sources, such as the 7,7′-epoxylignans odoratisol D, grandisin, machilin G and veraguensin depicted in Scheme 8, all contain a 2,4-diaryltetrahydrofuran skeleton and possess interesting biological activities such as anti-trypanosomal and anti-leishmanial activities. These molecules show a variety both in the nature of the substituents and in their relative configuration. Such tetrasubstituted 2,4-arylated tetrahydrofurans are possibly prepared in a straightforward manner from simple building blocks using the one-pot procedure previously described. The required benzylic homoallyl alcohols can be easily obtained as single diastereomers using classical Brown-type allylboration and related reactions (Scheme 8). Interestingly, this well-established and robust chemistry allows for the diastereoselective preparation of both the *syn* and *anti*-homoallyl alcohols, possibly in an enantioenriched form.^65^ Using our one-pot protocol, the *anti*-homoallyl alcohols were converted into 2,3,4,5-tetrasubstituted THFs presenting one or two aromatic substituents alpha to the oxygen atom. Using an α-chloroalkylBpin as the organoboron building block, 2,3,4,5-tetrasubstituted mono-aryl-THF 17o was obtained in 63% yield and excellent stereocontrol (dr 24 : 1). Similarly, 2,5-diaryl THFs 17p and 17q presenting the same substitution pattern and relative configurations as in neo-lignans odoratisol D and veraguensin, were obtained in 39% and 65% yields, respectively, with good levels of diastereoselectivity (dr ≥ 11 : 1). Although benzylic α-alkoxyalkyl radicals yielded promising results (Scheme 8, compounds 17p and 17q, R = Ph), the synthesis of natural neo-lignans has not yet been achieved. When the aryl group of the Bpin precursor bears strongly electron-donating alkoxy substituents (R = alkoxylated aryl), fragmentation products were observed. This issue has not yet been addressed.

The reaction mechanism presumably involves the boron-transesterification between the original Bpin species and TBC and proceeds following the previously reported reaction mechanism for simple protodeborylation.^56^ The high levels of diastereoselectivity observed for 2,3-substituted THF compounds prepared from anti-homoallylic alcohols can be rationalized by a chair-like transition state according to the Beckwith–Schiesser–Houk model (Scheme 8).^64,66^ The major product leading to 2,3-disubstituted THFs arises from a 5-*exo-trig* cyclization, with the substituents occupying a *pseudo*-equatorial position in the transition state to minimize the 1,3-diaxial interactions. The shorter bond lengths in chair-like transition states containing an oxygen atom increase the strain on the forming ring, providing good *cis*-selectivities.^67^

## Conclusion

A straightforward and effective method to produce a broad spectrum of α-alkoxyalkyl radicals was developed. It uses stable and readily accessible pinacol boronic ester precursors, which undergo transesterification with MeOBcat, followed by nucleohomolytic substitution at the boron centre with an oxygen-centred radical. This metal-free and non-oxidative method offers a practical approach for the mild and regioselective formation of α-alkoxyalkyl radicals to access dialkyl ether-containing target molecules, including natural product-like derivatives and other pharmacologically significant compounds.

## Author contributions

EAJ: methodology, investigation, writing initial draft. LB: methodology, investigation, writing initial draft. AF: investigations. FD: conceptualization, supervision, data curation, writing of the final version. PR: conceptualization, funding acquisition, supervision, writing of the final version.

## Conflicts of interest

There are no conflicts of interest to declare.

## Supplementary Material

QO-013-D6QO01025C-s001

QO-013-D6QO01025C-s002

## Data Availability

The data that support the findings of this study are openly available in the supplementary information (SI). Supplementary information: experimental procedures, NMR spectra, IR-spectra, and HRMS data. See DOI: https://doi.org/10.1039/d6qo01025c. CCDC 2564009 and 2564022 contain the supplementary crystallographic data for this paper.^68*a*,*b*^
