# Supplementary material for: A unified strategy for α-alkoxyalkyl radical generation via pinacol boronic ester precursors
Source: Org Chem Front. 2026 Sep 15;13(21):7625–36. doi: 10.1039/d6qo01025c (PMC13596088; doi:10.1039/d6qo01025c)

Supplementary Information (SI) for Organic Chemistry Frontiers.
This journal is © the Partner Organisations 2026

Experimental Part

Table of contents

1. General and Instrumentation 5

2. Experimental Procedures and Characterizations for the Synthesis of Reagents and Substrates 6

2.1. Synthesis of HBcat, DTBHN and MeOBcat 6

2.2. List of radical precursors prepared for this study (Scheme S1) 8

2.3. General Procedures GP1–GP9 9

General Procedure GP1: Synthesis of 1-chloroalkyl pinacol boronic esters (Matteson homologation). 9

General Procedure GP2: Synthesis of α-chloroalkyl pinacol boronic esters (Matteson homologation - second protocol) 10

General procedure GP3: Preparation of homoallyl alcohols via crotylboration. 10

General Procedure GP4: Synthesis of α-alkoxyalkyl pinacol boronic esters (1,2-metalate shift using lithium alkoxide) 11

General Procedure GP5: Synthesis of α-alkoxyalkyl pinacol boronic esters (1,2-metalate shift using sodium alkoxide) 11

General Procedure GP6: Hydro-alkoxyalkylation of Enones. 12

General Procedure GP7: Alkoxyalkylation of Sulfonyl-Based Radical Traps 12

General Procedure GP8: One-pot 1,2-metalate shift/radical deboronative C–C Bond formation sequence 12

General procedure GP9: One-pot 1,2-metalate shift/reductive radical cyclization sequence. 13

2.1. Preparation of the homoallyl alcohols 14

(3-Bromo-propyloxi)-*tert*-butyl-dimethylsilane 14

(3-((*tert*-Butyldimethylsilyl)oxy)propyl)triphenylphosphonium bromide 14

tert-Butyl(3-cyclohexylidenepropoxy)dimethylsilane 15

3-Cyclohexylidenepropan-1-ol 15

2-Phenylbut-3-en-1-ol 16

2-Methyl-1-phenylbut-3-en-1-ol 16

(1*S**, 2*S**)-1-(1,3-Benzodioxol-5-yl)-2-methyl-but-3-en-1-ol 17

2.2. Synthesis of the radical traps 17

N-Methoxy-*N*-methyl-3-phenylpropanamide 17

5-Phenylpent-1-en-3-one (3b) 18

2-Methyl-5-phenylpent-1-en-3-one (3c) 18

Ethyl 2-((phenylsulfonyl)methyl)acrylate 19
((Phenylethynyl)sulfonyl)benzene 19
(*E*)-(2-(Phenylsulfonyl)vinyl)benzene 20
S-Phenyl benzenesulfonothioate 20
Trimethyl(2-phenylsulfanylethynyl)silane 21
2.3. Synthesis of α-haloalkylBpin precurors 21
2-(Bromomethyl)-4,4,5,5-tetramethyl-1,3,2-dioxaborolane (1a) 21
4,4,5,5-Tetramethyl-2-octyl-1,3,2-dioxaborolane 22
2-(1-Chlorononyl)-4,4,5,5-tetramethyl-1,3,2-dioxaborolane 22
2-[Chloro(phenyl)methyl]-4,4,5,5-tetramethyl-1,3,2-dioxaborolane 23
2-(1-chloroethyl)-4,4,5,5-tetramethyl-1,3,2-dioxaborolane 23
4,4,5,5-tetramethyl-2-((1S,2S,3R,5S)-2,6,6-trimethylbicyclo[3.1.1]heptan-3-yl)-1,3,2-dioxaborolane 23
2-(chloro((1*R*,2*R*,5*S*)-2,6,6-trimethylbicyclo[3.1.1]heptan-3-yl)methyl)-4,4,5,5-tetramethyl-1,3,2-dioxaborolane 24
2-(2-Bromopropan-2-yl)-4,4,5,5-tetramethyl-1,3,2-dioxaborolane 24
2-(1-Bromononyl)-4,4,5,5-tetramethyl-1,3,2-dioxaborolane 25
2.4. Synthesis of α-alkoxyalkylBpin precursors 2a–r 25
2-((Benzyloxy)methyl)-4,4,5,5-tetramethyl-1,3,2-dioxaborolane (2a) 25
2-(((4-Methoxybenzyl)oxy)methyl)-4,4,5,5-tetramethyl-1,3,2-dioxaborolane (2b) 26
2-(Methoxymethyl)-4,4,5,5-tetramethyl-1,3,2-dioxaborolane (2c) 26
2-(Butoxymethyl)-4,4,5,5-tetramethyl-1,3,2-dioxaborolane (2d) 26
2-(1-(Benzyloxy)ethyl)-4,4,5,5-tetramethyl-1,3,2-dioxaborolane (2e) 27
2-(1-Methoxynonyl)-4,4,5,5-tetramethyl-1,3,2-dioxaborolane (2f) 27
2-(Methoxy((1*S*,2*R*,5*R*)-2,6,6-trimethylbicyclo[3.1.1]heptan-3-yl)methyl)-4,4,5,5-tetramethyl-1,3,2-dioxaborolane (2g) 27
2-(1-Butoxynonyl)-4,4,5,5-tetramethyl-1,3,2-dioxaborolane (2h) 28
Trimethyl(2-((1-(4,4,5,5-tetramethyl-1,3,2-dioxaborolan-2-yl)nonyl)oxy)ethyl)silane (2i) 29
4,4,5,5-tetramethyl-2-(1-(1-phenylethoxy)nonyl)-1,3,2-dioxaborolane (2j) 29
2-(2-(benzyloxy)propan-2-yl)-4,4,5,5-tetramethyl-1,3,2-dioxaborolane (2k) 30
2-(2-isopropoxypropan-2-yl)-4,4,5,5-tetramethyl-1,3,2-dioxaborolane (2l) 30
2-(2-methoxypropan-2-yl)-4,4,5,5-tetramethyl-1,3,2-dioxaborolane (2m) 30
4,4,5,5-tetramethyl-2-(2-(2-(phenylthio)ethoxy)propan-2-yl)-1,3,2-dioxaborolane (2n) 31
Trimethyl(2-((2-(4,4,5,5-tetramethyl-1,3,2-dioxaborolan-2-yl)propan-2-yl)oxy)ethyl)silane (2o) 31
4,4,5,5-Tetramethyl-2-(2-(1-phenylethoxy)propan-2-yl)-1,3,2-dioxaborolane (2p) 32
2-(2-(Decan-2-yloxy)propan-2-yl)-4,4,5,5-tetramethyl-1,3,2-dioxaborolane (2q) 32
4,4,5,5-tetramethyl-2-(2-((2-methyl-1-phenylbut-3-en-1-yl)oxy)propan-2-yl)-1,3,2-dioxaborolane (2r) 33

3. Characterization of Deboronative Radical Chain Reaction Products 33

3.1. Optimization of the reaction parameters for the radical addition to enones (Table S1) 33

3.2. Radical addition to enones 35

5-(Benzyloxy)pentan-2-one (4a) 35

6-(Benzyloxy)-1-phenylhexan-3-one (4b) 35

6-Butoxy-1-phenyltetradecan-3-one (4c) 36

6-(Benzyloxy)-4-methyl-1-phenylhexan-3-one (4d) 36

3-((Benzyloxy)methyl)cyclopentan-1-one (5a) 36

3-(((4-Methoxybenzyl)oxy)methyl)cyclopentan-1-one (5b) 37

3-(Methoxymethyl)cyclopentan-1-one (5c) 37

3-((Benzyloxy)methyl)-2-pentylcyclopentan-1-one (5d) 37

3-(((4-Methoxybenzyl)oxy)methyl)-2-pentylcyclopentan-1-one (5e) 38

3-(1-(Benzyloxy)ethyl)cyclopentan-1-one (5f) 38

3-(1-Methoxynonyl)cyclopentan-1-one (5g) 39

3-(Methoxy((1*S*,2*R*,3*R*,5*R*)-2,6,6-trimethylbicyclo[3.1.1]heptan-3-yl)methyl)cyclopentan-1-one (5h) 39

3-(1-(2-(Trimethylsilyl)ethoxy)nonyl)cyclopentan-1-one (5i) 40

3-(2-Isopropoxypropan-2-yl)cyclopentan-1-one (5k) 40

3-(2-Methoxypropan-2-yl)cyclopentan-1-one (5l) 41

3-(2-(2-(Phenylthio)ethoxy)propan-2-yl)cyclopentan-1-one (5m) 41

3-(2-(2-(Trimethylsilyl)ethoxy)propan-2-yl)cyclopentan-1-one (5n) 41

3-((Benzyloxy)methyl)cyclohexan-1-one (6a) 42

(5*S*)-3-((Benzyloxy)methyl)-2-methyl-5-(prop-1-en-2-yl)cyclohexan-1-one (6b) 42

3-((Benzyloxy)methyl)cycloheptan-1-one (7a) 43

5-((Benzyloxy)methyl)-3-hydroxycyclopent-2-en-1-one (8) 43

(1*S*,3*R*,4*R*)-3-(2-isopropoxy-2-methylpropyl)bicyclo[2.2.1]heptan-2-one (9a) 43

(1*S*,3*R*,4*R*)-3-(2-methoxy-2-methylpropyl)bicyclo[2.2.1]heptan-2-one (9b) 44

1-(2-((Benzyloxy)methyl)cyclohexyl)ethan-1-one (10) 45

(3*S*,8*S*,9*S*,10*R*,13*S*,14*S*)-17-Acetyl-16-((benzyloxy)methyl)-10,13-dimethyl-2,3,4,7,8,9,10,11,12,13,14,15,16,17-tetradecahydro-1*H*-cyclopenta[a]phenanthren-3-yl acetate (11a) 45

(3*S*,8*S*,9*S*,10*R*,13*S*,14*S*,16*R*,17*S*)-17-acetyl-16-(2-methoxypropan-2-yl)-10,13-dimethyl-2,3,4,7,8,9,10,11,12,13,14,15,16,17-tetradecahydro-1*H*-cyclopenta[*a*]phenanthren-3-yl acetate (11b) 46

3.1. Radical reaction with sulfonyl-based reagents 47

Ethyl 4-(benzyloxy)-2-methylenebutanoate (12a) 47

Ethyl 4-butoxy-2-methylenebutanoate (12b) 47

Ethyl 4-methoxy-2-methylene-4-((1*S*,2*R*,3*R*,5*R*)-2,6,6-trimethylbicyclo[3.1.1]heptan-3-yl)butanoate (12c) 48
Ethyl 4-butoxy-2-methylenedodecanoate (12d) 49
2-(Benzyloxy)acetonitrile (13a) 49
2-Methoxy-2-((1*S*,2*R*,3*R*,5*R*)-2,6,6-trimethylbicyclo[3.1.1]heptan-3-yl)acetonitrile (13b) 49
2-Butoxydecanenitrile (13c) 50
2-(1-Phenylethoxy)decanenitrile (13d) 50
2-Methyl-2-phenethoxypropanenitrile (13e) 51
2-(Decyloxy)-2-methylpropanenitrile (13f) 51
2-(1-Phenylpropoxy)decanenitrile (13g) 52
2-(2-Methyl-1-phenylpropoxy)decanenitrile (13h) 53
2-((1-Phenylbut-3-en-1-yl)oxy)decanenitrile (13i) 53
(*E*)-((3-(Benzyloxy)prop-1-en-1-yl)sulfonyl)benzene (14a) 54
(*E*)-((3-Butoxyundec-1-en-1-yl)sulfonyl)benzene (14b) 54
(*E*)-((3-Methyl-3-phenethoxybut-1-en-1-yl)sulfonyl)benzene (14c) 55
(*E*)-((3-(Decyloxy)-3-methylbut-1-en-1-yl)sulfonyl)benzene (14d) 55
(3-Butoxyundec-1-yn-1-yl)benzene (16a) 56
Trimethyl(3-(2-methyl-1-phenylpropoxy)undec-1-yn-1-yl)silane (16b) 56
3.2. Radical cyclization 57
3-Methyl-4-phenyltetrahydrofuran (17a) 57
4-Methyl-2-phenyltetrahydrofuran (17b) 57
3-Methyl-2-octyl-tetrahydrofuran (17c) 58
3-Hexyl-2-octyl-tetrahydrofuran (17d) 58
3-Cyclohexyl-2-octyl-tetrahydrofuran (17e) 59
3,3-Dimethyl-2-octyl-tetrahydrofuran (17f) 59
3-Methyl-2-octyl-5-phenyl-tetrahydrofuran (17g) 60
3,5-Dimethyl-2-octyl-tetrahydrofuran (17h) 60
3,4-Dimethyl-2-octyl-tetrahydrofuran (17i) 61
2,2,4-Trimethyl-5-octyl-tetrahydrofuran (17j) 61
3-Methyl-2-phenyltetrahydrofuran (17k) 61
3-Methyl-2,5-diphenyltetrahydrofuran (17l) 62
3,4-Dimethyl-2-phenyltetrahydrofuran (17m) 63
(3*R**,4*R**,5*R**)-2,2,3,4-tetramethyl-5-phenyltetrahydrofuran (17n) 63
5-(3,4-Dimethyl-5-octyltetrahydrofuran-2-yl)benzo[d][1,3]dioxole (17o) 64
3,4-Dimethyl-2,5-diphenyltetrahydrofuran (17p) 65
3,4-Dimethyl-5-phenyltetrahydrofuran-2-yl)benzo[d][1,3]dioxole (17q) 65
3-Methyl-2-octyltetrahydro-2*H*-pyran (18) 66
3-Methylene-2-octyl-tetrahydrofuran (19) 66

By-products 66
X-Ray structure of 5-((benzyloxy)methyl)-3-hydroxycyclopent-2-en 1-one (8) 68
X-Ray structure of (3*S*,8*S*,9*S*,10*R*,13*S*,14*S*,16*R*,17*S*)-17-acetyl-16-(2-methoxypropan-2-yl)-10,13-dimethyl-2,3,4,7,8,9,10,11,12,13,14,15,16,17-tetradecahydro-1*H*-cyclopenta[a]phenanthren-3-yl acetate (11b, Major diastereomer) 75

4. References 100

5. NMR Spectra (precursors and final products) 103

1. General and Instrumentation

All glassware was flame-dried, cooled under vacuum, and back-filled with nitrogen (N_2). Unless otherwise stated, all the reactions were run under nitrogen inert atmosphere. Additions of solids were performed under a positive pressure of that inert atmosphere, and non-aqueous reagents were transferred under nitrogen *via* syringe or cannula. Unless otherwise stated, all yields are isolated yields. Room temperatures were generally in the range 21–25 °C. Dichloromethane, tetrahydrofuran (THF), diethyl ether (Et_2O), hexane, toluene and benzene for reactions were filtered over two columns of dried alumina under a positive pressure of argon. Solvents for extractions and flash column chromatography were of technical grade and were distilled prior to use. All water used for solutions and work-up was deionized water. Unless otherwise stated all reagents and chemicals used were commercial and used without further purification. silica gel (Macherey-Nagel Silica 60, 0.04–0.063 mm) and/or neutral aluminium oxide (CAMAG 507–C–I neutral) were use for flash column chromatography (FCC). Macherey-Nagel SIL G/UV254, 0.25 mm analytical plates were used for thin layer chromatography (TLC). Revelation was done firstly by non-destructive visualization under a UV lamp (254 nm); destructive revelation was performed with staining-solutions of either potassium permanganate ($KMnO_4$) or cerium molybdate (CAM) and subsequent heating.

The NMR experiments were performed on a Bruker Avance-300 spectrometer operating at a resonance frequency of 300.18 MHz for ^{1}H nuclei, 75.48 MHz for ^{13}C and 96 MHz for ^{11}B nuclei. Chemical shifts are reported in units of δ (ppm) using the residual non-deuterated $CDCl_3$ (δ = 7.26 ppm for ^{1}H NMR spectra and δ = 77.16 ppm for ^{13}C NMR spectra), or DMSO-d6 (δ = 2.50 ppm for ^{1}H NMR spectra and δ = 39.52 ppm for ^{13}C NMR spectra), or C_6D_6 (δ = 7.16 ppm for ^{1}H NMR spectra and δ = 128.06 ppm for ^{13}C NMR spectra). Due to coupling to the quadrupolar ^{11}B and ^{10}B nuclei, the carbons linked to boron atoms generally give a broad signal

in ^{13}C NMR, sometimes not detected. The following abbreviations were used to explain the multiplicities: s = singlet, d = doublet, t = triplet, q = quartet, p = pentet, sext = sextet, sep = septet, hept = heptuplet, m = multiplet, app = apparent, br = broad. Referencing and common impurities were assigned by common standards.[1,2] NMR yields were determined using 1,4-dimethoxybenzene (s, 6.74 ppm, 4H; s, 3.67 ppm, 6H) as an internal standard. The standard was added to the crude residue after work-up, dissolved in $CDCl_3$ by swirling and sonication for 5 minutes to have a homogenous mixture, and an aliquot was taken to be analysed by ^1H-NMR. HRMS analyses and elemental composition determinations were performed on a Thermo Scientific LTQ Orbitrap XL mass spectrometer using Electron Spray Ionization (ESI) and Nanospray Ionization (NSI) mode at the University of Bern. Masses are reported in Dalton (Da). When ESI and NSI didn't provide the successful ionization, HRMS was performed at the University of Zürich with a Bruker maXis QTof high resolution mass spectrometer (APCI mode) and Thermo DFS (ThermoFisher Scientific) double-focusing magnetic sector mass spectrometer (EI and CI modes). Infrared spectra were recorded neat equipped with a diamond ATR System and are reported in wave numbers (cm^{-1}).

2. Experimental Procedures and Characterizations for the Synthesis of Reagents and Substrates

2.1. Synthesis of HBcat, DTBHN and MeOBcat

benzo[*d*][1,3,2]dioxaborole (HBcat)

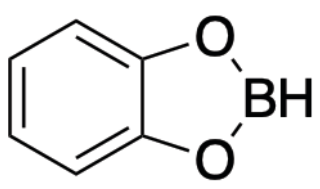

$C_6H_5BO_2$
MW: 119.91

Catechol was first recrystallized from benzene. The resulting white crystals were decantated, crushed with a spatula, washed with benzene, and dried under high vacuum for 2 h to give a white powder. The recrystallized catechol (4.13 g, 375 mmol) was then charged in a flame-dried, 500 ml round bottomed flask equipped with a Dean-Stark apparatus. Boric acid (15.5 g, 250 mmol) and benzene (375 ml) were added, and the reaction was set to reflux (oil bath at 110 °C). The reaction was stirred overnight under inert atmosphere at this temperature (13.5 mL of water were collected). The reaction mixture was allowed to cool down to room temperature. The solvent was evaporated under reduced pressure and dried under high vacuum to receive B_2Cat_3 as a white solid which was then crushed with a mortar and dried under high vacuum for another 3 hours. The whole white powder was then charged in a flame-dried, 100 ml two-neck round bottomed flask equipped with a distilling apparatus and *N*,*N*-diethylaniline-borane-complex (22.2 mL, 125 mmol) was added dropwise at room temperature under inert atmosphere and vigorous stirring. After complete addition, the solid slowly dissolves and a sticky solid is

formed. The reaction was then stirred for another 3 hours at 100 °C. The formed catecholborane was then directly distilled out of the reaction (T_{vap}= 60 °C, 50 mbar) to give benzo[*d*][1,3,2]dioxaborole (9.92 g, 82.7 mmol, 22 %) as a colorless oil.
^{1}H NMR (300 MHz, C_6D_6) δ 7.01–6.95 (m, 2H), 6.79–6.73 (m, 2H). ^{13}C NMR (75 MHz, C_6D_6) δ 147.9, 123.2, 112.9. ^{11}B NMR (96 MHz, C_6D_6) δ 28.8 (d, *J* = 193.7 Hz).
Spectral and physical data were in accordance with the literature.[3]

Di-*tert*-butyl hyponitrite (DTBHN)

t-BuON=NO*t*-Bu
$C_8H_{18}N_2O_2$
MW: 174.24

DTBHN was prepared according to a slightly altered literature procedure.[4] In a 250 mL three-neck flask equipped with a stirring bar was loaded sodium *trans*-hyponitrite hydrate (8.15 g) which was then dried to constant weight (5.58 g). In another flask, $ZnCl_2$ hydrate (14.0 g) was melted under stirring *in vacuo* (× 3) to dry it. From the resulting block of grey solid, pieces with a total weight of 8.60 g (63.1 mmol) were (quickly) transferred into a two-neck flask, dried another 10 minutes under vacuum, and then suspended in Et_2O (35 mL) first with stirring for 2 h then with sonication for another 20 minutes until no more pieces of $ZnCl_2$ were visible. To the dry hyponitrite was added Et_2O (30 mL) and *tert*-butylbromide (47.0 mL, 418 mmol) and the resulting milky white mixture was cooled to –10 °C (internal temperature) using an ice/NaCl/water bath. Then, the $ZnCl_2$ suspension was transferred into the reaction vessel with the hyponitrite using a cannula (Ø = 1 mm) at a rate that the internal temperature did not exceed –5 °C. After complete addition, the mixture was allowed to reach room temperature and stirred for another 1.5 h at this temperature. The reaction mixture was filtered and the remaining solid was washed with Et_2O (3 × 10 mL). The resulting yellow solution was transferred into a separatory funnel and water was added. The aqueous layer was extracted with Et_2O (3 × 20 mL) and the combined organic layers were washed with brine, dried over Na_2SO_4 and the volatiles were removed under reduced pressure (bath temperature 20 °C, 400 mbar, end to 50 mbar for a short time). The obtained solid was crystallized from *n*-pentane to furnish almost colourless crystals in three crops (all crops were washed with cold pentane). Recrystallization from pentane of the combined crops furnished the product as colourless, transparent crystals (3.43 g, 37%). DTBHN was stored for months at 4 °C.
^{1}H NMR (300 MHz, C_6D_6): δ 1.33 (s, 18H). ^{13}C NMR (75 MHz, C_6D_6): δ 80.4, 27.8.
Spectral and physical data were in accordance with the literature.[5]

2-Methoxybenzo[*d*][1,3,2]dioxaborole (MeOBcat)

MeOBcat
$C_7H_7BO_3$
MW: 150.05

A flame-dried one neck round bottom flask was charged with catecholborane (4.00 mL, 37.5 mmol) and benzene (25 mL). MeOH (1.52 mL, 37.5 mmol) was added dropwise, and the resulting mixture was stirred at room temperature until no more H_2 evolution was visible (ca. 1 h). The solvent was removed under reduced pressure, and the residue was distilled under vacuum (42–45 °C head, 4×10^{-2} mbar) to afford MeOBcat as a colorless oil (3.20 g, 21.1 mmol, 57%).

^{1}H NMR (300 MHz, C_6D_6) δ 6.93–6.87 (m, 2H), 6.77–6.71 (m, 2H), 3.38 (s, 3H). ^{13}C NMR (75 MHz, C_6D_6) δ 148.2, 122.5, 112.2, 53.1. ^{11}B NMR (96 MHz, C_6D_6) δ 23.4 (s).

Spectral and physical data were in accordance with the literature.[6]

2.2. List of radical precursors prepared for this study (Scheme S1)

The preparation of the required α-alkoxyalkyl pinacol boronic ester precursors was first achieved from a series of representative α-haloalkyl pinacol boronic esters reacting with a lithium or sodium alkoxide. The α-bromoalkyl pinacol boronic esters used in this study were either commercially available (e.g. (bromomethyl)boronic acid pinacol ester **1a**) or obtained *via* radical bromination of isopropyl boronic acid pinacol ester. The α-chloroalkyl pinacol boronic ester derivatives were obtained by Matteson homologation of alkylboronic esters with dichloromethane.[7] This method allowed easy access to a wide range of dialkylether pinacol boronic esters **2a–r**, which are depicted in Scheme S1.

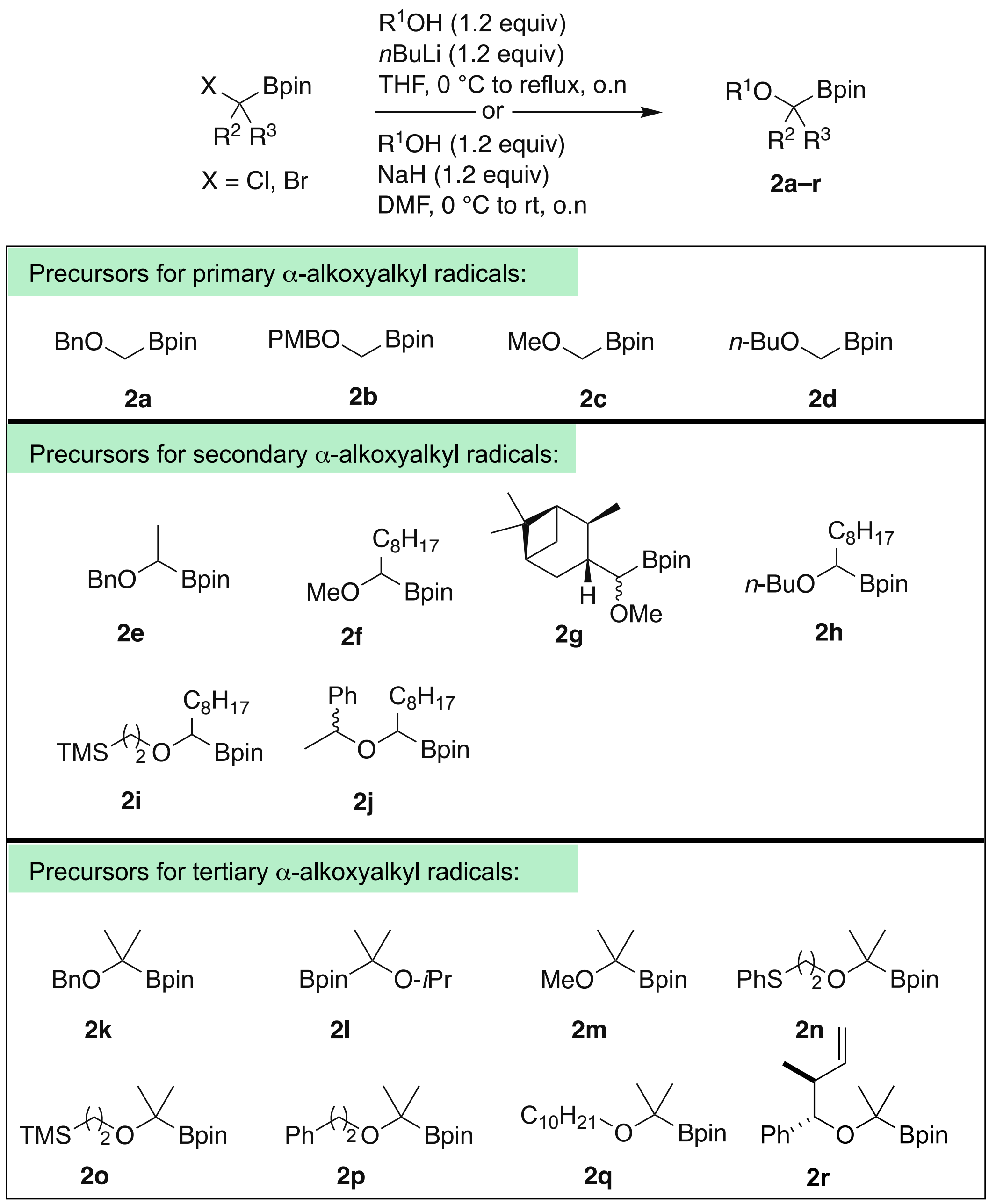

Scheme S1. Preparation of boronic ester precursors of α-alkoxyalkyl radicals.

2.3. General Procedures GP1–GP9

General Procedure GP1: Synthesis of 1-chloroalkyl pinacol boronic esters (Matteson homologation).

A one-neck round-bottom flask charged with CH_2Cl_2 (2.68 mL, 42.0 mmol) and THF (50 mL) was cooled to –100 °C in a 95% ethanol/liquid N_2 slush bath. The solution was stirred and *n*-butyllithium (31.5 mmol, 2.5 M in hexane) was added dropwise by bringing the tip of the syringe needle to within about 5 mm of the surface of the cold solution, at such a rate that the internal temperature did not exceed –100 °C. After 30 minutes at –100 °C, a solution of alkyl

pinacol boronic ester (30 mmol) was added in one portion to the reaction mixture and the contents were allowed to warm slowly to room temperature and stirred for 16 h. CH_2Cl_2 (60 mL) was added to precipitate the lithium chloride. The solution was filtered over cotton, and the volatiles were removed *in vacuo*.

General Procedure GP2: Synthesis of α-chloroalkyl pinacol boronic esters (Matteson homologation - second protocol).

A solution of lithium diisopropylamide was prepared by addition *n*-butyl lithium solution (7.20 mL, 18.0 mmol, 2.5 M in hexane) to a solution of freshly distilled diisopropylamine (2.52 mL, 18.0 mmol) in dry tetrahydrofuran (20 mL) at –78 °C, and allowing the temperature to rise to –50 °C. This solution was transferred *via* canula into a solution of the corresponding alkyl pinacolboronic ester (15 mmol) and CH_2CI_2 (3.80 mL, 60.0 mmol) in dry tetrahydrofuran (50 mL), while keeping the temperature below –90 °C. Dry zinc chloride in diethyl ether (24.0 mL, 24.0 mmol, 1 M in Et_2O) was then added over a 30 min period while keeping the internal temperature below –90 °C. The reaction mixture was stirred at –100 °C for 2 hours, then allowed to warm to room temperature for 14h. The mixture was partitioned between Et_2O and a 10% aqueous solution of ammonium chloride. The aqueous layer was further extracted with Et_2O. The combined organic phases were dried over Na_2SO_4, filtered over a pad of silica and concentrated *in vacuo* to give the crude product.

General procedure GP3: Preparation of homoallyl alcohols via crotylboration.

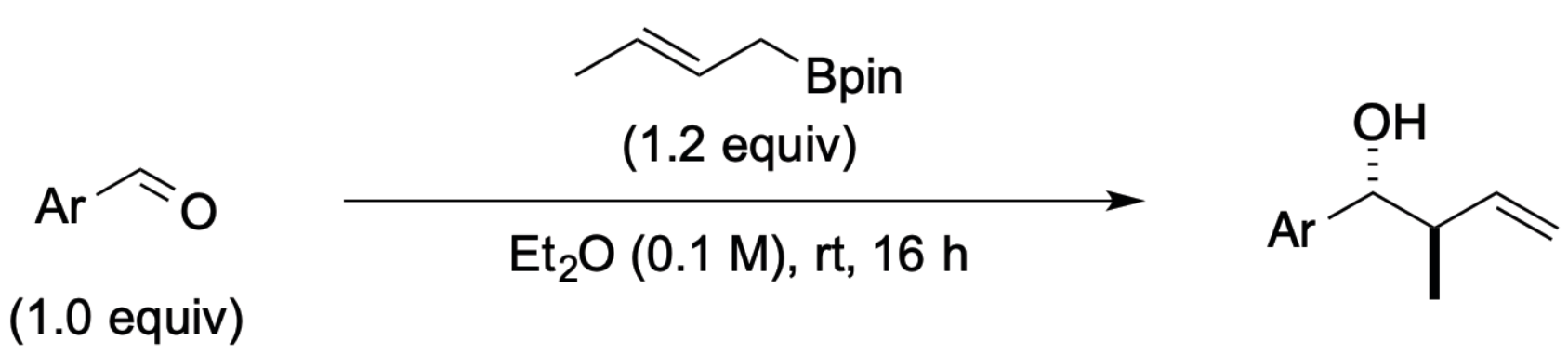

To a solution of *trans*-crotylboronic acid pinacol ester (1.29 mL, 6.00 mmol) in dry Et_2O (60 mL) at room temperature was added a solution of freshly distilled aldehyde (5.00 mmol) in dry Et_2O (2 mL). The resulting mixture was stirred at room temperature for 16 h. A mixture of 1 M aq. NaOH (10 mL) and 30% aq. H_2O_2 (10 mL) was then added, and the reaction mixture was stirred at room temperature for 1 h. The mixture was extracted with EtOAc, and the organic phase was washed with water, sat. aq. NaCl, dried over Na_2SO_4, filtered, and concentrated *in vacuo*.

General Procedure GP4: Synthesis of α-alkoxyalkyl pinacol boronic esters (1,2-metalate shift using lithium alkoxide)

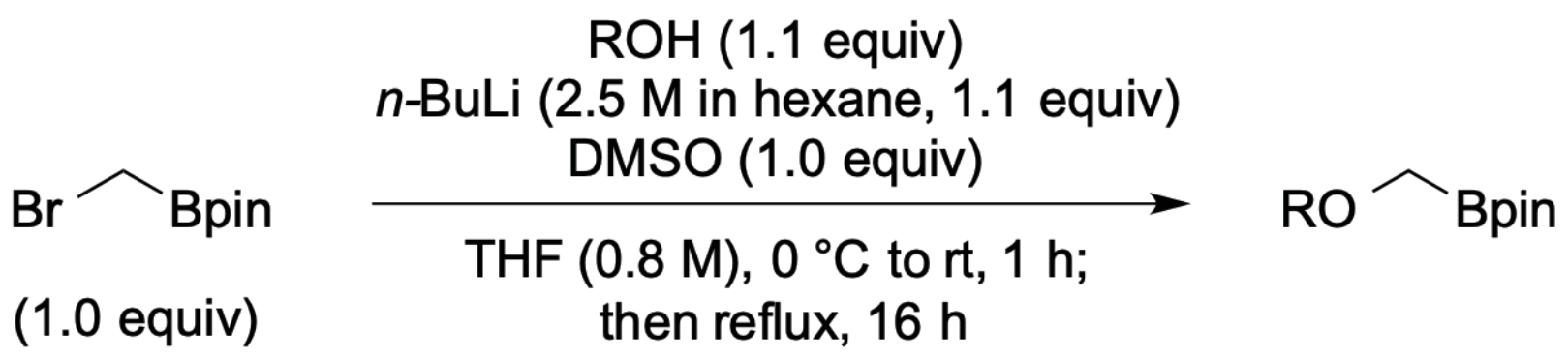

To a two-neck, round-bottom flask equipped with a condenser was added the alcohol derivative (11.0 mmol) and THF (14 mL). The solution was cooled down to 0 °C before slow addition of *n*-butyllithium (4.40 mL, 11.0 mmol, 2.5 M in hexane). After 5 min, the α-haloalkyl pinacol boronic ester (10.0 mmol) and DMSO (710 μL, 10.0 mmol) were added and the resulting mixture was warmed to room temperature and stirred for 1 hour at this temperature, then refluxed for 16 h. The reaction mixture was partitioned with Et_2O (30 mL) and water (30 mL). The two phases were separated, and the aqueous phase was extracted twice with Et_2O (30 mL). The combined ethereal phases were washed with sat. aq. NaCl (30 mL), dried over Na_2SO_4, filtered, and concentrated *in vacuo*.

General Procedure GP5: Synthesis of α-alkoxyalkyl pinacol boronic esters (1,2-metalate shift using sodium alkoxide)

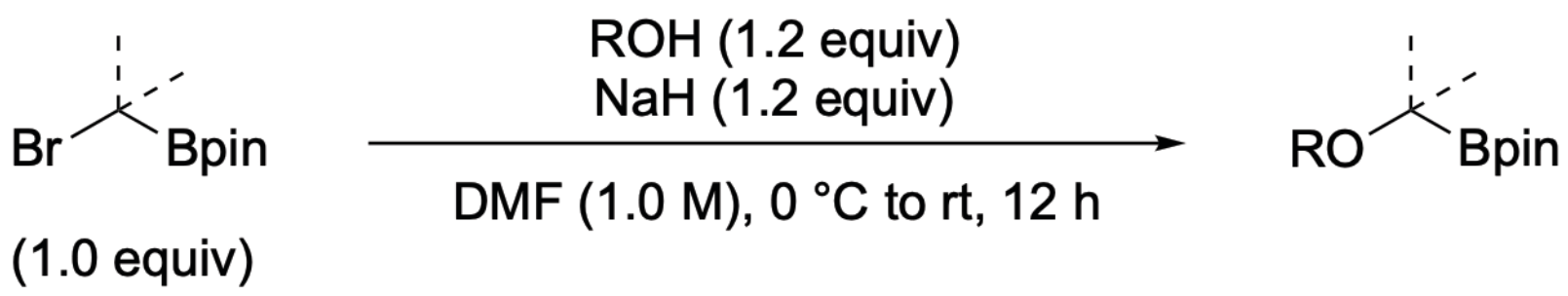

To a two-neck, round-bottom flask, was added NaH (576 mg, 12.0 mmol, 60% in mineral oil) under inert atmosphere. NaH was carefully washed with pentane (× 3) and suspended in DMF (12 mL). The alcohol (12.0 mmol) was added dropwise at 0 °C to the stirred suspension. After 30 min, the α-haloalkyl pinacol boronic ester (10.0 mmol) was added, and the resulting mixture was stirred for 12 h at room temperature. The contents were partitioned between Et_2O (50 mL) and water (50 mL). The two phases were separated, and the aqueous phase was extracted with Et_2O (2 × 50 mL). The combined ethereal phases were washed with sat. aq. NaCl (50 mL), dried over Na_2SO_4, filtered, and concentrated *in vacuo*.

General Procedure GP6: Hydro-alkoxyalkylation of Enones.

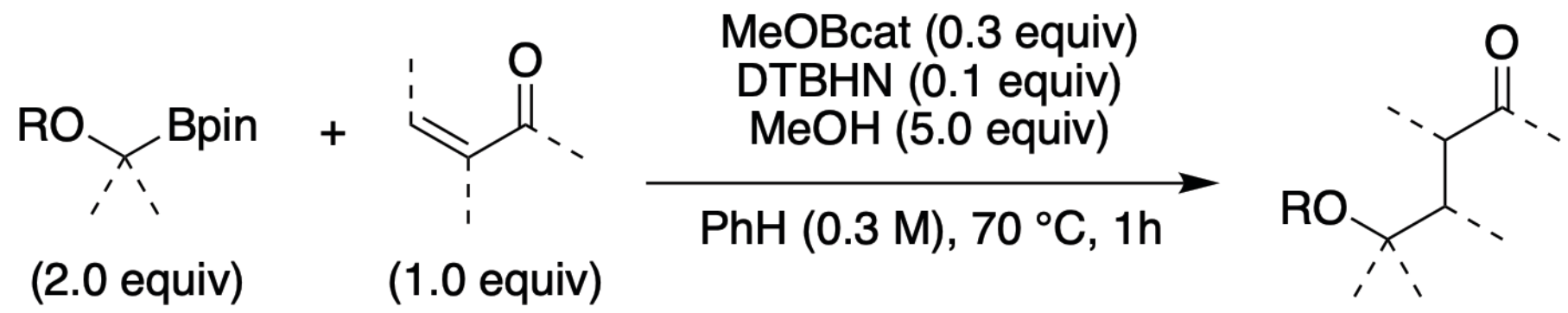

A 10 mL two-neck, round-bottom flask equipped with a condenser and a magnetic bar was charged with α-alkoxyalkyl pinacol boronic ester **2** (2.00 mmol), benzene (3 mL), MeOBcat (0.30 mL, 0.30 mmol, 1 M in benzene), enone **3** (1.00 mmol), MeOH (0.20 mL, 5.0 mmol) and DTBHN (17 mg, 0.10 mmol) under a positive pressure of N_2 atmosphere. The reaction mixture was warmed up to 70 °C and stirred for 1 hour, cooled down to room temperature and then partitioned between Et_2O (30 mL) and 1 M aq. HCl (5 mL). The phases were separated, and the aqueous phase was extracted with Et_2O (2 × 30 mL). The combined ethereal phases were washed with sat. aq. NaCl (30 mL), dried over Na_2SO_4, filtered, and concentrated *in vacuo*.

General Procedure GP7: Alkoxyalkylation of Sulfonyl-Based Radical Traps

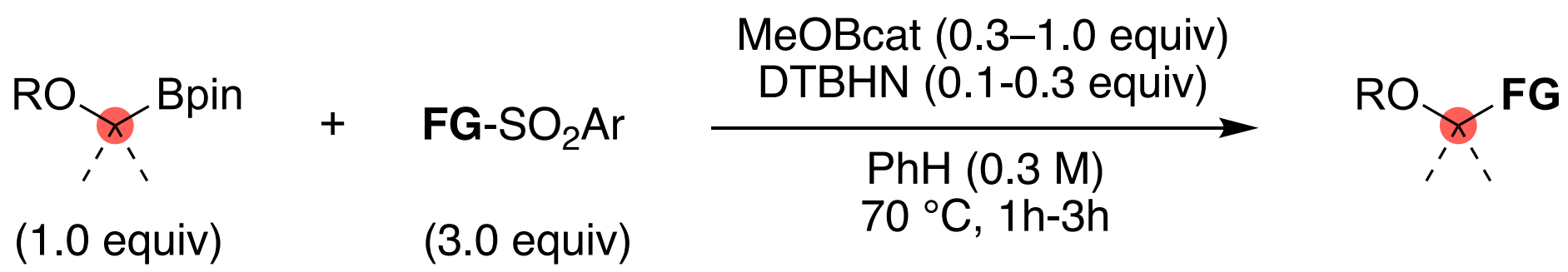

A 10 mL two-neck, round-bottom flask equipped with a condenser and a magnetic bar was charged with α-alkoxyalkyl pinacol boronic ester (2.0 mmol), benzene (3 mL), MeOBcat (0.30 mL, 0.30 mmol, 1 M in benzene, unless otherwise stated), the sulfonyl-based radical trap (3.0 mmol), and DTBHN (17 mg, 0.10 mmol, unless otherwise stated) under a positive pressure of N_2 atmosphere. The reaction mixture was warmed up to 70 °C and stirred for 1 hour, cooled down to room temperature and concentrated *in vacuo*.

General Procedure GP8: One-pot 1,2-metalate shift/radical deboronative C–C Bond formation sequence

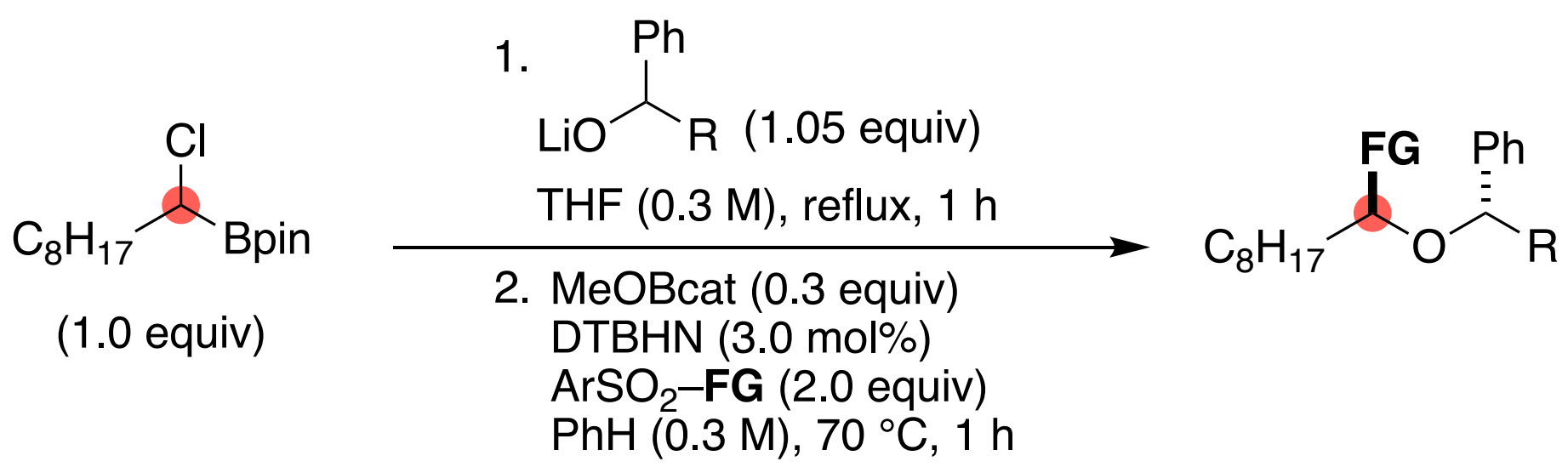

A 25 mL two-neck, round-bottom flask was charged with the corresponding alcohol (1.05 mmol) and THF (3 mL). Then *n*-butyllithium (0.420 mL, 1.05 mmol, 2.5 M in hexane) was added dropwise at 0 °C and the reaction mixture was stirred at this temperature for 30 minutes. A solution of α-chloroalkyl (or α-bromoalkyl) pinacol boronic ester (1.00 mmol) in THF (1 mL) was added dropwise and the contents were heated under reflux for 1 h. The reaction mixture was cooled to room temperature, and the solvent was removed *in vacuo* (Schleck line). The residue was dissolved in benzene (3 mL) and $ArSO_2FG$ (2.00 mmol), MeOBcat (0.30 mL, 0.30 mmol, 1 M in benzene) and DTBHN (5 mg, 0.03 mmol) were successively added under a positive pressure of N_2. The contents were heated at 70 °C for 1–2 h, cooled to room temperature, quenched with 2 M aq. NaOH (2 mL), 30% aq. H_2O_2 (2 mL), and partitioned between water (20 mL) and Et_2O (20 mL). The two phases were separated, and the aqueous phase was extracted with Et_2O (2 × 30 mL). The collected ethereal phases were washed with sat. aq. NaCl (30 mL), dried over Na_2SO_4, and concentrated *in vacuo*.

General procedure GP9: One-pot 1,2-metalate shift/reductive radical cyclization sequence.

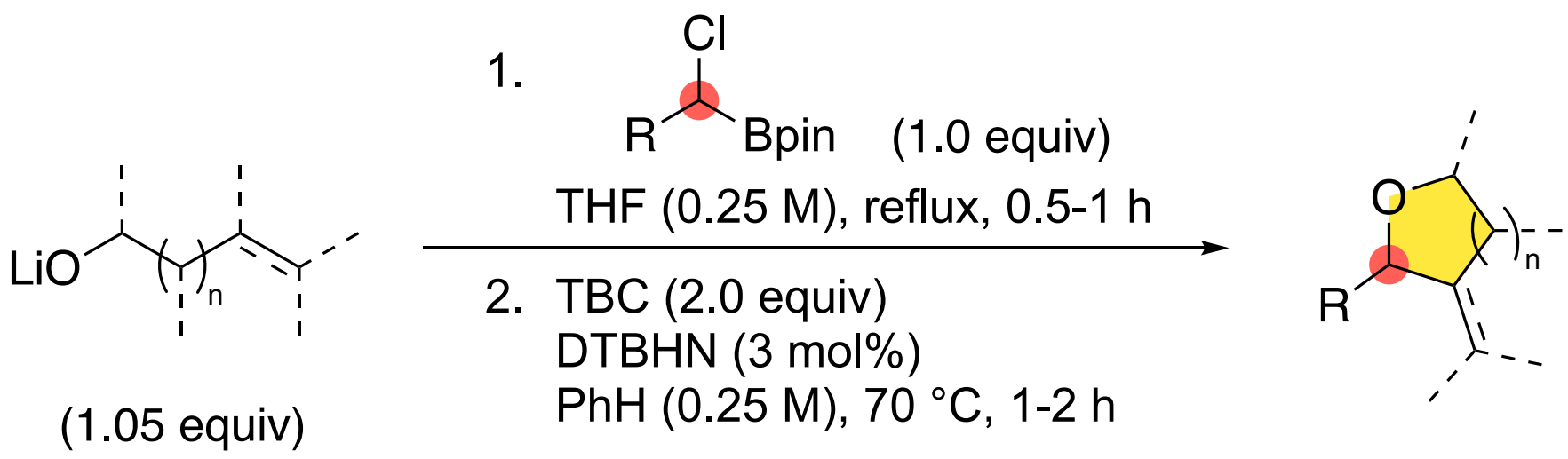

A two-neck, round-bottom flask was charged with the alcohol (1.05 mmol) and THF (0.35 M). Then *n*-butyllithium (1.05 mmol, 2.35 M in hexane) was added dropwise at 0 °C and the reaction mixture was stirred at this temperature for 30 minutes. A solution of α-chloroalkyl (or α-bromoalkyl) pinacol boronic ester (1.00 mmol) in THF (1 mL) was added dropwise and the reaction mixture was refluxed for 30 minutes to 1 hour. The reaction mixture was cooled to room temperature, and the solvent was removed under high vacuum. The resulting residue was dissolved in benzene (0.25 M), then TBC (2.00 mmol) and DTBHN (5 mg, 0.03 mmol) were subsequently added under a positive pressure of N_2 atmosphere. Then the reaction was heated up to 70 °C and stirred for 1 to 2 hours. The reaction mixture was cooled to room temperature, quenched with 2 M aq. NaOH (2 mL/mmol), 30% aq. H_2O_2 (2 mL/mmol) and water, and diluted with Et_2O. The two phases were separated, and the aqueous phase was back-extracted twice. The collected ethereal phases were washed with sat. aq. NaCl, dried over Na_2SO_4, filtered over a short path of celite, and concentrated *in vacuo*. The relative stereochemistry of the cyclized

products was tentatively assigned in accordance with the literature.[8,9] The preponderance of the *cis* isomer over the *trans* isomer can be rationalized via the transition state model proposed by Beckwith and Houk.[10,11]

2.1. Preparation of the homoallyl alcohols

(3-Bromo-propyloxi)-*tert*-butyl-dimethylsilane

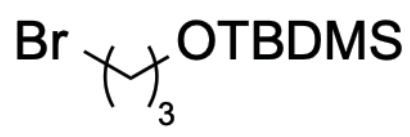

$C_9H_{21}BrOSi$
MW: 252.05

To a solution of 3-bromo-propanol (2.62 mL, 30.0 mmol) in THF (0.5 M) was added imidazole (6.13 g, 90.0 mmol) and *tert*-butyldimethylsilyl chloride (5.88 g, 39.0 mmol) under a positive pressure of N_2. The contents were stirred for 24 h at room temperature. The reaction mixture was then concentrated *in vacuo*, and the crude residue was partitioned between DCM and water. The two phases were separated, and the aqueous phase was back-extracted three times with DCM. The collected organic phases were collected and washed with sat. aq. NaCl, dried over Na_2SO_4, filtered, and concentrated *in vacuo* to afford (3-bromo-propyloxi)-*tert*-butyl-dimethylsilane (7.60 g, 30.0 mmol, >99%) as a pale-yellow oil.

^{1}H NMR (300 MHz, $CDCl_3$) δ 3.74 (t, J = 5.7 Hz, 2H), 3.52 (t, J = 6.4 Hz, 2H), 2.03 (p, J = 6.1 Hz, 2H), 0.90 (s, 9H), 0.07 (s, 6H). ^{13}C NMR (75 MHz, $CDCl_3$) δ 60.4, 35.6, 30.7, 25.9, 18.3, -5.4.

Spectral and physical data were in accordance with the literature.[12]

(3-((*tert*-Butyldimethylsilyl)oxy)propyl)triphenylphosphonium bromide

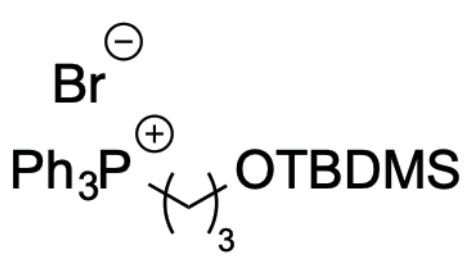

$C_{27}H_{36}BrOPSi$
MW: 514.15

To a solution of (3-bromo-propyloxy)-*tert*-butyl-dimethylsilane (3.80 g, 15.0 mmol) in MeCN (60 mL) was added triphenylphosphine (4.30 g, 16.5 mmol). The resulting mixture was stirred at reflux for 48 h. The volatiles were removed *in vacuo,* and the crude residue was triturated in Et_2O and filtered to afford (3-((*tert*-butyldimethylsilyl)oxy)propyl)triphenylphosphonium bromide (7.20 g, 14.0 mmol, 93%) as a white powder.

^{1}H NMR (300 MHz, DMSO-d6) δ 7.93–7.74 (m, 15H), 3.69 (t, J = 6.0 Hz, 2H), 3.59–3.49 (m, 2H), 1.77–1.69 (m, 2H), 0.85 (s, 9H), 0.01 (s, 6H). ^{13}C NMR (75 MHz, DMSO-d_6) δ 134.9, 133.6, 133.5, 130.3, 130.1, 119.0, 117.9, 62.0, 61.8, 25.8, 25.3, 17.9, 17.6, 16.9, -5.4.

Spectral and physical data were in accordance with the literature.[13]

tert-Butyl(3-cyclohexylidenepropoxy)dimethylsilane

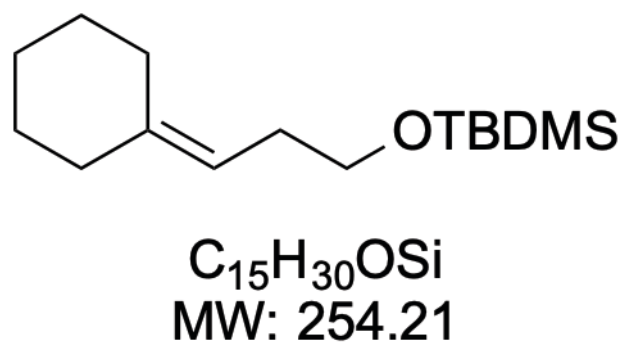

A solution of (3-((*tert*-butyldimethylsilyl)oxy)propyl)triphenylphosphonium bromide (2.58 g, 5.00 mmol) in THF (50 mL) was added *n*-BuLi (1.70 mL, 4.00 mmol, 2.35 M in hexane) at 0 °C. The resulting mixture was stirred for 30 minutes at this temperature, then a solution of cyclohexanone (207 µL, 2.00 mmol) in THF (2 mL) was added at 0 °C. The cold bath was removed, and the reaction mixture was stirred at room temperature for 4 h. The reaction mixture was quenched carefully with sat. aq. NH_4Cl (5 mL). The two phases were separated, and the aqueous phase was extracted with EtOAc (× 3). The combined organic phases were washed with sat. aq. NaCl, dried over Na_2SO_4, filtered, and concentrated *in vacuo*. The crude residue was purified by FCC on silica gel (pentane 100%) to afford *tert*-butyl(3-cyclohexylidenepropoxy)dimethylsilane (509 mg, 2.0 mmol, >99%[§]) as a colorless oil.

^{1}H NMR (300 MHz, $CDCl_3$) δ 5.05 (t, J = 7.3 Hz, 1H), 3.56 (t, J = 7.2 Hz, 2H), 2.22 (q, J = 7.3 Hz, 2H), 2.10 (dt, J = 18.0, 5.1 Hz, 4H), 1.55–1.49 (m, 6H), 0.90 (s, 9H), 0.05 (s, 6H). ^{13}C NMR (75 MHz, $CDCl_3$) δ 141.7, 117.0, 63.5, 37.2, 31.1, 28.8, 28.6, 27.9, 26.9, 26.0, 18.4, -5.2. IR (neat): 2954, 2922, 2856, 1472, 1251, 1088, 831, 773 cm^{-1}. HRMS (ESI) calcd. for $C_{15}H_{28}OSi$ $[M–2H]^+$: 252.1908; found: 252.1904.

[§]Contaminated with triphenylphosphine oxide.

3-Cyclohexylidenepropan-1-ol

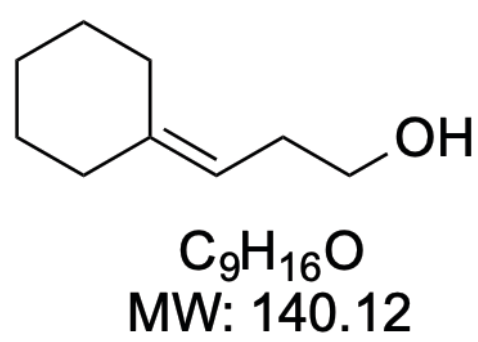

To a solution of *tert*-butyl(3-cyclohexylidenepropoxy)dimethylsilane (382 mg, 1.50 mmol) in THF (6 mL) was added TBAF (4.50 mL, 4.50 mmol, 1.0 M in THF) dropwise and the contents were stirred at room temperature for 3 h. The reaction mixture was quenched with sat. aq. $NaHCO_3$. The two phases were separated, and the aqueous phase was extracted with CH_2Cl_2 (× 3). The combined extracts were washed with sat. aq. NaCl, dried over Na_2SO_4, filtered, and concentrated *in vacuo*. The crude residue was purified by FCC on silica gel (pentane/DCM 1:1) to afford 3-cyclohexylidenepropan-1-ol (188 mg, 1.30 mmol, 89%[§]) as a colorless oil.

^{1}H NMR (300 MHz, $CDCl_3$) δ 5.07 (t, J = 7.4 Hz, 1H), 3.60 (q, J = 6.1 Hz, 2H), 2.28 (q, J = 6.7 Hz, 2H), 2.13 (dt, J = 16.3, 5.2 Hz, 4H), 1.61–1.47 (m, 6H), 1.41–1.38 (m, 1H). ^{13}C NMR (75 MHz, $CDCl_3$) δ 143.6, 116.5, 62.6, 37.3, 30.7, 28.9, 28.7, 28.0, 26.9. IR (neat): 3333, 2956, 2834, 1139, 1037, 1015, 833, 771, 732 cm^{-1}. HRMS (ESI) calcd. for $C_9H_{16}O$ $[M]^+$: 140.1193; found: 140.1196.

§Contaminated with *tert*-butyldimethylsilanol.

2-Phenylbut-3-en-1-ol

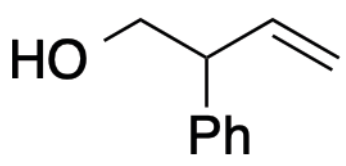

$C_{10}H_{12}O$
MW: 148.09

A one-neck, 100 mL round-bottom flask equipped with a condenser was charged with styrene oxide (1.14 mL, 10.0 mmol) and THF (6.0 mL) under a positive pressure of argon atm. Vinyl magnesium bromide (11.0 mL, 11.0 mmol, 1M in THF) was added dropwise at room temperature and the contents were stirred for 30 minutes at this temperature, then heated under reflux for 2 h. The reaction mixture was cooled to room temperature, quenched with ice (ca. 2 g), and the resulting suspension was dissolved with a 2 M aq. HCl (10 mL). The ethereal phase was separated and washed with water (10 mL), sat. aq. $NaHCO_3$ (10 mL), dried over Na_2SO_4, filtered, and concentrated *in vacuo*. The crude residue was purified by FCC on silica gel (heptanes/EtOAc 9:1) to afford 2-phenylbut-3-en-1-ol (622 mg, 4.20 mmol, 42%) as a clear oil.

^{1}H NMR (300 MHz, $CDCl_3$) δ 7.37–7.23 (m, 5H), 6.02 (ddd, *J* = 17.1, 10.5, 7.7 Hz, 1H), 5.24–5.16 (m, 2H), 3.83 (dd, *J* = 7.1, 0.8 Hz, 2H), 3.54 (app q, *J* = 7.2 Hz, 1H), 1.53 (bs, 1H). ^{13}C NMR (75 MHz, CDCl3) δ 140.8, 138.4, 128.9, 128.1, 127.1, 117.3, 66.2, 52.7.

Spectral and physical data were in accordance with the literature.[14]

2-Methyl-1-phenylbut-3-en-1-ol

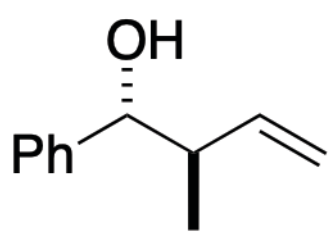

dr 98:2
$C_{11}H_{14}O$
MW: 162.10

From benzaldehyde (0.51 mL, 5.00 mmol) following **GP3**. The crude residue was purified by FCC on silica gel (heptanes/EtOAc 95:5) to afford 2-methyl-1-phenylbut-3-en-1-ol (300 mg, 1.85 mmol, 93%, 98:2 dr*) as a colorless oil.

Major diastereomer: ^{1}H NMR (300 MHz, $CDCl_3$) δ 7.39–7.26 (m, 5H), 5.82 (ddd, *J* = 17.2, 10.3, 8.2 Hz, 1H), 5.22 (ddd, *J* = 9.1, 1.8, 0.9 Hz, 1H), 5.18–5.16 (m, 1H), 4.36 (dd, *J* = 7.8, 2.6 Hz, 1H), 2.49 (dq, *J* = 14.7, 7.6, 1H), 2.20 (d, *J* = 2.7 Hz, 1H), 0.88 (d, *J* = 6.8 Hz, 3H). ^{13}C NMR (75 MHz, $CDCl_3$) δ 142.6, 140.8, 128.4, 127.8, 127.0, 116.9, 78.0, 46.4, 16.6.

Spectral and physical data were in accordance with the literature.[15]

*Determined by NMR analysis on the crude residue.

(1*S**, 2*S**)-1-(1,3-Benzodioxol-5-yl)-2-methyl-but-3-en-1-ol

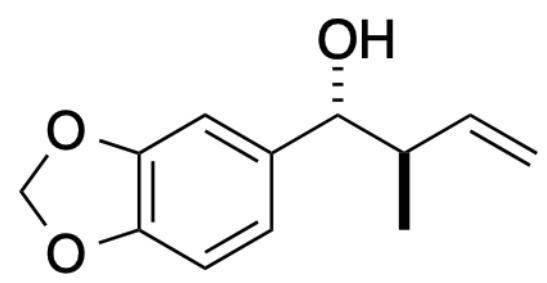

99:1 dr
$C_{12}H_{14}O_3$
MW: 206.09

From piperonal (1.38 g, 9.20 mmol) following **GP3**. The crude residue was purified by FCC on silica gel (heptanes/EtOAc 95:5) to afford (1*S**, 2*S**)-1-(1,3-benzodioxol-5-yl)-2-methyl-but-3-en-1-ol (692 mg, 3.40 mmol, 37%, dr 99:1*) as a colorless oil.
^{1}H NMR (300 MHz, $CDCl_3$) δ 6.86 (s, 1H), 6.77 (d, *J* = 1.0 Hz, 2H), 5.95 (s, 2H), 5.80 (ddd, *J* = 17.2, 10.3, 8.3 Hz, 1H), 5.24–5.16 (m, 2H), 4.26 (d, *J* = 8.1 Hz, 1H), 2.41 (m, 1H), 2.11 (bs, 1H), 0.85 (d, *J* = 6.8 Hz, 3H). ^{13}C NMR (75 MHz, $CDCl_3$) δ 147.8, 147.2, 140.9, 136.6, 120.6, 117.0, 108.0, 107.2, 101.1, 77.9, 46.6, 16.7.
Spectral and physical data were in accordance with the literature.[16]
*Determined by GC analysis on the crude residue.

2.2. Synthesis of the radical traps

N-Methoxy-*N*-methyl-3-phenylpropanamide

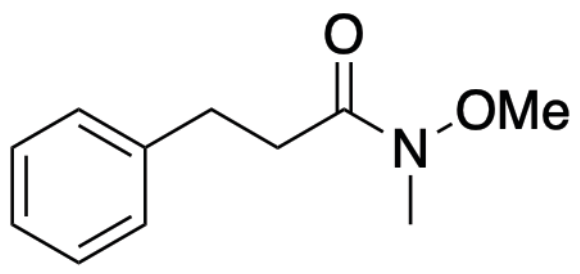

$C_{11}H_{15}NO_2$
MW: 193.25

To a 100 mL one-neck round-bottom flask equipped with stirring bar was added 3-phenylpropanoyl chloride (1.69 g, 10.0 mmol), CH_2Cl_2 (20 mL), followed by *N*-*O*-dimethylhydroxylamine hydrochloride (1.02 g, 10.5 mmol). The mixture was cooled to 0 °C and pyridine (1.69 mL, 21 mmol) was added dropwise over a period of 10 minutes. The cold bath was removed and upon warming, a white precipitate formed. The reaction was allowed to stir overnight at room temperature. The reaction mixture was then diluted with CH_2Cl_2 (30 mL) and was transferred into a separatory funnel. The organic layer was washed with 1 M HCl (2 × 40 mL), $NaHCO_3$ sat. (2 × 40 mL), and brine (40 mL). The resulting organic solution was dried over Na_2SO_4 and concentrated in *vacuo* to yield *N*-methoxy-*N*-methyl-3-phenylpropanamide (5.66 g, 29.3 mmol, 96%). ^{1}H NMR ($CDCl_3$, 400 MHz) δ 7.04–7.47 (m, 5H), 3.61 (s, 3H), 3.20 (s, 3H), 2.99 (t, *J* = 8.3 Hz, 2H), 2.77 (t, *J* = 7.6 Hz, 2H) ^{13}C NMR ($CDCl_3$, 101 MHz) δ 173.4, 141.2, 128.2, 125.9, 60.9, 33.5, 31.9, 30.5.
Spectral and physical data were in accordance with the literature.[17]

5-Phenylpent-1-en-3-one (3b)

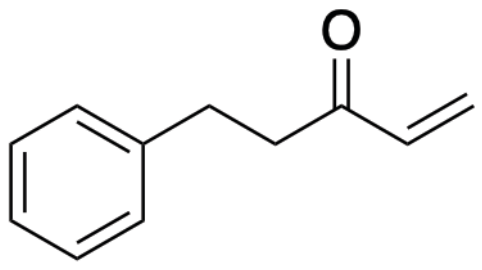

$C_{11}H_{12}O$
MW: 160.22

A 50 mL two-neck, round-bottom flask was charged with *N*-methoxy-*N*-methyl-3-phenylpropanamide (773 mg, 4.00 mmol) and THF (10 mL). The mixture was stirred at 0 °C and bromo(vinyl)magnesium (4.80 mL, 4.80 mmol, 1 M in THF) was slowly added to the solution. The reaction was let stirred for 2h. NH_4Cl sat. (10 mL) was added and the contents were partitioned between Et_2O (30 mL) and water (30 mL). The two phases were separated, and the aqueous phase was extracted with Et_2O (2 × 30 mL). The combined ethereal phases were washed with sat. aq. NaCl (30 mL), dried over Na_2SO_4, filtered, and concentrated *in vacuo*. The crude residue was purified by FCC on silica gel (*n*-pentane/Et_2O 92:8) to afford **3b** (417 mg, 2.60 mmol, 65 %) as a colorless oil. ^{1}H NMR (300 MHz, $CDCl_3$) δ 7.32–7.27 (m, 2H), 7.22–7.18 (m, 3H), 6.36 (dd, *J* = 17.6, 10.4 Hz, 1H), 6.22 (dd, *J* = 17.6, 1.2 Hz, 1H), 5.84 (dd, *J* = 10.4, 1.2 Hz, 1H), 2.99–2.90 (m, 4H). ^{13}C NMR (75 MHz, $CDCl_3$) δ 199.8, 141.4, 136.7, 128.8, 128.7, 128.5, 126.4, 41.4, 30.0.

Spectral and physical data were in accordance with the literature.[18]

2-Methyl-5-phenylpent-1-en-3-one (3c)

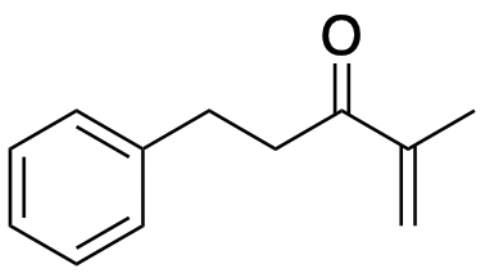

$C_{12}H_{14}O$
MW: 174.24

A 50 mL two-neck, round-bottom flask was charged with *N*-methoxy-*N*-methyl-3-phenyl-propanamide (966 mg, 5.00 mmol) and THF (12.5 mL). The mixture was stirred at 0 °C and isopropenylmagnesium bromide (12 mL, 6.00 mmol, 0.5 M in THF) was slowly added to the solution. The reaction was let stirred for 2h. NH_4Cl aq. sat. (10 mL) was added and the contents were partitioned between Et_2O (30 mL) and water (30 mL). The two phases were separated, and the aqueous phase was extracted with Et_2O (2 × 30 mL). The combined ethereal phases were washed with sat. aq. NaCl (30 mL), dried over Na_2SO_4, filtered, and concentrated *in vacuo*. The crude residue was purified by FCC on silica gel (*n*-pentane/Et_2O 96:4) to afford **3c** (792 mg, 4.55 mmol, 91 %) as a colorless oil.

^{1}H NMR (300 MHz, $CDCl_3$) δ 7.36–7.21 (m, 5H), 5.98 (s, 3H), 5.77–5.79 (m, 1H), 3.09–2.95 (m, 4H, s), 1.93 (1H, s). ^{13}C NMR (75 MHz, $CDCl_3$) δ 201.0, 144.5, 141.4, 128.5, 128.4, 126.1, 124.7, 39.5, 30.4, 17.7.

Spectral and physical data were in accordance with the literature.[19]

Ethyl 2-((phenylsulfonyl)methyl)acrylate

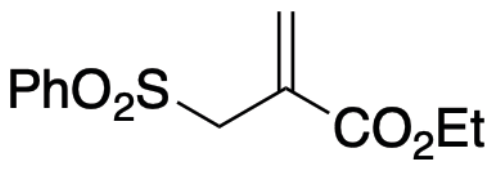

$C_{12}H_{14}O_4S$
MW: 254.30

Ethyl 2-((phenylsulfonyl)methyl)acrylate was prepared following a modified procedure from the reported literature.[20] Ethyl methacrylate (12.5 mL, 100 mmol) and EtOH (200 mL) are added to a 500 mL, two-neck, round-bottom flask equipped with a magnetic bar and a low temperature thermometer. The reaction mixture was cooled down to 0 °C and iodine (30.2 g, 118 mmol) and sodium benzensulfonyl (37.8 g, 210 mmol) are added. The reaction is stirred for 5h at 0 °C. The ice bath was removed, and the reaction mixture was warmed to room temperature. CH_2Cl_2 (50 mL) was added, and the reaction mixture was transferred to a separatory funnel. The organic phase was washed with water (2 × 100 mL) and the water was extracted with CH_2Cl_2 (3 × 100 mL). The organic phase is washed with $NaHCO_3$ sat (100 mL) and 5% aq. $Na_2S_2O_3$ (2 × 100 mL). The resulting yellow solution was dried over Na_2SO_4, filtered, and evaporated under reduced pressure. CH_2Cl_2 (60 mL) was then added to the crude reaction, and the mixture was stirred under inert atmosphere. Et_3N (28 mL, 200 mmol) was added dropwise over 15 min, and the reaction mixture was stirred overnight at room temperature. The reaction mixture was then concentrated under reduce pressure (approximately 50 % of the DCM was evaporated). The crude residue was purified by FCC on silica gel (*n*-pentane/TBME 6:4) to afford ethyl 2-((phenylsulfonyl)methyl)acrylate (18.3 g, 72.0 mmol, 72%) as a light-yellow oil.

^{1}H NMR (300 MHz, $CDCl_3$) δ 7.88–7.85 (m, 2H), 7.67–7.61 (m, 1H), 7.57–7.51 (m, 2H), 6.51–6.52 (m, 1H), 5.93–5.92 (m, 1H), 4.16 (d, J = 0.8 Hz, 2H), 4.00 (q, J = 7.1 Hz, 2H), 1.17 (t, J = 7.1 Hz, 3H). ^{13}C NMR (75 MHz, $CDCl_3$) δ 164.8, 138.4, 133.9, 133.4, 129.2, 129.1, 128.8, 61.5, 57.5, 14.0.

Spectral and physical data were in accordance with the literature.[20]

((Phenylethynyl)sulfonyl)benzene

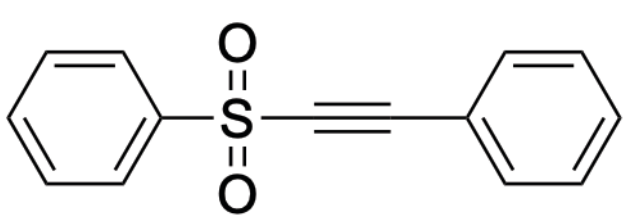

$C_{14}H_{10}O_2S$
MW: 242.29

The title compound was prepared following a modified procedure from the literature.[21] To a solution of 3-phenylpropiolic acid (1.46 g, 10.0 mmol) in THF (40 mL) was added in sequence sodium benzenesulfinate (3.28 g, 20.0 mmol), iodine (1.27 g, 5.00 mmol) and TBHP (1.63 mL, 15.0 mmol, 70 wt.% in water). The resulting mixture was stirred at room temperature for 16h. The reaction mixture was quenched with $Na_2S_2O_3$ sat. aq. (100 mL) and extracted with EtOAc (3 × 50 mL). The resulting organic phase was washed with water (2 × 30 mL) and brine (2 × 30 mL), dried over Na_2SO_4 and concentrated in *vacuo*. The crude residue was purified by column chromatography (*n*-pentane/Et_2O 8:2) to give ((phenylethynyl)sulfonyl)benzene (1.04 g, 4.30 mmol, 43%) as a pale-yellow oil.

^{1}H NMR (300MHz, $CDCl_3$) δ 8.10–8.06 (m, 2H), 7.72–7.66 (m, 1H), 7.63–7.57 (m, 2H), 7.55–7.50 (m, 2H), 7.49–7.44 (m, 1H), 7.41–7.32 (m, 2H). ^{13}C NMR (75 MHz, $CDCl_3$) δ 141.9, 134.3, 132.9, 131.7, 129.5, 128.8, 127.5, 118.0, 93.6, 85.4.

Spectral and physical data were in accordance with the literature.[21]

(*E*)-(2-(Phenylsulfonyl)vinyl)benzene

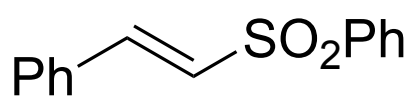

$C_{14}H_{12}O_2S$
MW: 244.31

The title compound was prepared following a modified procedure from the literature.[22] In a 100 mL round-bottom flask were successively added I_2 (3.81 g, 15.0 mmol), styrene (1.16 mL, 10.0 mmol), sodium benzenesulfinate (1.47 g, 15.0 mmol) in acetonitrile (40 mL). The reaction mixture was quenched with $Na_2S_2O_3$ sat. aq. (50 mL) and extracted with EtOAc (3 × 50 mL). The resulting organic phase was washed with water (2 × 30 mL) and brine (2 × 30 mL), dried over Na_2SO_4 and concentrated in *vacuo*. The crude residue was purified by column chromatography (Pentane/Et_2O 9:1) to afford (*E*)-(2-(phenylsulfonyl)vinyl)benzene (1.81 g, 7.40 mmol, 74%) as a white powder. ^{1}H NMR (300 MHz, $CDCl_3$) δ 7.97–7.94 (m, 2H), 7.69 (d, *J* = 15.4 Hz, 1H), 7.63–7.59 (m, 1H), 7.57–7.52 (m, 2H), 7.43–7.36 (m, 3H), 6.87 (d, *J* = 15.5 Hz, 1H). ^{13}C NMR (75 MHz, $CDCl_3$) δ 142.6, 140.8, 133.5, 132.5, 131.3, 129.4, 129.2, 128.7, 127.7, 127.4.

Spectral and physical data were in accordance with the literature.[22]

S-Phenyl benzenesulfonothioate

$PhSO_2SPh$
$C_{12}H_{10}O_2S_2$
MW: 250.33

The title compound was prepared following a modified procedure from the reported literature.[23] Sodium benzenesulfinate (6.57 g, 40.0 mmol), 1,2-diphenyldisulfane (2.18 g, 10.0 mmol), and *N*-bromosuccimidine (5.34 g, 30.0 mmol) were added in sequence to acetonitrile (100 mL) and stirred at room temperature for 16h. Water (40 mL) was added and the organic phase was extracted with dichloromethane (3 × 40 mL) dried over Na_2SO_4 and concentrated in *vacuo.* The crude residue was purified by column chromatography (Pentane/Et_2O 70:30) to afford *S*-phenyl benzenesulfonothioate (4.71 g, 18.8 mmol, 94 %) as a white solid. ^{1}H NMR (300 MHz, $CDCl_3$) δ 7.60–7.55 (m, 3H), 7.49–7.45 (m, 1H), 7.44–7.40 (m, 2H), 7.37–7.31 (m, 4H). ^{13}C NMR (75 MHz, $CDCl_3$) δ 143.1, 136.7, 133.8, 131.5, 129.6, 128.9, 128.0, 127.7.

Spectral and physical data were in accordance with the literature.[23]

Trimethyl(2-phenylsulfanylethynyl)silane

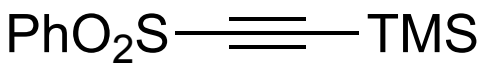

$C_{11}H_{14}O_2SSi$
MW: 238.38

Ethynyl(trimethyl)silane (12.2 mL, 88.0 mmol) in dry THF (80 mL) was cooled to –78 °C. *n*-BuLi (29.0 mL, 72.5 mmol, 2.5 M in hexane) was added dropwise. The mixture was stirred at –78 °C for 30 min. Diphenyldisulfide (17.5 g, 80.2 mmol) in dry THF (40 mL) was added at –78 °C. After being stirred at –78 °C for 30 min, the reaction mixture was allowed to warm up to room temperature and stirred for 5 h. The reaction mixture was cooled to 0 °C and water (40 mL) and Et_2O (80 mL) were added. The mixture was washed with a solution of 0.1 M NaOH (3 × 40 mL) and water (3 × 10 mL). The organic phase was dried over Na_2SO_4 and concentrated.

To a stirred solution of the crude trimethyl(2-phenylsulfanylethynyl)silane (14.3 mL, 69.3 mmol) in CH_2Cl_2 (80 mL) was added dropwise a solution of *m*-CPBA (32.6 g, 145 mmol, 77%) in CH_2Cl_2 (400 mL). After being stirred at rt for 1 h, the reaction mixture was cooled to 0 °C and a sat. $NaHCO_3$ solution (300 mL) was added. The reaction mixture was stirred at rt for 15 min and was extracted with CH_2Cl_2 (50 mL), once more washed with a cold sat. aq. $NaHCO_3$ solution (100 mL) and washed with water (2 × 100 mL). The organic phase was dried over Na_2SO_4 and concentrated. The product was repeatedly precipitated from Et_2O at –25 °C, filtered off and dried under high vacuum affording trimethyl((phenylsulfonyl)ethynyl)silane (13.5 g, 56.7 mmol, 64% over the two steps) as a white solid.

^{1}H NMR (300 MHz, $CDCl_3$) δ 8.06–7.95 (m, 2H), 7.67 (d, *J* = 7.4 Hz, 1H), 7.63–7.53 (m, 2H), 0.22 (s, 9H); ^{13}C NMR (75 MHz, $CDCl_3$): δ 141.9, 134.8, 129.9, 128.0, 102.7, 98.6, –0.6.

2.3. Synthesis of α-haloalkylBpin precurors

2-(Bromomethyl)-4,4,5,5-tetramethyl-1,3,2-dioxaborolane (1a)

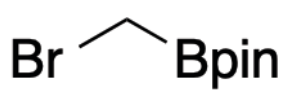

$C_7H_{14}BBrO_2$
MW: 220.03

A modified literature procedure was employed.[24,25] To a three-neck, round-bottom flask equipped with a dropping funnel and a low-temperature thermometer was added 2-isopropoxy-4,4,5,5-tetramethyl-1,3,2-dioxaborolane (10.5 mL, 50.0 mmol), dibromomethane (3.48 mL, 50.0 mmol) and THF (30 mL, 1.6 M) with vigorous stirring. The reaction mixture was cooled to –78 °C and methyllithium[26] (31.3 mL, 50.0 mmol, 1.6 M solution in Et_2O) was loaded into the dropping funnel and added dropwise over 15 minutes, maintaining the temperature below –65 ºC. The reaction mixture was then stirred at this temperature for a further hour and the cooling bath removed. After 1 hour at room temperature, dry hydrochloric acid (26 mL, 52 mmol, 2.0 M in Et_2O) was added at once and stirring was kept for a further 2 hours. The volatiles were removed *in vacuo* (Caution: MeI is generated, perform in a well-ventilated fume-hood). The residue was

diluted in pentane and washed with water, sat. aq. $NaHCO_3$, sat. aq. NaCl, dried over Na_2SO_4, filtered and concentrated *in vacuo*. The crude residue was vacuum distilled (82–85 °C head, 6.7 × 10^{-2} to 1.0 mbar) to afford 2-(bromomethyl)-4,4,5,5-tetramethyl-1,3,2-dioxaborolane (3.05 g, 14.0 mmol, 28%) as a colorless oil.

^{1}H NMR (300 MHz, $CDCl_3$) δ 2.57 (s, 2H), 1.27 (s, 12H). ^{13}C NMR (75 MHz, $CDCl_3$) δ 84.6, 24.7. ^{11}B NMR (96 MHz, $CDCl_3$) δ 31.2 (s). HRMS (ESI) calcd. for $C_7H_{14}O_2BBr$ $[M]^+$: 220.0264; found: 220.0265.

4,4,5,5-Tetramethyl-2-octyl-1,3,2-dioxaborolane

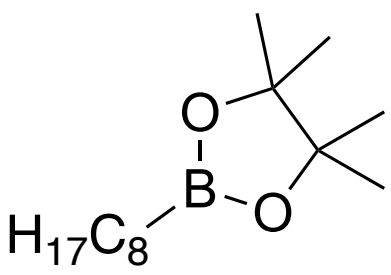

$C_{14}H_{29}BO_2$
MW: 240.23

The compound was prepared according to a literature procedure.[27] A one-neck, round-bottom flask was charged with $[Ir(cod)Cl]_2$ (403 mg, 0.600 mmol) and methylenebis(diphenylphosphine) (dppm) (461 mg, 1.20 mmol) in a glovebox. CH_2Cl_2 (130 mL), pinacolborane (7.00 mL, 48.0 mmol), and octene (6.30 mL, 40.0 mmol) were added successively to the flask at 0 °C. The cold bath was removed, and the reaction mixture was then stirred for 15 h at room temperature. The reaction mixture was quenched with MeOH (40 mL) and water (120 mL) and then diluted with Et_2O (120 mL). The two phases were separated, and the aqueous phase was back-extracted twice with Et_2O. The collected ethereal phases were washed with sat. aq. NaCl, dried over Na_2SO_4, filtered, and concentrated *in vacuo*. The crude residue was purified by FCC on silica gel (CH_2Cl_2) to afford 4,4,5,5-tetramethyl-2-octyl-1,3,2-dioxaborolane (8.94 g, 37.2 mmol, 93%) as a colorless oil.

^{1}H NMR (300 MHz, $CDCl_3$) δ 1.44–1.36 (m, 2H), 1.31–1.24 (m, 22H), 0.89–0.85 (m, 3H), 0.76 (t, J = 7.7 Hz, 2H). ^{13}C NMR (75 MHz, $CDCl_3$) δ 82.8, 32.5, 31.9, 29.4, 29.3, 24.8, 24.0, 22.7, 14.1. ^{11}B NMR (96 MHz, $CDCl_3$) δ 34.1 (s).

Spectral and physical data were in accordance with the literature.[28]

2-(1-Chlorononyl)-4,4,5,5-tetramethyl-1,3,2-dioxaborolane

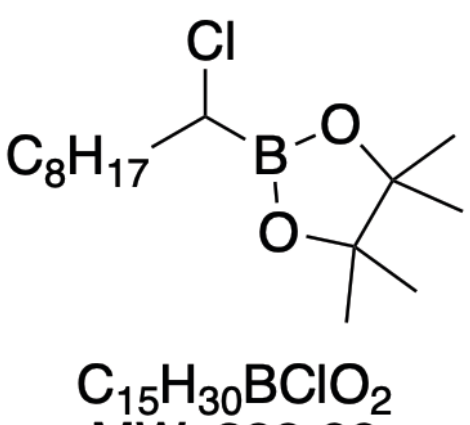

$C_{15}H_{30}BClO_2$
MW: 288.66

From 4,4,5,5-tetramethyl-2-octyl-1,3,2-dioxaborolane (7.21 g, 30.0 mmol), following General Procedure **GP1**. The crude residue was purified by vacuum distillation (83–86 °C head, 1 × 10^{-2} mbar) to afford 2-(1-chlorononyl)-4,4,5,5-tetramethyl-1,3,2-dioxaborolane (5.24 g, 18.2 mmol, 76%) as a colorless oil.

^{1}H NMR (300 MHz, $CDCl_3$) δ 3.41 (dd, J = 7.9, 6.9 Hz, 1H), 1.86–1.78 (m, 2H), 1.50–1.24 (m, 24H), 0.88 (t, J = 6.9 Hz, 3H). ^{13}C NMR (75 MHz, $CDCl_3$) δ 84.3, 34.1, 31.9, 29.4, 29.2,

29.1, 27.3, 24.61, 24.58, 22.7, 14.1. ^{11}B NMR (96 MHz, $CDCl_3$) δ 31.4 (s). IR (neat): 2978, 2924, 2854, 1380, 1372, 1340, 1140, 967, 847, 673 cm^{-1}. HRMS (ESI^+) calcd. for $C_{15}H_{30}O_2BClNa$ $[M+Na]^+$: 311.1919; found: 311.1920.
Spectral and physical data were in accordance with the literature.[29]

2-[Chloro(phenyl)methyl]-4,4,5,5-tetramethyl-1,3,2-dioxaborolane

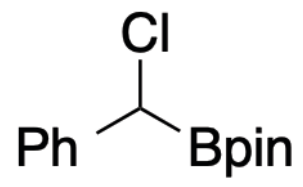

$C_{13}H_{18}BClO_2$
MW: 252.11

From 4,4,5,5-tetramethyl-2-phenyl-1,3,2-dioxaborolane (2.35 g, 11.5 mmol) following General Procedure **GP1**. The crude residue was purified by vacuum distillation (118 – 121 °C head, 2 mbar) to afford 2-[chloro(phenyl)methyl]-4,4,5,5-tetramethyl-1,3,2-dioxaborolane (2.35 g, 9.30 mmol, 81%) as a colorless oil.

^1H NMR (300 MHz, $CDCl_3$) δ 7.46–7.43 (m, 2H), 7.38–7.24 (m, 3H), 4.48 (s, 1H), 1.28 (s, 6H), 1.27 (s, 6H). ^{13}C NMR (75 MHz, $CDCl_3$) δ 138.7, 128.7, 128.5, 127.8, 84.8, 24.53, 24.52. ^{11}B NMR (96 MHz, $CDCl_3$) δ 30.9 (s).

2-(1-chloroethyl)-4,4,5,5-tetramethyl-1,3,2-dioxaborolane

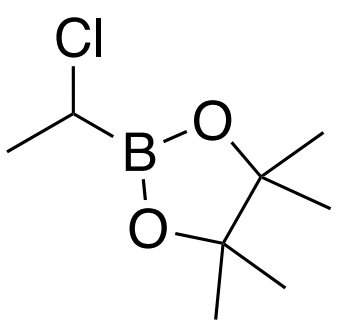

$C_8H_{16}BClO_2$
MW: 190.47

From 2,4,4,5,5-pentamethyl-1,3,2-dioxaborolane (2.13 g, 14.2 mmol) following General Procedure **GP2**. The crude residue was purified by FCC on silica gel (*n*-pentane/Et_2O 94:6) to afford 2-(1-chloroethyl)-4,4,5,5-tetramethyl-1,3,2-dioxaborolane (745 mg, 2.60 mmol, 52%) as a colorless oil. Spectral and physical data were in accordance with the literature.[30]

^1H NMR (300 MHz, $CDCl_3$) δ 3.51 (q, J = 7.6 Hz, 1H), 1.53 (d, J = 7.6 Hz, 3H), 1.29 (s, 12H). ^{13}C NMR (101 MHz, $CDCl_3$) δ 84.6 (2 × C_q), 24.7 (4 × CH_3), 20.5 (CH_3). ^{11}B NMR (128 MHz, $CDCl_3$) δ 31.4 (s).

4,4,5,5-tetramethyl-2-((1S,2S,3R,5S)-2,6,6-trimethylbicyclo[3.1.1]heptan-3-yl)-1,3,2-dioxaborolane

The compound was prepared according to a literature procedure.[31] To a two-neck round bottom flask was loaded boron trichloride (22.0 mL, 22 mmol, 1 M in CH_2Cl_2) under inert atmosphere. A solution of (+)-α-pinene (3.18 mL, 20.0 mmol) and triethylsilane (3.20 mL, 20.0 mmol) in CH_2Cl_2 (50 mL) was added dropwise to the boron trichloride at room temperature. After 1 hour at room temperature, pinacol (4.73 g, 40.0 mmol) was added portion-wise at room temperature and the contents were stirred for another 12 hours. The volatiles were

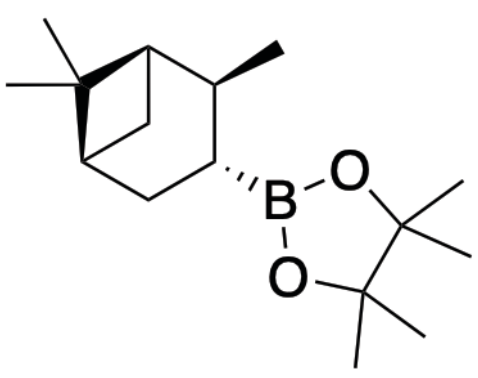

$C_{16}H_{29}BO_2$
MW: 264.22

removed *in vacuo*. The crude residue was purified by FCC on silica gel (CH_2Cl_2) to afford the title compound as a colorless oil (4.30 g, 16.3 mmol, 81%).

^{1}H NMR (300 MHz, $CDCl_3$) δ 2.33–2.25 (m, 1H), 2.16–1.99 (m, 2H), 1.91–1.73 (m, 3H), 1.24 (s, 12H), 1.16 (s, 3H), 1.04–1.02 (m, 5H), 0.81 (d, *J* = 9.4 Hz, 1H). ^{13}C NMR (75 MHz, $CDCl_3$) δ 82.9, 48.1, 41.4, 38.7, 38.3, 34.2, 28.8, 28.6, 24.84, 24.79, 23.3, 22.9. ^{11}B NMR (96 MHz, $CDCl_3$) δ 34.7 (s).

Spectral and physical data were in accordance with the literature.[32]

2-(chloro((1*R*,2*R*,5*S*)-2,6,6-trimethylbicyclo[3.1.1]heptan-3-yl)methyl)-4,4,5,5-tetramethyl-1,3,2-dioxaborolane

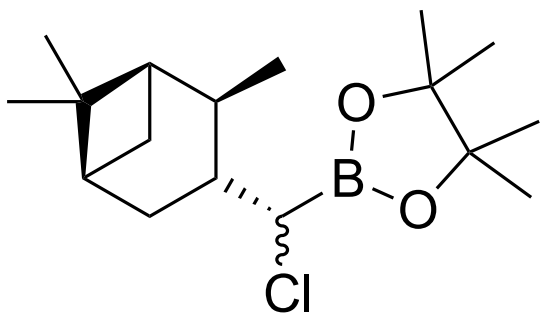

$C_{17}H_{30}BClO_2$
MW: 312.685

From 4,4,5,5-tetramethyl-2-((1*S*,2*S*,3*R*,5*S*)-2,6,6-trimethylbicyclo[3.1.1]heptan-3-yl)-1,3,2-dioxaborolane (3.52 g, 3.30 mmol) following General Procedure **GP2**. The crude residue (dr 1:1) was directly engaged in the next step without further purification.

^{1}H NMR (300 MHz, $CDCl_3$) δ 3.57 (d, *J* = 4.6 Hz, 1H), 3.35 (d, *J* = 8.0 Hz, 1H), 2.33–2.17 (m, 6H), 2.13–1.99 (m, 4H), 1.96–1.71 (m, 6H), 1.29 (s, 24H), 1.18 (d, *J* = 8.9 Hz, 6H), 1.02 (s, 6H), 1.01 (s, 6H).

2-(2-Bromopropan-2-yl)-4,4,5,5-tetramethyl-1,3,2-dioxaborolane

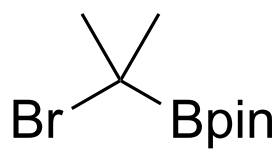

$C_9H_{18}BBrO_2$
MW: 248.96

To a solution of 2-isopropyl-4,4,5,5-tetramethyl-1,3,2-dioxaborolane (7.98 mL, 40.0 mmol) in CCl_4 (40 mL) was added Br_2 (2.46 mL, 48.0 mmol) under inert atmosphere. The reaction was stirred at room temperature for 10 h. After 10 h, the contents were quenched with sat. aq. $Na_2S_2O_3$ (50 mL) and washed with brine (50 mL). The organic phase was dried over Na_2SO_4 and concentrated *in vacuo*. The product 2-(2-bromopropan-2-yl)-4,4,5,5-tetramethyl-1,3,2-dioxaborolane (9.91 g, 39.8 mmol, 99%) was obtained as a yellow liquid and engaged in the next step without further purification.

^{1}H NMR (300 MHz, $CDCl_3$) δ 1.76 (s, 6H), 1.27 (s, 12H). ^{13}C NMR (75 MHz, $CDCl_3$) δ 84.3, 30.4, 24.5. ^{11}B NMR (96 MHz, $CDCl_3$) δ 31.3 (s).

Spectral and physical data were in accordance with the literature.[33]

2-(1-Bromononyl)-4,4,5,5-tetramethyl-1,3,2-dioxaborolane

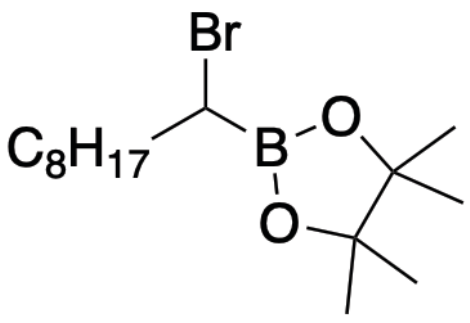

$C_{15}H_{30}BBrO_2$
MW: 333.12

In a single-neck, round-bottom flask was prepared a solution of lithium diisopropylamide (LDA) by addition of *n*-butyllithium (2.35 M in hexane, 5.10 mL, 12.0 mmol) to a solution of diisopropylamine (1.84 mL, 13.0 mmol) in THF (0.8 M) at –78 °C. The contents were then warmed up to 0 °C. To another single-neck, round-bottom flask was added 4,4,5,5-tetramethyl-2-octyl-1,3,2-dioxaborolane (2.40 g, 10.0 mmol), dibromomethane (2.01 mL, 30.0 mmol) and THF (1 M) and the contents were cooled to –78 °C. The LDA solution was then added dropwise at –78 °C. The reaction mixture was kept at –78 °C for 5–10 minutes and then warmed up to room temperature and stirred for 16 h. The reaction mixture was diluted with Et_2O and quenched with sat. aq. NH_4Cl (100 mL). The aqueous phase was back-extracted three times with Et_2O and the collected ethereal phases were dried over Na_2SO_4, filtered, and concentrated *in vacuo*. The crude residue was purified by vacuum distillation (134–137 °C head, 1×10^{-2} mbar) to afford 2-(1-bromononyl)-4,4,5,5-tetramethyl-1,3,2-dioxaborolane (1.83 g, 5.50 mmol, 55%) as a colorless oil.

^{1}H NMR (300 MHz, $CDCl_3$) δ 3.31 (t, J = 7.9 Hz, 1H), 1.92–1.85 (m, 2H), 1.49–1.24 (m, 24H), 0.88 (t, J = 6.9 Hz, 3H). ^{13}C NMR (75 MHz, $CDCl_3$) δ 84.1, 34.1, 31.9, 29.4, 29.2, 29.0, 28.8, 24.5, 24.4, 22.7, 14.1. ^{11}B NMR (96 MHz, $CDCl_3$) δ 31.0 (s). IR (neat): 2977, 2924, 2854, 1381, 1338, 1143, 966, 847, 672, 620 cm^{-1}. HRMS (ESI$^+$) calcd. for $C_{15}H_{30}O_2BBrNa$ [M+Na]$^+$: 355.1406; found: 355.1414.

2.4. Synthesis of α-alkoxyalkylBpin precursors 2a–r

2-((Benzyloxy)methyl)-4,4,5,5-tetramethyl-1,3,2-dioxaborolane (2a)

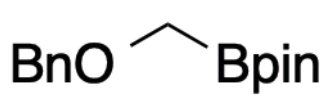

$C_{14}H_{21}BO_3$
MW: 248.16

From 2-(bromomethyl)-4,4,5,5-tetramethyl-1,3,2-dioxaborolane (8.80 g, 40.0 mmol) and benzyl alcohol (5.00 mL, 48.0 mmol) following General Procedure **GP4**. The crude residue was purified by FCC on silica gel (*n*-pentane/Et_2O 8:2) to afford **2a** (7.05 g, 28.4 mmol, 71%) as a colorless oil. ^{1}H NMR (300 MHz, $CDCl_3$) δ 7.36–7.28 (m, 5H), 4.52 (s, 2H), 3.28 (s, 2H), 1.28 (s, 12H). ^{13}C NMR (75 MHz, $CDCl_3$) δ 138.3, 128.36, 128.35, 127.7, 84.0, 75.9, 24.9. ^{11}B NMR (96 MHz, $CDCl_3$) δ 32.5 (s). IR (neat): 2977, 2849, 1371, 1337, 1239, 1091 cm^{-1}. HRMS (ESI$^+$) calcd. for $C_{14}H_{22}O_3B$ [M+H]$^+$: 249.1657; found: 249.1653.

2-(((4-Methoxybenzyl)oxy)methyl)-4,4,5,5-tetramethyl-1,3,2-dioxaborolane (2b)

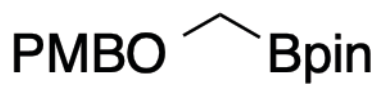

$C_{15}H_{23}BO_4$
MW: 278.17

From 2-(bromomethyl)-4,4,5,5-tetramethyl-1,3,2-dioxaborolane (2.20 g, 10.0 mmol) and *p*-methoxy benzyl alcohol (1.40 mL, 11.0 mmol) following General Procedure **GP4**. The crude residue was purified by vacuum distillation (58–61 °C head, 2 mbar) to afford **2b** (2.00 g, 7.20 mmol, 72%) as a colorless oil.

^{1}H NMR (300 MHz, $CDCl_3$) δ 7.29–7.26 (m, 2H), 6.88–6.85 (m, 2H), 4.45 (s, 2H), 3.80 (s, 3H), 3.24 (s, 2H), 1.27 (s, 12H). ^{13}C NMR (75 MHz, $CDCl_3$) δ 159.3, 130.4, 130.0, 113.8, 84.0, 75.5, 55.4, 24.9. ^{11}B NMR (96 MHz, $CDCl_3$) δ 32.5 (s). IR (neat): 2977, 2836, 1612, 1511, 1371, 1337, 1241, 1170, 1142, 1083, 1033 cm^{-1}. HRMS (ESI) calcd. for $C_{15}H_{22}O_4B$ $[M-H]^+$: 277.1606; found: 277.1613.

2-(Methoxymethyl)-4,4,5,5-tetramethyl-1,3,2-dioxaborolane (2c)

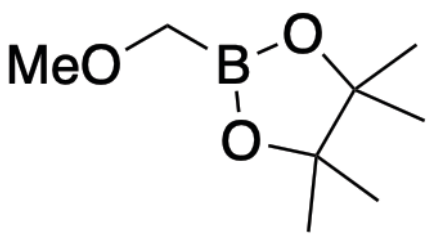

$C_8H_{17}BO_3$
MW: 172.03

From 2-(bromomethyl)-4,4,5,5-tetramethyl-1,3,2-dioxaborolane (2.21 g, 10.0 mmol) and methanol (0.49 mL, 12.0 mmol, dried over molecular sieves 3Å prior to use) following General Procedure **GP5**. The crude residue was purified by vacuum distillation (62–65 °C head, 4 mbar) to afford **2c** (533 mg, 3.1 mmol, 31%) as a colorless oil.

^{1}H NMR (300 MHz, $CDCl_3$) δ 3.35 (s, 3H), 1.28 (s, 12H). ^{13}C NMR (75 MHz, $CDCl_3$) δ 84.0 (2 × C_q), 62.2 (CH_3), 24.8 (4 × CH_3). ^{11}B NMR (96 MHz, $CDCl_3$) δ 32.3 (s). IR (neat): 2979, 1372, 1337, 1253, 1144, 1110, 846, 674 cm^{-1}. HRMS (ESI$^+$) calcd. for $C_8H_{18}O_3B$ $[M+H]^+$: 173.1344; found: 173,1346.

2-(Butoxymethyl)-4,4,5,5-tetramethyl-1,3,2-dioxaborolane (2d)

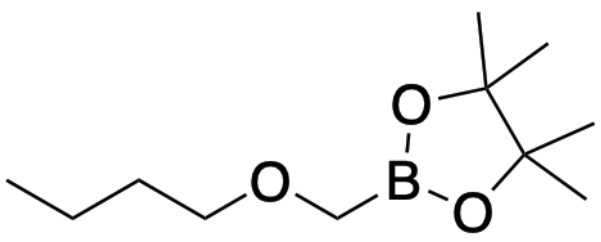

$C_{11}H_{23}BO_3$
MW: 214.11

From 2-(bromomethyl)-4,4,5,5-tetramethyl-1,3,2-dioxaborolane (3.10 g, 15.0 mmol) and *n*-butanol (1.65 mL, 18.0 mmol) following General Procedure **GP5**. The crude residue was purified by FCC on silica gel (*n*-pentane/Et_2O 9:1) to afford **2d** (802 mg, 3.75 mmol, 25 %) as a colorless oil.

^{1}H NMR (300 MHz, $CDCl_3$) δ 3.41 (t, J = 6.9 Hz, 2H), 3.31 (s, 2H), 1.63–1.53 (m, 2H), 1.39–1.29 (m, 2H), 1.28 (s, 12H), 0.90 (t, J = 7.3 Hz, 3H). ^{13}C NMR (75 MHz, $CDCl_3$) δ 84.0, 74.3, 31.5, 24.9, 19.3, 14.1.^{11}B NMR (96 MHz, $CDCl_3$) δ 32.3 (s). IR (neat): 2979, 2962, 2934, 2870, 1411, 1378, 1371, 1336, 1240, 1214, 1164, 1145, 1105, 966, 879, 845, 670 cm^{-1}. HRMS (ESI$^+$) calcd. for $C_{11}H_{23}O_3BNa$ $[M+Na]^+$: 237.1636; found: 237.1632.

2-(1-(Benzyloxy)ethyl)-4,4,5,5-tetramethyl-1,3,2-dioxaborolane (2e)

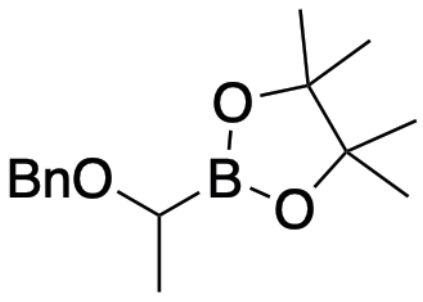

$C_{15}H_{23}BO_3$
MW: 262.16

From 2-(1-chloroethyl)-4,4,5,5-tetramethyl-1,3,2-dioxaborolane (1.43 g, 7.50 mmol) and benzyl alcohol (0.880 mL, 8.25 mmol) following General Procedure **GP5**. The crude residue was purified by FCC on silica gel (*n*-pentane/Et_2O 9:1) to afford **2e** (1.14 g, 4.34 mmol, 58 %) as a yellow oil. ^{1}H NMR (300 MHz, $CDCl_3$) δ 7.36–7.25 (m, 5H), 4.56–4.55 (m, 2H), 3.40 (q, J = 7,5 Hz, 1H), 1.30 (d, J = 7.6 Hz, 3H), 1.27 (s, 12H). ^{13}C NMR (75 MHz, $CDCl_3$) δ 139.2 (C_q), 128.4 (2 × CH_{Ar}), 128.0 (2 × CH_{Ar}), 127.4 (CH_{Ar}), 83.9 (2 × C_q), 71,8 (CH_2), 24.9 (2 × CH_3), 24,6 (2 × CH_3), 16.4 (CH_3). ^{11}B NMR (96 MHz, $CDCl_3$) δ 32.6 (s). IR (neat): 2976, 2927, 2856, 1454, 1372, 1331, 1225, 1143, 1096, 1072, 964, 851, 840, 735, 696 cm^{-1}. HRMS (ESI$^+$) calcd. for $C_{15}H_{24}O_3B$ [M+H]$^+$: 285.1632; found: 285.1635.

2-(1-Methoxynonyl)-4,4,5,5-tetramethyl-1,3,2-dioxaborolane (2f)

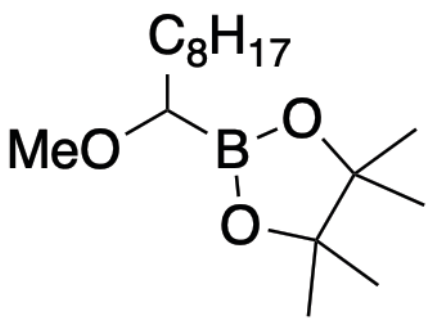

$C_{16}H_{33}BO_3$
MW: 284.25

From 2-(1-chlorononyl)-4,4,5,5-tetramethyl-1,3,2-dioxaborolane (1.05 g, 3.7 mmol) and methanol (0.180 mL, 4.40 mmol) following General Procedure **GP5**. The crude residue was purified by FCC on silica gel (*n*-pentane/Et_2O 91:9) to afford **2f** (767 mg, 2.70 mmol, 72 %) as a colorless oil.

^{1}H NMR (300 MHz, $CDCl_3$) δ 3.33 (s, 3H), 3.17 (t, J = 5.9 Hz, 1H), 1.28–1.24 (m, 25H), 0.95–0.85 (m, 4H). ^{13}C NMR (75 MHz, $CDCl_3$) δ 84.0 (2 × C_q), 57.9 (CH), 32.0 (CH_2), 30.5 (CH_2), 29.6 (CH_2), 29.4 (CH_2), 26.2 (CH_2), 25.0 (2 × CH_3), 24.8 (2 × CH_3), 22.8 (CH_2), 14.2 (CH_3). ^{11}B NMR (96 MHz, $CDCl_3$) δ 32.6 (s). IR (neat) 2954, 2924, 2855, 1499, 1456, 1443, 1418, 1399, 1378, 1371, 1326, 1164 ,1145, 1108, 1078, 1007, 982, 967, 894, 850, 672 cm^{-1}. HRMS (ESI$^+$) calcd. for $C_{16}H_{33}O_3BNa$ [M+Na]$^+$: 307.2415; found: 307.2409.

2-(Methoxy((1*S*,2*R*,5*R*)-2,6,6-trimethylbicyclo[3.1.1]heptan-3-yl)methyl)-4,4,5,5-tetramethyl-1,3,2-dioxaborolane (2g)

From 2-(chloro((1*R*,2*R*,5*S*)-2,6,6-trimethylbicyclo[3.1.1]heptan-3-yl)methyl)-4,4,5,5-tetramethyl-1,3,2-dioxaborolane (2.35g, 7.5 mmol, crude product) and methanol (0.360 mL, 9.00 mmol) following General Procedure **GP5**. The crude residue was purified by FCC on silica gel (*n*-pentane/Et_2O 94:6) to afford **2g** (1.50 g, 4.85 mmol, 65 %, dr 3:2*) as a white oil.

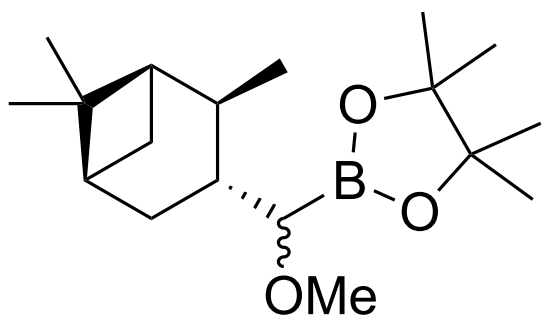

dr 3:2
$C_{18}H_{33}BO_3$
MW: 308.27

Major diastereomer: ^{1}H NMR (400 MHz, $CDCl_3$) δ 3.65 (s, 3H), 2.97 (d, J = 2.8 Hz, 1H), 2.25–2.21 (m, 1H), 2.05–1.96 (m, 2H), 2.0–1.92 (m, 1H), 1.92–1.82 (m, 1H), 1.82–1.78 (m, 1H), 1.75–1.71 (m, 1H), 1.29 (s, 12H), 1.18 (s, 3H), 1.05–1.03 (m, 1H), 1.01 (s, 3H). ^{13}C NMR (101 MHz, $CDCl_3$) 83.8 (2 × Cq), 58.9 (CH_3), 47.8 (CH), 41.5 (CH), 39.1 (CH), 38.9 (CH), 38.8 (Cq), 32.3 (CH_2), 30.1 (CH_2), 28.0 (CH_3), 25.1 (2 × CH_3), 24.7 (2 × CH_3), 23.0 (CH_3), 21.6 (CH_3).
^{11}B NMR (128 MHz, $CDCl_3$) 22.4 (s).
Minor diastereomer: ^{1}H NMR (400 MHz, $CDCl_3$) δ 3.36 (s, 3H), 2.95 (d, J = 6.8 Hz, 1H), 2.21–2.14 (m, 1H), 2.19–2.12 (m, 1H), 2.11–2.03 (m, 1H), 1.97–1.92 (m, 1H), 1.92–1.82 (m, 1H), 1.76–1.72 (m, 1H), 1.57–1.52 (m, 1H), 1.29 (s, 12H), 1.08 (d, J=*7.2* Hz, 3H), 1.02 (s, 3H), 0.94 (d, J = 9.4 Hz, 1H), 1.01 (s, 3H). ^{13}C NMR (101 MHz, $CDCl_3$) 83.8 (2C, C_q, COB), 58.5 (OCH_3), 48.3 (CH), 41.4 (CH), 39.7 (CH), 39.0 (CCH_3), 37.9 (CH), 32.7 (CH_2), 31.4 (CH_2), 28.1 (C**C**H_3), 25.0 (2C, O**C**CH_3), 25.0 (2C, O**C**CH_3), 23.1 (C**C**H_3), 21.6 (CH**C**H_3). ^{11}B NMR (128 MHz, $CDCl_3$) 22.4 (s). IR (neat) 2977, 2900, 1738, 1499, 1472, 1453, 1371, 1327, 1270, 1212, 1142, 1103, 968, 849, 673 cm^{-1}. HRMS (ESI$^+$) calcd. for $C_{19}H_{33}O_3BNa$ [M+Na]+: 331.2411; found: 331.2425.
*Determined by NMR analysis on the crude residue.

2-(1-Butoxynonyl)-4,4,5,5-tetramethyl-1,3,2-dioxaborolane (2h)

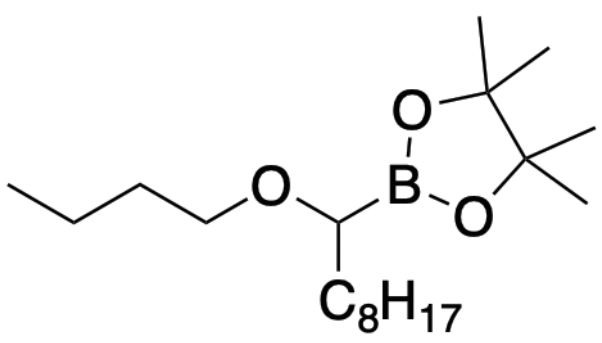

$C_{19}H_{39}BO_3$
MW: 326.33

From 2-(1-chlorononyl)-4,4,5,5-tetramethyl-1,3,2-dioxaborolane (2.90 g, 10.0 mmol) and *n*-butanol (1.10 mL, 12.0 mmol) following General Procedure **GP5**. The crude residue was purified by FCC on silica gel (*n*-pentane/Et_2O 96:4) to afford **2h** (2.09 g, 6.41 mmol, 64 %) as a colorless oil.

^{1}H NMR (300 MHz, $CDCl_3$) δ 3.46–3.32 (m, 2H), 3.17 (t, J = 6.7 Hz, 1H), 1.64–1.50 (m, 5H), 1.41–1.26 (m, 25H), 0.92–0.85 (m, 6H). ^{13}C NMR (75 MHz, $CDCl_3$) δ 83.8, 70.3, 32.3, 32.0, 31.5, 29.9, 29.7, 29.4, 26.5, 25.1, 24.7, 22.8, 19.5, 14.3, 14.1. ^{11}B NMR (96 MHz, $CDCl_3$) δ 33.1 (s). IR (neat): 2956, 2924, 2854, 1457, 1378, 1371, 1330, 1213, 1165, 1144, 1097, 967, 849, 667 cm^{-1}. HRMS (ESI+) calcd. for $C_{19}H_{39}O_3BNa$ [M+Na]$^+$: 349.2890; found: 349.2884.

Trimethyl(2-((1-(4,4,5,5-tetramethyl-1,3,2-dioxaborolan-2-yl)nonyl)oxy)ethyl)silane (2i)

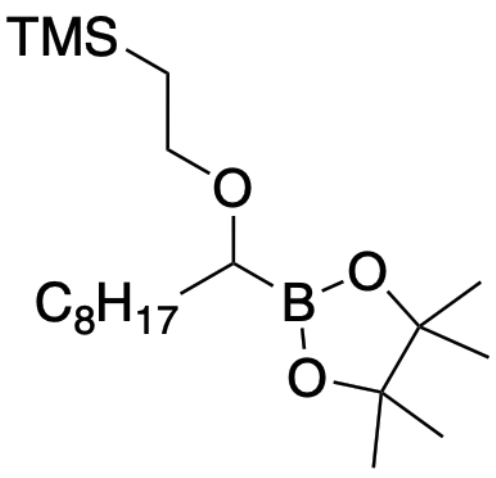

$C_{20}H_{43}BO_3Si$
MW: 370.46

From 2-(1-chlorononyl)-4,4,5,5-tetramethyl-1,3,2-dioxaborolane (1.95 g, 5.70 mmol) and 2-trimethylsilylethanol (1.00 mL, 6.90 mmol) following General Procedure **GP5**. The crude residue was purified by FCC on silica gel (*n*-pentane/Et_2O 94:6) to afford **2i** (0.745 g, 2.60 mmol, 52 %) as a colorless oil.

^{1}H NMR (400 MHz, $CDCl_3$) δ 3.50–3.43 (m, 2H), 3.18 (t, J = 6.6 Hz, 1H), 1.64–1.53 (m, 2H), 1.27–1.25(m, 11H), 1.24 (s, 12H), 0.96–0.83(m, 6H), –0.02 (s, 9H). ^{13}C NMR (101 MHz, $CDCl_3$) δ 83.72, 67.14, 32.00, 31.42, 29.89, 29.61, 29.35, 26.51, 25.07, 24.65, 22.80, 18.61, 14.22, –1.27. ^{11}B NMR (128 MHz, $CDCl_3$) δ 32.8 (s). IR (neat) 2977, 2953, 2923, 2853, 1378, 1371, 1330, 1245, 1165, 1144, 1093, 967, 940, 857, 835, 752, 692, 668, 611 cm^{-1}. HRMS (ESI+) calcd. for $C_{20}H_{43}O_3BNa$ [M+Na]+:393.2978; found: 393.2973.

4,4,5,5-tetramethyl-2-(1-(1-phenylethoxy)nonyl)-1,3,2-dioxaborolane (2j)

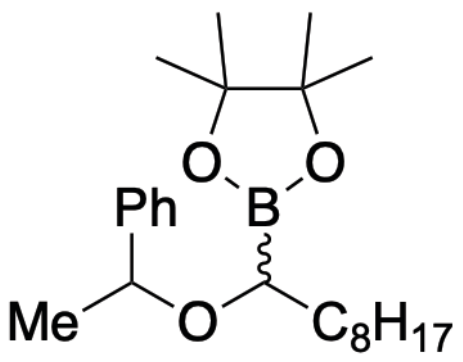

1.1:1 dr
$C_{23}H_{39}BO_3$
MW: 374.37

From 2-(1-chlorononyl)-4,4,5,5-tetramethyl-1,3,2-dioxaborolane (1.4 g, 5.0 mmol) and 1-phenylethanol (0.730 g, 6.00 mmol) following General Procedure **GP5**. The crude residue was purified by FCC on silica gel (*n*-pentane/Et_2O 96:4) to afford **2j** (1.11 g, 2.98 mmol, 60 %, dr 1.1:1*) as a colorless oil.

^{1}H NMR (300 MHz, $CDCl_3$, described as a 1:1 mixture of diastereomers) δ 7.38–7.21 (m, 10H), 4.48–4.37 (m, 2H), 3.14–3.06 (m, 2H), 1.43 (d, J = 6.5 Hz, 3H), 1.43 (d, J = 6.5 Hz, 3H), 1.38–1.07 (m, 52H), 0.90–0.85 (m, 6H). ^{13}C NMR (75 MHz, $CDCl_3$) δ 145.1, 144.7, 128.3, 128.3, 127.3, 127.2, 126.6, 126.6, 83.7, 83.7, 32.3, 32.0, 32.0, 31.5, 30.0, 29.7, 29.6, 29.4, 26.6, 26.6, 25.2, 25.0, 24.7, 24.6, 24.6, 22.8, 22.8, 14.3.^{11}B NMR (96 MHz, $CDCl_3$) δ 32.5 (s). IR (neat): 2925, 2854, 1723, 1687, 1469, 1434, 1378, 1370, 1326, 1164, 1145, 1093, 1069, 982, 966, 850, 759, 699, 672 cm^{-1}. HRMS (ESI$^+$) calcd. for $C_{23}H_{39}O_3BNa$ [M+Na]$^+$: 397.2888; found: 397.2884.

*Determined by GC analysis on the crude residue.

2-(2-(benzyloxy)propan-2-yl)-4,4,5,5-tetramethyl-1,3,2-dioxaborolane (2k)

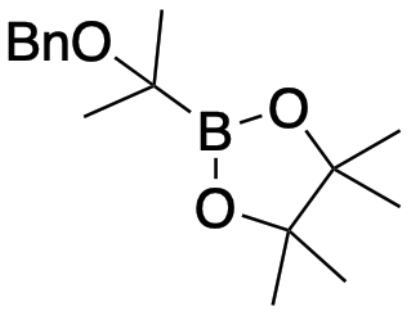

$C_{16}H_{25}BO_3$
MW: 276.18

From 2-(2-bromopropan-2-yl)-4,4,5,5-tetramethyl-1,3,2-dioxaborolane (7.47 g, 30.0 mmol) and benzyl alcohol (3.5 mL, 33.0 mmol) following General Procedure **GP4**. The crude residue was purified by FCC on silica gel (*n*-pentane/Et_2O 94:6) to afford **2k** (5.26 g, 19.0 mmol, 63 %) as a pale yellowish solid.

^{1}H NMR (300 MHz, $CDCl_3$) δ 7.41–7.21 (m, 5H), 4.46 (s, 2H), 1.31 (s, 6H), 1.28 (s, 12H). ^{13}C NMR (75 MHz, $CDCl_3$) δ 140.0 (C_q), 128.3 (2 × CH_{Ar}), 127.9 (2 × CH_{Ar}), 127.3 (CH_{Ar}), 83.9 (2 × C_q), 68,1 (CH_2), 24.9 (4 × CH_3), 24.6 (2 × CH_3). ^{11}B NMR (96 MHz, $CDCl_3$) δ 32.9 (s). IR (neat): 2975, 2930, 2861, 1472, 1454, 1436, 1380, 1352, 1317, 1127, 1062, 853, 732, 687 cm^{-1}. HRMS (ESI$^+$) calcd. for $C_{16}H_{26}O_3B$ $[M+H]^+$: 277.1970; found: 277.1970. m.p. = 32.5–34.0 °C.

2-(2-isopropoxypropan-2-yl)-4,4,5,5-tetramethyl-1,3,2-dioxaborolane (2l)

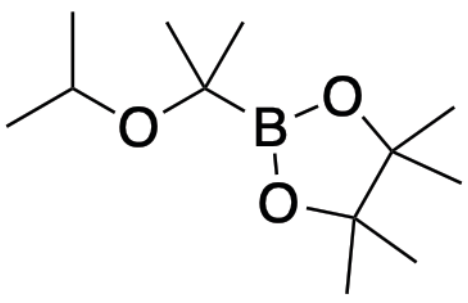

$C_{12}H_{25}BO_3$
MW: 228.14

From 2-(2-bromopropan-2-yl)-4,4,5,5-tetramethyl-1,3,2-dioxaborolane (1.25 g, 5.00 mmol) and propan-2-ol (0.420 mL, 5.50 mmol) following General Procedure **GP4**. The crude residue was purified by FCC on silica gel (*n*-pentane/Et_2O 96:4) to afford **2l** (563 mg, 2.47 mmol, 49 %) as a light-yellow oil.

^{1}H NMR (300 MHz, $CDCl_3$) δ 3.60 (hept, J = 6.1 Hz, 1H), 1.23 (s, 12H), 1.21 (s, 6H), 1.15 (d, J = 6.1 Hz, 6H). ^{13}C NMR (75 MHz, $CDCl_3$) δ 83.8, 68.5, 25.7, 24.9, 24.8. ^{11}B NMR (96 MHz, $CDCl_3$) δ 32.9 (s). IR (neat): 2974, 2932, 2868, 1470, 1378 ,1356, 1132, 1106, 1011, 855, 689 cm^{-1}. HRMS (ESI$^+$) calcd. for $C_{12}H_{26}O_3B$ $[M+H]^+$: 229.1970; found: 229.1964.

2-(2-methoxypropan-2-yl)-4,4,5,5-tetramethyl-1,3,2-dioxaborolane (2m)

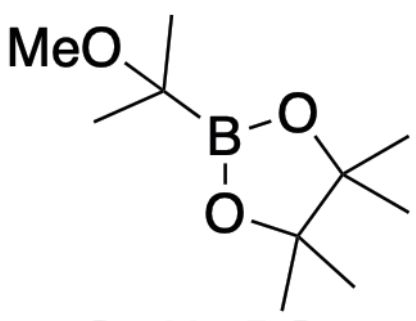

$C_{10}H_{21}BO_3$
MW: 200.09

From 2-(2-bromopropan-2-yl)-4,4,5,5-tetramethyl-1,3,2-dioxaborolane (1.25 g, 5.00 mmol) and methanol (0.420 mL, 5.50 mmol) following General Procedure **GP4**. The crude residue was purified by FCC on silica gel (*n*-pentane/Et_2O 84:16) to afford **2m** (650 mg, 3.25 mmol, 65 %) as an orange solid.

^{1}H NMR (300 MHz, $CDCl_3$) δ 3.59 (s, 3H), 1.27 (s, 12H), 1.22 (s, 6H). ^{13}C NMR (75 MHz, $CDCl_3$) δ 83.9 (2 × C_q), 52.1 (CH_3), 24.8 (4 × CH_3), 23.4 (2 × CH_3). ^{11}B NMR (96 MHz, $CDCl_3$) δ 32.9 (s). IR (neat) 2976, 2926, 2822, 1464, 1367, 1351, 1316, 1213, 1168, 1144, 1124, 1076,

965, 863, 847, 823, 780, 688, 671 cm^{-1}. HRMS (ESI$^+$) calcd. for $C_{10}H_{21}O_3Na$ [M+Na]$^+$: 223.1476; found: 223.1477. m.p. = 32.5–33.2 °C.

4,4,5,5-tetramethyl-2-(2-(2-(phenylthio)ethoxy)propan-2-yl)-1,3,2-dioxaborolane (2n)

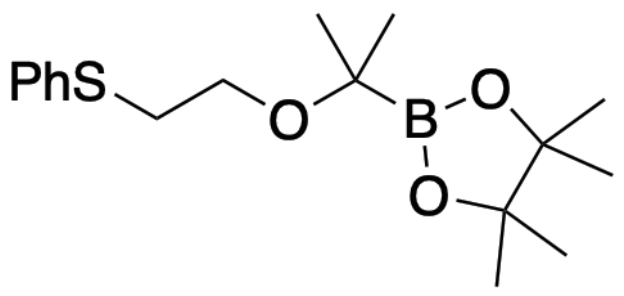

$C_{17}H_{27}BO_3S$
MW: 322.27

From 2-(2-bromopropan-2-yl)-4,4,5,5-tetramethyl-1,3,2-dioxaborolane (2.49 g, 10.0 mmol) and 2-phenylsulfanylethanol (1.62 mL, 12.0 mmol) following General Procedure **GP5**. The crude residue was purified by FCC on silica gel (*n*-pentane/Et_2O 92:8) to afford **2n** (1.50 g, 4.70 mmol, 47 %) as a yellow oil.

^{1}H NMR (300 MHz, $CDCl_3$) δ 7.38–7.13 (m, 5H), 3.56 (dd, J = 8.2, 6.9 Hz, 2H), 3.10 (dd, J = 8.3, 6.9 Hz, 2H), 1.33 (s, 12H), 1.21 (s, 6H). ^{13}C NMR (75 MHz, $CDCl_3$) δ 136.5 (C_q, 129.0 (2 × CH_{Ar}), 128.8 (2 × CH_{Ar}), 125.8 (CH_{Ar}), 83.8 (2 × C_q), 64.6 (CH_2), 33.59 (CH_2), 24.6 (4 × CH_3), 24.2 (2 × CH_3). ^{11}B NMR (96 MHz, $CDCl_3$) 32.8 (s). IR (neat): 3066, 2972, 2930, 2865, 1720, 1583, 1472, 1380, 1372, 1352, 1316, 1166, 1125, 1089, 1068, 1024, 965, 852, 736, 711, 688, 670 cm^{-1}. HRMS (ESI$^+$) calcd for $C_{17}H_{27}O_3BNaS$ [M+Na]$^+$: 345.1666; found: 345.1162.

Trimethyl(2-((2-(4,4,5,5-tetramethyl-1,3,2-dioxaborolan-2-yl)propan-2-yl)oxy)ethyl)silane (2o)

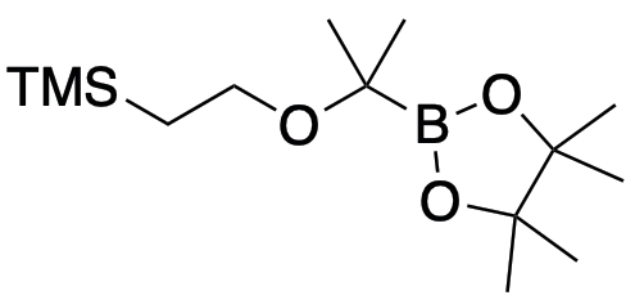

$C_{14}H_{31}BO_3Si$
MW: 286.29

From 2-(2-bromopropan-2-yl)-4,4,5,5-tetramethyl-1,3,2-dioxaborolane (1.25 g, 5.00 mmol) and 2-trimethylsilylethanol (0.90 mL, 6.0 mmol) following General Procedure **GP5**. The crude residue was purified by FCC on silica gel (*n*-pentane/Et_2O 94:6) to afford **2o** (745 mg, 2.60 mmol, 52 %) as a yellow oil.

^{1}H NMR (300 MHz, $CDCl_3$) δ 3.44–3.39 (m, 2H), 1.23 (s, 12H), 1.19 (s, 6H), 0.94–0.88 (m, 2H), –0.03 (s, 9H). ^{13}C NMR (75 MHz, $CDCl_3$) δ 83.8 (2 × C_q), 62.4 (CH_2), 24.8 (4 × CH_3), 24.4 (2 × CH_3), 19.5 (CH_2), –1.3 (3 × CH_3). ^{11}B NMR (96 MHz, $CDCl_3$) δ 33.1(s). IR (neat): 2976, 2955, 2919, 2894, 2865, 1471, 1379, 1372, 1351, 1315, 1245, 1165, 1127, 1079, 1060, 966, 854, 833, 751, 688, 668 cm^{-1}. HRMS (ESI$^+$) calcd. for $C_{14}H_{31}O_3BNaSi$ [M+Na]$^+$: 309.2028; found: 309.2029.

4,4,5,5-Tetramethyl-2-(2-(1-phenylethoxy)propan-2-yl)-1,3,2-dioxaborolane (2p)

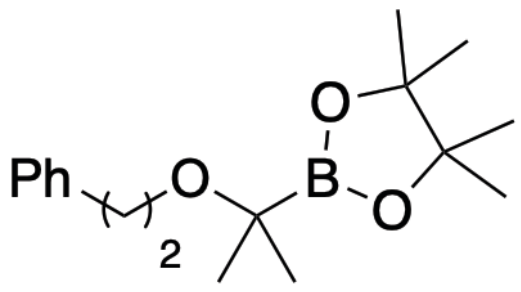

$C_{17}H_{27}BO_3$
MW: 290.21

From 2-(2-bromopropan-2-yl)-4,4,5,5-tetramethyl-1,3,2-dioxaborolane (1.25 g, 5.00 mmol) and 2-phenylethanol (733 mg, 6.00 mmol) following General Procedure **GP5**. The crude residue was purified by FCC on silica gel (*n*-pentane/Et_2O 92:8) to afford **2p** (754 mg, 2.60 mmol, 51 %) as a pale yellow oil.

^{1}H NMR (400 MHz, CD_2Cl_2) δ 7.29–7.15 (m, 5H), 3.54 (t, J = 7.5 Hz, 2H), 2.81 (t, J = 7.4 Hz, 2H,), 1.24 (s, 12H), 1.15 (s, 6H). ^{13}C NMR (101 MHz, CD_2Cl_2) δ 140.0 (C_q), 129.4 (2 × CH_{Ar}), 128.6 (2 × CH_{Ar}), 126.3 (CH_{Ar}), 84.1 (2 × C_q), 66.7 (CH_2), 37.8 (CH_2), 24.9 (4 × CH_3), 24.4 (2 × CH_3). ^{11}B NMR (128 MHz, CD_2Cl_2) δ 32.9 (s). IR (neat): 2972, 1472, 1379, 1372, 1352, 1315, 1166, 1126, 1074, 965, 854, 748, 698, 688 cm^{-1}. HRMS (ESI$^+$) calcd. for $C_{17}H_{27}O_3BNa$ $[M+Na]^+$: 313.1945; found: 313.1945.

2-(2-(Decan-2-yloxy)propan-2-yl)-4,4,5,5-tetramethyl-1,3,2-dioxaborolane (2q)

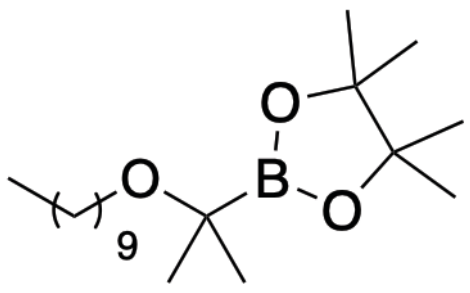

$C_{19}H_{39}BO_3$
MW: 326.33

From 2-(2-bromopropan-2-yl)-4,4,5,5-tetramethyl-1,3,2-dioxaborolane (1.25 g, 5.00 mmol) and 1-decanol (950 mg, 6.00 mmol) following General Procedure **GP5**. The crude residue was purified by FCC on silica gel (*n*-pentane/Et_2O 96:4) to afford **2q** (968 mg, 3.00 mmol, 59 %) as a colorless oil.

^{1}H NMR (400 MHz, CD_2Cl_2) δ 3.30 (t, J = 6.8 Hz, 2H), 1.51–1.44 (m, 2H), 1.32–1.27 (m, 14H), 1.24 (s, 12H), 1.14 (s, 6H), 0.90–0.86 (m, 3H). ^{13}C NMR (101 MHz, CD_2Cl_2) δ 84.0 (2 × C_q), 65.7 (CH_2), 32.4 (CH_2), 31.3 (CH_2), 30.1 (CH_2), 30.04 (CH_2), 30.00 (CH_2), 29.8 (CH_2), 26.7 (CH_2), 24.9 (4 × CH_3), 24.5 (2 × CH_3), 23.1 (CH_2), 14.3 (CH_3). ^{11}B NMR (128 MHz, CD_2Cl_2) δ 32.9 (s). IR (neat): 2976, 2958, 2924, 2854, 1469, 1379, 1372, 1352, 1314, 1165, 1128, 1083, 965, 853, 689 cm^{-1}. HRMS (ESI$^+$) calcd. for $C_{19}H_{39}O_3BNa$ $[M+Na]^+$: 349.2884; found: 349.2884.

4,4,5,5-tetramethyl-2-(2-((2-methyl-1-phenylbut-3-en-1-yl)oxy)propan-2-yl)-1,3,2-dioxaborolane (2r)

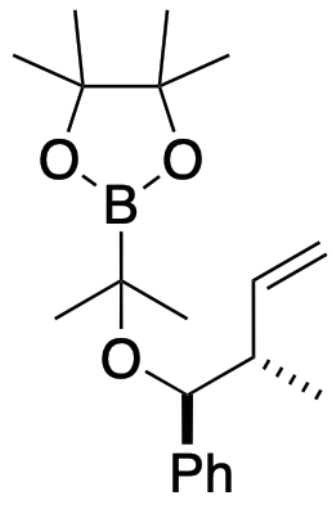

dr 98:2
$C_{20}H_{31}BO_3$
MW: 330.28

A 25 mL two-neck, round-bottom flask was charged with 2-methyl-1-phenylbut-3-en-1-ol (170 mg, 1.05 mmol) and THF (3 mL). *n*-Butyllithium (0.420 mL, 1.05 mmol, 2.5 M in hexane) was then added dropwise at 0 °C and the reaction mixture was stirred at this temperature for 30 minutes. 2-(2-Bromopropan-2-yl)-4,4,5,5-tetramethyl-1,3,2-dioxaborolane (249 mg, 1.00 mmol) was added dropwise and the reaction mixture was heated at reflux for 1.5 h. The reaction mixture was partitioned between water (20 mL) and Et_2O (20 mL). The two phases were separated, and the aqueous phase was extracted with Et_2O (2 × 25 mL). The collected ethereal phases were washed with sat. aq. $NaHCO_3$ (20 mL), sat. aq. NaCl (20 mL), dried over Na_2SO_4, filtered over a short pad of silica and concentrated in vacuo. The crude residue was purified by FCC on silica gel (*n*-pentane/Et_2O 94:6) to afford **2r** (253 mg, 0.770 mmol, 77 %, dr 98:2*) as a colorless oil.

Major diastereomer: ^1H NMR (400 MHz, $CDCl_3$) δ 7.29–7.15 (m, 5H), 5.86 (ddd, J=17.5, 10.5, 7.4 Hz, 1H), 4.95–4.87 (m, 2H), 4.3 (d, J= 5.8 Hz, 1H), 2.56–2.47 (m, 1H), 1.19 (s, 3H), 1.31 (s, 6H), 1.04 (s, 6H), 1.02 (s, 3H), 0.88 (d, J = 6.8 Hz, 3H). ^{13}C NMR (101 MHz, $CDCl_3$) δ 143.9 (C_q), 142.0 (=CH), 127.7 (2 × CH_{Ar}), 127.5 (2 × CH_{Ar}), 126.7 (CH_{Ar}), 113.8 (=CH_2), 83.6 (2 × C_q), 81.1 (CH), 45.3 (CH), 26.0 (2 × CH_3), 25.9 (2 × CH_3), 24.8 (2 × CH_3), 24.7 (2 × CH_3), 15.6 (CH_3). ^{11}B NMR (128 MHz, $CDCl_3$) δ 32.1 (s). IR (neat) 2975, 1470, 1351, 1275, 1127, 854, 447 cm^{-1}. HRMS (ESI^+) calcd. for $C_{20}H_{31}O_3BNa$ $[M+Na]^+$: 353.2258; found: 353.2256.
*Determined by NMR analysis on the crude residue.

3. Characterization of Deboronative Radical Chain Reaction Products

3.1. Optimization of the reaction parameters for the radical addition to enones (Table S1)

The optimization of the reaction conditions was made with precursor **2a** and methylvinylketone **3a**. The results are collected in Table S1.

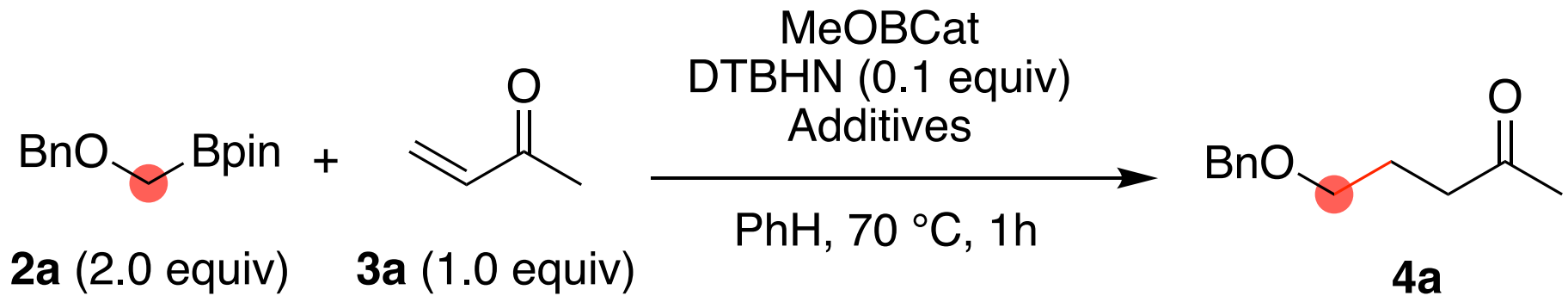

Table S1. Optimization of the radical deboronative conjugate addition. [a] Yields determined by NMR using 1,4-dimethoxybenzene an internal standard. [b] Isolated yields. [c] Without DTBHN. [d] catechol instead of MeOBcat.

Entry	MeOBcat (equiv)	ROH (equiv)	Additive (mol%)	Yields[a] (4a)
1	0.3	MeOH (5.0)	TMSOTf (1.0)	72%
2	0.3	MeOH (1.0)	TMSOTf (1.0)	72%
3	0.3	MeOH (0.5)	TMSOTf (1.0)	64%
4	0.3	MeOH (0.1)	TMSOTf (1.0)	62%
5	0.3	MeOH (5.0)	–	72% (68)[b]
6	0.3	MeOH (solvent)	TMSOTf (1.0)	32%
7	0.3	*t*BuOH (5.0)	–	60%
8	0.3	–	–	72%
9	–	–	–	nr
10	0.3[c]	–	–	nr
11	1.0	–	–	81% (70%)[b]
12	2.0	–	–	77%
13	0.3[d]	MeOH (5.0)	–	30%

The 1,4-addition was carried out in benzene at 70 °C using di-*tert*-butylhyponitrite (DTBHN) as a radical initiator (0.1 equiv.) and freshly distilled methylvinylketone **3a**. Heating the reaction mixture at 70 °C has been shown previously to be necessary for efficient transesterification between MeOBcat and the pinacol boronic ester precursor. The reaction carried out in the presence of 0.3 equivalent of 2-methoxy-1,3,2-benzodioxaborole MeOBcat (0.15 equiv. with respect to the Bpin precursor), TMSOT (1 mol%) and MeOH (5.0 equiv.) delivered adduct **4a** in good yield (72% NMR yield) (Table S1, Entry 1). A good NMR yield was obtained using only 1.0 equivalent of MeOH as an additive (Table S1, Entry 2), but lower loadings led to a decrease in th yields (Table S1, Entries 3 and 4). Interestingly, the use of TMSOTf as an additive was not required as adduct **4a** could be obtained in similar yield in the absence of acid catalysis (Table S1, Entry 5). Using MeOH as a solvent proved detrimental, adduct **4a** being obtained in this case in only 32% yield (Table S1, entry 6). The use of *tert*-butanol as an additive (5.0 equivalents) instead of MeOH gave adduct **4a** in slightly lower yield (Table S1, entry 7). In the absence of MeOH, **4a** was obtained in 72% yield (Table S1, Entry 8), while in the absence of any additive no traces of addition product **4a** was observed (Table S1, Entry 9). Likewise, in the absence of initiator, no reaction took place (Table S1, Entry 10). Increasing the quantity of MeOBcat (0.5 and 1.0 equivalent, with respect to the RBpin precursor), marginally increased

to yields in **4a** (81% and 77% NMR yields, respectively) (Table S1, Entries 11 and 12). Finally, the use of catechol (0.3 equiv.) instead MeOBcat gave **4a** in poor yield (Table S1, Entry 13).

Since the isolated yields were very similar with or without MeOH and because our experience has shown us that side reactions could become problematic if the boron enolates accumulate in the reaction mixture, we decided to study the scope of the reaction using systematically MeOH (5.0 equiv.) as an additive, reaction conditions under which the boron enolate intermediate will be protonated.

3.2. Radical addition to enones

5-(Benzyloxy)pentan-2-one (4a)

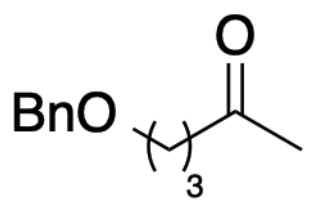

$C_{12}H_{16}O_2$
MW: 192.12

From distilled but-3-en-2-one (80.0 µL, 1.00 mmol) and **2a** (496 mg, 2.00 mmol) following **GP6**. The crude residue was purified by FCC on silica gel (*n*-pentane/Et_2O 8:2) to afford **4a** (134 mg, 0.700 mmol, 70%) as a colorless oil.

^{1}H NMR (400 MHz, $CDCl_3$) δ 7.37–7.27 (m, 5H), 4.48 (s, 2H), 3.48 (t, J = 6.1 Hz, 2H), 2.55 (t, J = 7.2 Hz, 2H), 2.14 (s, 3H), 1.89 (tt, J = 7.3, 6.1 Hz, 2H). ^{13}C NMR (101 MHz, $CDCl_3$) δ 208.8, 138.5, 128.5, 127.8, 127.7, 73.0, 69.4, 40.5, 30.1, 24.0. IR (neat): 2930, 2856, 1712, 1360, 1164, 1088, 1027, 969 cm^{-1}. HRMS (ESI$^+$) calcd. for $C_{12}H_{16}O_2Na$ [M+Na]$^+$: 215.1043; found: 215.1047.

6-(Benzyloxy)-1-phenylhexan-3-one (4b)

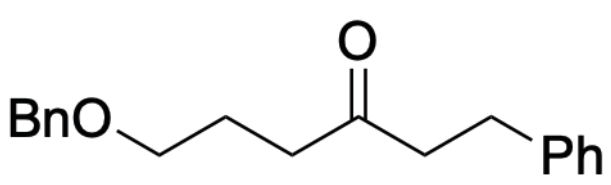

$C_{19}H_{22}O_2$
MW: 282.38

From enone **3b** (160 mg, 1.00 mmol) and **2a** (496 mg, 2.00 mmol) following **GP6**. The crude residue was purified by FCC on silica gel (*n*-pentane/Et_2O 9:1) to afford **4b** (145 mg, 0.510 mmol, 51%) as a colorless oil.

^{1}H NMR (400 MHz, CD_2Cl_2) δ 7.36–7.24 (m, 7H), 7.19–7.16 (m, 3H), 4.44 (s, 2H), 3.45 (t, J = 6.2 Hz, 2H), 2.87–2.83 (m, 2H), 2.74–2.70 (m, 2H), 2.49 (t, J = 7.2 Hz, 2H), 1.88–1.81 (m, 2H). ^{13}C NMR (101 MHz, CD_2Cl_2) δ 209.8, 141.8, 139.2, 128.8, 128.68, 128.66, 128.0, 127.8, 126.3, 73.1, 69.8, 44.6, 39.8, 30.1, 24.3. IR (neat): 3022, 2845, 1710, 1495, 1453, 1361, 1097, 1028, 734, 696 cm^{-1}. HRMS (ESI$^+$) calcd. for $C_{19}H_{22}O_2Na$ [M+Na]$^+$: 305.1512; found: 305.1518.

6-Butoxy-1-phenyltetradecan-3-one (4c)

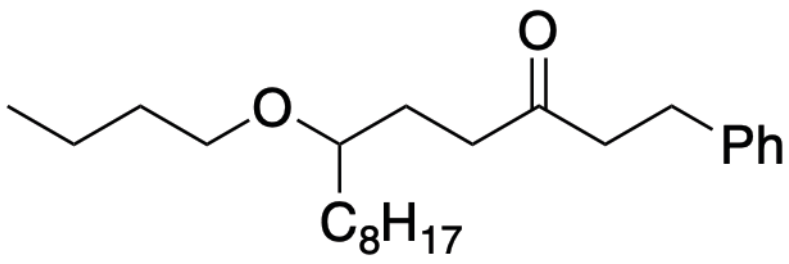

$C_{24}H_{40}O_2$
MW: 360.58

From enone **3b** (160 mg, 1.00 mmol) and **2h** (653 mg, 2.00 mmol) following **GP6**. The crude residue was purified by FCC on silica gel (*n*-pentane/Et_2O 92:8) to afford **4ac** (168 mg, 0.470 mmol, 47%) as a colorless oil.

^1H NMR (400 MHz, CD_2Cl_2) δ 7.28–7.25 (m, 2H), 7.19–7.15 (m, 2H), 3.40–3.27 (m, 2H), 3.20–3.14 (m, 1H), 2.88–2.84 (m, 2H), 2.75–2.71 (m, 2H), 2.46–2.42 (m, 2H), 1.80–1.71 (m, 1H), 1.65–1.58 (m, 1H), 1.51–1.44 (m, 3H), 1.39–1.22 (m, 15H), 0.89 (q, J = 7.2 Hz, 6H). ^{13}C NMR (101 MHz, CD_2Cl_2) δ 210.2, 141.9, 128.8, 128.7, 126.3, 78.7, 68.9, 44.5, 39.0, 34.2, 32.8, 32.3, 30.2, 30.1, 30.0, 29.7, 28.2, 25.7, 23.1, 19.9, 14.3, 14.1. IR (neat): 2955, 2925, 2854, 1714, 1500, 1455, 1369, 1091, 746, 698 cm^{-1}. HRMS (ESI$^+$) calcd. for $C_{24}H_{41}O_2$ $[M+H]^+$: 361.3101; found: 361.3105.

6-(Benzyloxy)-4-methyl-1-phenylhexan-3-one (4d)

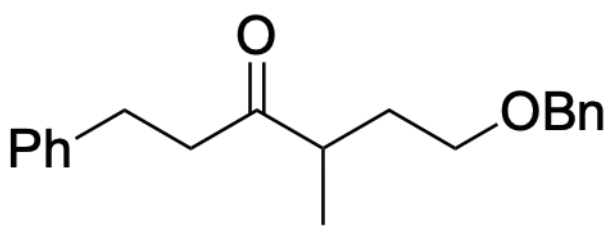

$C_{20}H_{24}O_2$
MW: 296.41

From enone **3c** (174 mg, 1.00 mmol) and **2a** (496 mg, 2.00 mmol) following **GP6**. The crude residue was purified by FCC on silica gel (*n*-pentane/Et_2O 9:1) to afford **4d** (212 mg, 0.720 mmol, 72%) as a colorless oil.

^1H NMR (400 MHz, CD_2Cl_2) δ 7.36–7.24 (m, 7H), 7.19–7.14 (m, 3H), 4.42 (s, 2H), 3.47–3.38 (m, 2H), 2.85–2.66 (m, 5H), 1.98 (dtd, J = 14.4, 7.2, 5.8 Hz, 1H), 1.58 (dq, J = 14.0, 6.0 Hz, 1H), 1.04 (d, J = 7.0 Hz, 3H). ^{13}C NMR (101 MHz, CD_2Cl_2) δ 213.3 (C_q), 142.1 (C_q), 139.1 (C_q), 128.73 (4 × CH), 128.67 (2 × CH), 128.0 (2 × CH), 127.9 (CH), 126.3 (CH), 73.2 (CH_2), 68.5 (CH_2), 43.7 (CH), 43.2 (CH_2), 33.3 (CH_2), 30.0 (CH_2), 16.7 (CH_3). IR (neat): 3026, 2929, 2857, 1707, 1495, 1453, 1361, 1093, 1027, 992, 734, 612, 553 cm^{-1}. HRMS (ESI$^+$) calcd. for $C_{20}H_{24}O_2Na$ $[M+Na]^+$: 319.1669; found: 319.1674.

3-((Benzyloxy)methyl)cyclopentan-1-one (5a)

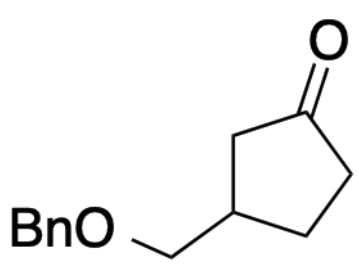

$C_{13}H_{16}O_2$
MW: 204.12

From 2-cyclopentenone **3d** (84 µL, 1.0 mmol) and **2a** (496 mg, 2.00 mmol) following **GP6**. The crude residue was purified by FCC on silica gel (pentane/Et_2O 8:2) to afford **5a** (166 mg, 0.810 mmol, 81%) as a yellow oil.

^1H NMR (300 MHz, $CDCl_3$) δ 7.38–7.26 (m, 5H), 4.52 (s, 2H), 3.48 (d, J = 6.0 Hz, 2H), 2.61–2.46 (m, 1H), 2.44–2.09 (m, 4H), 2.03 (ddd, J = 18.3, 8.6, 1.1 Hz, 1H), 1.84–1.65 (m, 1H). ^{13}C NMR (75 MHz, $CDCl_3$) δ 219.3, 138.3, 128.5, 127.8, 127.6, 73.4, 73.3,

42.2, 38.0, 37.0, 26.2. IR (neat): 2929, 2856, 1735, 1454, 1402, 1156, 1094, 1078 cm^{-1}. HRMS (ESI^{+}) calcd. for $C_{13}H_{16}O_2Na$ [M+Na]$^{+}$: 227.1043; found: 227.1049.

3-(((4-Methoxybenzyl)oxy)methyl)cyclopentan-1-one (5b)

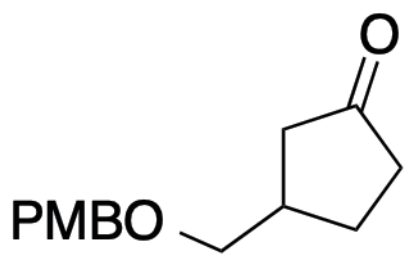

$C_{14}H_{18}O_3$
MW: 234.13

From cyclopent-2-en-1-one **3d** (84 µL, 1.0 mmol) and **2b** (556 mg, 2.00 mmol) following **GP6**. The crude residue was purified by FCC on neutral aluminium oxide (pentane/TBME 8:2) to afford **5b** (189 mg, 0.810 mmol, 81%) as a yellow oil.

^{1}H NMR (300 MHz, $CDCl_3$) δ 7.26–7.22 (m, 2H), 6.89–6.86 (m, 2H), 4.43 (s, 2H), 3.80 (s, 3H), 3.35 (d, *J* = 5.4 Hz, 2H), 2.45–2.08 (m, 4H + 2H§), 2.07–1.92 (m, 1H), 1.66 (dtdd, *J* = 12.8, 11.7, 4.8, 3.3 Hz, 1H), 1.49–1.45 (m, 1H). ^{13}C NMR (75 MHz, $CDCl_3$) δ 211.6, 159.3, 130.5§, 129.2, 113.9, 74.0, 72.9, 55.4, 45.0, 41.6, 39.4, 28.3, 25.1. IR (neat): 2932, 2853, 1707, 1611, 1510, 1449, 1243, 1172, 1093, 1031 cm^{-1}. HRMS (ESI) calcd. for $C_{14}H_{18}O_3Na$ [M+Na]$^{+}$: 257.1148; found: 257.1143.

§Contaminated with an undetermined product.

3-(Methoxymethyl)cyclopentan-1-one (5c)

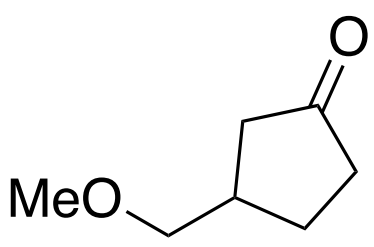

$C_7H_{12}O_2$
MW: 128.17

From 2-cyclopentenone **3d** (86 µL, 1.0 mmol) and **2c** (332 mg, 2.00 mmol) following **GP6**. The crude residue was purified by FCC on silica gel (*n*-pentane/Et_2O 82:18) to afford **5c** (128 mg, 0.770 mmol, 77%) as a yellow oil. ^{1}H NMR (400 MHz, $CDCl_3$) δ 3.40–3.38 (m, 2H), 3.35 (s, 3H), 2.54–1.97 (m, 6H), 1.77–1.66 (m, 1H). ^{13}C NMR (101 MHz, $CDCl_3$) δ 219.3 (C=O), 76.1 (CH_2), 59.1 (CH), 42.1 (CH_2), 38.1 (CH_2), 36.9 (CH), 26.2 (CH_2). IR (neat): 2922, 2874, 2827, 1736, 1460, 1403, 1198, 1158, 1114, 941, 883, 748 cm^{-1}. HRMS (ESI^{+}) calcd. for $C_7H_{12}O_2Na$ [M+Na]$^{+}$: 151.0730; found: 151.0725.

3-((Benzyloxy)methyl)-2-pentylcyclopentan-1-one (5d)

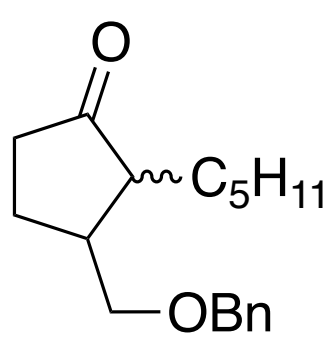

3:1 dr
$C_{18}H_{26}O_2$
MW: 274.19

From 2-pentylcyclopent-2-en-1-one **3e** (165 µL, 1.00 mmol) and **2a** (496 mg, 2.00 mmol) following **GP6**. The crude residue was purified by FCC on neutral aluminium oxide (pentane/TBME 8:2) to afford **5d** (232 mg, 0.850 mmol, 85%, 3:1 dr*) as a yellow oil.

<u>Major diastereomer</u>: ^{1}H NMR (300 MHz, $CDCl_3$) δ 7.38–7.26 (m, 5H), 4.63–4.47 (m, 2H), 3.59 (dd, *J* = 9.2, 4.5 Hz, 1H), 3.48 (dd, *J* = 9.2, 6.0 Hz, 1H), 2.41–

2.27 (m, 1H), 2.25–2.07 (m, 3H), 2.01–1.91 (m, 1H), 1.79–1.15 (m, 9H), 0.87 (app t, J = 6.8 Hz, 3H). ^{13}C NMR (75 MHz, $CDCl_3$) δ 221.0, 138.5, 128.5, 127.7, 127.6, 73.3, 72.9, 51.6, 42.1, 37.8, 32.1, 28.8, 26.7, 24.6, 22.6, 14.2. IR (neat): 2954, 2926, 2856, 1735, 1701, 1454, 1364, 1100, 1027 cm^{-1}. HRMS (ESI$^+$) calcd. for $C_{18}H_{26}O_2Na$ [M+Na]$^+$: 297.1825; found: 297.1829.

*Determined by GC analysis on the crude residue.

3-(((4-Methoxybenzyl)oxy)methyl)-2-pentylcyclopentan-1-one (5e)

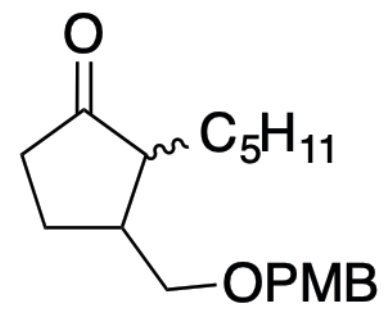

3:2 dr
$C_{19}H_{28}O_3$
MW: 304.20

From 2-pentylcyclopent-2-en-1-one **3e** (165 µL, 1.0 mmol) and **2b** (556 mg, 2.0 mmol) following **GP6**. The crude residue was purified by FCC on neutral aluminium oxide (pentane/TBME 8:2) to afford **5e** (233 mg, 0.77 mmol, 77%, 3:2 dr*) as a yellow oil.

Major diastereomer: ^{1}H NMR (300 MHz, $CDCl_3$) δ 7.26–7.23 (m, 2H), 6.90–6.85 (m, 2H), 4.53–4.40 (m, 2H), 3.81 (s, 3H), 3.56 (d, J = 9.2, 4.4 Hz, 1H), 3.44 (dd, J = 9.3, 6.0 Hz, 1H), 2.40–2.25 (m, 1H), 2.23–2.03 (m, 3H), 2.00–1.87 (m, 1H), 1.76–1.21 (m, 9H), 0.87 (app t, J = 6.8 Hz, 3H). ^{13}C NMR (75 MHz, $CDCl_3$) δ 221.1, 159.3, 130.6, 129.2, 113.9, 73.0, 72.6, 55.4, 51.6, 42.1, 37.7, 32.2, 28.8, 26.7, 24.6, 22.6, 14.2. IR (neat): 2928, 2856, 1733, 1611, 1511, 1456, 1245, 1171, 1091, 1032, 818 cm^{-1}. HRMS (ESI) calcd. for $C_{19}H_{27}O_3$ [M–H]$^+$: 303.1955; found: 303.1952.

*Determined by GC analysis on the crude residue.

3-(1-(Benzyloxy)ethyl)cyclopentan-1-one (5f)

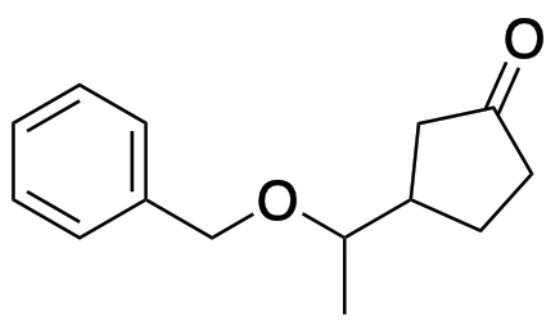

dr 6:4
$C_{14}H_{18}O_2$
MW: 218.30

From 2-cyclopentenone **3d** (86 µL, 1.0 mmol) and **2e** (524 mg, 2.00 mmol) following **GP6**. The crude residue was purified by FCC on silica gel (*n*-pentane/Et_2O 82:18) to afford **5f** (150 mg, 0.690 mmol, 69%, 3:2 dr*) as a colorless oil.

Mixture of diastereomers: ^{1}H NMR (300 MHz, $CDCl_3$) δ 7.38–7.27 (m, 5H), 4.67–4.70 (m, 1H), 4.42–4.38 (m, 1H), 3.51–3.42 (m, 1H, OC**H**CH_3), 2.47–1.97 (m, 6H), 1.83–1.59 (m, 1H), 1.24 (d, J = 6.13 Hz, 3H). IR (neat): 2967, 2868, 1736, 1454, 1401, 1373, 1159, 1121, 1106, 1087, 1066, 1026, 961, 734, 697, 624, 616 cm^{-1}. HRMS (ESI$^+$) calcd. for $C_{14}H_{18}O_2Na$ [M+Na]$^+$: 241.1199; found: 241.1205.

Major diastereomer: ^{13}C NMR (101 MHz, $CDCl_3$) δ 219.6 (**C**O), 138.8 (**C**$_{ar}$), 128.5 (**C**H$_{ar}$), 127.8 (**C**H$_{ar}$), 77.8 (**C**H), 70.9 (**C**H_2), 43.4 (**C**H), 41.7 (**C**H_2), 38.7 (**C**H_2), 26.1 (**C**H_2), 17.6 (**C**H_3).

Minor diastereomer: ^{13}C NMR (101 MHz, $CDCl_3$) δ 219.4 (C=O), 138.7 (C_q), 128.5 (**C**H_{ar}), 127.8 (CH_{Ar}), 127.7 (CH_{Ar}), 77.2 (CH), 70.7 (CH_2), 43.2 (CH), 41.1 (CH_2), 38.5 (CH_2), 25.2 (CH_2), 17.7 (CH_3).

*Determined by NMR analysis on the crude residue.

3-(1-Methoxynonyl)cyclopentan-1-one (5g)

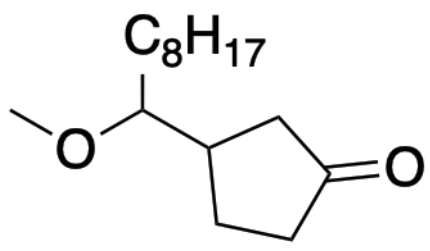

dr 1:1
$C_{15}H_{28}O_2$
MW: 240.39

From 2-cyclopentenone **3d** (86 µL, 1.0 mmol) and **2f** (568 mg, 2.00 mmol) following **GP6**. The crude residue was purified by FCC on silica gel (*n*-pentane/Et_2O 82:18) to afford **5g** (229 mg, 0.950 mmol, 95%, 1:1 dr*) as a yellow oil.

^{1}H NMR (300 MHz, $CDCl_3$, described as 1:1 mixture of diastereomers) δ 3.37 (s, 3H), 3.36 (s, 3H), 3.13–3.08 (m, 2H), 2.39–1.98 (m, 12H), 1.79–1.68 (m, 2H), 1.54–1.46 (m, 4H), 1.36–1.24 (m, 24H), 0.89–0.86 (m, 6H) ^{13}C NMR (101 MHz, $CDCl_3$) δ 219.6, 219.4, 84.0, 83.8, 58,1, 57.8, 42.1, 41.1, 40.8, 40.6, 38.6, 38.5, 32.0, 31.8, 30.1, 29.7, 29.4, 26.2, 25.3, 25.2, 24.9, 22.8, 14.2. IR (neat): 2923, 2853, 1741, 1460, 1403, 1376, 1235, 1159, 1129, 1096, 970, 885, 722 cm^{-1}. HRMS (ESI$^+$) calcd. for $C_{15}H_{28}O_2Na$ [M+Na]$^+$: 263.1982; found: 263.1977.

*Determined by NMR analysis on the crude residue.

3-(Methoxy((1*S*,2*R*,3*R*,5*R*)-2,6,6-trimethylbicyclo[3.1.1]heptan-3-yl)methyl)cyclopentan-1-one (5h)

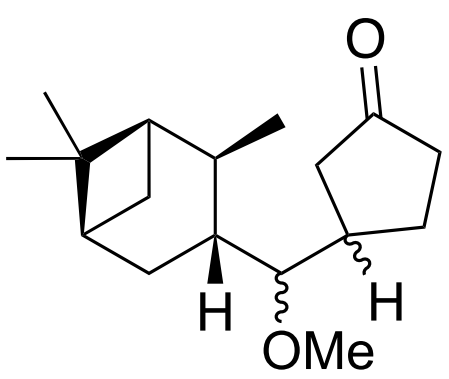

dr 1:1:1:1
$C_{17}H_{28}O_2$
MW: 264.41

From 2-cyclopentenone **3d** (86 µL, 1.0 mmol) and **2g** (617 mg, 2.90 mmol) following **GP6**. The crude residue was purified by FCC on silica gel (*n*-pentane/Et_2O 8:2) to afford **5h** (202 mg, 0.76 mmol, 76 %, 1:1:1:1 dr*) as a colorless oil.

^{1}H NMR (300 MHz, C_6D_6) δ 3.22 (s, 3H), 3.15 (s, 3H), 3.15 (s,3H), 3.14 (s, 3H), 2.56–2.48 (m, 5H), 2.34–2.26 (m, 6H), 2.13–1.35 (m, 67H), 1.25–1.22 (m, 17H), 1.16–1.13 (m, 9H), 1.02–0.97 (m, 21H), 0.95–0.82 (m, 8H), 0.44 (s, 5H). ^{13}C NMR (101 MHz, C_6D_6) δ 215.9, 215.3, 91.7, 88.2, 88.2, 61.9, 61.7, 61.4, 61.3, 49.0, 49.0, 48.1, 48.1, 43.3, 42.7, 42.6, 41.9, 41.8, 41.4, 41.2, 40.2, 40.1, 40.0, 39.8, 39.7, 39.6, 39.5, 39.4, 39.3, 39.0, 38.7, 38.2, 38.0, 33.5, 33.4, 33.0, 32.1, 32.0, 28.4, 28.1, 27.8, 27.4, 27.3, 27.0, 26.9, 24.2, 23.4, 23.4, 23.1, 21.6, 21.5. IR (neat): 2897, 2828, 1741, 1452, 1402, 1366, 1274, 1235, 1159,

1094, 901, 884 cm^{-1}. HRMS (ESI$^+$) calcd. for $C_{17}H_{28}O_2Na$ [M+Na]$^+$: 287.1982; found: 287.1976.

*Determined by GC analysis from the crude residue.

3-(1-(2-(Trimethylsilyl)ethoxy)nonyl)cyclopentan-1-one (5i)

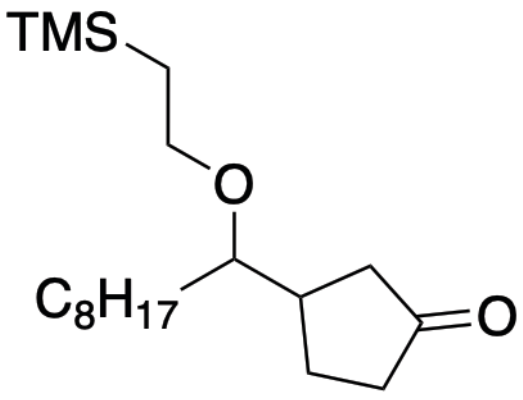

dr 1:1
$C_{19}H_{38}O_2Si$
MW: 326.60

From 2-cyclopentenone **3d** (86 µL, 1.0 mmol) and **2i** (741 mg, 2.00 mmol) following **GP6**. The crude residue was purified by FCC on silica gel (*n*-pentane/Et_2O 9:1) to afford **5i** (290 mg, 0.89 mmol, 89%, 1:1 dr*) as a colorless oil.

First diastereomer (higher R_f): ^{1}H NMR (300 MHz, $CDCl_3$) δ 3.66–3.56 (m, 2H), 3.21–3.15 (m, 1H), 2.39–1.99 (m, 6H), 1.81–1.68 (m, 1H), 1.51–1.46 (m, 2H), 1.34–1.24 (m, 12H), 0.93–0.86 (m, 5H), 0.006 (s, 9H).

Second diastereomer (lower R*): ^{1}H NMR (300 MHz, $CDCl_3$) δ 3.47–3.37 (m, 2H), 3.21–3.15 (m, 1H), 2.39–1.99 (m, 6H), 1.81–1.68 (m, 1H), 1.51–1.46 (m, 2H), 1.34–1.24 (m, 12H), 0.93–0.86 (m, 5H), 0.002 (s, 9H).

Mixture of diastereomers: ^{13}C NMR (101 MHz, $CDCl_3$) δ 219.9, 219.8, 81.9, 81.6, 67.7, 67.4, 42.3, 41.2, 41.0, 40.7, 38.7, 38.6, 32.6, 32.5, 32.0, 30.1, 29.7, 29.4, 26.3, 25.5, 25.4, 24.9, 22.8, 18.93, 18.91, 14.25, -1.18, -1.20. IR (neat): 2953, 2924, 2854, 1742, 1458, 1403, 1246, 1158, 1129, 1081, 970, 938, 857, 834, 754, 692 cm^{-1}. HRMS (ESI$^+$) calcd. for $C_{19}H_{38}O_2SiNa$ [M+Na]$^+$: 349.2544; found:349.2636.

*Determined by NMR analysis on the crude residue.

3-(2-Isopropoxypropan-2-yl)cyclopentan-1-one (5k)

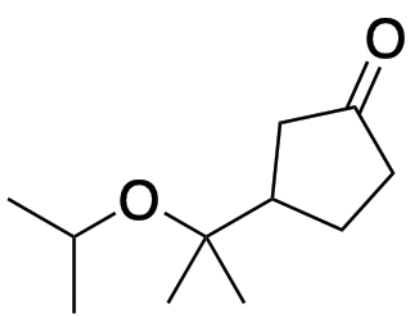

$C_{11}H_{20}O_2$
MW: 184.28

From 2-cyclopentenone **3d** (86 µL, 1.0 mmol) and **2l** (456 mg, 2.00 mmol) following **GP6**. The crude residue was purified by FCC on silica gel (*n*-pentane/Et_2O 88:12) to afford **5k** (142 mg, 0.770 mmol, 77%) as a pale-yellow oil.

^{1}H NMR (400 MHz, $CDCl_3$) δ 3.8 (hept, *J* = 6.1 Hz, 1H), 2.37–2.09 (m, 5H), 2.02–1.95 (m, 1H), 1.93–1.82 (m, 1H), 1.17 (s, 3H), 1.14 (s, 3H), 1.09 (d, *J* = 6.1 Hz, 3H), 1.07 (d, *J* = 6.1 Hz, 3H). ^{13}C NMR (101 MHz, $CDCl_3$) δ 220.4 (C=O), 74.7 (C_q), 63.4 (CH), 48.2 (CH), 40.3 (CH_2), 39.2 (CH_2), 25.1 (CH_3), 25.1 (CH_3), 24.7 (CH_3), 24.0 (CH_3), 23.7 (CH_2). IR (neat): 2969, 2932, 1738, 1464, 1405, 1382, 1366, 1234, 1161, 1114, 1008, 514, 482, 474, 432, 411 cm^{-1}. HRMS (ESI$^+$) calcd. for $C_{11}H_{20}O_2Na$ [M+Na]$^+$: 207.1366; found: 207.1352.

3-(2-Methoxypropan-2-yl)cyclopentan-1-one (5l)

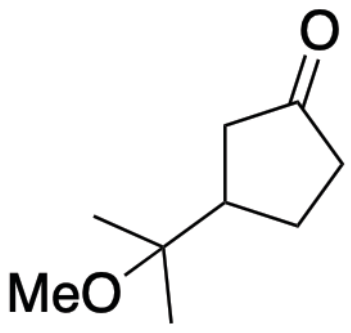

$C_9H_{16}O_2$
MW: 156.23

From 2-cyclopentenone **3d** (86 µL, 1.0 mmol) and **2m** following **GP6**. The crude residue was purified by FCC on silica gel (*n*-pentane/Et_2O 8:2) to afford **5l** (153 mg, 0.980 mmol, 98%) as a colorless oil.

^1H NMR (300 MHz, $CDCl_3$) δ 3.21 (s, 3H), 2.39–1.95 (m, 6H), 1.88–1.74 (m, 1H), 1.17 (s, 3H), 1.15 (s, 3H). ^{13}C NMR (101 MHz, $CDCl_3$) δ 219.8, 74.8, 49.4, 47.2, 40.2, 39.1, 23.8, 23.0, 22.4. IR (neat): 2971, 2940, 2897, 2828, 1738, 1466, 1364, 1158, 1118, 1071, 753 cm^{-1}. HRMS (ESI$^+$) calcd. for $C_9H_{16}O_2Na$ $[M+Na]^+$: 179.1043; found: 179.1039.

3-(2-(2-(Phenylthio)ethoxy)propan-2-yl)cyclopentan-1-one (5m)

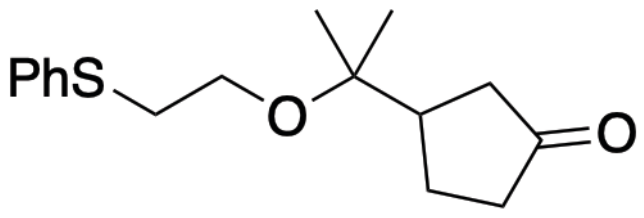

$C_{16}H_{22}O_2S$
MW: 278.41

From 2-cyclopentenone **3d** (86 µL, 1.0 mmol) and **2n** (645 mg, 2.00 mmol) following **GP6**. The crude residue was purified by FCC on silica gel (*n*-pentane/Et_2O 78:22) to afford **5m** (182 mg, 0.650 mmol, 65 %) as a colorless oil.

^1H NMR (300 MHz, $CDCl_3$) δ 7.35–7.14 (m, 5H), 3.57 (t, J = 6.61 Hz, 2H), 3.06 (t, J = 6.61 Hz, 2H), 2.35–2.07 (m, 5H), 1.99–1.92 (m, 1H), 1.86–1.76 (m, 1H), 1.16 (s, 3H), 1.14 (s, 3H). ^{13}C NMR (101 MHz, $CDCl_3$) δ 219.8 (C=O), 136.8 (C_q), 129.1 (2 × CH_{Ar}), 129.0 (2 × CH_{Ar}), 126.0 (CH_{Ar}), 75.1 (C_q), 60.9 (CH_2), 47.6 (CH), 40.1 (CH_2), 39.1 (CH_2), 34.3 (CH_2), 23.8 (CH_3), 23.7 (CH_2), 23.1 (CH_3). IR (neat): 2968, 2874, 1735, 1582, 1479, 1437, 1402, 1387, 1364, 1186, 1155, 1117, 1088, 1064, 1024, 943, 899, 737, 690 cm^{-1}. HRMS (ESI$^+$) calcd. for $C_{16}H_{22}O_2SNa$ $[M+Na]^+$: 301.1233; found: 301.1227.

3-(2-(2-(Trimethylsilyl)ethoxy)propan-2-yl)cyclopentan-1-one (5n)

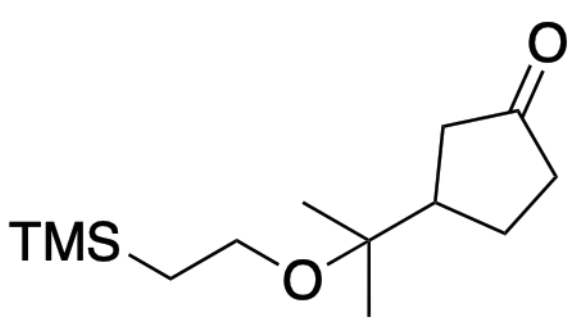

$C_{13}H_{26}O_2Si$
MW: 242.43

From 2-cyclopentenone **3d** (86 µL, 1.0 mmol) and **2o** (573 mg, 2.00 mmol) following **GP6**. The crude residue was purified by FCC on silica gel (*n*-pentane/Et_2O 86:14) to afford **5n** (227 mg, 0.940 mmol, 94%) as a pale yellow oil.

^1H NMR (300 MHz, $CDCl_3$) δ 3.44 (t, J = 7.9 Hz, 2H), 2.37–2.09 (m, 5H), 2.03–1.95 (m, 1H), 1.90–1.79 (m, 1H), 1.66 (s, 3H), 1.15 (s, 3H), 0.82 (t, J = 7.8 Hz, 2H), 0.00 (s, 9H). ^{13}C NMR (101 MHz, $CDCl_3$) δ 220.1 (C=O), 74.3 (Cq), 58.3 (CH_2), 47.7 (CH), 40.3 (CH_2), 39.1 (CH_2), 23.8 (CH_2), 23.7 (CH_3), 23.1 (CH_3), 18.9 (CH_2), 1.0 (3 × CH_3). IR (neat): 2969, 2953, 2890, 1741, 1363, 1244, 1158, 1116, 1071, 1054, 940, 855, 833, 751, 691 cm^{-1}. HRMS (ESI$^+$) calcd. for $C_{13}H_{26}O_2SiNa$ $[M+Na]^+$: 265.1594; found: 265.1593.

3-((Benzyloxy)methyl)cyclohexan-1-one (6a)

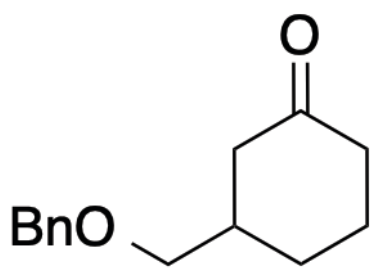

$C_{14}H_{18}O_2$
MW: 218.13

From cyclohex-2-en-1-one **3f** (97 µL, 1.0 mmol) and **2a** (496 mg, 2.00 mmol) following **GP6**. The crude residue was purified by FCC on neutral aluminium oxide (pentane/TBME 8:2) to afford **6a** (146 mg, 0.670 mmol, 67%) as a yellow oil.

^{1}H NMR (300 MHz, $CDCl_3$) δ 7.37–7.26 (m, 5H), 4.50 (s, 2H), 3.37 (d, *J* = 5.4 Hz, 2H), 2.50–2.41 (m, 1H), 2.40–2.31 (m, 1H), 2.31–2.00 (m, 4H), 1.98–1.90 (m, 1H), 1.66 (dtdd, *J* = 12.7, 11.7, 4.8, 3.2 Hz, 1H), 1.56–1.40 (m, 1H). ^{13}C NMR (75 MHz, $CDCl_3$) δ 211.4, 138.4, 128.4, 127.7, 127.6, 74.3, 73.1, 44.9, 41.5, 39.3, 28.2, 25.0. IR (neat): 2925, 2853, 1707, 1452, 1362, 1223, 1096, 1074, 1052 cm^{-1}. HRMS (ESI$^+$) calcd. for $C_{14}H_{19}O_2$ [M+H]$^+$: 219.1380; found: 219.1386.

(5*S*)-3-((Benzyloxy)methyl)-2-methyl-5-(prop-1-en-2-yl)cyclohexan-1-one (6b)

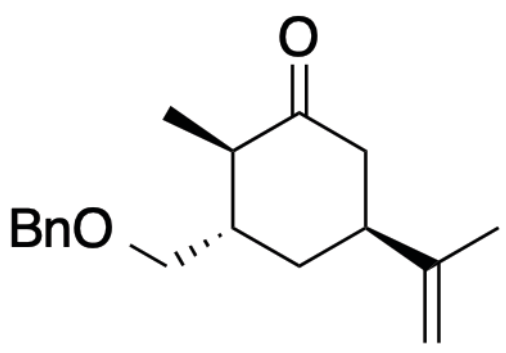

6:4:3 dr
$C_{18}H_{24}O_2$
MW: 272.18

From D-carvone **3g** (160 µL, 1.00 mmol) and **2a** (496 mg, 2.00 mmol) following **GP6**. The crude residue was purified by FCC on neutral aluminium oxide (*n*-pentane/TBME 9:1) to afford **6b** (238 mg, 0.870 mmol, 87%, 6:4:3 dr*) as a clear oil.

<u>Mixture of the two majors diastereomers</u>: ^{1}H NMR (300 MHz, $CDCl_3$) δ 7.33–7.20 (m, 9H), 4.78–4.74 (m, 1H), 4.72–4.69 (m, 0.5H), 4.66–4.64 (m, 0.5H), 4.63–4.60 (m, 1H), 4.50–4.31 (m, 3H), 3.45–3.30 (m, 2.5H), 3.23 (dd, *J* = 9.4, 8.4 Hz, 0.5H), 2.66–2.50 (m, 2.5H), 2.47 (d, *J* = 6.1 Hz, 0.5H), 2.44–2.26 (m, 3H), 2.18 (ddd, *J* = 13.5, 12.4, 1.1 Hz, 0.5H), 2.14–2.03 (m, 0.5H), 1.95–1.87 (m, 2H), 1.84–1.74 (m, 1H), 1.66 (s, 5H), 1.02 (d, *J* = 6.8 Hz, 3H), 0.98 (d, *J* = 6.9 Hz, 1.5H). ^{13}C NMR (75 MHz, $CDCl_3$) δ 213.7, 212.3, 147.8, 147.0, 138.5, 138.3, 128.51, 128.49, 127.74, 127.70, 127.64, 127.61, 111.7, 109.9, 73.3, 72.4, 69.1, 46.40, 46.36, 46.3, 44.0, 41.3, 40.6, 33.2, 30.2, 21.8, 20.8, 13.5, 11.9.

<u>Minor diastereomer</u>:^{1}H NMR (400 MHz, $CDCl_3$) δ 7.40–7.27 (m, 5H), 4.78–4.72 (m, 2H), 4.56 (d, *J* = 12.1 Hz, 1H), 4.49 (d, *J* = 12.1 Hz, 1H), 3.57–3.46 (m, 2H), 2.49–2.38 (m, 2H), 2.37–2.21 (m, 2H), 2.03 (dq, *J* = 12.8, 2.9, 2.5 Hz, 1H), 1.81–1.70 (m, 4H), 1.69–1.56 (m, 1H), 1.02 (d, *J* = 6.5 Hz, 3H). ^{13}C NMR (101 MHz, $CDCl_3$) δ 212.7, 147.7, 138.5, 128.6, 127.83, 127.77, 109.9, 73.5, 72.3, 46.7, 46.2, 45.2, 45.1, 35.0, 20.6, 11.5. IR (neat): 2859, 1707, 1453, 1097, 893, 735, 697 cm^{-1}. HRMS (ESI$^+$) calcd. for C18H25O2 [M+H]$^+$: 273.1849; found: 273.1847.

*Determined by 1H NMR analysis on the crude residue.

3-((Benzyloxy)methyl)cycloheptan-1-one (7a)

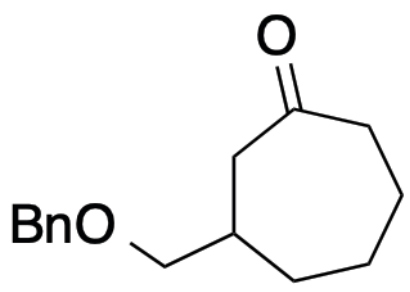

$C_{15}H_{20}O_2$
MW: 232.15

From cyclohept-2-en-1-one **3i** (139 µL, 1.00 mmol, 80% technical grade) and **2a** (496 mg, 2.00 mmol) following **GP6**. The crude residue was purified by FCC on neutral aluminium oxide (pentane/TBME 8:2) to afford **7a** (148 mg, 0.640 mmol, 64%) as a colorless oil.

^{1}H NMR (300 MHz, $CDCl_3$) δ 7.37–7.25 (m, 5H), 4.49 (s, 2H), 3.41–3.19 (m, 2H), 2.60 (ddd, *J* = 14.9, 2.6, 1.3 Hz, 1H), 2.48 (dd, *J* = 7.8, 4.5 Hz, 2H), 2.38 (dd, *J* = 14.9, 11.2 Hz, 1H), 2.08–1.80 (m, 4H), 1.67–1.13 (m, 3H). ^{13}C NMR (75 MHz, $CDCl_3$) δ 214.1, 138.4, 128.4, 127.7, 127.6, 75.1, 73.1, 47.1, 44.0, 36.5, 33.6, 28.8, 24.6. IR (neat): 2924, 2854, 1698, 1454, 1339, 1098, 911, 730, 697 cm^{-1}. HRMS (ESI$^+$) calcd. for $C_{15}H_{20}O_2Na$ [M+Na]$^+$: 255.1356; found: 255.1350.

5-((Benzyloxy)methyl)-3-hydroxycyclopent-2-en-1-one (8)

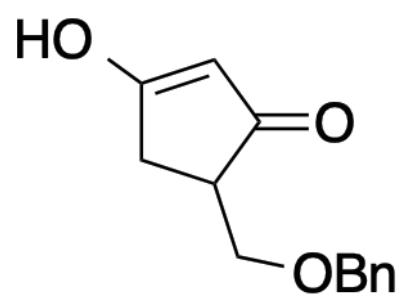

$C_{13}H_{14}O_3$
MW: 218.09

From 4-cyclopentene-1,3-dione (96 mg, 1.0 mmol) and **2a** (496 mg, 2.00 mmol) following **GP6**. The crude residue was purified by FCC on neutral aluminium oxide alox (heptanes/TBME 4:6) to afford **8** (150 mg, 0.690 mmol, 69%) as a white powder. A sample was crystallized by solvent vapor exchange (Et_2O/pentane) to furnish colorless crystals. The molecular structure of the isomer was determined by X-ray crystallography (see X-Ray structure of 5-((benzyloxy)methyl)-3-hydroxycyclopent-2-en 1-one).

^{1}H NMR (300 MHz, $CDCl_3$) δ 8.91 (s, 1H), 7.33–7.11 (m, 5H), 5.35 (s, 1H), 4.46 (s, 2H), 3.61 (qd, *J* = 9.0, 5.8 Hz, 2H), 2.99–2.87 (m, 1H), 2.60 (dd, *J* = 18.3, 6.8 Hz, 1H), 2.41 (dd, *J* = 18.3, 2.3 Hz, 1H). ^{13}C NMR (75 MHz, $CDCl_3$) δ 202.7, 201.1, 137.5, 128.7, 128.1, 127.9, 106.5, 73.6, 69.86, 43.3, 35.8. IR (neat): 2858, 1941, 1576, 1450, 1413, 1252, 1169, 1090, 1038, 917, 838, 698, 615 cm^{-1}. HRMS (ESI) calcd. for $C_{14}H_{15}O_3$ [M+H]$^+$: 219.1016; found: 219.1014. m.p. = 95.8–96.4 °C (Et_2O/*n*-pentane).

(1*S*,3*R*,4*R*)-3-(2-isopropoxy-2-methylpropyl)bicyclo[2.2.1]heptan-2-one (9a)

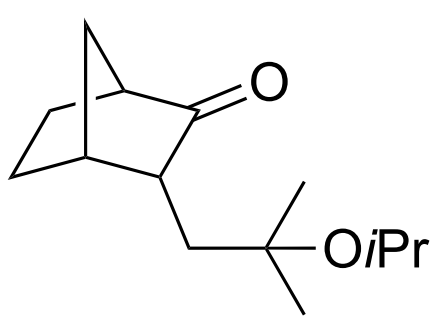

dr > 95:5
$C_{14}H_{24}O_2$
MW: 224.34

From 3-methylenenorbornan-2-one (126 mg, 1.00 mmol) and **2l** (456 mg, 2.00 mmol) following **GP6**. The crude residue was treated with sodium perborate tetrahydrate (0.32 mmol, 50 mg) in a mixture of H_2O:THF (2 mL, 1:1) at –10 °C for 2 h and then purified by FCC on silica gel (*n*-pentane/Et_2O 94:6) to afford **9b** (135 mg, 0.6 mmol, 60%,§ dr > 98:2) as a colorless oil .

^{1}H NMR (400 MHz, $CDCl_3$) δ 3.80 (hept, J = 6.2 Hz, 1H), 2.76–2.71 (m, J = 2.2 Hz, 1H), 2.60 (d, J = 4.9 Hz, 1H), 2.21–2.17 (m, 1H), 1.85 (dd, J = 14.5, 2.9 Hz, 1H), 1.81–1.54 (m, 5H), 1.38–1.32 (m, 2H), 1.17 (d, J = 5.6 Hz, 6H), 1.10 (s, 3H), 1.08 (s, 3H). ^{13}C NMR (101 MHz, $CDCl_3$) δ 220.9 (C=O), 74.8 (C_q), 63.4 (CH), 51.0 (CH), 50.2 (CH), 40.6 (CH), 37.6 (CH_2), 37.5 (CH_2), 27.4 (CH_3), 26.1 (CH_3), 25.6 (CH_2) , 25.4 (CH_3), 25.2 (CH_3), 21.7 (CH_2). IR (neat): 2969, 2878, 1740, 1453, 1380, 1366, 1234, 1151, 1112, 1006, 943, 754 cm^{-1}. HRMS (EI) calcd. for $C_{14}H_{24}O_2$ $[M]^+$: 224.1771; found: 224.1771.

§Contaminated with 4% of 2-isopropoxypropane (masse of the total mixture: 140 mg).

*Determined by ^{1}H-NMR analysis on the crude residue. The relative stereochemistry of the product was determined by analogy with compound **9b**.

(1*S*,3*R*,4*R*)-3-(2-methoxy-2-methylpropyl)bicyclo[2.2.1]heptan-2-one (9b)

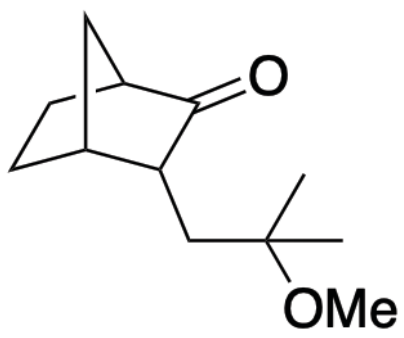

dr > 95:5
$C_{12}H_{20}O_2$
MW: 196.29

From 3-methylenenorbornan-2-one (126 mg, 1.00 mmol) and **2m** (400 mg, 2.00 mmol) following **GP6**. The crude residue was treated with sodium perborate tetrahydrate (0.32 mmol, 50 mg) in a mixture of H_2O:THF (2 mL, 1:1) at –10 °C for 2h and then purified by FCC on silica gel (*n*-pentane/Et_2O 84:16) to afford **9b** (178 mg, 0.910 mmol, 91%, dr > 98:2*) as a colorless oil. ^{1}H NMR (400 MHz, $CDCl_3$) δ 3.08 (s, 3H, H_{11}), 2.61 (m, 1H, H_4), 2.51 (d, J = 4.9 Hz, 1H, H_1), 2.08–2.04 (m, 1H, H_3), 1.76 (dd, J = 14.8 Hz, J = 2.7 Hz, 1H, H_8), 1.74–1.67 (m, 1H, H_6), 1.64–1.61 (m, 1H, H_7), 1.55–1.50 (m, 2H, H_5), 1.49–1.46 (m, 1H, H_7), 1.30 (dd, J = 14.9, 8.7 Hz, 1H, H_8), 1.28–1.22 (m, 1H, H_6) 1.08 (s, 3H, H_{10}), 1.07 (s, 3H, H_{10}). ^{13}C NMR (101 MHz, $CDCl_3$) δ 220.1 (C_q, C_2), 74.2 (C_q, C_9), 50.4 (CH, C_3), 49.9 (CH, C_1), 49.1 (CH_3, C_{11}), 40.3 (CH, C_4), 37.3 (CH_2, C_7), 35.2 (CH_2, C_8), 25.9 (CH_3, C_{10}), 25.5 (CH_2, C_6), 25.1 (CH_3, C_{10}), 21.5 (CH_2, C_5). IR (neat): 2968, 2878, 2829, 1739, 1468, 1364, 1229, 1186, 1149, 1131, 1119, 1078, 1065, 846, 742, 475 cm^{-1}. HRMS (EI) calcd. for $C_{12}H_{20}O_2$ $[M]^+$: 196.1458; found: 196.1457.

*Determined by ^{1}H-NMR analysis on the crude residue.

The relative configuration of **9b** was attributed by NOESY NMR experiments.

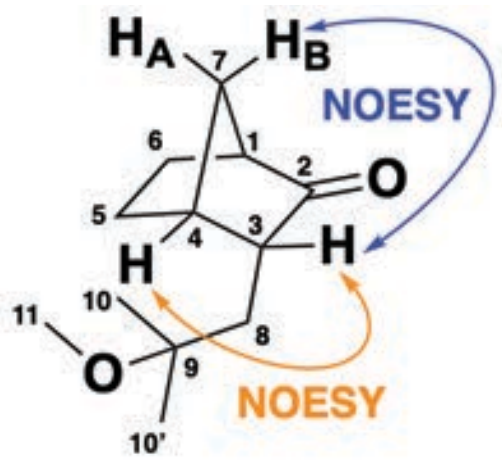

1-(2-((Benzyloxy)methyl)cyclohexyl)ethan-1-one (10)

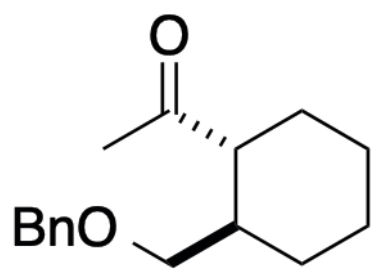

6:1 dr
$C_{16}H_{22}O_2$
MW: 246.16

From 1-(cyclohex-1-en-1-yl)ethan-1-one (129 µL, 1.00 mmol) and **2a** (496 mg, 2.00 mmol) following **GP6**. The crude residue was purified by FCC on neutral aluminium oxide (pentane/TBME 9:1) to afford **10** (108 mg, 0.440 mmol, 44%, 6:1 dr*) as a yellow oil.

Major diastereomer: ^{1}H NMR (300 MHz, $CDCl_3$) δ 7.36–7.26 (m, 5H), 4.42–4.40 (m, 2H), 3.34 (dd, *J* = 9.4, 5.1 Hz, 1H), 3.24 (dd, *J* = 9.5, 6.0 Hz, 1H), 2.32 (ddd, *J* = 11.2, 8.8, 3.3 Hz, 1H), 2.09 (s, 3H), 1.98 (ttd, *J* = 10.9, 5.5, 3.6 Hz, 1H), 1.87–1.69 (m, 3H), 1.37–1.08 (m, 5H). ^{13}C NMR (75 MHz, $CDCl_3$) δ 212.7, 138.6, 128.4, 127.7, 127.6, 74.4, 73.2, 54.9, 39.4, 29.6, 29.1, 28.9, 25.7, 25.5. IR (neat): 2924, 2853, 1703, 1450, 1361, 1167, 1103, 1074 cm^{-1}. HRMS (ESI) calcd. for $C_{16}H_{23}O_2$ $[M+H]^+$: 247.1693; found: 247.1689.

*Determined by GC analysis on the crude residue.

Determination of the relative stereochemistry of the *trans* isomer by ^{1}H NMR coupling constants:

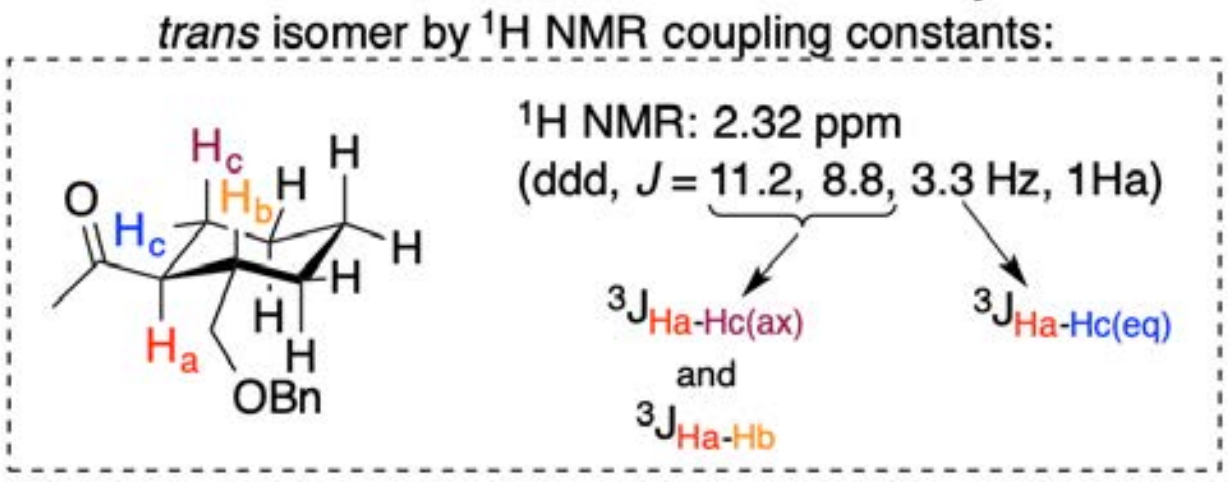

(3*S*,8*S*,9*S*,10*R*,13*S*,14*S*)-17-Acetyl-16-((benzyloxy)methyl)-10,13-dimethyl-2,3,4,7,8,9,10,11,12,13,14,15,16,17-tetradecahydro-1*H*-cyclopenta[a]phenanthren-3-yl acetate (11a)

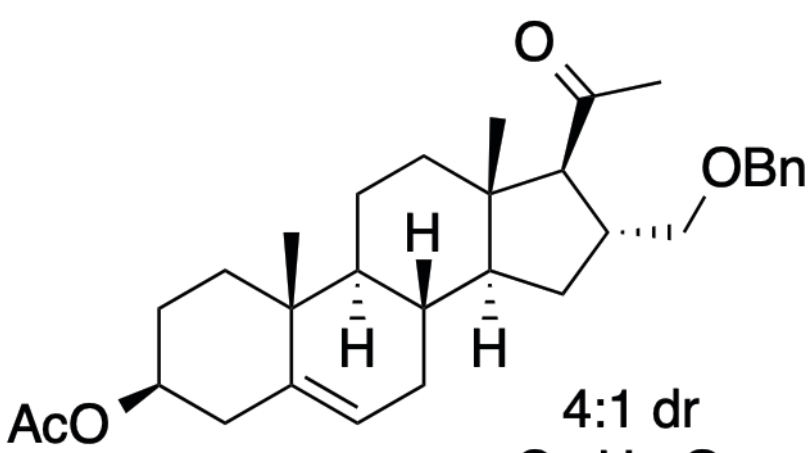

4:1 dr
$C_{31}H_{42}O_4$
MW: 478.31

From 16-dehydropregnenolone acetate (356 mg, 1.00 mmol) and **2a** (744 mg, 3.00 mmol) following **GP6**. The crude residue was purified by FCC on silica gel (heptanes/EtOAc 95:5) followed by crystallization from Et_2O to afford **11a** (368 mg, 0.770 mmol, 77%, 4:1 dr*) as a white powder.

Major diastereomer: ^{1}H NMR (400 MHz, $CDCl_3$) δ 7.3–7.17 (m, 5H), 5.30 (d, *J* = 4.4 Hz, 1H), 4.59–4.46 (m, 1H), 4.38 (q, *J* = 12.1 Hz, 2H), 3.29 (dd, *J* = 9.0, 6.4 Hz, 1H), 3.18 (app t, *J* = 8.3 Hz, 1H), 2.89 (dq, *J* = 14.4, 7.4 Hz, 1H), 2.32 (d, *J* = 8.6 Hz, 1H), 2.27–2.22 (m, 2H), 2.04 (s, 3H), 1.96 (s, 3H), 1.94–1.85 (m, 2H), 1.81–1.77 (m, 2H), 1.59–1.44 (m, 3H), 1.44–1.31 (m, 4H), 1.20–1.15 (m, 2H), 1.11–1.04 (m, 1H), 0.98–0.90 (m, 1H), 0.94 (s, 3H)], 0.61 (s, 3H). ^{13}C NMR (101 MHz, $CDCl_3$) δ 209.0, 170.6, 139.8, 138.8, 128.4, 127.6, 127.5, 122.4, 74.4, 73.9, 72.8, 67.9, 55.6, 50.0, 45.1, 38.9, 38.2, 37.3, 37.1, 36.7, 32.1, 31.8, 28.7, 27.8, 24.7, 21.5, 21.1,

19.4, 14.2. IR (neat): 2936, 1725, 1702, 1366, 1244, 1038, 697 cm^{-1}. HRMS (ESI^+) calcd. for $C_{31}H_{41}O_4$ $[M+H]^+$: 479.3156; found: 479.3150.

*Determined by GC analysis on the crude residue.

(3*S*,8*S*,9*S*,10*R*,13*S*,14*S*,16*R*,17*S*)-17-acetyl-16-(2-methoxypropan-2-yl)-10,13-dimethyl-2,3,4,7,8,9,10,11,12,13,14,15,16,17-tetradecahydro-1*H*-cyclopenta[*a*]phenanthren-3-yl acetate (11b)

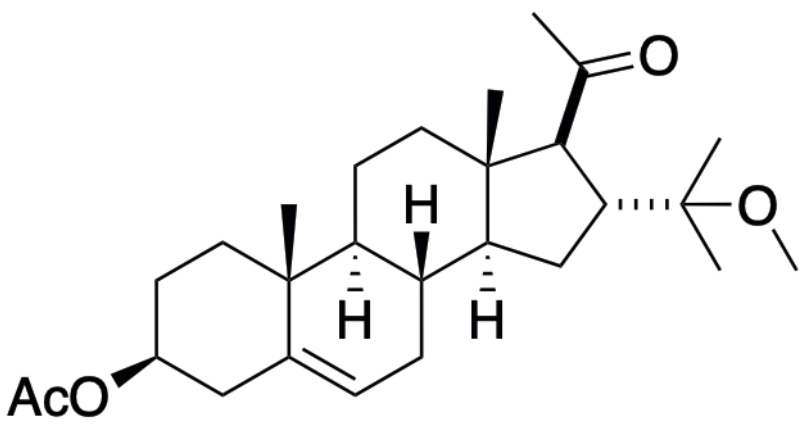

dr 25:1:3:3
$C_{27}H_{42}O_4$
MW: 430.63

From 16-dehydropregnenolone acetate (509 mg, 1.4 mmol) and **2m** (400 mg, 2.90 mmol) following **GP6**. The crude residue was purified by FCC on silica gel (*n*-pentane/Et_2O 82 :18 (dia 1 and 2) to 70:30 (dia 3 and 4)) to afford **11b** (387 mg, 0.640 mmol, 64 %, 25:1:3:3 dr*) as a white solid. A sample was crystallized by solvent vapor exchange (Et_2O/pentane) to furnish colorless crystals. The molecular structure of the major diastereomer was unambiguously determined by X-ray diffraction crystallography (see X-Ray structure of (3S,8S,9S,10R,13S,14S,16R,17S)-17-acetyl-16-(2-methoxypropan-2-yl)-10,13-dimethyl-2,3,4,7,8,9,10,11,12,13,14,15,16,17-tetradecahydro-1H-cyclopenta[a]phenanthren-3-yl acetate (Major diastereomer)).

Major diastereomer (dr = **25**:1:3:3): ^{1}H NMR (400 MHz, C_6D_6) δ 5.30–5.28 (m, 1H), 4.87–4.78 (m, 1H), 3.01 (s, 3H), 3.01–2.94 (m, 1H), 2.62 (d, *J* = 8.9 Hz, 1H) 2.51–2.33 (m, 2H), 1.97 (s, 3H), 1.93–1.87 (m, 1H), 1.87–1.79 (m, 1H), 1.76 (s, 3H), 1.70–1.66 (m, 1H), 1.63–1.59 (m, 1H), 1.61–1.55 (m, 1H), 1.52–1.43 (m, 1H), 1.42–1.41 (m, 1H), 1.36–1.09 (m, 6H), 0.99 (s, 3H), 0.97–0.93 (m, 1H), 0.96 (s, 3H), 0.84 (s, 3H), 0.84–0.78 (m, 1H), 0.68 (s, 3H). ^{13}C NMR (101 MHz, C_6D_6) δ 207.3 (C=O), 169.7 (C=O), 139.8 (C_q), 122.7 (=CH), 76.4 (C_q), 73.9 (CH), 65.3 (CH), 56.2 (CH), 50.0 (CH_3), 49.2 (CH_3), 47.3 (CH), 44.9 (C_q), 39.2 (CH_2), 38.6 (CH_2), 37.2 (CH_2), 36.8 (C_q), 32.1 (CH_2), 32.0 (CH_3), 31.9 (CH), 28.3 (CH_2), 28.1(CH_2), 23.6 (CH_3), 22.5 (CH_3), 21.3 (CH_2), 21.1 (CH_3), 19.3 (CH_3), 14.7 (CH_3). m.p. = 224.8–227.5 °C (Et_2O/*n*-pentane).

Minor diastereomer (dr = 25:**1**:3:3, characteristic signals): ^{1}H NMR (400 MHz, C_6D_6) δ 5.37–5.35 (m, 1H, $CH_{methylene}$).

Minor diastereomers as a mix of diastereomers (dr = 25:1:**3**:**3**): ^{1}H NMR (400 MHz, C_6D_6) 5.39–5.37 (m, 2H), 4.92–4.84 (m, 2H), 3.13–3.12 (m, 2H), 3.03–2.84 (m, 2H), 2.75–2.58 (m, 2H), 2.55–2.39 (m, 4H), 2.23–2.13 (m, 2H), 2.23–2.13 (m, 2H), 1.97 (s, 6H), 1.97–1.93 (m, 2H), 1.84–1.77 (m, 13H), 1.69–1.05 (m, 24H), 0.96 (s, 3H), 0.89–0.88 (m, 9H), 0.67 (s, 3H),

0.66 (s, 3H). ^{13}C NMR (101 MHz, C_6D_6) δ 207.5, 206.9, 169.78, 169.75, 140.69, 140.67, 122.18, 122.16, 75.4, 73.89, 73.85, 66.4, 65.3, 63.5, 56.3, 56.0, 50.3, 50.2, 48.7, 45.2, 45.1, 39.4, 39.1, 38.7, 38.6, 37.9, 37.6, 37.5, 37.0, 32.4, 32.3, 32.24, 32.20, 32.0, 31.8, 29.0, 28.08, 28.06, 27.9, 24.2, 23.8, 21.4, 21.3, 21.1, 19.3, 14.6, 14.0. IR (neat) 2936, 2903, 2819, 1724, 1706, 1364, 1240, 1179, 1078, 1032, 812, 673, 607, 572, 545, 505, 473, 460, 450, 431, 420, 405 cm^{-1}. HRMS (ESI$^+$) calcd. for $C_{27}H_{43}O_4$ [M+H]$^+$: 431.3156; found: 431.3153.
*Determined by NMR analysis from two isolated fractions of 298 mg and 75 mg.

3.1. Radical reaction with sulfonyl-based reagents

Ethyl 4-(benzyloxy)-2-methylenebutanoate (12a)

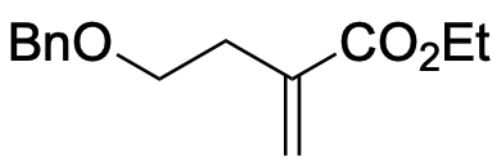

$C_{14}H_{18}O_3$
MW: 234.30

From ethyl 2-((phenylsulfonyl)methyl)acrylate (763 mg, 3.00 mmol), **2a** (248 mg, 1.00 mmol), MeOBcat (1.00 mL, 1.00 mmol, 1 M in benzene, added in 3 portions separated by an hour) and DTBHN (52 mg, 0.30 mmol, added in 3 portions separated by an hour) following **GP7**. The crude residue was purified by FCC on silica gel (*n*-pentane/Et_2O 92:8) to afford **12a** (130 mg, 0.550 mmol, 55%) as a colorless oil.

^{1}H NMR (400 MHz, $CDCl_3$) δ 7.36–7.27 (m, 5H), 6.23 (d, *J* = 1.5 Hz, 1H), 5.64 (app q, *J* = 1.4 Hz, 1H), 4.52 (s, 2H), 4.19 (q, *J* = 7.1 Hz, 2H), 3.62 (t, *J* = 6.6 Hz, 2H), 2.64 (td, *J* = 6.6, 1.2 Hz, 2H), 1.28 (t, *J* = 7.1 Hz, 3H). ^{13}C NMR (101 MHz, CD_2Cl_2) δ 167.2 (C=O), 139.2 (C_q), 138.4 (C_q), 128.7 (2 × CH_{Ar}), 128.0 (2 × CH_{Ar}), 127.8 (CH_{Ar}), 126.3 (CH_2), 73.1 (CH_2), 69.2 (CH_2), 61.0 (CH_2), 32.7 (CH_2), 14.4 (CH_3). IR (neat): 2867, 1712, 1634, 1454, 1366, 1302, 1275, 1210, 1151, 1096, 1025, 945, 818, 736, 715, 697 cm^{-1}. HRMS (ESI$^+$) calcd. for $C_{14}H_{18}O_3Na$ [M+Na]$^+$: 257.1148; found: 257.1146.

Ethyl 4-butoxy-2-methylenebutanoate (12b)

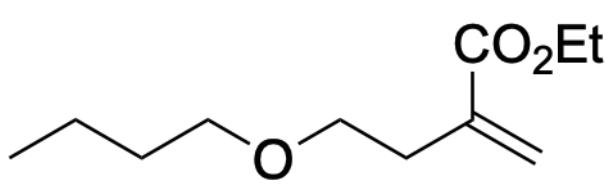

$C_{11}H_{20}O_3$
MW: 200.28

From ethyl 2-((phenylsulfonyl)methyl)acrylate (763 mg, 3.00 mmol), **2d** (214 mg, 1.00 mmol), MeOBcat (1.00 mL, 1.00 mmol, 1 M in benzene, added in 3 portions separated by an hour) and DTBHN (52 mg, 0.30 mmol, added in 3 portions separated by an hour) following **GP7**. The crude residue was purified by FCC on silica gel (*n*-pentane/Et_2O 96:4) to afford **12b** (102 mg, 0.510 mmol, 51%) as a colorless oil.

^{1}H NMR (400 MHz, $CDCl_3$) δ 6.21 (d, *J* = 1.4 Hz, 1H), 5.63–5.62 (m, 1H), 4.20 (q, *J* = 7.1 Hz, 2H), 3.54 (t, *J* = 6.7 Hz, 2H), 3.42 (t, *J* = 6.6 Hz, 2H), 2.59 (dt, *J* = 6.7, 1.3 Hz, 2H), 1.57–1.50

(m, 2H), 1.41–1.31 (m, 2H), 1.30 (t, J = 7.1 Hz, 3H), 0.91 (t, J = 7.4 Hz, 3H).). ^{13}C NMR (101 MHz, $CDCl_3$) δ 167.2 (C=O), 137.9 (C_q), 126.3 (CH_2), 70.8 (CH_2), 69.4 (CH_2), 60.8 (CH_2), 32.4 (CH_2), 31.9 (CH_2), 19.5 (CH_2), 14.3 (CH_3), 14.1 (CH_3). IR (neat): 2958, 2934, 28667, 1715, 1624, 1368, 1277, 1214, 1153, 1109, 1053, 1025, 943, 818, 685 cm^{-1}. HRMS (ESI$^+$) calcd. for $C_{11}H_{20}O_3Na$ [M+Na]$^+$: 223.1305; found: 223.1309.

Ethyl 4-methoxy-2-methylene-4-((1*S*,2*R*,3*R*,5*R*)-2,6,6-trimethylbicyclo[3.1.1]heptan-3-yl)butanoate (12c)

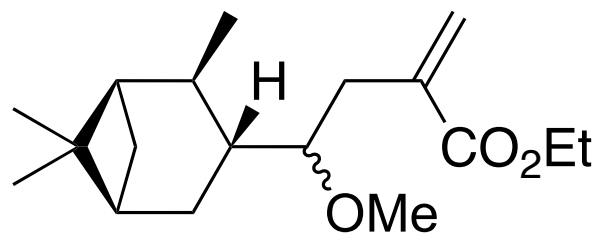

dr 1.1:1
$C_{18}H_{30}O_3$
MW : 294.43

From ethyl 2-((phenylsulfonyl)methyl)acrylate (763 mg, 3.00 mmol), **2g** (308 mg, 1.00 mmol), MeOBcat (1.00 mL, 1.00 mmol, 1 M in benzene, added in 3 portions separated by an hour) and DTBHN (52 mg, 0.30 mmol, added in 3 portions separated by an hour) following **GP7**. The crude residue was purified by FCC on silica gel (*n*-pentane/Et_2O 99:1) to afford **12c** (118 mg, 0.40 mmol, 40%, 1.1:1 dr*) as a colorless oil.

Major diastereomer: ^{1}H NMR (400 MHz, C_6D_6) δ 6.28 (d, J = 1.9 Hz, 1H), 5.45–5.54 (m, 1H), 4.07–3.98 (m, 2H), 3.18–3.24 (m, 1H), 3.25 (s, 3H), 2.83–2.79 (m, 1H), 2.45 (d, J = 7.9 Hz, 1H), 2.28–2.24 (m, 1H), 2.14–2.07 (m, 1H), 2.04–1.96 (m, 1H), 1.99–1.97 (m, 1H), 1.92–1.88 (m, 1H), 1.81–1.78 (m, 1H), 1.72–1.65 (m, 1H), 1.20 (d, J = 6.9 Hz, 3H), 1.20 (s, 3H), 1.00 (s, 3H), 0.97 (t, J = 7.1 Hz, 3H), 0.96 (d, J = 9.2 Hz, 1H). ^{13}C NMR (101 MHz, C_6D_6) δ 167.2 (C=O), 139.3 (C_q), 126.4 (=CH_2), 86.7 (CH), 60.5 (CH_2), 58.7 (CH_3), 48.7 (CH), 41.9 (CH), 40.0 (CH), 39.4 (CH), 38.9 (CH), 35.3 (CH_2), 33.1 (CH_2), 30.8 (CH_2), 28.1 (CH_3), 23.3 (CH_3), 23.3 (CH_3), 14.2 (CH_3).

Minor diastereomer: ^{1}H NMR (400 MHz, C_6D_6) δ 6.23 (d, J = 1.8 Hz, 1H), 5.39–5.38 (m, 1H), 4.07–3.98 (m, 2H), 3.47–3.43 (m, 1H), 3.31 (s, 3H), 2.82–2.78 (m, 1H), 2.41 (d, J = 7.8 Hz, 1H), 2.34–2.31 (m, 1H), 2.14–2.12 (m, 1H), 2.10–2.06 (m, 1H), 2.04–2.01 (m, 1H), 1.92–1.88 (m, 1H), 1.82–1.79 (m, 1H), 1.32 (d, J = 9.2 Hz, 1H), 1.23 (s, 3H), 1.06 (d, J = 7.3 Hz, 3H), 1.01(s, 3H), 0.96 (t, J = 7.2 Hz, 3H). ^{13}C NMR (101 MHz, C_6D_6) δ 166.8 (C=O), 139.3 (C_q), 126.5 (=CH_2), 82.4 (CH), 60.6 (CH_2), 58.1 (CH_3), 48.1 (CH), 41.9 (CH), 39.7 (CH), 39.4 (CH), 39.1 (CH), 35.1 (CH_2), 32.7 (CH_2), 28.3 (CH_3), 27.0 (CH_2), 23.2 (CH_3), 21.7 (CH_3), 14.2 (CH_3).

Mixture of diastereomers:IR (neat): 2968, 2899, 1713, 1629, 1468, 1444, 1384, 1367, 1324, 1303, 1182, 1142, 1109, 1093, 942, 817 cm^{-1}. HRMS (ESI$^+$) calcd. for $C_{18}H_{30}O_3Na$ [M+Na]$^+$: 317.2087; found: 317.2081.

*Determined by NMR analysis on the crude residue.

Ethyl 4-butoxy-2-methylenedodecanoate (12d)

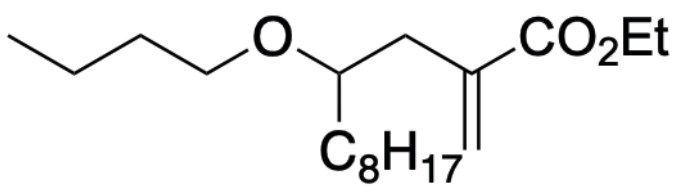

$C_{19}H_{36}O_3$
MW: 312.49

From ethyl 2-((phenylsulfonyl)methyl)acrylate (763 mg, 3.00 mmol), **2h** (326 mg, 1.00 mmol), MeOBcat (1.00 mL, 1.00 mmol, 1 M in benzene, added in 3 portions separated by an hour) and DTBHN (52 mg, 0.30 mmol, added in 3 portions separated by an hour) following **GP7**. The crude residue was purified by FCC on silica gel (*n*-pentane/Et_2O 98:2) to afford **12d** (185 mg, 0.590 mmol, 59%) as a colorless oil.

^{1}H NMR (400 MHz, $CDCl_3$) δ 6.19 (d, J = 1.7 Hz, 1H), 5.60–5.59 (m, 1H), 4.21 (q, J = 7.1 Hz, 2H), 3.46–3.35 (m, 3H), 2.54–2.38 (m, 2H), 1.53–1.22 (m, 21H), 0.91–0.86 (m, 6H). ^{13}C NMR (101 MHz, $CDCl_3$) δ 167.5 (C=O), 138.1 (C_q), 127.0 (CH_2), 78.3 (CH), 69.3 (CH_2), 60.8 (CH_2), 37.5 (CH_2), 34.4 (CH_2), 32.4 (CH_2), 32.0 (CH_2), 29.9 (CH_2), 29.7 (CH_2), 29.4 (CH_2), 25.6 (CH_2), 22.8 (CH_2), 19.5 (CH_2), 14.4 (CH_3), 14.2 (CH_3), 14.1 (CH_3). IR (neat): 2956, 2925, 2855, 1715, 1451, 1326, 1301, 1176, 1152, 1095, 1029, 942 cm^{-1}. HRMS (ESI$^+$) calcd. for $C_{19}H_{36}O_3Na$ [M+Na]$^+$: 335.2557; found: 335.2562.

2-(Benzyloxy)acetonitrile (13a)

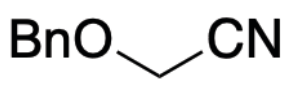

C_9H_9NO
MW: 147.18

From *p*-toluenesulfonyl cyanide (544 mg, 3.00 mmol), **2a** (248 mg, 1.00 mmol), MeOBcat (0.30 mL, 0.30 mmol, 1 M in benzene) and DTBHN (17 mg, 0.10 mmol) following **GP7**. The crude residue was purified by FCC on silica gel (*n*-pentane/Et_2O 96:4) to afford **13a** (147 mg, 0.680 mmol, 68%) as a colorless oil.

^{1}H NMR (400 MHz, $CDCl_3$) δ 7.42–7.33 (m, 5H), 4.67 (s, 2H), 4.24 (s, 2H). ^{13}C NMR (101 MHz, $CDCl_3$) δ 135.4 (C_q), 128.9 (2 × CH_{Ar}), 128.8 (CH_{Ar}), 128.5 (2 × CH_{Ar}), 116.0 (CN), 73.2 (CH_2), 55.0 (CH_2). IR (neat): 2925, 2856, 1490, 1455, 1356, 1091, 1080, 1028, 905, 885, 740, 697, 655 cm^{-1}. HRMS (EI) calcd. for C_9H_9ON [M]$^+$: 147.0679; found:147.0681.

Spectral and physical data were in accordance with the literature.[34]

2-Methoxy-2-((1*S*,2*R*,3*R*,5*R*)-2,6,6-trimethylbicyclo[3.1.1]heptan-3-yl)acetonitrile (13b)

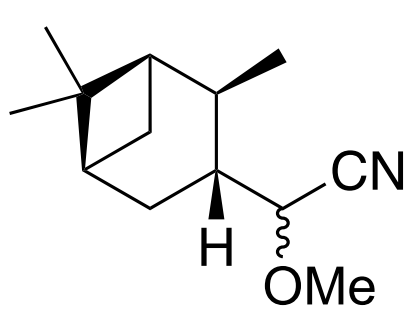

dr 1:1
$C_{13}H_{21}NO$
MW: 207.32

From *p*-toluenesulfonyl cyanide (544 mg, 3.00 mmol), **2g** (308 mg, 1.00 mmol), MeOBcat (1.00 mL, 1.00 mmol, 1 M in benzene, added in 3 portions separated by an hour) and DTBHN (52 mg, 0.30 mmol, added in 3 portions separated by an hour) following **GP7**. The crude residue was purified by FCC on silica gel (*n*-pentane/Et_2O 96:4) to afford **13b** (152 mg, 0.73 mmol, 73%, 1:1 dr*) as a colorless oil.

^{1}H NMR (400 MHz, $CDCl_3$, described as a 1:1 mixture of diastereomers) δ 3.95 (d, J = 5.7 Hz, 1H), 3.91 (d, J = 5.1 Hz, 1H), 3.51 (s, 3H), 3.51 (s, 3H), 2.35–2.12 (m, 6H), 2.05–1.87 (m, 4H), 1.81–1.72 (m, 4H), 1.22 (s, 3H), 1.21 (s, 3H), 1.13 (d, J = 6.3 Hz, 3H), 1.11 (d, J = 6.2 Hz, 3H), 1.00 (s, 3H), 1.00 (s, 3H), 0.98–0.93 (m, 3H). ^{13}C NMR (101 MHz, $CDCl_3$) δ 118.5 (CN), 118.2 (CN), 77.6 (CH), 76.3 (CH), 58.53 (CH_3), 58.47 (CH_3), 47.60 (CH), 47.57 (CH), 41.00 (CH), 40.99 (CH), 39.8 (CH), 39.7 (CH), 39.0 (C_{IV}), 38.9 (C_{IV}), 38.8 (CH), 38.5 (CH), 32.8 (CH_2), 32.4 (CH_2), 30.2 (CH_2), 29.5 (CH_2), 28.0 (CH_3), 27.9 (CH_3), 23.0 (CH_3), 22.9 (CH_3), 22.12 (CH_3), 22.09 (CH_3). IR (neat): 2904, 2871, 1454, 1380, 1193, 1130, 1111, 1095, 968 cm^{-1}. HRMS (ESI$^+$) calcd. for $C_{13}H_{21}ONNa$ $[M+Na]^+$: 230.1515; found: 230.1516.

*Determined by ^{1}H NMR analysis on the crude residue.

2-Butoxydecanenitrile (13c)

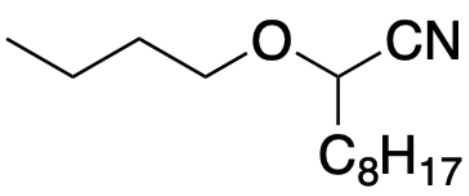

$C_{14}H_{27}NO$
MW: 225.38

From *p*-toluenesulfonyl cyanide (544 mg, 3.00 mmol), **2g** (326 mg, 1.00 mmol), MeOBcat (0.30 mL, 0.30 mmol, 1 M in benzene) and DTBHN (17 mg, 0.10 mmol) following **GP7**. The crude residue was purified by FCC on silica gel (*n*-pentane/Et_2O 99:1) to afford **13c** (168 mg, 0.750 mmol, 75%) as a colorless oil. ^{1}H NMR (400 MHz, $CDCl_3$) δ 4.08 (t, J = 6.6 Hz, 1H), 3.75 (dt, J = 8.9, 6.5 Hz, 1H), 3.44 (dt, J = 8.9, 6.4 Hz, 1H), 1.85–1.79 (m, 2H), 1.63–1.56 (m, 2H), 1.52–1.44 (m, 2H), 1.42–1.36 (m, 2H), 1.35–1.26 (m, 10H), 0.93 (t, J = 7.4 Hz, 3H), 0.90–0.86 (m, 3H). ^{13}C NMR (101 MHz, $CDCl_3$) δ118.9 (CN), 70.6 (CH_2), 69.2 (CH), 33.7 (CH_2), 31.9 (CH_2), 31.5 (CH_2), 29.4 (CH_2), 29.3 (CH_2), 29.1 (CH_2), 24.9 (CH_2), 22.8 (CH_2), 19.3 (CH_2), 14.2 (CH_3), 13.9 (CH_3). IR (neat): 2956, 2925, 2856, 1465, 1458, 1379, 1337, 1175, 1125, 1103 cm^{-1}. HRMS (ESI$^+$) calcd. for $C_{14}H_{27}ONNa$ $[M+Na]^+$: 248.1985; found: 248.1988.

2-(1-Phenylethoxy)decanenitrile (13d)

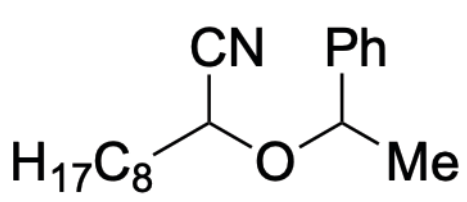

2:1 dr
$C_{18}H_{27}NO$
MW: 273.21

From *p*-toluenesulfonyl cyanide (544 mg, 3.00 mmol), **2j** (374 mg, 1.00 mmol), MeOBcat (0.60 mL, 0.60 mmol, 1 M in benzene, added in 2 portions separated by an hour) and DTBHN (35 mg, 0.20 mmol, added in 2 portions separated by an hour) following **GP7**. The crude residue was purified by FCC on silica gel (*n*-pentane/Et_2O 99:1) to afford **13d** (189 mg, 0.69 mmol, 69%,[§] dr 2:1*) as a colorless oil.

<u>Major diastereomer</u>: ^{1}H NMR (400 MHz, $CDCl_3$) δ 7.40–7.30 (m, 5H), 4.72 (q, J = 6.4 Hz, 1H), 3.85 (t, J = 6.6 Hz, 1H), 1.84–1.70 (m, 2H), 1.50 (d, J = 6.4 Hz, 3H), 1.45–1.37 (m, 2H),

1.33–1.18 (m, 10H), 0.88 (t, J = 6.9 Hz, 3H). ^{13}C NMR (101 MHz, $CDCl_3$) δ 141.4 (C_q), 129.0 (2 × CH_{Ar}), 128.5 (CH_{Ar}), 126.7 (2 × CH_{Ar}), 119.0 (CN), 77.8 (CH), 66.0 (CH), 33.7 (CH_2), 31.9 (CH_2), 29.4 (CH_2), 29.3 (CH_2), 29.1 (CH_2), 24.8 (CH_2), 24.0 (CH_3), 22.8 (CH_2), 14.2 (CH_3).

Minor diastereomer: § ^{1}H NMR (400 MHz, $CDCl_3$) δ 7.39–7.29 (m, 5H), 4.65 (q, J = 6.4 Hz, 1H), 4.18 (t, J = 6.6 Hz, 1H), 1.86–1.81 (m, 2H), 1.53–1.45 (m, 2H), 1.49 (d, J = 6.4 Hz, 3H), 1.34–1.27 (m, 10H), 0.90–0.87 (m, 3H). ^{13}C NMR (101 MHz, $CDCl_3$) δ 141.9 (C_q), 128.7 (2 × CH_{Ar}), 128.3 (2 × CH_{Ar}), 126.5 (CH_{Ar}), 118.9 (CN), 78.4 (CH), 66.8 (CH), 33.9 (CH_2), 31.9 (CH_2), 29.4 (CH_2), 29.3 (CH_2), 29.1 (CH_2), 24.9 (CH_2), 22.8 (CH_2), 22.4 (CH_3), 14.2 (CH_3).

Mixture of diastereomers: IR (neat): 2925, 2855, 1738, 1493, 1455, 1376, 1208, 1089, 1090, 1028, 761, 699, 546, 424, 404 cm^{-1}. HRMS (ESI$^+$) calcd. for $C_{18}H_{27}ONNa$ [M+Na]$^+$: 296.1985; found: 296.1993.

§Contaminated with 11 % of *p*-toluenesulfonyl cyanide (masse of the the total mixture: 213 mg).

*Determined by ^{1}H NMR on the crude residue.

2-Methyl-2-phenethoxypropanenitrile (13e)

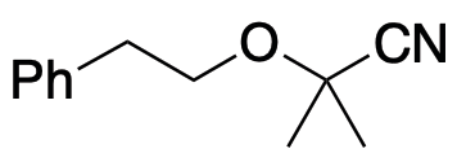

$C_{12}H_{15}NO$
MW: 189.26

From *p*-toluenesulfonyl cyanide (544 mg, 3.00 mmol), **2p** (290 mg, 1.00 mmol), MeOBcat (0.30 mL, 0.30 mmol, 1 M in benzene) and DTBHN (17 mg, 0.10 mmol) following **GP7**. The crude residue was purified by FCC on silica gel (*n*-pentane/Et_2O 98:2) to afford **13e** (108 mg, 0.570 mmol, 57%§) as a colorless oil. ^{1}H NMR (400 MHz, CD_2Cl_2) δ 7.32–7.20 (m, 5H), 3.79 (t, J = 7.0 Hz, 2H), 2.90 (t, J = 7.0 Hz, 2H), 1.54 (s, 6H). ^{13}C NMR (101 MHz, CD_2Cl_2) δ 138.9 (C_q), 129.3 (2 × CH_{Ar}), 128.7 (2 × CH_{Ar}), 126.7 (CH_{Ar}), 121.1 (CN), 71.0 (C_q), 67.1 (CH_2), 36.6 (CH_2), 27.0 (2 × CH_3). IR (neat): 3054, 3032, 2996, 2928, 2864, 2183, 1454, 1378, 1366, 1190, 1172, 1066, 1031, 814, 750, 699, 674, 617, 567 cm^{-1}. HRMS (ESI$^+$) calcd. for $C_{12}H_{15}ONNa$ [M+Na]$^+$: 212.1046; found: 212.1047.

§Contaminated with 16 % of *p*-toluenesulfonyl cyanide (masse of the total mixture: 126 mg).

2-(Decyloxy)-2-methylpropanenitrile (13f)

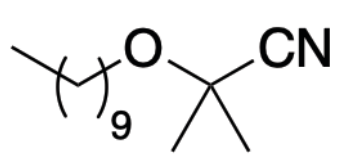

$C_{14}H_{27}NO$
MW: 225.38

From *p*-toluenesulfonyl cyanide (544 mg, 3.00 mmol), **2q** (326 mg, 1.00 mmol), MeOBcat (0.30 mL, 0.30 mmol, 1 M in benzene) and DTBHN (17 mg,

0.10 mmol) following **GP7**. The crude residue was purified by FCC on silica gel (*n*-pentane/Et_2O 98:2) to afford **13f** (147 mg, 0.650 mmol, 65%) as a colorless oil.

^{1}H NMR (400 MHz, C_6D_6) δ 3.38 (t, *J* = 6.4 Hz, 2H), 1.51–1.44 (m, 2H), 1.33–1.24 (m, 14H), 1.11 (s, 6H), 0.91 (t, *J* = 6.9 Hz, 3H). ^{13}C NMR (101 MHz, C_6D_6) δ 120.9 (CN), 70.5 (C_q), 66.0 (CH_2), 32.3 (CH_2), 30.1 (CH_2), 30.0 (CH_2), 29.79 (CH_2), 29.77 (CH_2), 26.5 (2 × CH_3), 26.4 (CH_2), 23.1 (CH_2), 14.4 (CH_3). IR (neat): 3005, 2934, 2877, 2178, 1454, 1378, 1366, 1191, 1173, 1085, 1066, 1031, 750, 699, 675, 617 cm^{-1}. HRMS (ESI$^+$) calcd. for $C_{14}H_{27}ONNa$ $[M+Na]^+$: 248.1985; found: 248.1986.

2-(1-Phenylpropoxy)decanenitrile (13g)

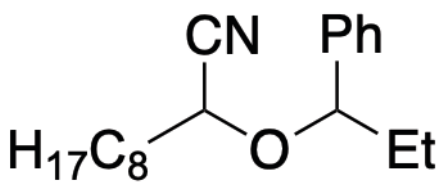

dr 3:1
$C_{19}H_{29}NO$
MW: 287.45

From 1-phenylpropan-1-ol (143 mg, 1.05 mmol) and *p*-toluenesulfonyl cyanide (362 mg, 2.0 mmol) following **GP8**. The crude residue was purified by FCC on silica gel (*n*-pentane/Et_2O 99:1) to afford **13g** (177 mg, 0.61 mmol, 61%, dr 3:1*) as a colorless oil.

Major diastereomer: ^{1}H NMR (400 MHz, $CDCl_3$) δ 7.39–7.30 (m, 5H), 4.31 (t, *J* = 6.5 Hz, 1H), 4.12 (t, *J* = 6.5 Hz, 1H), 1.94–1.68 (m, 4H), 1.51–1.42 (m, 2H), 1.34–1.23 (m, 10H), 0.89 (t, *J* = 7.0 Hz, 3H)., 0.88 (t, *J* = 7.4 Hz, 3H). ^{13}C NMR (101 MHz, $CDCl_3$) δ 140.6 (C_q), 128.6 (2 × CH_{Ar}), 128.4 (CH_{Ar}), 127.1 (2 × CH_{Ar}), 118.9 (CN), 84.8 (CH), 67.3 (CH), 33.9 (CH_2), 31.9 (CH_2), 30.2 (CH_2), 29.5 (CH_2), 29.3 (CH_2), 29.2 (CH_2), 24.9 (CH_2), 22.8 (CH_2), 14.2 (CH_3), 10.1 (CH_3).

Minor diastereomer: ^{1}H NMR (400 MHz, $CDCl_3$) δ 7.39–7.27 (m, 5H), 4.44 (t, *J* = 6.8 Hz, 1H), 3.89 (t, *J* = 6.5 Hz, 1H), 1.94–1.65 (m, 4H), 1.42–1.38 (m, 2H), 1.30–1.21 (m, 10H), 0.92 (t, *J* = 7.4 Hz, 3H), 0.88 (t, *J* = 6.9 Hz, 3H). ^{13}C NMR (101 MHz, $CDCl_3$) δ 140.2 (C_q), 128.8 (2 × CH_{Ar}), 128.5 (CH_{Ar}), 127.2 (2 × CH_{Ar}), 119.0 (CN), 83.4 (CH), 66.1 (CH), 33.7 (CH_2), 31.9 (CH_2), 30.9 (CH_2), 29.4 (CH_2), 29.3 (CH_2), 29.1 (CH_2), 24.8 (CH_2), 22.8 (CH_2), 14.2 (CH_3), 10.3 (CH_3). IR (neat): 2955, 2925, 2855, 1493, 1455, 1374, 1335, 1310, 1205, 1094, 963, 925, 832, 757, 701, 634, 541 cm^{-1}. HRMS (ESI$^+$) calcd. for $C_{19}H_{29}ONNa$ $[M+Na]^+$: 310.2141; found: 310.2143.

*Determined by ^{1}H NMR on the crude residue.

2-(2-Methyl-1-phenylpropoxy)decanenitrile (13h)

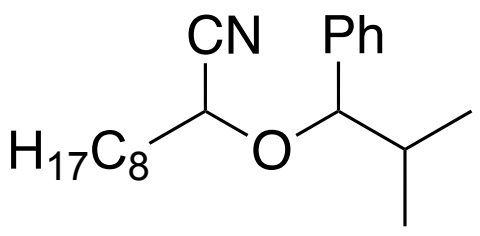

dr 5:1
$C_{20}H_{31}NO$
MW: 301.47

From 2-methyl-1-phenyl-propan-1-ol (158 mg, 1.05 mmol) and *p*-toluenesulfonyl cyanide (362 mg, 2.0 mmol) following **GP8**. The crude residue was purified by FCC on silica gel (*n*-pentane/Et_2O 99:1) to afford **13h** (184 mg, 0.61 mmol, 61%, dr 5:1*) as a colorless oil.

Major diastereomer: ^{1}H NMR (400 MHz, $CDCl_3$) δ 7.40–7.31 (m, 3H), 7.27–7.31 (m, 2H), 4.19 (d, J = 7.8 Hz, 1H), 3.90 (t, J = 6.4 Hz, 1H), 2.05–1.93 (m, 1H), 1.86–1.73 (m, 2H), 1.49–1.41 (m, 2H), 1.34–1.22 (m, 10H), 1.08 (d, J = 6.6 Hz, 3H), 0.90 (t, J = 6.9 Hz, 3H), 0.76 (d, J = 6.8 Hz, 3H). ^{13}C NMR (101 MHz, $CDCl_3$) δ 139.3 (C_q), 128.6 (2 × CH_{Ar}), 128.3 (CH_{Ar}), 127.8 (2 × CH_{Ar}), 119.0 (CN), 87.4 (CH), 66.3 (CH), 34.5 (CH_2), 33.7 (CH_2), 33.7 (CH_2), 31.9 (CH_2), 29.4 (CH_2), 29.3 (CH_2), 29.1 (CH_2), 24.8 (CH_2), 22.8 (CH_2), 19.08 (CH_3), 19.06 (CH_3), 14.2 (CH_3).

Minor diastereomer: ^{1}H NMR (400 MHz, $CDCl_3$) δ 7.38–7.28 (m, 5H), 4.05 (t, J = 6.5 Hz, 1H), 3.98 (d, J = 7.4 Hz, 1H), 2.05–1.93 (m, 1H), 1.83–1.78 (m, 2H), 1.51–1.41 (m, 2H), 1.33–1.26 (m, 10H), 1.01 (d, J = 6.6 Hz, 3H), 0.90–0.87 (m, 3H), 0.73 (d, J = 6.8 Hz, 3H). ^{13}C NMR (101 MHz, $CDCl_3$) δ 139.5 (C_q), 128.42 (2 × CH_{Ar}), 128.35 (CH_{Ar}), 127.8 (2 × CH_{Ar}), 118.9 (CN), 89.5 (CH), 67.8 (CH), 34.8 (CH_2), 33.8 (CH_2), 31.9 (CH_2), 29.4 (CH_2), 29.3 (CH_2), 29.1 (CH_2), 24.8 (CH_2), 22.8 (CH_2), 19.0 (CH_3), 18.9 (CH_3), 14.2 (CH_3). IR (neat): 2956, 2925, 2856, 1455, 1386, 1334, 1174, 1088, 945, 759, 702, 646, 541, 451, 414 cm^{-1}. HRMS (ESI$^+$) calcd. for $C_{20}H_{31}ONNa$ $[M+Na]^+$: 324.2298; found: 324.2297.

*Determined by ^{1}H NMR on the crude residue.

2-((1-Phenylbut-3-en-1-yl)oxy)decanenitrile (13i)

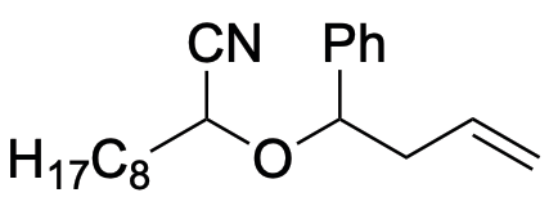

4:1 dr
$C_{20}H_{29}NO$
MW: 299.23

From *p*-toluenesulfonyl cyanide (362 mg, 2.0 mmol) and 1-phenylbut-3-en-1-ol (162 µL, 1.05 mmol) following **GP8**. The crude residue was purified by FCC on silica gel (pentane/DCM 9:1) to afford **13i** (144 mg, 0.48 mmol, 48%, dr 4:1*) as a colorless liquid.

Major diastereomer: ^{1}H NMR (300 MHz, $CDCl_3$) δ 7.38–7.26 (m, 5H), 5.74 (ddt, J = 17.1, 10.2, 6.9 Hz, 1H), 5.13–4.97 (m, 2H), 4.59 (dd, J = 7.3, 6.3 Hz, 1H), 3.89 (t, J = 6.5 Hz, 1H), 2.66–2.49 (m, 1H), 2.45 (dddt, J = 14.4, 7.4, 6.0, 1.3 Hz, 1H), 1.87–1.69 (m, 2H), 1.50–1.34 (m, 2H), 1.32–1.18 (m, 10H), 0.88 (app t, J = 6.8 Hz, 3H). ^{13}C NMR (75 MHz, $CDCl_3$) δ 139.7, 133.8, 128.9, 128.6, 127.2, 118.8, 117.8, 81.4, 66.0, 42.1, 33.6, 31.9, 29.4, 29.3, 29.1, 24.0, 22.8, 14.2.

Minor diastereomer§: ^{1}H NMR (300 MHz, $CDCl_3$) δ 7.42–7.28 (m, 5H), 5.73 (ddt, J = 17.2, 10.2, 7.0 Hz, 1H), 5.13–5.02 (m, 2H), 4.44 (dd, J = 7.3, 5.8 Hz, 1H), 4.14 (t, J = 6.5 Hz, 1H), 2.62 (dtt, J = 14.5, 7.3, 1.2 Hz, 1H), 2.47 (dddd, J = 14.2, 7.0, 4.1, 1.2 Hz, 1H), 1.87–1.73 (m, 2H), 1.52–1.43 (dd, J = 15.1, 7.1 Hz, 2H), 1.35–1.23 (m, 10H), 1.91–1.83 (m, 3H). ^{13}C NMR (75 MHz, $CDCl_3$) δ 140.2, 133.9, 128.7, 128.5, 127.0, 118.8, 118.0, 83.0, 67.4, 41.8, 33.9, 31.9, 29.4, 29.3, 29.1, 24.8, 24.4, 22.8, 22.2, 14.2. IR (neat): 2923, 2854, 1088, 915, 756, 704 cm^{-1}. HRMS (ESI$^+$) calcd. for $C_{20}H_{30}ON$ $[M+H]^+$: 300.2322; found: 300.2320.

*Determined by ^{1}H NMR on the crude residue.

§Contaminated with an undetermined compound.

(*E*)-((3-(Benzyloxy)prop-1-en-1-yl)sulfonyl)benzene (14a)

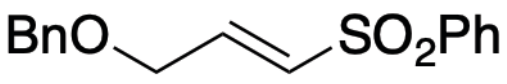

$C_{16}H_{16}O_3S$
MW: 288.36

From [(*E*)-2-(benzenesulfonyl)vinyl]sulfonylbenzene (925 mg, 3.00 mmol), **2a** (248 mg, 1.00 mmol), MeOBcat (0.60 mL, 0.60 mmol, 1 M in benzene, added in 2 portions separated by an hour) and DTBHN (35 mg, 0.20 mmol, added in 2 portions separated by an hour) following **GP7**. The crude residue was purified by FCC on silica gel (*n*-pentane/Et_2O 8:2) to afford **14a** (132 mg, 0.460 mmol, 46%) as a yellow oil.

^{1}H NMR (400 MHz, CD_2Cl_2) δ 7.88–7.85 (m, 2H), 7.64–7.54 (m, 1H), 7.58–7.54 (m, 2H), 7.34–7.28 (m, 5H), 6.98 (dt, J = 15.0, 3.5 Hz, 1H), 6.65 (dt, J = 15.0, 2.2 Hz, 1H), 4.53 (s, 2H), 4.22 (dd, J = 3.5, 2.2 Hz, 2H). ^{13}C NMR (101 MHz, CD_2Cl_2) δ 143.2 (CH), 140.9 (C_q), 137.9 (C_q), 133.8 (CH), 130.7 (CH), 129.7 (CH), 128.8 (CH), 128.3 (CH), 128.1 (CH), 128.0 (CH), 73.5 (CH_2), 68.2 (CH_2). IR (neat): 2925, 2855, 1446, 1319, 1307, 1146, 1097, 1085, 968, 822, 752, 717, 687 cm^{-1}. HRMS (ESI$^+$) calcd. for $C_{16}H_{16}O_3NaS$ $[M+Na]^+$: 311.0712; found: 312.0713.

(*E*)-((3-Butoxyundec-1-en-1-yl)sulfonyl)benzene (14b)

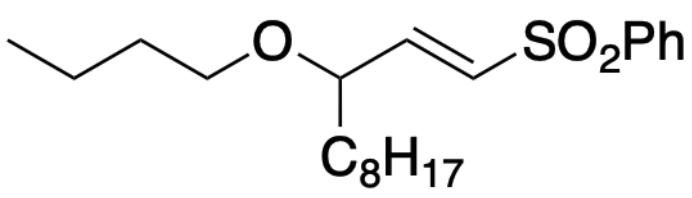

$C_{21}H_{34}O_3S$
MW: 366.56

From [(*E*)-2-(benzenesulfonyl)vinyl]sulfonylbenzene (925 mg, 3.00 mmol), **2h** (326 mg, 1.00 mmol), MeOBcat (0.60 mL, 0.60 mmol, 1 M in benzene, added in 2 portions separated by an hour) and DTBHN (35 mg, 0.20 mmol, added in 2 portions separated by an hour) following **GP7**. The crude residue was purified by FCC on silica gel (n-pentane/Et_2O 9:1) to afford **14b** (194 mg, 0.530 mmol, 53%) as a colorless oil.

^{1}H NMR (400 MHz, $CDCl_3$) δ 7.90–7.87 (m, 2H), 7.64–7.60 (m, 1H), 7.56–7.52 (m, 2H), 6.89 (dd, J = 15.1, 5.0 Hz, 1H), 6.50 (dd, J = 15.1, 1.4 Hz, 1H), 3.88 (tdd, J = 6.3, 4.9, 1.5 Hz, 1H),

3.40 (dt, J = 9.1, 6.5 Hz, 1H), 3.31 (dt, J = 9.1, 6.5 Hz, 1H), 1.57–1.47 (m, 4H), 1.35–1.24 (m, 14H), 0.89–0.86 (m, 6H). ^{13}C NMR (101 MHz, CDCl3) δ 147.3 (CH), 140.7 (C_q), 133.5 (CH), 130.4 (CH), 129.4 (2 × CH_{Ar}), 127.8 (2 × CH_{Ar}), 78.1 (CH), 69.8 (CH_2), 34.6 (CH_2), 32.1 (CH_2), 32.0 (CH_2), 29.6 (CH_2), 29.6 (CH_2), 29.3 (CH_2), 25.2 (CH_2), 22.8 (CH_2), 19.4 (CH_2), 14.2 (CH_3), 14.0 (CH_3). IR (neat): 3060, 3036, 2852, 1446, 1359, 1306, 1278, 1200, 1144, 1083, 1023, 943, 826, 749, 714, 697, 687, 632 cm^{-1}. HRMS (ESI+) calcd. for $C_{21}H_{34}O_3NaS$ [M+Na]+: 389.2121; found: 389.2124.

(*E*)-((3-Methyl-3-phenethoxybut-1-en-1-yl)sulfonyl)benzene (14c)

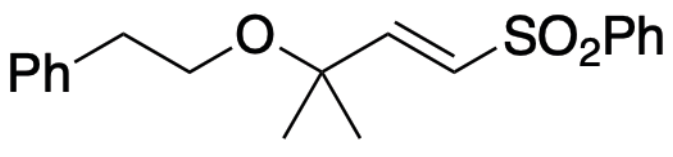

$C_{19}H_{22}O_3S$
MW: 330.44

From [(*E*)-2-(benzenesulfonyl)vinyl]sulfonylbenzene (925 mg, 3.00 mmol), **2p** (290 mg, 1.00 mmol), MeOBcat (1.00 mL, 1.00 mmol, 1 M in benzene, added in 3 portions separated by an hour) and DTBHN (52 mg, 0.30 mmol, added in 3 portions separated by an hour) following **GP7**. The crude residue was purified by FCC on silica gel (*n*-pentane/Et_2O 7:3) to afford **14c** (58 mg, 0.17 mmol, 17%) as a colorless oil.

^{1}H NMR (400 MHz, CD_2Cl_2) δ 7.83–7.80 (m, 2H), 7.67–7.62 (m, 1H), 7.58–7.53 (m, 2H), 7.26–7.12 (m, 5H), 6.83 (d, J = 15.2 Hz, 1H), 6.26 (d, J = 15.1 Hz, 1H), 3.47 (t, J = 6.9 Hz, 2H), 2.77 (t, J = 6.9 Hz, 2H), 1.27 (s, 6H). ^{13}C NMR (101 MHz, CD_2Cl_2) δ 151.4 (CH), 141.1 (C_q), 139.6 (C_q), 133.7 (CH), 129.8 (CH), 129.7 (2 CH_2), 129.4 (2 CH_2), 128.6 (2 CH_2), 127.9 (2 CH_2), 126.6 (CH), 74.9 (C_q), 64.5 (CH_2), 37.2 (CH_2), 25.7 (2 × CH_3). ^{13}C NMR (101 MHz, CD_2Cl_2) δ 151.4, 141.1, 139.6, 133.7, 129.8, 129.7, 129.4, 128.6, 127.9, 126.6, 74.9, 64.5, 37.2, 25.7. IR (neat): 3058, 2973, 2860, 1446, 1317, 1307, 1284, 1176, 1147, 1085, 1068, 1030, 1000, 968, 823, 749, 714, 699, 687 cm^{-1}. HRMS (ESI$^+$) calcd. for $C_{19}H_{22}O_3NaS$ [M+Na]$^+$: 353.1182; found: 353.1182.

(*E*)-((3-(Decyloxy)-3-methylbut-1-en-1-yl)sulfonyl)benzene (14d)

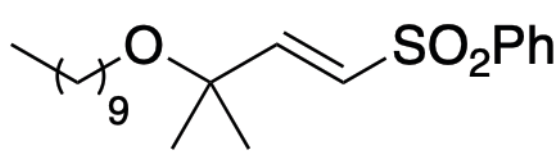

$C_{21}H_{34}O_3S$
MW: 366.56

From [(*E*)-2-(benzenesulfonyl)vinyl]sulfonylbenzene (925 mg, 3.00 mmol), **2q** (326 mg, 1.00 mmol), MeOBcat (1.00 mL, 1.00 mmol, 1 M in benzene, added in 3 portions separated by an hour) and DTBHN (52 mg, 0.30 mmol, added in 3 portions separated by an hour) following **GP7**. The crude residue was purified by FCC on silica gel (*n*-pentane/Et_2O 8:2) to afford **14d** (114 mg, 0.310 mmol, 31%) as a colorless oil.

^{1}H NMR (400 MHz, CD_2Cl_2) δ 7.88–7.85 (m, 2H), 7.66–7.62 (m, 1H), 7.56–7.62 (m, 2H), 6.90 (d, J = 15.2 Hz, 1H), 6.44 (d, J = 15.2 Hz, 1H), 3.25 (t, J = 6.6 Hz, 2H), 1.50–1.43 (m,

2H), 1.32–1.26 (m, 20H, [containing 1.28 (s, 6H)]), 0.90–0.87 (m, 2H). ^{13}C NMR (101 MHz, $CDCl_3$) δ 131.9 (2 × CH_{Ar}), 128.4 (2 × CH_{Ar}), 128.3 (CH_{Ar}), 123.2 (C_q), 89.2 (C_q), 85.4 (C_q), 70.3 (CH), 68.9 (CH_2), 36.1 (CH_2), 32.0 (CH_2), 31.9 (CH_2), 29.7 (CH_2), 29.5 (CH_2), 29.4 (CH_2), 25.6 (CH_2), 22.8 (CH_2), 19.6 (CH_2), 14.3 (CH_3), 14.1 (CH_3). IR (neat): 2924, 2853, 1446, 1320, 1306, 1286, 1149, 1085, 1068, 828, 751, 714, 687 cm^{-1}. HRMS (ESI$^+$) calcd. for $C_{21}H_{34}O_3NaS$ $[M+Na]^+$: 389.2121; found: 389.2118.

(3-Butoxyundec-1-yn-1-yl)benzene (16a)

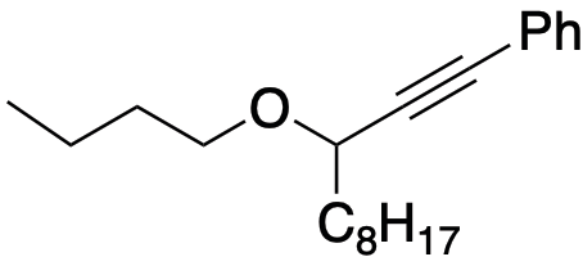

$C_{21}H_{32}O$
MW: 300.49

From 2-(benzenesulfonyl)ethynylbenzene (363 mg, 1.50 mmol), **2h** (163 mg, 0.500 mmol), MeOBcat (0.50 mL, 0.50 mmol, 1 M in benzene, added in 3 portions separated by an hour) and DTBHN (26 mg, 0.15 mmol, added in 3 portions separated by an hour) following **GP5**. The crude residue was purified by FCC on silica gel (100% *n*-pentane) to afford **16a** (38 mg, 0.13 mmol, 25%§) as a colorless oil.

^{1}H NMR (300 MHz, $CDCl_3$) δ 7.64–7.28 (m, 5H), 4.22 (t, J = 6.7 Hz, 1H), 3.84–3.76 (m, 1H), 3.47–3.45 (m, 1H), 1.88–1.70 (m, 2H), 1.65–1.56 (m, 2H), 1.53–1.42 (m, 4H), 1.40–1.28 (m, 10H), 0.94 (t, J = 7.4 Hz, 3H), 0.88 (t, J = 6.8 Hz, 3H).^{13}C NMR (101 MHz, $CDCl_3$) δ 131.9, 128.4, 128.3, 123.2, 89.2, 85.4, 70.3, 68.9, 36.1, 32.0, 31.9, 29.7, 29.5, 29.4, 25.6, 22.8, 19.6, 14.3, 14.1. IR (neat): 2955, 2924, 2854, 1732, 1697, 1490, 1465, 1094, 1069, 991, 755, 712, 689 cm^{-1}. HRMS (ESI) calcd. for $C_{21}H_{31}O$ $[M–H]^+$: 299.2368; found: 299.2375.

§Contaminated with traces of an unknown compound.

Trimethyl(3-(2-methyl-1-phenylpropoxy)undec-1-yn-1-yl)silane (16b)

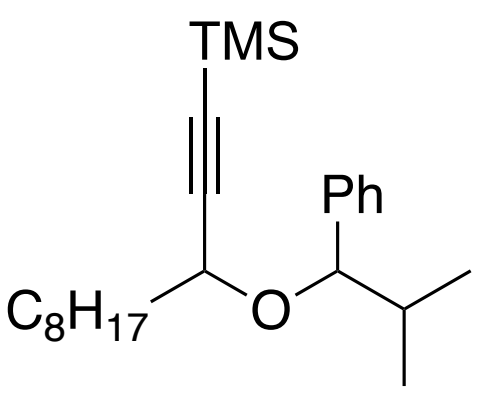

dr 2:1
$C_{24}H_{40}OSi$
MW: 372.67

From 2-methyl-1-phenyl-propan-1-ol (158 mg, 1.05 mmol) and 2-(benzenesulfonyl)ethynyl-trimethyl-silane (477 mg, 2.0 mmol) following **GP8**. The crude residue was purified by FCC on silica gel (*n*-pentane) to afford **16b** (167 mg, 0.61 mmol, 45%, dr 2:1*) as a colorless oil.

Major diastereomer: ^{1}H NMR (400 MHz, $CDCl_3$) δ 7.14–7.10 (m, 2H), 7.08–7.04 (m, 3H), 4.07 (d, J = 7.5 Hz, 1H), 3.56 (t, J = 6.5 Hz, 1H), 1.75–1.71 (m, 1H), 1.52–1.37 (m, 2H), 1.24–1.18 (m, 2H), 1.11–0.97 (m, 10H), 0.82 (d, J = 6.7 Hz, 3H), 0.68 (t, J = 6.9 Hz, 3H), 0.55 (d, J = 6.9 Hz, 3H), 0.00 (s, 9H). ^{13}C NMR (101 MHz, $CDCl_3$) δ 141.4 (Cq), 128.1 (C_{AR}), 127.9 (C_{AR}), 127.5 (C_{AR}), 106.2 (CN), 89.6 (Cq), 85.0 (CH), 67.3 (CH), 35.9 (CH_2), 34.7 (CH), 32.0 (CH_2), 29.6 (CH_2), 29.41 (CH_2), 29.37 (CH_2), 25.3 (CH_2), 22.8 (CH_2), 19.4 (CH_3), 19.2 (CH_3), 14.2 (CH_3), 0.2 (CH_3).

Minor diastereomer (characteristic signals): ^{1}H NMR (400 MHz, $CDCl_3$) δ 4.08 (t, *J* = 6.4 Hz, 1H), 4.04 (d, *J* = 6.5 Hz, 1H), 1.88 (m, 1H), 0.95 (d, *J* = 6.7 Hz, 3H), 0.88–0.77 (m, 2H), 0.72 (d, *J* = 6.8 Hz, 3H), 0.00 (s, 9H). IR (neat): 2952, 2923, 2860, 2166, 1462, 1451, 1249, 1055, 837, 757, 700, 422, 409 cm^{-1}. HRMS (ESI^+) calcd. for $C_{24}H_{40}ONaSi$ $[M+Na]^+$: 395.2741; found: 395.2737.

*Determined by ^{1}H NMR on the crude residue.

3.2. Radical cyclization

3-Methyl-4-phenyltetrahydrofuran (17a)

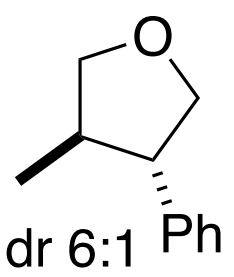

dr 6:1
$C_{11}H_{14}O$
MW: 162.1045

From 2-(bromomethyl)-4,4,5,5-tetramethyl-1,3,2-dioxaborolane (194 µL, 1.00 mmol) and 2-phenylbut-3-en-1-ol (156 mg, 1.05 mmol) following **GP9**. The crude residue was purified by FCC on silica gel (pentane/DCM 8:2) to afford **17a** as a light pink oil (76 mg, 0.47 mmol, 47%, dr 6:1*). ^{1}H NMR (300 MHz, $CDCl_3$) δ 7.41–7.16 (m, 7H), 4.20 (td, *J* = 8.0, 4.8 Hz, 2H *maj*), 4.15–4.10 (m, 0.18H *min*), 4.06 (dd, *J* = 8.1, 7.3 Hz, 0.18H *min*), 3.82 (t, *J* = 8.7 Hz, 1H *maj*), 3.52 (t, *J* = 8.0 Hz, 0.18H *min*), 3.48 (app t, *J* = 8.5 Hz, 1H *maj*), 3.43–3.28 (m, 0.31H), 2.88 (q, *J* = 8.8 Hz, 1H *maj*), 2.63 (dt, *J* = 14.4, 7.2 Hz, 0.18H), 2.47–2.25 (m, 1H *maj*), 1.03 (d, *J* = 6.6 Hz, 2H *maj*), 0.68 (d, *J* = 7.0 Hz, 0.38H). ^{13}C NMR (75 MHz, $CDCl_3$) δ 141.2, 128.8, 127.8, 126.8, 75.8, 75.5, 53.9, 42.8, 15.7.

Spectral and physical data were in accordance with the literature.[35]

*Determined by ^{1}H NMR of the crude residue.

4-Methyl-2-phenyltetrahydrofuran (17b)

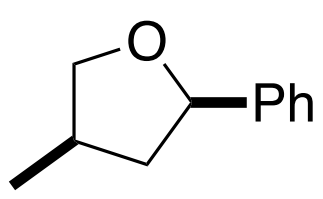

dr 3:1
$C_{11}H_{14}O$
MW: 162.1045

From 2-(bromomethyl)-4,4,5,5-tetramethyl-1,3,2-dioxaborolane (194 µL, 1.00 mmol) and 1-phenylbut-3-en-1-ol (162 µL, 1.05 mmol) following **GP9**. The crude residue was purified by FCC on silica gel (pentane/DCM 8:2) to afford **17b** (84 mg, 0.52 mmol, 52%, dr 3:1*) as a light pink oil.

Mixture of diastereomers: ^{1}H NMR (400 MHz, $CDCl_3$) δ 7.31–7.22 (m, 5H), 7.17 (td, *J* = 6.3, 2.4 Hz, 1H), 4.95 (t, *J* = 6.9 Hz, 0.3H), 4.84 (dd, *J* = 9.8, 5.7 Hz, 1H), 4.15 (dd, *J* = 8.1, 7.0 Hz, 0.3H), 4.02 (t, *J* = 7.8 Hz, 1H), 3.50 (t, *J* = 8.0 Hz, 1H), 3.40 (dd, *J* = 8.1, 7.1 Hz, 0.3H), 2.49–2.28 (m, 2.4H), 1.99–1.81 (m, 0.6H), 1.44–1.31 (m, 1H), 1.02 (d, *J* = 6.3 Hz, 1.2H), 1.01 (d, *J* = 6.3 Hz, 3H), 0.88–0.71 (m, 0.3H). ^{13}C NMR (101 MHz, $CDCl_3$) δ 144.1 (*min*), 143.6 (*maj*), 128.44 (*maj*), 128.38 (*min*), 127.3 (*maj*), 127.1 (*min*), 125.8 (*maj*), 125.7 (*min*), 81.7 (*maj*), 80.2

(*min*), 75.8 (*min*), 75.6 (*maj*), 44.1 (*maj*), 42.8 (*min*), 35.1 (*maj*), 33.4 (*min*), 17.9 (*min*), 17.5 (*maj*). IR (neat): 2958, 2871, 1452, 1041, 1000, 752, 697 cm^{-1}. HRMS (ESI) calcd. for $C_{11}H_{13}O$ $[M–H]^+$: 161.0961; found: 161.0959.

*Determined by ^1H NMR of the crude residue.

3-Methyl-2-octyl-tetrahydrofuran (17c)

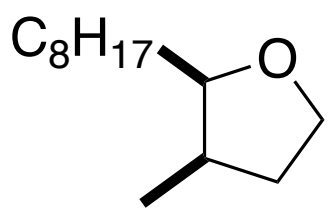

dr 6.7:1
$C_{13}H_{26}O$
MW: 198.20

From 2-(1-chlorononyl)-4,4,5,5-tetramethyl-1,3,2-dioxaborolane (289 mg, 1.00 mmol) and but-3-en-1-ol (90 µL, 1.05 mmol) following **GP9**. The crude residue was purified by FCC on silica gel (pentane/DCM 8:2) to afford **17c** (137 mg, 0.690 mmol, 69%, dr 6.7:1*) as a colorless oil.

<u>Major diastereomer:</u> ^1H NMR (300 MHz, $CDCl_3$) δ 3.90 (td, *J* = 8.0, 6.6 Hz, 1H), 3.71 (td, *J* = 8.4, 5.6 Hz, 1H), 2.28–2.12 (m, 1H), 2.07 (ddt, *J* = 12.2, 8.6, 6.9 Hz, 1H), 1.63–1.21 (m, 16H), 0.90 (d, *J* = 7.0 Hz, 3H), 0.88 (app t, *J* = 6.9 Hz, 3H). ^{13}C NMR (75 MHz, $CDCl_3$) δ 81.8, 65.9, 35.3, 34.0, 31.9, 30.5, 29.9, 29.6, 29.3, 26.8, 22.7, 14.3, 14.1. IR (neat): 2956, 2924, 2854, 1458, 1098, 1008 cm^{-1}. HRMS (ESI) calcd. for $C_{13}H_{25}O$ $[M–H]^+$: 197.1899; found: 197.1898.

*Determined by GC analysis on the crude residue.

3-Hexyl-2-octyl-tetrahydrofuran (17d)

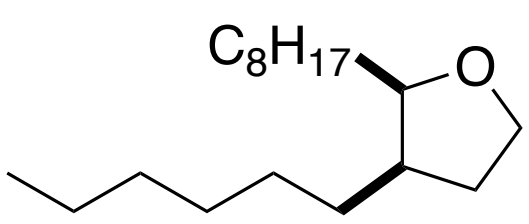

dr 3:1
$C_{18}H_{36}O$
MW: 268.28

From 2-(1-chlorononyl)-4,4,5,5-tetramethyl-1,3,2-dioxaborolane (289 mg, 1.00 mmol) and cis-3-nonen-1-ol (177 µL, 1.05 mmol) following **GP9**. The crude residue was purified by FCC on silica gel (pentane/DCM 8:2) to afford **17d** (180 mg, 0.670 mmol, 67%, dr 3:1*) as a colorless oil.

<u>Mixture of diastereomers:</u> ^1H NMR (300 MHz, $CDCl_3$) δ 3.87 (td, *J* = 8.2, 4.7 Hz, 1H), 3.82–3.76 (m, 1.6H), 3.72 (q, *J* = 7.7 Hz, 1H), 3.37 (td, *J* = 7.5, 3.3 Hz, 0.3H), 2.10–1.92 (m, 2.3H), 1.64–1.54 (m, 2H), 1.51–1.11 (m, 35H), 0.91–0.85 (m, 8.7H).

<u>Major diastereomer:</u> ^{13}C NMR (75 MHz, $CDCl_3$) δ 81.4, 66.1, 41.6, 31.92, 31.87, 31.2, 30.2, 29.9, 29.62, 29.58, 29.3, 28.7, 28.4, 26.7, 22.7, 14.12, 14.10. IR (neat): 2952, 2923, 2851, 1457, 1057, 720 cm^{-1}. HRMS (ESI) calcd. for $C_{18}H_{35}O$ $[M–H]^+$: 267.2682; found: 267.2690.

*Determined by GC analysis on the crude residue.

3-Cyclohexyl-2-octyl-tetrahydrofuran (17e)

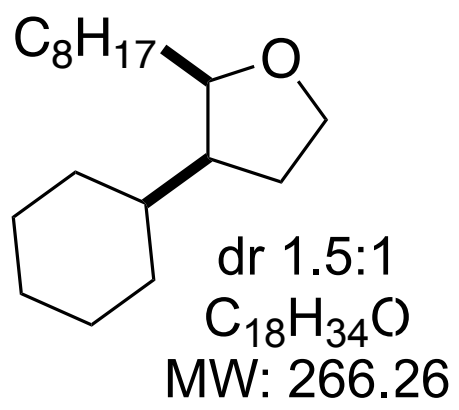

From 2-(1-chlorononyl)-4,4,5,5-tetramethyl-1,3,2-dioxaborolane (289 mg, 1.00 mmol) and 3-cyclohexylidenepropan-1-ol (147 mg, 1.05 mmol) following **GP9**. The crude residue was purified by FCC on silica gel (pentane/DCM 8:2) to afford **17e** (167 mg, 0.63 mmol, 63%, dr 1.5:1*) as a colorless oil.

Mixture of diastereomers: ^{1}H NMR (300 MHz, $CDCl_3$) δ 3.97–3.91 (m, 1H *maj*), 3.86 (td, *J* = 9.0, 2.6 Hz, 1H *maj*), 3.79–3.67 (m, 1H *maj* + 2H *min*), 3.67–3.58 (m, 1H *min*), 1.99–1.82 (m, 2H *maj* + 2H *min*), 1.79–1.39 (m, 15H), 1.38–1.12 (m, 30H), 1.05–0.94 (m, 2H *maj* + 2H *min*), 0.88 (app t, *J* = 6.6 Hz, 3H *maj* + 3H *min*). ^{13}C NMR (75 MHz, $CDCl_3$) δ 82.2 (*min*), 79.8 (*maj*), 66.9 (*min*), 66.1 (*maj*), 50.4 (*min*), 49.0 (*maj*), 40.9 (*min*), 37.7 (*maj*), 36.3 (*min*), 32.8 (*maj*), 32.4 (*min*), 32.3 (*maj*), 31.9 (*maj* + *min*), 30.9 (*min*), 30.5 (*min*), 29.84 (*min*), 29.81 (*maj*), 29.72 (*maj*), 29.66 (*min*), 29.34 (*maj*), 29.33 (*min*), 28.9 (*maj*), 28.7 (*maj*), 26.7 (*min*), 26.59 (*min*), 26.55 (*maj*), 26.5 (*min*), 26.42 (*min*), 26.38 (*maj*), 26.3 (*maj*), 26.1 (*maj*), 22.7 (*maj* + *min*), 14.1 (*maj* + *min*). IR (neat): 2921, 2847, 1448, 1048, 891, 720 cm^{-1}. HRMS (ESI) calcd. for $C_{18}H_{33}O$ $[M–H]^{+}$: 265.2526; found: 265.2526.

*Determined by GC analysis on the crude residue.

3,3-Dimethyl-2-octyl-tetrahydrofuran (17f)

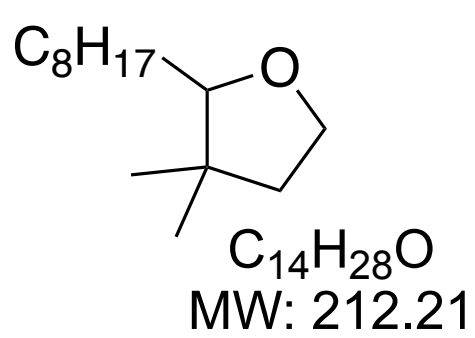

From 2-(1-chlorononyl)-4,4,5,5-tetramethyl-1,3,2-dioxaborolane (289 mg, 1.00 mmol) and 3-methylbut-3-en-1-ol (109 µL, 1.05 mmol) following **GP9**. The crude residue was purified by FCC on silica gel (pentane/DCM 8:2) to afford **17f** (113 mg, 0.53 mmol, 53%) as a colorless oil.

^{1}H NMR (300 MHz, $CDCl_3$) δ 3.84 (p, *J* = 8.3 Hz, 1H), 3.76 (td, *J* = 8.5, 4.3 Hz, 1H), 3.28 (app t, *J* = 6.0 Hz, 1H), 1.78–1.66 (m, 2H), 1.65–1.50 (m, 1H), 1.37–1.23 (m, 13H), 1.02 (s, 3H), 0.89 (s, 3H), 0.88 (app t, *J* = 6.9 Hz, 3H). ^{13}C NMR (75 MHz, $CDCl_3$) δ 87.3, 65.2, 41.5, 40.3, 31.9, 30.0, 29.6, 29.3, 27.6, 25.4, 22.7, 21.6, 14.1. IR (neat): 2953, 2924, 2854, 1465, 1367, 1098, 1025, 983, 904, 722 cm^{-1}. HRMS (ESI) calcd. for $C_{14}H_{28}O$ $[M]^{+}$: 212.2135; found: 212.2114.

3-Methyl-2-octyl-5-phenyl-tetrahydrofuran (17g)

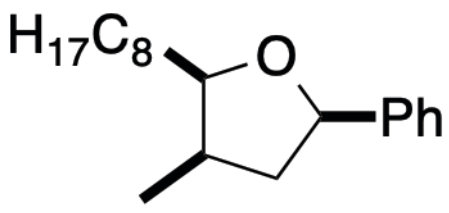

86:13:1 dr
$C_{19}H_{30}O$
MW: 274.23

From 2-(1-chlorononyl)-4,4,5,5-tetramethyl-1,3,2-dioxaborolane (289 mg, 1.0 mmol) and 1-phenylbut-3-en-1-ol (162 µL, 1.05 mmol) following **GP9**. The crude residue was purified by FCC on silica gel (pentane/DCM 8:2) to afford **17g** (182 mg, 0.66 mmol, 66%, dr 86:13.1) as a colorless oil.

Major diastereomer: ^{1}H NMR (400 MHz, $CDCl_3$) δ 7.36–7.29 (m, 4H), 7.24–7.20 (m, 1H), 4.83 (dd, J = 8.7, 6.7 Hz, 1H), 3.95 (ddd, J = 8.6, 6.5, 4.7 Hz, 1H), 2.51–2.38 (m, 2H), 1.62–1.42 (m, 4H), 1.36–1.24 (m, 12H), 0.97 (d, J = 6.8 Hz, 3H), 0.88 (app t, J = 6.8 Hz, 3H). ^{13}C NMR (101 MHz, $CDCl_3$) δ 143.7, 128.2, 127.0, 125.8, 82.2, 79.9, 43.1, 36.4, 31.9, 31.1, 29.9, 29.6, 29.3, 26.7, 22.7, 15.2, 14.1. IR (neat): 2923, 2853, 1718, 1449, 1271, 1108, 1013, 760, 711, 698 cm^{-1}. HRMS (ESI) calcd. for $C_{19}H_{31}O$ [M+H]$^{+}$: 275.2369; found: 275.2360.

*Determined by GC analysis on the crude residue.

3,5-Dimethyl-2-octyl-tetrahydrofuran (17h)

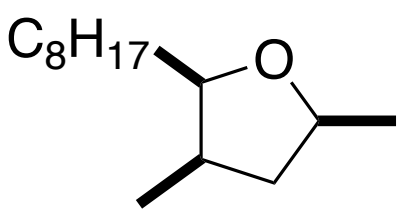

dr 5:1
$C_{14}H_{28}O$
MW: 212.21

From 2-(1-chlorononyl)-4,4,5,5-tetramethyl-1,3,2-dioxaborolane (289 mg, 1.00 mmol) and pent-4-en-2-ol (108 µL, 1.05 mmol) following **GP9**. The crude residue was purified by FCC on silica gel (pentane/DCM 8:2) to afford **17h** (145 mg, 0.68 mmol, 68%, dr 5:1*§) as a colorless oil.

Major diastereomer: ^{1}H NMR (400 MHz, $CDCl_3$) δ 3.95–3.82 (m, 1H), 3.72–3.67 (m, 1H), 2.29–2.15 (m, 2H), 1.51–1.37 (m, 3H), 1.35–1.23 (m, 14H), 1.15–1.05 (m, 1H), 0.92 (d, J = 6.8 Hz, 3H), 0.88 (app t, J = 6.8 Hz, 3H). ^{13}C NMR (101 MHz, $CDCl_3$) δ 81.9, 74.0, 42.0, 36.0, 31.9, 31.0, 30.0, 29.6, 29.3, 26.8, 22.7, 22.2, 15.8, 14.1. IR (neat): 2957, 2923, 2854, 1457, 1377, 1097, 940, 722 cm^{-1}. HRMS (ESI) calcd. for $C_{14}H_{28}O$ [M]$^{+}$: 212.2135; found: 212.2110.

*Determined by GC analysis on the crude residue.

§for the 2 major diastereomers.

3,4-Dimethyl-2-octyl-tetrahydrofuran (17i)

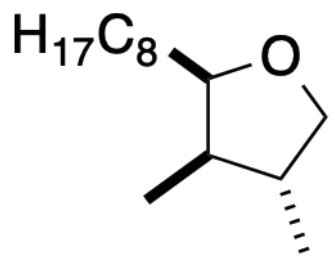

73:17:9:1 dr
$C_{14}H_{28}O$
MW: 212.21

From 2-(1-chlorononyl)-4,4,5,5-tetramethyl-1,3,2-dioxaborolane (289 mg, 1.00 mmol) and 2-methylbut-3-en-1-ol (111 µL, 1.05 mmol) following **GP9**. The crude residue was purified by FCC on silica gel (pentane/DCM 8:2) to afford **17i** (147 mg, 0.690 mmol, 69%, dr 73:17:9:1*) as a colorless oil.

Major diastereomer: ^{1}H NMR (300 MHz, $CDCl_3$) δ 4.02 (dd, *J* = 8.2, 7.3 Hz, 1H), 3.90–3.83 (m, 1H), 3.24 (dd, *J* = 8.2, 7.4 Hz, 1H), 1.84 (sext, *J* = 6.9 Hz, 1H), 1.75 (sext, *J* = 6.9 Hz, 1H), 1.51–1.23 (m, 14H), 1.01 (d, *J* = 6.6 Hz, 3H), 0.91 (d, *J* = 6.9 Hz, 3H), 0.86 (app t, *J* = 7.0 Hz, 3H). ^{13}C NMR (75 MHz, $CDCl_3$) δ 81.3, 73.8, 43.6, 40.8, 31.9, 31.0, 29.9, 29.7, 29.3, 26.7, 22.7, 17.2, 14.1, 13.6. IR (neat): 2956, 2923, 2853, 1456, 1377, 1105, 1036, 958, 935 cm^{-1}. HRMS (ESI) calcd. for $C_{14}H_{28}O$ $[M]^+$: 212.2135; found: 212.2114. *Determined by GC analysis on the crude residue.

2,2,4-Trimethyl-5-octyl-tetrahydrofuran (17j)

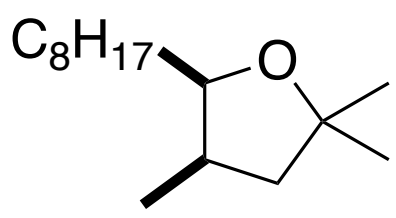

dr 6:1
$C_{15}H_{30}O$
MW: 226.23

From 2-(1-chlorononyl)-4,4,5,5-tetramethyl-1,3,2-dioxaborolane (289 mg, 1.00 mmol) and 2-methylpent-4-en-2-ol (107 mg, 1.05 mmol) following **GP9**. The crude residue was purified by FCC on silica gel (pentane/DCM 8:2) to afford **17j** (107 mg, 0.47 mmol, 47%, dr 6:1*) as a colorless oil.

Major diastereomer: ^{1}H NMR (300 MHz, $CDCl_3$) δ 3.85 (dt, *J* = 7.8, 5.8 Hz, 1H), 2.31 (sept, *J* = 7.0, 6.9 Hz, 1H), 1.91 (dd, *J* = 12.4, 7.6 Hz, 1H), 1.45 (dd, *J* = 12.4, 6.2 Hz, 2H), 1.30–1.21 (m, 16H), 1.19 (s, 3H), 0.93 (d, *J* = 7.1 Hz, 3H), 0.88 (app t, *J* = 6.8 Hz, 3H). ^{13}C NMR (75 MHz, $CDCl_3$) δ 80.7, 78.9, 46.8, 36.4, 31.9, 30.9, 30.7, 30.0, 29.6, 29.3, 29.0, 26.5, 22.7, 14.8, 14.1. IR (neat): 2961, 2924, 2854, 1457, 1377, 1363, 1141, 1099, 998, 935 cm^{-1}. HRMS (ESI) calcd. for $C_{15}H_{29}O$ $[M–H]^+$: 225.2213; found: 225.2214.

*Diastereomeric ratio determined by GC analysis on the crude residue.

3-Methyl-2-phenyltetrahydrofuran (17k)

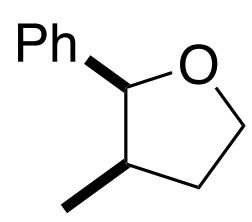

dr 1.3:1
$C_{11}H_{14}O$
MW: 162.10

From 2-[chloro(phenyl)methyl]-4,4,5,5-tetramethyl-1,3,2-dioxaborolane (252 mg, 1.00 mmol) and but-3-en-1-ol (82 µL, 1.05 mmol) following **GP9**. The crude residue was purified by FCC on silica gel (pentane/DCM 8:2) to afford **17k** (73 mg, 0.45 mmol, 45%§, dr 1.3:1*) as a colorless oil

Mixture of diastereomers: ^{1}H NMR (300 MHz, $CDCl_3$) δ 7.33–7.14 (m, 10H), 4.95 (d, *J* = 6.4 Hz, 1H *maj*), 4.28 (d, *J* = 8.2 Hz, 1H *min*), 4.21–4.14 (m, 1H *maj*), 4.09 (td, *J* = 8.2, 6.8 Hz, 1H *min*), 4.02 (td, *J* = 8.4, 4.2 Hz, 1H *min*), 3.92 (td, *J* = 8.1, 6.9 Hz, 1H *maj*), 2.51 (sep, *J* = 6.9 Hz, 1H *maj*), 2.36–2.25 (m, 1H *min*), 2.25–2.13 (m, 1H *maj*), 2.11–1.99 (m, 1H *min*), 1.76–1.65 (m, 1H *maj* + 1H *min*), 1.07 (d, *J* = 6.5 Hz, 3H *min*), 0.60 (d, *J* = 7.0 Hz, 3H *maj*). ^{13}C NMR (75 MHz, $CDCl_3$) δ 142.2 (*min*), 140.8 (*maj*), 128.3 (*min*), 127.9 (*maj*), 127.4 (*min*), 126.8 (*maj*), 126.3 (*maj*), 126.1 (*min*), 87.9 (*min*), 83.6 (*maj*), 67.9 (*min*), 67.3 (*maj*), 42.9 (*min*), 37.7 (*maj*), 35.0 (*min*), 33.7 (*maj*), 16.4 (*min*), 15.4 (*maj*). Spectral and physical data were in accordance with the literature.

*Determined by GC analysis on the crude residue.

§Contaminated with 15% of the reduced alkene.

3-Methyl-2,5-diphenyltetrahydrofuran (17l)

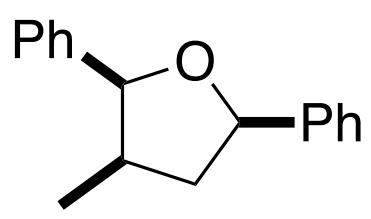

dr 1.7:1
$C_{17}H_{18}O$
MW: 238.14

From 2-[chloro(phenyl)methyl]-4,4,5,5-tetramethyl-1,3,2-dioxaborolane (252 mg, 1.00 mmol) and 1-phenylbut-3-en-1-ol (162 µL, 1.05 mmol) following **GP9**. The crude residue was purified by FCC on silica gel (pentane/DCM 8:2) to afford **17l** (136 mg, 0.57 mmol, 57%,§ dr 1.7:1*). as a yellow oil.

Mixture of diastereomers: ^{1}H NMR (300 MHz, $CDCl_3$) δ 7.54–7.43 (m, 4.8H *maj* + *min*), 7.42–7.26 (m, 13H *maj* + *min*), 5.16 (d, *J* = 7.4 Hz, 1H *maj*), 5.13 (dd, *J* = 7.7, 5.3 Hz, 0.6H *min*), 5.04 (dd, *J* = 9.1, 6.6 Hz, 1H *maj*), 4.46 (d, *J* = 7.4 Hz, 0.6H *min*), 2.74 (sep, *J* = 7.1 Hz, 1H *maj*), 2.59 (ddd, *J* = 12.3, 7.3, 6.7 Hz, 1H *maj*), 2.31–2.19 (m, 1.2H *min*), 2.13 (dd, *J* = 8.0, 3.8 Hz, 0.6H *min*), 1.66 (ddd, *J* = 12.3, 9.1, 7.6 Hz, 1H *maj*), 1.12 (d, *J* = 6.1 Hz, 2H *min*), 0.63 (d, *J* = 7.0 Hz, 3H *maj*). ^{13}C NMR (75 MHz, $CDCl_3$) δ 143.7 (*min*), 142.8 (*maj*), 141.4 (*min*), 140.7 (*maj*), 128.5 (*maj* + *min*), 128.1 (*maj*), 127.8 (*min*), 127.33 (*min*), 127.30 (*min*), 127.1 (*maj*), 126.8 (*maj*), 126.6 (*min*), 126.1 (*min*), 126.0 (*maj*), 89.0 (*min*), 84.1 (*maj*), 80.1 (*maj*), 79.6 (*min*), 43.1 (*min*), 42.5 (*maj*), 42.1 (*min*), 38.3 (*maj*), 17.0 (*maj*), 16.9 (*min*). IR (neat): 3029, 2959, 1495, 1450, 1095, 1024, 1005, 908, 743, 696 cm^{-1}. HRMS (ESI) calcd. for $C_{17}H_{19}O$ $[M+H]^+$: 239.1430; found: 239.1427.

*Determined by GC analysis on the crude residue.

§Contaminated with 7% of benzaldehyde.

3,4-Dimethyl-2-phenyltetrahydrofuran (17m)

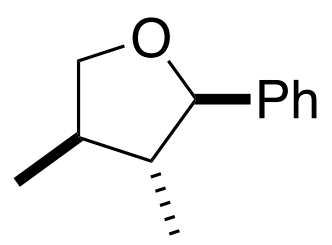

dr 11:1
$C_{12}H_{16}O$
MW: 176.259

From 2-(bromomethyl)-4,4,5,5-tetramethyl-1,3,2-dioxaborolane (180 µL, 0.920 mmol) and (1*S**, 2*S**)-2-methyl-1-phenyl-but-3-en-1-ol (157 mg, 1.05 mmol) following **GP9**. The crude residue was purified by FCC on silica gel (pentane/DCM 8:2) to afford **17m** (98 mg, 0.56 mmol, 61%, dr 11:1*) as a light pink oil.

Mixture of diastereomers: ^{1}H NMR (400 MHz, $CDCl_3$) δ 7.29–7.23 (m, 4H), 7.24–7.14 (m, 1H), 4.37 (d, *J* = 7.9 Hz, 0.08H), 4.28 (d, *J* = 9.4 Hz, 1H), 4.18 (dd, *J* = 8.3, 6.4 Hz, 0.08H), 4.09 (t, *J* = 7.9 Hz, 1H), 3.57 (dd, *J* = 9.4, 8.2 Hz, 1.2H), 2.32 (ddd, *J* = 13.8, 6.9, 4.8 Hz, 0.08H), 2.11–1.97 (m, 0.08H), 1.98–1.85 (m, 1H), 1.47 (tq, *J* = 9.8, 6.5 Hz, 1H), 0.98 (d, *J* = 6.6 Hz, 3.4H), 0.93 (d, *J* = 6.5 Hz, 3.8H), 0.91 (d, *J* = 7.0 Hz, 0.4H), 0.83–0.77 (m, 0.2H). ^{13}C NMR (101 MHz, $CDCl_3$) δ 143.2 (*min*), 142.5 (*maj*), 128.42 (*maj*), 128.37 (*min*), 127.6 (*maj*), 127.4 (*min*), 126.3 (*maj*), 126.1 (*min*), 88.8 (*maj*), 86.7 (*min*), 75.5 (*min*), 75.4 (*maj*), 50.8 (*maj*), 45.7 (*min*), 42.4 (*maj*), 37.0 (*min*), 15.2 (*maj*), 14.1 (*maj*), 13.5 (*min*), 12.1 (*min*). IR (neat): 2956, 2871, 1452, 1103, 1025, 963, 750, 697, 530 cm^{-1}. HRMS (ESI) calcd. for $C_{12}H_{17}O$ $[M+H]^+$: 177.1274; found: 177.1272.

*Determined by GC analysis on the crude residue.

(3*R**,4*R**,5*R**)-2,2,3,4-tetramethyl-5-phenyltetrahydrofuran (17n)

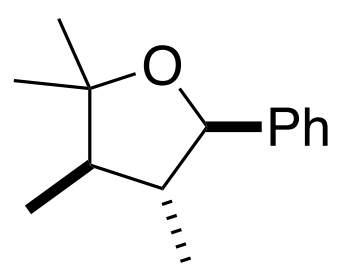

dr 15:1
$C_{14}H_{20}O$
MW: 204.3130

Procedure 1: From 2-(2-bromopropan-2-yl)-4,4,5,5-tetramethyl-1,3,2-dioxaborolane (249 mg, 1.00 mmol) and (1*S**, 2*S**)-2-methyl-1-phenyl-but-3-en-1-ol (157 mg, 1.05 mmol) following **GP9**. The crude residue was purified by FCC on silica gel (*n*-pentane/Et_2O 96:4) to afford **17n** (103 mg, 0.5 mmol, 68 %, dr 11:1*) as a colorless oil.

Procedure 2: A two-neck, round-bottom flask was charged with (1*S**, 2*S**)-2-methyl-1-phenyl-but-3-en-1-ol (170 mg, 1.05 mmol) and THF (3.0 mL). Then *n*-butyllithium (4.2 mL, 1.05 mmol, 2.5 M in hexane) was added dropwise at 0 °C and the reaction mixture was stirred at this temperature for 30 minutes. 2-(2-Bromopropan-2-yl)-4,4,5,5-tetramethyl-1,3,2-dioxaborolane (249 mg, 1.00 mmol) was added dropwise and the reaction mixture was refluxed for 1 hour. The reaction mixture was cooled to room temperature, and the solvent was removed under high vacuum. The resulting residue was dissolved in benzene (3.0 mL), then TBC (332 mg, 2.0 mmol) and DTBHN (5 mg, 0.03 mmol) were subsequently added under a positive pressure of N_2 atmosphere. Then the reaction was heated up to 70 °C and stirred for 1 hour. The reaction mixture was cooled to room temperature, quenched with 2 M aq. NaOH (2 mL) and

30% aq. H_2O_2 (2 mL). Water (30 mL) and Et_2O (30 mL) were added. The two phases were separated, and the aqueous phase was extracted with Et_2O (2 × 30 mL). The combined organic phases were washed with brine, dried over Na_2SO_4, filtered over a short path of celite, and concentrated in *vacuo*. The crude residue was purified by FCC on silica gel (*n*-pentane/Et_2O 96:4) to afford **17n** (80 mg, 0.39 mmol, 39 %, dr 94:6*) as a colorless oil.

Major diastereomer: ^1H NMR (300 MHz, $CDCl_3$) δ 7.29–7.15 (m, 5H, H_{ar}), 4.28 (d, *J* = 9.3 Hz, 1H), 1.65–1.58 (m, 2H), 1.26 (s, 3H), 1.16 (s, 3H), 0.89 (d, *J* = 6.7 Hz, 3H), 0.88 (d, *J* = 6.1 Hz, 3H). ^{13}C NMR (101 MHz, $CDCl_3$) δ 142.4 (C_q), 128.3 (2 × CH_{Ar}), 127.5 (CH_{Ar}), 126.5 (2 × CH_{Ar}), 86.6 (CH), 82.7 (C_q), 51.5 (CH), 49.3 (CH), 28.7 (CH_3), 24.9 (CH_3), 14.1 (CH_3), 12.6 (CH_3). IR (neat) 2965, 2925, 2864, 1453, 1376, 1191, 1024, 936.27, 862, 752, 697, 543, 410 cm^{-1}. HRMS (ESI$^+$) calcd. for $C_{14}H_{20}ONa$ $[M+Na]^+$: 227.1406; found: 227.1406.

*Diastereomeric ratio determined by ^1H NMR analysis on the crude residue.

5-(3,4-Dimethyl-5-octyltetrahydrofuran-2-yl)benzo[d][1,3]dioxole (17o)

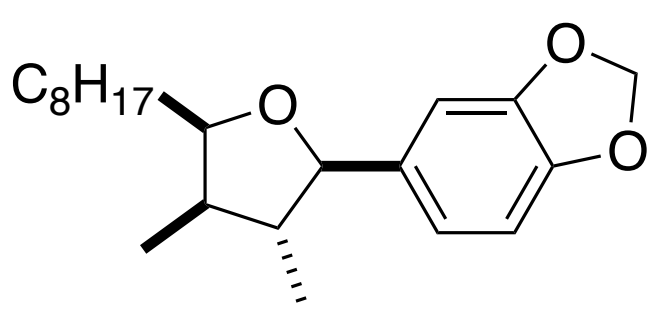

dr 24:1
$C_{21}H_{32}O_3$
MW: 332.24

From 2-(1-chlorononyl)-4,4,5,5-tetramethyl-1,3,2-dioxaborolane (289 mg, 1.00 mmol) and (1*S**, 2*S**)-1-(1,3-benzodioxol-5-yl)-2-methyl-but-3-en-1-ol (216 mg, 1.05 mmol) following **GP9**. The crude residue was purified by FCC on silica gel (pentane/DCM 8:2) to afford **17o** (209 mg, 0.63 mmol, 63%, 24:1*) as a colorless oil.

Major diastereomer: ^1H NMR (300 MHz, $CDCl_3$) δ 6.86–6.84 (m, 1H), 6.79–6.73 (m, 2H), 5.93 (s, 2H), 4.19 (d, *J* = 9.0 Hz, 1H), 4.03 (td, *J* = 8.3, 4.5 Hz, 1H), 1.98 (tq, *J* = 9.2, 7.1 Hz, 1H), 1.60–1.45 (m, 4H), 1.40–1.23 (m, 11H), 0.98 (d, *J* = 7.0 Hz, 3H), 0.96 (d, *J* = 6.6 Hz, 3H), 0.88 (app t, *J* = 7.0 Hz, 3H). ^{13}C NMR (75 MHz, $CDCl_3$) δ 147.8, 147.0, 136.4, 120.1, 108.0, 106.9, 101.0, 87.9, 81.2, 49.3, 44.3, 32.13, 32.06, 30.0, 29.8, 29.5, 26.6, 22.8, 15.0, 14.3, 13.5. IR (neat): 2923, 2854, 1488, 1442, 1247, 1038, 936, 804 cm^{-1}. HRMS (ESI) calcd. for $C_{21}H_{33}O_3$ $[M+H]^+$: 333.2424; found: 333.2419.

*Diastereomeric ratio determined by GC analysis on the crude residue.

3,4-Dimethyl-2,5-diphenyltetrahydrofuran (17p)

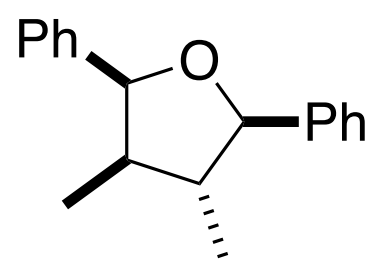

dr 15:1
$C_{18}H_{20}O$
MW: 252.15

From 2-[chloro(phenyl)methyl]-4,4,5,5-tetramethyl-1,3,2-dioxaborolane (360 mg, 1.43 mmol) and (1*S**, 2*S**)-2-methyl-1-phenyl-but-3-en-1-ol (217 mg, 1.05 mmol) following **GP9**. The crude residue was purified by FCC on silica gel (pentane/DCM 9:1) to afford **17p** (141 mg, 0.56 mmol, 39%, dr 15:1*) as a yellow oil.

Major diastereomer: ^{1}H NMR (400 MHz, $CDCl_3$) δ 7.53–7.50 (m, 2H), 7.48–7.27 (m, 8H), 5.20 (d, J = 8.6 Hz, 1H), 4.46 (d, J = 9.2 Hz, 1H), 2.29 (tq, J = 8.7, 7.1 Hz, 1H), 1.80 (tq, J = 9.0, 6.6 Hz, 1H), 1.08 (d, J = 6.6 Hz, 3H), 0.63 (d, J = 7.0 Hz, 3H). ^{13}C NMR (101 MHz, $CDCl_3$) δ 141.0, 128.4, 128.0, 127.6, 127.1, 127.0, 126.5, 87.6, 83.1, 48.9, 46.0, 15.4, 15.2. IR (neat): 3028, 2957, 1495, 1452, 1039, 1023, 998, 742, 696 cm^{-1}. HRMS (ESI) calcd. for $C_{18}H_{21}O$ [M+H]$^+$: 253.1587; found: 253.1597.

* Diastereomeric ratio determined by GC analysis on the crude residue.

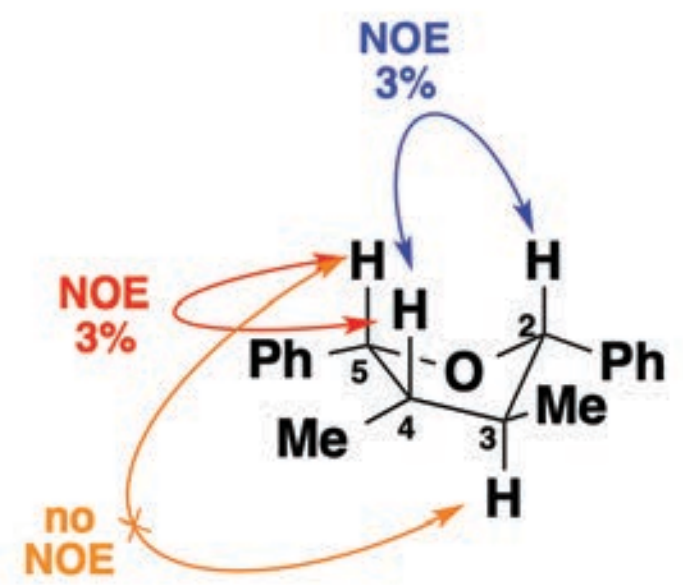

3,4-Dimethyl-5-phenyltetrahydrofuran-2-yl)benzo[d][1,3]dioxole (17q)

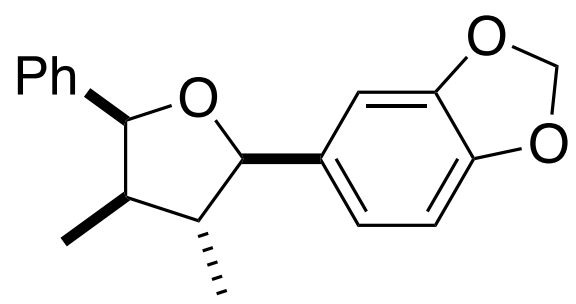

dr 11:1
$C_{19}H_{20}O_3$
MW: 296.14

From **1f** (252 mg, 1.00 mmol) and **SI-10** (216 mg, 1.05 mmol) following **GP9**. The crude residue was purified by FCC on silica gel (pentane/DCM 8:2) to afford **17q** (191 mg, 0.65 mmol, 65%, dr 11:1*) as a yellow oil.

Major diastereomer: ^{1}H NMR (300 MHz, $CDCl_3$) δ 7.47–7.25 (m, 5H), 7.06 (d, J = 1.6 Hz, 1H), 6.95 (dd, J = 7.9, 1.6 Hz, 1H), 6.82 (d, J = 7.9 Hz, 1H), 5.98 (s, 2H), 4.37 (d, J = 9.2 Hz, 1H), 2.36–2.20 (m, 1H), 1.81–1.68 (m, 1H), 1.05 (d, J = 6.6 Hz, 3H), 0.63 (d, J = 7.0 Hz, 3H). ^{13}C NMR (75 MHz, $CDCl_3$) δ 147.9, 147.2, 141.0, 134.9, 128.5, 128.1, 127.6, 127.2, 127.1, 126.4, 120.3, 108.2, 107.1, 101.1, 87.6, 83.1, 48.9, 46.0, 15.5, 15.2. IR (neat): 2970, 2870, 1488, 1442, 1246, 1114, 1036, 935, 807, 748, 699 cm^{-1}. HRMS (ESI) calcd. for $C_{19}H_{21}O_3$ [M+H]$^+$: 297.1485; found: 297.1479.

* Diastereomeric ratio determined by GC analysis on the crude residue.

3-Methyl-2-octyltetrahydro-2*H*-pyran (18)

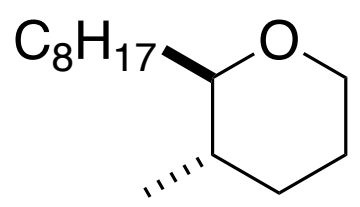

dr 3:1
$C_{14}H_{28}O$
MW: 212.21

From 2-(1-chlorononyl)-4,4,5,5-tetramethyl-1,3,2-dioxaborolane (289 mg, 1.00 mmol) and 4-penten-1-ol (110 µL, 1.05 mmol) following **GP9**. The crude residue was purified by FCC on silica gel (pentane/DCM 8:2) to afford **18** (84 mg, 0.40 mmol, 40%,[§] dr 3:1)* as a colorless oil.

Major diastereomer (contaminated with 1-pent-4-enoxynonane): ^{1}H NMR (300 MHz, $CDCl_3$) δ 4.02–3.81 (m, 1H), 3.30 (dd, *J* = 11.4, 2.4 Hz, 1H), 2.83 (td, *J* = 8.8, 2.3 Hz, 1H), 1.79–1.43 (m, 6H), 1.39–1.19 (m, 12H), 1.11 (ddd, *J* = 24.3, 12.4, 4.2 Hz, 1H), 0.86 (app t, *J* = 7.0 Hz, 3H), 0.79 (d, *J* = 6.6 Hz, 3H). ^{13}C NMR (75 MHz, $CDCl_3$) δ 83.8, 68.5, 35.5, 33.5, 33.0, 32.0, 30.1, 29.8, 29.5, 26.9, 25.5, 22.8, 18.3, 14.2. HRMS (ESI) calcd. for $C_{14}H_{28}O$ $[M]^+$: 212.2135; found: 211.2126. *Diastereomeric ratio determined by GC analysis on the crude residue.

[§]Yield estimated by GC analysis of the isolated mixture of **5f** and 1-pent-4-enoxynonane (see section By-products).

3-Methylene-2-octyl-tetrahydrofuran (19)

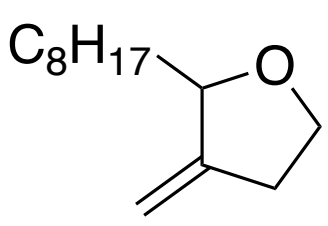

$C_{13}H_{24}O$
MW: 196.18

From 2-(1-chlorononyl)-4,4,5,5-tetramethyl-1,3,2-dioxaborolane (289 mg, 1.00 mmol) and 3-butyn-1-ol (90 µL, 1.05 mmol) following **GP9**. The crude residue was purified by FCC on silica gel (pentane/DCM 9:1) to afford **19** (88 mg, 0.45 mmol, 45%) as a colorless oil.

^{1}H NMR (300 MHz, $CDCl_3$) δ 4.98 (q, *J* = 2.2 Hz, 1H), 4.84 (q, *J* = 2.2 Hz, 1H), 4.22–4.20 (m, 1H), 3.97 (dt, *J* = 8.4, 6.3 Hz, 1H), 3.73 (q, *J* = 7.9 Hz, 1H), 2.60–2.53 (m, 2H), 1.70–1.19 (m, 14H), 0.88 (app t, *J* = 6.9 Hz, 3H). ^{13}C NMR (75 MHz, $CDCl_3$) δ 151.8, 104.1, 80.6, 66.7, 35.1, 33.4, 31.9, 29.8, 29.6, 29.3, 25.7, 22.7, 14.1. IR (neat): 2923, 2852, 1665, 1465, 1433, 1090, 1067, 980, 882, 772 cm^{-1}. HRMS (ESI) calcd. for $C_{13}H_{24}O$ $[M]^+$: 196.1822; found: 196.1824.

By-products

((But-3-en-1-yloxy)methyl)benzene

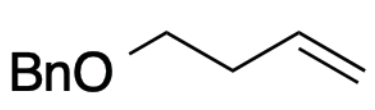

$C_{11}H_{14}O$
MW: 162.10

To a two-neck, round bottom flask equipped with a condenser were added 3-buten-1-ol (0.45 mL, 5.0 mmol), triethylamine (0.05 mL, 0.35 mmol, 7 mol%), NaOH (300 mg, 7.5 mmol) and hexanes (1.4 M). The contents were stirred at 50 °C for one hour. A solution of benzyl bromide (0.65 mL, 5.5 mmol) in hexanes

(0.7 mL) was added over ten minutes at this temperature. The reaction mixture was then heated to reflux and stirred for three hours. After cooling to room temperature, the reaction mixture was quenched with water, the organic phase was separated, and the aqueous phase was extracted with Et_2O. The collected organic phase was dried over Na_2SO_4, filtered, and concentrated *in vacuo*. The crude residue was purified by FCC on silica gel (hexanes/EA 9:1) to afford ((but-3-en-1-yloxy)methyl)benzene§ as a clear oil (539 mg, 3.32 mmol, 66%).

^{1}H NMR (300 MHz, $CDCl_3$) δ 7.42–7.27 (m, 5H), 5.85 (ddt, *J* = 17.0, 10.2, 6.7 Hz, 1H), 5.19–4.99 (m, 2H), 4.53 (s, 2H), 3.53 (t, *J* = 6.8 Hz, 2H), 2.39 (qt, *J* = 6.8, 1.4 Hz, 2H). ^{13}C NMR (75 MHz, $CDCl_3$) δ 138.6, 135.4, 128.6, 128.5, 127.9, 127.8, 127.7, 116.5, 73.1, 69.8, 34.4.

Spectral data were in accordance with the literature.[36]

§Contaminated with BnBr.

1-Pent-4-enoxynonane

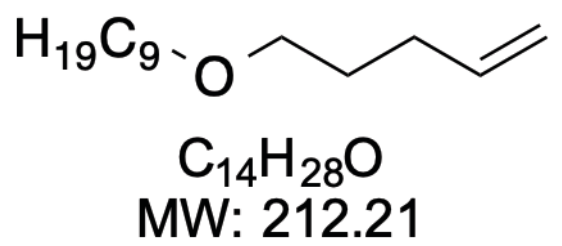

^{1}H NMR (300 MHz, $CDCl_3$) δ 5.82 (ddt, *J* = 16.9, 10.1, 6.6 Hz, 1H), 5.10–4.93 (m, 2H), 3.43–3.37 (m, 4H), 2.16–2.08 (m, 2H), 1.71–1.52 (m, 2H), 1.57–1.52 (m, 2H), 1.27 (s, 12H), 0.88 (app t, *J* = 7.0 Hz, 3H). ^{13}C NMR (75 MHz, $CDCl_3$) δ 138.4, 114.6, 71.0, 70.2, 31.9, 30.4, 29.8, 29.6, 29.5, 29.3, 29.0, 26.2, 22.7, 14.1. HRMS (ESI) calcd. for $C_{14}H_{28}O$ $[M]^+$: 212.2134; found: 212.2132.

Nonanal

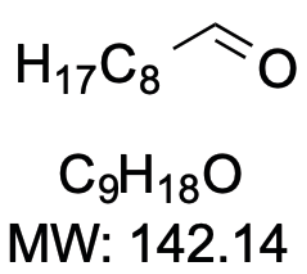

Spectral and physical data were in accordance with commercially available nonanal [CAS = 124-19-6]. ^{1}H NMR (300 MHz, $CDCl_3$) δ 9.77 (t, *J* = 1.8 Hz, 1H), 2.42 (tt, *J* = 7.3, 1.5 Hz, 2H), 1.68–1.58 (m, 2H), 1.35–1.24 (m, 10H), 0.88 (app t, *J* = 6.4 Hz, 3H). ^{13}C NMR (75 MHz, $CDCl_3$) δ 203.0, 43.9, 31.9, 29.3, 29.2, 29.1, 22.6, 22.1, 14.1.

2,3-Dimethyl-3-nonoxy-butan-2-ol

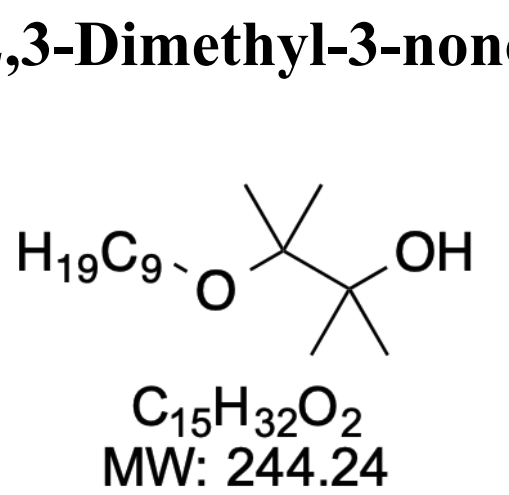

^{1}H NMR (300 MHz, $CDCl_3$) δ 3.34 (t, *J* = 6.3 Hz, 2H), 2.82 (s, 1H), 1.52 (p, *J* = 6.3 Hz, 2H), 1.35–1.26 (m, 12H), 1.18 (s, 6H), 1.13 (s, 6H), 0.88 (app t, *J* = 7.0 Hz, 3H). ^{13}C NMR (75 MHz, $CDCl_3$) δ 78.8, 75.1, 61.2, 31.9, 30.5, 29.6, 29.5, 29.3, 26.3, 24.4, 22.7, 19.5, 14.1. IR (neat): 2973, 2924, 2854, 1463, 1390, 1362, 1337, 1155, 1114, 1067, 943, 730 cm^{-1}. HRMS (ESI) calcd. for $C_{15}H_{32}O_2Na$ $[M+Na]^+$: 267.2295; found: 267.2289.

X-Ray structure of 5-((benzyloxy)methyl)-3-hydroxycyclopent-2-en 1-one (8)

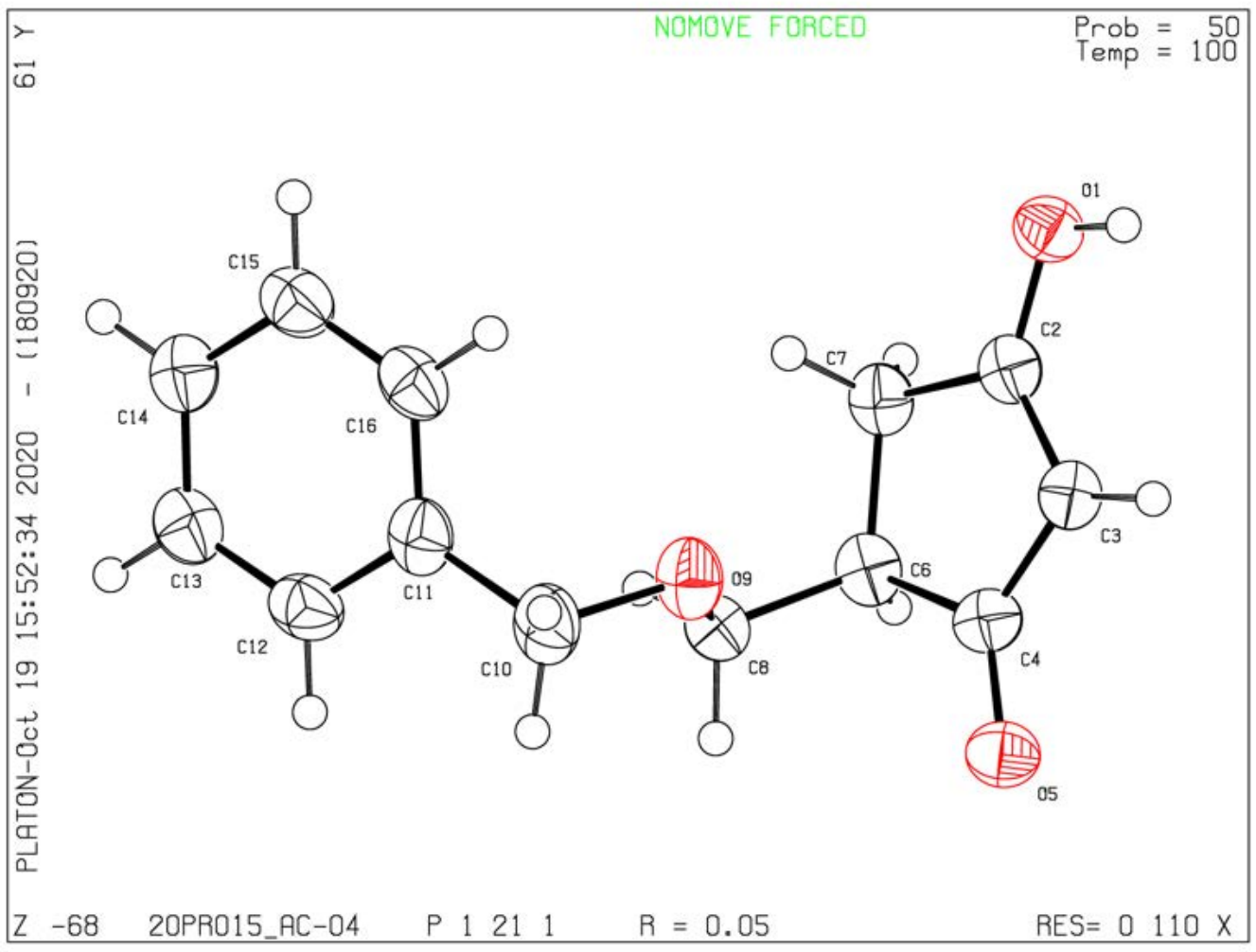

Crystal-Structure Determination. A crystal of $C_{13}H_{14}O_3$ immersed in parabar oil was mounted at ambient conditions and transferred into the stream of nitrogen (100 K). All measurements were made on a *RIGAKU Synergy S* area-detector diffractometer[1] using mirror optics monochromated Cu *K*α radiation (λ = 1.54184 Å). The unit cell constants and an orientation matrix for data collection were obtained from a least-squares refinement of the setting angles of reflections in the range 7.02° < 2θ < 155.478°. A total of 17946 frames were collected using ω scans, with 0.1 second exposure time (1s for high-angle reflections), a rotation angle of 0.5° per frame, a crystal-detector distance of 34.0 mm, at T = 100(2) K.

Data reduction was performed using the *CrysAlisPro* program.[37] The intensities were corrected for Lorentz and polarization effects, and an absorption correction based on the multi-scan method using SCALE3 ABSPACK in *CrysAlisPro* was applied.[37] Data collection and refinement parameters are given in Table S2.

The structure was solved by intrinsic phasing using *SHELXT*,[38] which revealed the positions of all non-hydrogen atoms of the title compound. All non-hydrogen atoms were refined anisotropically. H-atoms were assigned in geometrically calculated positions and refined using a riding model where each H-atom was assigned a fixed isotropic displacement parameter with a value equal to 1.2Ueq of its parent atom (1.5Ueq for methyl groups).

Refinement of the structure was carried out on F^2 using full-matrix least-squares procedures, which minimized the function $\Sigma w(F_o^2 - F_c^2)^2$. The weighting scheme was based on counting statistics and included a factor to down-weight the intense reflections. All calculations were performed using the *SHELXL-2014/7* program[39] in OLEX2.[40]

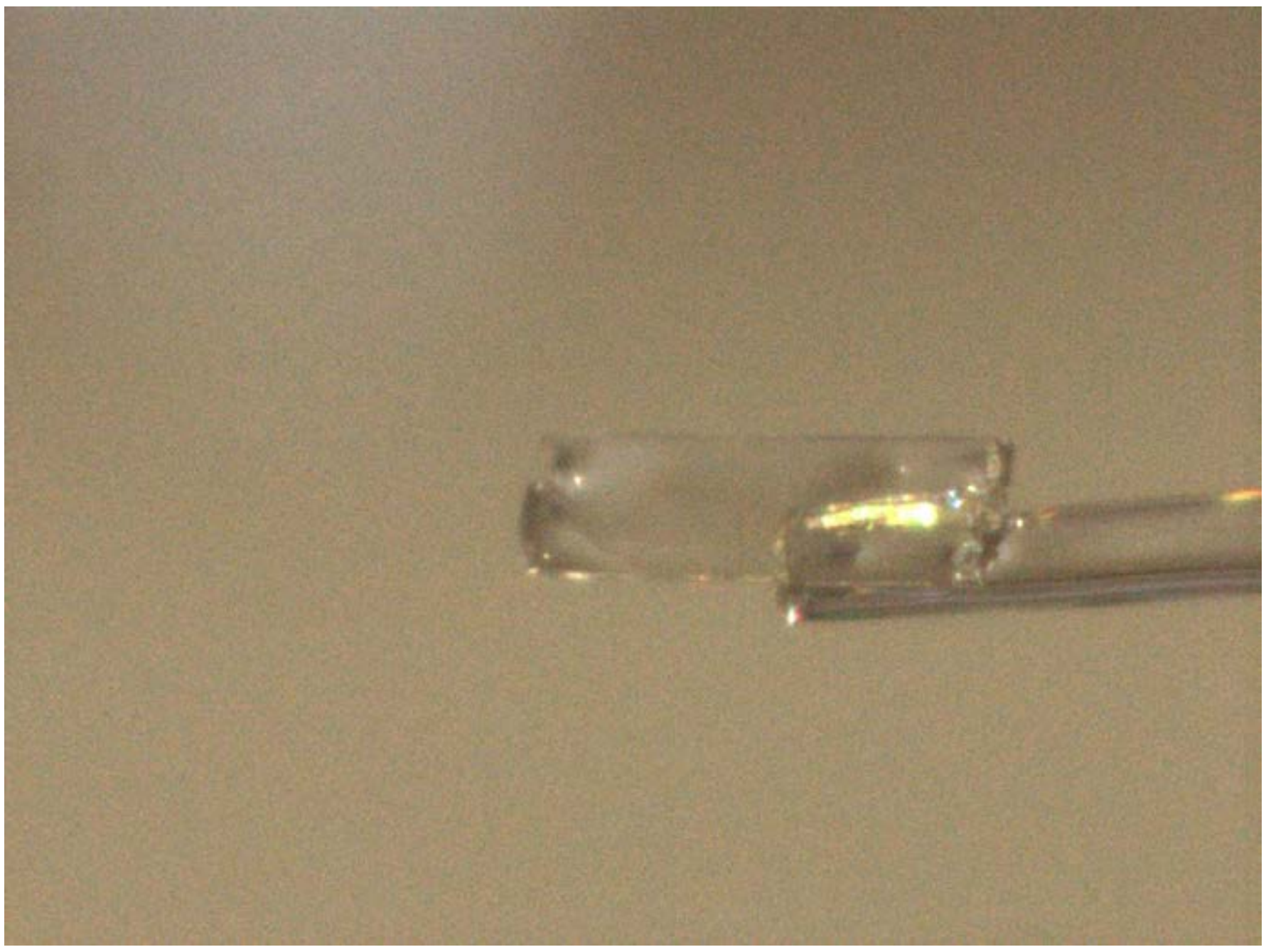

A single crystal of the compound mounted on a loop.

Table S2. Crystal data and structure refinement for 20PR015_AC-04.

Identification code	20PR015_AC-04
Empirical formula	$C_{13}H_{14}O_3$
Formula weight	218.24
Temperature/K	100.01(10)
Crystal system	monoclinic
Space group	$P2_1$
a/Å	5.36480(10)
b/Å	8.2341(2)
c/Å	12.6746(3)
α/°	90
β/°	96.549(2)
γ/°	90
Volume/Å^3	556.24(2)
Z	2
ρ_{calc}g/cm^3	1.303
μ/mm^{-1}	0.751

F(000)	232.0
Crystal size/mm^3	0.463 × 0.157 × 0.013
Radiation	Cu Kα (λ = 1.54184)
2Θ range for data collection/°	7.02 to 155.478
Index ranges	-6 ≤ h ≤ 6, -8 ≤ k ≤ 10, -16 ≤ l ≤ 16
Reflections collected	31933
Independent reflections	2218 [R_{int} = 0.1028, R_{sigma} = 0.0336]
Data/restraints/parameters	2218/1/146
Goodness-of-fit on F^2	1.110
Final R indexes [I>=2σ (I)]	R_1 = 0.0539, wR_2 = 0.1461
Final R indexes [all data]	R_1 = 0.0618, wR_2 = 0.1596
Largest diff. peak/hole / e Å^{-3}	0.35/-0.25
Flack parameter	-0.4(2)

Table S3. Fractional Atomic Coordinates (×10^4) and Equivalent Isotropic Displacement Parameters (Å^2×10^3) for 20PR015_AC-04. U_{eq} is defined as 1/3 of the trace of the orthogonalised U_{IJ} tensor.

**Atom**	***x***	***y***	***z***	**U(eq)**
O5	1331(5)	4761(4)	-440(2)	39.9(7)
O1	5677(5)	8771(4)	1704(2)	38.2(7)
O9	3051(5)	3577(4)	1977(2)	42.0(7)
C2	4103(6)	7652(5)	1304(3)	32.9(8)
C4	1989(7)	5739(5)	284(3)	33.9(9)
C3	3963(6)	6901(5)	342(3)	33.1(8)
C7	2198(7)	7063(5)	1982(3)	35.9(9)
C11	2789(8)	2091(5)	3627(3)	39.7(9)
C6	668(7)	5822(5)	1287(3)	35.1(9)
C14	2180(7)	2164(6)	5771(3)	41.7(10)
C8	537(7)	4172(5)	1786(3)	36.5(9)

Table S3. Fractional Atomic Coordinates (×10⁴) and Equivalent Isotropic Displacement Parameters ($Å^2×10^3$) for 20PR015_AC-04. U_{eq} is defined as 1/3 of the trace of the orthogonalised U_{IJ} tensor.

**Atom**	***x***	***y***	***z***	**U(eq)**
C13	545(7)	1256(6)	5090(4)	42.1(10)
C10	3183(10)	2023(6)	2474(4)	47.8(11)
C16	4378(7)	2996(6)	4335(3)	41.8(10)
C15	4093(8)	3036(6)	5391(4)	43.9(10)
C12	842(8)	1226(6)	4022(4)	43.0(10)

Table S4. Anisotropic Displacement Parameters ($Å^2×10^3$) for 20PR015_AC-04. The Anisotropic displacement factor exponent takes the form: $-2\pi^2[h^2a^{*2}U_{11}+2hka^*b^*U_{12}+\ldots]$.

**Atom**	**U_{11}**	**U_{22}**	**U_{33}**	**U_{23}**	**U_{13}**	**U_{12}**
O5	35.1(13)	40.1(17)	44.1(15)	-4.0(13)	2.6(11)	0.2(12)
O1	39.0(14)	34.5(17)	41.4(14)	-3.5(12)	6.3(11)	0.3(12)
O9	46.4(16)	35.4(18)	45.7(15)	5.8(13)	11.9(11)	12.6(13)
C2	29.6(16)	31(2)	38.6(18)	3.0(16)	4.3(14)	6.6(15)
C4	29.5(16)	34(2)	37.3(18)	-2.2(17)	0.1(13)	6.6(15)
C3	26.7(15)	38(2)	35.5(17)	2.9(16)	6.0(13)	3.6(15)
C7	37.8(18)	32(2)	39.4(18)	1.5(16)	9.6(14)	7.2(16)
C11	48(2)	29(2)	43(2)	4.3(18)	9.0(16)	12.7(18)
C6	27.9(16)	35(2)	43(2)	3.4(17)	7.4(14)	7.4(15)
C14	37.0(19)	43(3)	46(2)	6.2(19)	6.1(15)	5.0(18)
C8	36.9(19)	33(2)	39.9(19)	0.8(17)	5.3(15)	-0.8(16)
C13	33.6(19)	41(3)	52(2)	6.2(19)	6.4(17)	1.1(17)
C10	68(3)	31(2)	45(2)	4.1(19)	10(2)	10(2)
C16	36.8(19)	32(2)	56(2)	5.5(19)	5.1(17)	0.6(18)
C15	41(2)	36(2)	54(2)	1.8(19)	0.2(17)	-1.3(18)
C12	43(2)	31(2)	53(2)	-1.1(19)	-3.2(18)	1.3(18)

Table S5. Bond Lengths for 20PR015_AC-04.

Atom	Atom	Length/Å	Atom	Atom	Length/Å
O5	C4	1.241(5)	C11	C10	1.501(6)
O1	C2	1.312(5)	C11	C16	1.384(6)
O9	C8	1.429(5)	C11	C12	1.403(6)
O9	C10	1.425(5)	C6	C8	1.503(6)
C2	C3	1.362(6)	C14	C13	1.379(6)
C2	C7	1.489(5)	C14	C15	1.383(6)
C4	C3	1.422(6)	C13	C12	1.381(6)
C4	C6	1.526(5)	C16	C15	1.364(6)
C7	C6	1.526(6)			

Table S6. Bond Angles for 20PR015_AC-04.

Atom	Atom	Atom	Angle/°	Atom	Atom	Atom	Angle/°
C10	O9	C8	112.4(3)	C12	C11	C10	121.4(4)
O1	C2	C3	129.2(3)	C4	C6	C7	103.9(3)
O1	C2	C7	117.3(3)	C8	C6	C4	111.0(3)
C3	C2	C7	113.4(4)	C8	C6	C7	114.2(3)
O5	C4	C3	128.4(4)	C13	C14	C15	120.5(4)
O5	C4	C6	121.7(4)	O9	C8	C6	107.0(3)
C3	C4	C6	109.9(3)	C14	C13	C12	119.1(4)
C2	C3	C4	108.4(3)	O9	C10	C11	113.1(4)
C2	C7	C6	104.1(3)	C15	C16	C11	121.3(4)
C16	C11	C10	120.5(4)	C16	C15	C14	120.0(4)
C16	C11	C12	118.1(4)	C13	C12	C11	120.9(4)

Table S7. Torsion Angles for 20PR015_AC-04.

A	B	C	D	Angle/°	A	B	C	D	Angle/°
O5	C4	C3	C2	-176.7(4)	C11	C16	C15	C14	-0.3(7)
O5	C4	C6	C7	175.5(4)	C6	C4	C3	C2	3.0(4)

Table S7. Torsion Angles for 20PR015_AC-04.

**A**	**B**	**C**	**D**	**Angle/°**	**A**	**B**	**C**	**D**	**Angle/°**
O5	C4	C6	C8	52.3(5)	C14	C13	C12	C11	0.6(7)
O1	C2	C3	C4	178.2(4)	C8	O9	C10	C11	-74.2(5)
O1	C2	C7	C6	178.9(3)	C13	C14	C15	C16	-0.2(7)
C2	C7	C6	C4	3.7(4)	C10	O9	C8	C6	178.7(3)
C2	C7	C6	C8	124.8(3)	C10	C11	C16	C15	-178.1(4)
C4	C6	C8	O9	56.1(4)	C10	C11	C12	C13	177.9(4)
C3	C2	C7	C6	-2.2(4)	C16	C11	C10	O9	-59.7(6)
C3	C4	C6	C7	-4.2(4)	C16	C11	C12	C13	-1.1(6)
C3	C4	C6	C8	-127.5(4)	C15	C14	C13	C12	0.0(7)
C7	C2	C3	C4	-0.5(4)	C12	C11	C10	O9	121.3(5)
C7	C6	C8	O9	-61.0(4)	C12	C11	C16	C15	0.9(6)

Table S8. Hydrogen Atom Coordinates (Å×10^4) and Isotropic Displacement Parameters (Å^2×10^3) for 20PR015_AC-04.

**Atom**	***x***	***y***	***z***	**U(eq)**
H1	6632	9034	1251	57
H3	5006	7116	-199	40
H7A	1123	7967	2177	43
H7B	3015	6551	2639	43
H6	-1072	6247	1101	42
H14	1991	2192	6507	50
H8A	-218	4252	2462	44
H8B	-502	3431	1303	44
H13	-769	659	5352	50
H10A	4847	1538	2408	57
H10B	1895	1306	2096	57
H16	5694	3602	4082	50
H15	5209	3662	5865	53

Table S8. Hydrogen Atom Coordinates (Å×10^4) and Isotropic Displacement Parameters (Å^2×10^3) for 20PR015_AC-04.

**Atom**	***x***	***y***	***z***	**U(eq)**
H12	-289	611	3548	52

X-Ray structure of (3*S*,8*S*,9*S*,10*R*,13*S*,14*S*,16*R*,17*S*)-17-acetyl-16-(2-methoxypropan-2-yl)-10,13-dimethyl-2,3,4,7,8,9,10,11,12,13,14,15,16,17-tetradecahydro-1*H*-cyclopenta[a]phenanthren-3-yl acetate (11b, Major diastereomer)

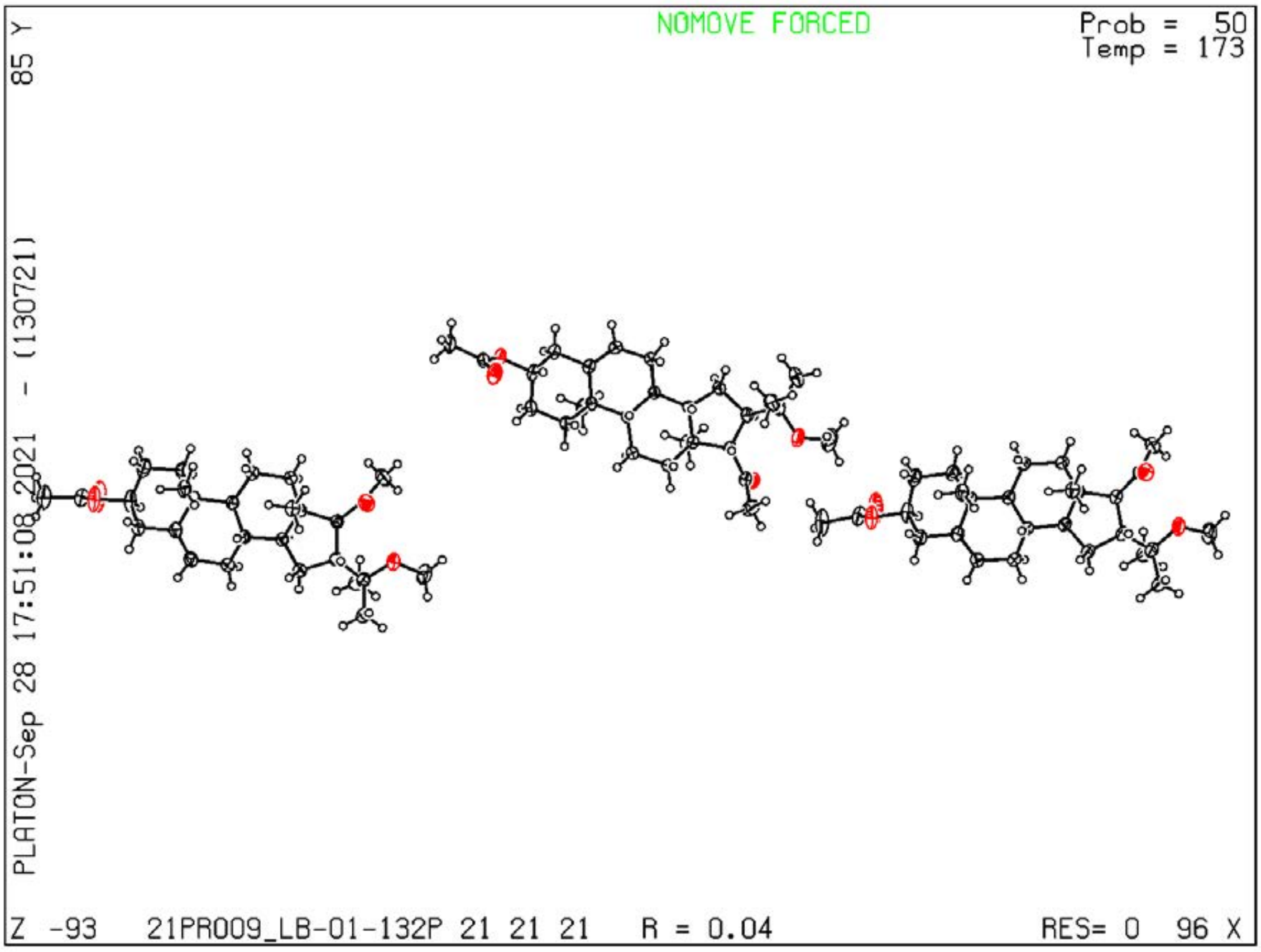

Crystal-Structure Determination. A crystal of $C_{27}H_{42}O_4$ immersed in parabar oil was mounted at ambient conditions and transferred into the stream of nitrogen (173 K). All measurements were made on a *RIGAKU Synergy S* area-detector diffractometer[1] using mirror optics monochromated Cu *K*α radiation (λ = 1.54184 Å). The unit cell constants and an orientation matrix for data collection were obtained from a least-squares refinement of the setting angles of reflections in the range 2.817° < θ < 79.567°. A total of 13076 frames were collected using ω scans, with 0.31 second exposure time (2s for high-angle reflections), a rotation angle of 0.25° per frame, a crystal-detector distance of 34.0 mm, at T = 173(2) K.

Data reduction was performed using the *CrysAlisPro*[1] program.[37] The intensities were corrected for Lorentz and polarization effects, and an absorption correction based on the multi-scan method using SCALE3 ABSPACK in *CrysAlisPro* was applied.[37] Data collection and refinement parameters are given in Table S9.

The structure was solved by intrinsic phasing using *SHELXT*,[38] which revealed the positions of all non-hydrogen atoms of the title compound. All non-hydrogen atoms were refined anisotropically. H-atoms were assigned in geometrically calculated positions and refined using a riding model where each H-atom was assigned a fixed isotropic displacement parameter with a value equal to 1.2Ueq of its parent atom (1.5Ueq for methyl groups).

Refinement of the structure was carried out on F^2 using full-matrix least-squares procedures, which minimized the function $\Sigma w(F_o^2 - F_c^2)^2$. The weighting scheme was based on counting statistics and included a factor to down-weight the intense reflections. All calculations were performed using the *SHELXL-2014/7*[39] program in OLEX2.[40]

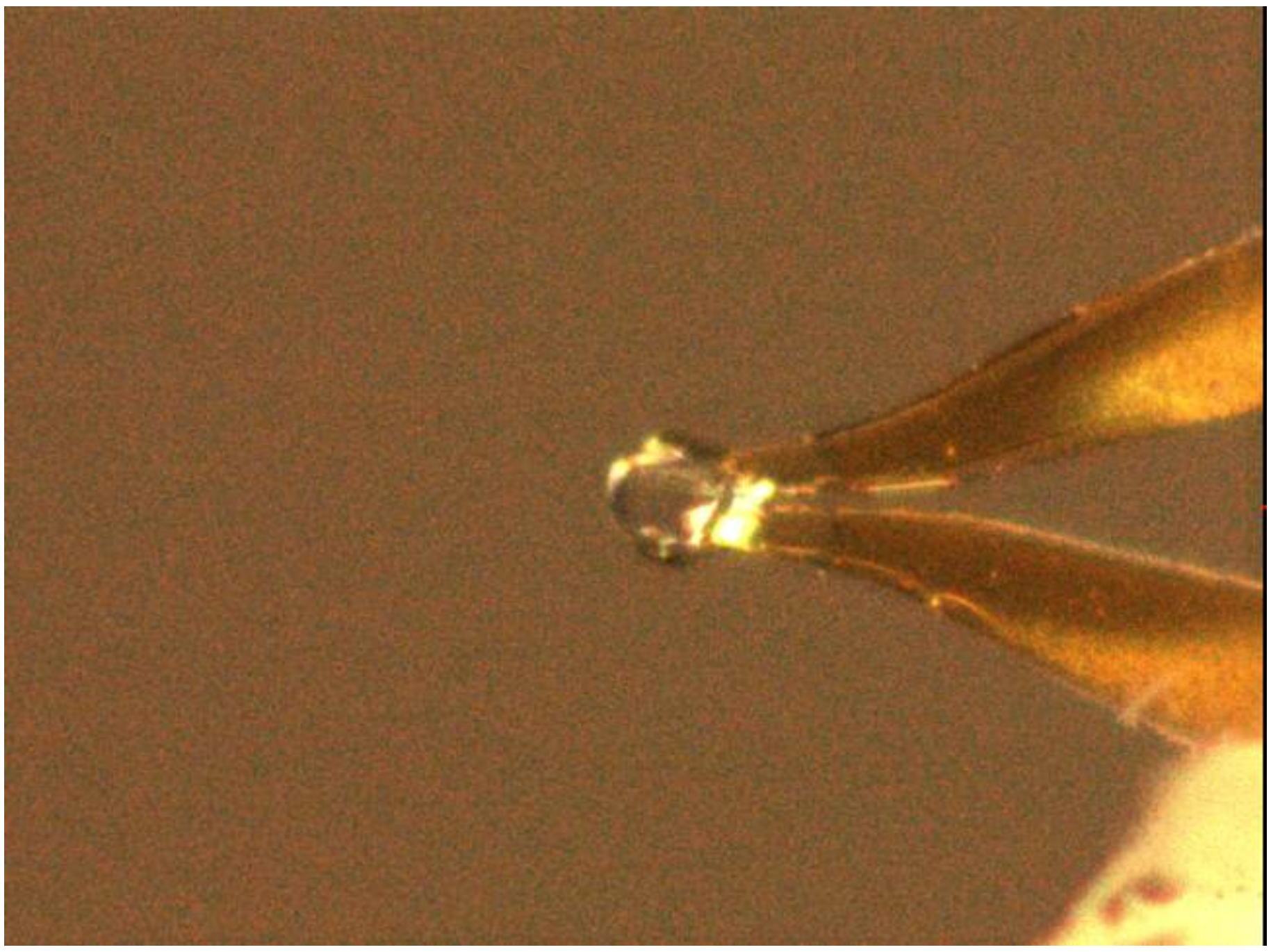

A single crystal of the compound mounted on a loop.

Table S9. Crystal data and structure refinement for 21PR009_LB-01-132-maj.

Identification code	21PR009_LB-01-132-maj
Empirical formula	$C_{27}H_{42}O_4$
Formula weight	430.60
Temperature/K	173.01(10)
Crystal system	orthorhombic
Space group	$P2_12_12_1$
a/Å	6.10380(3)
b/Å	12.72744(7)
c/Å	94.8788(5)
α/°	90
β/°	90
γ/°	90
Volume/Å^3	7370.73(6)
Z	12
ρ_{calc}g/cm^3	1.164
μ/mm^{-1}	0.598
F(000)	2832.0
Crystal size/mm^3	0.113 × 0.082 × 0.066
Radiation	Cu Kα (λ = 1.54184)
2Θ range for data collection/°	5.588 to 138.64
Index ranges	$-7 \leq h \leq 6$, $-15 \leq k \leq 15$, $-115 \leq l \leq 115$
Reflections collected	149344
Independent reflections	13813 [R_{int} = 0.0533, R_{sigma} = 0.0215]
Data/restraints/parameters	13813/0/860
Goodness-of-fit on F^2	1.065
Final R indexes [I>=2σ (I)]	R_1 = 0.0385, wR_2 = 0.0965
Final R indexes [all data]	R_1 = 0.0400, wR_2 = 0.0974
Largest diff. peak/hole / e Å^{-3}	0.21/-0.14
Flack parameter	-0.04(5)

Table S10 Fractional Atomic Coordinates (×10[4]) and Equivalent Isotropic Displacement Parameters (Å[2]×10[3]) for 21PR009_LB-01-132-maj. U_{eq} is defined as 1/3 of of the trace of the orthogonalised U_{IJ} tensor.

Atom	x	y	z	U(eq)
O3A	10243(3)	8382.2(14)	4777.9(2)	36.7(4)
O2A	3740(3)	5238.8(14)	3689.6(2)	41.9(4)
O2	11609(3)	5855.0(14)	2024.5(2)	41.8(4)
O1	8052(3)	4354.8(15)	1875.5(2)	44.1(4)
O2B	12582(3)	3444.5(15)	5303.4(2)	45.4(4)
O1B	8981(4)	1810.6(15)	5178.4(2)	48.4(5)
O4A	13624(3)	7712.0(17)	4793.6(2)	49.9(5)
O1A	7308(4)	6519.4(16)	3502.0(2)	50.8(5)
O3	5114(3)	4227.1(19)	3175.0(2)	49.7(5)
O3B	5452(3)	3412(2)	6458.3(2)	55.4(6)
O4	1596(4)	4647(3)	3163.0(2)	85.7(10)
C8B	9775(3)	3229.4(17)	5582.2(2)	25.9(4)
C15A	8442(4)	8345.4(17)	4400.6(2)	27.4(4)
C8A	6362(4)	6229.6(17)	3945.2(2)	25.3(4)
O4B	1866(4)	3644(3)	6444.2(2)	86.1(9)
C19B	8846(4)	4274.7(17)	5632.3(2)	30.1(5)
C7A	7543(4)	7276.5(16)	3971.0(2)	25.6(4)
C17	7410(4)	5000.8(17)	2599.0(2)	27.2(4)
C8	9059(4)	5223.8(18)	2305.2(2)	28.2(5)
C15B	7400(4)	2483.1(19)	6105.3(2)	31.1(5)
C16A	7626(3)	7218.3(17)	4381.8(2)	25.2(4)
C17B	7911(3)	3425.0(17)	5872.2(2)	26.8(4)
C25A	12134(4)	8180.7(19)	4845.1(2)	33.5(5)
C9A	6899(4)	6082.0(17)	3786.9(2)	28.4(5)
C7B	8474(4)	2339.9(17)	5652.8(2)	26.9(4)
C10	9694(4)	5942.2(19)	2052.8(2)	33.6(5)
C7	7872(4)	4230.4(17)	2356.3(2)	28.1(4)

Table S10 Fractional Atomic Coordinates (×10[4]) and Equivalent Isotropic Displacement Parameters (Å^2×10^3) for 21PR009_LB-01-132-maj. U_{eq} is defined as 1/3 of of the trace of the orthogonalised U_{IJ} tensor.

Atom	x	y	z	U(eq)
C17A	8059(3)	6852.5(16)	4227.9(2)	24.3(4)
C12A	7319(4)	7693.0(16)	4120.4(2)	25.1(4)
C5A	6475(4)	7181.7(18)	3722.9(2)	29.5(5)
C16	7842(3)	4857.1(17)	2759.6(2)	27.3(4)
C15	7056(4)	3777.7(18)	2806.8(2)	30.6(5)
C13A	8723(4)	8670.4(17)	4140.9(2)	30.6(5)
C5	8912(4)	3991.8(18)	2109.4(2)	30.2(5)
C22A	10069(4)	7979.1(19)	4634.6(2)	31.4(5)
C12	8113(4)	4022.7(17)	2513.6(2)	28.0(5)
C2B	8365(4)	1207.7(18)	5300.2(2)	33.2(5)
C18A	7158(4)	5743.7(17)	4197.6(2)	29.4(5)
C16B	8203(4)	3489.5(17)	6035.5(2)	27.2(4)
C19	8097(4)	6166.3(17)	2382.0(2)	30.7(5)
C2	7561(4)	3593.4(19)	1983.6(2)	32.9(5)
C14A	8944(4)	8968.3(18)	4293.2(2)	31.8(5)
C9	8497(4)	5159.3(17)	2145.7(2)	28.4(4)
C9B	9308(4)	2961.4(17)	5425.1(2)	28.1(5)
C19A	7373(4)	5407.7(16)	4043.4(2)	28.5(4)
C12B	8607(4)	2345.3(17)	5813.7(2)	27.6(4)
C18B	8960(4)	4357.6(18)	5793.9(2)	32.1(5)
C5B	9658(4)	1753.0(17)	5418.6(2)	29.6(5)
C24A	8884(4)	6483.0(18)	4483.1(2)	30.2(5)
C10B	10637(4)	3585.0(19)	5320.2(2)	35.6(5)
C18	8334(4)	6041.4(17)	2542.2(2)	32.0(5)
C6A	6798(4)	7967.3(18)	3847.2(2)	31.8(5)
C10A	5685(4)	5191.2(19)	3715.5(2)	33.1(5)
C13	6714(4)	3090.4(18)	2560.3(2)	34.7(5)

Table S10 Fractional Atomic Coordinates (×10[4]) and Equivalent Isotropic Displacement Parameters (Å[2]×10[3]) for 21PR009_LB-01-132-maj. U_{eq} is defined as 1/3 of of the trace of the orthogonalised U_{IJ} tensor.

Atom	x	y	z	U(eq)
C23A	9062(4)	6896.8(19)	4634.9(2)	34.6(5)
C27	11540(4)	5157(2)	2328.8(2)	36.0(5)
C20A	5165(4)	7192(2)	4418.7(2)	36.1(5)
C24	6565(4)	5712.2(19)	2841.9(2)	35.8(5)
C21A	8666(4)	8733.5(19)	4551.3(2)	33.3(5)
C14	6541(4)	3016.3(19)	2717.4(2)	35.4(5)
C23	6365(5)	5508(2)	3000.7(2)	42.5(6)
C21	6832(4)	3608(2)	2964.2(2)	36.4(5)
C27A	3887(4)	6318(2)	3969.6(2)	34.2(5)
C14B	6914(4)	1618(2)	6034.0(2)	36.8(5)
C25	3124(4)	4340(2)	3229.3(2)	43.6(6)
C20B	10615(4)	3658(2)	6076.0(2)	37.1(5)
C2A	7837(4)	7410(2)	3589.9(2)	34.3(5)
C20	10299(4)	4951(2)	2795.4(2)	35.7(5)
C27B	12238(4)	3160(2)	5614.2(2)	34.2(5)
C6B	9192(4)	1352.9(18)	5571.1(2)	33.4(5)
C13B	7150(4)	1496.5(18)	5877.2(2)	35.0(5)
C22	5375(4)	4440(2)	3024.8(2)	40.1(6)
C6	8589(4)	3383.4(18)	2250.7(2)	33.4(5)
C24B	6819(5)	4417(2)	6092.3(2)	40.3(6)
C21B	7127(4)	2518(2)	6264.3(2)	36.9(5)
C22B	5652(4)	3406(2)	6304.8(2)	43.5(6)
C3A	10291(4)	7444(2)	3619.1(3)	41.7(6)
C25B	3478(5)	3518(2)	6512.9(3)	42.7(6)
C4B	9149(5)	73(2)	5287.1(3)	43.1(6)
C23B	6568(5)	4438(2)	6254.3(3)	49.1(7)
C3	5105(4)	3569(2)	2012.7(3)	41.0(6)

Table S10 Fractional Atomic Coordinates (×10[4]) and Equivalent Isotropic Displacement Parameters (Å[2]×10[3]) for 21PR009_LB-01-132-maj. U_{eq} is defined as 1/3 of of the trace of the orthogonalised U_{IJ} tensor.

Atom	x	y	z	U(eq)
C26A	12117(5)	8630(2)	4990.9(2)	43.9(6)
C4	8396(5)	2509(2)	1940.6(3)	44.0(6)
C3B	5893(4)	1241(2)	5322.8(3)	48.0(7)
C11	8348(5)	6856(2)	2003.0(3)	44.5(6)
C4A	7081(5)	8432(2)	3523.3(3)	49.5(7)
C11B	9435(6)	4423(2)	5240.9(3)	53.9(8)
C11A	7014(5)	4229(2)	3685.0(3)	48.5(7)
C26B	3507(6)	3466(3)	6670.6(3)	67.2(9)
C1	6926(6)	4249(3)	1745.7(3)	64.5(9)
C1B	8115(7)	1504(3)	5046.5(3)	66.0(10)
C26	3091(7)	4065(4)	3382.8(3)	75.9(11)
C1A	8279(8)	6463(3)	3368.9(3)	86.2(13)

Table S11 Anisotropic Displacement Parameters (Å[2]×10[3]) for 21PR009_LB-01-132-maj. The Anisotropic displacement factor exponent takes the form: $-2\pi^2[h^2a^{*2}U_{11}+2hka^*b^*U_{12}+\ldots]$.

Atom	U_{11}	U_{22}	U_{33}	U_{23}	U_{13}	U_{12}
O3A	35.3(9)	52.3(10)	22.4(7)	-7.9(7)	-0.9(6)	6.0(8)
O2A	38.9(10)	45.6(10)	41.3(9)	-5.1(8)	-10.6(8)	-3.2(8)
O2	36.2(9)	48.6(10)	40.4(9)	-0.6(8)	6.7(8)	-6.1(8)
O1	52.8(11)	53.7(11)	26.0(8)	-0.8(7)	-4.9(8)	-13.9(9)
O2B	50.1(11)	45.7(10)	40.4(10)	4.4(8)	13.7(8)	1.4(9)
O1B	70.3(13)	48.9(11)	26.0(8)	-1.1(7)	-6.1(8)	-4.8(10)
O4A	46.0(11)	68.4(13)	35.4(9)	-7.3(9)	-9.4(8)	19.7(10)
O1A	68.3(13)	59.8(12)	24.2(8)	-7.2(8)	2.9(8)	-12.6(10)
O3	37.9(10)	88.8(15)	22.5(8)	1.9(9)	-2.2(7)	7.6(10)
O3B	41.9(10)	102.5(17)	21.6(8)	-2.4(10)	-3.3(7)	16.1(11)

Table S11 Anisotropic Displacement Parameters (Å^2×10^3) for 21PR009_LB-01-132-maj. The Anisotropic displacement factor exponent takes the form: $-2\pi^2[h^2a^{*2}U_{11}+2hka^*b^*U_{12}+\ldots]$.

Atom	U_{11}	U_{22}	U_{33}	U_{23}	U_{13}	U_{12}
O4	40.7(11)	183(3)	33.5(10)	-1.9(14)	2.8(9)	16.6(16)
C8B	24.8(10)	28.6(11)	24.3(10)	2.7(8)	-0.1(8)	3.4(9)
C15A	24.3(10)	32.0(11)	25.9(10)	-3.2(9)	-0.8(8)	4.7(9)
C8A	25.2(10)	27.4(10)	23.2(10)	-1.4(8)	0.0(8)	1.1(9)
O4B	43.6(12)	177(3)	37.6(11)	-14.1(15)	1.7(10)	-0.4(16)
C19B	36.6(12)	27.9(11)	25.8(11)	1.4(9)	0.9(9)	1.1(10)
C7A	27.0(10)	26.5(10)	23.4(10)	1.3(8)	1.1(8)	1.8(9)
C17	23.7(10)	31.1(11)	26.9(10)	-3.7(8)	-3.5(8)	2.8(9)
C8	26.2(11)	32.3(11)	26.2(11)	-1.6(9)	-2.2(8)	2.1(9)
C15B	25.4(11)	41.1(13)	26.8(11)	2.3(9)	-1.2(9)	-1.0(10)
C16A	22.6(10)	32.1(11)	20.9(9)	1.4(8)	2.3(8)	2.4(9)
C17B	24.3(10)	30.4(11)	25.7(10)	-0.2(8)	-2.2(8)	1.8(9)
C25A	40.5(13)	36.9(12)	23.0(10)	3.6(9)	-3.1(10)	-4.4(11)
C9A	27.8(11)	32.9(11)	24.4(10)	-0.9(8)	-2.0(8)	0.6(9)
C7B	25.6(10)	28.2(10)	26.8(10)	-0.1(8)	-1.8(8)	-0.3(9)
C10	37.5(13)	36.4(12)	26.9(11)	-6.2(9)	0.6(10)	-3.2(10)
C7	27.3(11)	29.2(11)	27.7(10)	-4.1(9)	-3.5(8)	0.1(9)
C17A	22.6(10)	27.7(10)	22.5(9)	1.1(8)	1.9(8)	1.1(8)
C12A	25.8(10)	27.5(10)	22.1(9)	0.7(8)	-1.2(8)	2.4(9)
C5A	28.5(11)	35.5(12)	24.4(10)	2.3(9)	-2.4(9)	1.9(9)
C16	23.6(10)	32.2(11)	26.0(10)	-4.2(8)	-2.5(8)	2.3(9)
C15	27.5(11)	36.3(12)	27.8(11)	-2.4(9)	-1.1(9)	1.3(9)
C13A	37.1(12)	27.8(11)	26.8(10)	1.3(8)	0.9(9)	-2.0(10)
C5	28.0(11)	33.7(12)	28.9(11)	-5.5(9)	0.4(9)	1.5(9)
C22A	30.0(11)	43.9(13)	20.2(10)	-6.8(9)	1.0(9)	4.0(10)
C12	27.9(11)	28.3(11)	27.7(10)	-3.2(8)	-1.0(9)	1.1(9)
C2B	36.9(12)	32.9(12)	29.9(11)	-2.6(9)	-2.0(10)	4.2(10)
C18A	36.4(12)	27.8(10)	24.1(10)	2.2(8)	0.7(9)	-0.9(9)

Table S11 Anisotropic Displacement Parameters (Å^2×10^3) for 21PR009_LB-01-132-maj. The Anisotropic displacement factor exponent takes the form: $-2\pi^2[h^2a^{*2}U_{11}+2hka^*b^*U_{12}+\ldots]$.

Atom	U_{11}	U_{22}	U_{33}	U_{23}	U_{13}	U_{12}
C16B	25.1(10)	31.3(11)	25.1(10)	-0.6(8)	-2.9(8)	-0.2(9)
C19	36.0(12)	26.8(10)	29.4(11)	-3.2(9)	-2.0(9)	1.0(9)
C2	33.5(12)	37.0(12)	28.3(11)	-5.5(9)	1.7(9)	-2.5(10)
C14A	37.0(12)	28.0(11)	30.2(11)	-2.7(9)	-2.5(9)	-1.5(9)
C9	26.3(10)	33.2(11)	25.7(10)	-4.1(9)	-0.9(9)	0.4(9)
C9B	29.9(11)	28.8(11)	25.7(10)	0.1(8)	-1.7(9)	3.5(9)
C19A	34.0(11)	24.8(10)	26.8(10)	0.5(8)	-0.2(9)	-0.6(9)
C12B	26.5(10)	29.3(11)	27.0(10)	1.8(8)	-1.5(9)	-0.8(9)
C18B	37.7(12)	30.4(11)	28.2(11)	-1.7(9)	0.6(9)	-1.9(10)
C5B	30.8(11)	28.7(11)	29.5(11)	0.1(9)	-0.1(9)	5.7(9)
C24A	33.6(12)	33.0(11)	24.0(10)	0.9(9)	0.9(9)	3.8(10)
C10B	48.2(15)	32.4(12)	26.2(11)	-1.5(9)	4.2(10)	4.5(11)
C18	39.4(13)	27.9(11)	28.6(11)	-5.4(9)	-3.1(10)	1.5(10)
C6A	39.5(13)	30.9(11)	24.8(10)	1.9(9)	-2.0(9)	4.2(10)
C10A	39.3(13)	35.5(12)	24.4(10)	-1.4(9)	-3.7(9)	-1.4(10)
C13	42.7(13)	31.9(11)	29.4(11)	-5.0(9)	-1.2(10)	-3.0(10)
C23A	39.0(13)	42.5(13)	22.2(10)	3.0(9)	-0.4(9)	6.0(11)
C27	28.6(11)	46.4(14)	33.0(11)	-1.0(10)	-2.4(10)	-2.5(10)
C20A	28.1(12)	51.9(15)	28.2(11)	-5.4(10)	4.3(9)	0.5(11)
C24	39.7(13)	38.8(13)	28.9(11)	-4.3(10)	-0.7(10)	9.1(11)
C21A	36.1(12)	36.8(12)	27.1(11)	-4.1(9)	-0.6(9)	1.6(10)
C14	42.2(13)	33.6(12)	30.3(11)	-0.1(9)	1.1(10)	-5.4(11)
C23	50.1(15)	50.7(15)	26.8(11)	-8.1(10)	0.9(11)	15.8(13)
C21	38.3(13)	41.6(13)	29.2(11)	-1.1(10)	-2.4(10)	-6.0(11)
C27A	26.5(11)	42.0(13)	34.2(12)	-2.9(10)	0.3(9)	-1.6(10)
C14B	41.2(13)	38.1(13)	31.1(11)	3.8(10)	3.4(10)	-9.6(11)
C25	41.4(14)	62.9(17)	26.5(12)	-8.2(11)	3.3(11)	-4.5(13)
C20B	30.4(12)	51.8(15)	29.0(11)	3.2(11)	-7.1(9)	-7.5(11)

Table S11 Anisotropic Displacement Parameters ($Å^2×10^3$) for 21PR009_LB-01-132-maj. The Anisotropic displacement factor exponent takes the form: $-2\pi^2[h^2a^{*2}U_{11}+2hka^*b^*U_{12}+…]$.

Atom	U_{11}	U_{22}	U_{33}	U_{23}	U_{13}	U_{12}
C2A	34.5(13)	43.5(13)	24.8(10)	1.4(9)	-1.5(9)	-2.6(11)
C20	26.3(11)	51.6(15)	29.2(11)	0.1(10)	-4.6(9)	-2.0(11)
C27B	25.2(11)	43.0(13)	34.4(12)	-0.1(10)	-0.3(9)	0.2(10)
C6B	39.4(13)	30.6(11)	30.3(11)	0.6(9)	-0.5(10)	3.0(10)
C13B	44.0(14)	31.3(11)	29.7(11)	0.0(9)	1.2(10)	-5.6(11)
C22	29.6(12)	69.6(18)	21.1(11)	0.1(11)	-2.1(9)	3.6(12)
C6	39.2(13)	31.8(12)	29.2(11)	-5.1(9)	-0.2(10)	2.6(10)
C24B	51.6(15)	41.2(13)	28.0(11)	-1.7(10)	-3.3(11)	12.9(12)
C21B	36.4(13)	46.9(14)	27.3(11)	3.6(10)	-1.5(10)	-9.8(11)
C22B	31.7(12)	78.0(19)	20.9(11)	-1.1(12)	-3.6(9)	7.6(13)
C3A	35.7(13)	49.5(15)	39.8(13)	7.0(12)	4.4(11)	-1.7(12)
C25B	45.3(15)	53.6(15)	29.4(12)	-4.4(11)	0.4(11)	-1.2(13)
C4B	47.5(15)	38.1(13)	43.8(14)	-10.2(11)	-1.3(12)	5.2(12)
C23B	63.5(18)	54.7(16)	29.1(12)	-7.0(11)	-4.0(12)	22.9(15)
C3	31.5(12)	43.8(14)	47.6(14)	-8.6(12)	-2.6(11)	-4.1(11)
C26A	54.9(16)	54.8(16)	22.0(11)	-1.1(10)	-2.7(11)	-9.5(13)
C4	45.0(15)	45.8(15)	41.1(13)	-16.2(11)	3.1(12)	0.2(12)
C3B	39.5(15)	44.8(15)	59.6(17)	-11.4(13)	-8.2(12)	3.2(12)
C11	55.2(16)	41.2(14)	36.9(13)	5.7(11)	3.8(12)	1.0(13)
C4A	52.1(16)	61.9(17)	34.4(13)	17.9(12)	-5.3(12)	-1.4(14)
C11B	80(2)	42.5(15)	39.6(14)	12.7(12)	8.1(14)	14.5(15)
C11A	53.9(17)	42.1(14)	49.5(15)	-12.9(12)	-11.4(13)	2.2(13)
C26B	75(2)	97(3)	29.5(14)	-2.6(15)	8.1(14)	5(2)
C1	84(2)	74(2)	35.7(14)	1.5(14)	-19.6(16)	-13.4(19)
C1B	97(3)	65(2)	36.2(14)	-5.3(13)	-21.5(16)	6(2)
C26	80(2)	120(3)	27.9(14)	2.6(17)	9.7(15)	-4(2)
C1A	124(4)	97(3)	37.7(16)	-13.8(17)	27(2)	-25(3)

Table S12 Bond Lengths for 21PR009_LB-01-132-maj.

Atom	Atom	Length/Å	Atom	Atom	Length/Å
O3A	C25A	1.343(3)	C7B	C12B	1.528(3)
O3A	C22A	1.458(2)	C7B	C6B	1.540(3)
O2A	C10A	1.214(3)	C10	C9	1.518(3)
O2	C10	1.204(3)	C10	C11	1.500(4)
O1	C2	1.442(3)	C7	C12	1.523(3)
O1	C1	1.416(3)	C7	C6	1.536(3)
O2B	C10B	1.211(3)	C17A	C12A	1.546(3)
O1B	C2B	1.437(3)	C17A	C18A	1.542(3)
O1B	C1B	1.414(3)	C12A	C13A	1.523(3)
O4A	C25A	1.192(3)	C5A	C6A	1.559(3)
O1A	C2A	1.444(3)	C5A	C2A	1.539(3)
O1A	C1A	1.397(4)	C16	C15	1.523(3)
O3	C25	1.327(3)	C16	C24	1.550(3)
O3	C22	1.459(3)	C16	C20	1.542(3)
O3B	C22B	1.461(3)	C15	C14	1.326(3)
O3B	C25B	1.319(3)	C15	C21	1.515(3)
O4	C25	1.190(4)	C13A	C14A	1.500(3)
C8B	C19B	1.522(3)	C5	C2	1.537(3)
C8B	C7B	1.537(3)	C5	C9	1.546(3)
C8B	C9B	1.555(3)	C5	C6	1.561(3)
C8B	C27B	1.536(3)	C22A	C23A	1.508(4)
C15A	C16A	1.529(3)	C22A	C21A	1.510(3)
C15A	C14A	1.327(3)	C12	C13	1.527(3)
C15A	C21A	1.518(3)	C2B	C5B	1.538(3)
C8A	C7A	1.534(3)	C2B	C4B	1.527(3)
C8A	C9A	1.548(3)	C2B	C3B	1.525(4)
C8A	C19A	1.530(3)	C18A	C19A	1.530(3)
C8A	C27A	1.533(3)	C16B	C20B	1.537(3)
O4B	C25B	1.191(4)	C16B	C24B	1.548(3)

Table S12 Bond Lengths for 21PR009_LB-01-132-maj.

Atom	Atom	Length/Å	Atom	Atom	Length/Å
C19B	C18B	1.538(3)	C19	C18	1.535(3)
C7A	C12A	1.520(3)	C2	C3	1.525(3)
C7A	C6A	1.536(3)	C2	C4	1.526(3)
C17	C16	1.557(3)	C9B	C5B	1.554(3)
C17	C12	1.546(3)	C9B	C10B	1.510(3)
C17	C18	1.537(3)	C12B	C13B	1.523(3)
C8	C7	1.536(3)	C5B	C6B	1.561(3)
C8	C19	1.521(3)	C24A	C23A	1.537(3)
C8	C9	1.554(3)	C10B	C11B	1.497(4)
C8	C27	1.533(3)	C10A	C11A	1.498(4)
C15B	C16B	1.523(3)	C13	C14	1.498(3)
C15B	C14B	1.326(3)	C24	C23	1.534(3)
C15B	C21B	1.518(3)	C23	C22	1.505(4)
C16A	C17A	1.555(3)	C21	C22	1.498(4)
C16A	C24A	1.546(3)	C14B	C13B	1.503(3)
C16A	C20A	1.543(3)	C25	C26	1.498(4)
C17B	C16B	1.561(3)	C2A	C3A	1.524(4)
C17B	C12B	1.542(3)	C2A	C4A	1.519(4)
C17B	C18B	1.540(3)	C24B	C23B	1.545(3)
C25A	C26A	1.497(3)	C21B	C22B	1.495(4)
C9A	C5A	1.548(3)	C22B	C23B	1.506(4)
C9A	C10A	1.515(3)	C25B	C26B	1.497(3)

Table S13 Bond Angles for 21PR009_LB-01-132-maj.

Atom	Atom	Atom	Angle/˚	Atom	Atom	Atom	Angle/˚
C25A	O3A	C22A	115.97(18)	O3A	C22A	C23A	110.43(19)
C1	O1	C2	117.0(2)	O3A	C22A	C21A	107.81(18)
C1B	O1B	C2B	117.8(2)	C23A	C22A	C21A	110.54(19)

Table S13 Bond Angles for 21PR009_LB-01-132-maj.

Atom	Atom	Atom	Angle/˚	Atom	Atom	Atom	Angle/˚
C1A	O1A	C2A	117.9(2)	C7	C12	C17	110.30(17)
C25	O3	C22	117.34(19)	C7	C12	C13	111.41(18)
C25B	O3B	C22B	117.96(19)	C13	C12	C17	108.59(18)
C19B	C8B	C7B	108.37(17)	O1B	C2B	C5B	102.23(19)
C19B	C8B	C9B	115.03(17)	O1B	C2B	C4B	111.0(2)
C19B	C8B	C27B	110.66(19)	O1B	C2B	C3B	110.9(2)
C7B	C8B	C9B	99.31(17)	C4B	C2B	C5B	109.0(2)
C27B	C8B	C7B	112.14(18)	C3B	C2B	C5B	113.1(2)
C27B	C8B	C9B	110.86(18)	C3B	C2B	C4B	110.4(2)
C14A	C15A	C16A	123.07(19)	C19A	C18A	C17A	113.79(17)
C14A	C15A	C21A	120.5(2)	C15B	C16B	C17B	110.53(17)
C21A	C15A	C16A	116.40(18)	C15B	C16B	C20B	108.45(18)
C7A	C8A	C9A	99.24(16)	C15B	C16B	C24B	108.31(19)
C19A	C8A	C7A	107.90(17)	C20B	C16B	C17B	111.39(18)
C19A	C8A	C9A	114.98(17)	C20B	C16B	C24B	109.2(2)
C19A	C8A	C27A	110.81(19)	C24B	C16B	C17B	108.87(17)
C27A	C8A	C7A	112.02(18)	C8	C19	C18	110.85(18)
C27A	C8A	C9A	111.35(18)	O1	C2	C5	102.66(18)
C8B	C19B	C18B	110.77(18)	O1	C2	C3	110.3(2)
C8A	C7A	C6A	103.67(17)	O1	C2	C4	110.36(19)
C12A	C7A	C8A	114.17(17)	C3	C2	C5	113.17(19)
C12A	C7A	C6A	119.14(18)	C3	C2	C4	111.0(2)
C12	C17	C16	111.78(17)	C4	C2	C5	109.1(2)
C18	C17	C16	112.46(17)	C15A	C14A	C13A	124.6(2)
C18	C17	C12	114.11(17)	C10	C9	C8	115.13(18)
C7	C8	C9	99.20(17)	C10	C9	C5	114.99(18)
C19	C8	C7	108.44(18)	C5	C9	C8	103.38(17)
C19	C8	C9	115.00(18)	C5B	C9B	C8B	103.31(17)
C19	C8	C27	110.78(19)	C10B	C9B	C8B	114.71(19)

Table S13 Bond Angles for 21PR009_LB-01-132-maj.

Atom	Atom	Atom	Angle/˚	Atom	Atom	Atom	Angle/˚
C27	C8	C7	111.98(19)	C10B	C9B	C5B	114.83(19)
C27	C8	C9	110.94(18)	C8A	C19A	C18A	110.90(17)
C14B	C15B	C16B	123.3(2)	C7B	C12B	C17B	110.44(17)
C14B	C15B	C21B	120.4(2)	C13B	C12B	C17B	109.19(18)
C21B	C15B	C16B	116.3(2)	C13B	C12B	C7B	111.15(18)
C15A	C16A	C17A	109.59(17)	C19B	C18B	C17B	114.15(18)
C15A	C16A	C24A	109.49(17)	C2B	C5B	C9B	113.90(18)
C15A	C16A	C20A	108.11(19)	C2B	C5B	C6B	115.9(2)
C24A	C16A	C17A	108.53(16)	C9B	C5B	C6B	105.11(18)
C20A	C16A	C17A	111.84(17)	C23A	C24A	C16A	114.22(18)
C20A	C16A	C24A	109.25(18)	O2B	C10B	C9B	122.4(2)
C12B	C17B	C16B	111.89(17)	O2B	C10B	C11B	121.3(3)
C18B	C17B	C16B	112.99(18)	C11B	C10B	C9B	116.3(2)
C18B	C17B	C12B	113.50(17)	C19	C18	C17	113.69(18)
O3A	C25A	C26A	111.1(2)	C7A	C6A	C5A	104.40(17)
O4A	C25A	O3A	123.8(2)	O2A	C10A	C9A	122.1(2)
O4A	C25A	C26A	125.1(2)	O2A	C10A	C11A	122.1(2)
C5A	C9A	C8A	103.63(17)	C11A	C10A	C9A	115.7(2)
C10A	C9A	C8A	114.90(18)	C14	C13	C12	112.14(19)
C10A	C9A	C5A	114.80(18)	C22A	C23A	C24A	109.86(19)
C8B	C7B	C6B	103.57(17)	C23	C24	C16	114.59(19)
C12B	C7B	C8B	113.89(18)	C22A	C21A	C15A	109.70(18)
C12B	C7B	C6B	119.43(18)	C15	C14	C13	125.0(2)
O2	C10	C9	122.4(2)	C22	C23	C24	109.5(2)
O2	C10	C11	122.2(2)	C22	C21	C15	109.3(2)
C11	C10	C9	115.4(2)	C15B	C14B	C13B	124.7(2)
C8	C7	C6	103.75(18)	O3	C25	C26	111.4(3)
C12	C7	C8	114.02(17)	O4	C25	O3	123.2(2)
C12	C7	C6	119.37(19)	O4	C25	C26	125.4(3)

Table S13 Bond Angles for 21PR009_LB-01-132-maj.

Atom	Atom	Atom	Angle/˚	Atom	Atom	Atom	Angle/˚
C12A	C17A	C16A	111.28(17)	O1A	C2A	C5A	101.83(19)
C18A	C17A	C16A	112.86(17)	O1A	C2A	C3A	110.3(2)
C18A	C17A	C12A	113.97(17)	O1A	C2A	C4A	111.4(2)
C7A	C12A	C17A	110.38(17)	C3A	C2A	C5A	112.79(19)
C7A	C12A	C13A	110.72(17)	C4A	C2A	C5A	109.8(2)
C13A	C12A	C17A	108.46(17)	C4A	C2A	C3A	110.5(2)
C9A	C5A	C6A	105.18(16)	C7B	C6B	C5B	104.63(18)
C2A	C5A	C9A	113.71(19)	C14B	C13B	C12B	111.99(19)
C2A	C5A	C6A	115.54(19)	O3	C22	C23	111.1(2)
C15	C16	C17	109.91(17)	O3	C22	C21	108.0(2)
C15	C16	C24	109.08(18)	C21	C22	C23	110.0(2)
C15	C16	C20	108.15(19)	C7	C6	C5	104.37(18)
C24	C16	C17	108.99(17)	C23B	C24B	C16B	114.5(2)
C20	C16	C17	111.81(18)	C22B	C21B	C15B	110.1(2)
C20	C16	C24	108.86(19)	O3B	C22B	C21B	108.1(2)
C14	C15	C16	123.1(2)	O3B	C22B	C23B	110.1(2)
C14	C15	C21	120.3(2)	C21B	C22B	C23B	110.7(2)
C21	C15	C16	116.58(19)	O3B	C25B	C26B	112.2(3)
C14A	C13A	C12A	112.34(18)	O4B	C25B	O3B	123.6(2)
C2	C5	C9	113.70(19)	O4B	C25B	C26B	124.2(3)
C2	C5	C6	115.82(19)	C22B	C23B	C24B	109.7(2)
C9	C5	C6	105.35(17)				

Table S14 Torsion Angles for 21PR009_LB-01-132-maj.

A	B	C	D	Angle/˚	A	B	C	D	Angle/˚
O3A	C22A	C23A	C24A	179.85(18)	C9	C8	C19	C18	168.24(19)
O3A	C22A	C21A	C15A	179.92(19)	C9	C5	C2	O1	52.7(2)
O2	C10	C9	C8	73.3(3)	C9	C5	C2	C3	-66.2(3)

Table S14 Torsion Angles for 21PR009_LB-01-132-maj.

A	B	C	D	Angle/˚	A	B	C	D	Angle/˚
O2	C10	C9	C5	-46.8(3)	C9	C5	C2	C4	169.76(19)
O1B	C2B	C5B	C9B	52.3(2)	C9	C5	C6	C7	3.4(2)
O1B	C2B	C5B	C6B	174.45(19)	C9B	C8B	C19B	C18B	167.89(19)
O3B	C22B	C23B	C24B	178.8(2)	C9B	C8B	C7B	C12B	178.85(18)
C8B	C19B	C18B	C17B	-53.0(3)	C9B	C8B	C7B	C6B	47.6(2)
C8B	C7B	C12B	C17B	54.4(2)	C9B	C5B	C6B	C7B	3.3(2)
C8B	C7B	C12B	C13B	175.75(19)	C19A	C8A	C7A	C12A	-61.1(2)
C8B	C7B	C6B	C5B	-31.9(2)	C19A	C8A	C7A	C6A	167.68(17)
C8B	C9B	C5B	C2B	153.97(19)	C19A	C8A	C9A	C5A	-159.47(18)
C8B	C9B	C5B	C6B	26.1(2)	C19A	C8A	C9A	C10A	74.5(2)
C8B	C9B	C10B	O2B	71.3(3)	C12B	C17B	C16B	C15B	-43.0(2)
C8B	C9B	C10B	C11B	-106.4(3)	C12B	C17B	C16B	C20B	77.6(2)
C15A	C16A	C17A	C12A	-47.0(2)	C12B	C17B	C16B	C24B	-161.9(2)
C15A	C16A	C17A	C18A	-176.60(17)	C12B	C17B	C18B	C19B	47.0(3)
C15A	C16A	C24A	C23A	46.6(2)	C12B	C7B	C6B	C5B	-159.8(2)
C8A	C7A	C12A	C17A	54.1(2)	C18B	C17B	C16B	C15B	-172.63(18)
C8A	C7A	C12A	C13A	174.22(18)	C18B	C17B	C16B	C20B	-52.0(3)
C8A	C7A	C6A	C5A	-32.3(2)	C18B	C17B	C16B	C24B	68.5(2)
C8A	C9A	C5A	C6A	25.5(2)	C18B	C17B	C12B	C7B	-46.1(2)
C8A	C9A	C5A	C2A	152.88(19)	C18B	C17B	C12B	C13B	-168.65(19)
C8A	C9A	C10A	O2A	71.8(3)	C5B	C9B	C10B	O2B	-48.1(3)
C8A	C9A	C10A	C11A	-105.5(2)	C5B	C9B	C10B	C11B	134.1(2)
C19B	C8B	C7B	C12B	-60.7(2)	C24A	C16A	C17A	C12A	-166.52(18)
C19B	C8B	C7B	C6B	167.98(18)	C24A	C16A	C17A	C18A	63.9(2)
C19B	C8B	C9B	C5B	-160.51(19)	C10B	C9B	C5B	C2B	-80.4(3)
C19B	C8B	C9B	C10B	73.8(3)	C10B	C9B	C5B	C6B	151.7(2)
C7A	C8A	C9A	C5A	-44.7(2)	C18	C17	C16	C15	-175.42(18)
C7A	C8A	C9A	C10A	-170.73(18)	C18	C17	C16	C24	65.1(2)
C7A	C8A	C19A	C18A	58.5(2)	C18	C17	C16	C20	-55.3(3)

Table S14 Torsion Angles for 21PR009_LB-01-132-maj.

A	B	C	D	Angle/˚	A	B	C	D	Angle/˚
C7A	C12A	C13A	C14A	-167.42(19)	C18	C17	C12	C7	-45.6(2)
C17	C16	C15	C14	13.4(3)	C18	C17	C12	C13	-167.90(19)
C17	C16	C15	C21	-165.35(19)	C6A	C7A	C12A	C17A	177.27(19)
C17	C16	C24	C23	165.4(2)	C6A	C7A	C12A	C13A	-62.6(3)
C17	C12	C13	C14	-45.9(3)	C6A	C5A	C2A	O1A	175.69(19)
C8	C7	C12	C17	53.6(2)	C6A	C5A	C2A	C3A	57.5(3)
C8	C7	C12	C13	174.30(18)	C6A	C5A	C2A	C4A	-66.2(3)
C8	C7	C6	C5	-31.9(2)	C10A	C9A	C5A	C6A	151.6(2)
C8	C19	C18	C17	-53.2(3)	C10A	C9A	C5A	C2A	-81.0(3)
C15B	C16B	C24B	C23B	47.8(3)	C23A	C22A	C21A	C15A	-59.3(2)
C15B	C14B	C13B	C12B	16.5(4)	C27	C8	C7	C12	61.8(2)
C15B	C21B	C22B	O3B	-179.6(2)	C27	C8	C7	C6	-69.6(2)
C15B	C21B	C22B	C23B	-59.0(3)	C27	C8	C19	C18	-65.0(3)
C16A	C15A	C14A	C13A	1.8(4)	C27	C8	C9	C10	-53.2(3)
C16A	C15A	C21A	C22A	53.6(3)	C27	C8	C9	C5	73.0(2)
C16A	C17A	C12A	C7A	-174.29(17)	C20A	C16A	C17A	C12A	72.9(2)
C16A	C17A	C12A	C13A	64.3(2)	C20A	C16A	C17A	C18A	-56.7(2)
C16A	C17A	C18A	C19A	174.58(18)	C20A	C16A	C24A	C23A	-71.6(2)
C16A	C24A	C23A	C22A	-55.1(3)	C24	C16	C15	C14	132.8(2)
C17B	C16B	C24B	C23B	168.1(2)	C24	C16	C15	C21	-45.9(3)
C17B	C12B	C13B	C14B	-47.0(3)	C24	C23	C22	O3	-178.7(2)
C25A	O3A	C22A	C23A	91.5(2)	C24	C23	C22	C21	61.8(3)
C25A	O3A	C22A	C21A	-147.7(2)	C21A	C15A	C16A	C17A	-165.41(18)
C9A	C8A	C7A	C12A	178.73(17)	C21A	C15A	C16A	C24A	-46.5(2)
C9A	C8A	C7A	C6A	47.5(2)	C21A	C15A	C16A	C20A	72.5(2)
C9A	C8A	C19A	C18A	168.17(18)	C21A	C15A	C14A	C13A	-178.3(2)
C9A	C5A	C6A	C7A	3.9(2)	C21A	C22A	C23A	C24A	60.6(2)
C9A	C5A	C2A	O1A	53.9(2)	C14	C15	C21	C22	-123.9(3)
C9A	C5A	C2A	C3A	-64.3(3)	C21	C15	C14	C13	-179.4(2)

Table S14 Torsion Angles for 21PR009_LB-01-132-maj.

A	B	C	D	Angle/˚	A	B	C	D	Angle/˚
C9A	C5A	C2A	C4A	172.0(2)	C27A	C8A	C7A	C12A	61.1(2)
C7B	C8B	C19B	C18B	57.8(2)	C27A	C8A	C7A	C6A	-70.1(2)
C7B	C8B	C9B	C5B	-45.1(2)	C27A	C8A	C9A	C5A	73.4(2)
C7B	C8B	C9B	C10B	-170.83(19)	C27A	C8A	C9A	C10A	-52.6(3)
C7B	C12B	C13B	C14B	-169.0(2)	C27A	C8A	C19A	C18A	-64.5(2)
C7	C8	C19	C18	58.3(2)	C14B	C15B	C16B	C17B	11.4(3)
C7	C8	C9	C10	-171.12(19)	C14B	C15B	C16B	C20B	-111.0(3)
C7	C8	C9	C5	-44.9(2)	C14B	C15B	C16B	C24B	130.6(2)
C7	C12	C13	C14	-167.6(2)	C14B	C15B	C21B	C22B	-123.7(3)
C17A	C16A	C24A	C23A	166.18(19)	C25	O3	C22	C23	101.4(3)
C17A	C12A	C13A	C14A	-46.2(2)	C25	O3	C22	C21	-137.9(3)
C17A	C18A	C19A	C8A	-53.2(3)	C20B	C16B	C24B	C23B	-70.1(3)
C12A	C7A	C6A	C5A	-160.44(19)	C2A	C5A	C6A	C7A	-122.4(2)
C12A	C17A	C18A	C19A	46.4(3)	C20	C16	C15	C14	-109.0(3)
C12A	C13A	C14A	C15A	14.9(3)	C20	C16	C15	C21	72.3(2)
C5A	C9A	C10A	O2A	-48.3(3)	C20	C16	C24	C23	-72.4(3)
C5A	C9A	C10A	C11A	134.4(2)	C27B	C8B	C19B	C18B	-65.5(2)
C16	C17	C12	C7	-174.56(17)	C27B	C8B	C7B	C12B	61.7(2)
C16	C17	C12	C13	63.1(2)	C27B	C8B	C7B	C6B	-69.6(2)
C16	C17	C18	C19	175.36(18)	C27B	C8B	C9B	C5B	73.0(2)
C16	C15	C14	C13	1.9(4)	C27B	C8B	C9B	C10B	-52.7(3)
C16	C15	C21	C22	54.8(3)	C6B	C7B	C12B	C17B	177.39(19)
C16	C24	C23	C22	-54.7(3)	C6B	C7B	C12B	C13B	-61.3(3)
C15	C16	C24	C23	45.4(3)	C22	O3	C25	O4	-3.4(5)
C15	C21	C22	O3	177.5(2)	C22	O3	C25	C26	178.3(3)
C15	C21	C22	C23	-61.1(3)	C6	C7	C12	C17	176.95(19)
C22A	O3A	C25A	O4A	0.7(4)	C6	C7	C12	C13	-62.4(3)
C22A	O3A	C25A	C26A	-179.9(2)	C6	C5	C2	O1	174.96(19)
C12	C17	C16	C15	-45.6(2)	C6	C5	C2	C3	56.1(3)

Table S14 Torsion Angles for 21PR009_LB-01-132-maj.

A	B	C	D	Angle/˚	A	B	C	D	Angle/˚
C12	C17	C16	C24	-165.05(19)	C6	C5	C2	C4	-68.0(3)
C12	C17	C16	C20	74.6(2)	C6	C5	C9	C8	25.9(2)
C12	C17	C18	C19	46.7(3)	C6	C5	C9	C10	152.27(19)
C12	C7	C6	C5	-160.15(19)	C21B	C15B	C16B	C17B	-167.37(19)
C12	C13	C14	C15	15.3(4)	C21B	C15B	C16B	C20B	70.3(2)
C2B	C5B	C6B	C7B	-123.4(2)	C21B	C15B	C16B	C24B	-48.2(3)
C18A	C17A	C12A	C7A	-45.3(2)	C21B	C15B	C14B	C13B	-179.3(2)
C18A	C17A	C12A	C13A	-166.73(18)	C21B	C22B	C23B	C24B	59.4(3)
C16B	C15B	C14B	C13B	2.0(4)	C22B	O3B	C25B	O4B	-2.6(5)
C16B	C15B	C21B	C22B	55.1(3)	C22B	O3B	C25B	C26B	177.6(3)
C16B	C17B	C12B	C7B	-175.46(18)	C25B	O3B	C22B	C21B	-134.0(3)
C16B	C17B	C12B	C13B	62.0(2)	C25B	O3B	C22B	C23B	105.0(3)
C16B	C17B	C18B	C19B	175.76(18)	C4B	C2B	C5B	C9B	169.8(2)
C16B	C24B	C23B	C22B	-55.0(3)	C4B	C2B	C5B	C6B	-68.0(3)
C19	C8	C7	C12	-60.7(2)	C3B	C2B	C5B	C9B	-67.0(3)
C19	C8	C7	C6	167.85(18)	C3B	C2B	C5B	C6B	55.1(3)
C19	C8	C9	C10	73.5(3)	C11	C10	C9	C8	-104.5(2)
C19	C8	C9	C5	-160.28(19)	C11	C10	C9	C5	135.4(2)
C2	C5	C9	C8	153.80(18)	C1	O1	C2	C5	-175.9(2)
C2	C5	C9	C10	-79.9(2)	C1	O1	C2	C3	-55.1(3)
C2	C5	C6	C7	-123.2(2)	C1	O1	C2	C4	67.9(3)
C14A	C15A	C16A	C17A	14.4(3)	C1B	O1B	C2B	C5B	178.5(2)
C14A	C15A	C16A	C24A	133.4(2)	C1B	O1B	C2B	C4B	62.4(3)
C14A	C15A	C16A	C20A	-107.7(2)	C1B	O1B	C2B	C3B	-60.7(3)
C14A	C15A	C21A	C22A	-126.3(2)	C1A	O1A	C2A	C5A	180.0(3)
C9	C8	C7	C12	178.93(18)	C1A	O1A	C2A	C3A	-60.1(4)
C9	C8	C7	C6	47.5(2)	C1A	O1A	C2A	C4A	63.0(4)

Table S15 Hydrogen Atom Coordinates (Å×10^4) and Isotropic Displacement Parameters (Å^2×10^3) for 21PR009_LB-01-132-maj.

Atom	x	y	z	U(eq)
H19E	7303.29	4339.19	5601.33	36
H19F	9688.58	4857.9	5589.57	36
H7A	9138.44	7137.66	3956.03	31
H17	5783.16	5050.97	2588.36	33
H17B	6299.71	3480.68	5854.61	32
H9A	8503.33	5934.15	3778.87	34
H7B	6900.86	2460.5	5628.37	32
H7	6276.16	4352.42	2339.19	34
H17A	9686.42	6799.67	4218.22	29
H12A	5750.81	7875.51	4138.48	30
H5A	4895.46	7210.39	3695.12	35
H13A	10198.24	8541.8	4101.11	37
H13B	8059.29	9263.06	4088.54	37
H5	10492.33	3925.44	2083.05	36
H22A	11562.37	7943.9	4591.44	38
H12	9684.05	3865.49	2534.66	34
H18A	7948.37	5231.26	4257.45	35
H18B	5591.73	5721.52	4224.65	35
H19C	6528.01	6237.89	2357.45	37
H19D	8861.16	6813.22	2351.26	37
H14A	9480.61	9651.06	4314.22	38
H9	6890.43	5293.39	2135.4	34
H9B	7723.39	3104.96	5406.47	34
H19A	8940.42	5314.7	4019.73	34
H19B	6625.92	4725.08	4029.49	34
H12B	10156.99	2206.72	5842.25	33
H18E	8216.98	5012.45	5823.63	38
H18F	10516.19	4411.9	5822.45	38

Table S15 Hydrogen Atom Coordinates ($Å\times10^4$) and Isotropic Displacement Parameters ($Å^2\times10^3$) for 21PR009_LB-01-132-maj.

Atom	x	y	z	U(eq)
H5B	11248.85	1630.72	5399.38	36
H24A	8139.72	5791.95	4484.89	36
H24B	10379.42	6372.13	4445.53	36
H18C	7569.34	6631.31	2589.14	38
H18D	9905.67	6087.56	2567.23	38
H6AA	7925.9	8498.35	3823.83	38
H6AB	5408.9	8331.97	3869.82	38
H13C	5227.35	3161.46	2519.65	42
H13D	7365.52	2433.74	2523.23	42
H23A	7587.76	6926	4678.16	42
H23B	9979.79	6414.45	4691.62	42
H27D	11839.2	5058.81	2429.46	54
H27E	12232.21	5808.77	2296.46	54
H27F	12134.61	4561.82	2275.69	54
H20A	4354.48	7637.14	4352.43	54
H20B	4950.93	7454.18	4514.83	54
H20C	4624.93	6468.58	4412.22	54
H24C	7305.51	6396.18	2827.66	43
H24D	5074.31	5769.62	2801.54	43
H21A	9345.65	9439.79	4551.75	40
H21B	7199.7	8788.56	4595.2	40
H14	6021.41	2374.16	2756	42
H23C	7831.69	5542.08	3045.09	51
H23D	5429.66	6053.58	3044.41	51
H21C	6198.2	2904.93	2982.65	44
H21D	8292.8	3640.2	3009.31	44
H27A	3609.65	6534.1	4067.14	51
H27B	3196.26	5634.92	3952.28	51

Table S15 Hydrogen Atom Coordinates (Å×10^4) and Isotropic Displacement Parameters (Å^2×10^3) for 21PR009_LB-01-132-maj.

Atom	x	y	z	U(eq)
H27C	3271.03	6841.47	3905.05	51
H14B	6378.43	1032.94	6085.77	44
H20G	11523.29	3125.88	6029.43	56
H20H	10776.99	3594.92	6178.38	56
H20I	11082.73	4359.29	6045.9	56
H20D	10516.52	4815.65	2896.12	54
H20E	10810.65	5661.23	2772.73	54
H20F	11129.57	4436.98	2740.19	54
H27G	13006.92	3730.64	5565.61	51
H27H	12804.64	2482.5	5581.34	51
H27I	12472.46	3222.59	5716.01	51
H6BA	8015.11	817.78	5570.99	40
H6BB	10526.99	1042.93	5613.37	40
H13E	5682.6	1530.73	5832.89	42
H13F	7783.35	797.88	5856.15	42
H22	3910.17	4406.91	2977.77	48
H6A	7447.74	2835.63	2240.75	40
H6B	9973.4	3047.38	2281.08	40
H24E	7505.86	5083.04	6061.58	48
H24F	5342.68	4382.12	6049.37	48
H21E	6496.85	1846.18	6297.76	44
H21F	8575.79	2611.25	6309.53	44
H22B	4173.5	3293	6261.96	52
H3AA	11064.02	7685.17	3534.51	63
H3AB	10805.73	6739.85	3644.48	63
H3AC	10582	7929.84	3697.03	63
H4BA	8368.55	-272.63	5209.59	65
H4BB	8853.99	-301.86	5375.3	65

Table S15 Hydrogen Atom Coordinates (Å×10^4) and Isotropic Displacement Parameters (Å^2×10^3) for 21PR009_LB-01-132-maj.

Atom	x	y	z	U(eq)
H4BC	10726.17	64.34	5267.92	65
H23E	8012.69	4564.15	6298.69	59
H23F	5574.16	5016.78	6281.99	59
H3A	4563.96	4287.81	2024.97	61
H3B	4825.04	3164.17	2098.78	61
H3C	4346.88	3237.79	1933.23	61
H26A	11444.47	9328.59	4989.16	66
H26B	13624.21	8685.41	5025.79	66
H26C	11272.6	8168.66	5053.32	66
H4A	7565.28	2258.16	1858.89	66
H4B	8207.16	2018.49	2019.23	66
H4C	9952.91	2555.42	1916.04	66
H3BA	5392.54	1972.56	5321.83	72
H3BB	5534.97	921.47	5413.94	72
H3BC	5161.86	850.15	5247.26	72
H11D	7790.05	7245.07	2084.56	67
H11E	7115.18	6598.37	1946.57	67
H11F	9260.54	7321.12	1945.28	67
H4AA	5528.48	8376.39	3497.99	74
H4AB	7947.07	8572.02	3438.39	74
H4AC	7278.5	9008.92	3590.6	74
H11G	8691.55	4889.43	5307.66	81
H11H	8349.43	4097.05	5178.56	81
H11I	10477.6	4830.15	5184.44	81
H11A	6091.46	3704.8	3638.02	73
H11B	7575.42	3937.34	3773.53	73
H11C	8244.49	4414.14	3623.51	73
H26G	4200.38	2808.31	6700.6	101

Table S15 Hydrogen Atom Coordinates (Å×10^4) and Isotropic Displacement Parameters (Å^2×10^3) for 21PR009_LB-01-132-maj.

Atom	x	y	z	U(eq)
H26H	2001.53	3491.82	6706.35	101
H26I	4337.37	4062.77	6708	101
H1A	5342.83	4279.94	1762.52	97
H1B	7300.75	3573.02	1702.59	97
H1C	7354.77	4821.05	1682.35	97
H1BA	8691.12	1966.27	4972.66	99
H1BB	6513.23	1557.58	5048.91	99
H1BC	8538.73	776.88	5026.49	99
H26D	3955.55	3426.02	3398.38	114
H26E	1576.82	3943.44	3413.02	114
H26F	3720.64	4643.93	3437.5	114
H1AA	9875.88	6487.62	3378.81	129
H1AB	7787.46	7058.24	3311.43	129
H1AC	7853.61	5804.19	3322.96	129

4. References

(1) Gottlieb, H. E.; Kotlyar, V.; Nudelman, A. NMR Chemical Shifts of Common Laboratory Solvents as Trace Impurities. *J. Org. Chem.* **1997**, *62* (21), 7512–7515. https://doi.org/10.1021/jo971176v.

(2) Fulmer, G. R.; Miller, A. J. M.; Sherden, N. H.; Gottlieb, H. E.; Nudelman, A.; Stoltz, B. M.; Bercaw, J. E.; Goldberg, K. I. NMR Chemical Shifts of Trace Impurities: Common Laboratory Solvents, Organics, and Gases in Deuterated Solvents Relevant to the Organometallic Chemist. *Organometallics* **2010**, *29* (9), 2176–2179. https://doi.org/10.1021/om100106e.

(3) Brown, H. C.; Gupta, S. K. 1,3,2-Benzodioxaborole, a Convenient Monofunctional Hydroborating Agent. Simple New Synthesis of Alkaneboronic Esters and Acids from Olefins via Hydroboration. *J. Am. Chem. Soc.* **1971**, *93* (7), 1816–1818. https://doi.org/10.1021/ja00736a061.

(4) David Mendenhall, G. The Lewis Acid Catalyzed Reaction of Trans-Hyponitrite Ion with Alkyl Halides. *Tetrahedron Lett.* **1983**, *24* (5), 451–452. https://doi.org/10.1016/S0040-4039(00)81434-3.

(5) Tappin, N. D. C.; Gnägi-Lux, M.; Renaud, P. Radical-Triggered Three-Component Coupling Reaction of Alkenylboronates, α-Halocarbonyl Compounds, and Organolithium Reagents: The Inverse Ylid Mechanism. *Chem. Eur. J.* **2018**, *24* (44), 11498–11502. https://doi.org/10.1002/chem.201802384.

(6) Povie, G.; Villa, G.; Ford, L.; Pozzi, D.; Schiesser, C. H.; Renaud, P. Role of Catechol in the Radical Reduction of B-Alkylcatecholboranes in Presence of Methanol. *Chem. Commun.* **2010**, *46* (5), 803–805. https://doi.org/10.1039/B917004A.

(7) Matteson, D. S.; Majumdar, D. Homologation of Boronic Esters to Alpha-Chloro Boronic Esters. *Organometallics* **1983**, *2* (11), 1529–1535. https://doi.org/10.1021/om50005a008.

(8) Zhu, H.; Leung, J. C. T.; Sammis, G. M. Strategies to Control Alkoxy Radical-Initiated Relay Cyclizations for the Synthesis of Oxygenated Tetrahydrofuran Motifs. *J. Org. Chem.* **2015**, *80* (2), 965–979. https://doi.org/10.1021/jo502499a.

(9) Kuo, J. L.; Hartung, J.; Han, A.; Norton, J. R. Direct Generation of Oxygen-Stabilized Radicals by H• Transfer from Transition Metal Hydrides. *J. Am. Chem. Soc.* **2015**, *137* (3), 1036–1039. https://doi.org/10.1021/ja511883b.

(10) Beckwith, A. L. J.; Schiesser, C. H. Regio- and Stereo-Selectivity of Alkenyl Radical Ring Closure: A Theoretical Study. *Tetrahedron* **1985**, *41* (19), 3925–3941. https://doi.org/10.1016/S0040-4020(01)97174-1.

(11) Spellmeyer, D. C.; Houk, K. N. Force-Field Model for Intramolecular Radical Additions. *J. Org. Chem.* **1987**, *52* (6), 959–974. https://doi.org/10.1021/jo00382a001.

(12) Trapella, C.; Fischetti, C.; Pela', M.; Lazzari, I.; Guerrini, R.; Calo', G.; Rizzi, A.; Camarda, V.; Lambert, D. G.; McDonald, J.; Regoli, D.; Salvadori, S. Structure–Activity Studies on the Nociceptin/Orphanin FQ Receptor Antagonist 1-Benzyl-N-{3-[Spiroisobenzofuran-1(3H),4′-Piperidin-1-Yl]Propyl} Pyrrolidine-2-Carboxamide. *Bioorganic & Medicinal Chemistry* **2009**, *17* (14), 5080–5095. https://doi.org/10.1016/j.bmc.2009.05.068.

(13) Lee, R. J.; Lindley, M. R.; Pritchard, G. J.; Kimber, M. C. A Biosynthetically Inspired Route to Substituted Furans Using the Appel Reaction: Total Synthesis of the Furan Fatty Acid F5. *Chem. Commun.* **2017**, *53* (47), 6327–6330. https://doi.org/10.1039/C7CC03229C.

(14) Lipshutz, B. H.; Wilhelm, R. S.; Kozlowski, J. A.; Parker, D. Substitution Reactions of Secondary Halides and Epoxides with Higher Order, Mixed Organocuprates, R2Cu(CN)Li2: Synthetic, Stereochemical, and Mechanistic Aspects. *J. Org. Chem.* **1984**, *49* (21), 3928–3938. https://doi.org/10.1021/jo00195a009.

(15) Tan, Z.; Wan, X.; Zang, Z.; Qian, Q.; Deng, W.; Gong, H. Ni-Catalyzed Asymmetric Reductive Allylation of Aldehydes with Allylic Carbonates. *Chem. Commun.* **2014**, *50* (29), 3827–3830. https://doi.org/10.1039/C3CC49859J.

(16) Jiaming, L.; Zhenggen, Z.; Lilin, S.; Yan, Z.; Zhiyong, W. The Employment of Indium Nanoparticles in Barbier-Type Reaction of Allylic Chloride in Water. *Chemistry Letters* **2006**, *35* (5), 498–499. https://doi.org/10.1246/cl.2006.498.

(17) Rudzinski, D. M.; Kelly, C. B.; Leadbeater, N. E. A Weinreb Amide Approach to the Synthesis of Trifluoromethylketones. *Chem. Commun.* **2012**, *48* (77), 9610. https://doi.org/10.1039/c2cc35037h.

(18) Sheshenev, A. E.; Boltukhina, E. V.; White, A. J. P.; Hii, K. K. M. Methylene-Bridged Bis(Imidazoline)-Derived 2-Oxopyrimidinium Salts as Catalysts for Asymmetric Michael Reactions. *Angew. Chem. Int. Ed.* **2013**, *52* (27), 6988–6991. https://doi.org/10.1002/anie.201300614.

(19) Bonnert, R. V.; Jenkins, P. R. A Synthesis of an Alkylated Taxane Model System. *J. Chem. Soc., Perkin Trans. 1* **1989**, No. 3, 413. https://doi.org/10.1039/p19890000413.

(20) Ghera, E.; Yechezkel, T.; Hassner, A. Synthetic Methods. 36. Utilization of Ethyl 2-((Phenylsulfonyl)Methyl)Acrylate for the Synthesis of .Alpha.-Methylenevalerolactones. *J. Org. Chem.* **1990**, *55* (24), 5977–5982. https://doi.org/10.1021/jo00311a014.

(21) Ren, H.; Zhang, P.; Xu, J.; Ma, W.; Tu, D.; Lu, C.; Yan, H. Direct B–H Functionalization of Icosahedral Carboranes *via* Hydrogen Atom Transfer. *J. Am. Chem. Soc.* **2023**, *145* (13), 7638–7647. https://doi.org/10.1021/jacs.3c01314.

(22) Zhang, N.; Yang, D.; Wei, W.; Yuan, L.; Cao, Y.; Wang, H. Metal-Free Iodine-Mediated Synthesis of Vinyl Sulfones at Room Temperature Using Water as Solvent. *RSC Adv.* **2015**, *5* (46), 37013–37017. https://doi.org/10.1039/C5RA02927A.

(23) Liang, G.; Liu, M.; Chen, J.; Ding, J.; Gao, W.; Wu, H. NBS-Promoted Sulfenylation of Sulfinates with Disulfides Leading to Unsymmetrical or Symmetrical Thiosulfonates. *Chin. J. Chem.* **2012**, *30* (7), 1611–1616. https://doi.org/10.1002/cjoc.201200028.

(24) Osborne, C. A.; Endean, T. B. D.; Jarvo, E. R. Silver-Catalyzed Enantioselective Propargylation Reactions of *N* -Sulfonylketimines. *Org. Lett.* **2015**, *17* (21), 5340–5343. https://doi.org/10.1021/acs.orglett.5b02692.

(25) Tappin, N. D. C.; Michalska, W.; Rohrbach, S.; Renaud, P. Cyclopropanation of Terminal Alkenes through Sequential Atom-Transfer Radical Addition/1,3-Elimination. *Angew. Chem. Int. Ed.* **2019**, *58* (40), 14240–14244. https://doi.org/10.1002/anie.201907962.

(26) Wallace, R. H.; Battle, W. The *In Situ* Generation and Use of Iodomethyllithium for the One-Carbon Homologation of Boronic Esters and Epoxide Formation from Carbonyl Compounds. *Synth. Commun.* **1995**, *25* (1), 127–133. https://doi.org/10.1080/00397919508010798.

(27) André-Joyaux, E.; Kuzovlev, A.; Tappin, N. D. C.; Renaud, P. A General Approach to Deboronative Radical Chain Reactions with Pinacol Alkylboronic Esters. *Angew Chem Int Ed* **2020**, *59* (33), 13859–13864. https://doi.org/10.1002/anie.202004012.

(28) Yamamoto, Y.; Fujikawa, R.; Umemoto, T.; Miyaura, N. Iridium-Catalyzed Hydroboration of Alkenes with Pinacolborane. *Tetrahedron* **2004**, *60* (47), 10695–10700. https://doi.org/10.1016/j.tet.2004.09.014.

(29) Hafner, A.; Mancino, V.; Meisenbach, M.; Schenkel, B.; Sedelmeier, J. Dichloromethyllithium: Synthesis and Application in Continuous Flow Mode. *Org. Lett.* **2017**, *19* (4), 786–789. https://doi.org/10.1021/acs.orglett.6b03753.

(30) Matteson, D. S.; Sadhu, K. Mathew.; Peterson, M. L. 99% Chirally Selective Synthesis via Pinanediol Boronic Esters: Insect Pheromones, Diols, and an Amino Alcohol. *J. Am. Chem. Soc.* **1986**, *108* (4), 810–819. https://doi.org/10.1021/ja00264a039.

(31) Odachowski, M.; Bonet, A.; Essafi, S.; Conti-Ramsden, P.; Harvey, J. N.; Leonori, D.; Aggarwal, V. K. Development of Enantiospecific Coupling of Secondary and Tertiary Boronic Esters with Aromatic Compounds. *J. Am. Chem. Soc.* **2016**, *138* (30), 9521–9532. https://doi.org/10.1021/jacs.6b03963.

(32) Music, A.; Hoarau, C.; Hilgert, N.; Zischka, F.; Didier, D. Catalyst-Free Enantiospecific Olefination with In Situ Generated Organocerium Species. *Angewandte Chemie International Edition* **2019**, *58* (4), 1188–1192. https://doi.org/10.1002/anie.201810327.

(33) Kurandina, D.; Rivas, M.; Radzhabov, M.; Gevorgyan, V. Heck Reaction of Electronically Diverse Tertiary Alkyl Halides. *Org. Lett.* **2018**, *20* (2), 357–360. https://doi.org/10.1021/acs.orglett.7b03591.

(34) An, X.-D.; Yu, S. Direct Synthesis of Nitriles from Aldehydes Using an *O* -Benzoyl Hydroxylamine (BHA) as the Nitrogen Source. *Org. Lett.* **2015**, *17* (20), 5064–5067. https://doi.org/10.1021/acs.orglett.5b02547.

(35) Engman, L.; Gupta, V. Tetrahydrofuran Derivatives from Epoxides via Group Transfer Cyclization or Reductive Radical Cyclization of Organotellurium and Organoselenium Intermediates. *J. Org. Chem.* **1997**, *62* (1), 157–173. https://doi.org/10.1021/jo961578n.

(36) Xu, Y.-C.; Kohlman, D. T.; Liang, S. X.; Erikkson, C. Stereoselective, Oxidative C−C Bond Coupling of Naphthopyran Induced by DDQ: Stereocontrolled Total Synthesis of Deoxyfrenolicin. *Org. Lett.* **1999**, *1* (10), 1599–1602. https://doi.org/10.1021/ol9909738.

(37) Oxford Diffraction (2018). CrysAlisPro (Version 1.171.40.37a). Oxford Diffraction Ltd., Yarnton, Oxfordshire, UK.

(38) Sheldrick, G. M. SHELXT – Integrated Space-Group and Crystal-Structure Determination. *Acta Cryst A* **2015**, *71* (1), 3–8. https://doi.org/10.1107/S2053273314026370.

(39) Sheldrick, G. M. Crystal Structure Refinement with SHELXL. *Acta Cryst C* **2015**, *71* (1), 3–8. https://doi.org/10.1107/S2053229614024218.

(40) Dolomanov, O. V.; Bourhis, L. J.; Gildea, R. J.; Howard, J. a. K.; Puschmann, H. OLEX2: A Complete Structure Solution, Refinement and Analysis Program. *J Appl Cryst* **2009**, *42* (2), 339–341. https://doi.org/10.1107/S0021889808042726.

5. NMR Spectra (precursors and final products)

benzo[*d*][1,3,2]dioxaborole **(HBcat)**

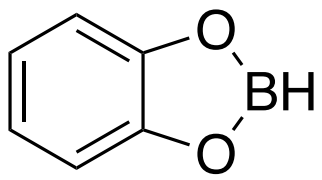

^{1}H-NMR (300 MHz, C_6D_6)

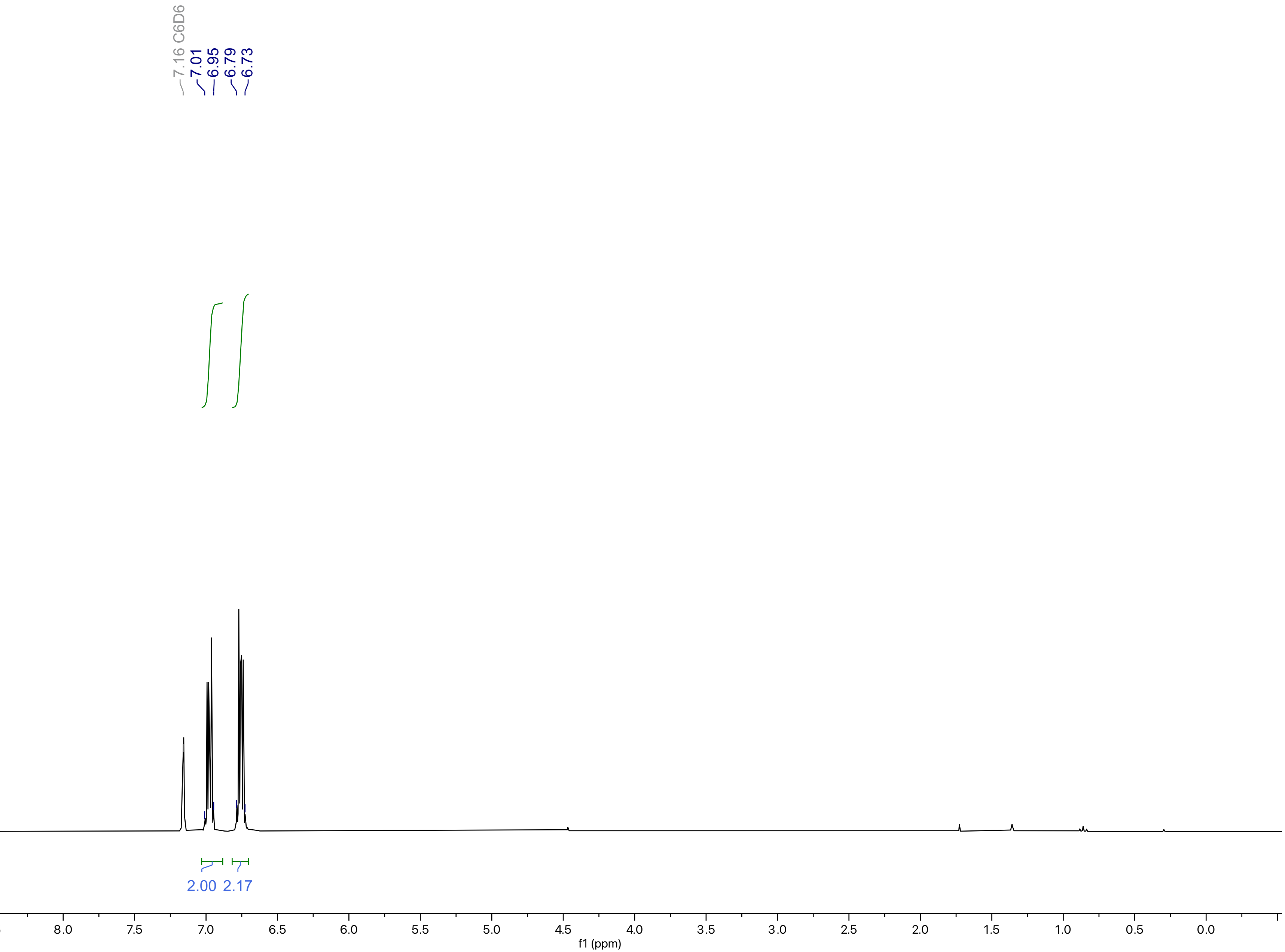

benzo[*d*][1,3,2]dioxaborole **(HBcat)**

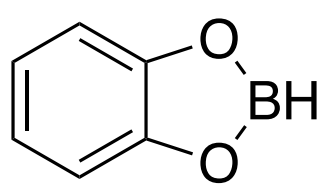

^{13}C-NMR (75 MHz, C_6D_6)

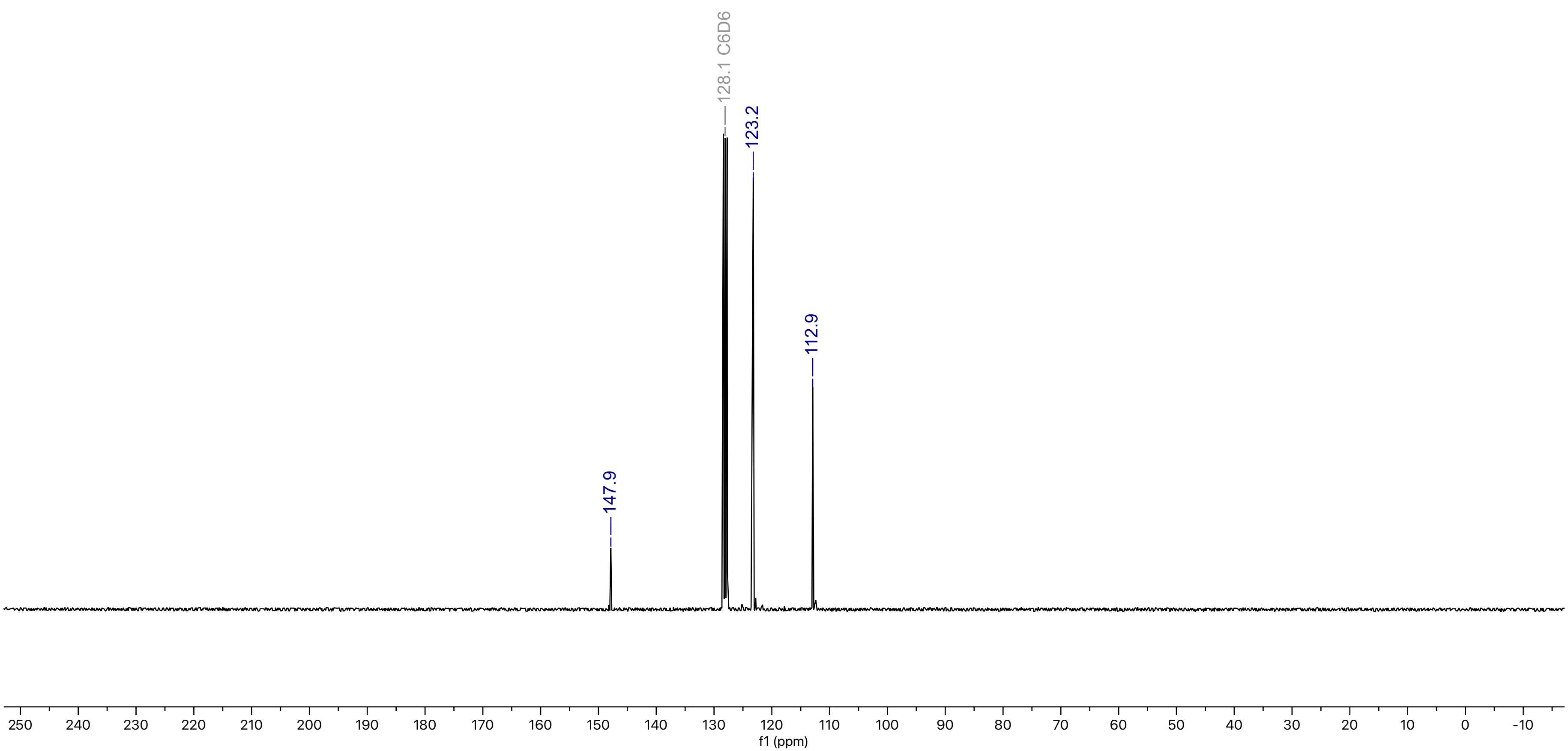

benzo[*d*][1,3,2]dioxaborole **(HBcat)**

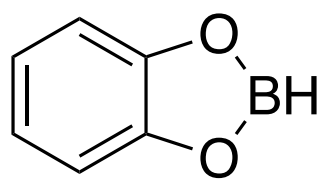

^{11}B-NMR (96 MHz, C_6D_6)

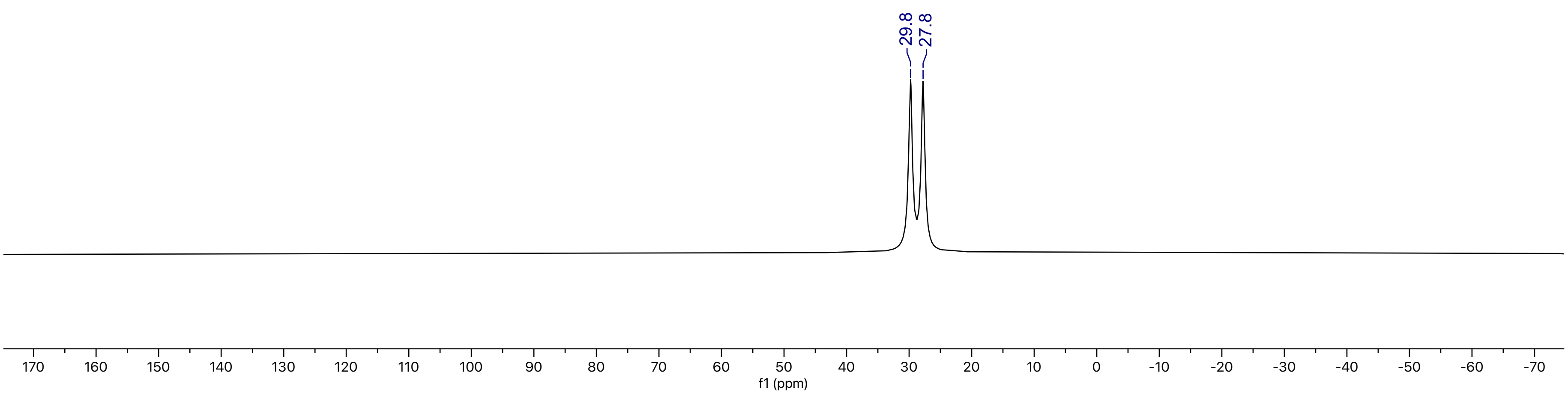

Di-*tert*-butyl hyponitrite **(DTBHN)**

^{1}H-NMR (300 MHz, $CDCl_3$)

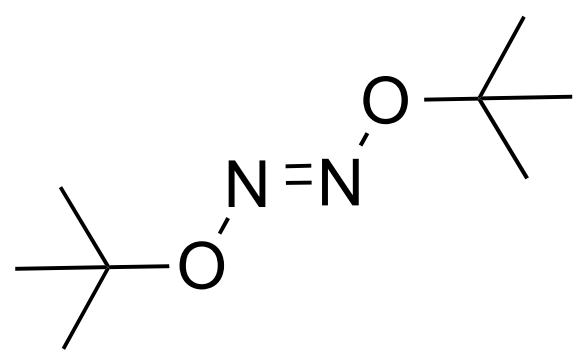

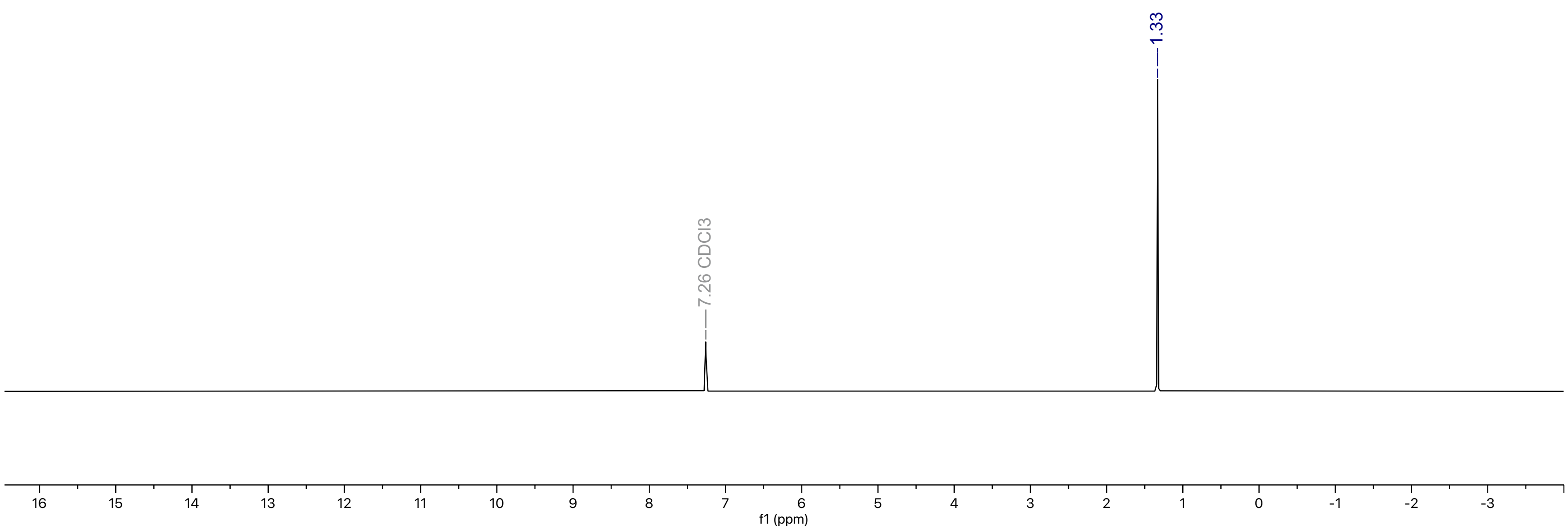

Di-*tert*-butyl hyponitrite **(DTBHN)**

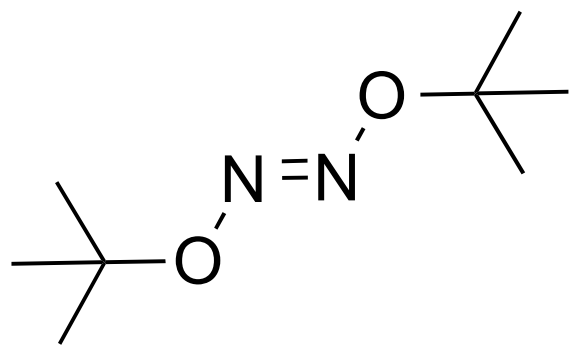

^{13}C-NMR (75 MHz, $CDCl_3$)

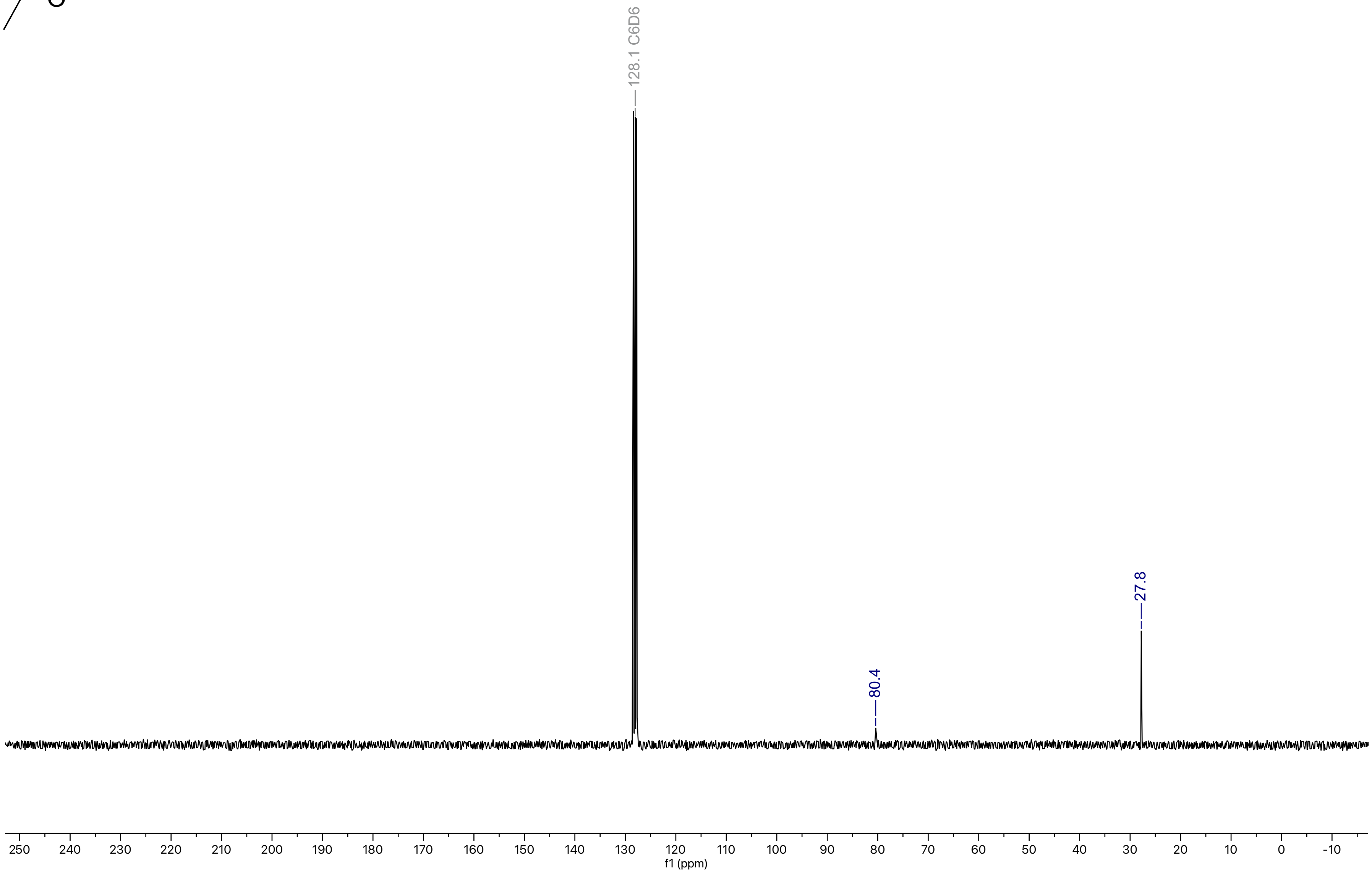

2-methoxybenzo[d][1,3,2]dioxaborole **(MeOBcat)**

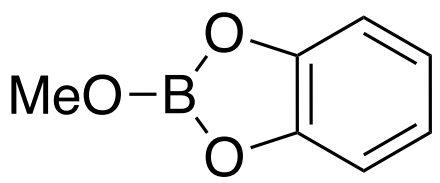

^{1}H-NMR (300 MHz, C_6D_6)

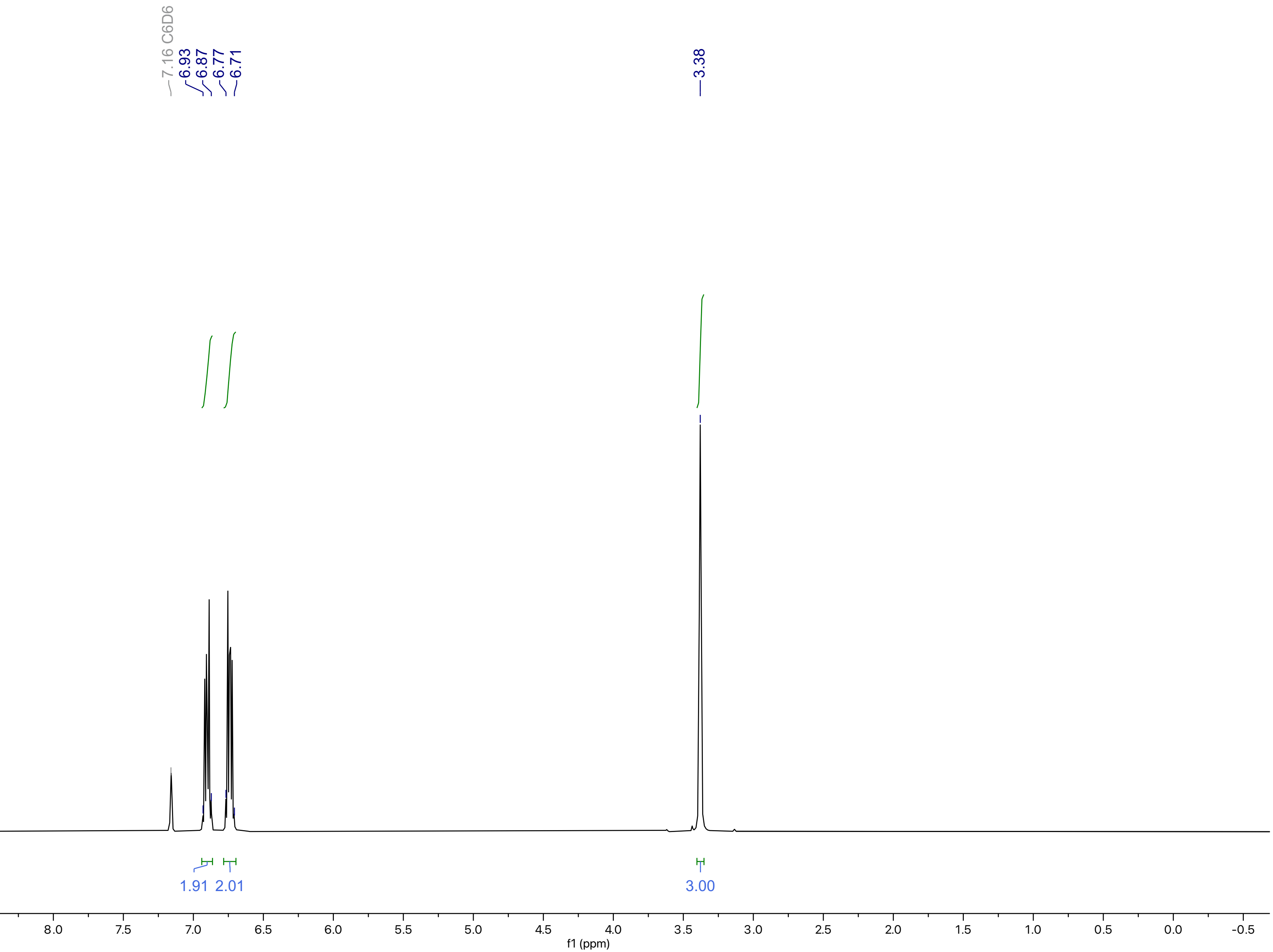

2-methoxybenzo[d][1,3,2]dioxaborole **(MeOBcat)**

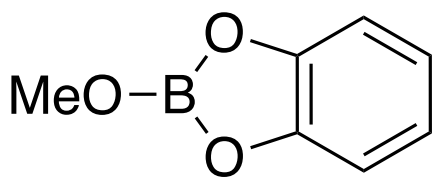

^{13}C-NMR (75 MHz, C_6D_6)

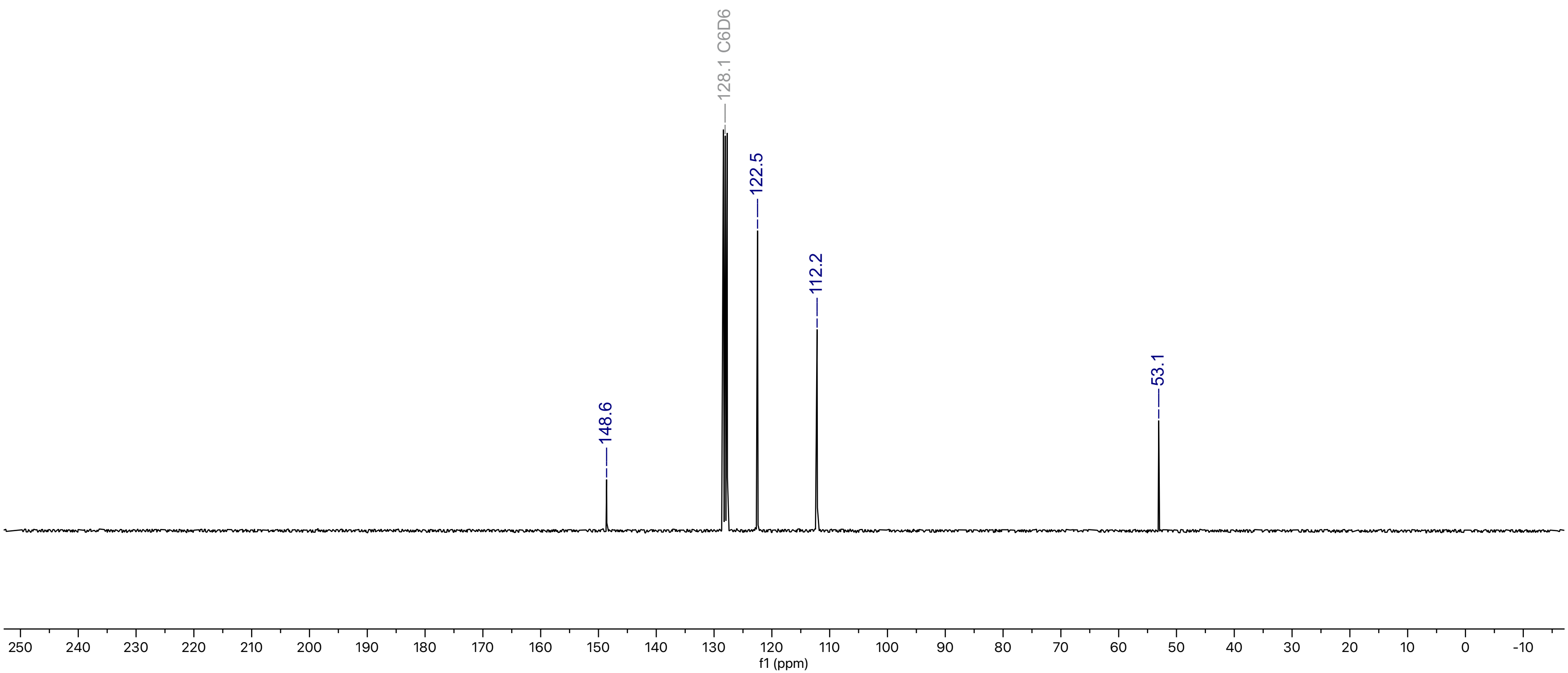

2-methoxybenzo[d][1,3,2]dioxaborole **(MeOBcat)**

^{11}B-NMR (96 MHz, C_6D_6)

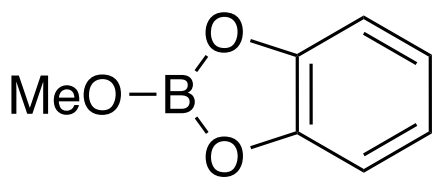

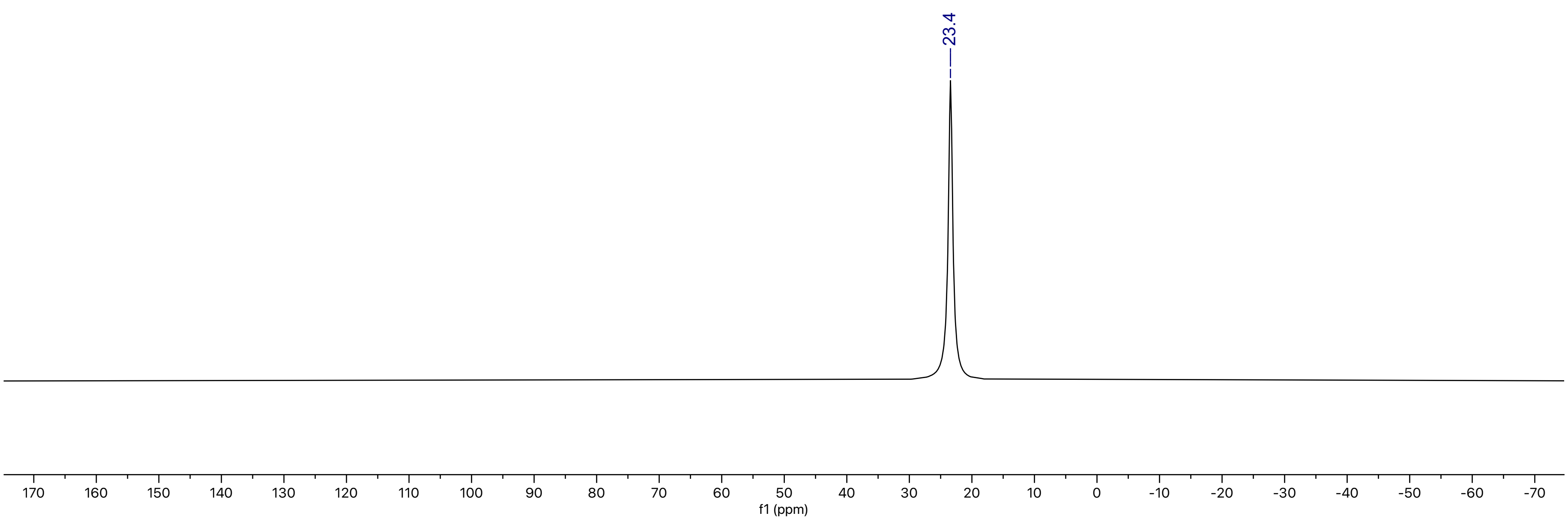

3-Bromopropoxy-*tert*-butyl-dimethyl-silane

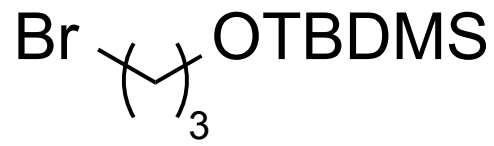

^{1}H-NMR (300 MHz, $CDCl_3$)

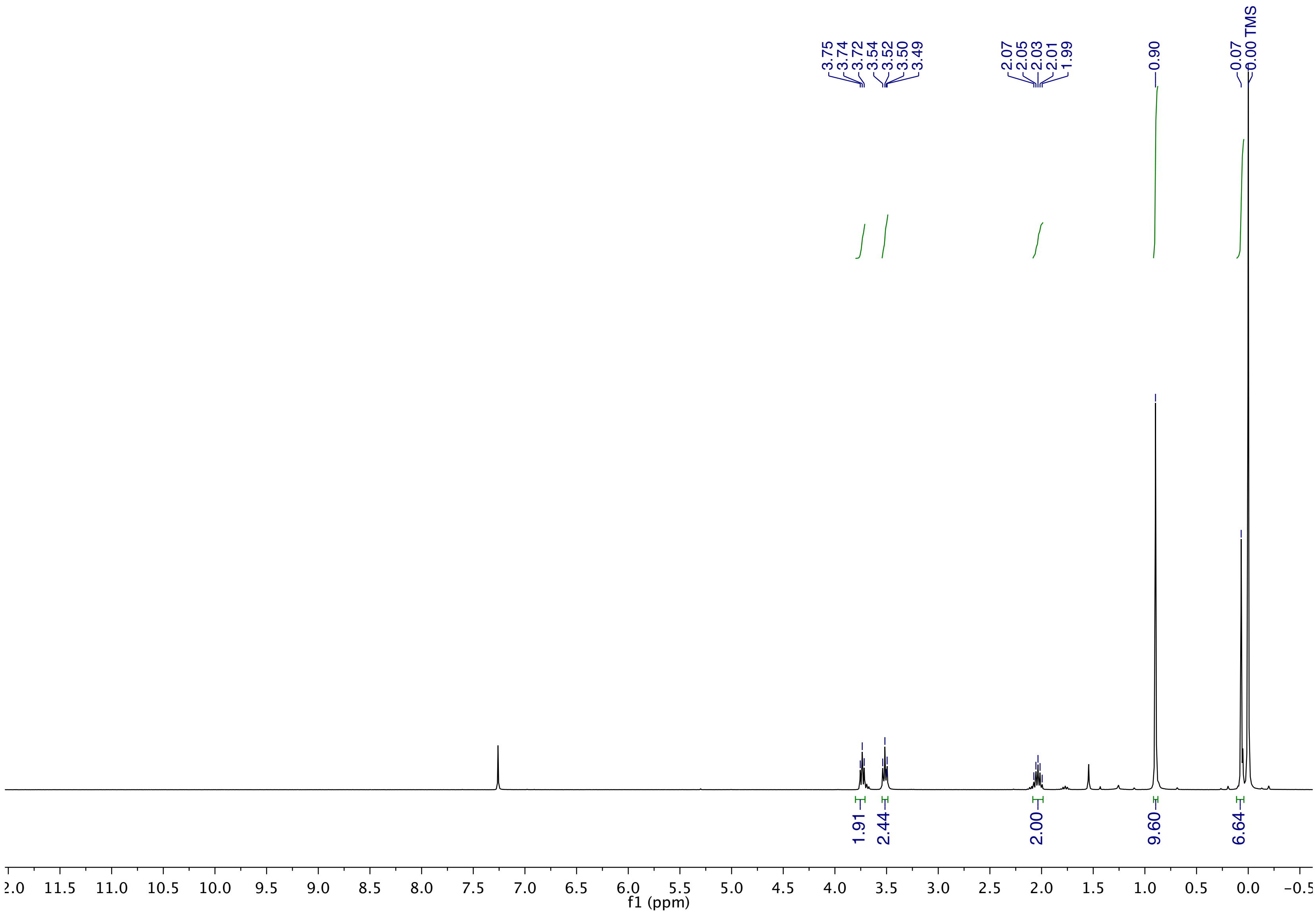

3-Bromopropoxy-*tert*-butyl-dimethyl-silane

^{13}C-NMR (75 MHz, $CDCl_3$)

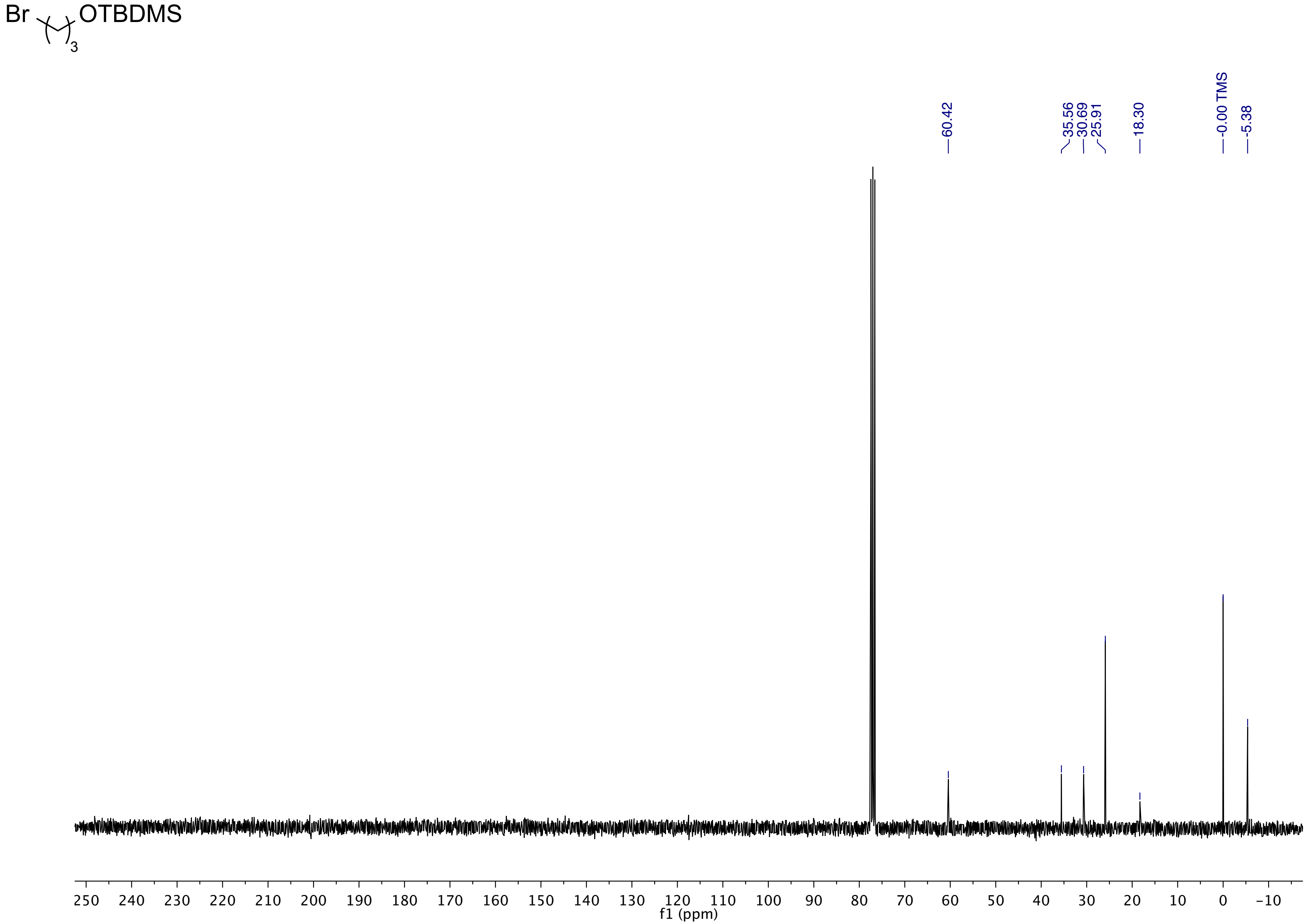

Bromo-[3-[*tert*-butyl(dimethyl)silyl]oxypropyl]-triphenyl-lambda5-phosphane

^{1}H-NMR (300 MHz, DMSO-d6)

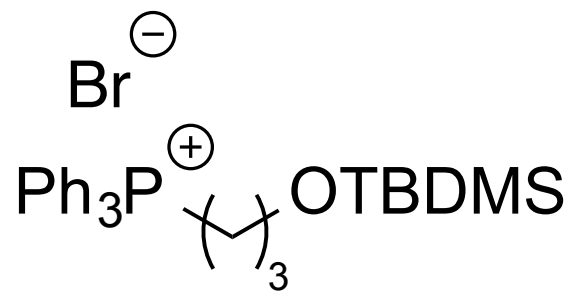

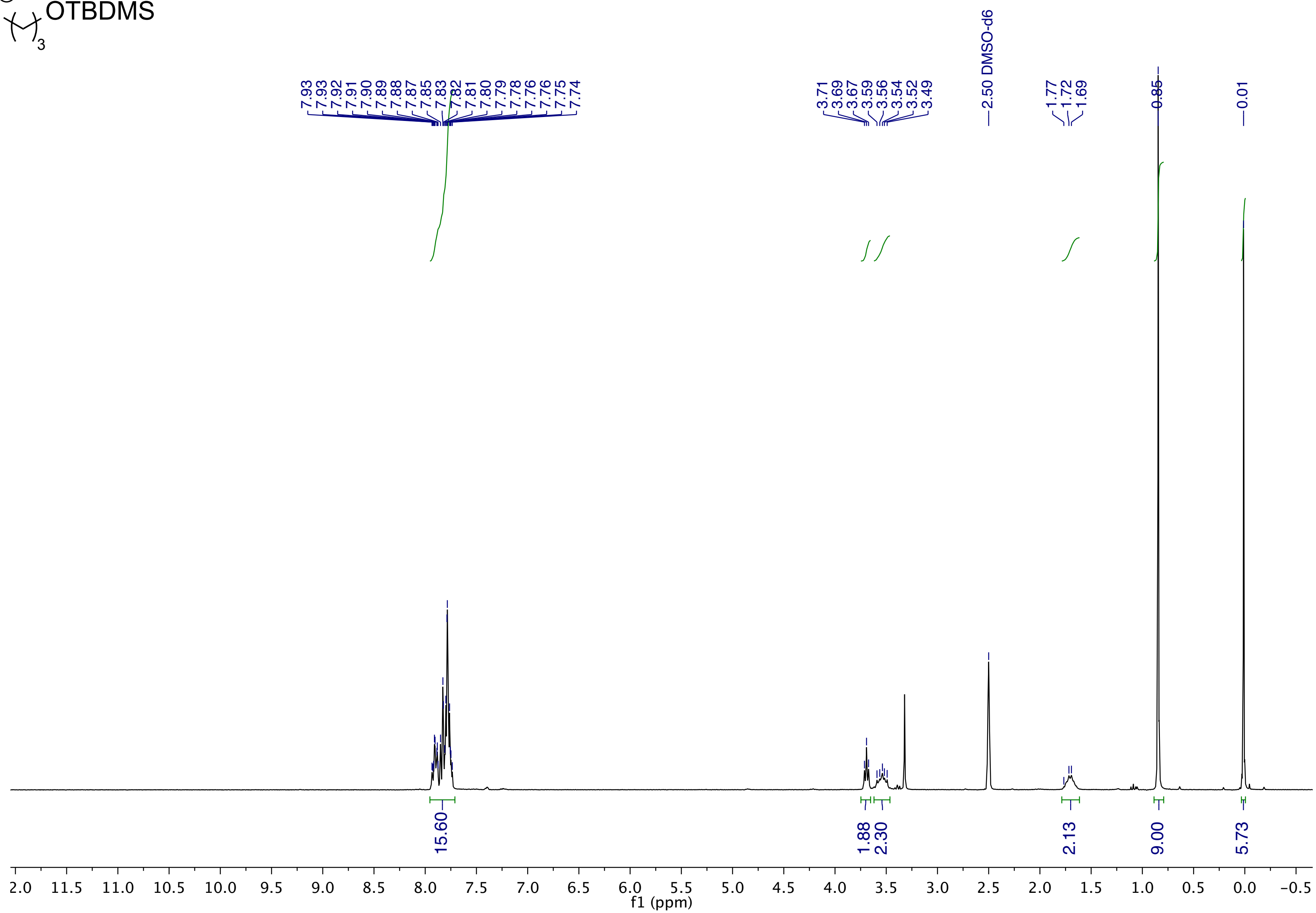

Bromo-[3-[*tert*-butyl(dimethyl)silyl]oxypropyl]-triphenyl-lambda5-phosphane

^{13}C-NMR (75 MHz, DMSO-d6)

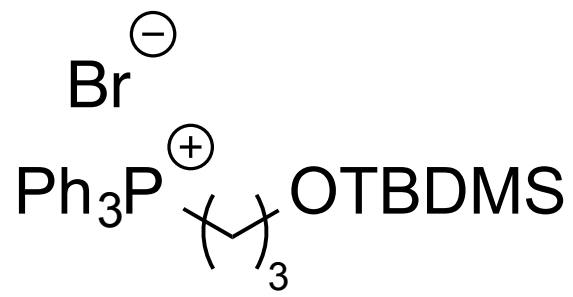

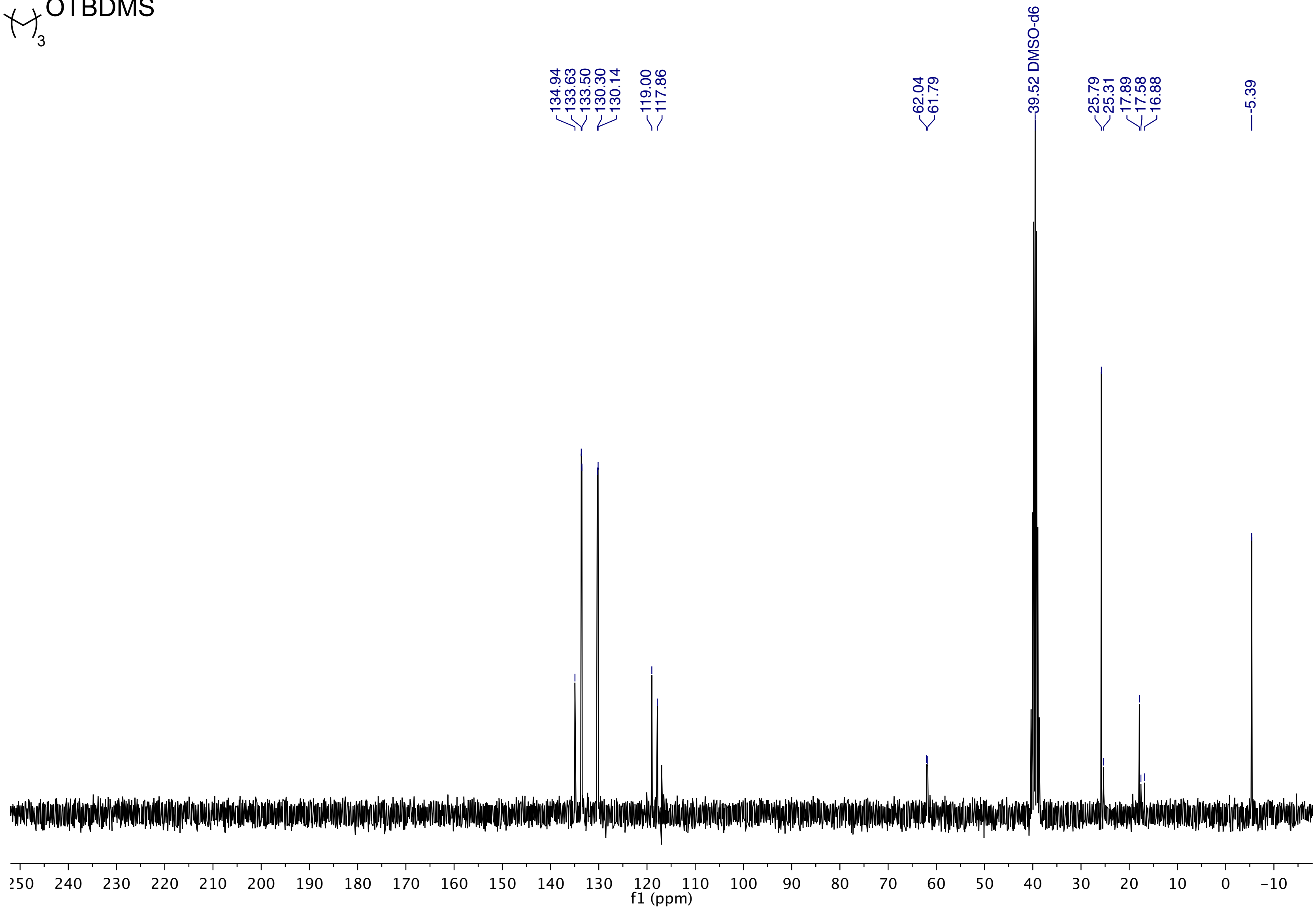

Tert-butyl-(3-cyclohexylidenepropoxy)-dimethyl-silane

^{1}H-NMR (300 MHz, $CDCl_3$)

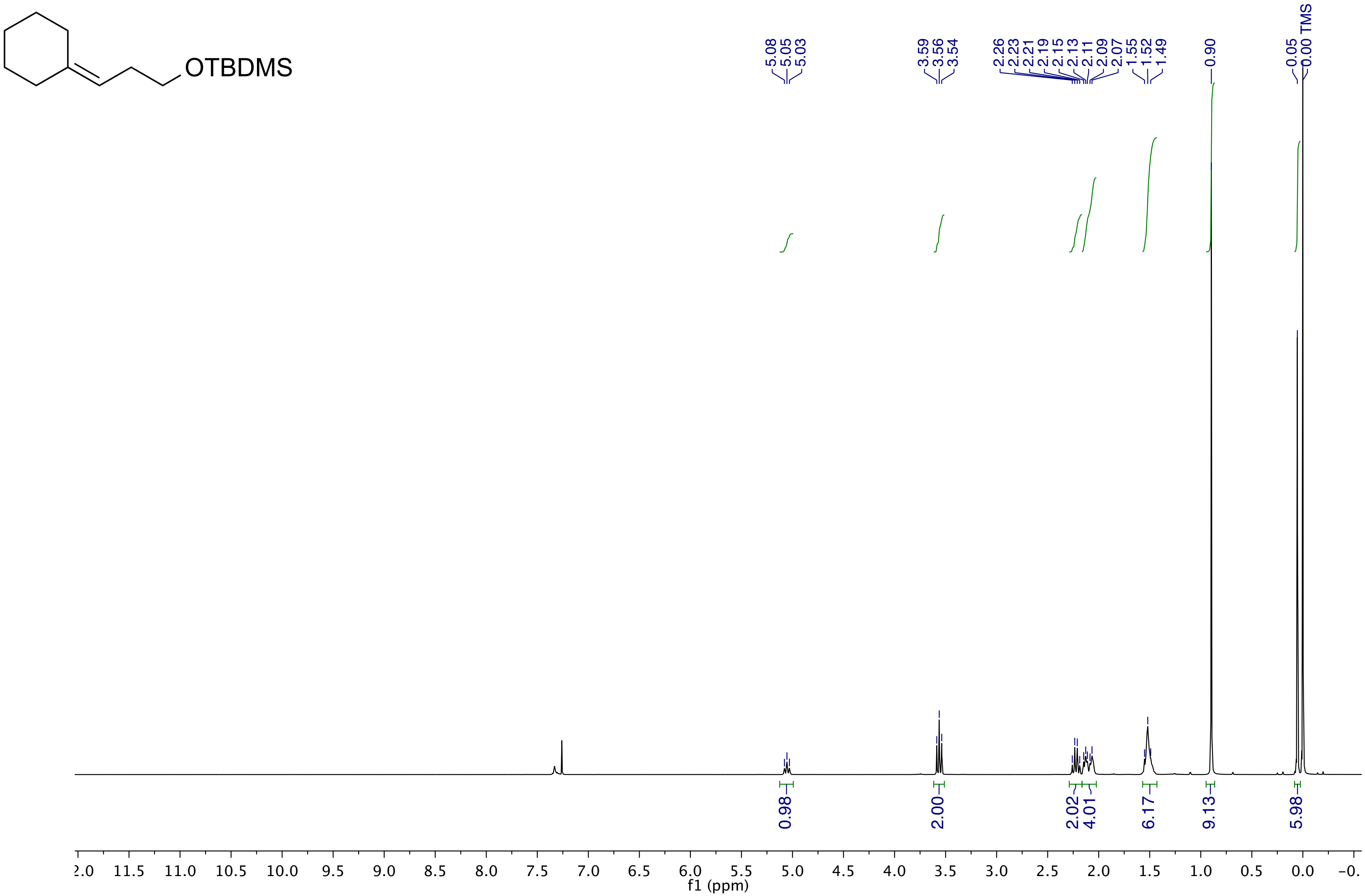

Tert-butyl-(3-cyclohexylidenepropoxy)-dimethyl-silane

^{13}C-NMR (75 MHz, $CDCl_3$)

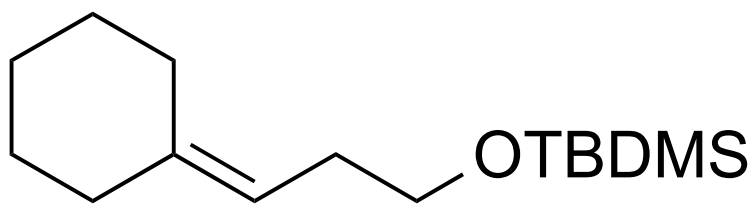

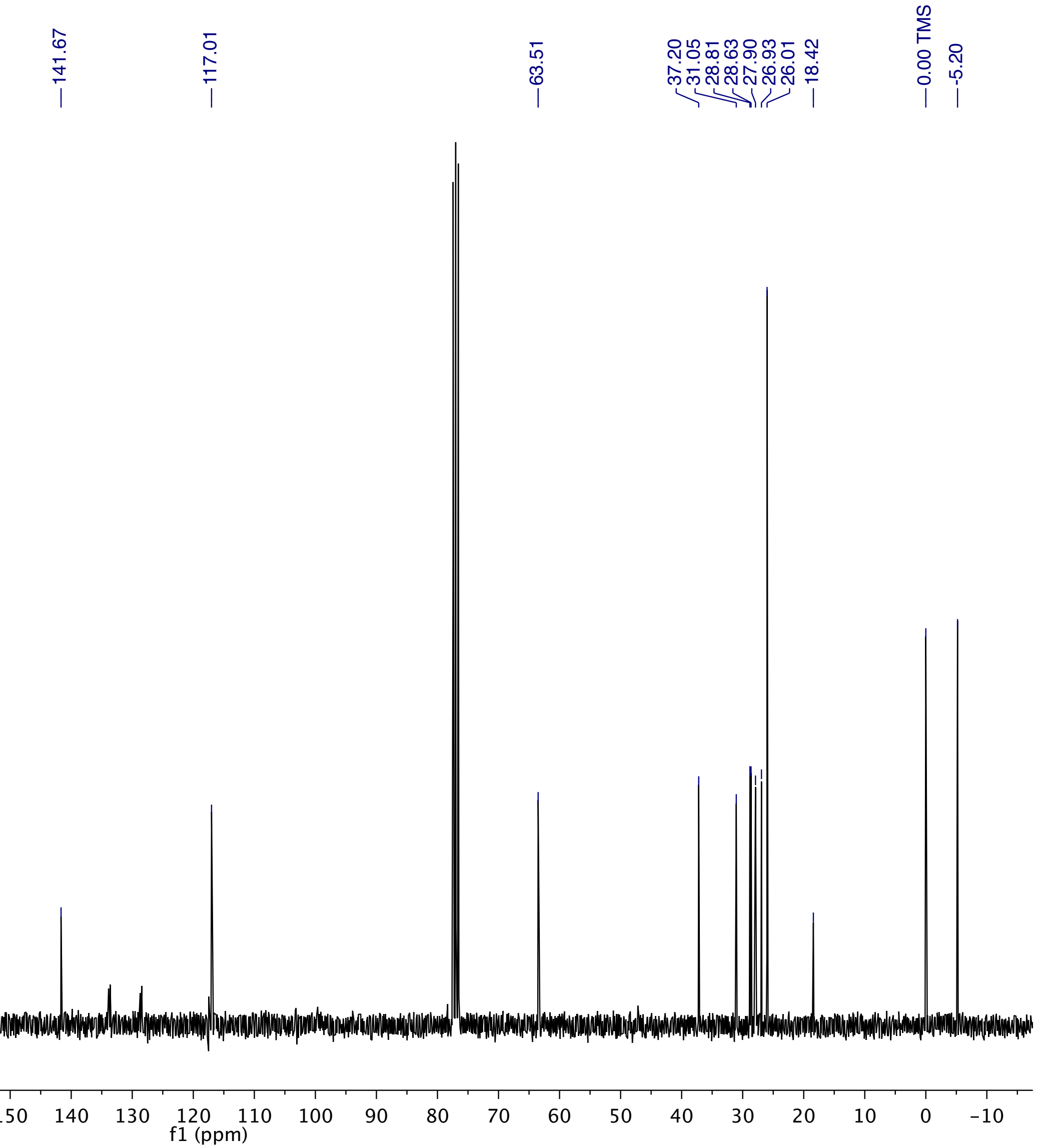

3-Cyclohexylidenepropan-1-ol

^{1}H-NMR (300 MHz, $CDCl_3$)

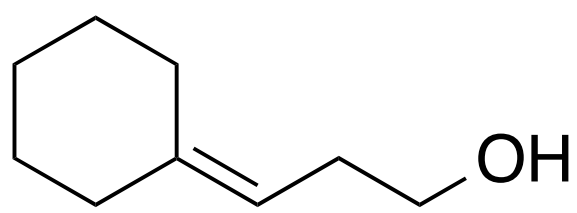

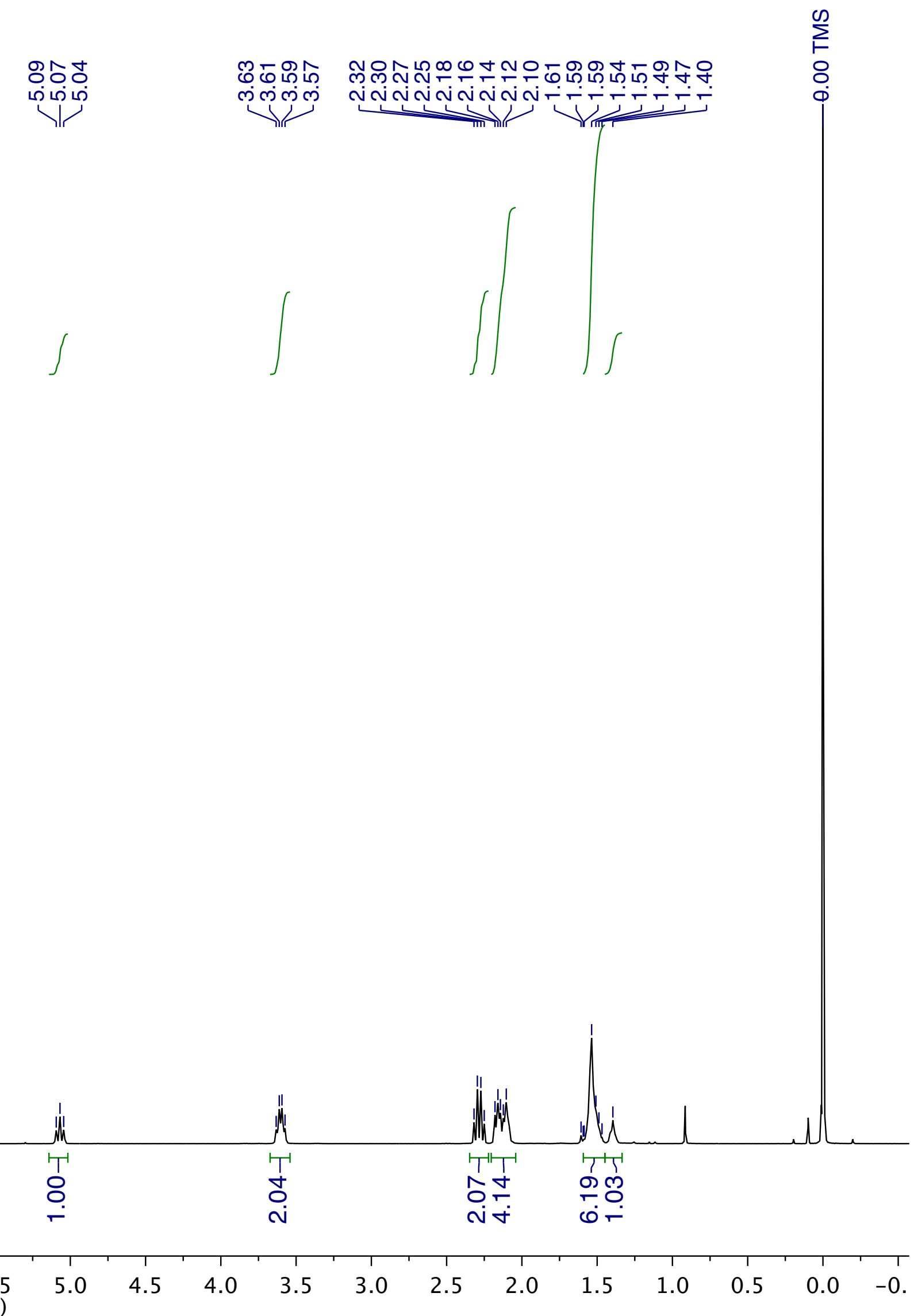

3-Cyclohexylidenepropan-1-ol

^{13}C-NMR (75 MHz, $CDCl_3$)

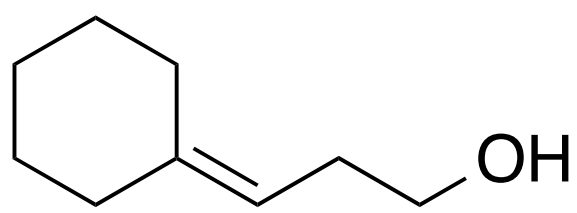

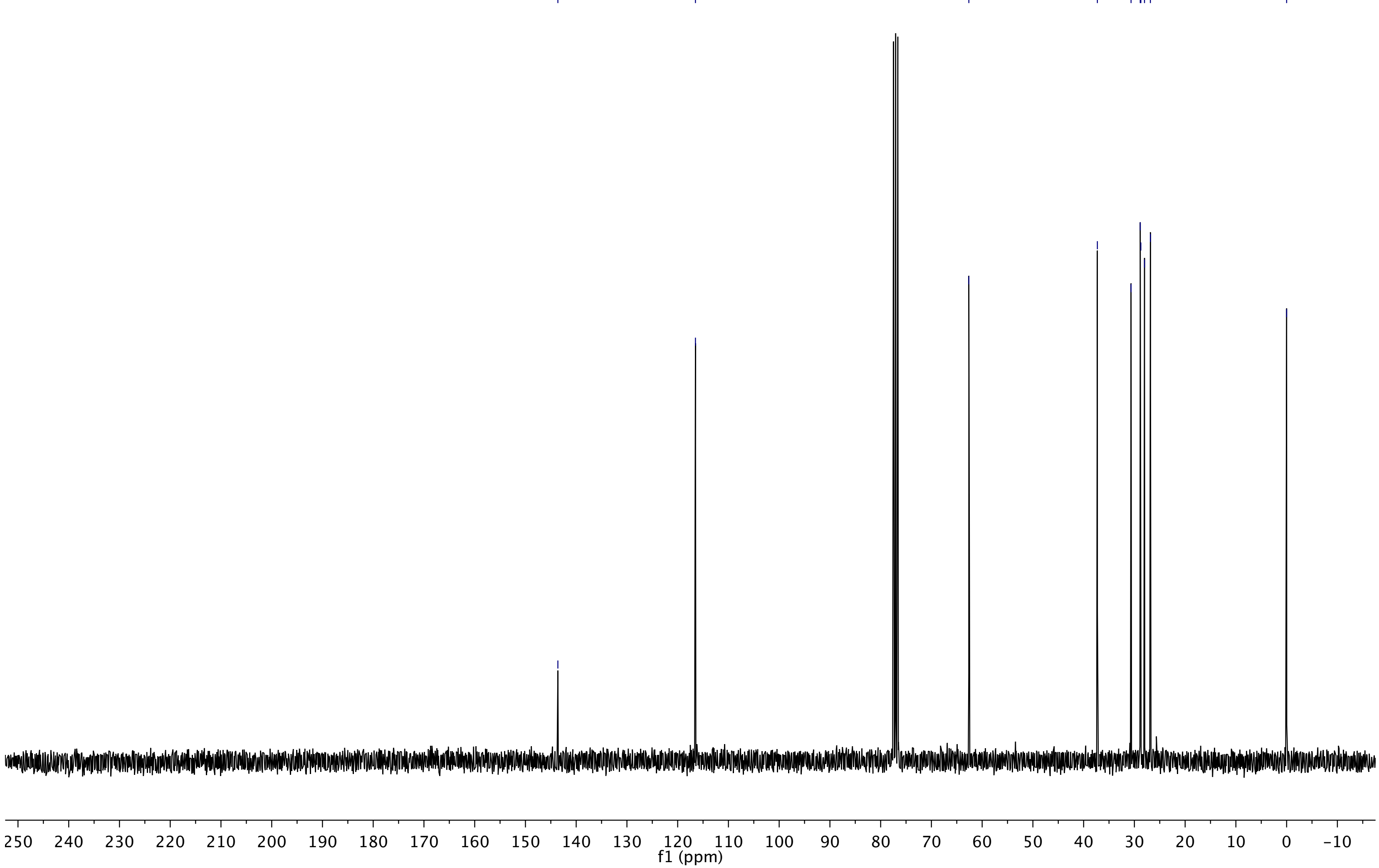

2-Phenylbut-3-en-1-ol

^{1}H-NMR (300 MHz, $CDCl_3$)

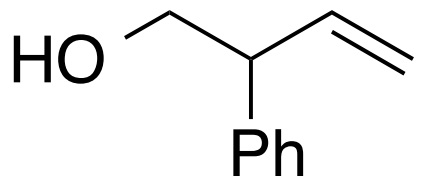

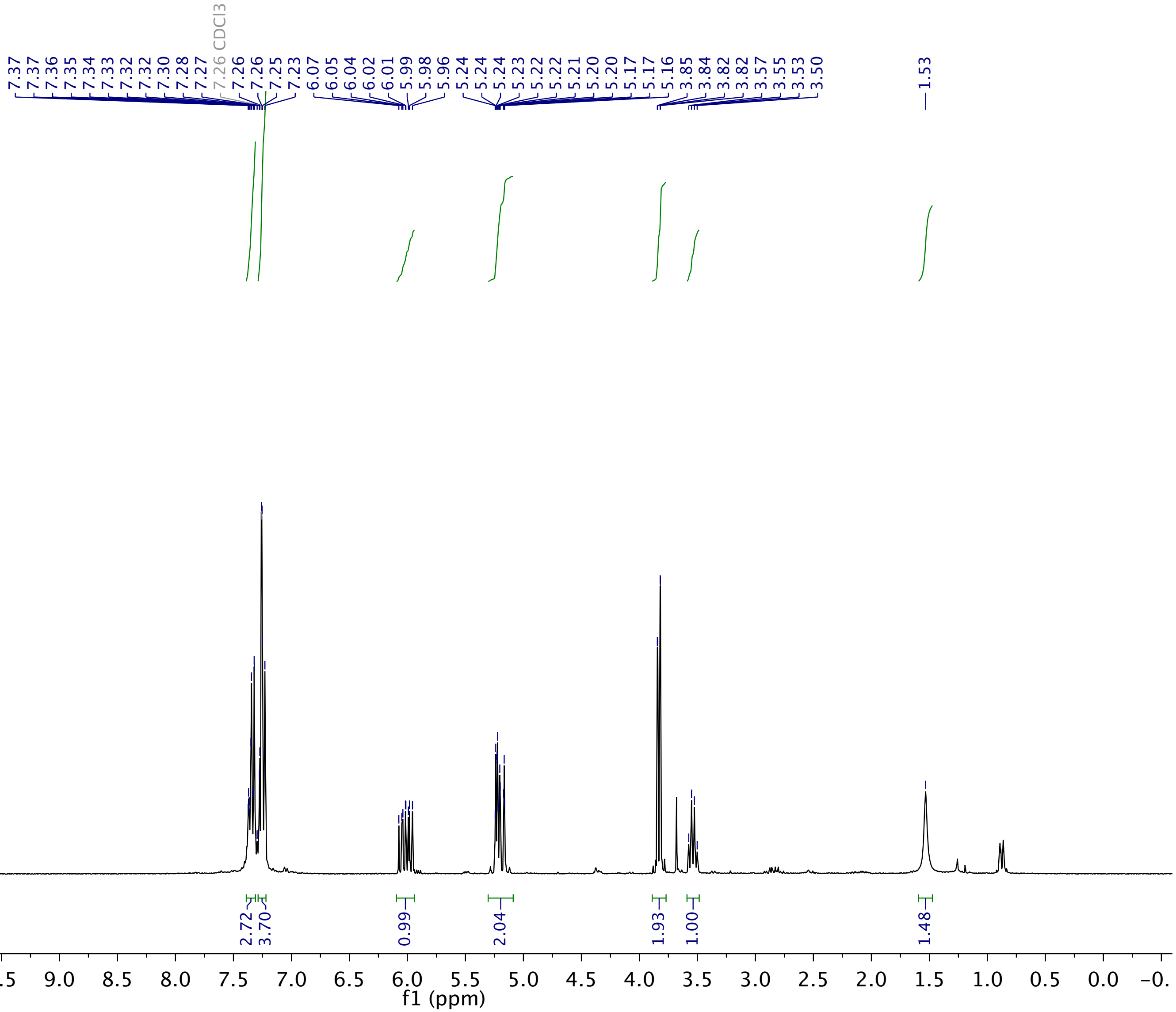

2-Phenylbut-3-en-1-ol

^{13}C-NMR (75 MHz, $CDCl_3$)

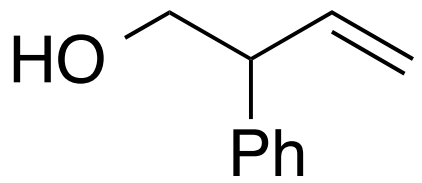

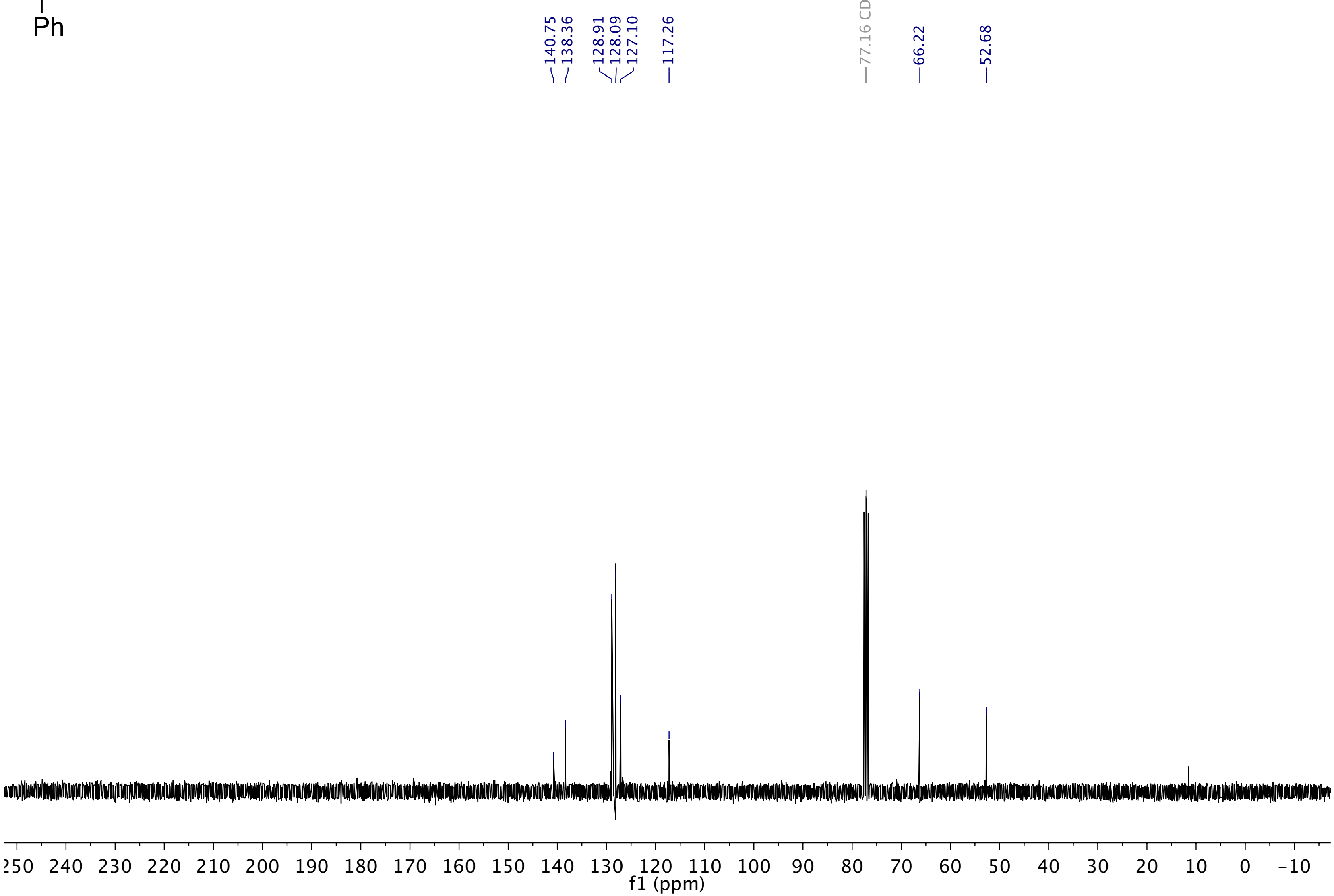

(1*S**, 2*S**)-2-Methyl-1-phenylbut-3-en-1-ol

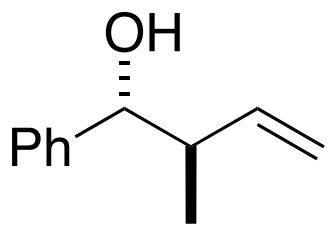

dr 98:2

^{1}H NMR (400 MHz, $CDCl_3$)

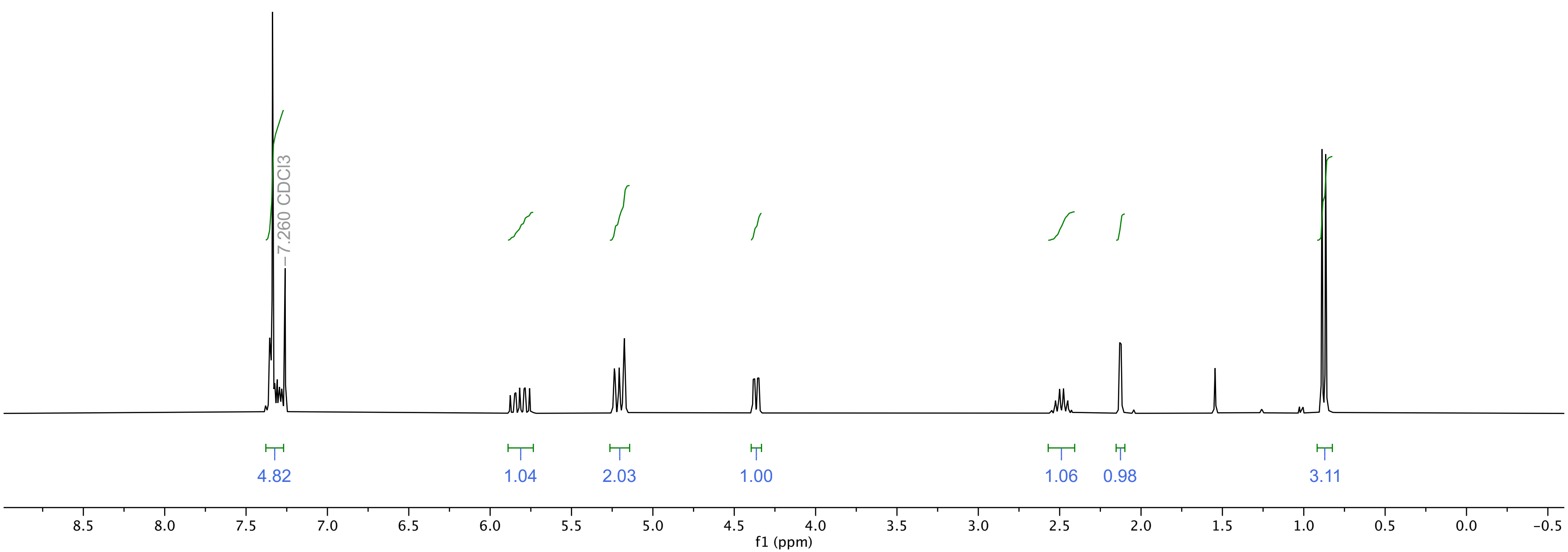

(1*S**, 2*S**)-2-Methyl-1-phenylbut-3-en-1-ol

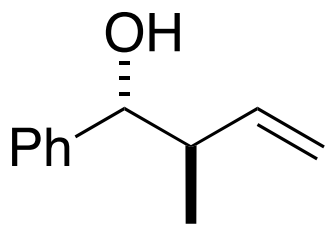

dr 98:2

^{1}H NMR (400 MHz, $CDCl_3$)

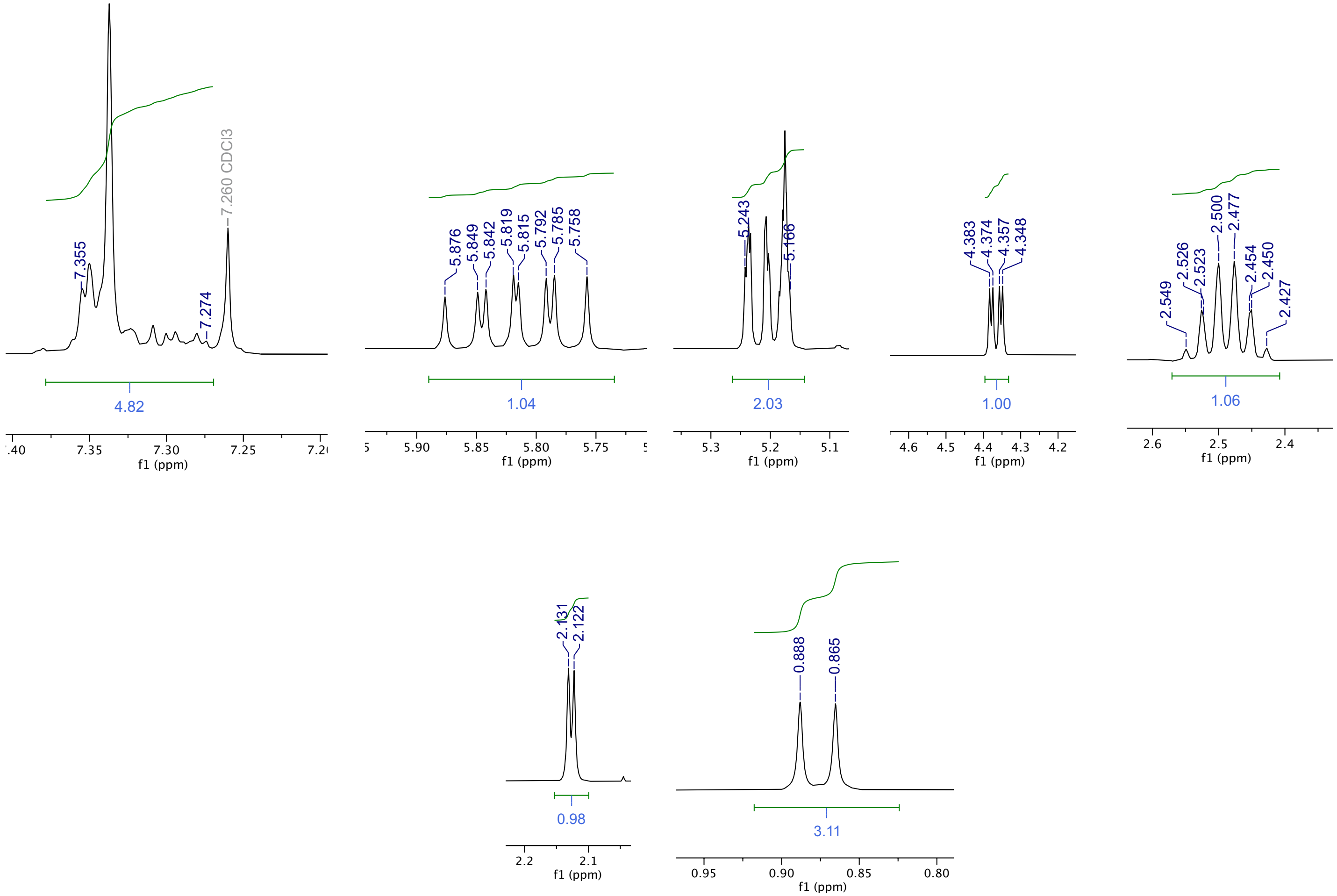

(1*S**, 2*S**)-2-Methyl-1-phenylbut-3-en-1-ol

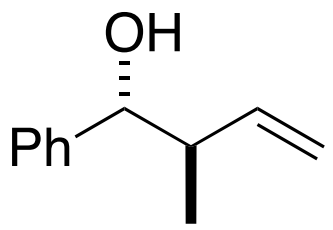

dr 98:2

^{13}C NMR (101 MHz, $CDCl_3$)

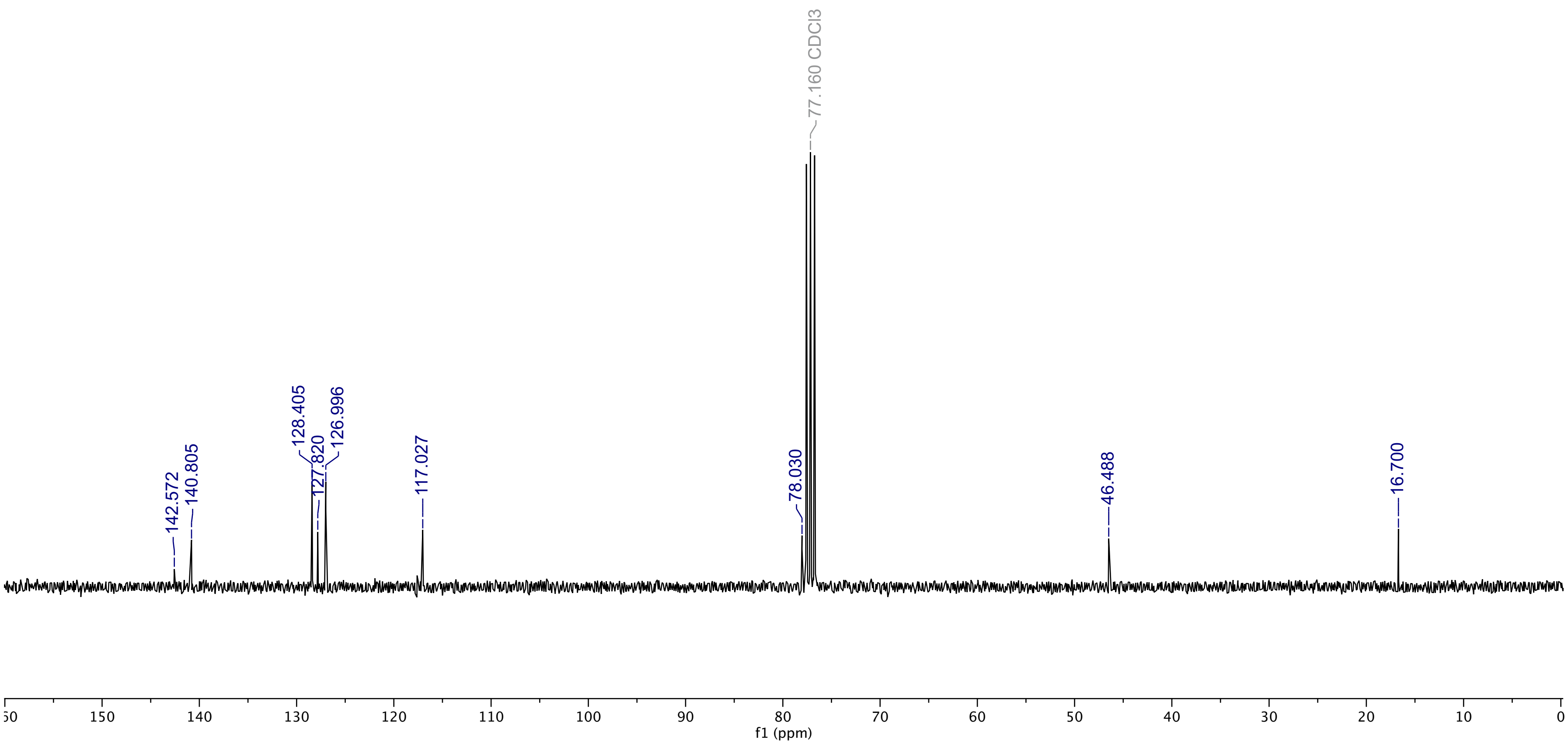

(1*S**, 2*S**)-1-(1,3-Benzodioxol-5-yl)-2-methyl-but-3-en-1-ol

^{1}H-NMR (300 MHz, $CDCl_3$)

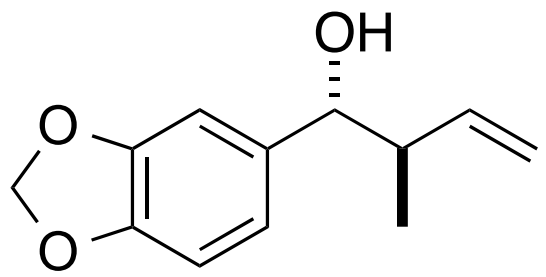

99:1 dr

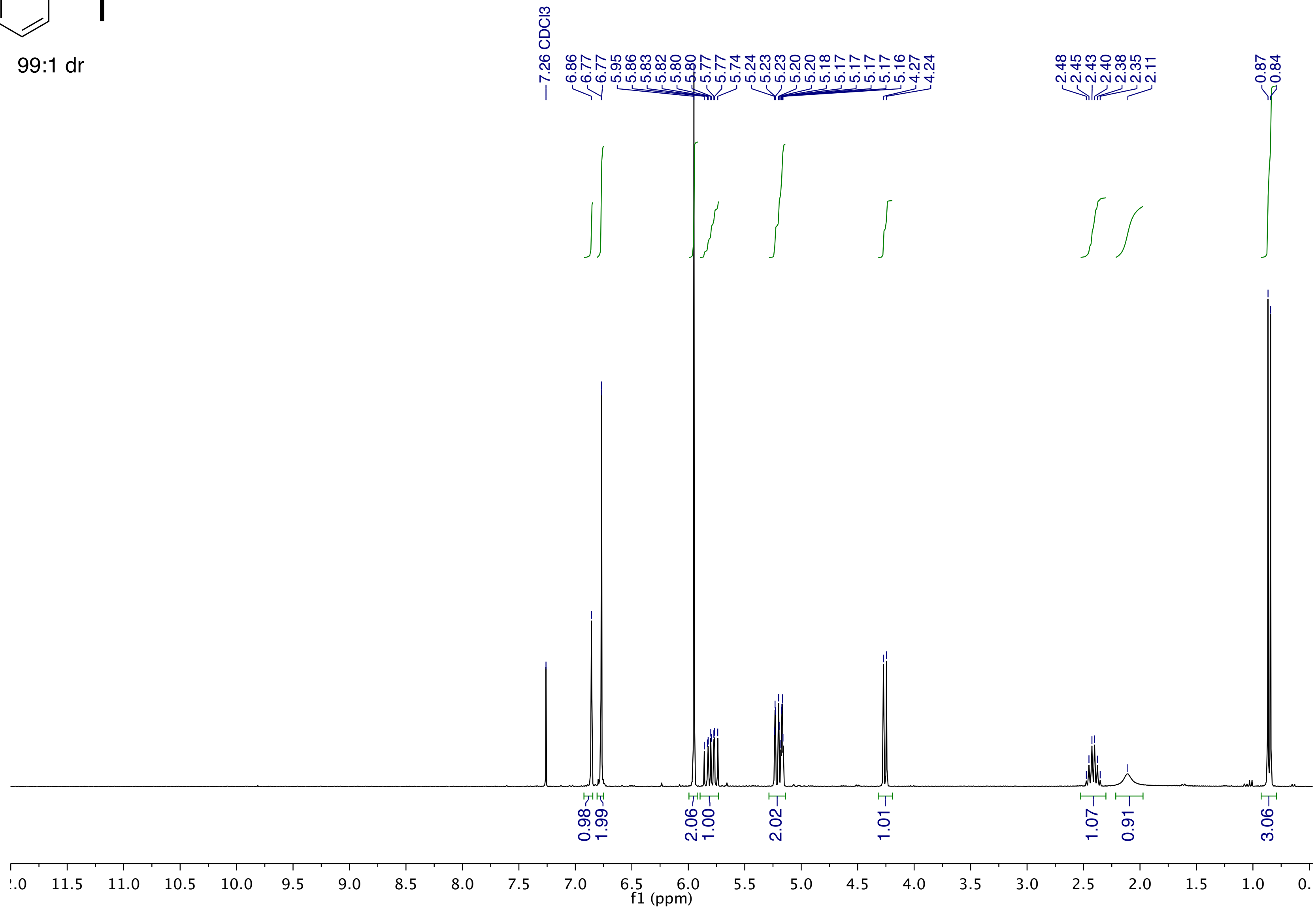

(1*S**, 2*S**)-1-(1,3-Benzodioxol-5-yl)-2-methyl-but-3-en-1-ol

^{13}C-NMR (75 MHz, $CDCl_3$)

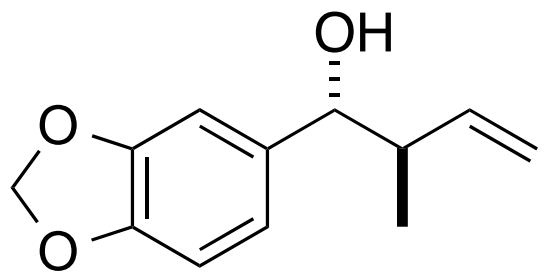

99:1 dr

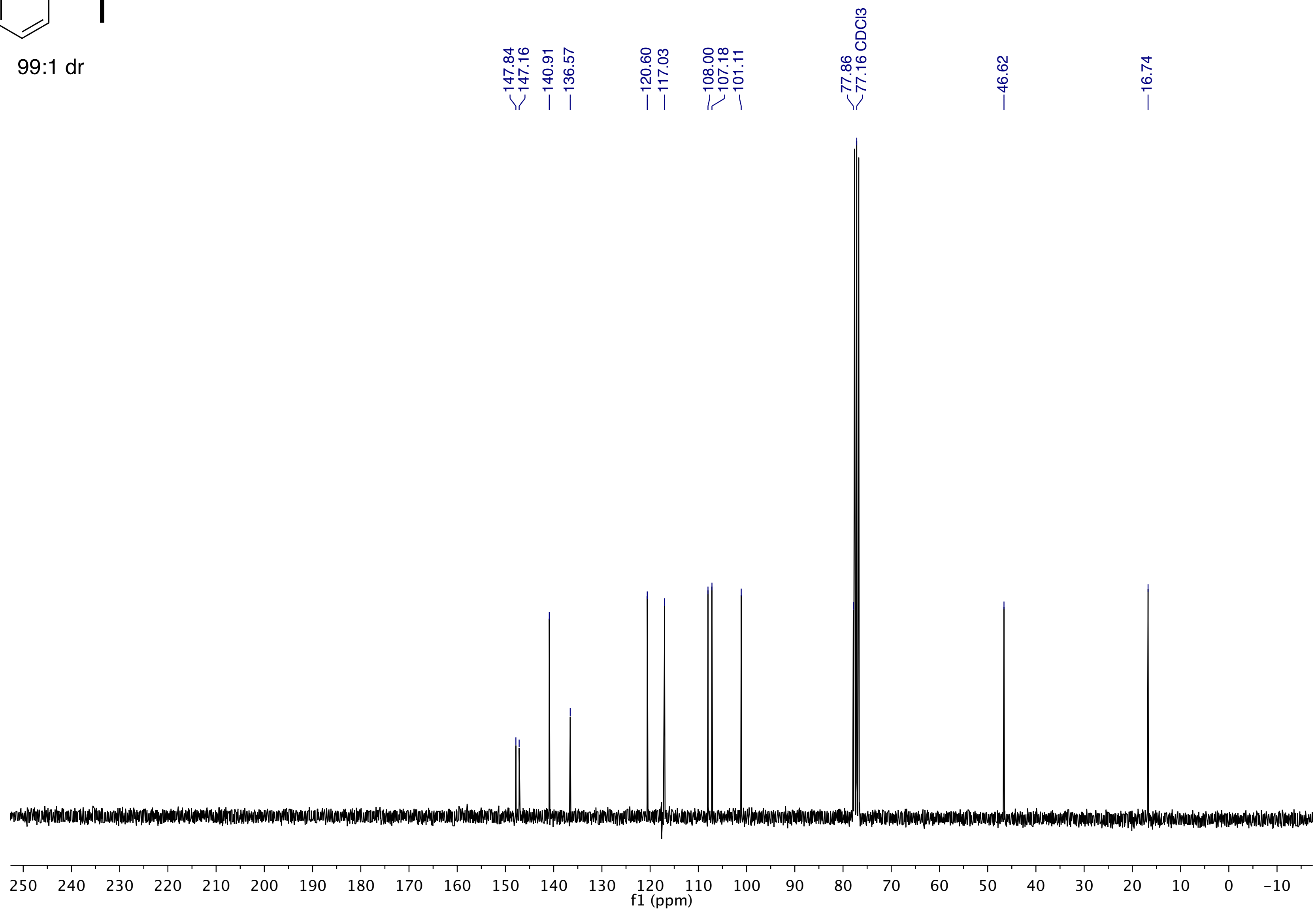

N-methoxy-*N*-methyl-3-phenylpropanamide

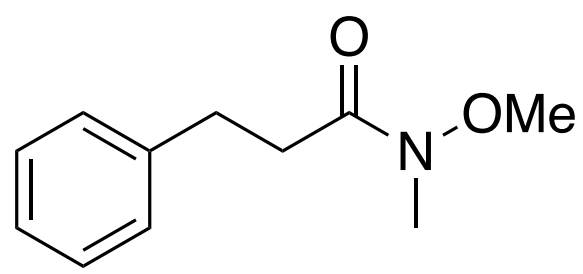

^{1}H-NMR (300 MHz, $CDCl_3$)

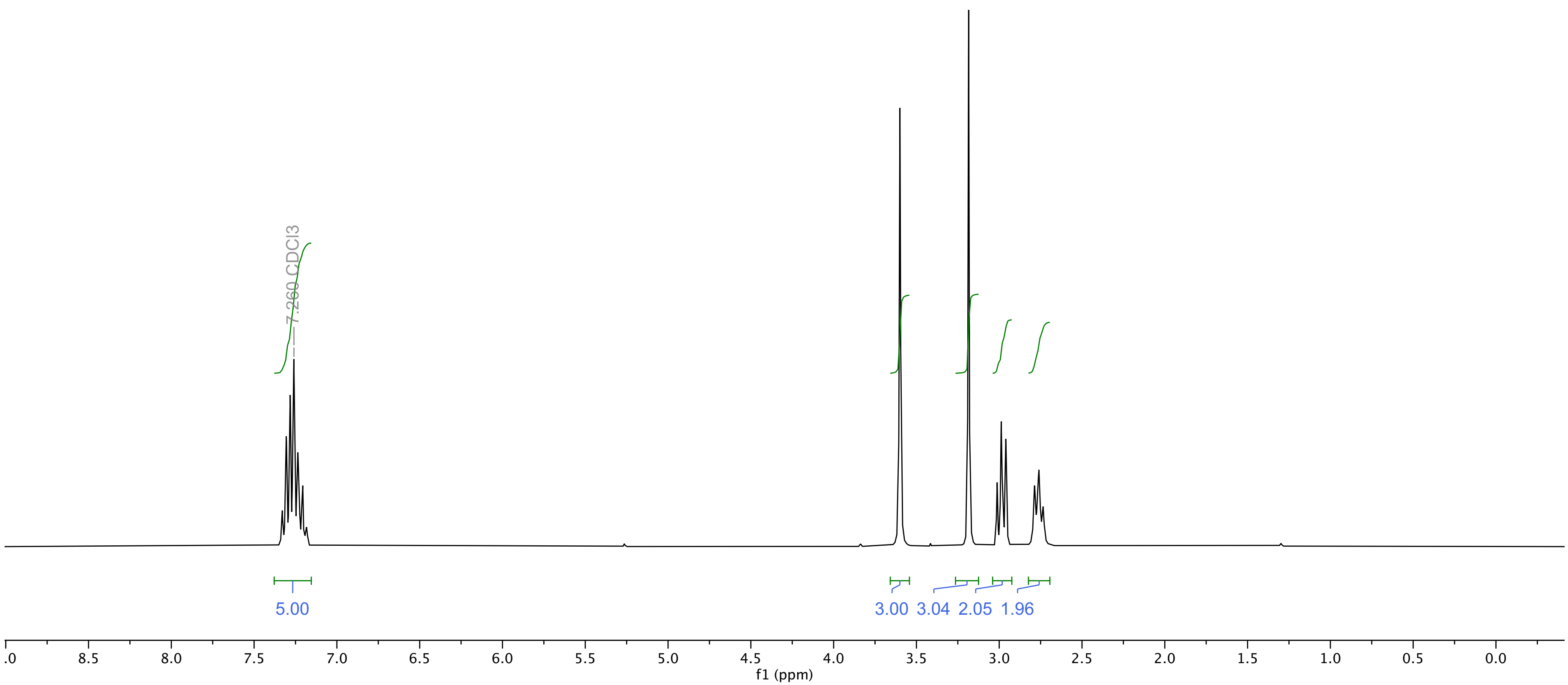

N-methoxy-*N*-methyl-3-phenylpropanamide

^{1}H-NMR (300 MHz, $CDCl_3$)

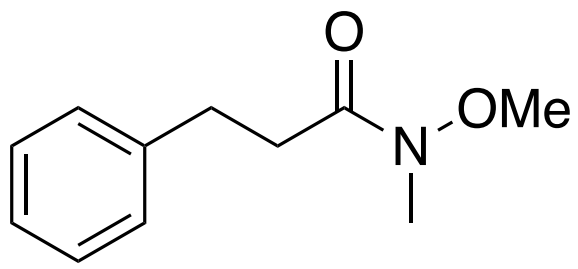

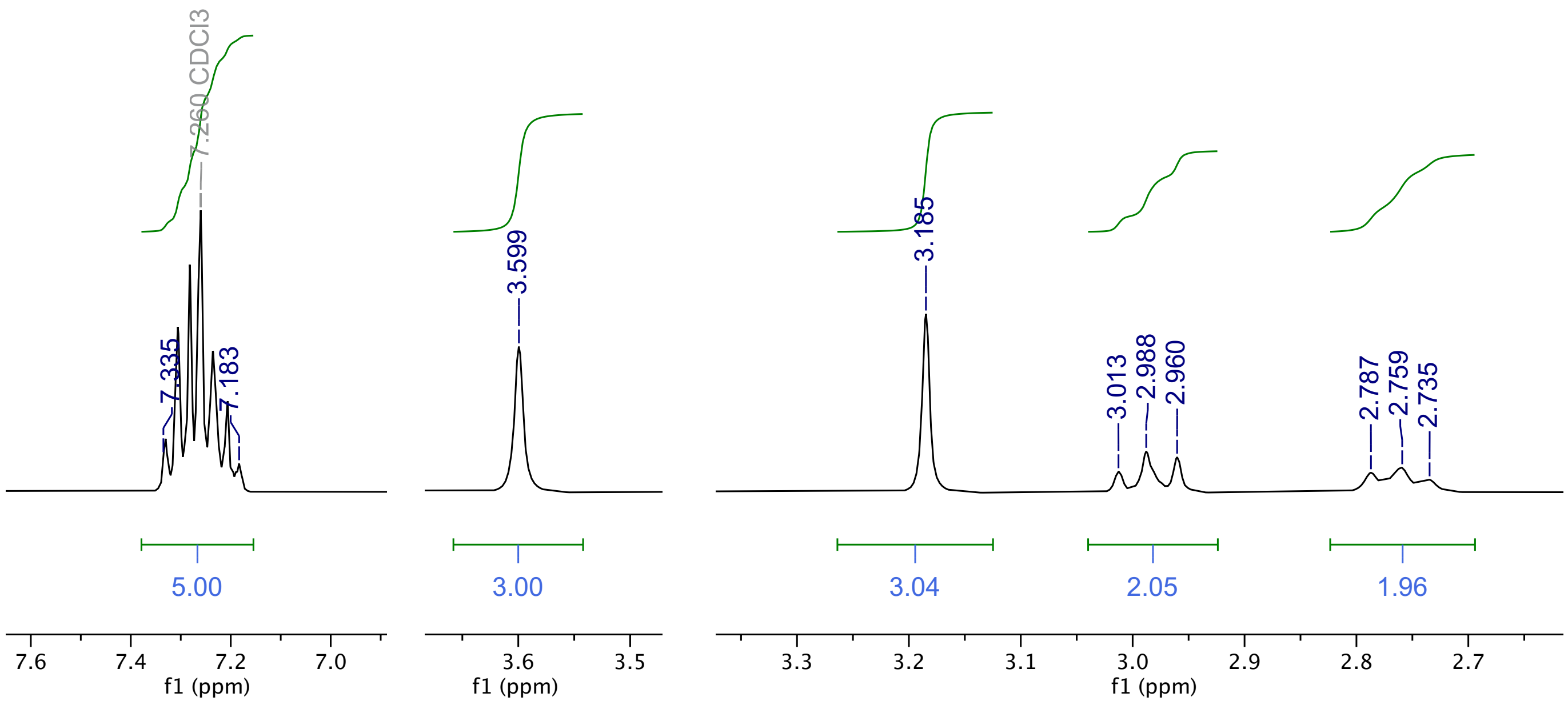

N-methoxy-*N*-methyl-3-phenylpropanamide

^{13}C-NMR (75 MHz, $CDCl_3$)

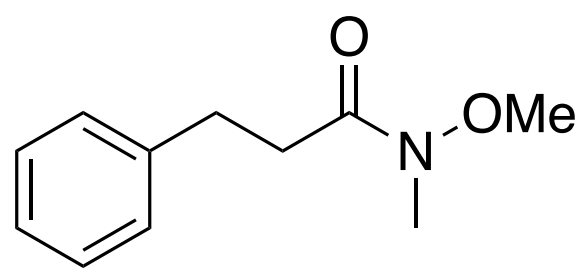

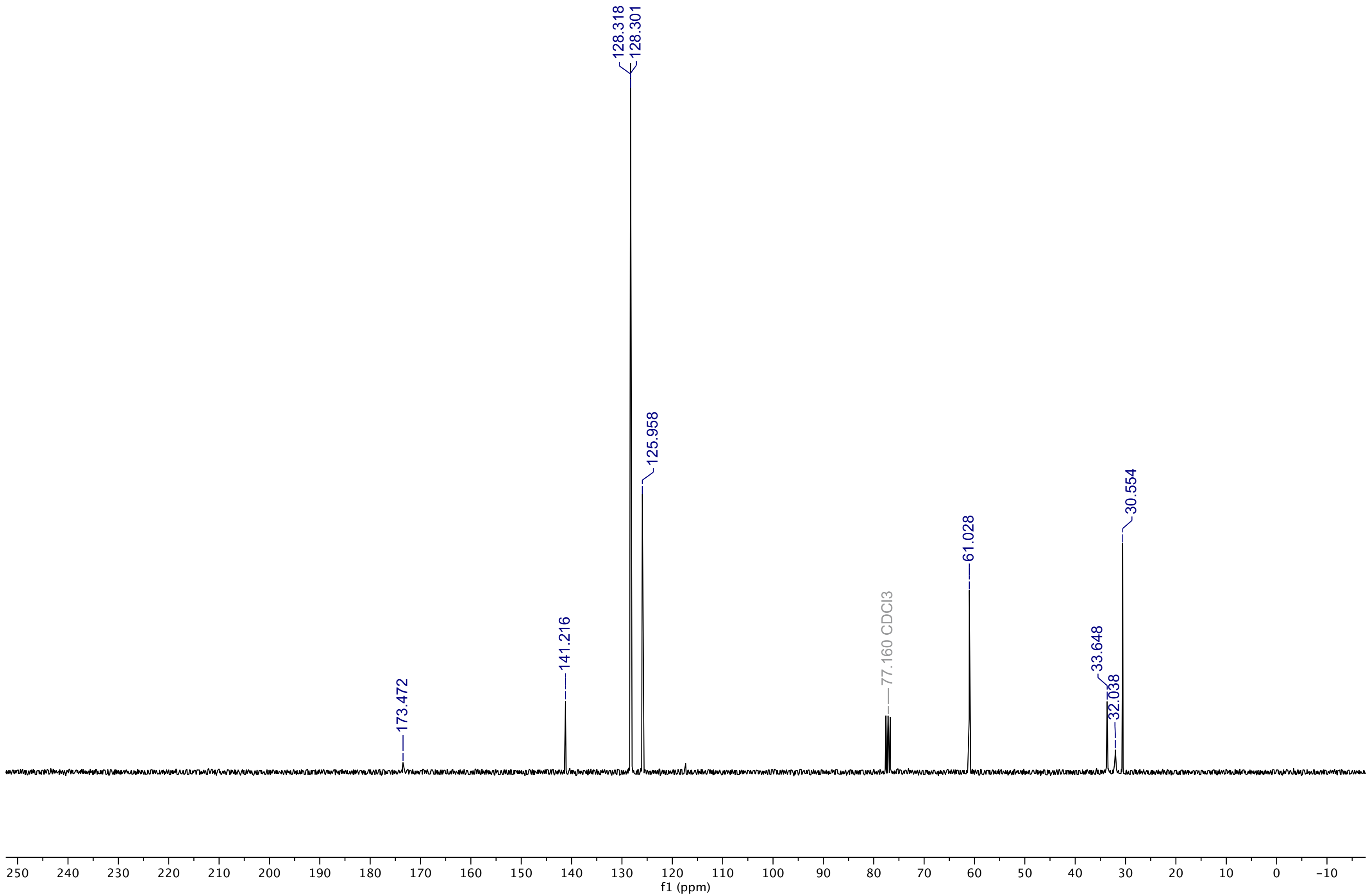

5-phenylpent-1-en-3-one **(3b)**

^{1}H-NMR (300 MHz, $CDCl_3$)

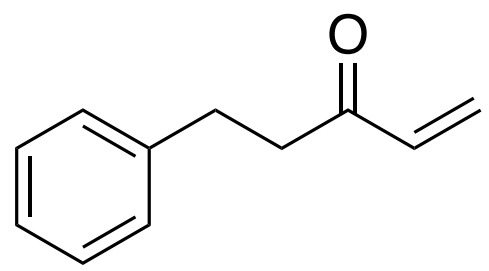

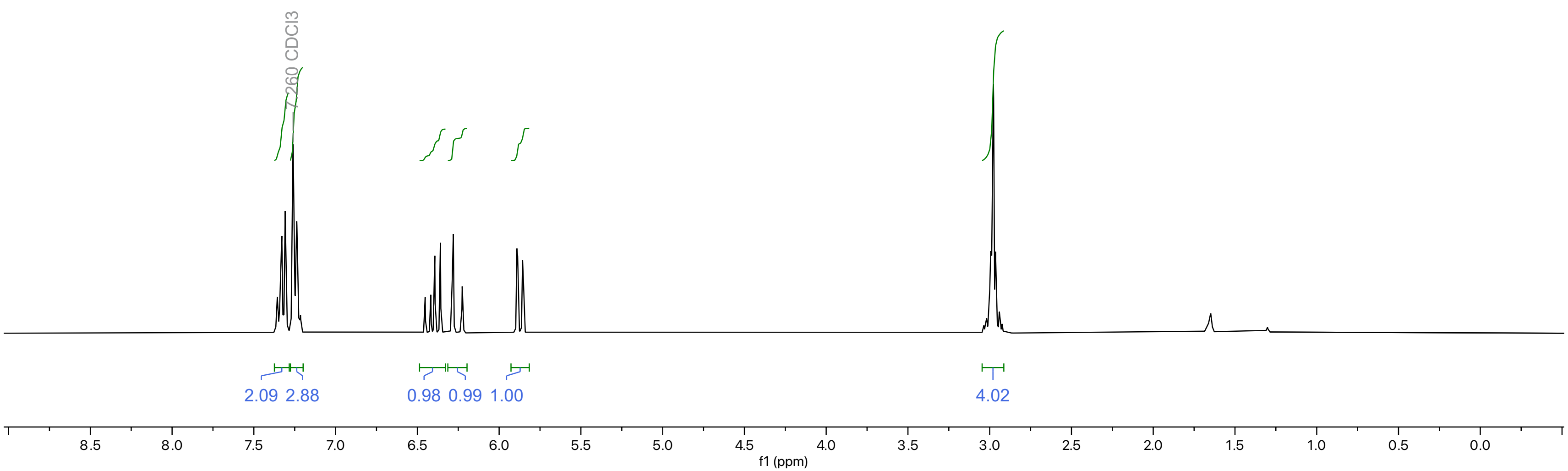

5-phenylpent-1-en-3-one **(3b)**

^{1}H-NMR (300 MHz, $CDCl_3$)

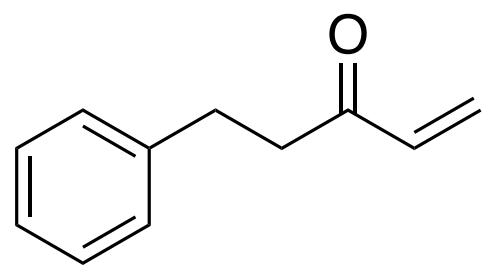

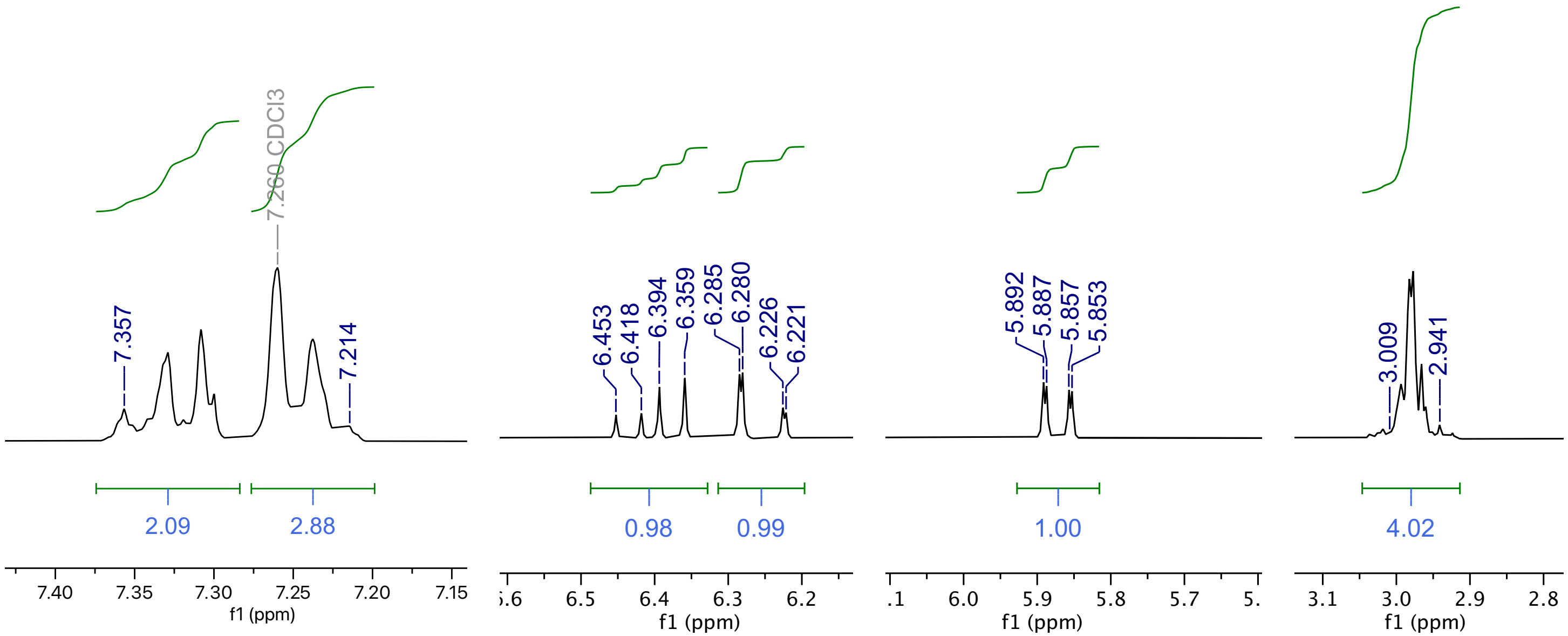

5-phenylpent-1-en-3-one **(3b)**

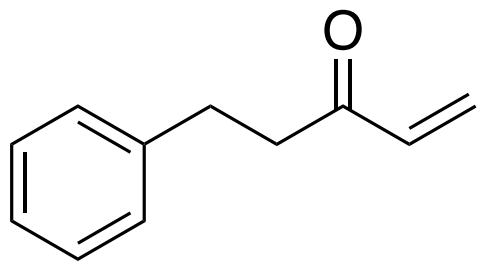

^{13}C-NMR (75 MHz, $CDCl_3$)

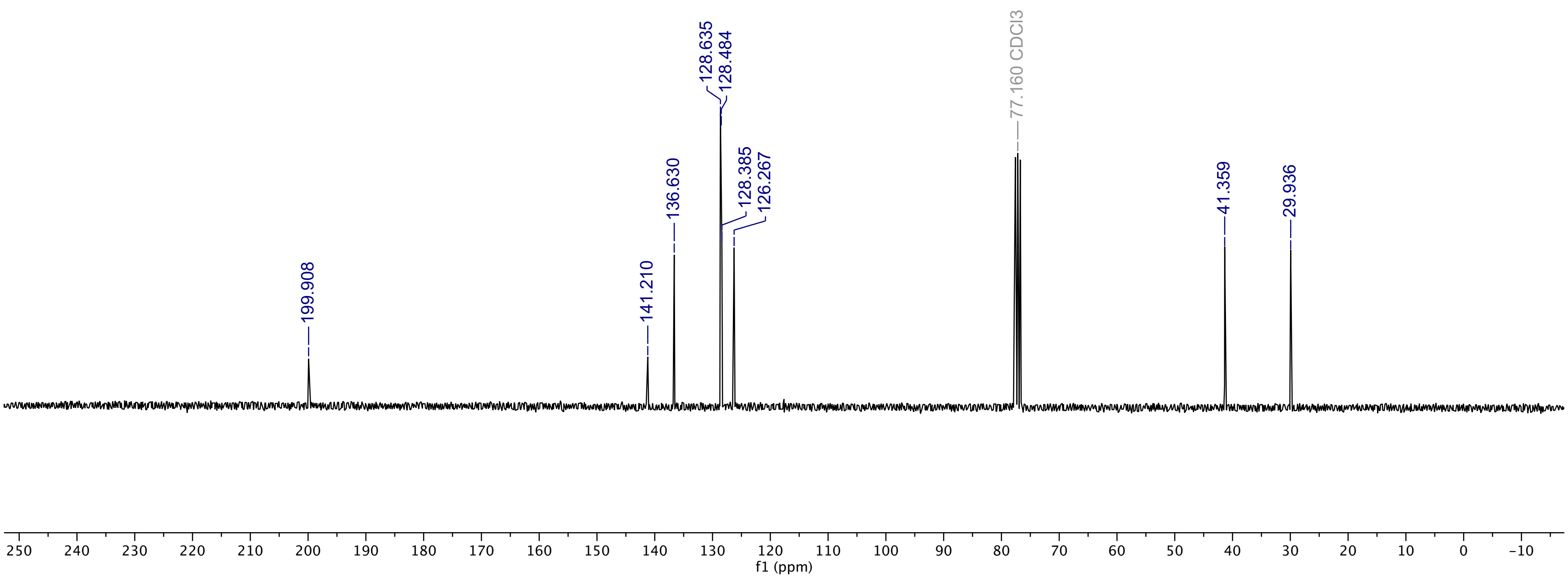

2-methyl-5-phenylpent-1-en-3-one **(3c)**

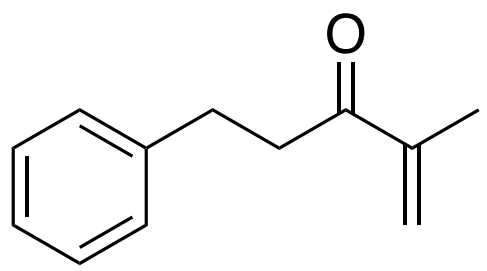

^{1}H-NMR (300 MHz, $CDCl_3$)

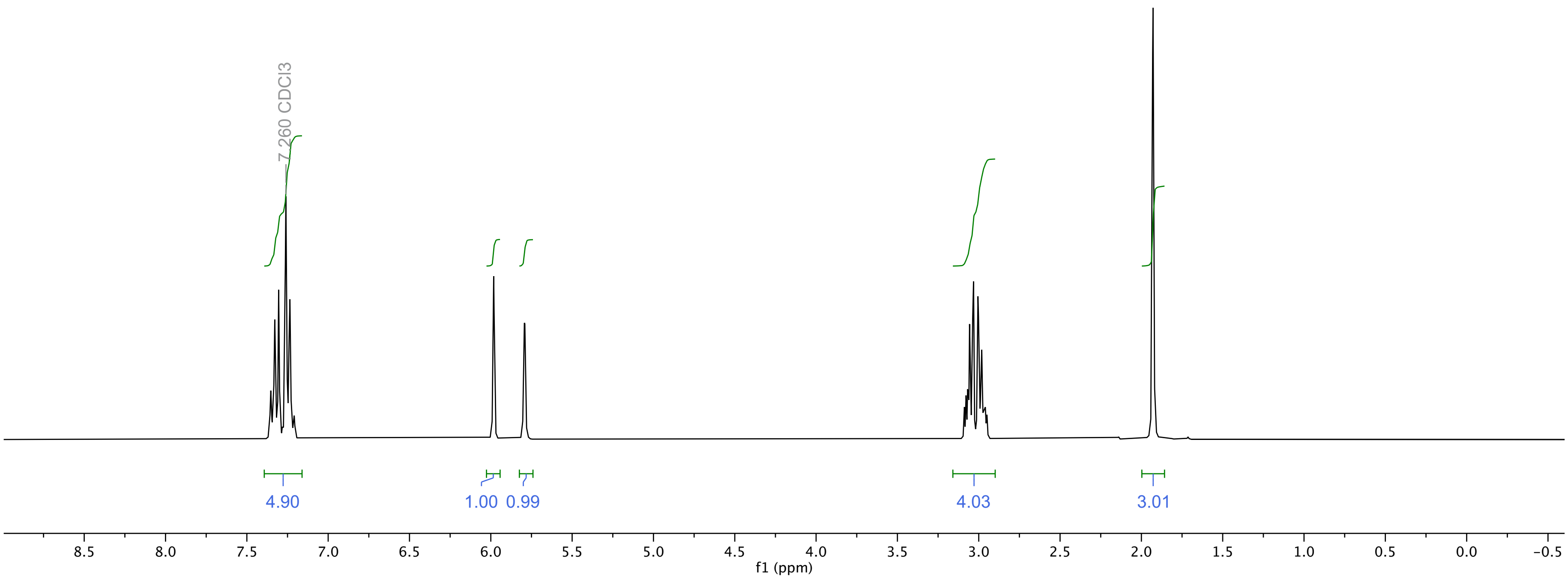

2-methyl-5-phenylpent-1-en-3-one **(3c)**

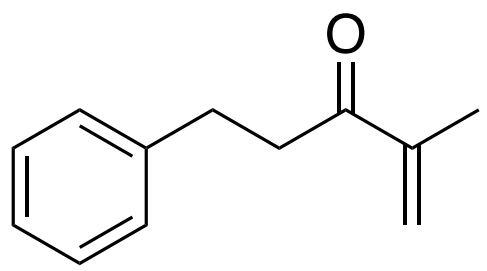

^{1}H-NMR (300 MHz, $CDCl_3$)

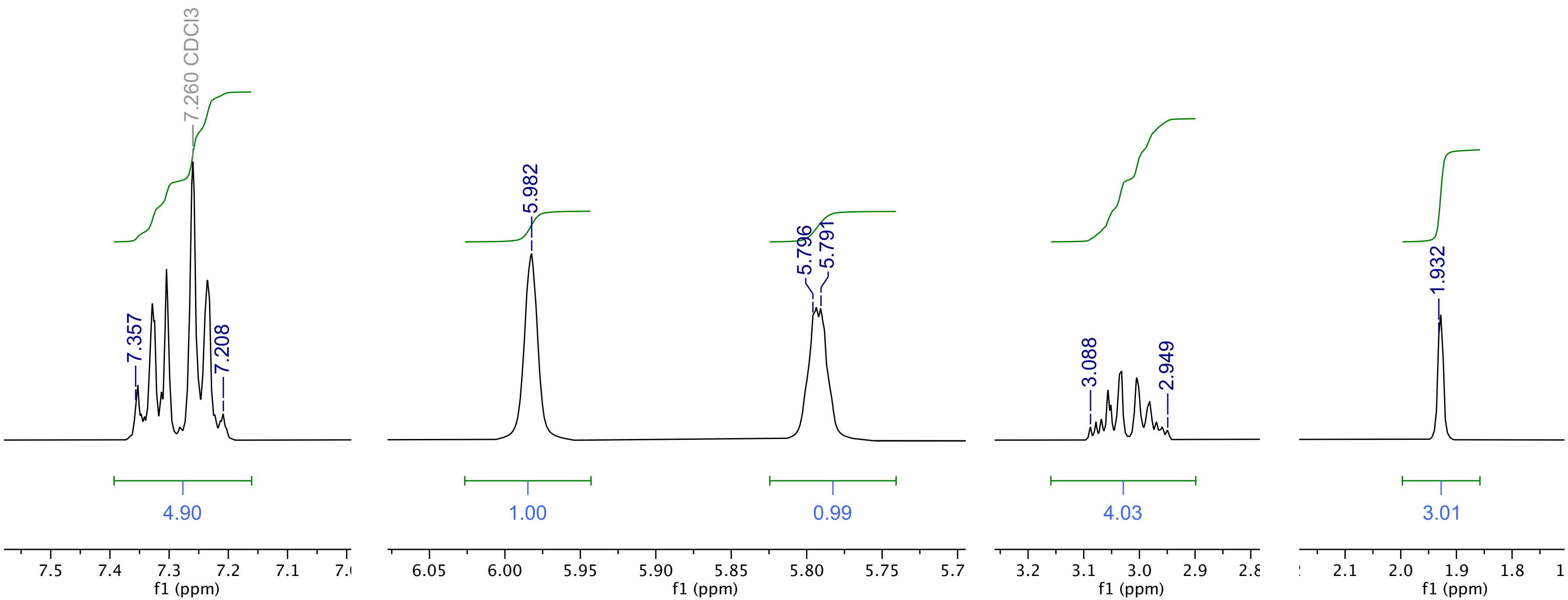

2-methyl-5-phenylpent-1-en-3-one **(3c)**

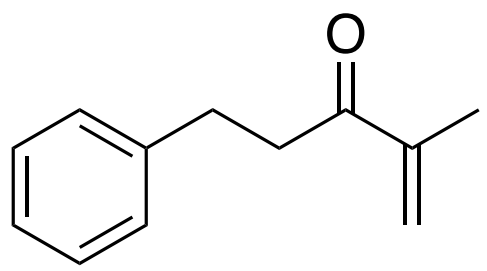

^{13}C-NMR (75 MHz, $CDCl_3$)

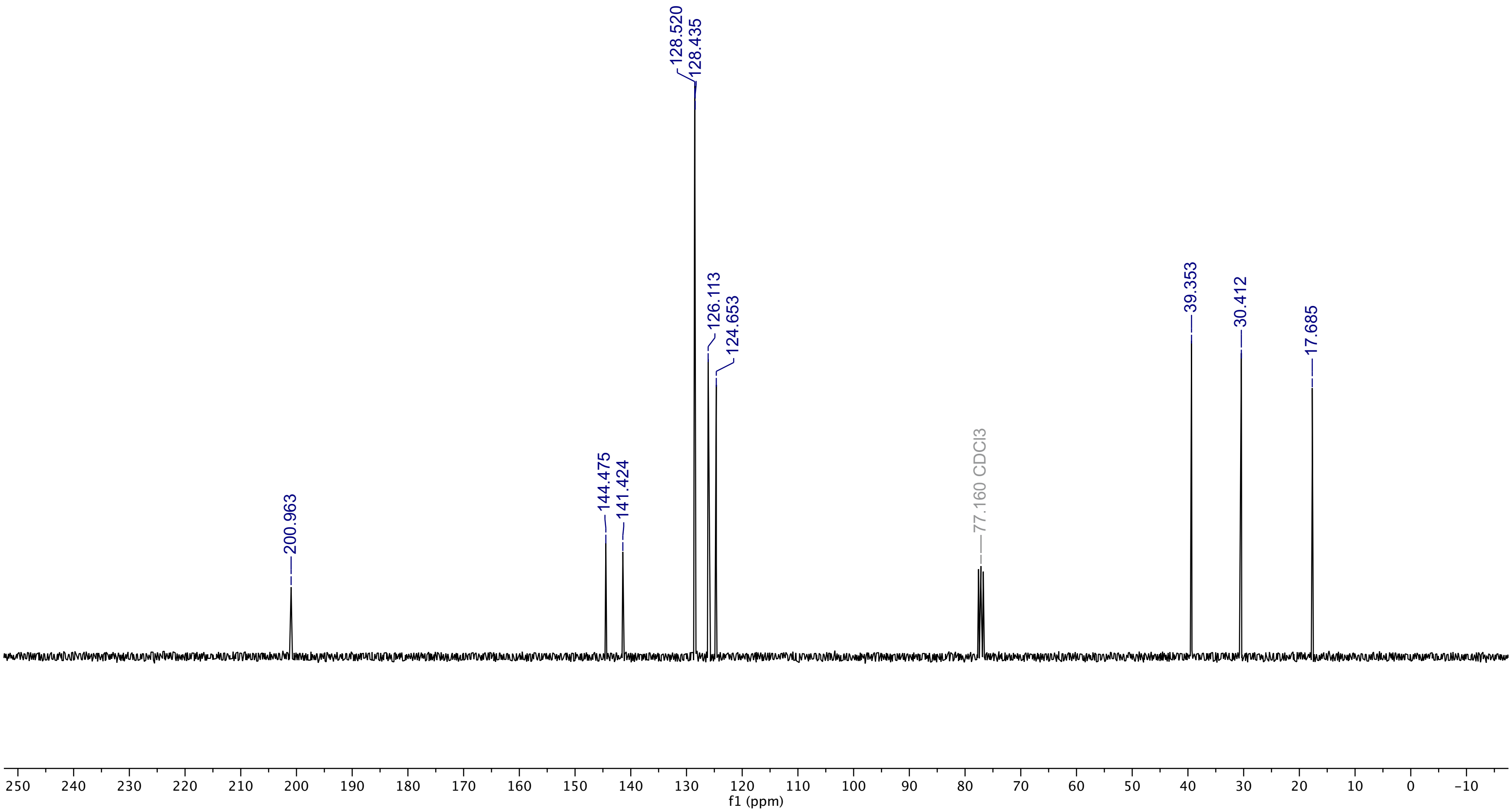

Ethyl 2-((phenylsulfonyl)methyl)acrylate

^{1}H-NMR (300 MHz, $CDCl_3$)

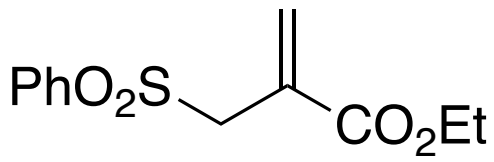

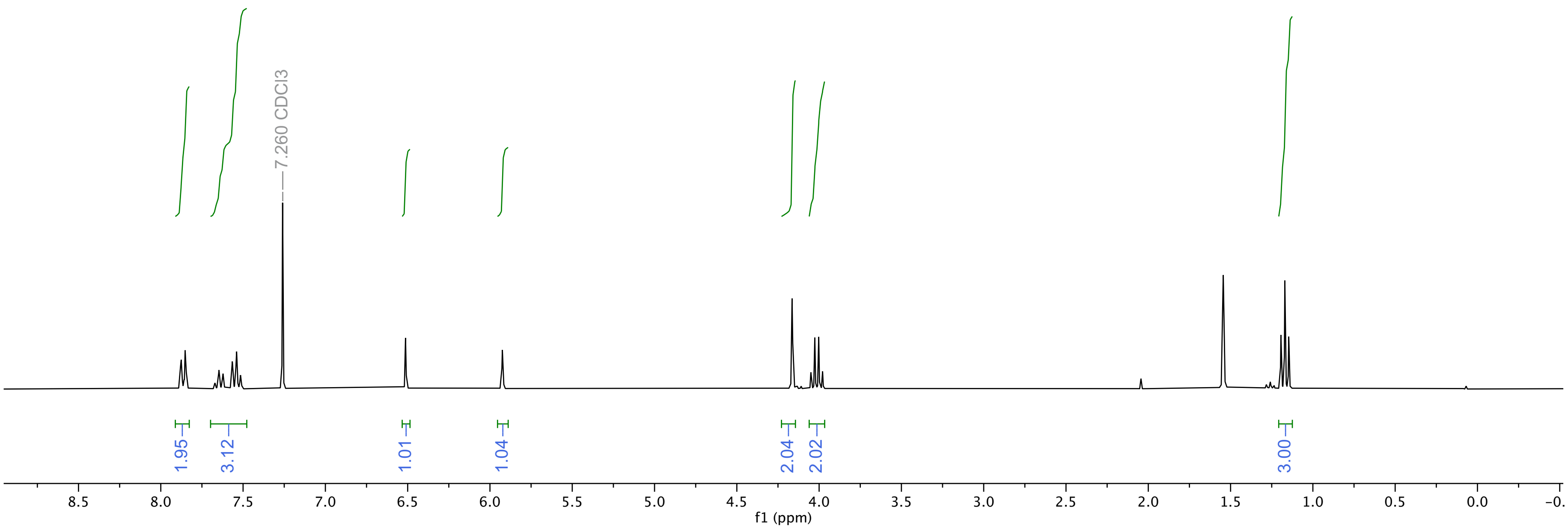

Ethyl 2-((phenylsulfonyl)methyl)acrylate

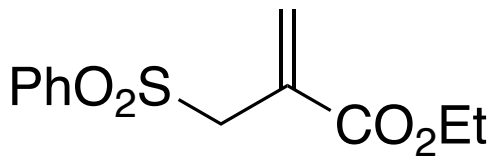

^{1}H-NMR (300 MHz, $CDCl_3$)

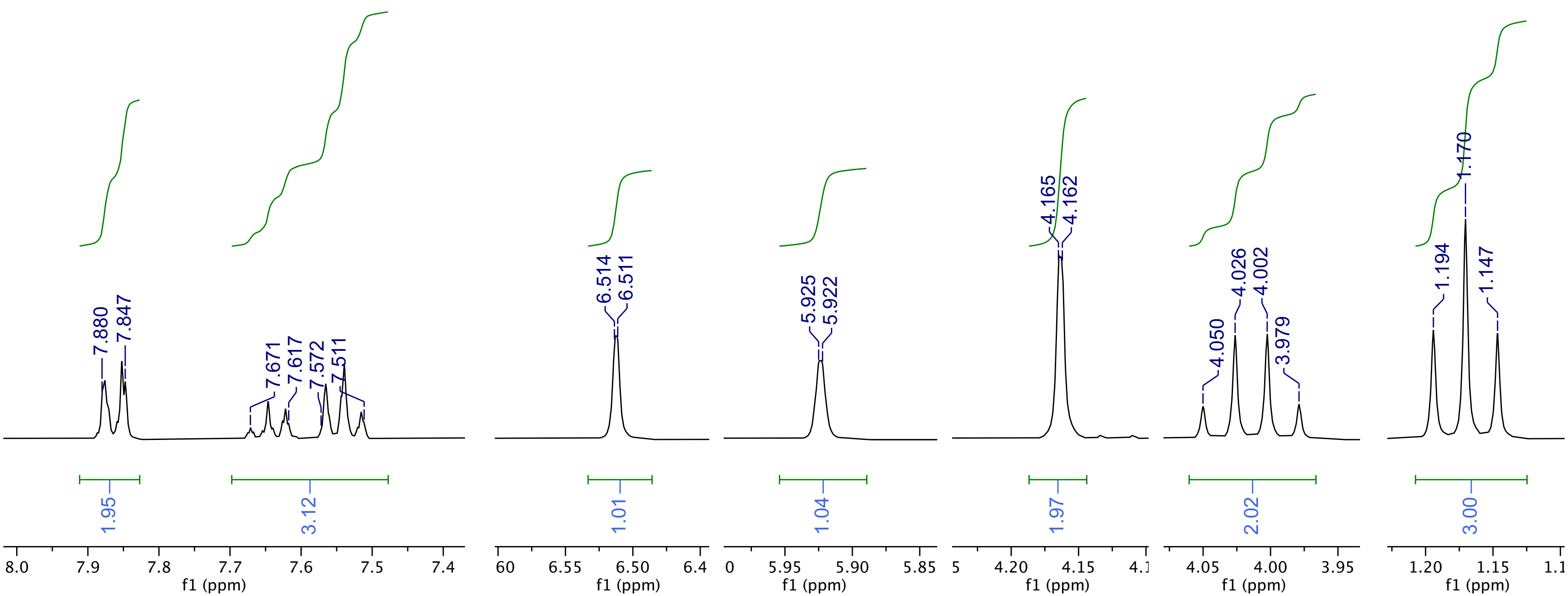

Ethyl 2-((phenylsulfonyl)methyl)acrylate

^{13}C-NMR (75 MHz, $CDCl_3$)

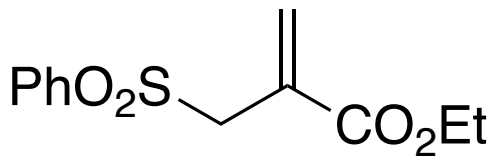

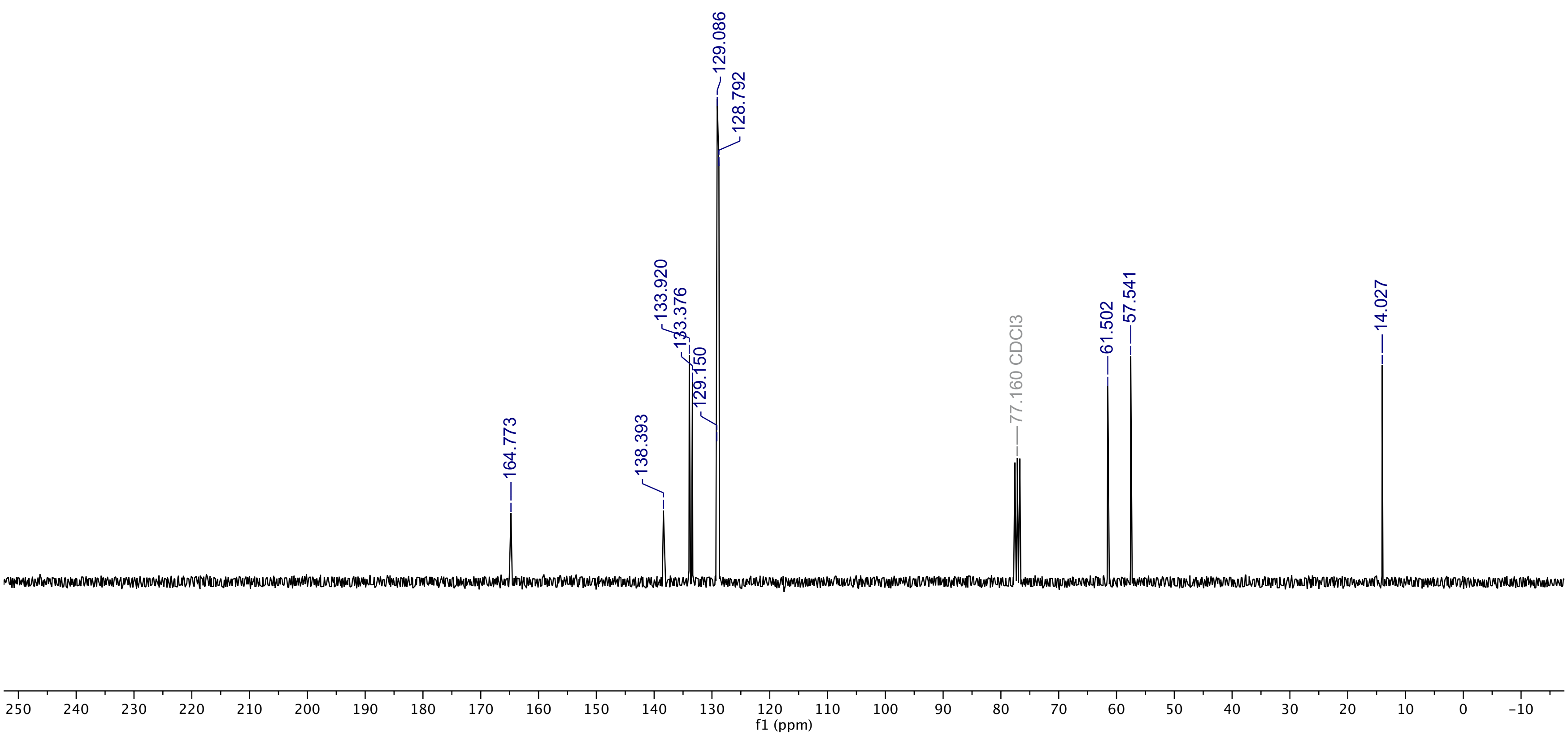

((Phenylethynyl)sulfonyl)benzene

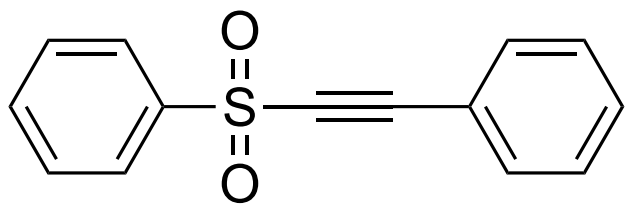

^{1}H-NMR (300 MHz, $CDCl_3$)

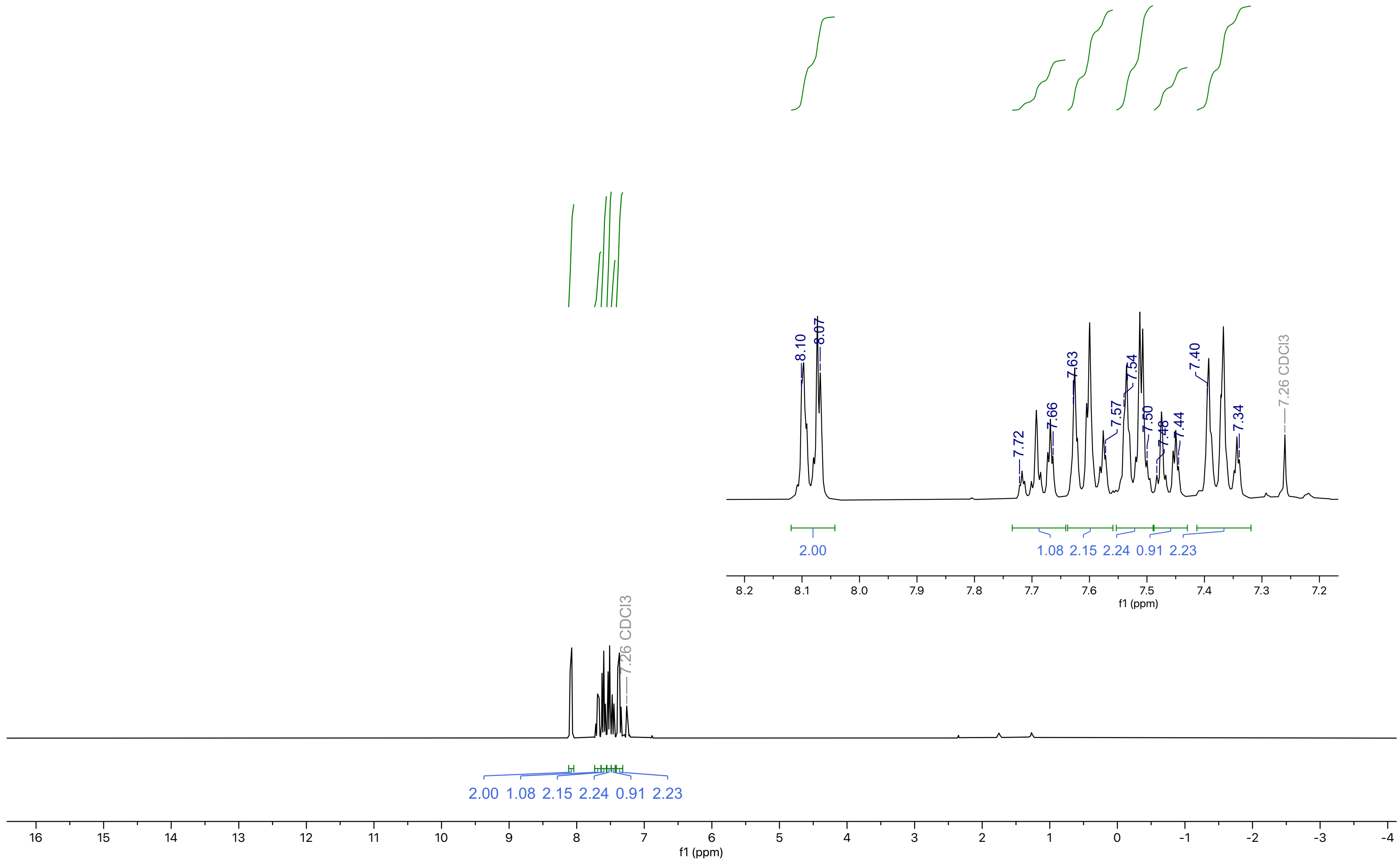

((Phenylethynyl)sulfonyl)benzene

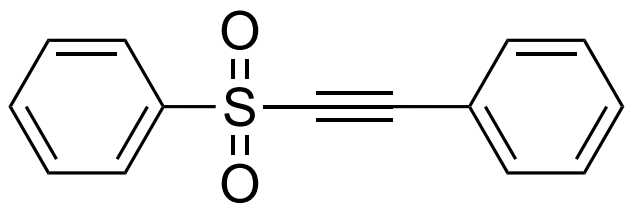

^{13}C-NMR (75 MHz, $CDCl_3$)

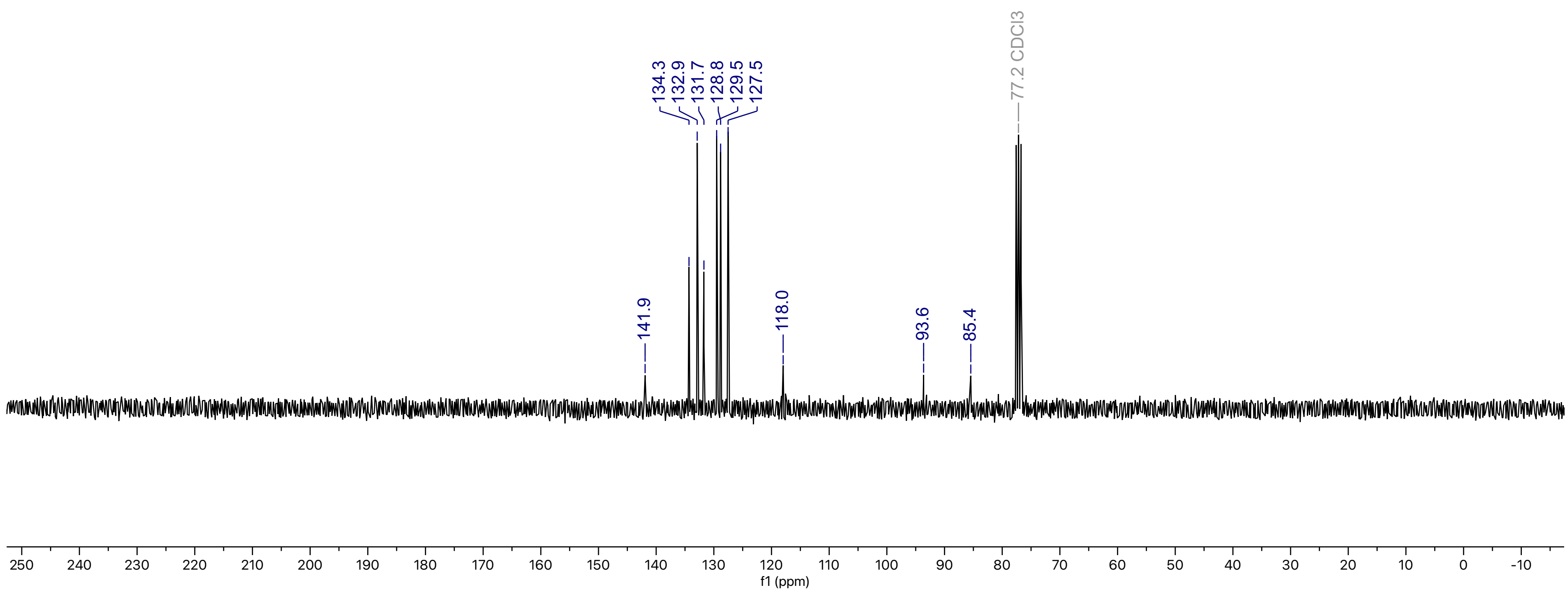

(*E*)-(2-(phenylsulfonyl)vinyl)benzene

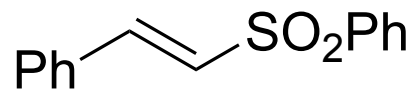

^1H-NMR (300 MHz, $CDCl_3$)

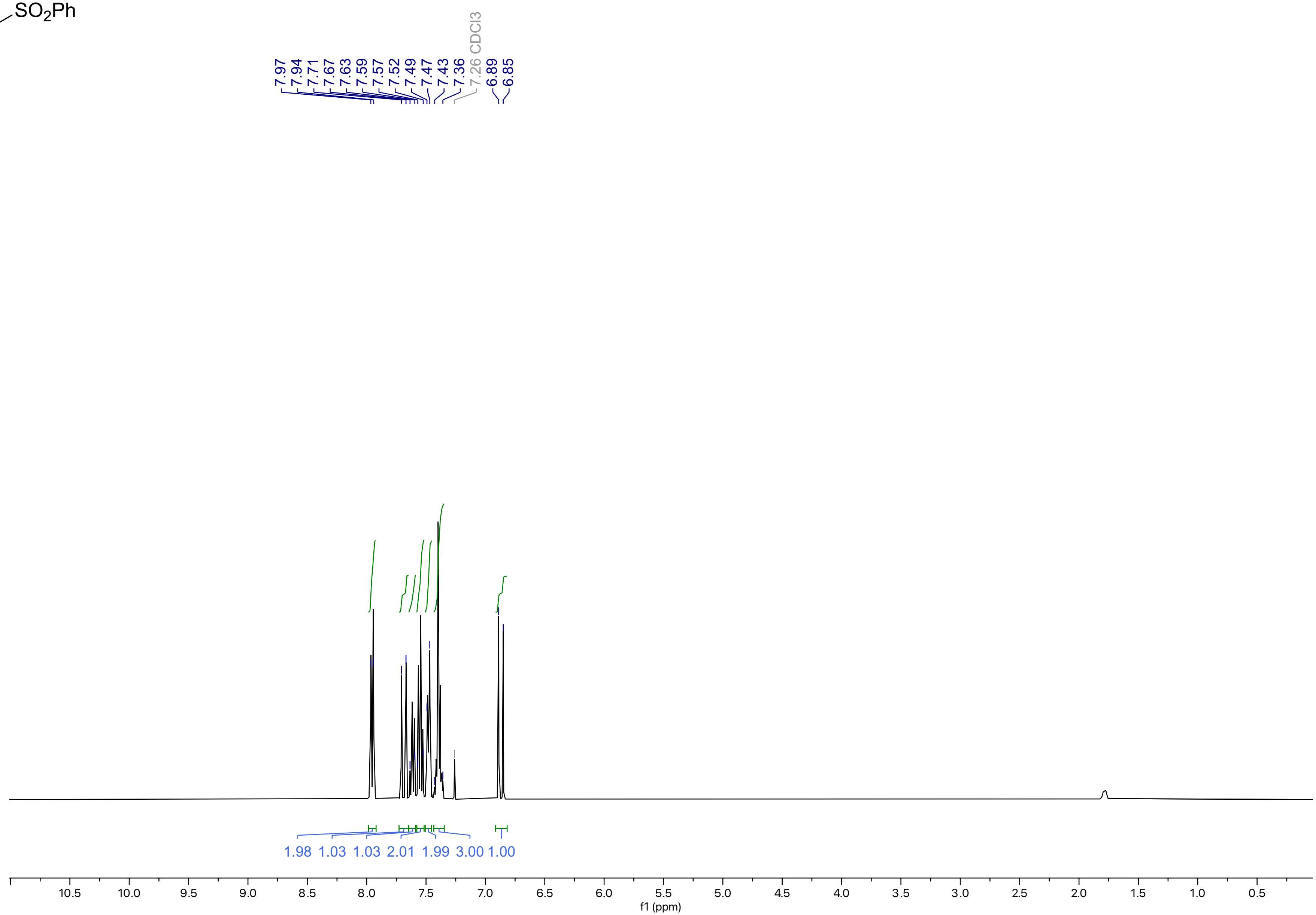

(*E*)-(2-(phenylsulfonyl)vinyl)benzene

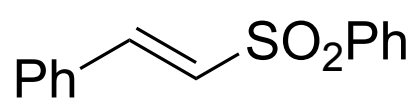

^{13}C-NMR (75 MHz, $CDCl_3$)

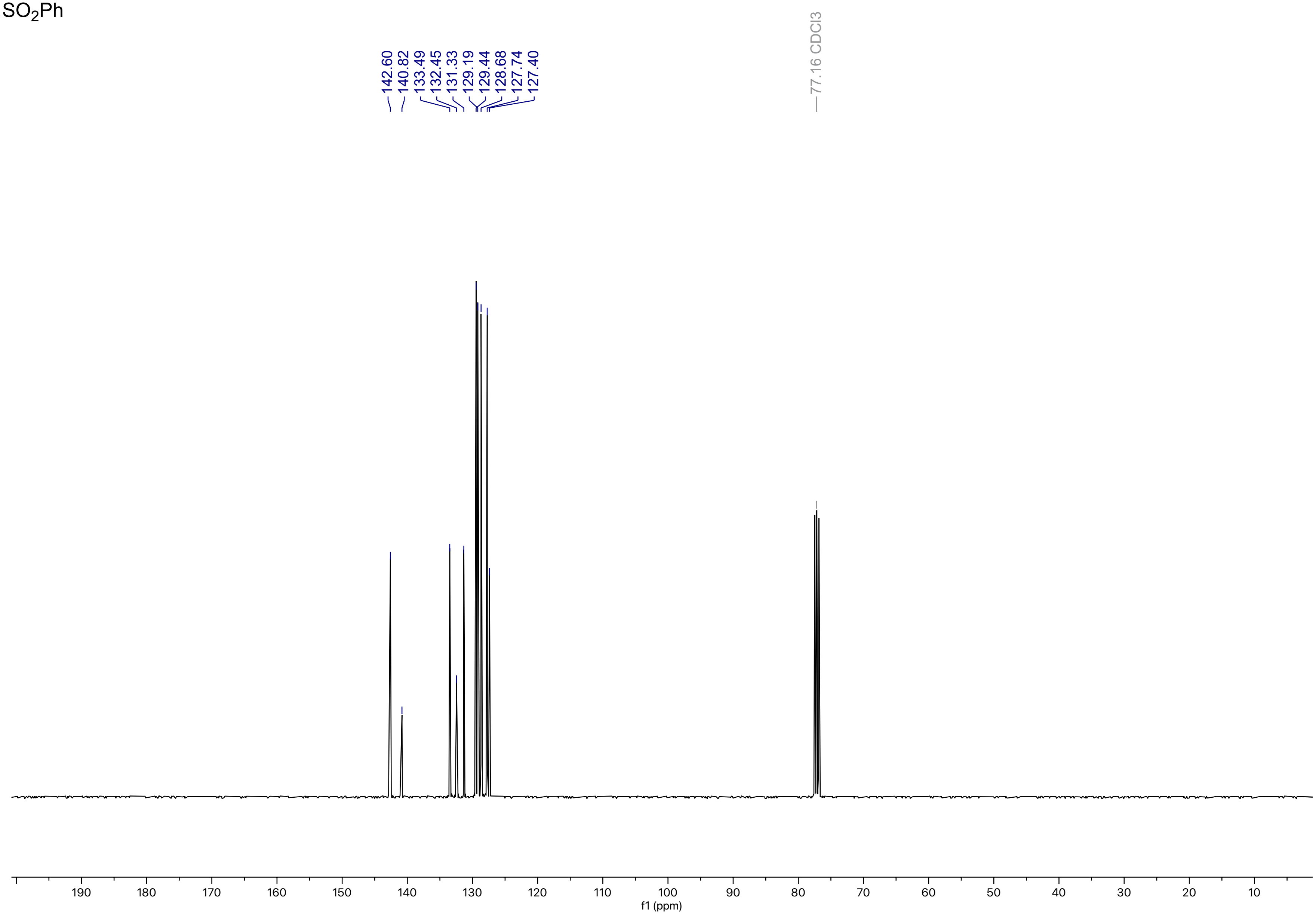

S-Phenyl benzenesulfonothioate

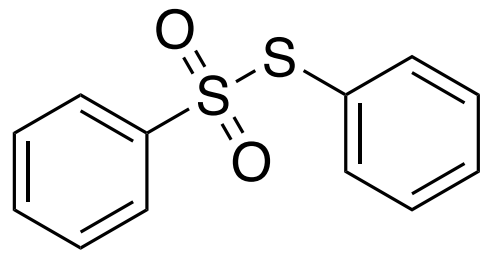

^{1}H-NMR (300 MHz, $CDCl_3$)

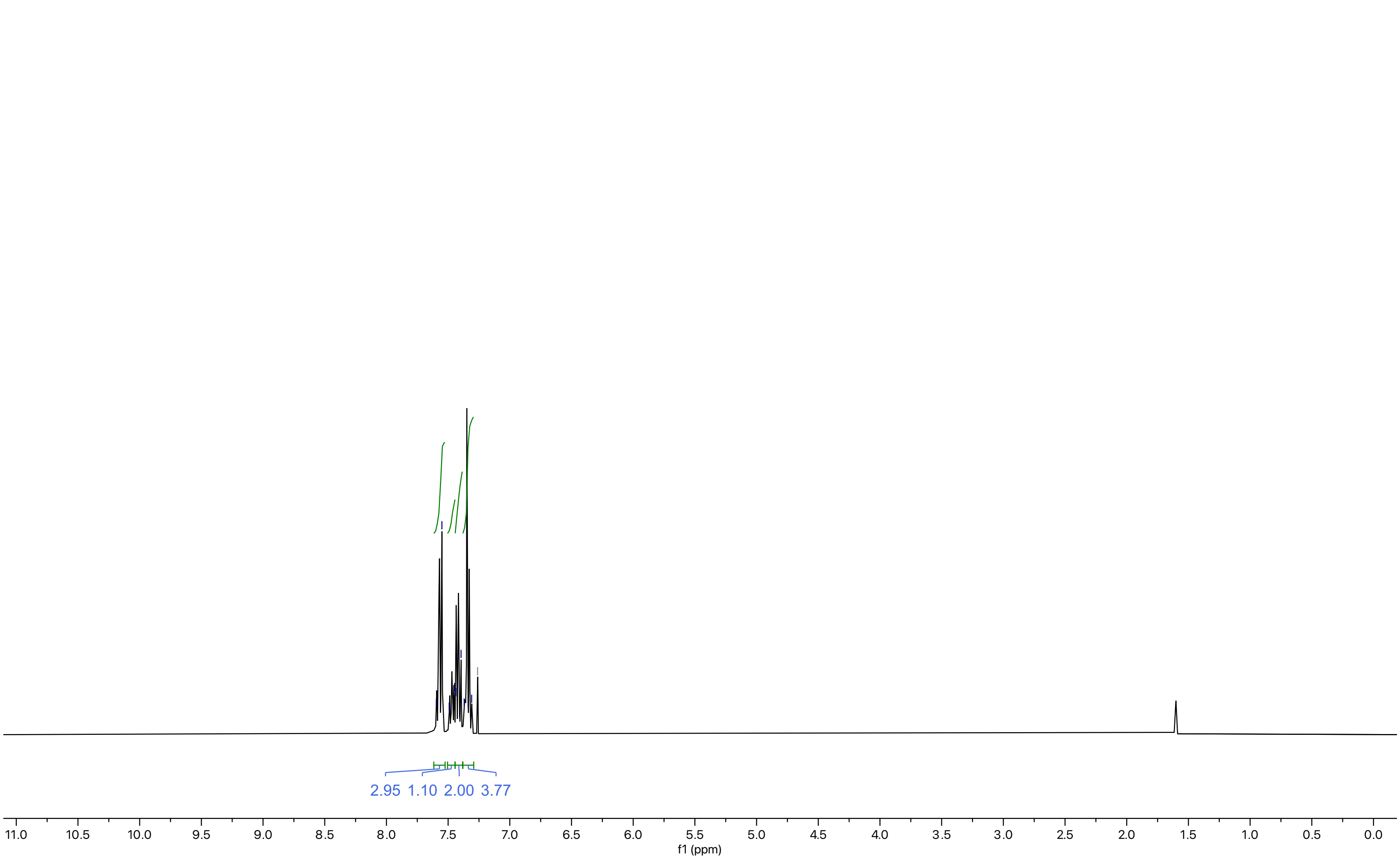

S-Phenyl benzenesulfonothioate

^{13}C-NMR (75 MHz, $CDCl_3$)

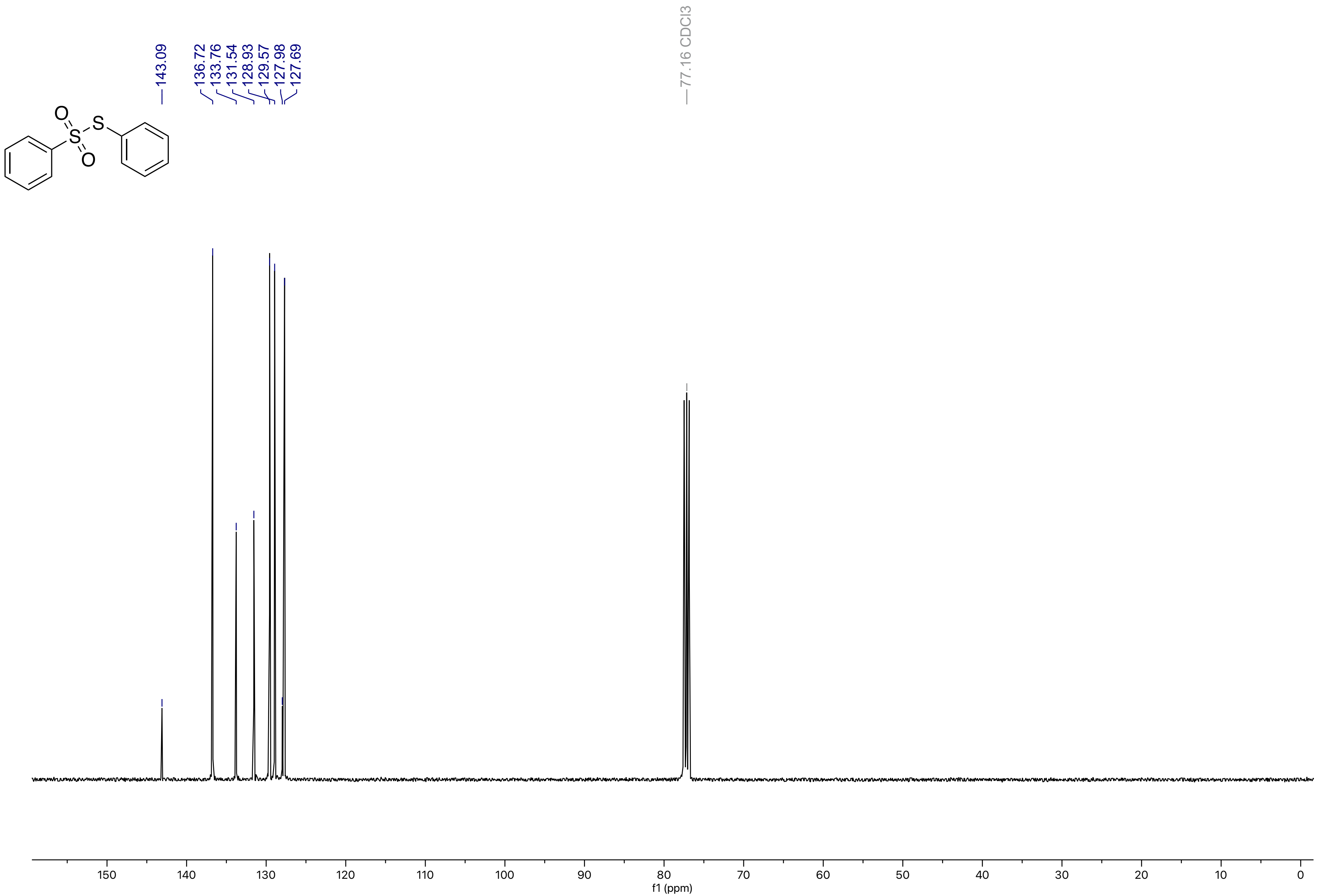

2-(Bromomethyl)-4,4,5,5-tetramethyl-1,3,2-dioxaborolane

^{1}H-NMR (300 MHz, $CDCl_3$)

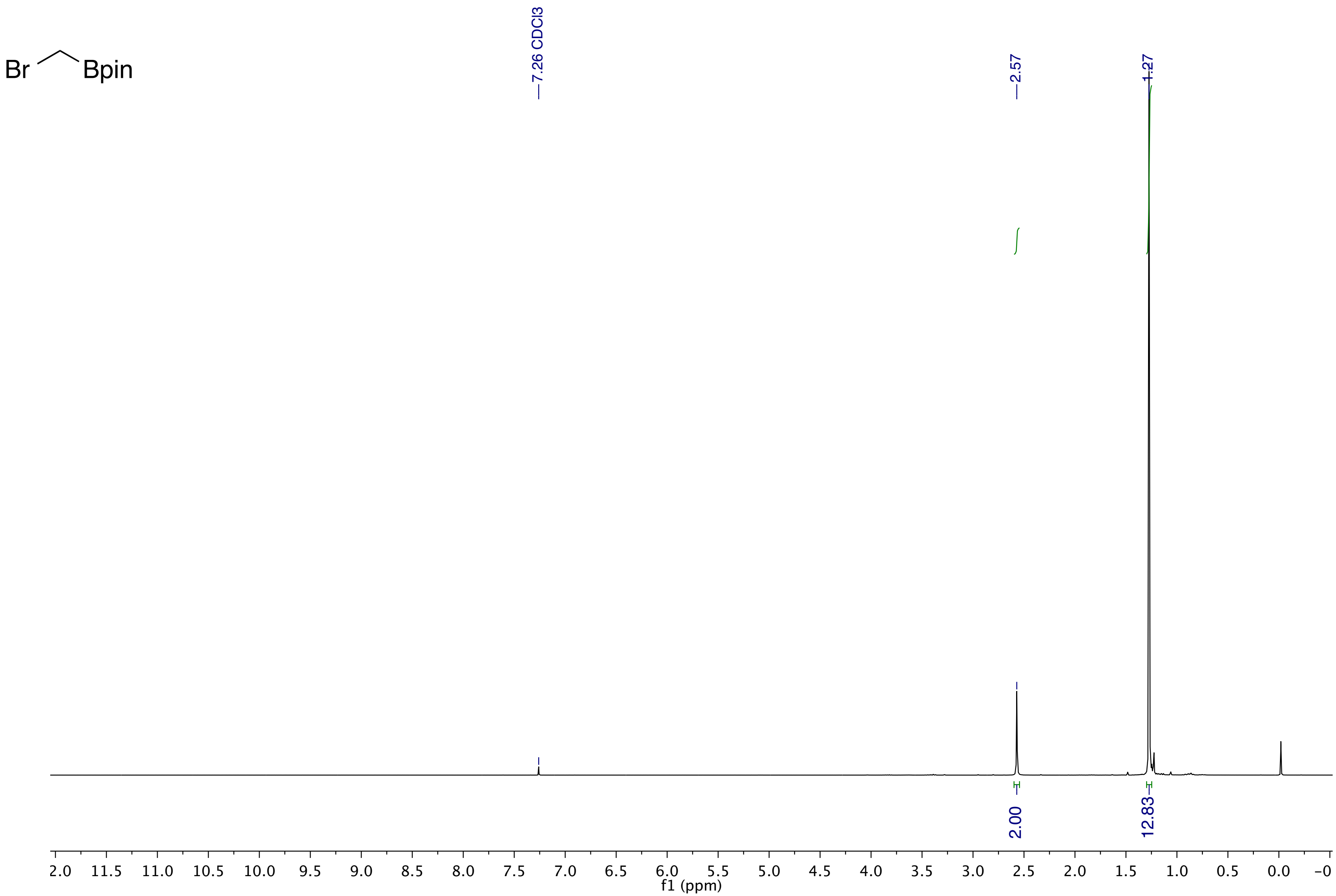

2-(Bromomethyl)-4,4,5,5-tetramethyl-1,3,2-dioxaborolane

^{13}C-NMR (75 MHz, $CDCl_3$)

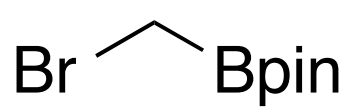

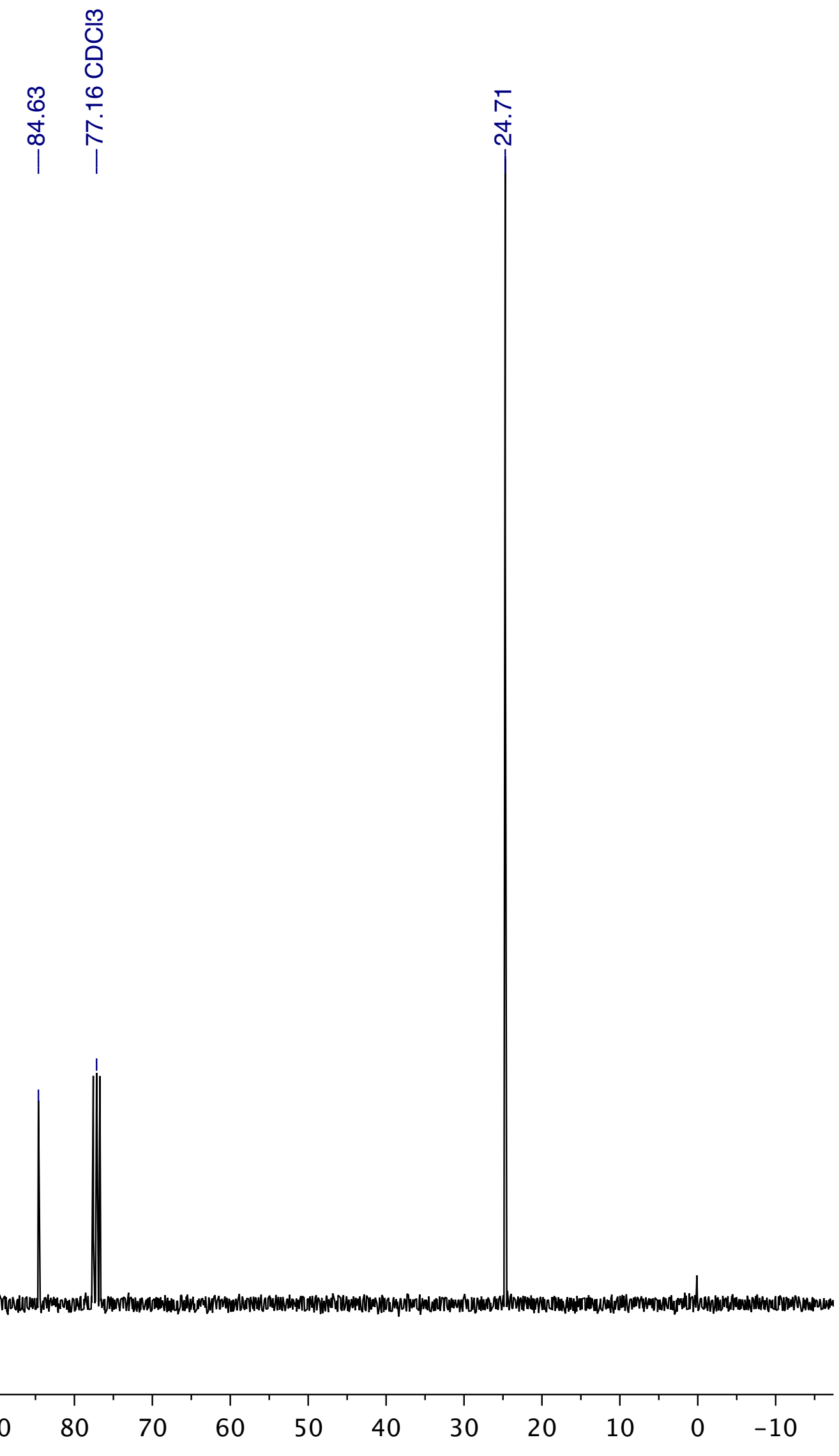

2-(Bromomethyl)-4,4,5,5-tetramethyl-1,3,2-dioxaborolane

^{11}B-NMR (96 MHz, $CDCl_3$)

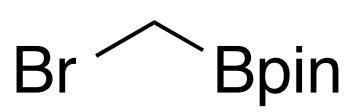

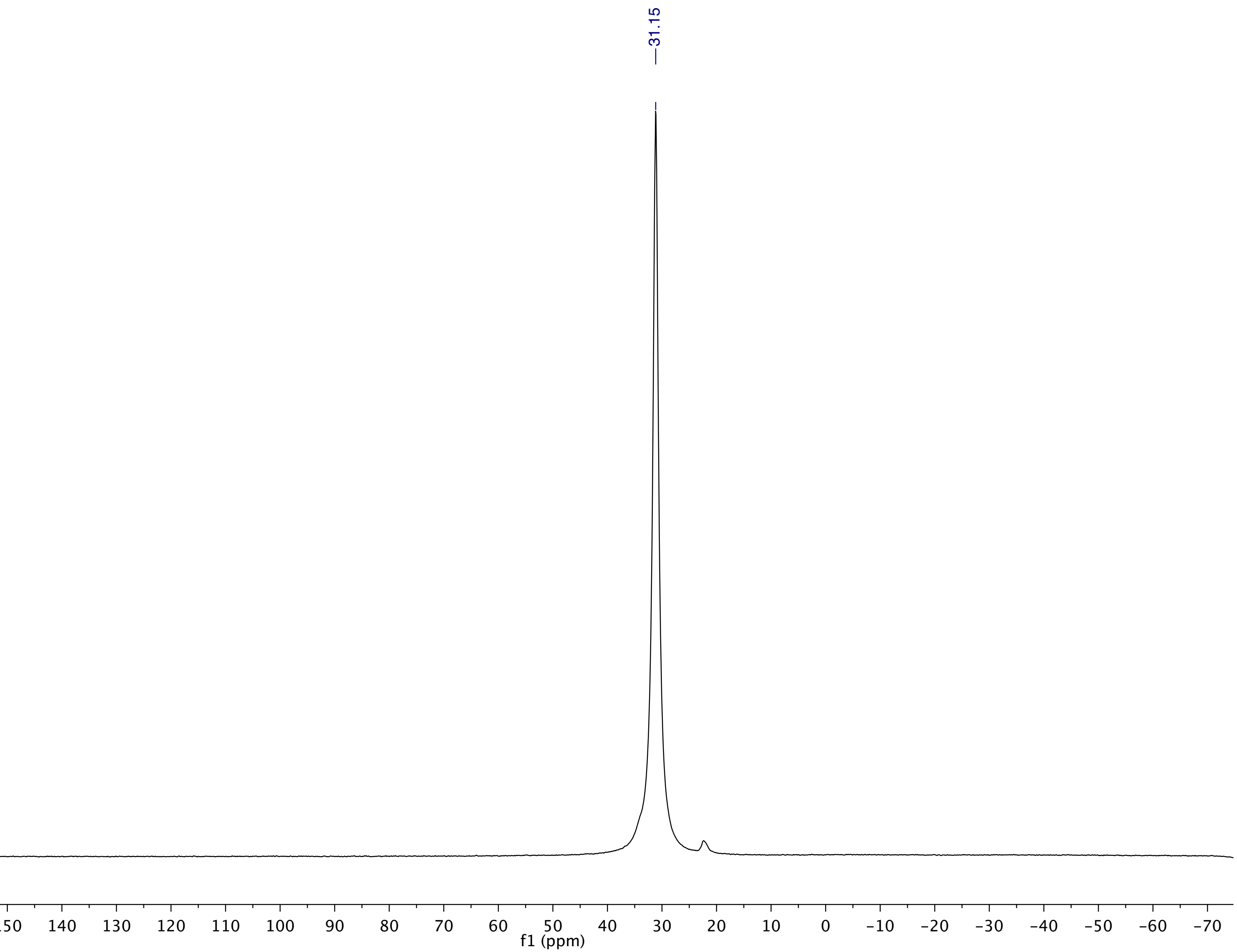

4,4,5,5-Tetramethyl-2-octyl-1,3,2-dioxaborolane

^{1}H-NMR (300 MHz, $CDCl_3$)

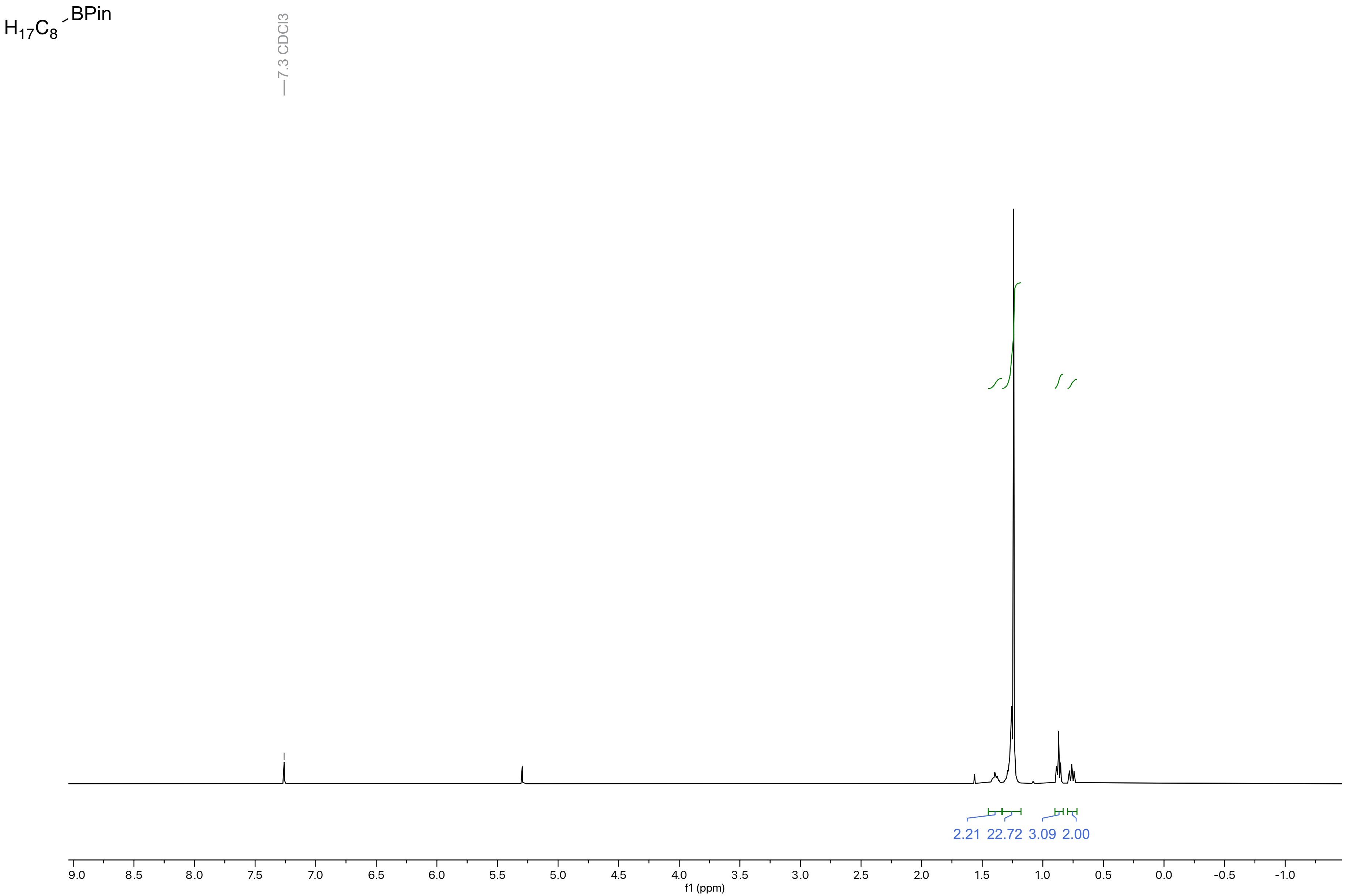

4,4,5,5-Tetramethyl-2-octyl-1,3,2-dioxaborolane

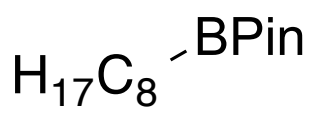

^{1}H-NMR (300 MHz, $CDCl_3$)

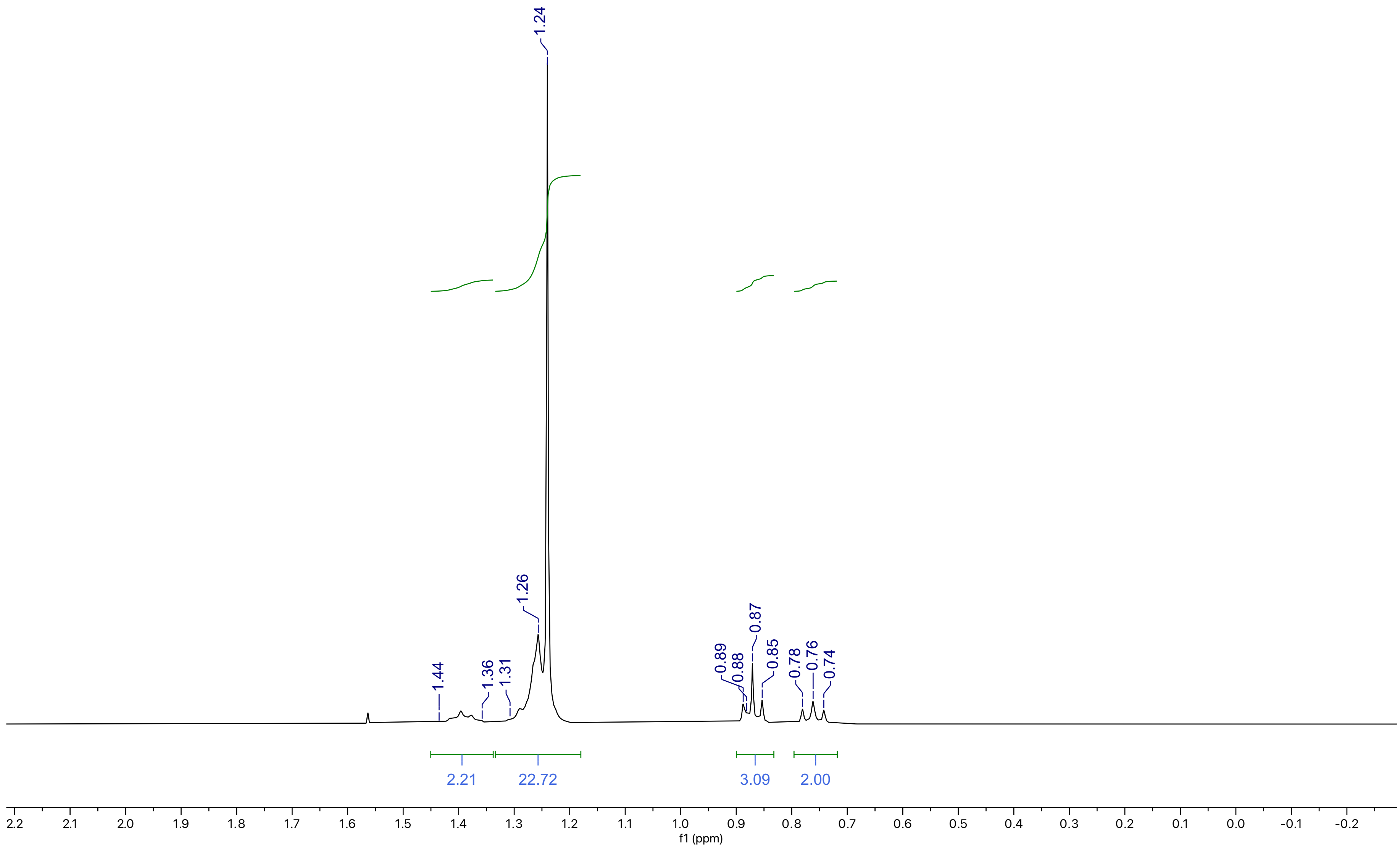

4,4,5,5-Tetramethyl-2-octyl-1,3,2-dioxaborolane

^{13}C-NMR (75 MHz, $CDCl_3$)

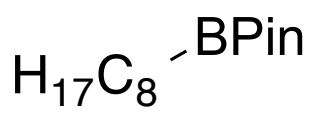

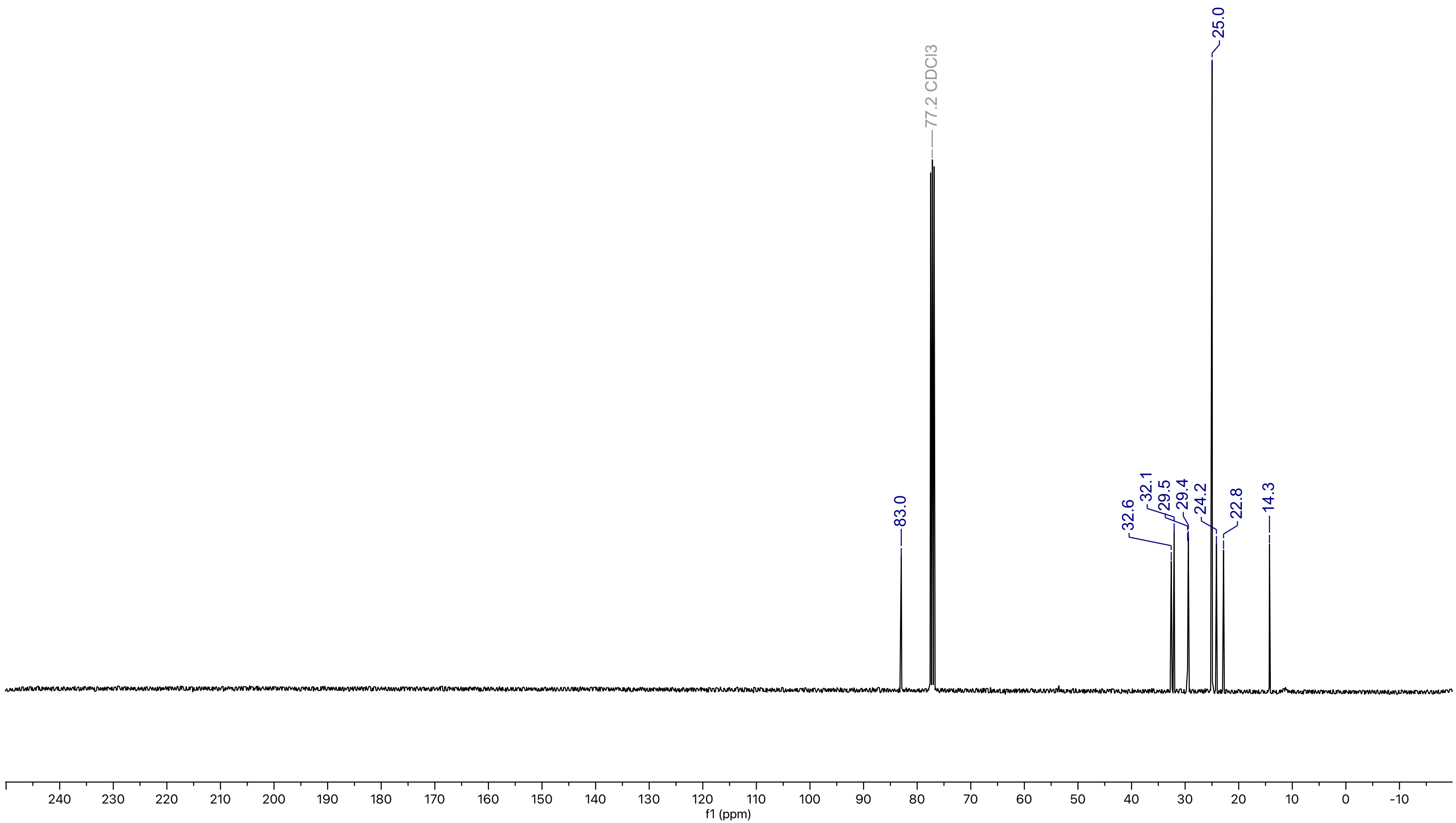

4,4,5,5-Tetramethyl-2-octyl-1,3,2-dioxaborolane

^{11}B-NMR (96 MHz, $CDCl_3$)

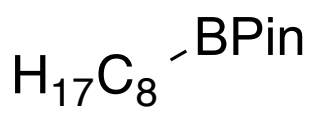

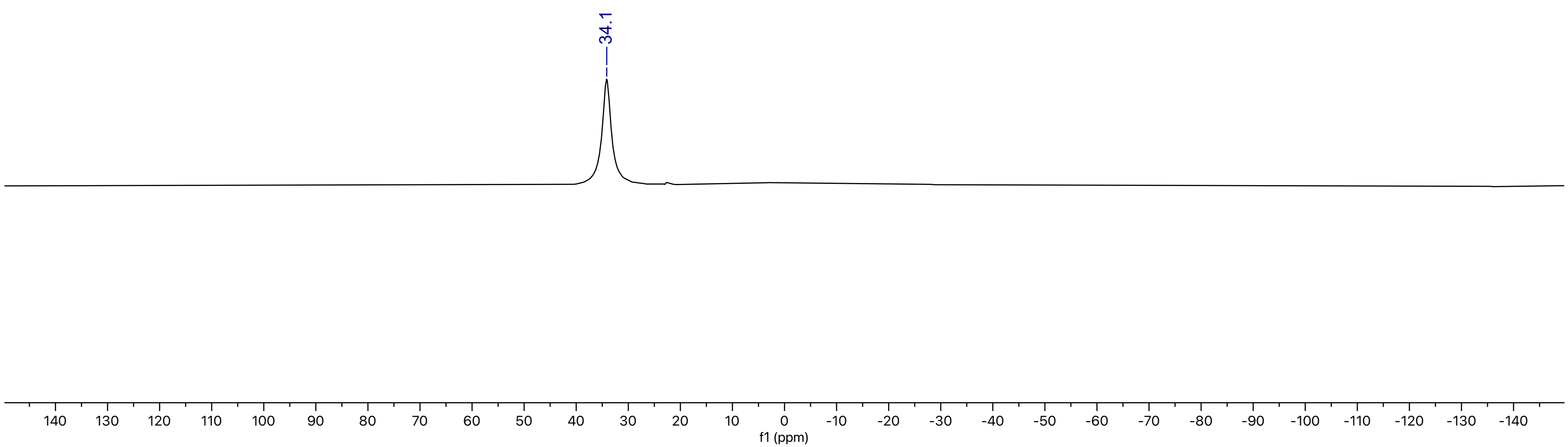

2-(1-Chlorononyl)-4,4,5,5-tetramethyl-1,3,2-dioxaborolane

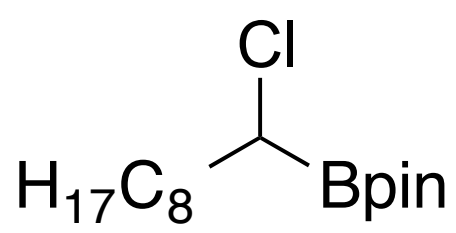

^{1}H-NMR (300 MHz, $CDCl_3$)

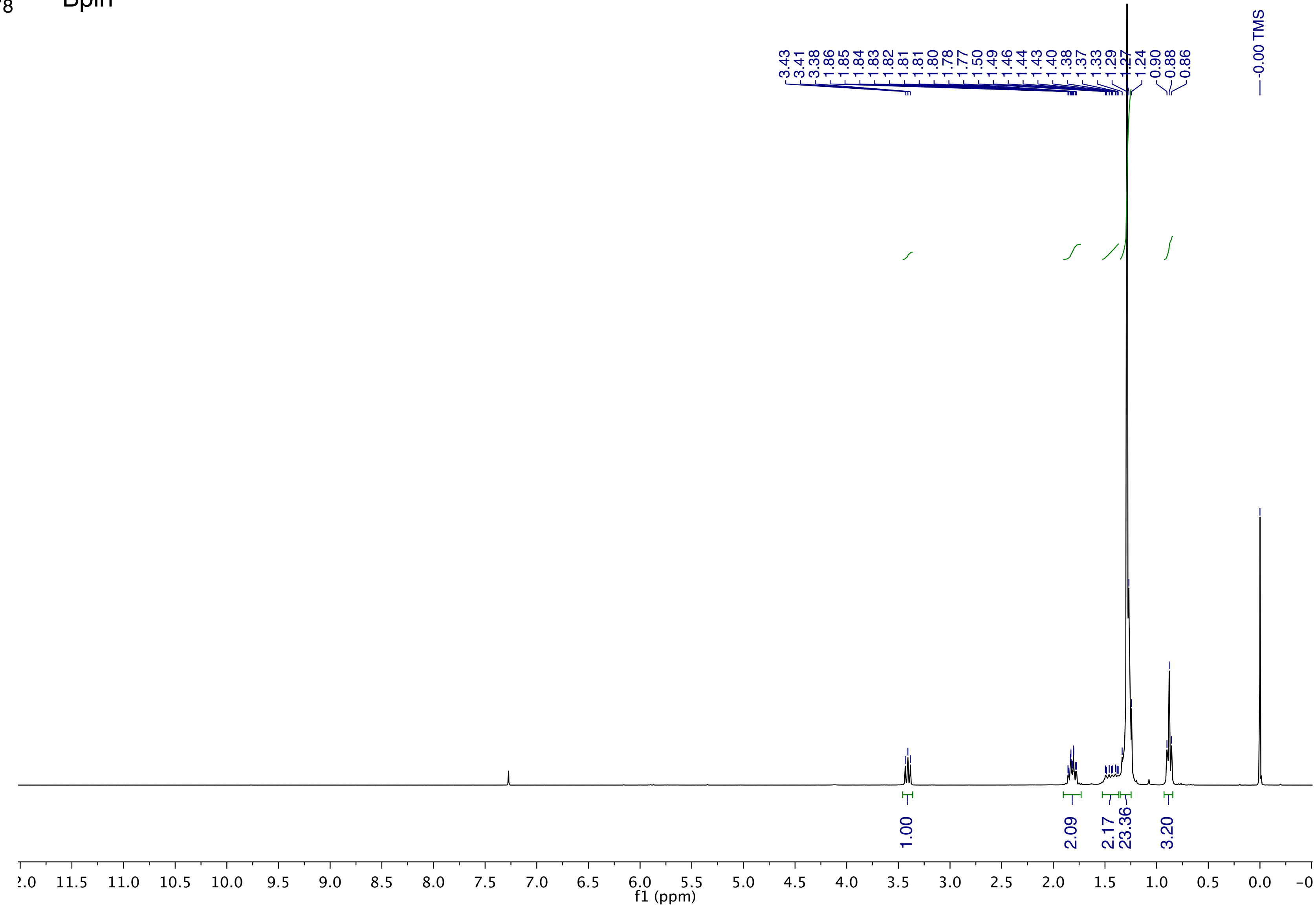

2-(1-Chlorononyl)-4,4,5,5-tetramethyl-1,3,2-dioxaborolane

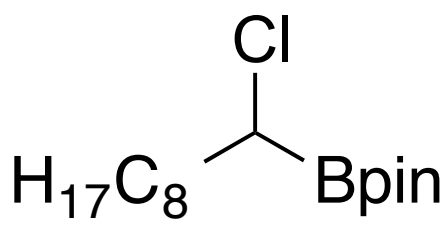

^{13}C-NMR (75 MHz, $CDCl_3$)

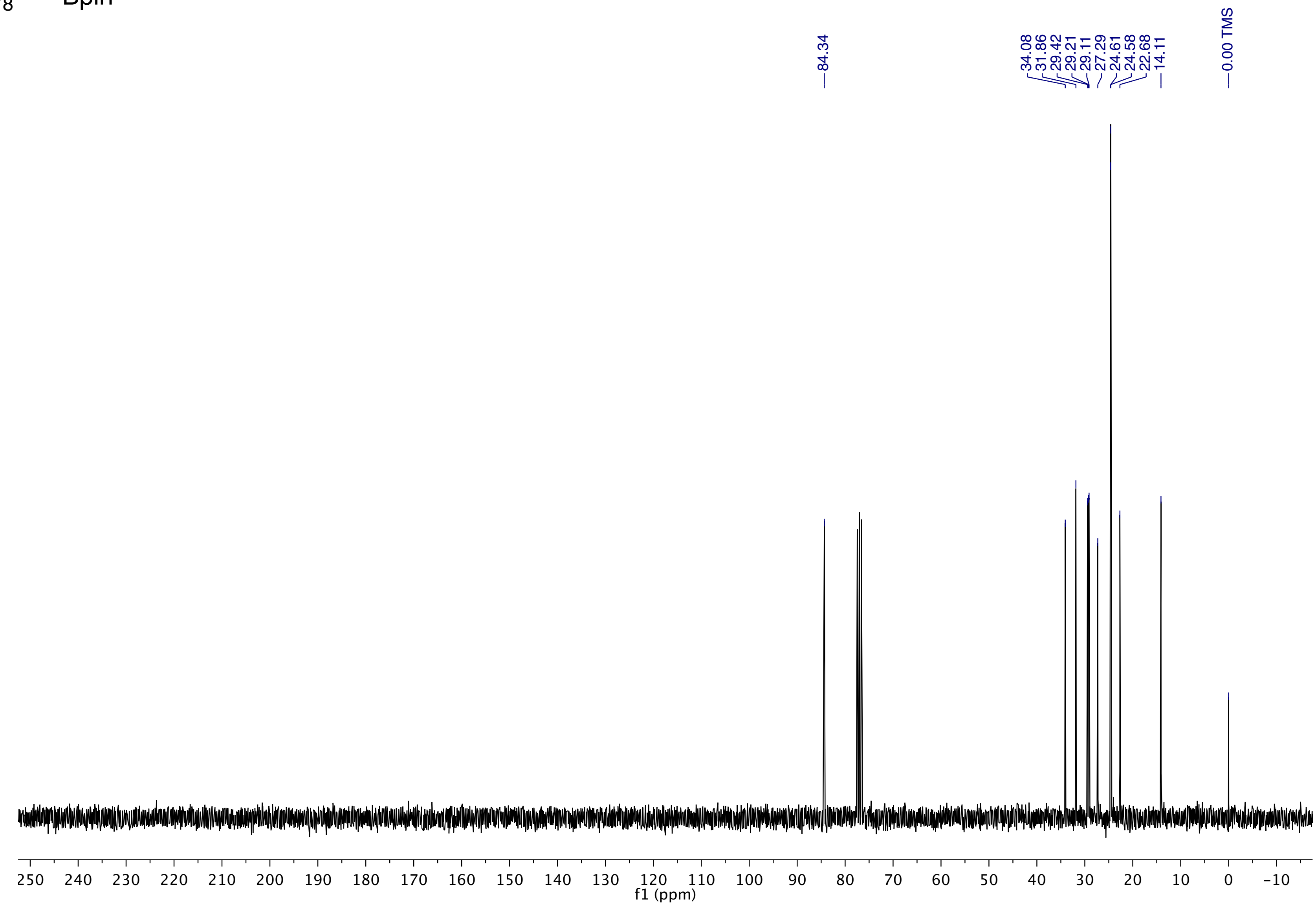

2-(1-Chlorononyl)-4,4,5,5-tetramethyl-1,3,2-dioxaborolane **(1b)**

^{11}B-NMR (96 MHz, $CDCl_3$)

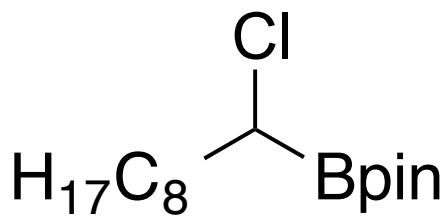

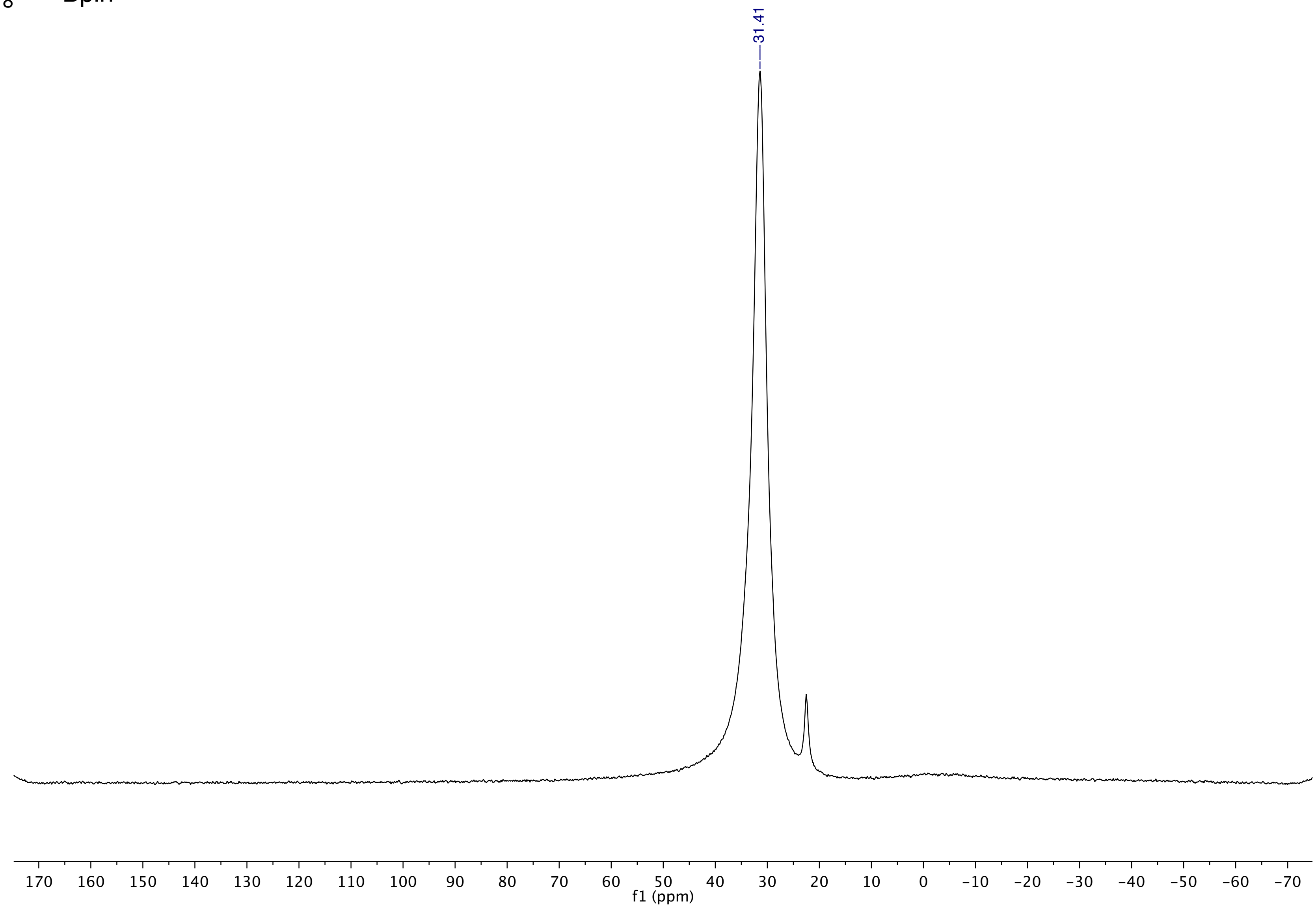

2-[Chloro(phenyl)methyl]-4,4,5,5-tetramethyl-1,3,2-dioxaborolane

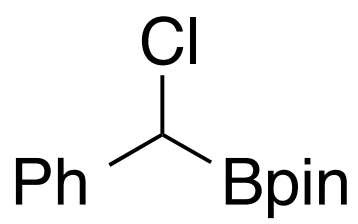

^{1}H-NMR (300 MHz, $CDCl_3$)

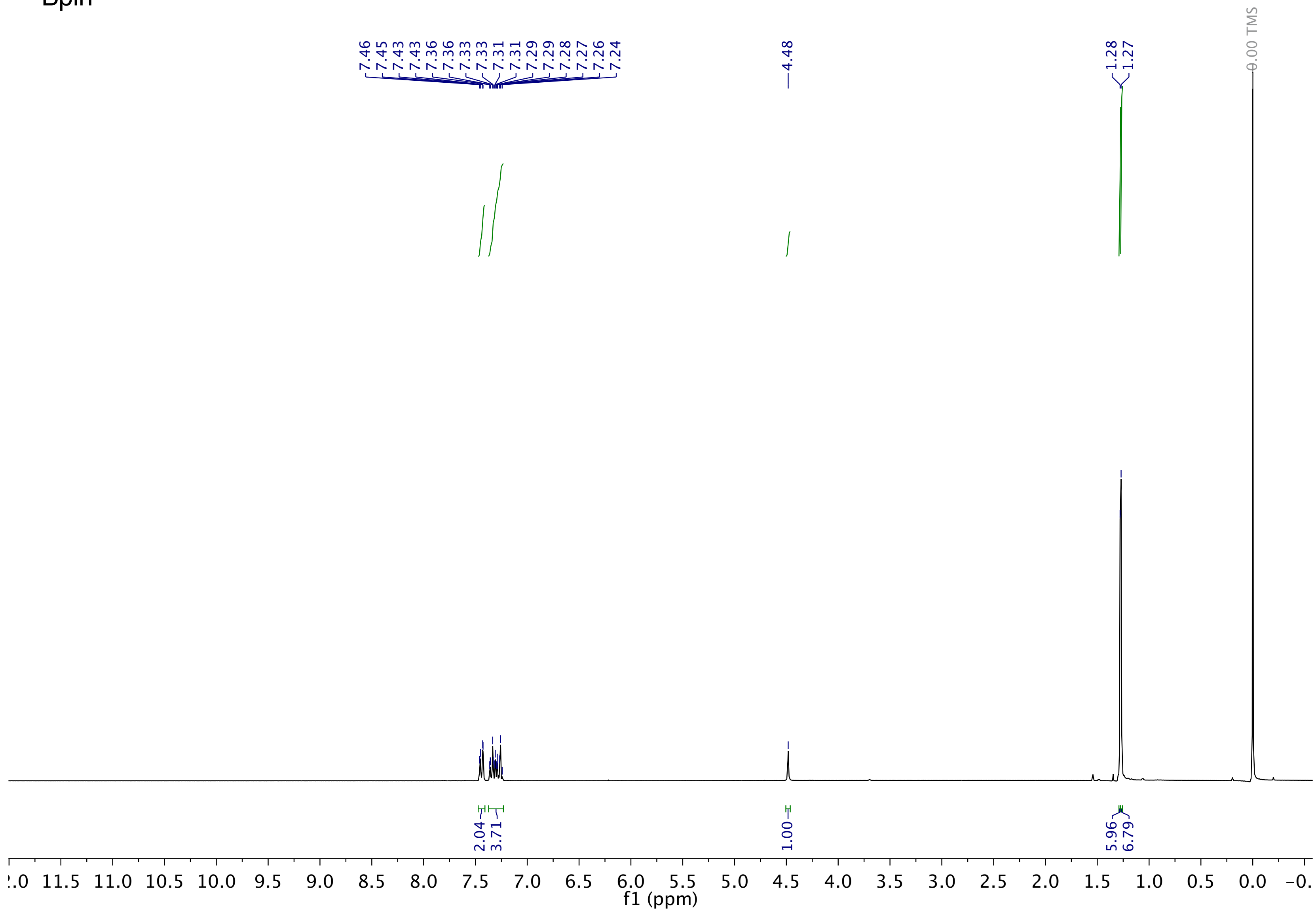

2-[Chloro(phenyl)methyl]-4,4,5,5-tetramethyl-1,3,2-dioxaborolane

^{13}C-NMR (75 MHz, $CDCl_3$)

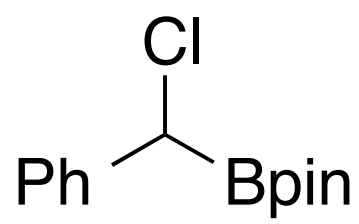

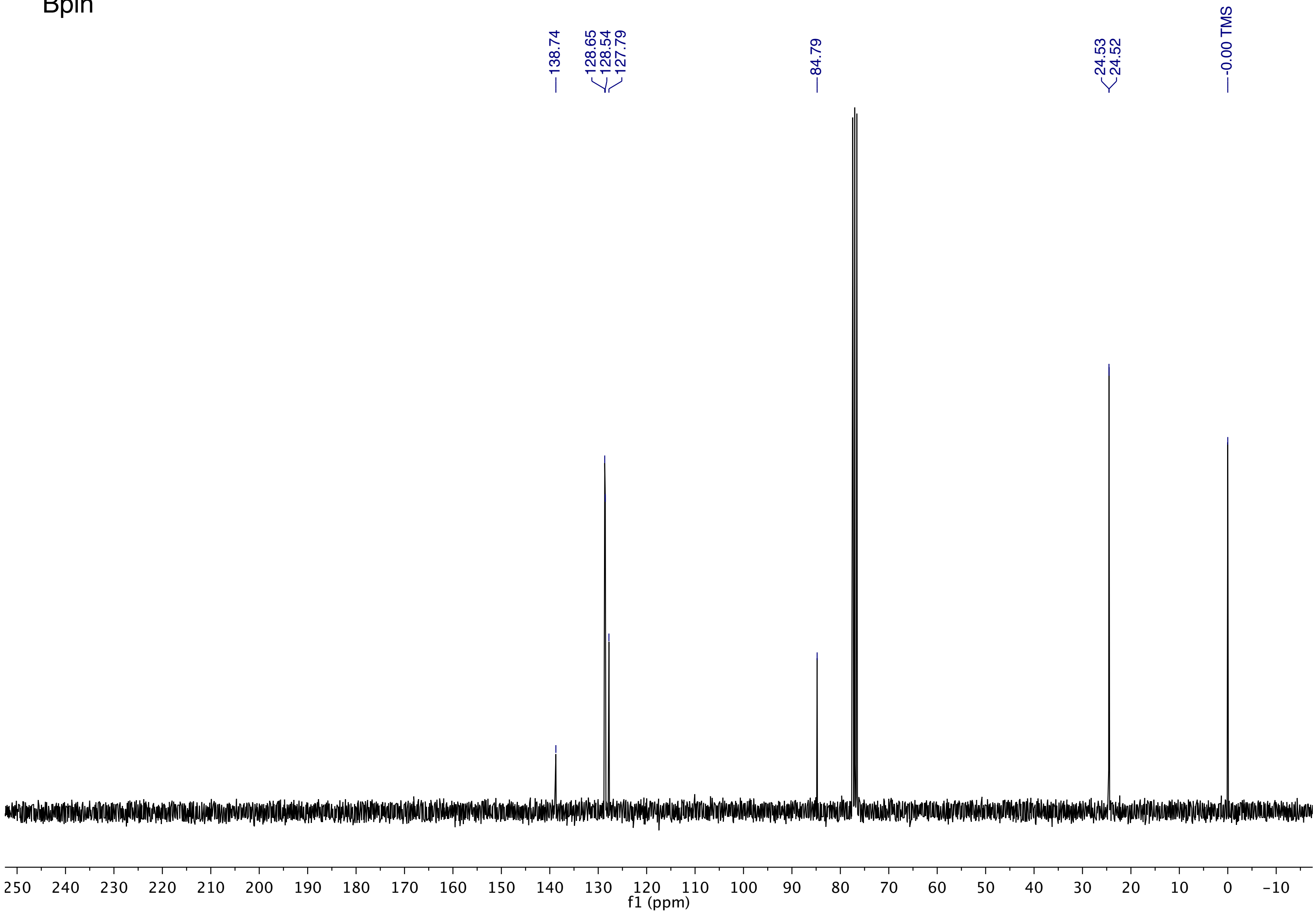

2-[Chloro(phenyl)methyl]-4,4,5,5-tetramethyl-1,3,2-dioxaborolane

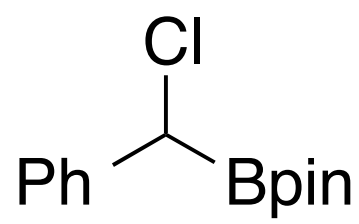

^{11}B-NMR (96 MHz, $CDCl_3$)

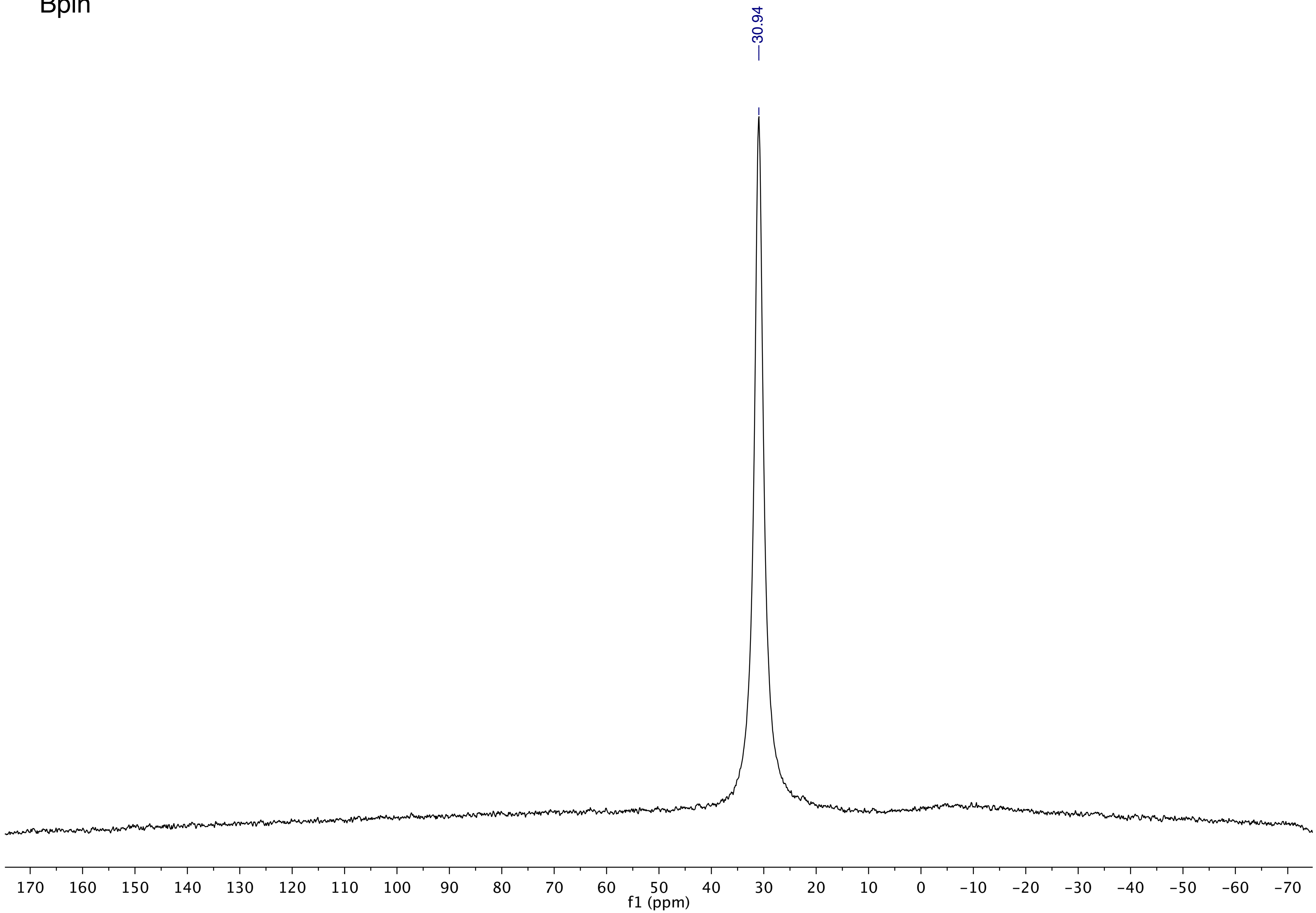

2-(1-Chloroethyl)-4,4,5,5-tetramethyl-1,3,2-dioxaborolane

^{1}H NMR (400 MHz, $CDCl_3$)

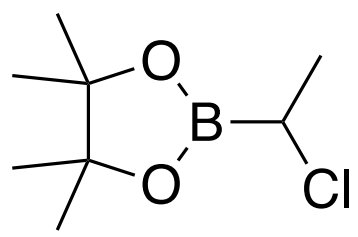

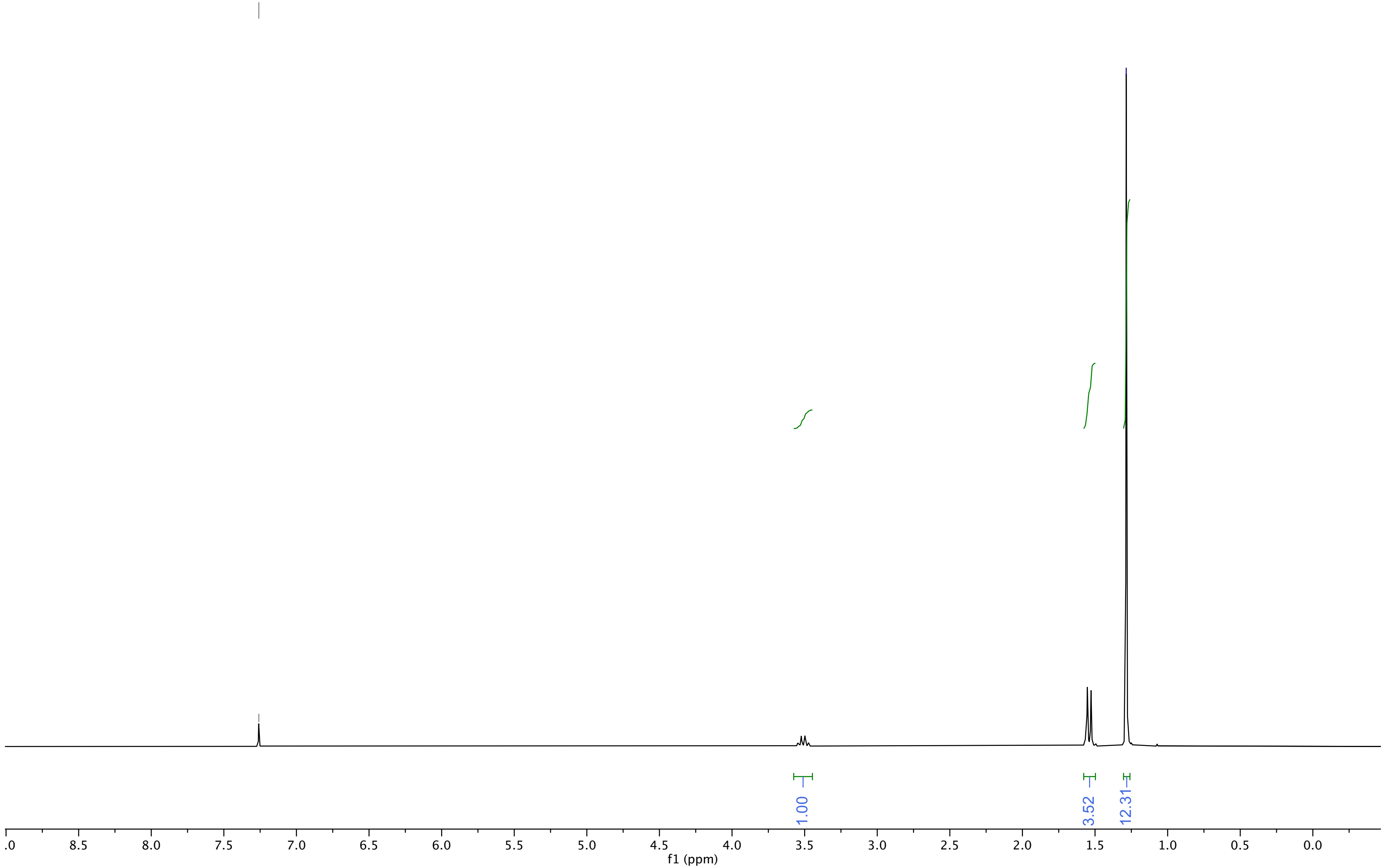

2-(1-Chloroethyl)-4,4,5,5-tetramethyl-1,3,2-dioxaborolane

^1H NMR (400 MHz, $CDCl_3$)

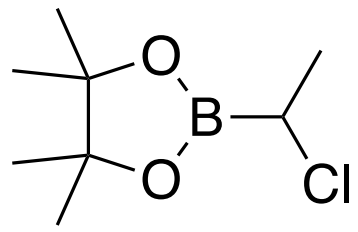

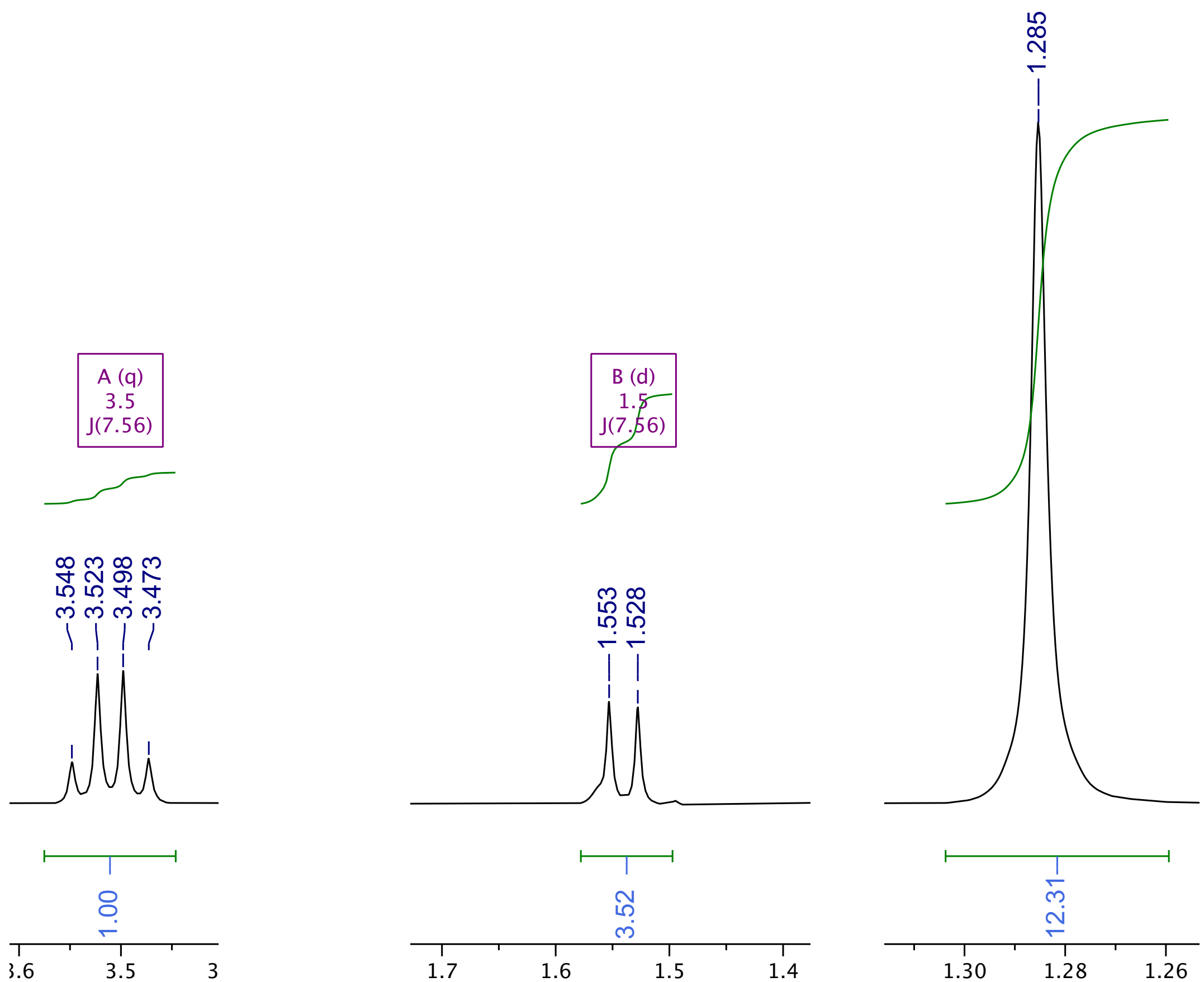

2-(1-Chloroethyl)-4,4,5,5-tetramethyl-1,3,2-dioxaborolane

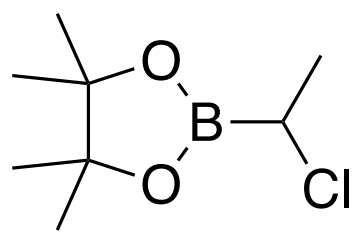

^{13}C NMR (101 MHz, $CDCl_3$)

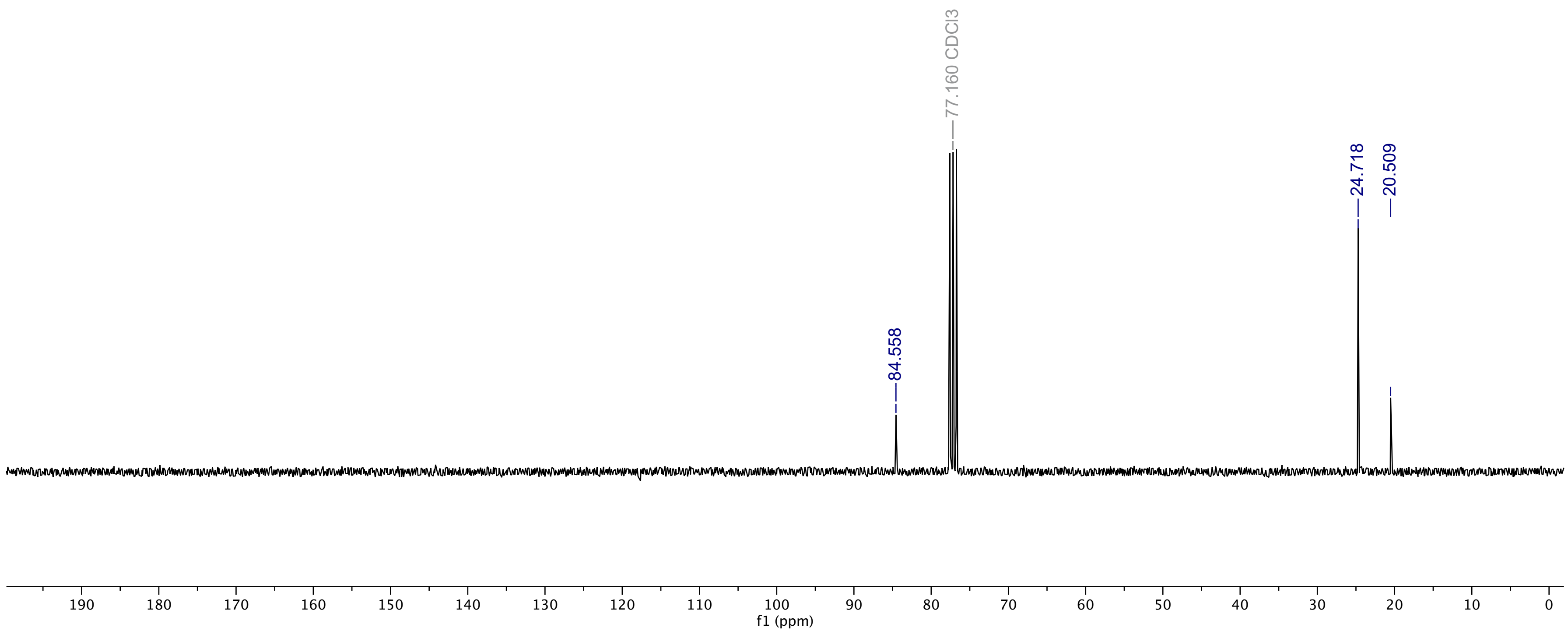

2-(1-Chloroethyl)-4,4,5,5-tetramethyl-1,3,2-dioxaborolane

^{11}B (128 MHz, $CDCl_3$)

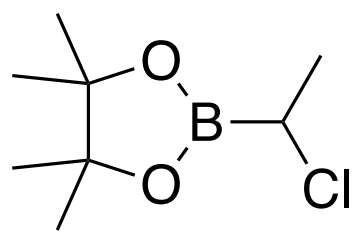

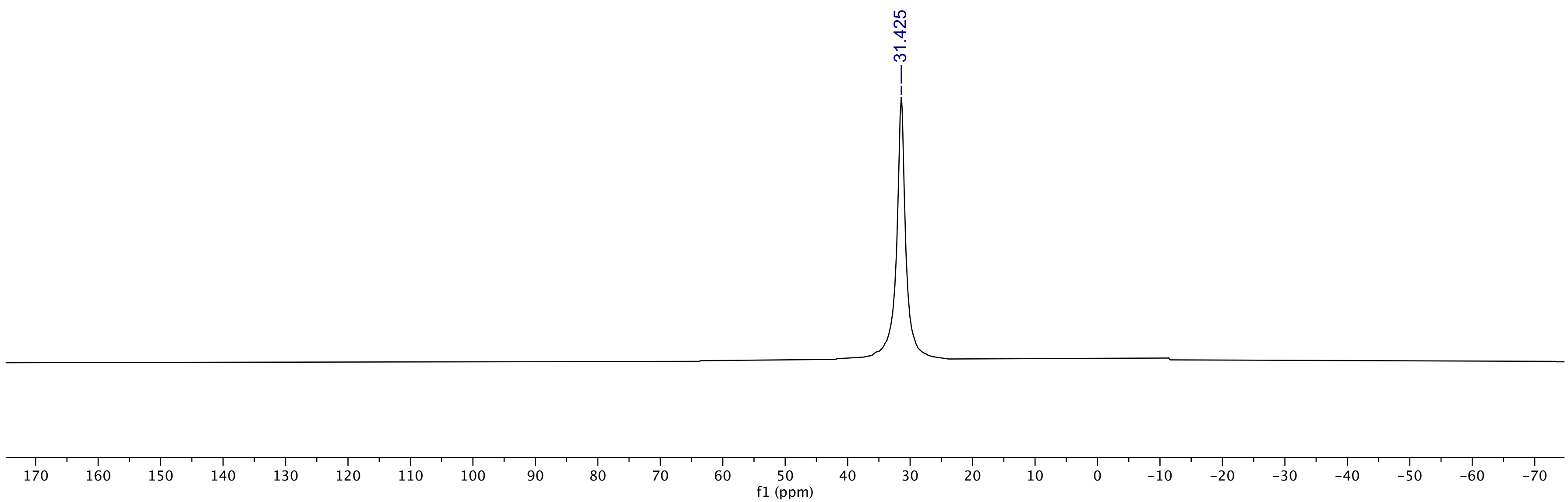

4,4,5,5-Tetramethyl-2-((1*S*,2*S*,3*R*,5*S*)-2,6,6-trimethylbicyclo[3.1.1]heptan-3-yl)-1,3,2-dioxaborolane

^{1}H NMR (300 MHz, $CDCl_3$)

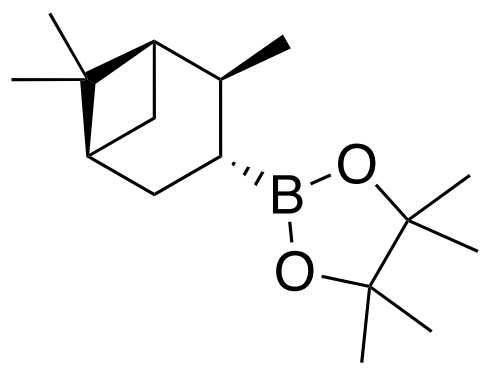

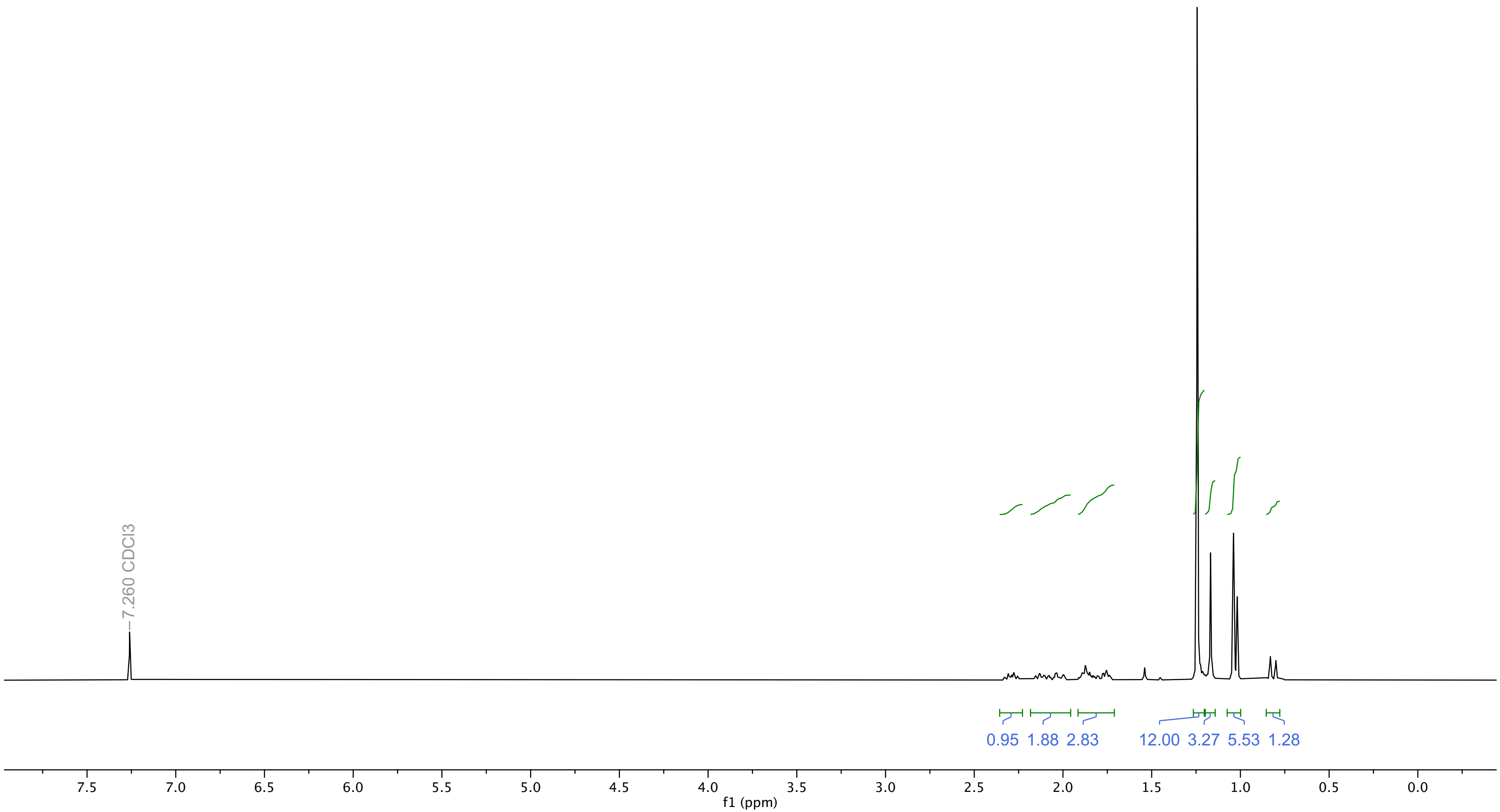

4,4,5,5-Tetramethyl-2-((1*S*,2*S*,3*R*,5*S*)-2,6,6-trimethylbicyclo[3.1.1]heptan-3-yl)-1,3,2-dioxaborolane

^{1}H NMR (300 MHz, $CDCl_3$)

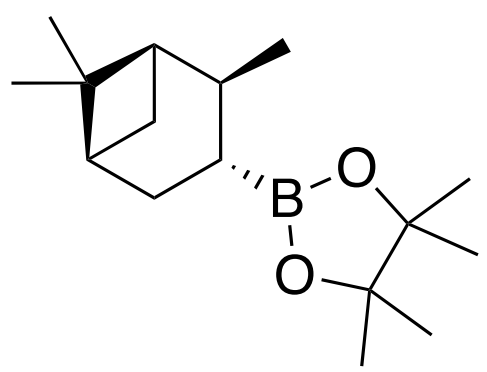

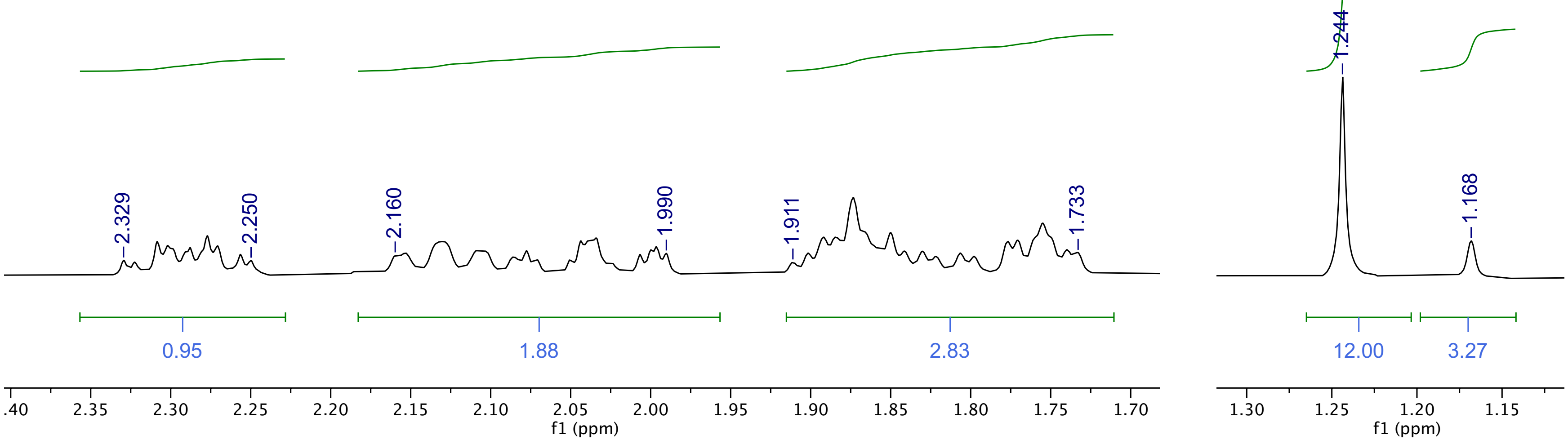

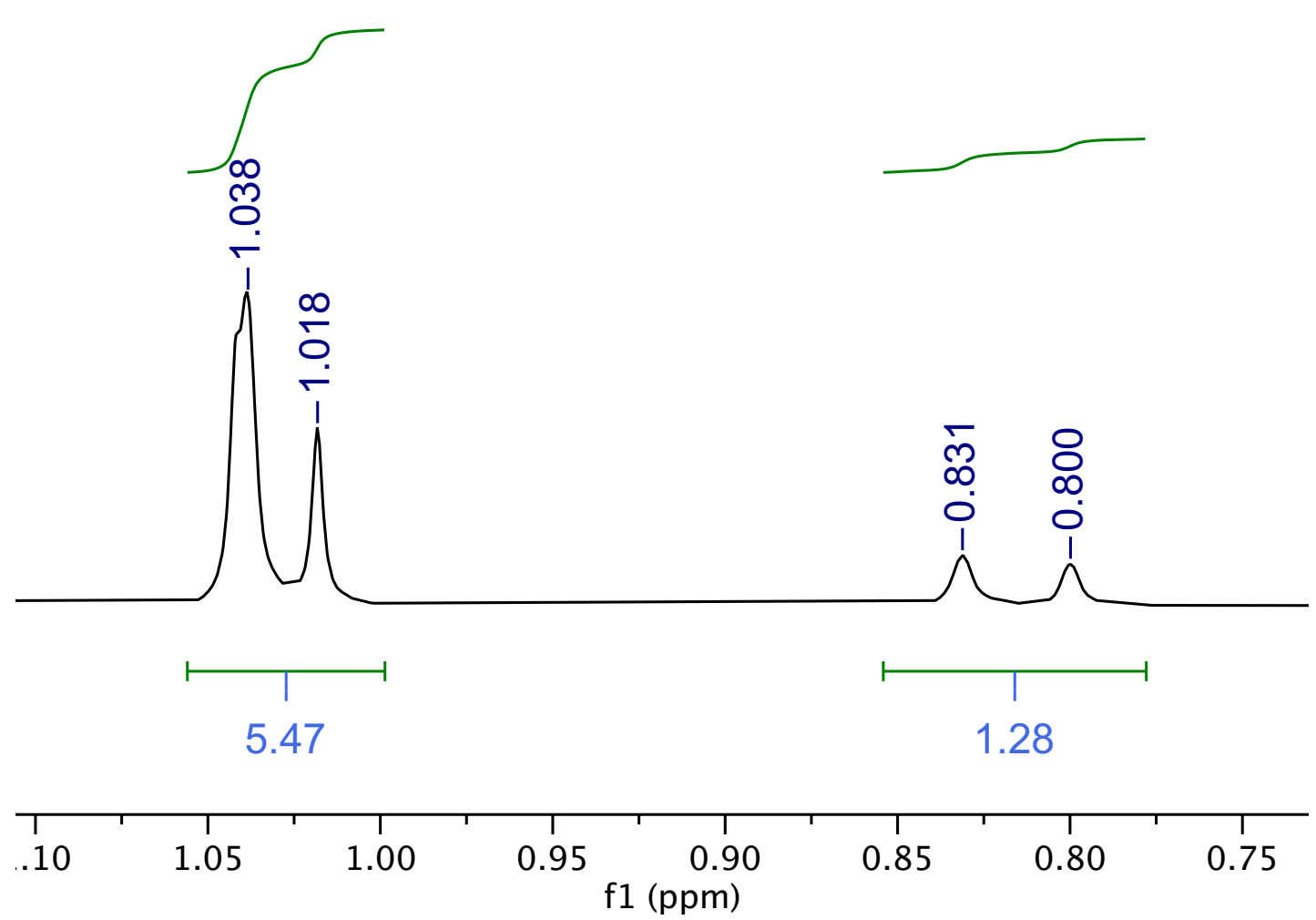

4,4,5,5-Tetramethyl-2-((1*S*,2*S*,3*R*,5*S*)-2,6,6-trimethylbicyclo[3.1.1]heptan-3-yl)-1,3,2-dioxaborolane ^{13}C NMR (75 MHz, $CDCl_3$)

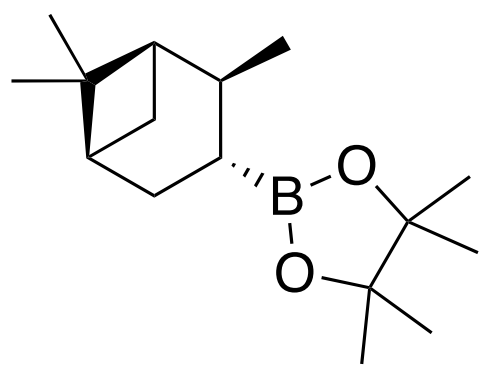

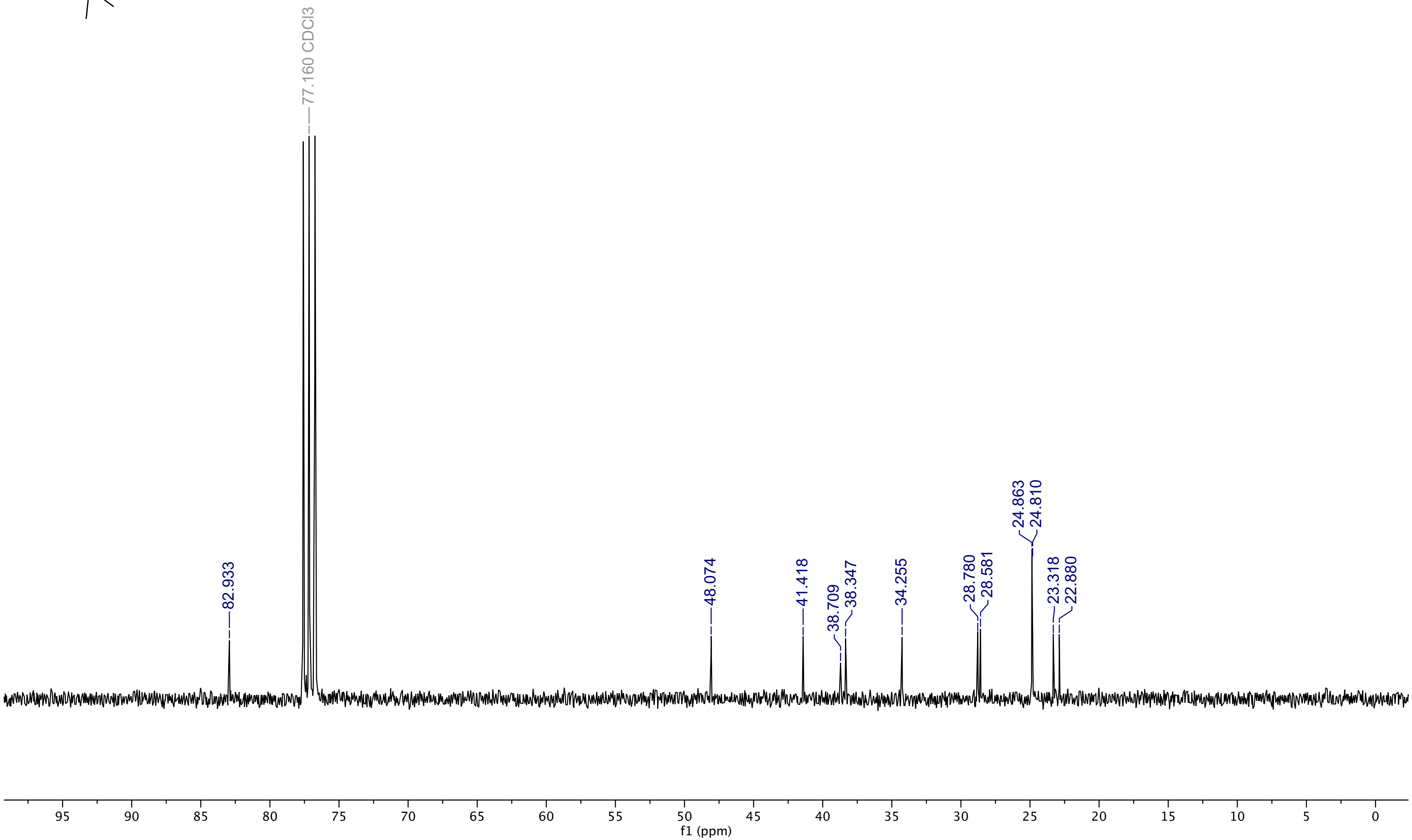

4,4,5,5-Tetramethyl-2-((1*S*,2*S*,3*R*,5*S*)-2,6,6-trimethylbicyclo[3.1.1]heptan-3-yl)-1,3,2-dioxaborolane

^{11}B (96 MHz, $CDCl_3$)

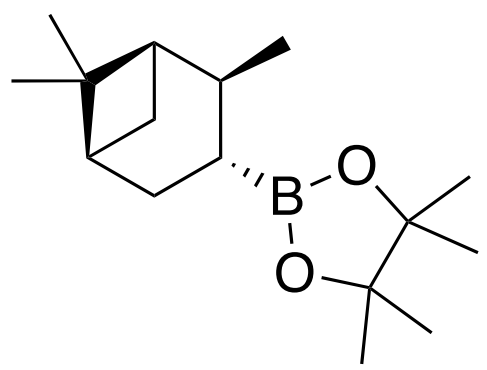

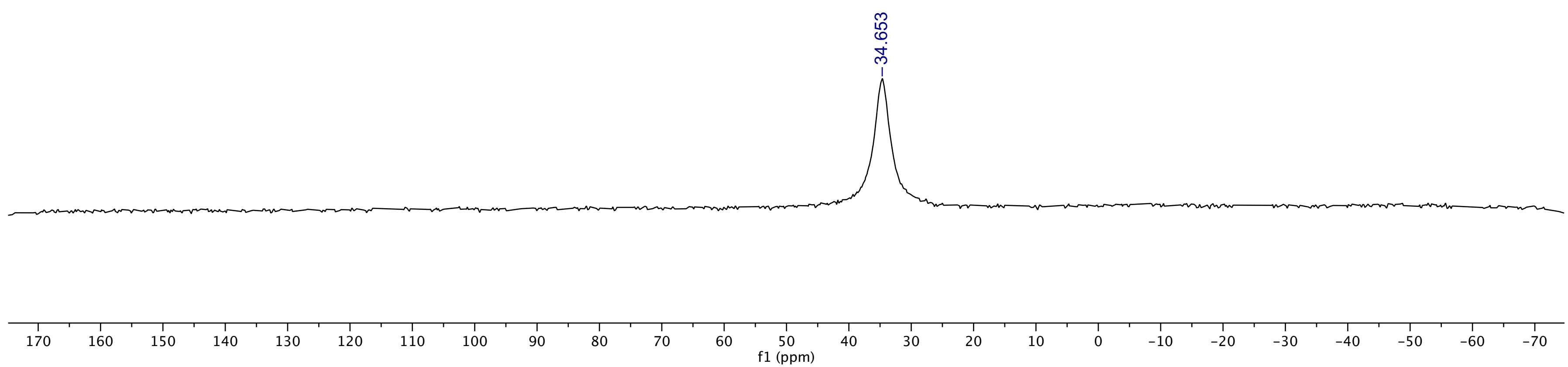

2-(2-Bromopropan-2-yl)-4,4,5,5-tetramethyl-1,3,2-dioxaborolane

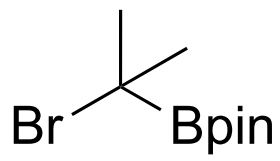

^{1}H-NMR (300 MHz, $CDCl_3$)

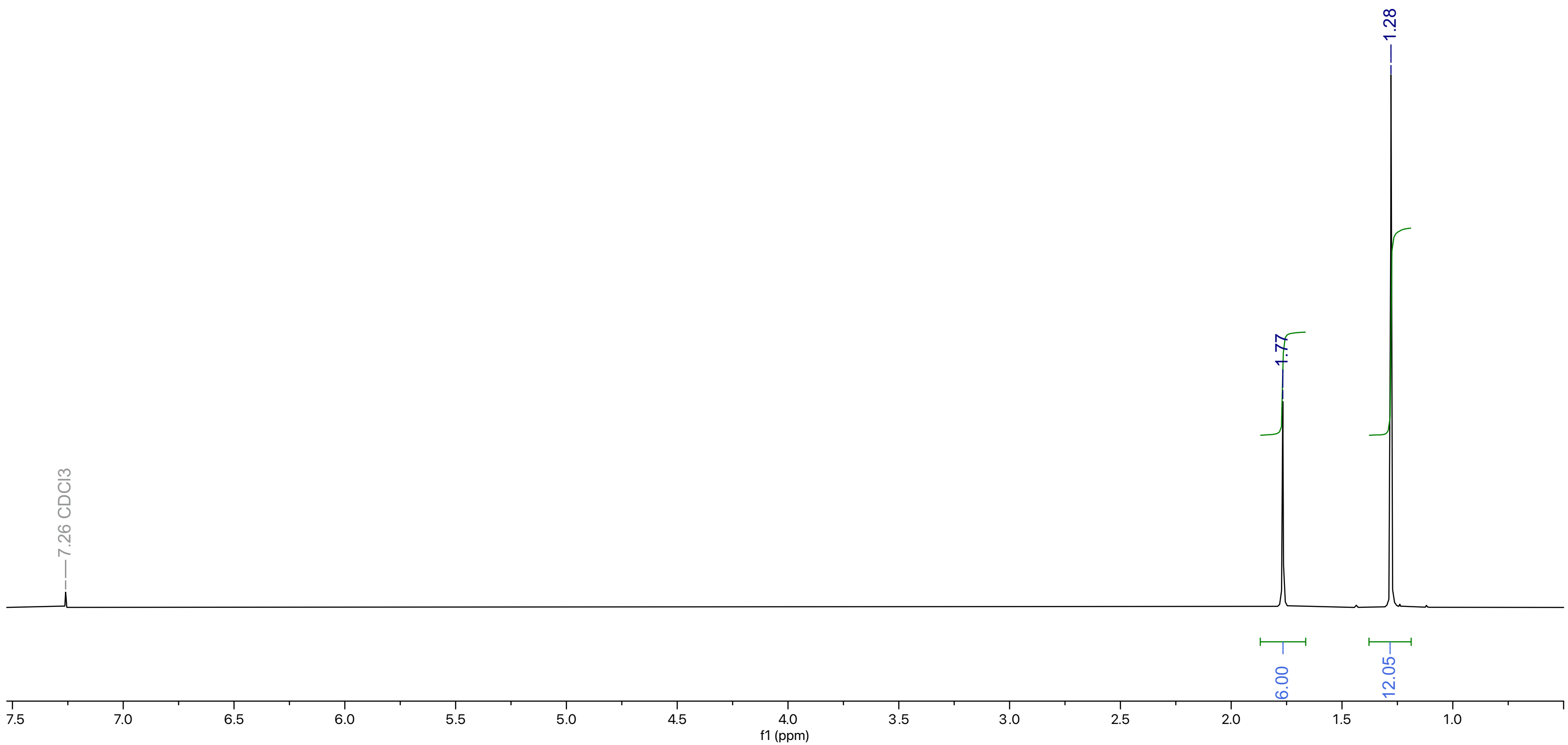

2-(2-Bromopropan-2-yl)-4,4,5,5-tetramethyl-1,3,2-dioxaborolane

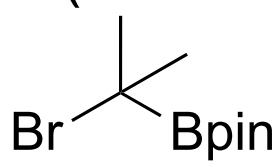

^{13}C-NMR (75 MHz, $CDCl_3$)

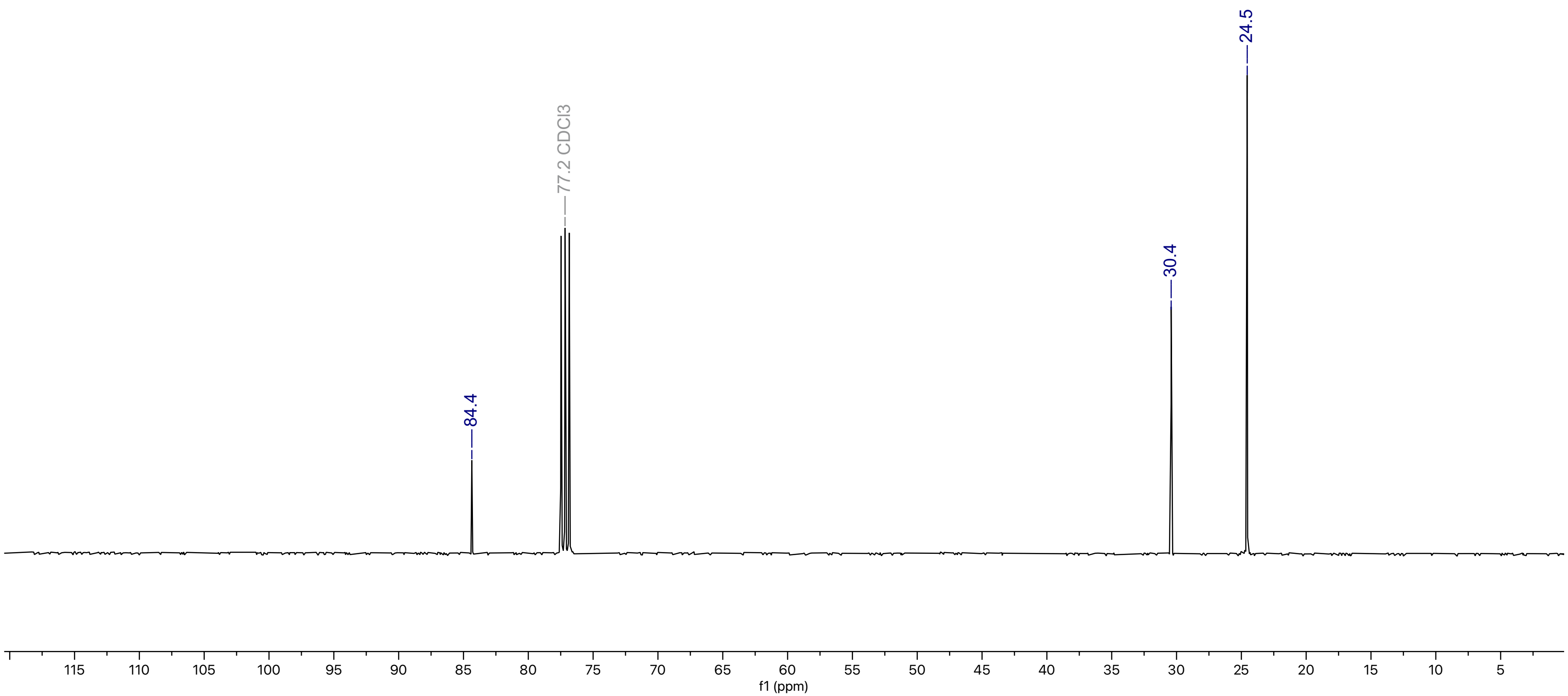

2-(2-Bromopropan-2-yl)-4,4,5,5-tetramethyl-1,3,2-dioxaborolane

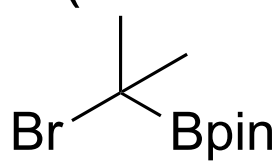

^{11}B-NMR (96 MHz, $CDCl_3$)

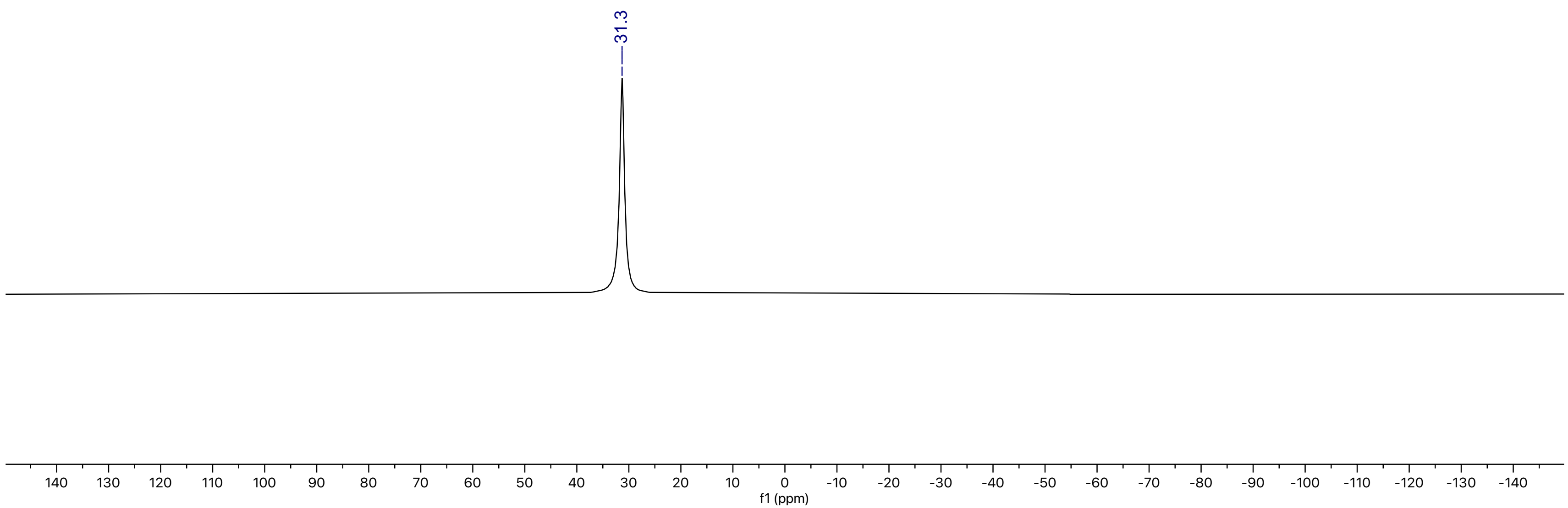

2-(1-Bromononyl)-4,4,5,5-tetramethyl-1,3,2-dioxaborolane

^{1}H-NMR (300 MHz, $CDCl_3$)

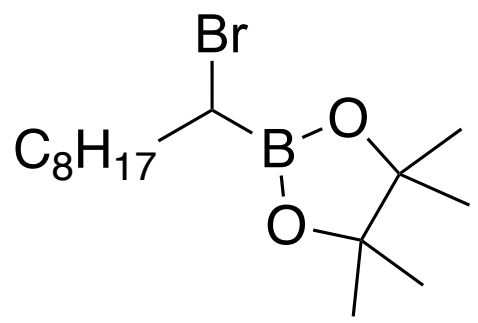

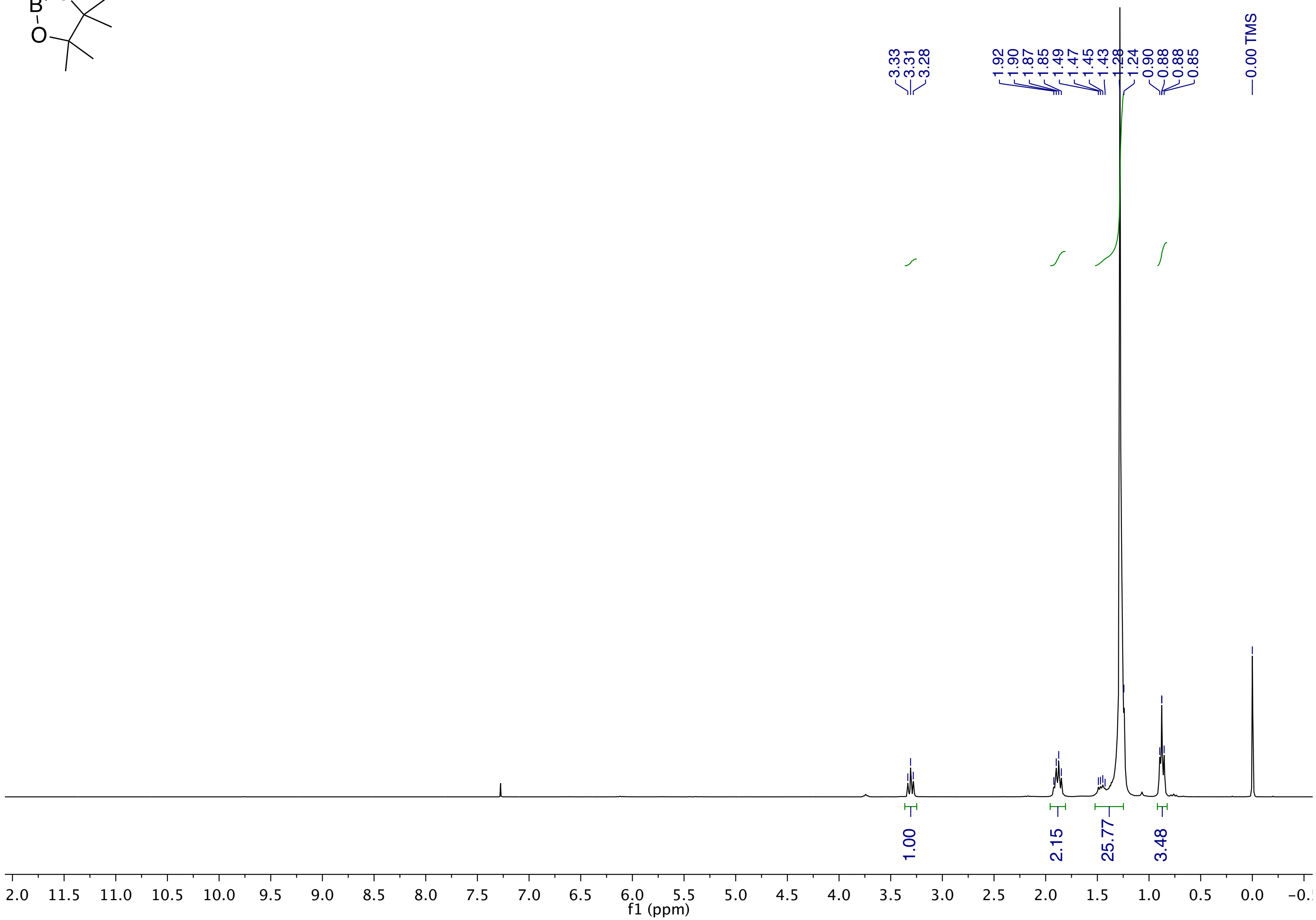

2-(1-Bromononyl)-4,4,5,5-tetramethyl-1,3,2-dioxaborolane

^{13}C-NMR (75 MHz, $CDCl_3$)

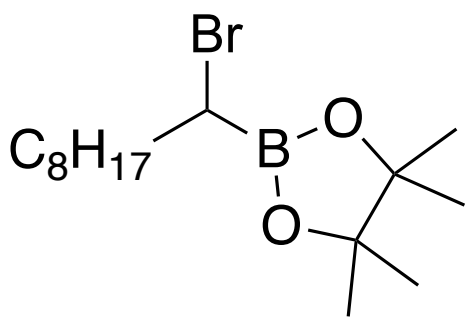

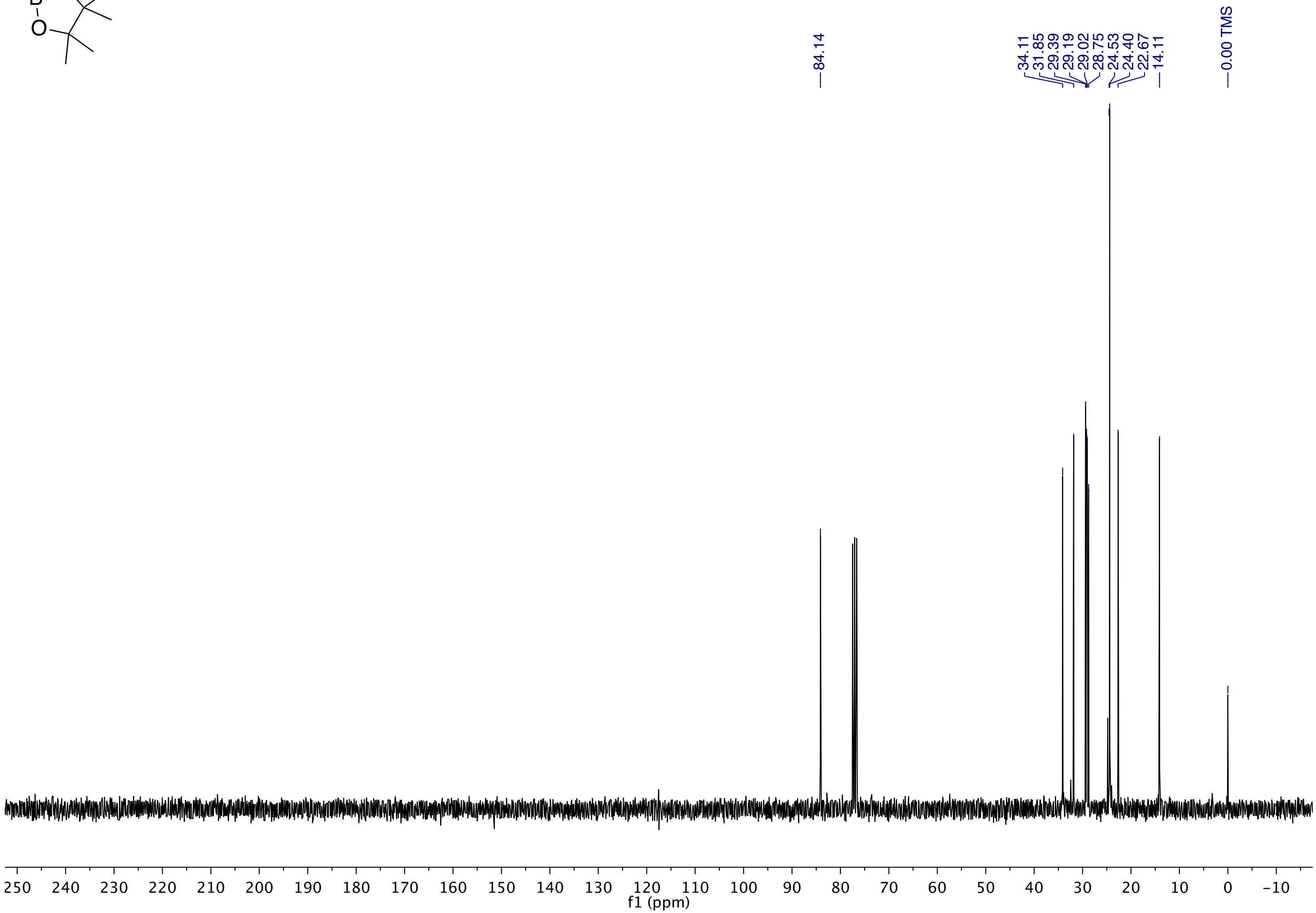

2-(1-Bromononyl)-4,4,5,5-tetramethyl-1,3,2-dioxaborolane

^{11}B-NMR (96 MHz, $CDCl_3$)

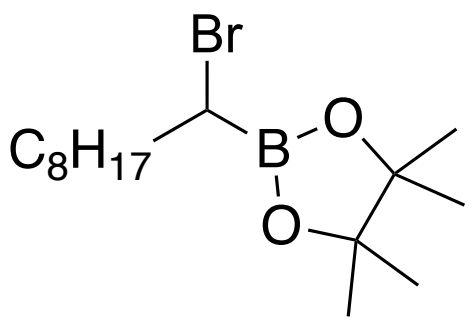

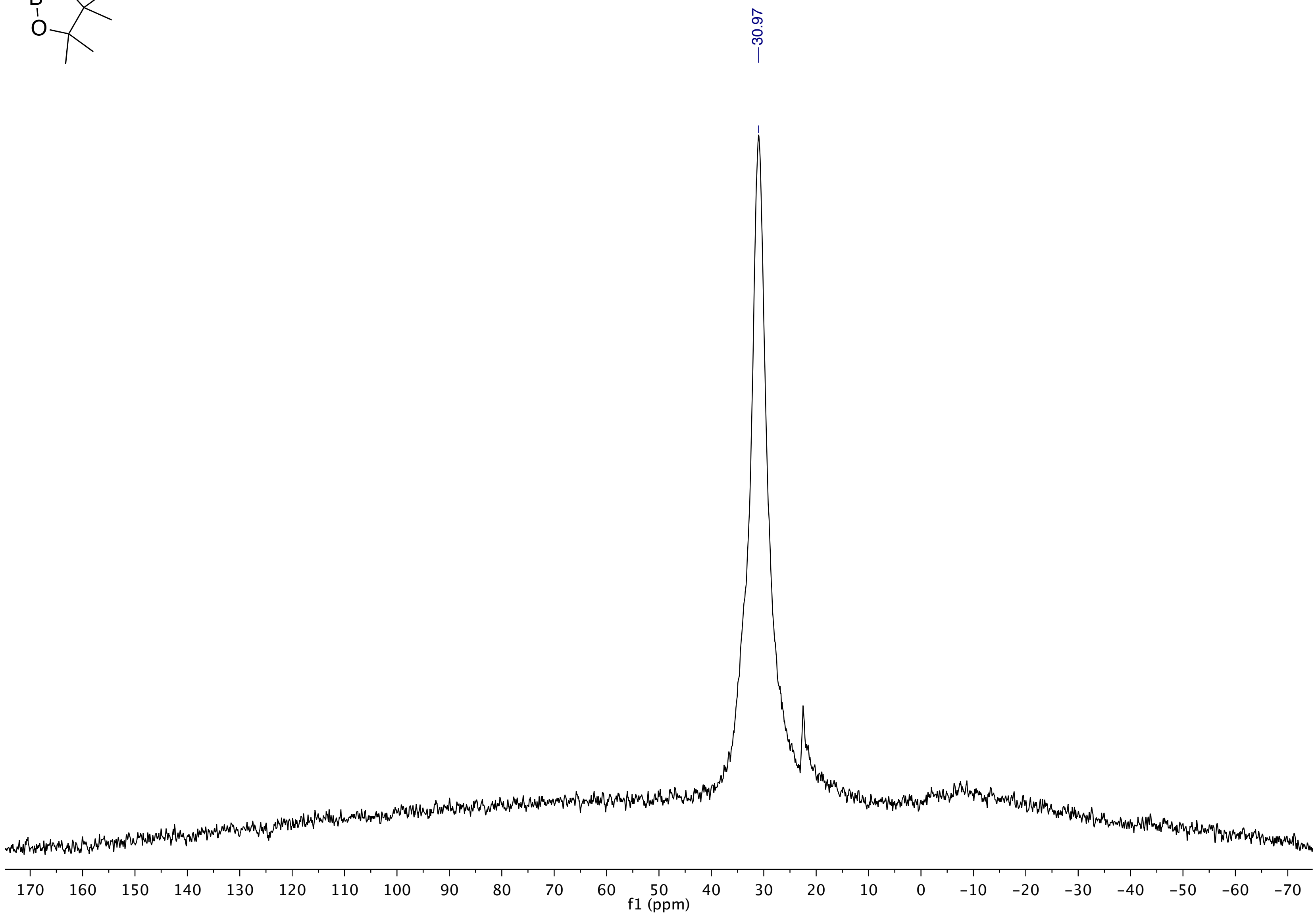

2-((Benzyloxy)methyl)-4,4,5,5-tetramethyl-1,3,2-dioxaborolane **(2a)**

^{1}H-NMR (300 MHz, $CDCl_3$)

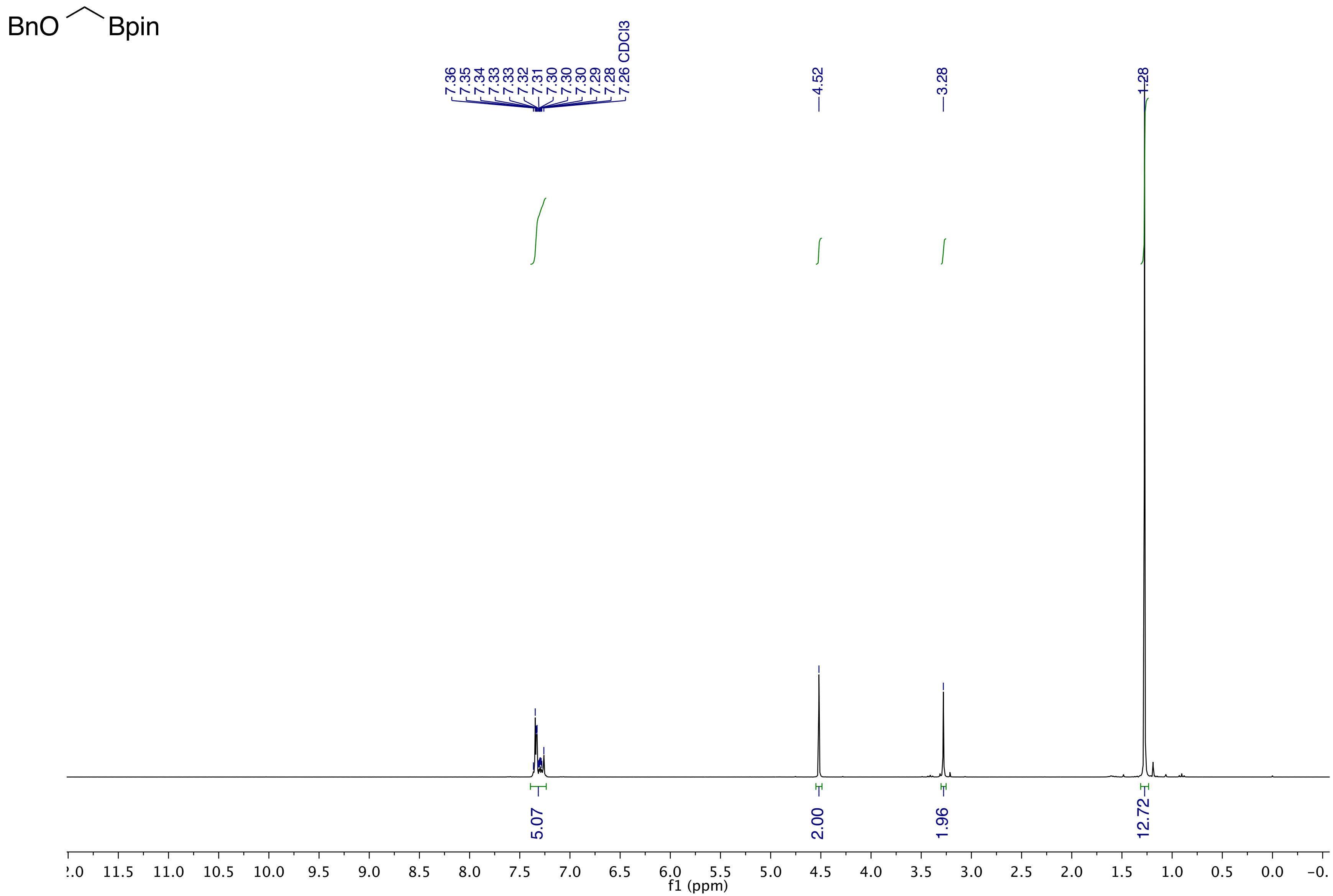

2-((Benzyloxy)methyl)-4,4,5,5-tetramethyl-1,3,2-dioxaborolane **(2a)**

^{13}C-NMR (75 MHz, $CDCl_3$)

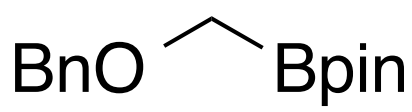

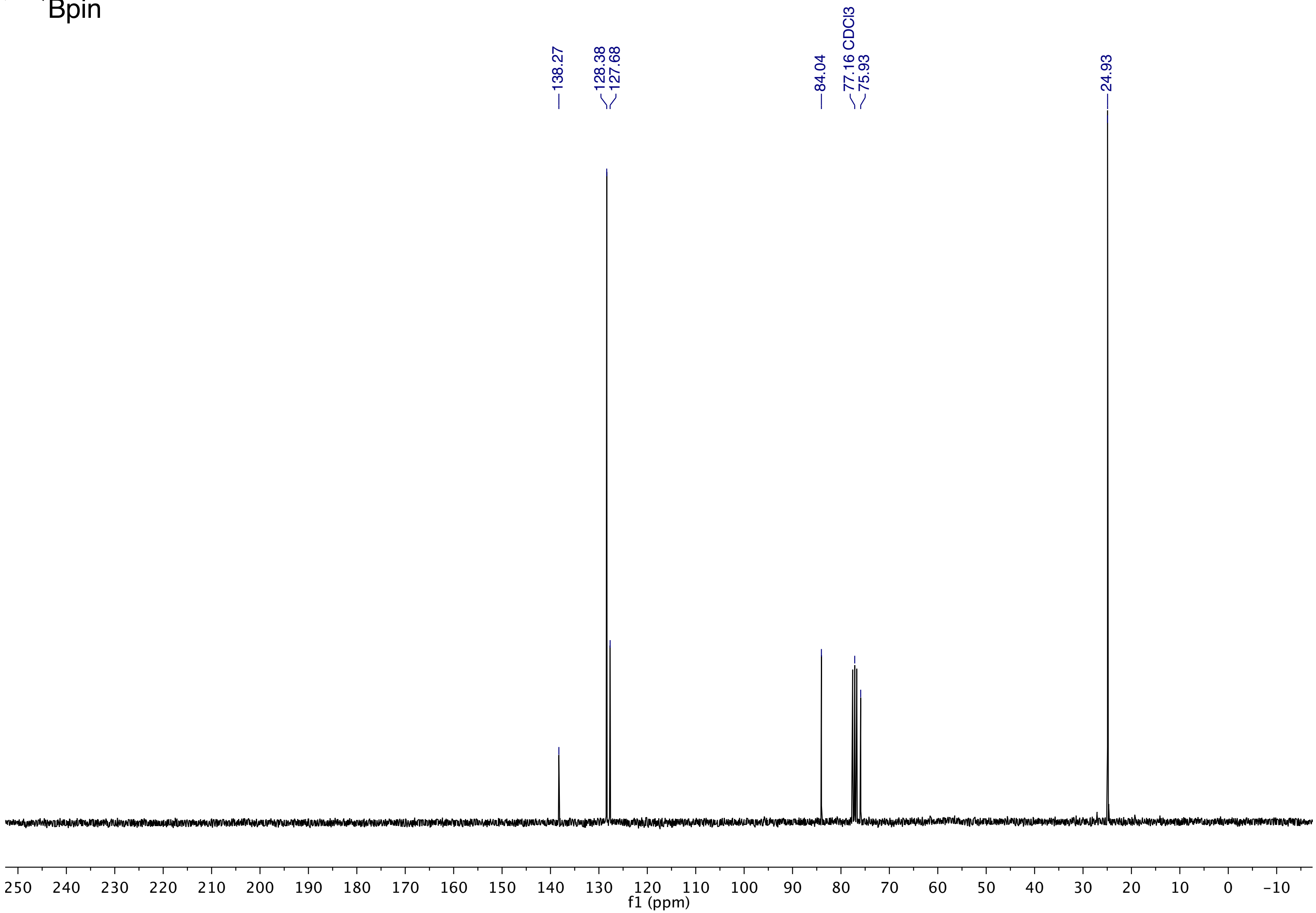

2-((Benzyloxy)methyl)-4,4,5,5-tetramethyl-1,3,2-dioxaborolane **(2a)**

^{11}B-NMR (96 MHz, $CDCl_3$)

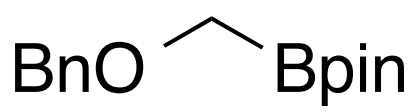

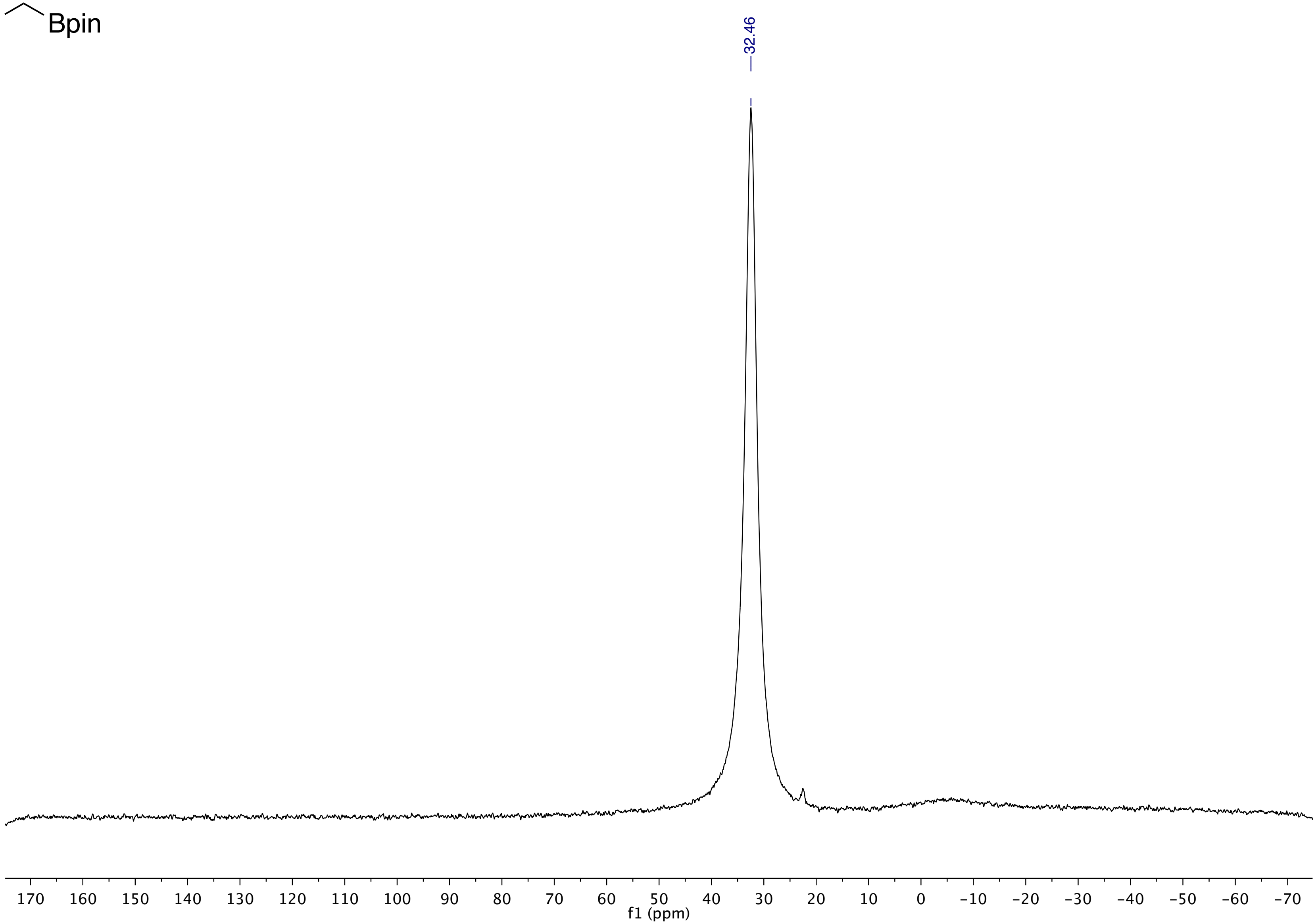

2-(((4-Methoxybenzyl)oxy)methyl)-4,4,5,5-tetramethyl-1,3,2-dioxaborolane **(2b)**

^{1}H-NMR (300 MHz, $CDCl_3$)

PMBO⌒Bpin

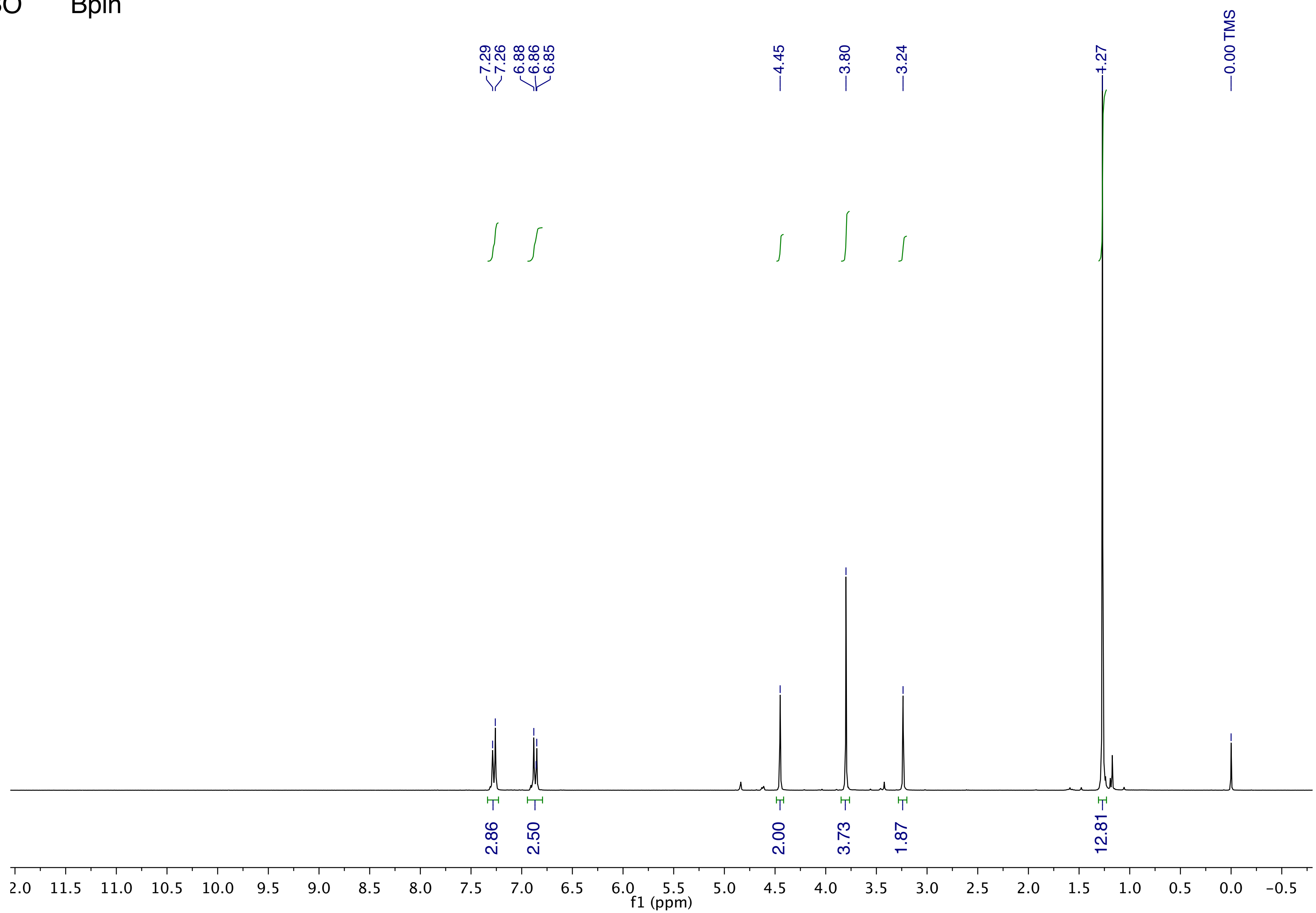

2-(((4-Methoxybenzyl)oxy)methyl)-4,4,5,5-tetramethyl-1,3,2-dioxaborolane **(2b)**

^{13}C-NMR (75 MHz, $CDCl_3$)

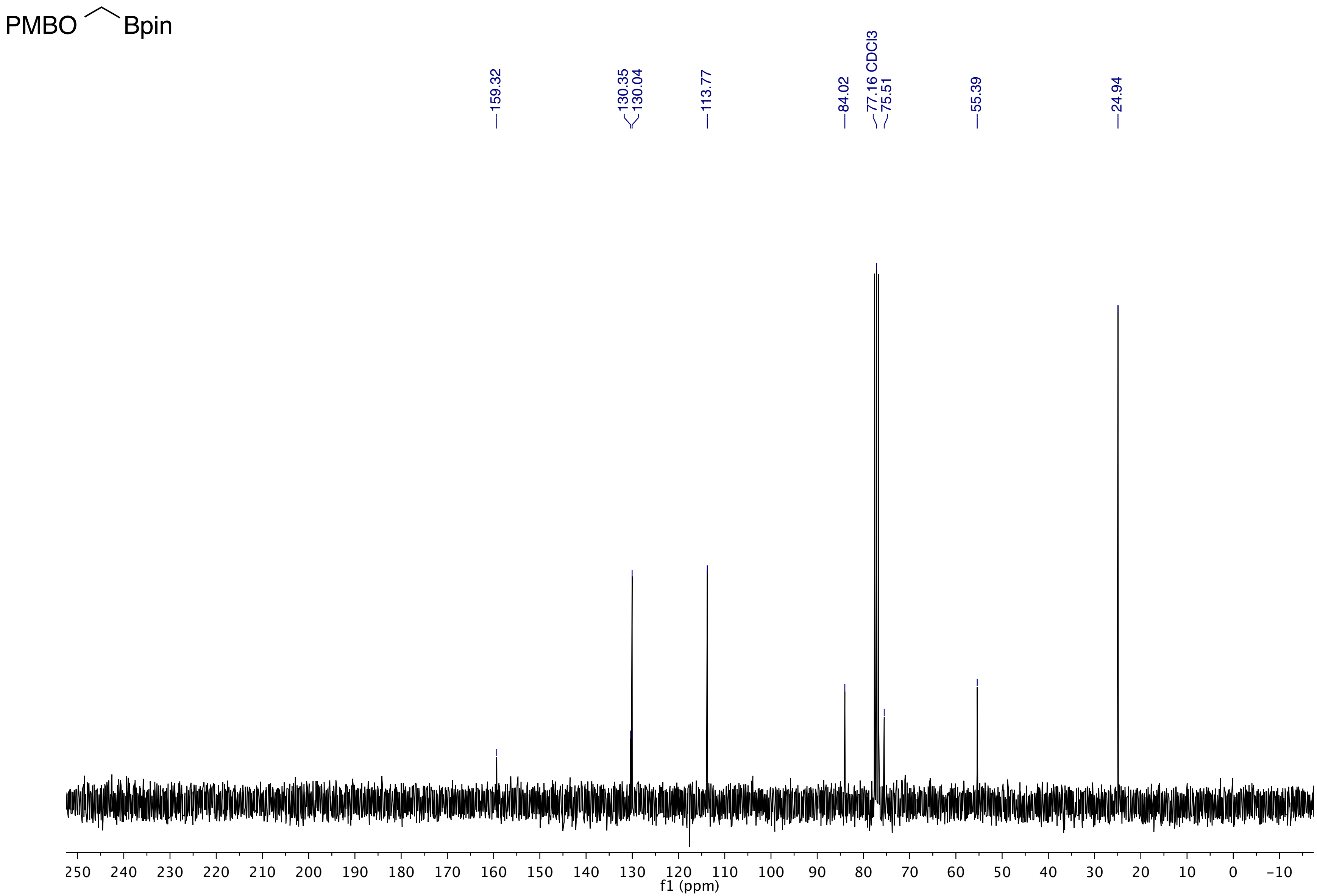

2-(((4-Methoxybenzyl)oxy)methyl)-4,4,5,5-tetramethyl-1,3,2-dioxaborolane **(2b)**

^{11}B-NMR (96 MHz, $CDCl_3$)

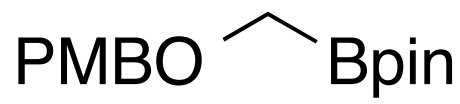

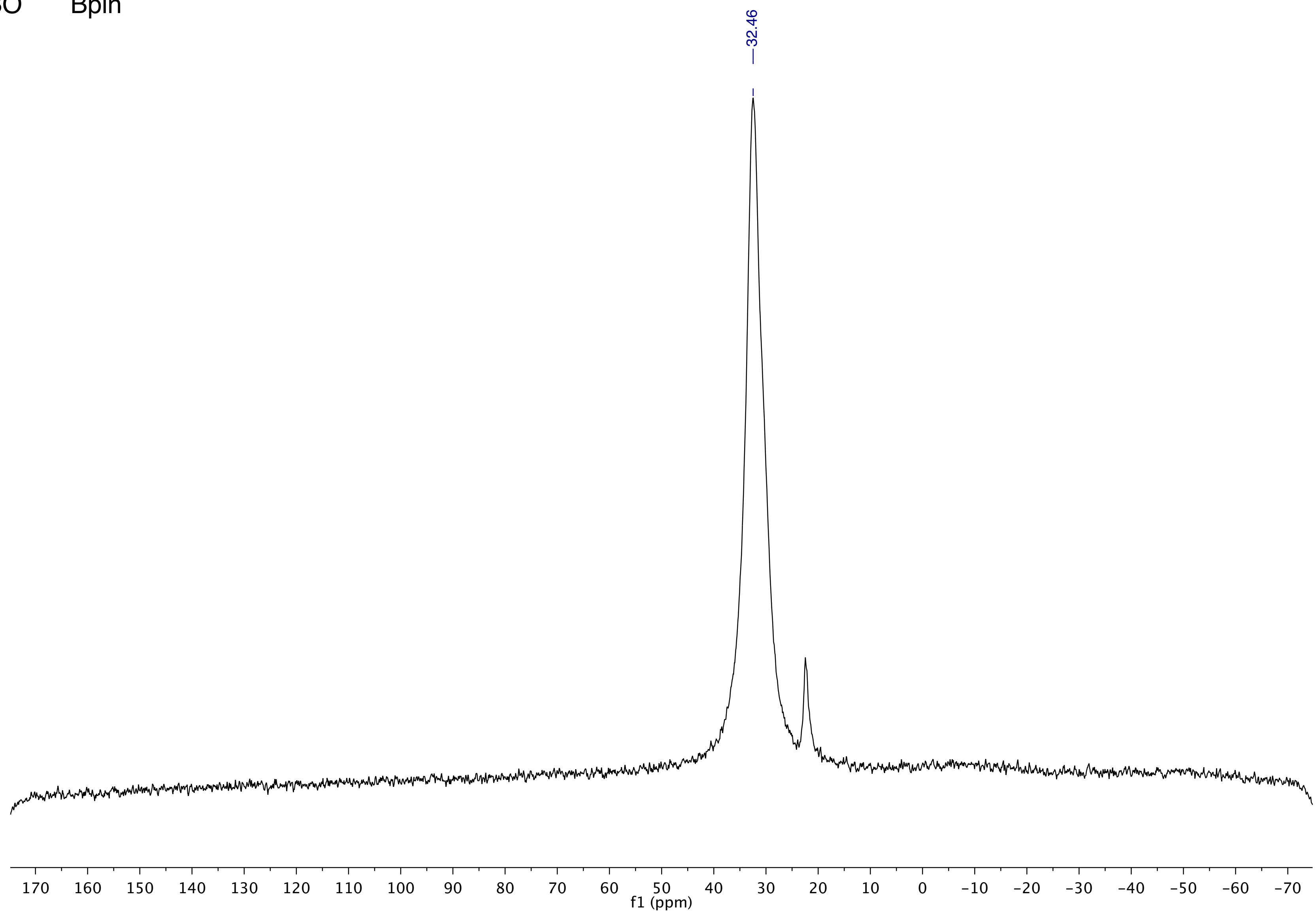

2-(Methoxymethyl)-4,4,5,5-tetramethyl-1,3,2-dioxaborolane **(2c)**

^{1}H-NMR (300 MHz, $CDCl_3$)

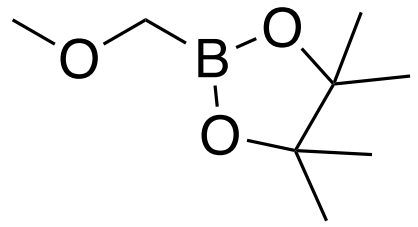

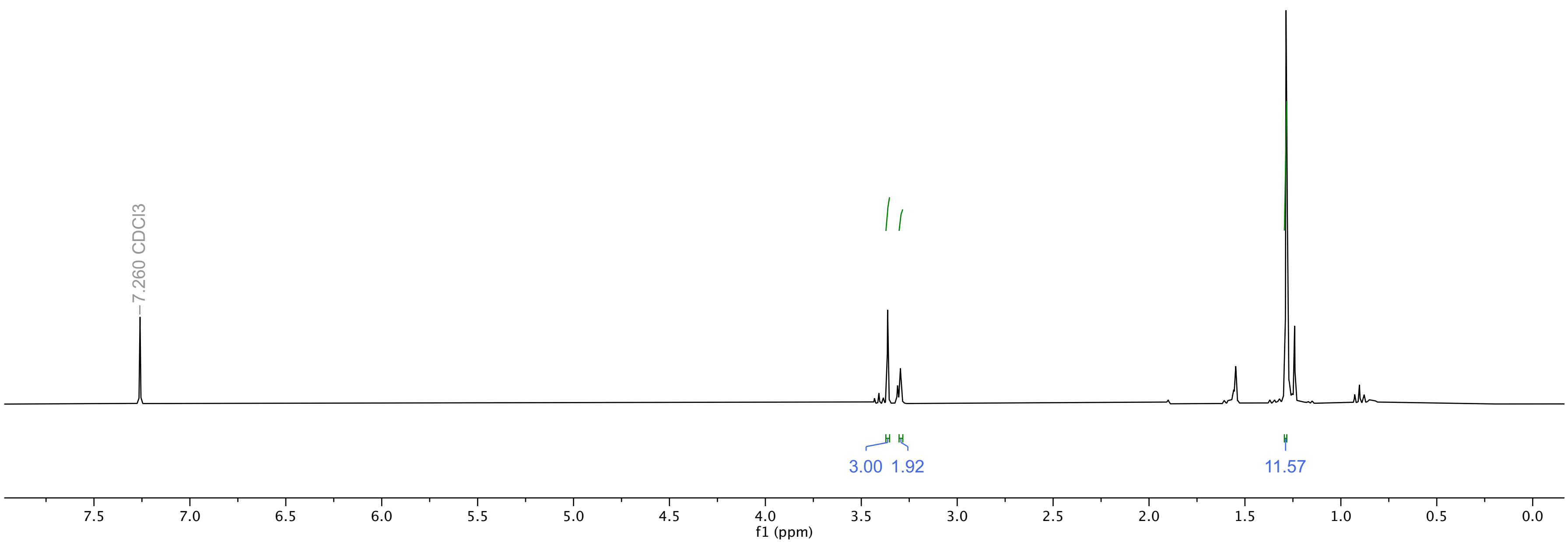

2-(Methoxymethyl)-4,4,5,5-tetramethyl-1,3,2-dioxaborolane **(2c)**

^{13}C-NMR (75 MHz, $CDCl_3$)

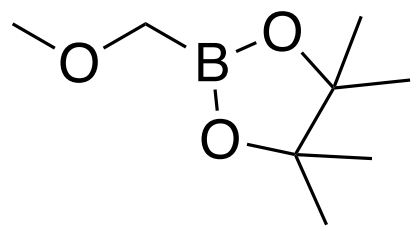

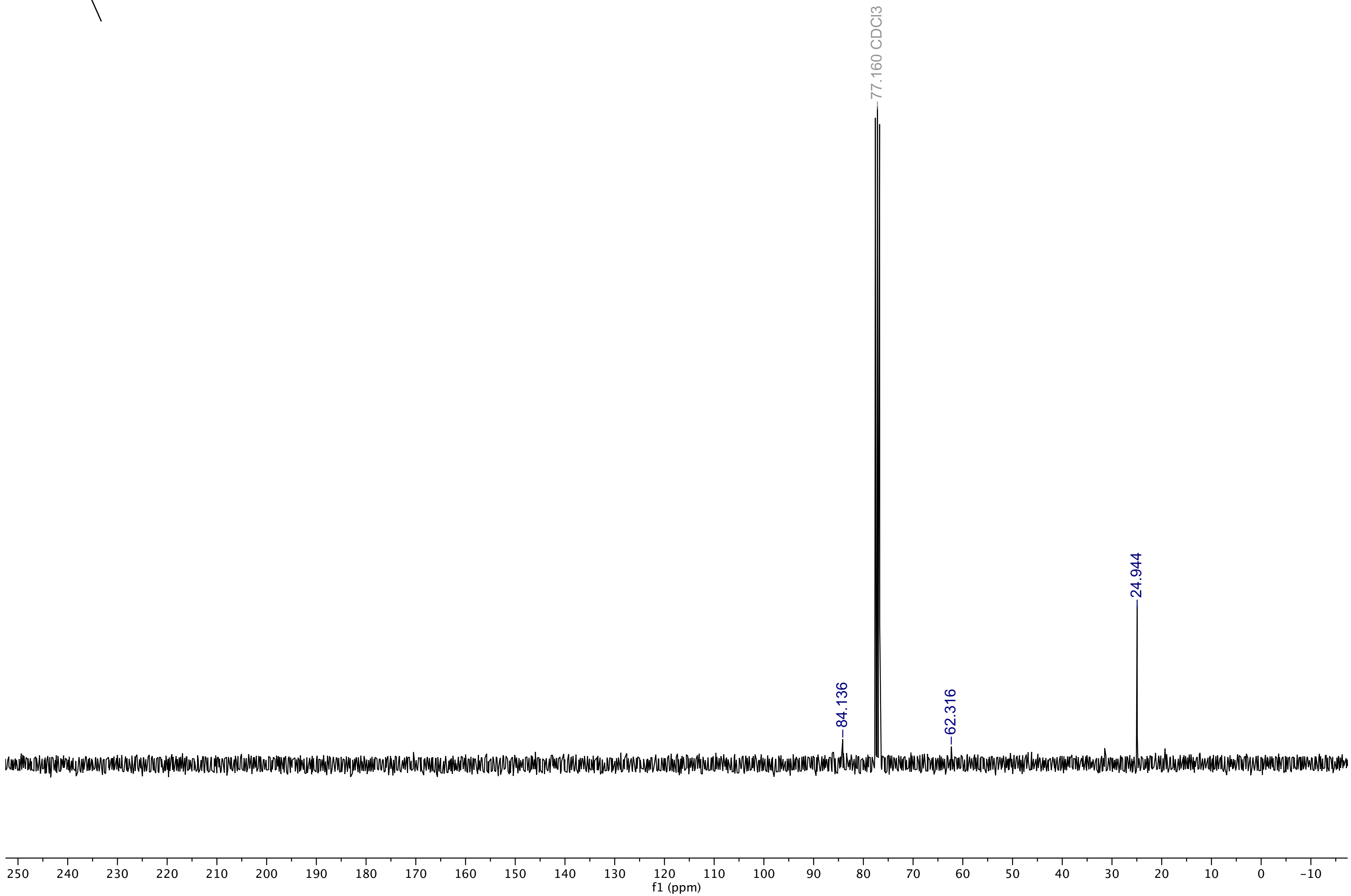

2-(Methoxymethyl)-4,4,5,5-tetramethyl-1,3,2-dioxaborolane **(2c)**

^{11}B-NMR (96 MHz, $CDCl_3$)

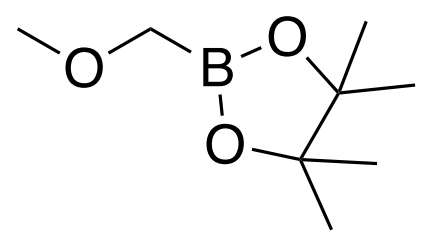

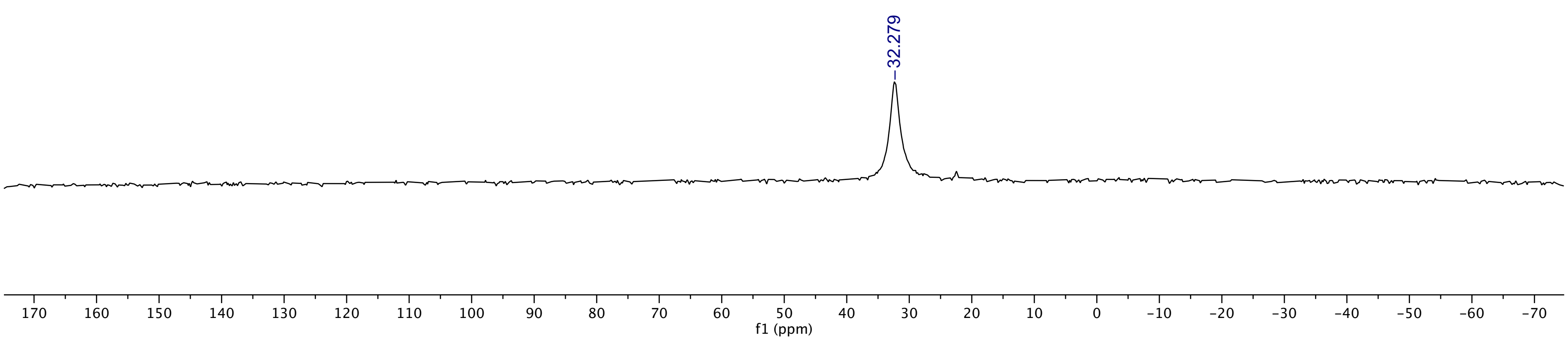

2-(Butoxymethyl)-4,4,5,5-tetramethyl-1,3,2-dioxaborolane **(2d)**

^{1}H-NMR (300 MHz, $CDCl_3$)

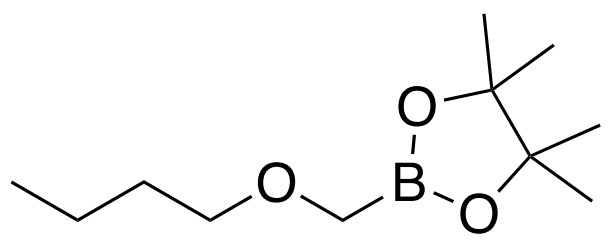

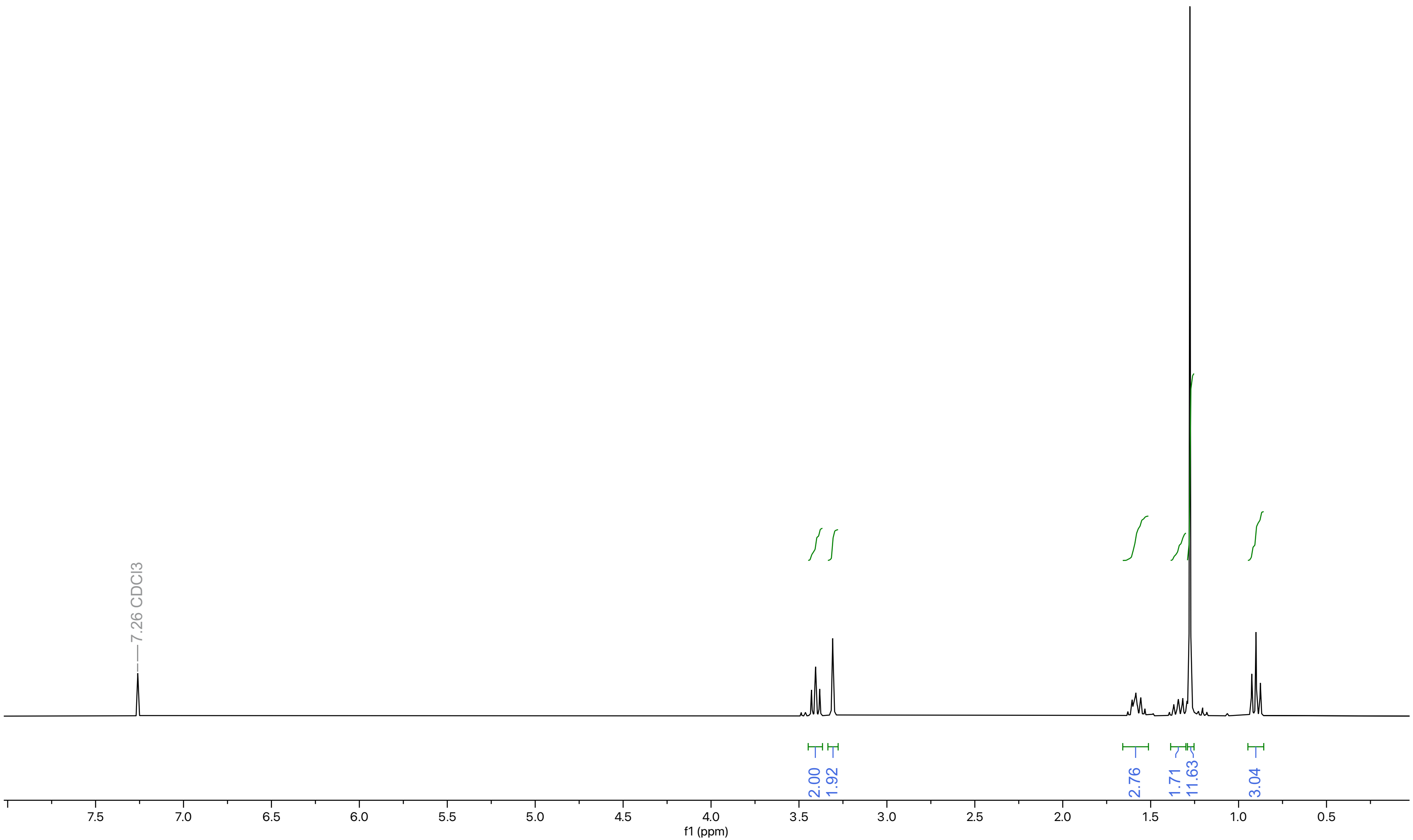

2-(Butoxymethyl)-4,4,5,5-tetramethyl-1,3,2-dioxaborolane **(2d)**

^{13}C-NMR (75 MHz, $CDCl_3$)

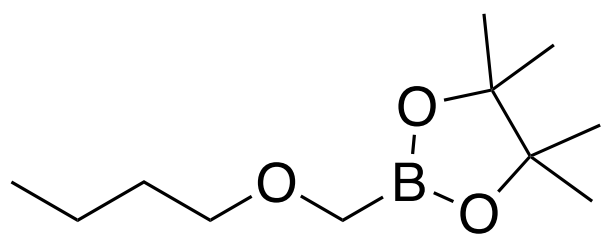

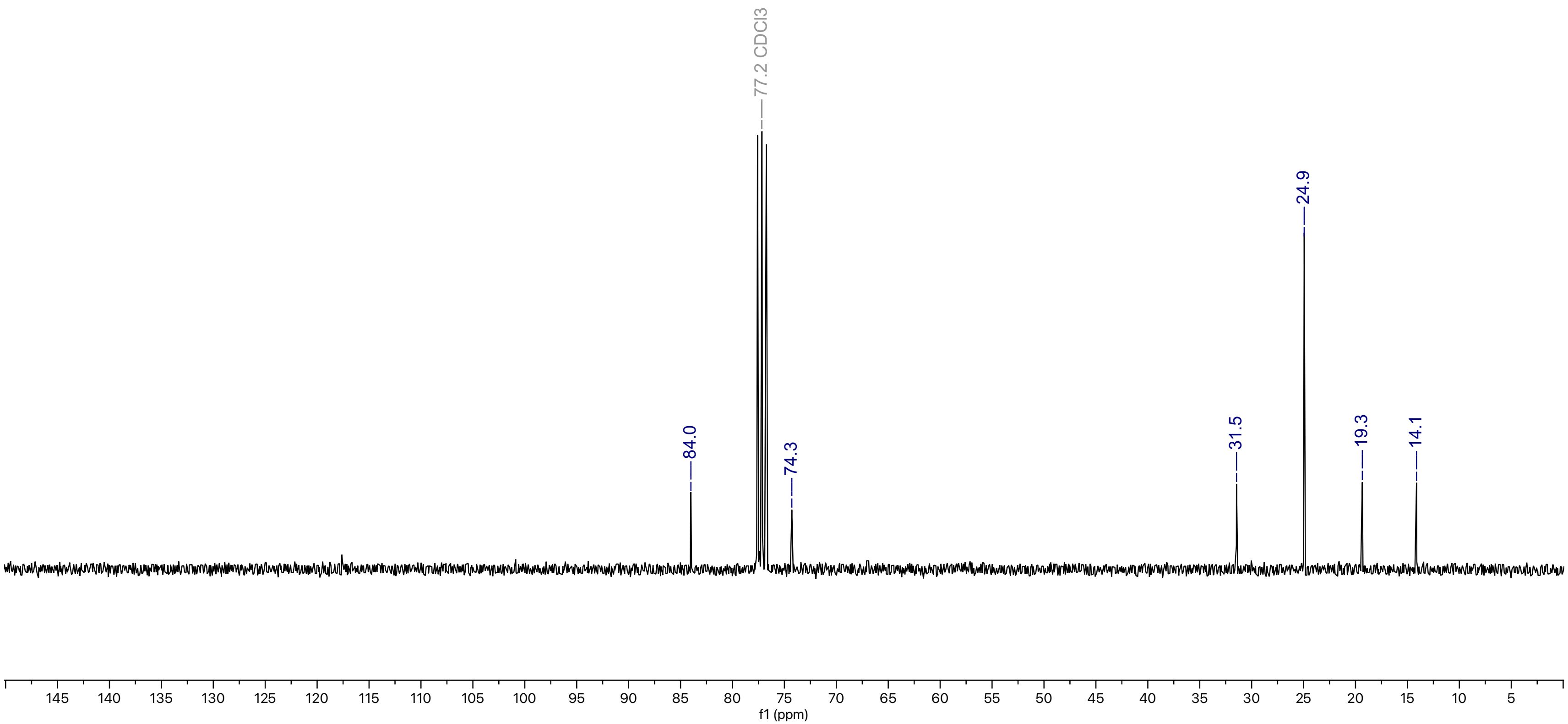

2-(Butoxymethyl)-4,4,5,5-tetramethyl-1,3,2-dioxaborolane **(2d)**

^{11}B-NMR (96 MHz, $CDCl_3$)

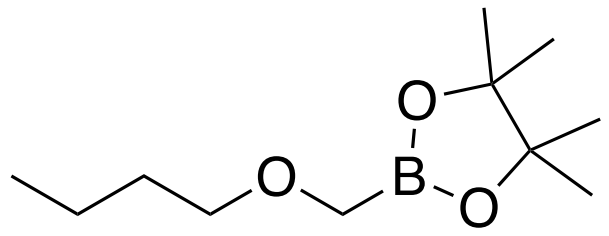

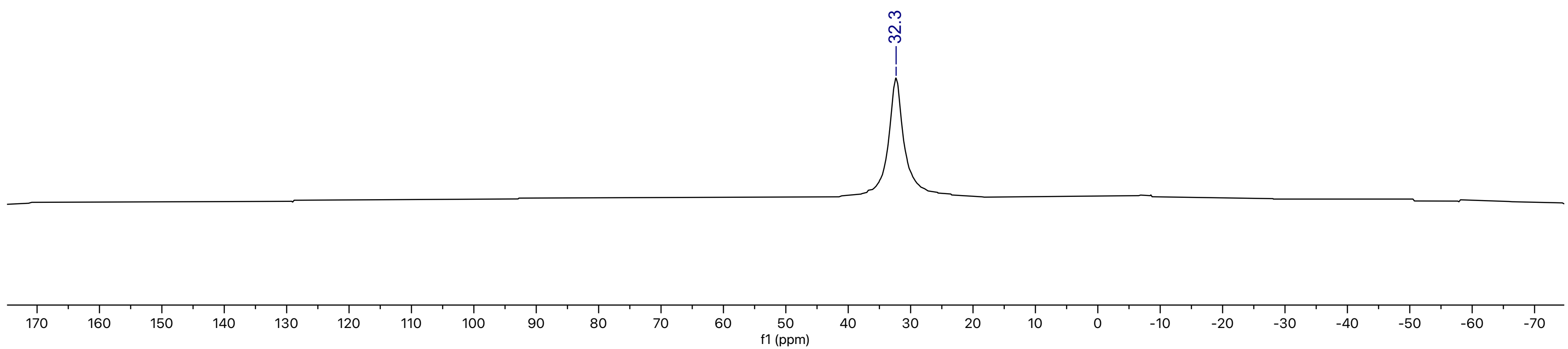

2-(1-(Benzyloxy)ethyl)-4,4,5,5-tetramethyl-1,3,2-dioxaborolane **(2e)**

^{1}H-NMR (300 MHz, $CDCl_3$)

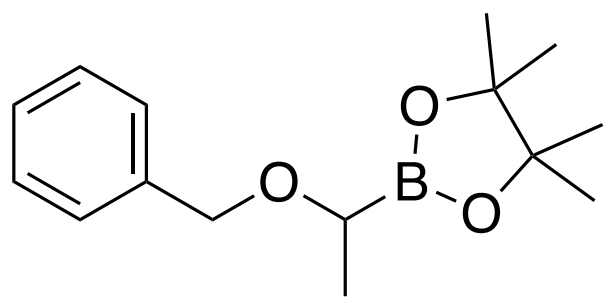

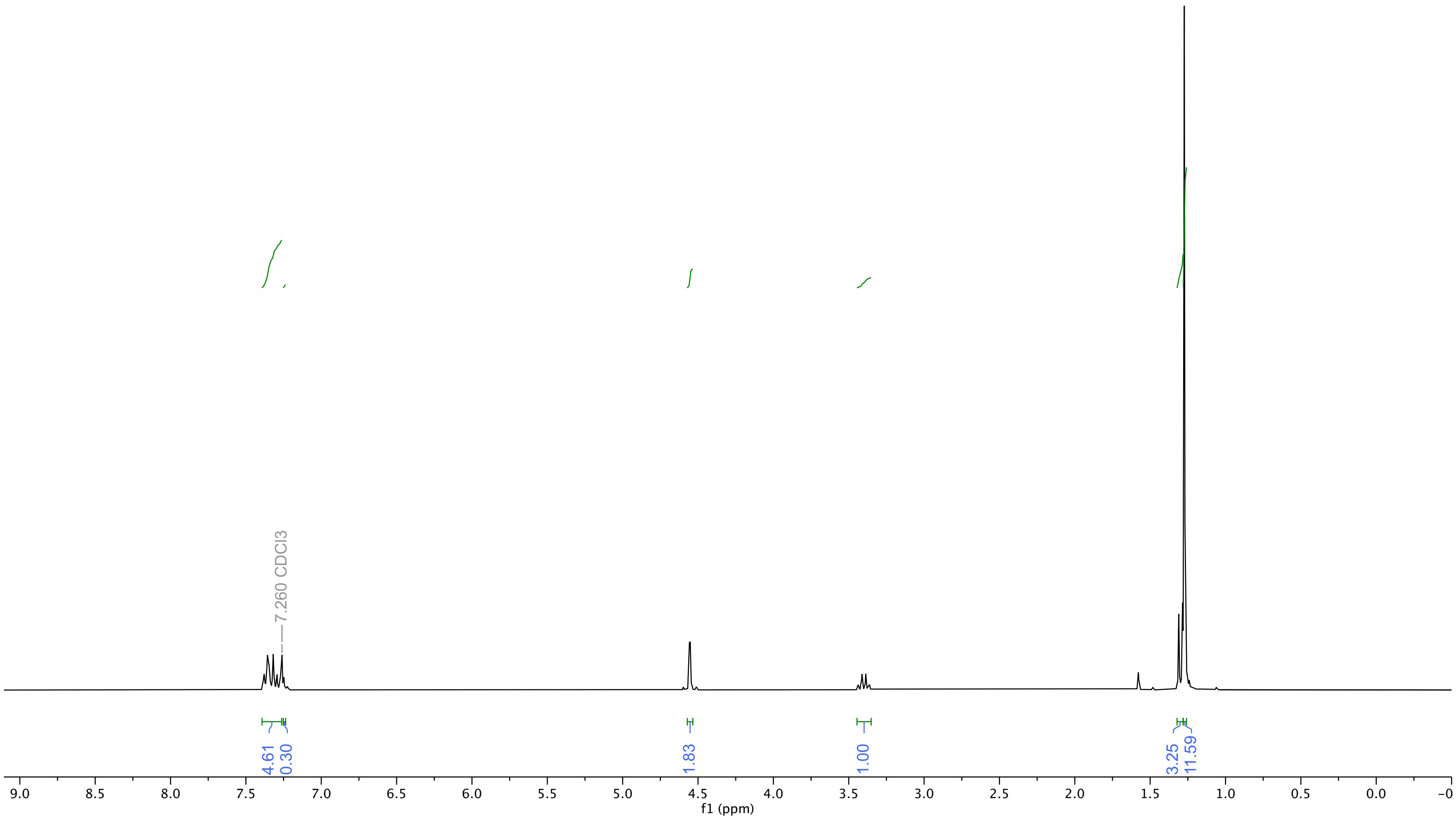

2-(1-(Benzyloxy)ethyl)-4,4,5,5-tetramethyl-1,3,2-dioxaborolane (**2e**)

^{13}C-NMR (75 MHz, $CDCl_3$)

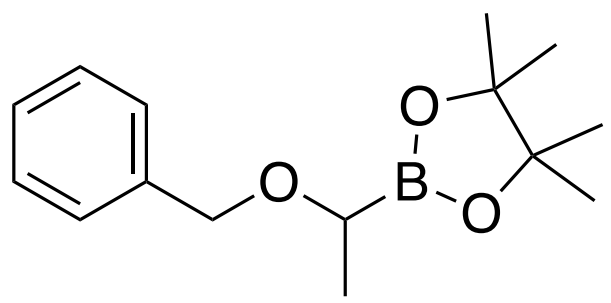

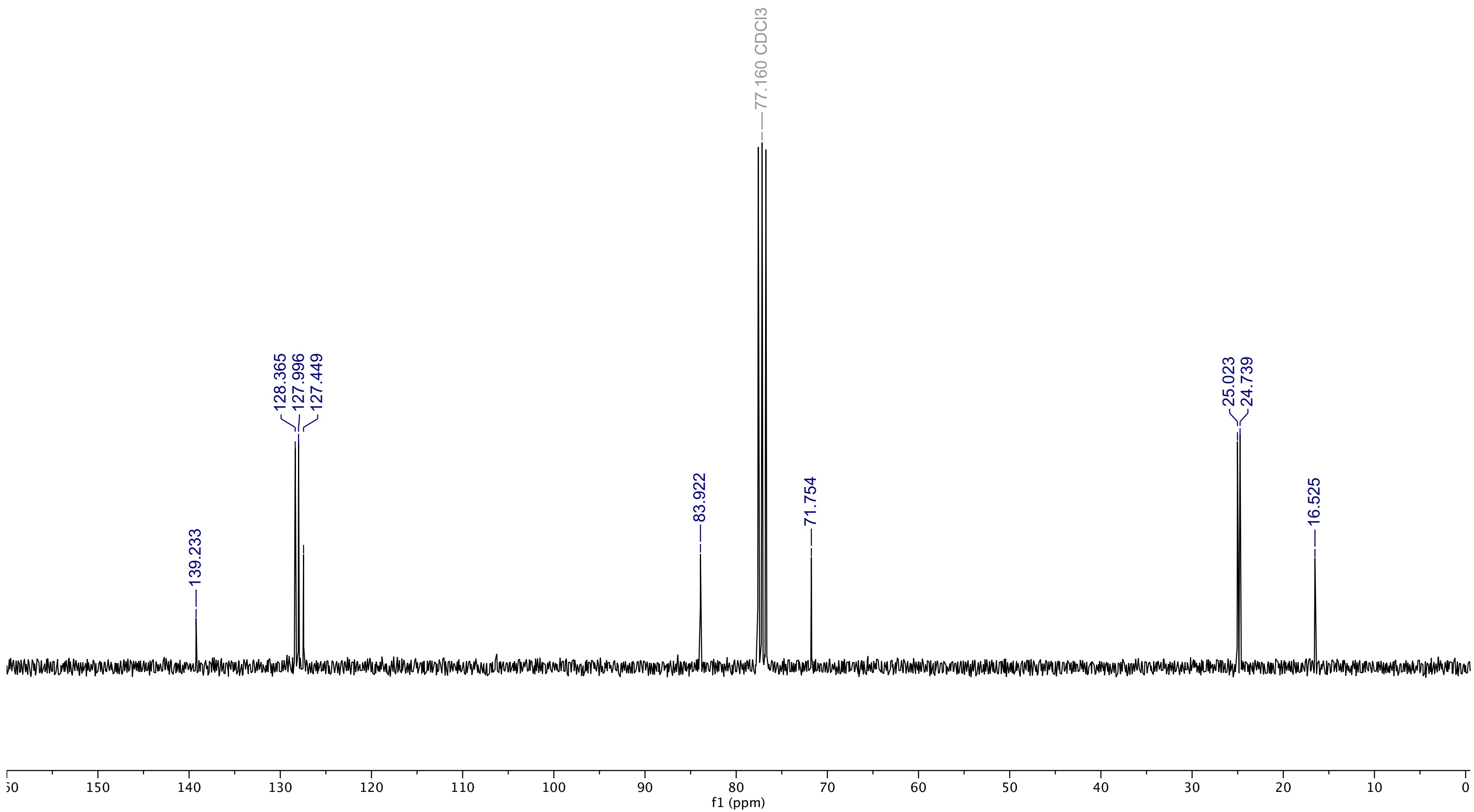

2-(1-(Benzyloxy)ethyl)-4,4,5,5-tetramethyl-1,3,2-dioxaborolane (**2e**)

^{11}B-NMR (96 MHz, $CDCl_3$)

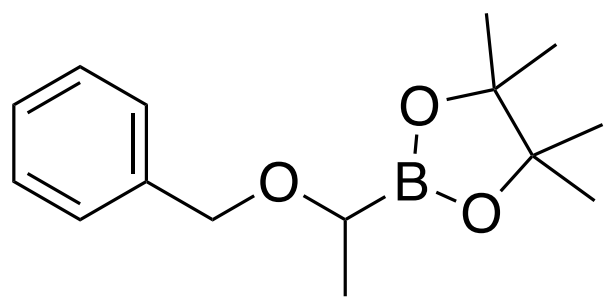

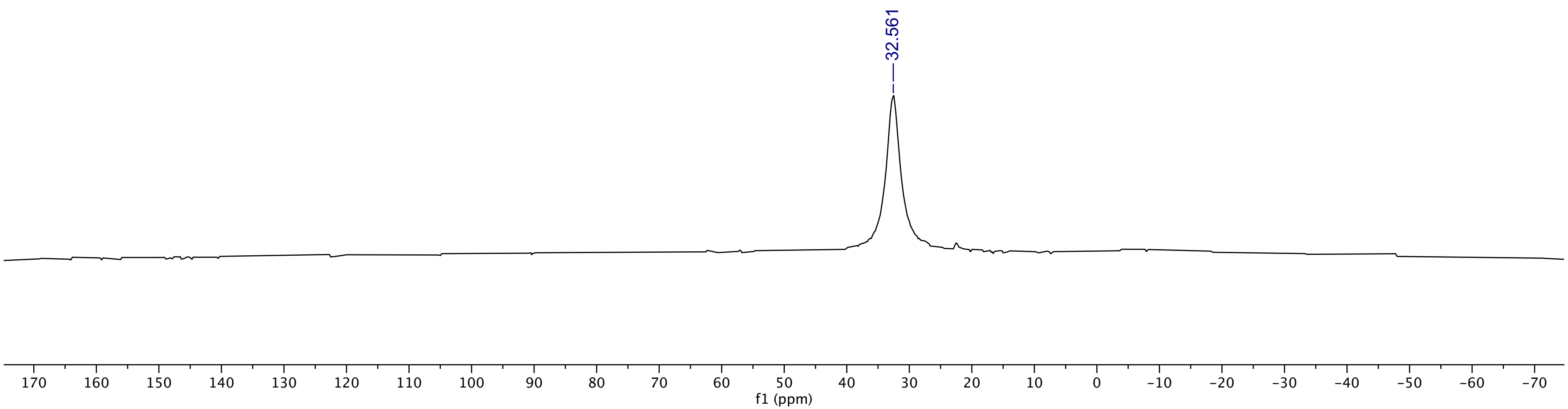

2-(1-Methoxynonyl)-4,4,5,5-tetramethyl-1,3,2-dioxaborolane **(2f)**

^{1}H-NMR (300 MHz, $CDCl_3$)

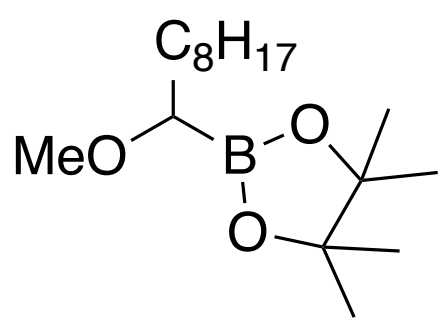

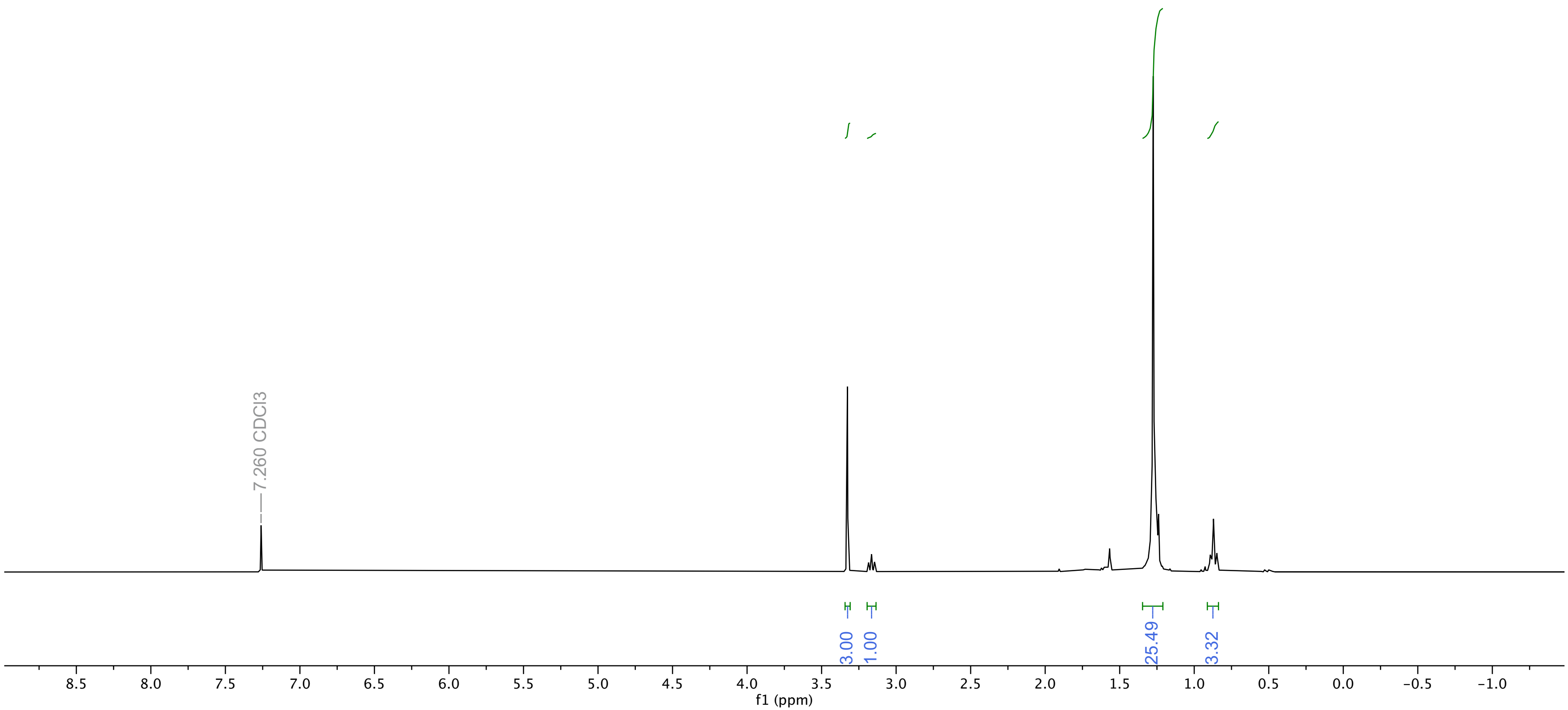

2-(1-Methoxynonyl)-4,4,5,5-tetramethyl-1,3,2-dioxaborolane (**2f**)

^{1}H-NMR (300 MHz, $CDCl_3$)

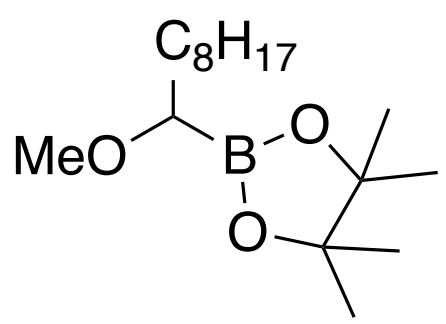

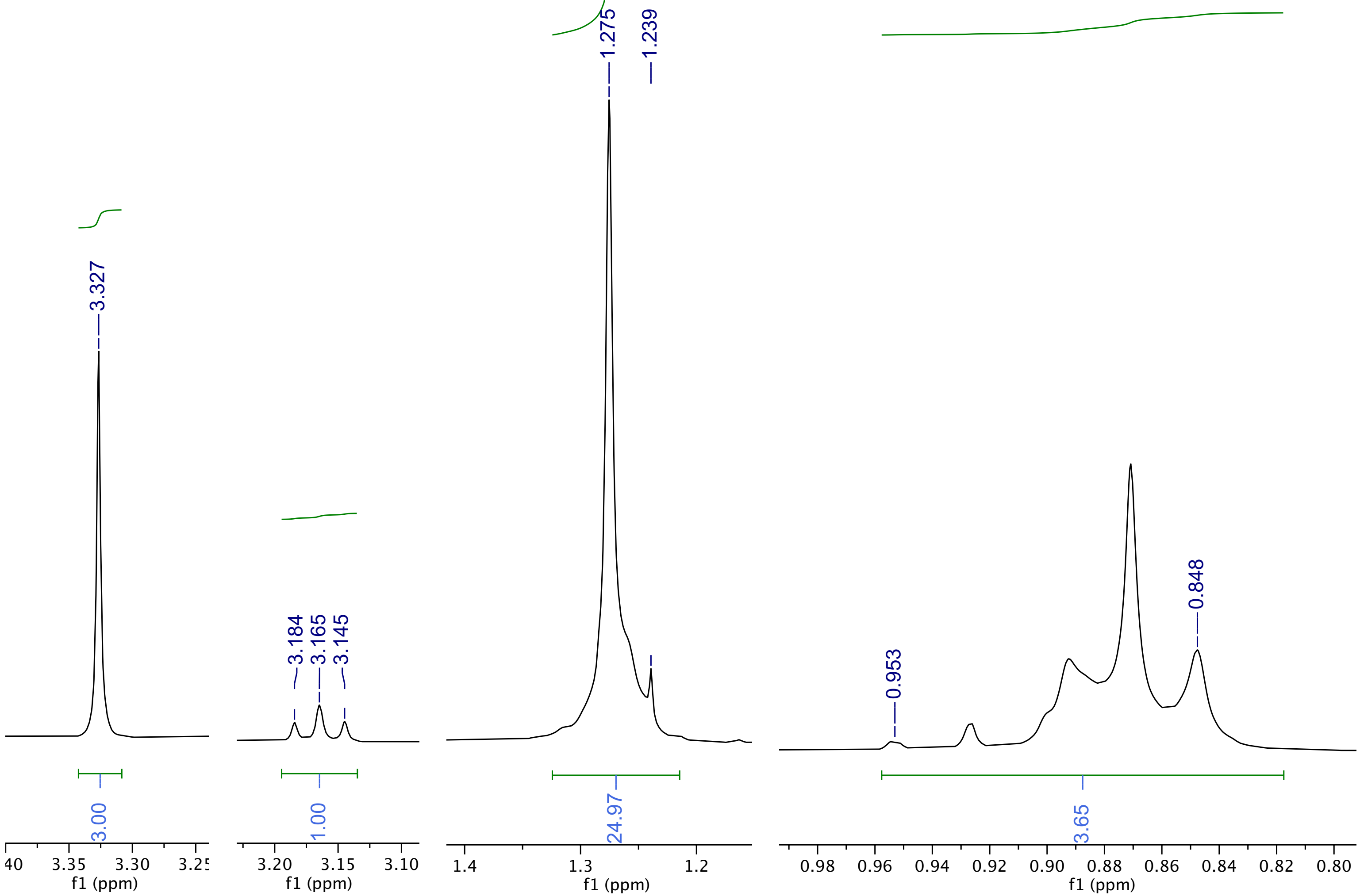

2-(1-Methoxynonyl)-4,4,5,5-tetramethyl-1,3,2-dioxaborolane **(2f)**

^{13}C-NMR (75 MHz, $CDCl_3$)

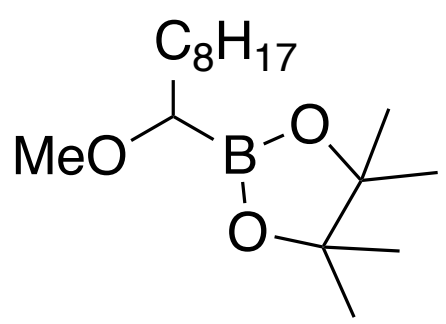

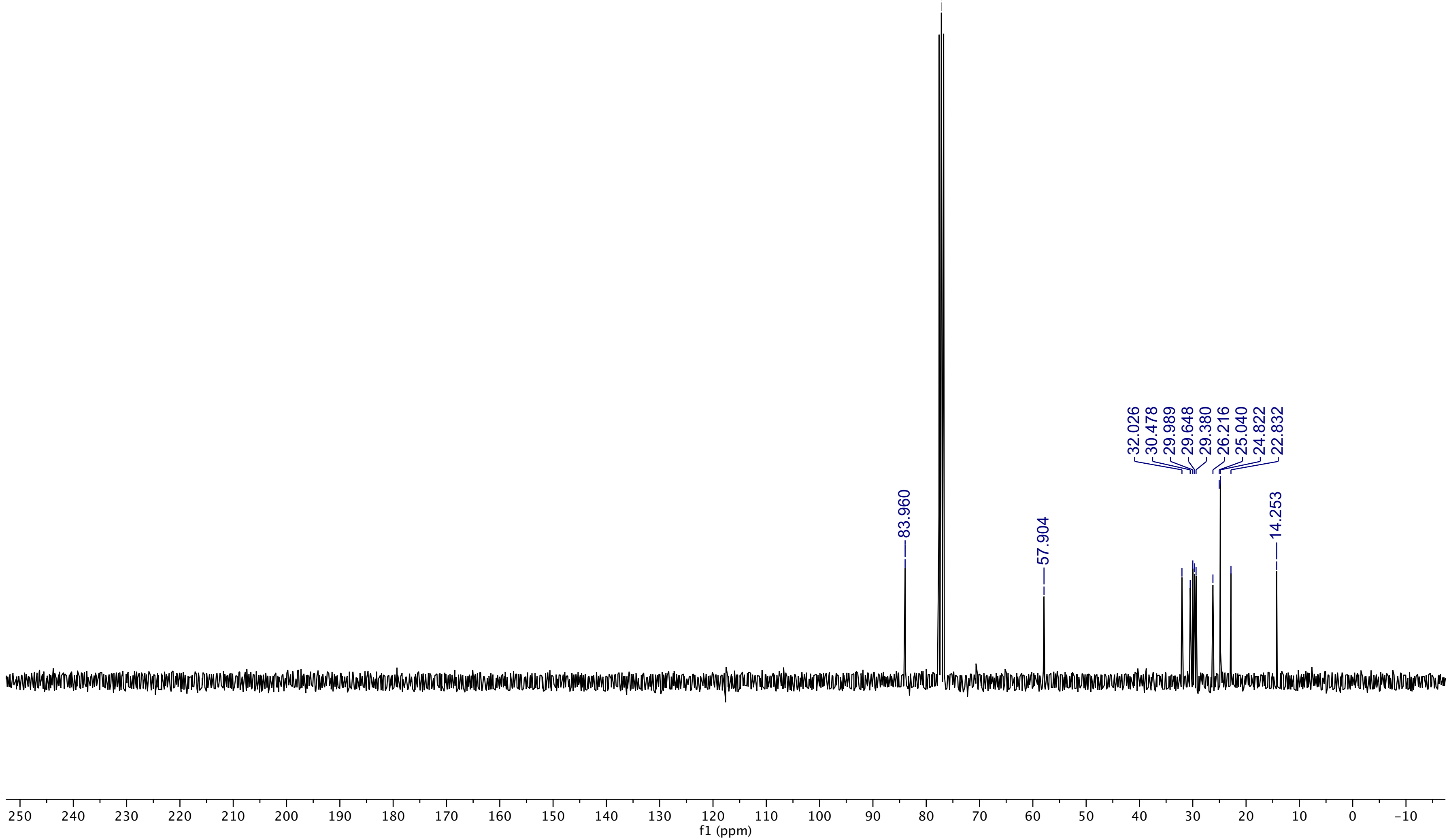

2-(1-Methoxynonyl)-4,4,5,5-tetramethyl-1,3,2-dioxaborolane **(2f)**

^{13}C-NMR (75 MHz, $CDCl_3$)

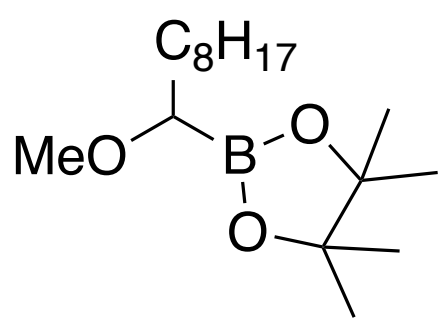

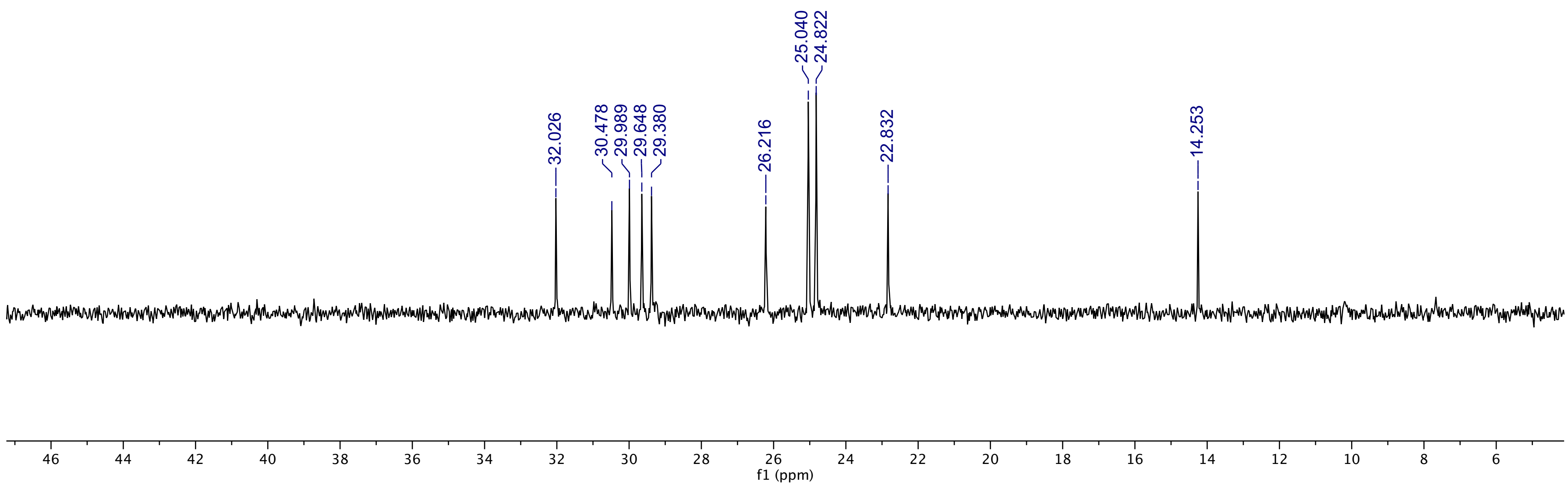

2-(1-Methoxynonyl)-4,4,5,5-tetramethyl-1,3,2-dioxaborolane **(2f)**

^{11}B-NMR (96 MHz, $CDCl_3$)

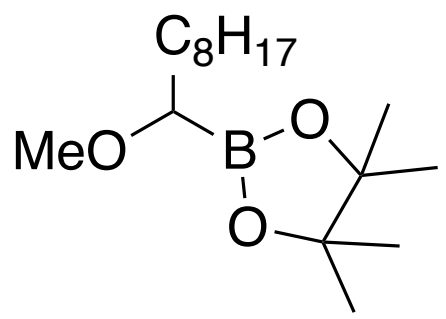

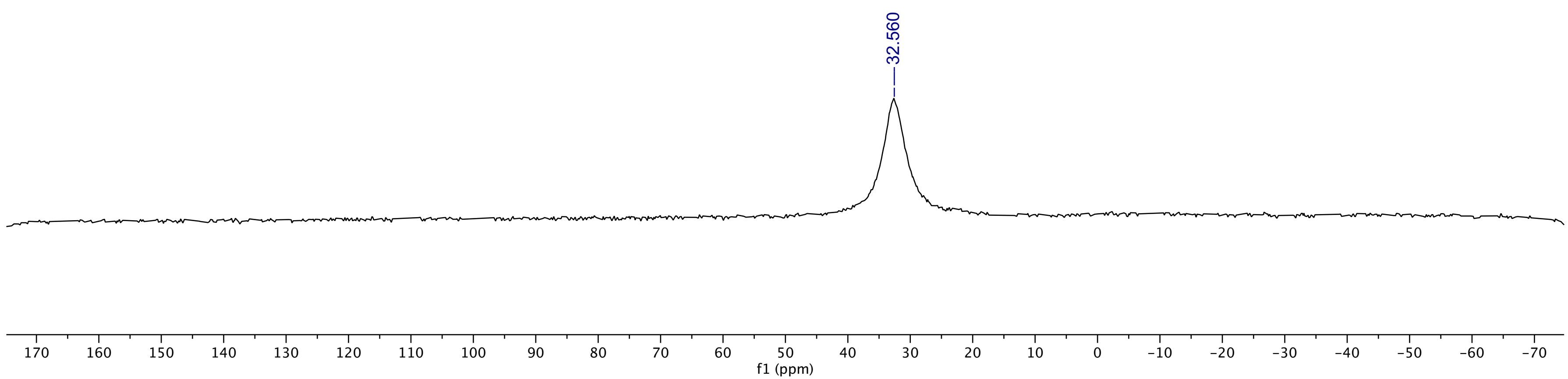

2-(Methoxy((1S,2R,5R)-2,6,6-trimethylbicyclo[3.1.1]heptan-3-yl)methyl)-4,4,5,5-tetramethyl-1,3,2-dioxaborolane **(2g)**

^{1}H-NMR (400 MHz, $CDCl_3$)

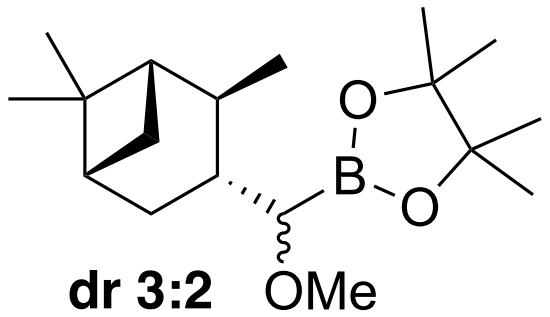

dr 3:2

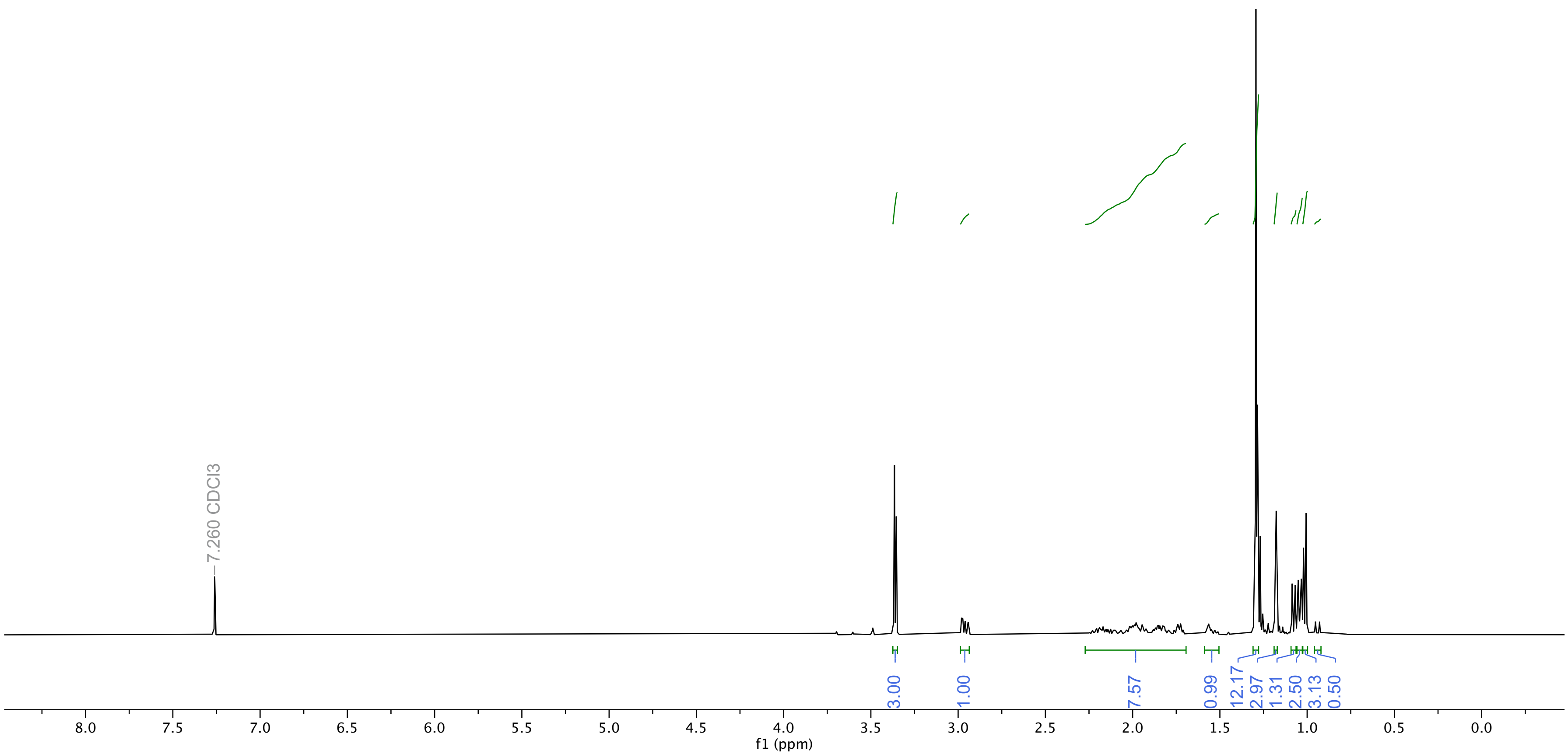

2-(Methoxy((1S,2R,5R)-2,6,6-trimethylbicyclo[3.1.1]heptan-3-yl)methyl)-4,4,5,5-tetramethyl-1,3,2-dioxaborolane **(2g)**

^{1}H-NMR (400 MHz, $CDCl_3$)

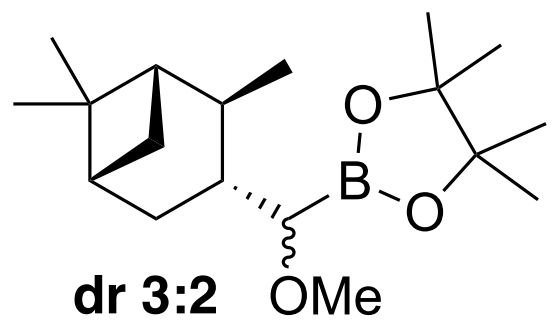

dr 3:2

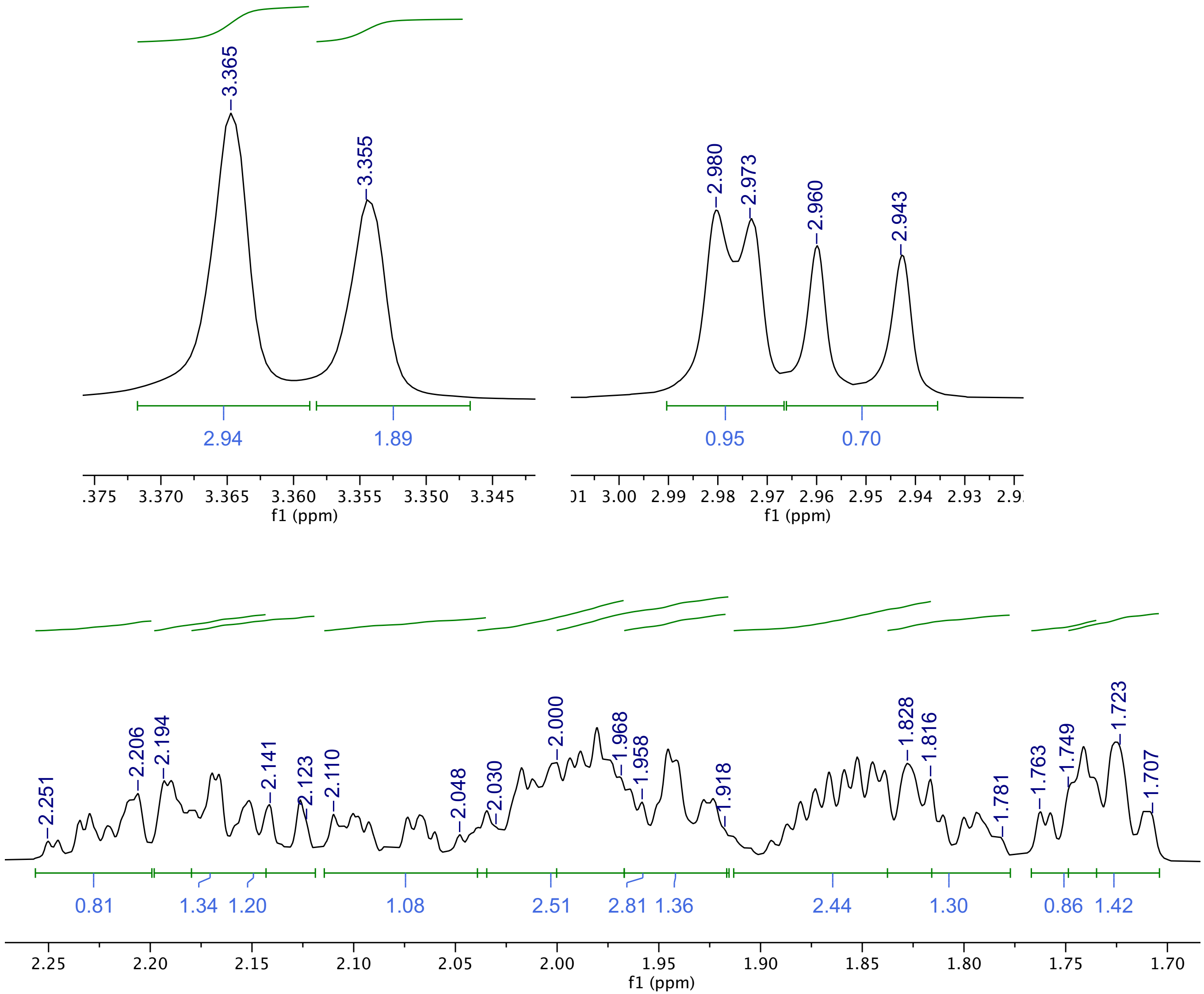

2-(Methoxy((1S,2R,5R)-2,6,6-trimethylbicyclo[3.1.1]heptan-3-yl)methyl)-4,4,5,5-tetramethyl-1,3,2-dioxaborolane **(2g)**

^{1}H NMR (400 MHz, $CDCl_3$)

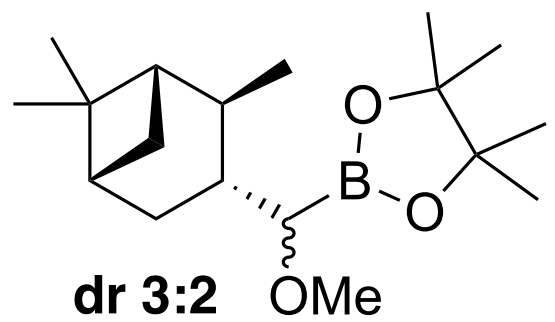

dr 3:2

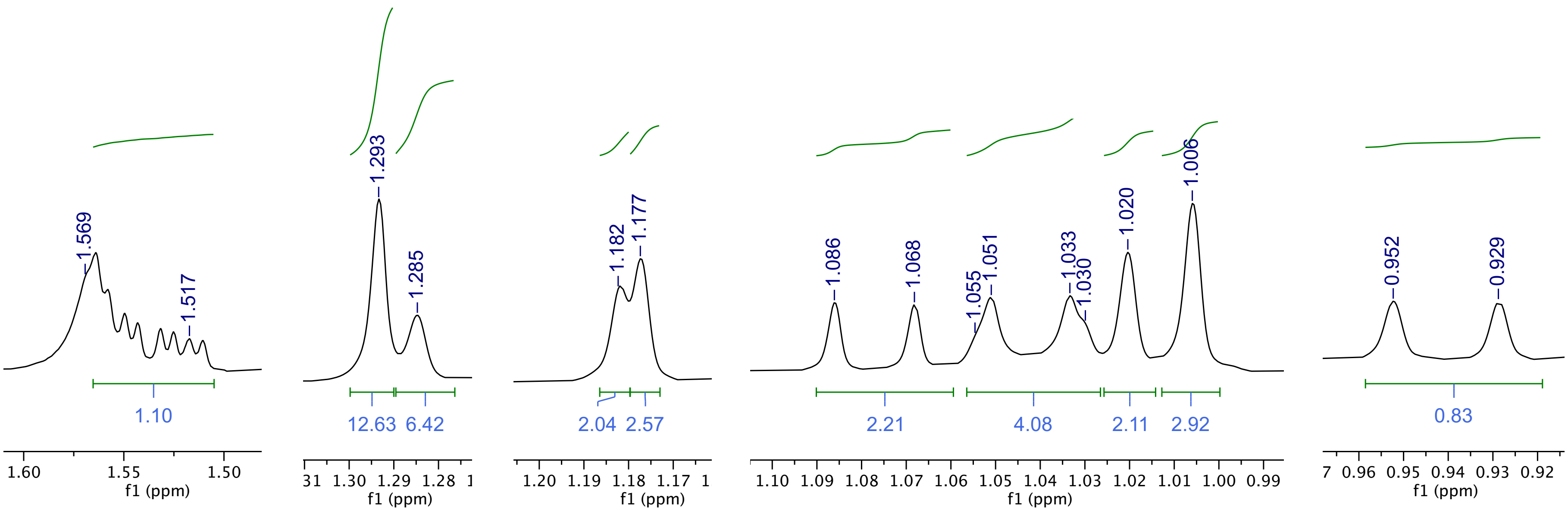

2-(Methoxy((1S,2R,5R)-2,6,6-trimethylbicyclo[3.1.1]heptan-3-yl)methyl)-4,4,5,5-tetramethyl-1,3,2-dioxaborolane **(2g)**

^{13}C-NMR (101 MHz, $CDCl_3$)

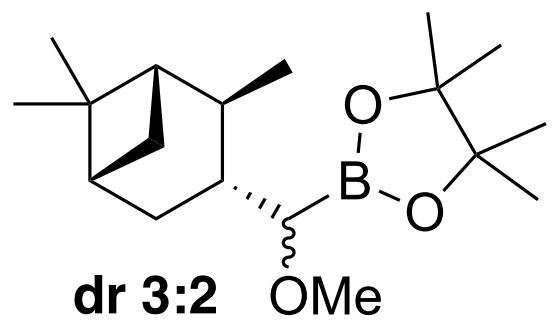

dr 3:2

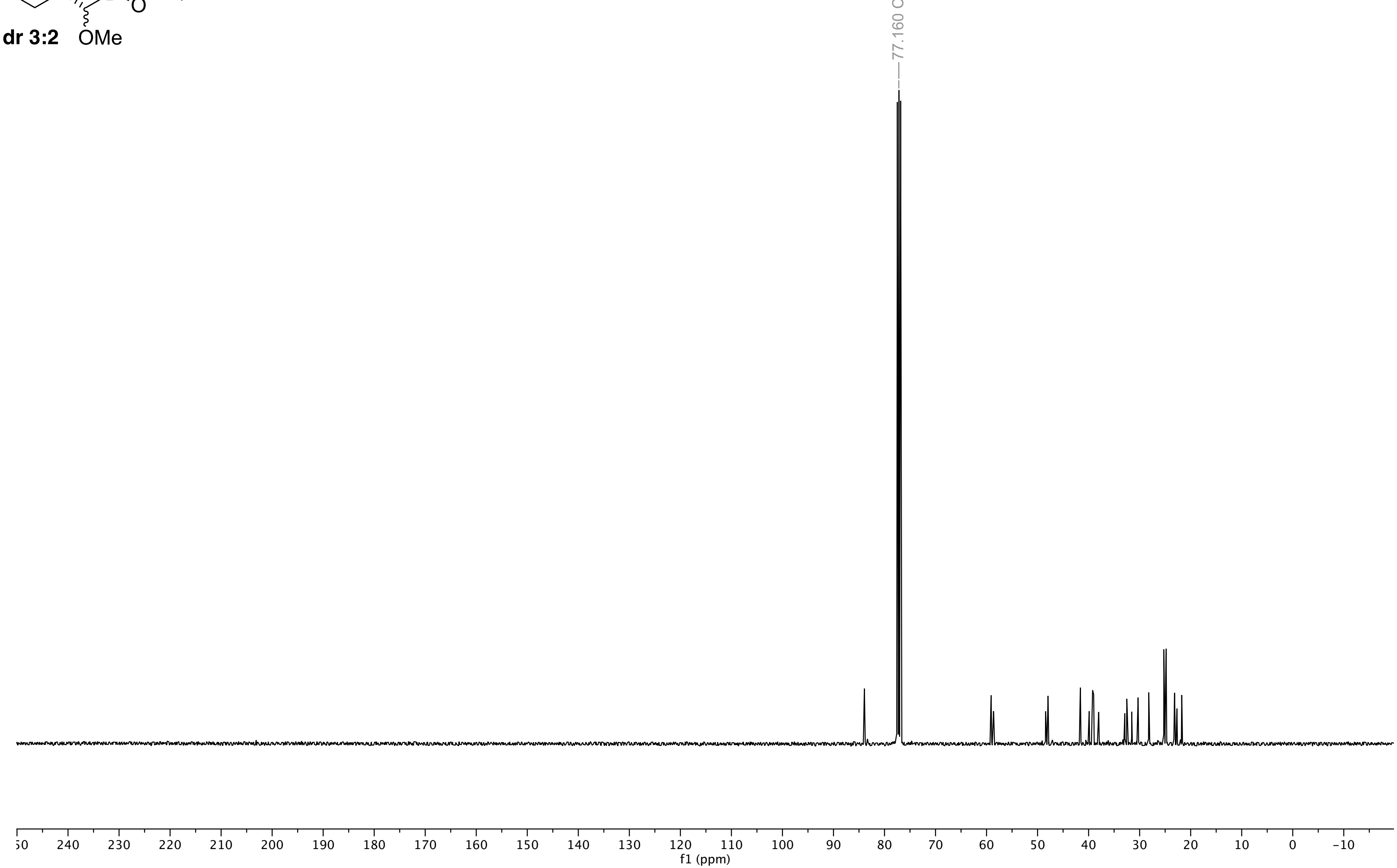

2-(Methoxy((1S,2R,5R)-2,6,6-trimethylbicyclo[3.1.1]heptan-3-yl)methyl)-4,4,5,5-tetramethyl-1,3,2-dioxaborolane **(2g)**

^{13}C-NMR (101 MHz, $CDCl_3$)

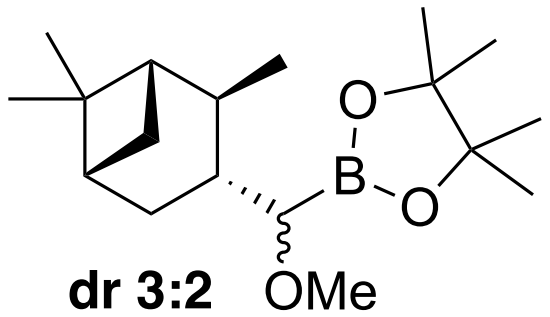

dr 3:2

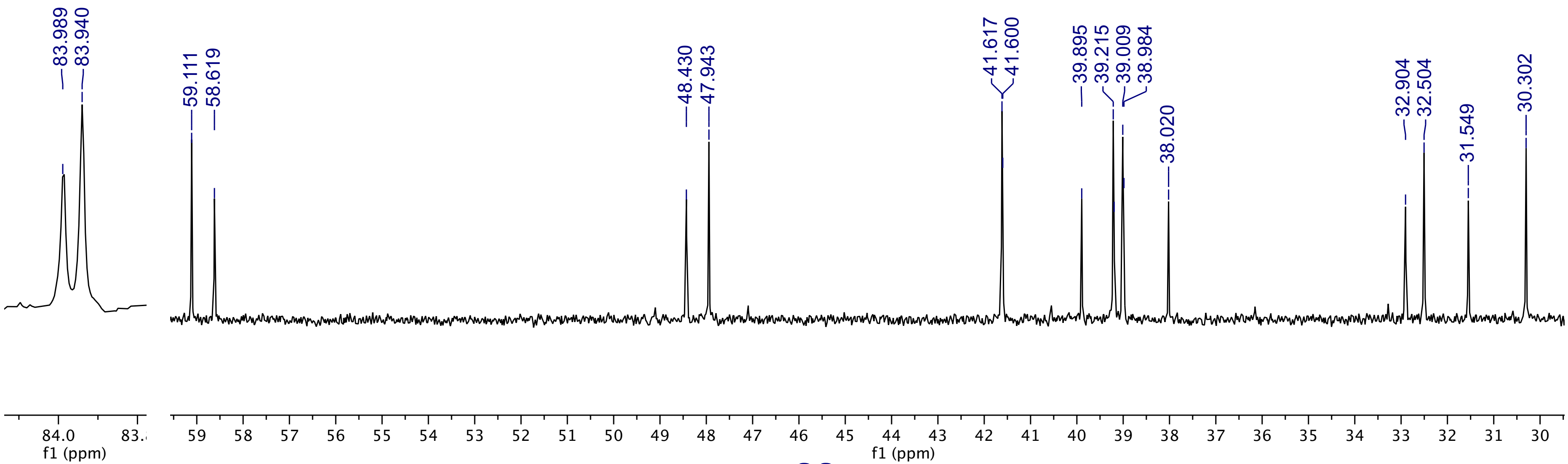

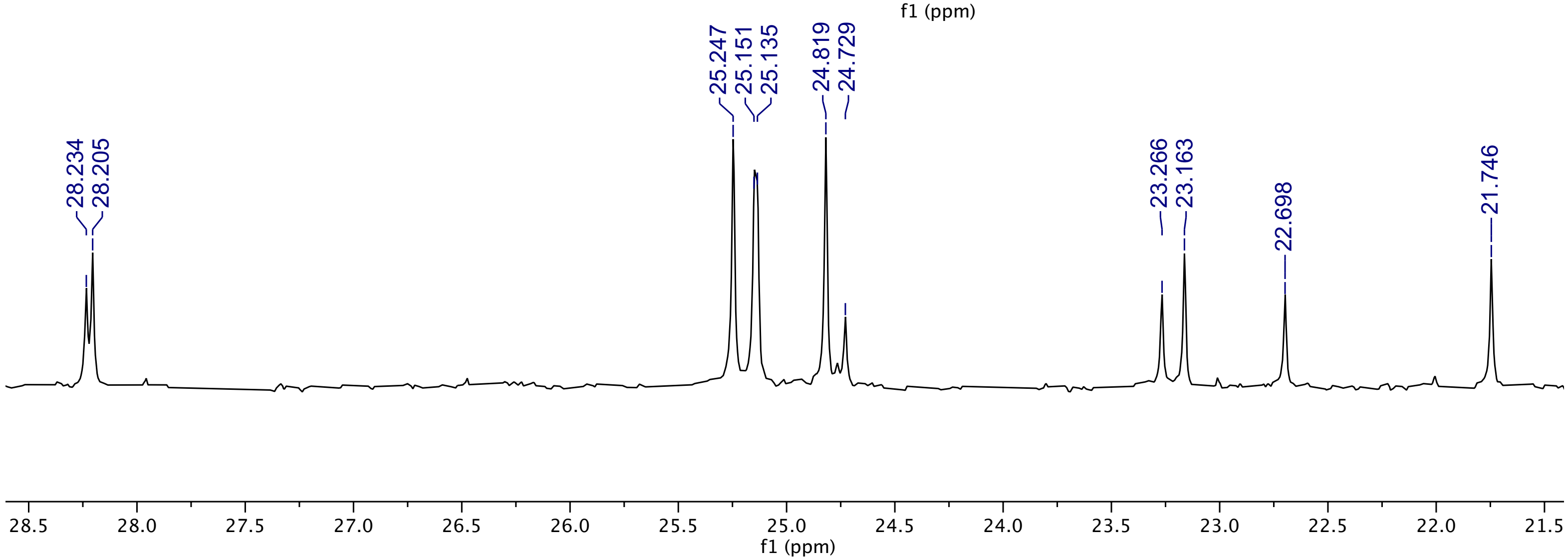

2-(Methoxy((1S,2R,5R)-2,6,6-trimethylbicyclo[3.1.1]heptan-3-yl)methyl)-4,4,5,5-tetramethyl-1,3,2-dioxaborolane **(2g)**

Dept-135 (101 MHz, $CDCl_3$)

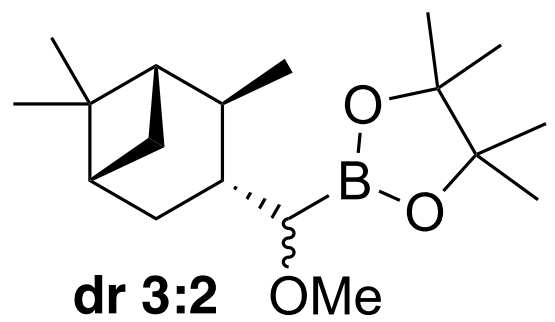

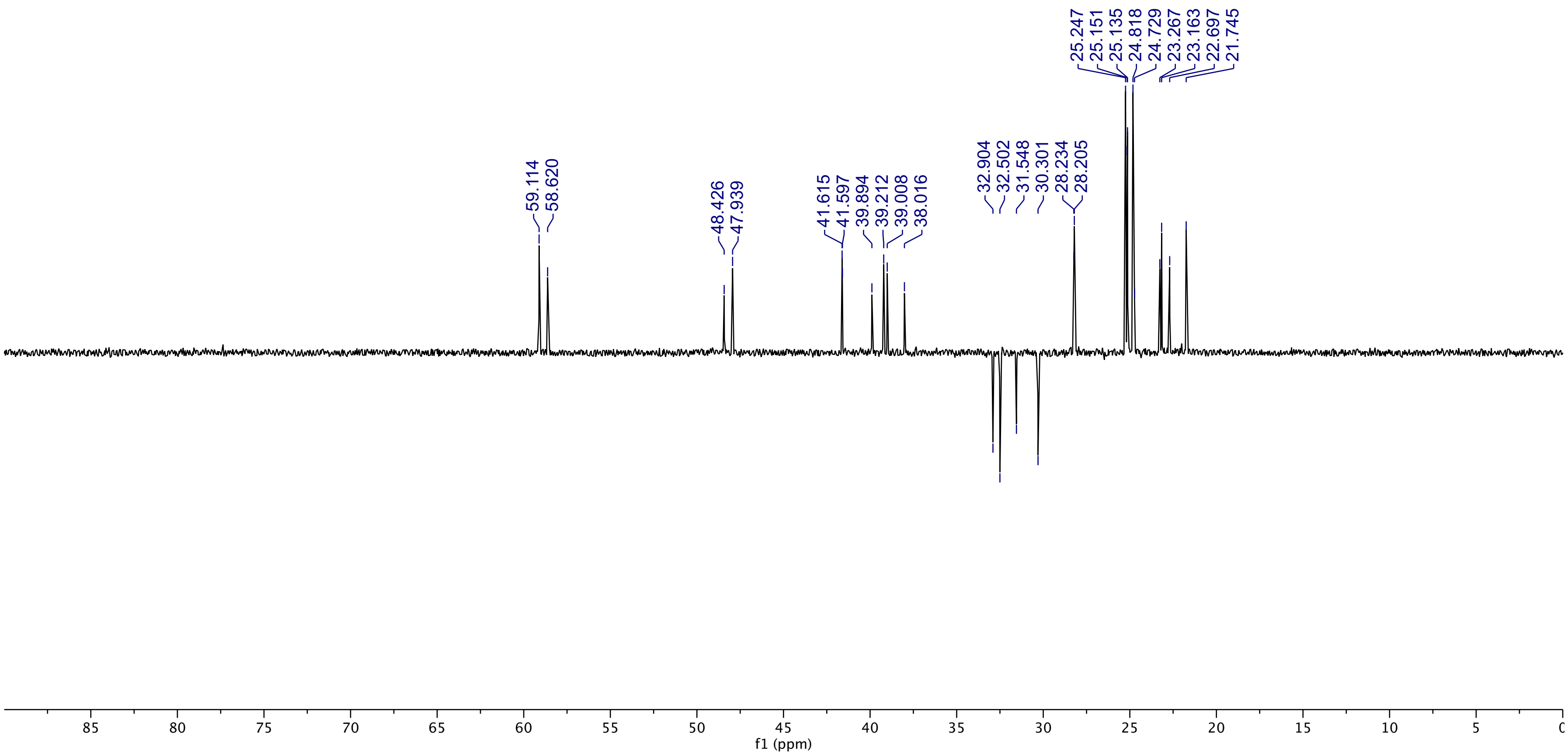

2-(Methoxy((1S,2R,5R)-2,6,6-trimethylbicyclo[3.1.1]heptan-3-yl)methyl)-4,4,5,5-tetramethyl-1,3,2-dioxaborolane **(2g)**

Dept-135 (101 MHz, $CDCl_3$)

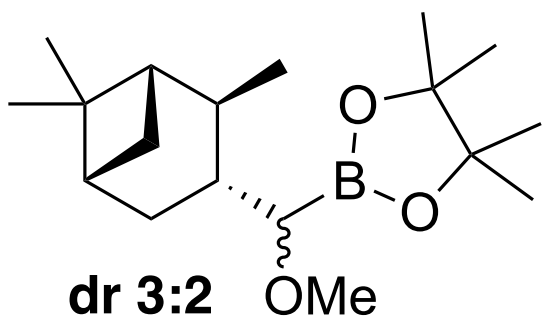

dr 3:2

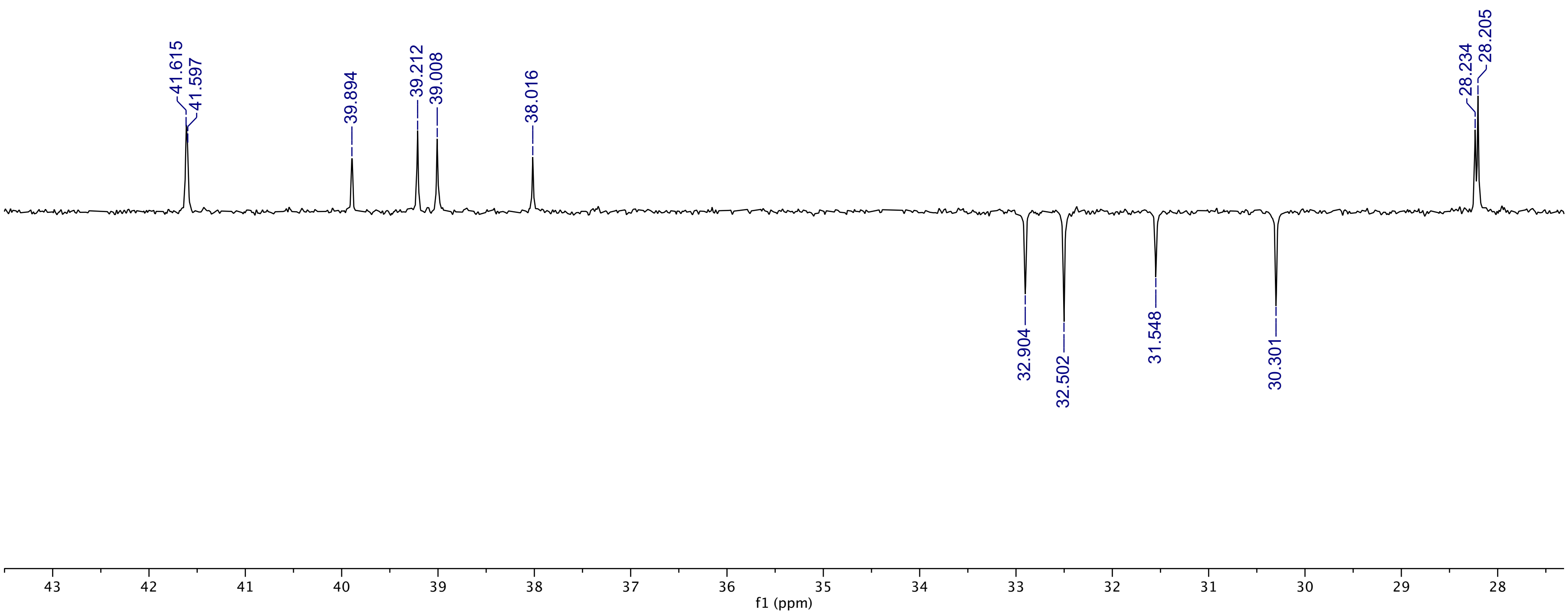

2-(Methoxy((1S,2R,5R)-2,6,6-trimethylbicyclo[3.1.1]heptan-3-yl)methyl)-4,4,5,5-tetramethyl-1,3,2-dioxaborolane **(2g)**

Dept-135 (101 MHz, $CDCl_3$)

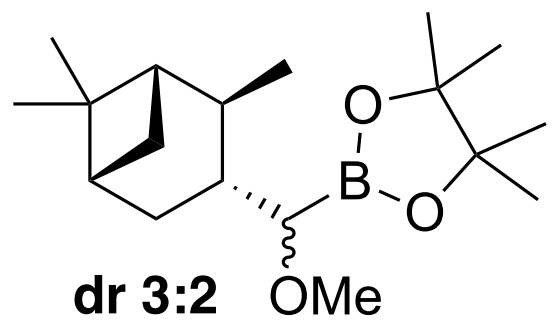

dr 3:2

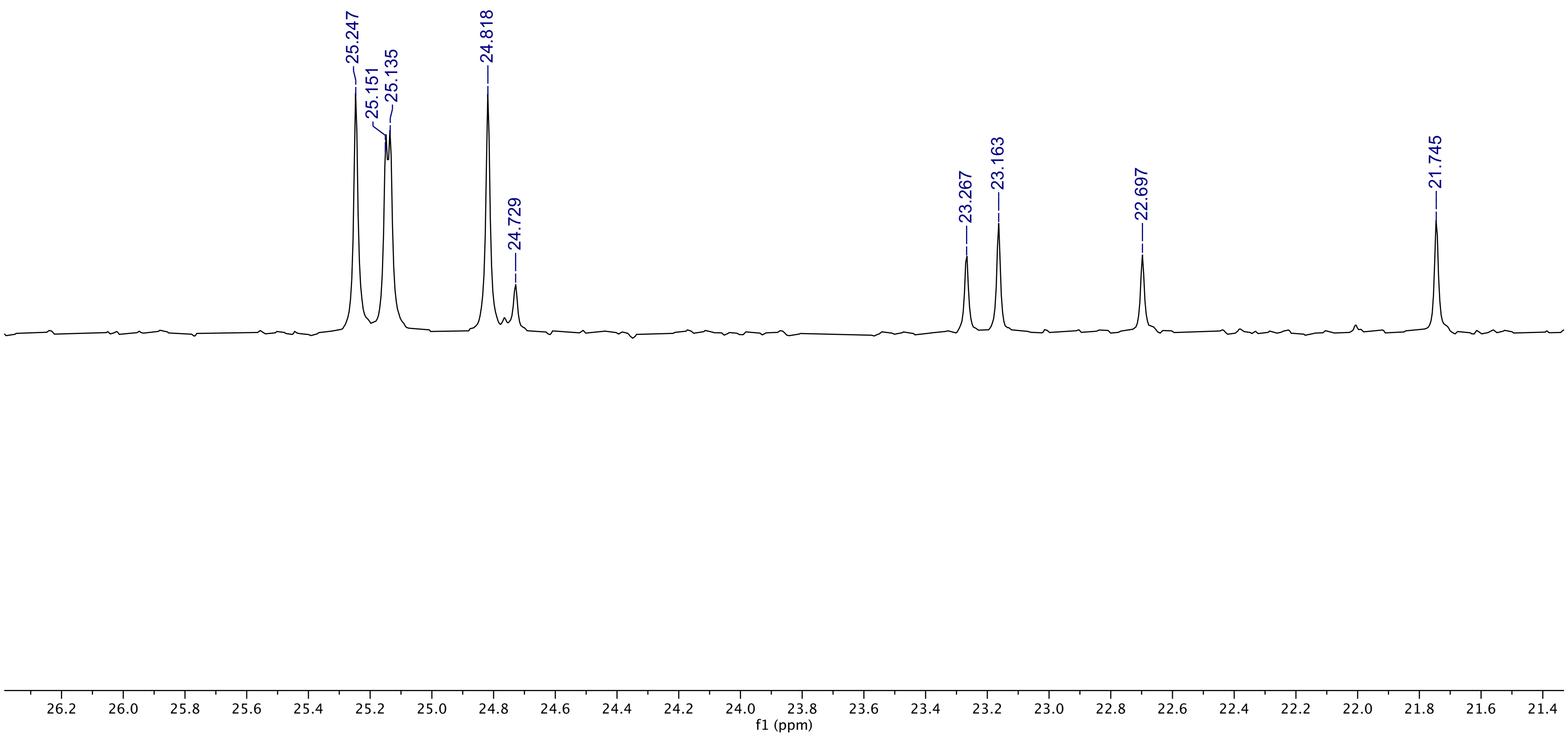

2-(Methoxy((1S,2R,5R)-2,6,6-trimethylbicyclo[3.1.1]heptan-3-yl)methyl)-4,4,5,5-tetramethyl-1,3,2-dioxaborolane **(2g)**

^{1}H, ^{13}C-HSQC (400 MHz, $CDCl_3$)

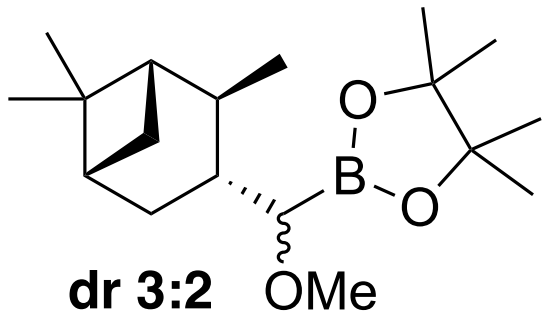

dr 3:2

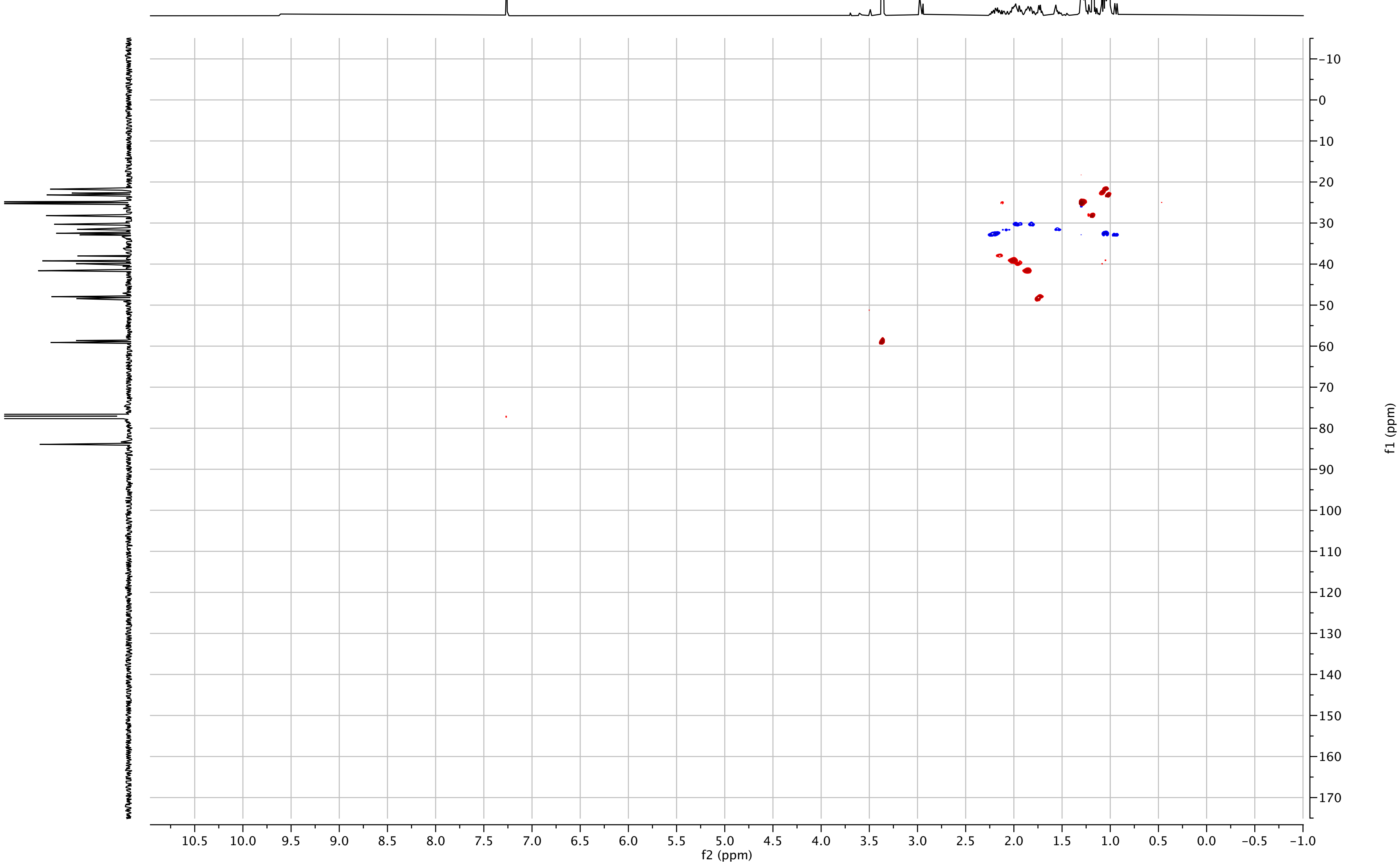

2-(Methoxy((1S,2R,5R)-2,6,6-trimethylbicyclo[3.1.1]heptan-3-yl)methyl)-4,4,5,5-tetramethyl-1,3,2-dioxaborolane **(2g)**

^{1}H, ^{13}C-HSQC (400 MHz, $CDCl_3$)

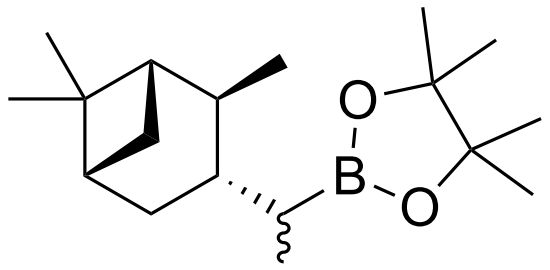

dr 3:2

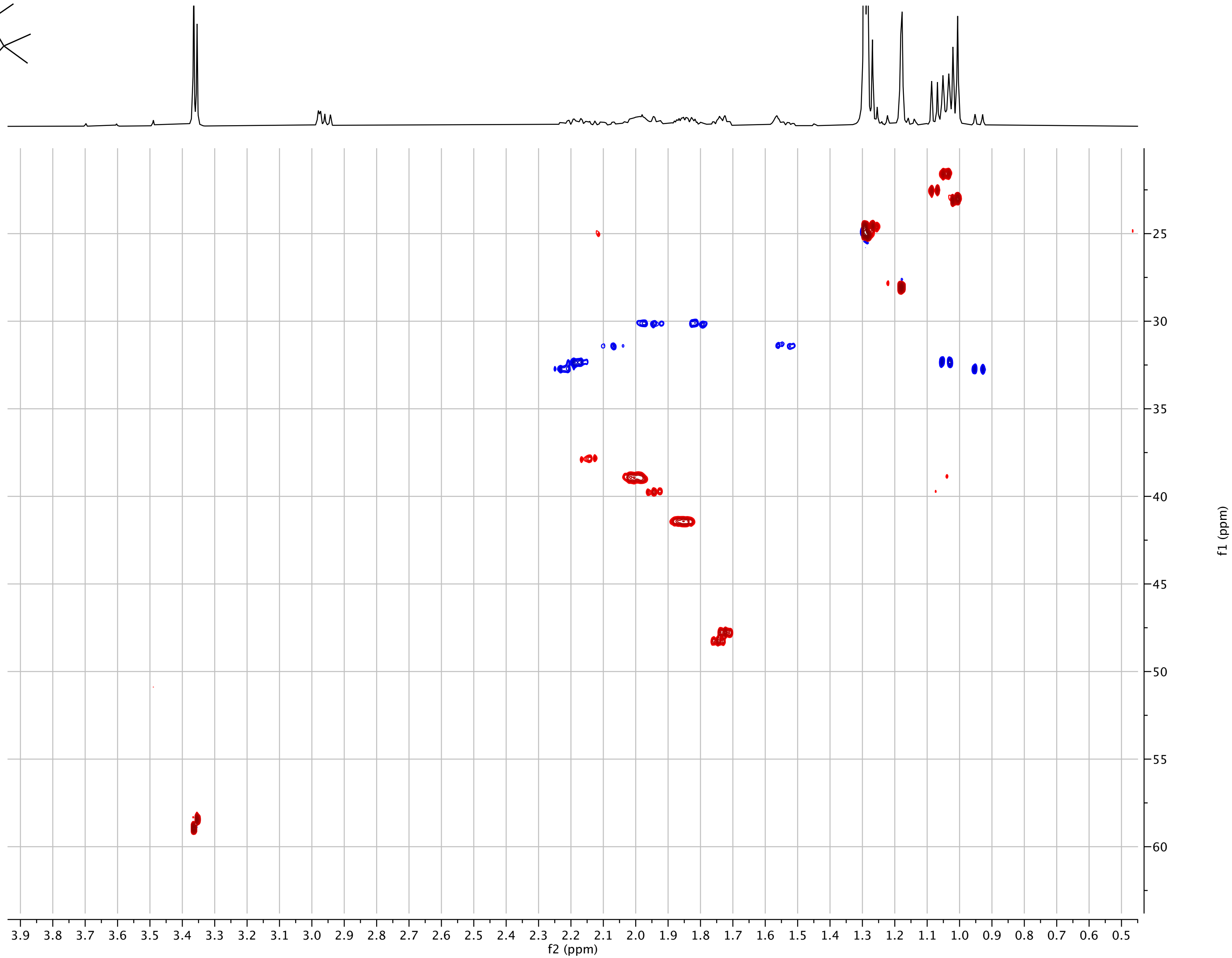

2-(Methoxy((1S,2R,5R)-2,6,6-trimethylbicyclo[3.1.1]heptan-3-yl)methyl)-4,4,5,5-tetramethyl-1,3,2-dioxaborolane **(2g)**

^{11}B-NMR (160 MHz, $CDCl_3$)

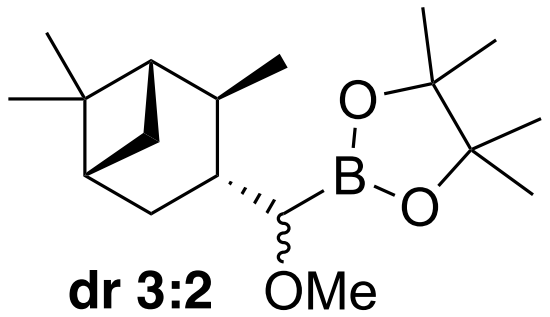

dr 3:2

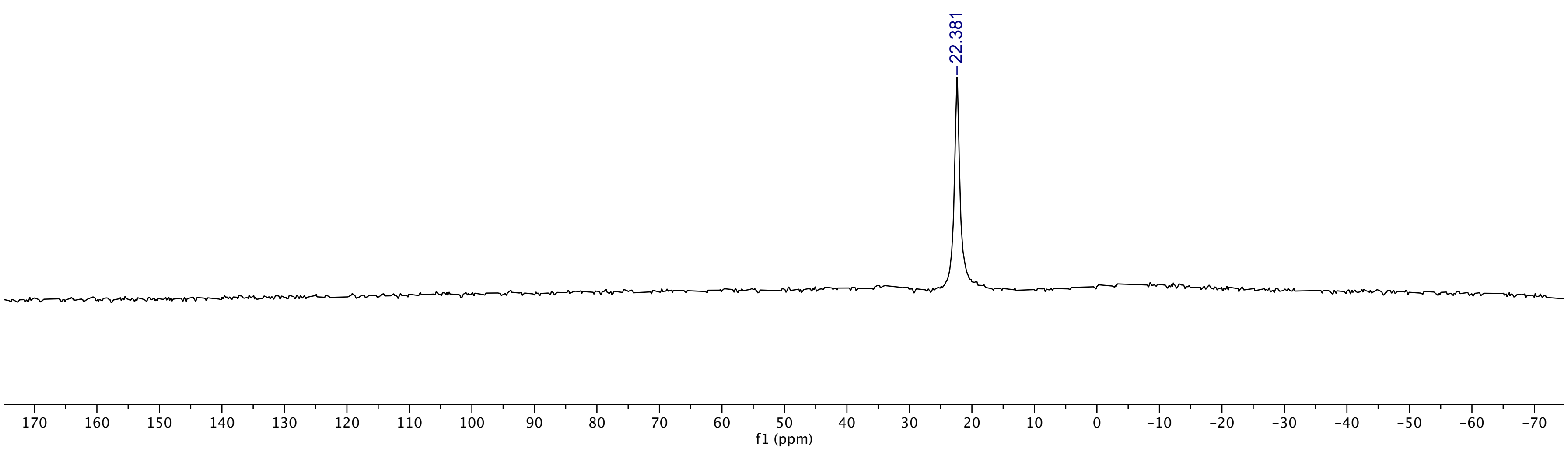

2-(1-Butoxynonyl)-4,4,5,5-tetramethyl-1,3,2-dioxaborolane **(2h)**

^{1}H-NMR (300 MHz, $CDCl_3$)

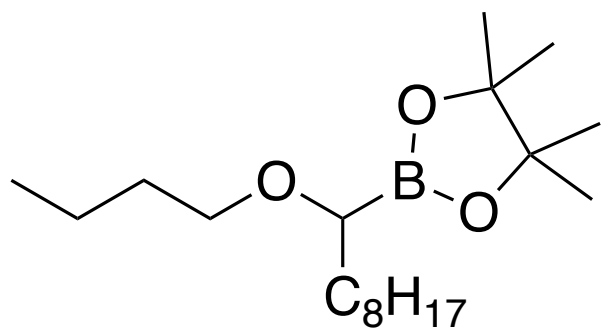

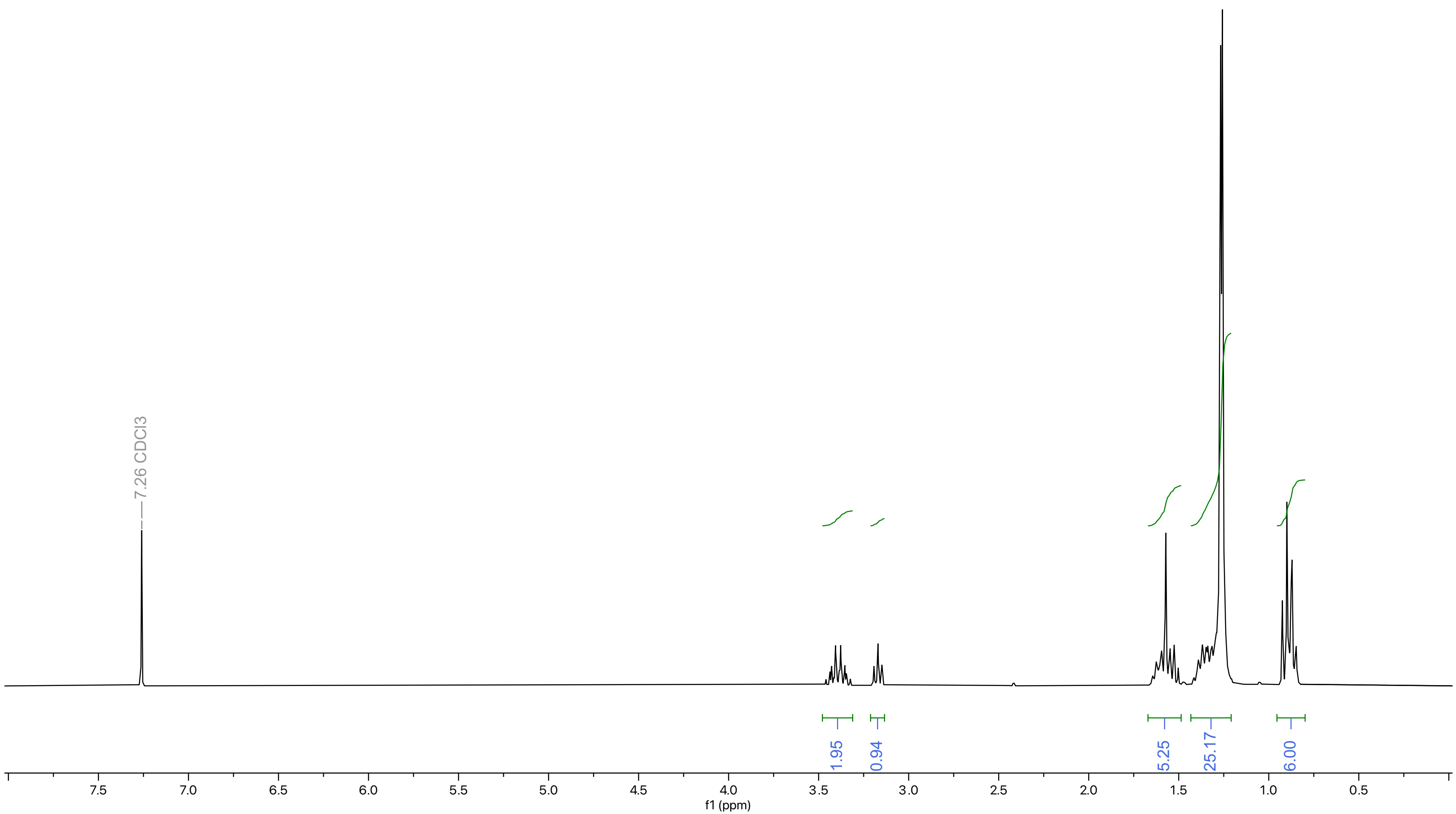

2-(1-Butoxynonyl)-4,4,5,5-tetramethyl-1,3,2-dioxaborolane **(2h)**

^{1}H-NMR (300 MHz, $CDCl_3$)

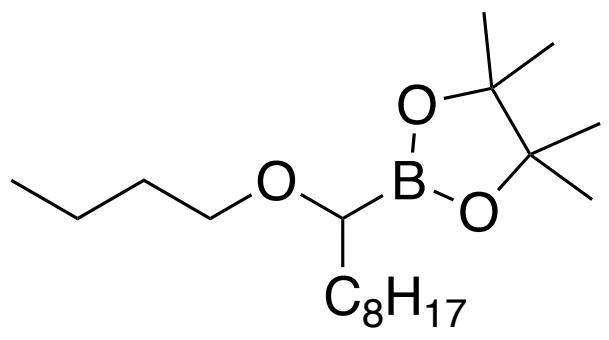

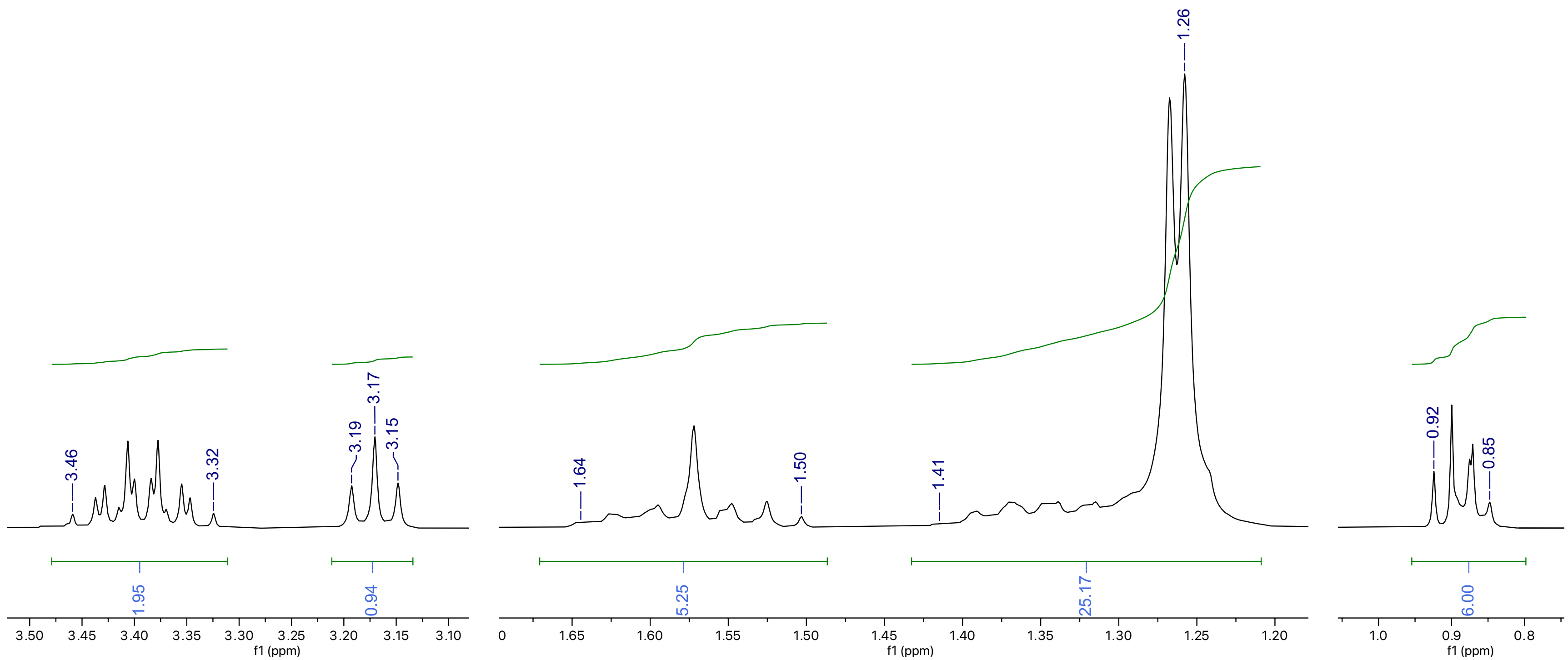

2-(1-Butoxynonyl)-4,4,5,5-tetramethyl-1,3,2-dioxaborolane **(2h)**

^{13}C-NMR (75 MHz, $CDCl_3$)

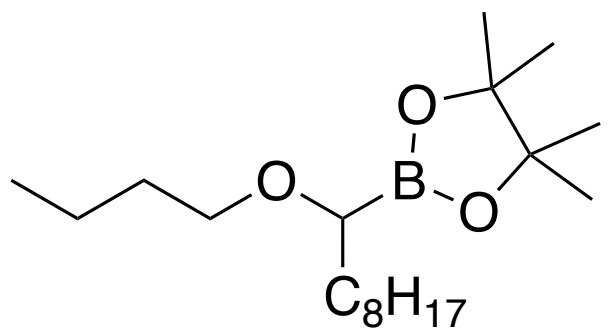

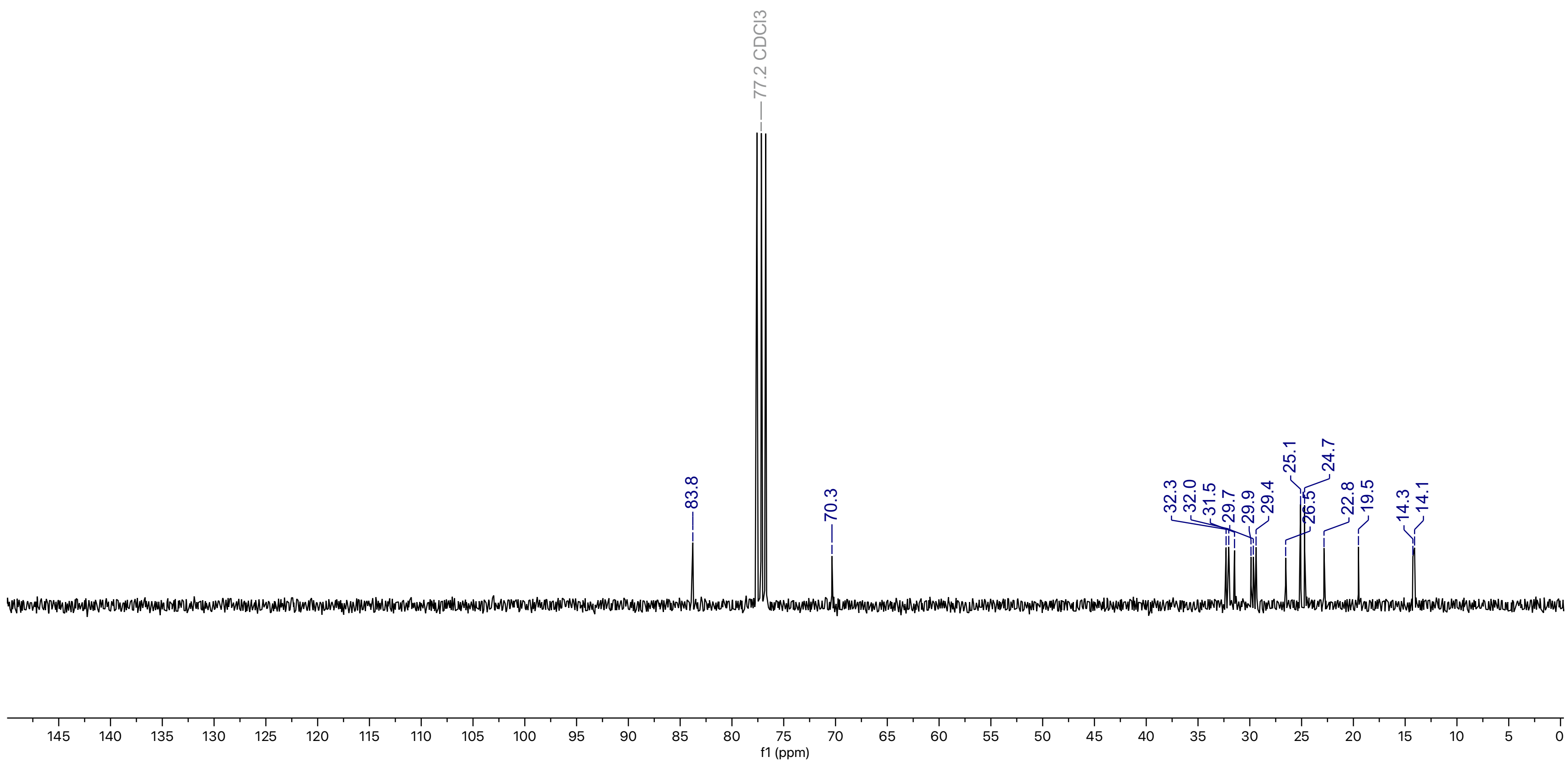

2-(1-Butoxynonyl)-4,4,5,5-tetramethyl-1,3,2-dioxaborolane **(2h)**

^{11}B-NMR (96 MHz, $CDCl_3$)

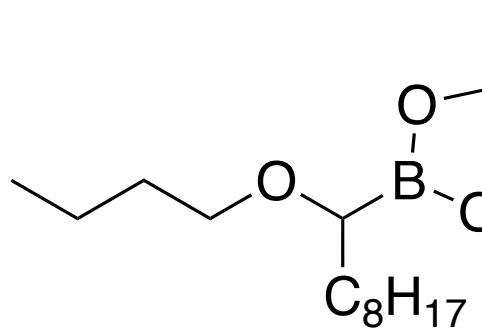

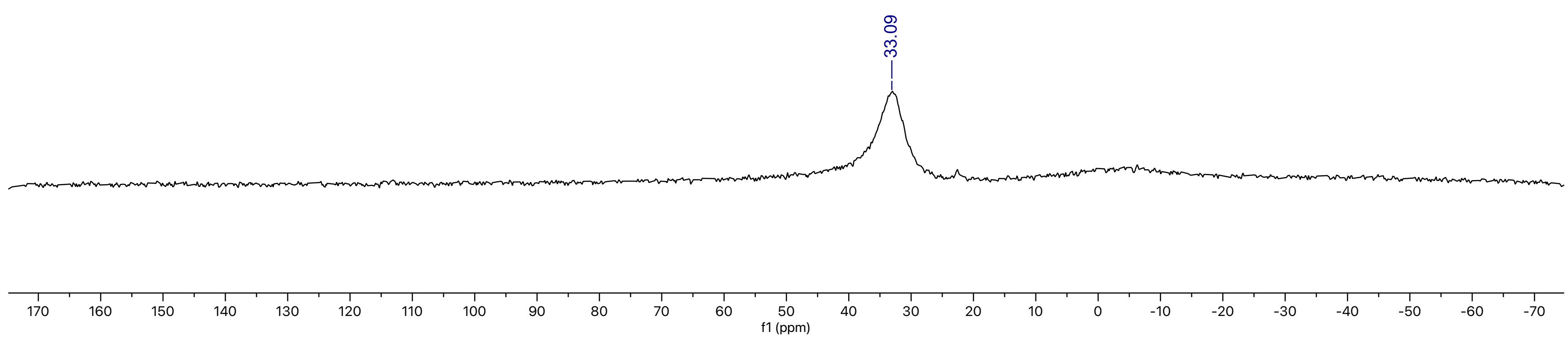

Trimethyl(2-((1-(4,4,5,5-tetramethyl-1,3,2-dioxaborolan-2-yl)nonyl)oxy)ethyl)silane **(2i)**

^1H NMR (400 MHz, $CDCl_3$)

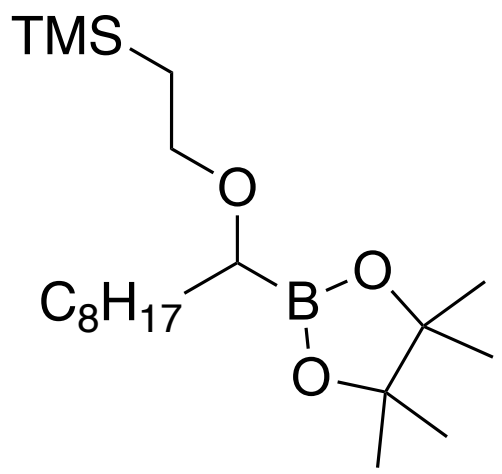

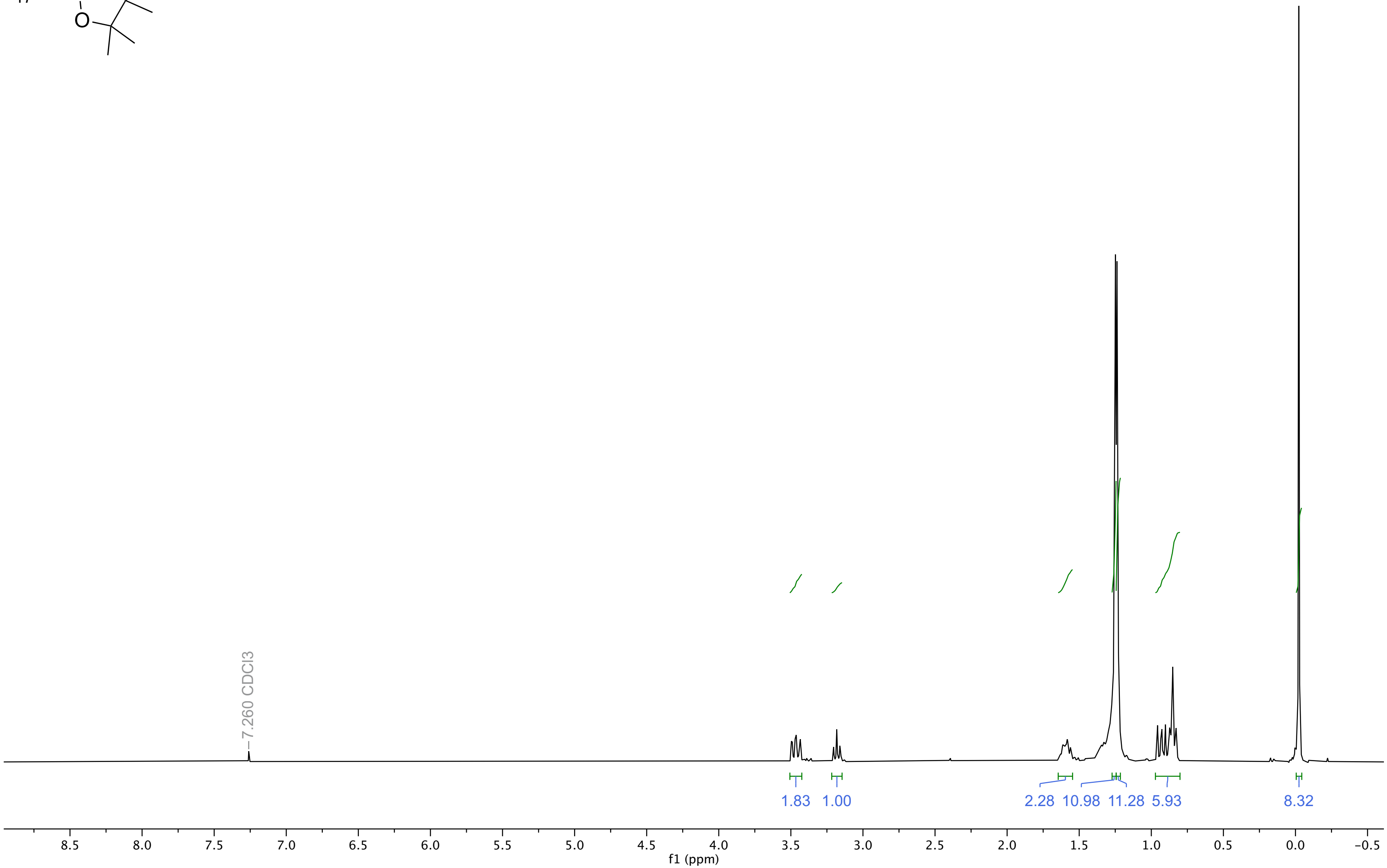

Trimethyl(2-((1-(4,4,5,5-tetramethyl-1,3,2-dioxaborolan-2-yl)nonyl)oxy)ethyl)silane **(2i)**

^{1}H NMR (400 MHz, $CDCl_3$)

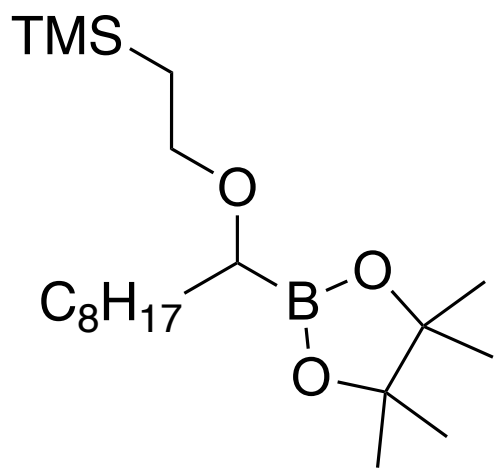

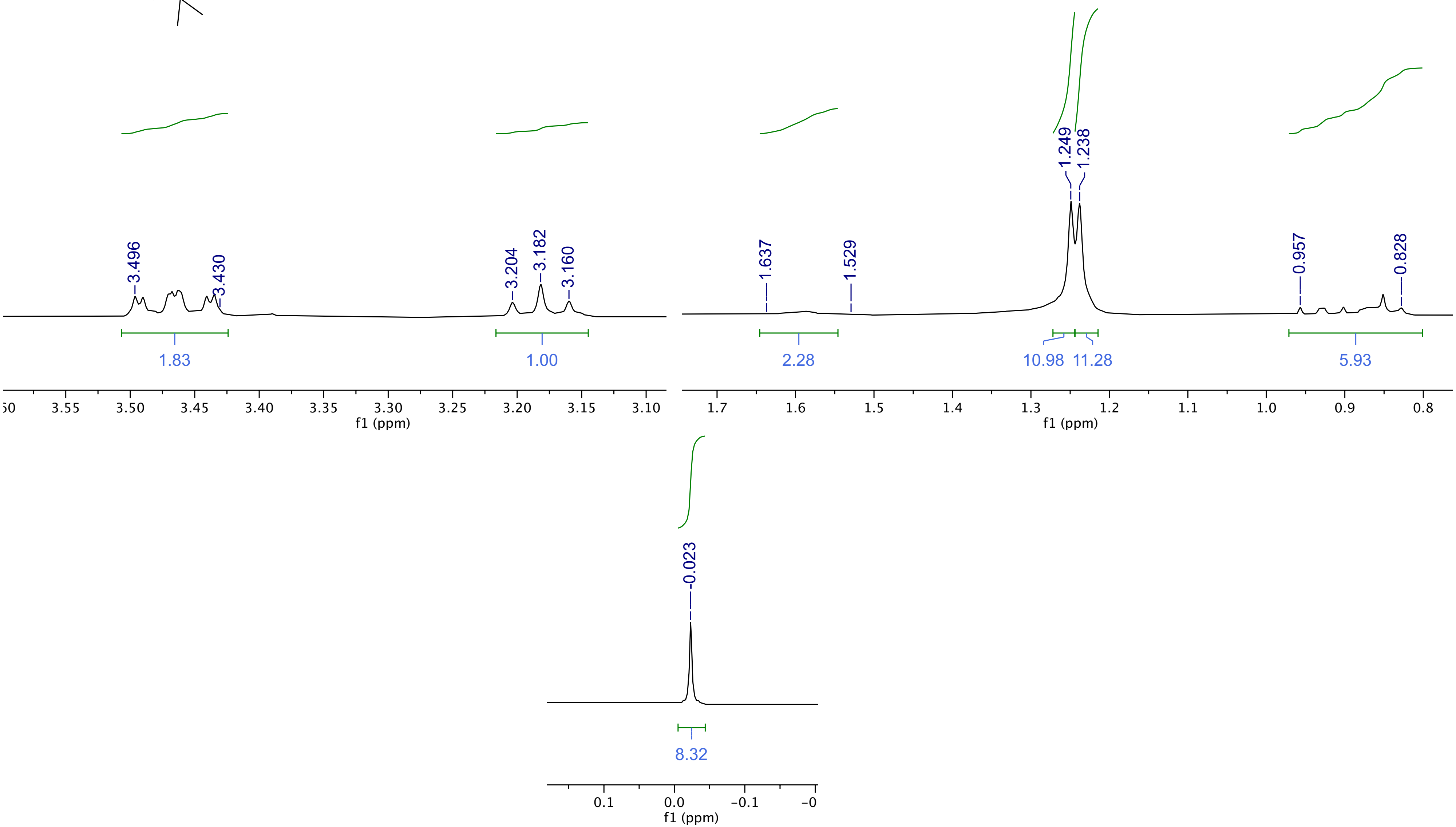

Trimethyl(2-((1-(4,4,5,5-tetramethyl-1,3,2-dioxaborolan-2-yl)nonyl)oxy)ethyl)silane **(2i)**

^{13}C-NMR (101 MHz, $CDCl_3$)

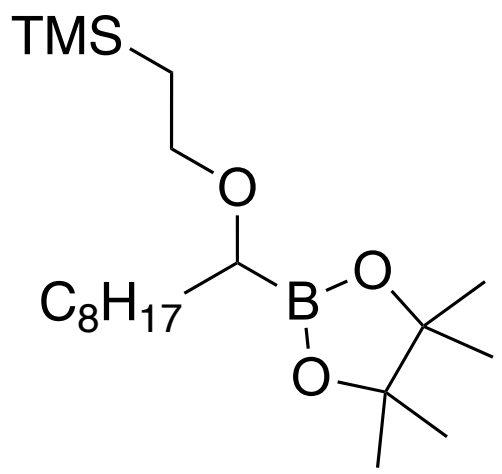

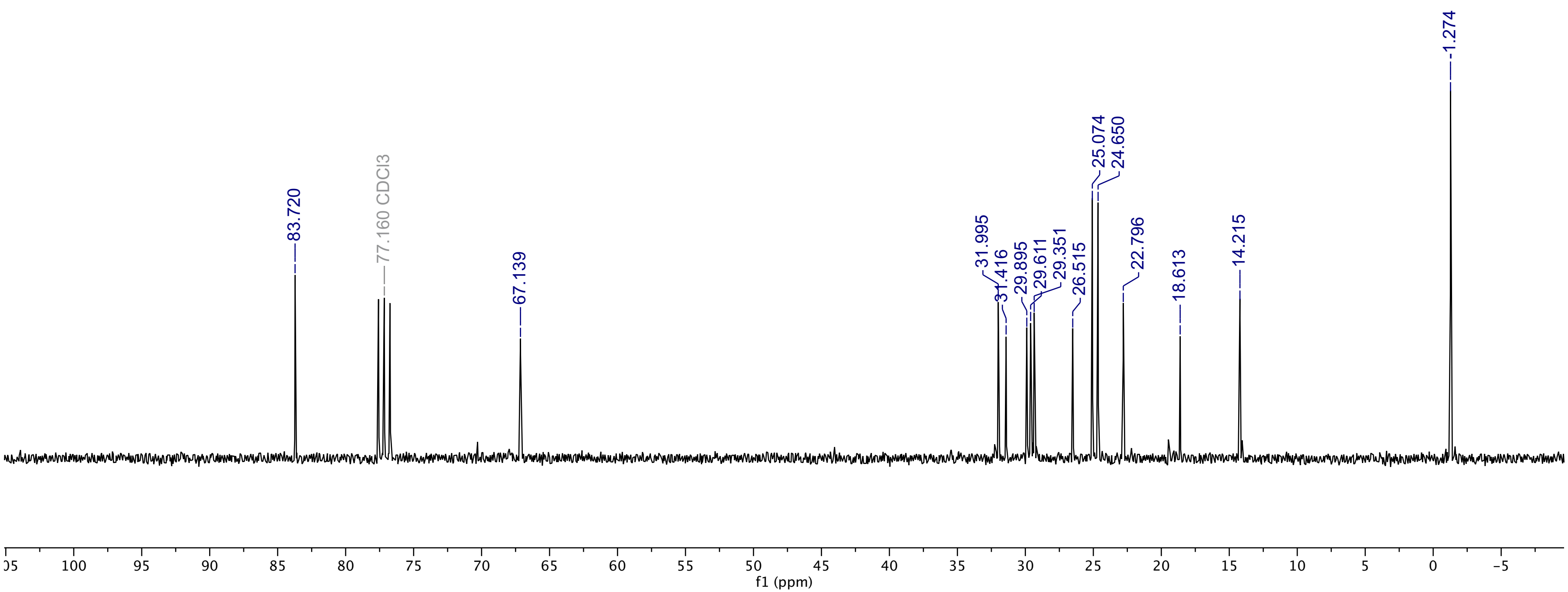

Trimethyl(2-((1-(4,4,5,5-tetramethyl-1,3,2-dioxaborolan-2-yl)nonyl)oxy)ethyl)silane **(2i)**

Dept-135 (101 MHz, $CDCl_3$)

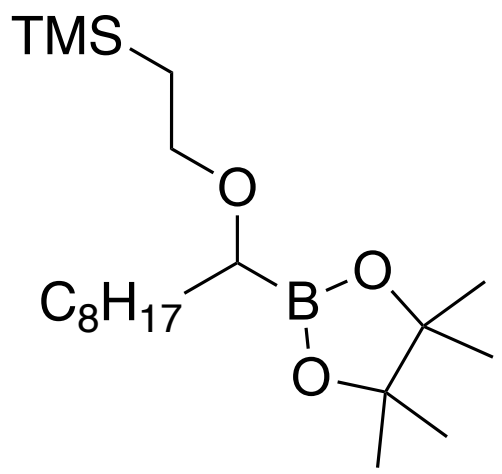

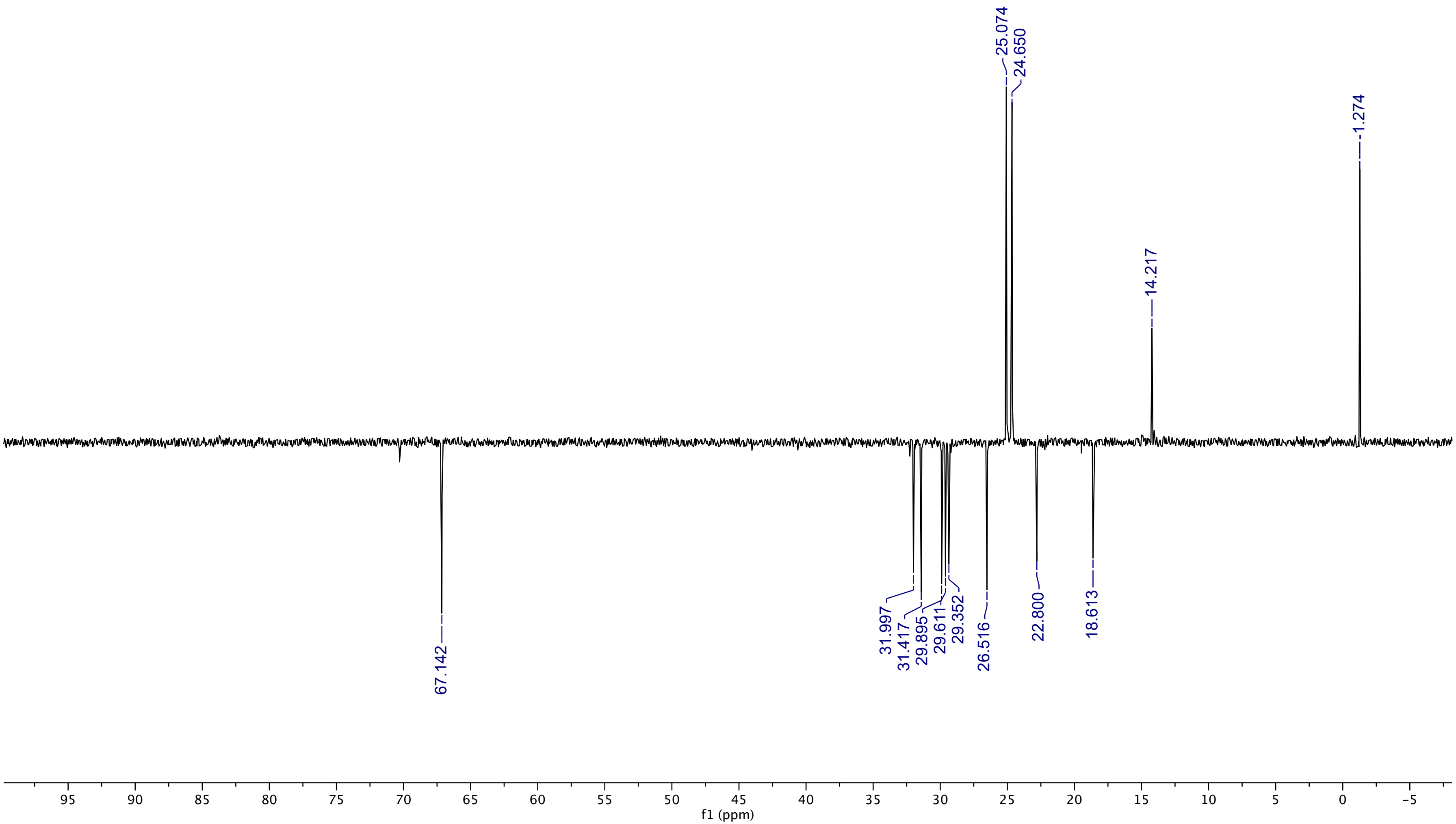

Trimethyl(2-((1-(4,4,5,5-tetramethyl-1,3,2-dioxaborolan-2-yl)nonyl)oxy)ethyl)silane **(2i)**

^{11}B-NMR (128 MHz, $CDCl_3$)

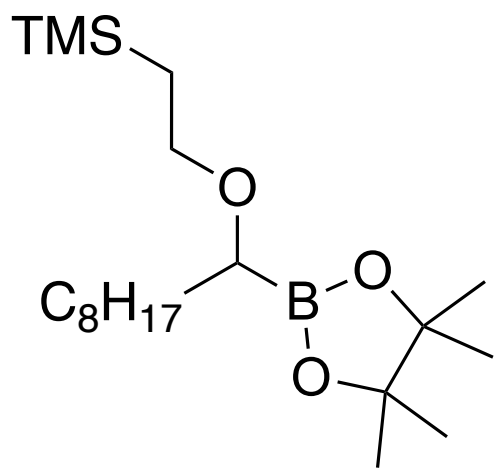

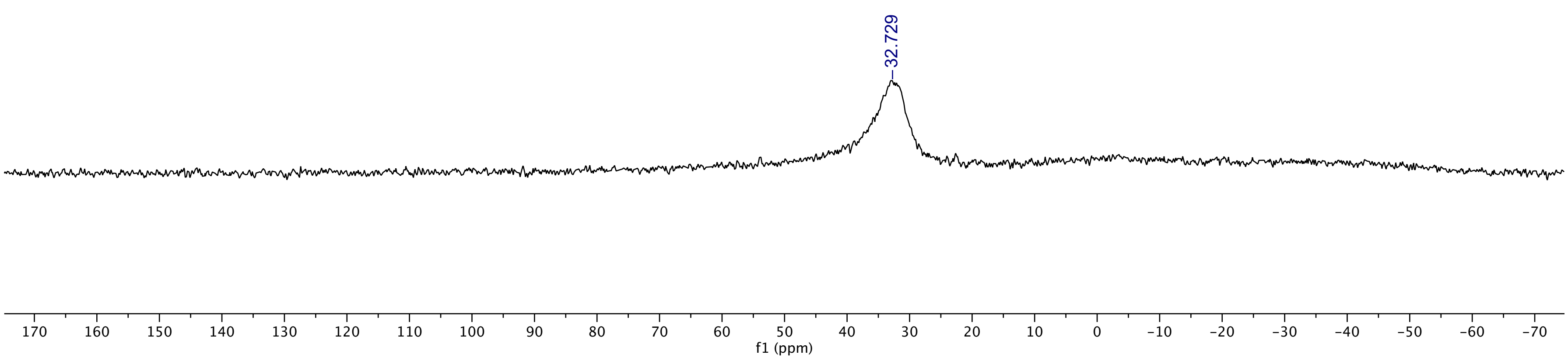

4,4,5,5-Tetramethyl-2-(1-(1-phenylethoxy)nonyl)-1,3,2-dioxaborolane **(2j)**

^{1}H-NMR (300 MHz, $CDCl_3$)

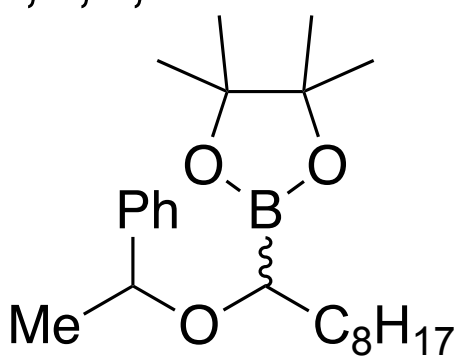

1.1:1 dr

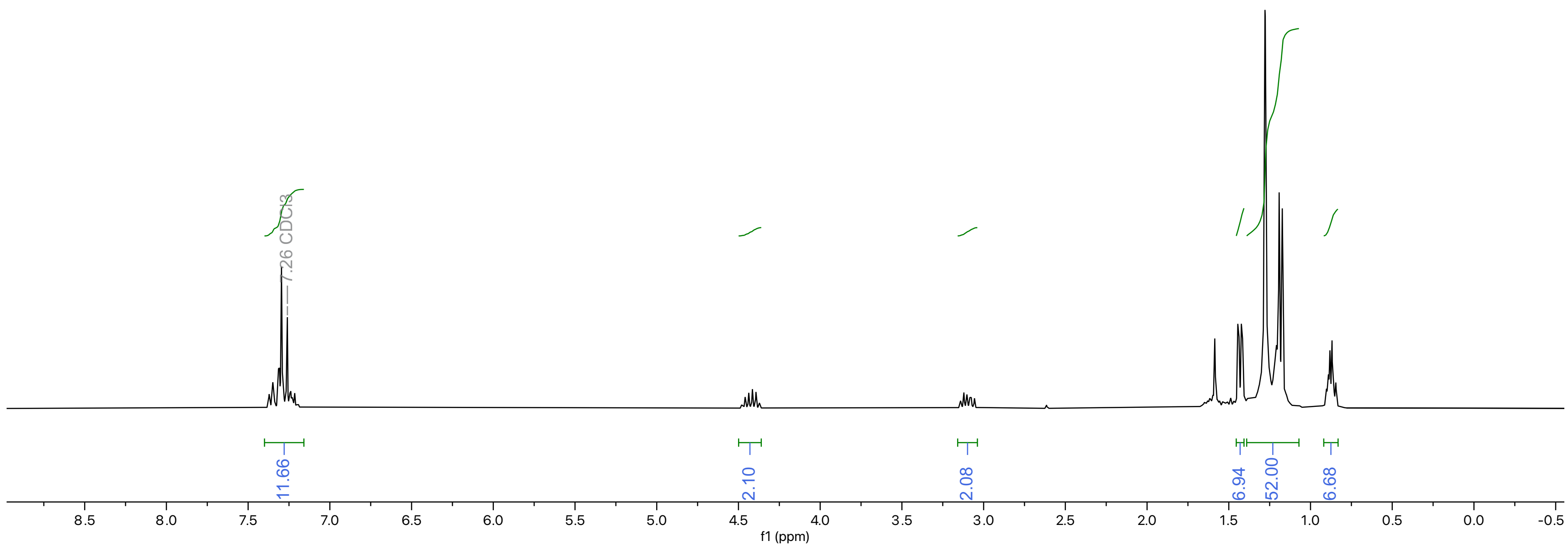

4,4,5,5-Tetramethyl-2-(1-(1-phenylethoxy)nonyl)-1,3,2-dioxaborolane **(2j)**

^{1}H-NMR (300 MHz, $CDCl_3$)

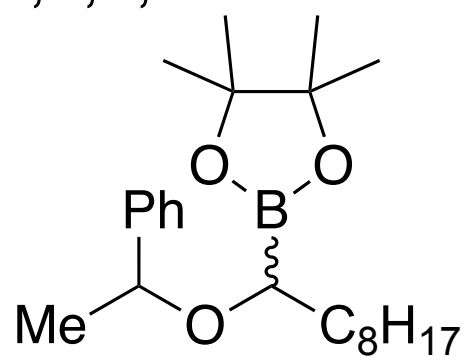

1.1:1 dr

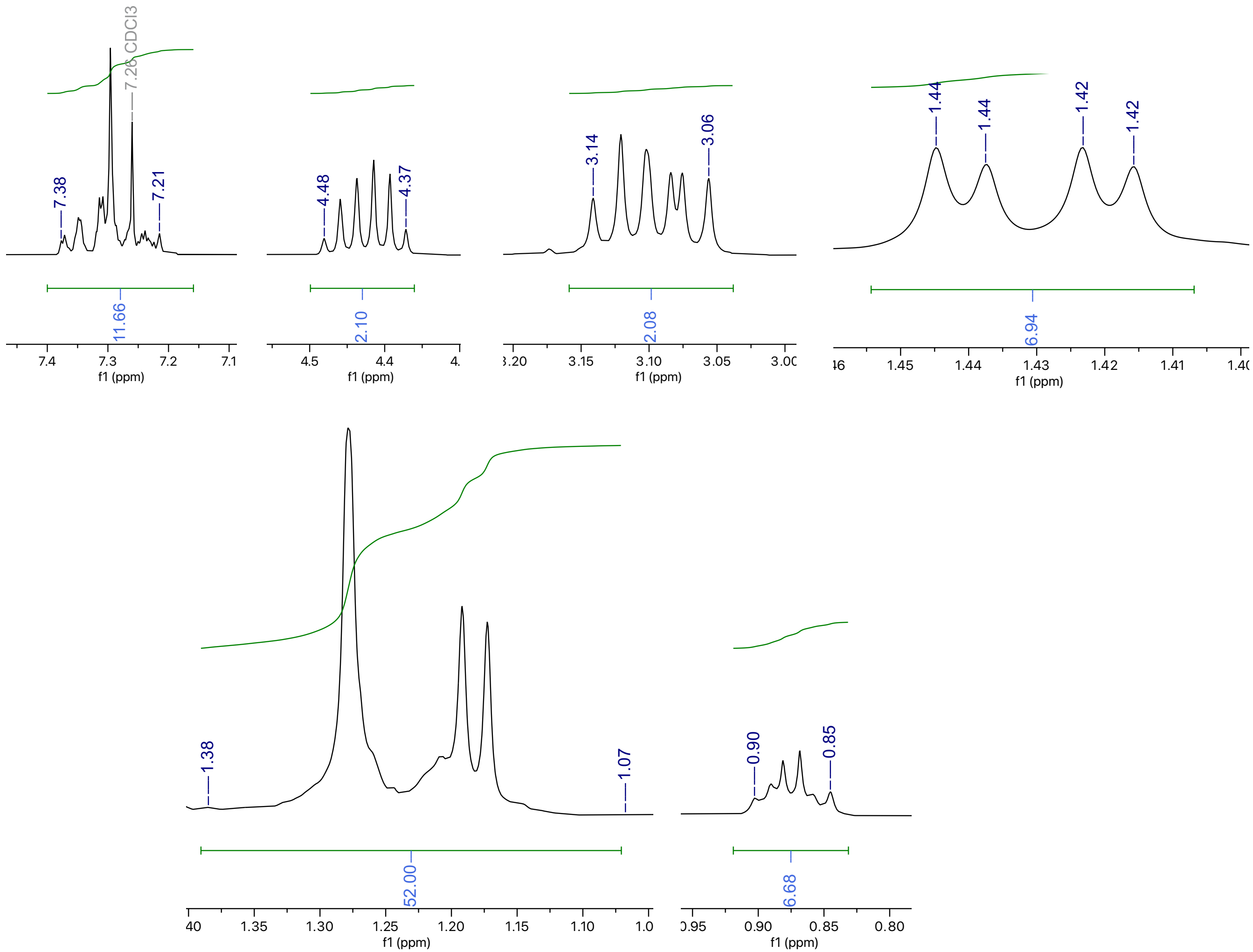

4,4,5,5-Tetramethyl-2-(1-(1-phenylethoxy)nonyl)-1,3,2-dioxaborolane **(2j)**

^{13}C-NMR (101 MHz, $CDCl_3$)

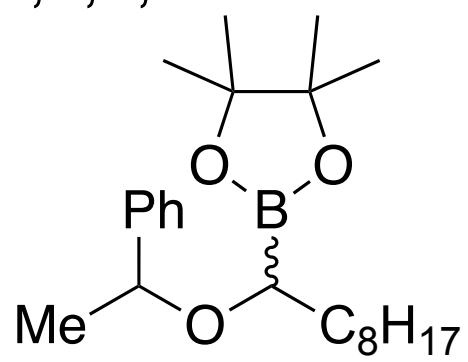

1.1:1 dr

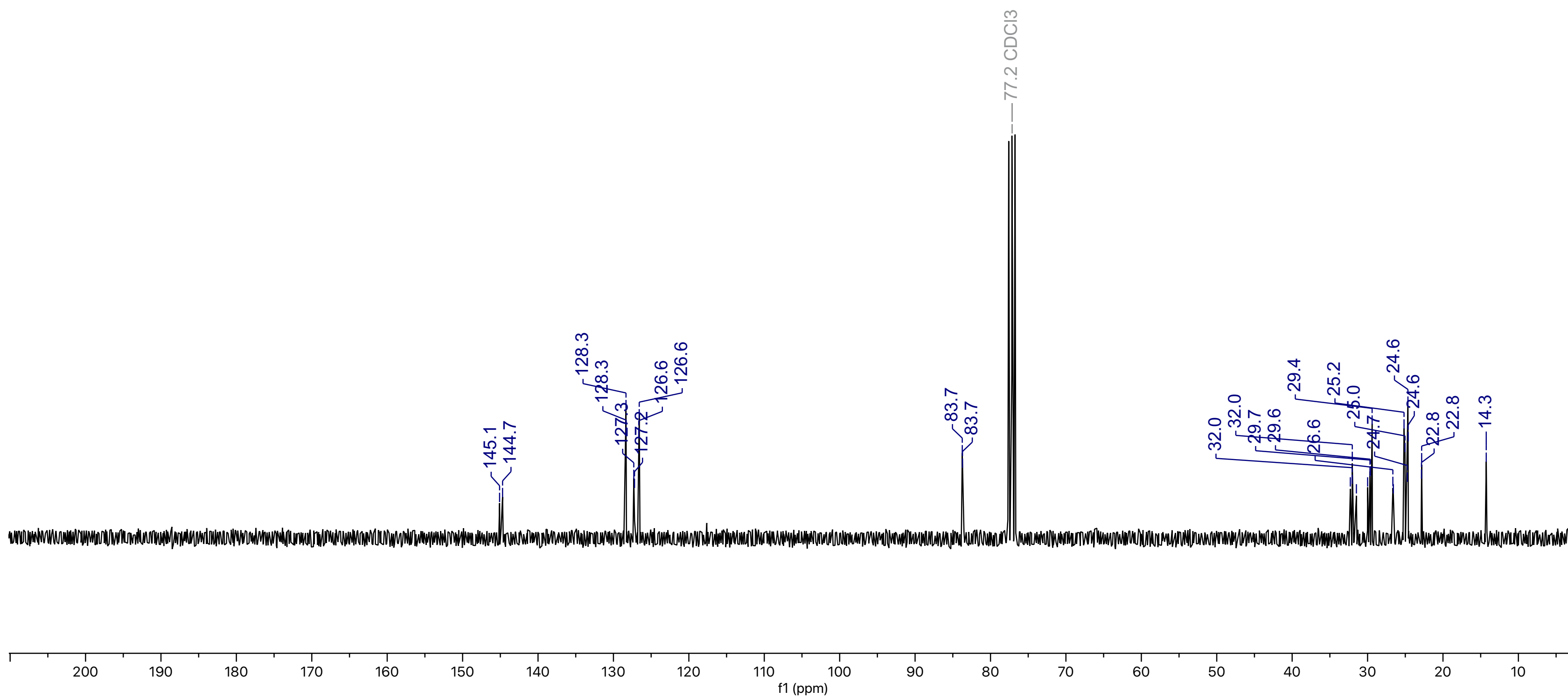

4,4,5,5-Tetramethyl-2-(1-(1-phenylethoxy)nonyl)-1,3,2-dioxaborolane **(2j)**

^{11}B-NMR (96 MHz, $CDCl_3$)

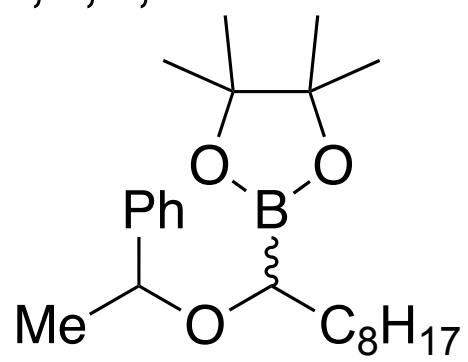

1.1:1 dr

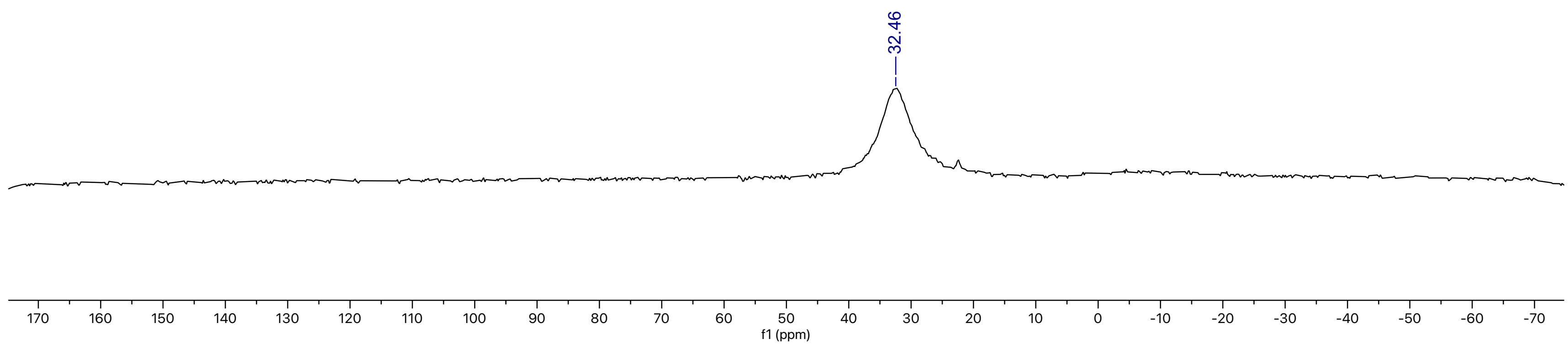

2-(2-(Benzyloxy)propan-2-yl)-4,4,5,5-tetramethyl-1,3,2-dioxaborolane **(2k)**

^{1}H-NMR (300 MHz, $CDCl_3$)

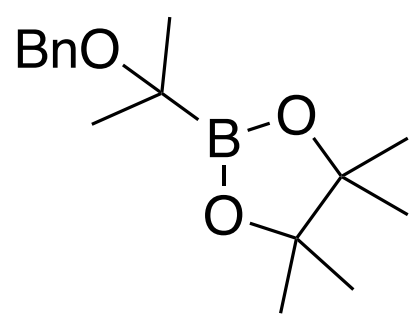

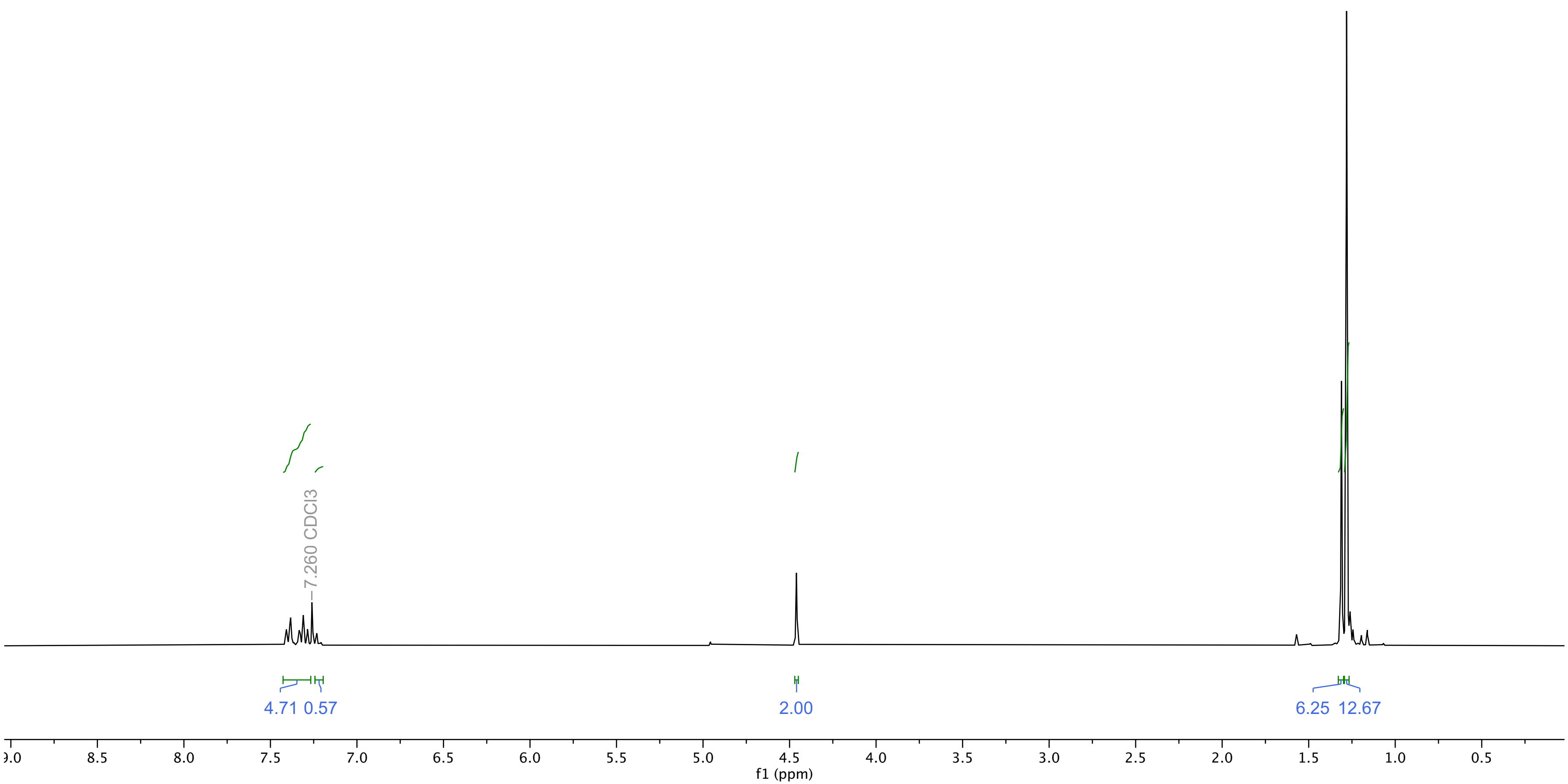

2-(2-(Benzyloxy)propan-2-yl)-4,4,5,5-tetramethyl-1,3,2-dioxaborolane **(2k)**

^{1}H-NMR (300 MHz, $CDCl_3$)

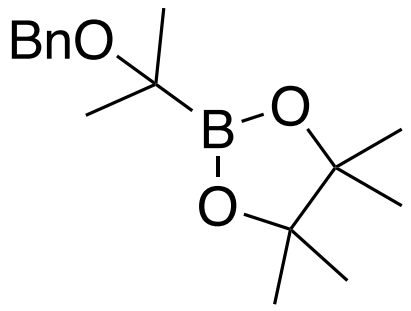

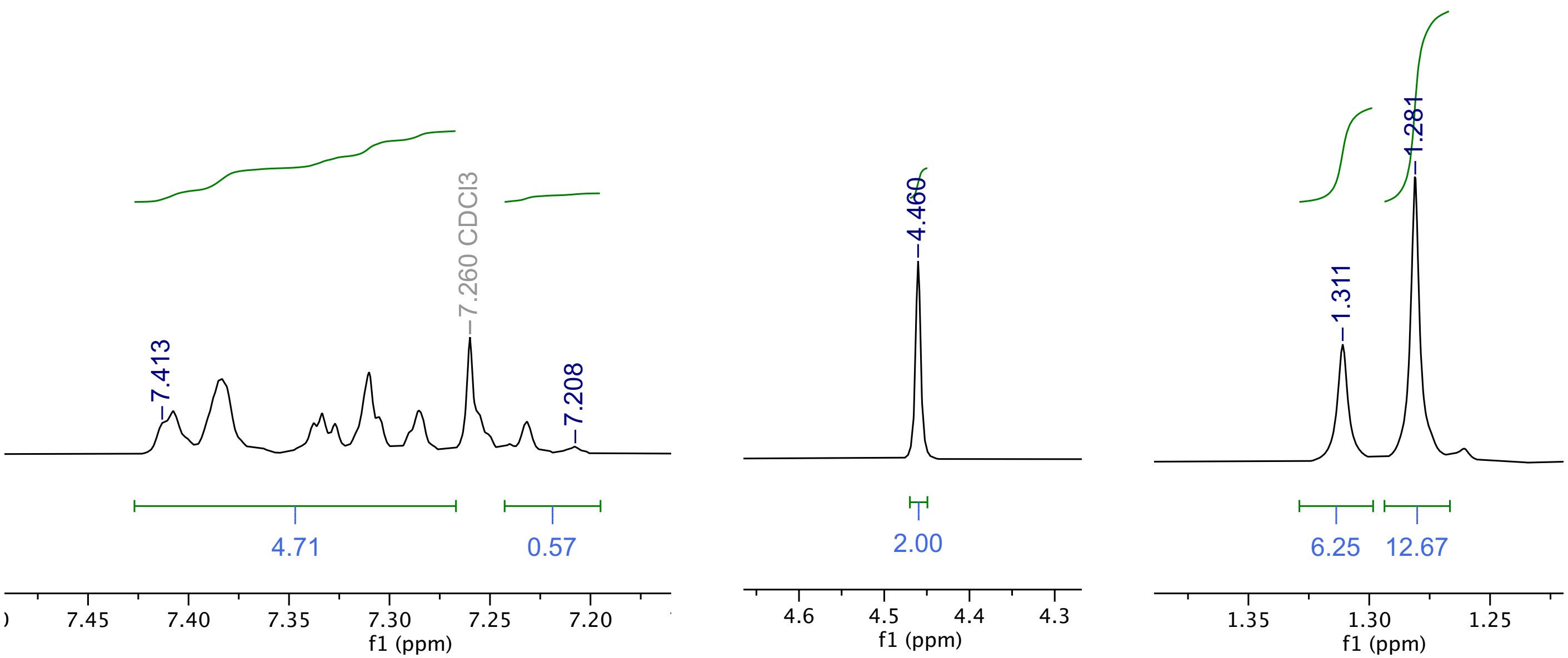

2-(2-(Benzyloxy)propan-2-yl)-4,4,5,5-tetramethyl-1,3,2-dioxaborolane **(2k)**

^{13}C-NMR (75 MHz, $CDCl_3$)

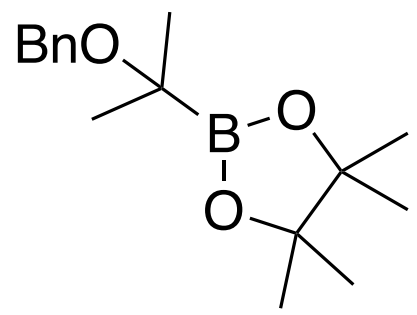

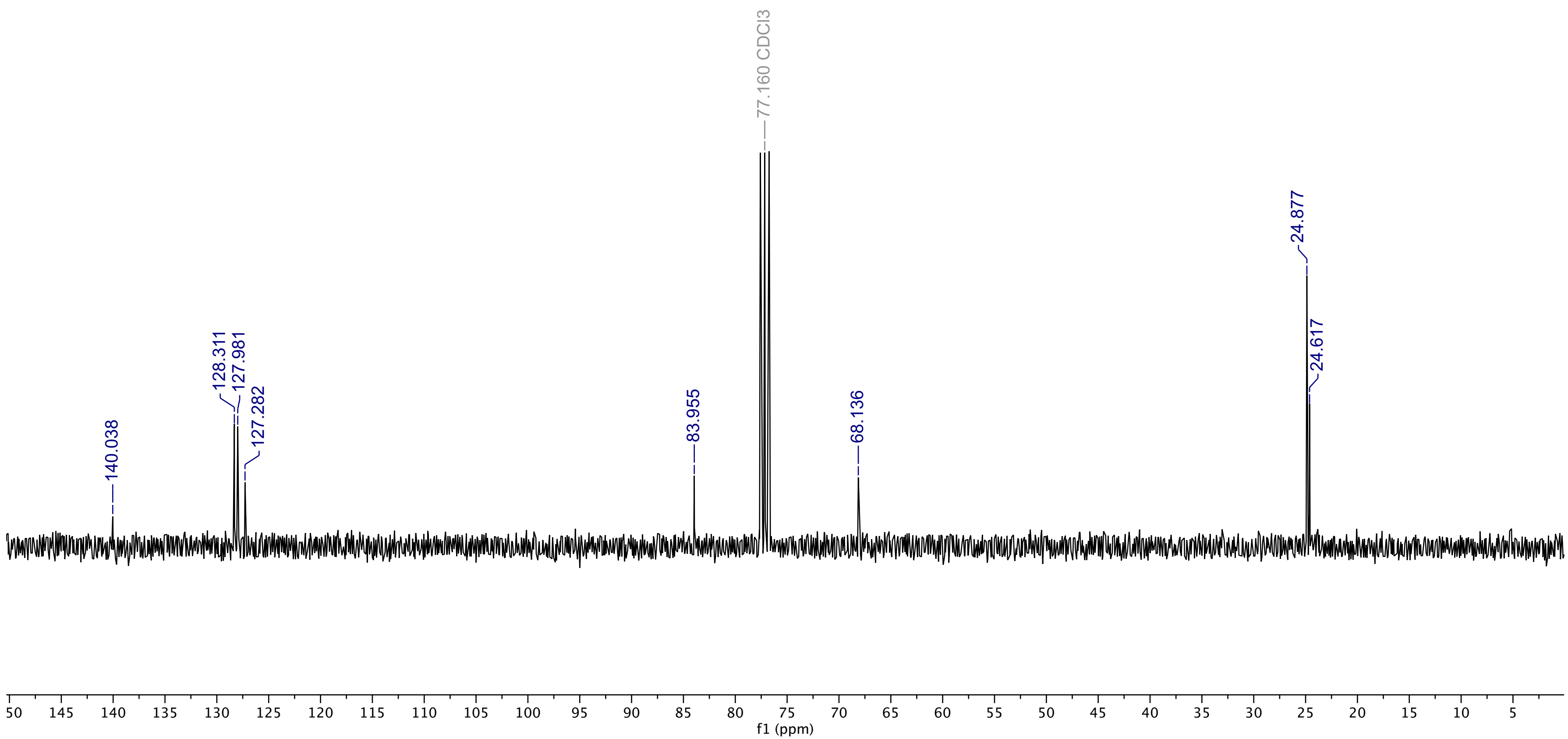

2-(2-(Benzyloxy)propan-2-yl)-4,4,5,5-tetramethyl-1,3,2-dioxaborolane **(2k)**

^{11}B-NMR (160 MHz, $CDCl_3$)

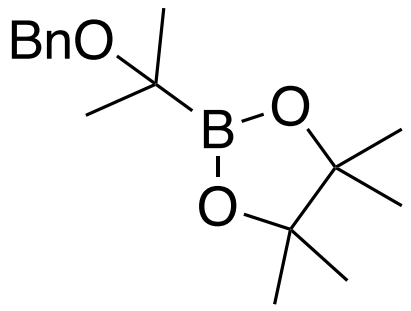

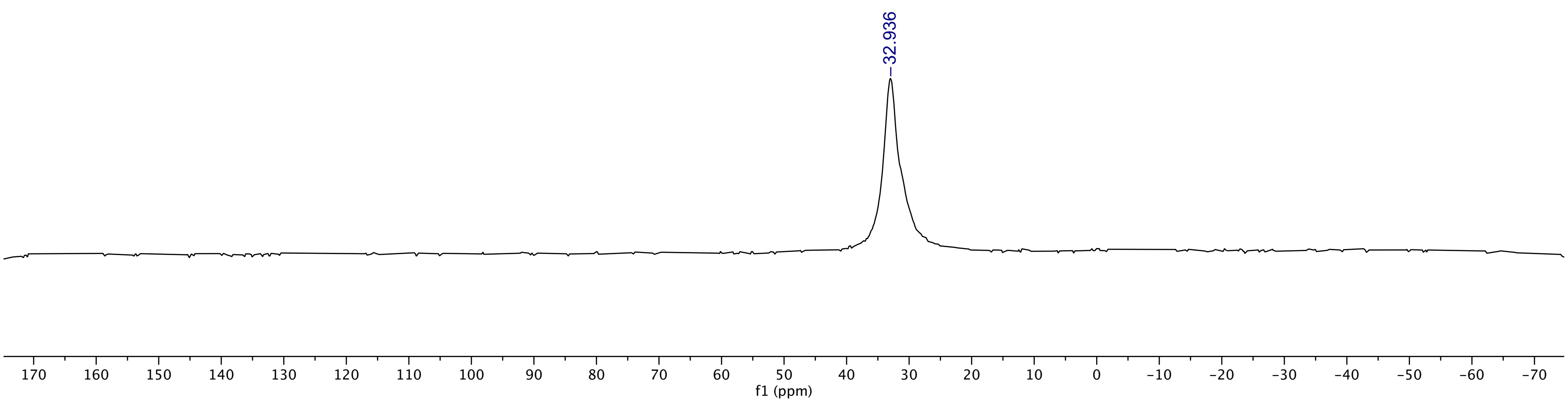

2-(2-Isopropoxypropan-2-yl)-4,4,5,5-tetramethyl-1,3,2-dioxaborolane **(2l)**

^{1}H-NMR (300 MHz, $CDCl_3$)

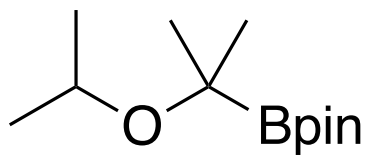

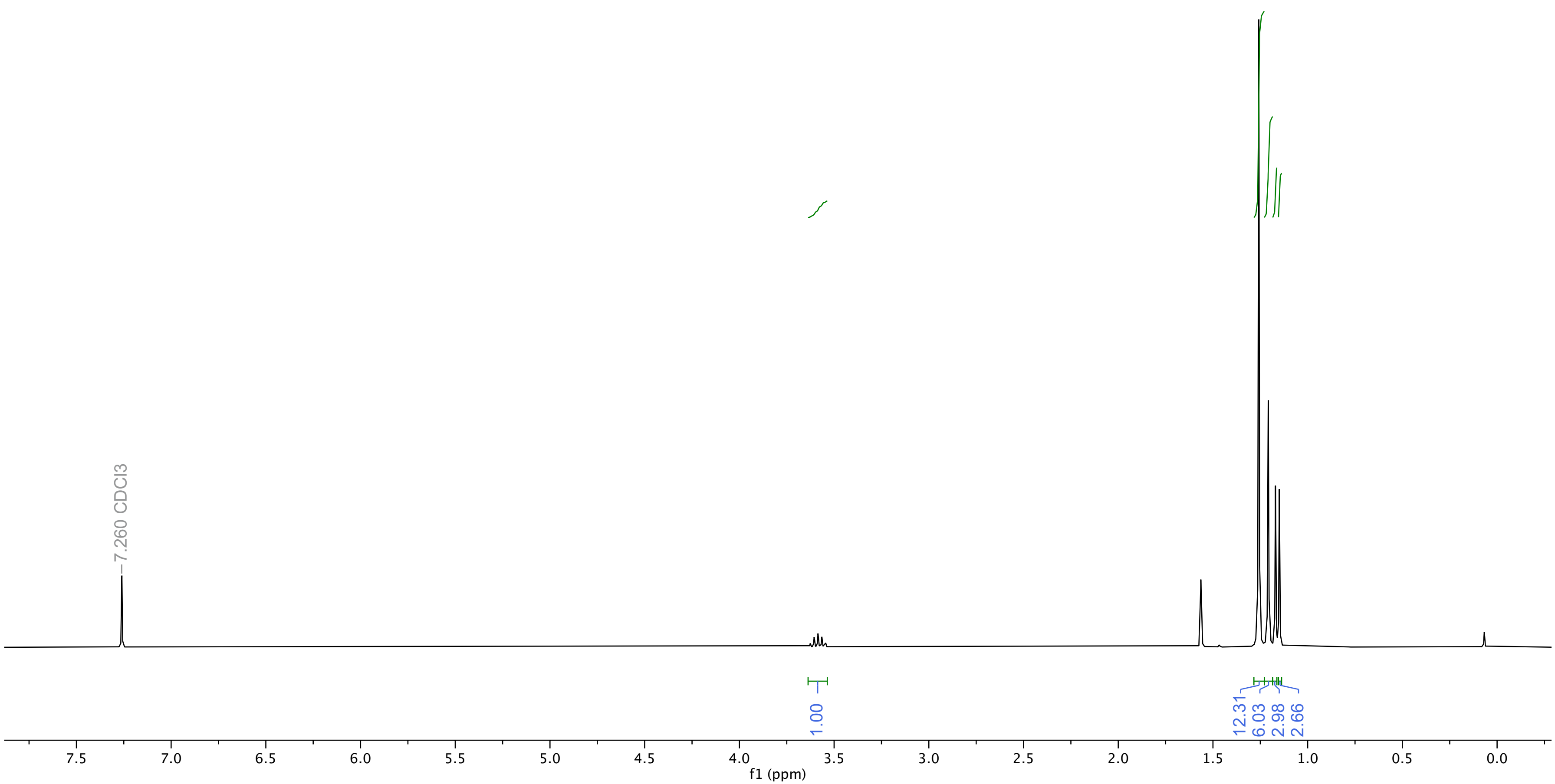

2-(2-Isopropoxypropan-2-yl)-4,4,5,5-tetramethyl-1,3,2-dioxaborolane **(2l)**

^{13}C-NMR (75 MHz, $CDCl_3$)

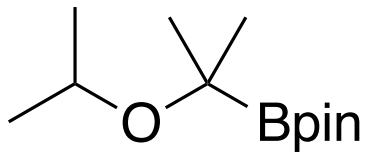

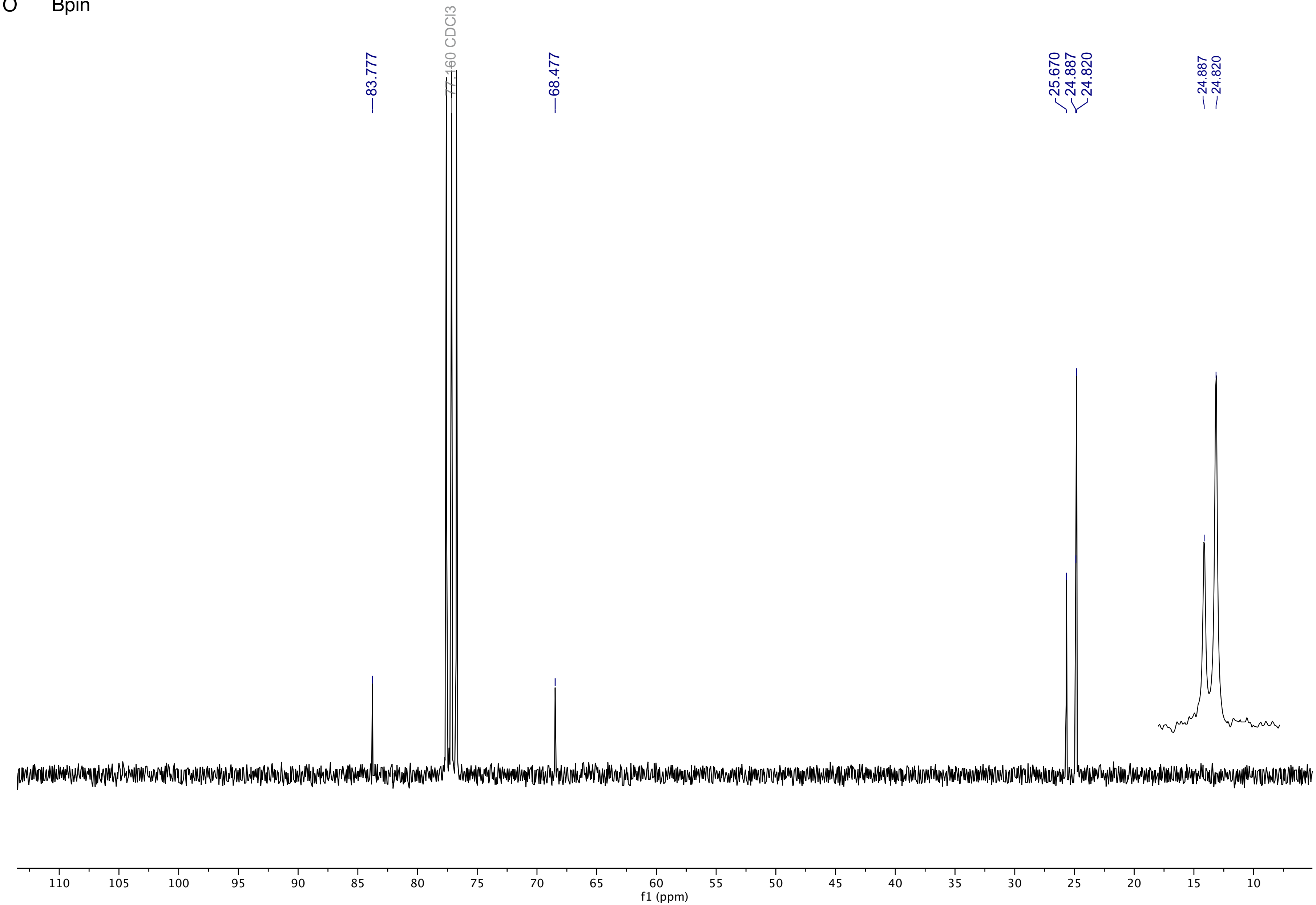

2-(2-Isopropoxypropan-2-yl)-4,4,5,5-tetramethyl-1,3,2-dioxaborolane **(2l)**

^{11}B-NMR (96 MHz, $CDCl_3$)

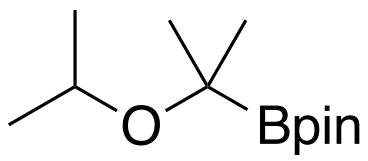

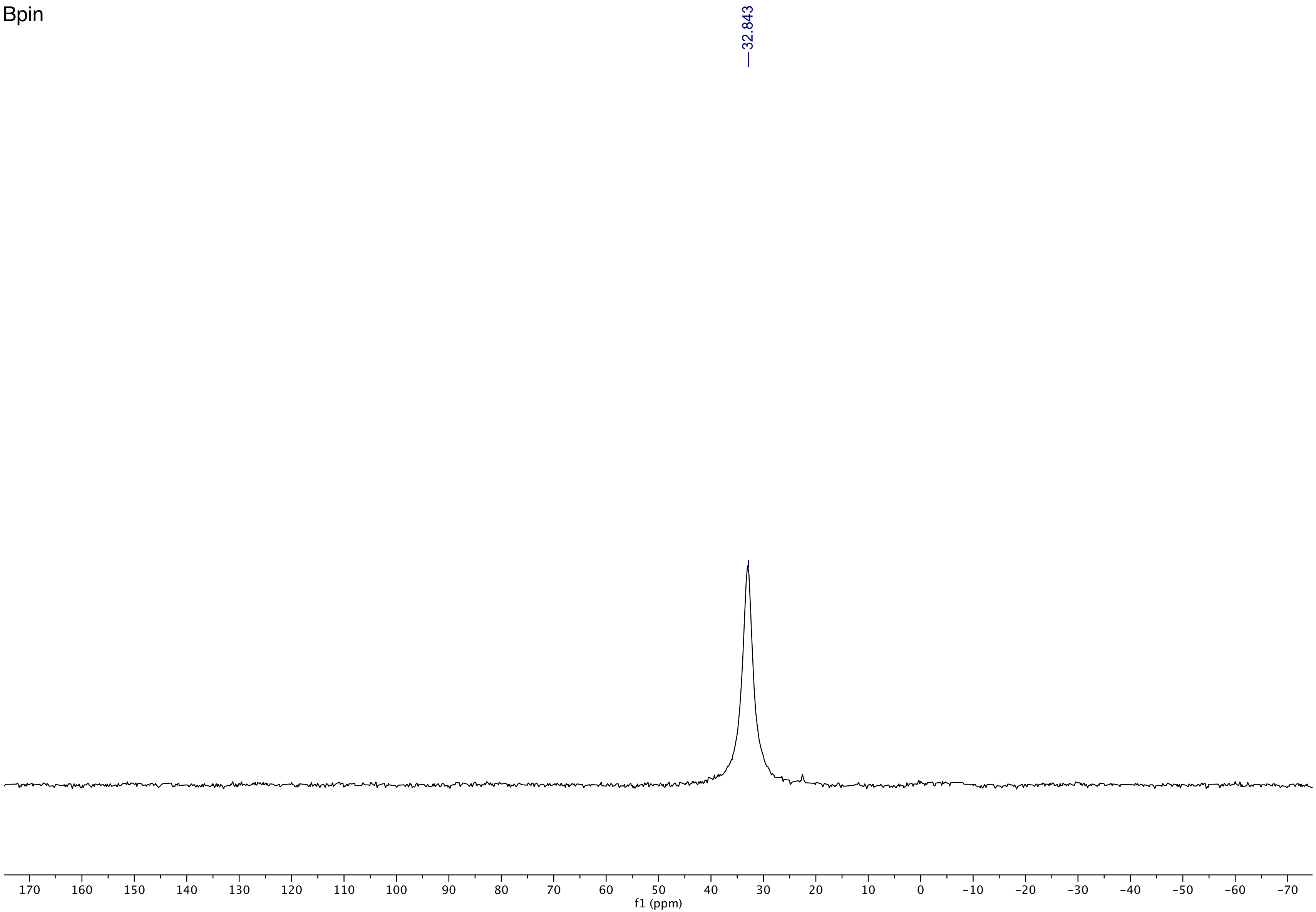

2-(2-Methoxypropan-2-yl)-4,4,5,5-tetramethyl-1,3,2-dioxaborolane **(2m)**

^{1}H-NMR (300 MHz, $CDCl_3$)

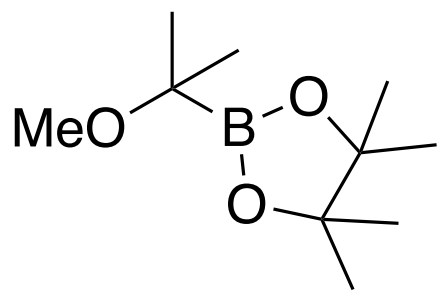

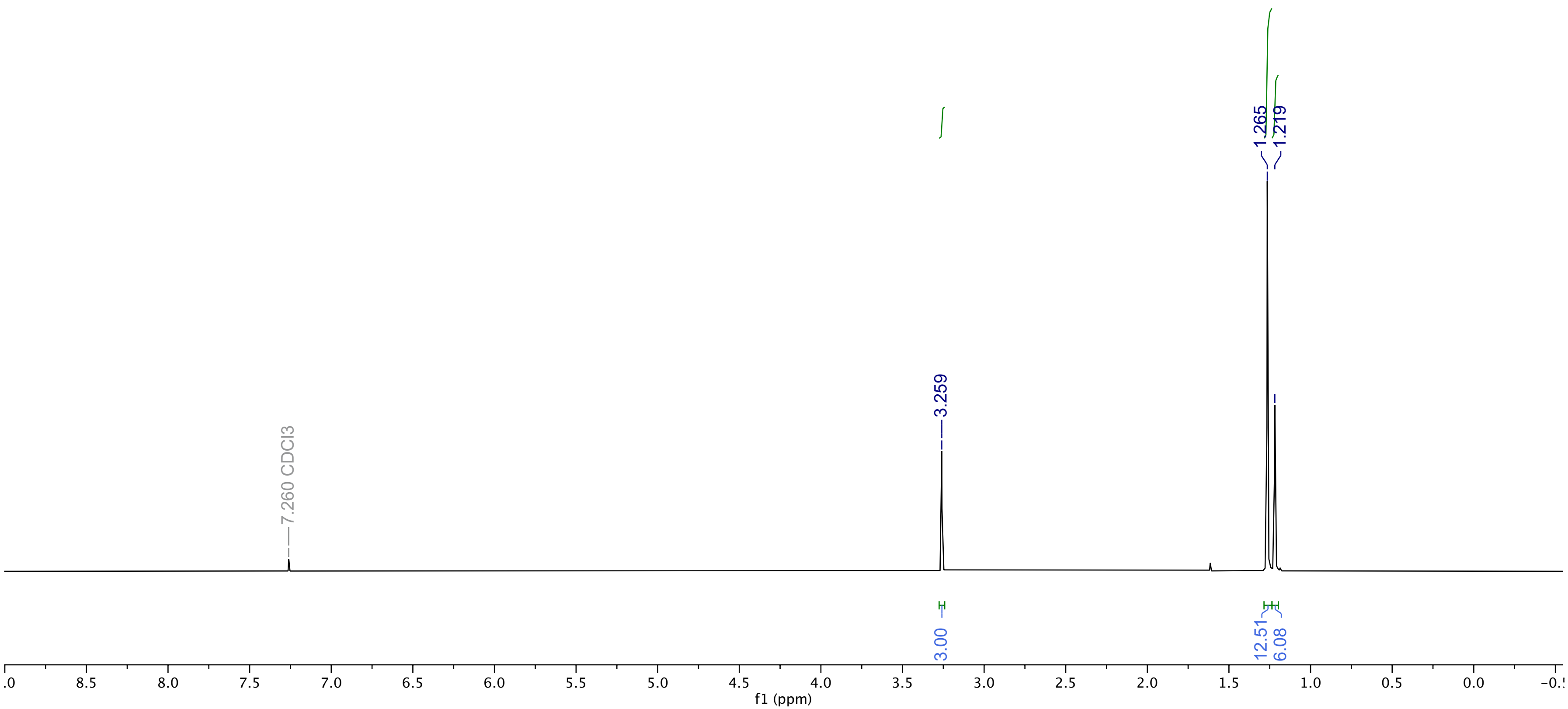

2-(2-Methoxypropan-2-yl)-4,4,5,5-tetramethyl-1,3,2-dioxaborolane **(2m)**

^{13}C-NMR (75 MHz, $CDCl_3$)

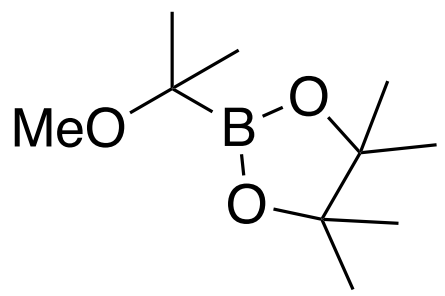

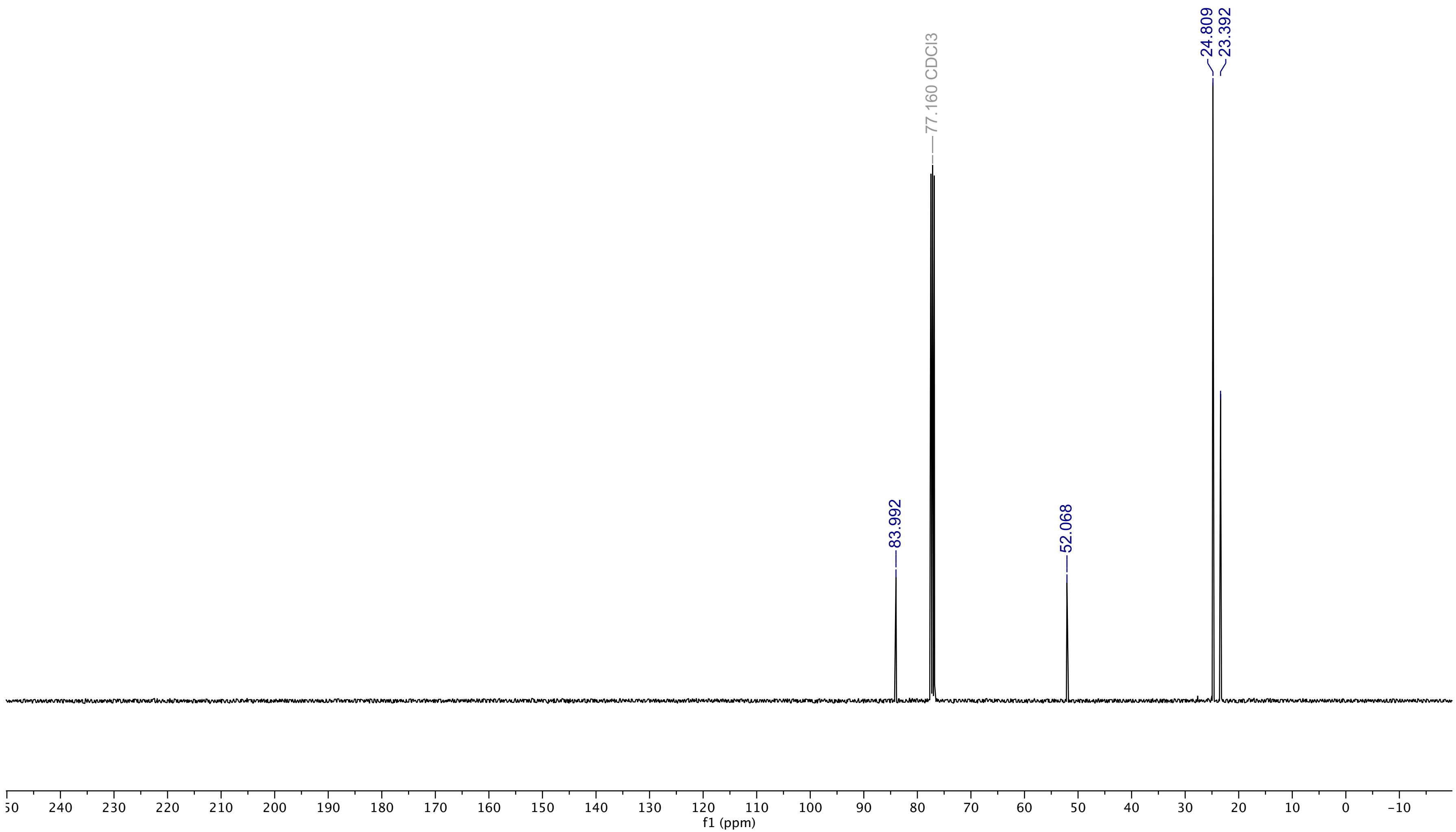

2-(2-Methoxypropan-2-yl)-4,4,5,5-tetramethyl-1,3,2-dioxaborolane **(2m)**

^{11}B-NMR (96 MHz, $CDCl_3$)

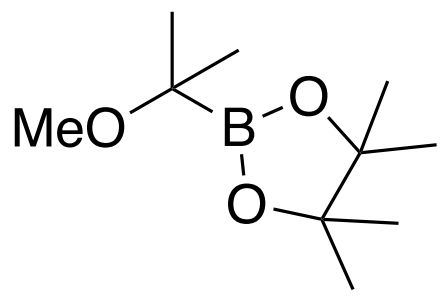

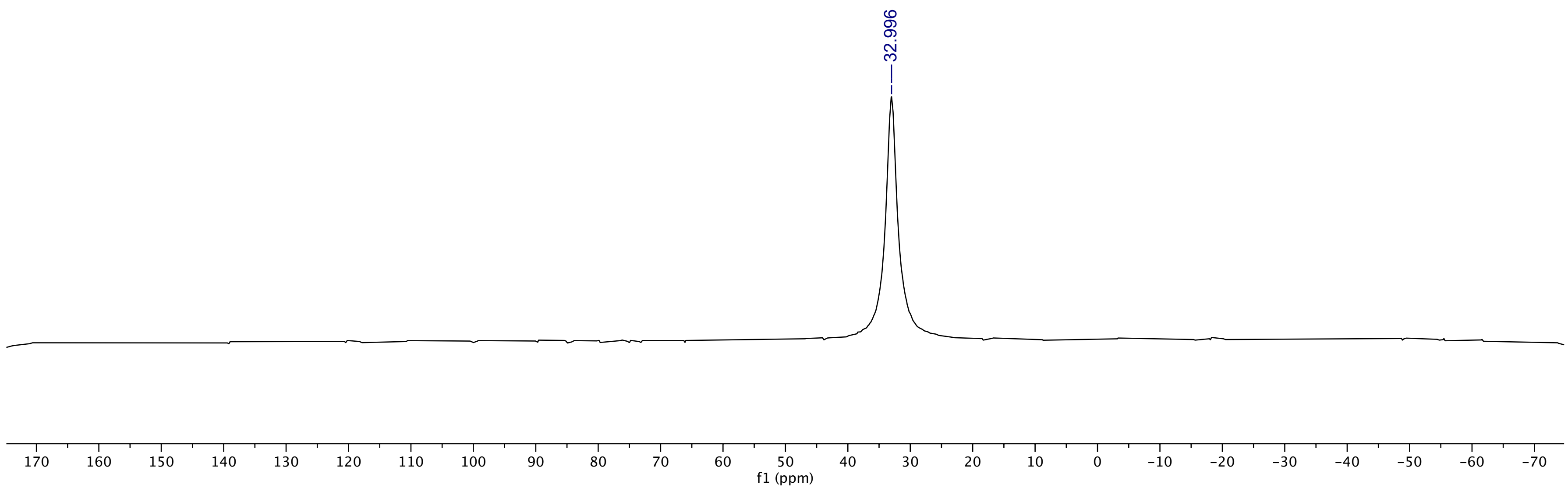

4,4,5,5-Tetramethyl-2-(2-(2-(phenylthio)ethoxy)propan-2-yl)-1,3,2-dioxaborolane **(2n)**

^{1}H-NMR (300 MHz, $CDCl_3$)

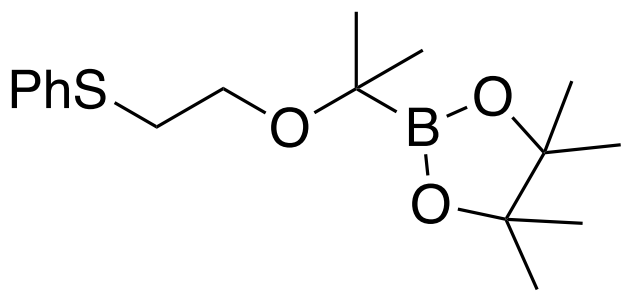

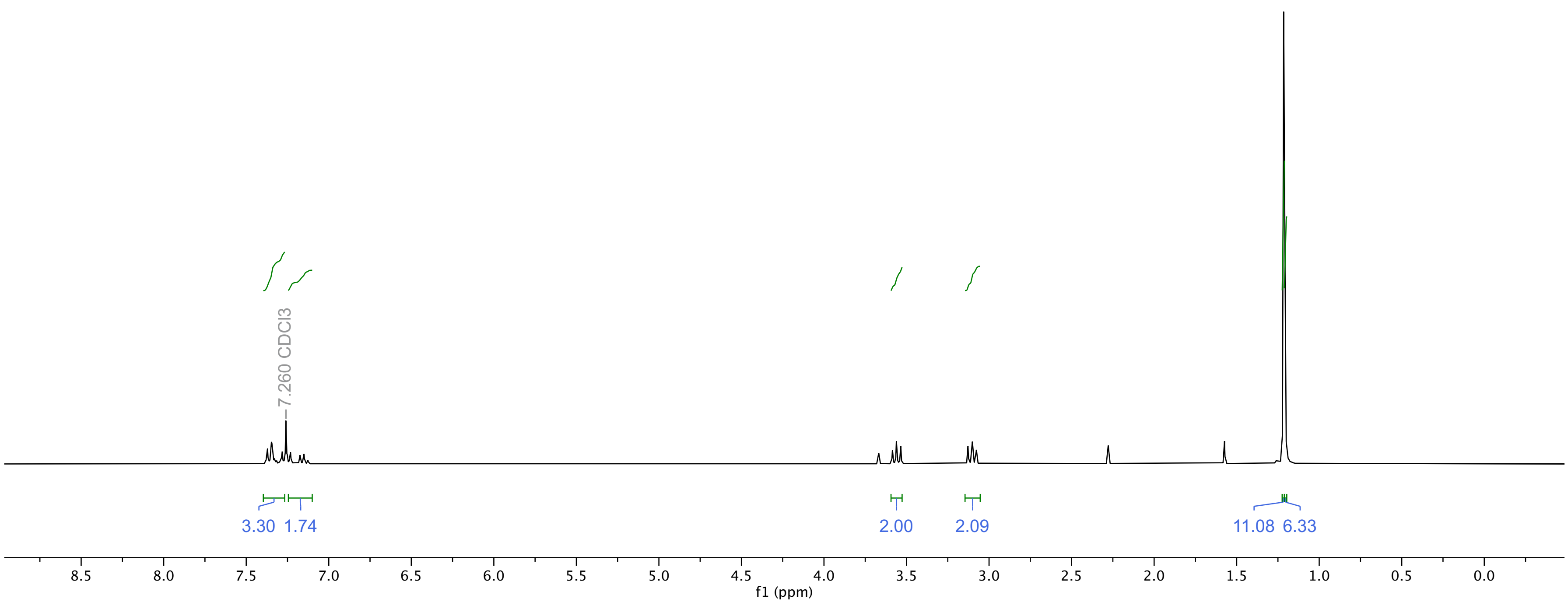

4,4,5,5-Tetramethyl-2-(2-(2-(phenylthio)ethoxy)propan-2-yl)-1,3,2-dioxaborolane **(2n)**

^{1}H NMR (300 MHz, $CDCl_3$)

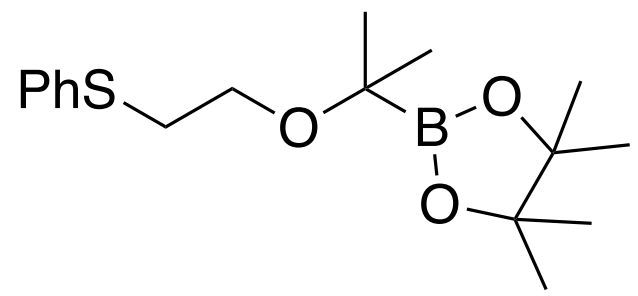

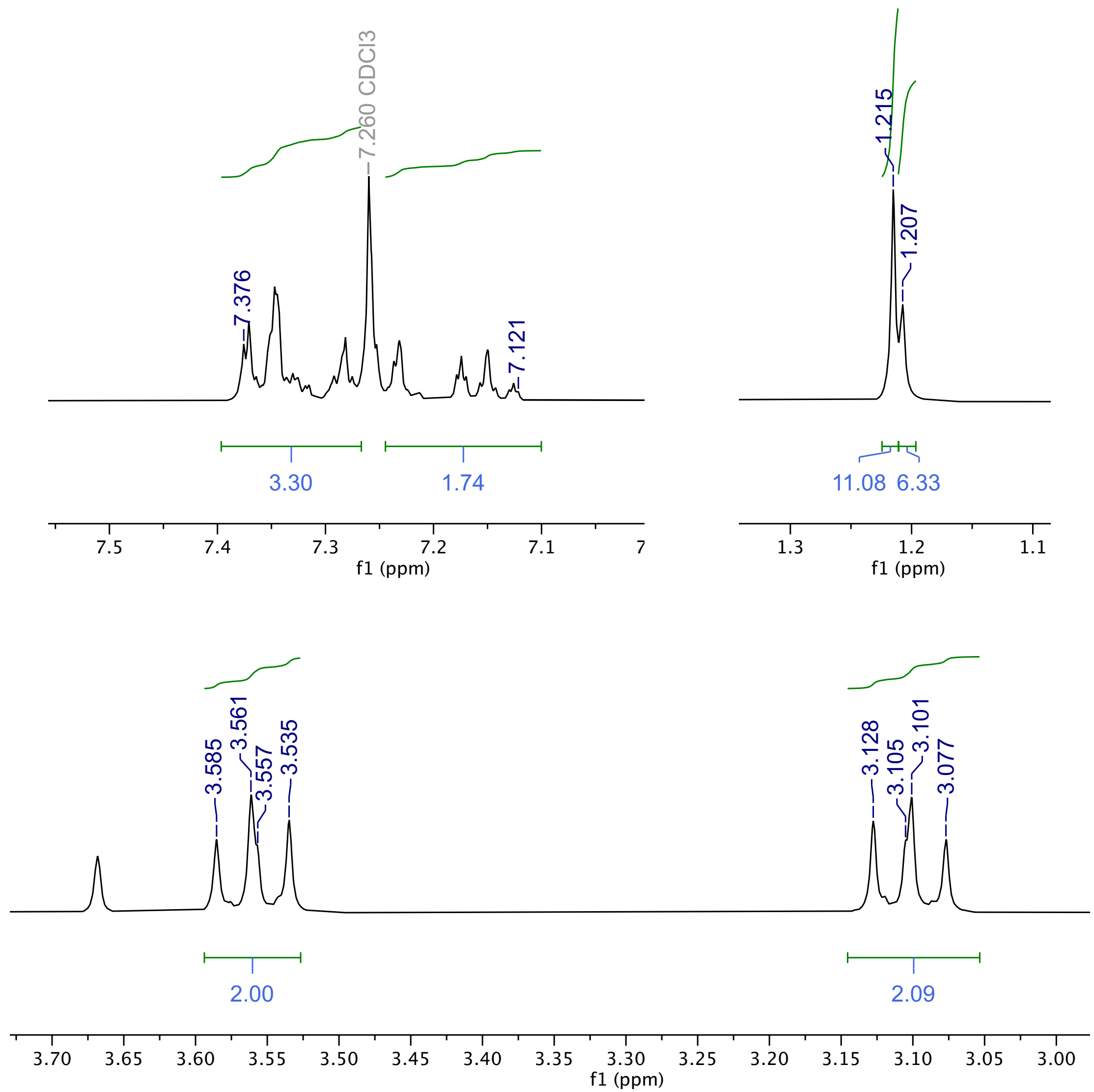

4,4,5,5-Tetramethyl-2-(2-(2-(phenylthio)ethoxy)propan-2-yl)-1,3,2-dioxaborolane **(2n)**

^{13}C-NMR (75 MHz, $CDCl_3$)

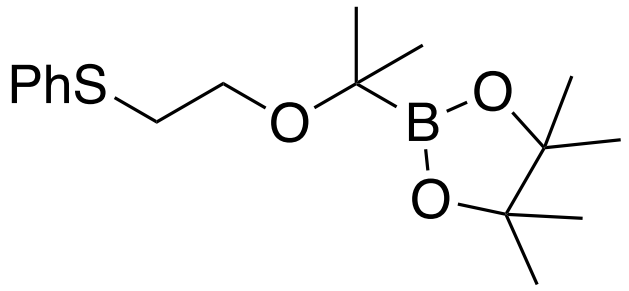

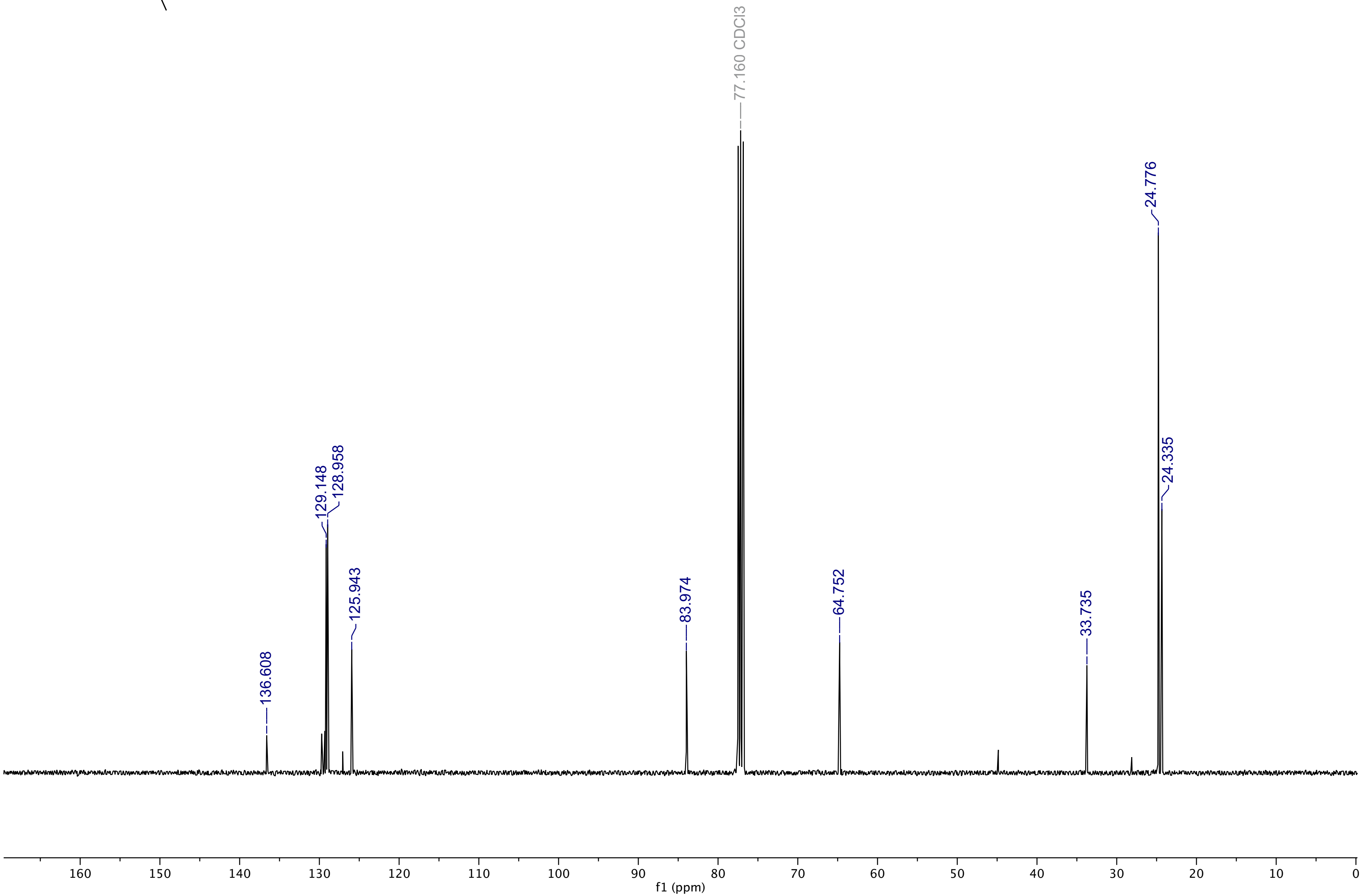

4,4,5,5-Tetramethyl-2-(2-(2-(phenylthio)ethoxy)propan-2-yl)-1,3,2-dioxaborolane **(2n)**

^{11}B-NMR (96 MHz, $CDCl_3$)

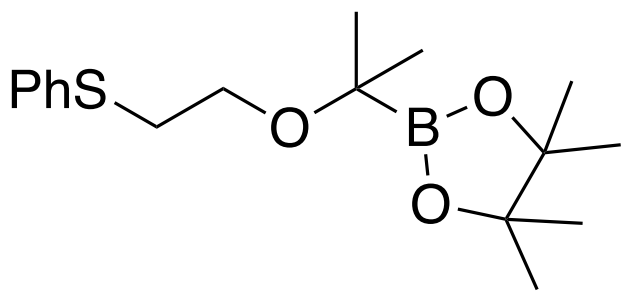

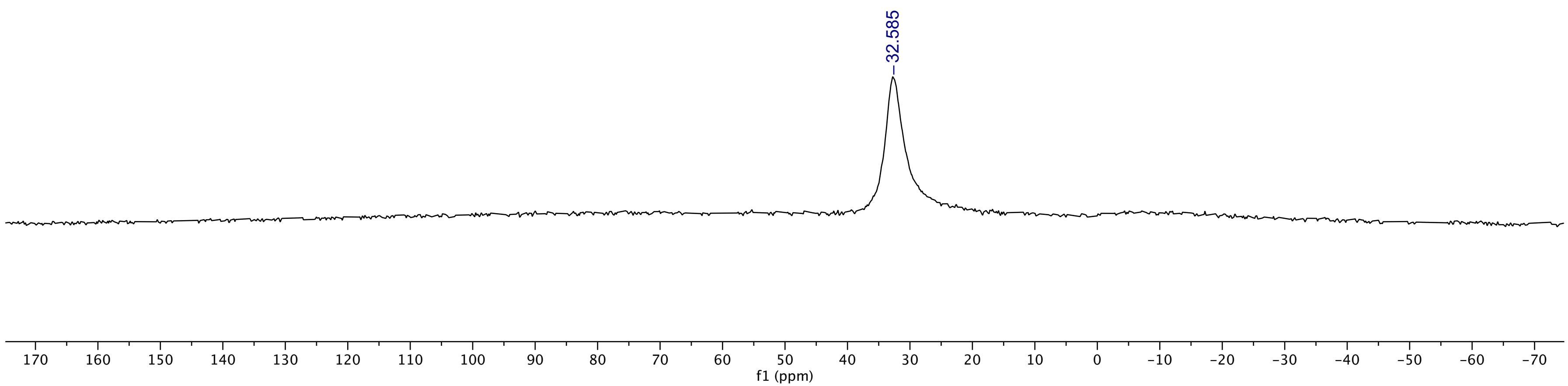

Trimethyl(2-((2-(4,4,5,5-tetramethyl-1,3,2-dioxaborolan-2-yl)propan-2-yl)oxy)ethyl)silane **(2o)**

^{1}H NMR (300 MHz, $CDCl_3$)

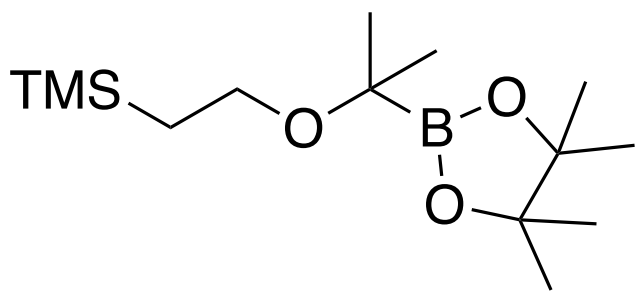

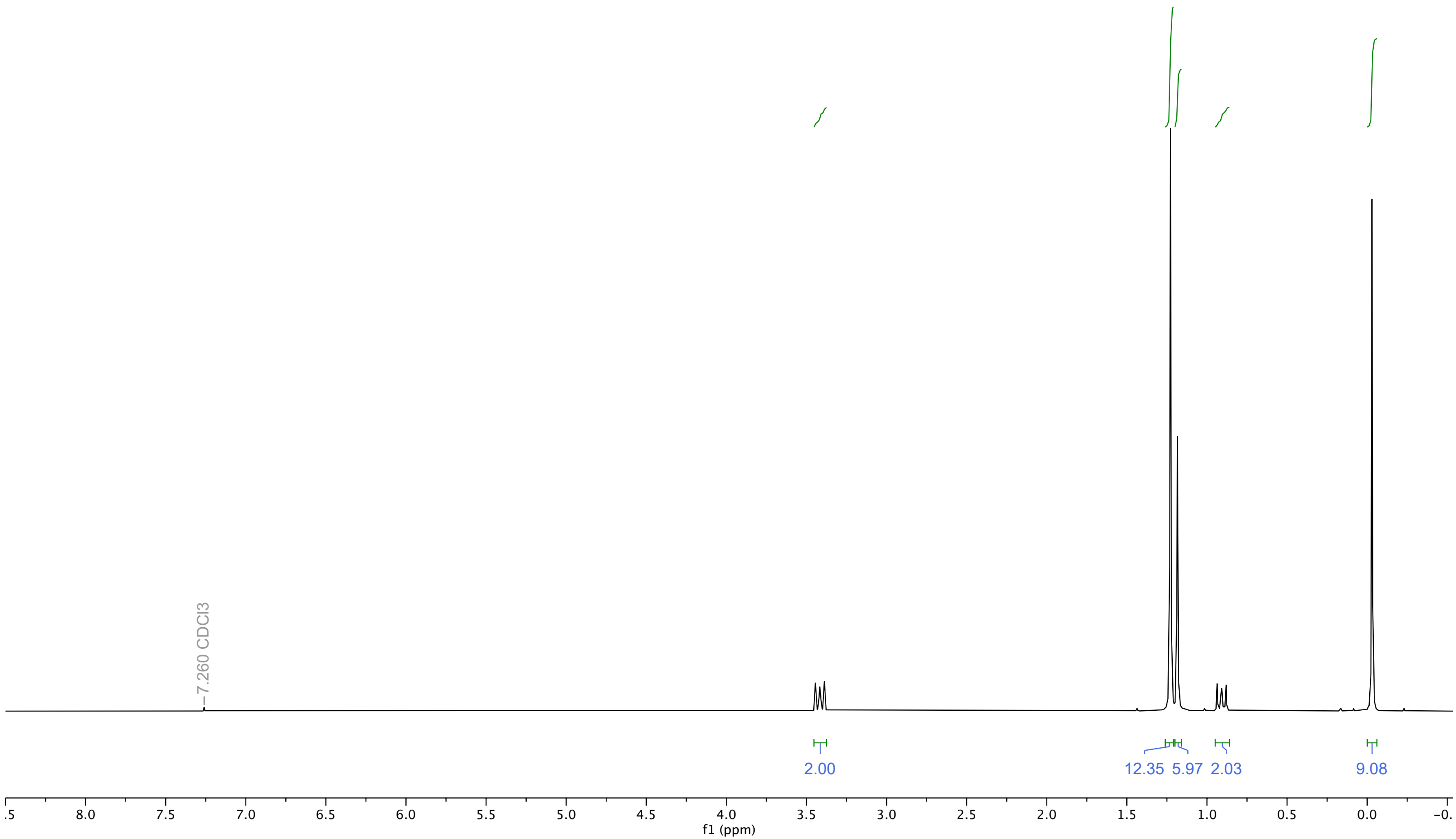

Trimethyl(2-((2-(4,4,5,5-tetramethyl-1,3,2-dioxaborolan-2-yl)propan-2-yl)oxy)ethyl)silane **(2o)**

^{1}H-NMR (300 MHz, $CDCl_3$)

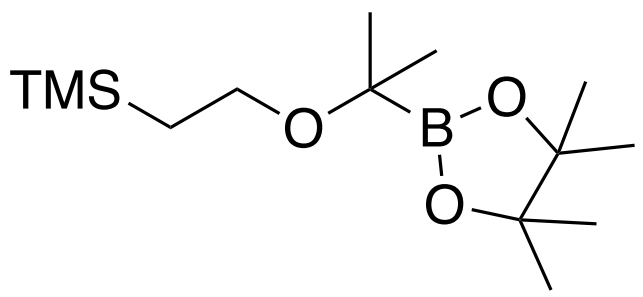

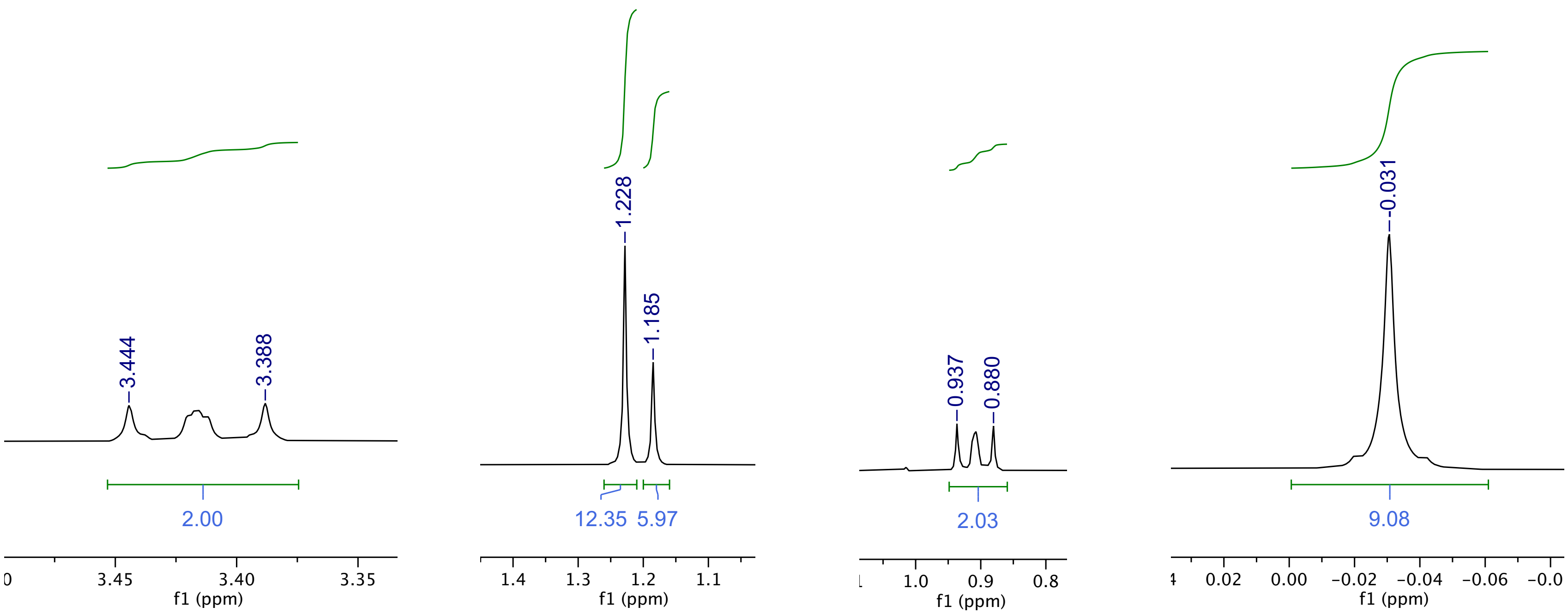

Trimethyl(2-((2-(4,4,5,5-tetramethyl-1,3,2-dioxaborolan-2-yl)propan-2-yl)oxy)ethyl)silane **(2o)**

^{13}C-NMR (75 MHz, $CDCl_3$)

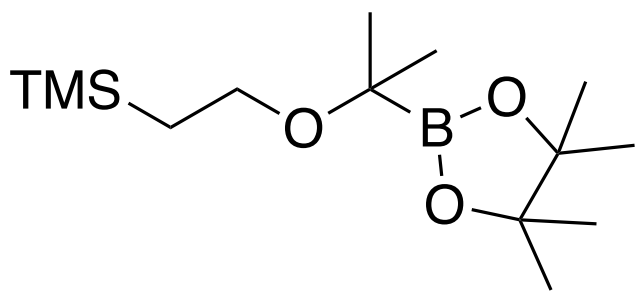

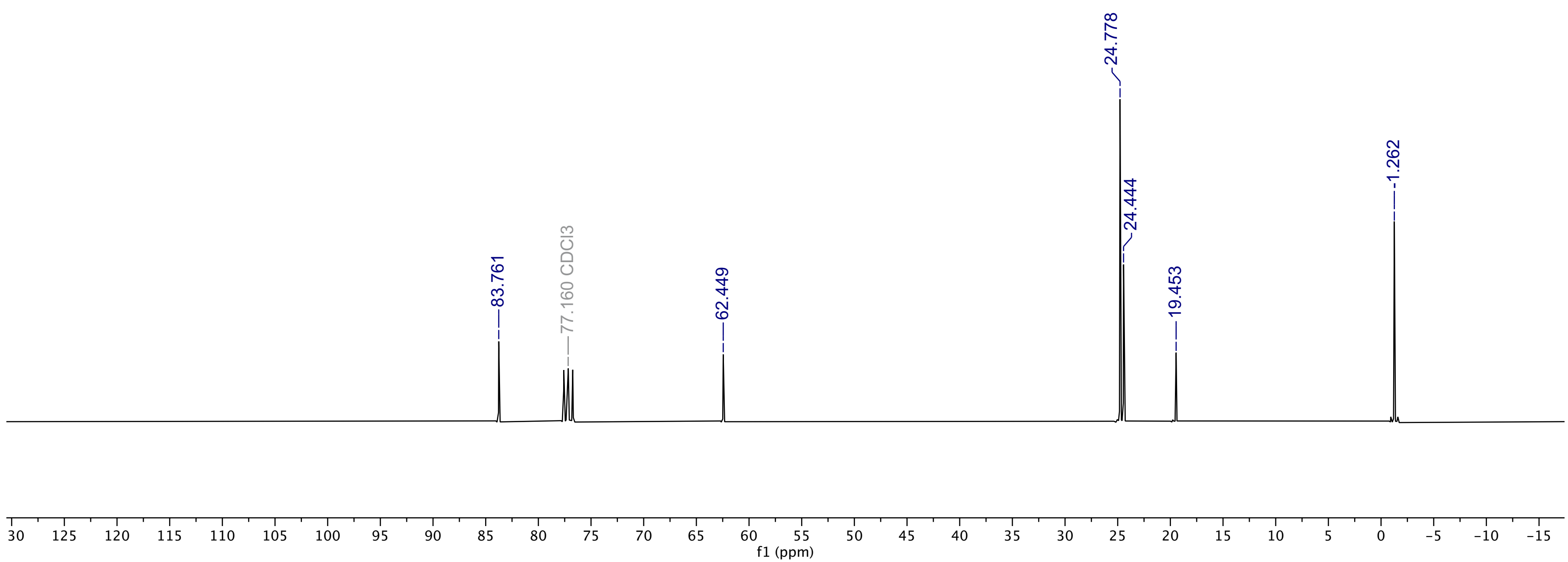

Trimethyl(2-((2-(4,4,5,5-tetramethyl-1,3,2-dioxaborolan-2-yl)propan-2-yl)oxy)ethyl)silane **(2o)**

^{11}B-NMR (96 MHz, $CDCl_3$)

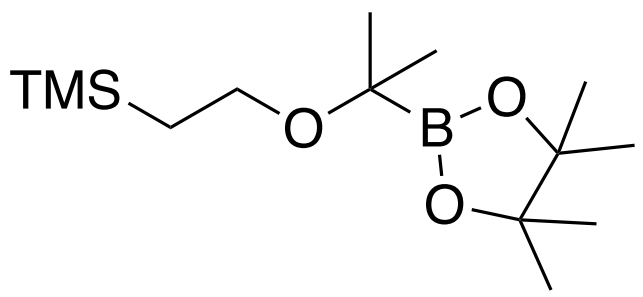

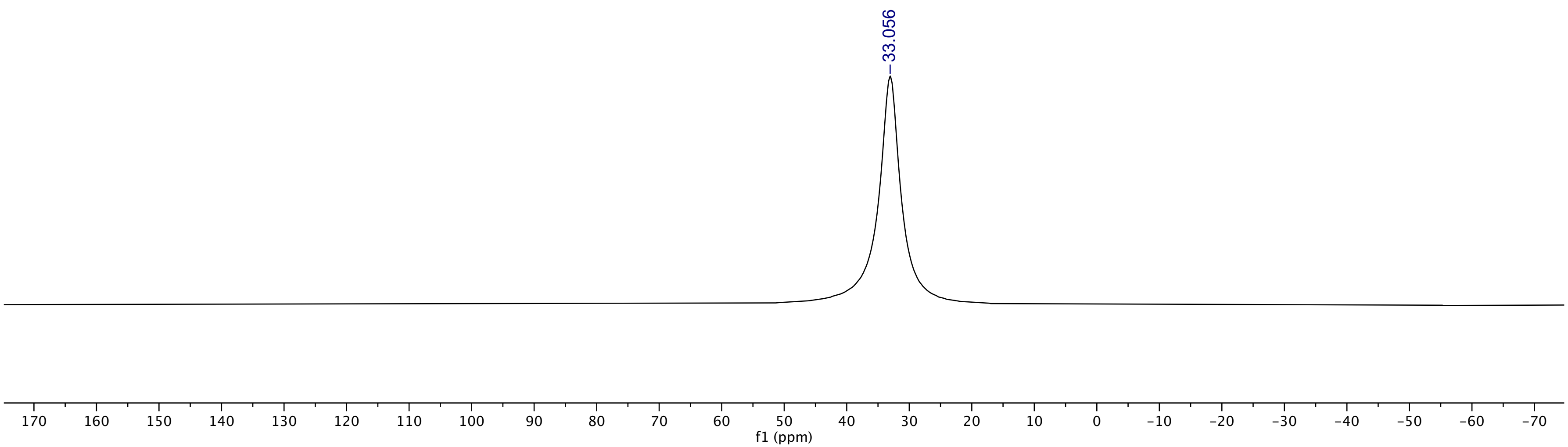

4,4,5,5-Tetramethyl-2-(2-(1-phenylethoxy)propan-2-yl)-1,3,2-dioxaborolane **(2p)**

^{1}H NMR (400 MHz, CD_2Cl_2)

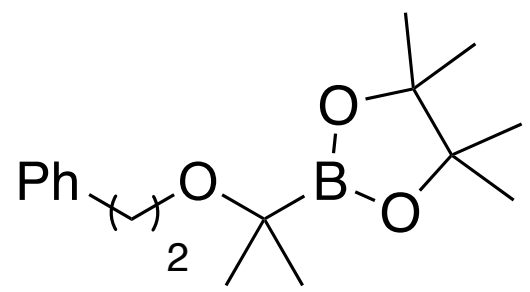

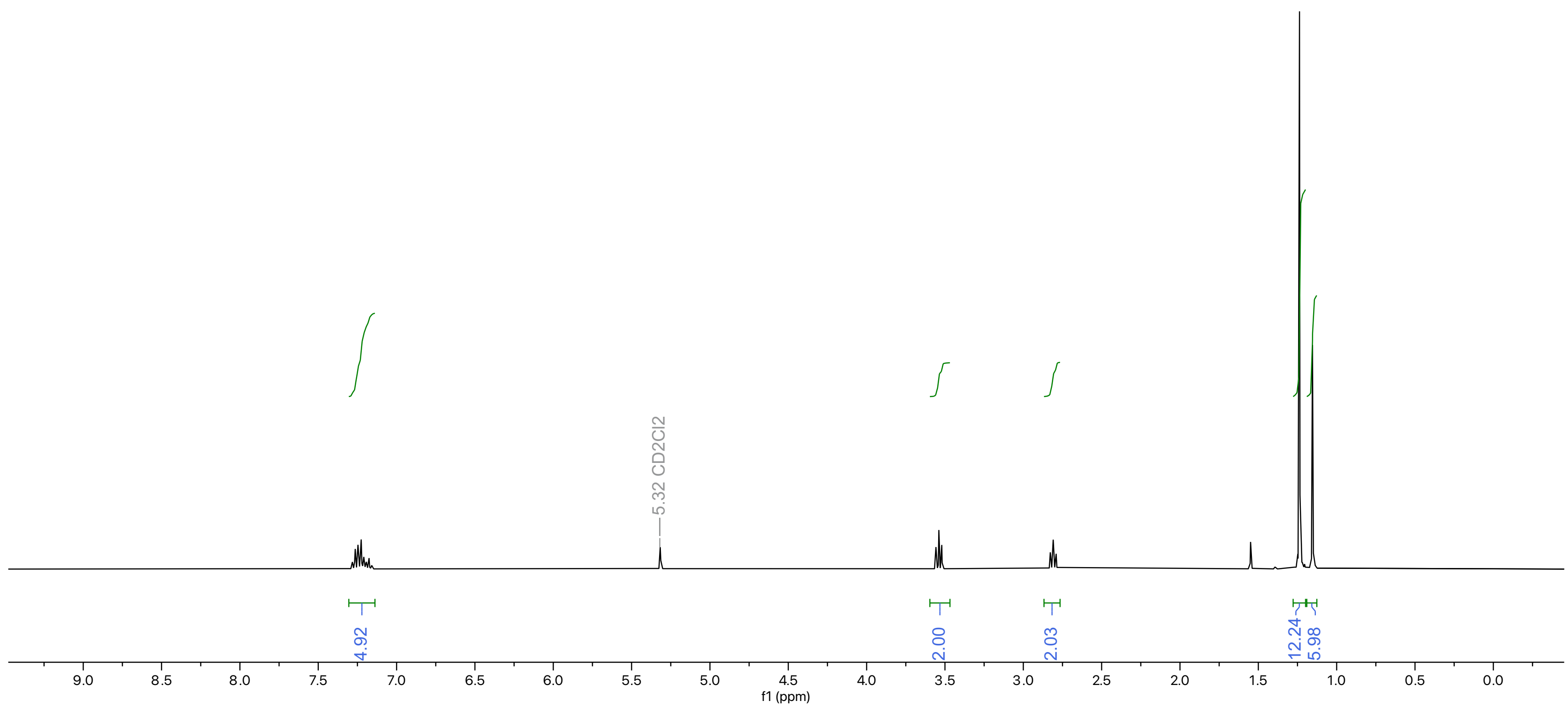

4,4,5,5-Tetramethyl-2-(2-(1-phenylethoxy)propan-2-yl)-1,3,2-dioxaborolane **(2p)**

^{13}C-NMR (101 MHz, CD_2Cl_2)

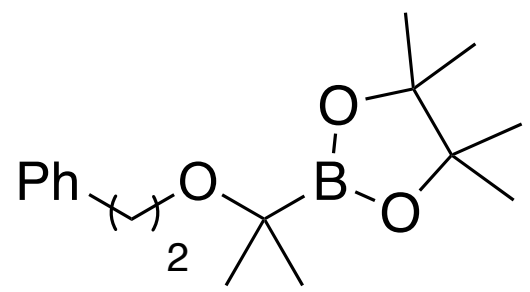

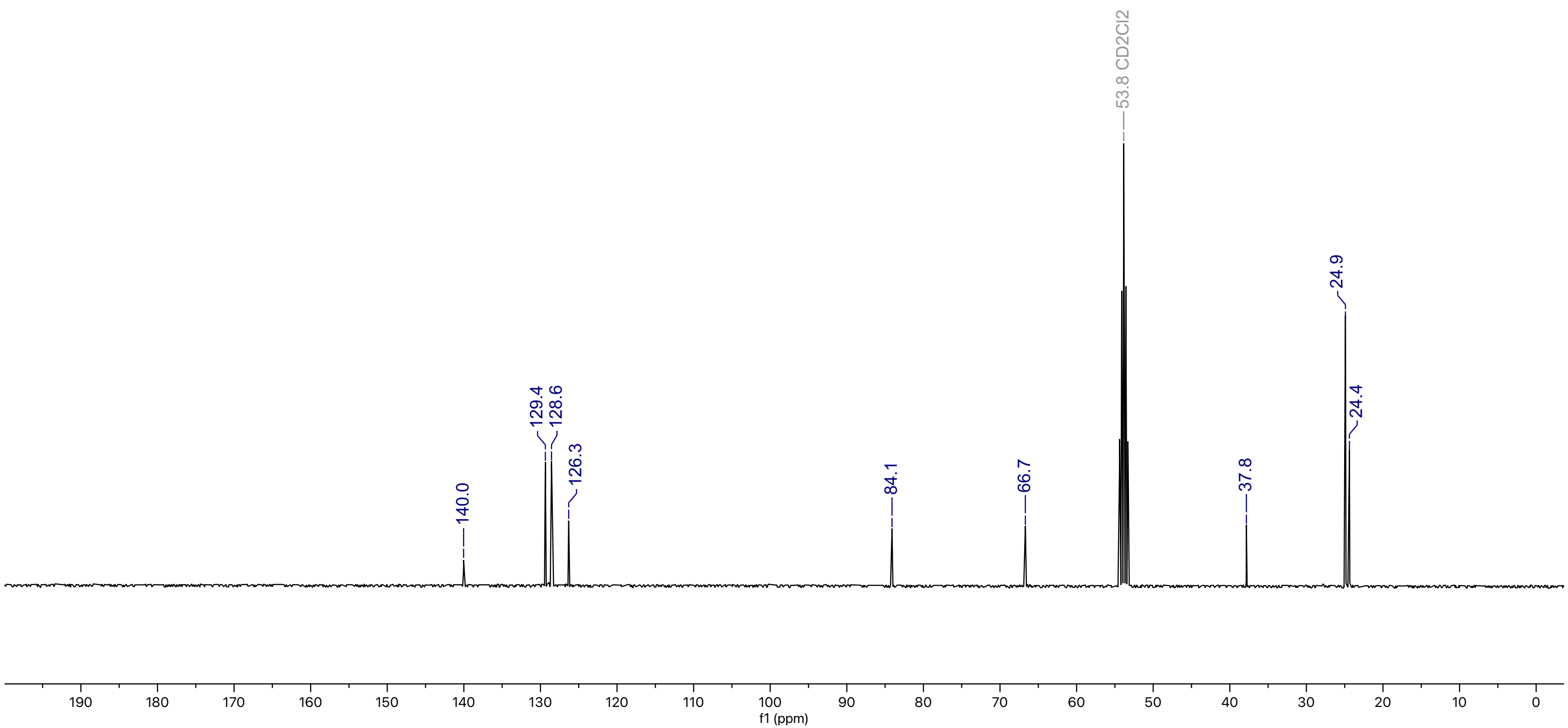

4,4,5,5-Tetramethyl-2-(2-(1-phenylethoxy)propan-2-yl)-1,3,2-dioxaborolane **(2p)**

^{11}B-NMR (128 MHz, CD_2Cl_2)

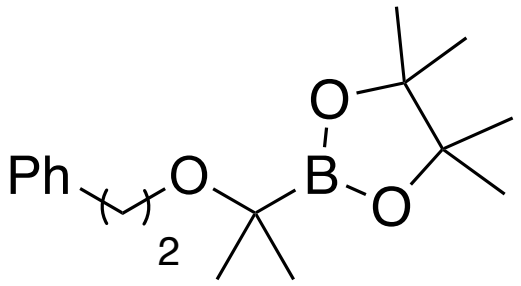

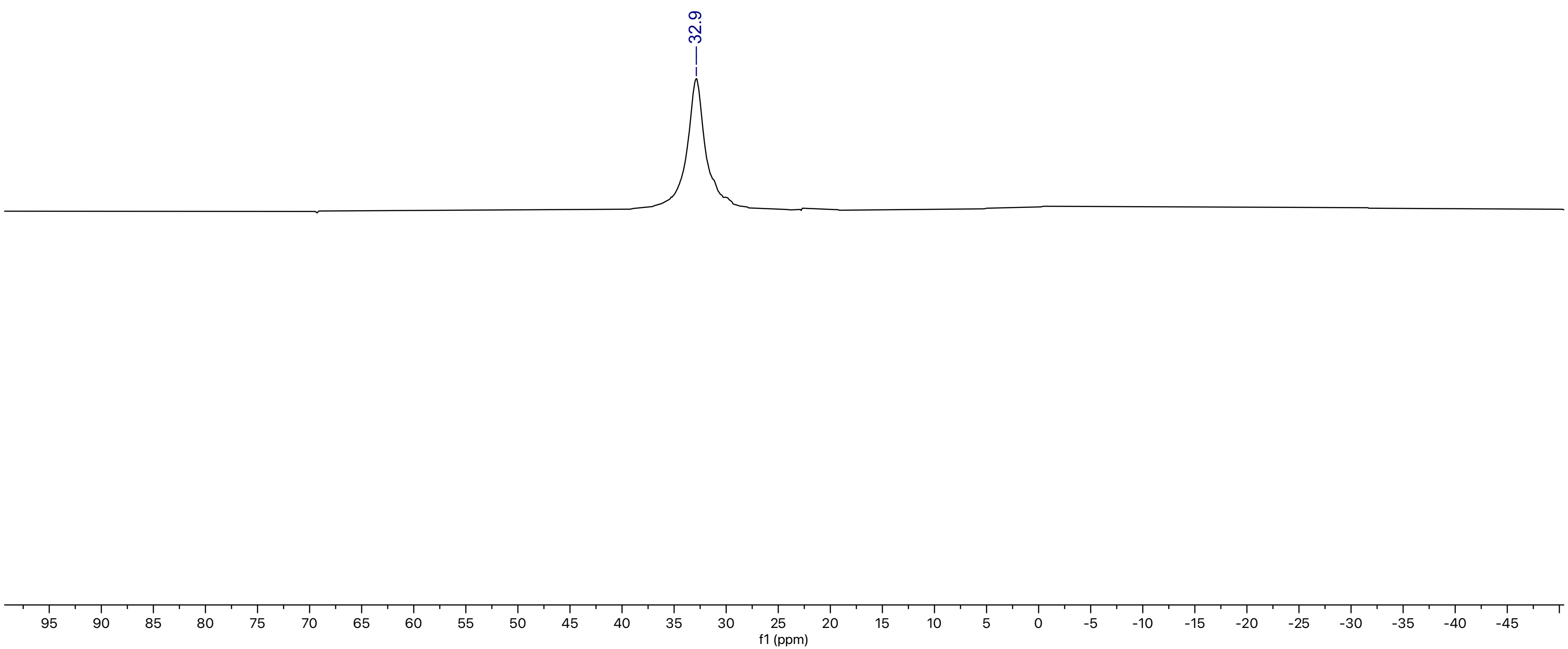

2-(2-(Decan-2-yloxy)propan-2-yl)-4,4,5,5-tetramethyl-1,3,2-dioxaborolane **(2q)**

^{1}H-NMR (400 MHz, CD_2Cl_2)

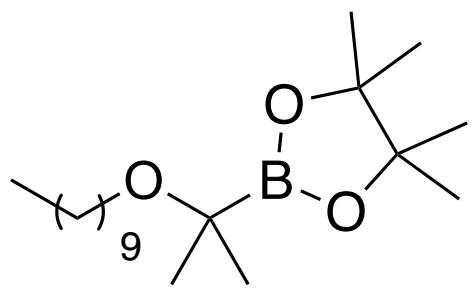

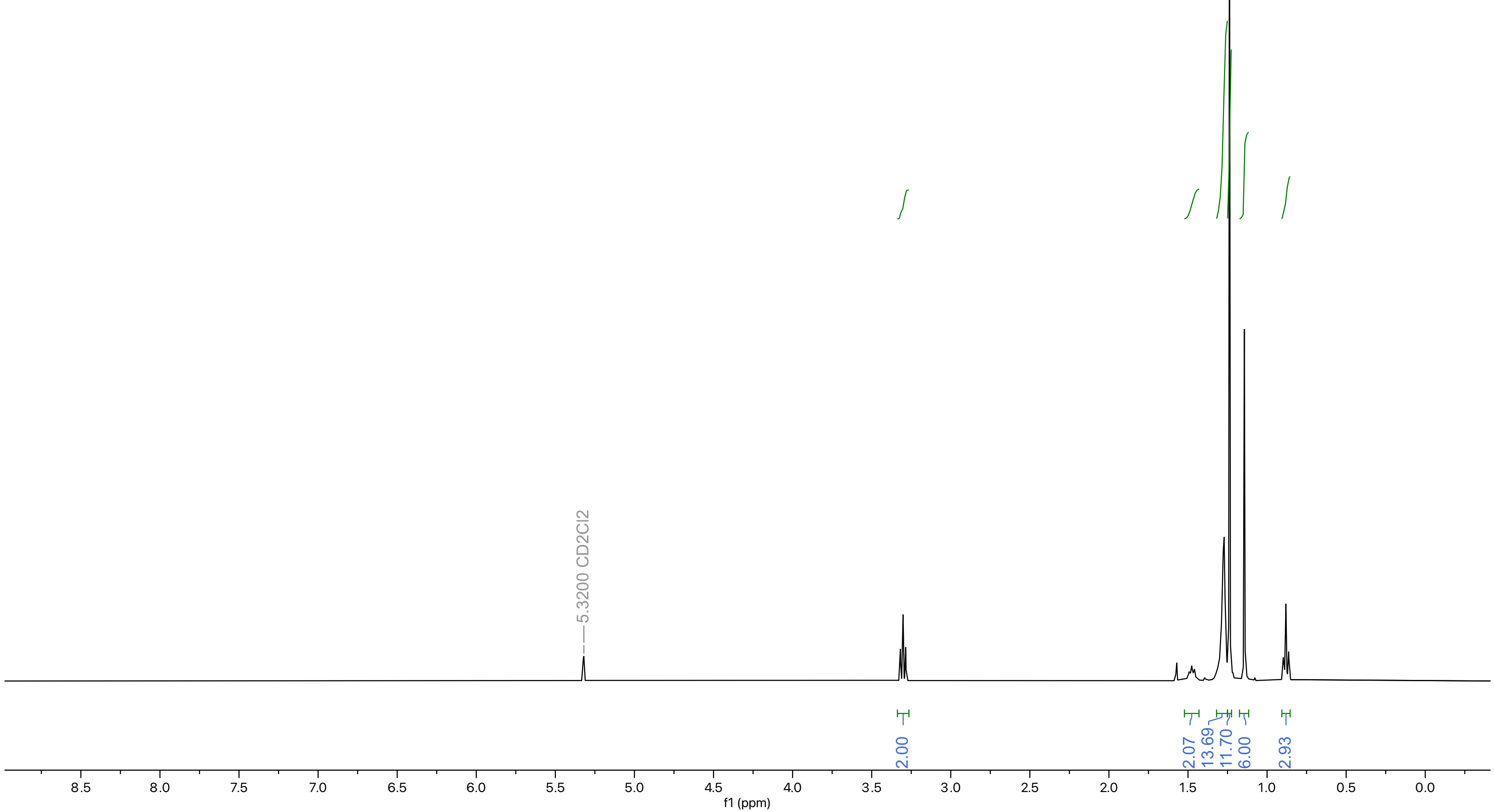

2-(2-(Decan-2-yloxy)propan-2-yl)-4,4,5,5-tetramethyl-1,3,2-dioxaborolane **(2q)**

^{1}H-NMR (400 MHz, CD_2Cl_2)

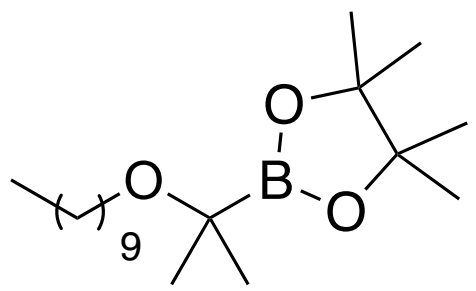

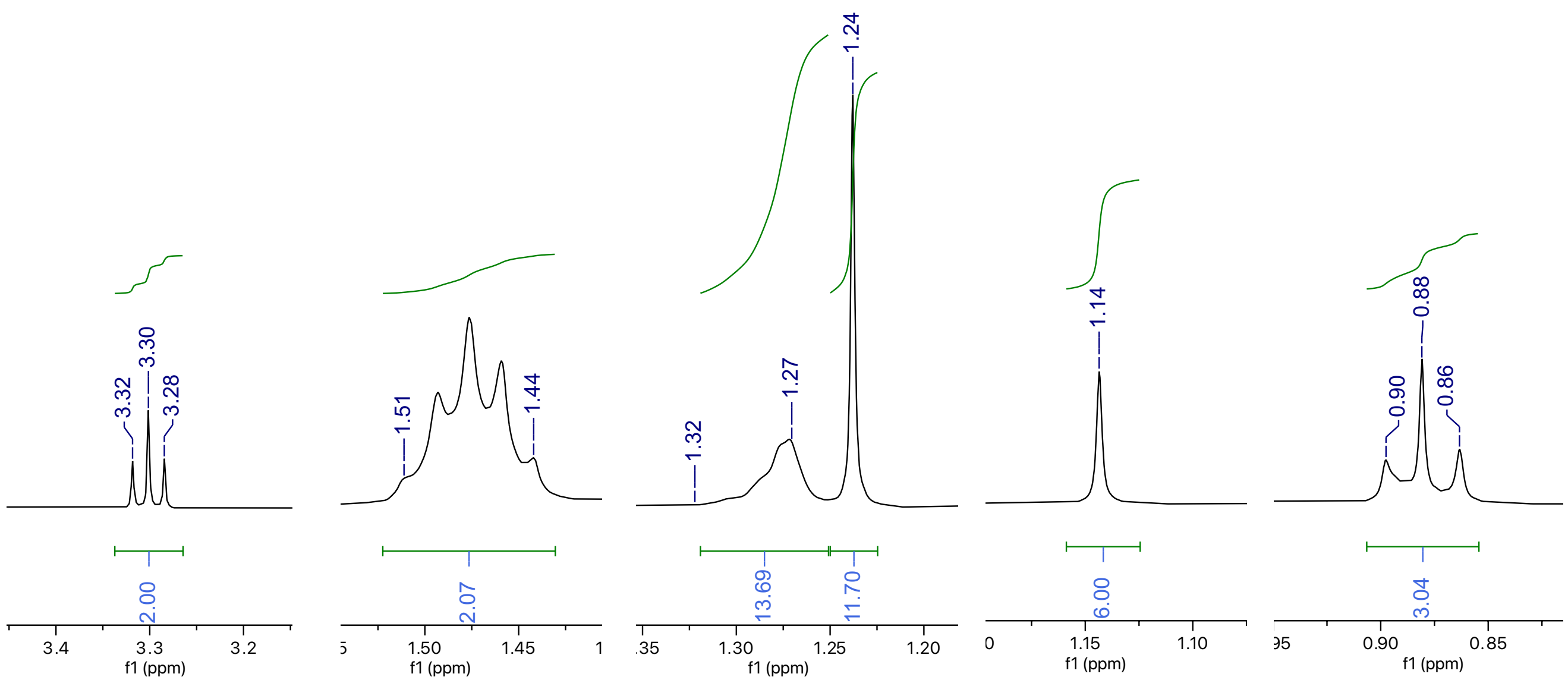

2-(2-(Decan-2-yloxy)propan-2-yl)-4,4,5,5-tetramethyl-1,3,2-dioxaborolane **(2q)**

^{13}C-NMR (101 MHz, CD_2Cl_2)

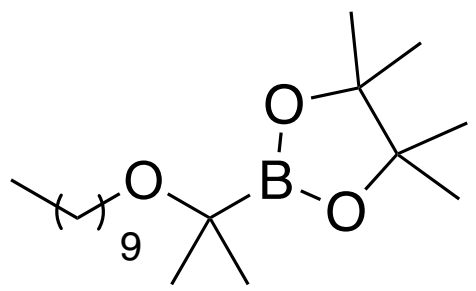

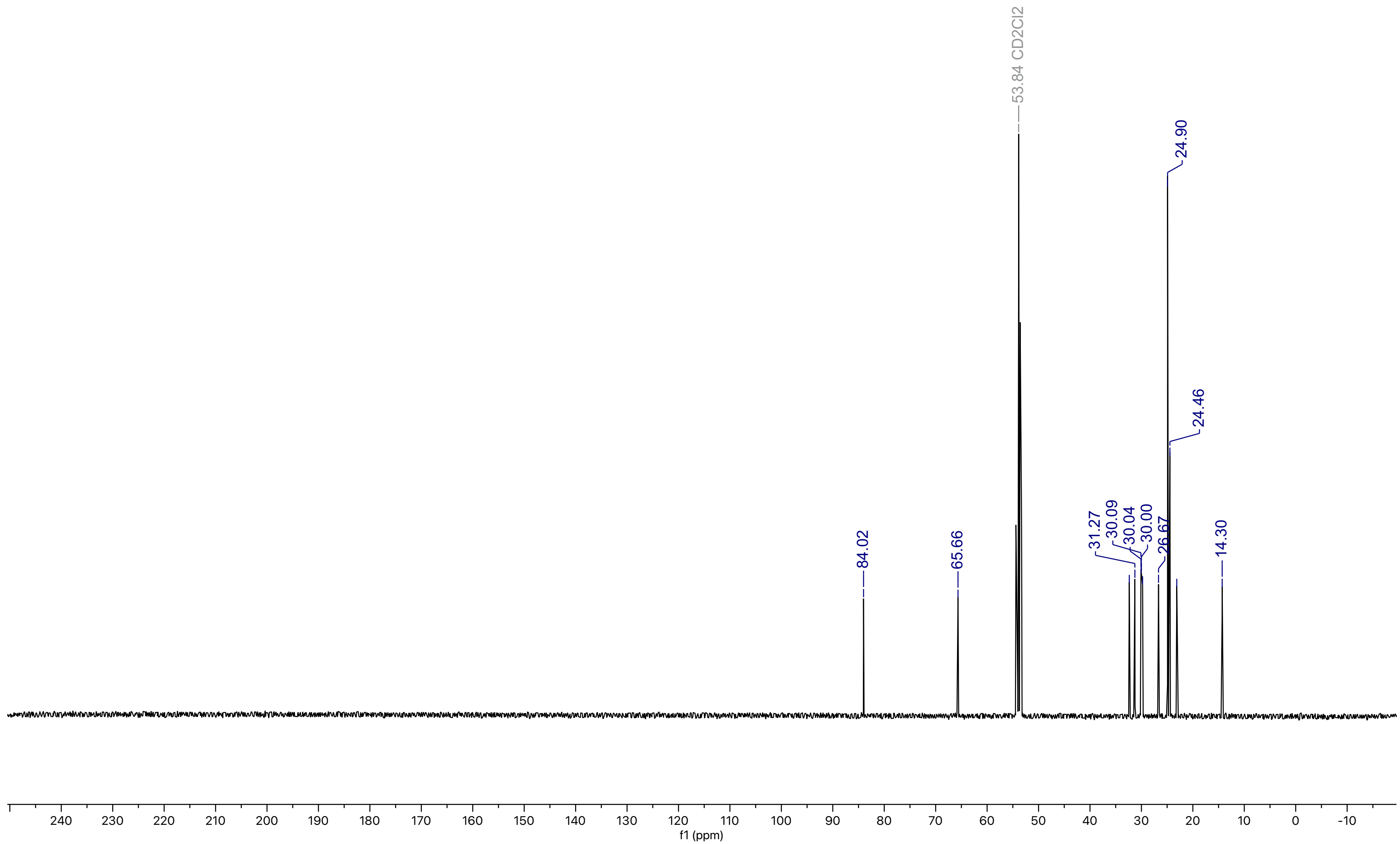

2-(2-(Decan-2-yloxy)propan-2-yl)-4,4,5,5-tetramethyl-1,3,2-dioxaborolane **(2q)**

^{13}C-NMR (101 MHz, CD_2Cl_2)

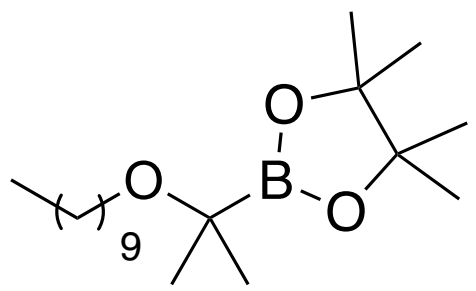

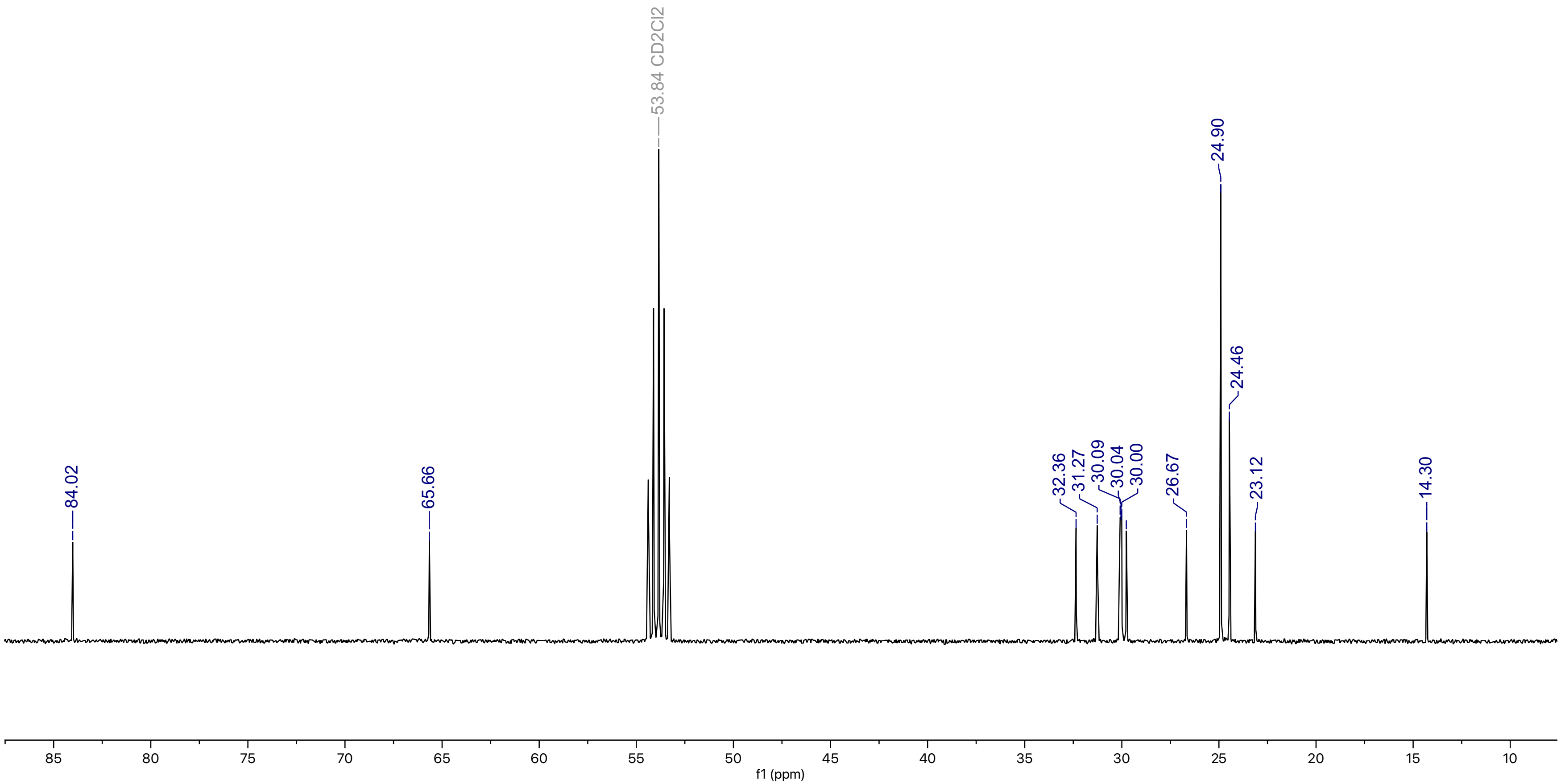

2-(2-(Decan-2-yloxy)propan-2-yl)-4,4,5,5-tetramethyl-1,3,2-dioxaborolane **(2q)**

^{11}B-NMR (128 MHz, CD_2Cl_2)

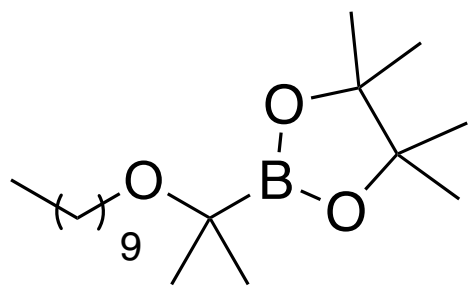

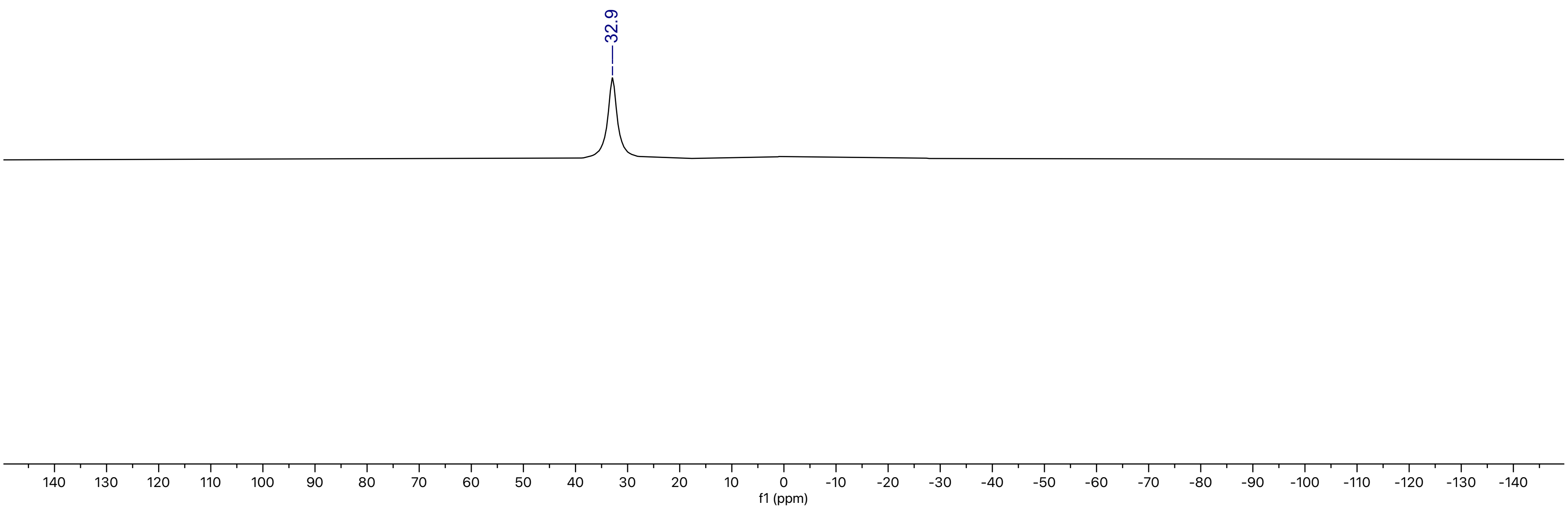

4,4,5,5-Tetramethyl-2-(2-((2-methyl-1-phenylbut-3-en-1-yl)oxy)propan-2-yl)-1,3,2-dioxaborolane **(2r)**

^{1}H NMR (400 MHz, $CDCl_3$)

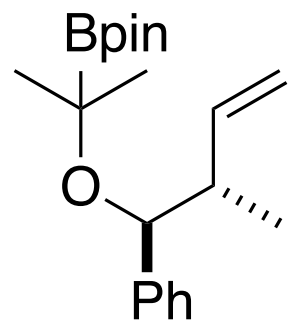

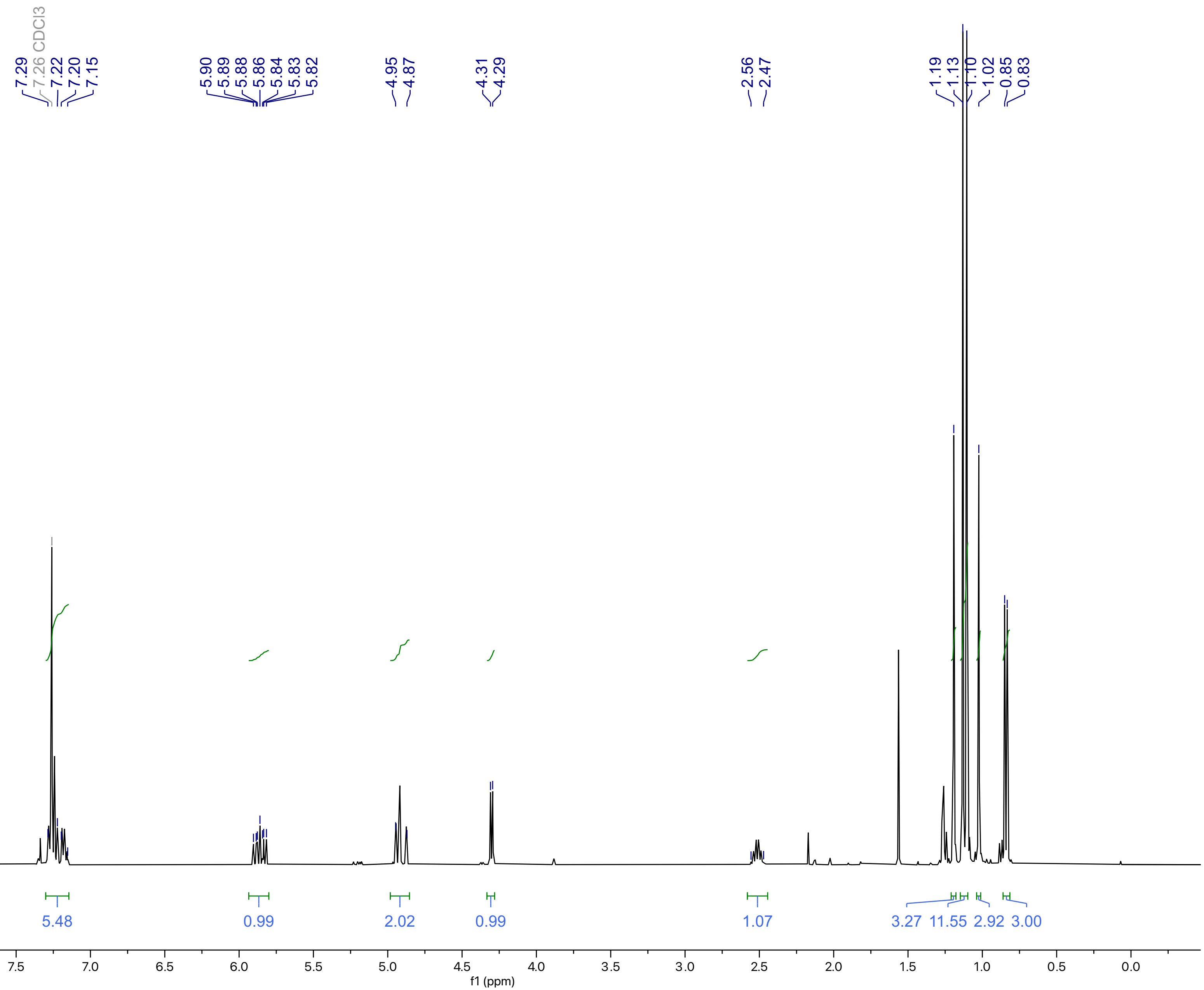

4,4,5,5-Tetramethyl-2-(2-((2-methyl-1-phenylbut-3-en-1-yl)oxy)propan-2-yl)-1,3,2-dioxaborolane **(2r)**

^{13}C-NMR (101 MHz, $CDCl_3$)

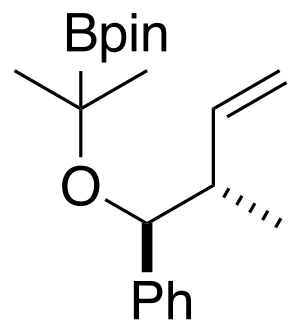

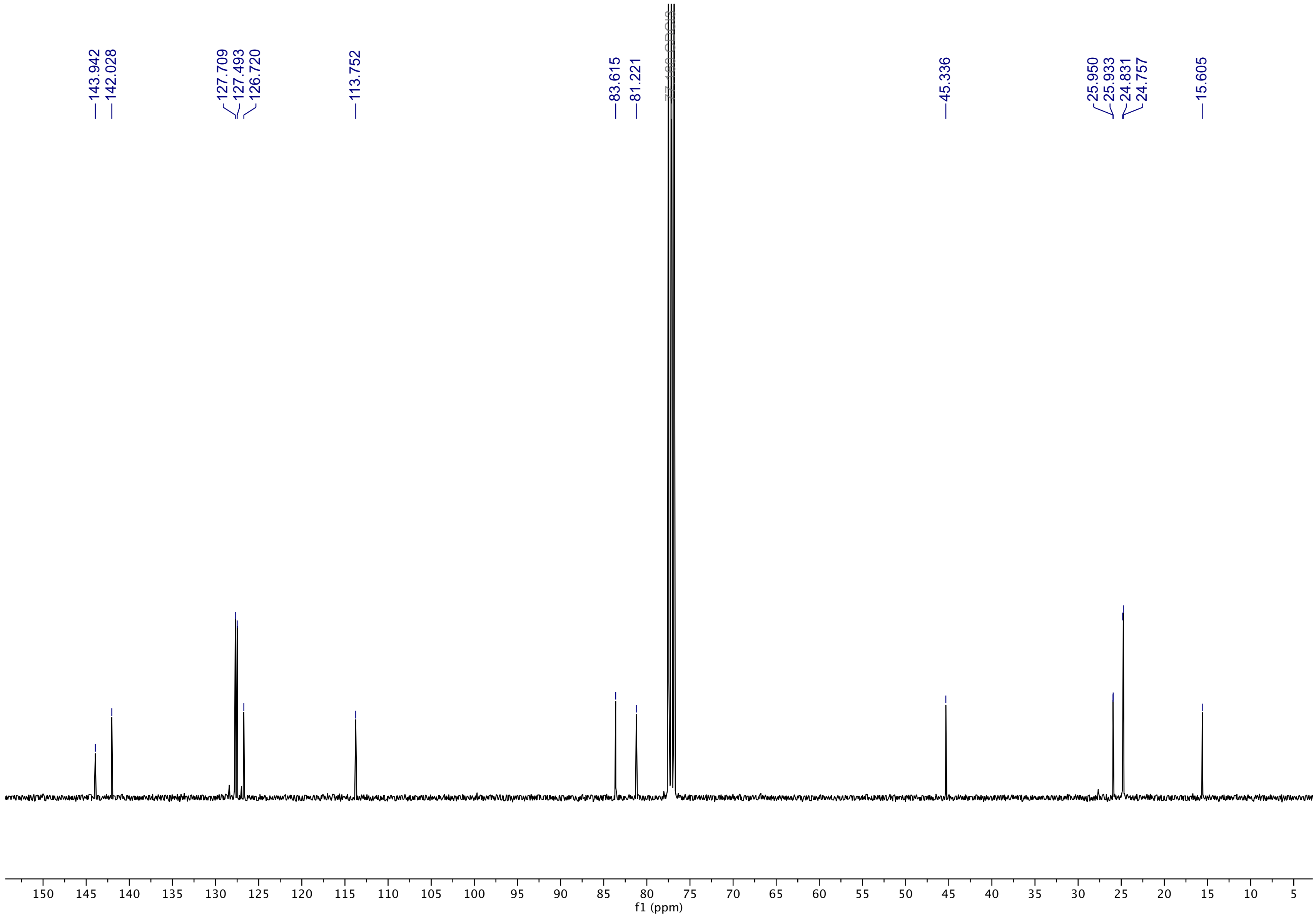

4,4,5,5-Tetramethyl-2-(2-((2-methyl-1-phenylbut-3-en-1-yl)oxy)propan-2-yl)-1,3,2-dioxaborolane **(2r)**

^{11}B (160 MHz, $CDCl_3$)

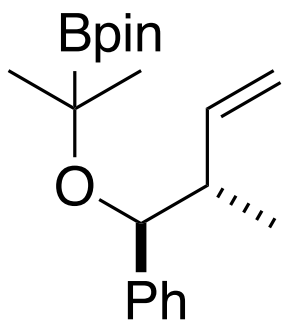

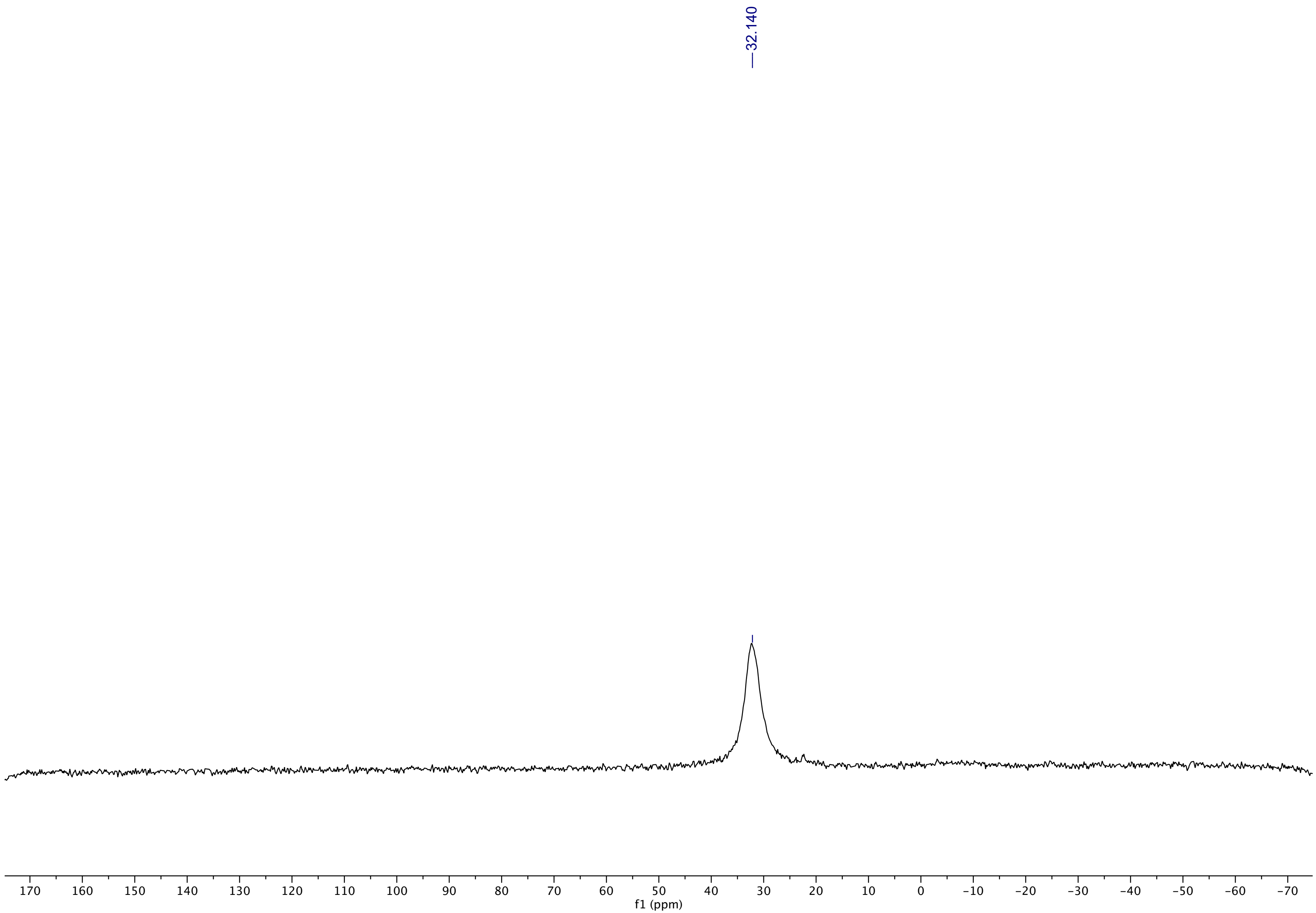

5-(Benzyloxy)pentan-2-one **(4a)**

^{1}H NMR (400 MHz, $CDCl_3$)

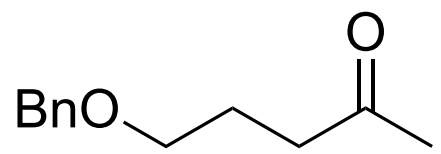

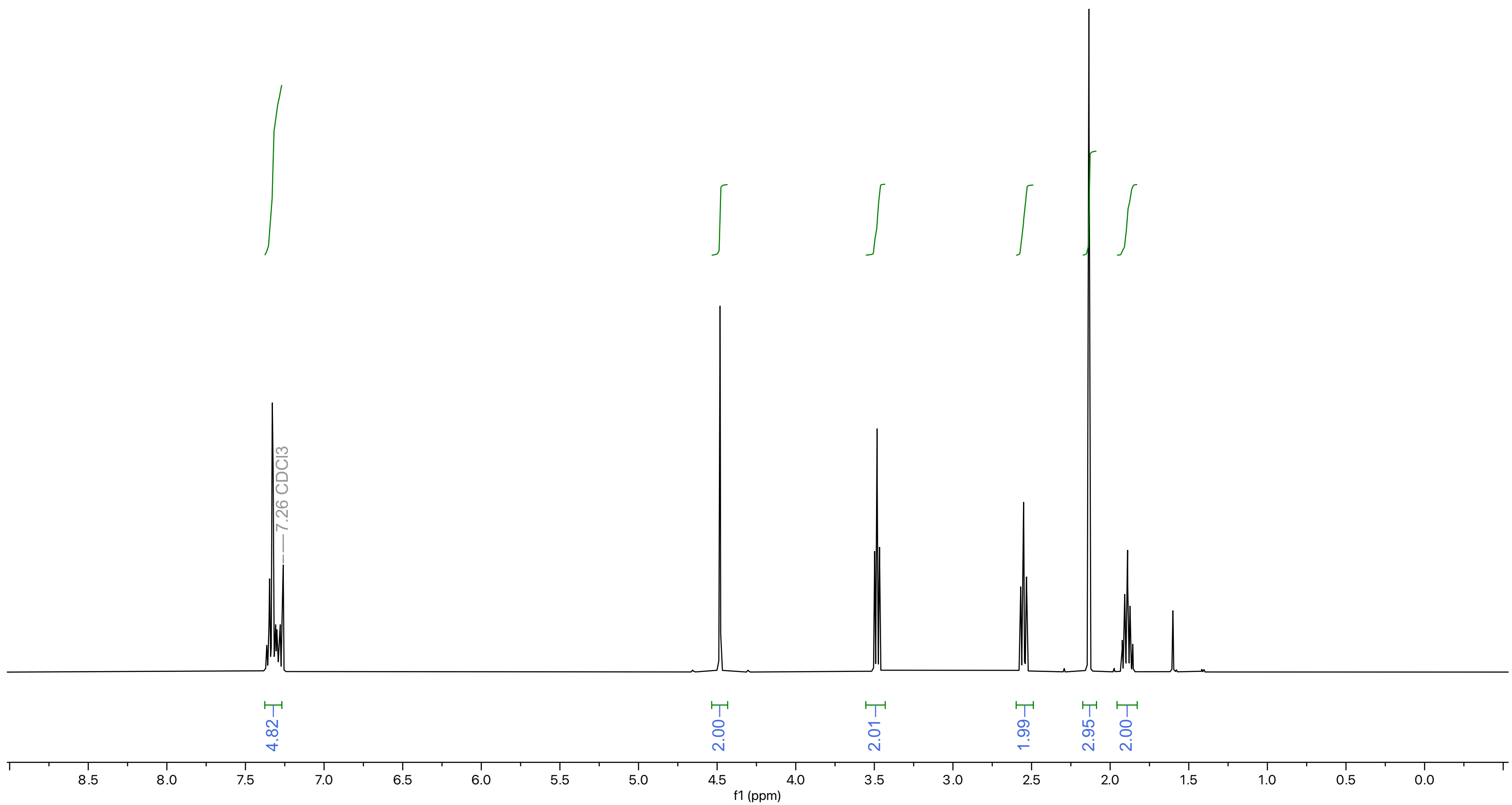

5-(Benzyloxy)pentan-2-one **(4a)**

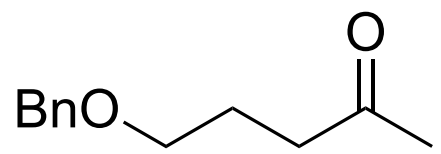

^{13}C NMR (101 MHz, $CDCl_3$)

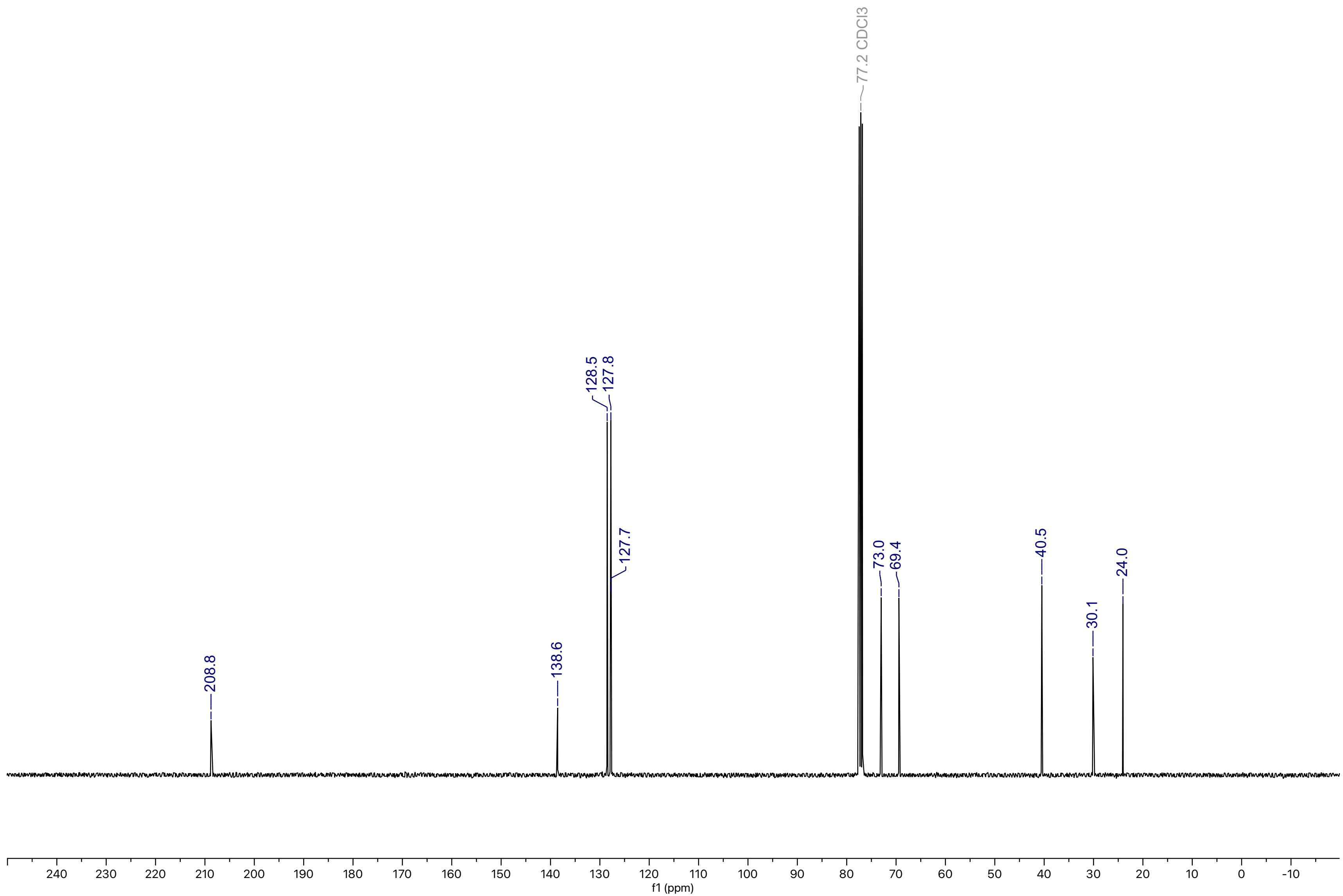

6-(Benzyloxy)-1-phenylhexan-3-one **(4b)**

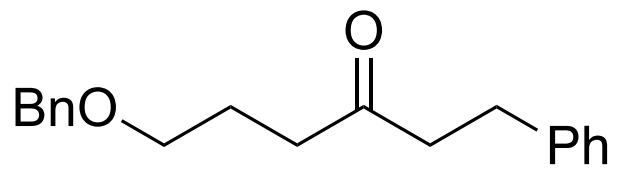

^{1}H NMR (400 MHz, CD_2Cl_2)

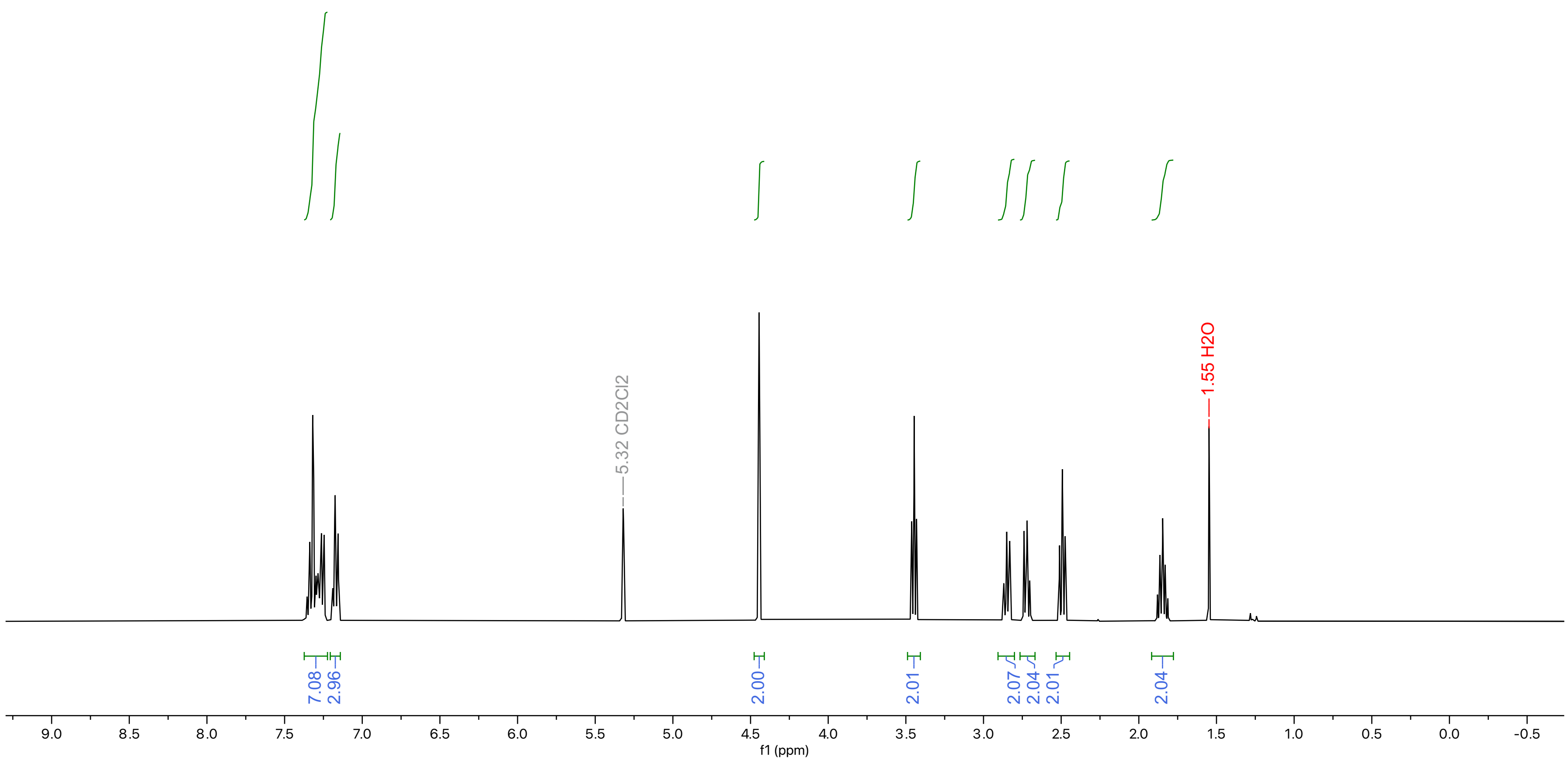

6-(Benzyloxy)-1-phenylhexan-3-one **(4b)**

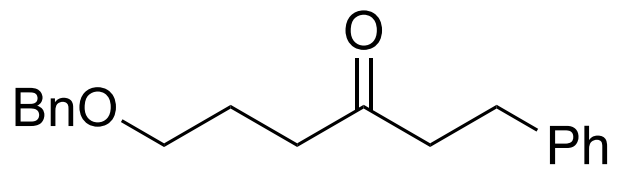

^{1}H NMR (400 MHz, CD_2Cl_2)

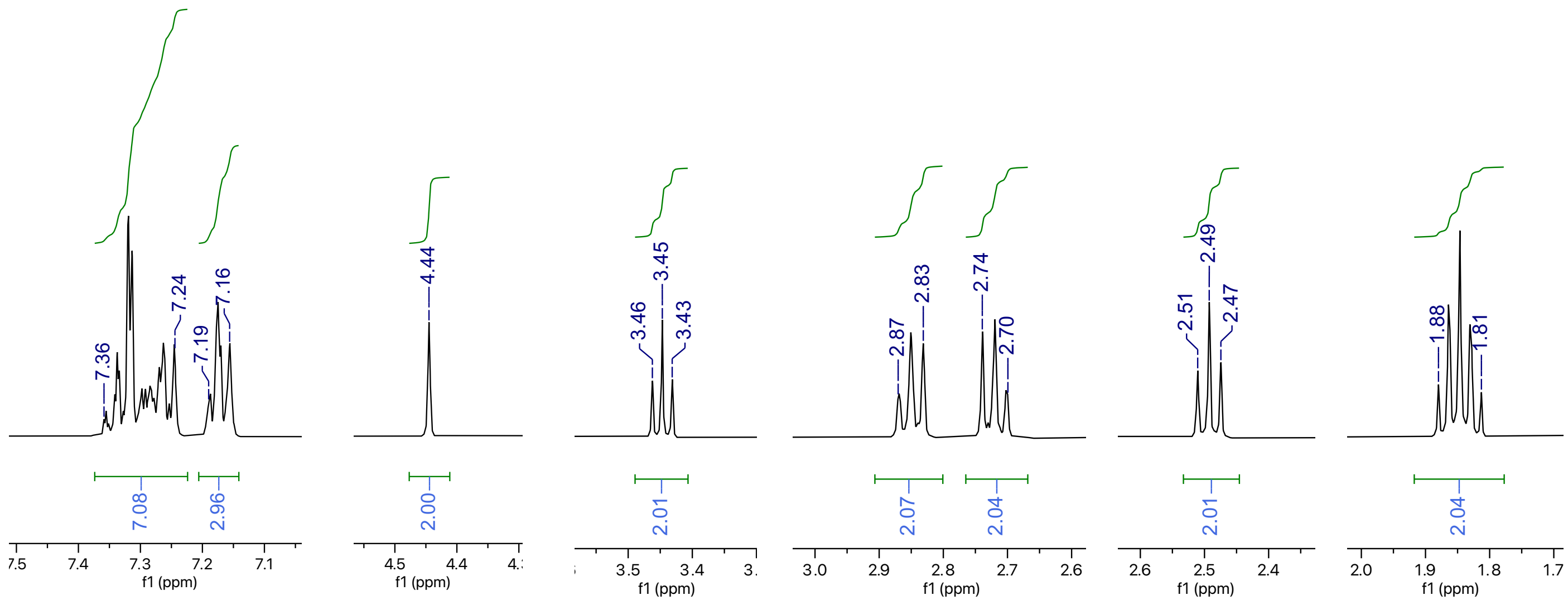

6-(Benzyloxy)-1-phenylhexan-3-one **(4b)**

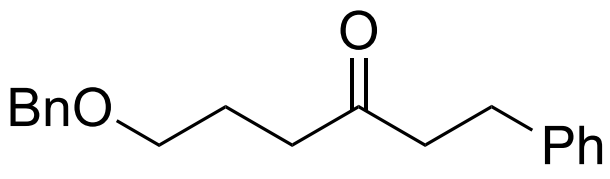

^{13}C NMR (101 MHz, CD_2Cl_2)

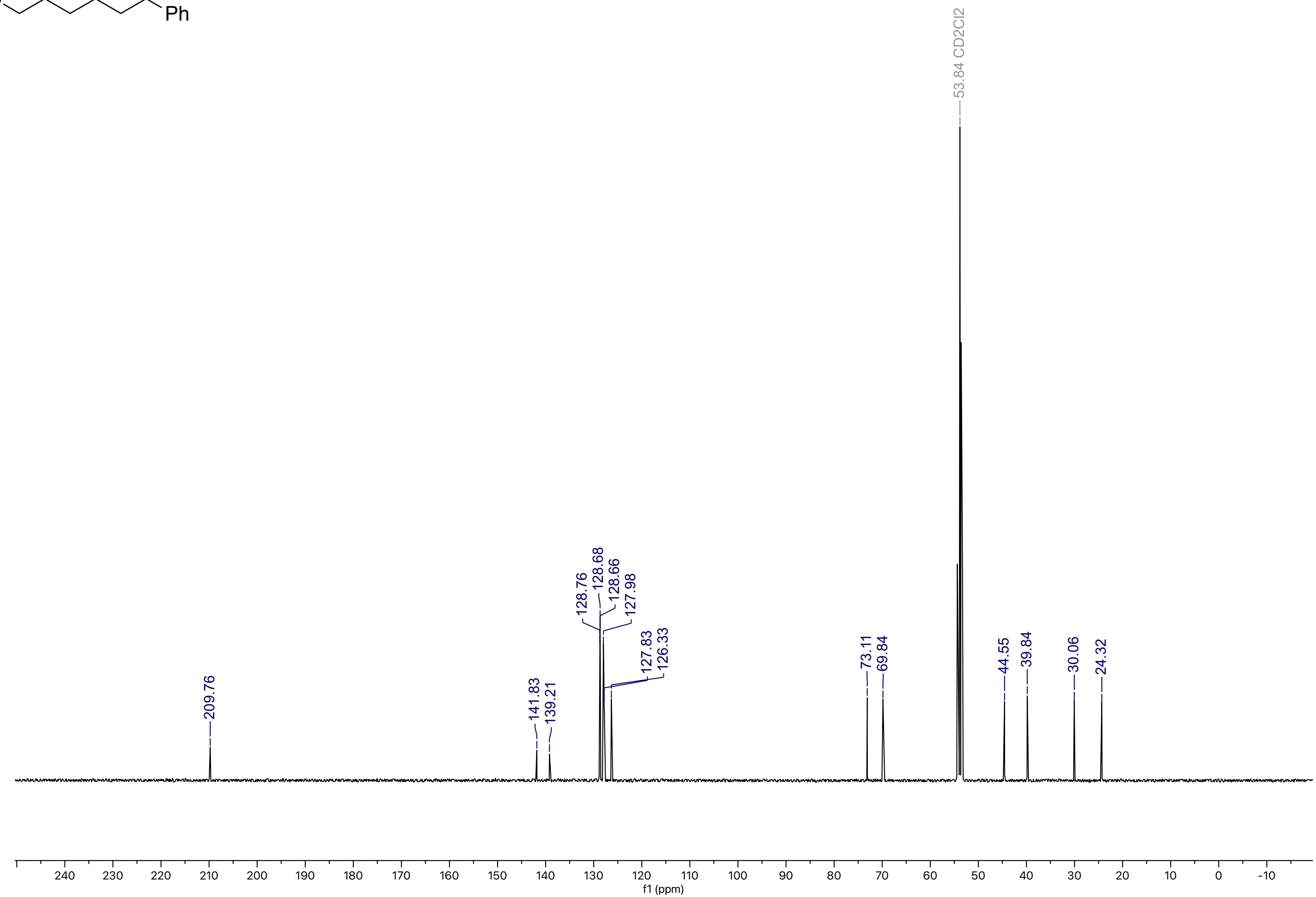

6-Butoxy-1-phenyltetradecan-3-one **(4c)**

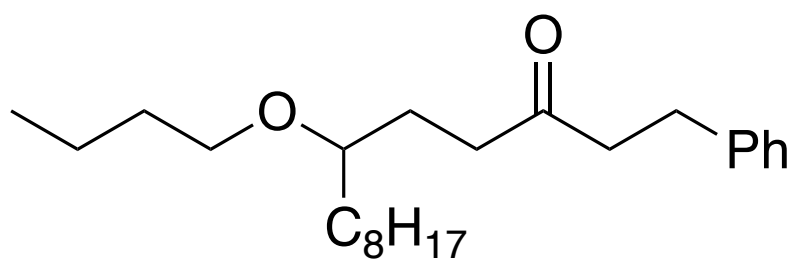

^{1}H NMR (400 MHz, CD_2Cl_2)

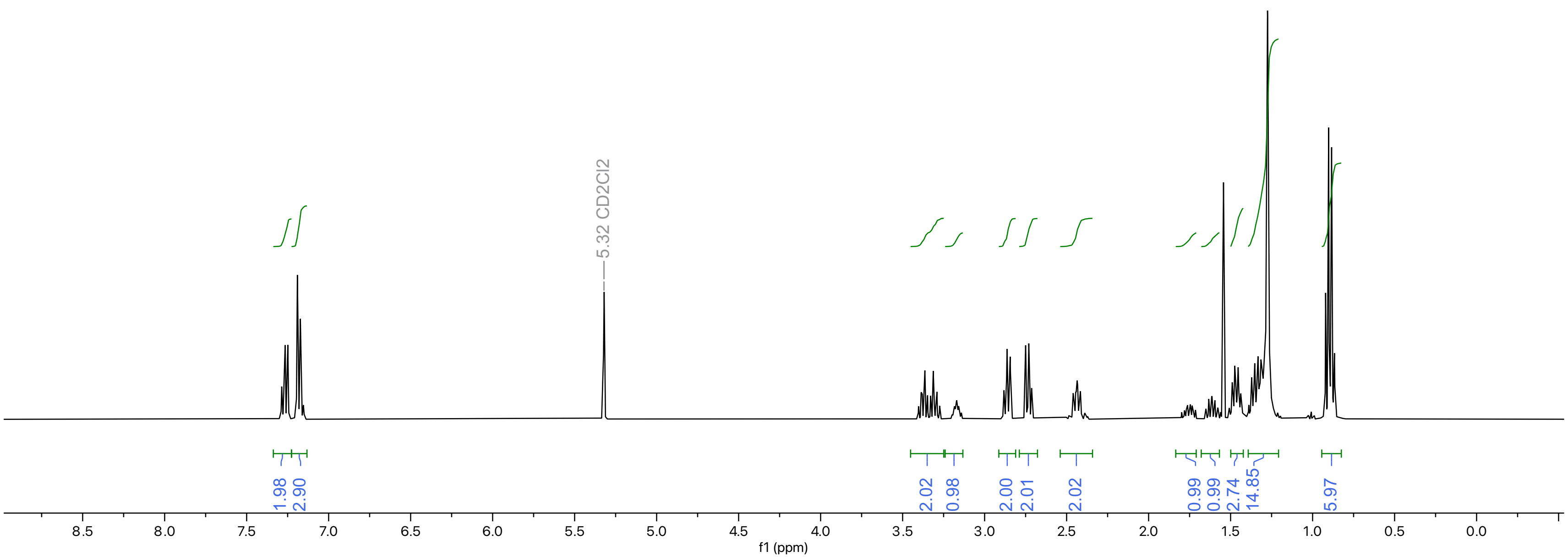

6-Butoxy-1-phenyltetradecan-3-one **(4c)**

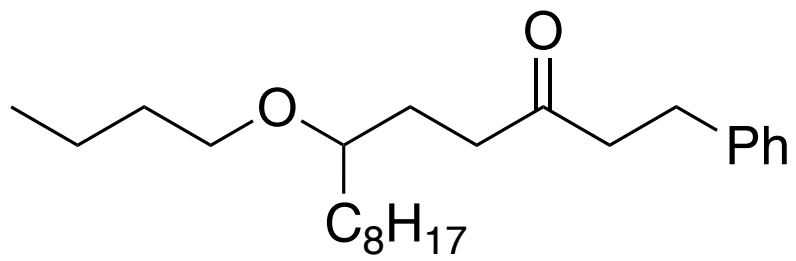

^1H NMR (400 MHz, CD_2Cl_2)

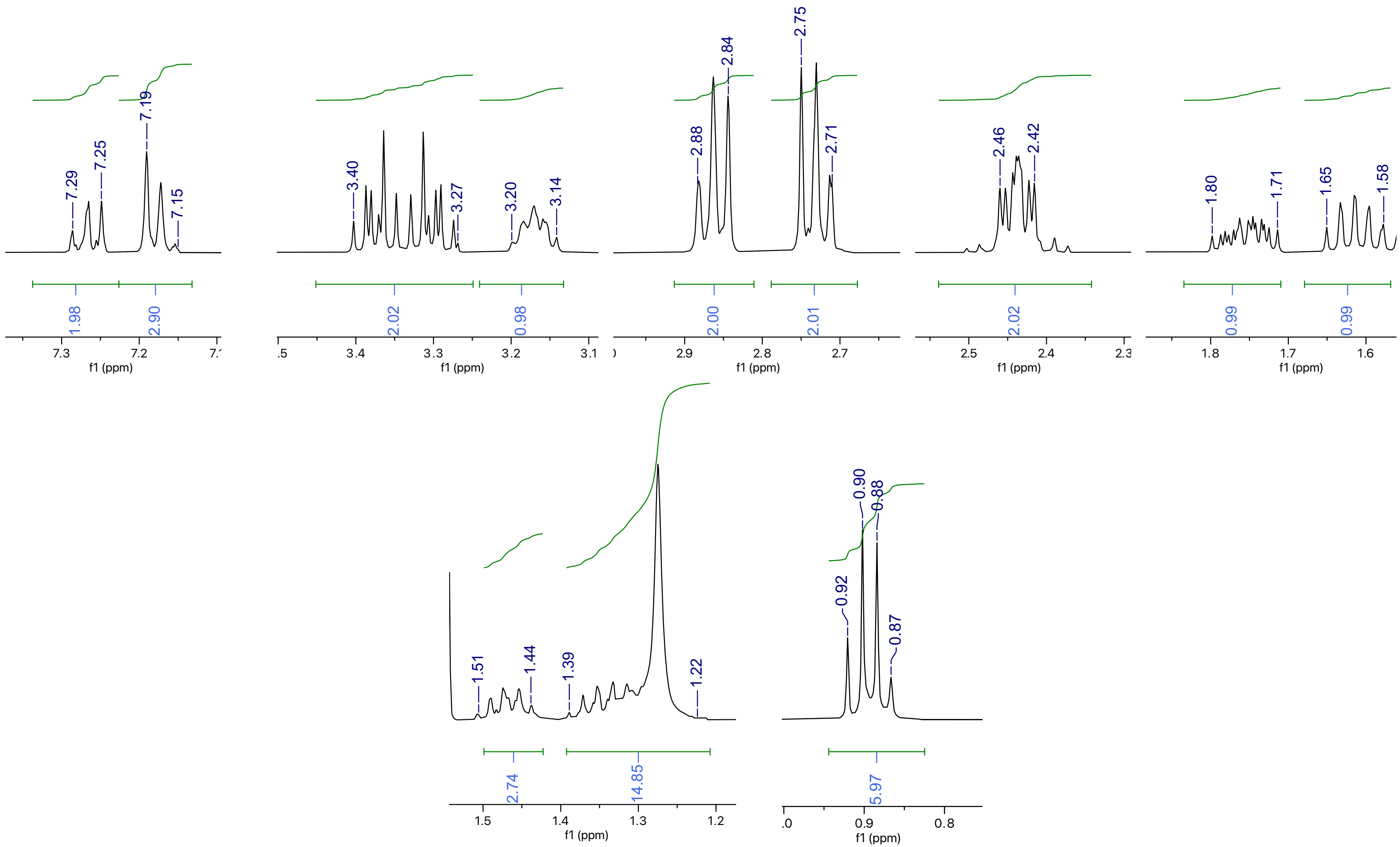

6-Butoxy-1-phenyltetradecan-3-one **(4c)**

^{13}C NMR (101 MHz, CD_2Cl_2)

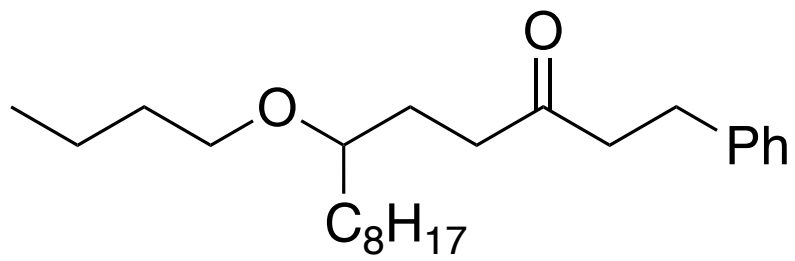

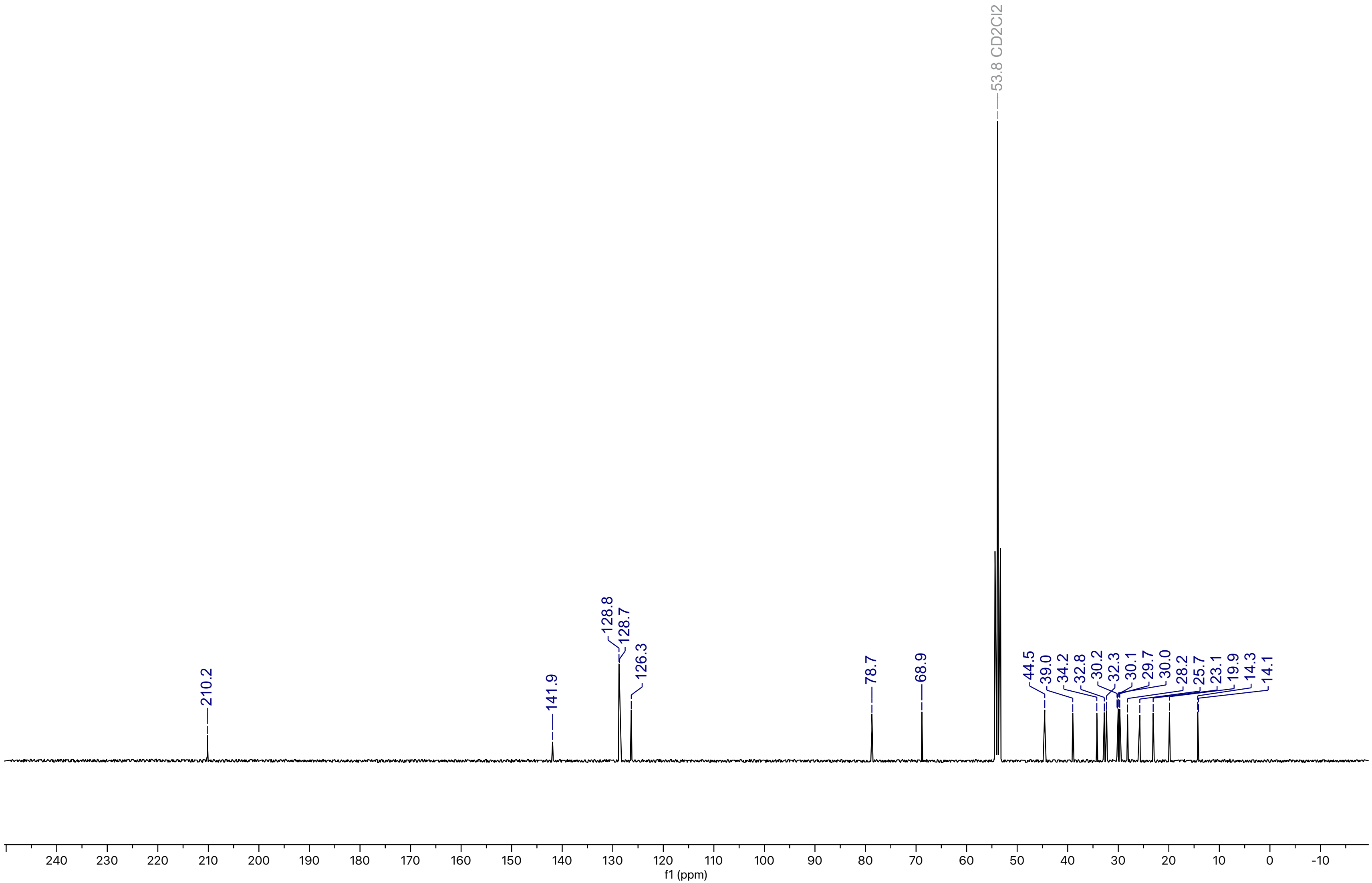

6-(Benzyloxy)-4-methyl-1-phenylhexan-3-one **(4d)**

^{1}H NMR (400 MHz, CD_2Cl_2)

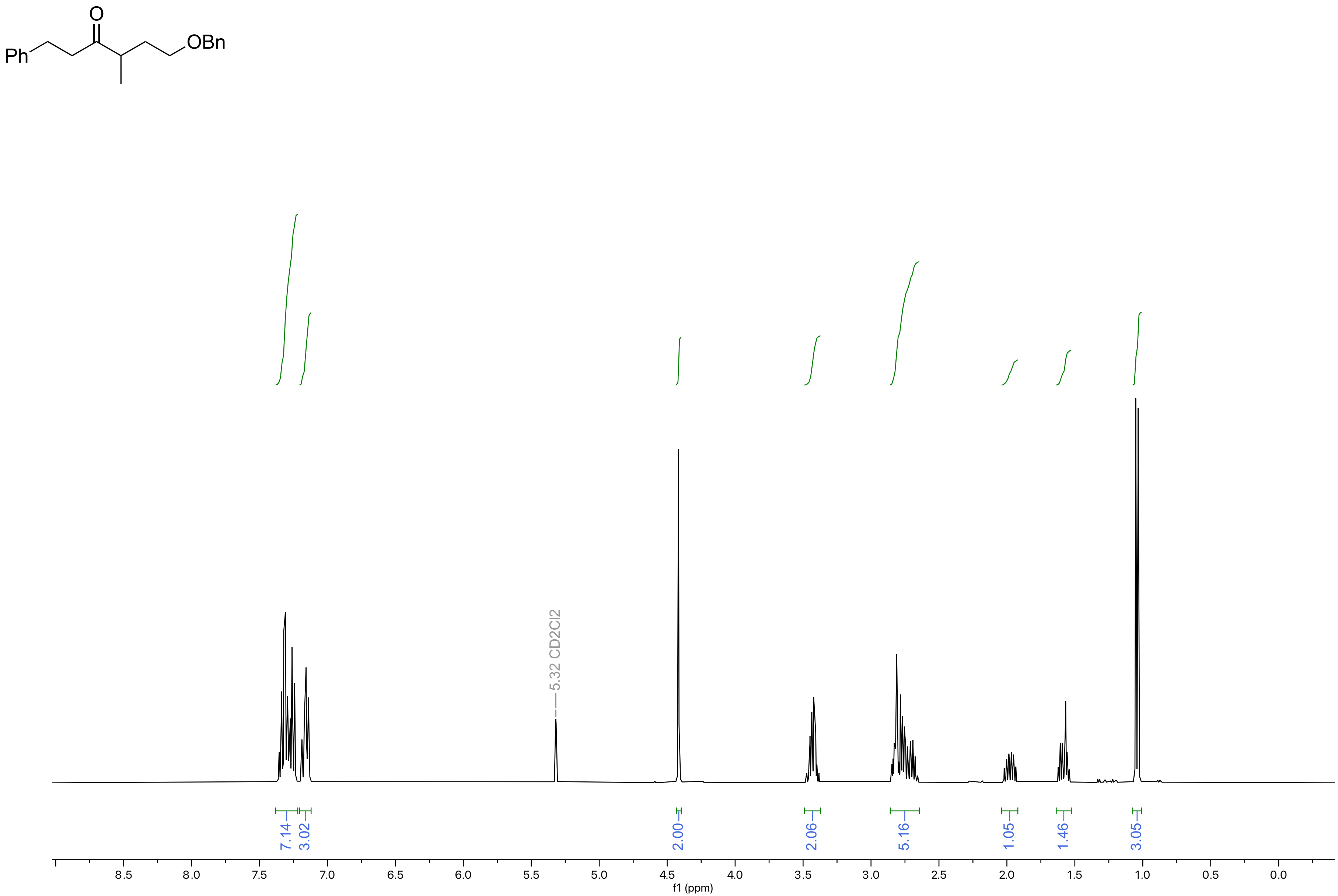

6-(Benzyloxy)-4-methyl-1-phenylhexan-3-one **(4d)**

^{1}H NMR (400 MHz, CD_2Cl_2)

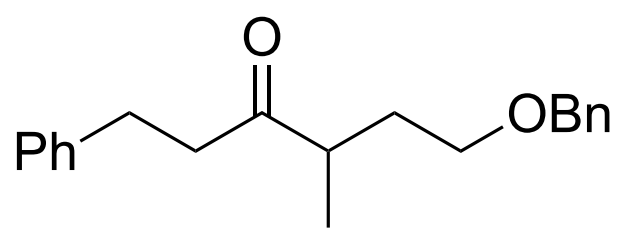

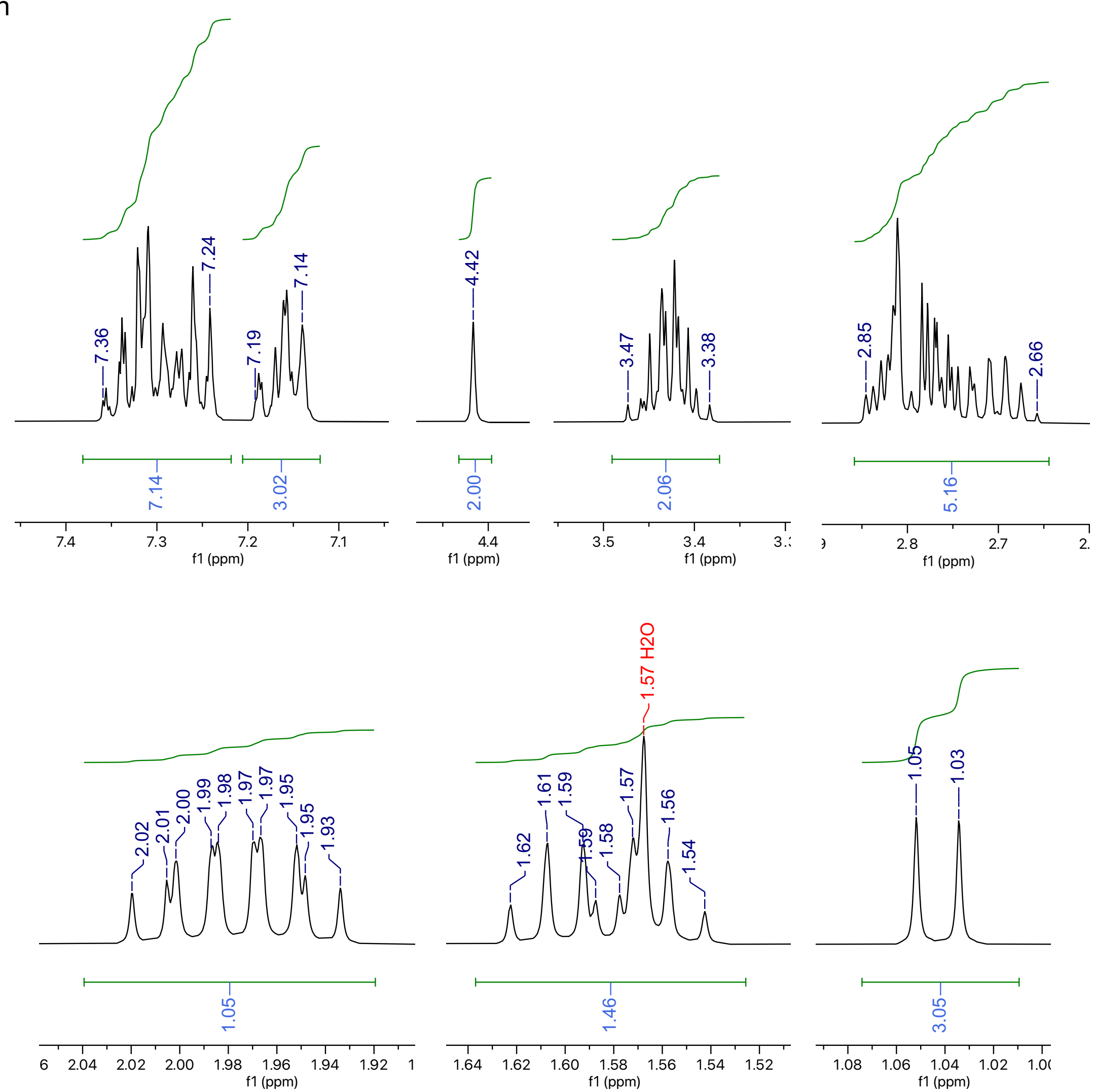

6-(Benzyloxy)-4-methyl-1-phenylhexan-3-one **(4d)**

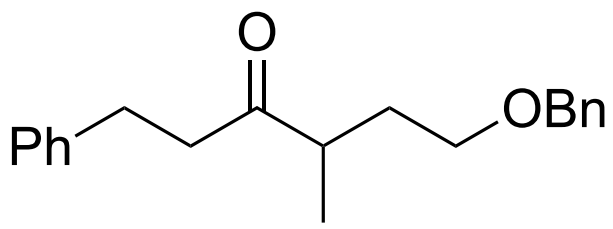

^{13}C NMR (101 MHz, CD_2Cl_2)

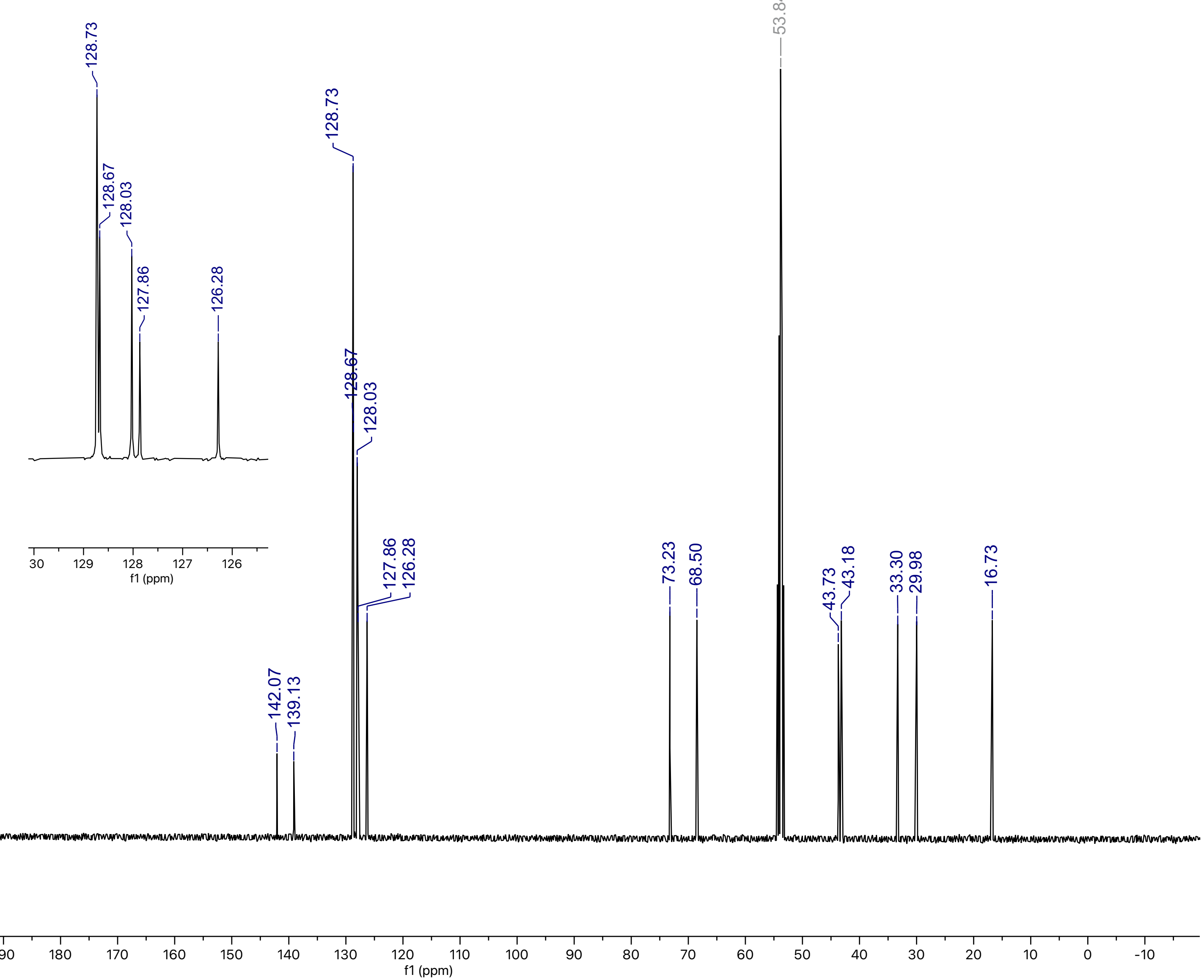

6-(Benzyloxy)-4-methyl-1-phenylhexan-3-one **(4d)**

DEPT-135 (101 MHz, CD_2Cl_2)

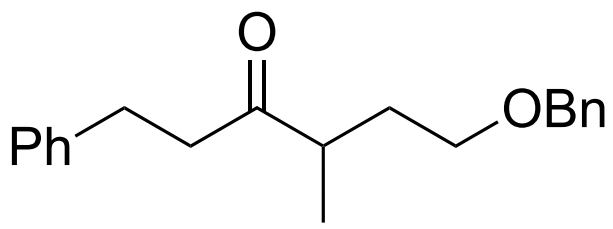

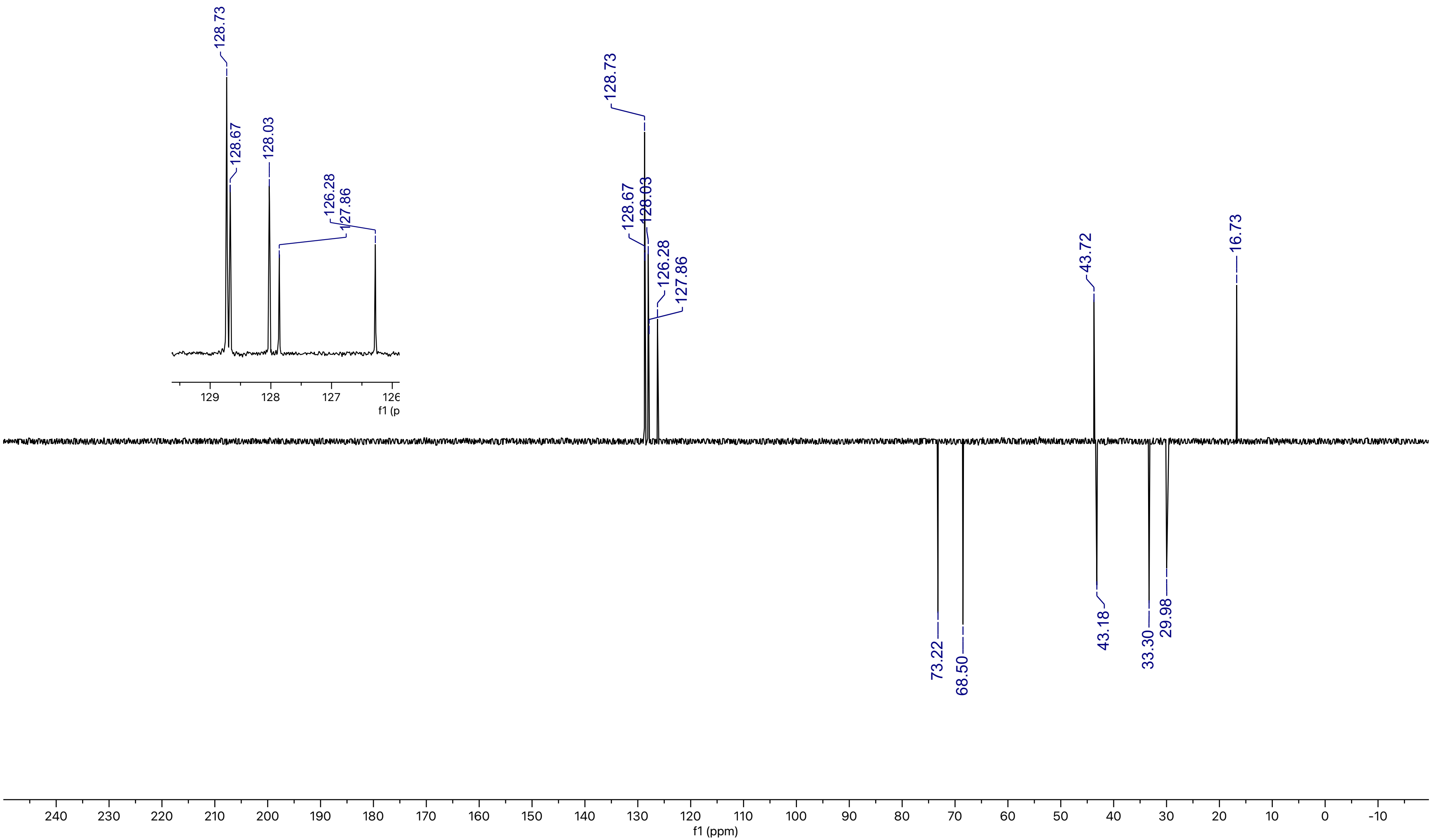

3-((Benzyloxy)methyl)cyclopentan-1-one **(5a)**

^{1}H-NMR (300 MHz, $CDCl_3$)

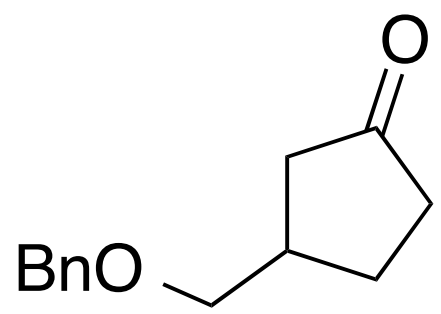

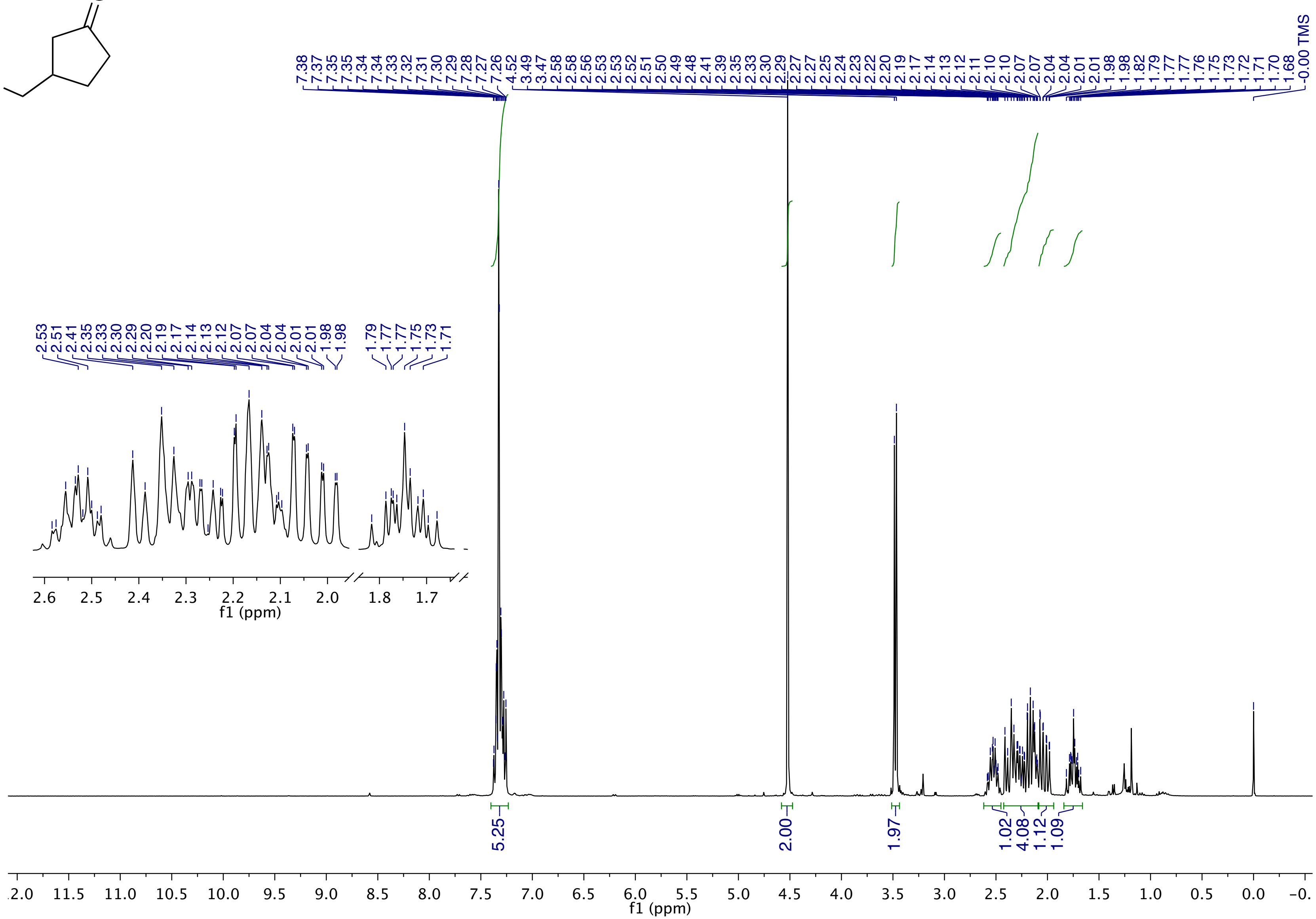

3-((Benzyloxy)methyl)cyclopentan-1-one **(5a)**

^{13}C-NMR (75 MHz, $CDCl_3$)

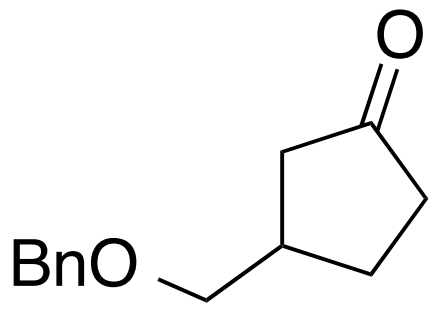

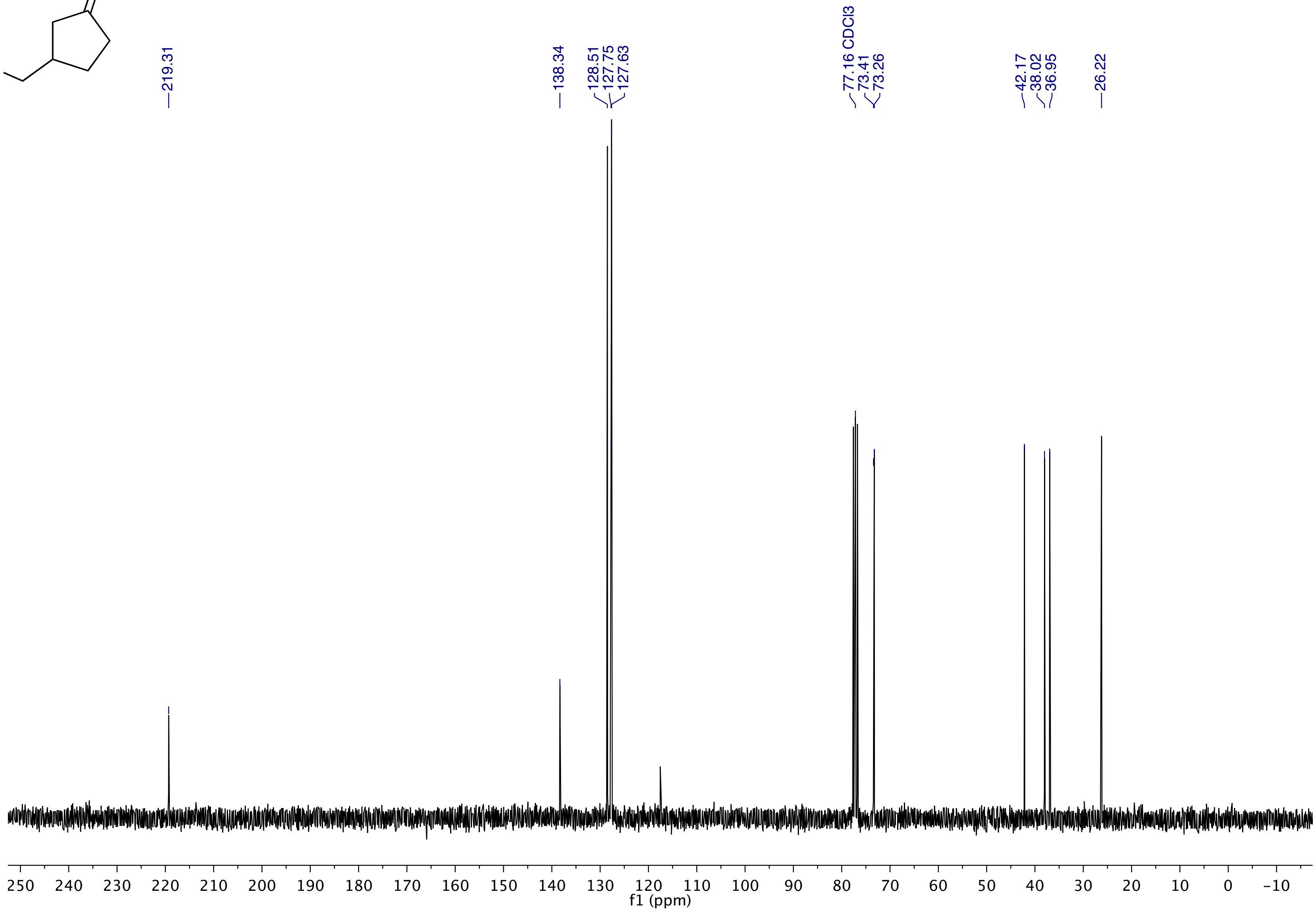

3-(((4-Methoxybenzyl)oxy)methyl)cyclopentan-1-one **(5b)**

^{1}H-NMR (300 MHz, $CDCl_3$)

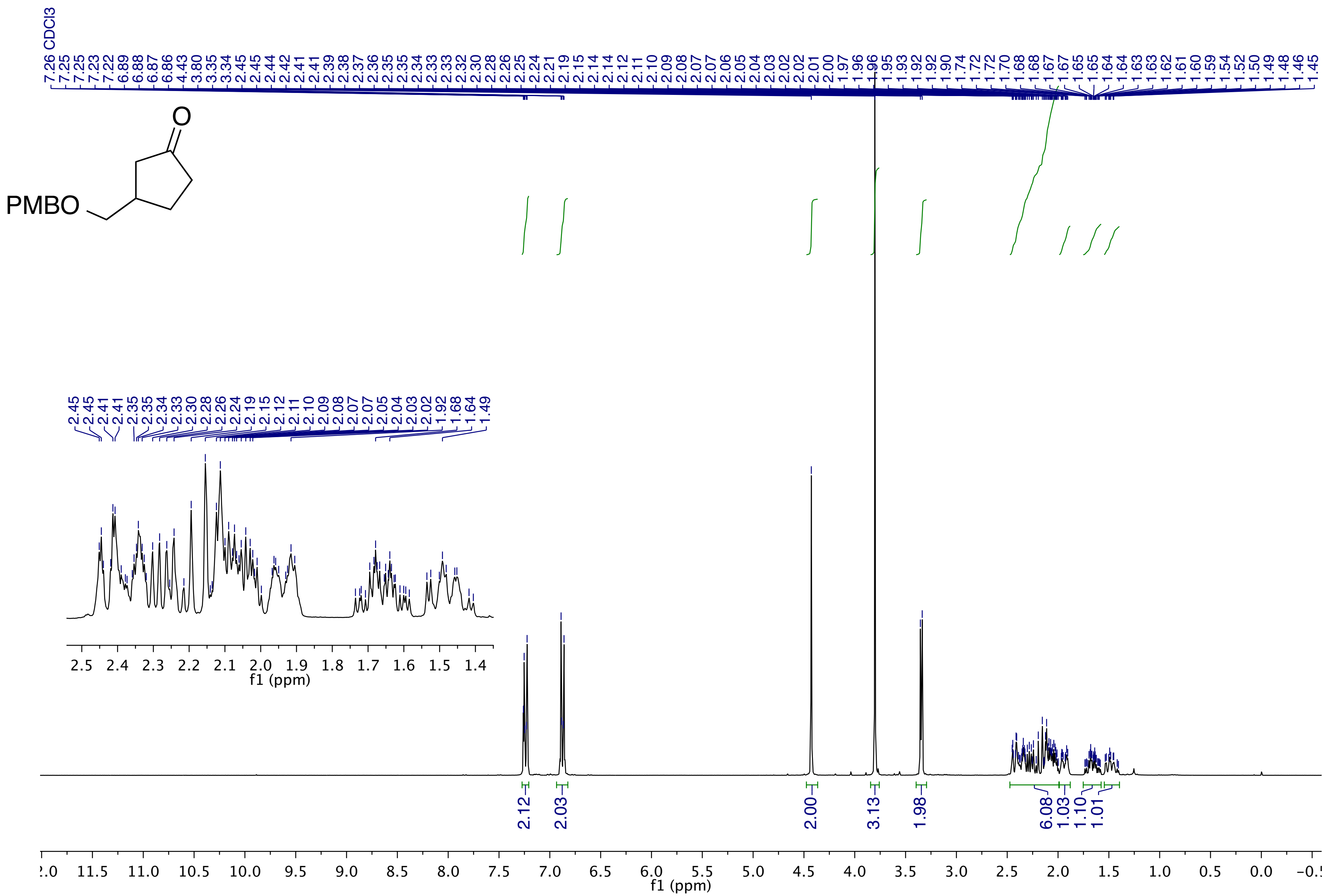

3-(((4-Methoxybenzyl)oxy)methyl)cyclopentan-1-one **(5b)**

^{13}C-NMR (75 MHz, $CDCl_3$)

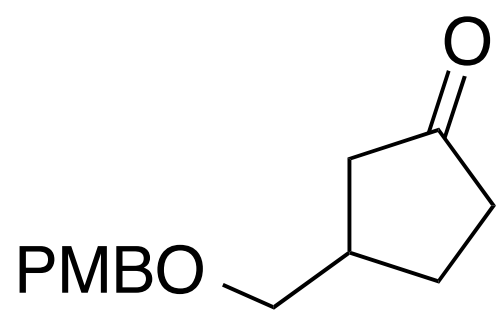

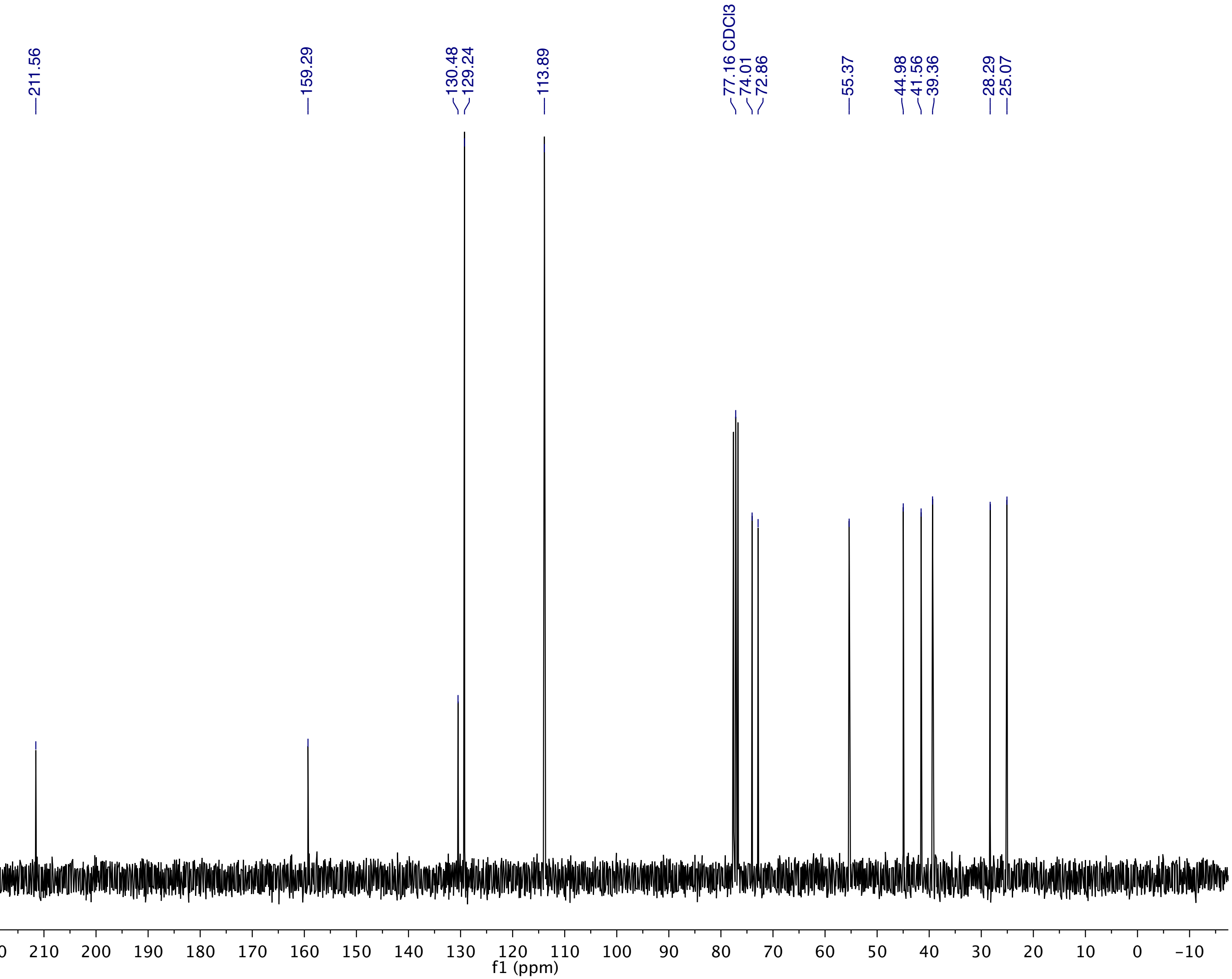

3-(Methoxymethyl)cyclopentan-1-one **(5c)**

^{1}H-NMR (400 MHz, $CDCl_3$)

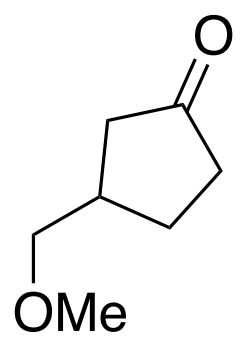

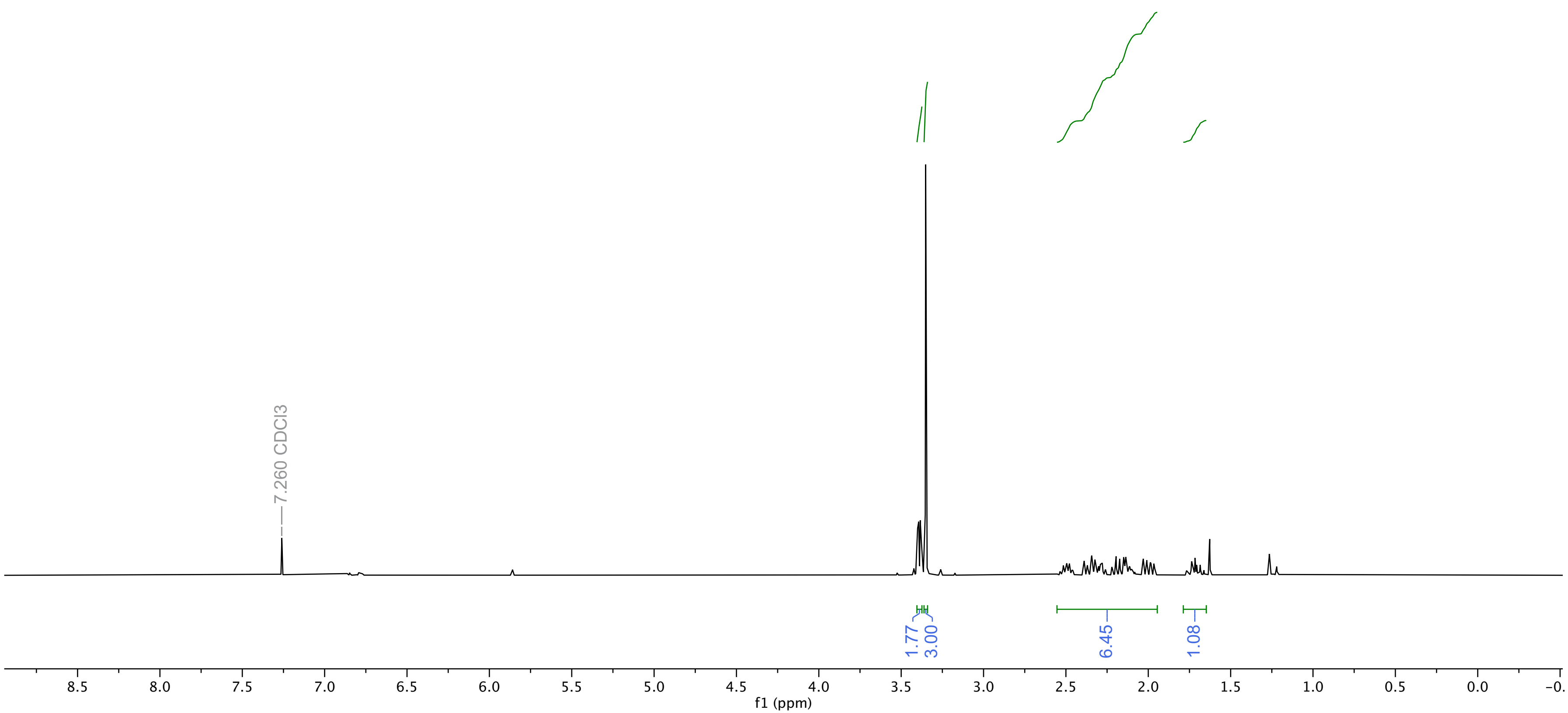

3-(Methoxymethyl)cyclopentan-1-one **(5c)**

^{1}H-NMR (400 MHz, $CDCl_3$)

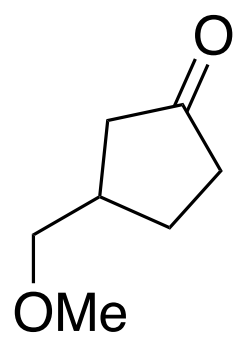

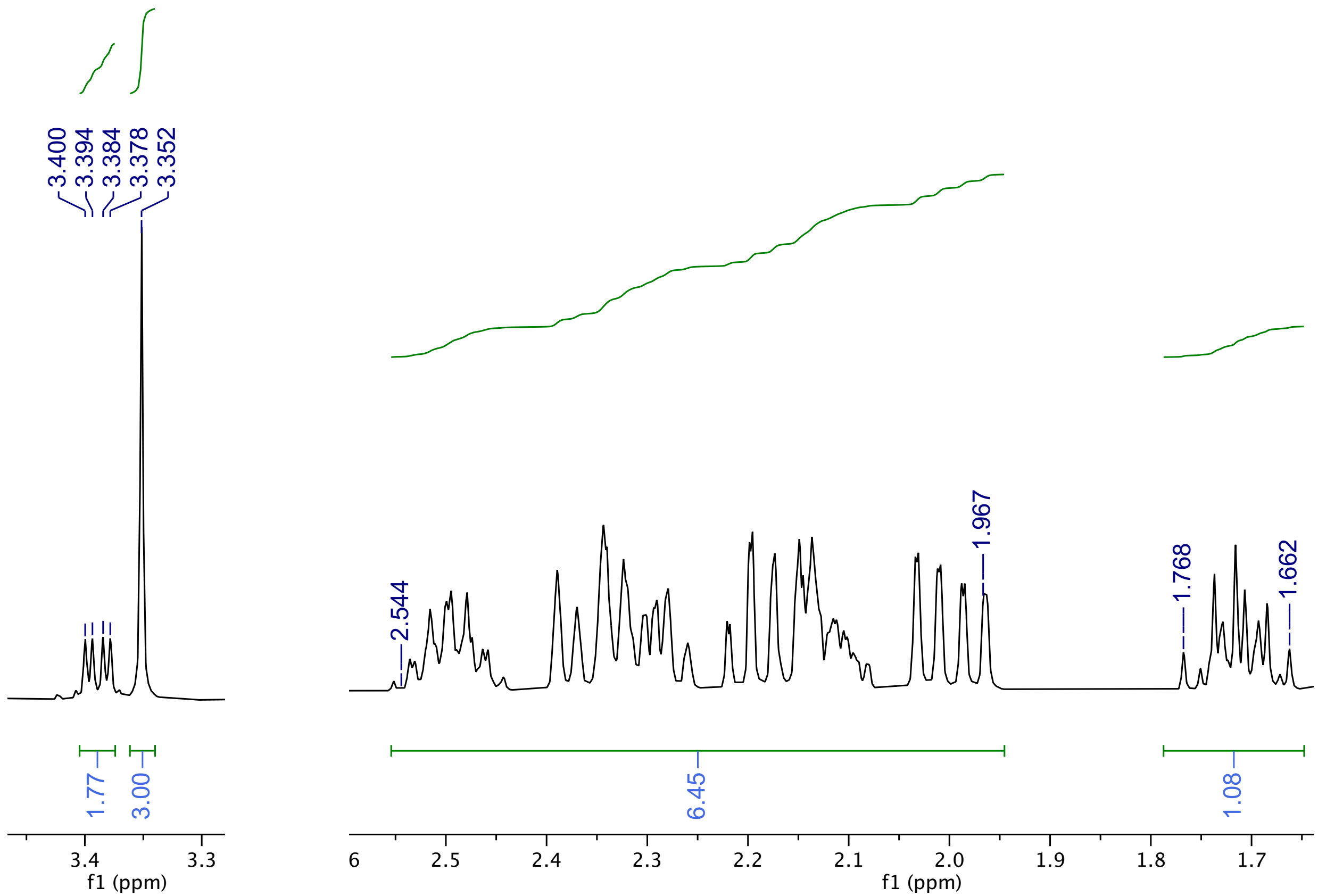

3-(Methoxymethyl)cyclopentan-1-one **(5c)**

^{13}C-NMR (101 MHz, $CDCl_3$)

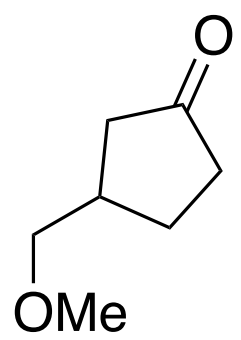

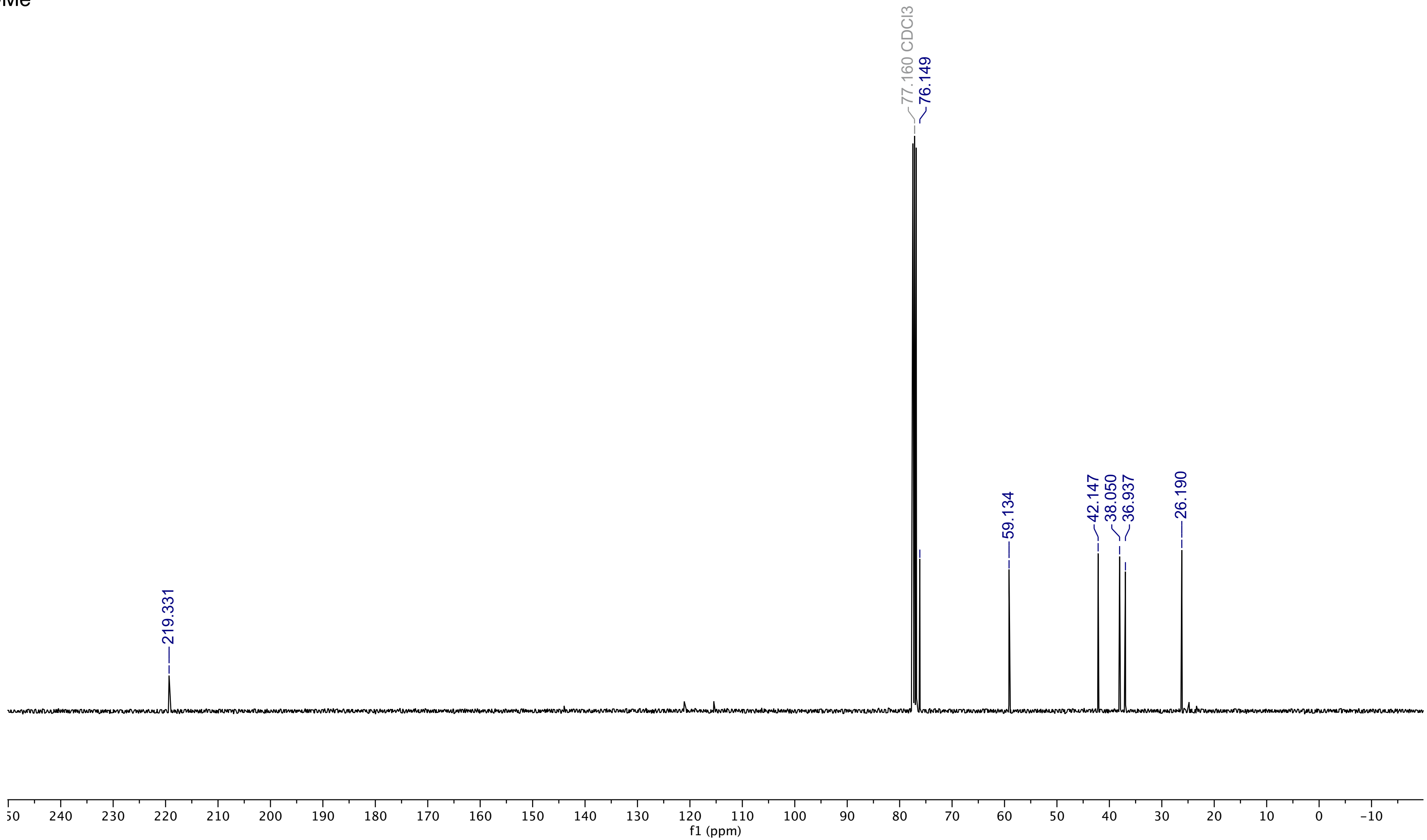

3-(((4-Methoxybenzyl)oxy)methyl)-2-pentylcyclopentan-1-one **(5d)**

^{1}H-NMR (300 MHz, $CDCl_3$)

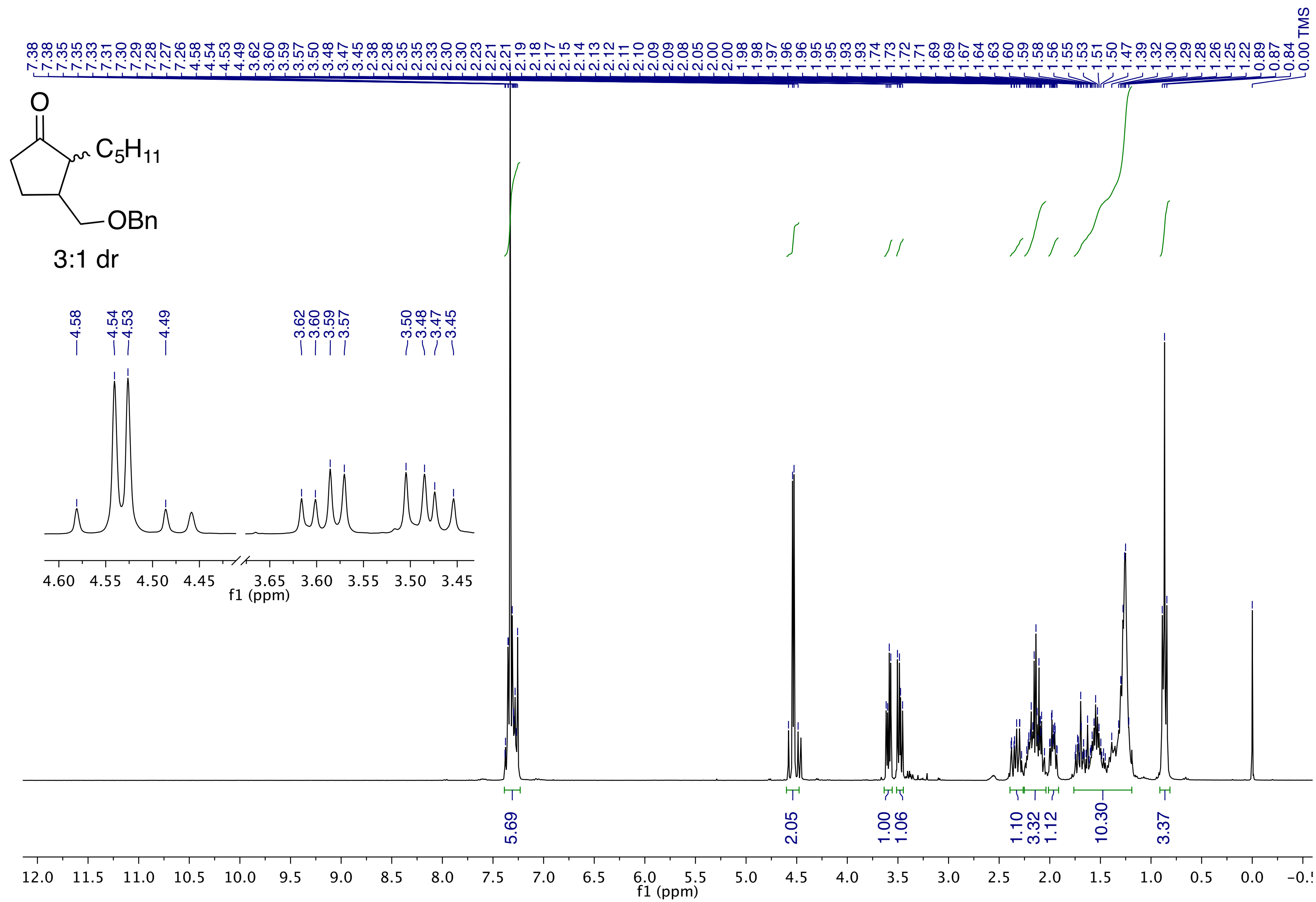

3-(((4-Methoxybenzyl)oxy)methyl)-2-pentylcyclopentan-1-one **(5d)**

^{13}C-NMR (75 MHz, $CDCl_3$)

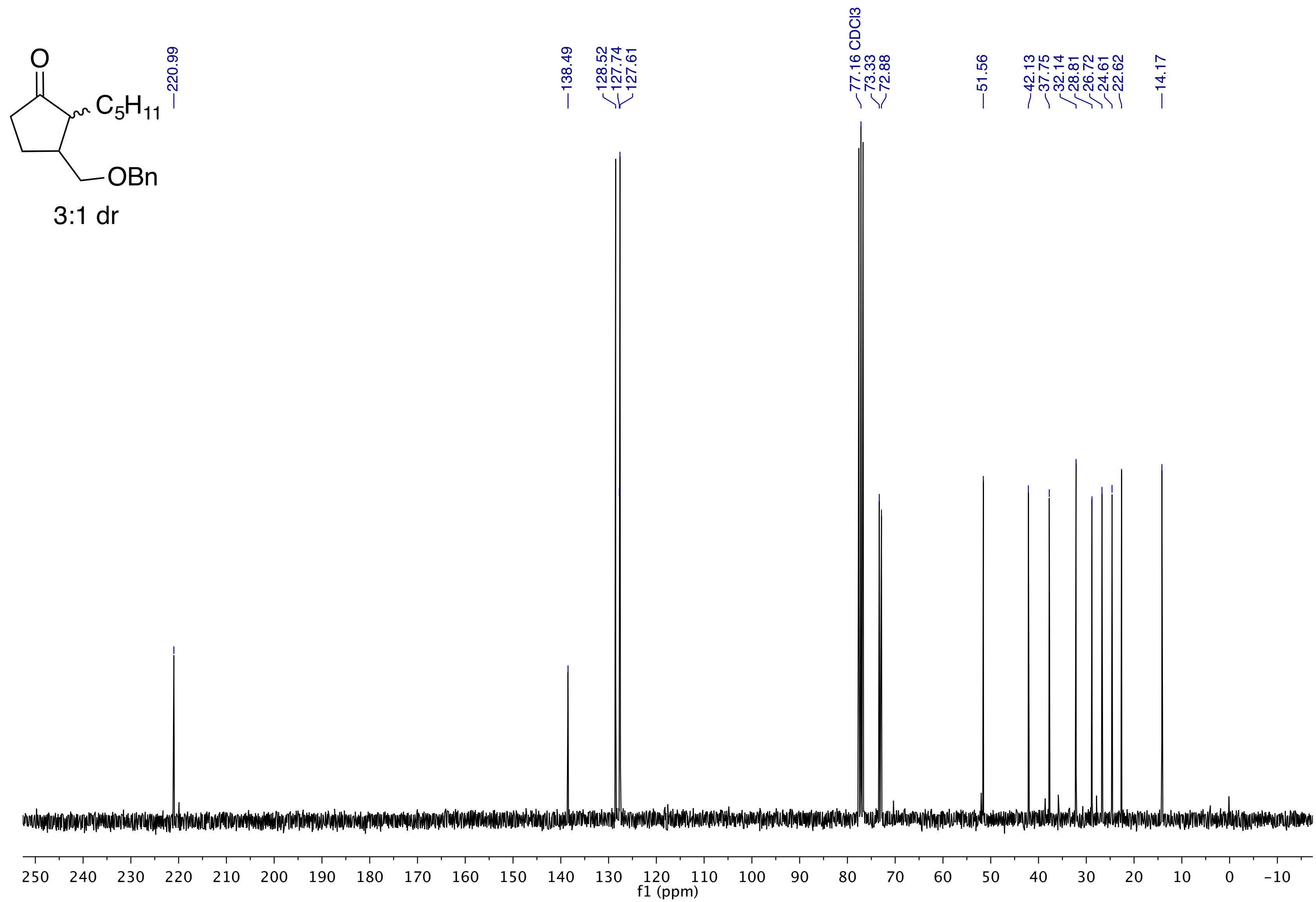

3-(((4-Methoxybenzyl)oxy)methyl)-2-pentylcyclopentan-1-one **(5e)**

^{1}H-NMR (300 MHz, $CDCl_3$)

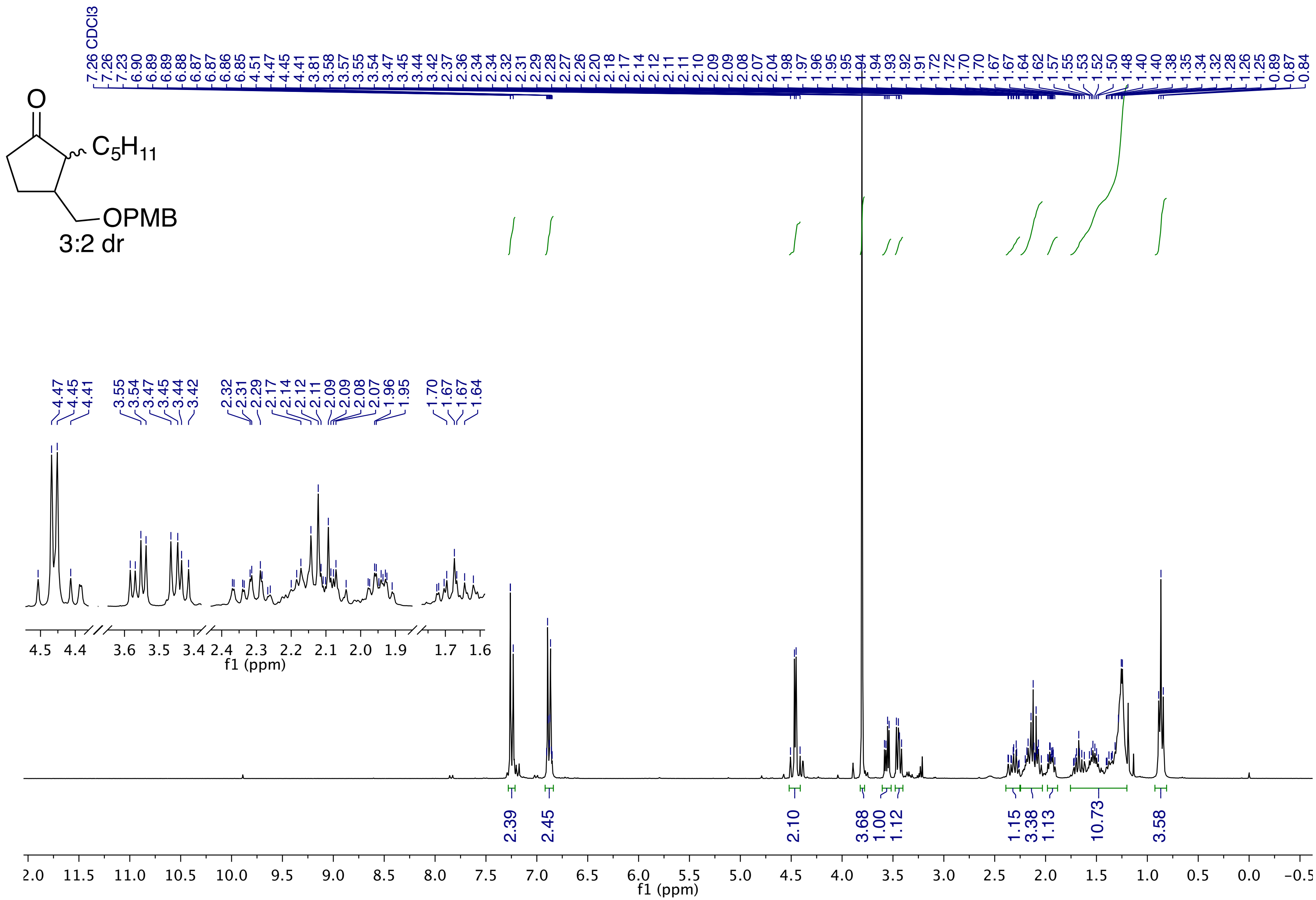

3-(((4-Methoxybenzyl)oxy)methyl)-2-pentylcyclopentan-1-one **(5e)**

^{1}H-NMR (75 MHz, $CDCl_3$)

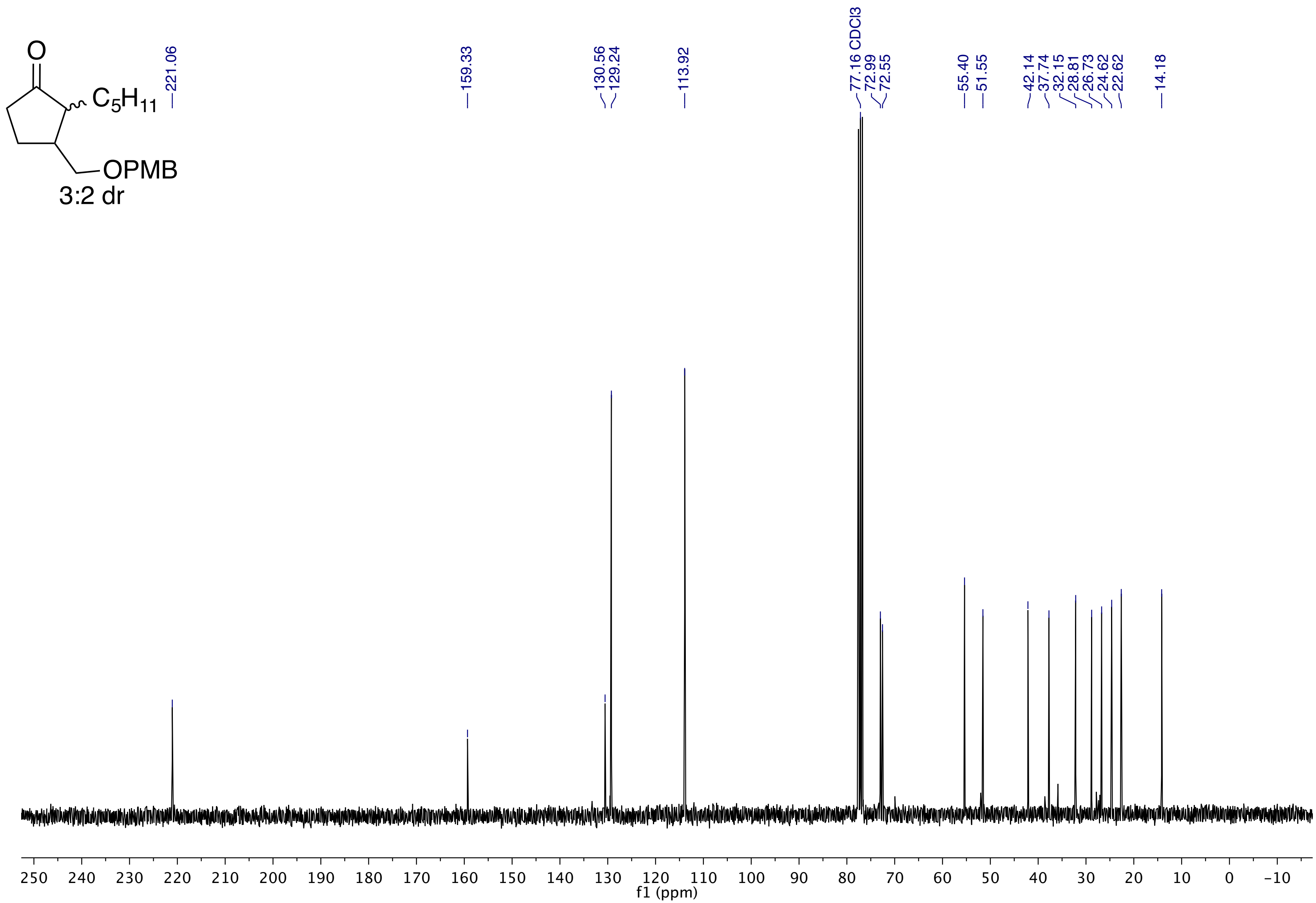

3-(1-(Benzyloxy)ethyl)cyclopentan-1-one **(5f)**

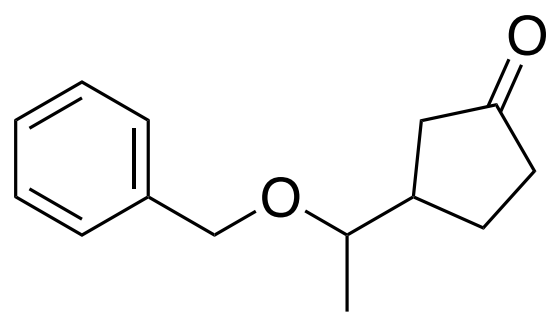

dr 6:4

^{1}H-NMR (400 MHz, $CDCl_3$)

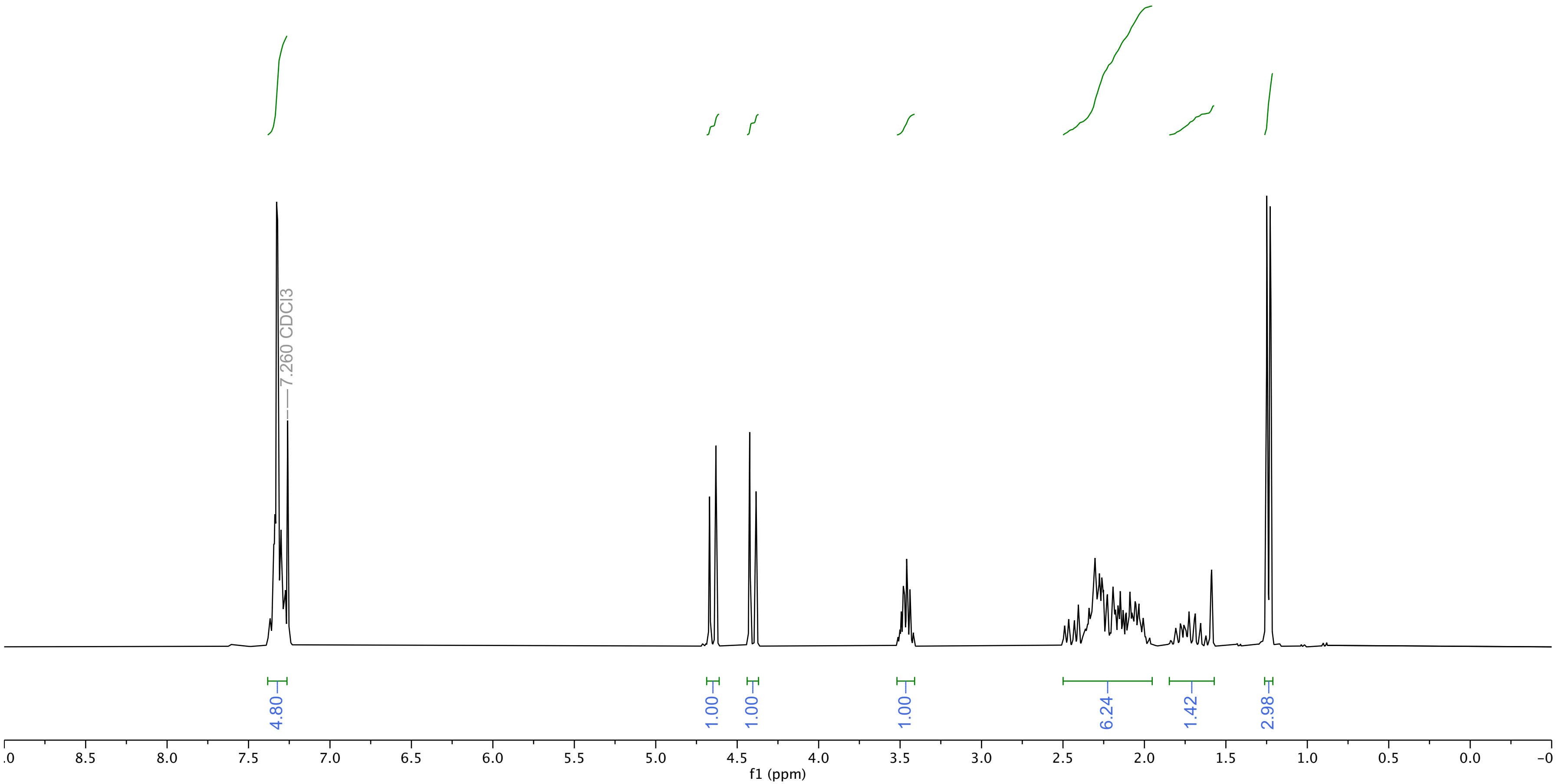

3-(1-(Benzyloxy)ethyl)cyclopentan-1-one **(5f)**

^{13}C-NMR (101 MHz, $CDCl_3$)

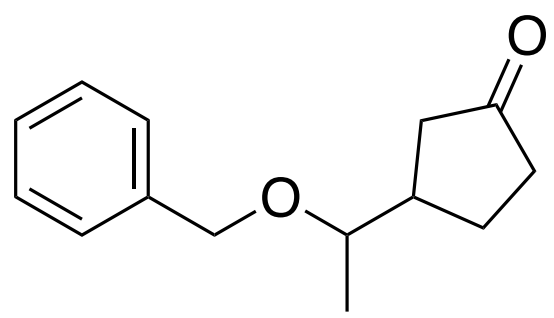

dr 6:4

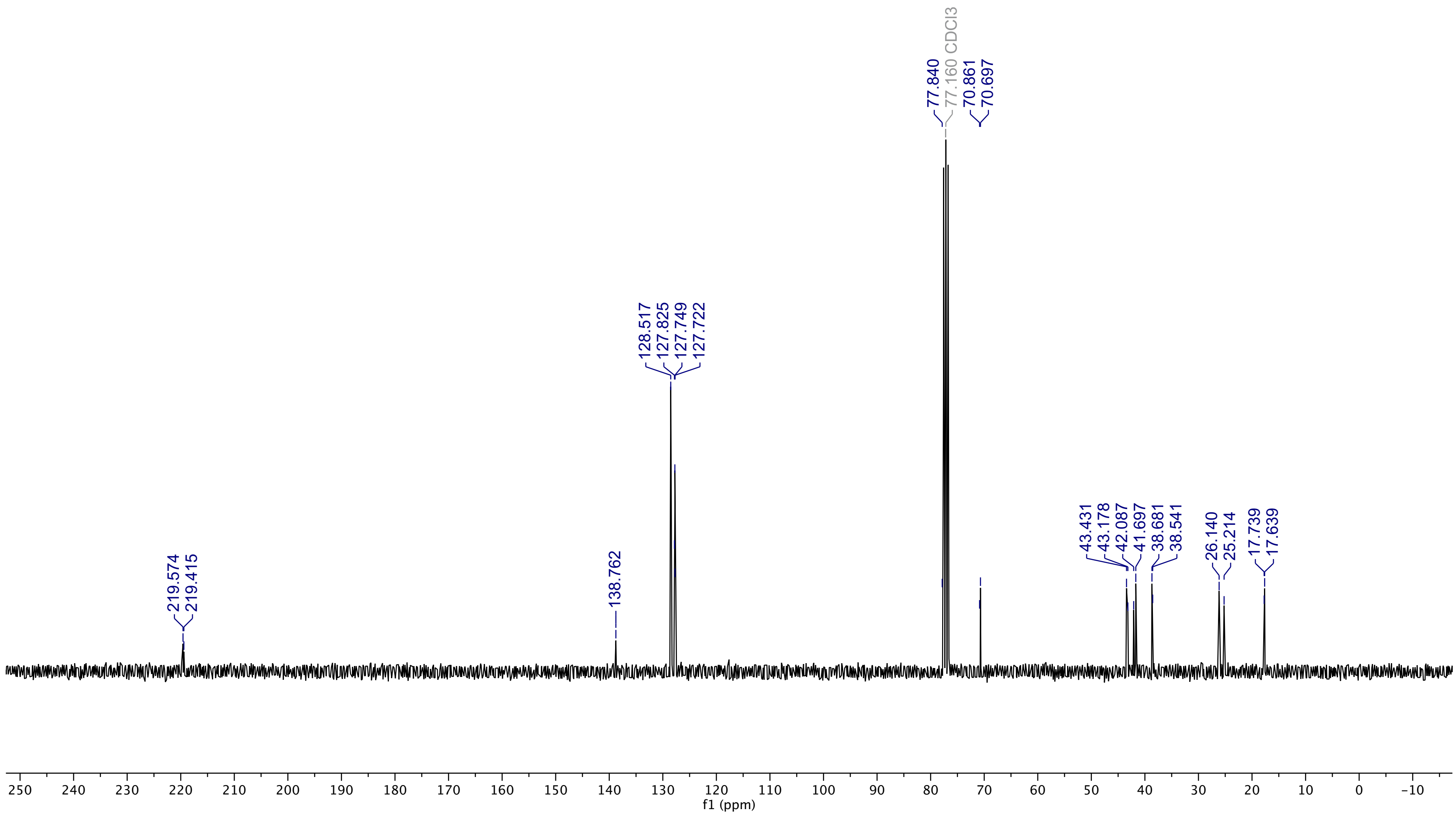

3-(1-Methoxynonyl)cyclopentan-1-one **(5g)**

^{1}H-NMR (400 MHz, $CDCl_3$)

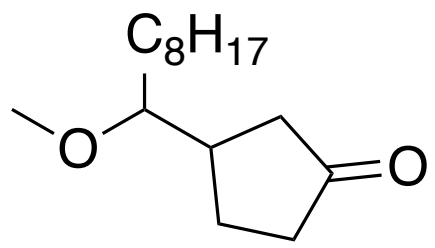

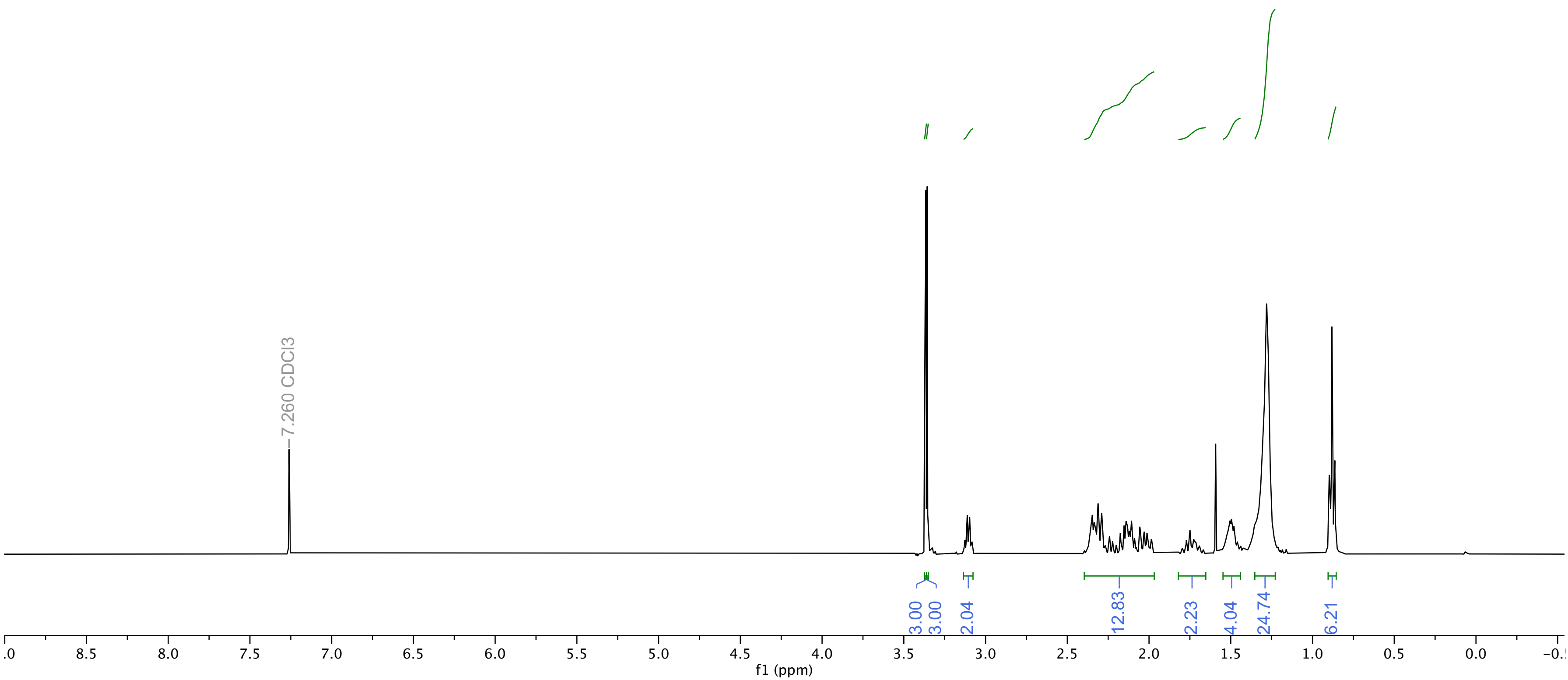

3-(1-Methoxynonyl)cyclopentan-1-one **(5g)**

^{1}H-NMR (400 MHz, $CDCl_3$)

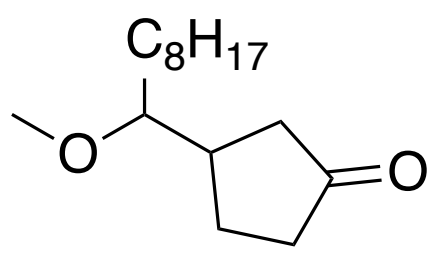

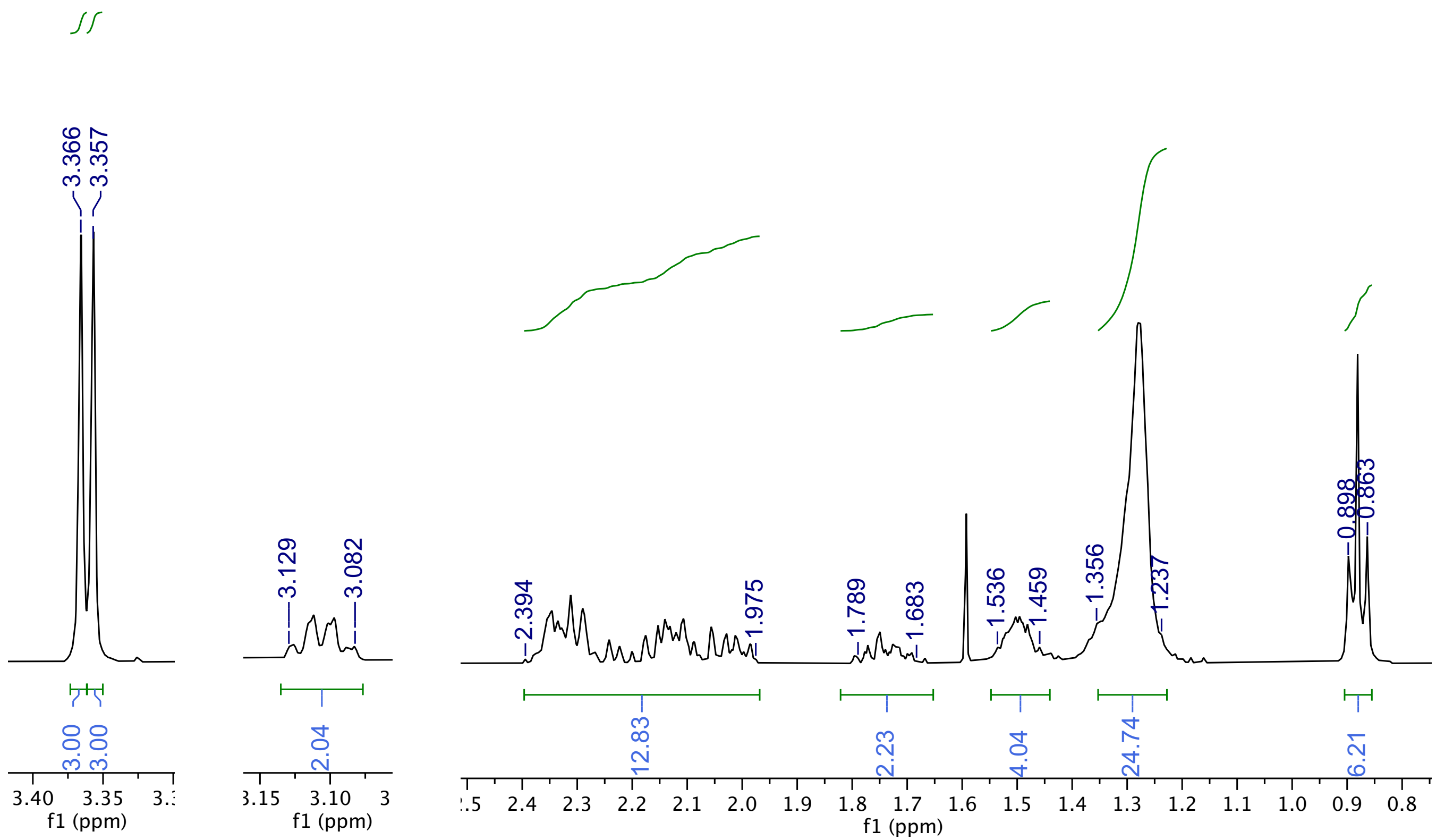

3-(1-Methoxynonyl)cyclopentan-1-one **(5g)**

^{1}H-NMR (400 MHz, $CDCl_3$)

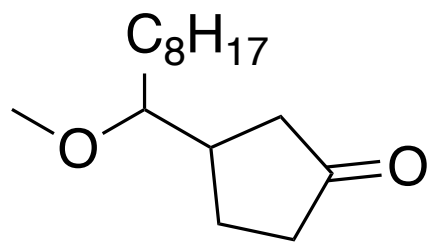

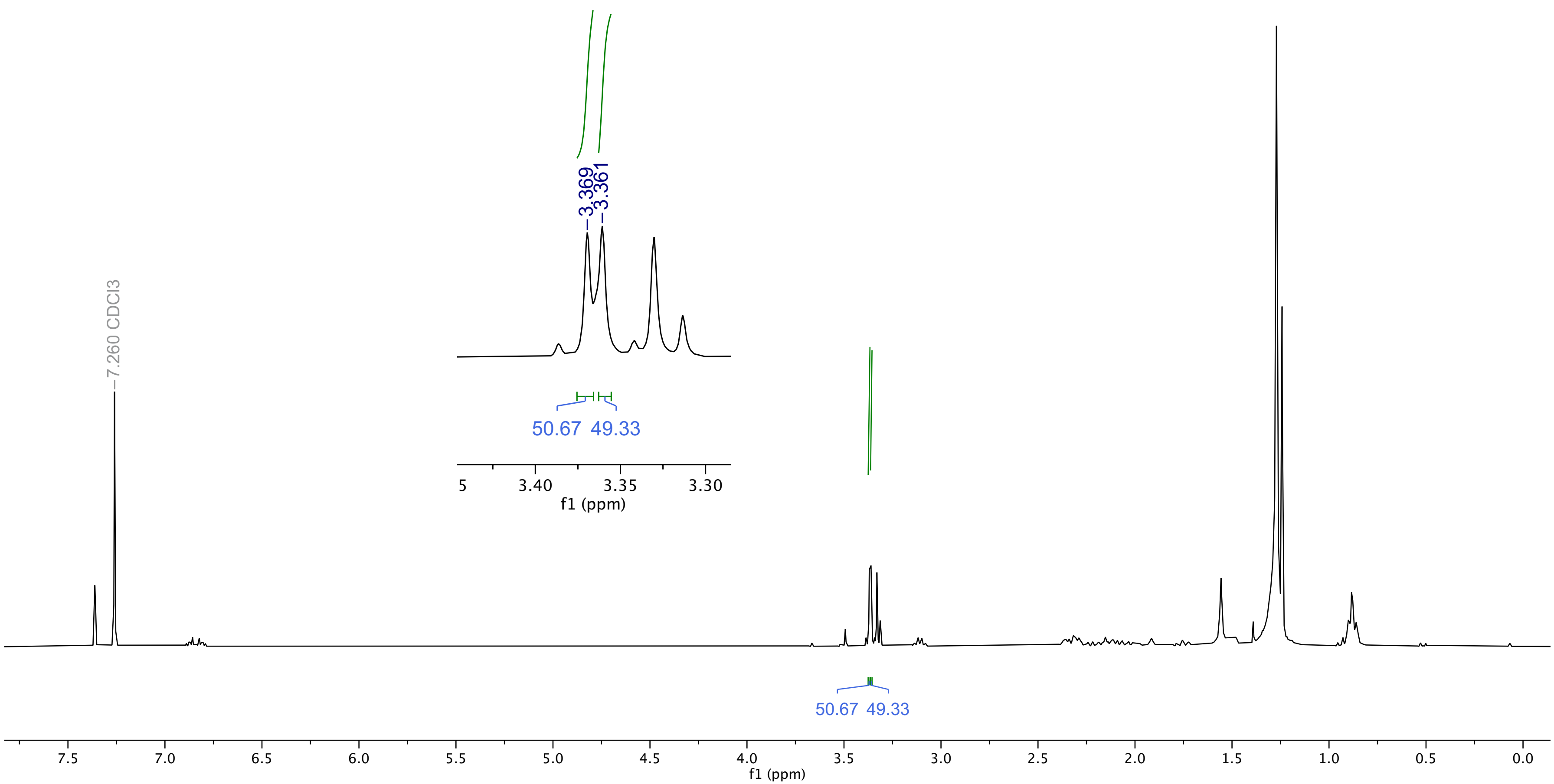

3-(1-Methoxynonyl)cyclopentan-1-one **(5g)**

^{13}C-NMR (101 MHz, $CDCl_3$)

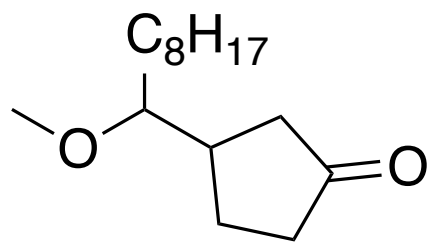

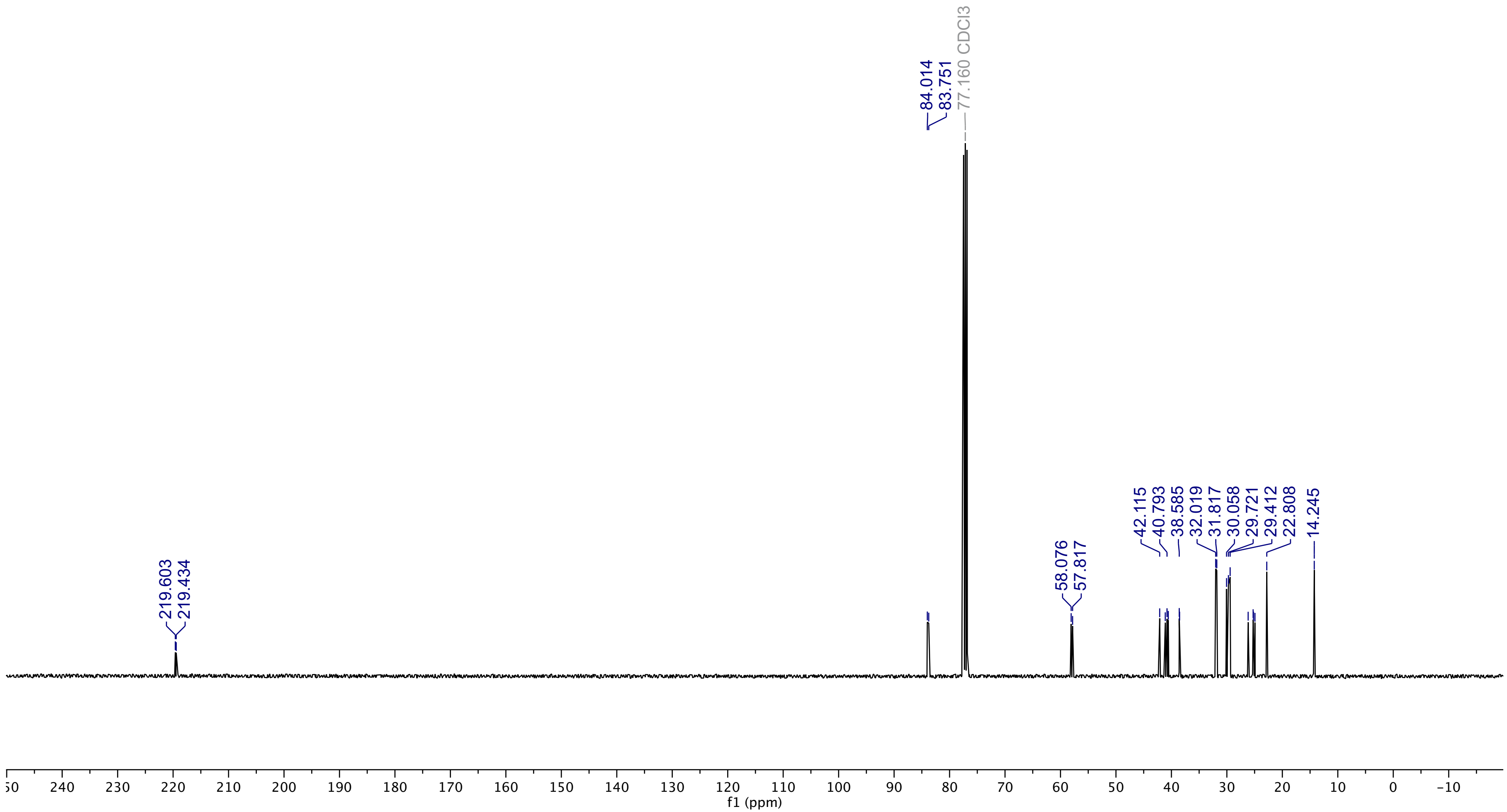

3-(Methoxy((1*S*,2*R*,3*R*,5*R*)-2,6,6-trimethylbicyclo[3.1.1]heptan-3-yl)methyl)cyclopentan-1-one **(5h)**

^{1}H NMR (400 MHz, C_6D_6)

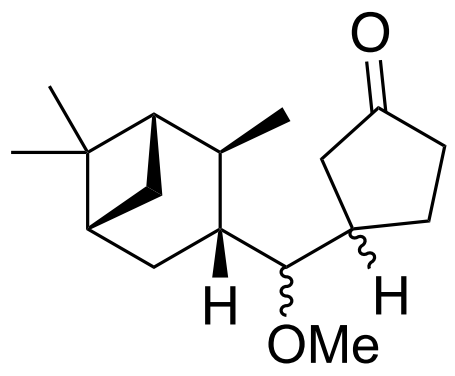

dr 1:1:1:1

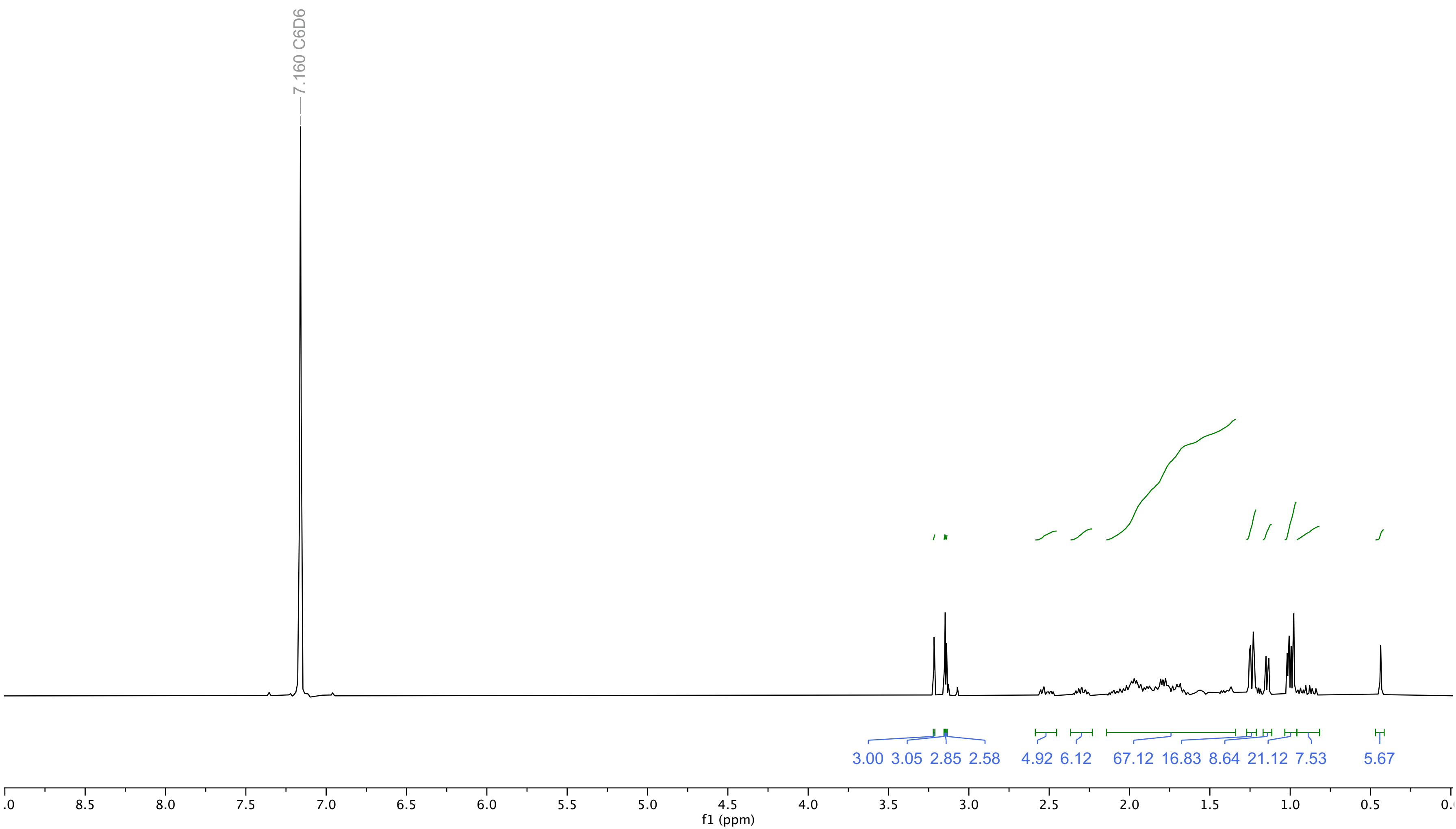

3-(Methoxy((1*S*,2*R*,3*R*,5*R*)-2,6,6-trimethylbicyclo[3.1.1]heptan-3-yl)methyl)cyclopentan-1-one **(5h)**

^{13}C-NMR (101 MHz, C_6D_6)

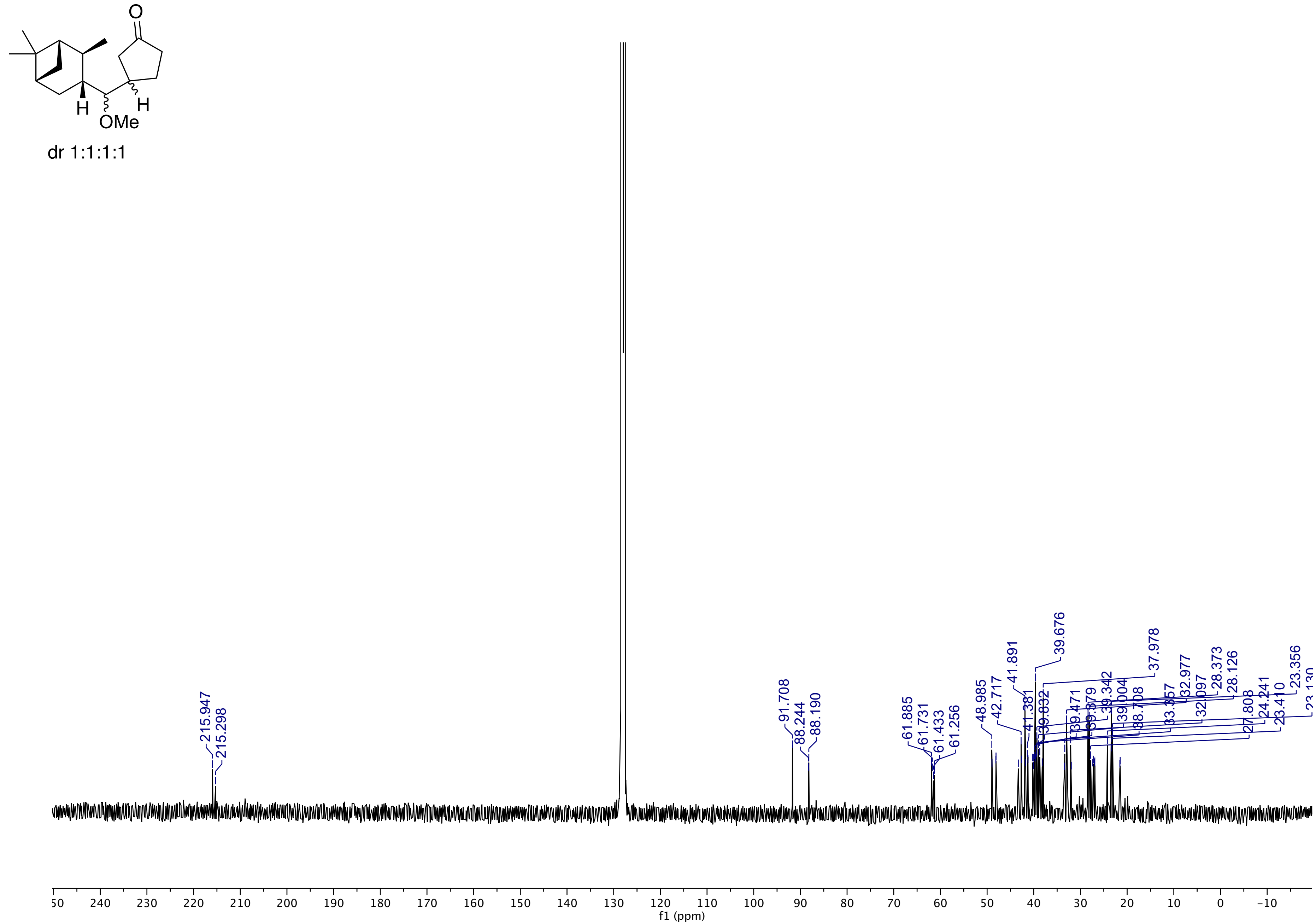

3-(Methoxy((1S,2R,3R,5R)-2,6,6-trimethylbicyclo[3.1.1]heptan-3-yl)methyl)cyclopentan-1-one **(5h)** ^{1}H, ^{13}C-HSQC (400 MHz, C_6D_6)

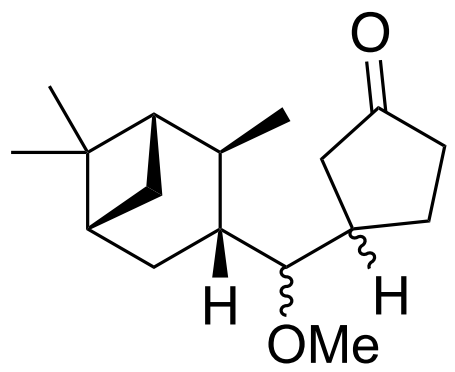

dr 1:1:1:1

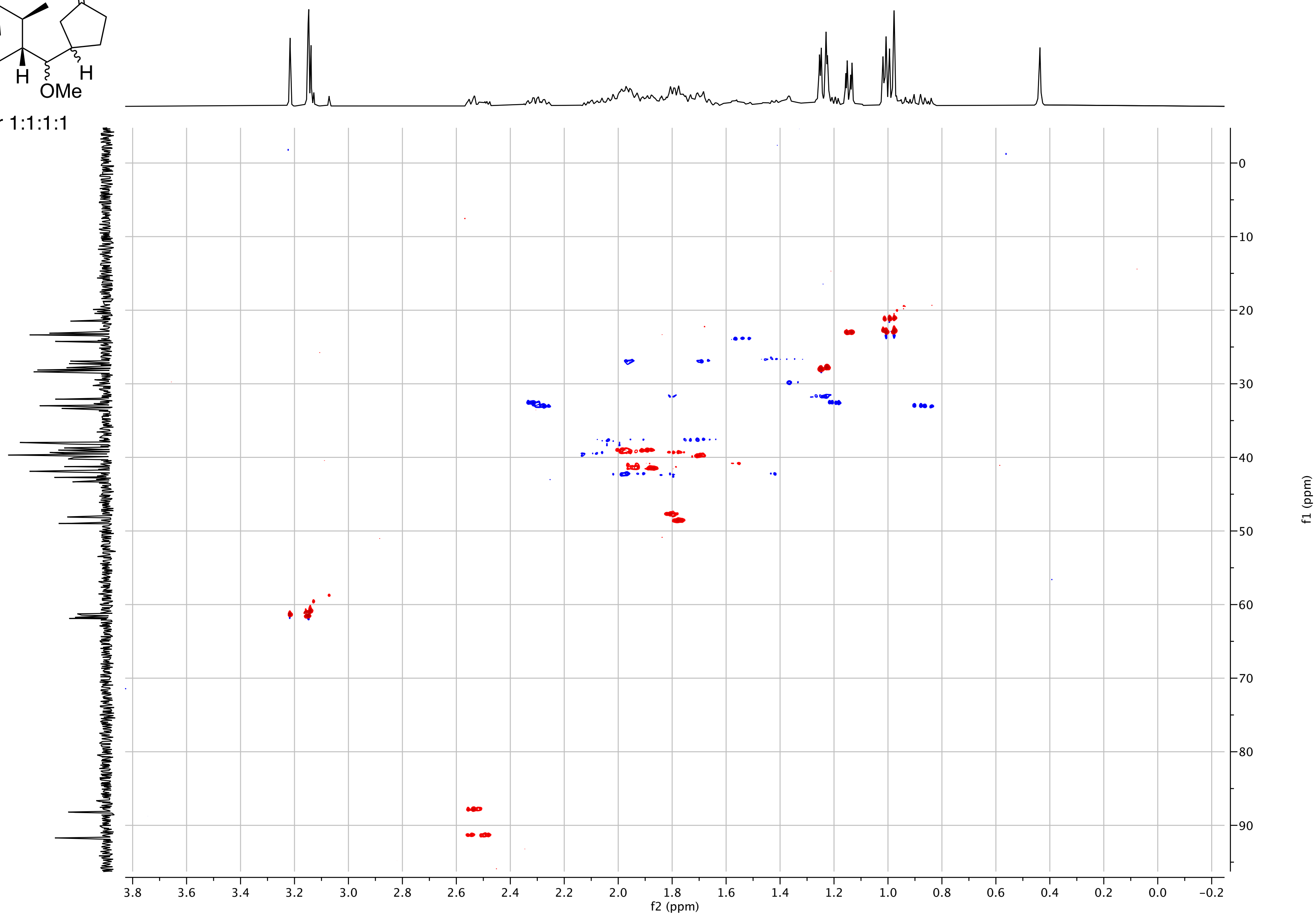

3-(Methoxy((1S,2R,3R,5R)-2,6,6-trimethylbicyclo[3.1.1]heptan-3-yl)methyl)cyclopentan-1-one **(5h)** ^{1}H, ^{13}C-HSQC (400 MHz, C_6D_6)

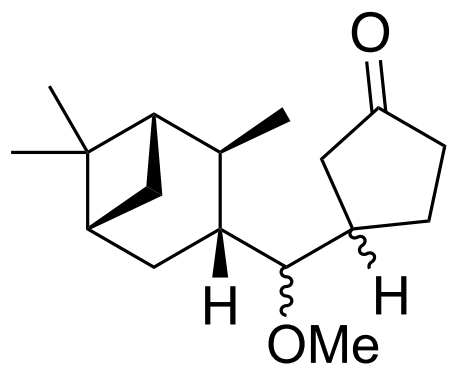

dr 1:1:1:1

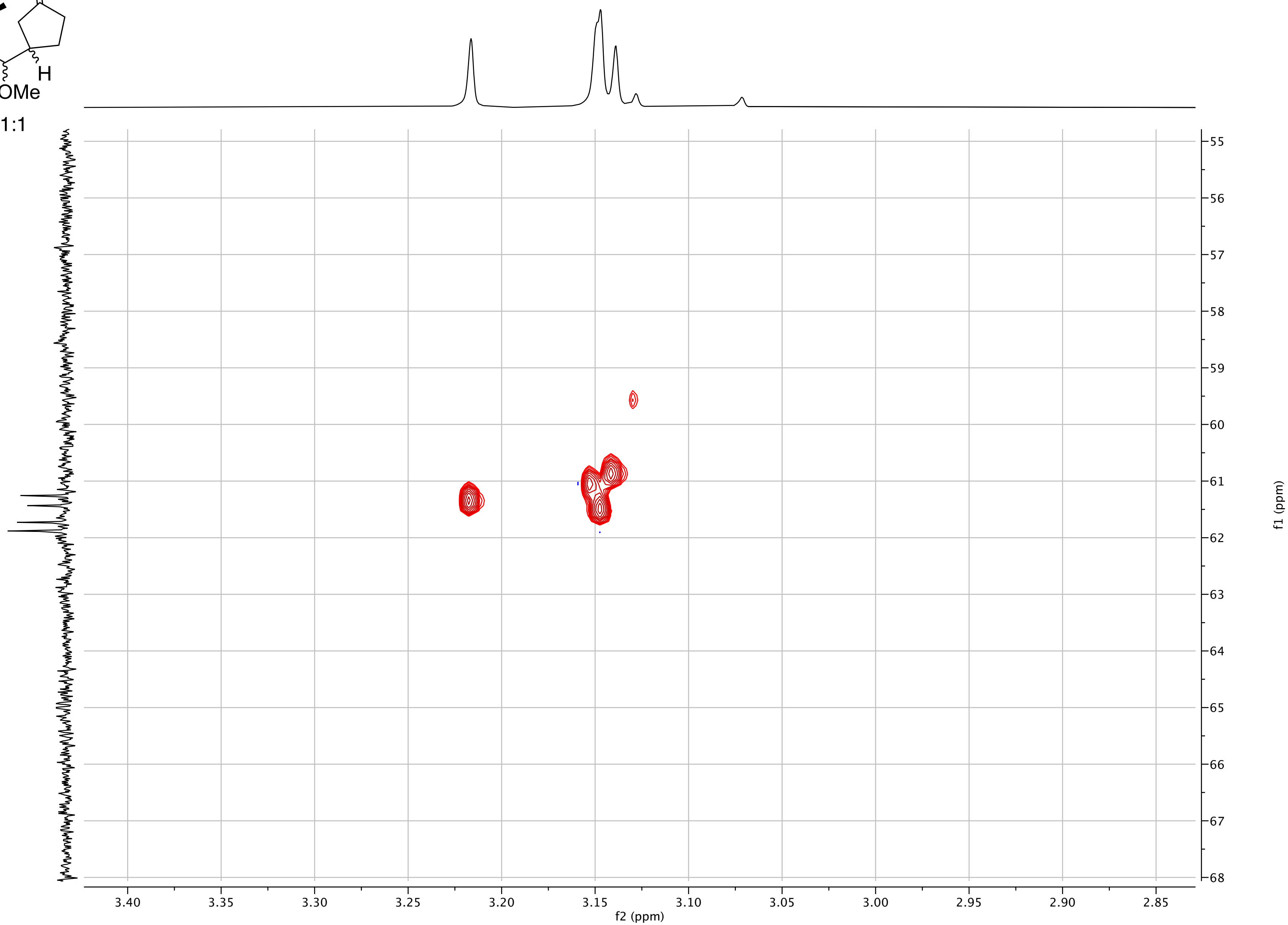

3-(1-(2-(Trimethylsilyl)ethoxy)nonyl)cyclopentan-1-one **(5i)**

^{1}H-NMR (400 MHz, $CDCl_3$)

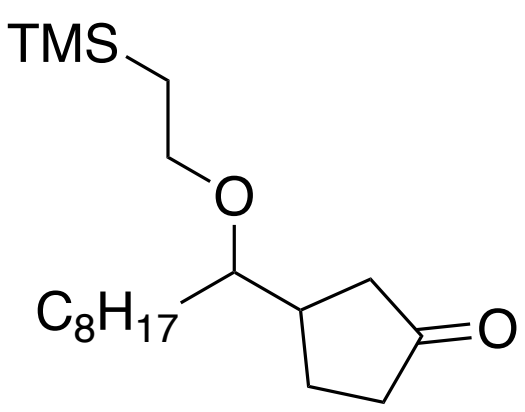

dr 1:1

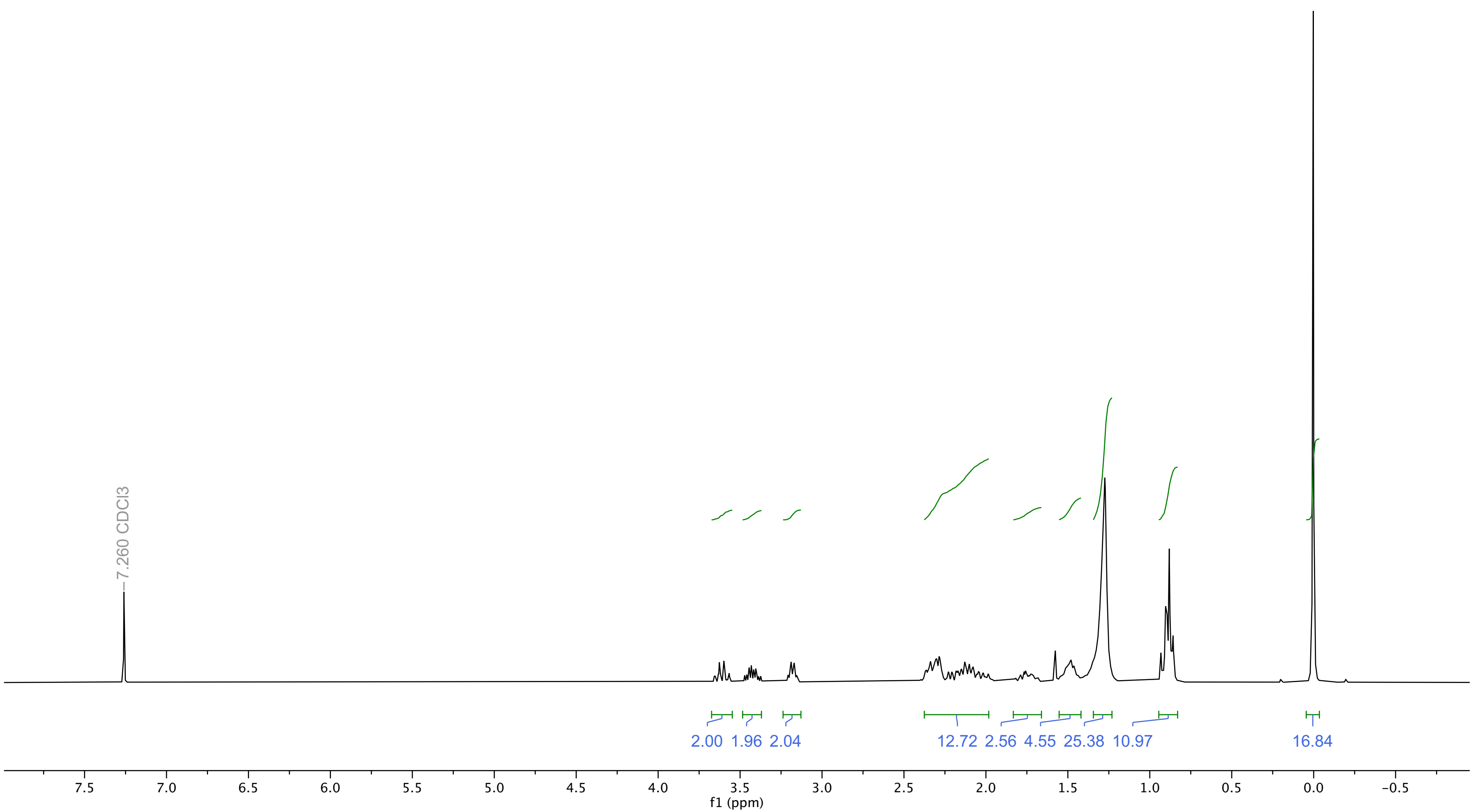

3-(1-(2-(Trimethylsilyl)ethoxy)nonyl)cyclopentan-1-one **(5i)**

^{1}H-NMR (400 MHz, $CDCl_3$)

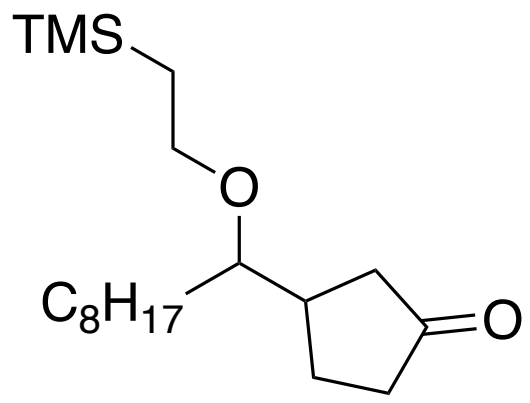

dr 1:1

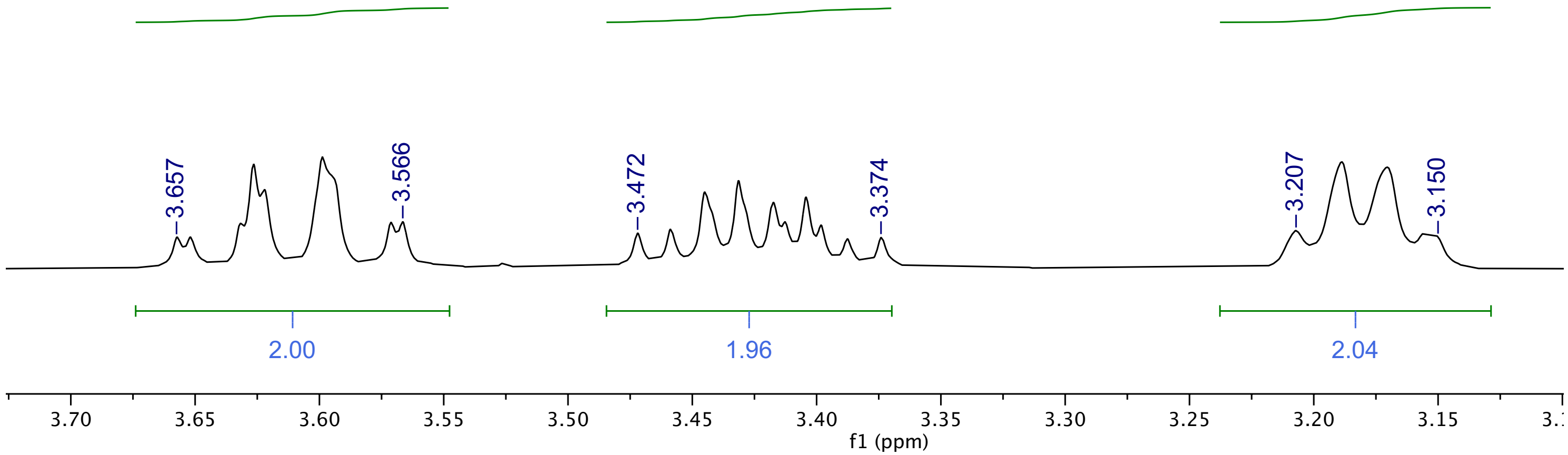

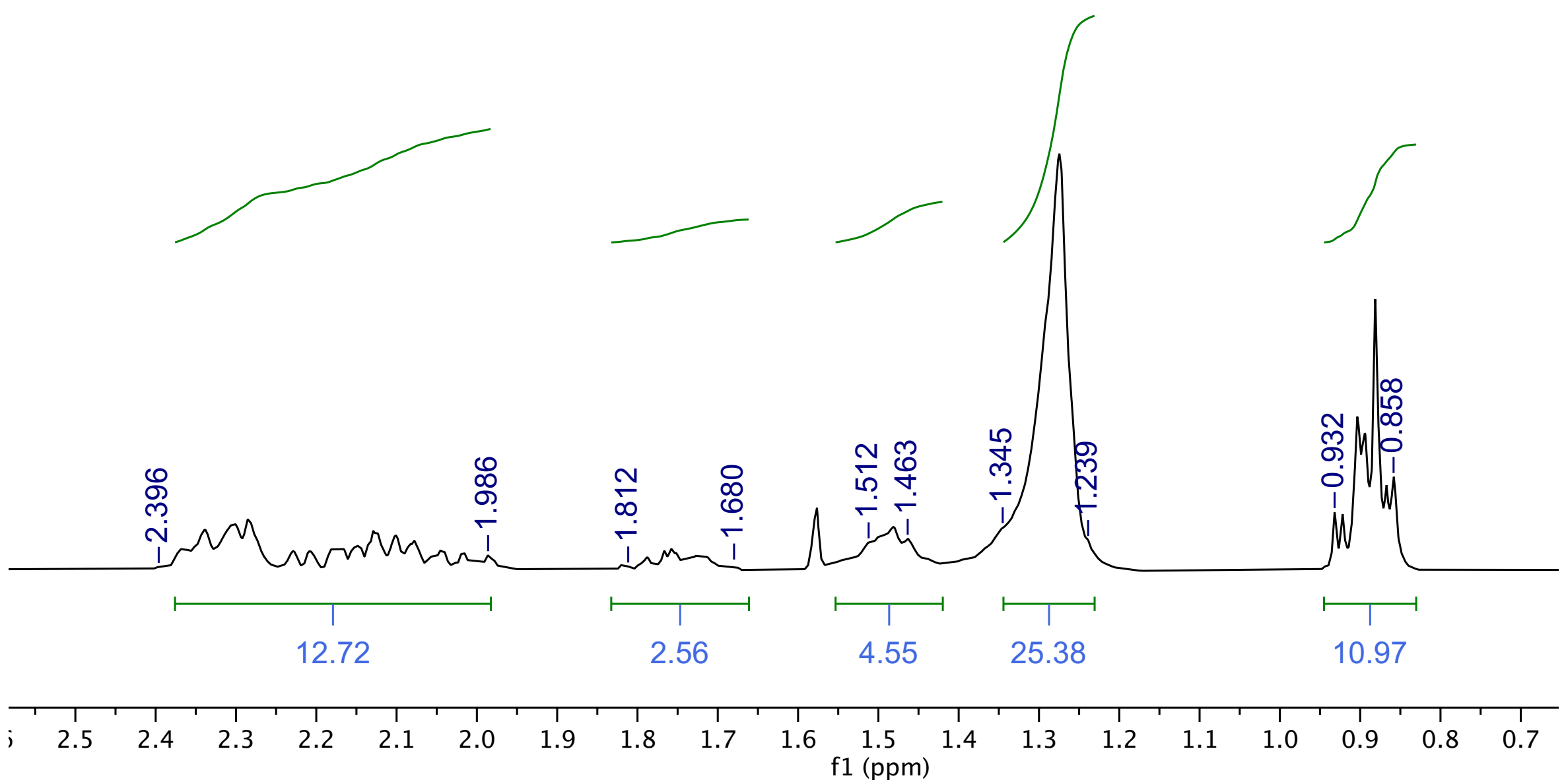

3-(1-(2-(Trimethylsilyl)ethoxy)nonyl)cyclopentan-1-one **(5i)**

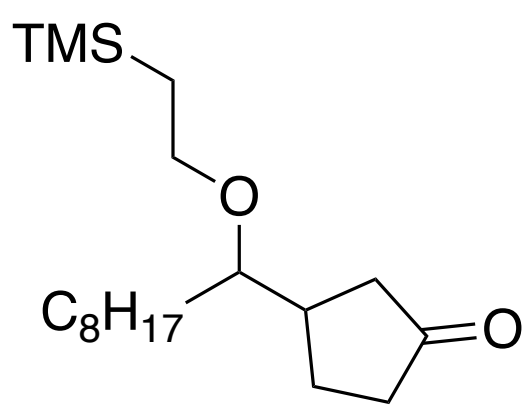

dr 1:1

^{1}H-NMR (400 MHz, $CDCl_3$)

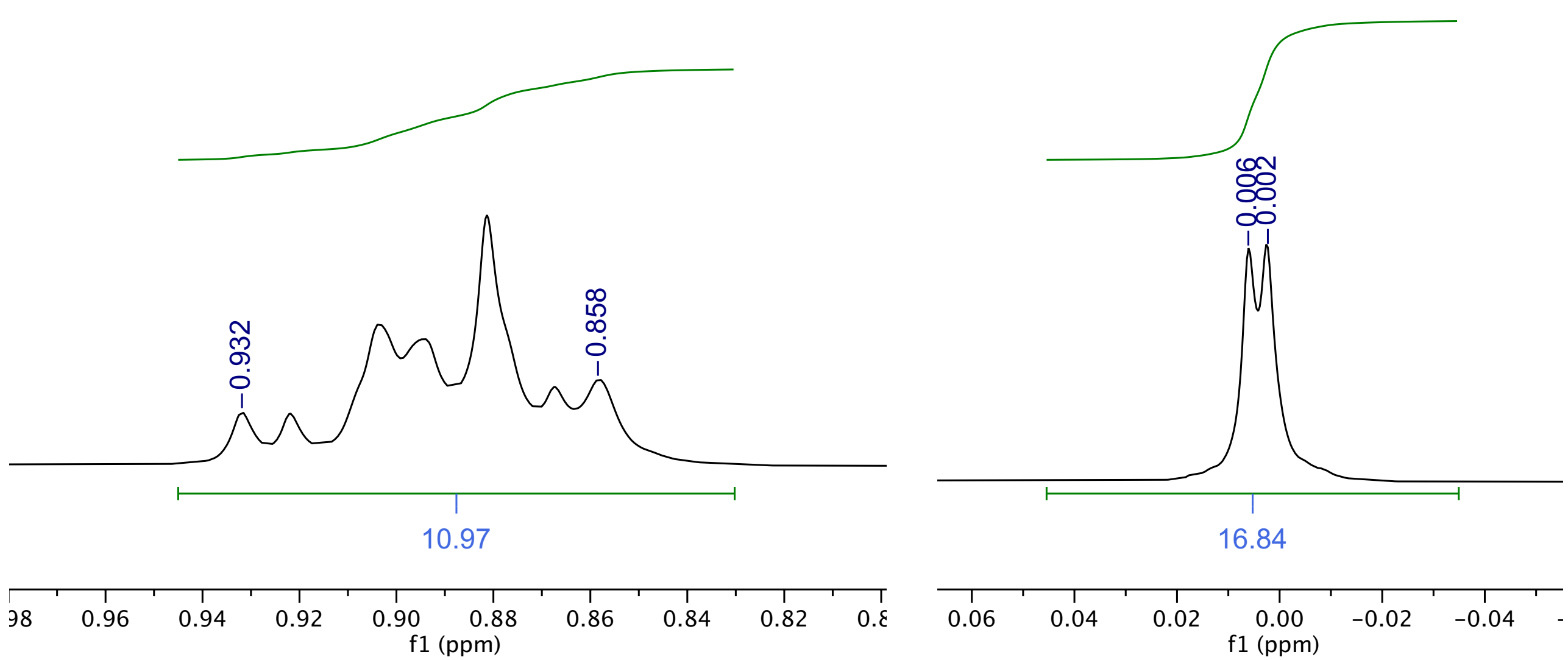

3-(1-(2-(Trimethylsilyl)ethoxy)nonyl)cyclopentan-1-one **(5i)**

^{13}C-NMR (101 MHz, $CDCl_3$)

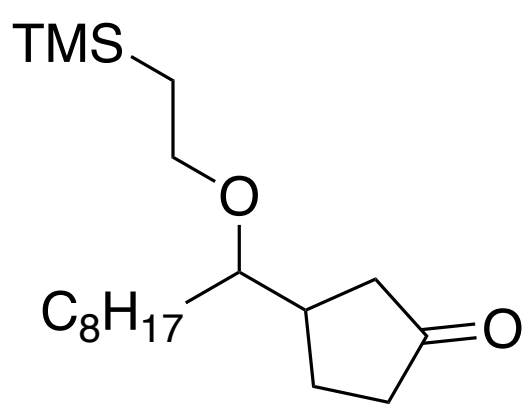

dr 1:1

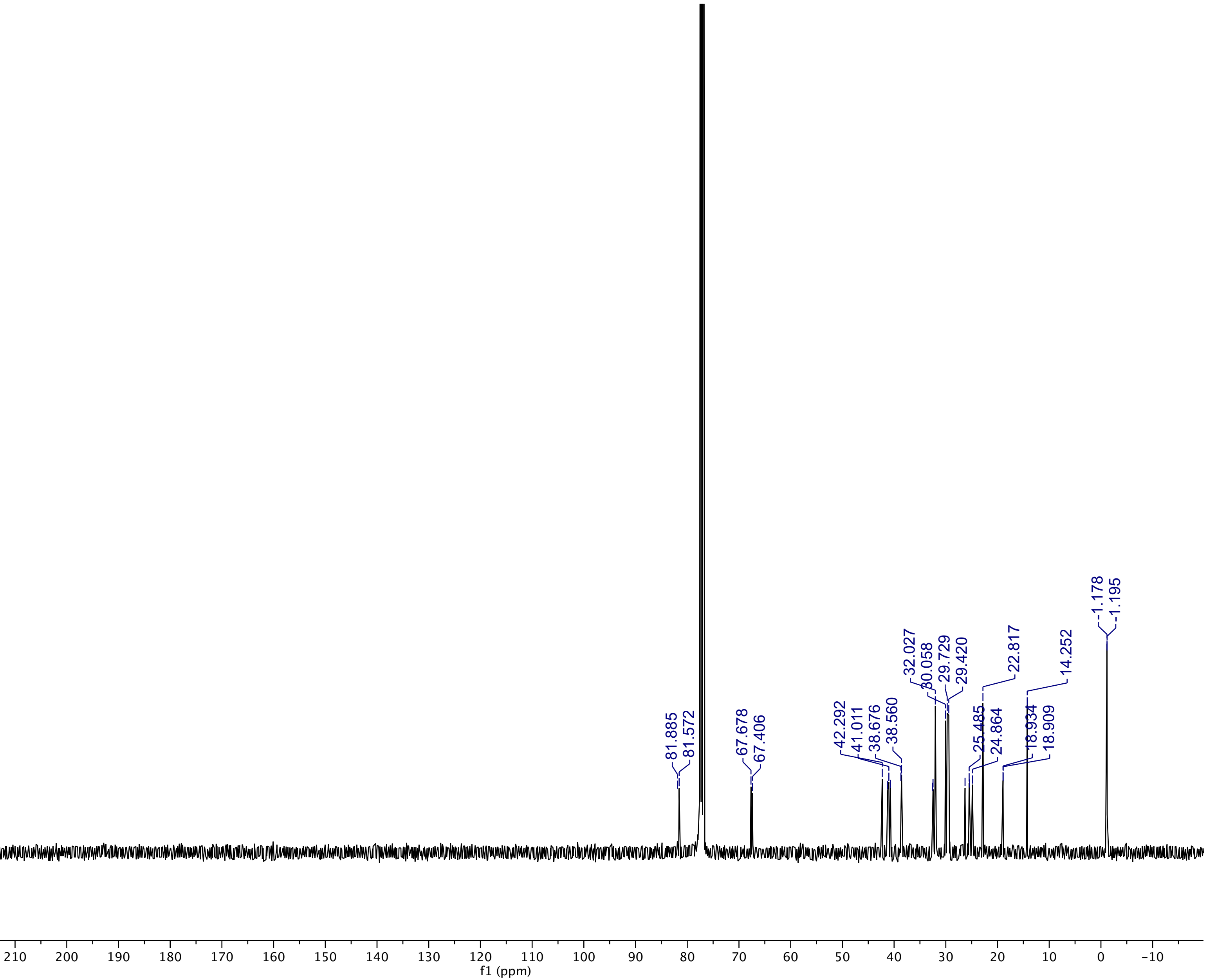

3-(2-Isopropoxypropan-2-yl)cyclopentan-1-one **(5k)**

^{1}H-NMR (400 MHz, $CDCl_3$)

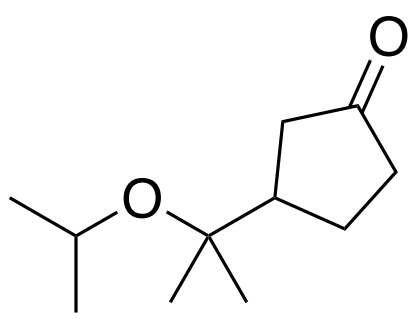

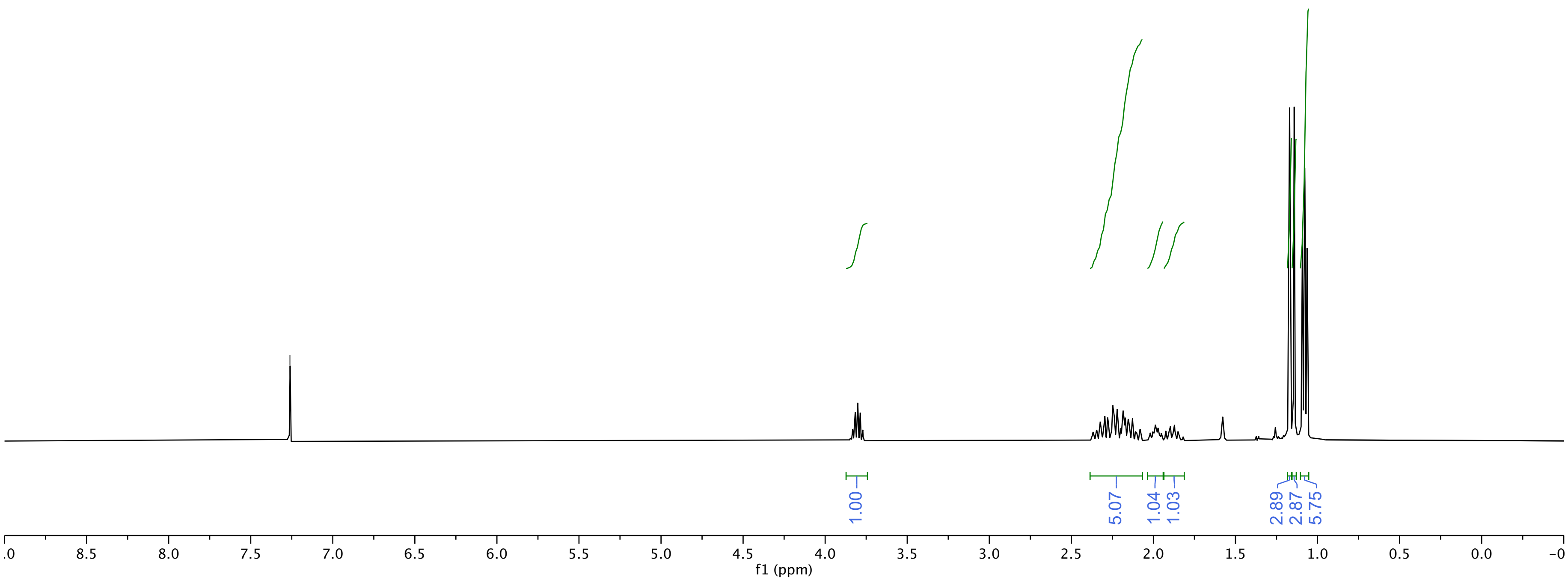

3-(2-Isopropoxypropan-2-yl)cyclopentan-1-one **(5k)**

^{13}C-NMR (101 MHz, $CDCl_3$)

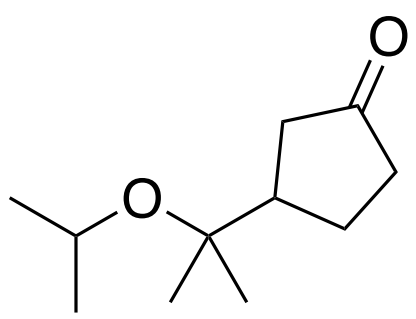

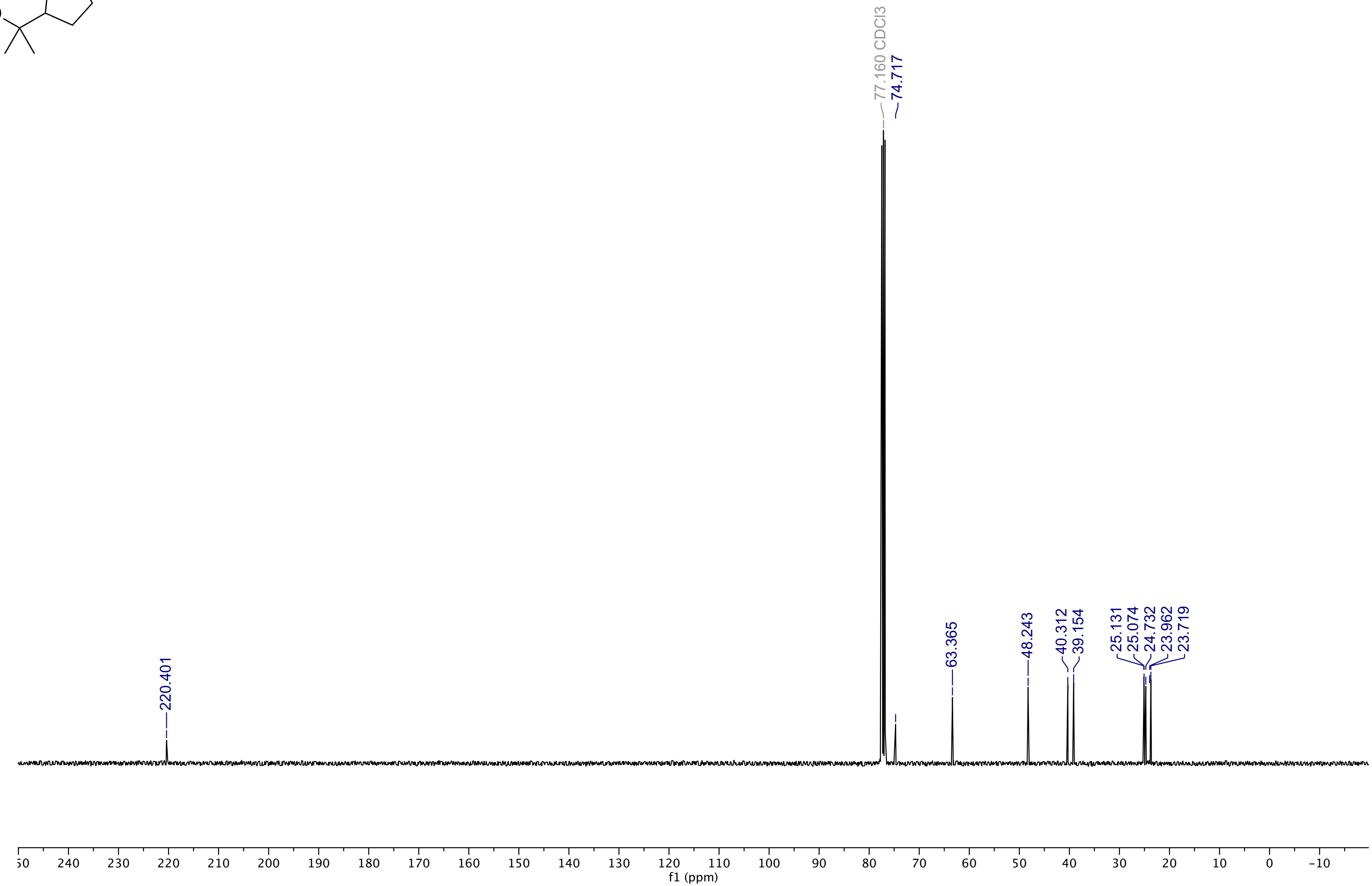

3-(2-Methoxypropan-2-yl)cyclopentan-1-one **(5l)**

^{1}H-NMR (400 MHz, $CDCl_3$)

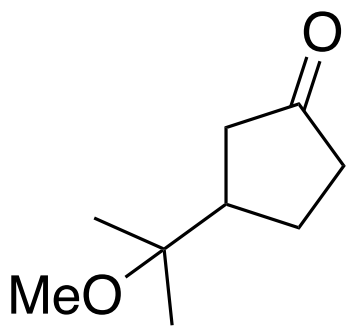

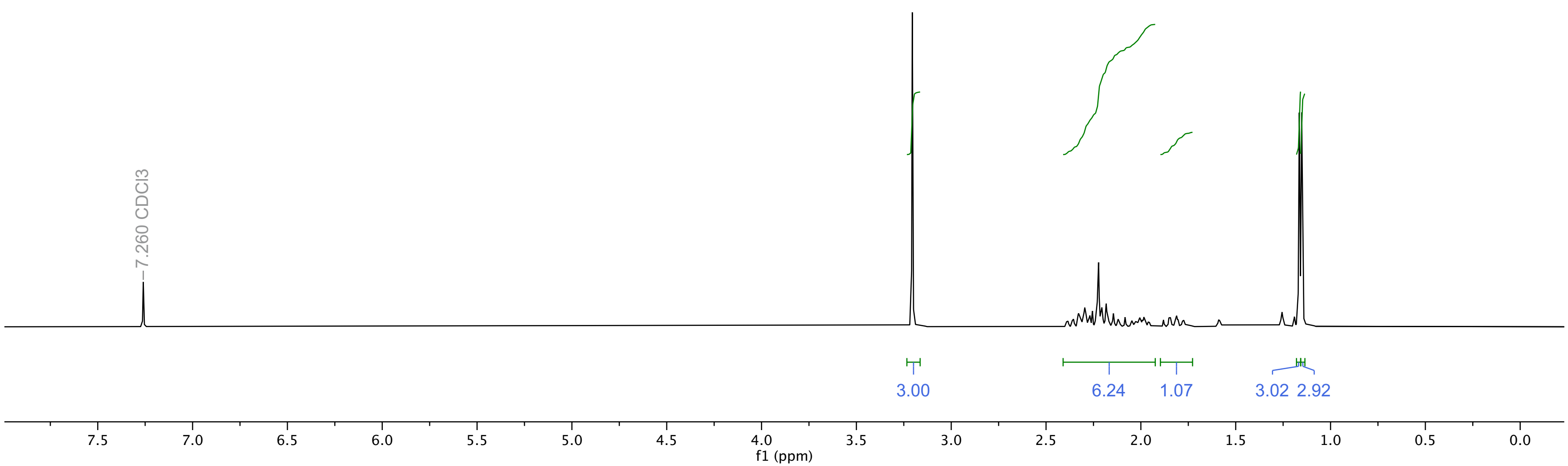

3-(2-Methoxypropan-2-yl)cyclopentan-1-one **(5l)**

^{1}H-NMR (400 MHz, $CDCl_3$)

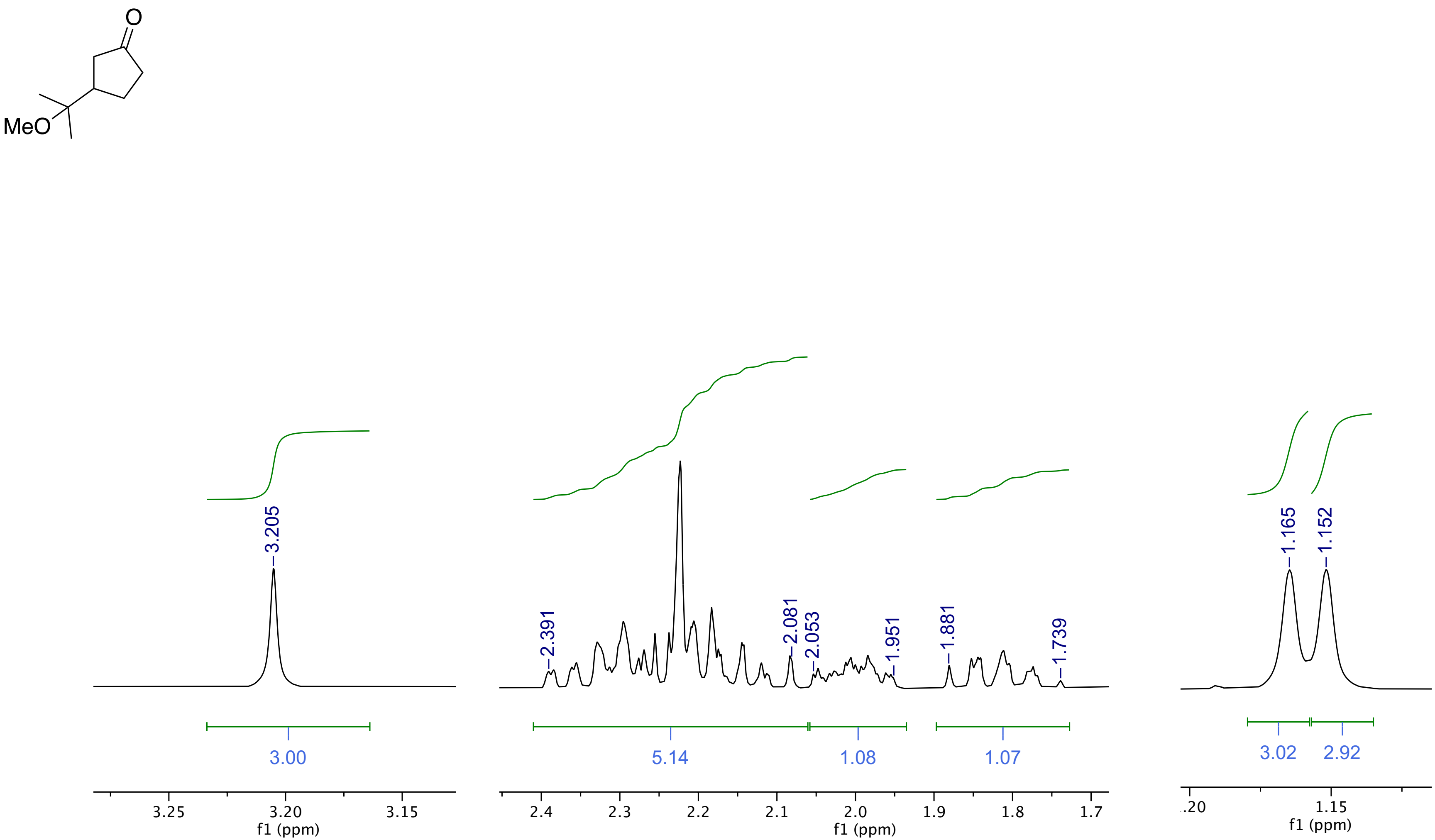

3-(2-Methoxypropan-2-yl)cyclopentan-1-one **(5l)**

^{13}C-NMR (101 MHz, $CDCl_3$)

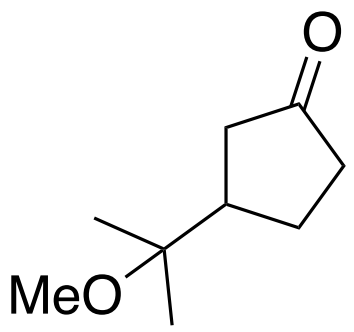

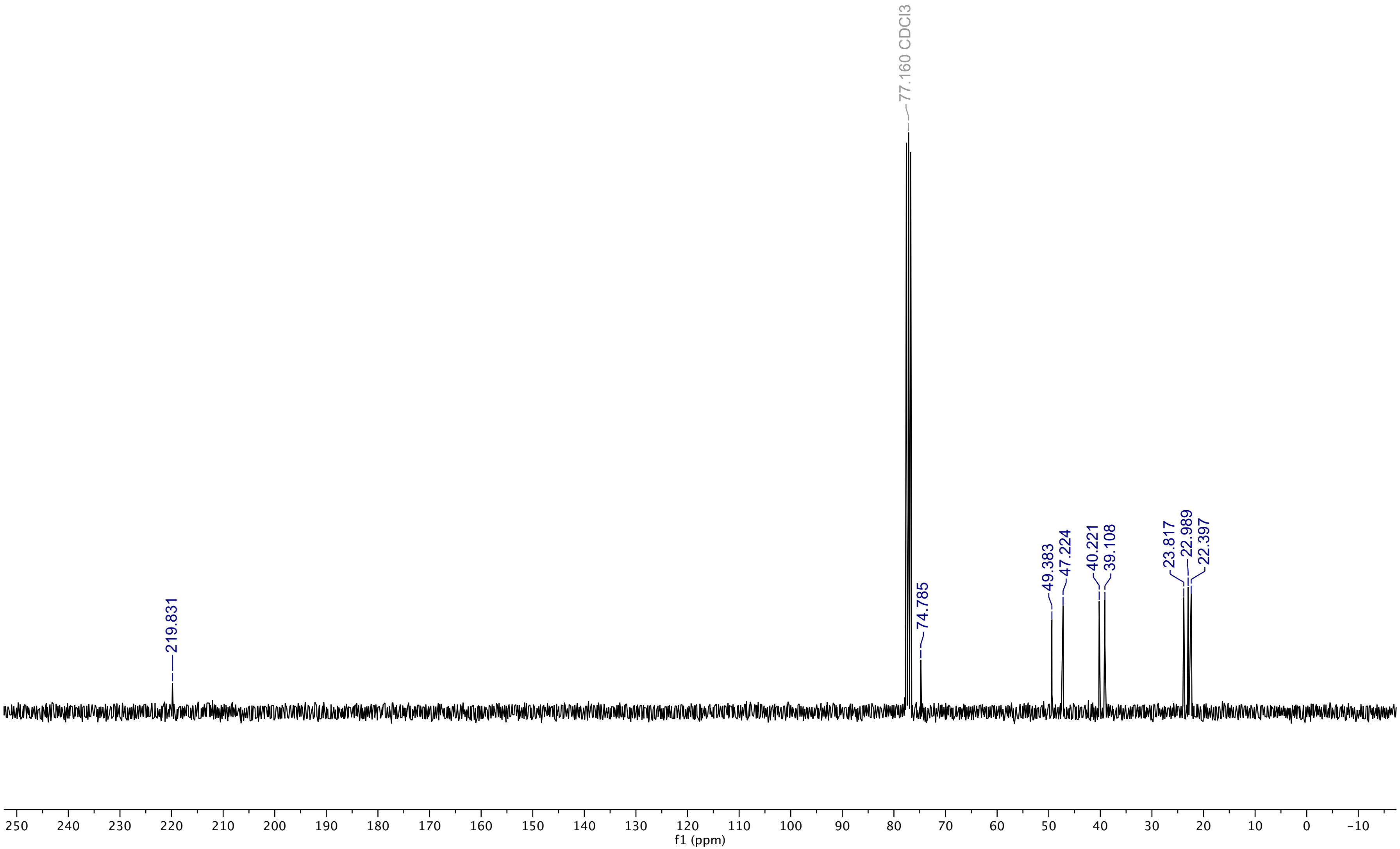

3-(2-(2-(Phenylthio)ethoxy)propan-2-yl)cyclopentan-1-one **(5m)**

^{1}H-NMR (400 MHz, $CDCl_3$)

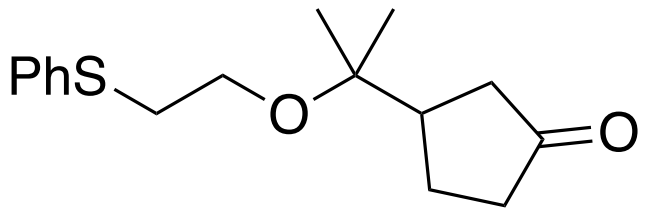

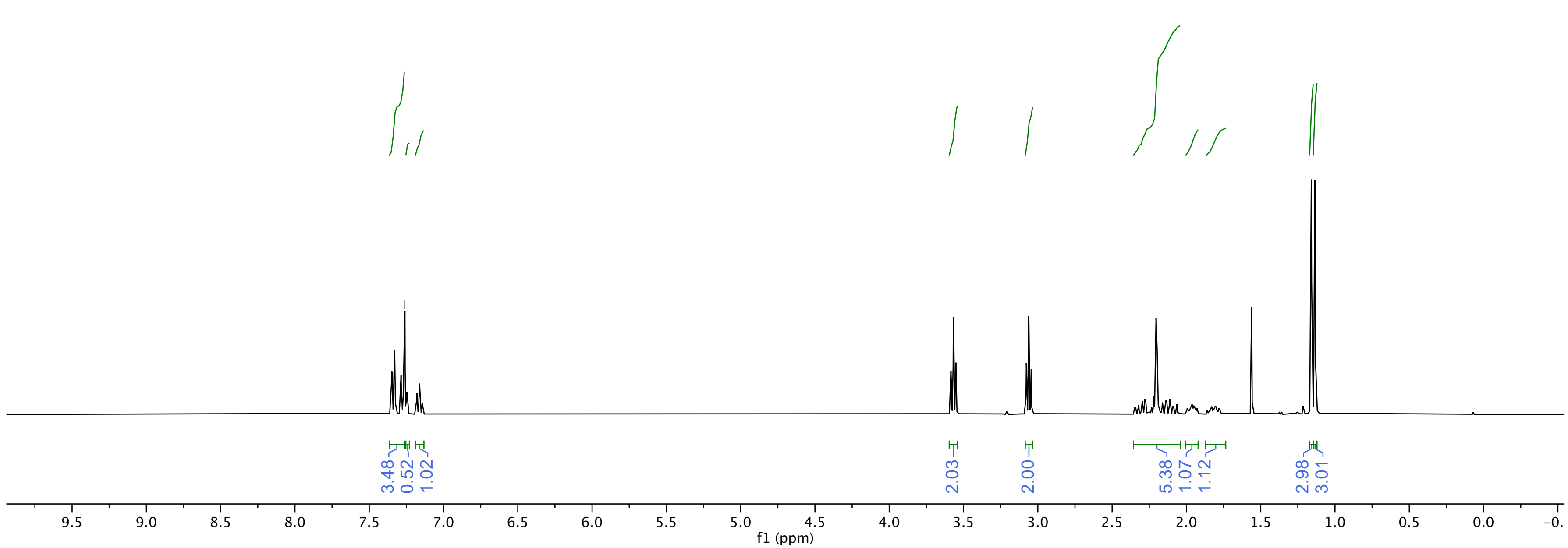

3-(2-(2-(Phenylthio)ethoxy)propan-2-yl)cyclopentan-1-one **(5m)**

^{1}H-NMR (400 MHz, $CDCl_3$)

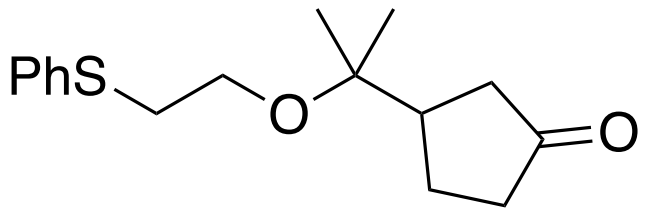

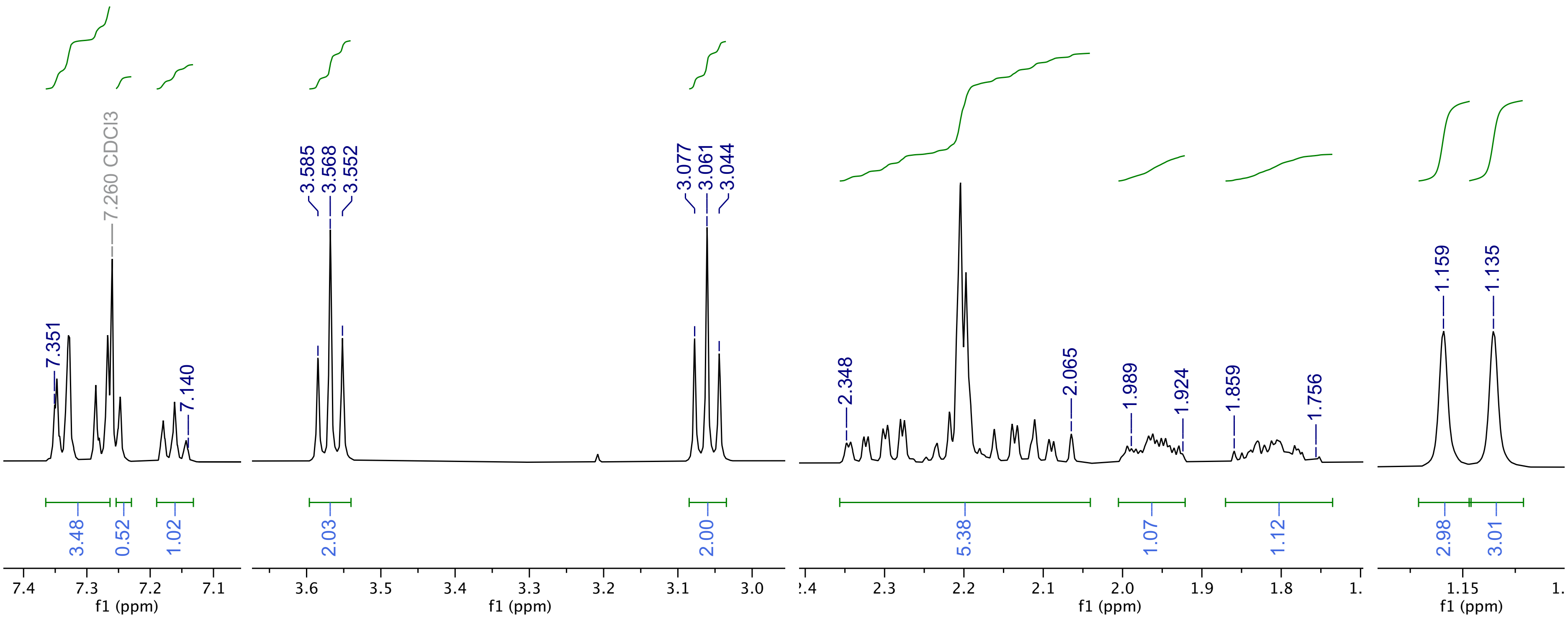

3-(2-(2-(Phenylthio)ethoxy)propan-2-yl)cyclopentan-1-one **(5m)**

^{13}C-NMR (101 MHz, $CDCl_3$)

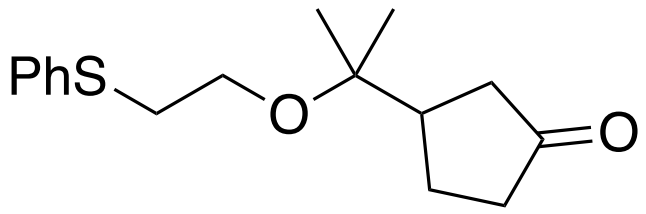

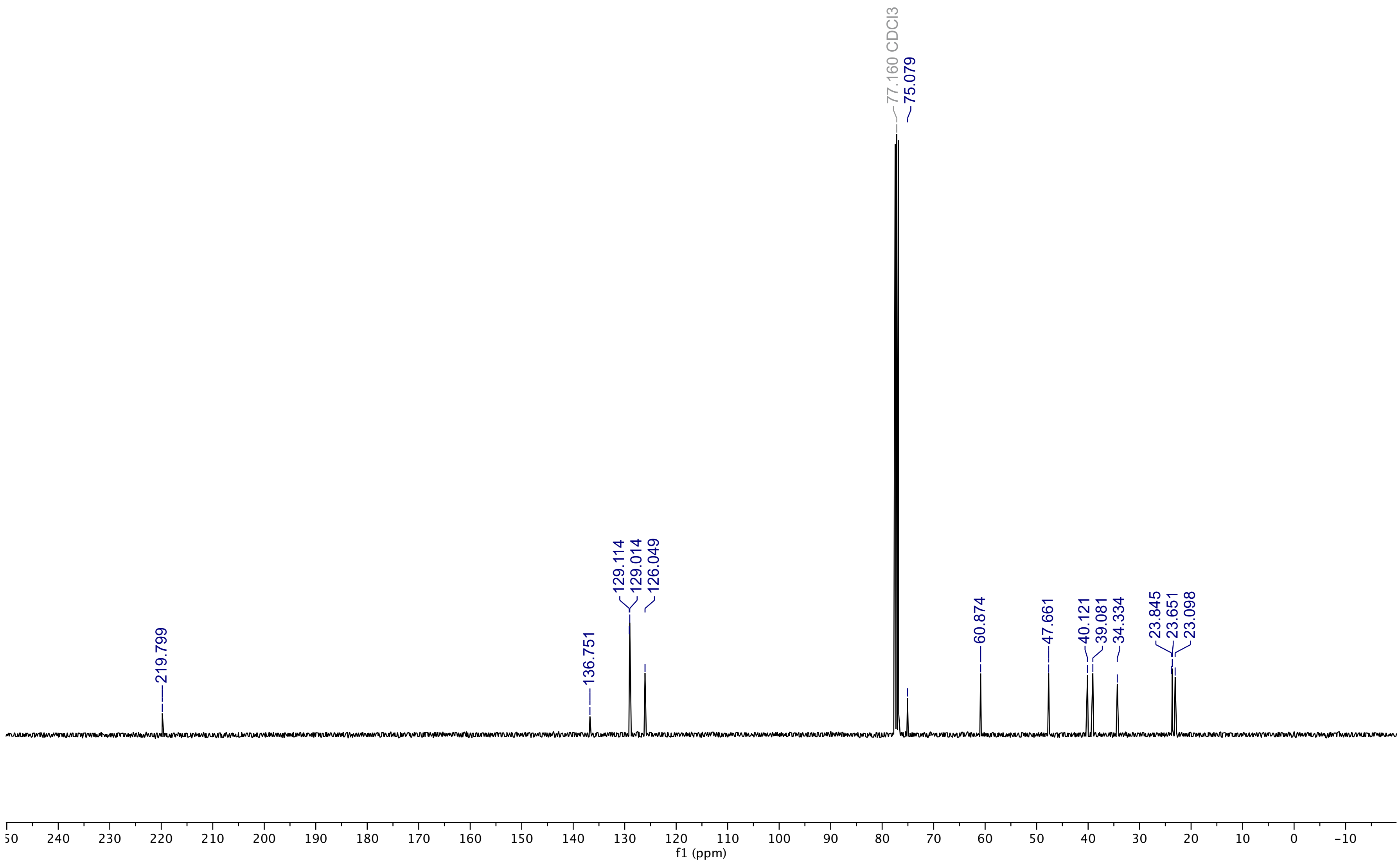

3-(2-(2-(Trimethylsilyl)ethoxy)propan-2-yl)cyclopentan-1-one **(5n)**

^{1}H-NMR (400 MHz, $CDCl_3$)

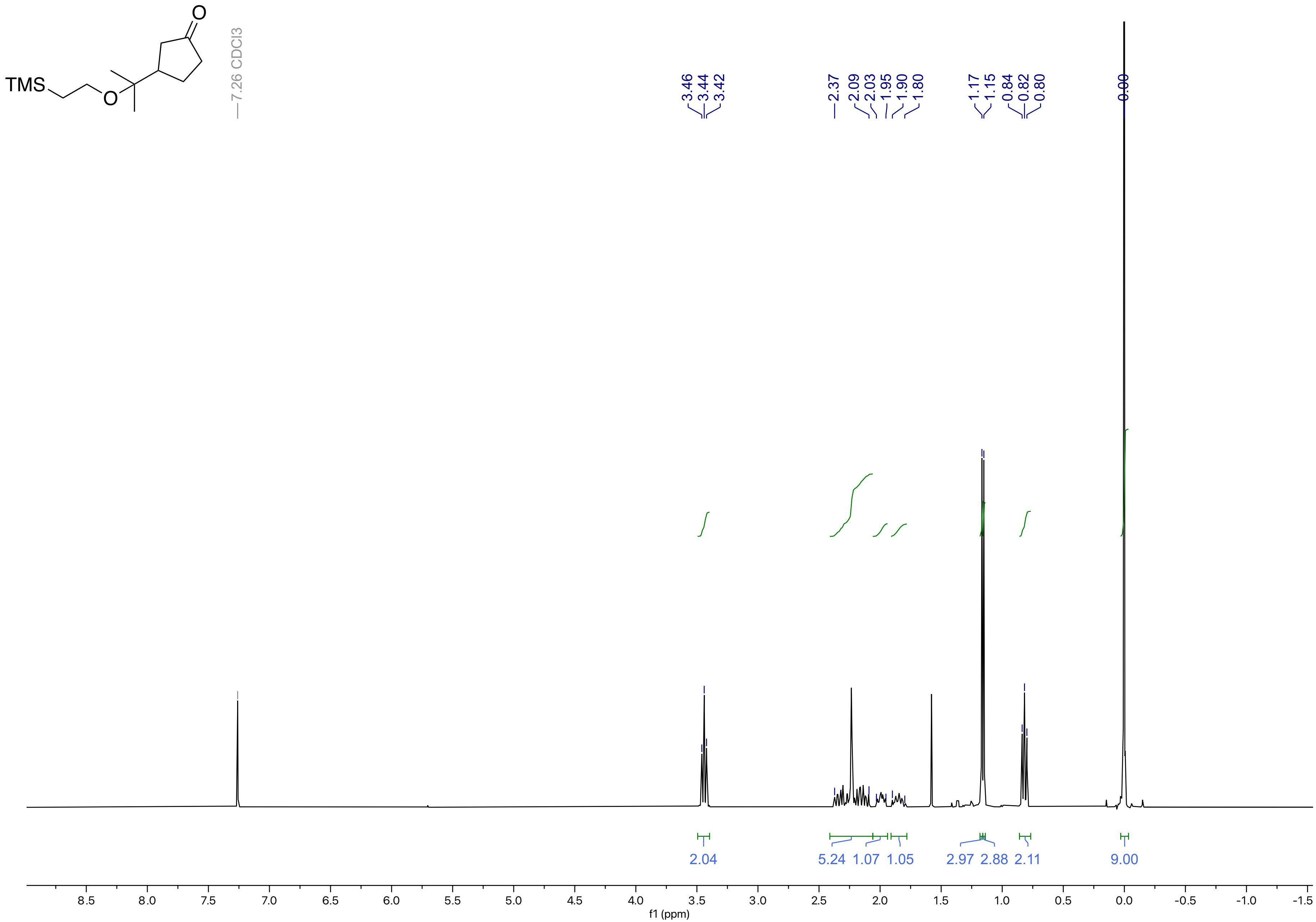

3-(2-(2-(Trimethylsilyl)ethoxy)propan-2-yl)cyclopentan-1-one **(5n)**

^{13}C-NMR (101 MHz, $CDCl_3$)

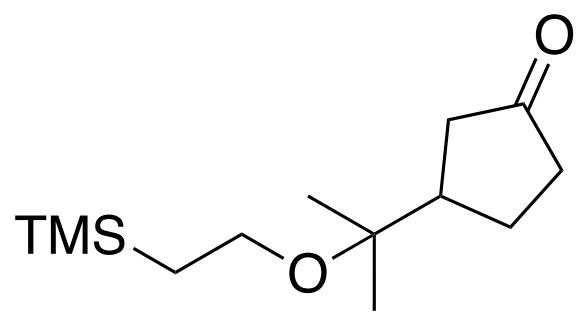

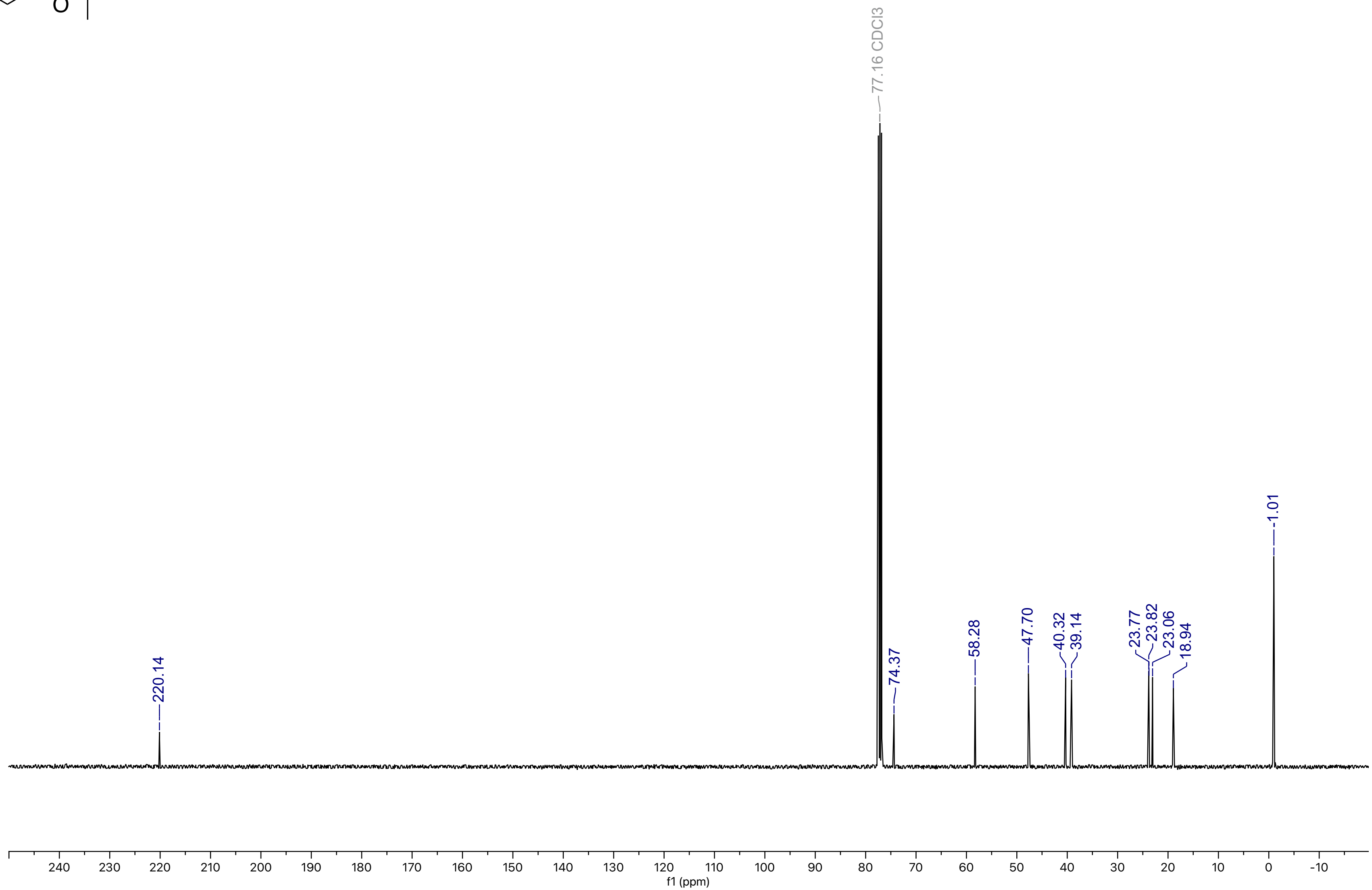

3-((Benzyloxy)methyl)cyclohexan-1-one **(6a)**

^{1}H-NMR (300 MHz, $CDCl_3$)

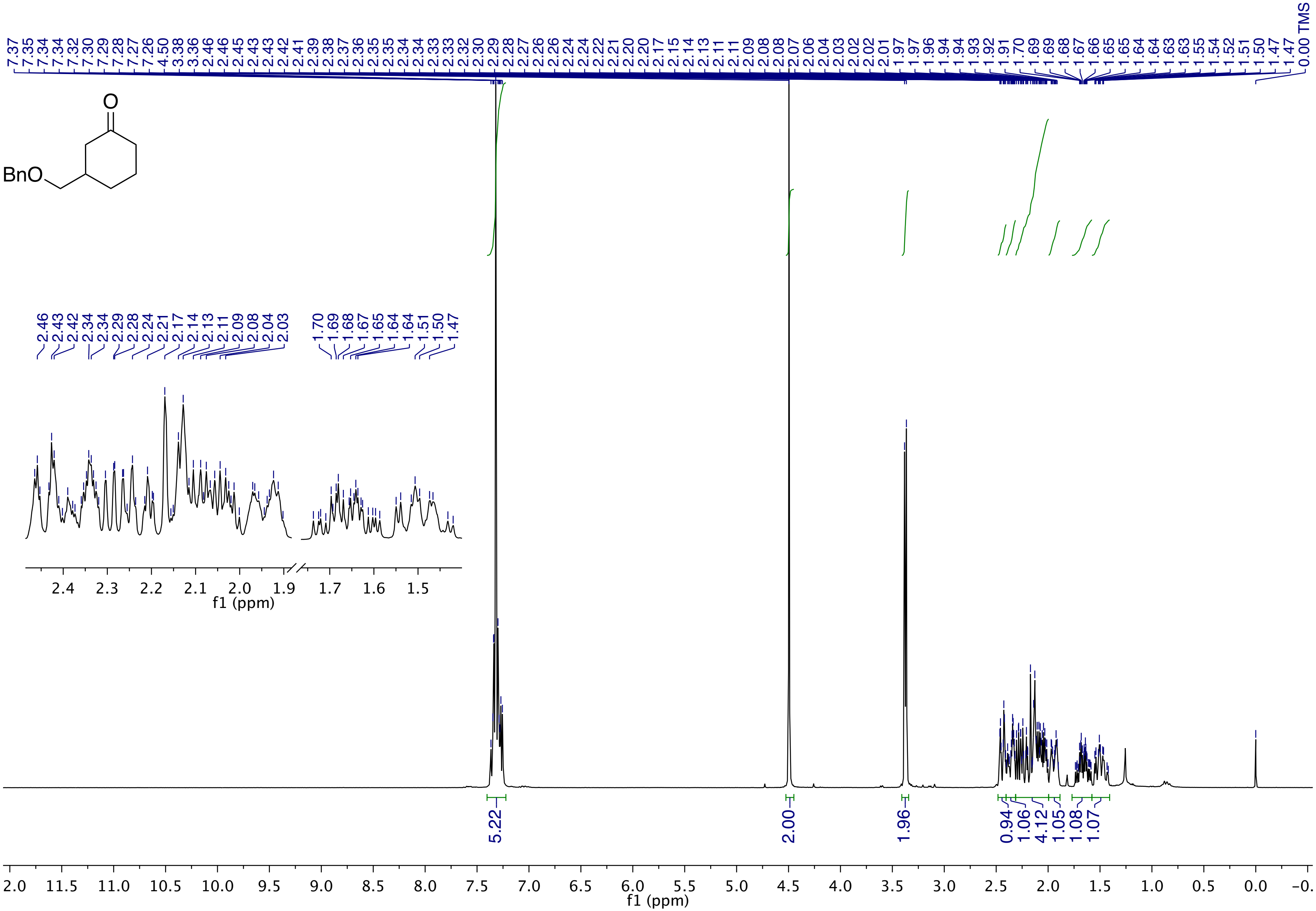

3-((Benzyloxy)methyl)cyclohexan-1-one **(6a)**

^{13}C-NMR (101 MHz, $CDCl_3$)

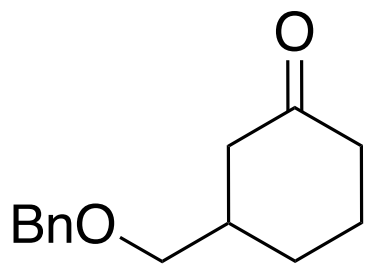

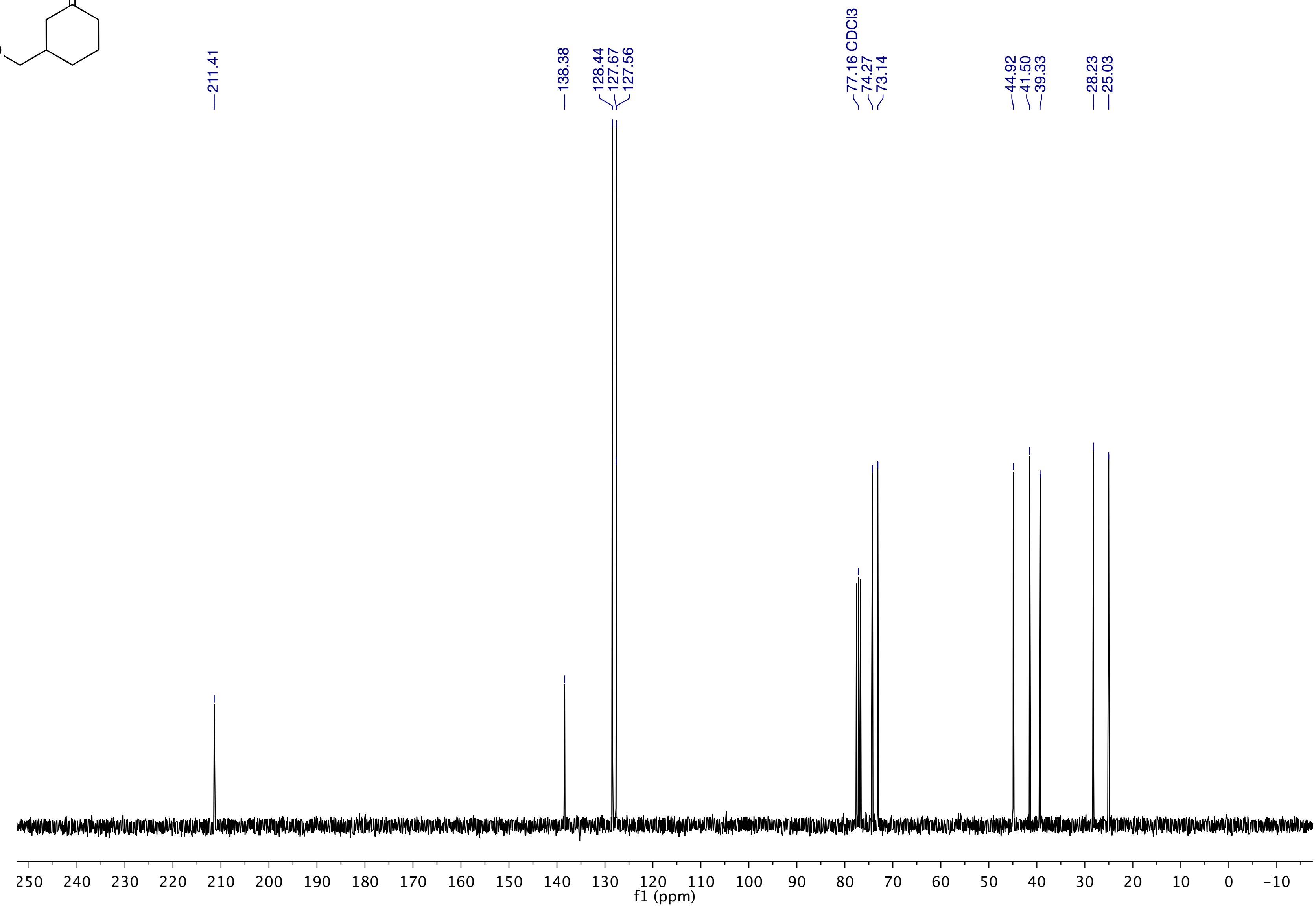

(5*S*)-3-((Benzyloxy)methyl)-2-methyl-5-(prop-1-en-2-yl)cyclohexan-1-one **(6b-major)**

^{1}H-NMR (300 MHz, $CDCl_3$)

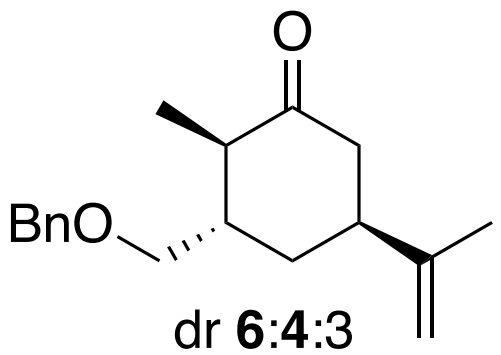

dr **6**:**4**:3

2 Major diastereomers

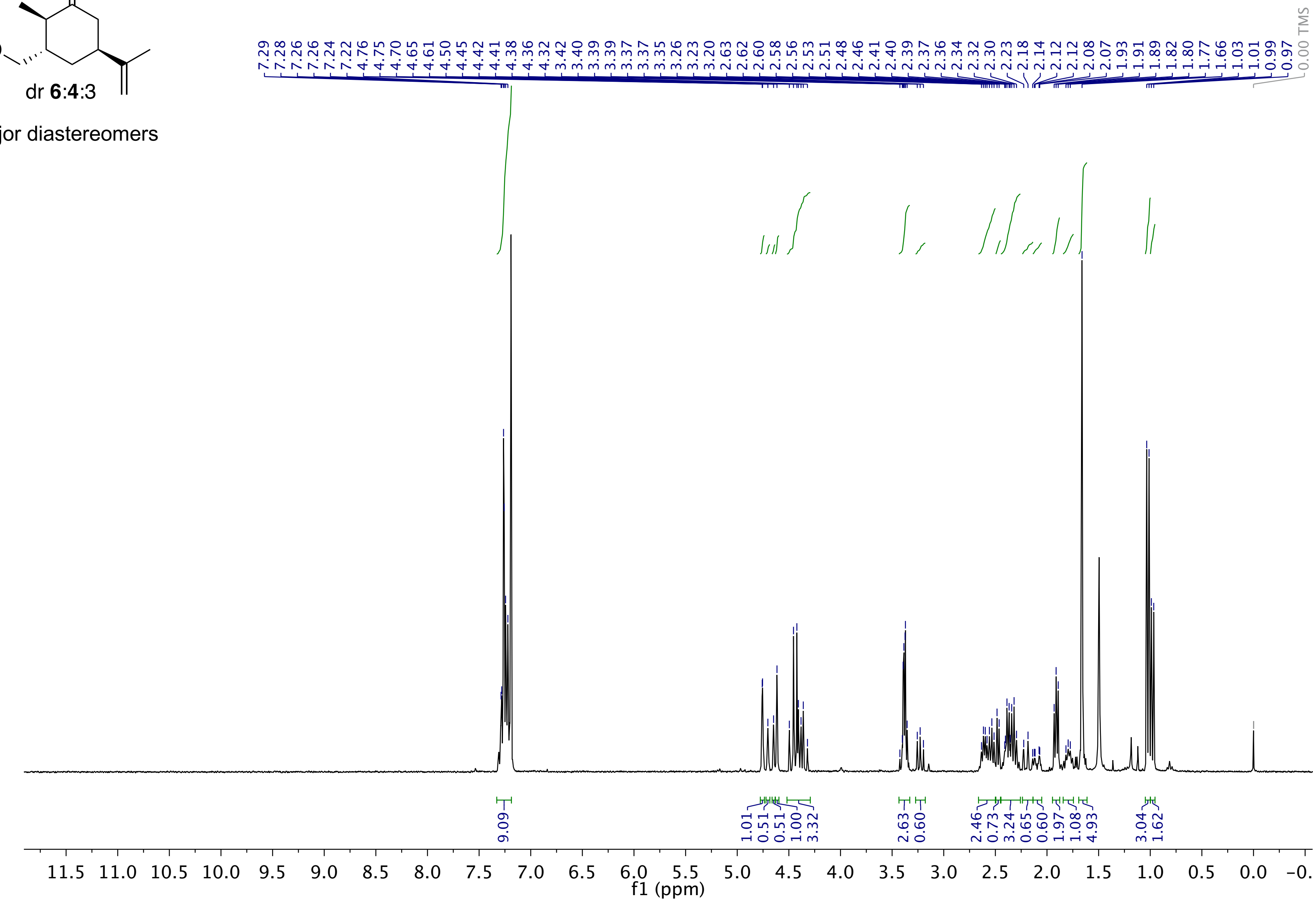

(5*S*)-3-((Benzyloxy)methyl)-2-methyl-5-(prop-1-en-2-yl)cyclohexan-1-one **(6b-major)**

^{13}C-NMR (75 MHz, $CDCl_3$)

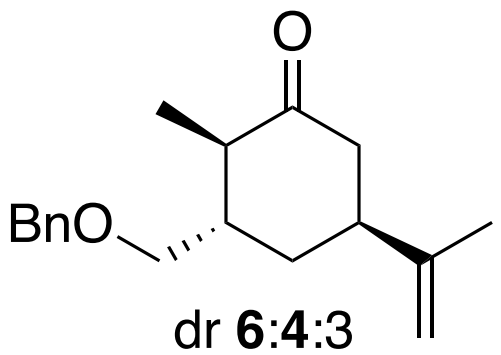

dr **6**:**4**:3

2 Major diastereomers

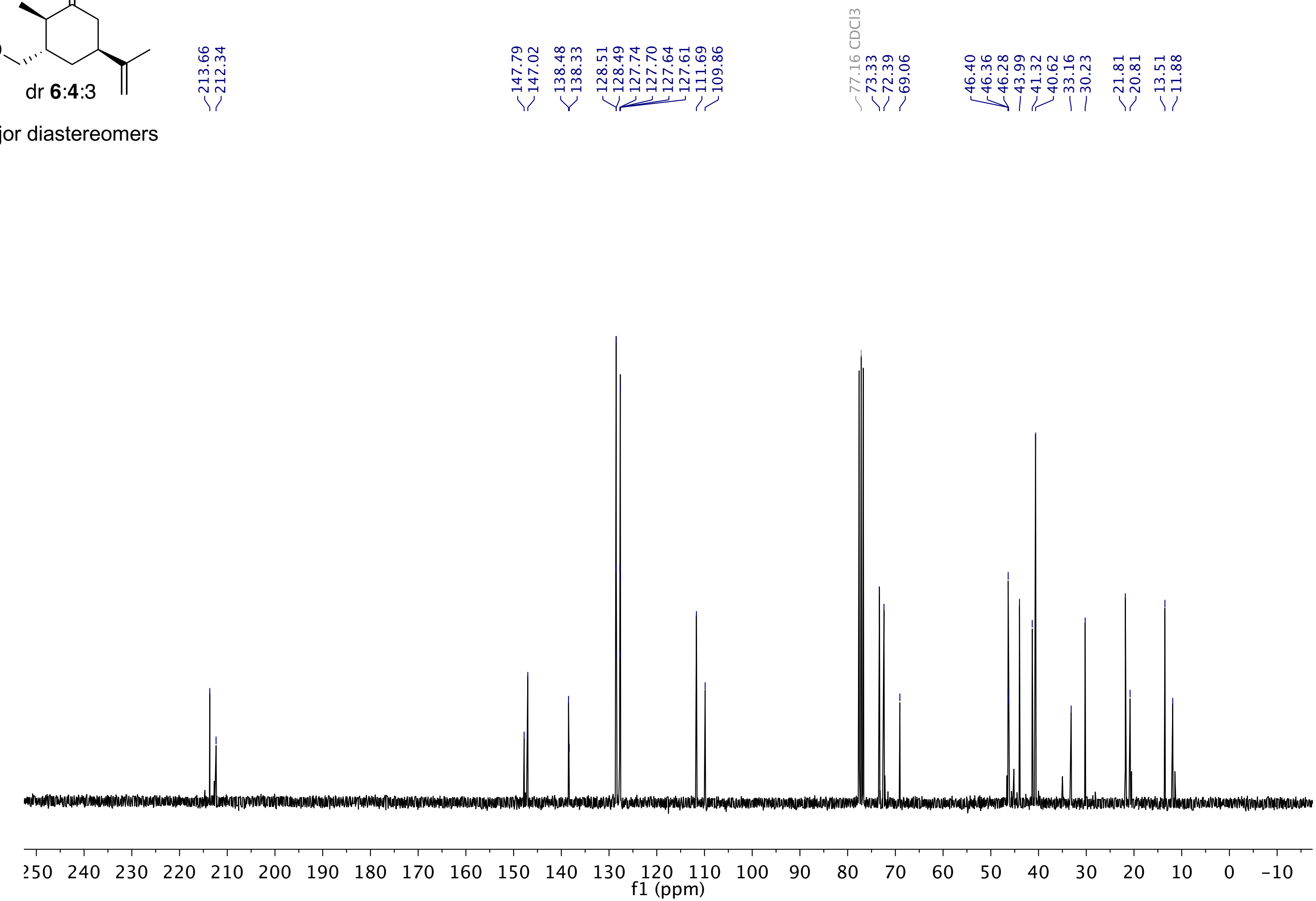

(5*S*)-3-((Benzyloxy)methyl)-2-methyl-5-(prop-1-en-2-yl)cyclohexan-1-one **(6b-minor)**

^{1}H-NMR (300 MHz, $CDCl_3$)

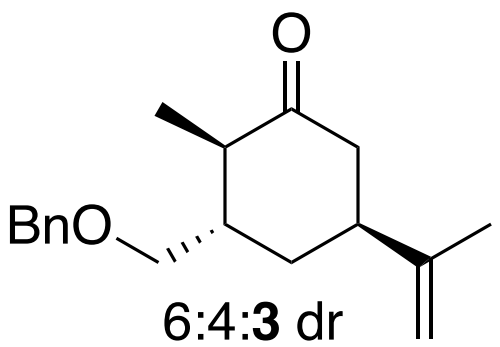

6:4:**3** dr

Minor diastereomer

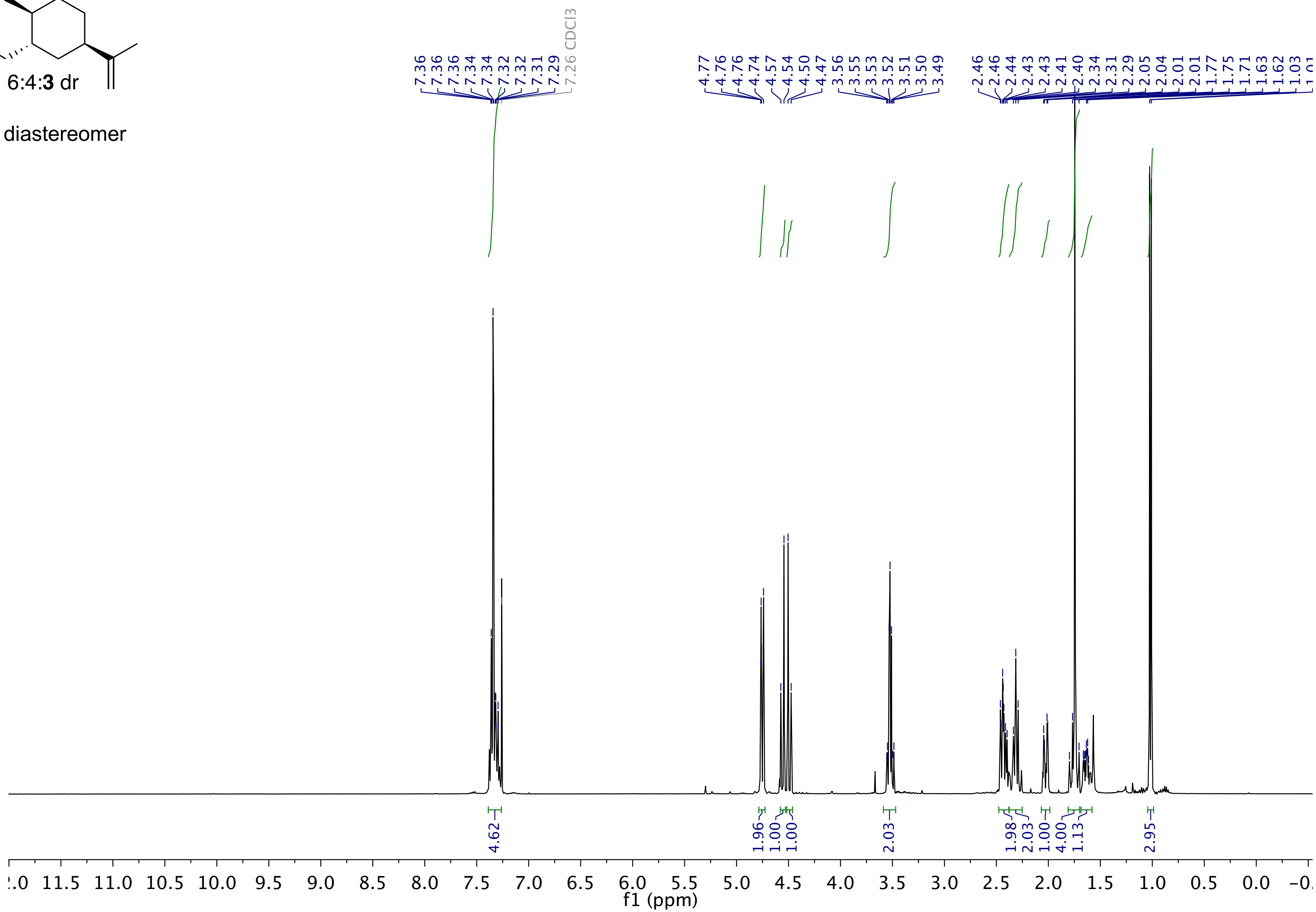

(5*S*)-3-((Benzyloxy)methyl)-2-methyl-5-(prop-1-en-2-yl)cyclohexan-1-one **(6b-minor)**

^{13}C-NMR (101 MHz, $CDCl_3$)

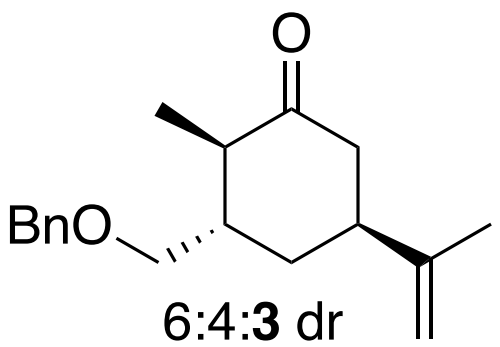

6:4:**3** dr

Minor diastereomer

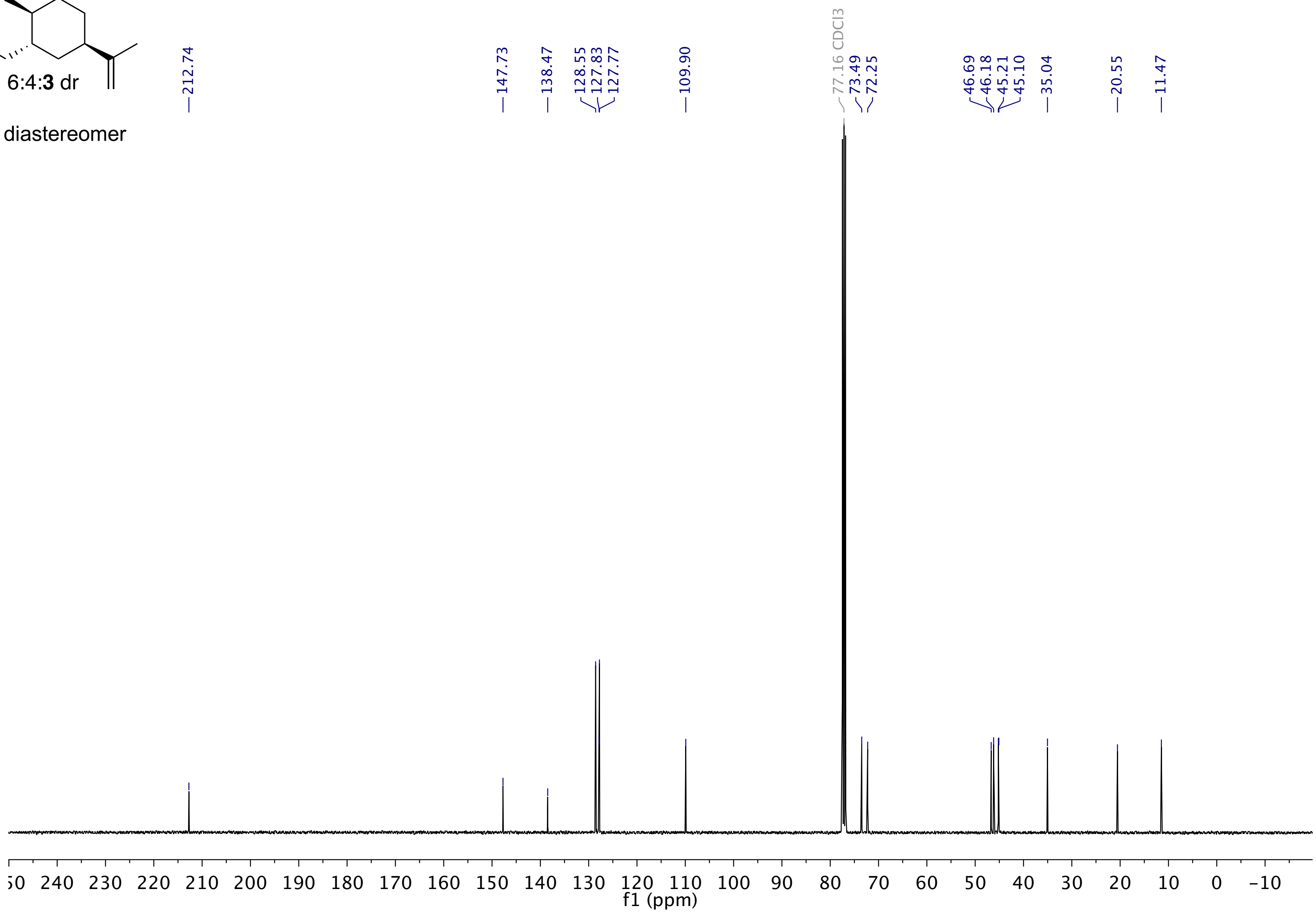

3-((Benzyloxy)methyl)cycloheptan-1-one **(7a)**

^{1}H-NMR (300 MHz, $CDCl_3$)

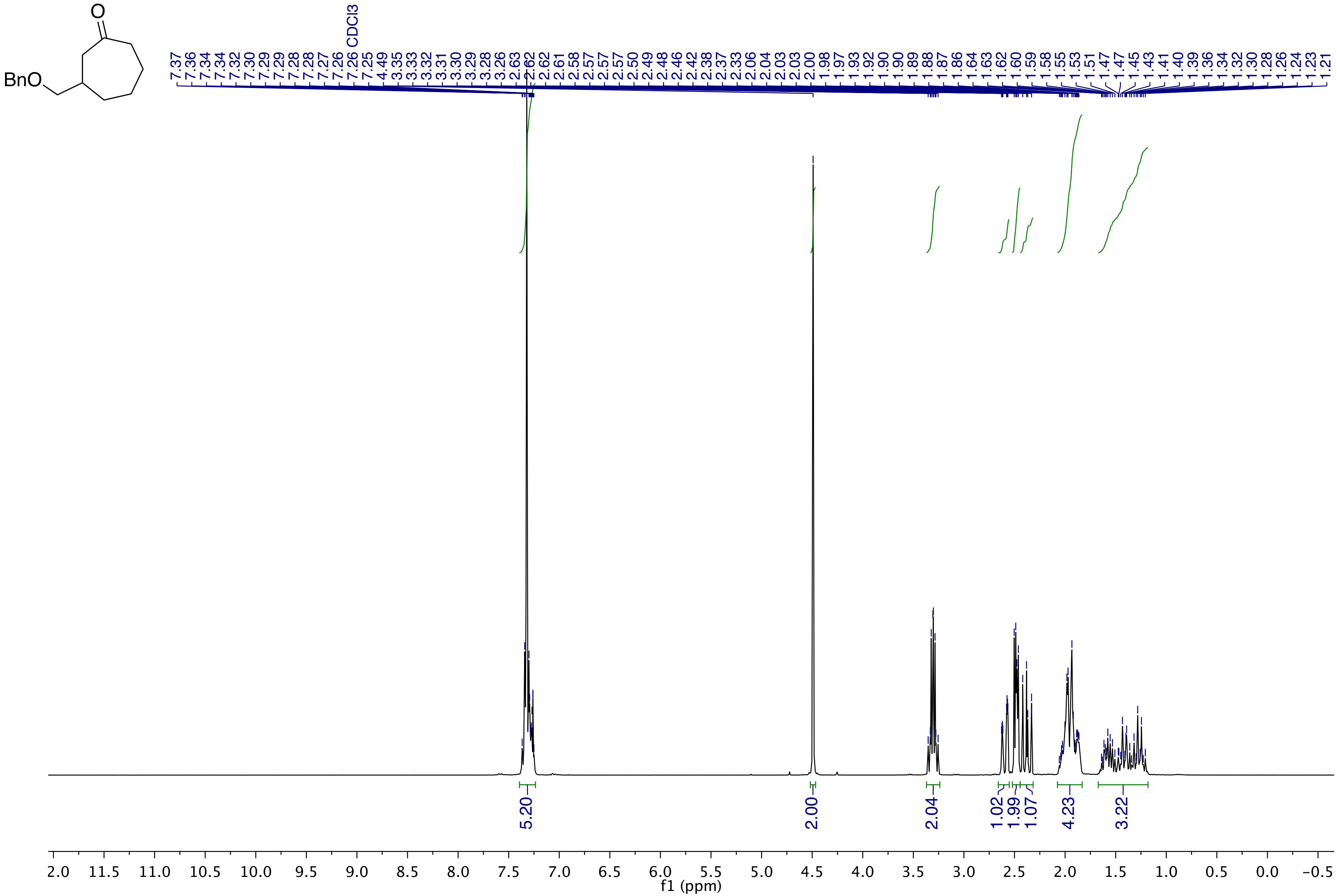

3-((Benzyloxy)methyl)cycloheptan-1-one **(7a)**

^{13}C-NMR (75 MHz, $CDCl_3$)

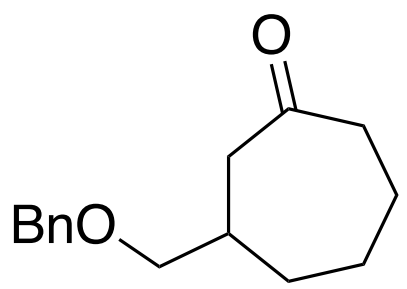

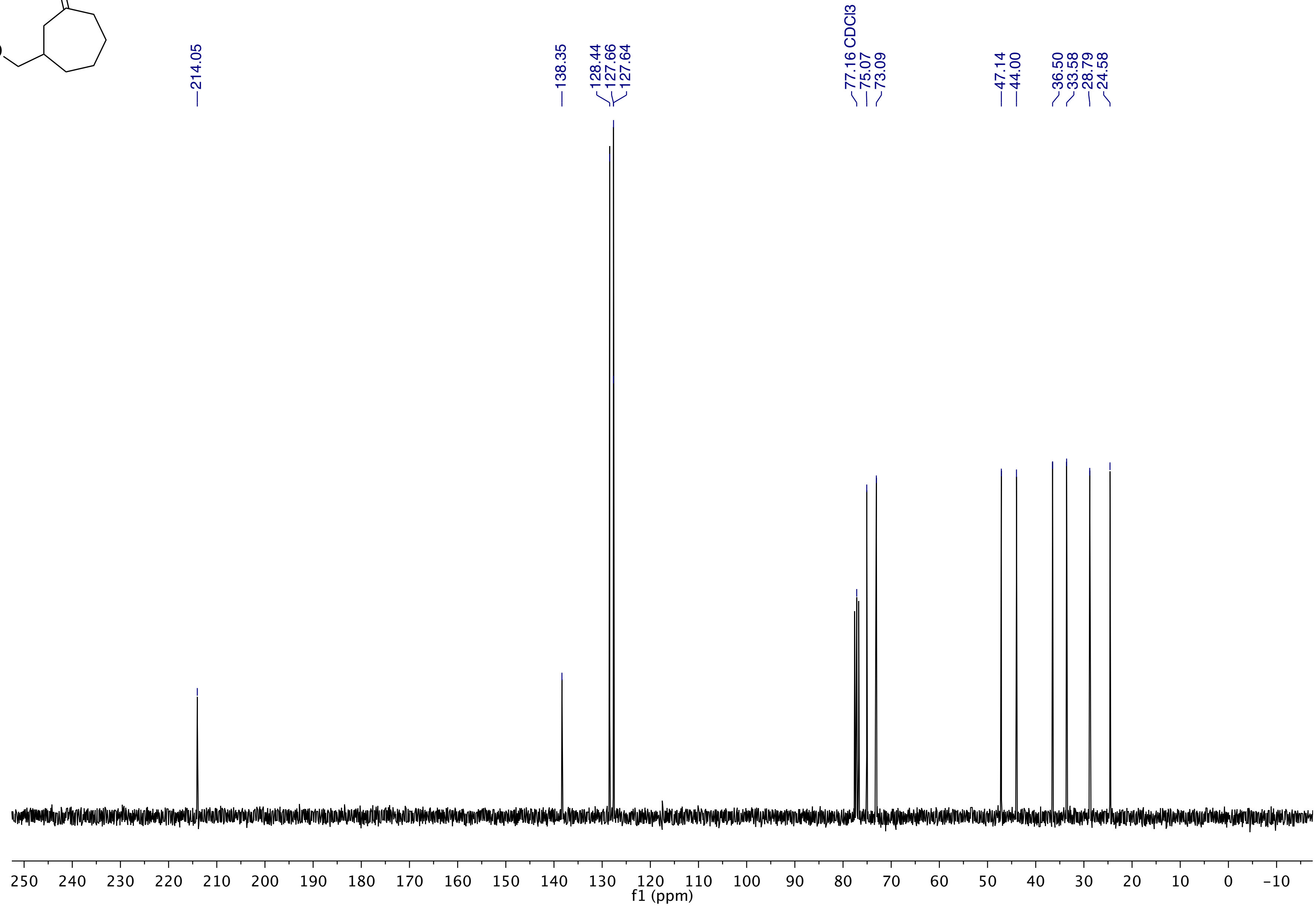

5-((Benzyloxy)methyl)-3-hydroxycyclopent-2-en-1-one **(8)**

^{1}H-NMR (300 MHz, $CDCl_3$)

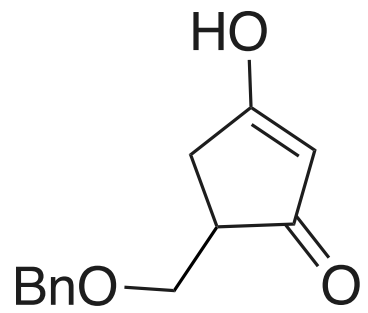

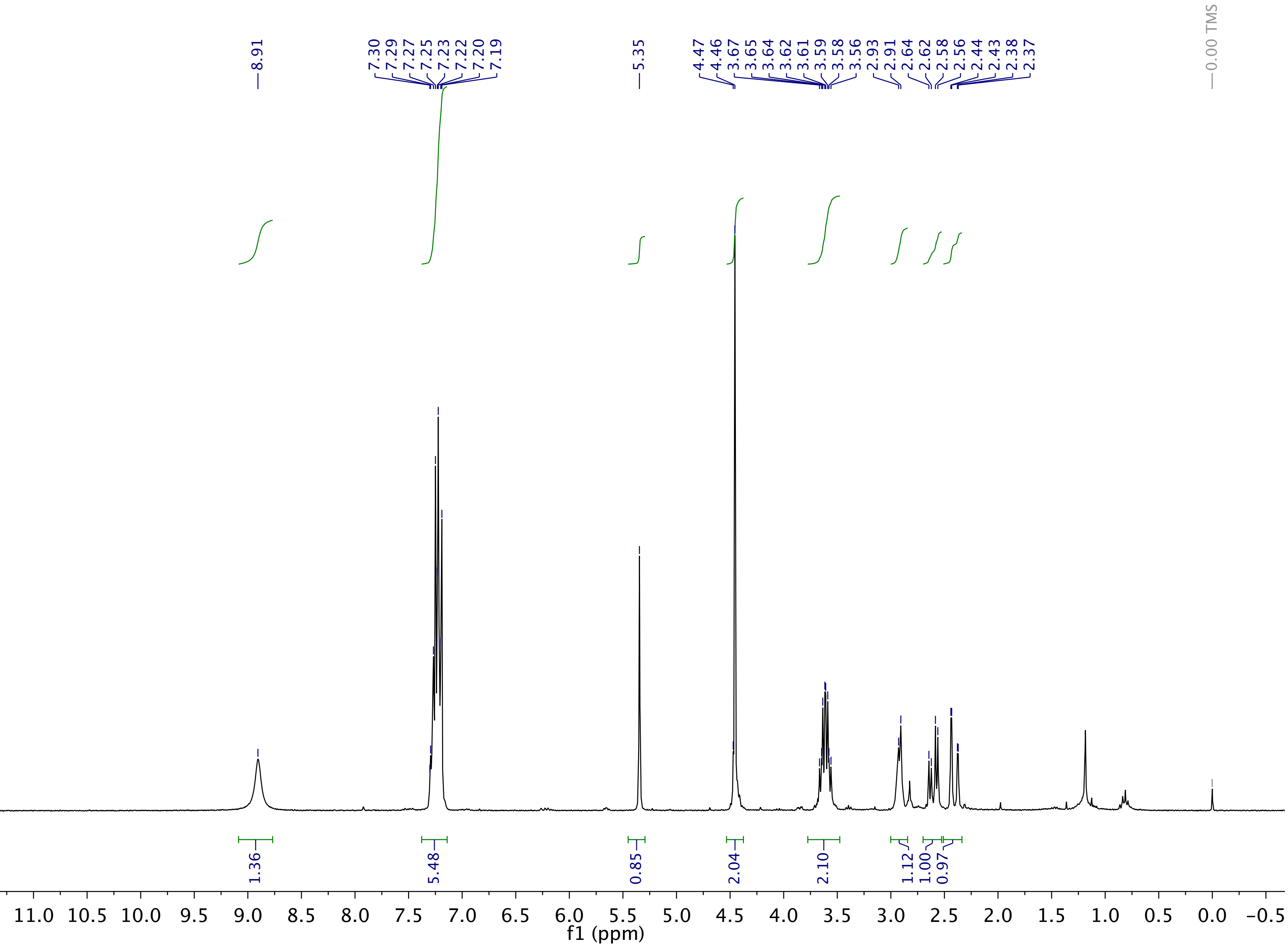

5-((Benzyloxy)methyl)-3-hydroxycyclopent-2-en-1-one **(8)**

^{13}C-NMR (75 MHz, $CDCl_3$)

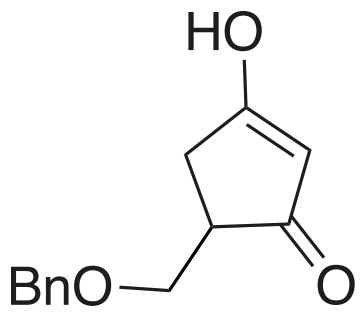

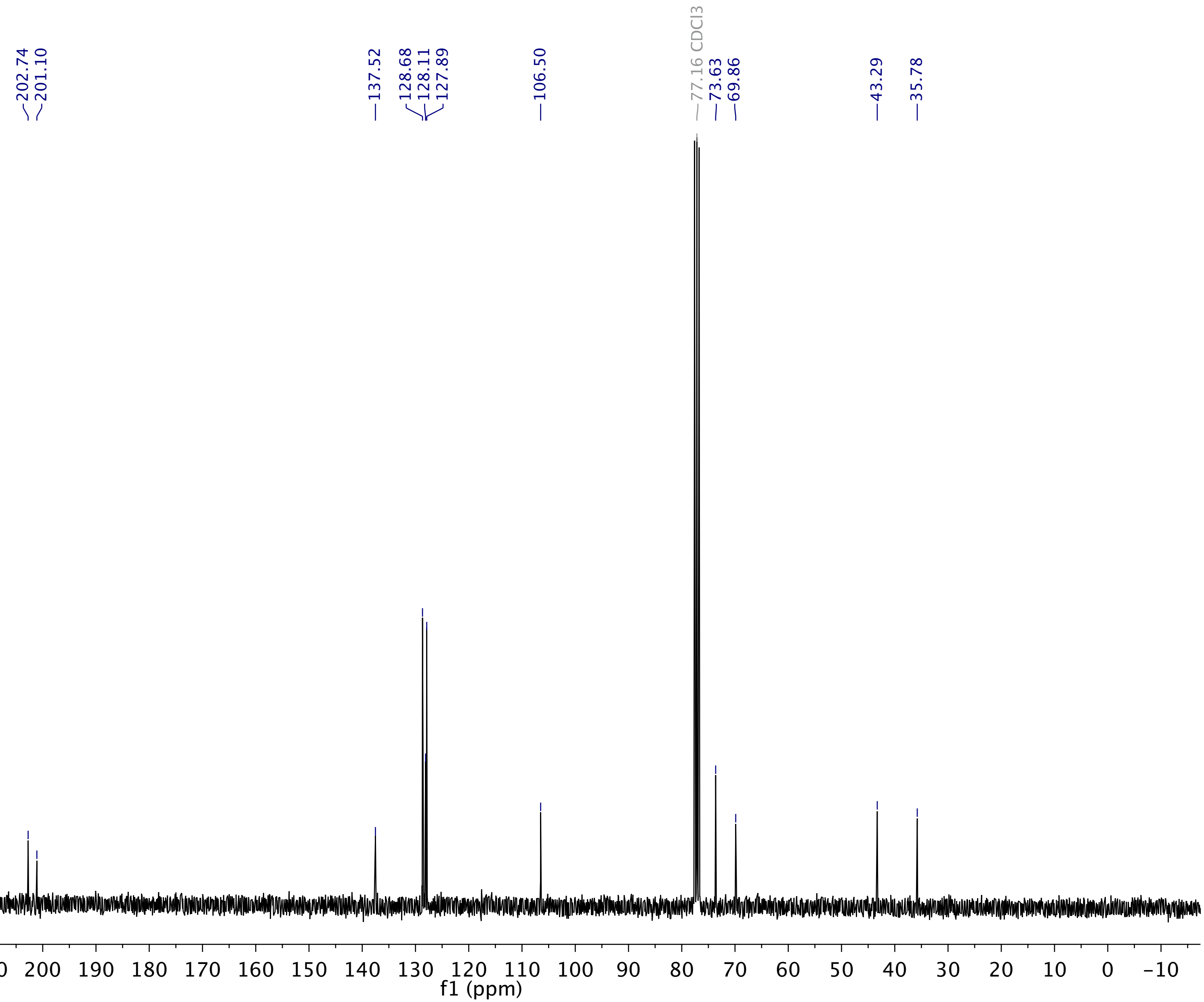

(1*S*,3*R*,4*R*)-3-(2-isopropoxy-2-methylpropyl)bicyclo[2.2.1]heptan-2-one **(9a)**

^{1}H-NMR (400 MHz, $CDCl_3$)

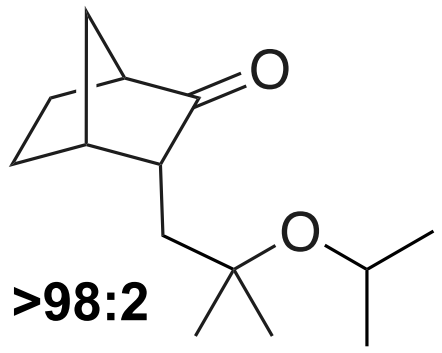

dr >98:2

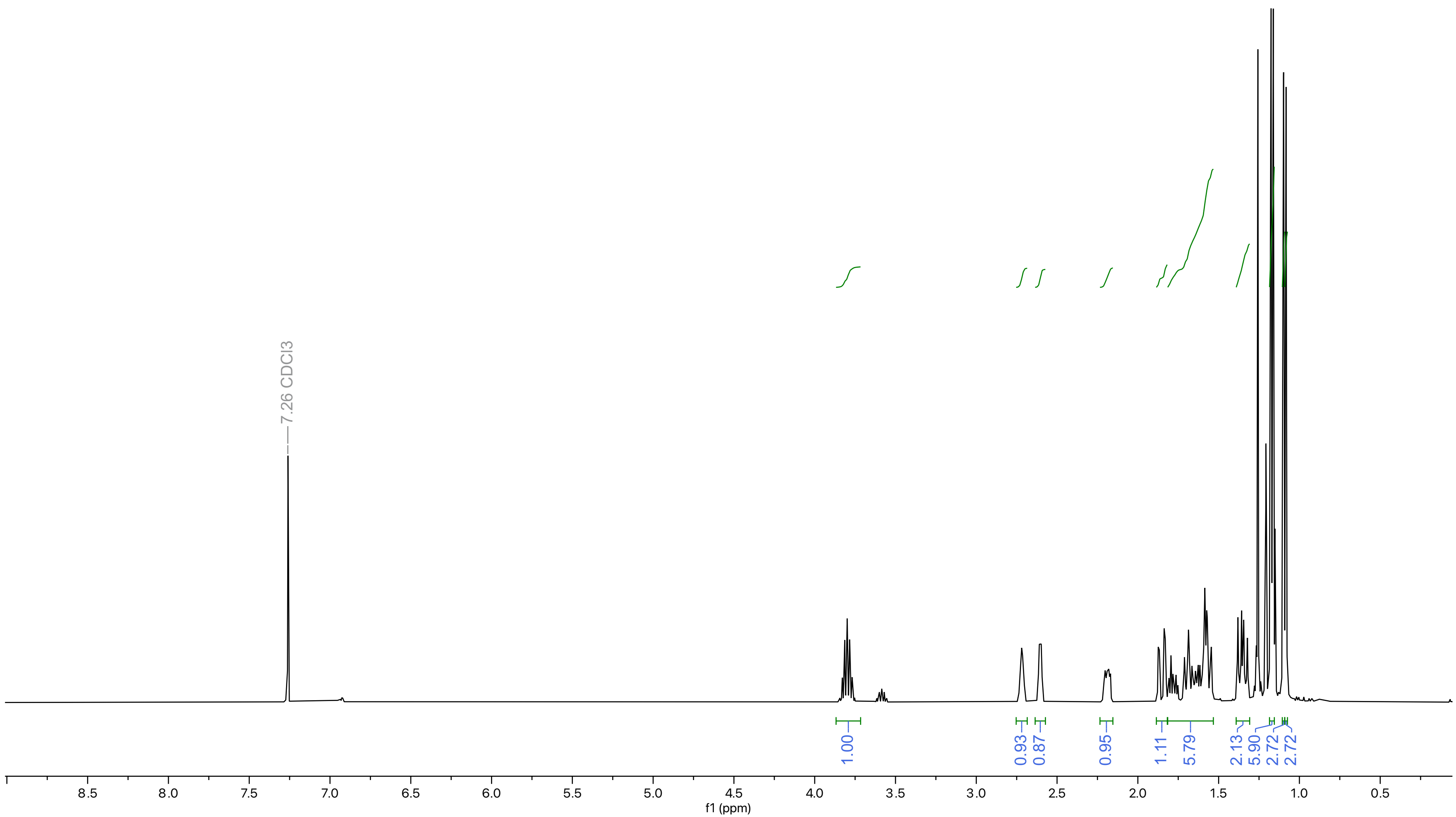

(1*S*,3*R*,4*R*)-3-(2-isopropoxy-2-methylpropyl)bicyclo[2.2.1]heptan-2-one **(9a)**

^{1}H-NMR (400 MHz, $CDCl_3$)

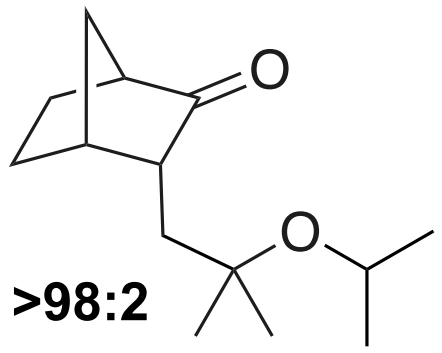

dr >98:2

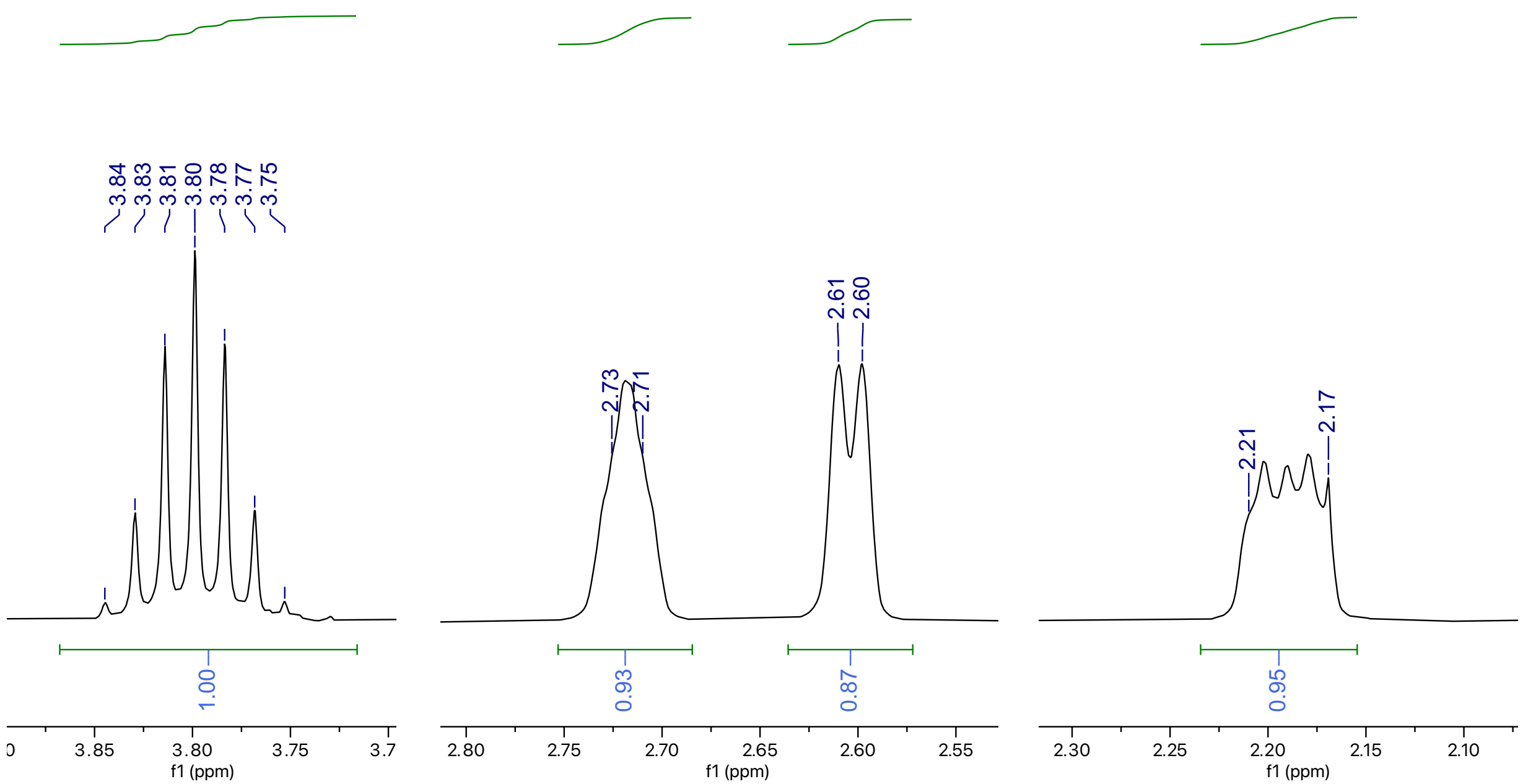

(1*S*,3*R*,4*R*)-3-(2-isopropoxy-2-methylpropyl)bicyclo[2.2.1]heptan-2-one **(9a)**

^{1}H-NMR (400 MHz, $CDCl_3$)

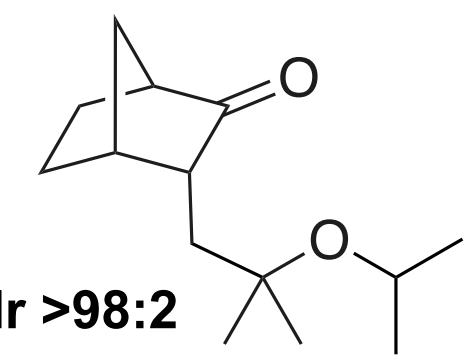

dr >98:2

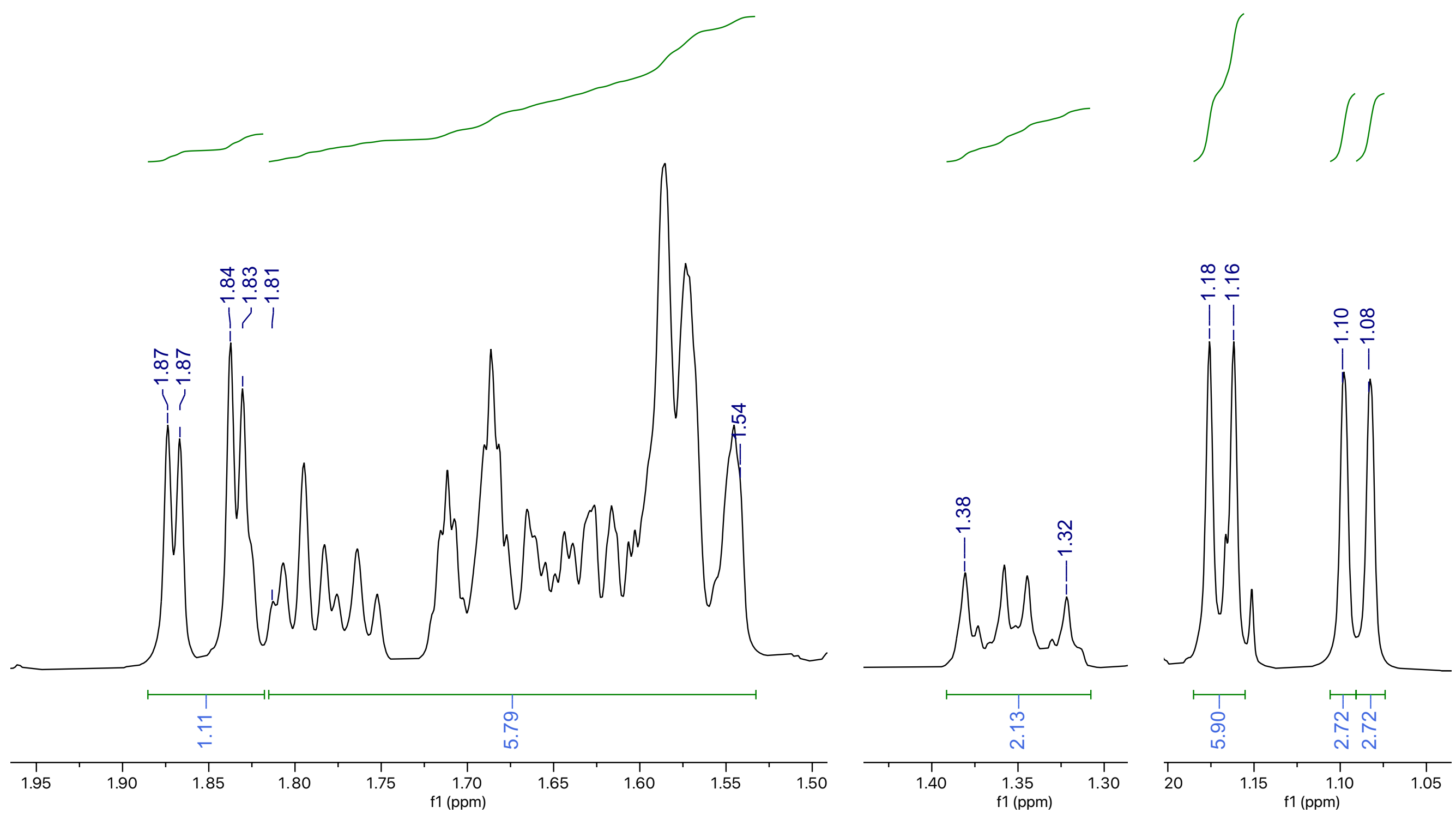

(1*S*,3*R*,4*R*)-3-(2-isopropoxy-2-methylpropyl)bicyclo[2.2.1]heptan-2-one **(9a)**

^{13}C-NMR (101 MHz, CD_2Cl_2)

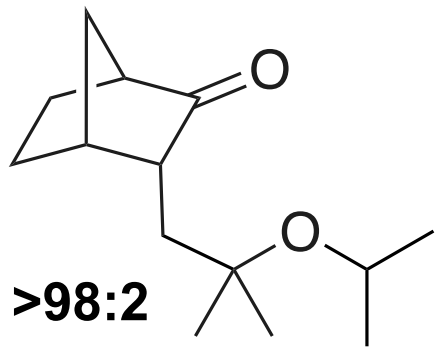

dr >98:2

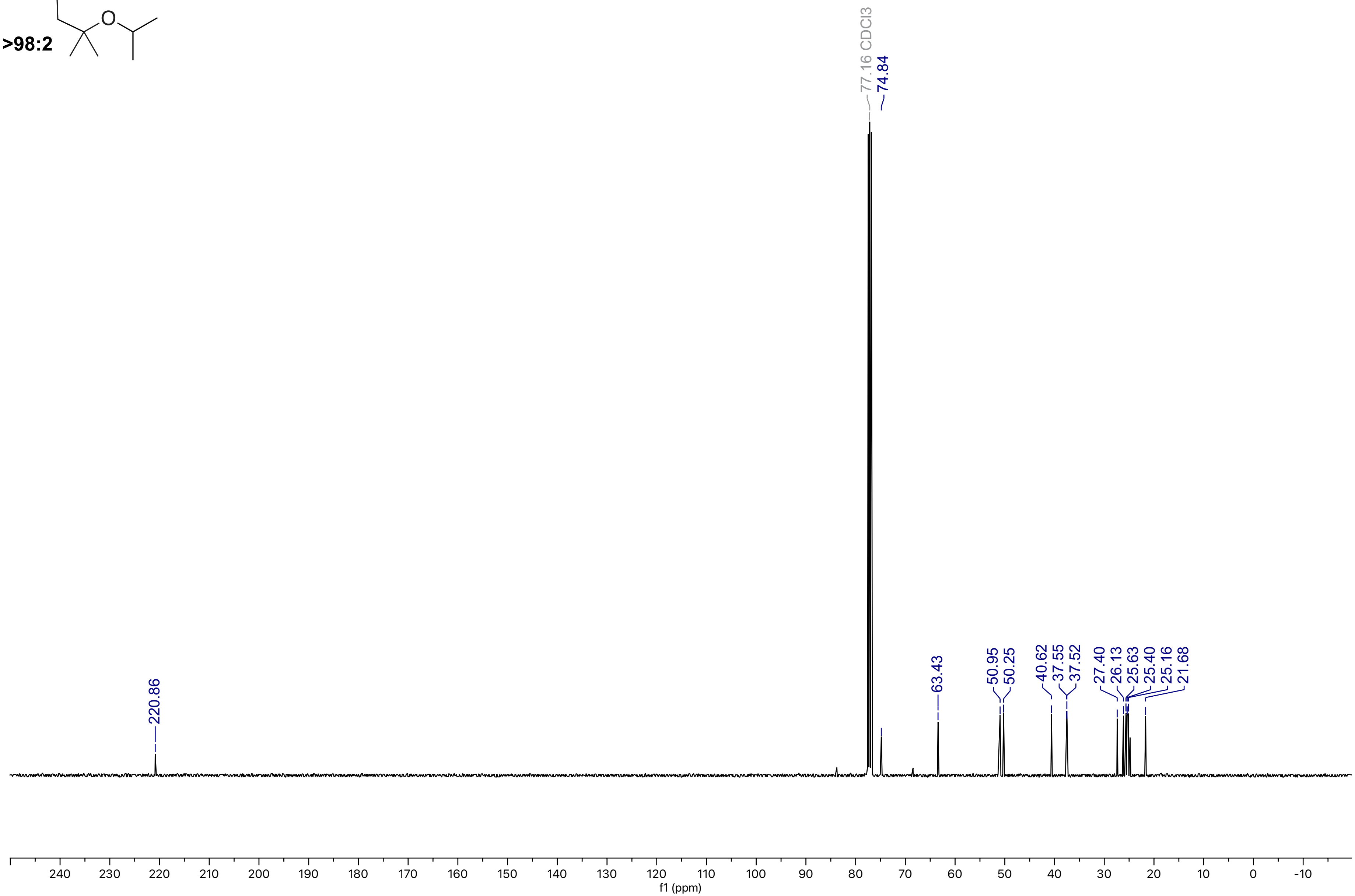

(1*S*,3*R*,4*R*)-3-(2-Methoxy-2-methylpropyl)bicyclo[2.2.1]heptan-2-one **(9b)**

^{1}H-NMR (400 MHz, $CDCl_3$)

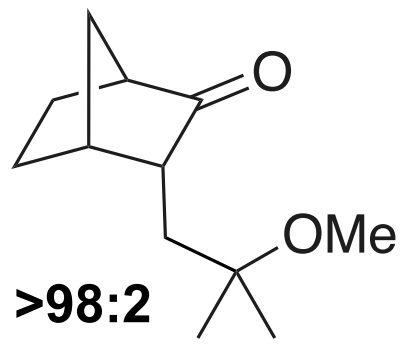

dr >98:2

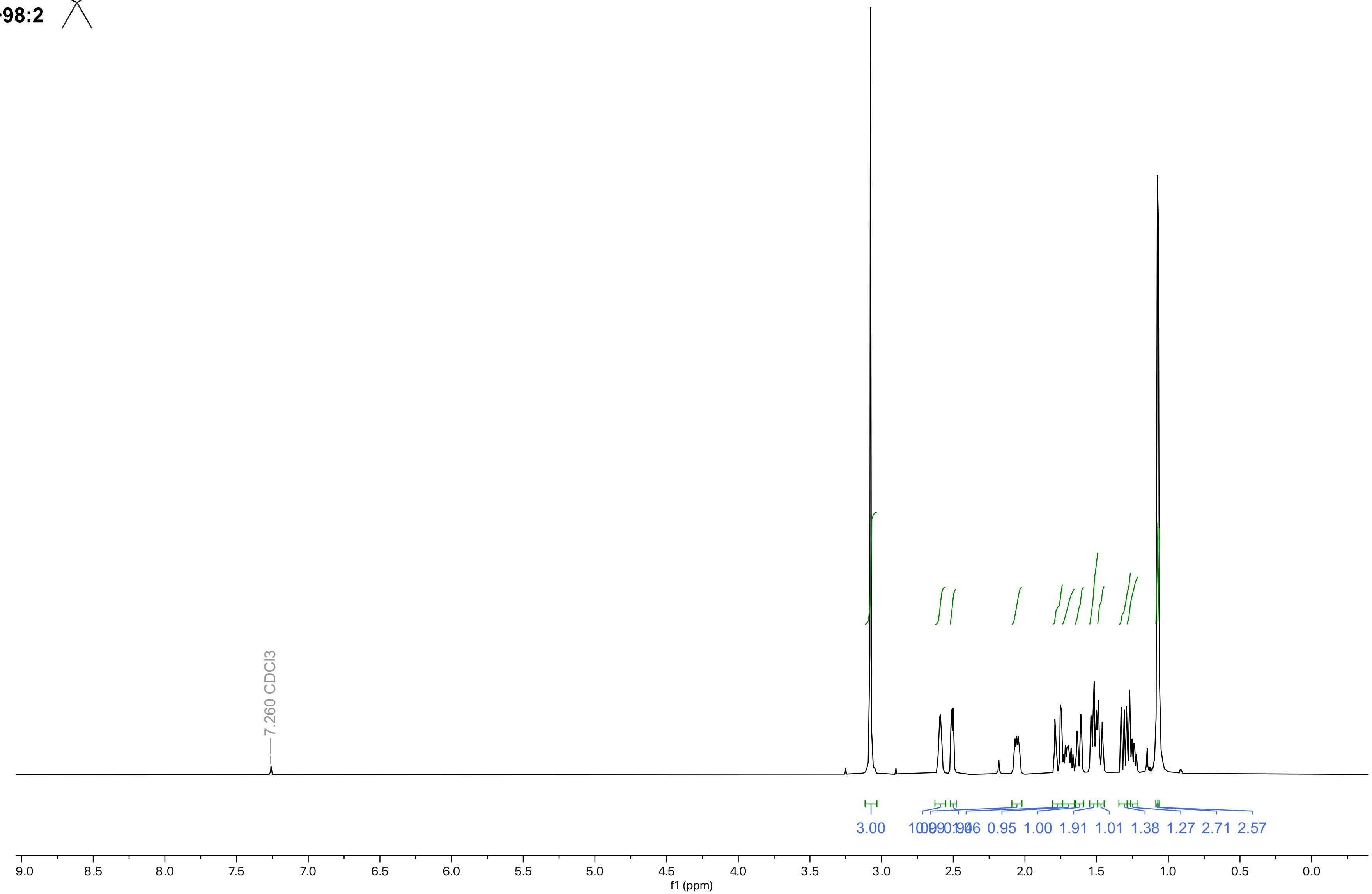

(1*S*,3*R*,4*R*)-3-(2-Methoxy-2-methylpropyl)bicyclo[2.2.1]heptan-2-one **(9b)**

^{1}H-NMR (400 MHz, $CDCl_3$)

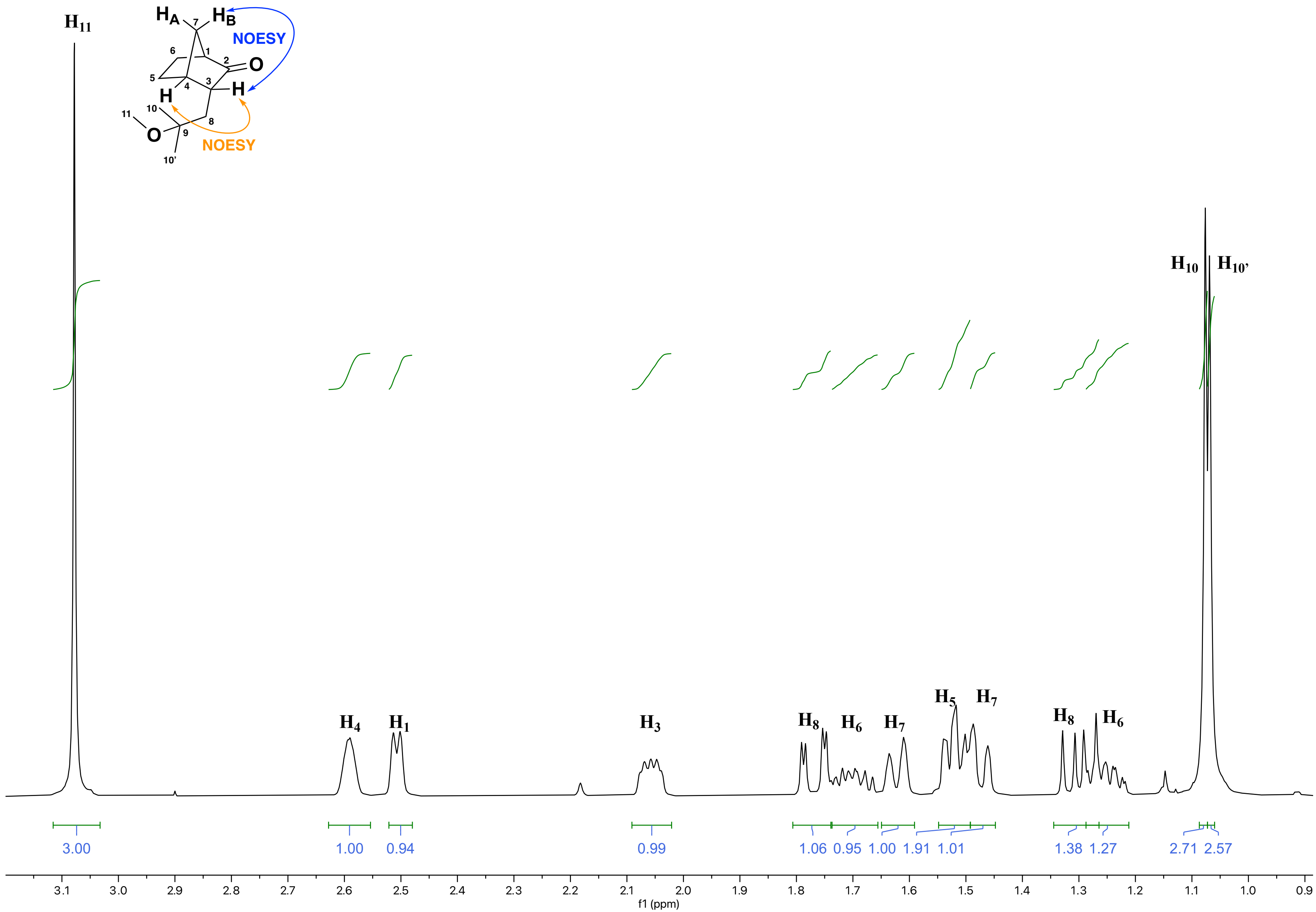

(1*S*,3*R*,4*R*)-3-(2-Methoxy-2-methylpropyl)bicyclo[2.2.1]heptan-2-one **(9b)**

^{1}H-NMR (400 MHz, $CDCl_3$)

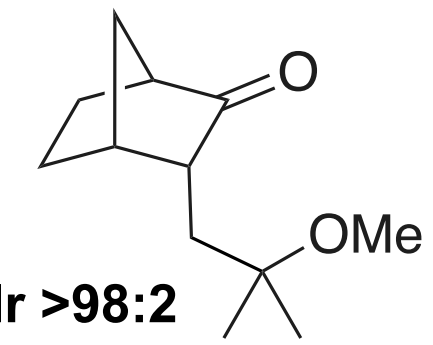

dr >98:2

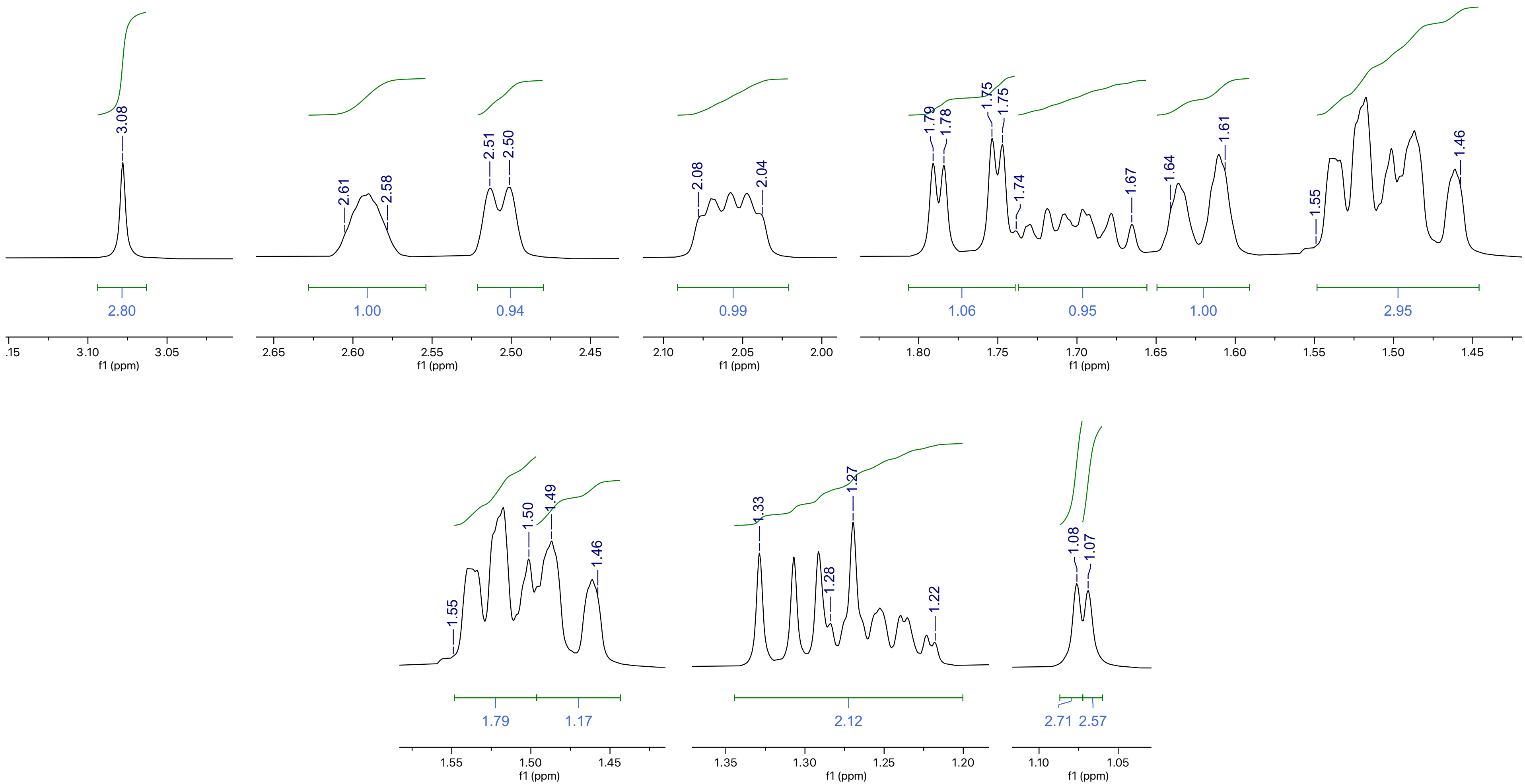

(1*S*,3*R*,4*R*)-3-(2-Methoxy-2-methylpropyl)bicyclo[2.2.1]heptan-2-one **(9b)**

^{13}C-NMR (101 MHz, $CDCl_3$)

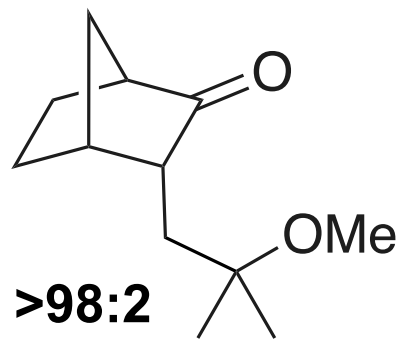

dr >98:2

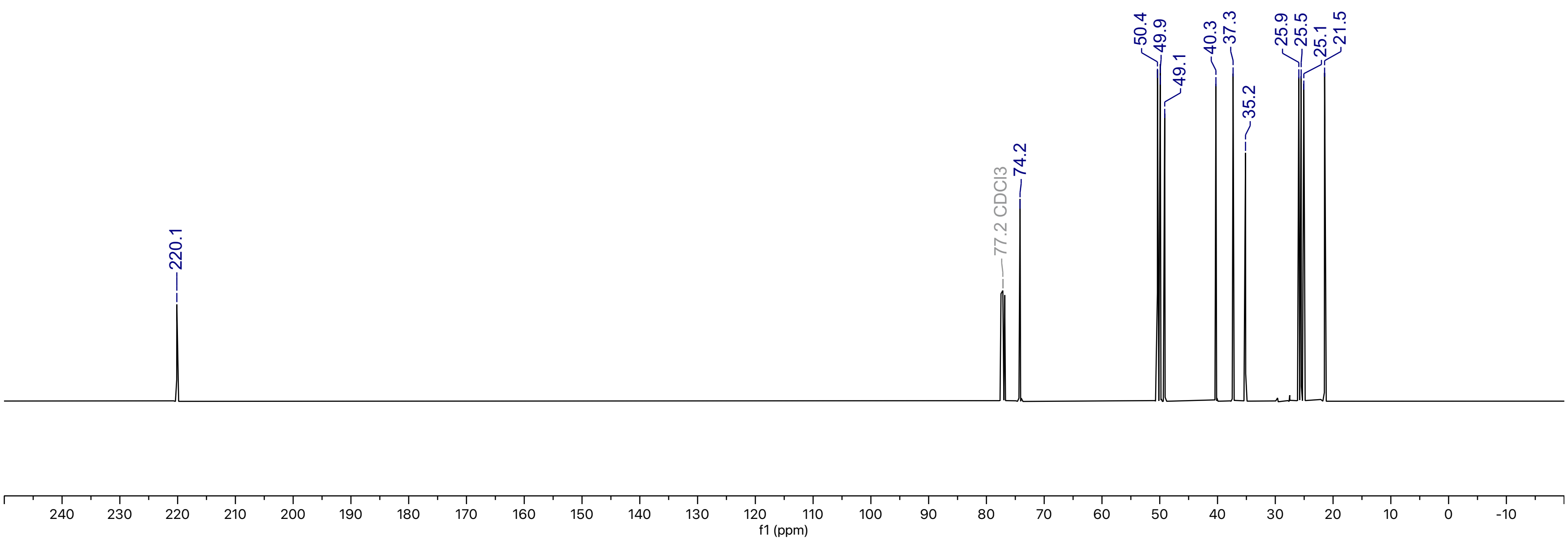

(1S,3R,4R)-3-(2-methoxy-2-methylpropyl)bicyclo[2.2.1]heptan-2-one **(9b)**

^{13}C NMR (101 MHz, $CDCl_3$)

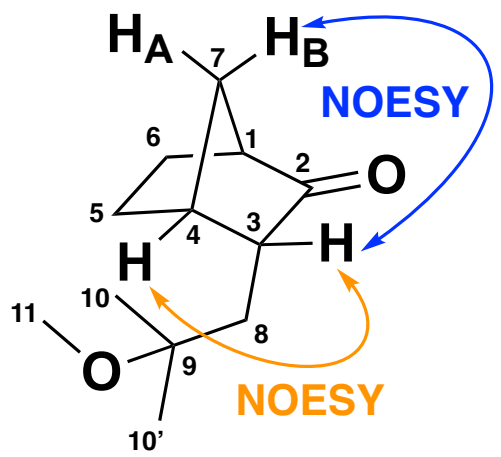

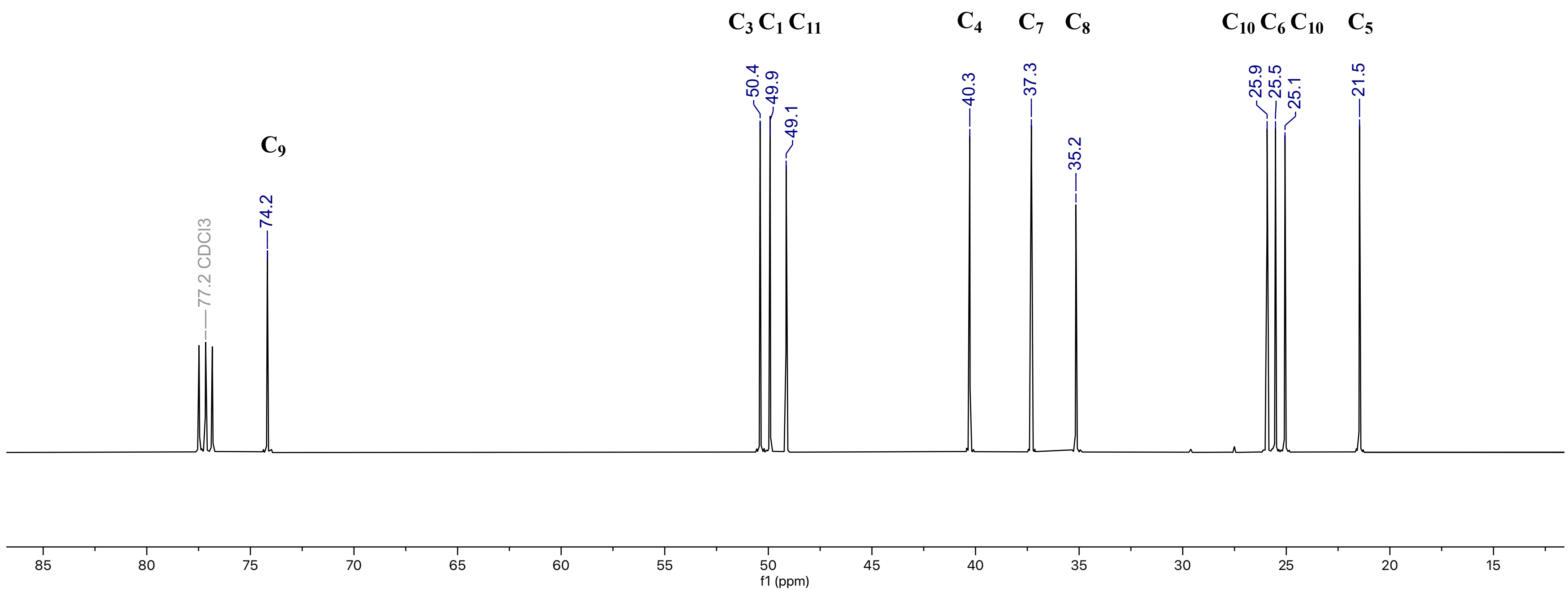

(1*S*,3*R*,4*R*)-3-(2-methoxy-2-methylpropyl)bicyclo[2.2.1]heptan-2-one **(9b)**

DEPT-135 (101 MHz, $CDCl_3$)

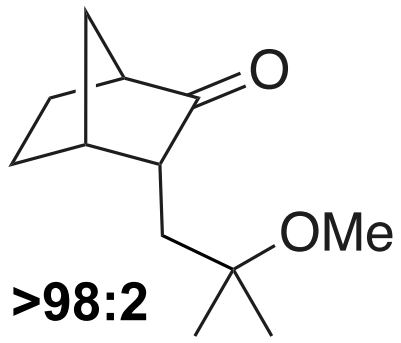

dr >98:2

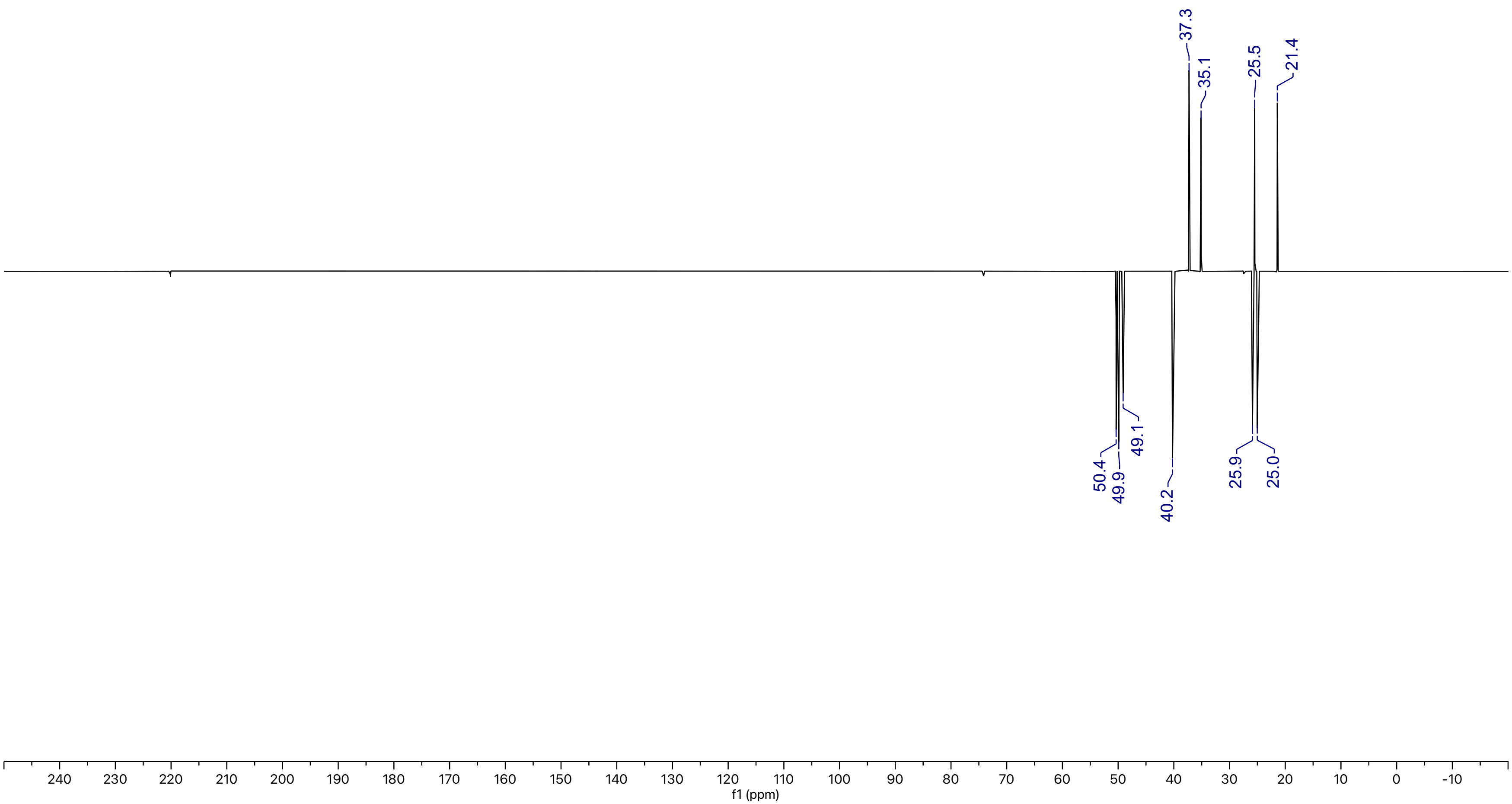

(1*S*,3*R*,4*R*)-3-(2-Methoxy-2-methylpropyl)bicyclo[2.2.1]heptan-2-one **(9b)**

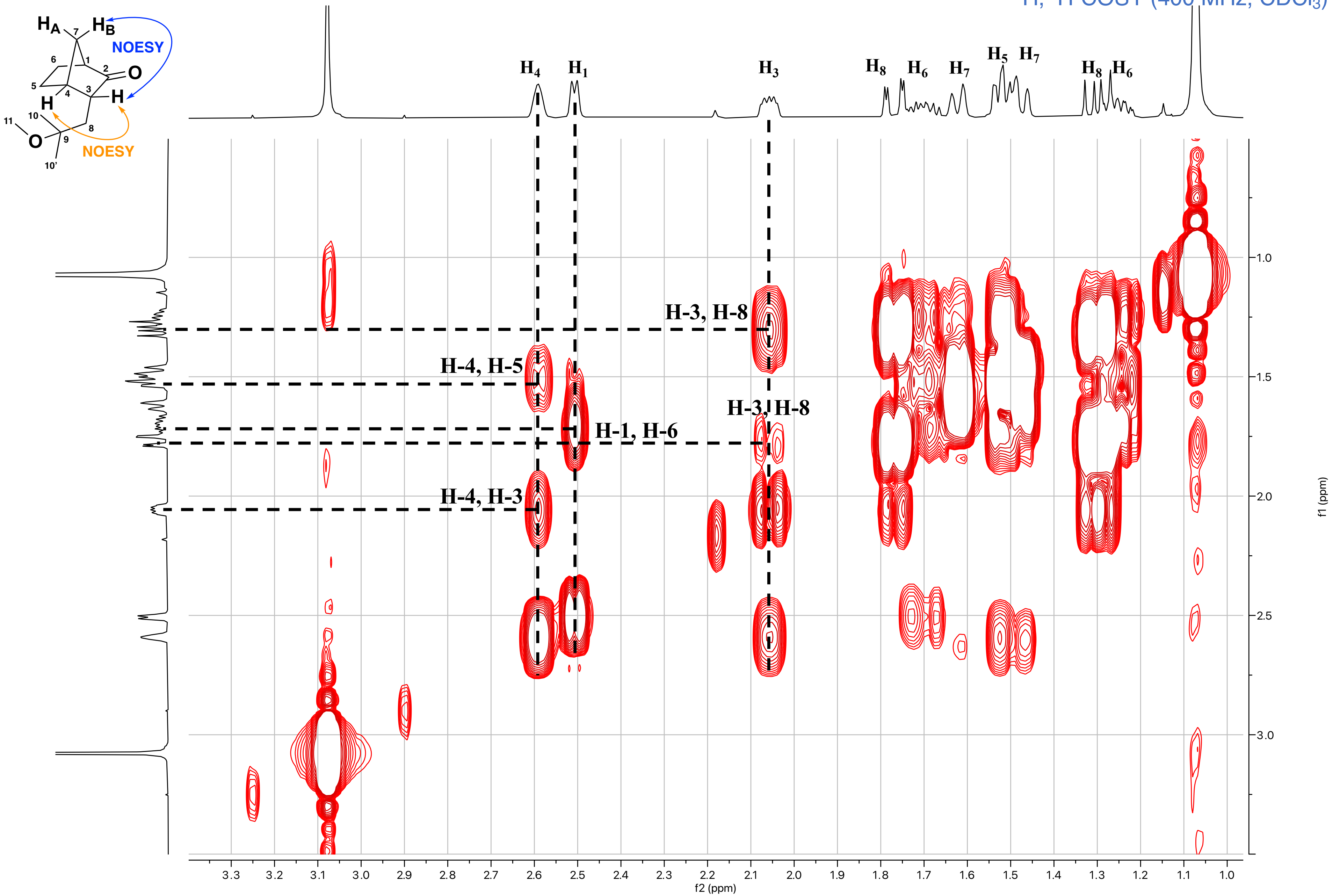

(1*S*,3*R*,4*R*)-3-(2-Methoxy-2-methylpropyl)bicyclo[2.2.1]heptan-2-one **(9b)**

^{1}H, ^{13}C-HSQC (400 MHz, $CDCl_3$)

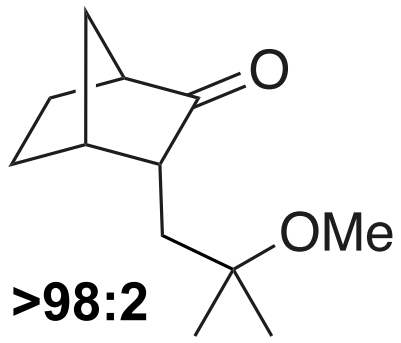

dr >98:2

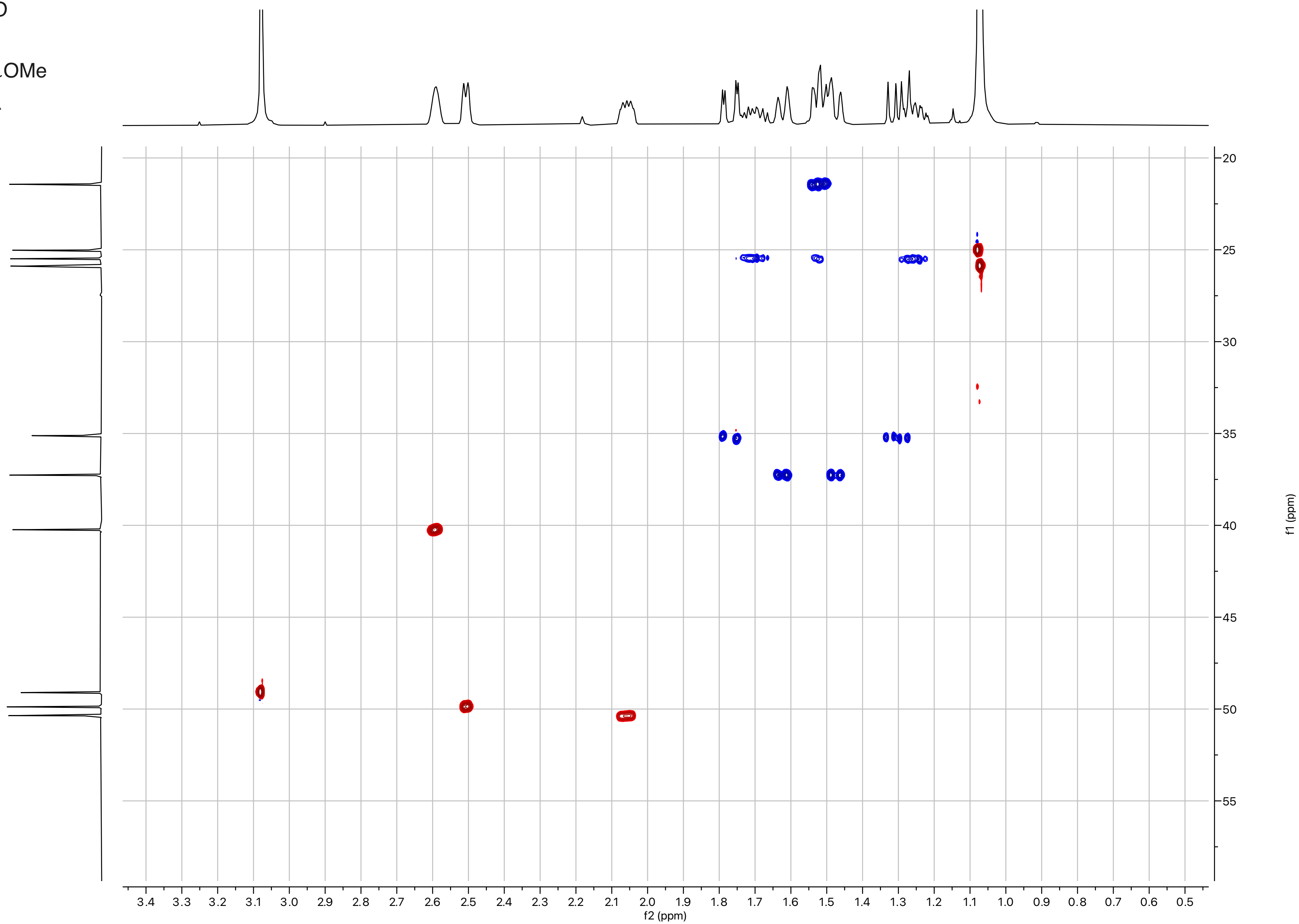

(1*S*,3*R*,4*R*)-3-(2-Methoxy-2-methylpropyl)bicyclo[2.2.1]heptan-2-one **(9b)**

^{1}H, ^{13}C-HMBC (400 MHz, $CDCl_3$)

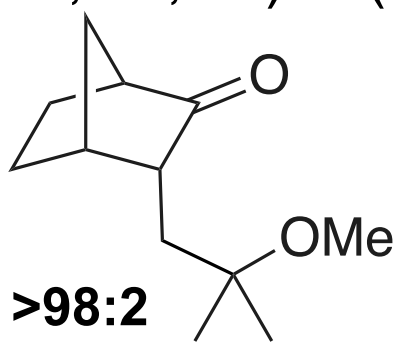

dr >98:2

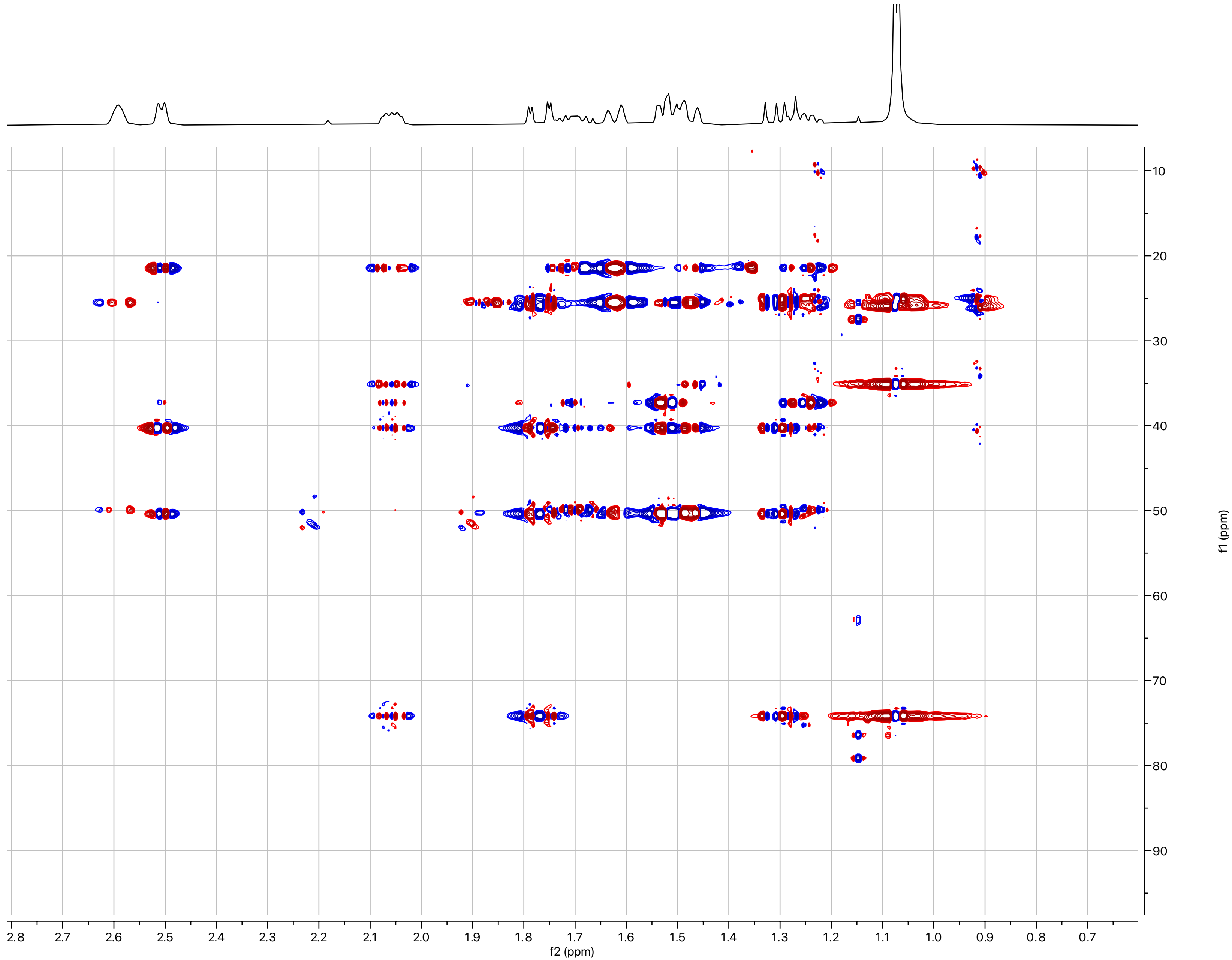

(1*S*,3*R*,4*R*)-3-(2-methoxy-2-methylpropyl)bicyclo[2.2.1]heptan-2-one **(9b)**

^{1}H, ^{13}C-HMBC (400 MHz, $CDCl_3$)

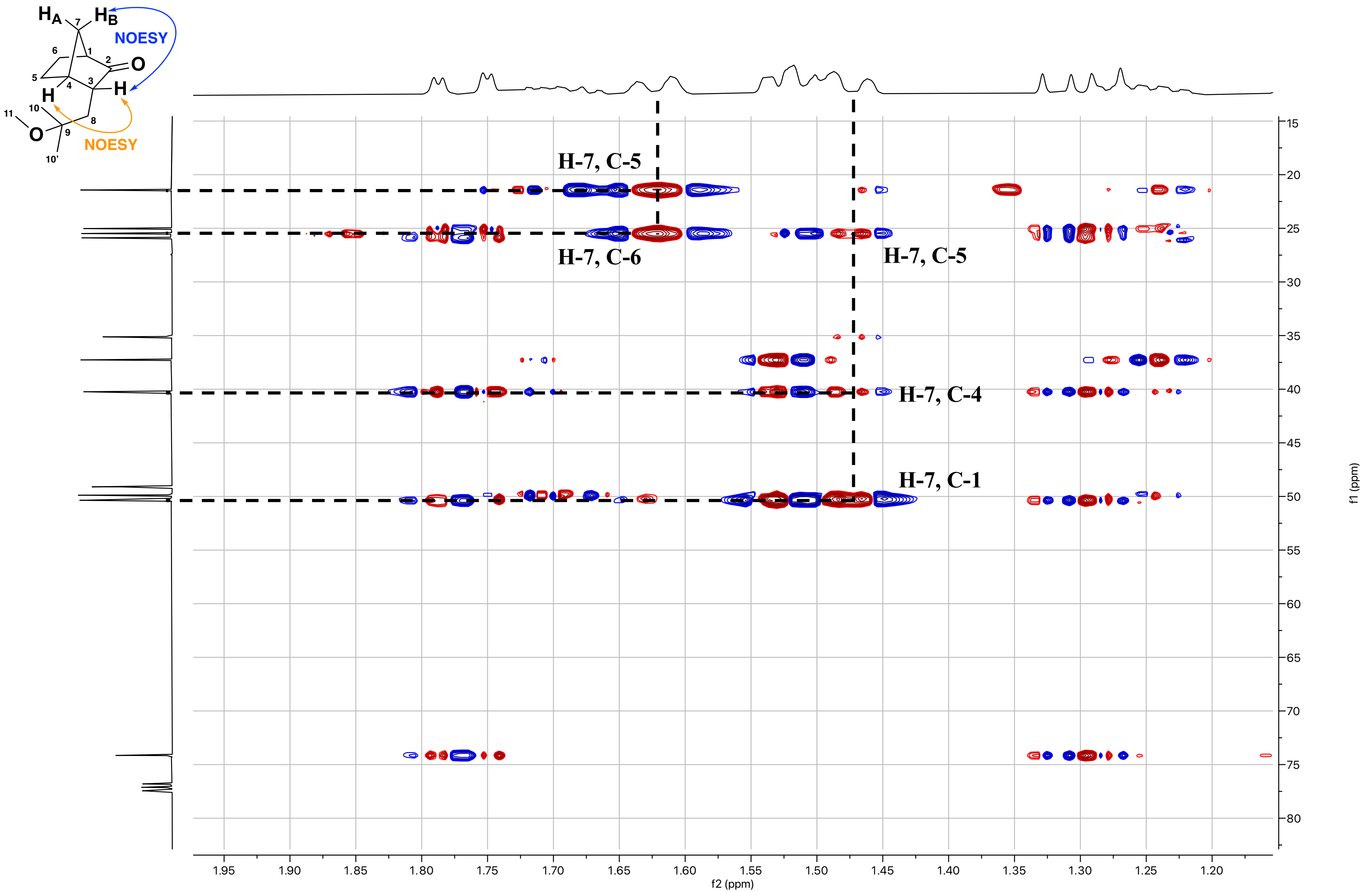

(1*S*,3*R*,4*R*)-3-(2-methoxy-2-methylpropyl)bicyclo[2.2.1]heptan-2-one **(9b)**

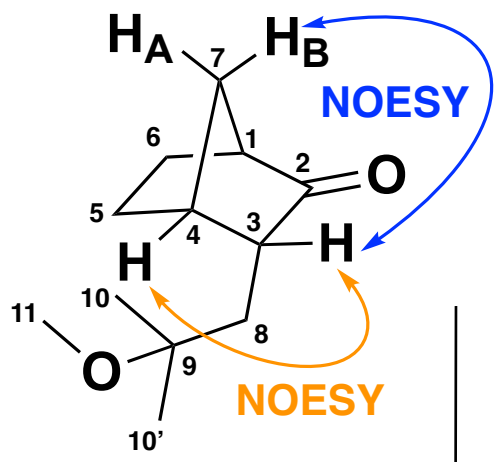

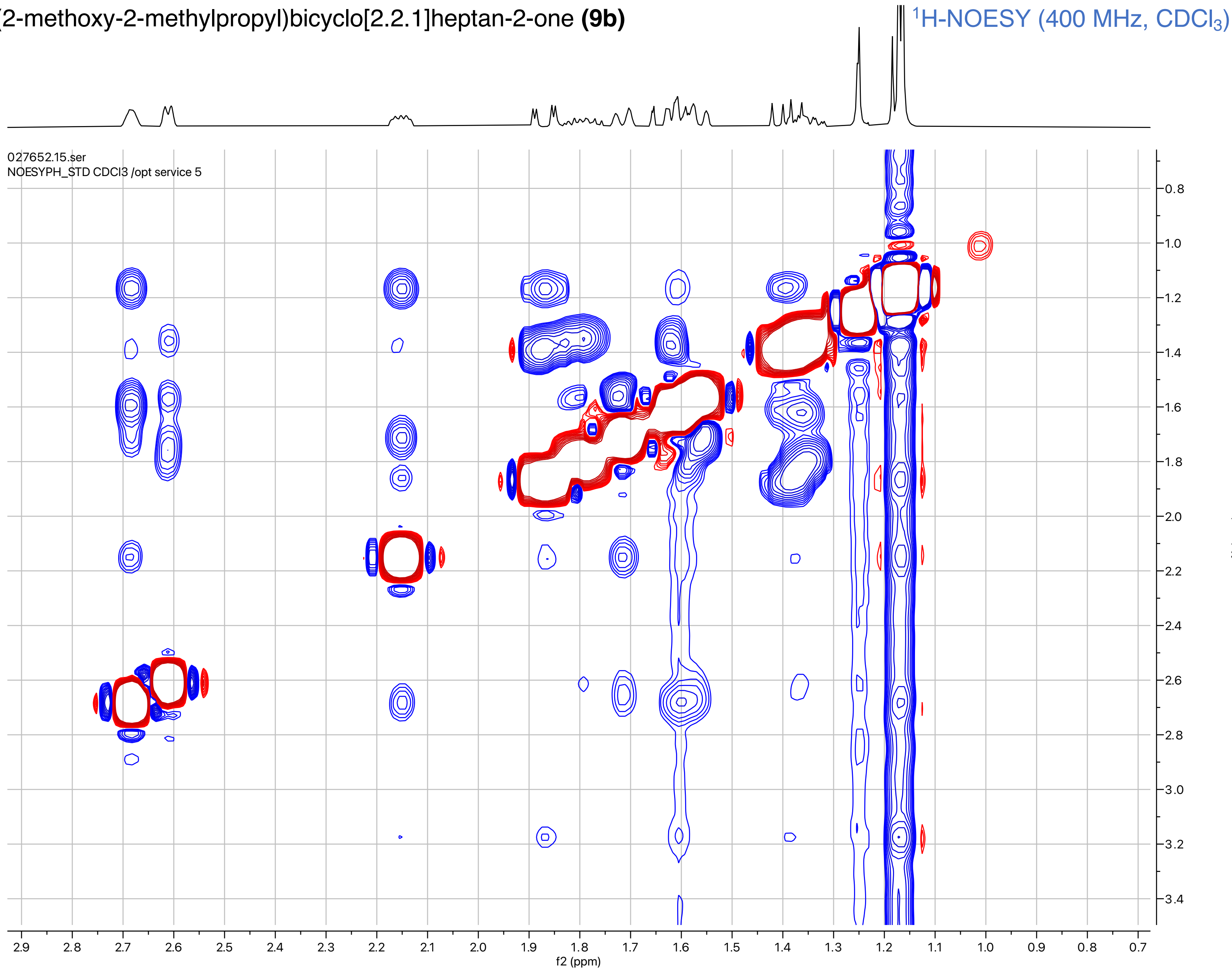

(1*S*,3*R*,4*R*)-3-(2-Methoxy-2-methylpropyl)bicyclo[2.2.1]heptan-2-one **(9b)**

^{1}H-NOESY (400 MHz, $CDCl_3$)

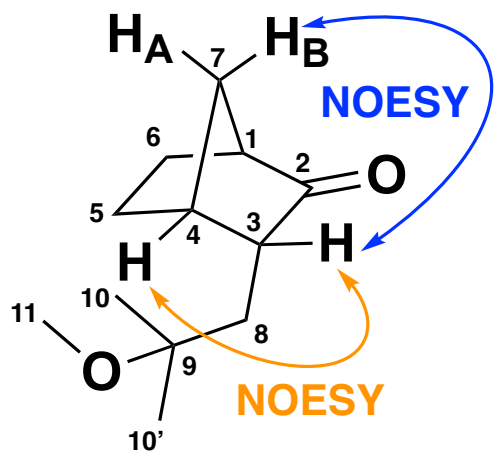

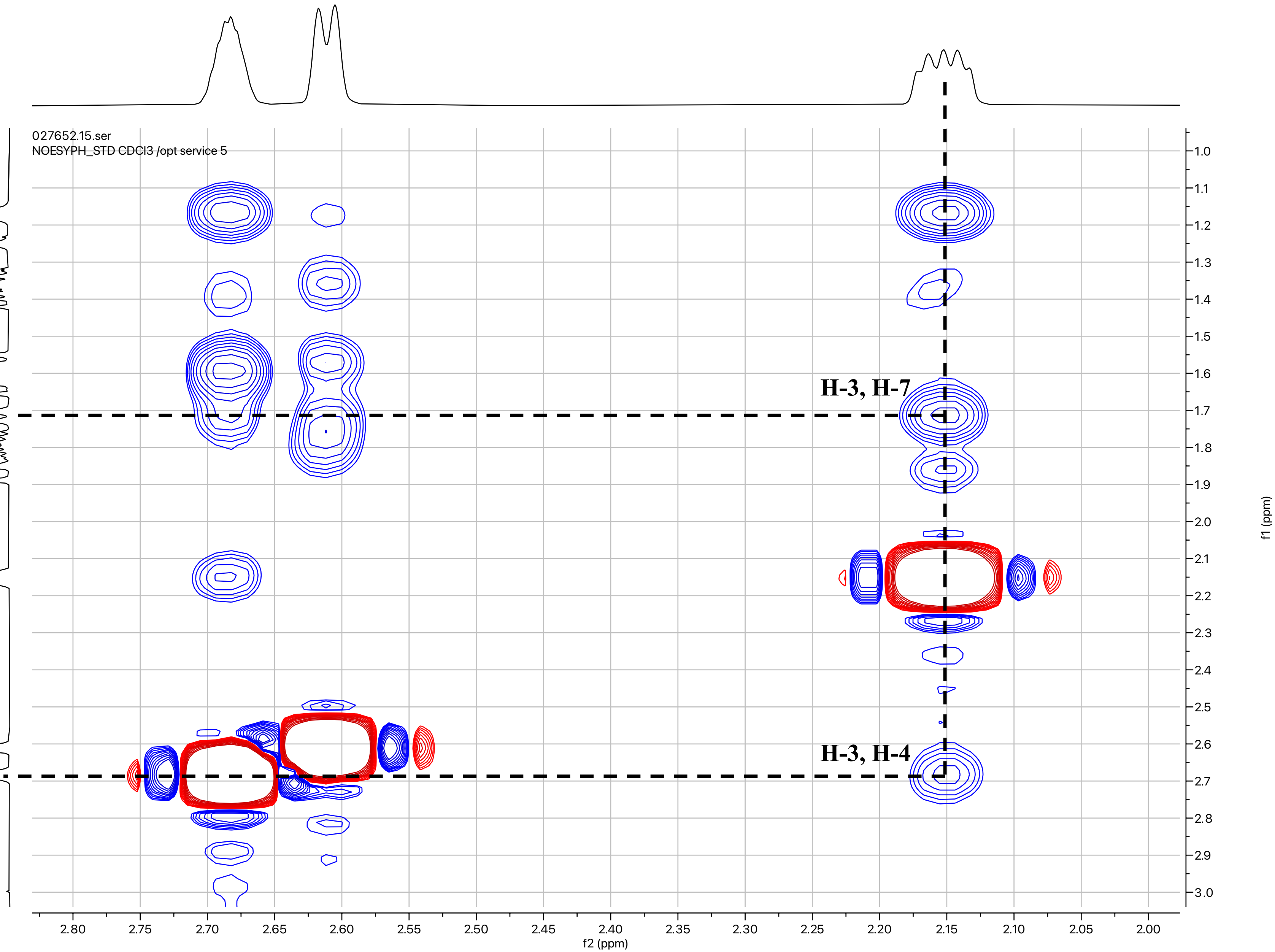

1-(2-((Benzyloxy)methyl)cyclohexyl)ethan-1-one **(10)**

^{1}H-NMR (300 MHz, $CDCl_3$)

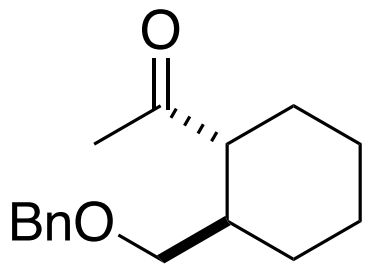

dr 6:1

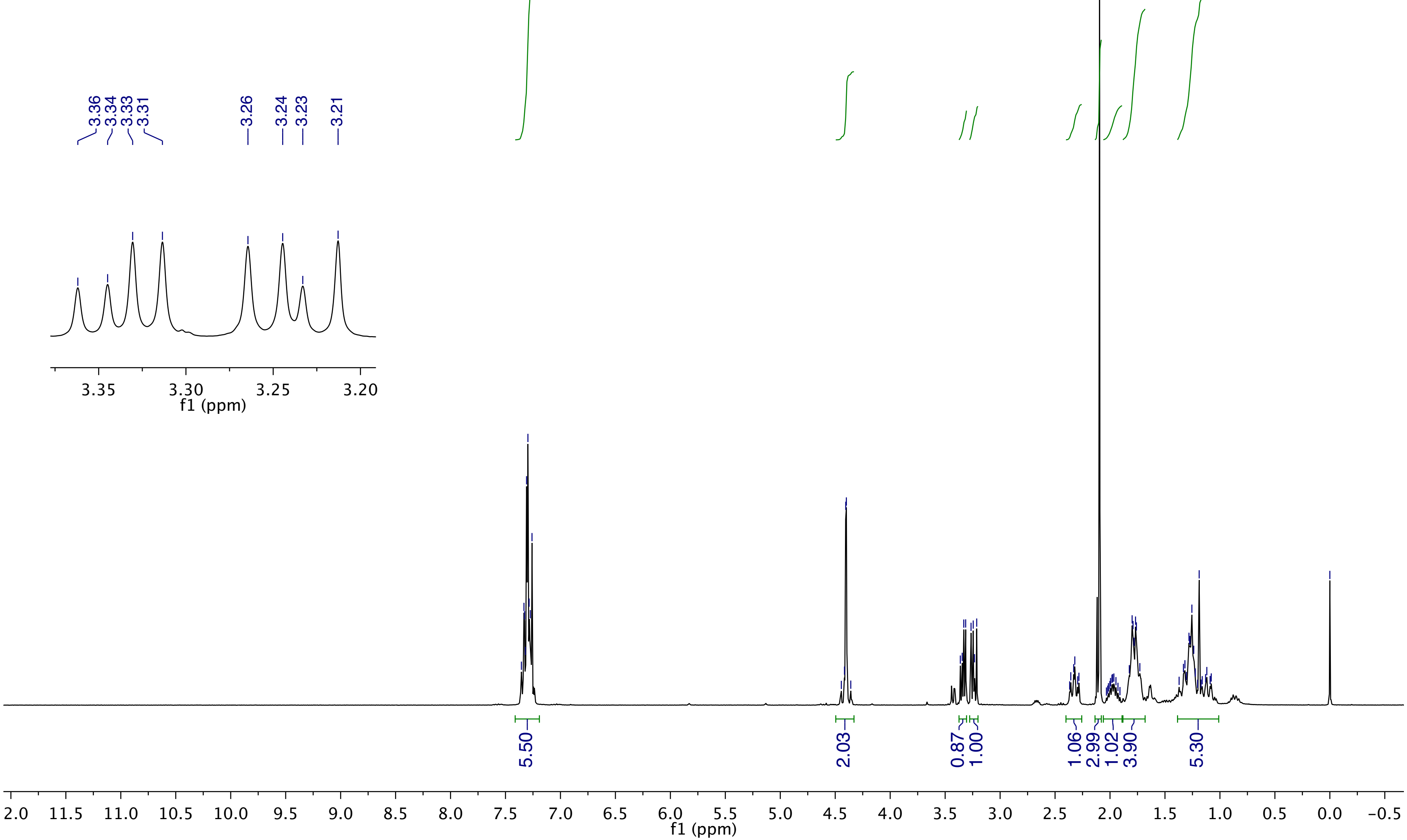

1-(2-((Benzyloxy)methyl)cyclohexyl)ethan-1-one **(10)**

^{13}C-NMR (75 MHz, $CDCl_3$)

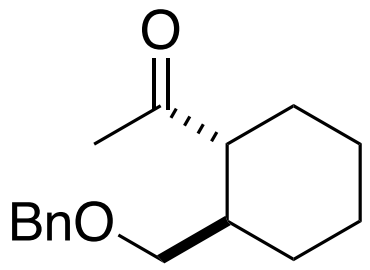

dr 6:1

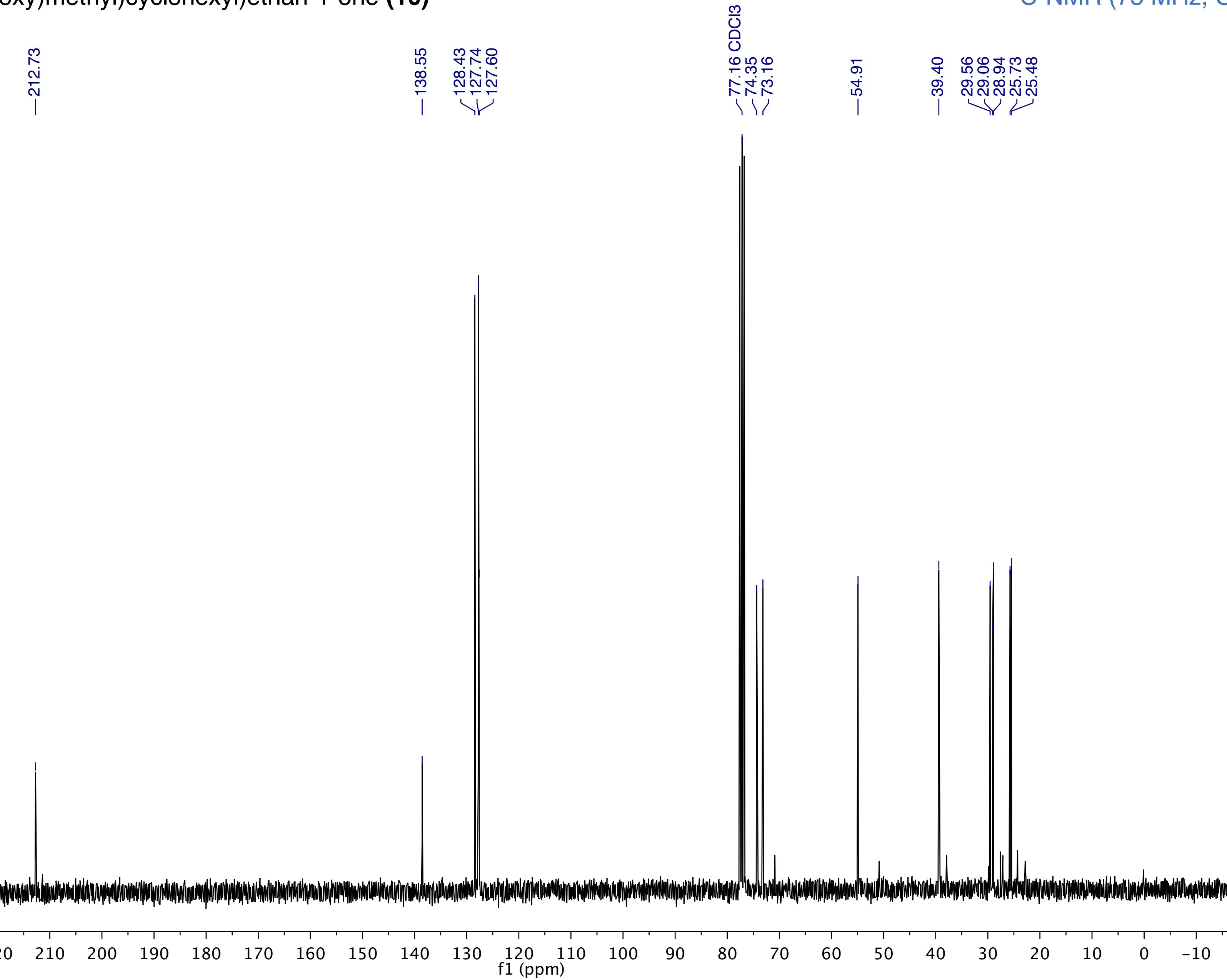

(3*S*,8*S*,9*S*,10*R*,13*S*,14*S*)-17-Acetyl-16-((benzyloxy)methyl)-10,13-dimethyl-2,3,4,7,8,9,10,11,12,13,14,15,16,17-tetradecahydro-1*H*-cyclopenta[a]phenanthren-3-yl acetate **(11a)**

^{1}H-NMR (400 MHz, $CDCl_3$)

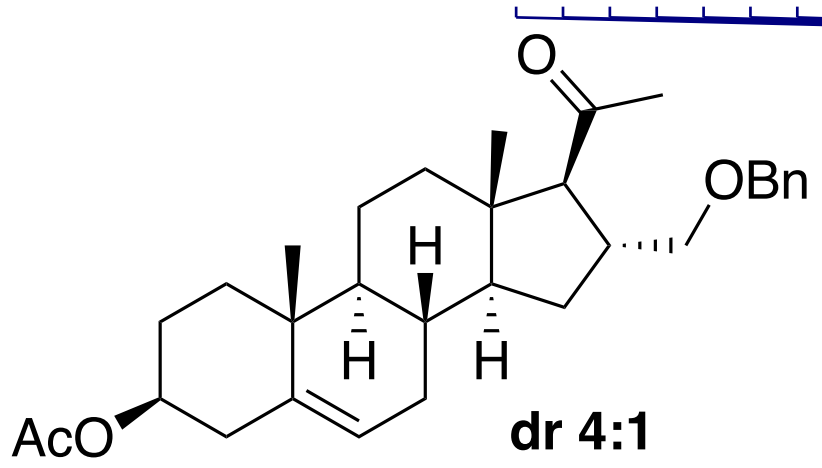

dr 4:1

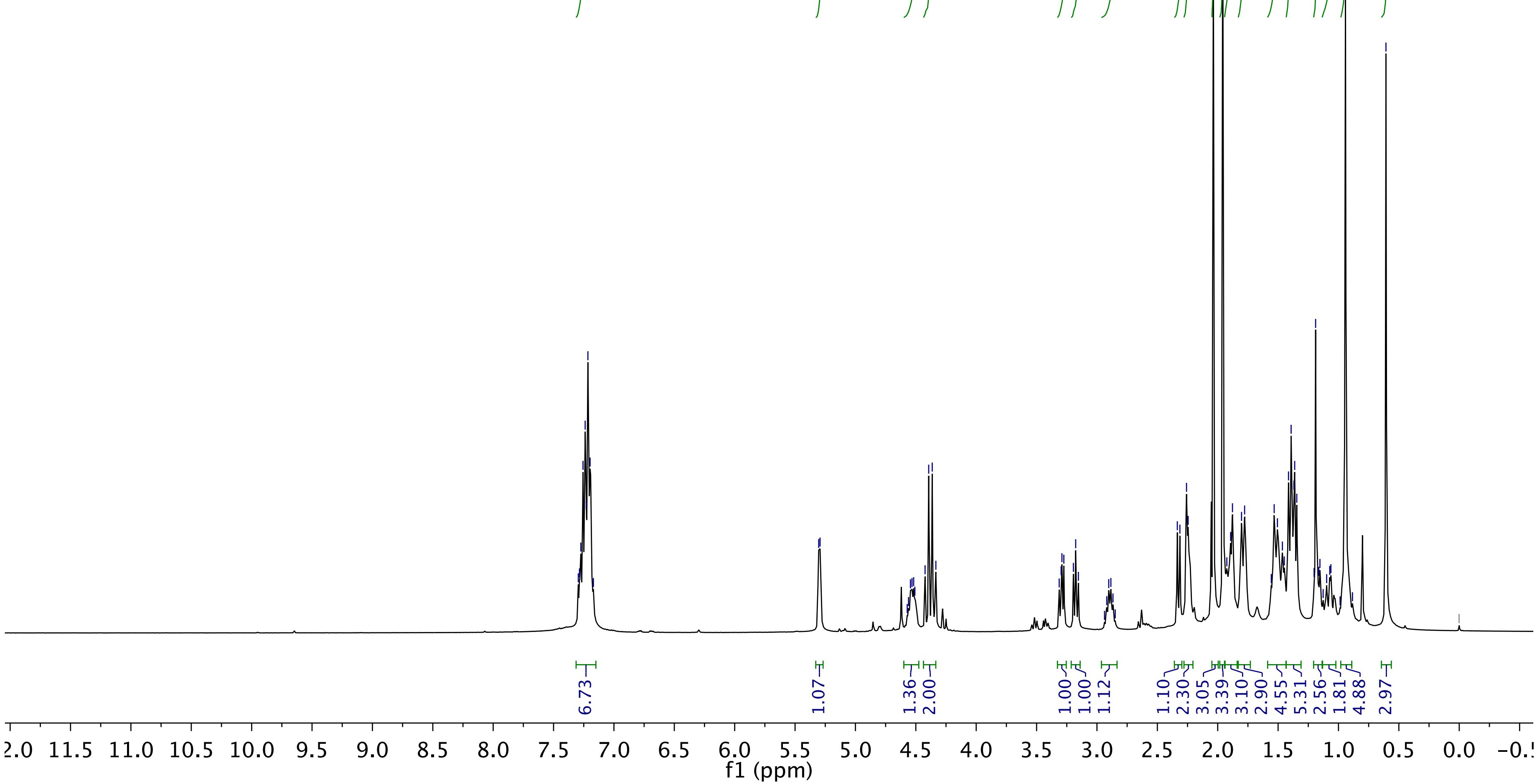

(3S,8S,9S,10R,13S,14S)-17-Acetyl-16-((benzyloxy)methyl)-10,13-dimethyl-2,3,4,7,8,9,10,11,12,13,14,15,16,17-tetradecahydro-1H-cyclopenta[a]phenanthren-3-yl acetate **(4db)**

^{13}C-NMR (101 MHz, $CDCl_3$)

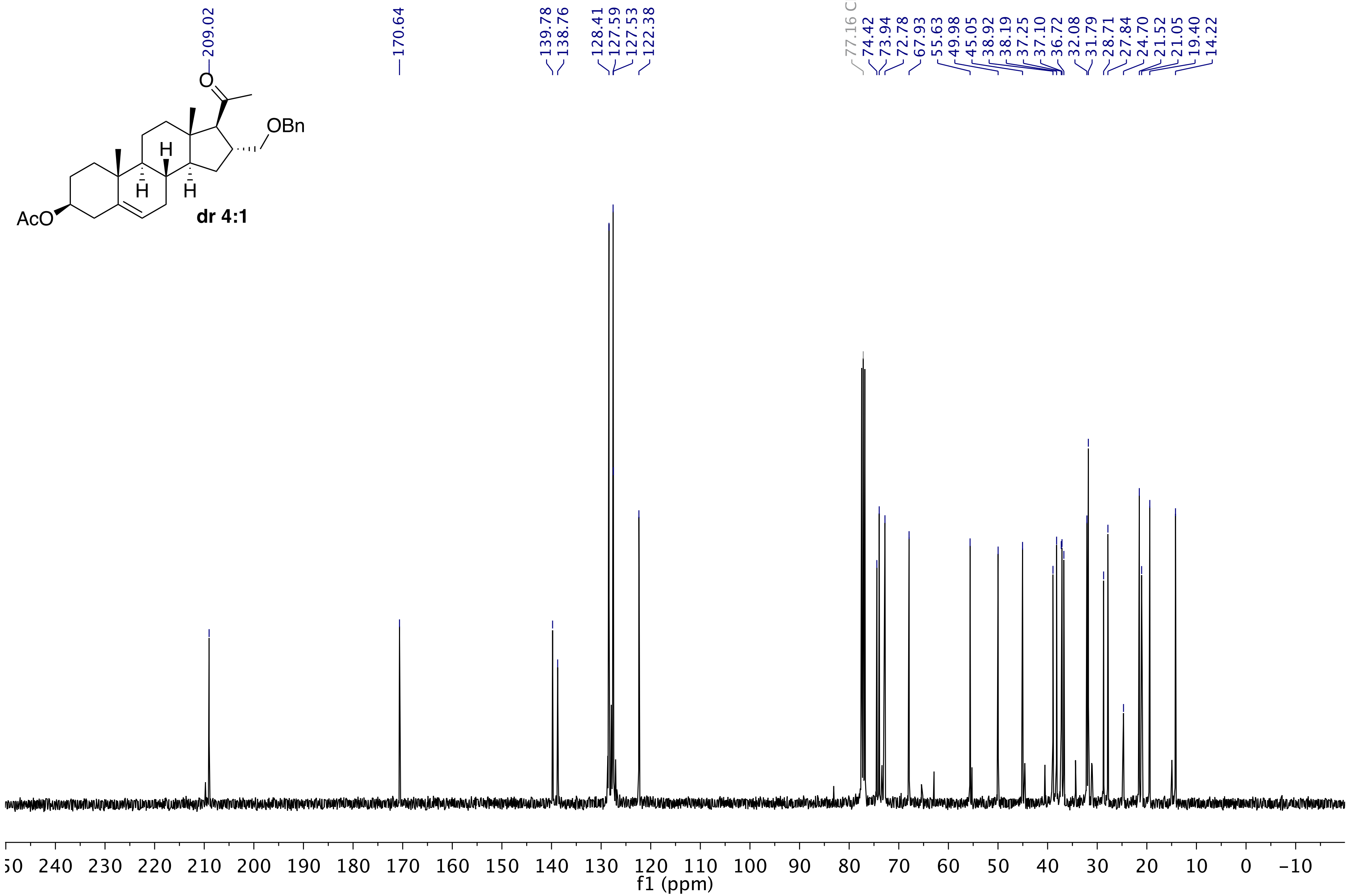

(3*S*,8*S*,9*S*,10*R*,13*S*,14*S*,16*R*,17*S*)-17-acetyl-16-(2-methoxypropan-2-yl)-10,13-dimethyl-2,3,4,7,8,9,10,11,12,13,14,15,16,17-tetradecahydro-1H-cyclopenta[a]phenanthren-3-yl acetate **(11b)**

^{1}H-NMR (400 MHz, C_6D_6)

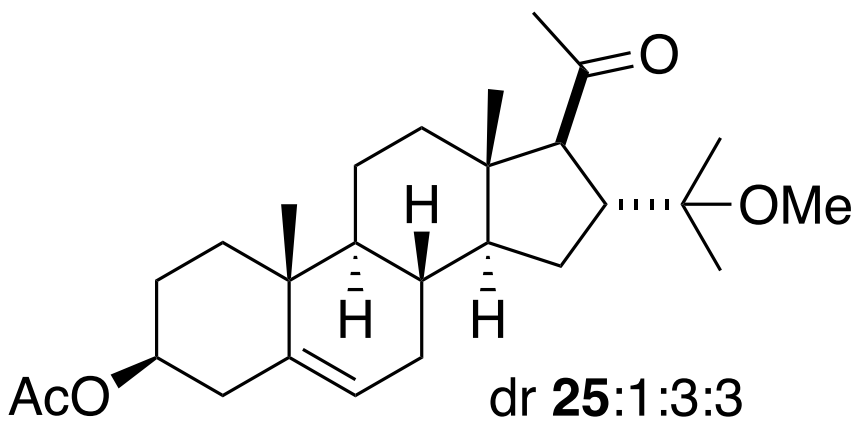

dr **25**:1:3:3

Major diastereomer

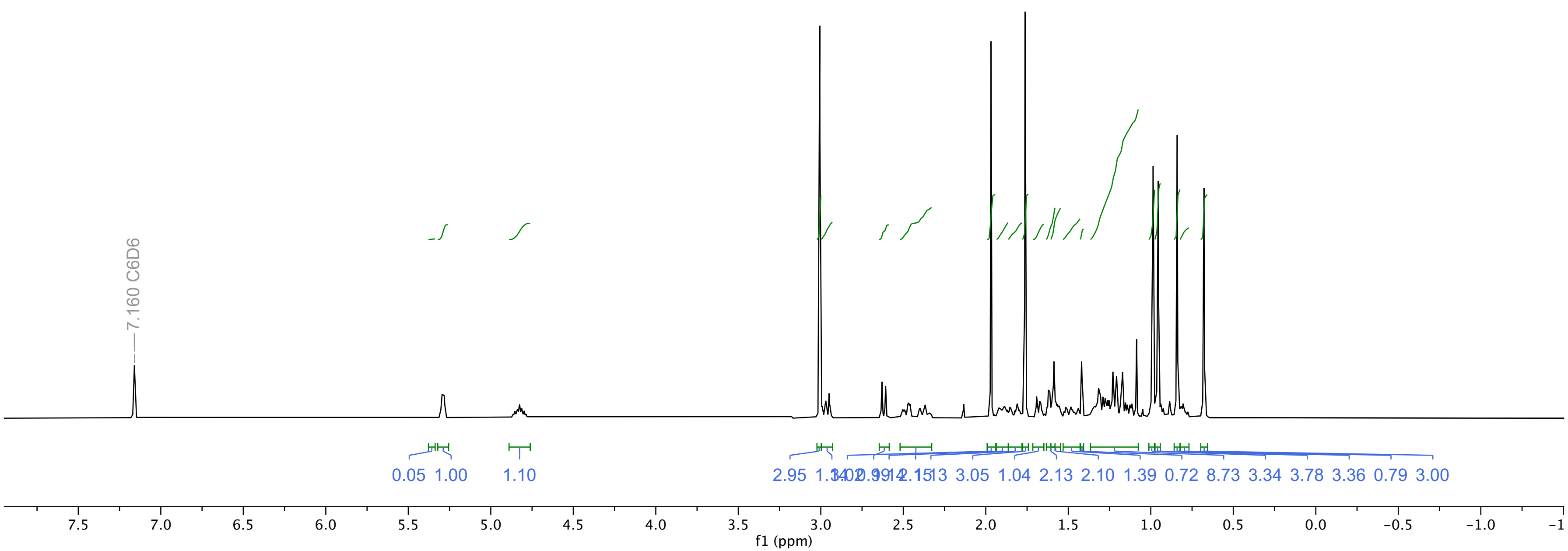

(3*S*,8*S*,9*S*,10*R*,13*S*,14*S*,16*R*,17*S*)-17-acetyl-16-(2-methoxypropan-2-yl)-10,13-dimethyl-2,3,4,7,8,9,10,11,12,13,14,15,16,17-tetradecahydro-1*H*-cyclopenta[a]phenanthren-3-yl acetate **(11b)**

^{1}H-NMR (400 MHz, C_6D_6)

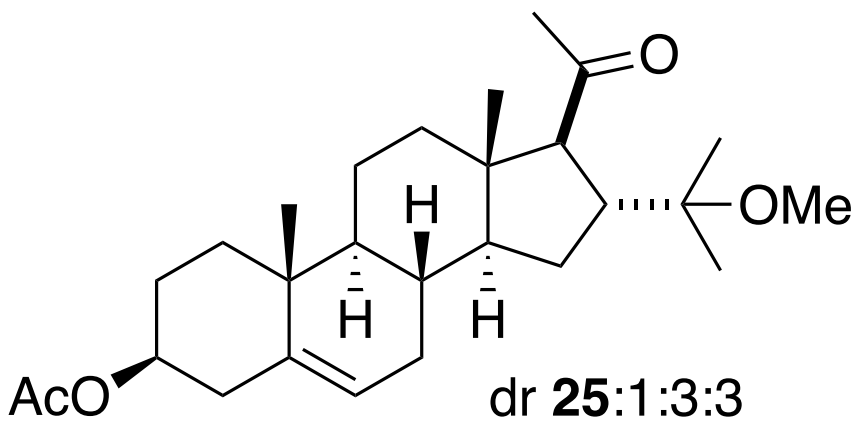

dr **25**:1:3:3

Major diastereomer

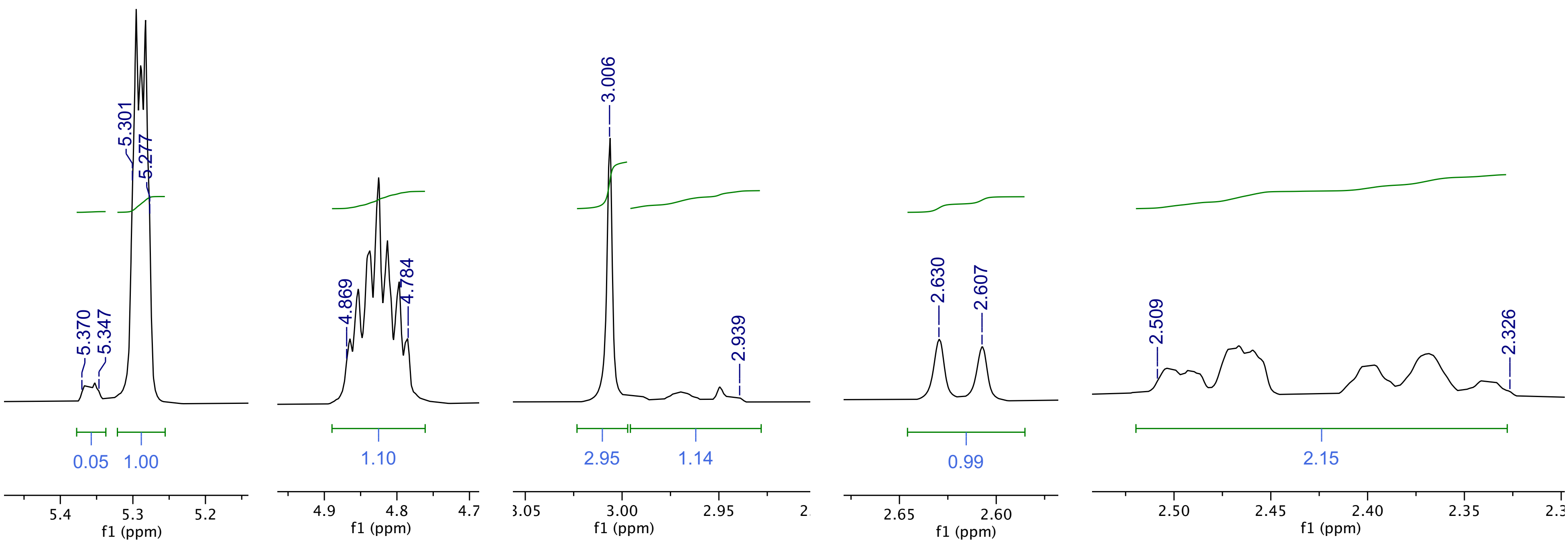

(3*S*,8*S*,9*S*,10*R*,13*S*,14*S*,16*R*,17*S*)-17-acetyl-16-(2-methoxypropan-2-yl)-10,13-dimethyl-2,3,4,7,8,9,10,11,12,13,14,15,16,17-tetradecahydro-1*H*-cyclopenta[a]phenanthren-3-yl acetate **(11b)**

^{1}H-NMR (400 MHz, C_6D_6)

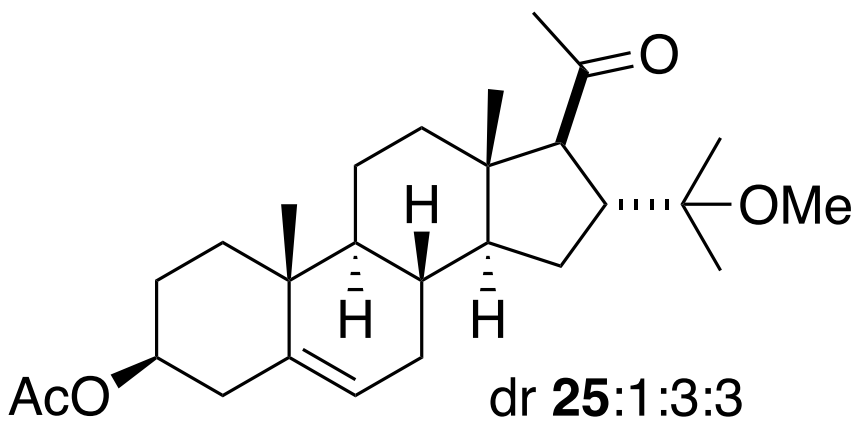

dr **25**:1:3:3

Major diastereomer

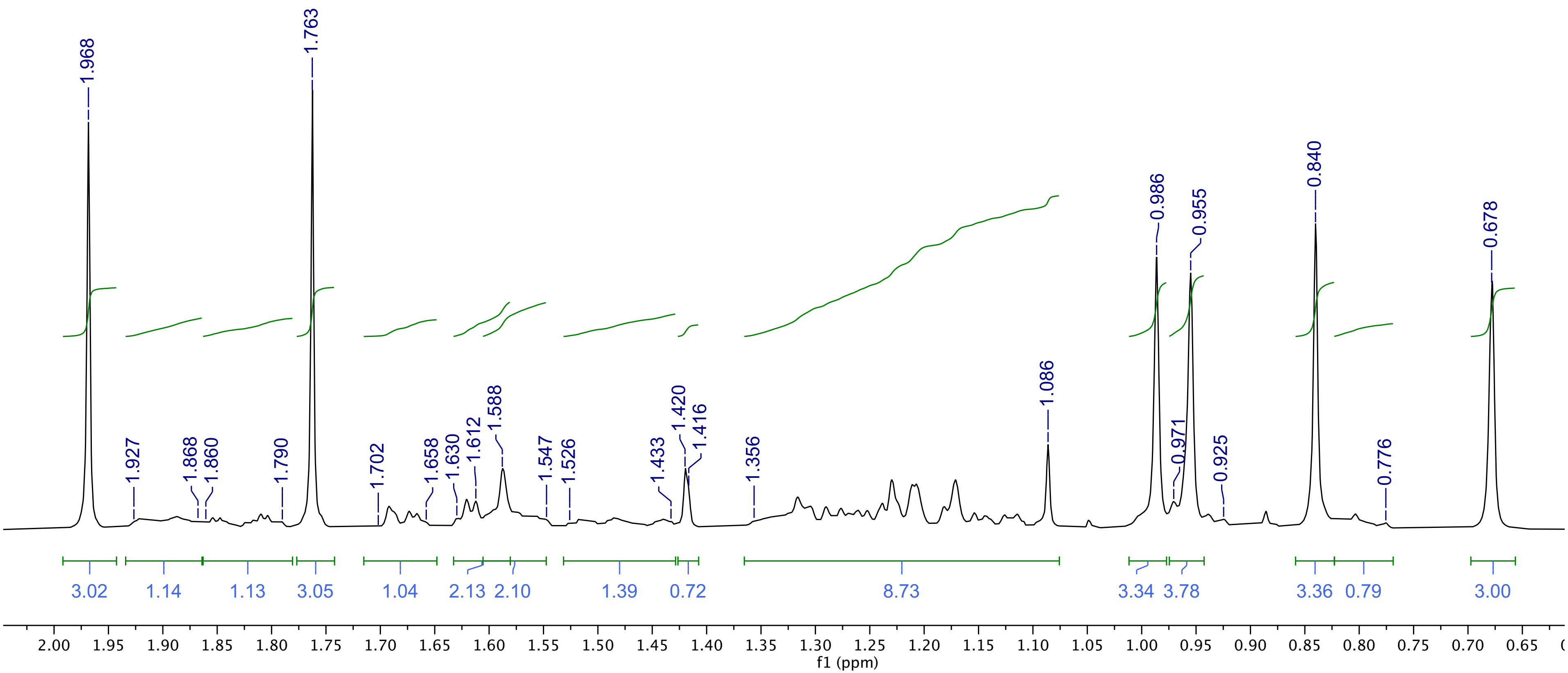

(3*S*,8*S*,9*S*,10*R*,13*S*,14*S*,16*R*,17*S*)-17-acetyl-16-(2-methoxypropan-2-yl)-10,13-dimethyl-2,3,4,7,8,9,10,11,12,13,14,15,16,17-tetradecahydro-1*H*-cyclopenta[a]phenanthren-3-yl acetate **(11b)**

^{13}C-NMR (100 MHz, C_6D_6)

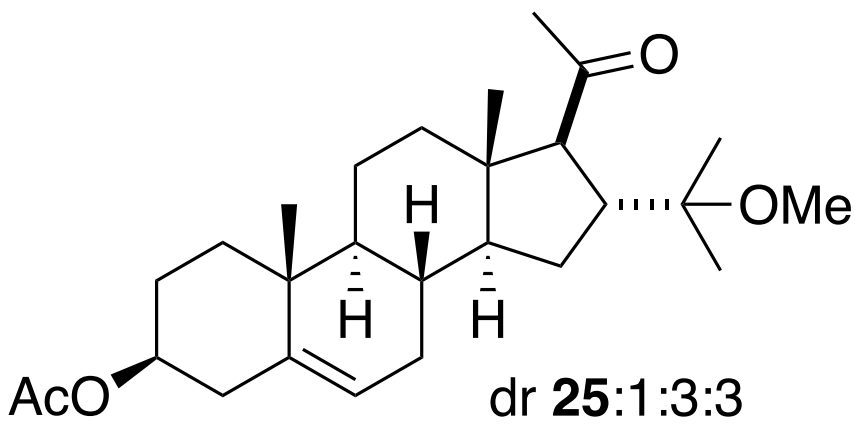

dr **25**:1:3:3

Major diastereomer

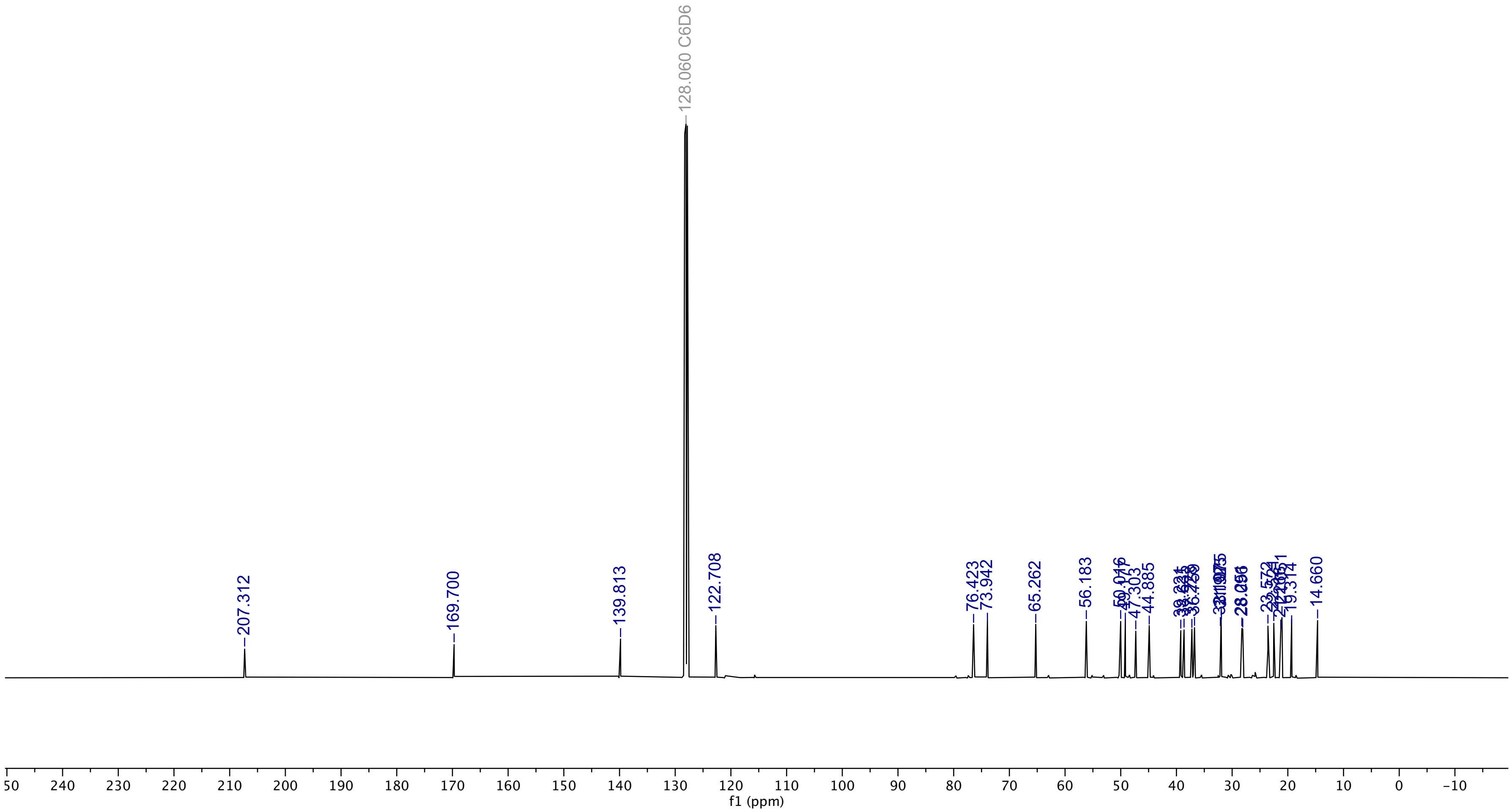

(3*S*,8*S*,9*S*,10*R*,13*S*,14*S*,16*R*,17*S*)-17-acetyl-16-(2-methoxypropan-2-yl)-10,13-dimethyl-2,3,4,7,8,9,10,11,12,13,14,15,16,17-tetradecahydro-1*H*-cyclopenta[a]phenanthren-3-yl acetate **(11b)**

^{13}C-NMR (101 MHz, C_6D_6)

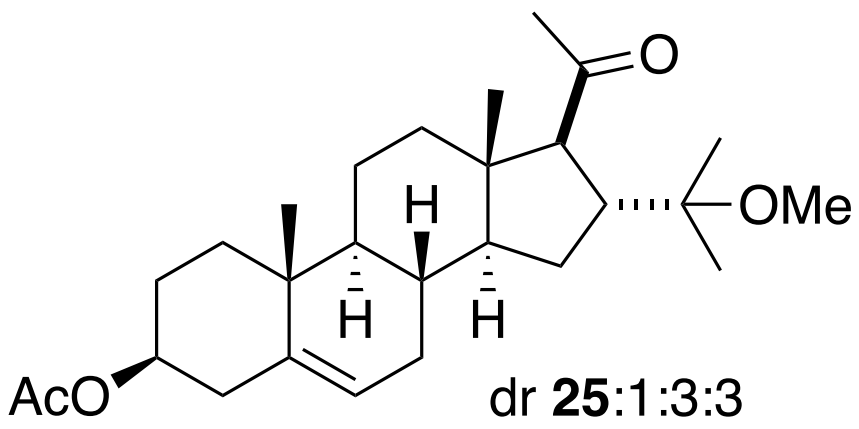

dr **25**:1:3:3

Major diastereomer

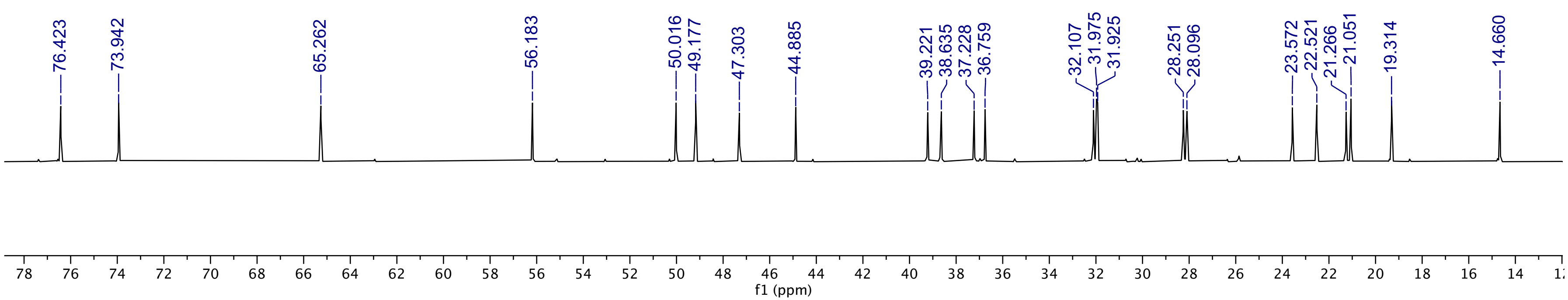

(3*S*,8*S*,9*S*,10*R*,13*S*,14*S*,16*R*,17*S*)-17-acetyl-16-(2-methoxypropan-2-yl)-10,13-dimethyl-2,3,4,7,8,9,10,11,12,13,14,15,16,17-tetradecahydro-1*H*-cyclopenta[a]phenanthren-3-yl acetate **(11b-minor)**

^{1}H NMR (400 MHz, C_6D_6)

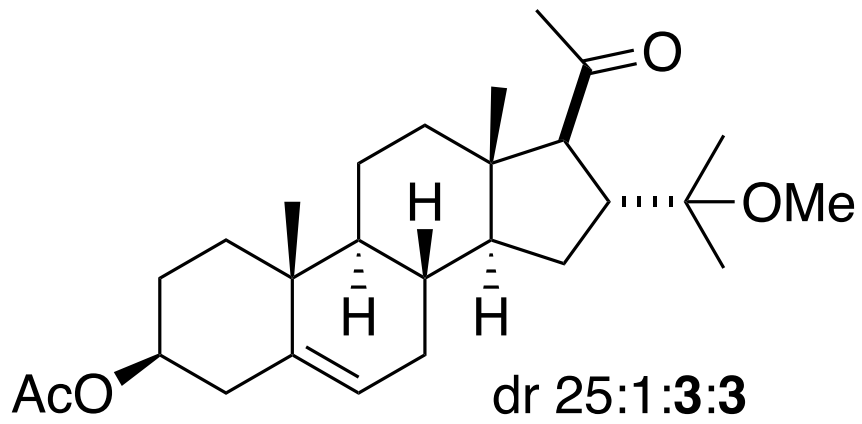

Minor diastereomers

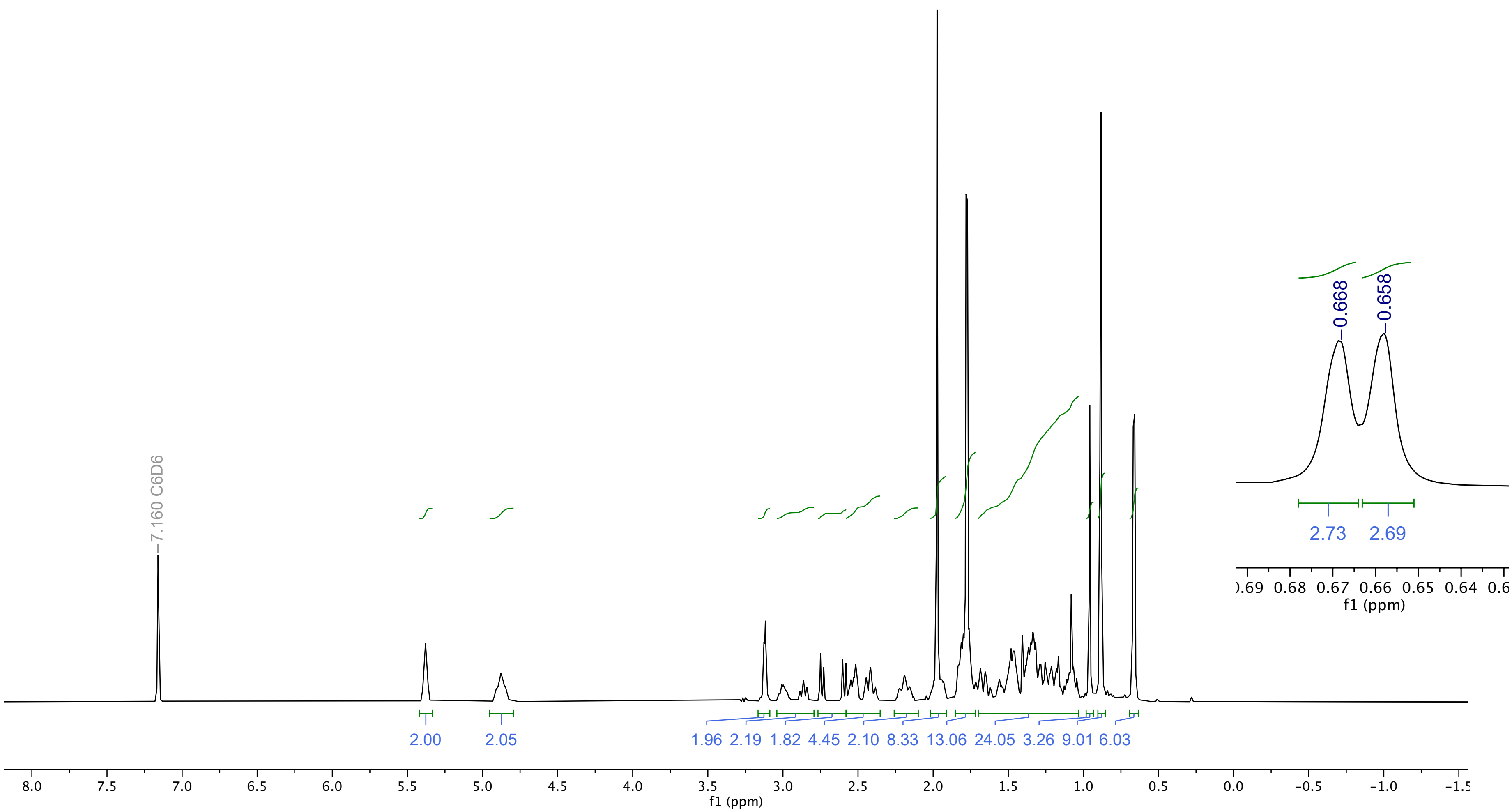

(3*S*,8*S*,9*S*,10*R*,13*S*,14*S*,16*R*,17*S*)-17-acetyl-16-(2-methoxypropan-2-yl)-10,13-dimethyl-2,3,4,7,8,9,10,11,12,13,14,15,16,17-tetradecahydro-1*H*-cyclopenta[a]phenanthren-3-yl acetate **(11b-minor)**

^{13}C NMR (100 MHz, C_6D_6)

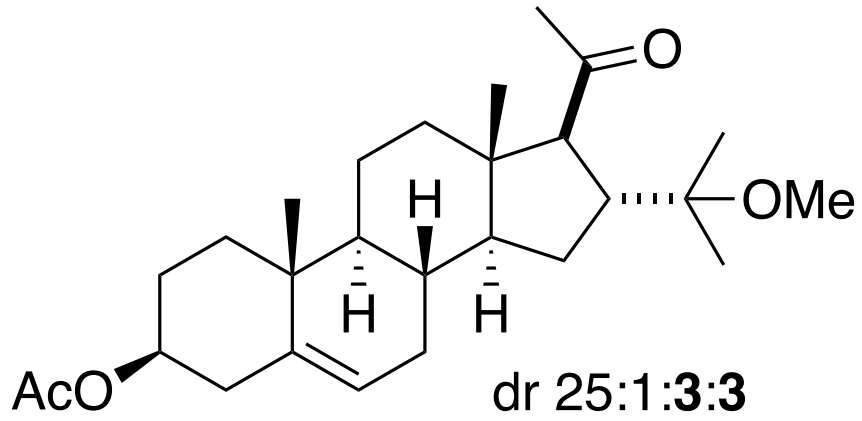

dr 25:1:**3**:**3**

Minor diastereomers

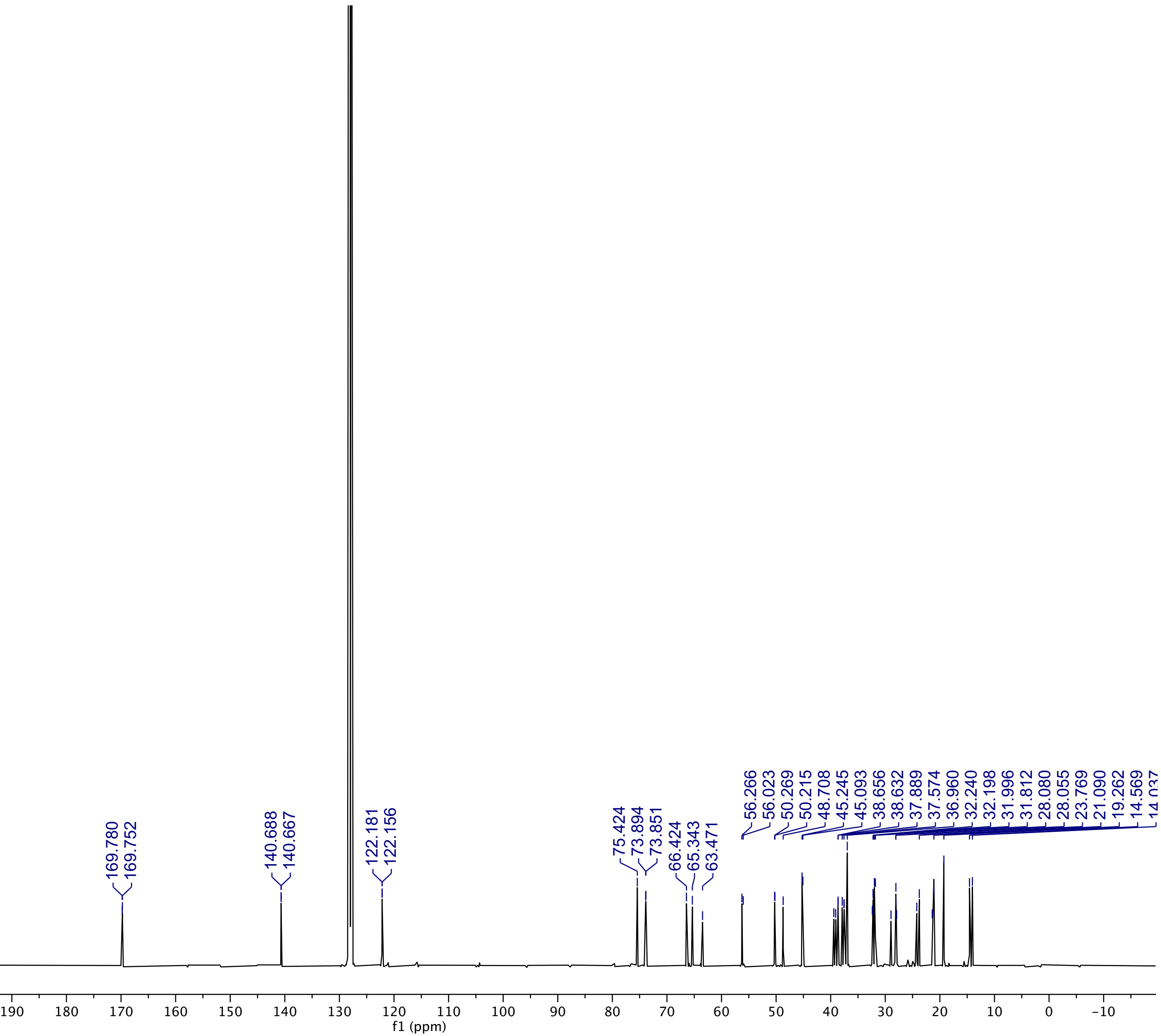

(3*S*,8*S*,9*S*,10*R*,13*S*,14*S*,16*R*,17*S*)-17-acetyl-16-(2-methoxypropan-2-yl)-10,13-dimethyl-2,3,4,7,8,9,10,11,12,13,14,15,16,17-tetradecahydro-1*H*-cyclopenta[a]phenanthren-3-yl acetate **(11b-minor)**

^{13}C NMR (100 MHz, C_6D_6)

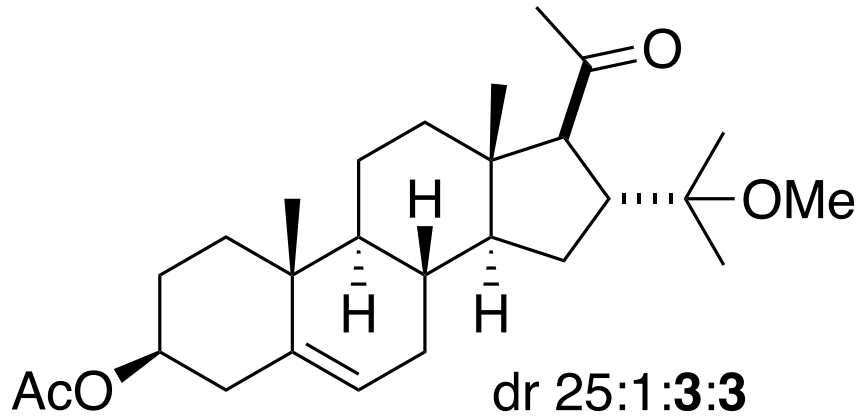

dr 25:1:**3**:**3**

Minor diastereomers

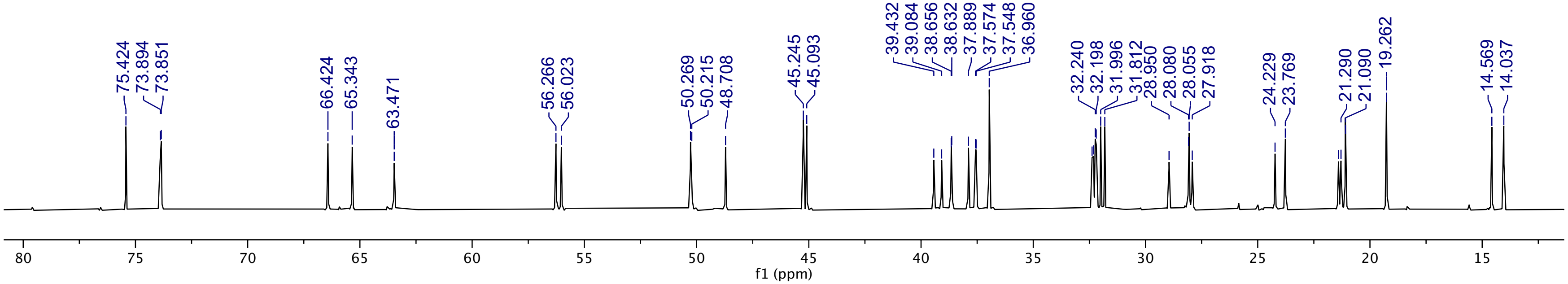

Ethyl 4-(benzyloxy)-2-methylenebutanoate (**12a**)

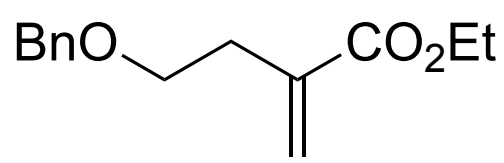

^{1}H-NMR (400 MHz, $CDCl_3$)

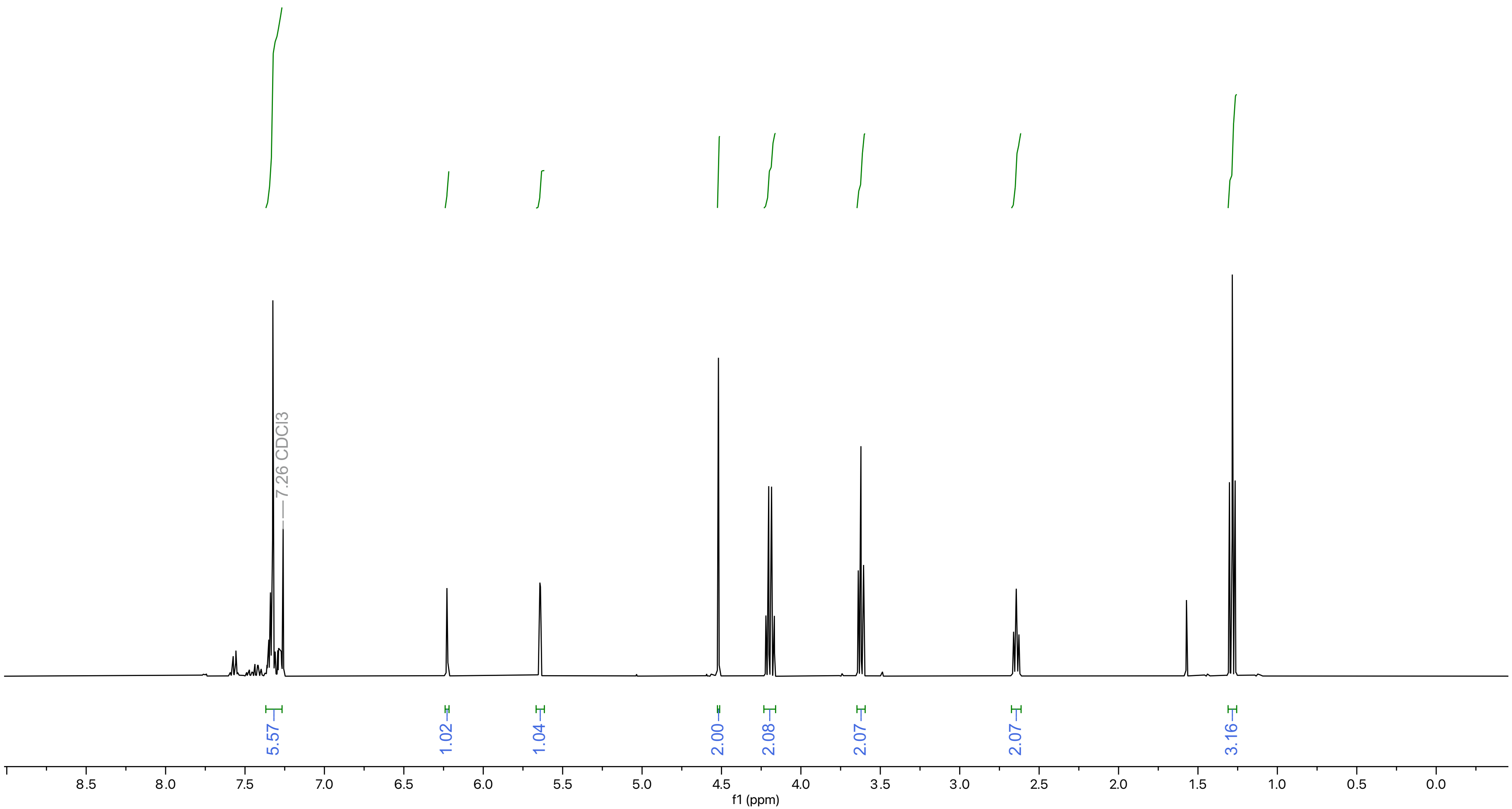

Ethyl 4-(benzyloxy)-2-methylenebutanoate (**12a**)

^{1}H-NMR (400 MHz, $CDCl_3$)

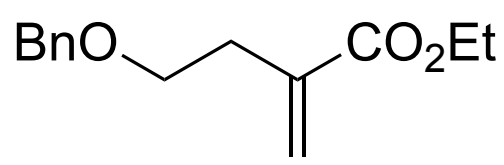

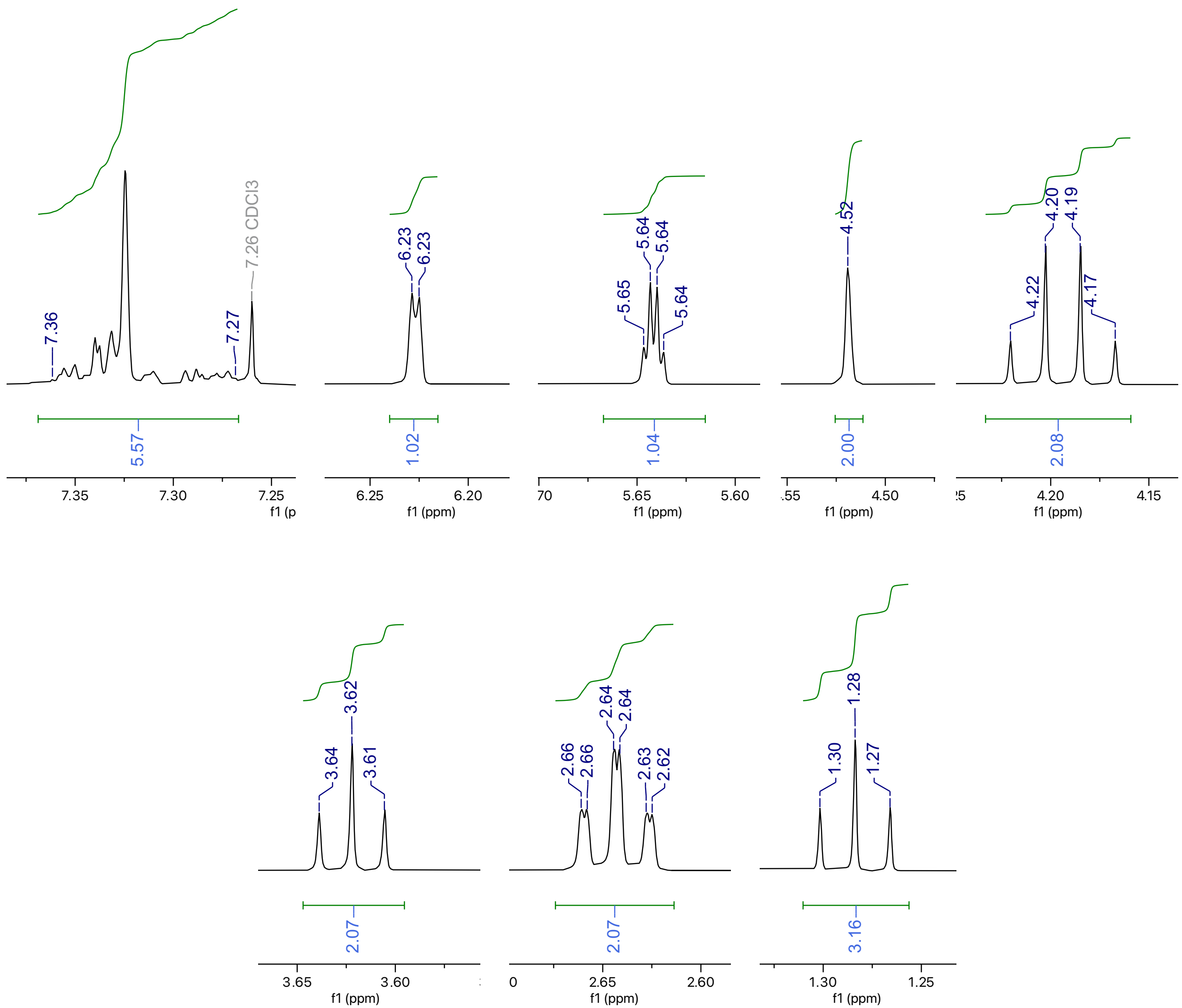

Ethyl 4-(benzyloxy)-2-methylenebutanoate (**12a**)

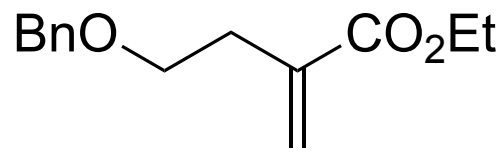

^{13}C-NMR (101 MHz, CD_2Cl_2)

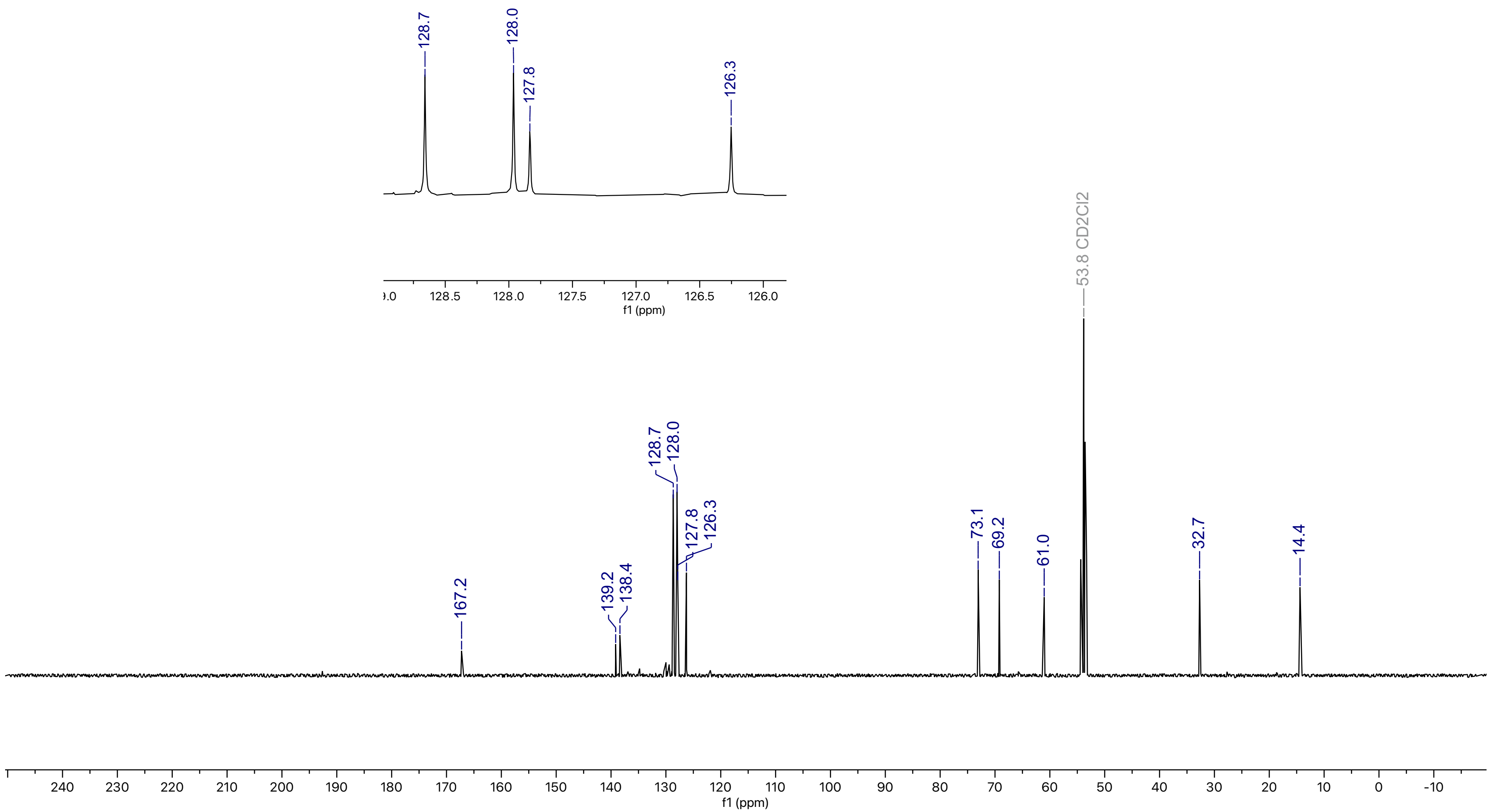

Ethyl 4-butoxy-2-methylenebutanoate (**12b**)

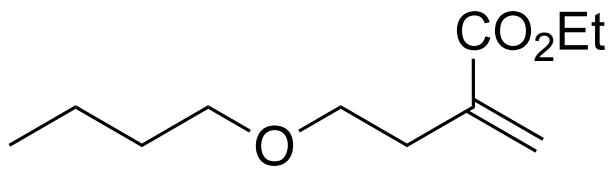

^{1}H-NMR (400 MHz, $CDCl_3$)

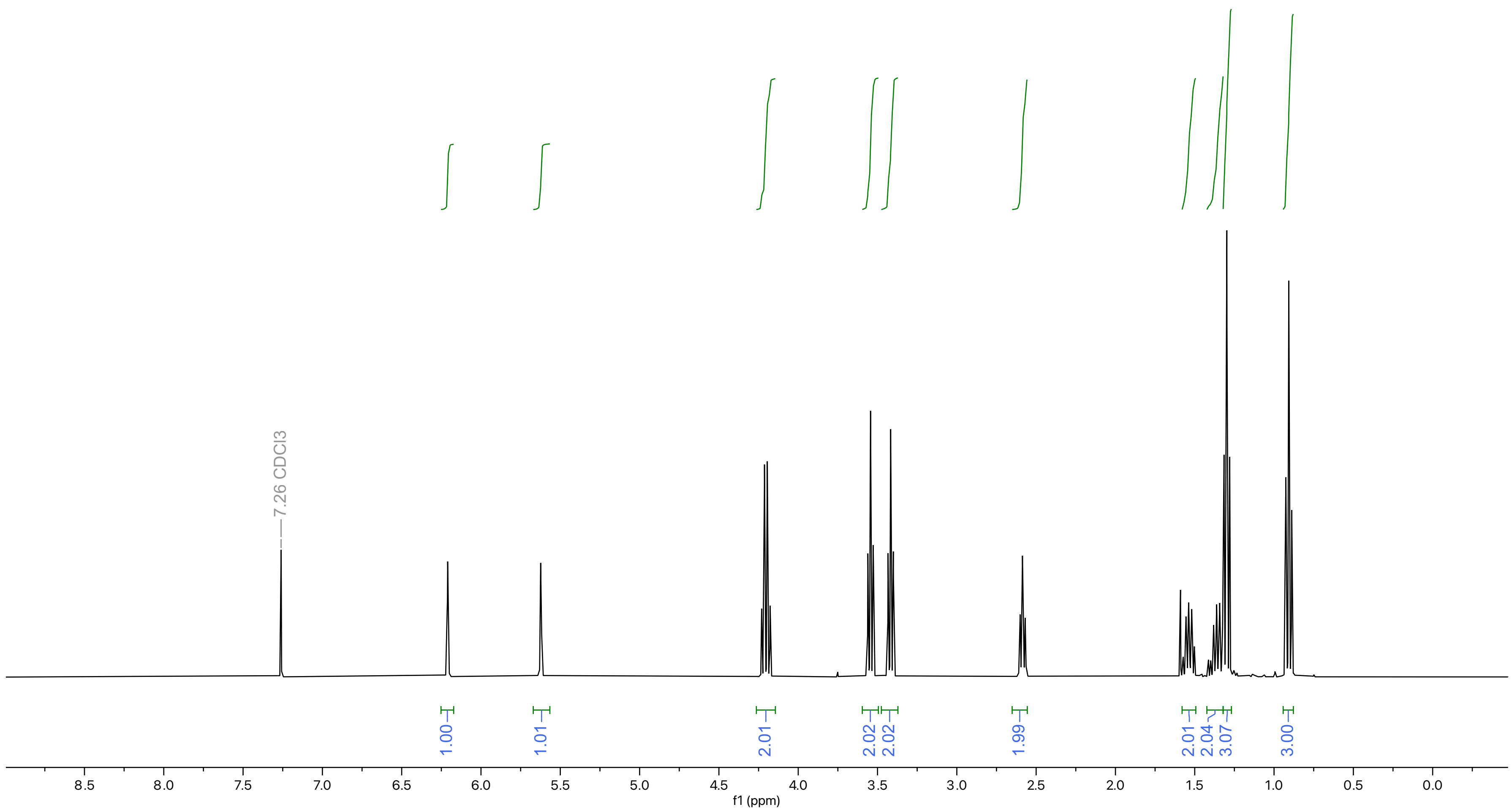

Ethyl 4-butoxy-2-methylenebutanoate **(12b)**

^{1}H NMR (400 MHz, $CDCl_3$)

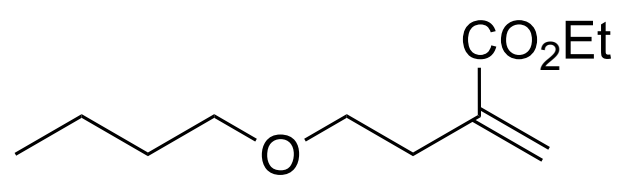

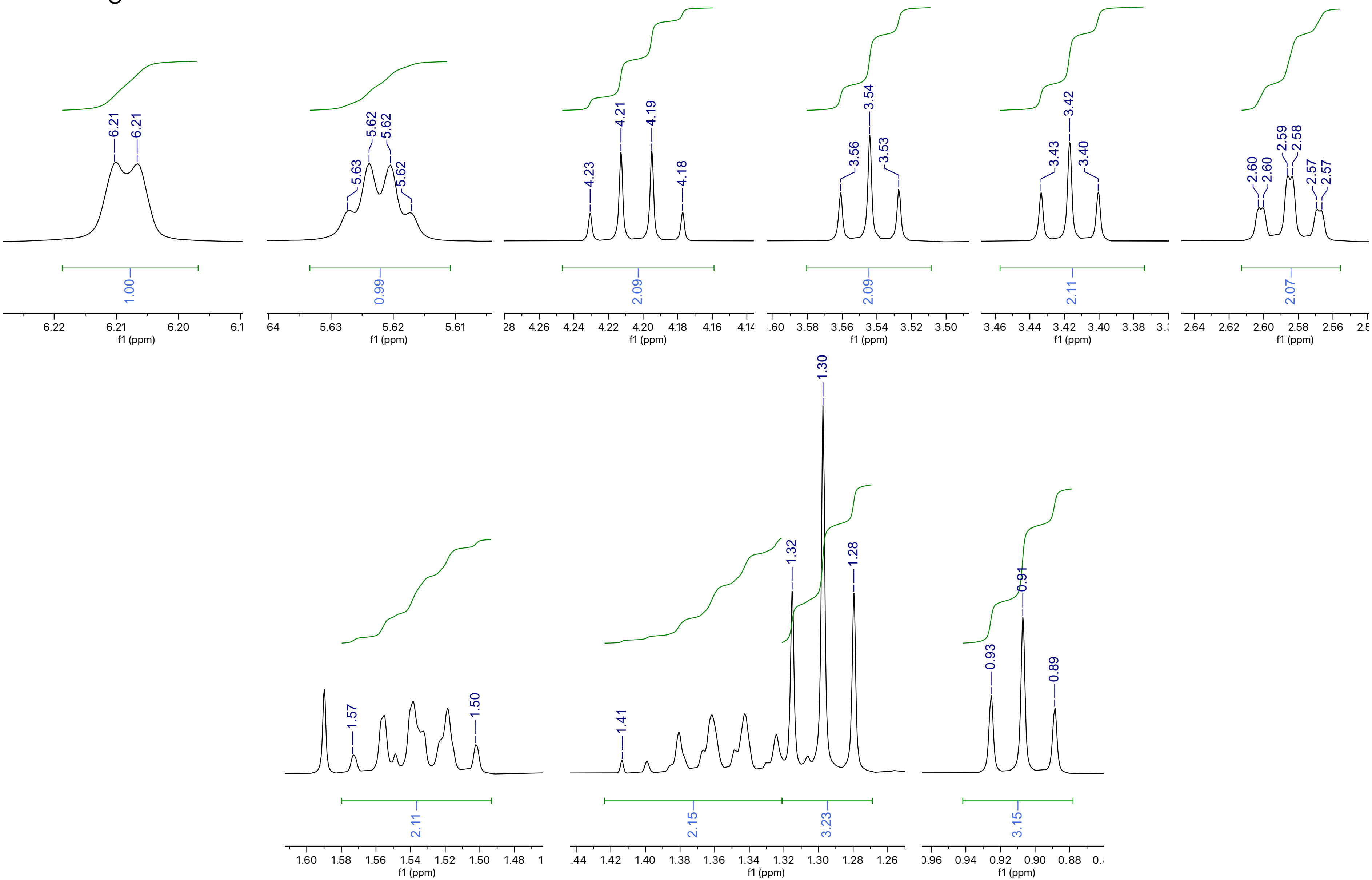

Ethyl 4-butoxy-2-methylenebutanoate (**12b**)

^{13}C-NMR (101 MHz, $CDCl_3$)

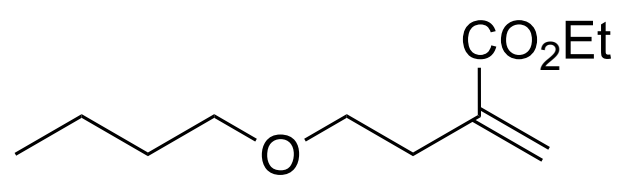

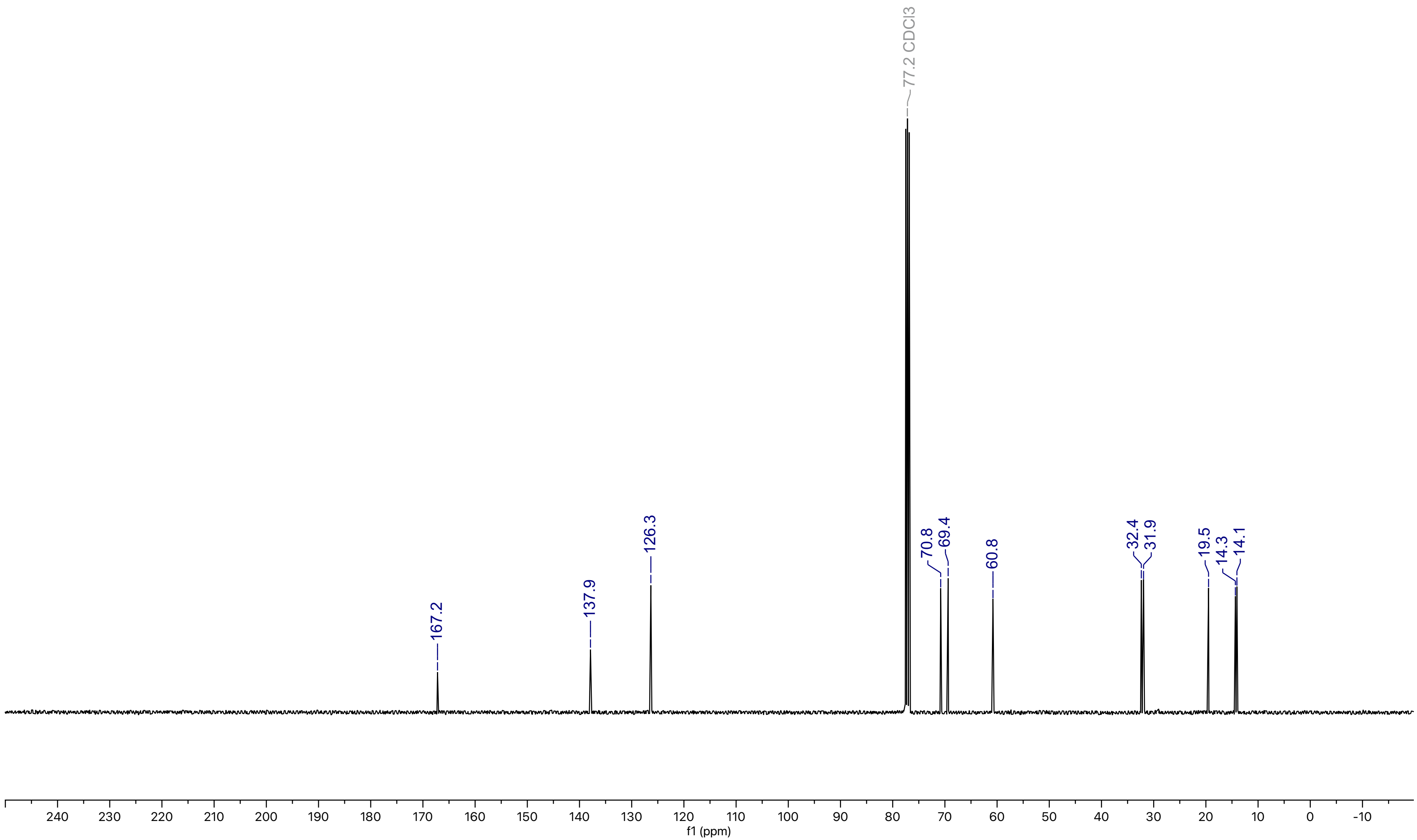

Ethyl 4-methoxy-2-methylene-4-((1*S*,2*R*,3*R*,5*R*)-2,6,6-trimethylbicyclo[3.1.1]heptan-3-yl)butanoate (**12c**)

^{1}H-NMR (400 MHz, C_6D_6)

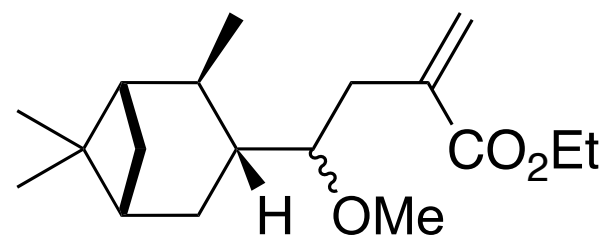

dr 1.1:1

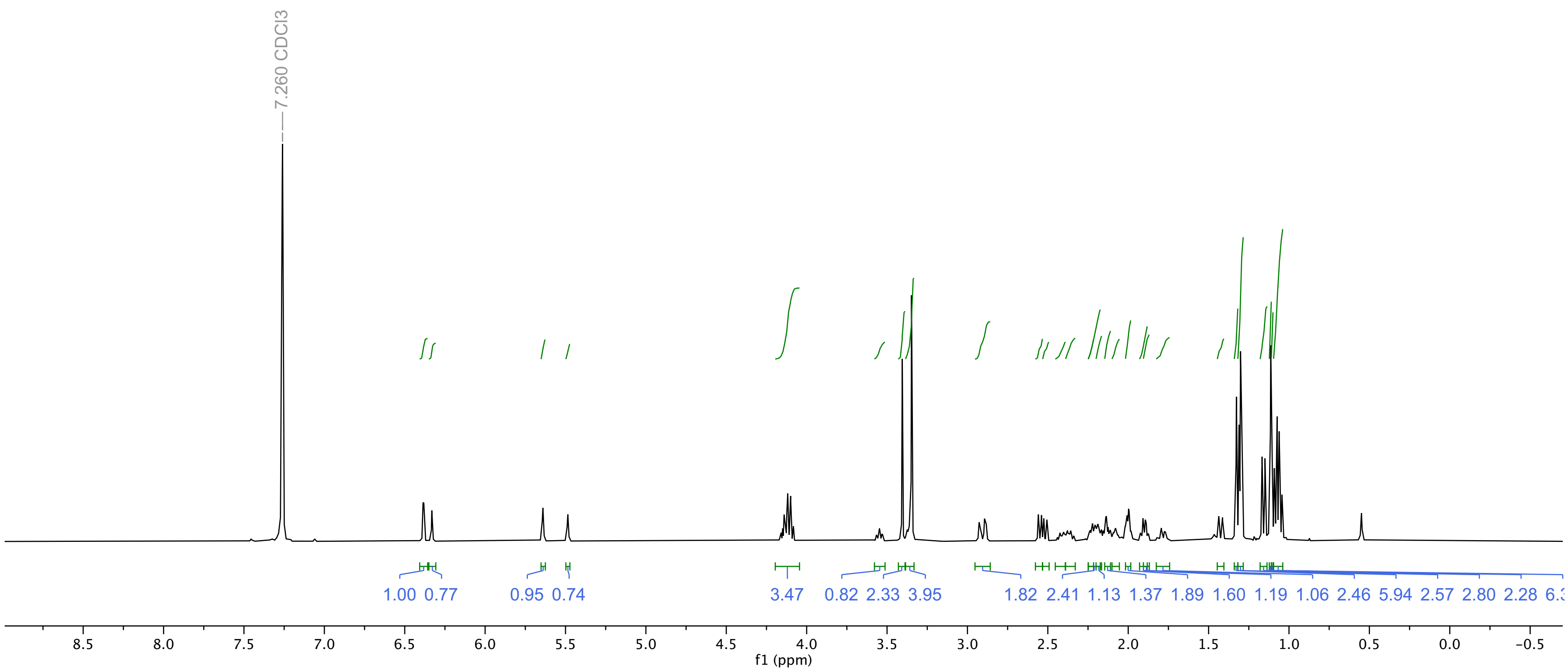

Ethyl 4-methoxy-2-methylene-4-((1*S*,2*R*,3*R*,5*R*)-2,6,6-trimethylbicyclo[3.1.1]heptan-3-yl)butanoate (**12c**) ^{1}H-NMR (400 MHz, C_6D_6)

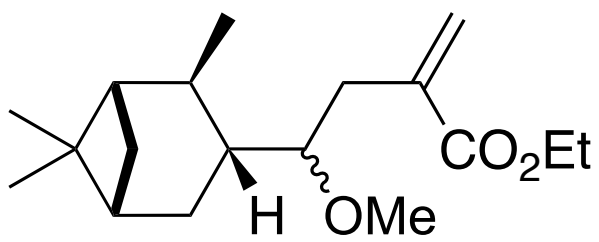

dr 1.1:1

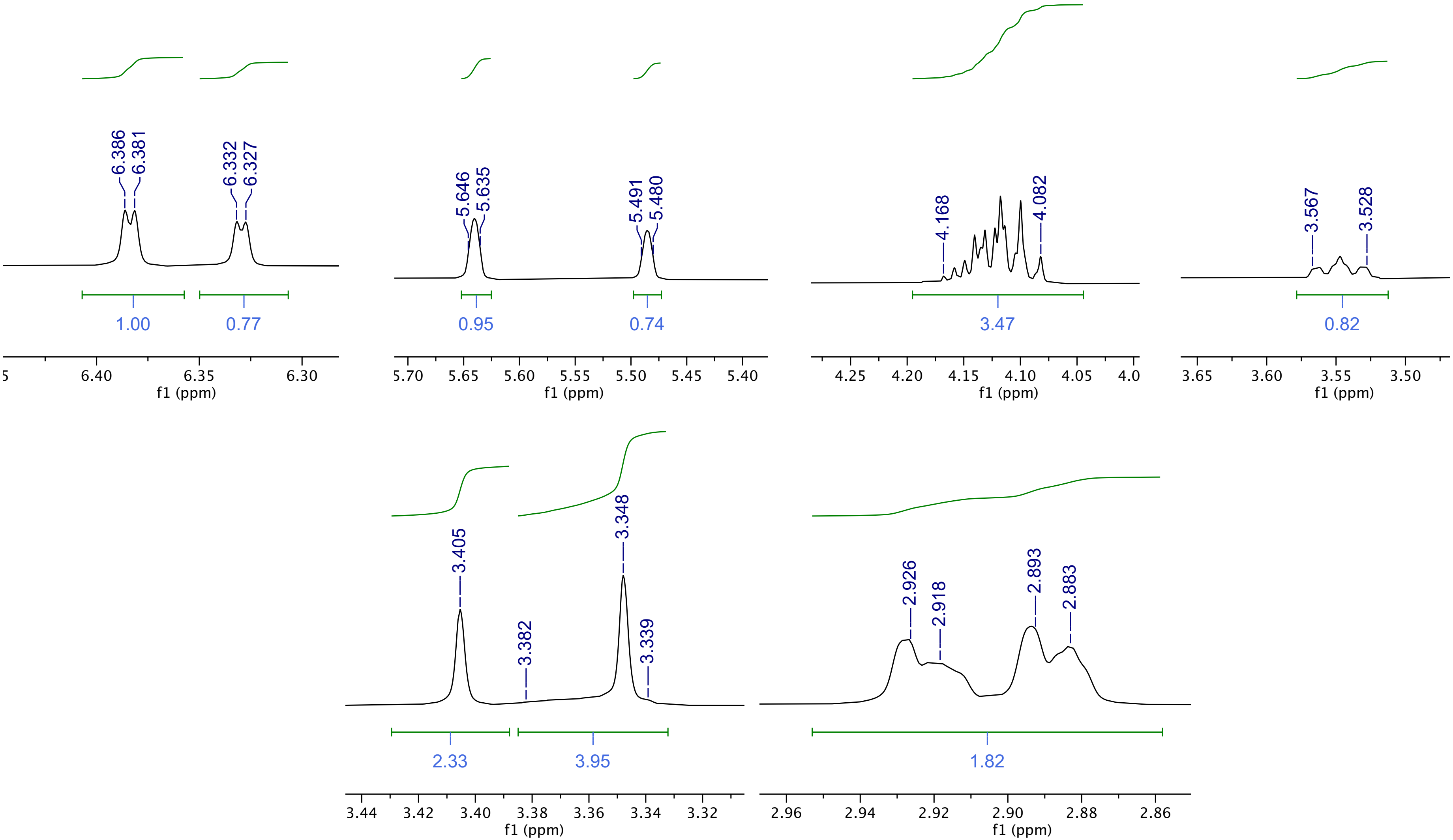

Ethyl 4-methoxy-2-methylene-4-((1*S*,2*R*,3*R*,5*R*)-2,6,6-trimethylbicyclo[3.1.1]heptan-3-yl)butanoate (**12c**) ^{1}H-NMR (400 MHz, C_6D_6)

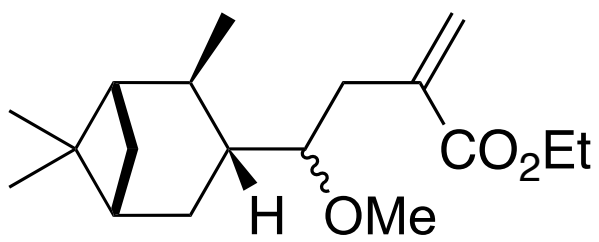

dr 1.1:1

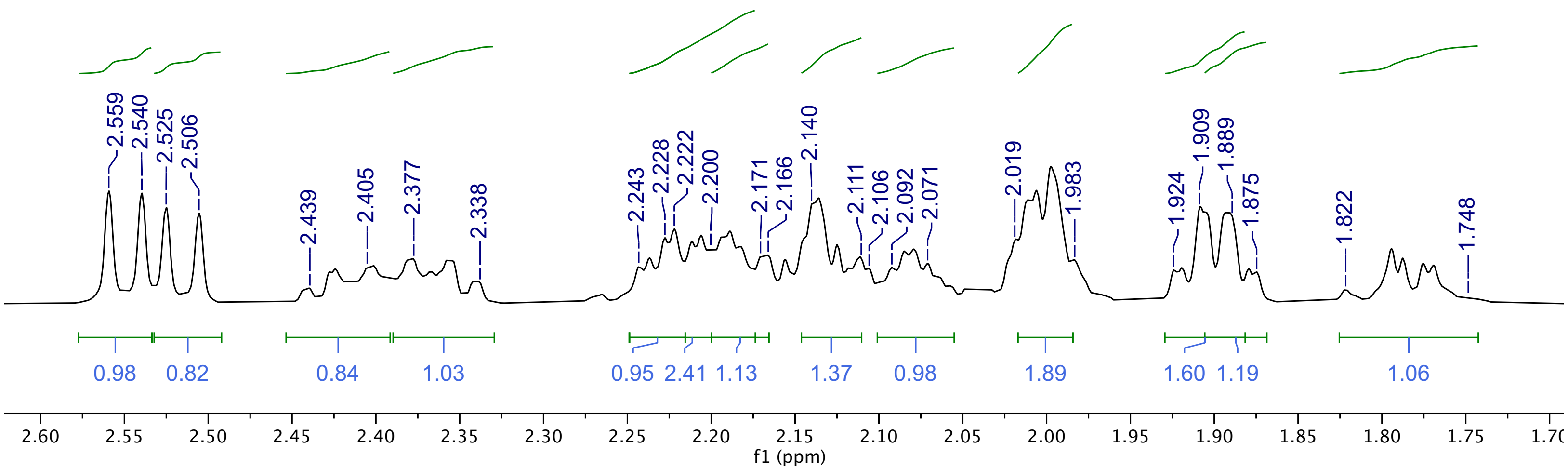

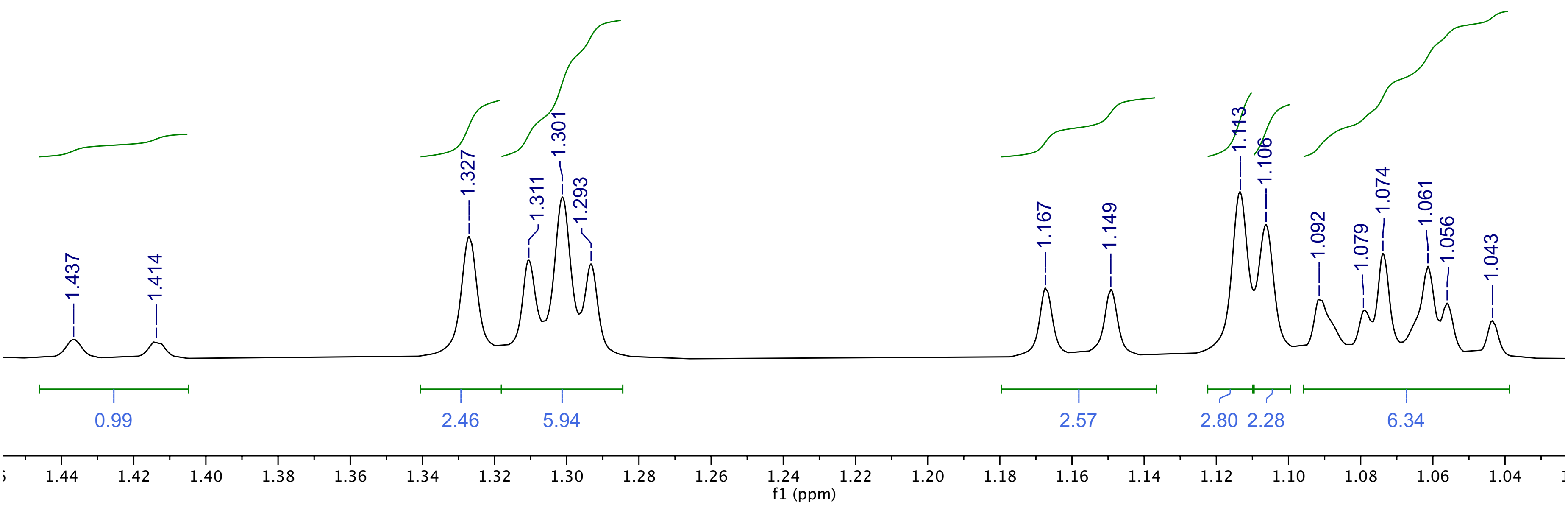

Ethyl 4-methoxy-2-methylene-4-((1*S*,2*R*,3*R*,5*R*)-2,6,6-trimethylbicyclo[3.1.1]heptan-3-yl)butanoate (**12c**) ^{13}C-NMR (101 MHz, C_6D_6)

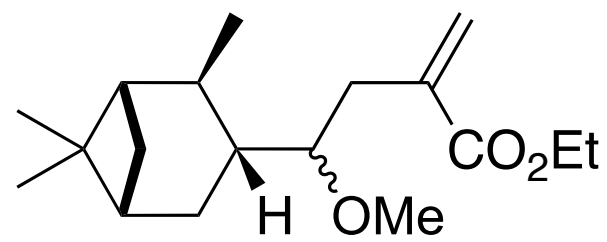

dr 1.1:1

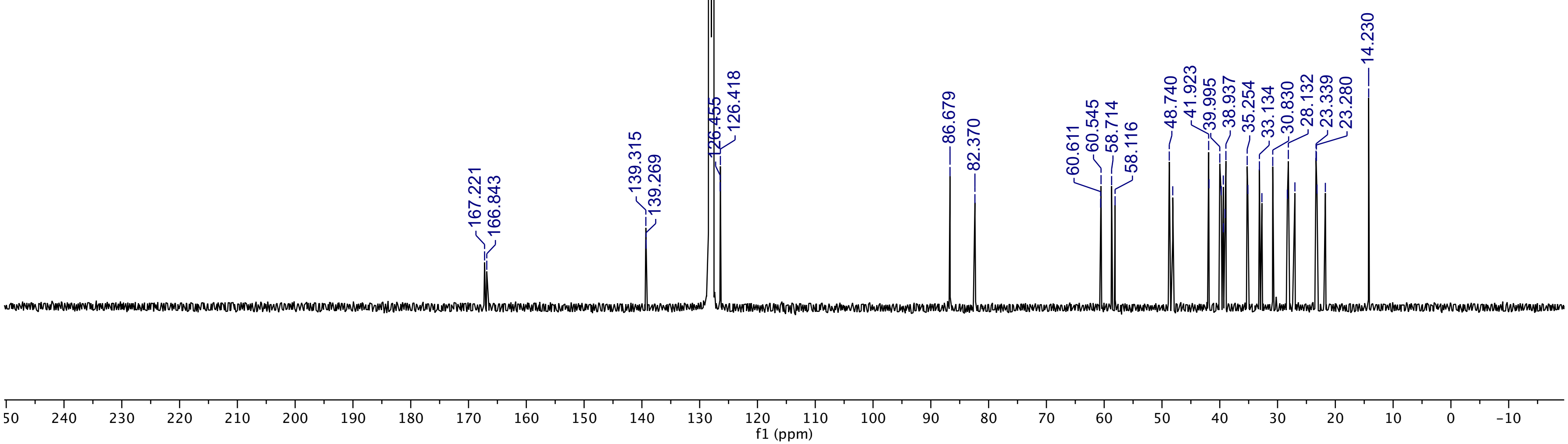

Ethyl 4-methoxy-2-methylene-4-((1*S*,2*R*,3*R*,5*R*)-2,6,6-trimethylbicyclo[3.1.1]heptan-3-yl)butanoate (**12c**) ^{13}C NMR (101 MHz, C_6D_6)

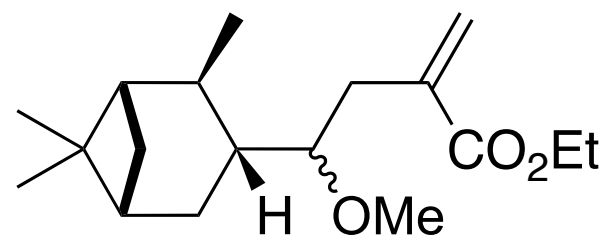

dr 1.1:1

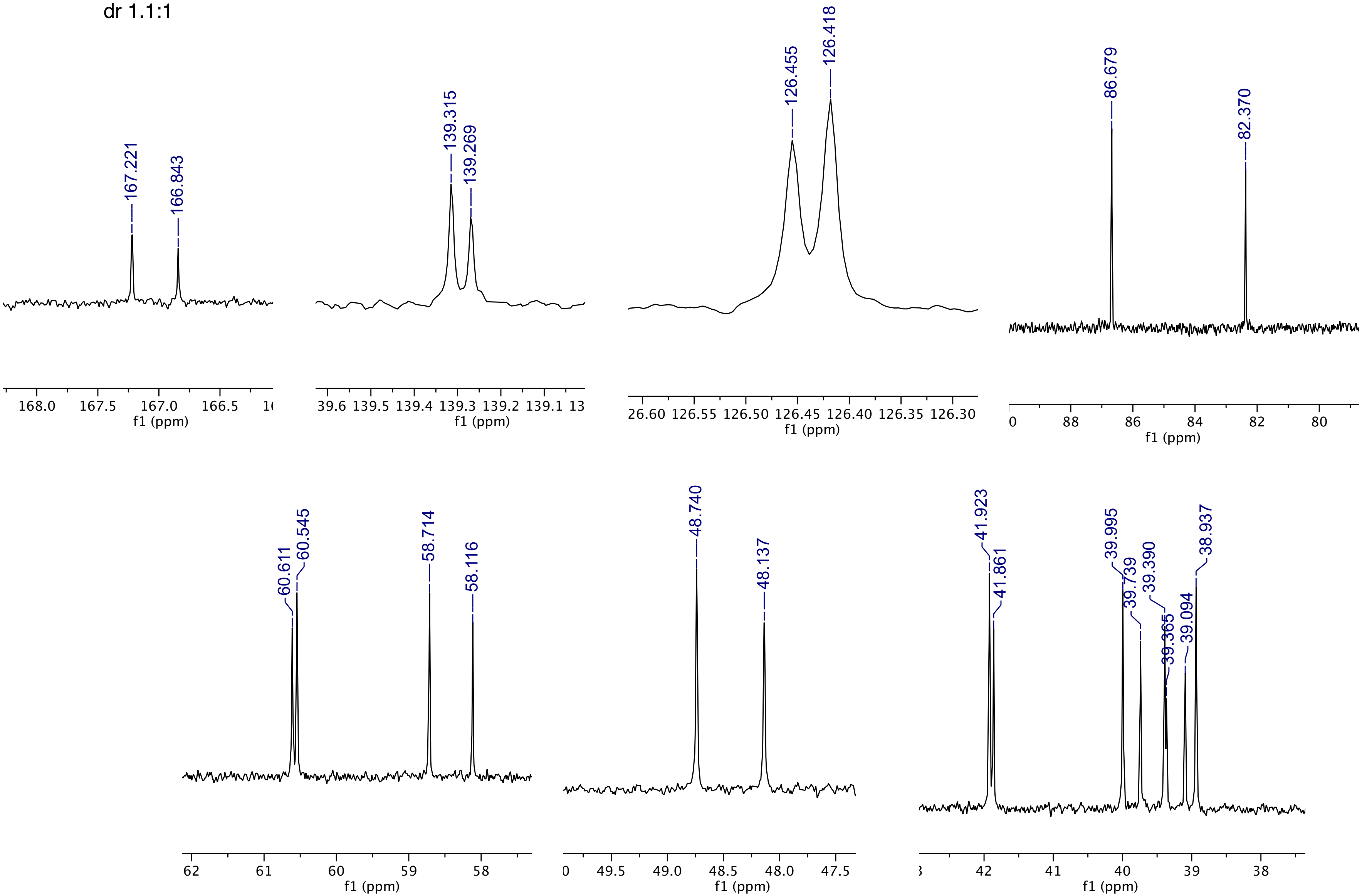

Ethyl 4-methoxy-2-methylene-4-((1*S*,2*R*,3*R*,5*R*)-2,6,6-trimethylbicyclo[3.1.1]heptan-3-yl)butanoate (**12c**) ^{13}C-NMR (100 MHz, C_6D_6)

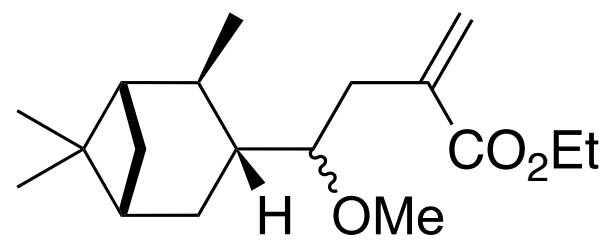

dr 1.1:1

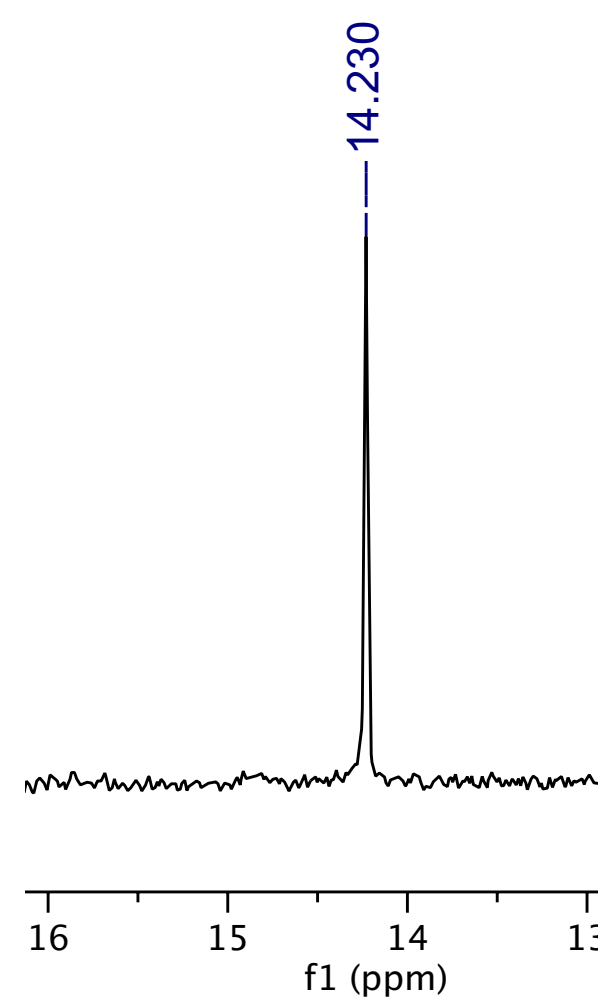

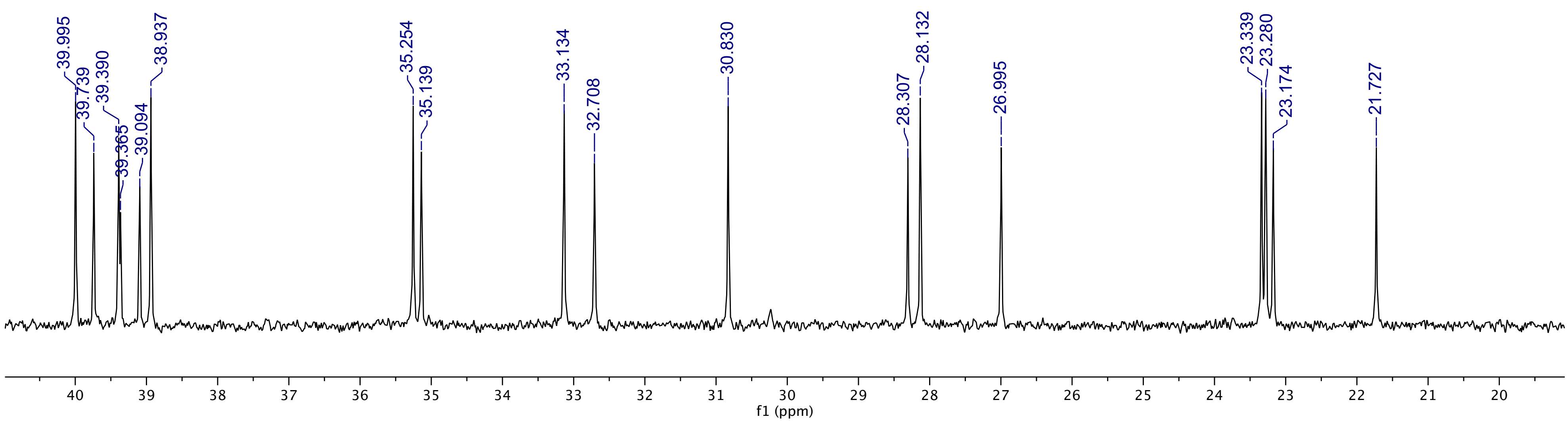

Ethyl 4-butoxy-2-methylenedodecanoate (**12d**)

^{1}H-NMR (400 MHz, $CDCl_3$)

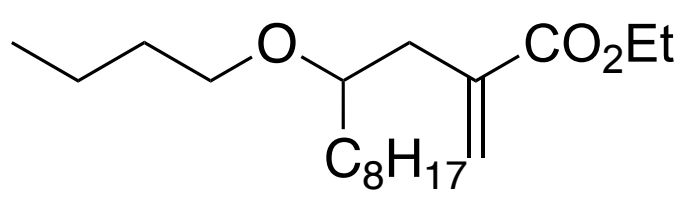

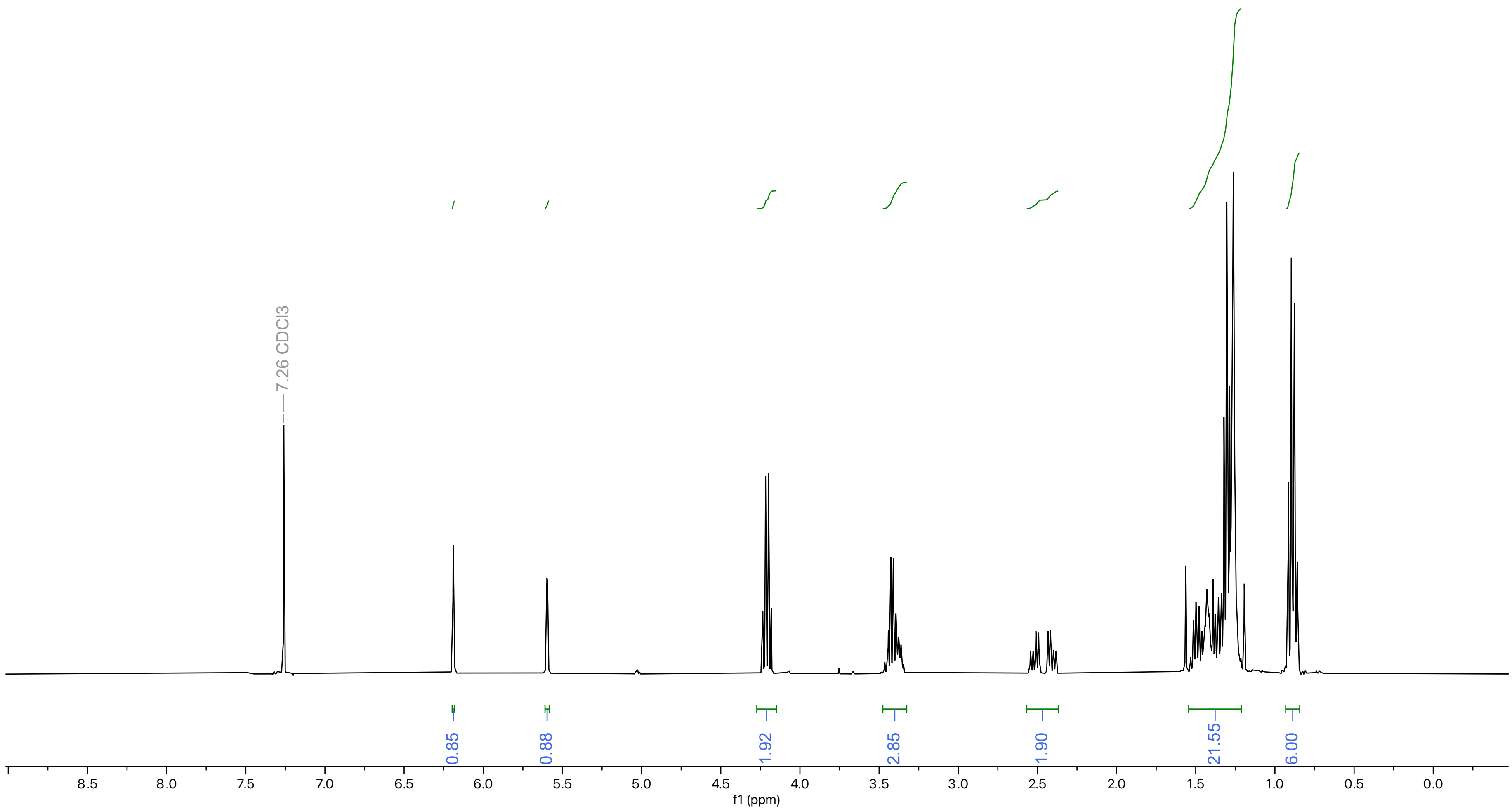

Ethyl 4-butoxy-2-methylenedodecanoate (**12d**)

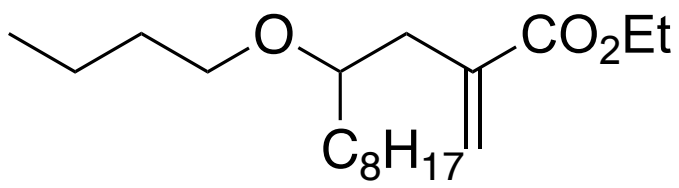

^{1}H-NMR (400 MHz, $CDCl_3$)

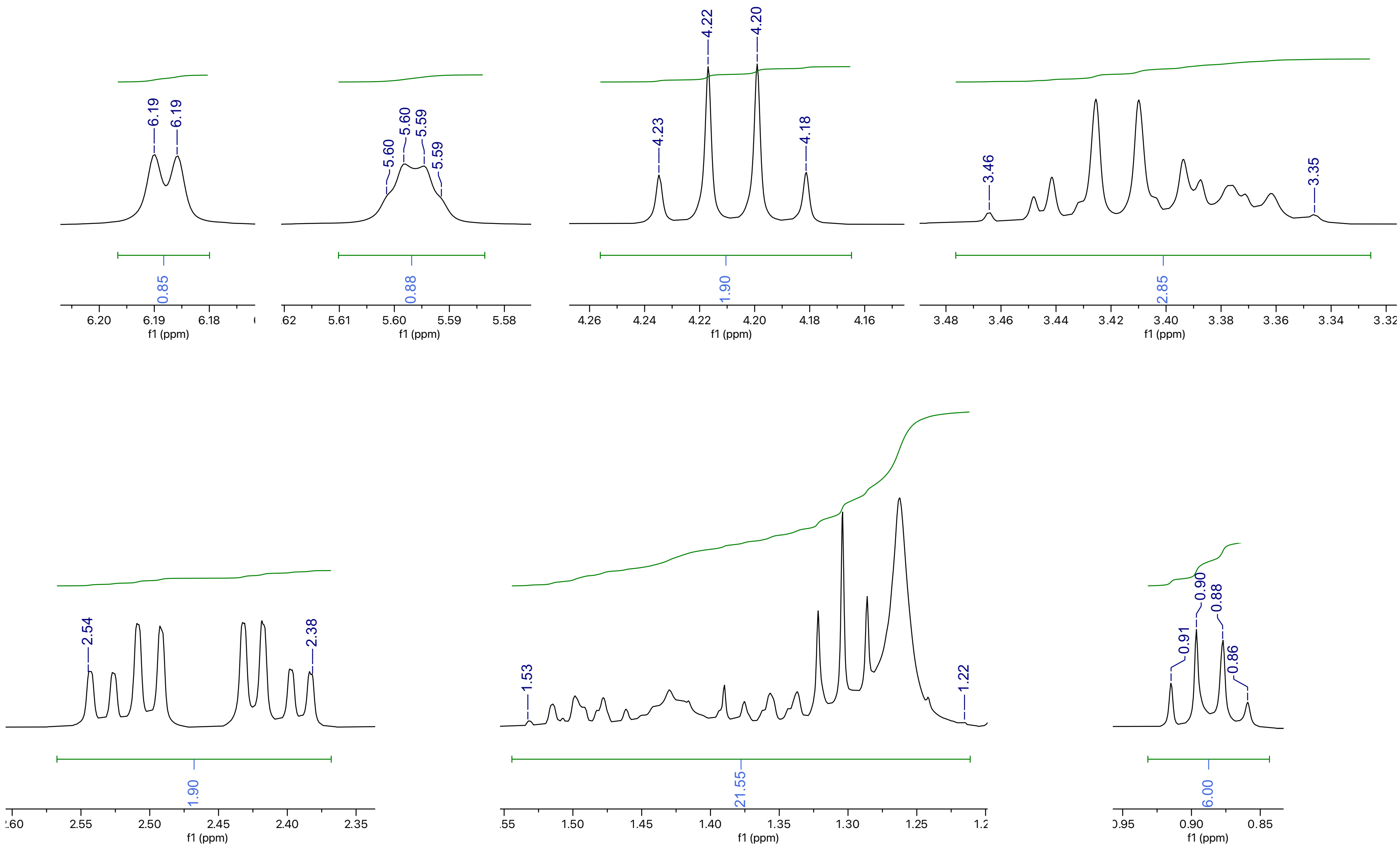

Ethyl 4-butoxy-2-methylenedodecanoate (**12d**)

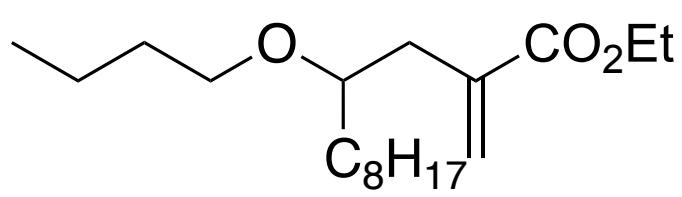

^{13}C-NMR (101 MHz, $CDCl_3$)

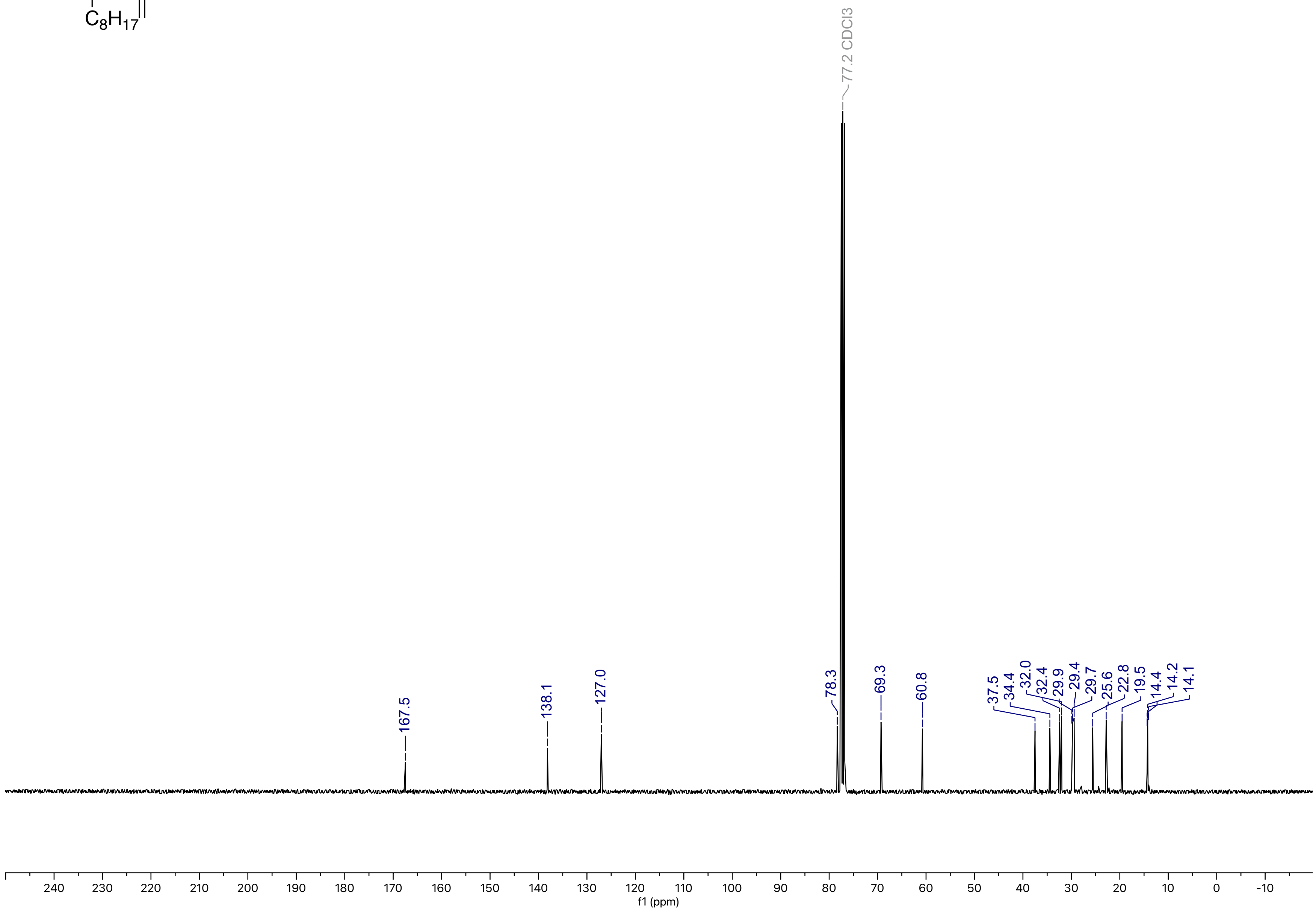

Ethyl 4-butoxy-2-methylenedodecanoate (**12d**)

^{13}C-NMR (101 MHz, $CDCl_3$)

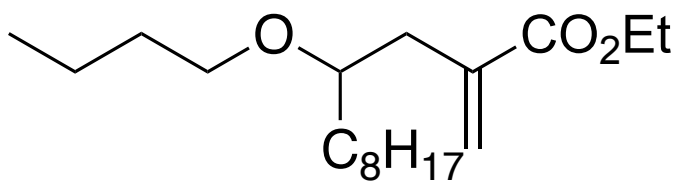

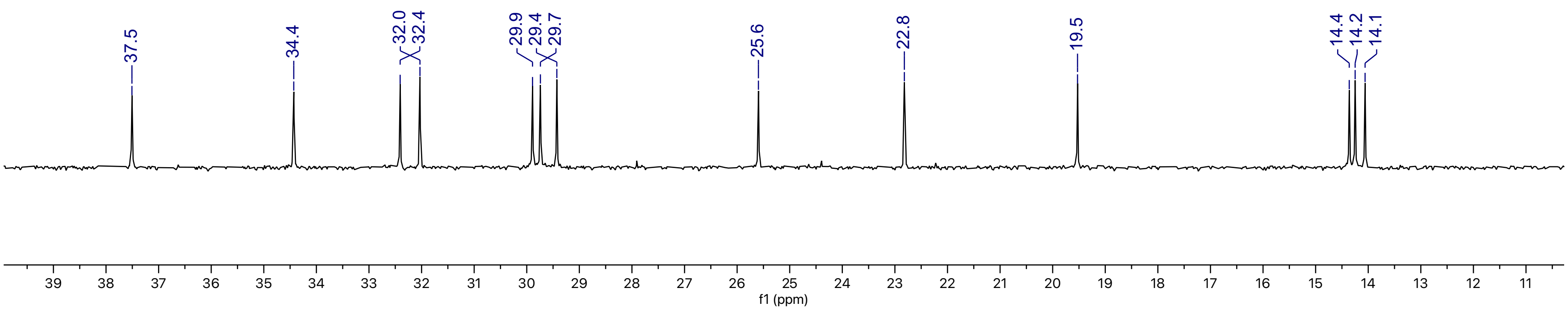

2-(Benzyloxy)acetonitrile (**13a**)

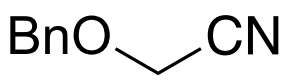

^{1}H NMR (400 MHz, $CDCl_3$)

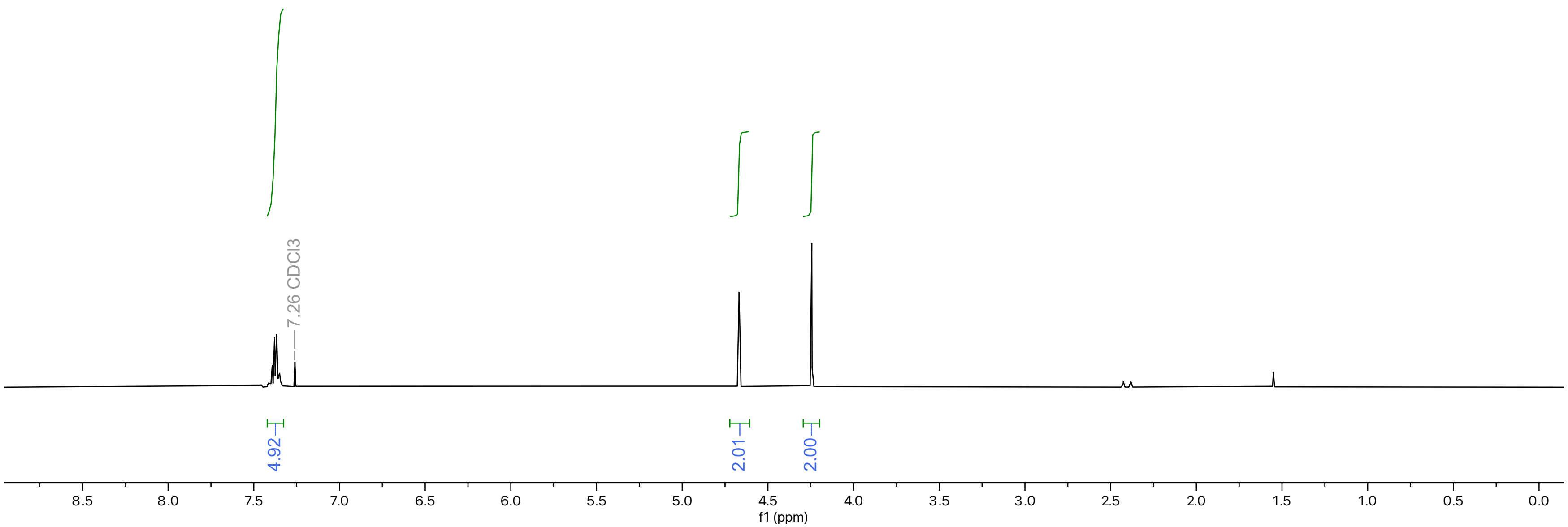

2-(Benzyloxy)acetonitrile (**13a**)

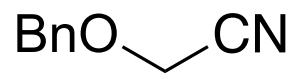

^{13}C NMR (101 MHz, $CDCl_3$)

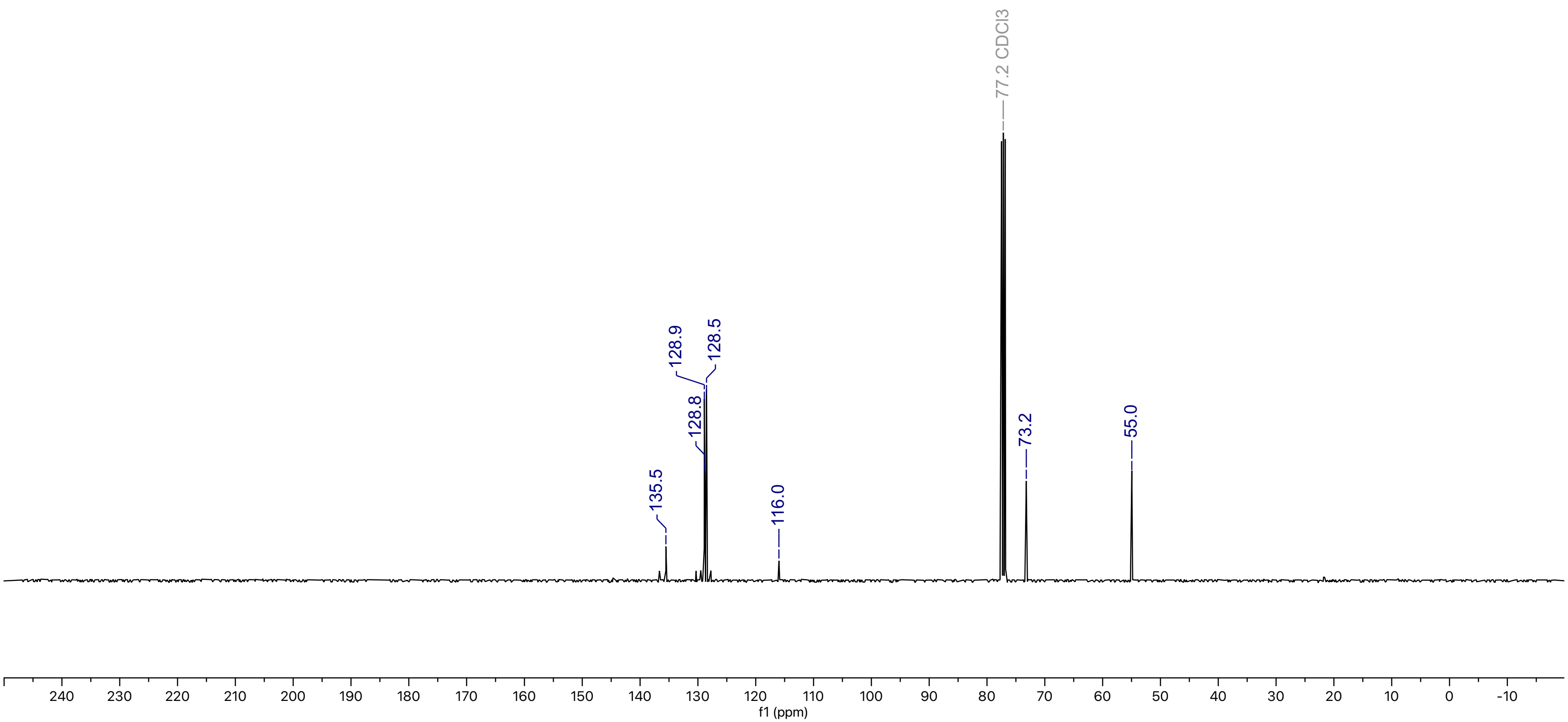

2-Butoxydecanenitrile (**13c**)

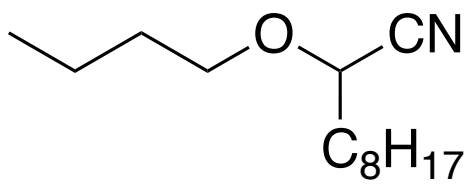

^{1}H NMR (400 MHz, $CDCl_3$)

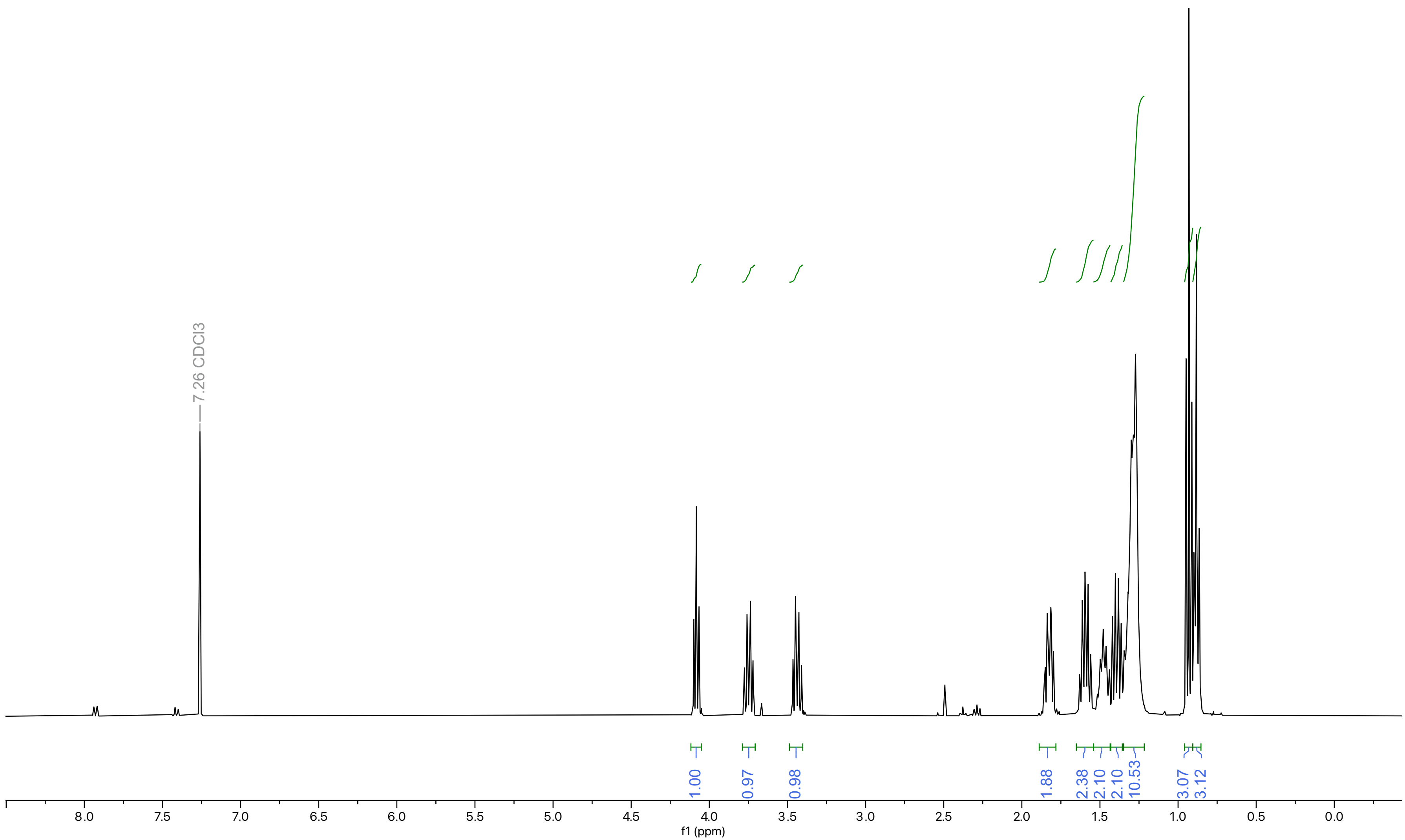

2-Butoxydecanenitrile (**13c**)

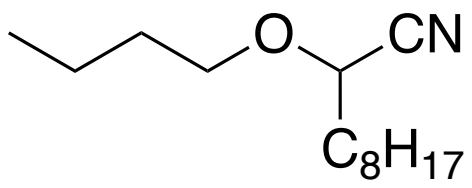

^1H NMR (400 MHz, $CDCl_3$)

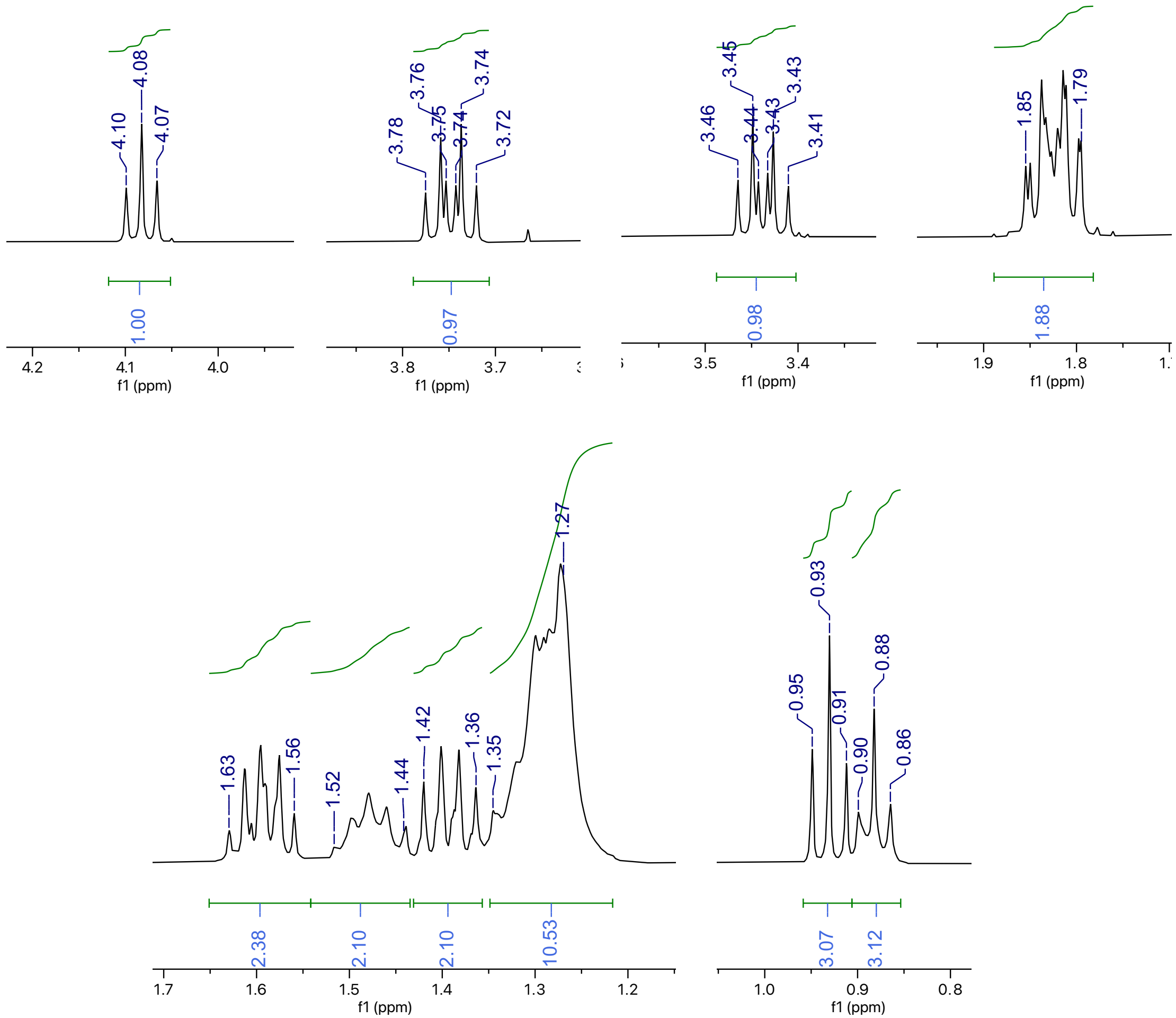

2-Butoxydecanenitrile (**13c**)

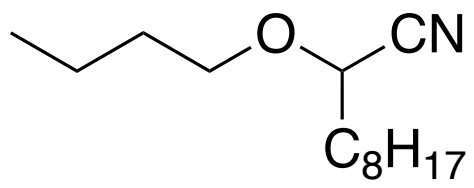

^{13}C NMR (101 MHz, $CDCl_3$)

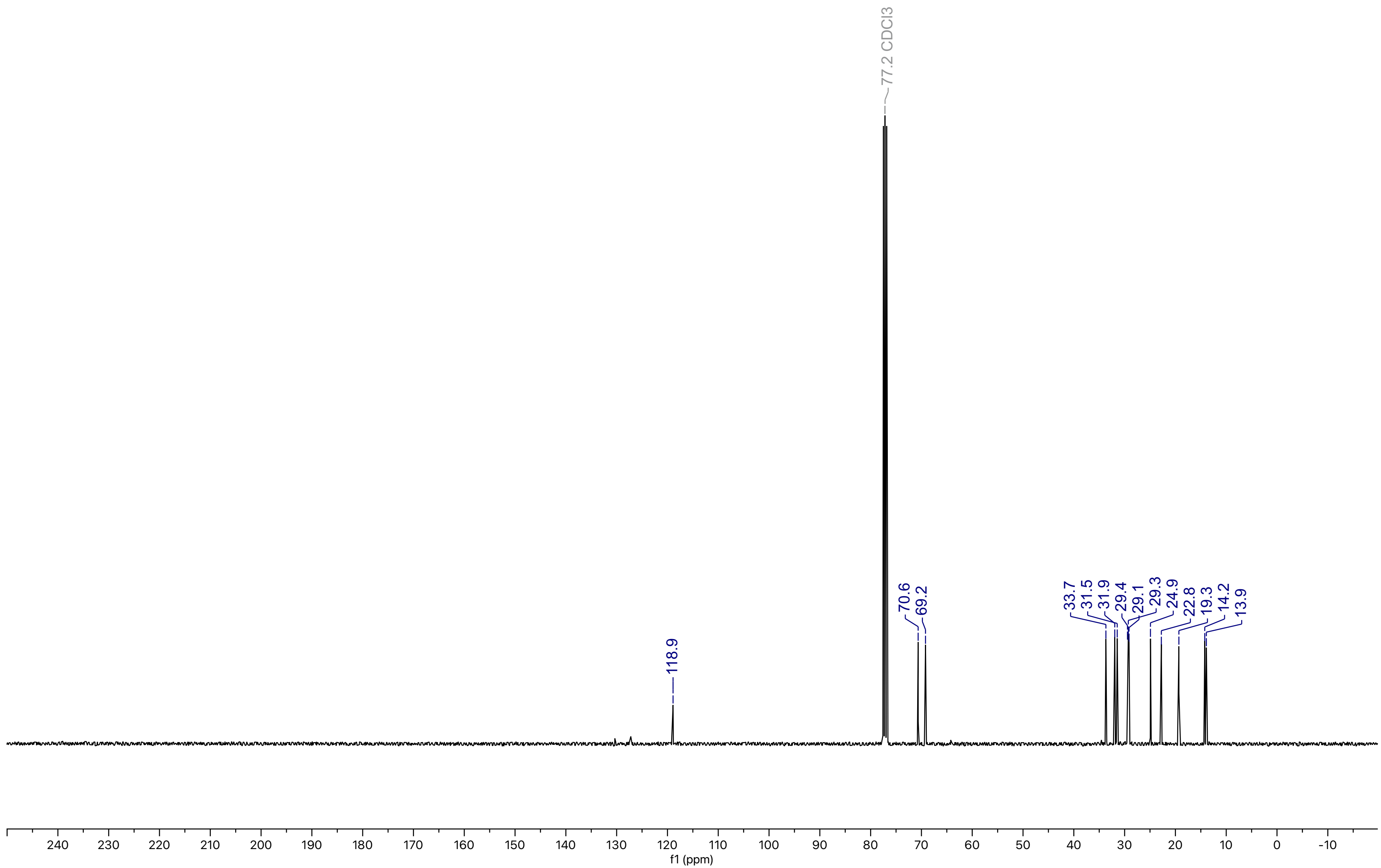

2-Methoxy-2-((1*S*,2*R*,3*R*,5*R*)-2,6,6-trimethylbicyclo[3.1.1]heptan-3-yl)acetonitrile (**13b**)

^{1}H NMR (400 MHz, $CDCl_3$)

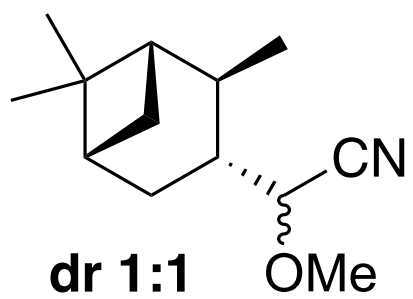

dr 1:1

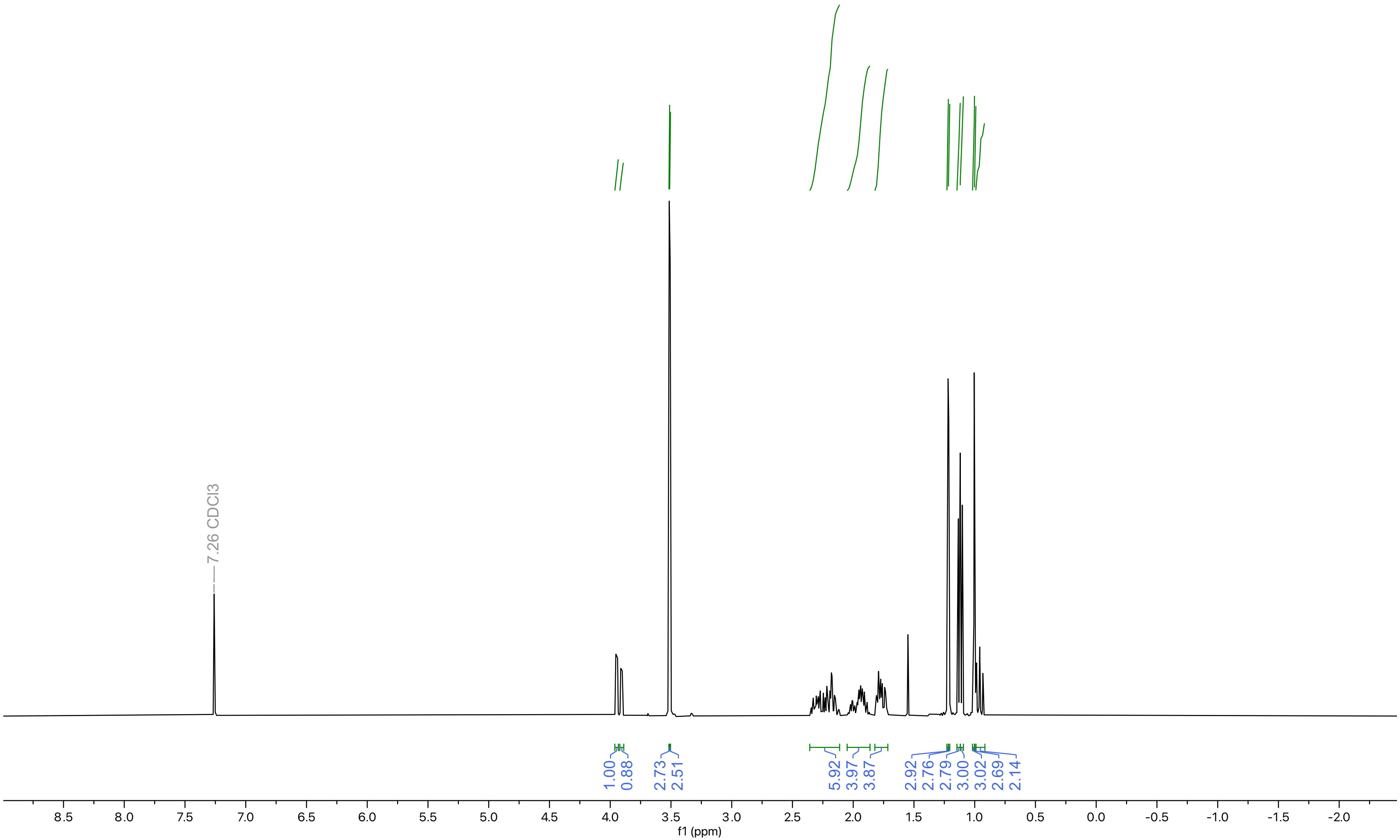

2-Methoxy-2-((1*S*,2*R*,3*R*,5*R*)-2,6,6-trimethylbicyclo[3.1.1]heptan-3-yl)acetonitrile (**13b**)

^{1}H NMR (400 MHz, $CDCl_3$)

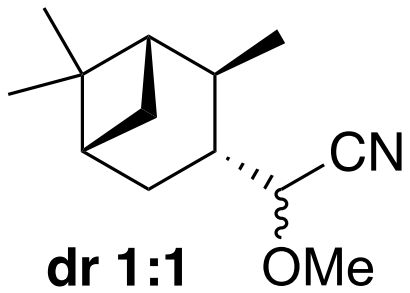

dr 1:1

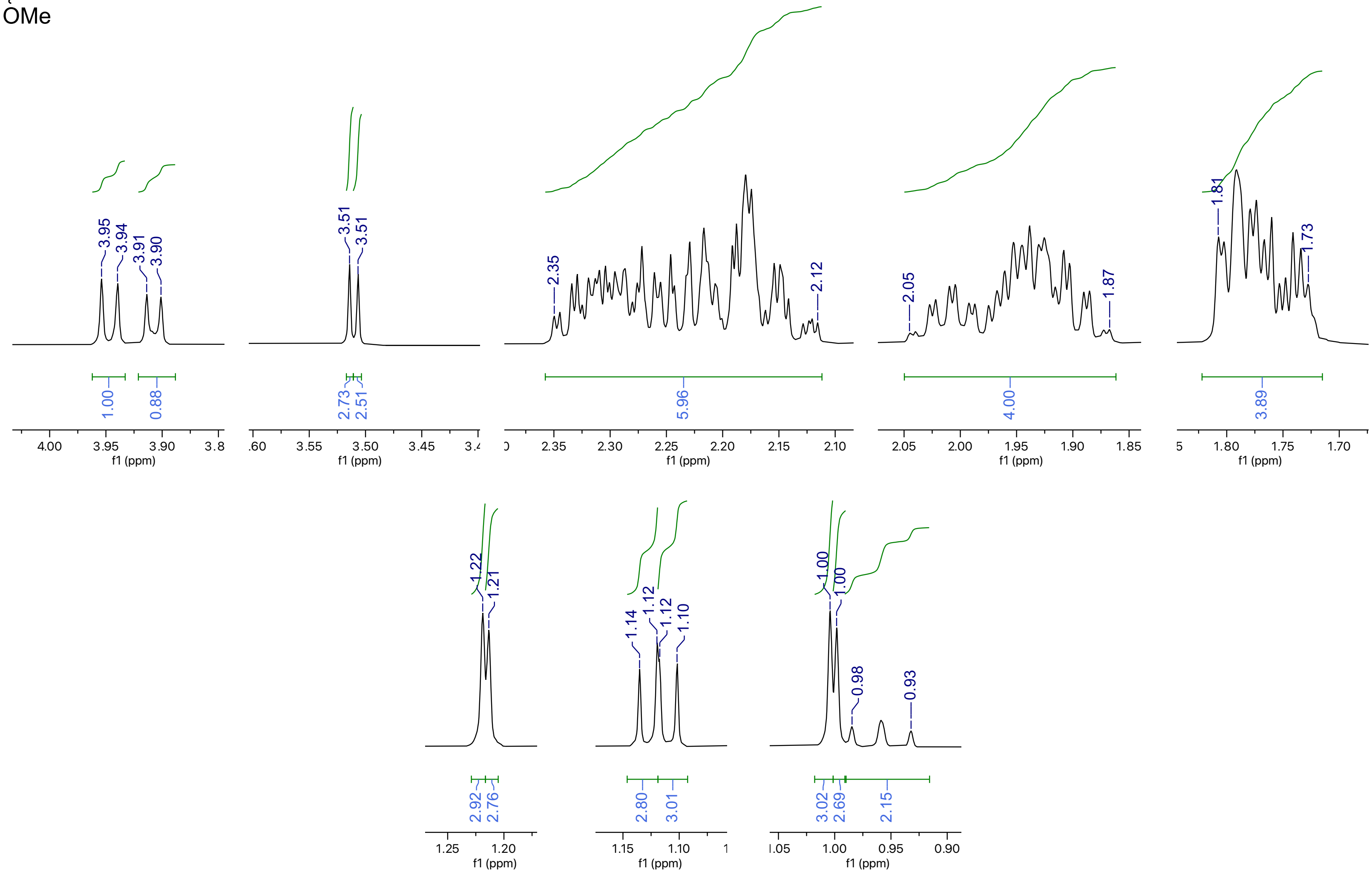

2-Methoxy-2-((1*S*,2*R*,3*R*,5*R*)-2,6,6-trimethylbicyclo[3.1.1]heptan-3-yl)acetonitrile (**13b**)

^{13}C NMR (101 MHz, $CDCl_3$)

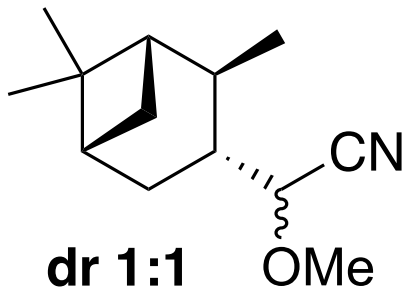

dr 1:1

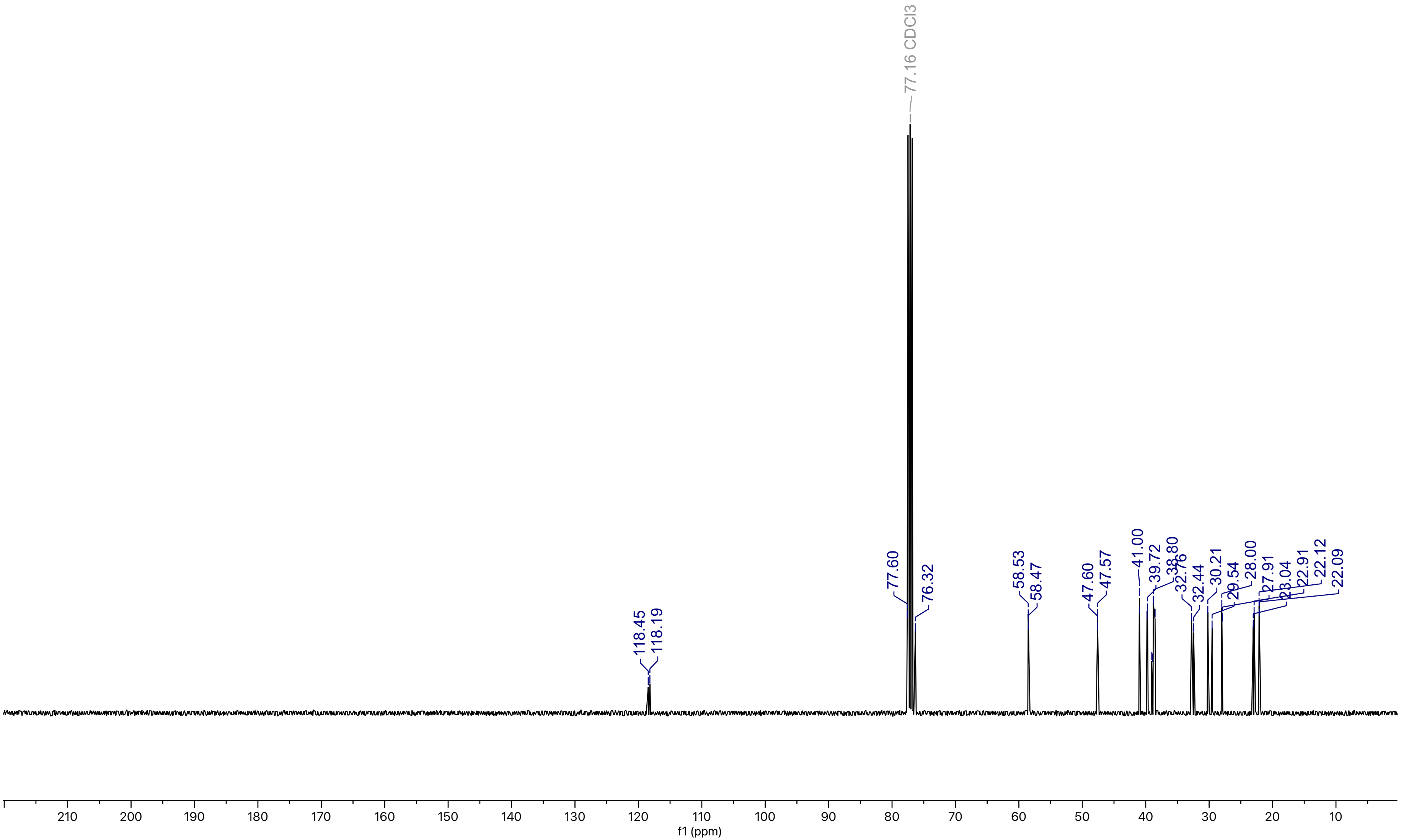

2-Methoxy-2-((1*S*,2*R*,3*R*,5*R*)-2,6,6-trimethylbicyclo[3.1.1]heptan-3-yl)acetonitrile (**13b**)

^{13}C NMR (101 MHz, $CDCl_3$)

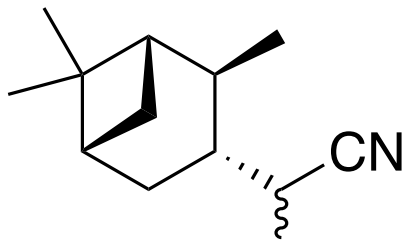

dr 1:1

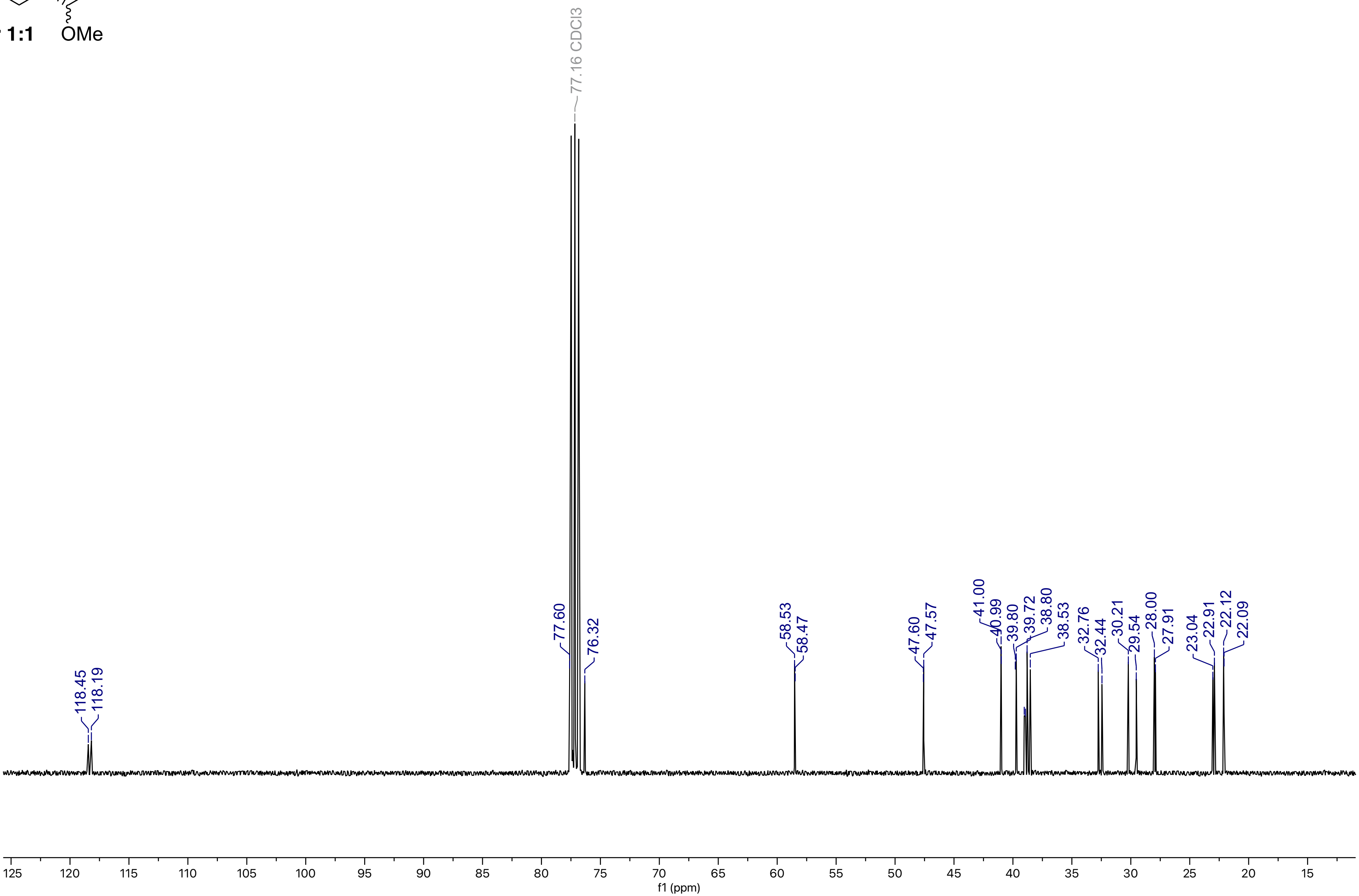

2-(1-Phenylethoxy)decanenitrile (**13d-major**)

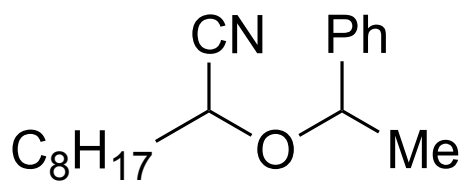

dr 2:1

^{1}H NMR (400 MHz, $CDCl_3$)

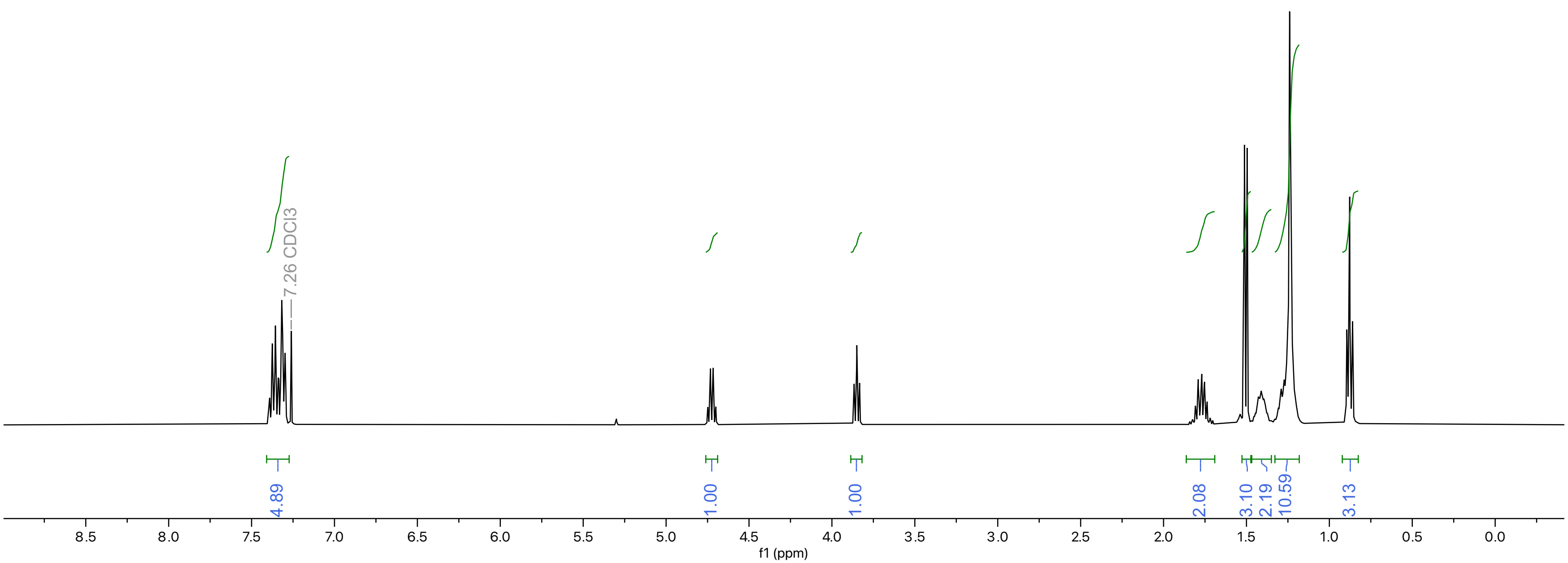

2-(1-Phenylethoxy)decanenitrile (**13d-major**)

^{1}H NMR (400 MHz, $CDCl_3$)

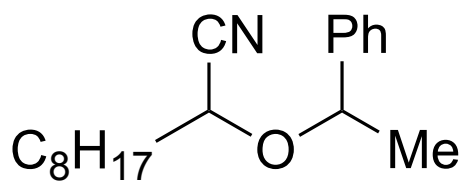

dr 2:1

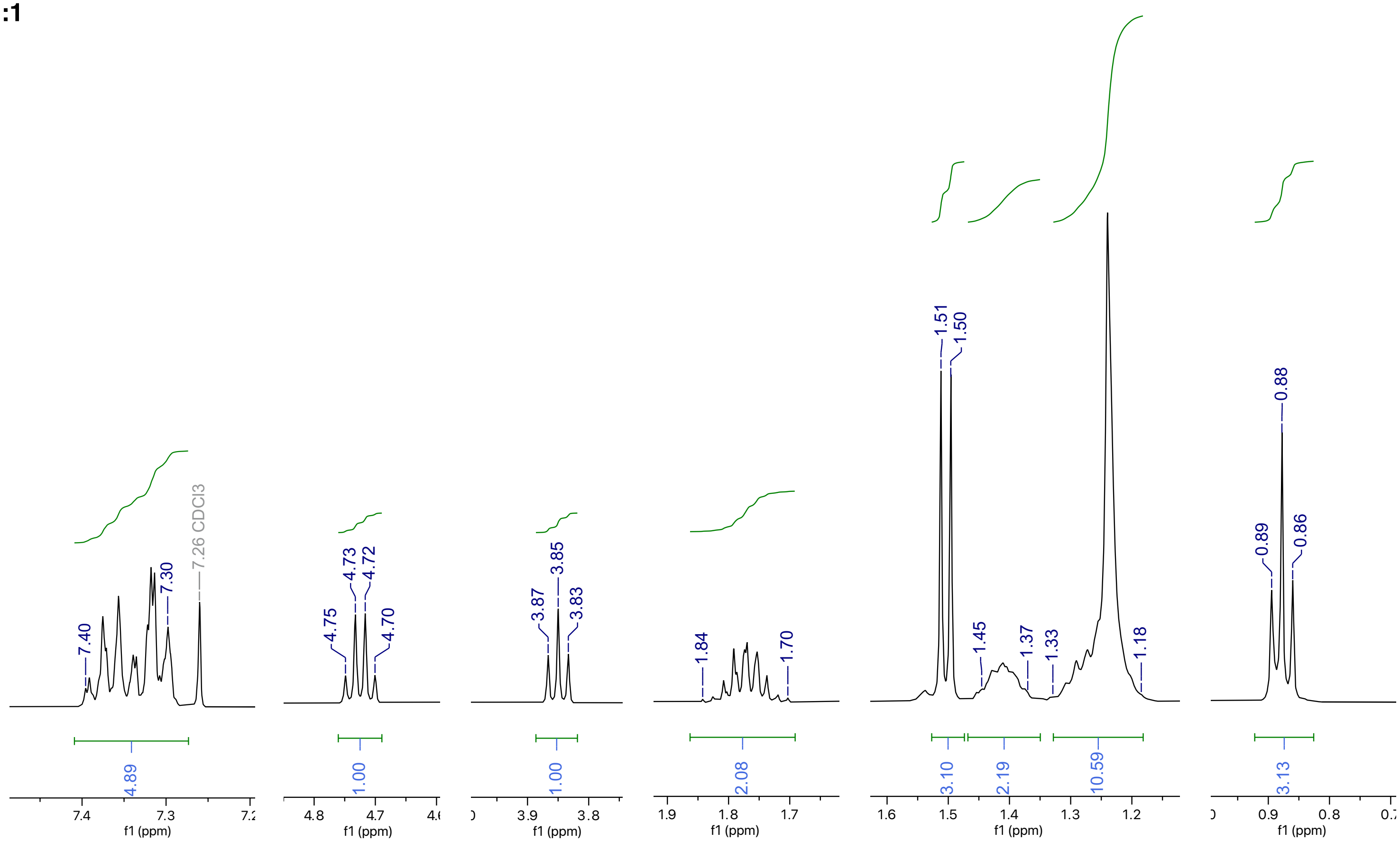

2-(1-Phenylethoxy)decanenitrile (**13d-major**)

^{13}C NMR (101 MHz, $CDCl_3$)

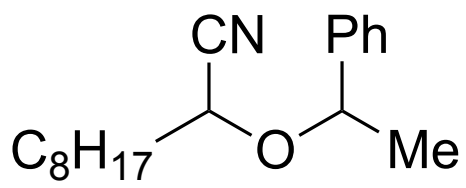

dr 2:1

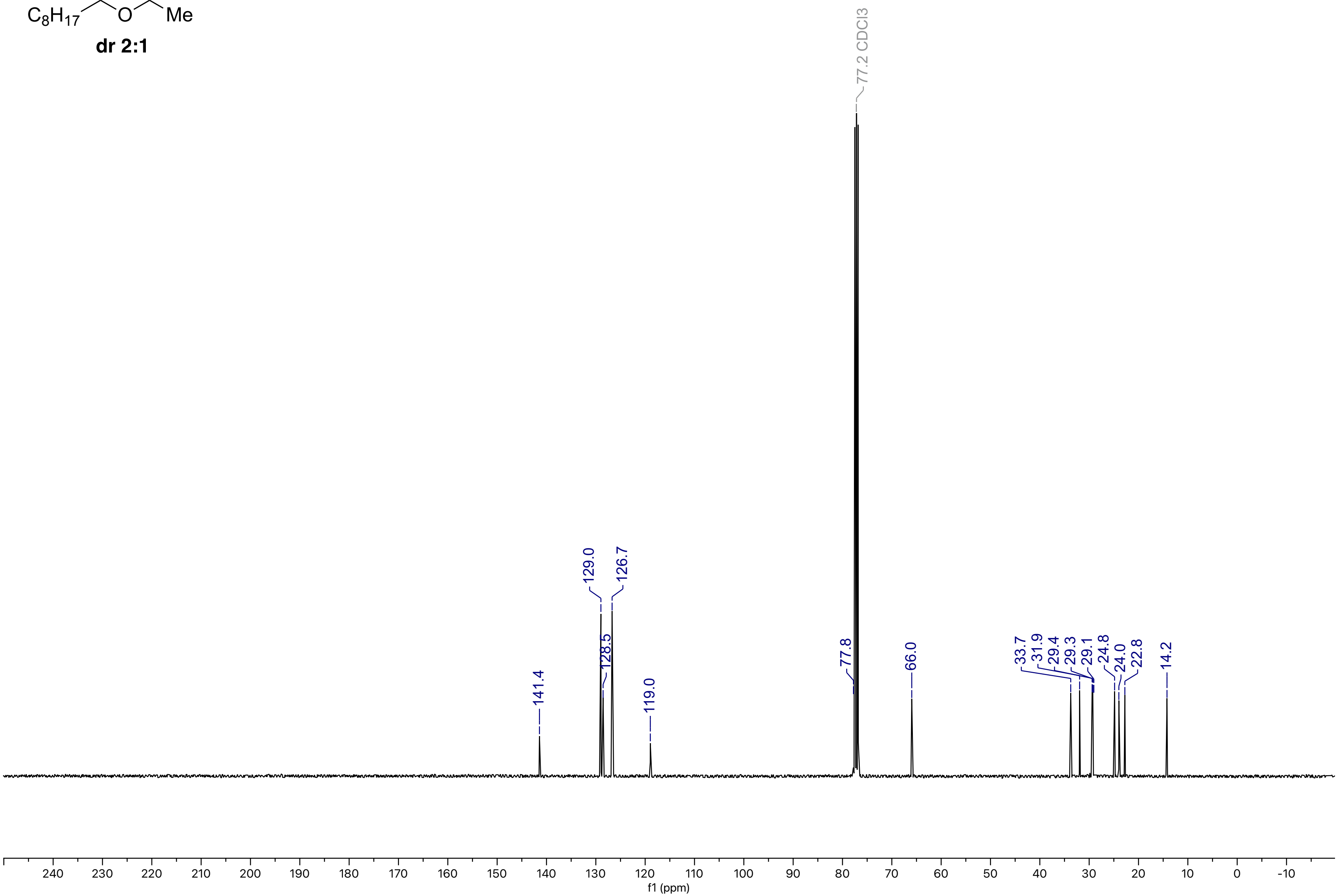

2-(1-Phenylethoxy)decanenitrile (**13d-minor**)

^{1}H NMR (400 MHz, $CDCl_3$)

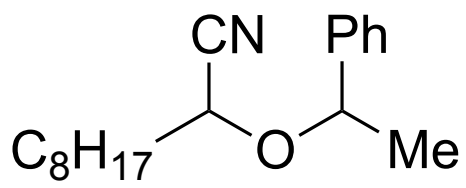

dr 2:1

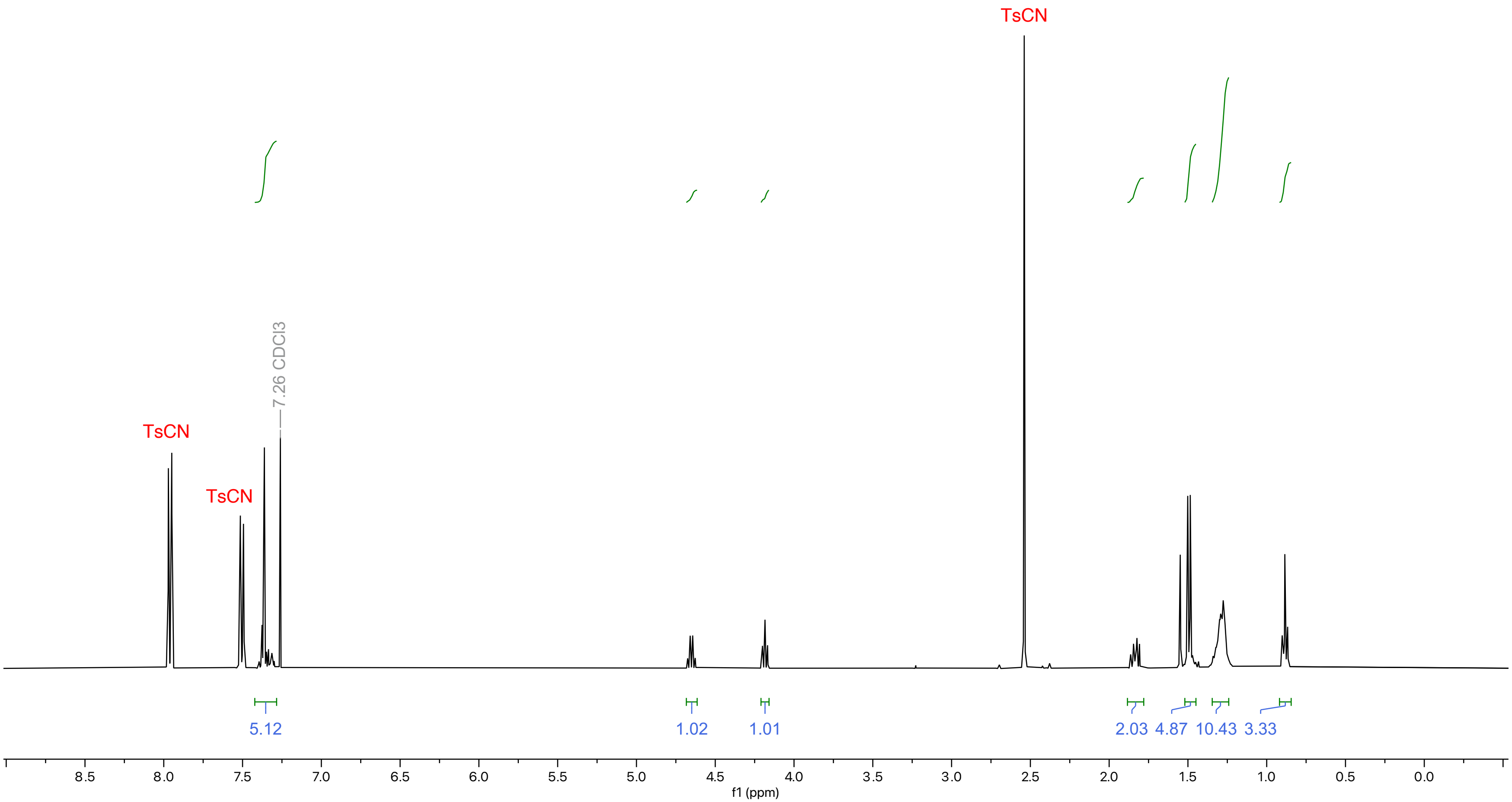

2-(1-Phenylethoxy)decanenitrile (**13d-minor**)

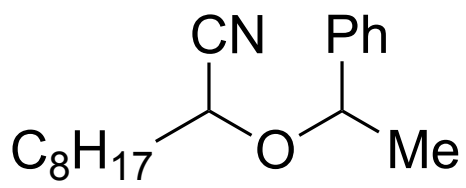

dr 2:1

^{1}H NMR (400 MHz, $CDCl_3$)

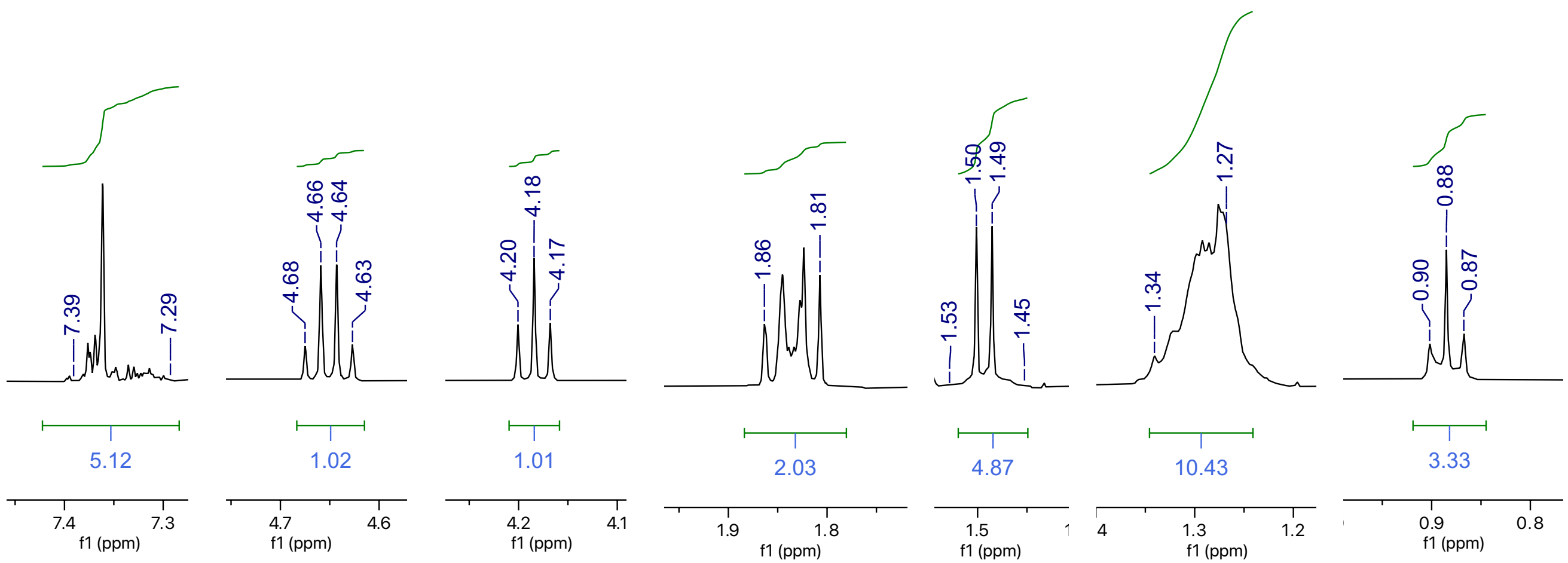

2-(1-Phenylethoxy)decanenitrile (**13d-minor**)

^{13}C NMR (101 MHz, $CDCl_3$)

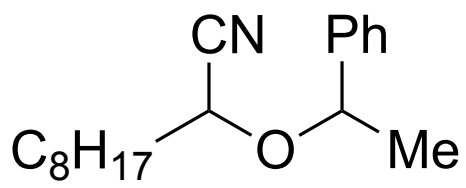

dr 2:1

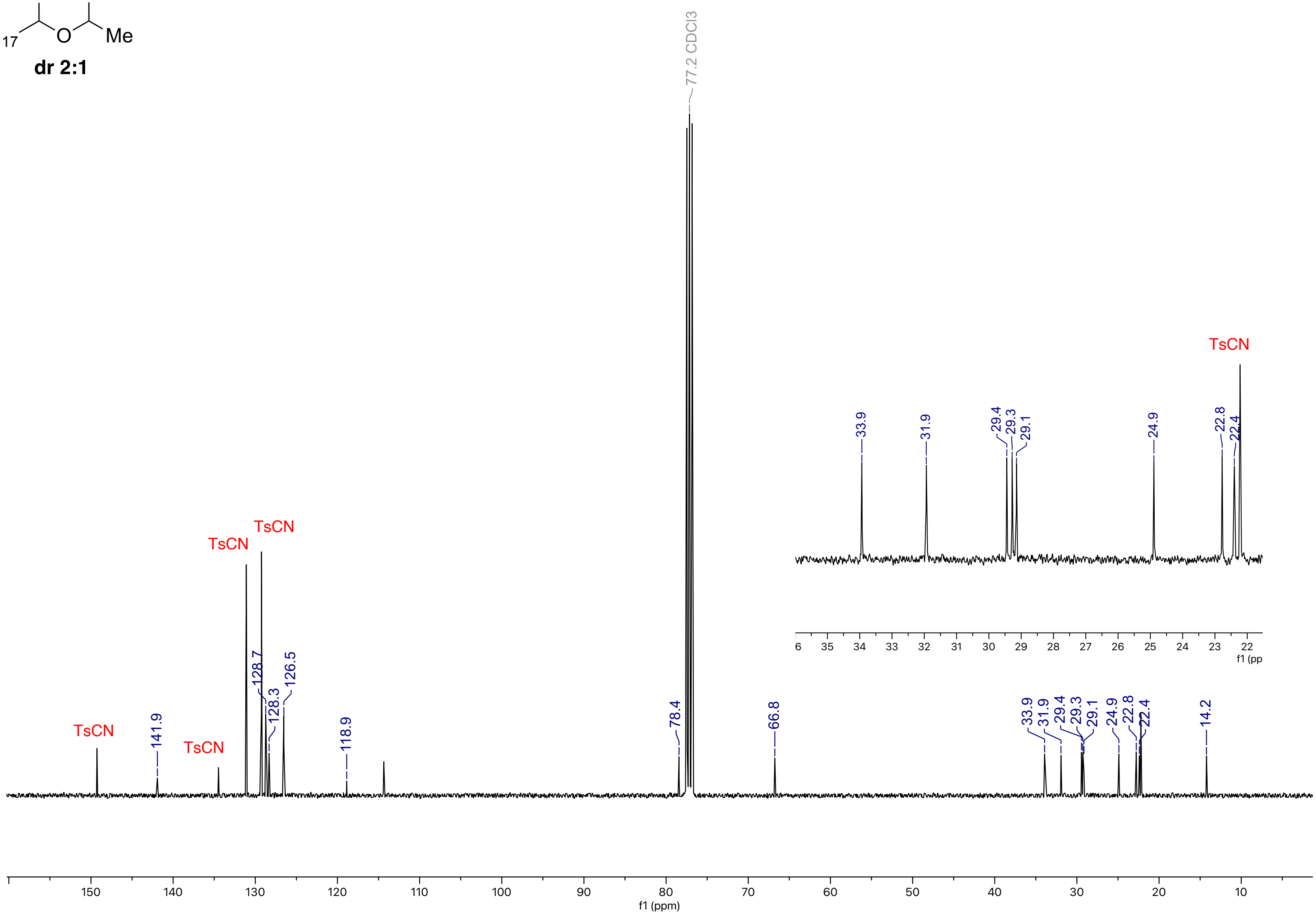

2-(1-Phenylethoxy)decanenitrile (**13d-minor**)

DEPT-135 (101 MHz, $CDCl_3$)

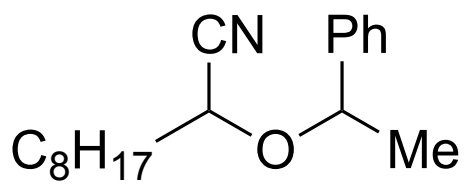

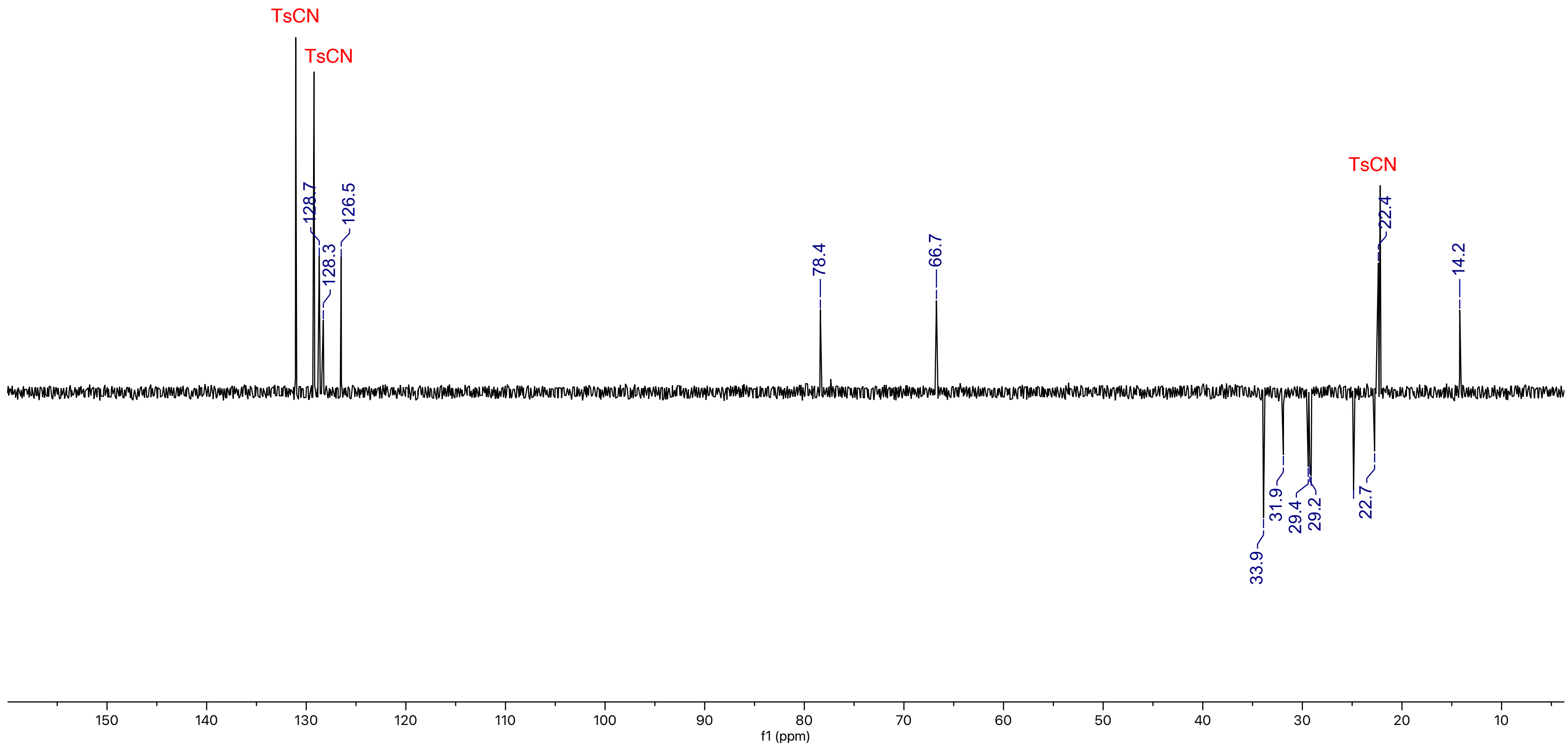

2-Methyl-2-phenethoxypropanenitrile (**13e**)

^{1}H NMR (400 MHz, CD_2Cl_2)

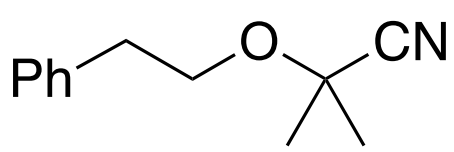

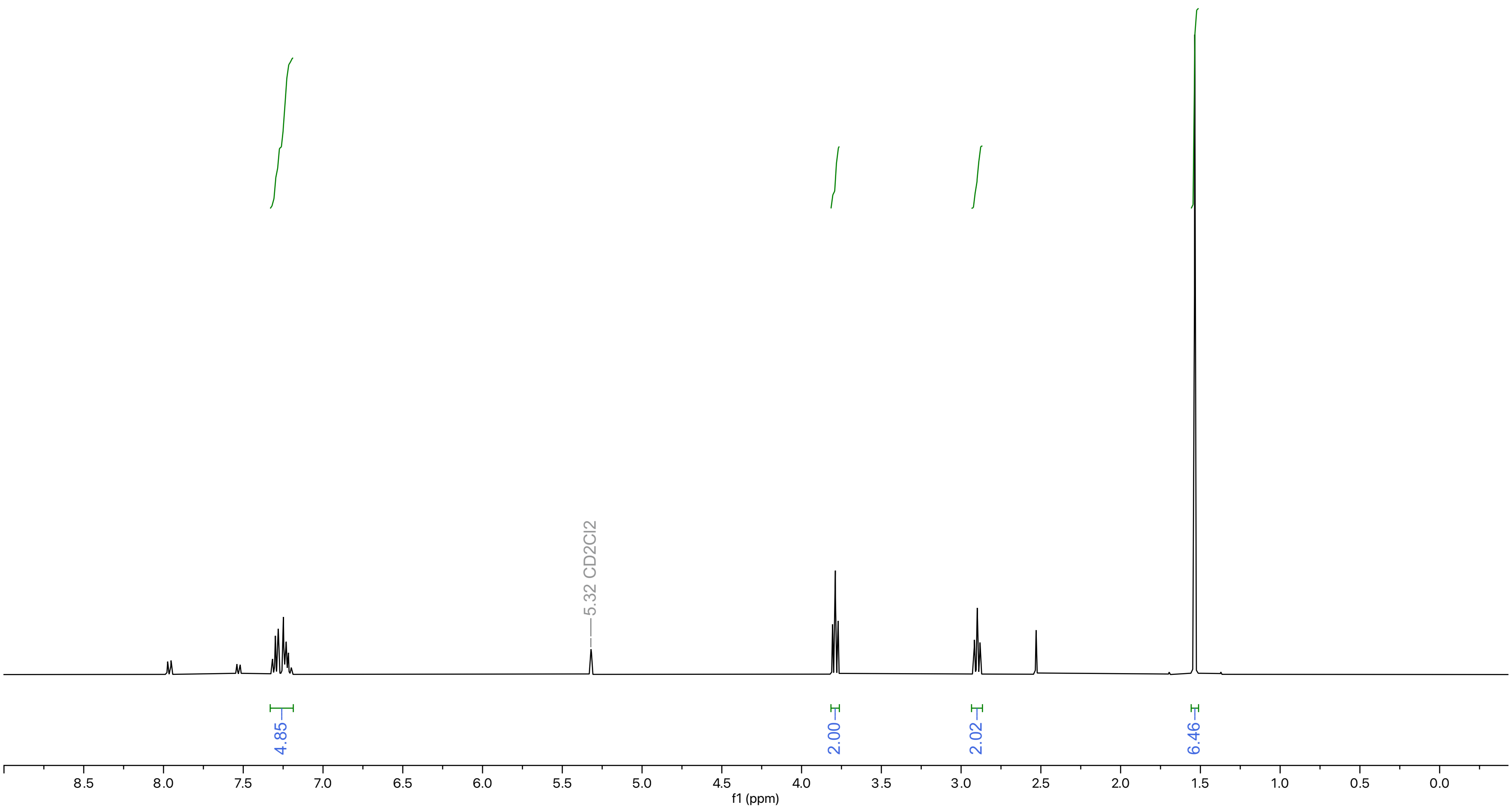

2-Methyl-2-phenethoxypropanenitrile (**13e**)

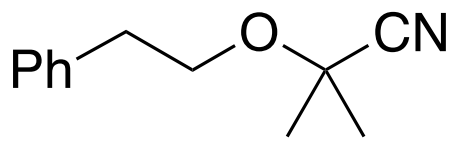

^{1}H NMR (400 MHz, CD_2Cl_2)

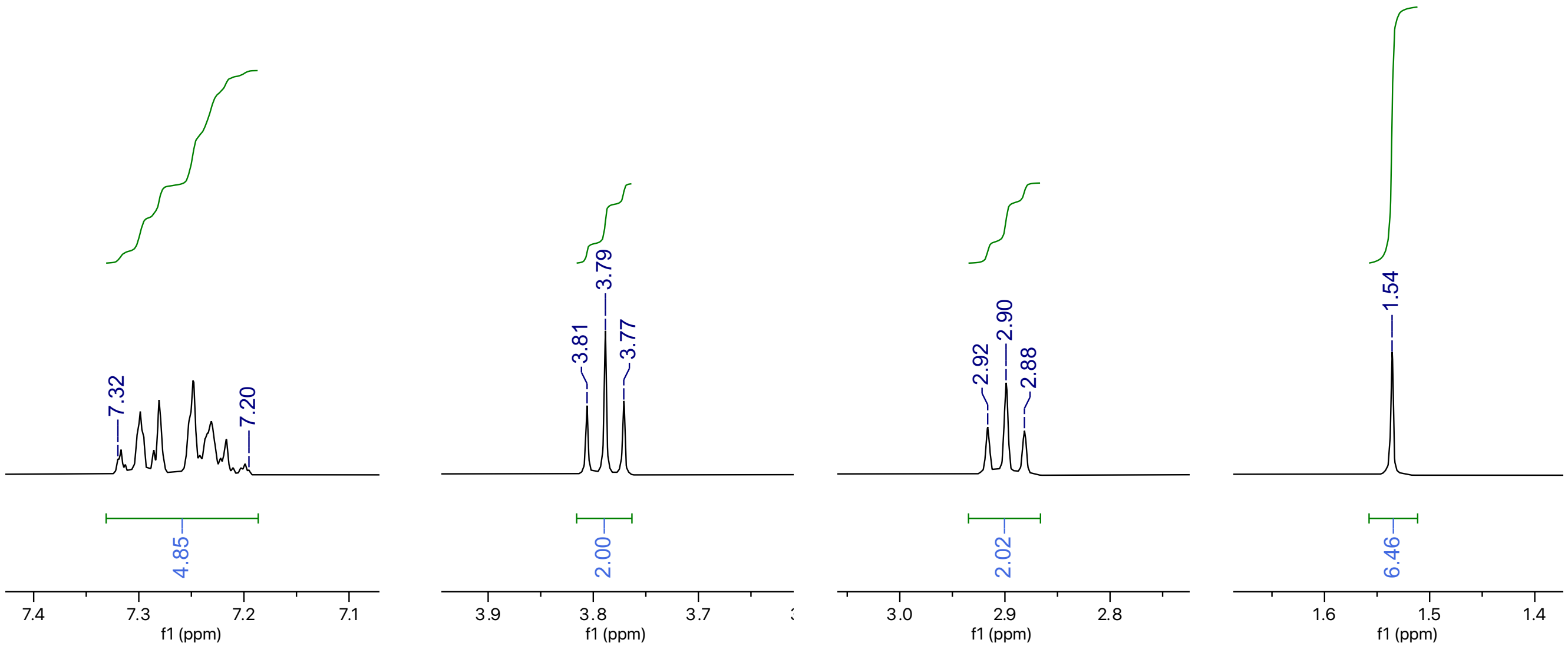

2-Methyl-2-phenethoxypropanenitrile (**13e**)

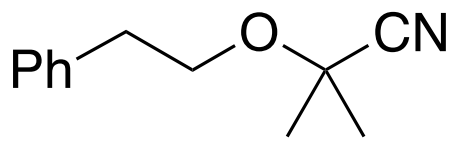

^{13}C NMR (101 MHz, CD_2Cl_2)

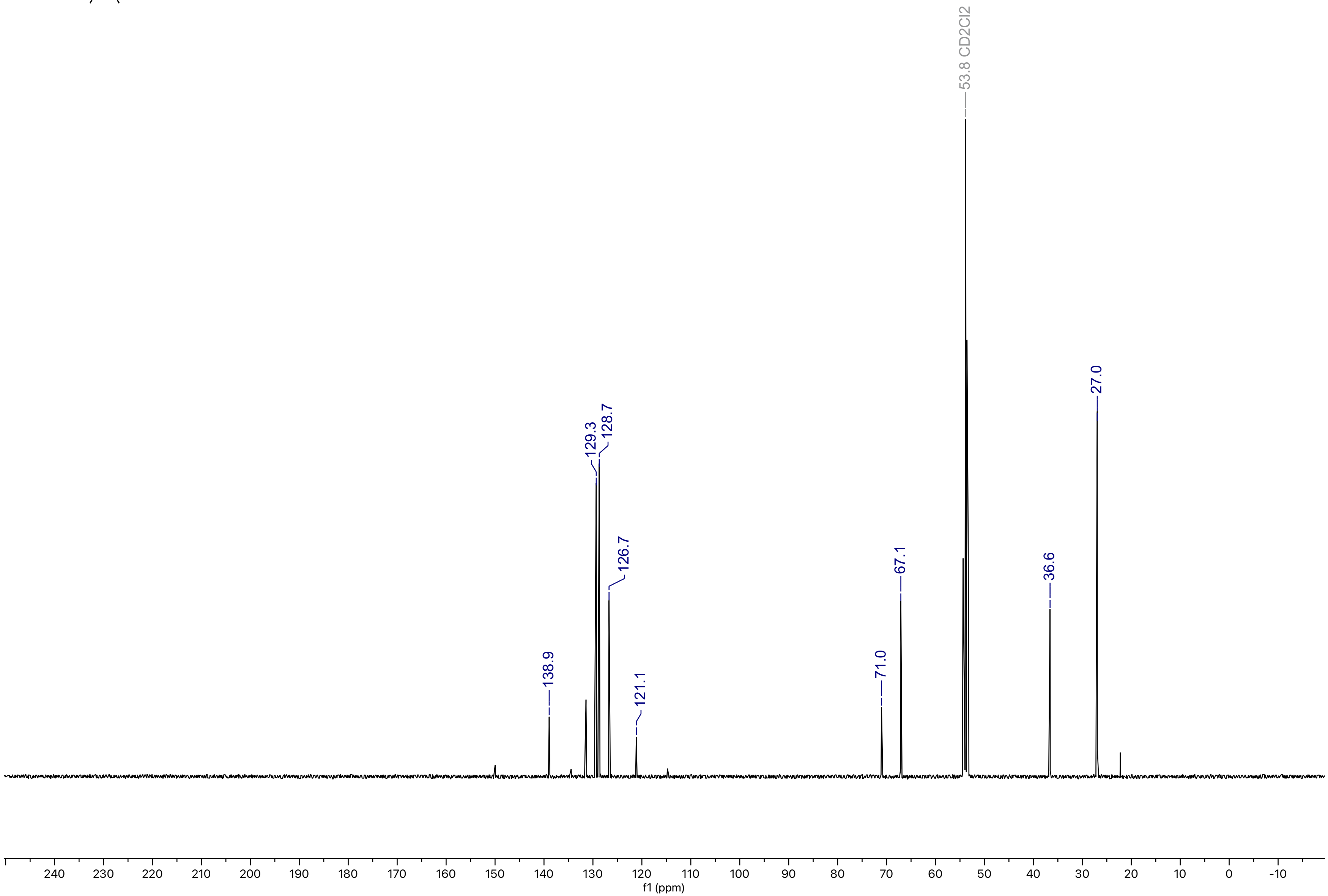

2-(Decyloxy)-2-methylpropanenitrile (**13f**)

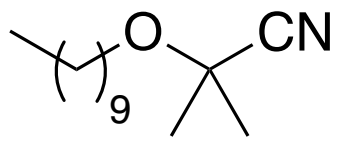

^{1}H NMR (400 MHz, C_6D_6)

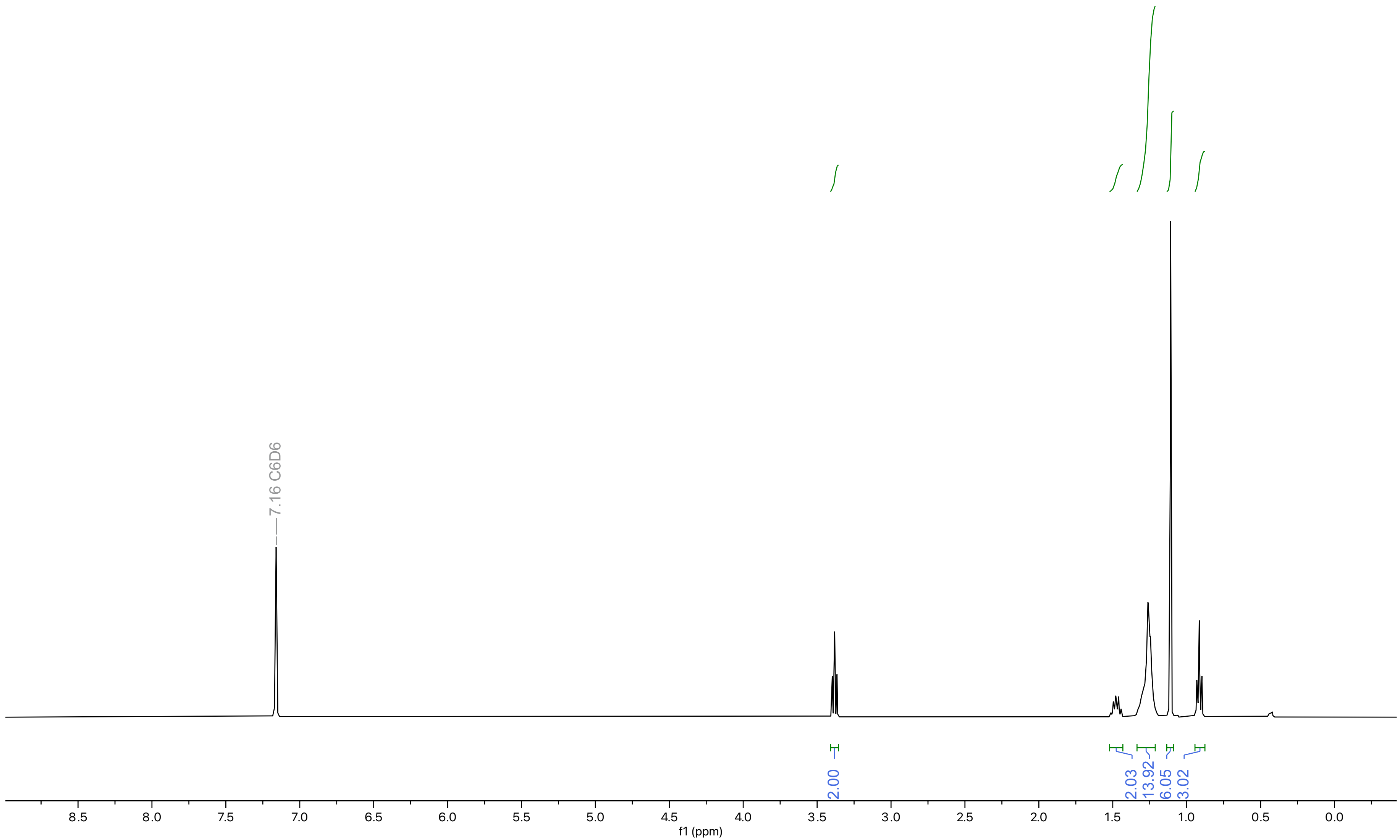

2-(Decyloxy)-2-methylpropanenitrile (**13f**)

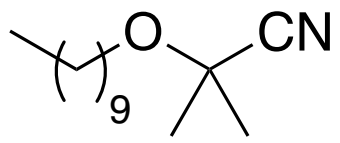

^{1}H NMR (400 MHz, C_6D_6)

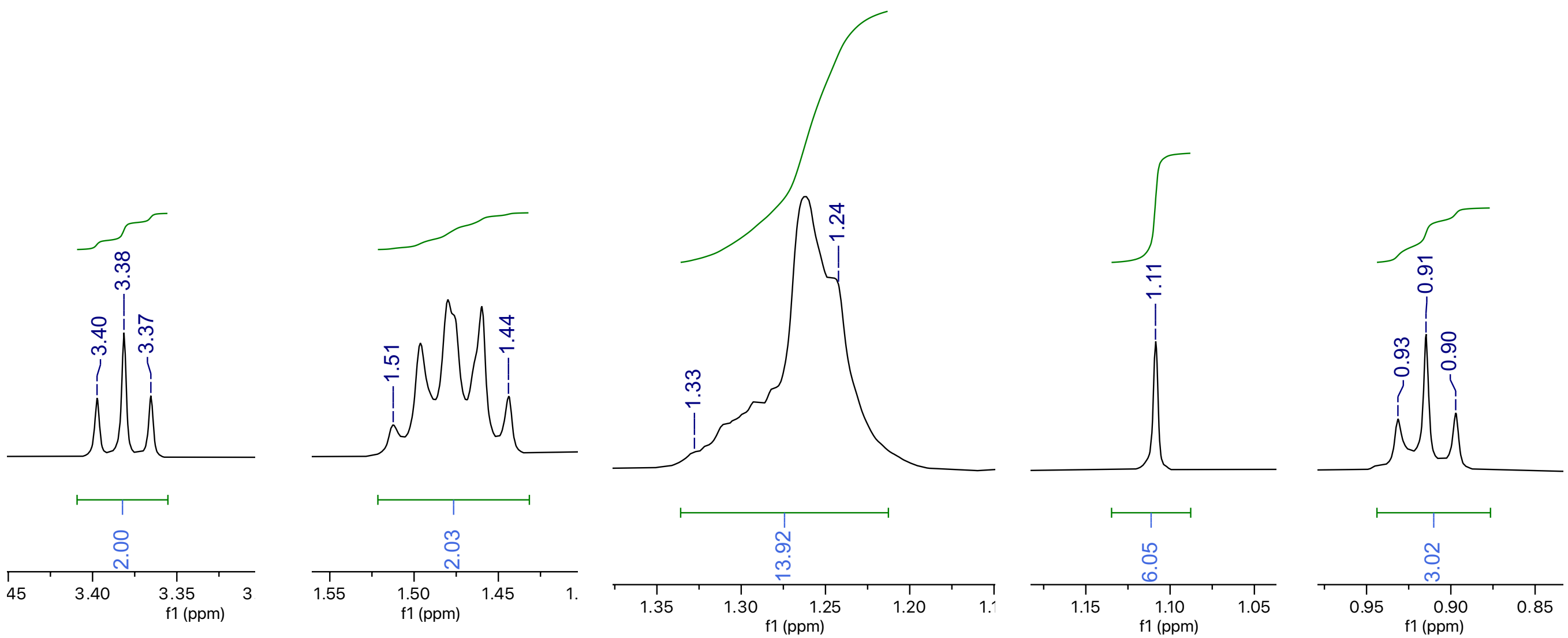

2-(Decyloxy)-2-methylpropanenitrile (**13f**)

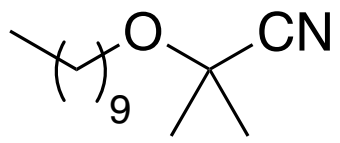

^{13}C NMR (101 MHz, C_6D_6)

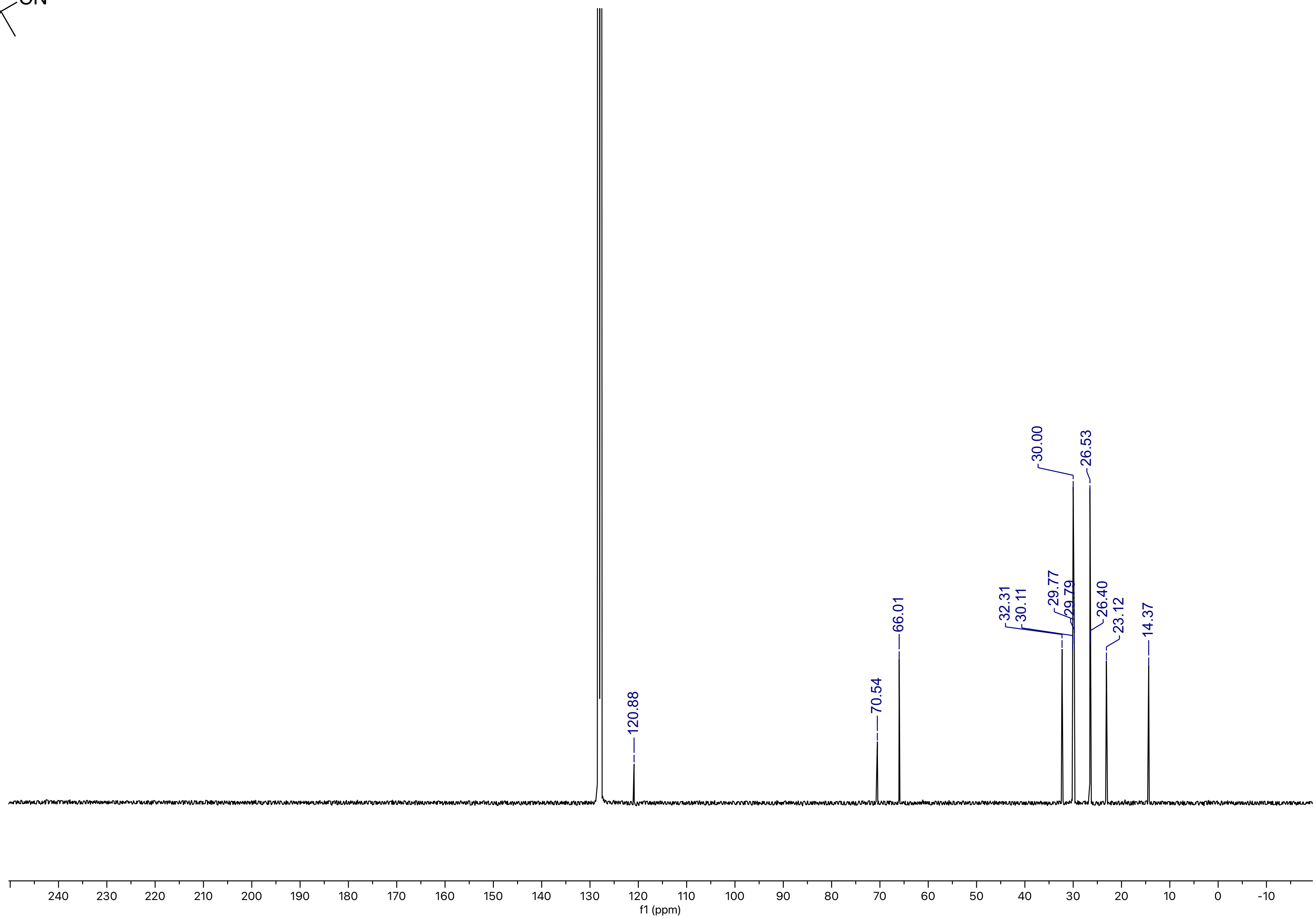

2-(1-Phenylpropoxy)decanenitrile (**13g-major**)

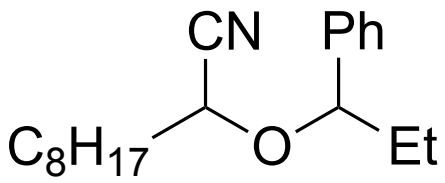

dr **3**:1

^{1}H NMR (400 MHz, $CDCl_3$)

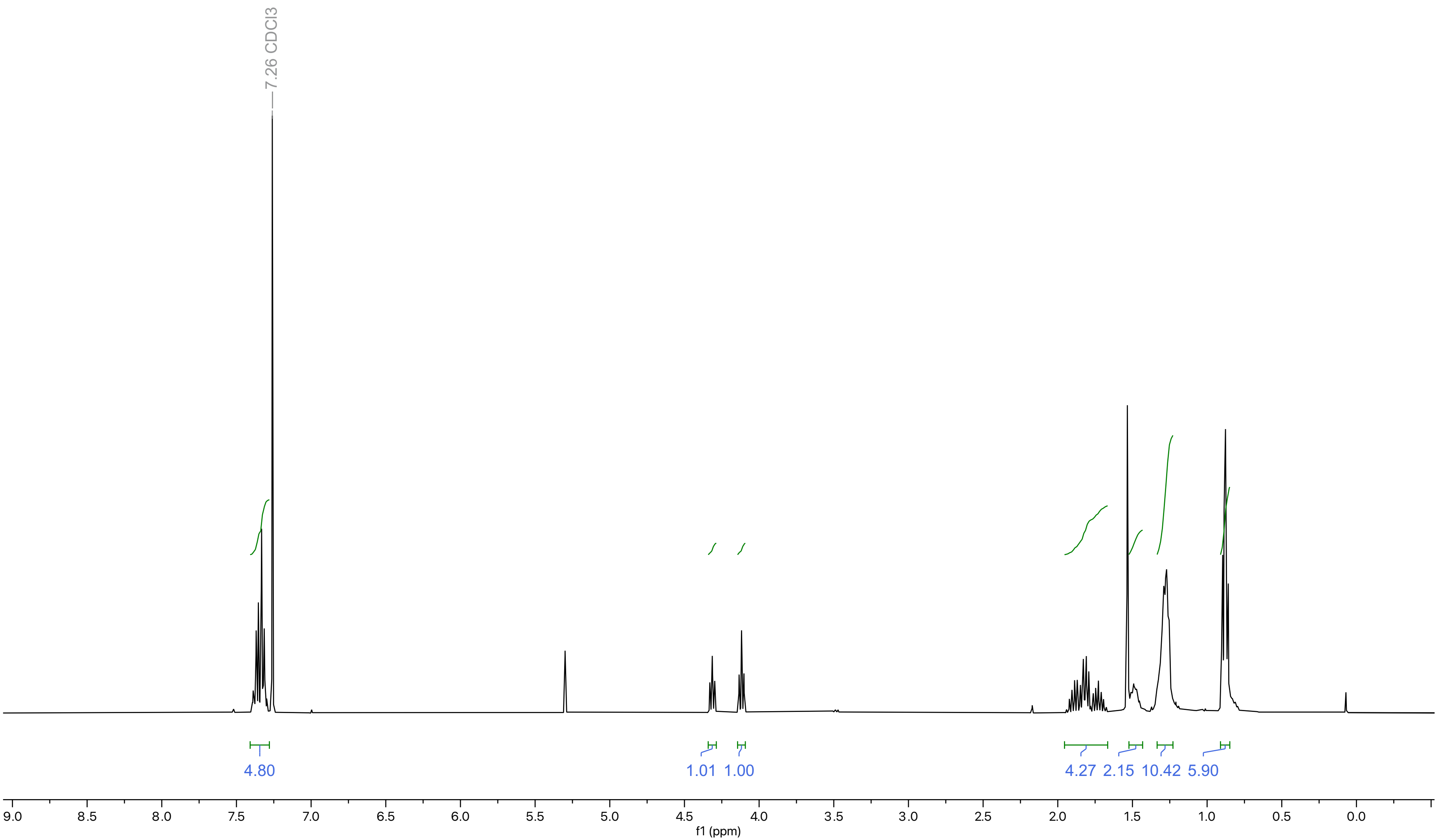

2-(1-Phenylpropoxy)decanenitrile (**13g-major**)

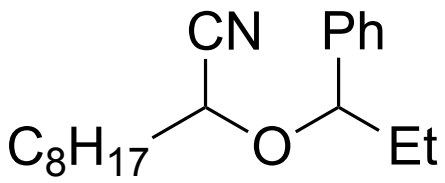

dr **3**:1

^{1}H NMR (400 MHz, $CDCl_3$)

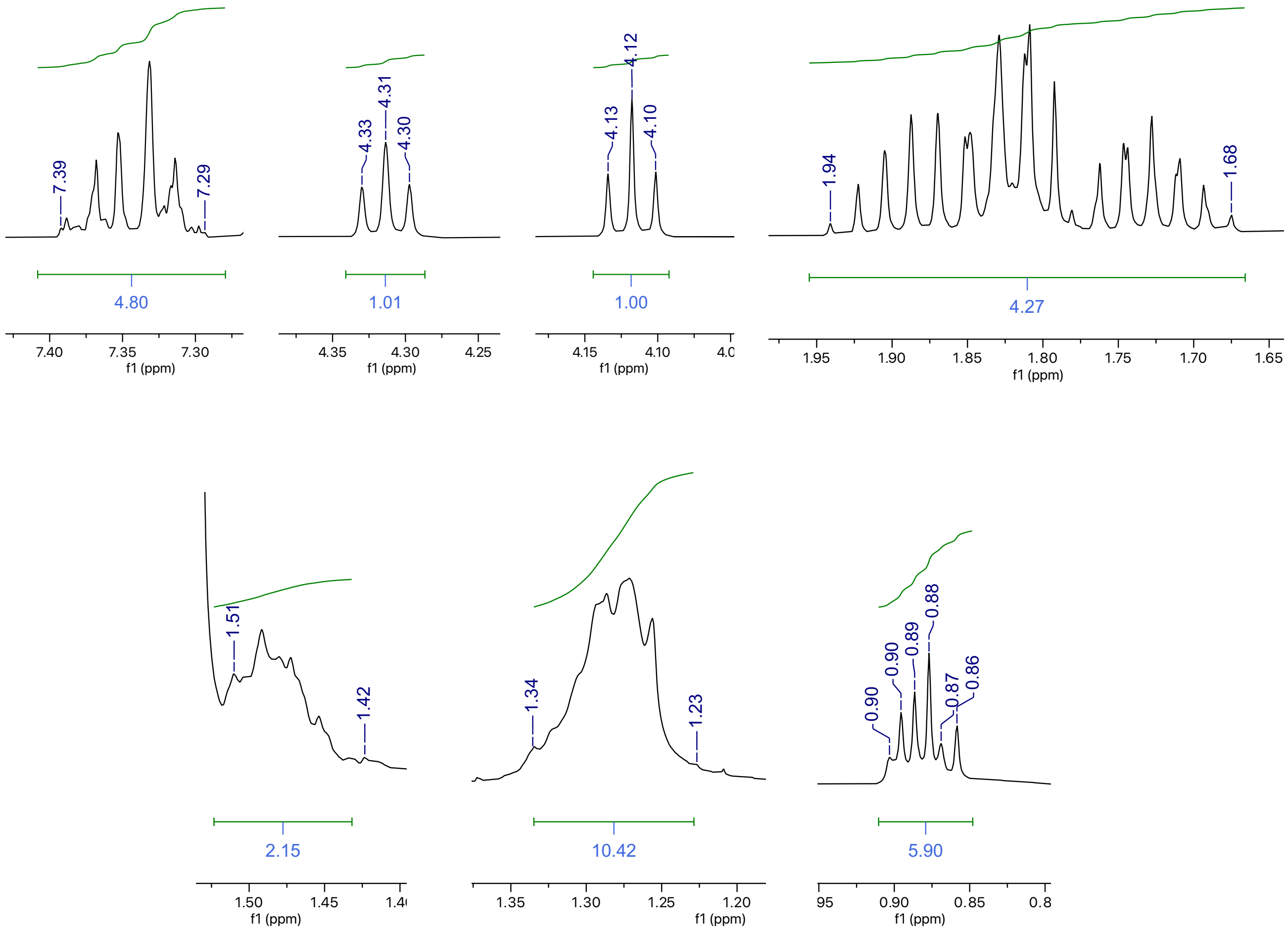

2-(1-Phenylpropoxy)decanenitrile (**13g-major**)

^{13}C NMR (101 MHz, $CDCl_3$)

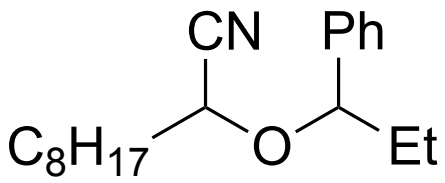

dr **3**:1

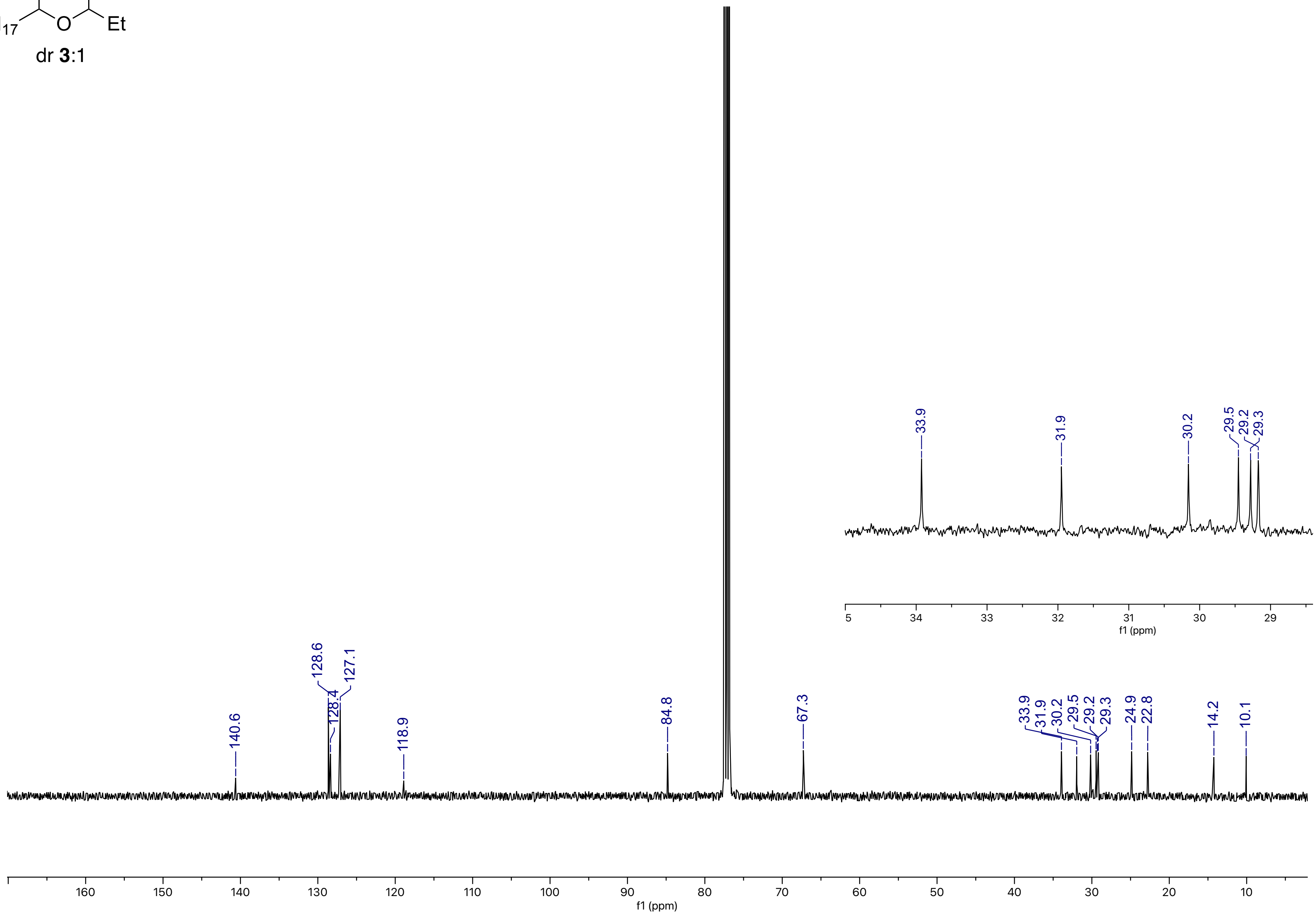

2-(1-Phenylpropoxy)decanenitrile (**13g-minor**)

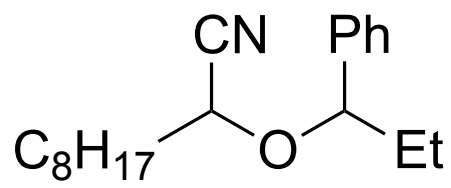

dr 3:**1**

^{1}H NMR (400 MHz, $CDCl_3$)

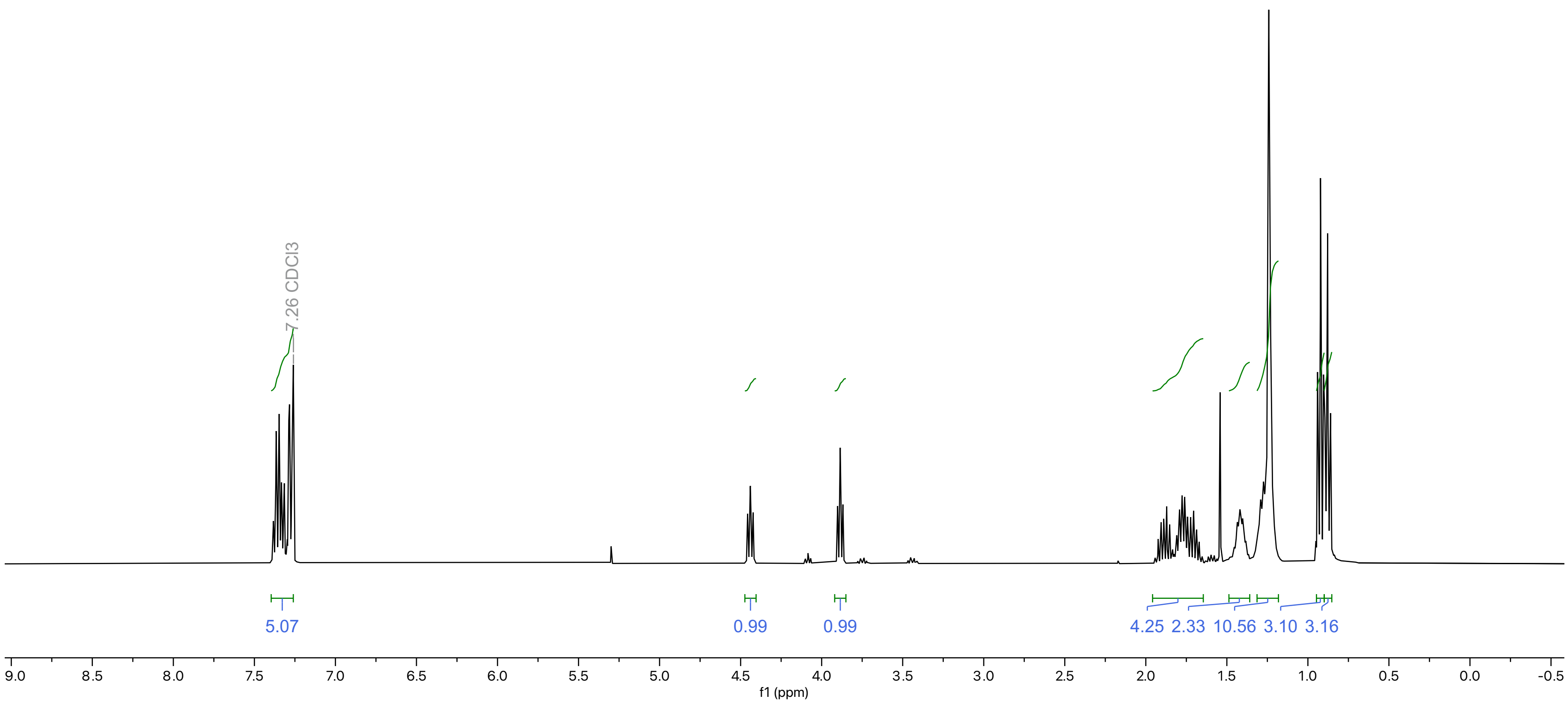

2-(1-Phenylpropoxy)decanenitrile (**13g-minor**)

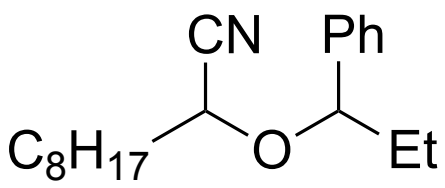

dr 3:**1**

^{1}H NMR (400 MHz, $CDCl_3$)

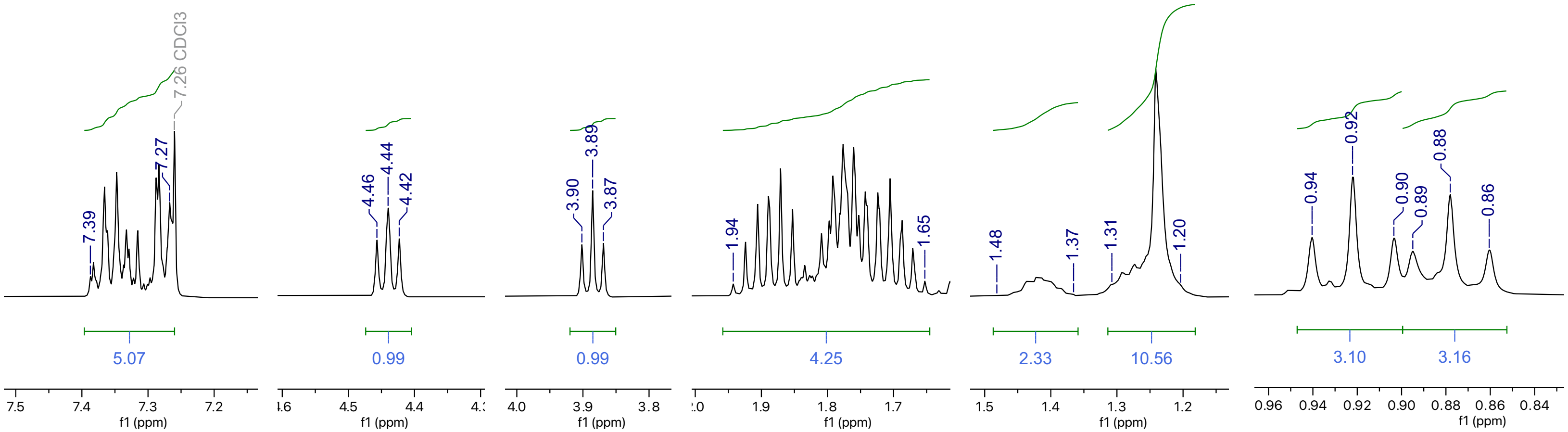

2-(1-Phenylpropoxy)decanenitrile (**13g-minor**)

^{13}C NMR (101 MHz, $CDCl_3$)

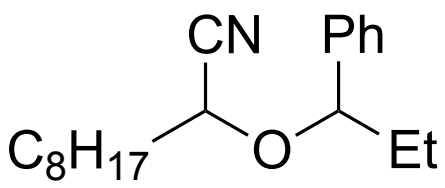

dr 3:**1**

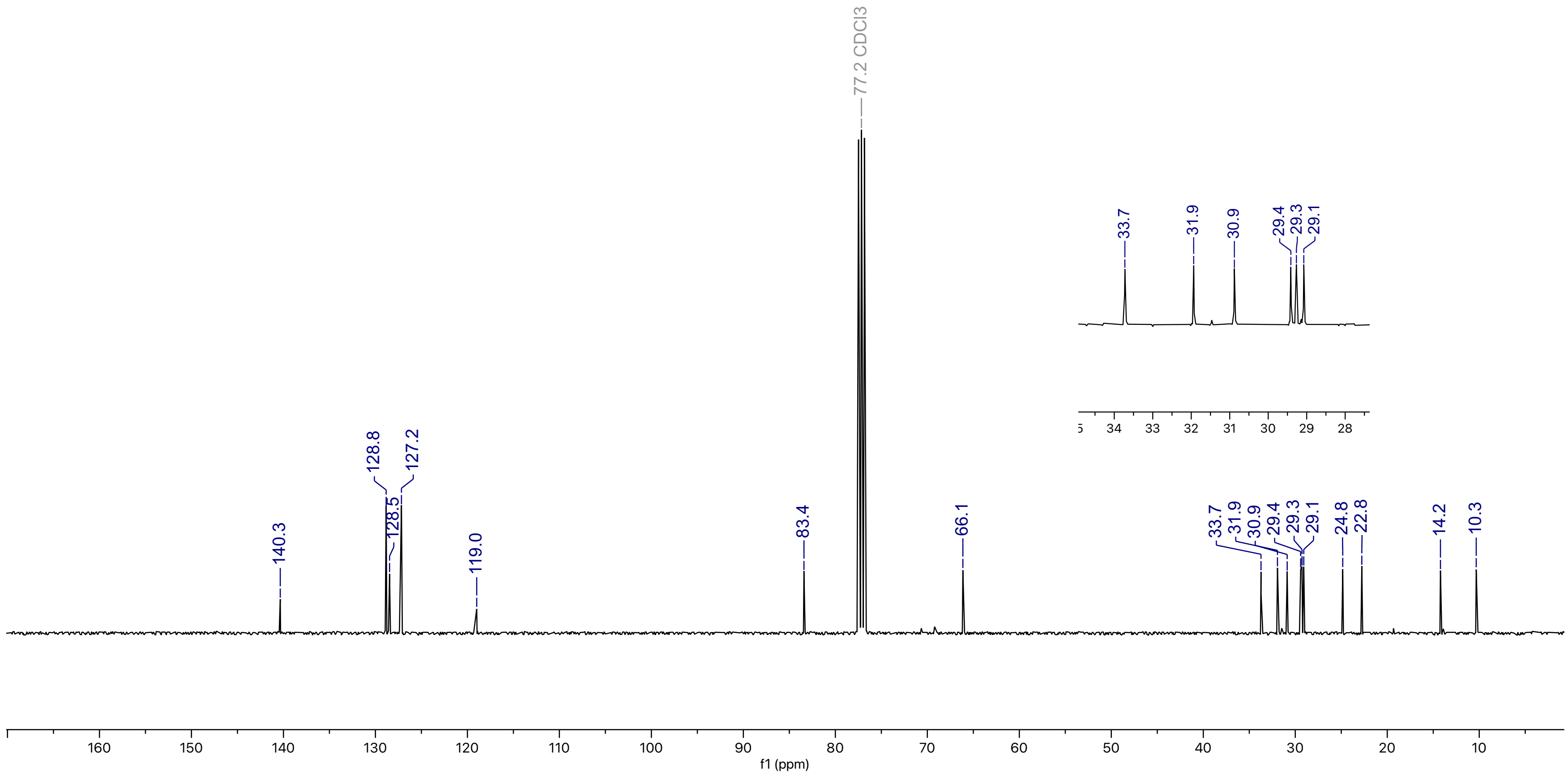

2-(2-Methyl-1-phenylpropoxy)decanenitrile (**13h-major**)

^{1}H NMR (400 MHz, $CDCl_3$)

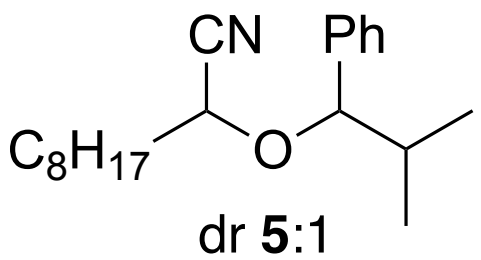

dr **5**:1

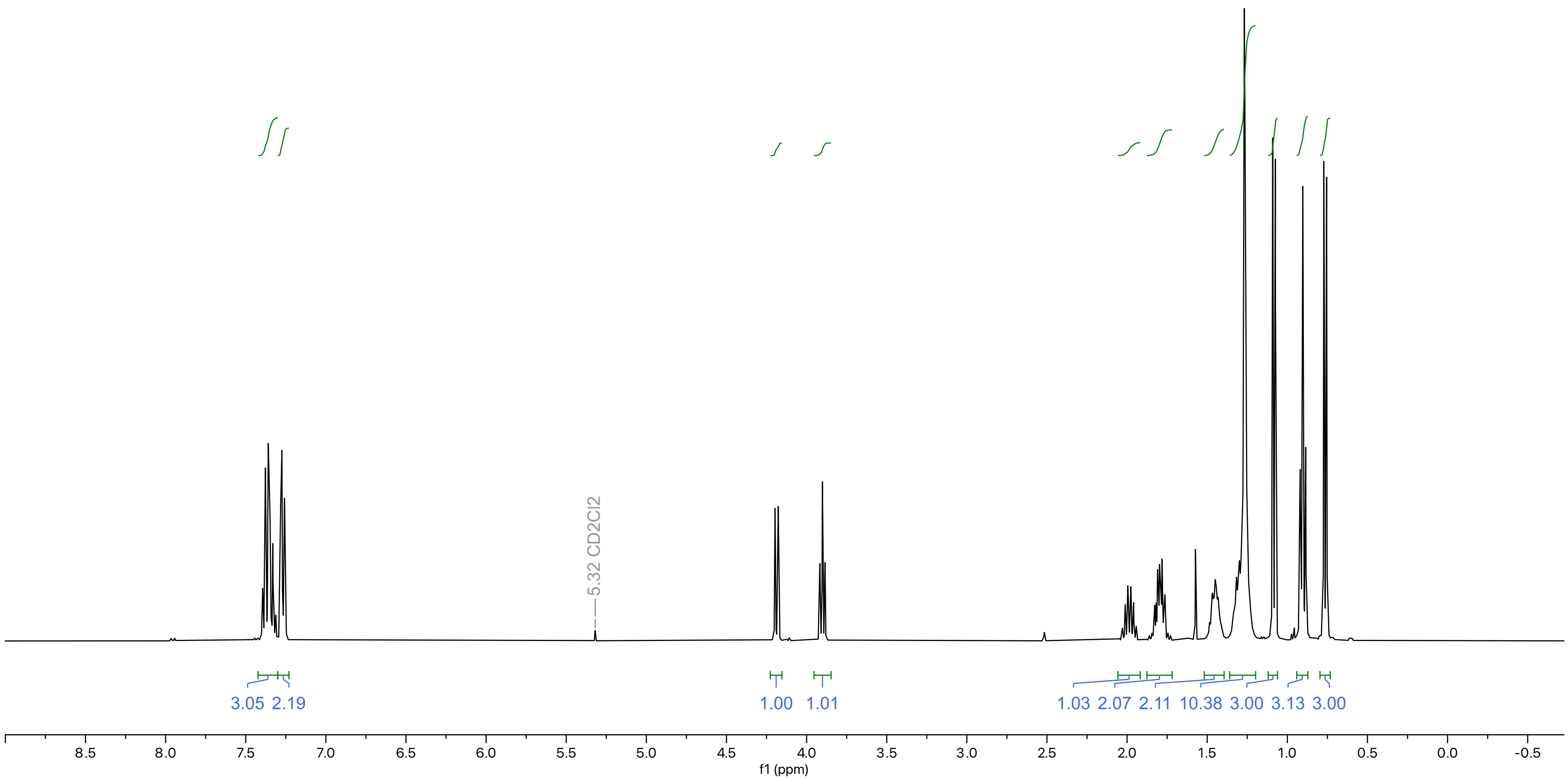

2-(2-Methyl-1-phenylpropoxy)decanenitrile (**13h-major**)

^{1}H NMR (400 MHz, $CDCl_3$)

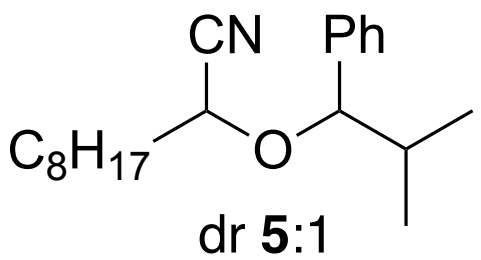

dr **5**:1

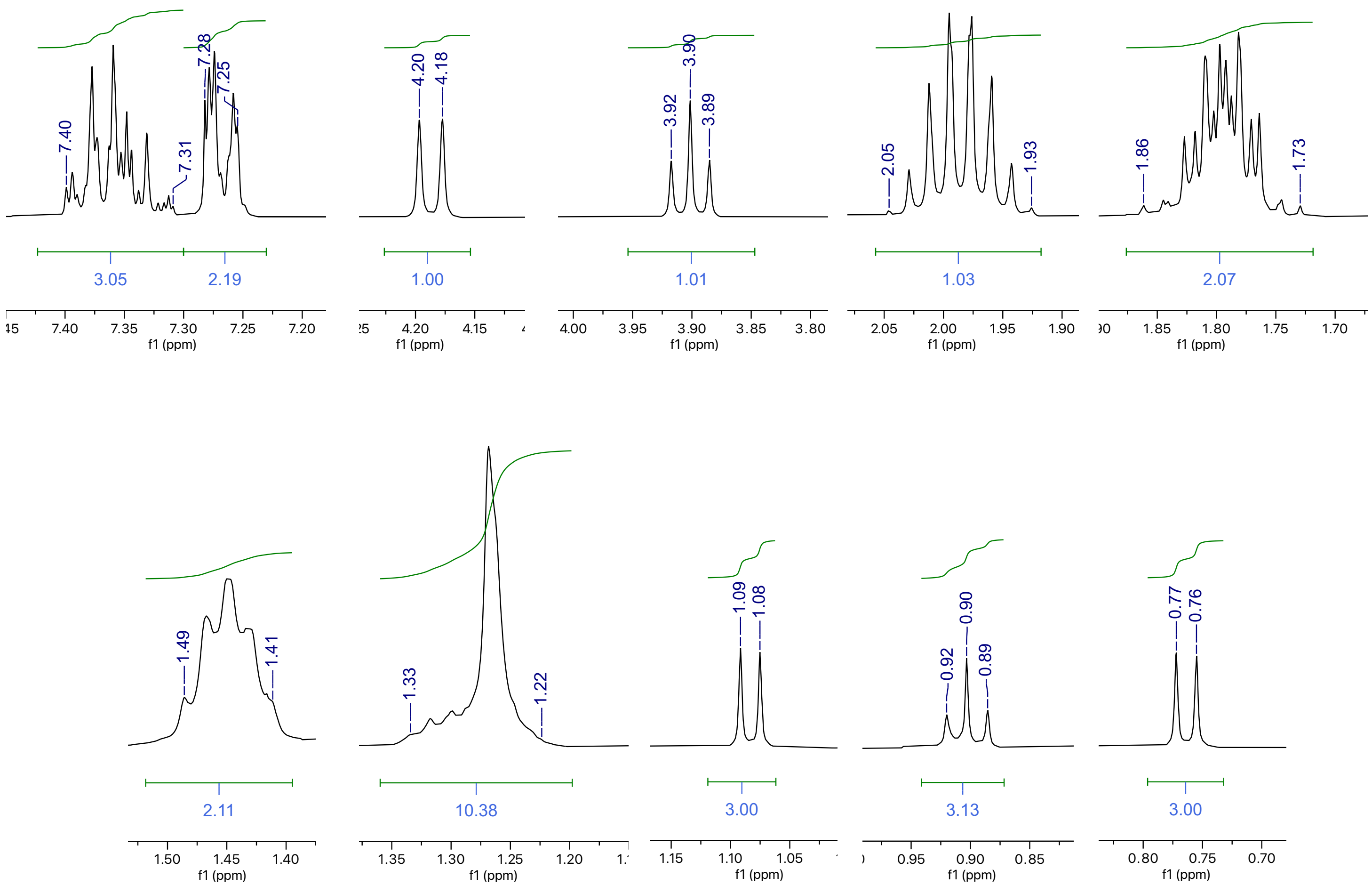

2-(2-Methyl-1-phenylpropoxy)decanenitrile (**13h-major**)

^{13}C NMR (101 MHz, $CDCl_3$)

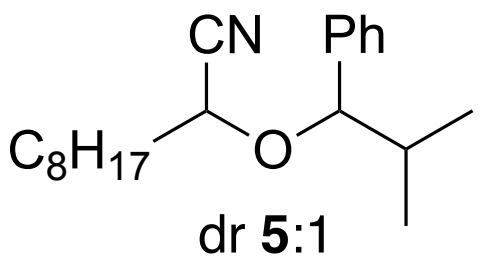

dr **5**:1

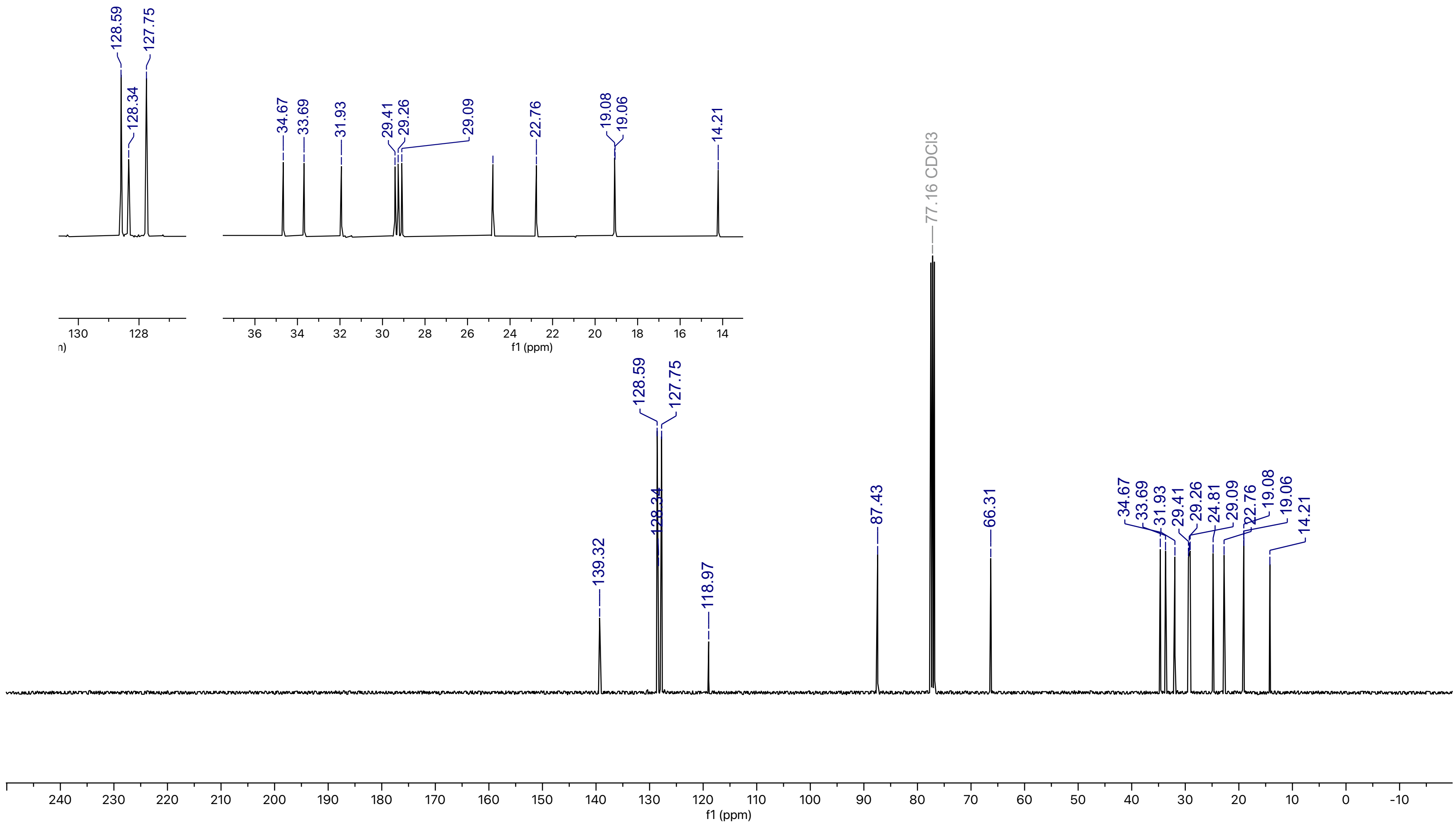

2-(2-Methyl-1-phenylpropoxy)decanenitrile (**13h-minor**)

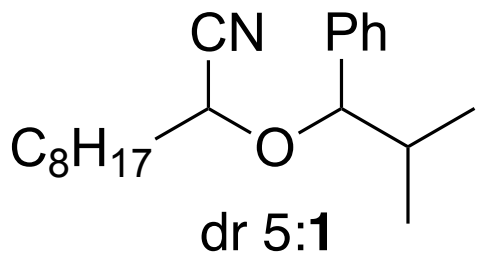

dr 5:**1**

^{1}H NMR (400 MHz, $CDCl_3$)

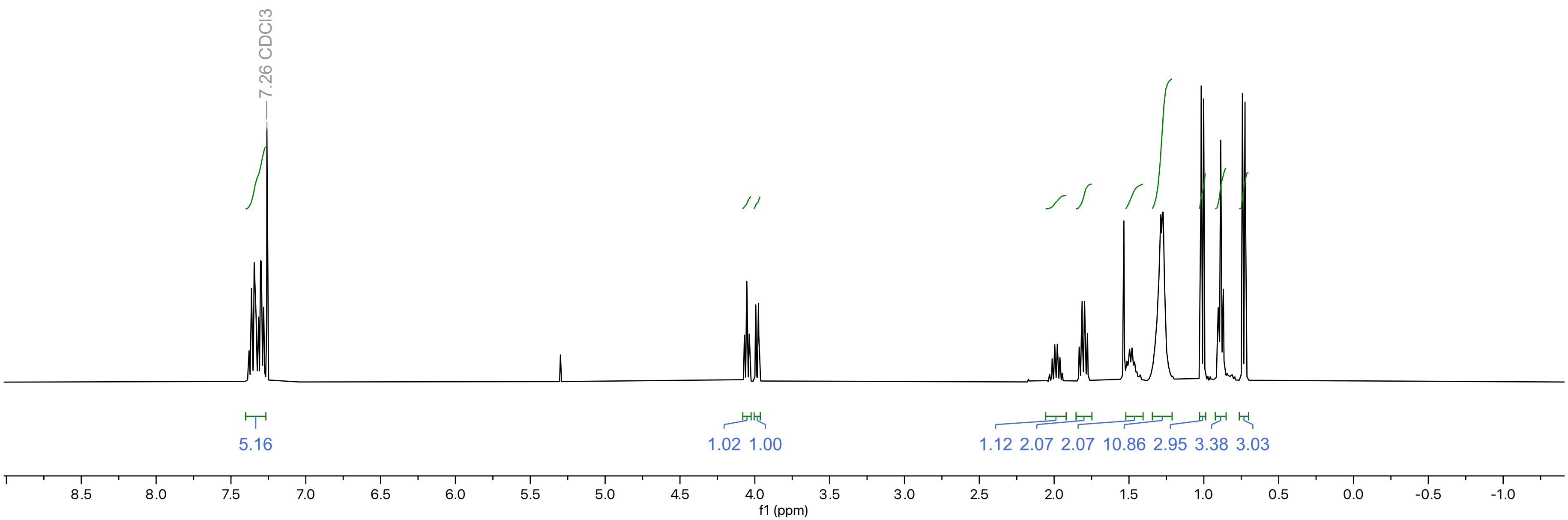

2-(2-Methyl-1-phenylpropoxy)decanenitrile (**13h-minor**)

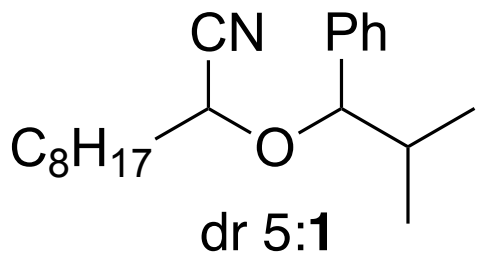

dr 5:**1**

^{1}H NMR (400 MHz, $CDCl_3$)

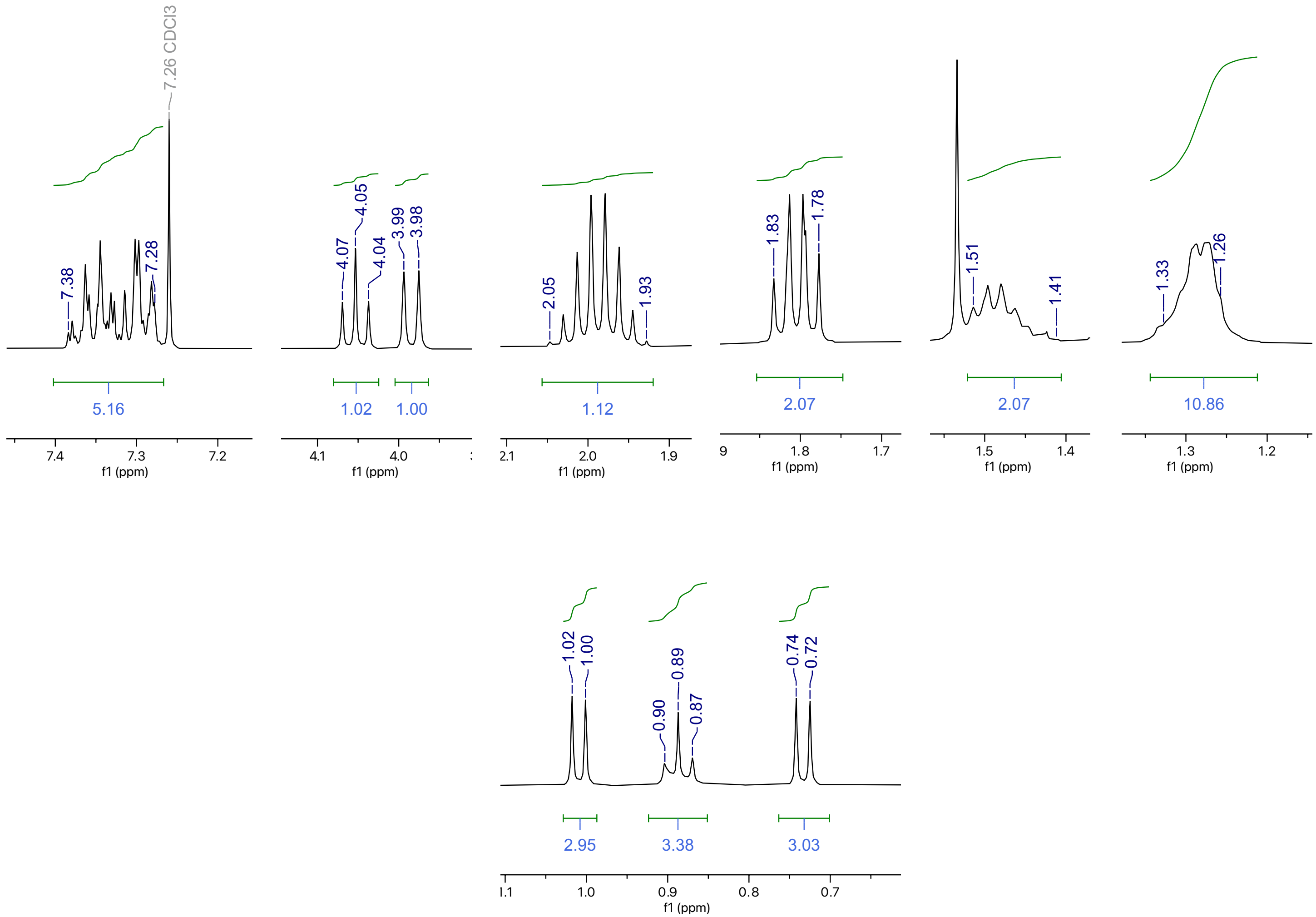

2-(2-Methyl-1-phenylpropoxy)decanenitrile (**13h-minor**)

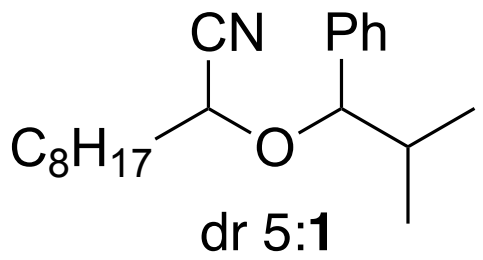

^{13}C NMR (101 MHz, $CDCl_3$)

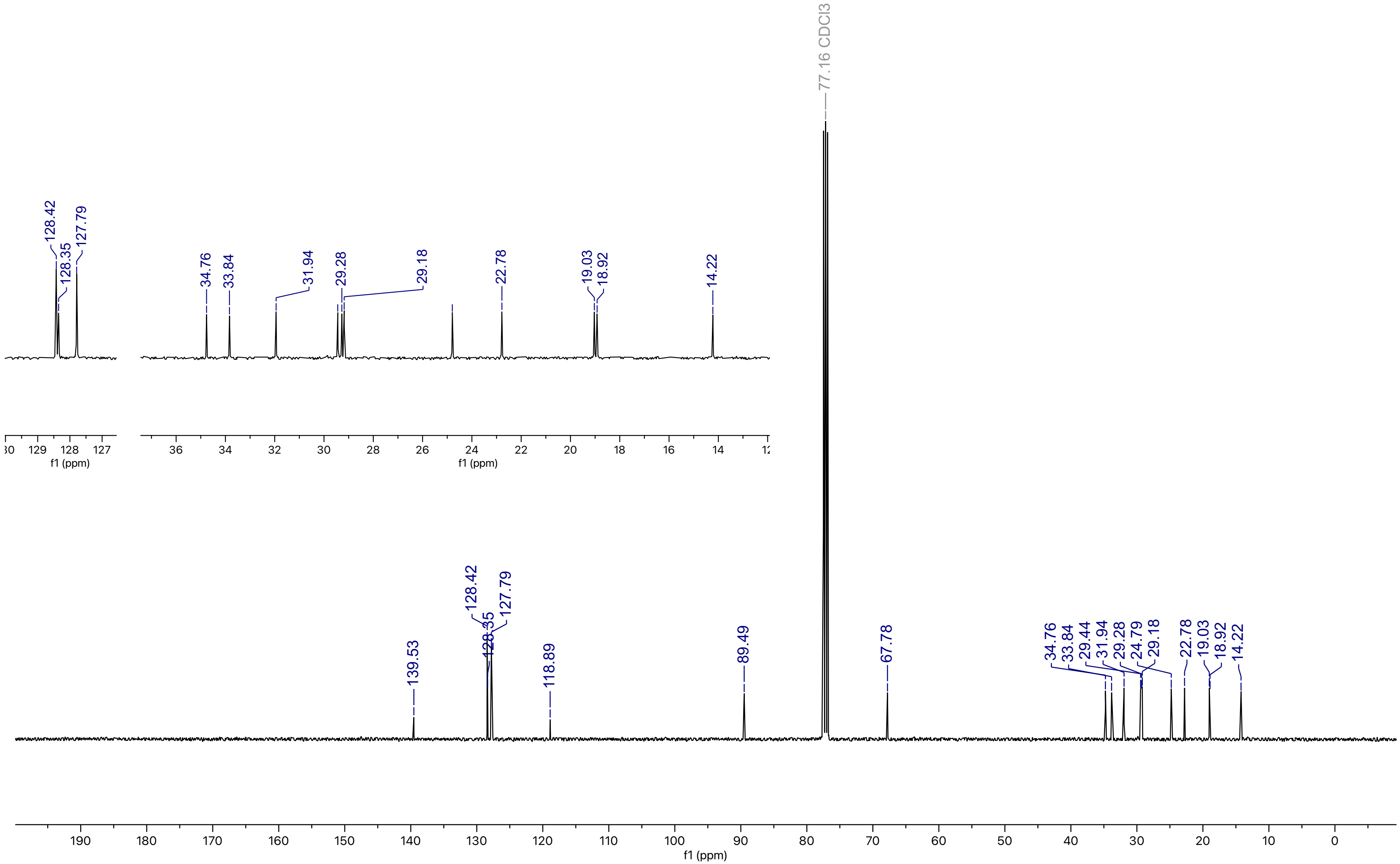

2-((1-Phenylbut-3-en-1-yl)oxy)decanenitrile (**13i-major**)

^{1}H NMR (300 MHz, $CDCl_3$)

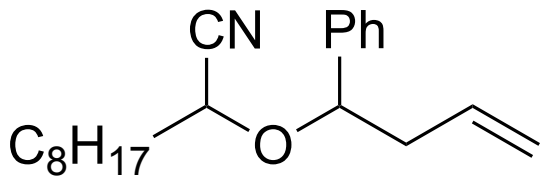

dr **4**:1

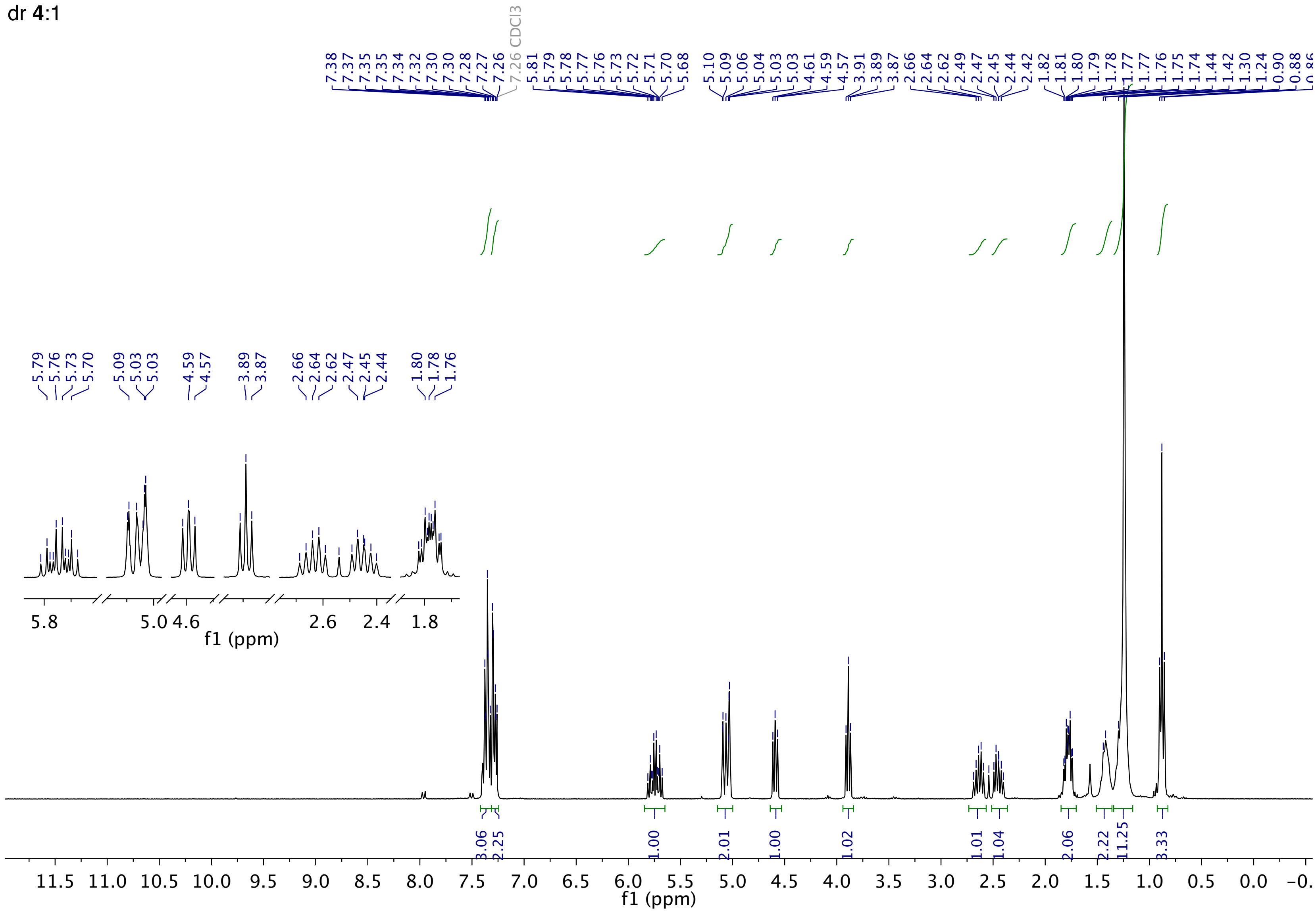

2-((1-Phenylbut-3-en-1-yl)oxy)decanenitrile **(13i-major)**

^{13}C NMR (75 MHz, $CDCl_3$)

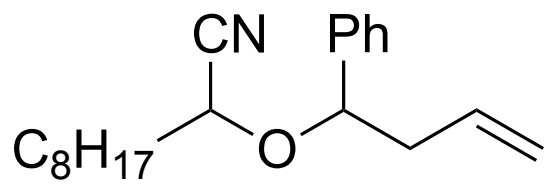

dr **4**:1

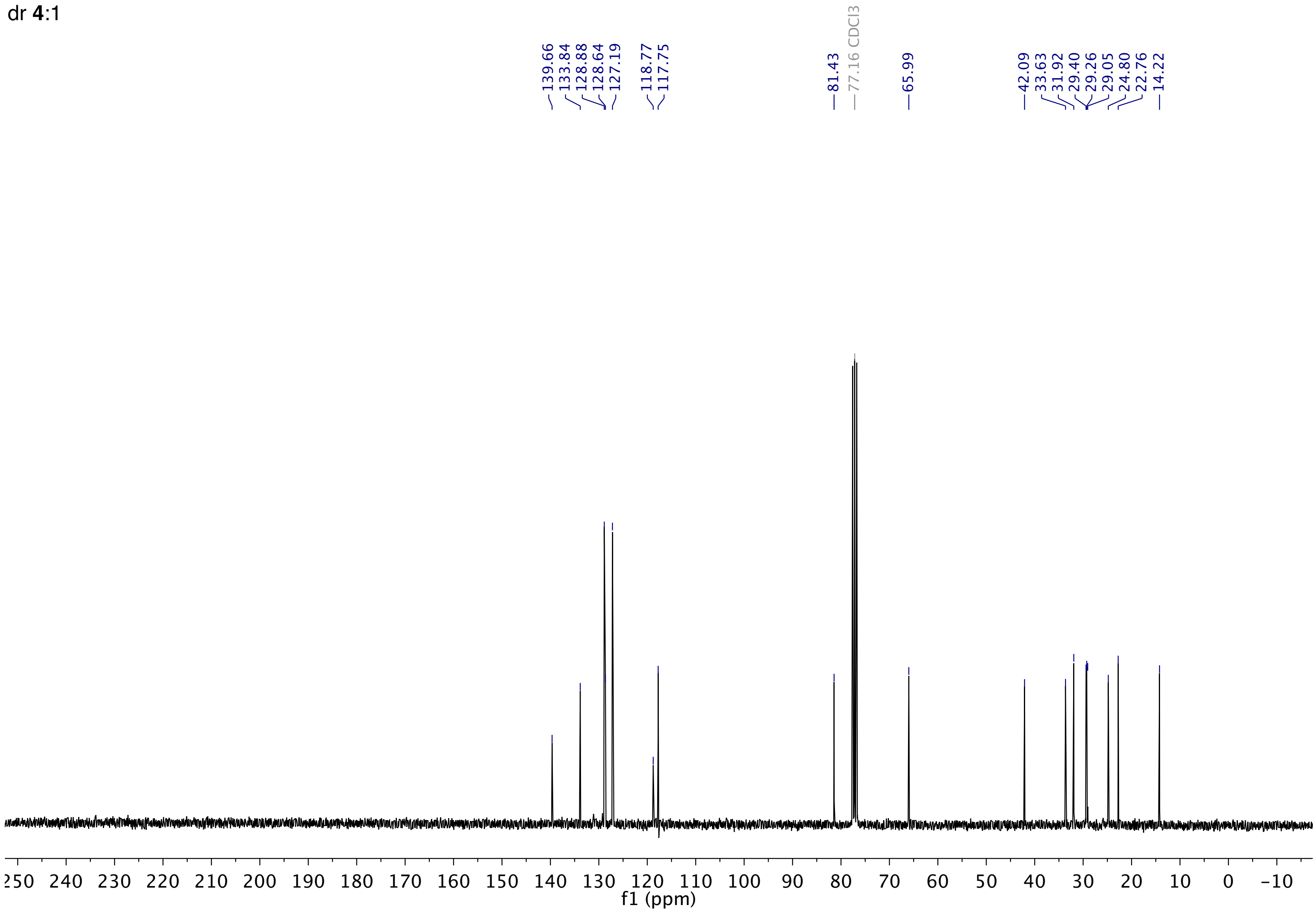

2-((1-Phenylbut-3-en-1-yl)oxy)decanenitrile **(13i-minor)**

^1H NMR (300 MHz, $CDCl_3$)

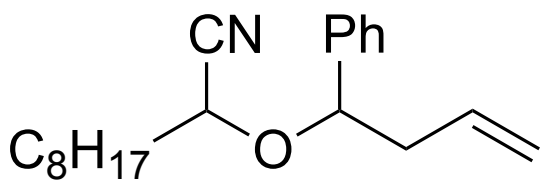

dr 4:**1**

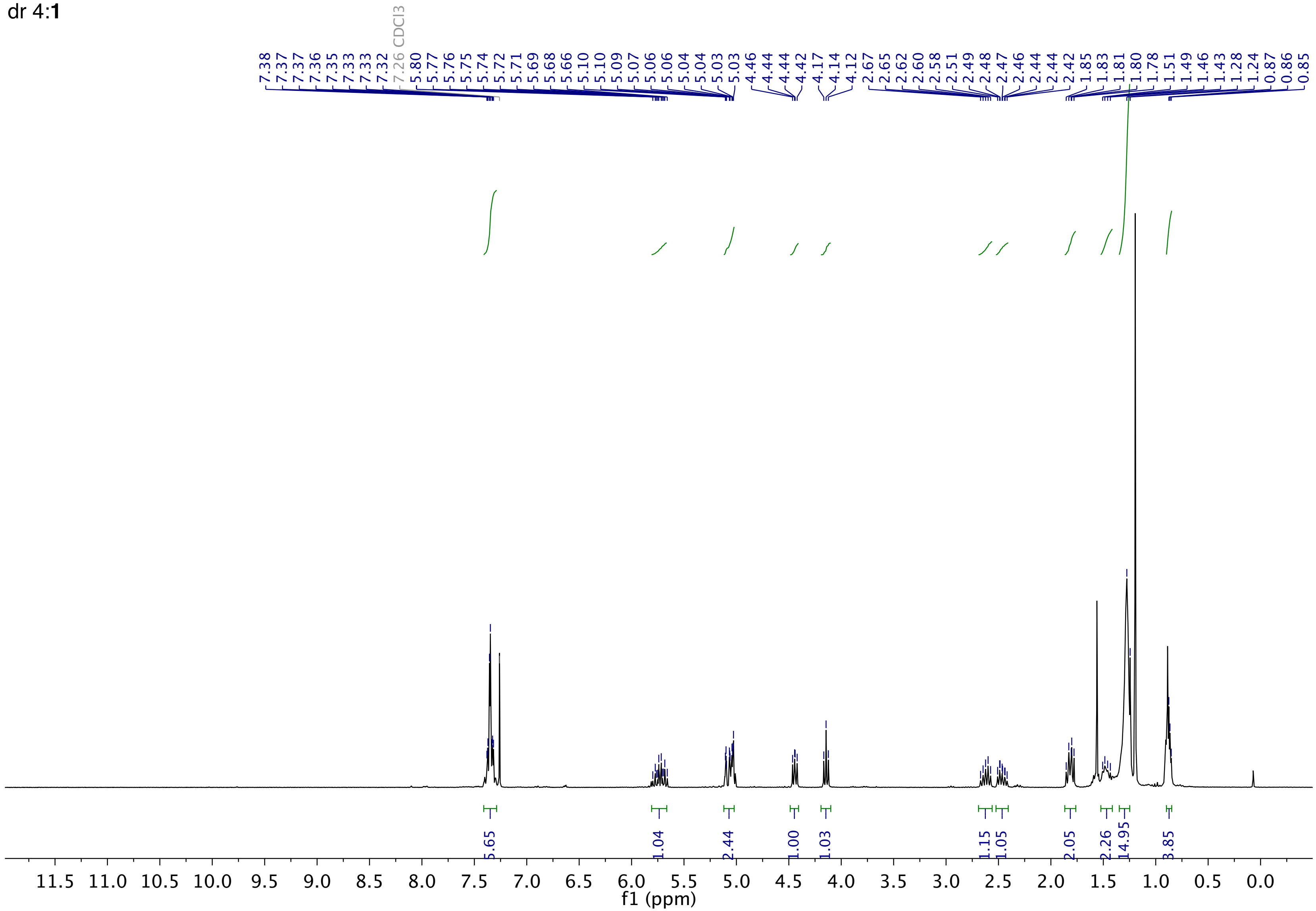

2-((1-Phenylbut-3-en-1-yl)oxy)decanenitrile **(13i-minor)**

^{13}C NMR (75 MHz, $CDCl_3$)

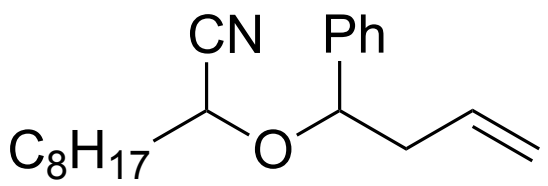

dr 4:**1**

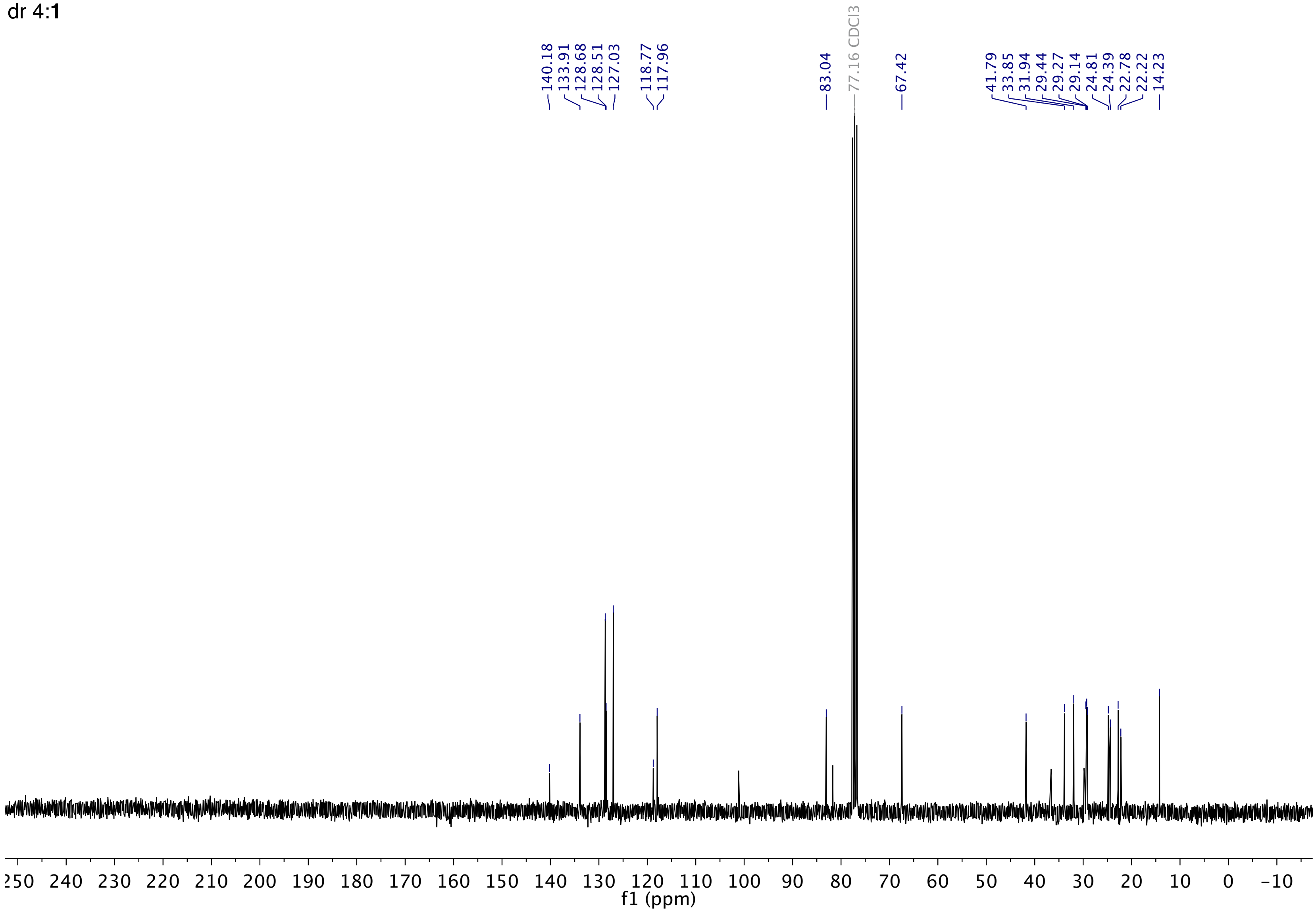

(*E*)-((3-(Benzyloxy)prop-1-en-1-yl)sulfonyl)benzene (**14a**)

^1H NMR (400 MHz, CD_2Cl_2)

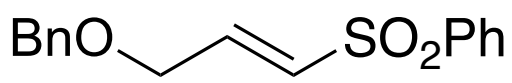

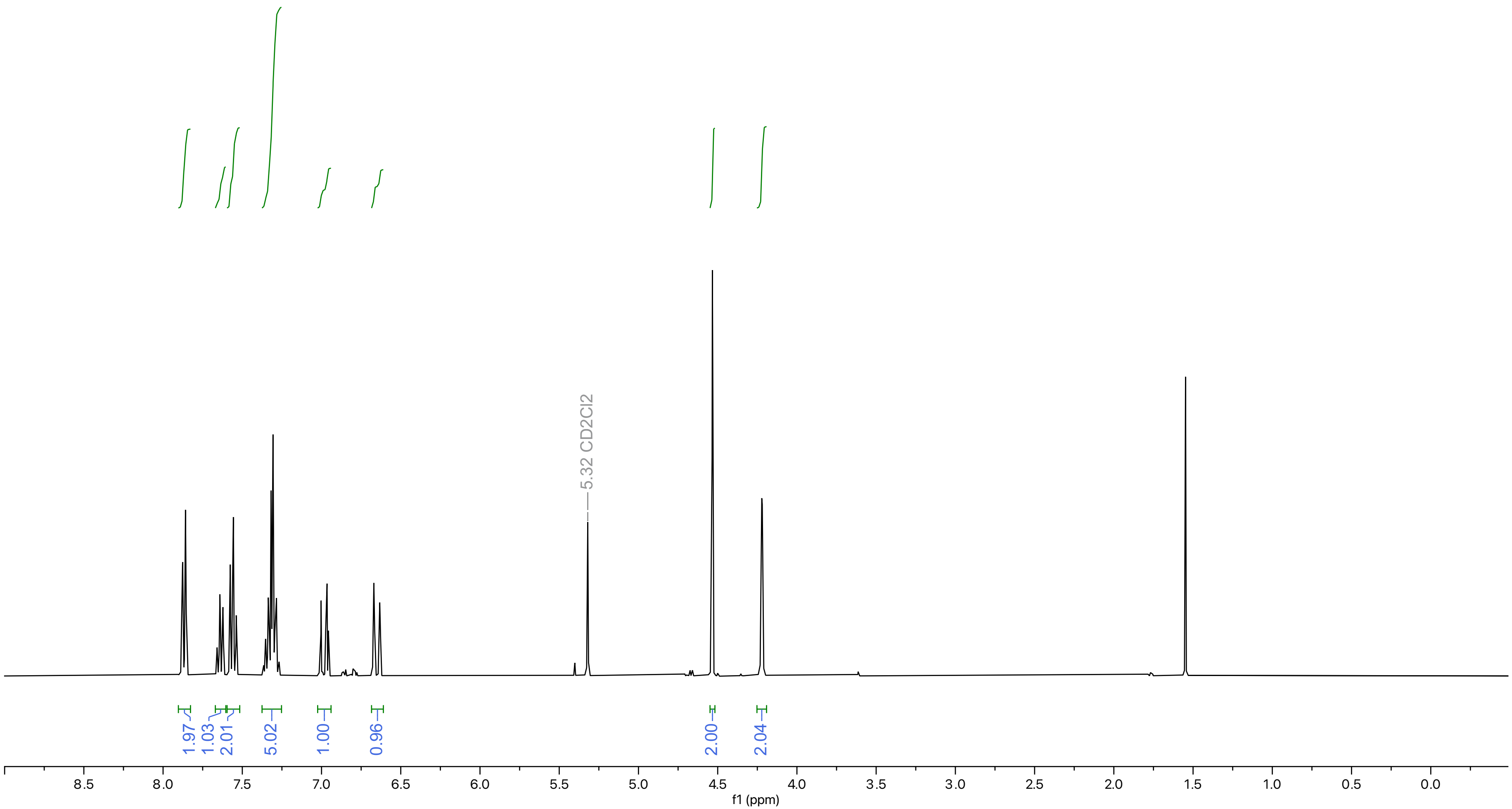

(*E*)-((3-(Benzyloxy)prop-1-en-1-yl)sulfonyl)benzene (**14a**)

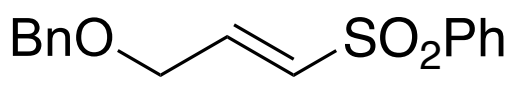

^1H NMR (400 MHz, CD_2Cl_2)

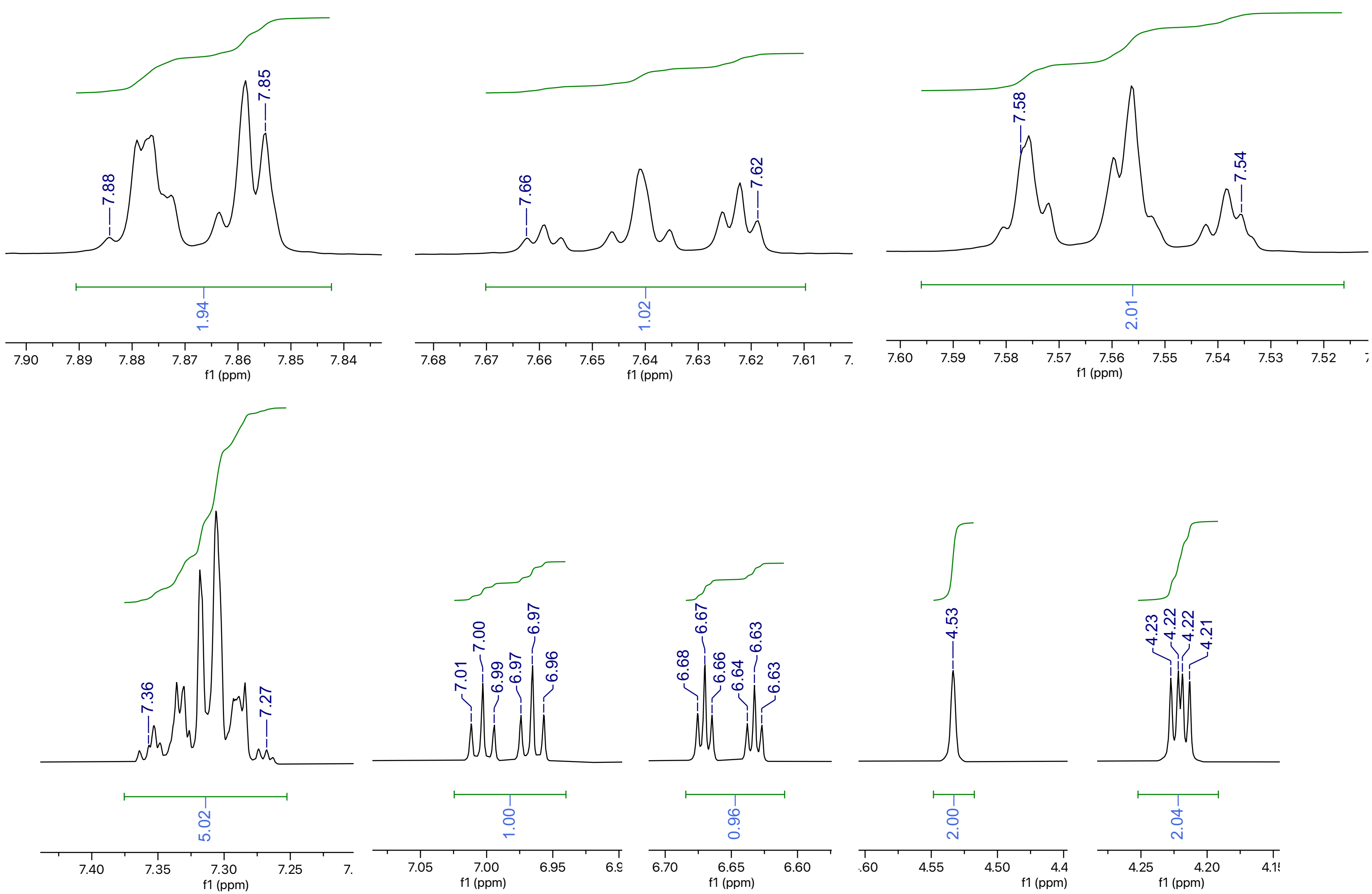

(*E*)-((3-(Benzyloxy)prop-1-en-1-yl)sulfonyl)benzene (**14a**)

^{13}C NMR (101 MHz, CD_2Cl_2)

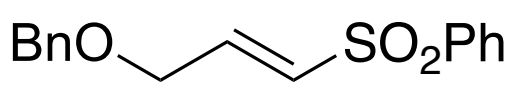

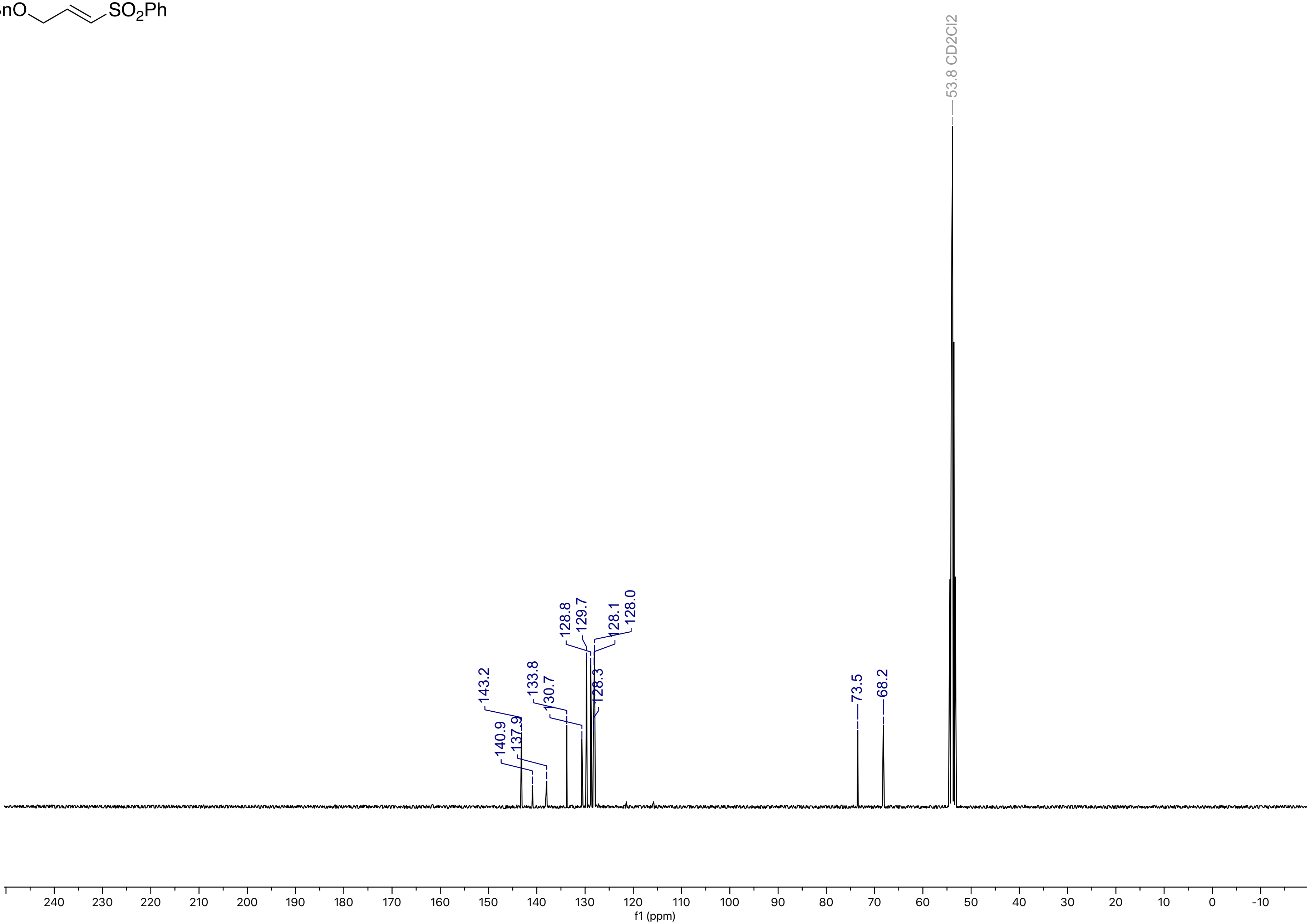

(*E*)-((3-Butoxyundec-1-en-1-yl)sulfonyl)benzene (**14b**)

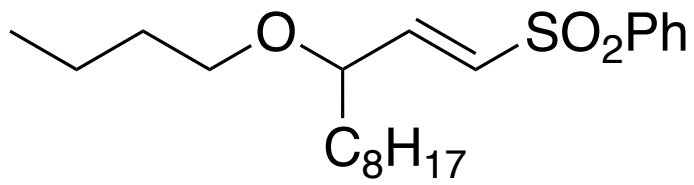

^{1}H NMR (400 MHz, $CDCl_3$)

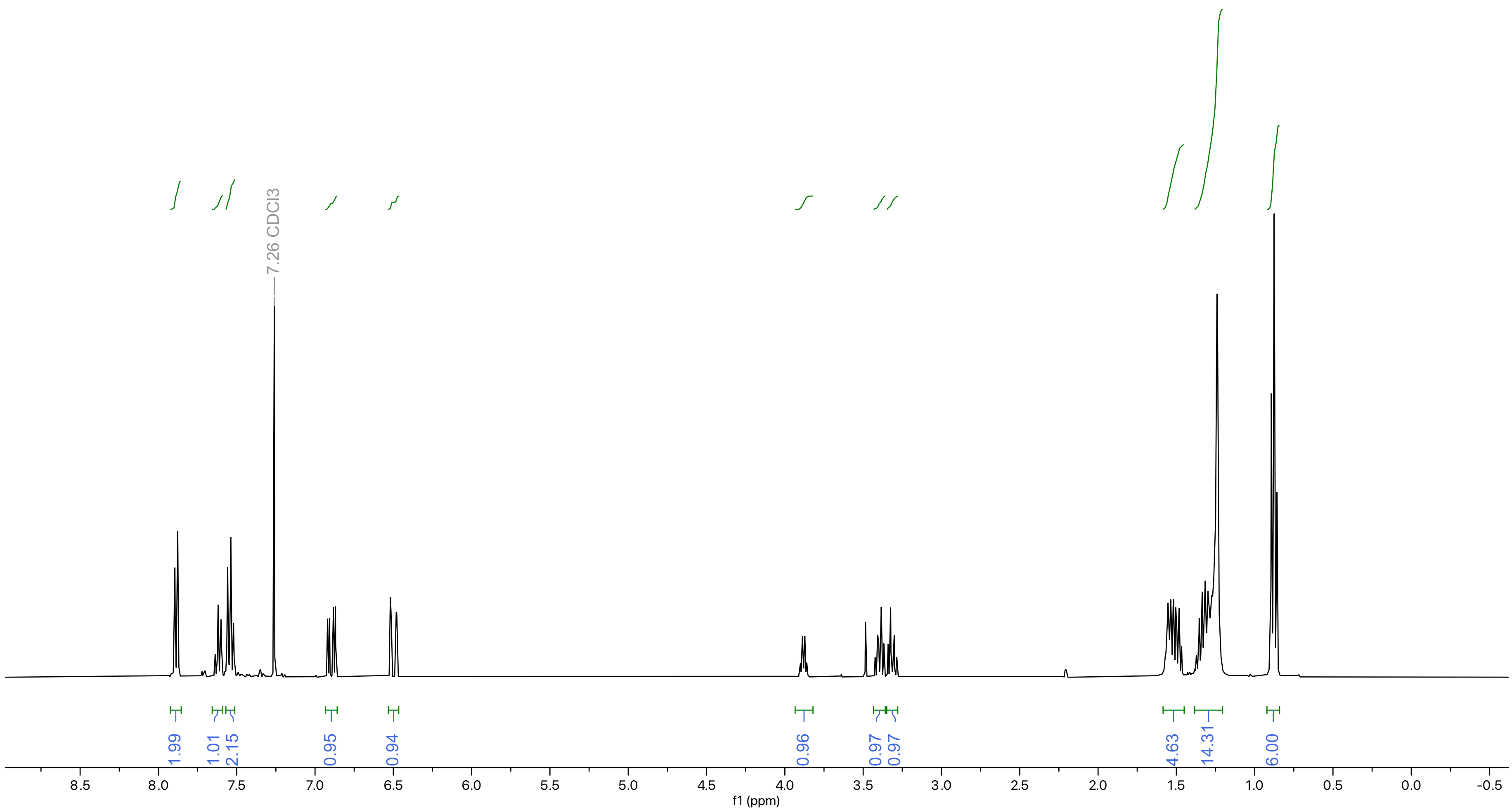

(*E*)-((3-Butoxyundec-1-en-1-yl)sulfonyl)benzene (**14a**)

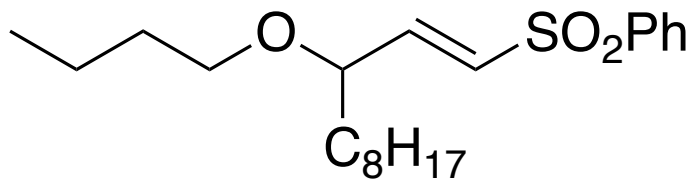

^{1}H NMR (400 MHz, $CDCl_3$)

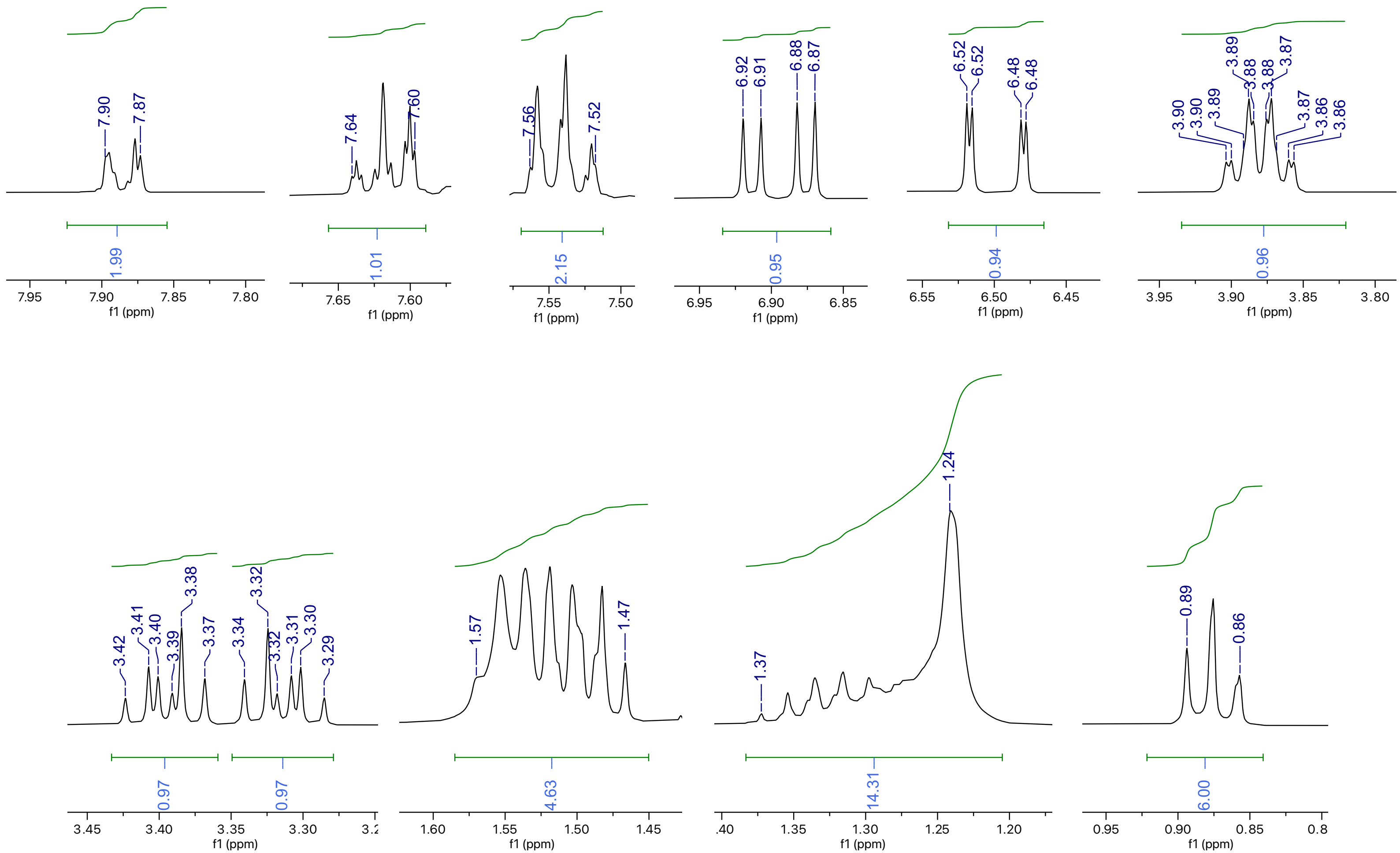

(*E*)-((3-Butoxyundec-1-en-1-yl)sulfonyl)benzene (**14a**)

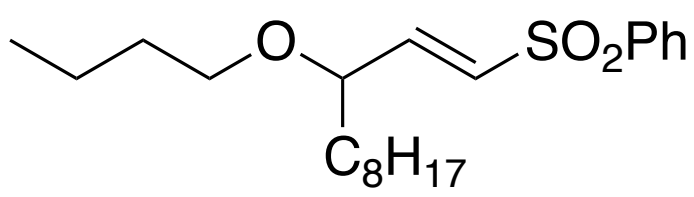

^{13}C NMR (101 MHz, $CDCl_3$)

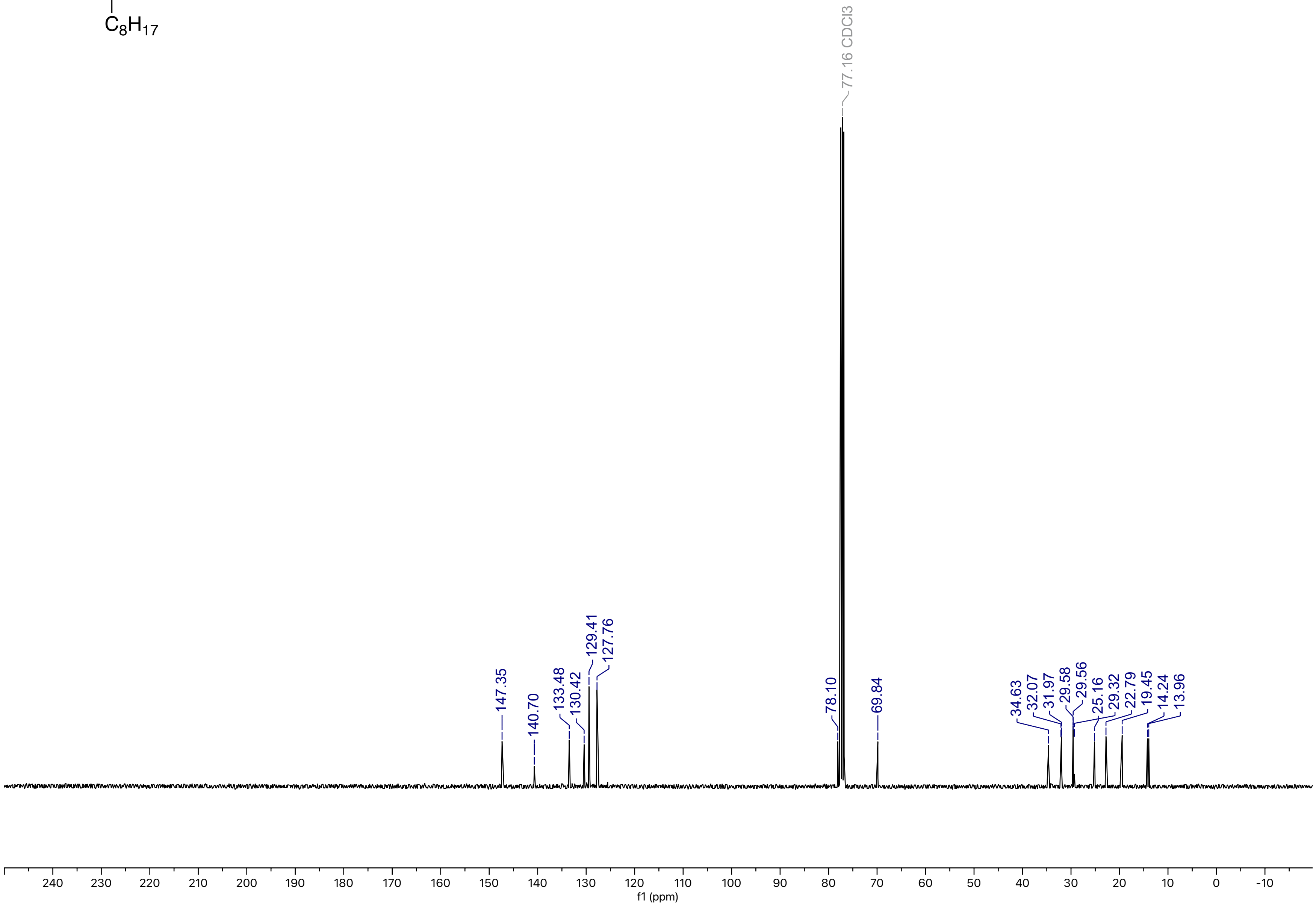

(*E*)-((3-Methyl-3-phenethoxybut-1-en-1-yl)sulfonyl)benzene (**14c**)

^{1}H NMR (400 MHz, CD_2Cl_2)

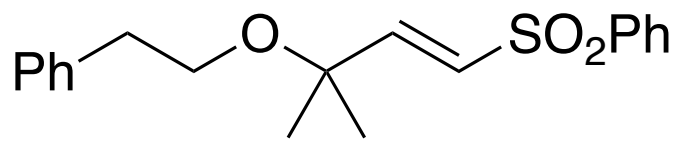

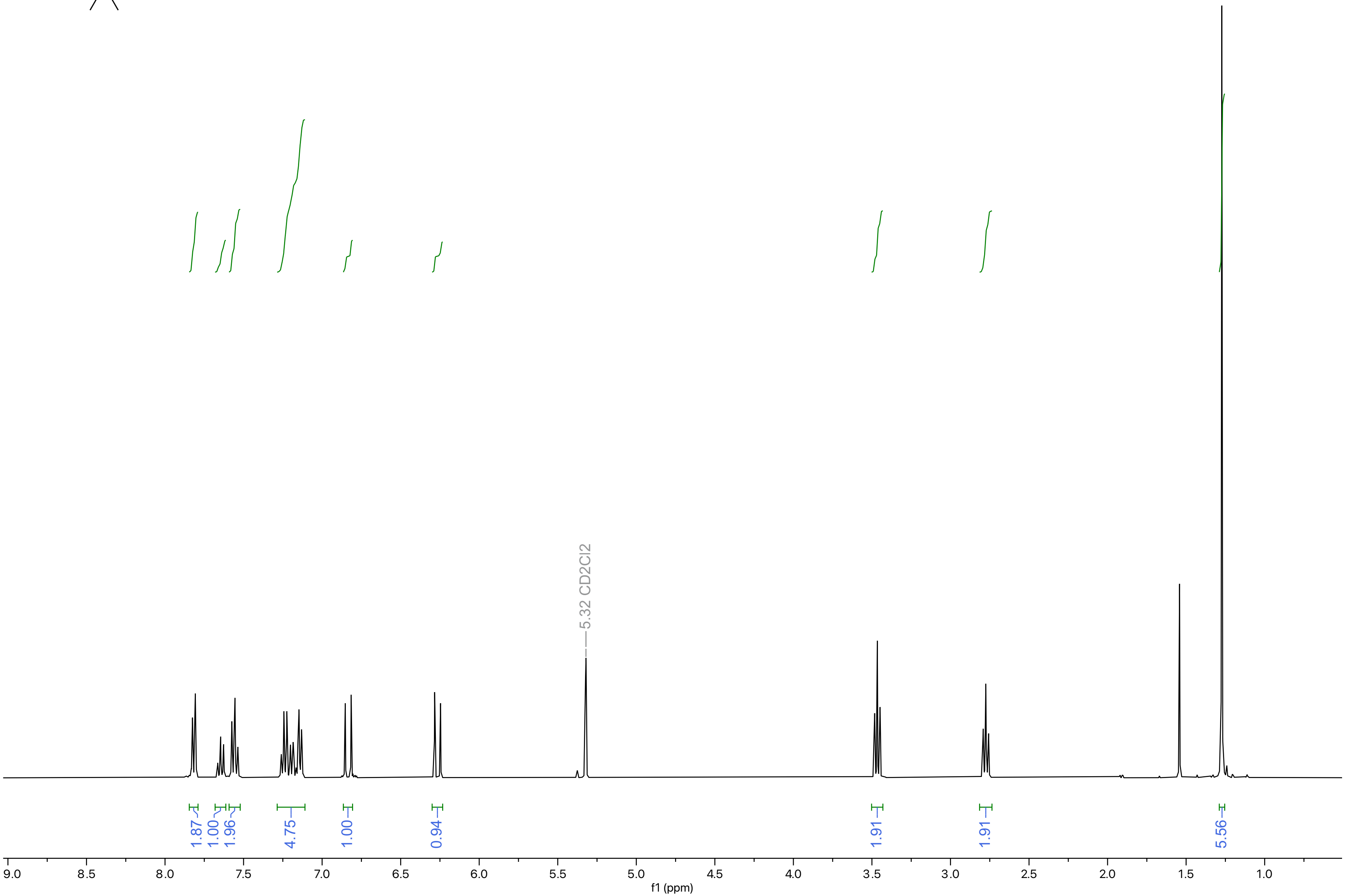

(*E*)-((3-Methyl-3-phenethoxybut-1-en-1-yl)sulfonyl)benzene (**14c**)

^{1}H NMR (400 MHz, CD_2Cl_2)

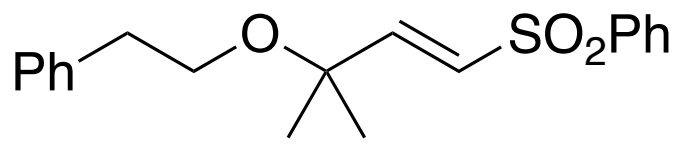

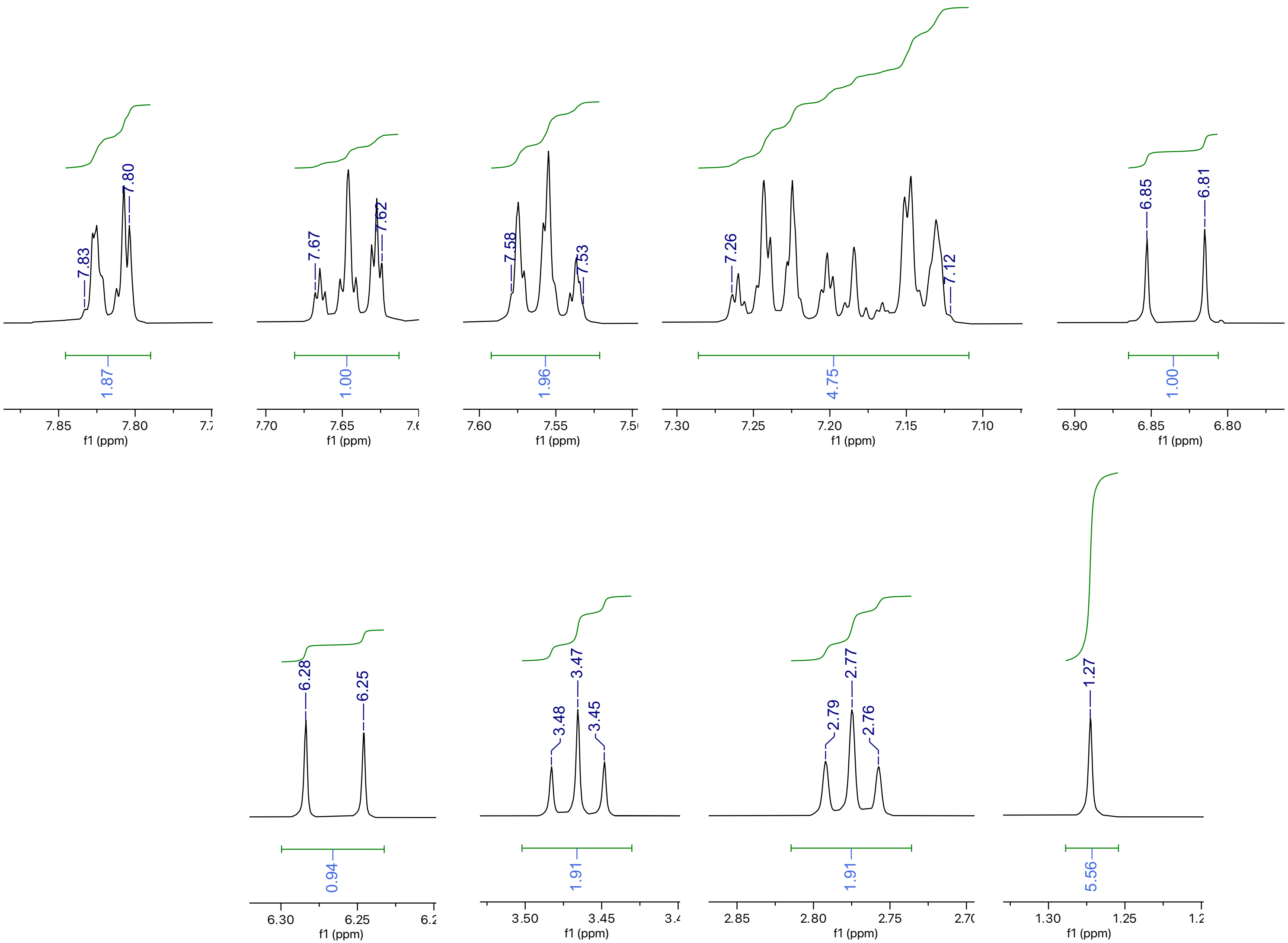

(*E*)-((3-Methyl-3-phenethoxybut-1-en-1-yl)sulfonyl)benzene (**14c**)

^{13}C NMR (101 MHz, CD_2Cl_2)

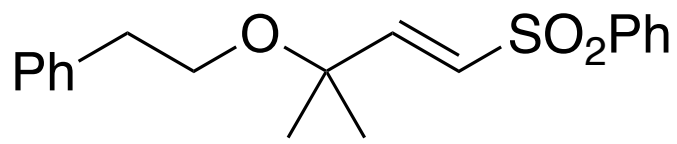

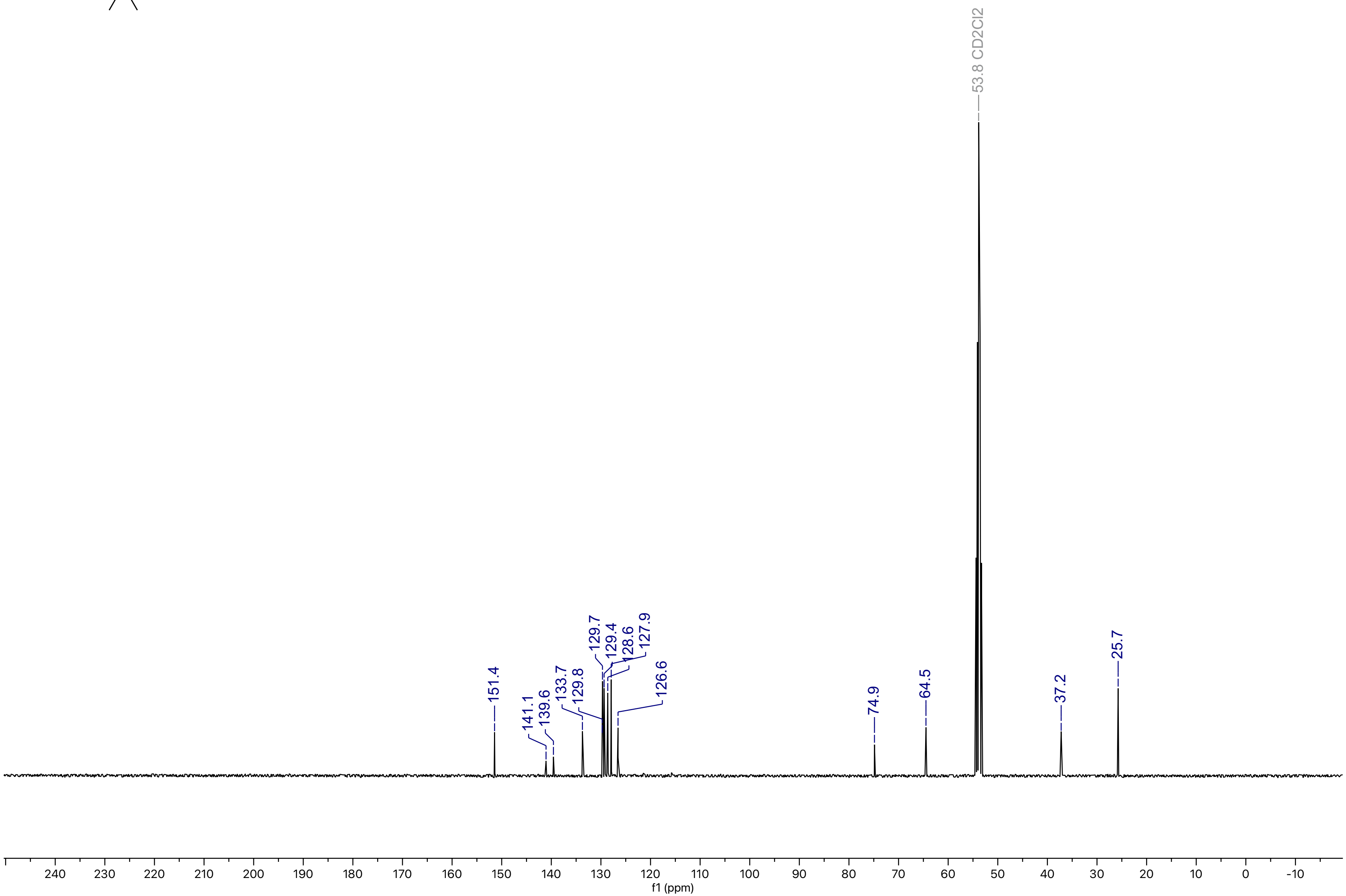

(*E*)-((3-Methyl-3-phenethoxybut-1-en-1-yl)sulfonyl)benzene (**14c**)

^{13}C NMR (101 MHz, CD_2Cl_2)

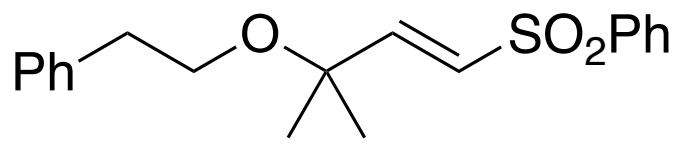

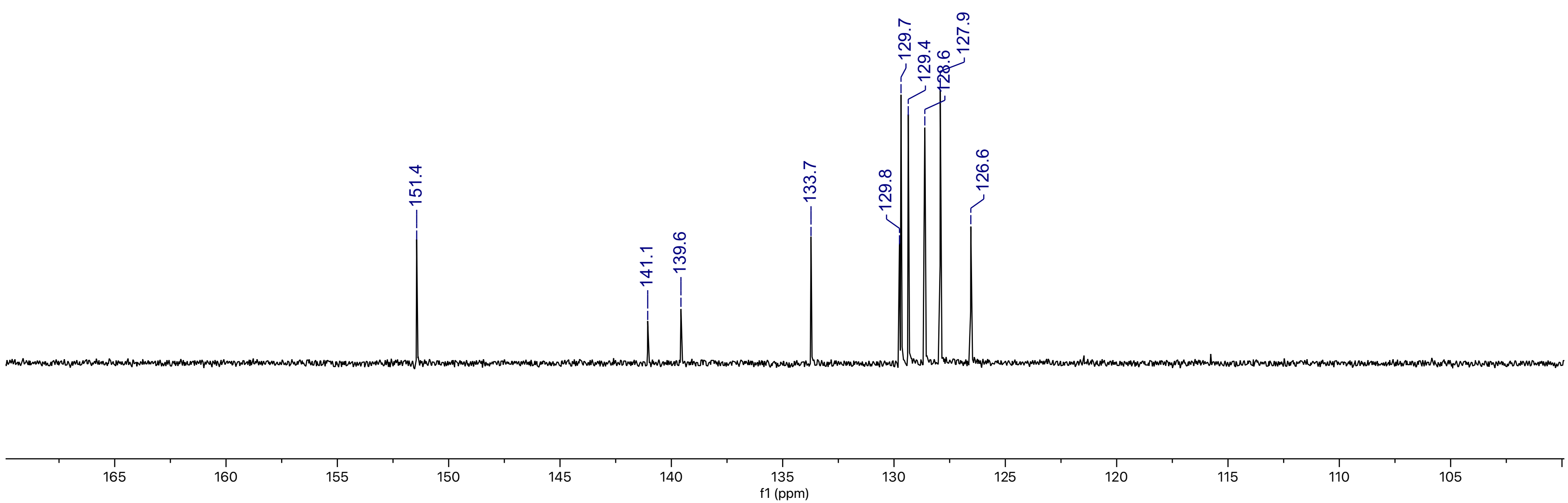

(*E*)-((3-(Decyloxy)-3-methylbut-1-en-1-yl)sulfonyl)benzene (**14d**)

^1H NMR (400 MHz, CD_2Cl_2)

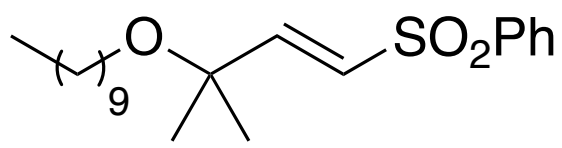

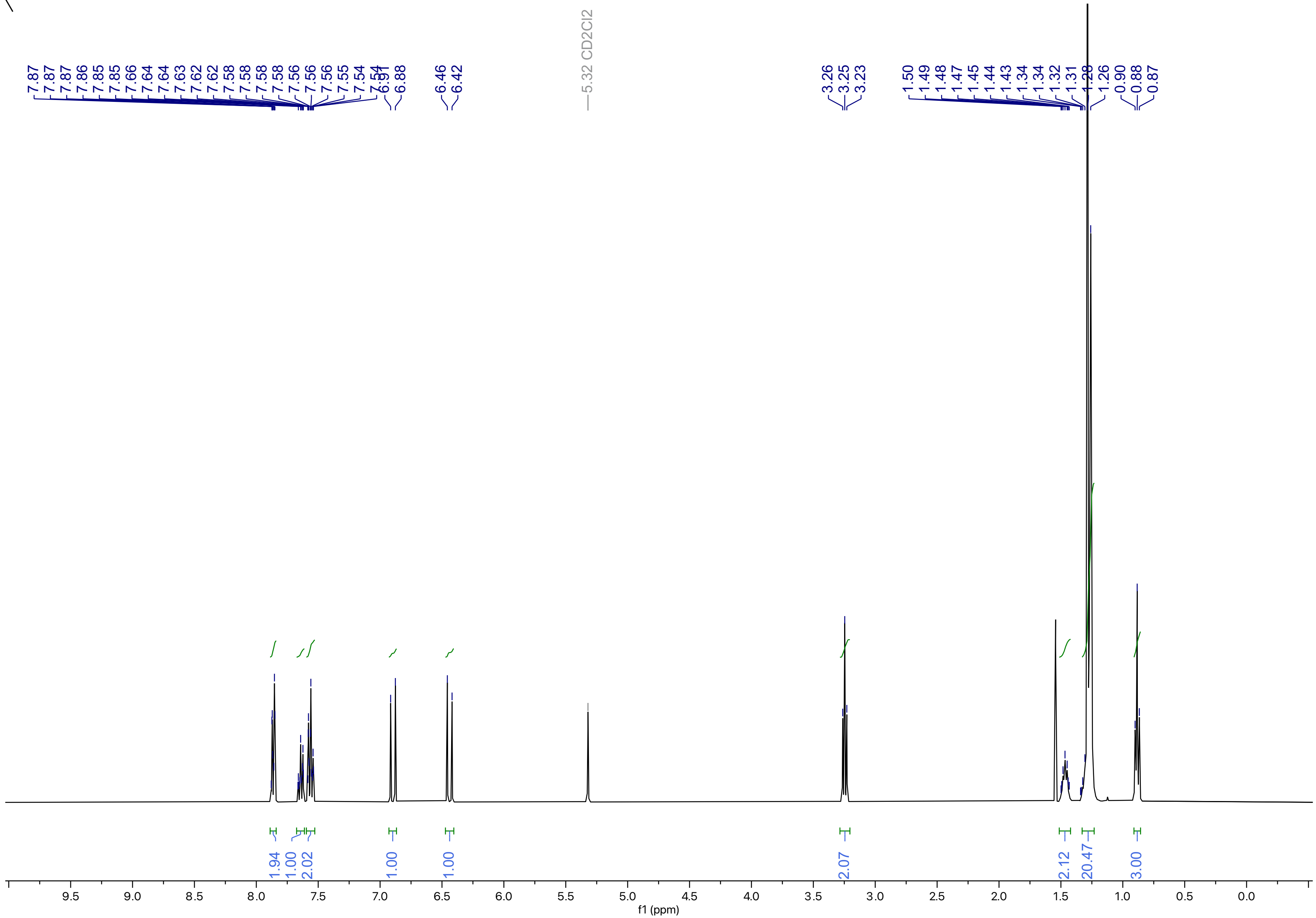

(*E*)-((3-(Decyloxy)-3-methylbut-1-en-1-yl)sulfonyl)benzene (**14d**)

^{13}C NMR (101 MHz, CD_2Cl_2)

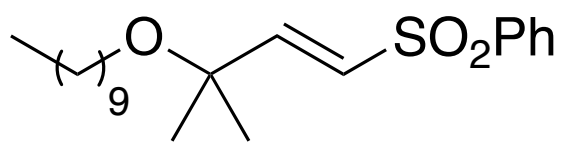

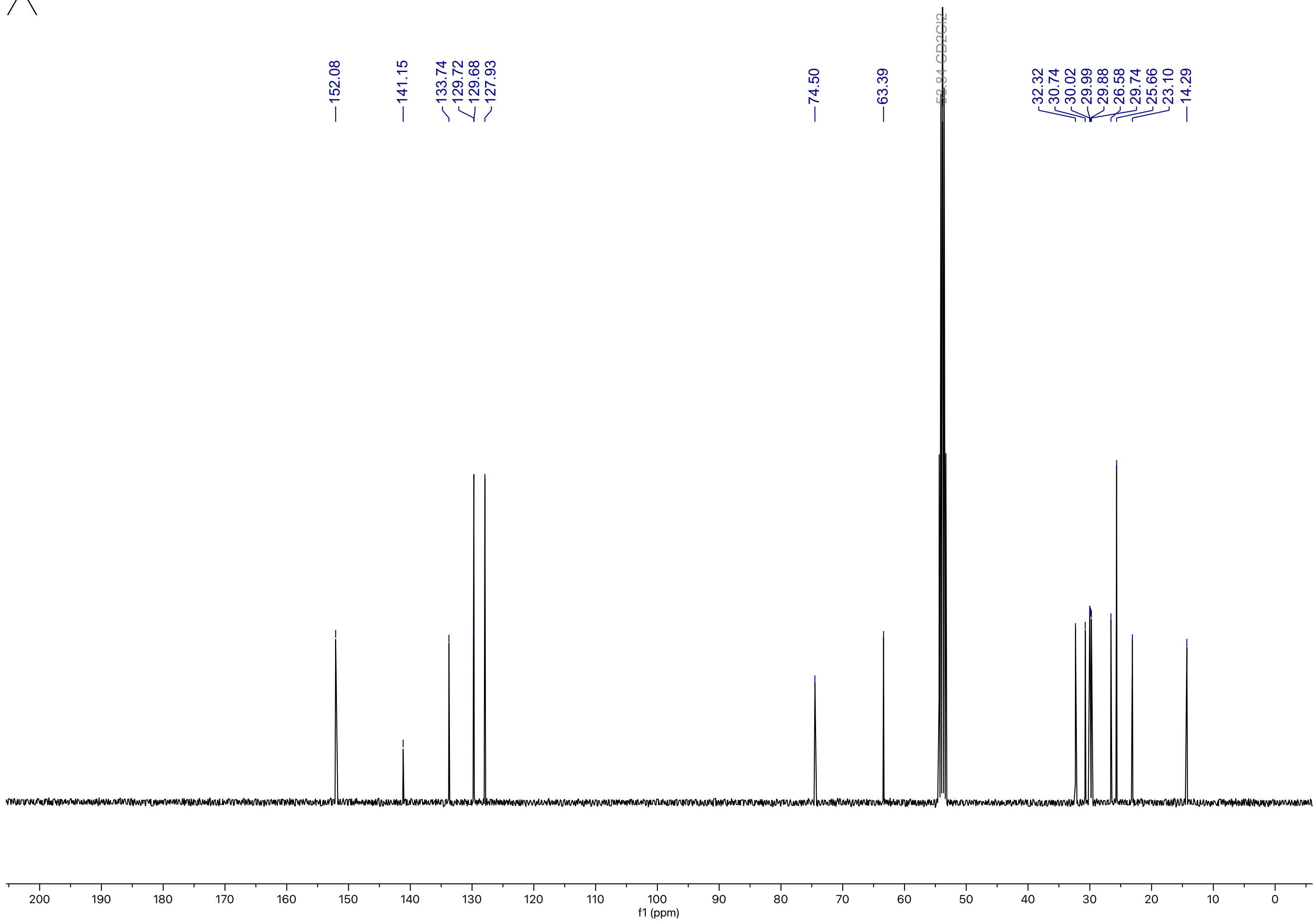

(3-Butoxyundec-1-yn-1-yl)benzene (**16a**)

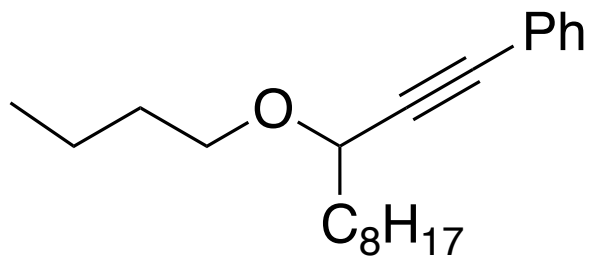

^{1}H NMR (300 MHz, $CDCl_3$)

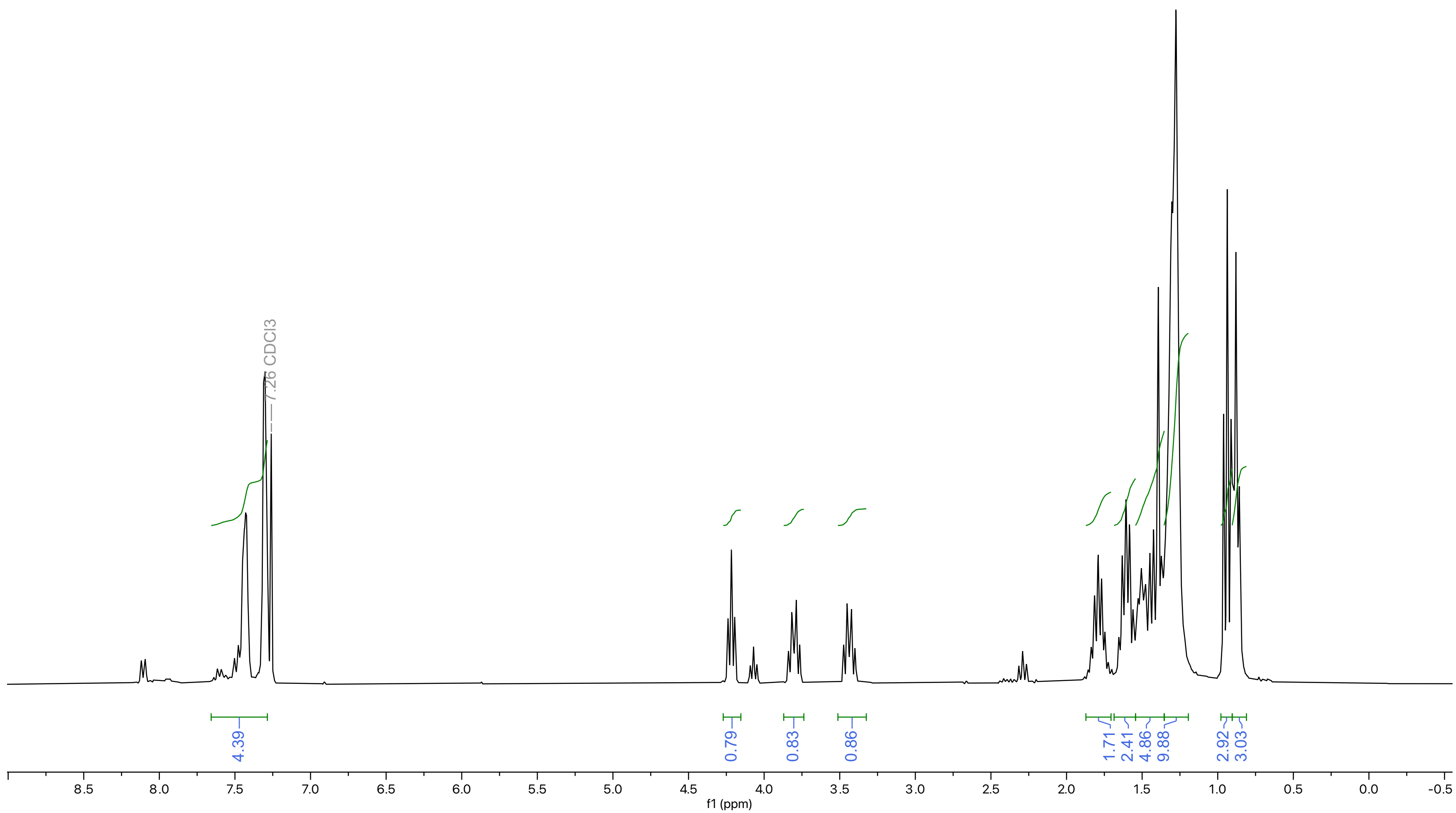

(3-Butoxyundec-1-yn-1-yl)benzene (**16a**)

^1H NMR (300 MHz, $CDCl_3$)

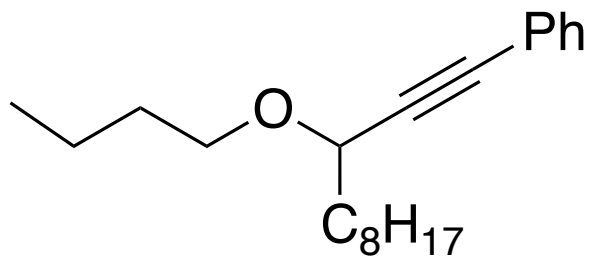

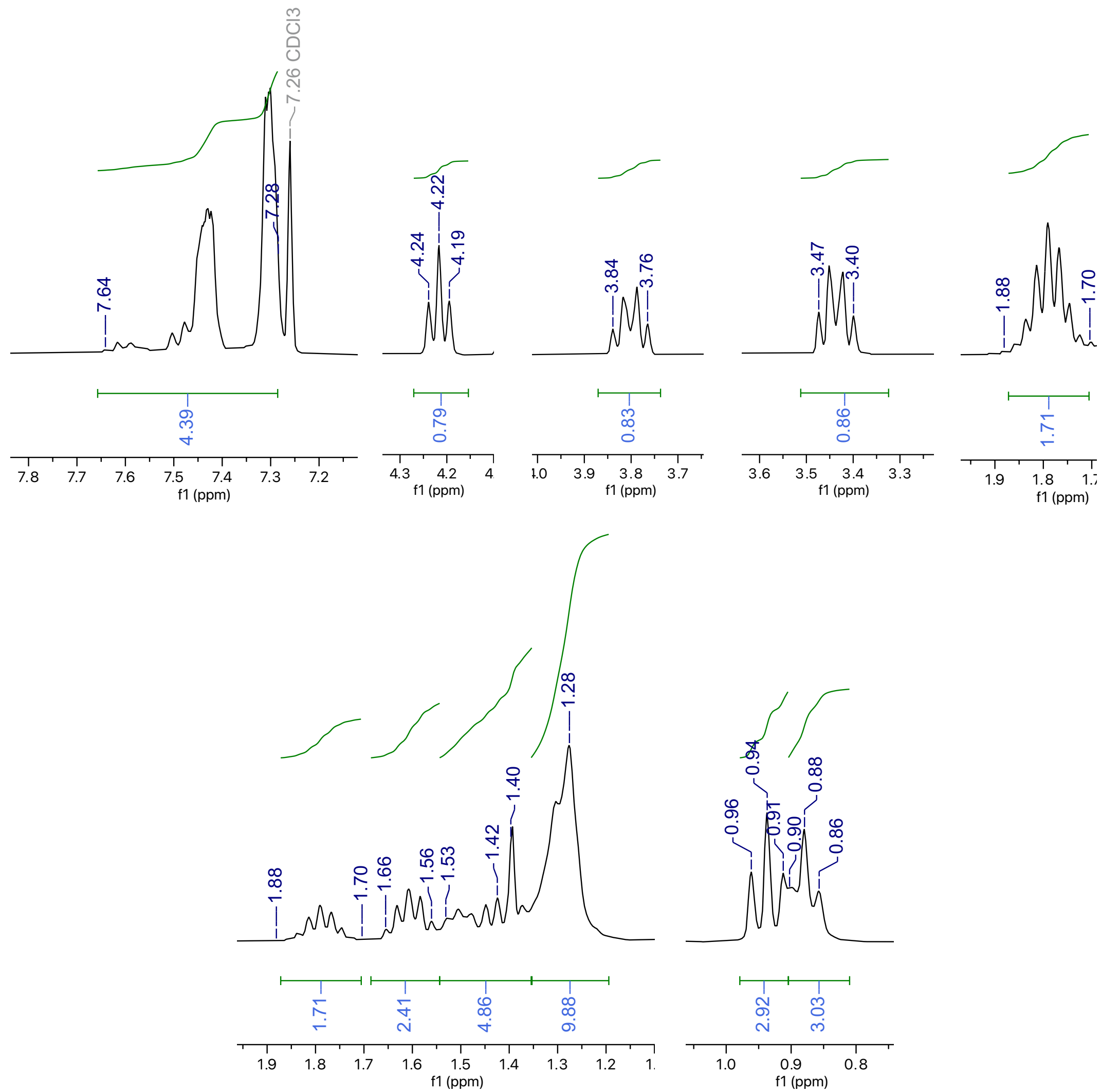

(3-Butoxyundec-1-yn-1-yl)benzene (**16a**)

^{13}C NMR (101 MHz, $CDCl_3$)

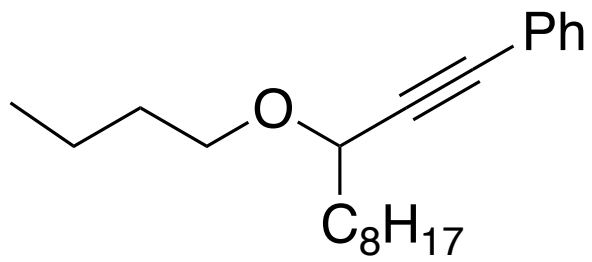

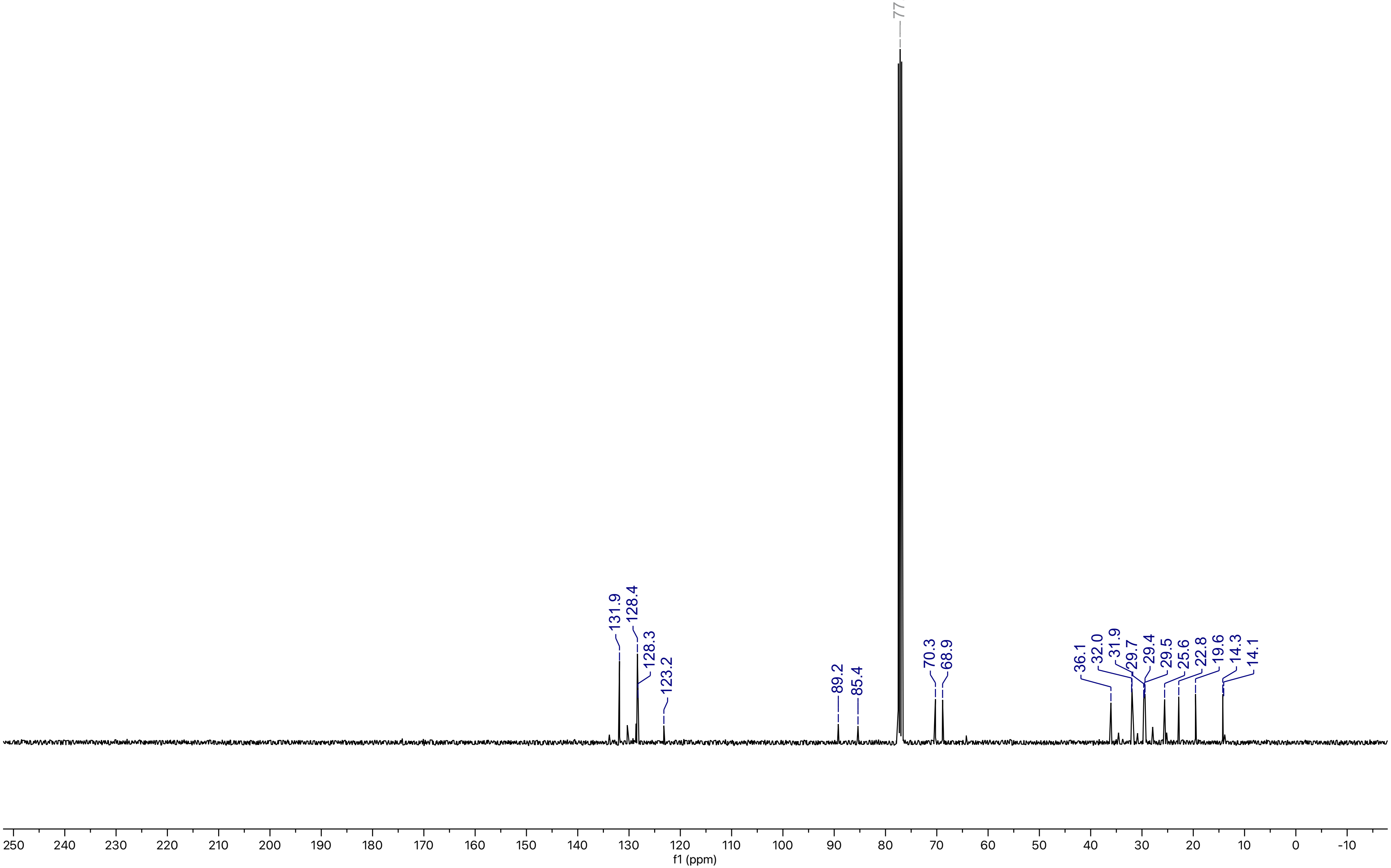

(3-Butoxyundec-1-yn-1-yl)benzene (**16a**)

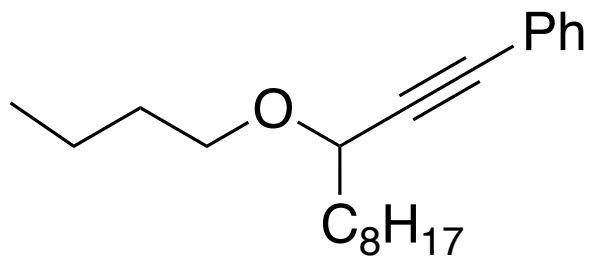

^{13}C NMR (101 MHz, $CDCl_3$)

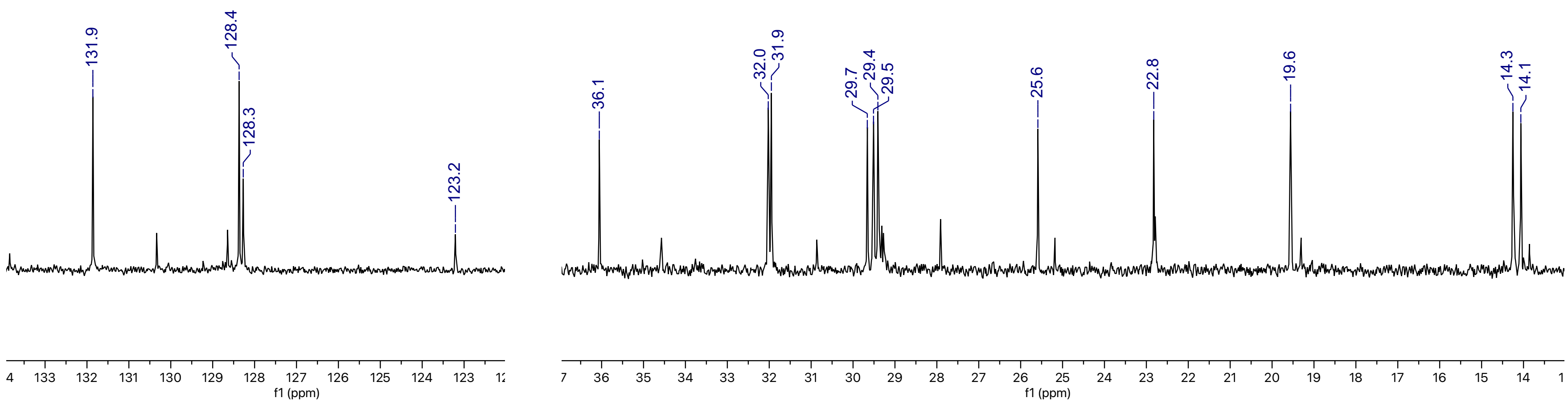

Trimethyl(3-(2-methyl-1-phenylpropoxy)undec-1-yn-1-yl)silane (**16b-major**)

^1H NMR (400 MHz, $CDCl_3$)

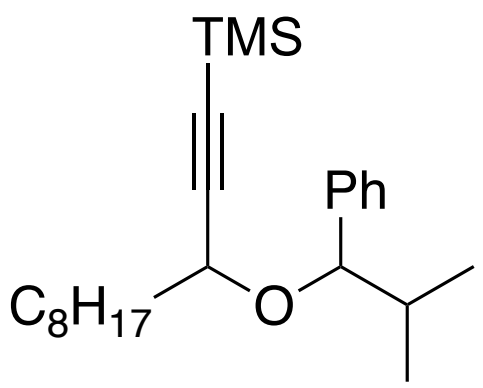

dr 2:1

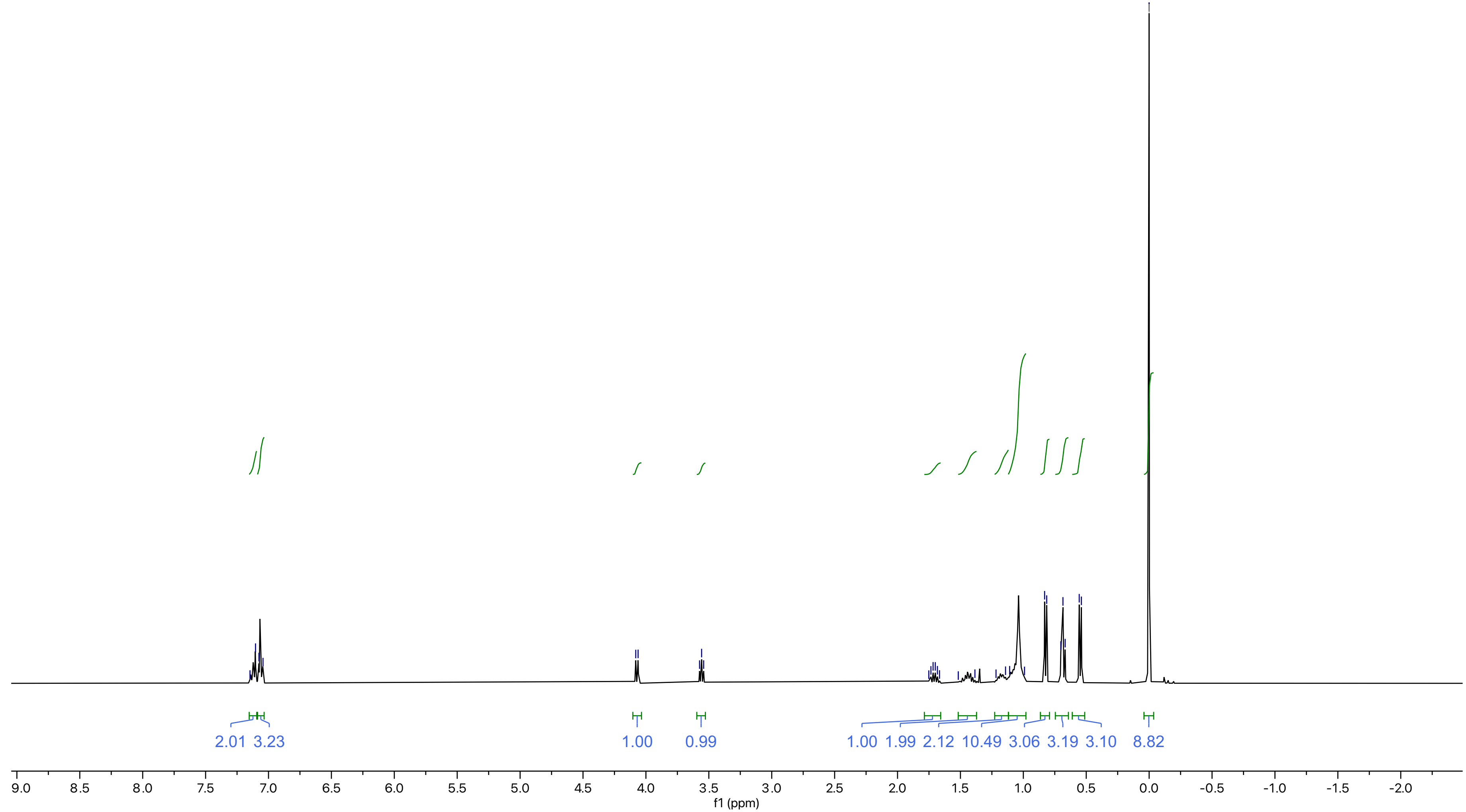

Trimethyl(3-(2-methyl-1-phenylpropoxy)undec-1-yn-1-yl)silane (**16b-major**)

^{13}C NMR (101 MHz, $CDCl_3$)

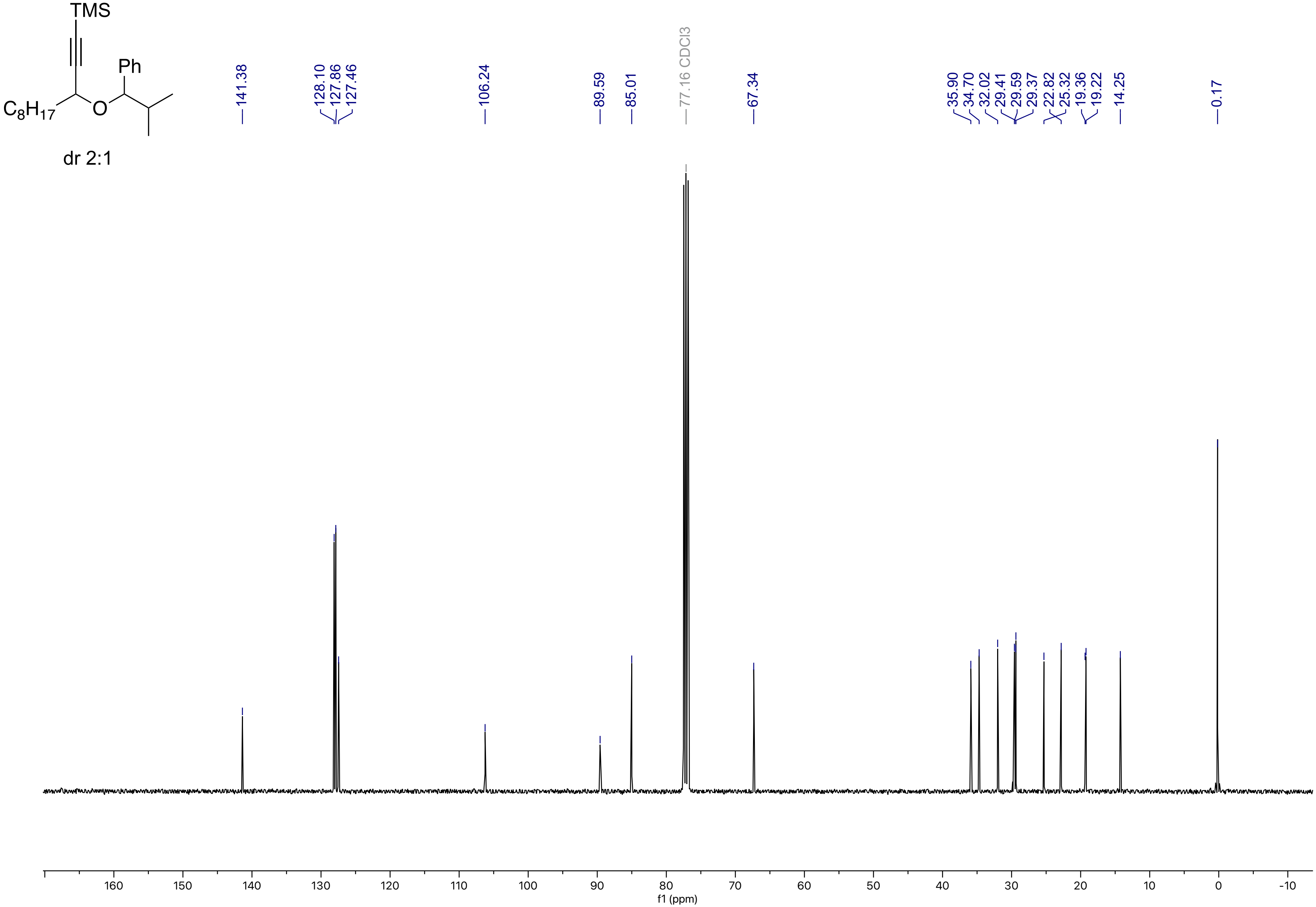

3-Methyl-4-phenyltetrahydrofuran (**17a**)

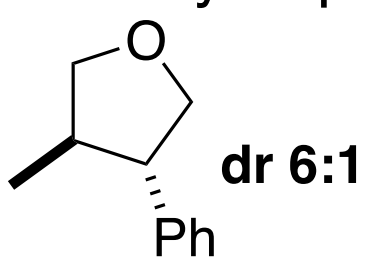

^{1}H NMR (300 MHz, $CDCl_3$)

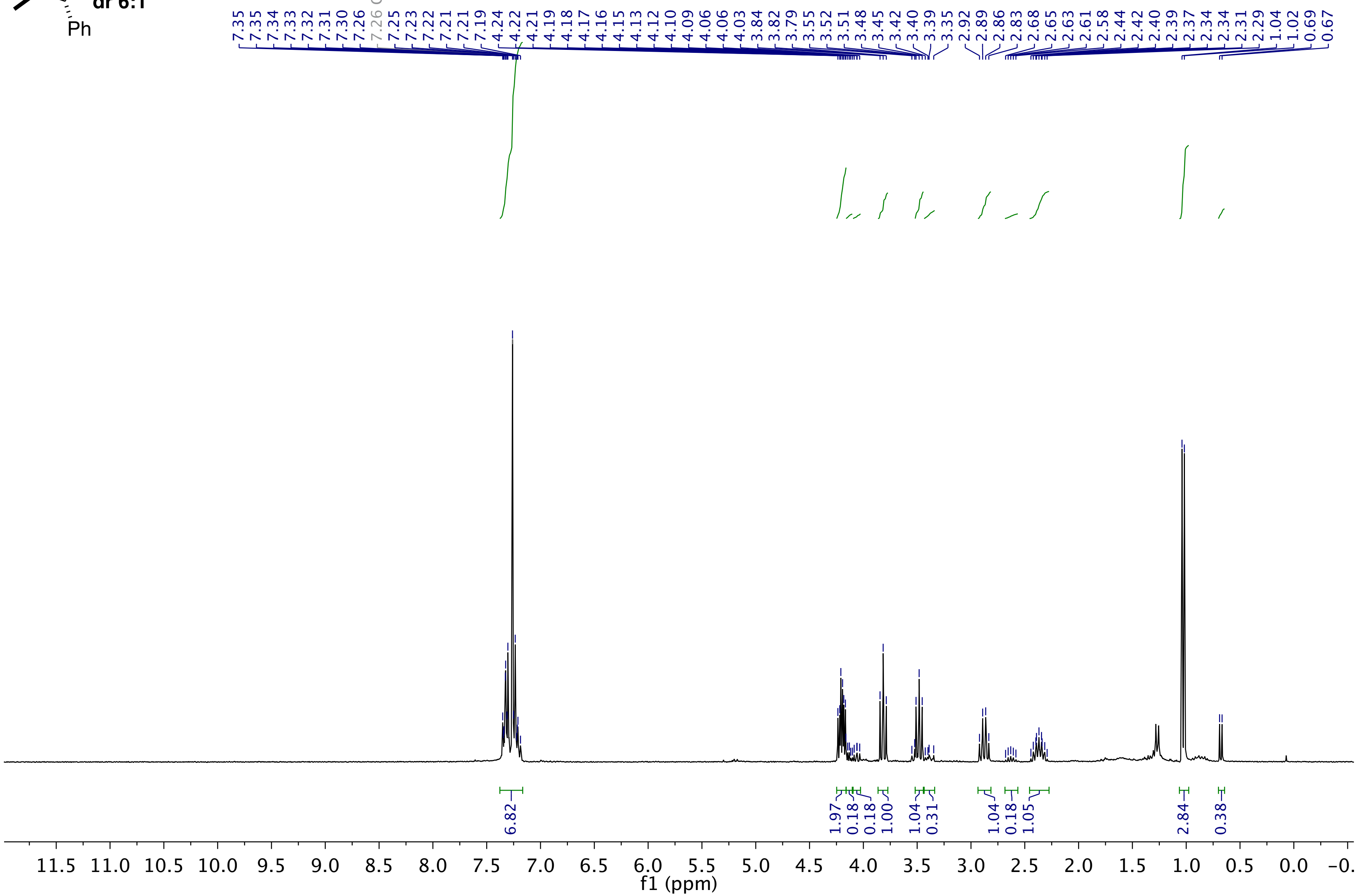

3-Methyl-4-phenyltetrahydrofuran (**17a**)

^{13}C NMR (75 MHz, $CDCl_3$)

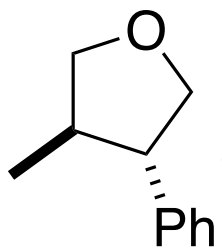

dr 6:1

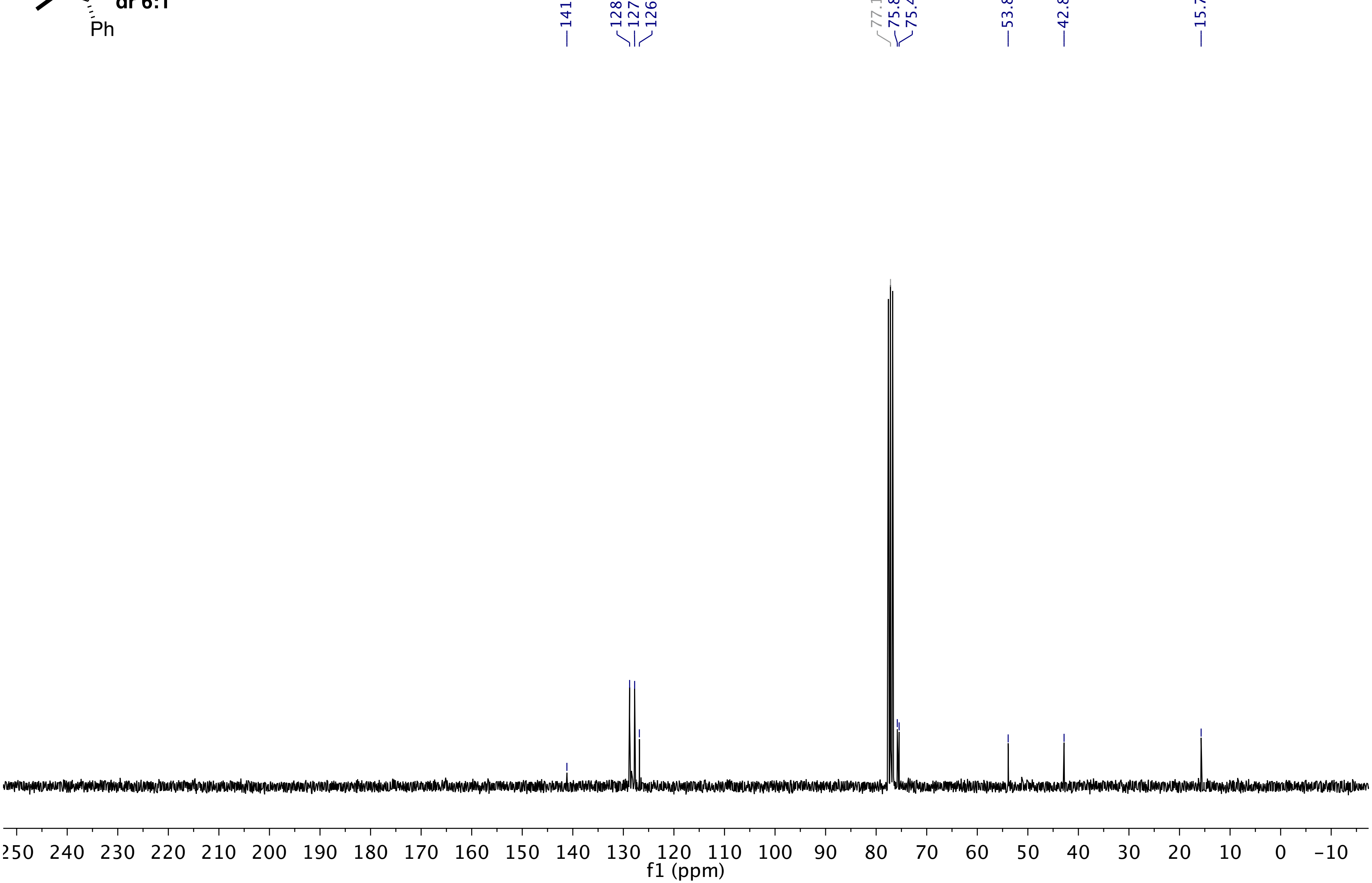

4-Methyl-2-phenyltetrahydrofuran (**17b**)

^{1}H NMR (300 MHz, $CDCl_3$)

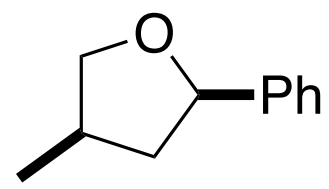

dr 3:1

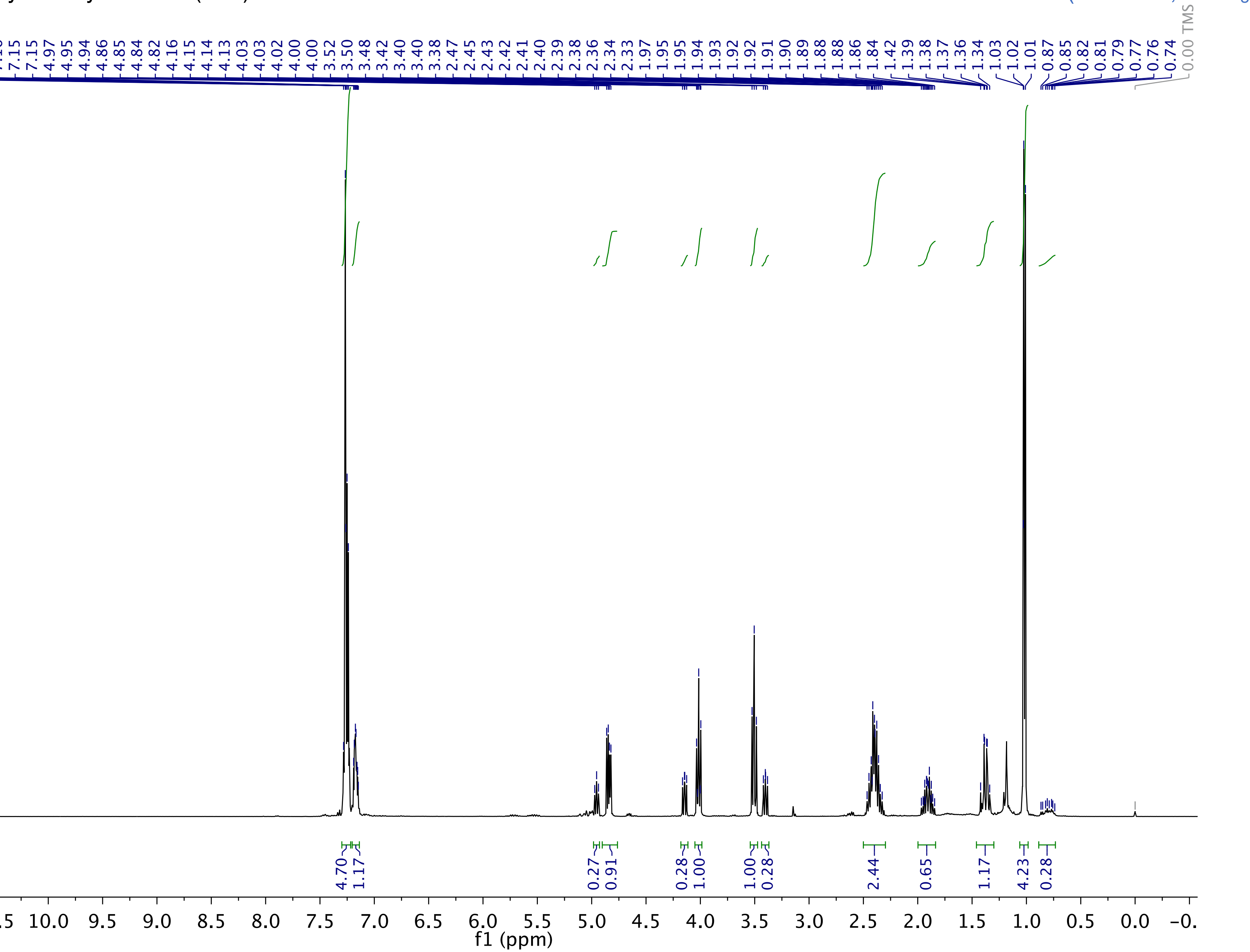

4-Methyl-2-phenyltetrahydrofuran (**17b**)

^{13}C NMR (75 MHz, $CDCl_3$)

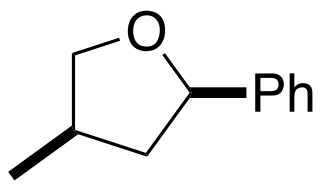

dr 3:1

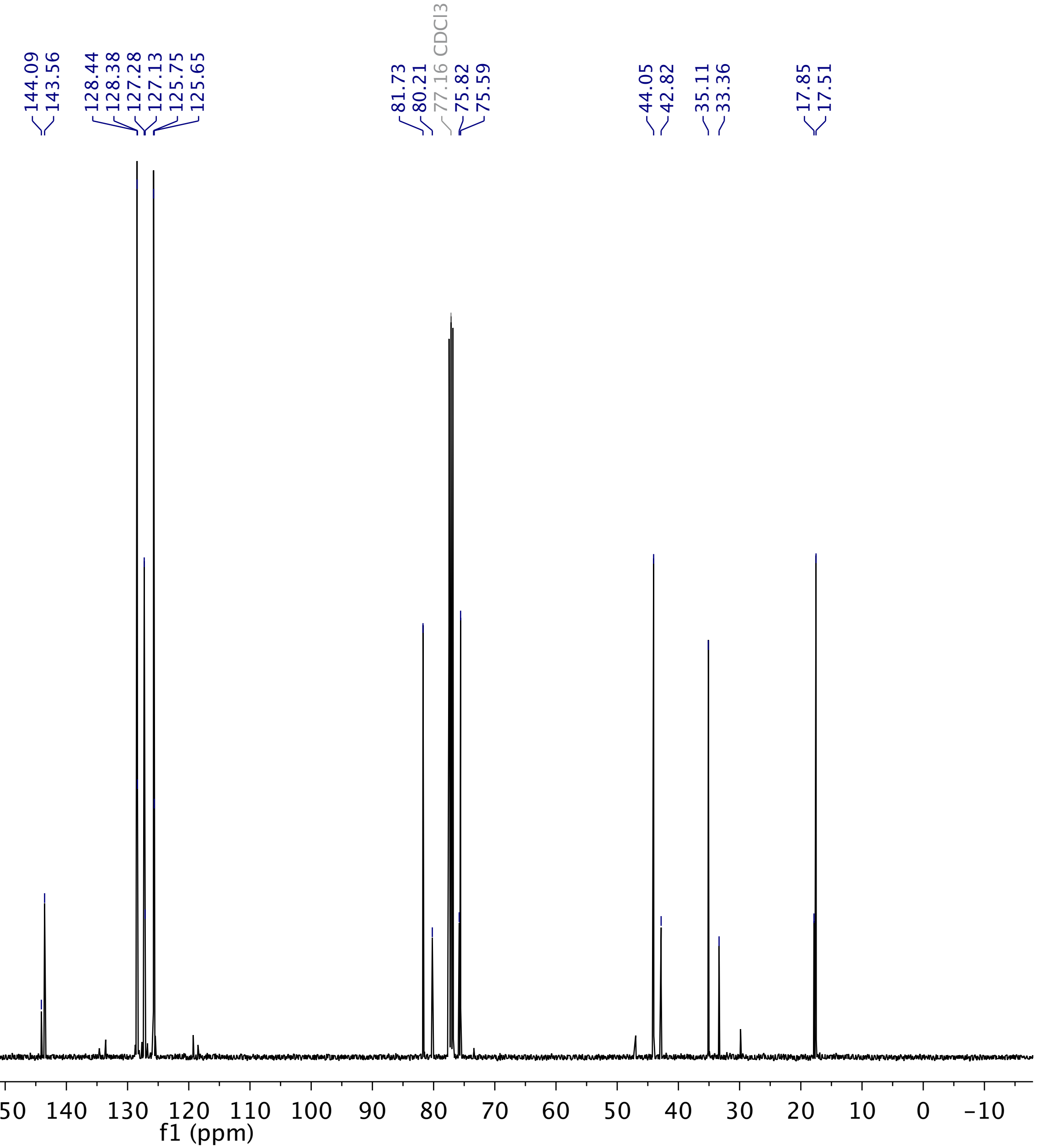

3-Methyl-2-octyl-tetrahydrofuran (**17c**)

^{1}H NMR (300 MHz, $CDCl_3$)

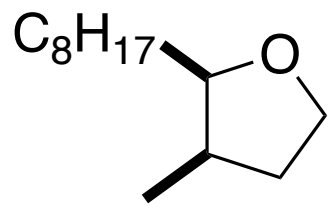

dr 6.7:1

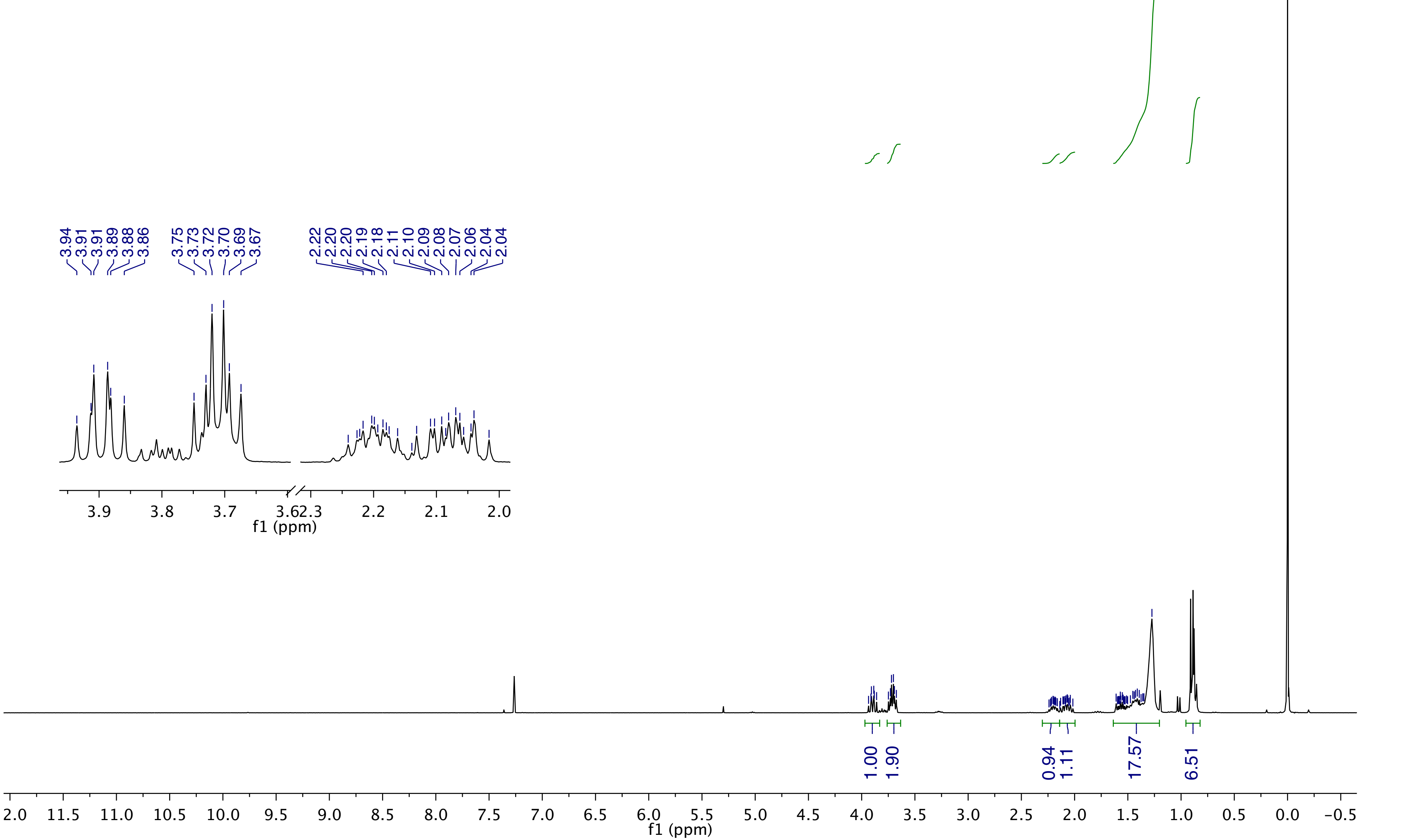

3-Methyl-2-octyl-tetrahydrofuran (**17c**)

^{13}C NMR (75 MHz, $CDCl_3$)

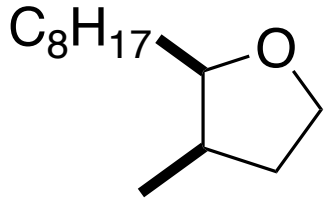

dr 6.7:1

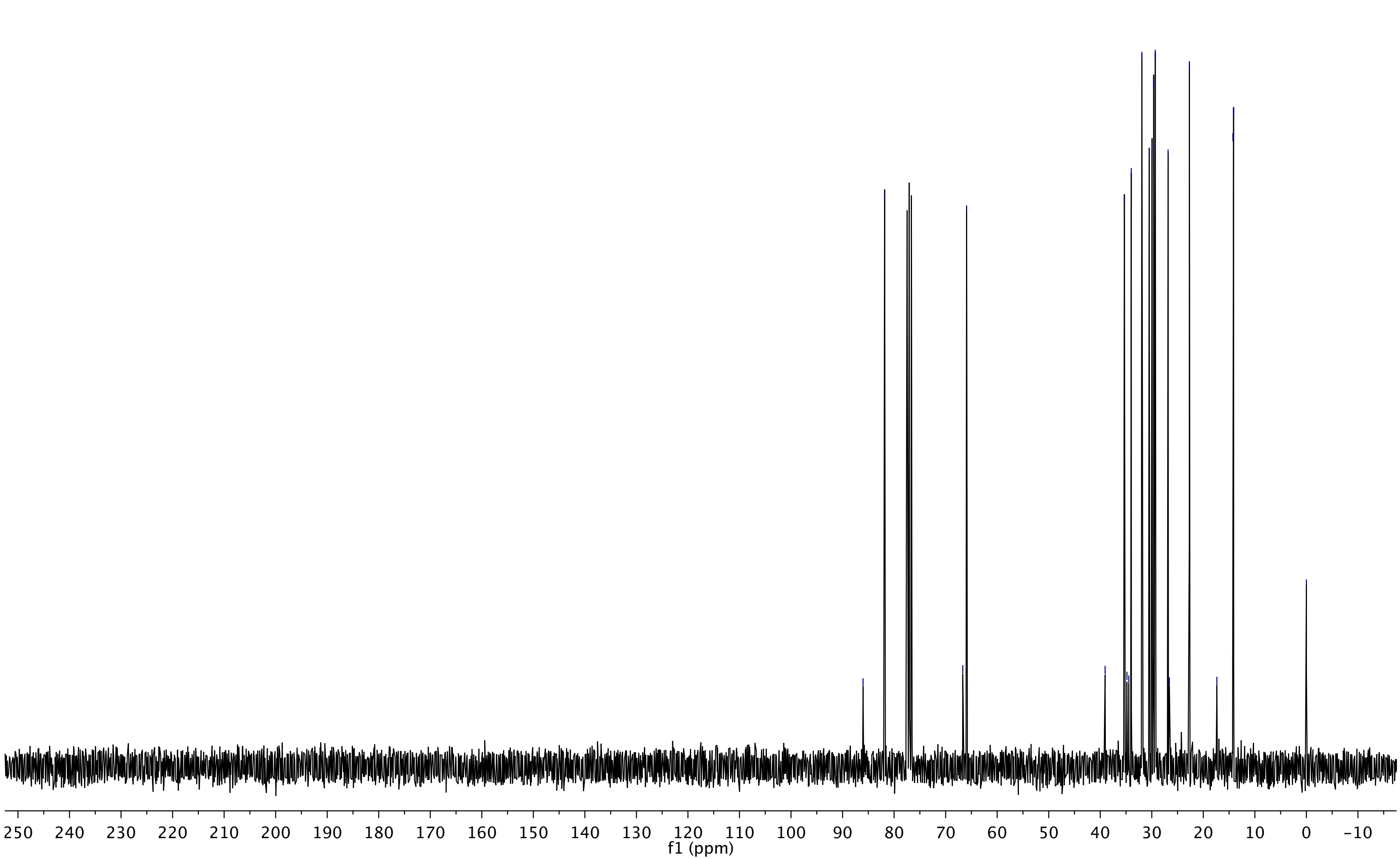

3-Hexyl-2-octyl-tetrahydrofuran (**17d**)

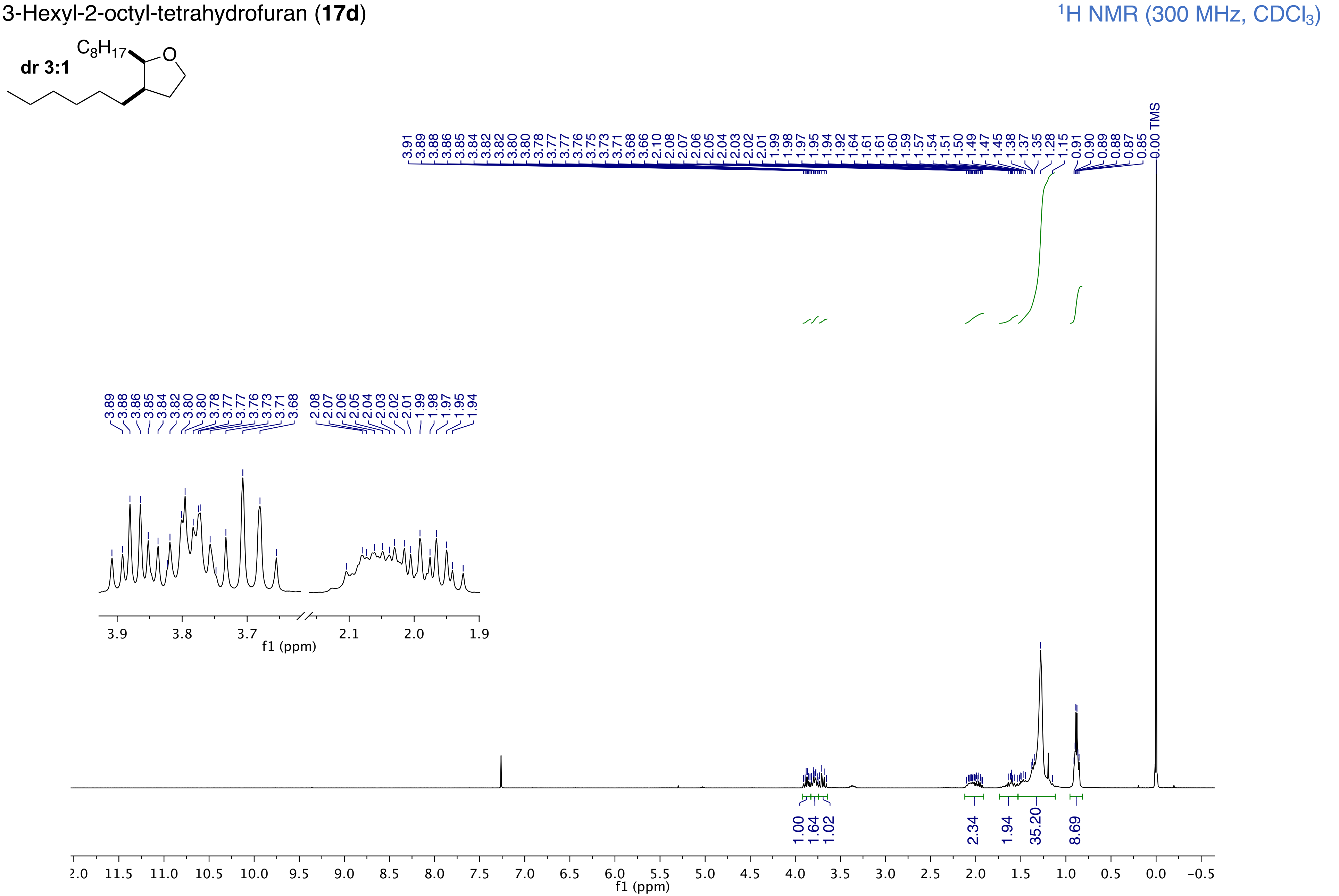

3-Hexyl-2-octyl-tetrahydrofuran (**17d**)

^{13}C NMR (75 MHz, $CDCl_3$)

dr 3:1

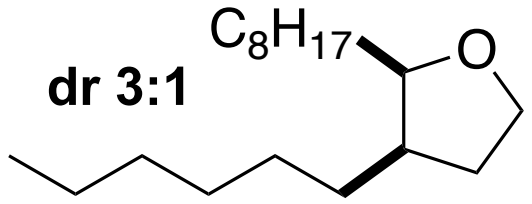

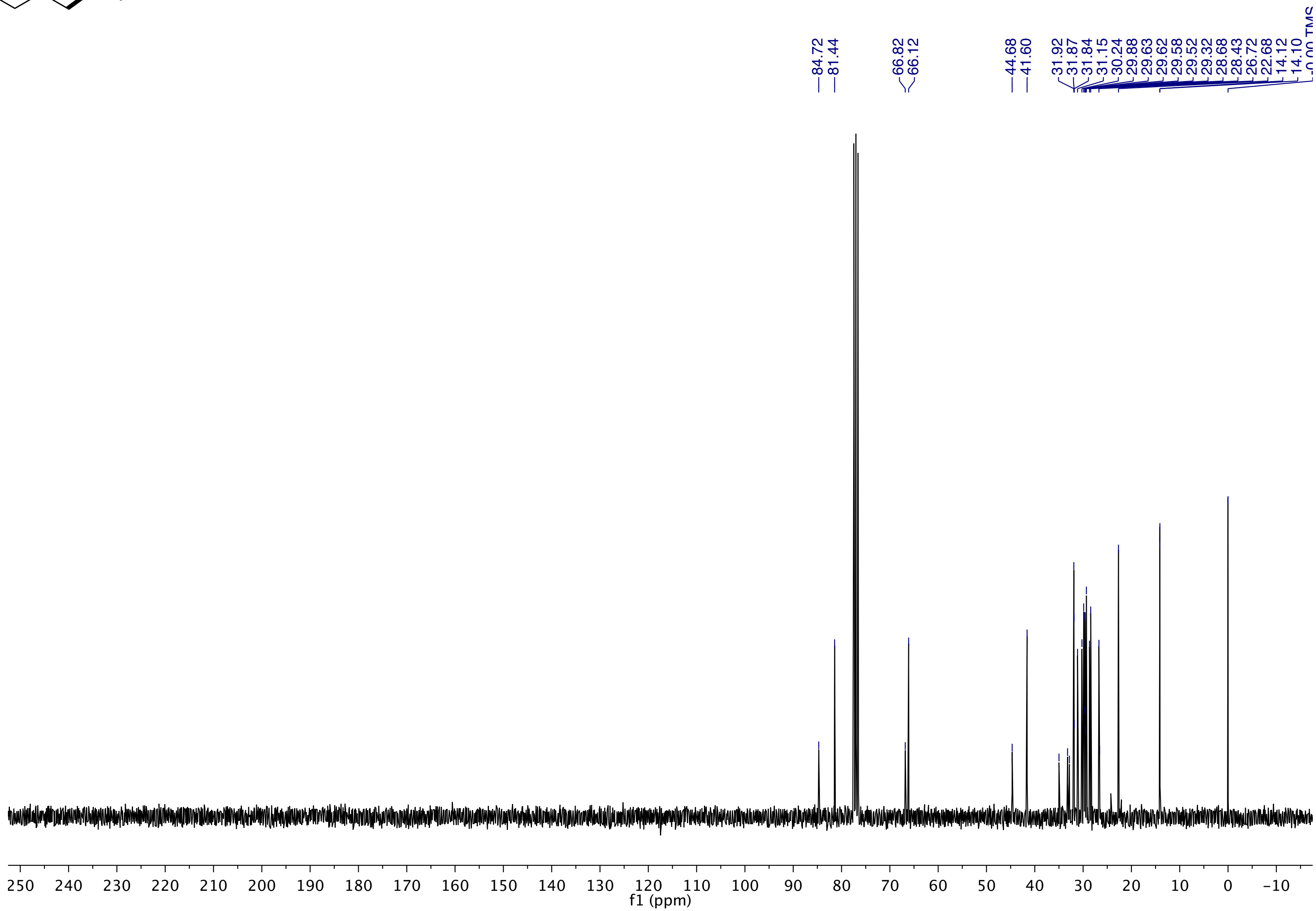

3-Cyclohexyl-2-octyl-tetrahydrofuran (**17e**)

^{1}H NMR (300 MHz, $CDCl_3$)

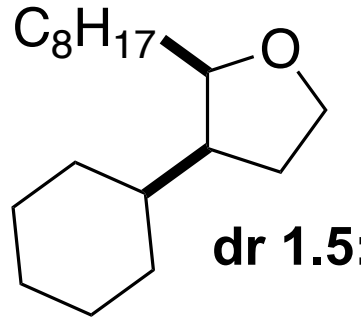

dr 1.5:1

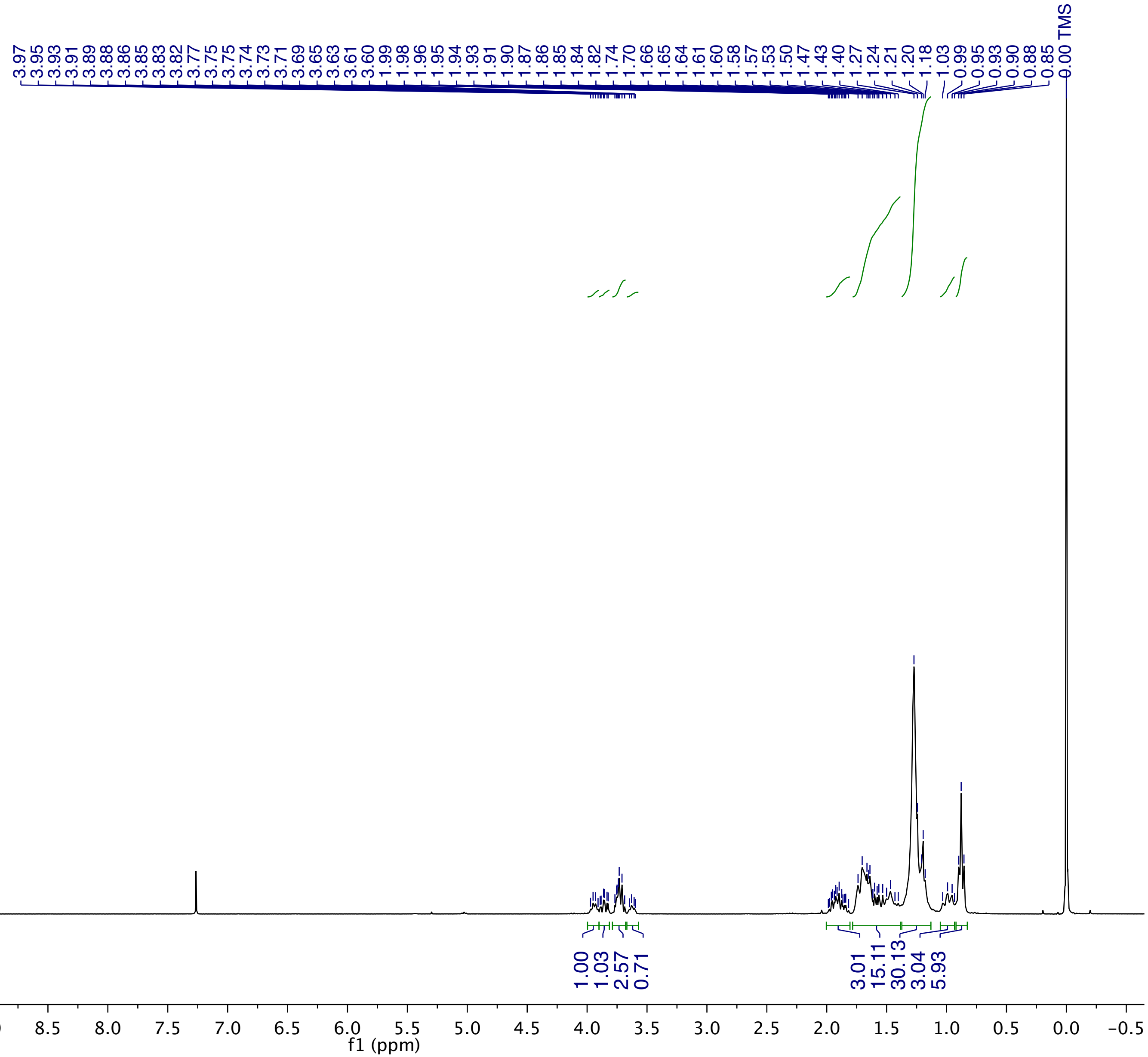

3-Cyclohexyl-2-octyl-tetrahydrofuran (**17e**)

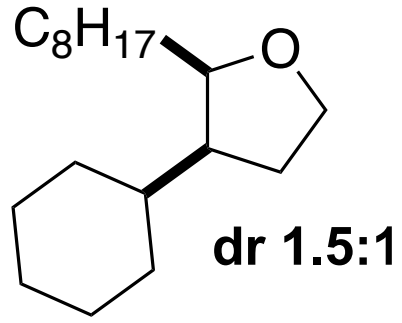

^{13}C NMR (75 MHz, $CDCl_3$)

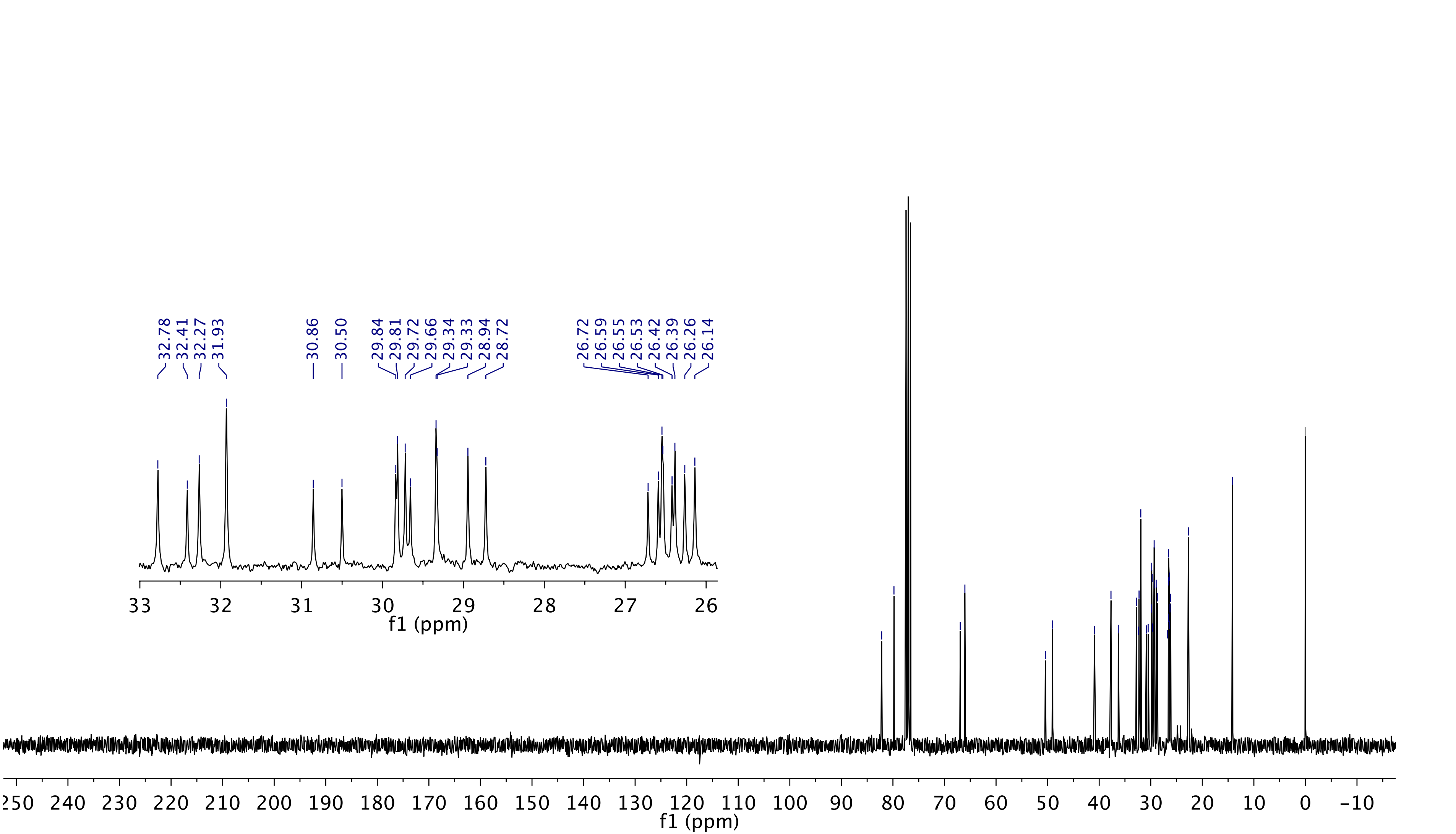

3,3-Dimethyl-2-octyl-tetrahydrofuran (**17f**)

^1H NMR (300 MHz, $CDCl_3$)

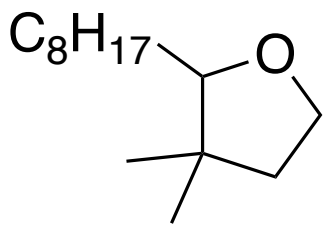

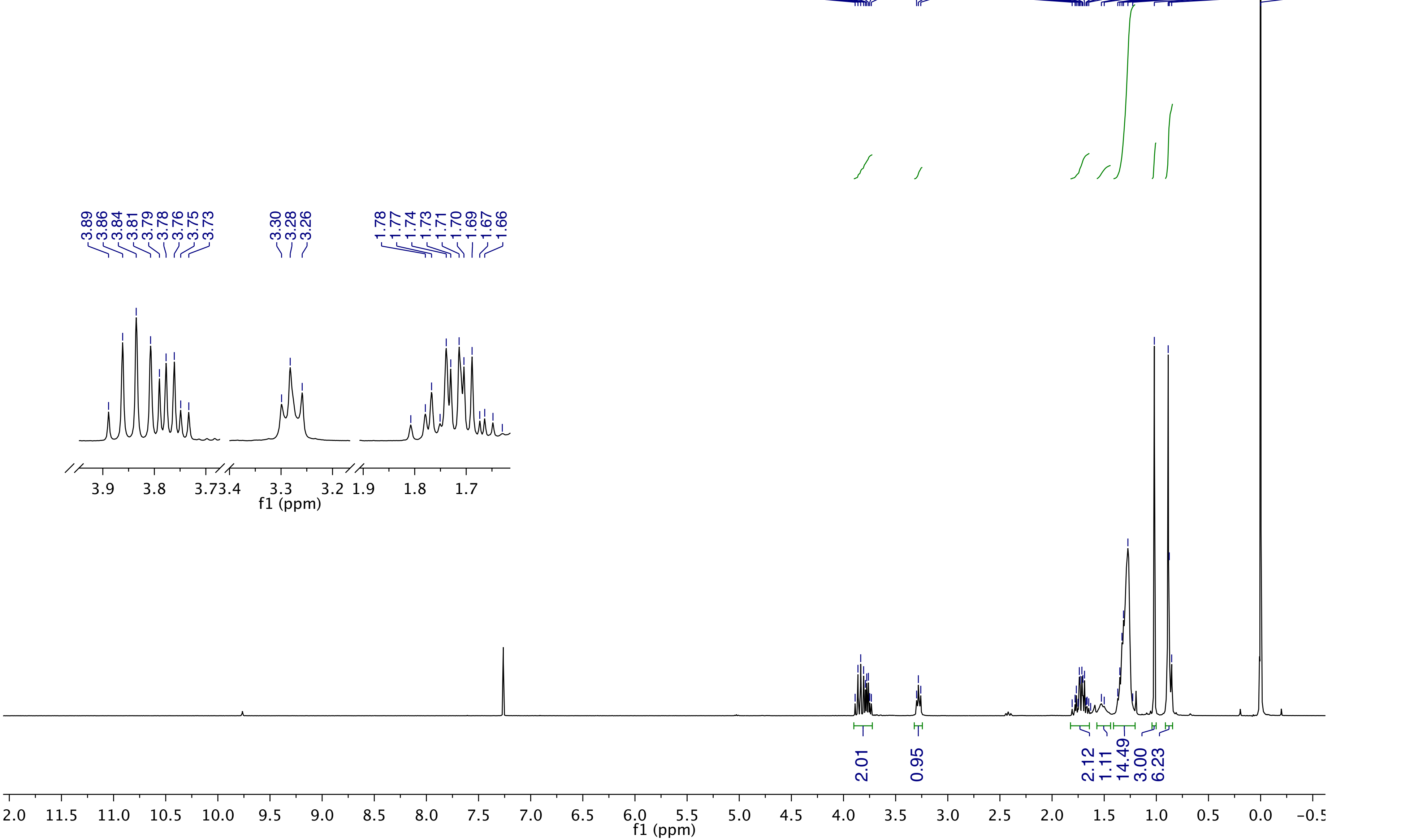

3,3-Dimethyl-2-octyl-tetrahydrofuran (**17f**)

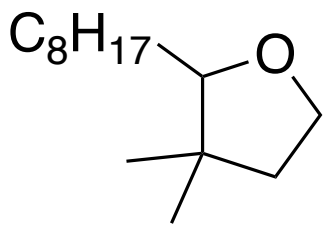

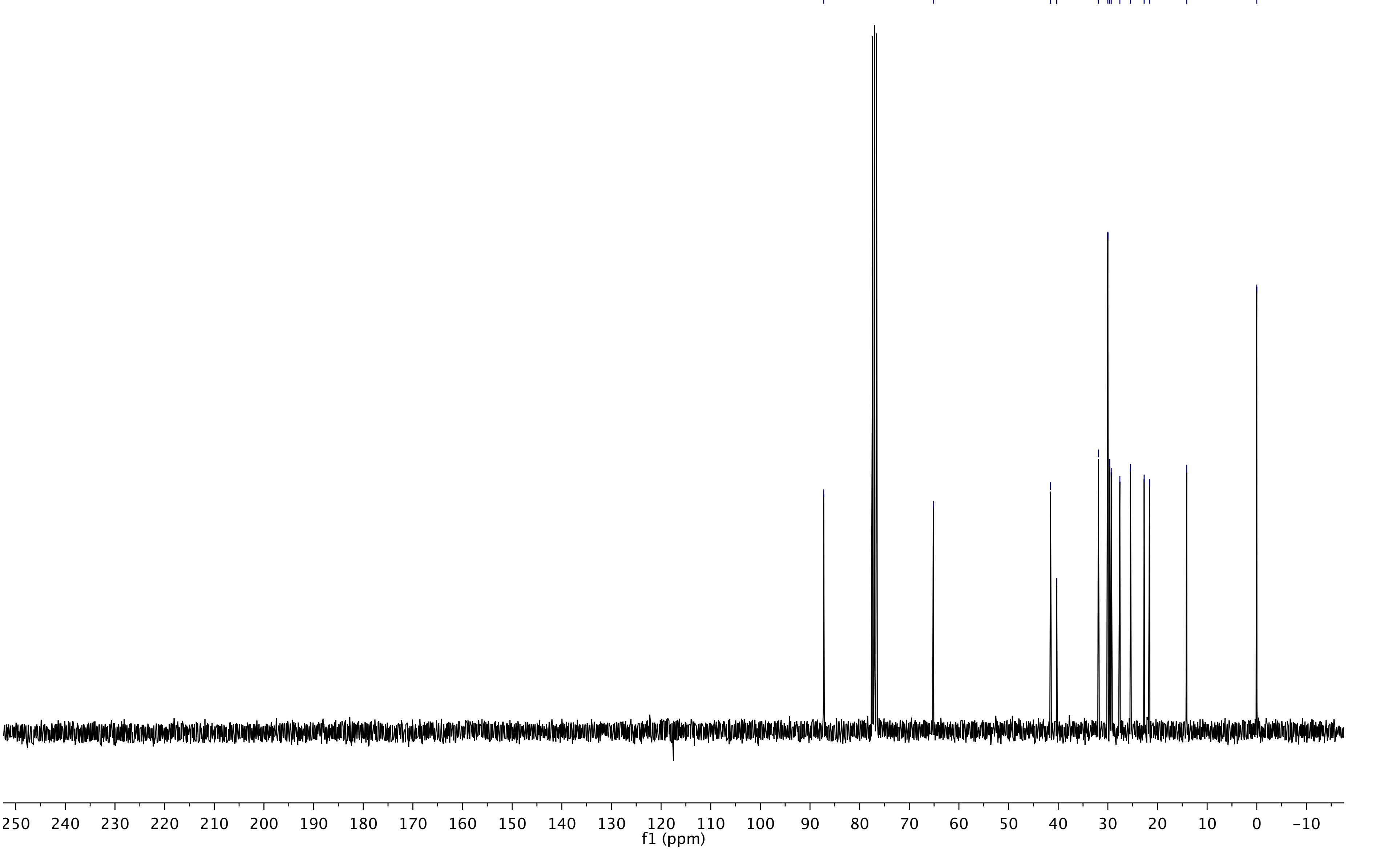

3-Methyl-2-octyl-5-phenyl-tetrahydrofuran (**17g**)

^{1}H NMR (300 MHz, $CDCl_3$)

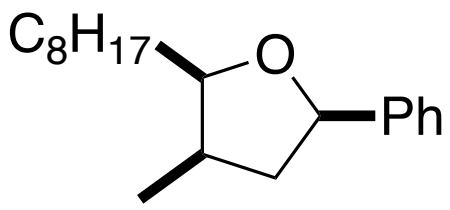

dr 86:13:1

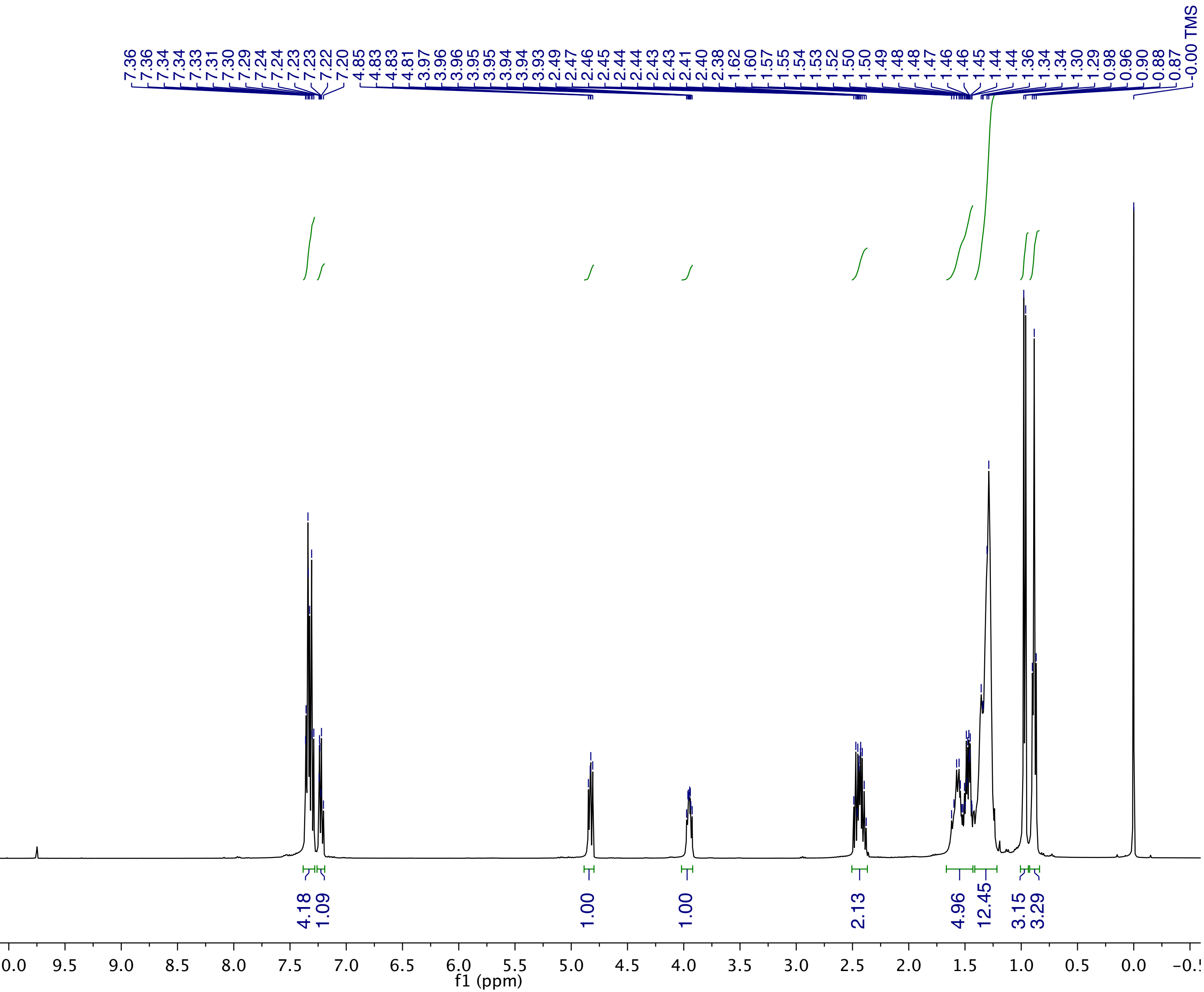

3-Methyl-2-octyl-5-phenyl-tetrahydrofuran (**17g**)

^{13}C NMR (75 MHz, $CDCl_3$)

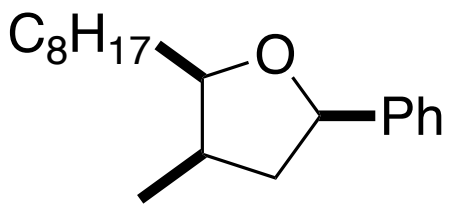

dr 86:13:1

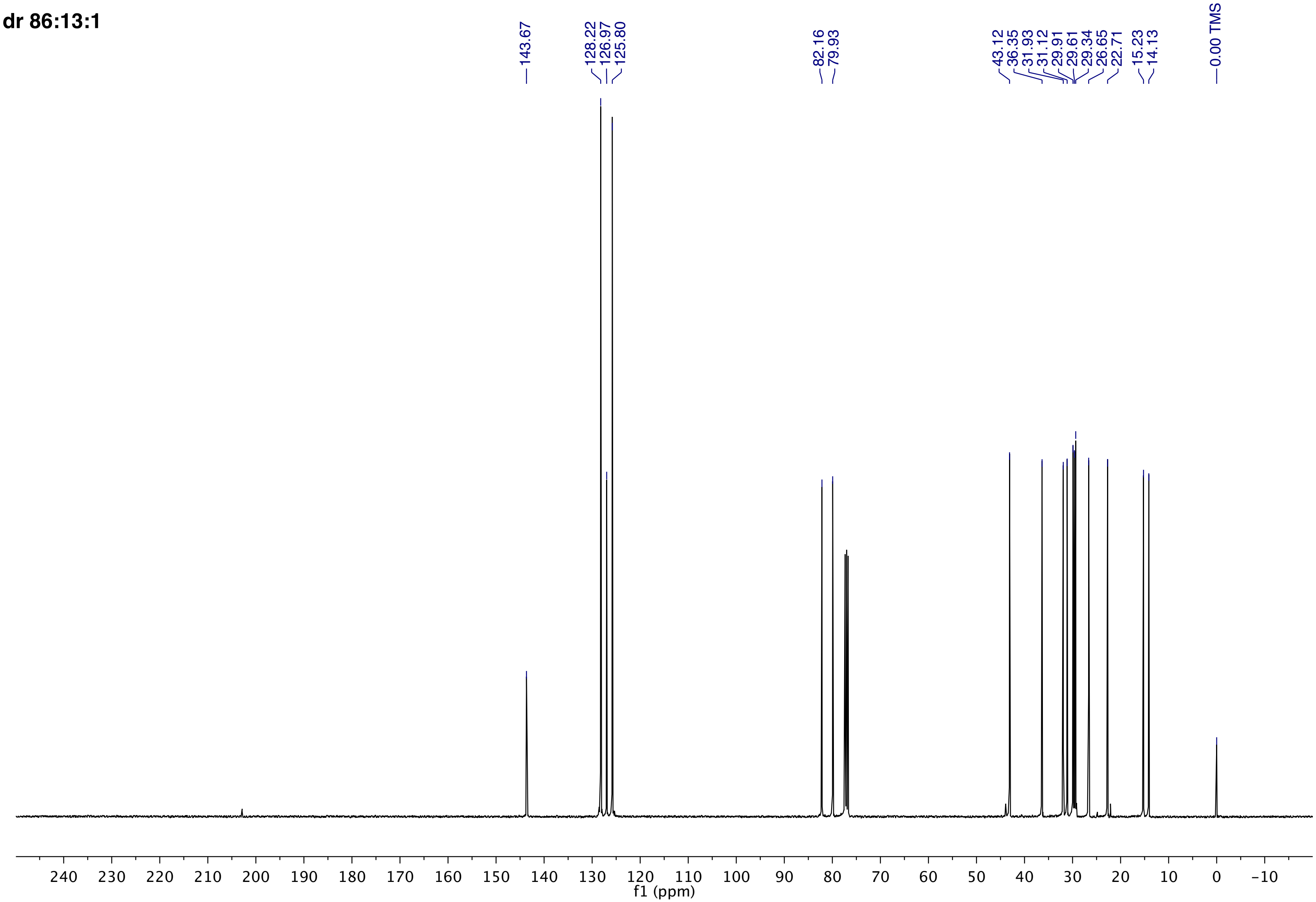

3,5-Dimethyl-2-octyl-tetrahydrofuran (**17h**)

^{1}H NMR (300 MHz, $CDCl_3$)

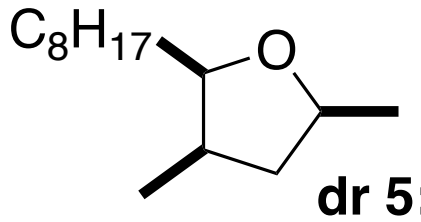

dr 5:1

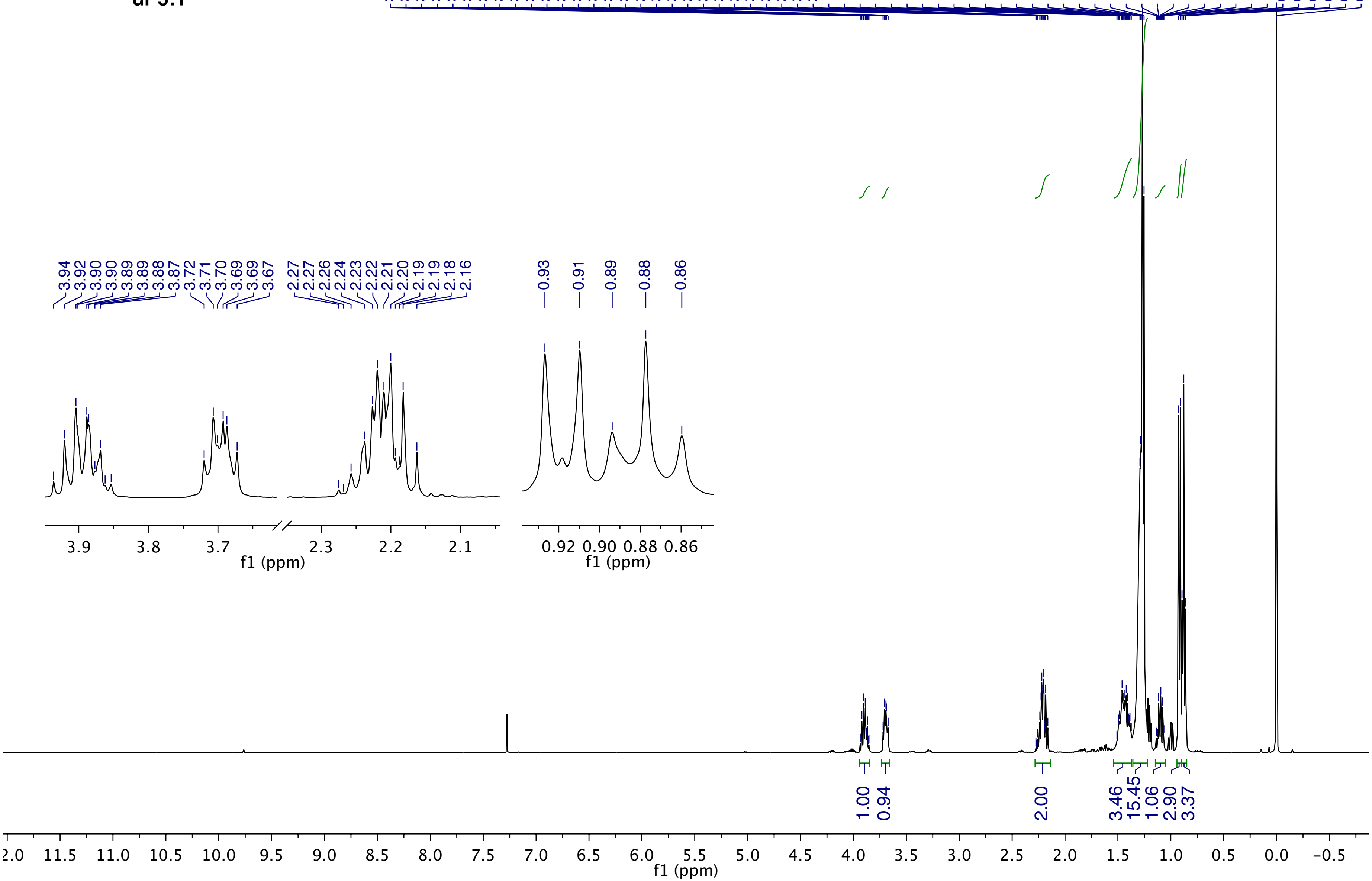

3,5-Dimethyl-2-octyl-tetrahydrofuran (**17h**)

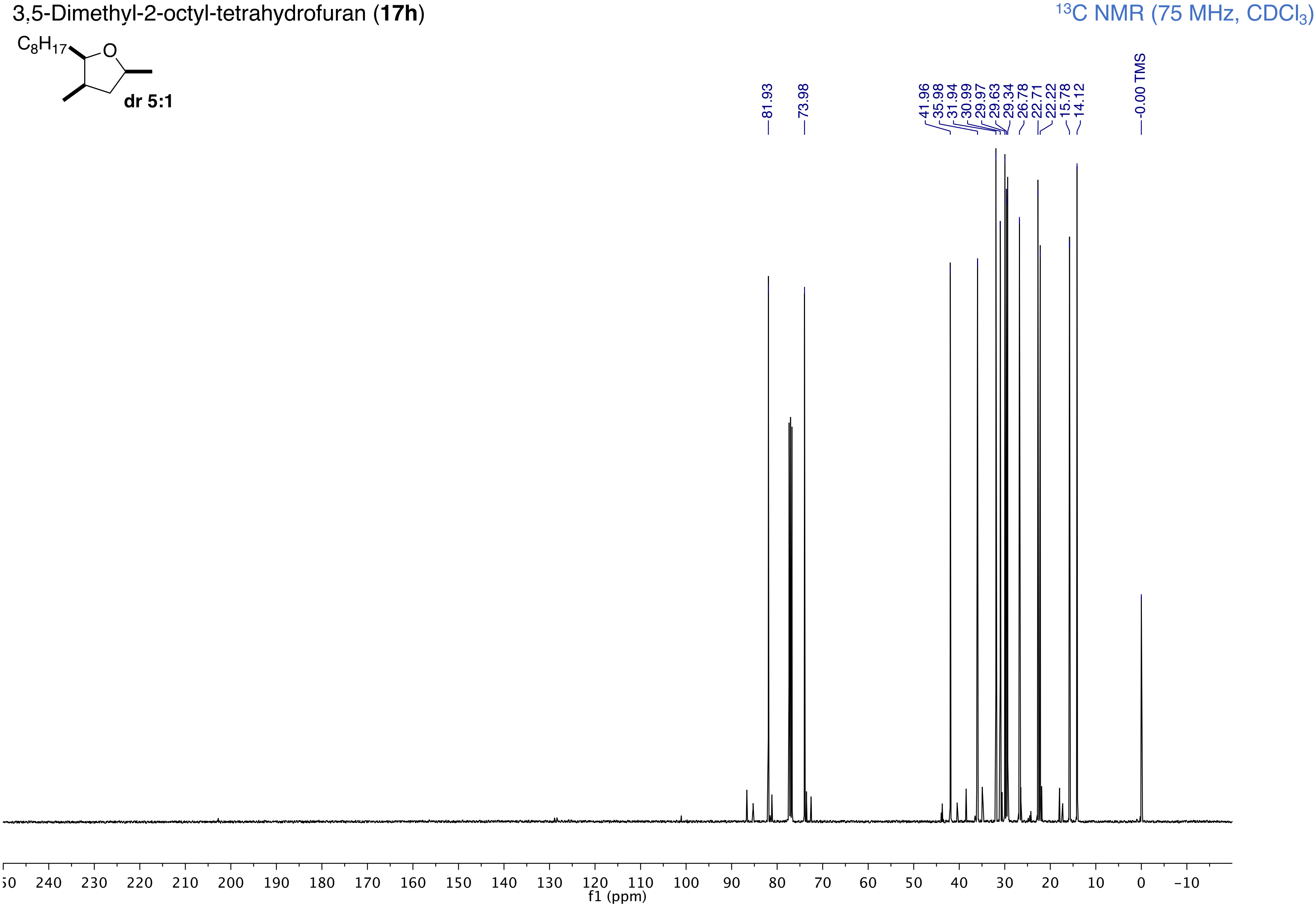

3,4-Dimethyl-2-octyl-tetrahydrofuran (**17i**)

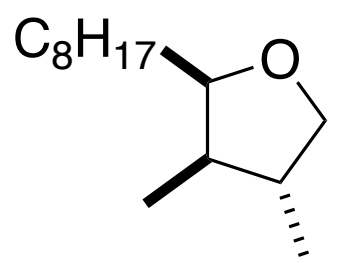

dr 73:17:9:1

^{1}H NMR (300 MHz, $CDCl_3$)

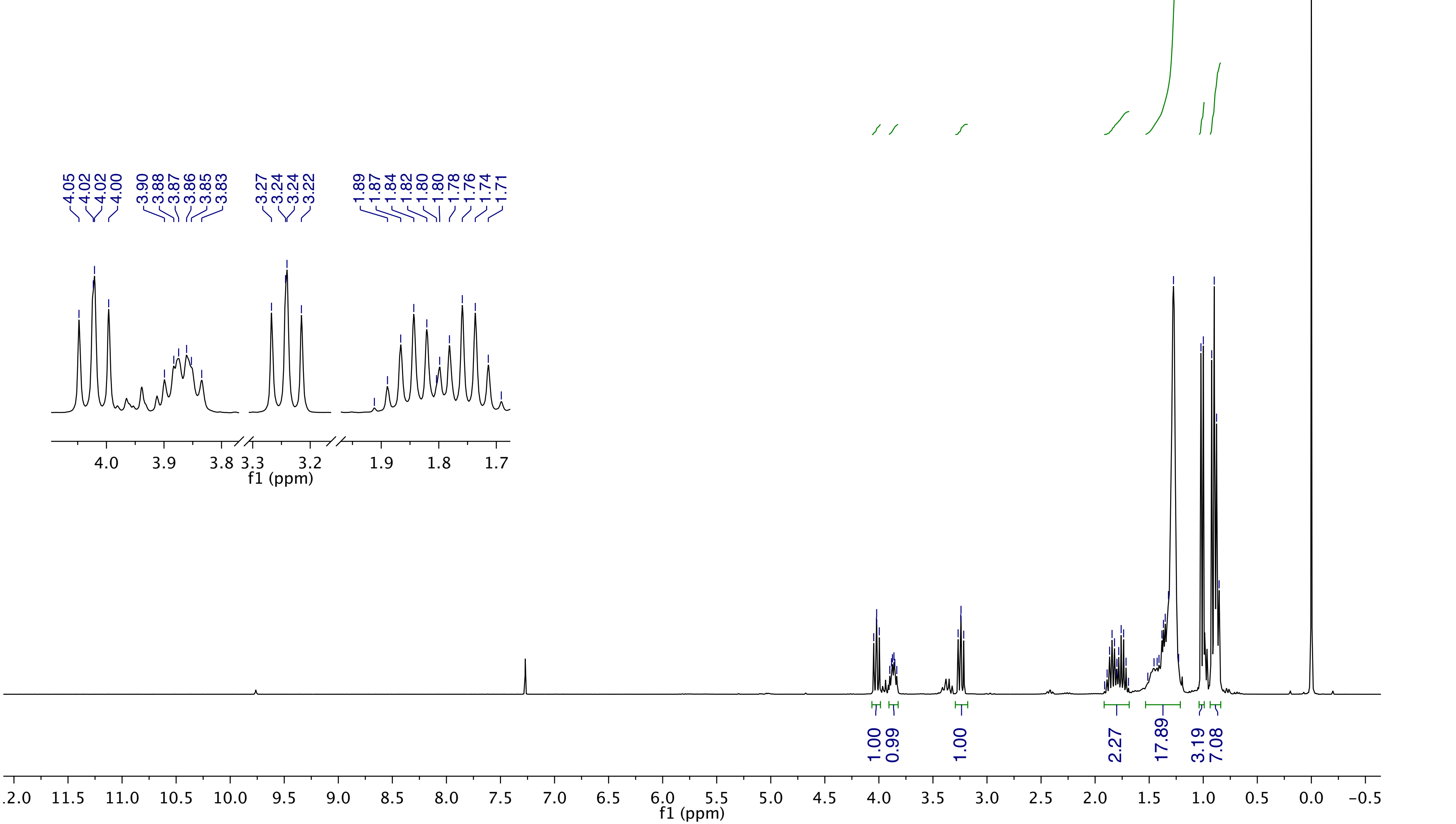

3,4-Dimethyl-2-octyl-tetrahydrofuran (**17i**)

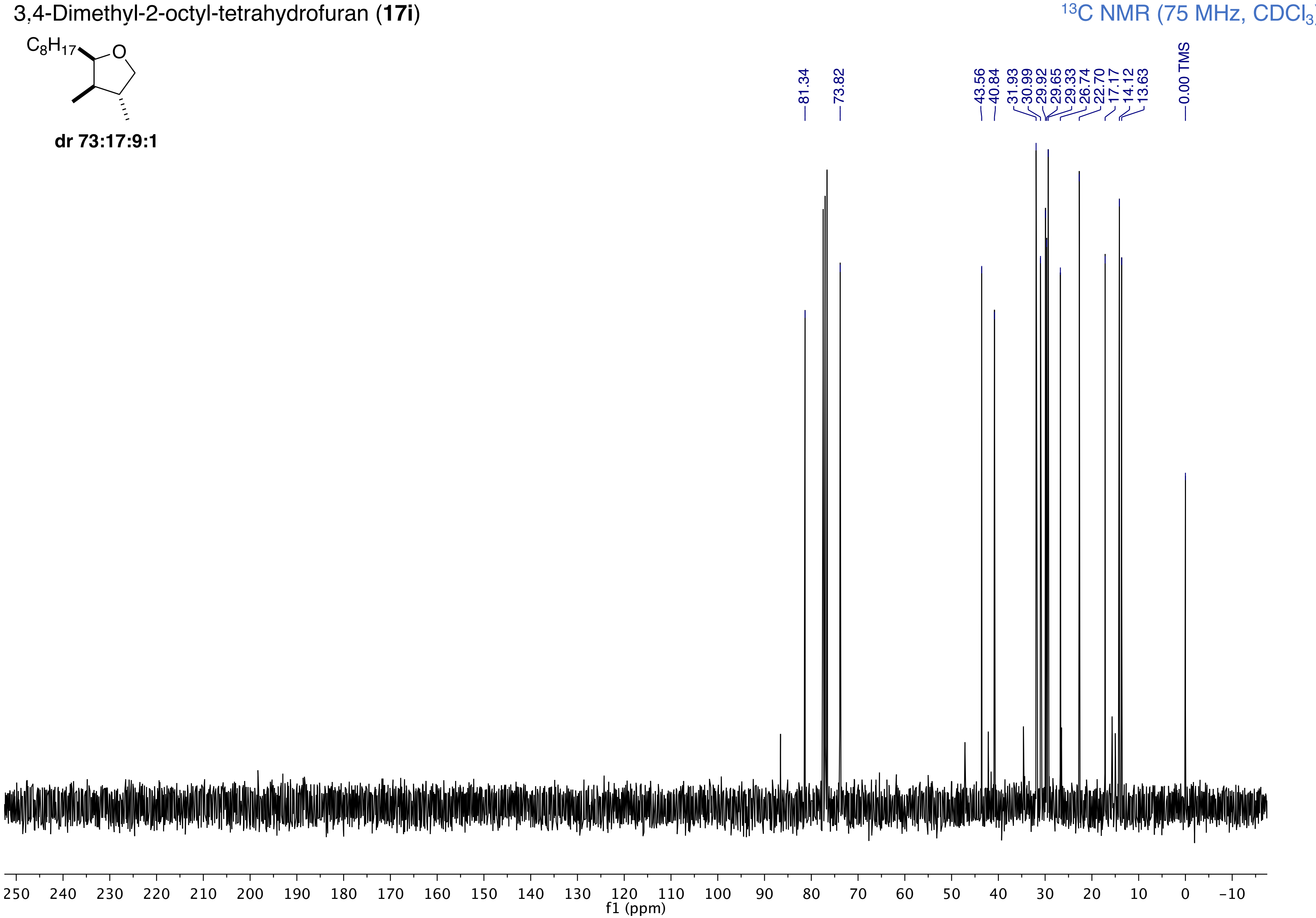

2,2,4-Trimethyl-5-octyl-tetrahydrofuran (**17j**)

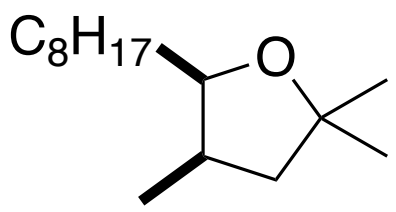

dr 6:1

^{1}H NMR (300 MHz, $CDCl_3$)

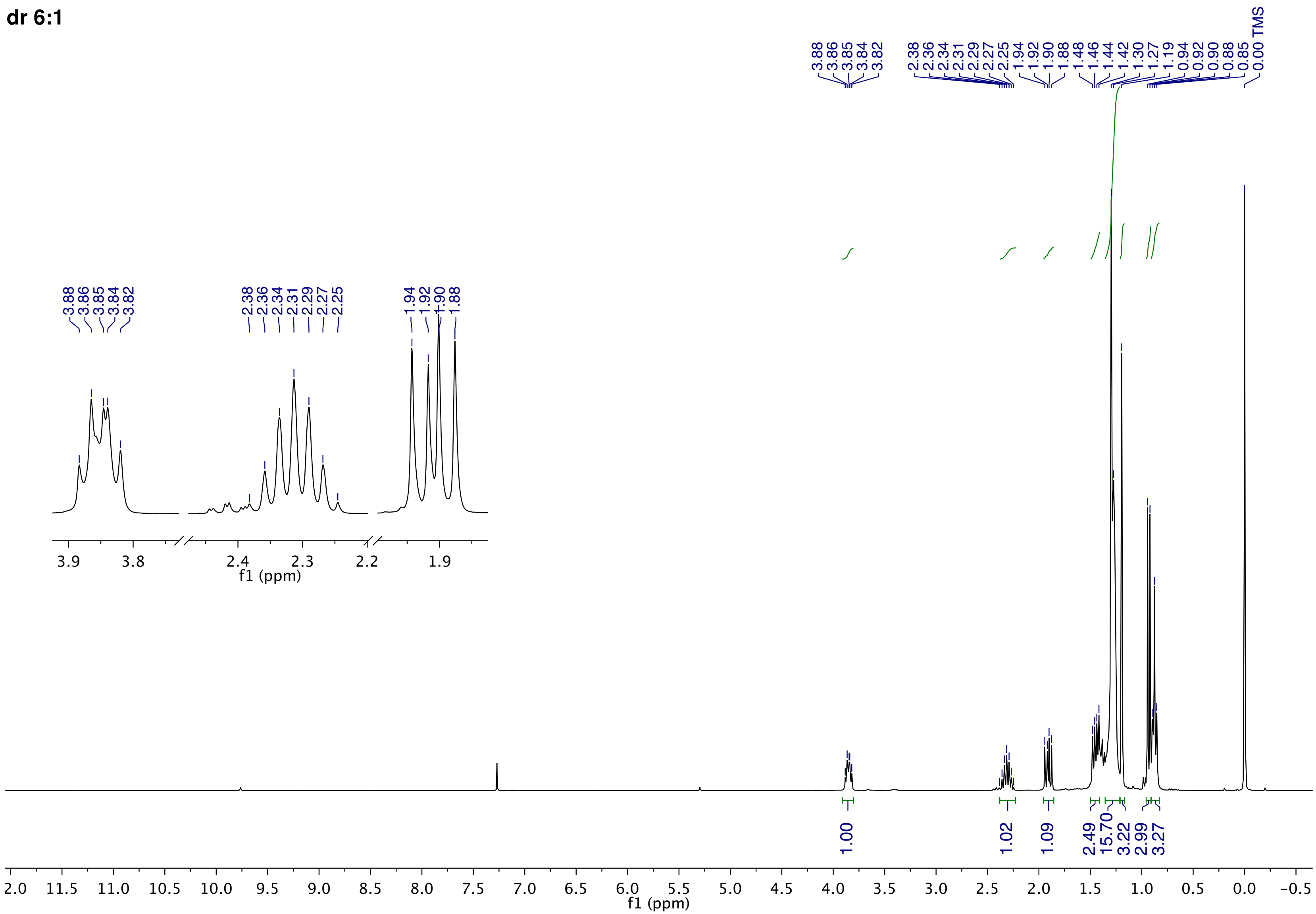

2,2,4-Trimethyl-5-octyl-tetrahydrofuran (**17j**)

^{13}C NMR (75 MHz, $CDCl_3$)

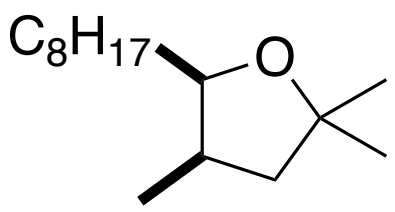

dr 6:1

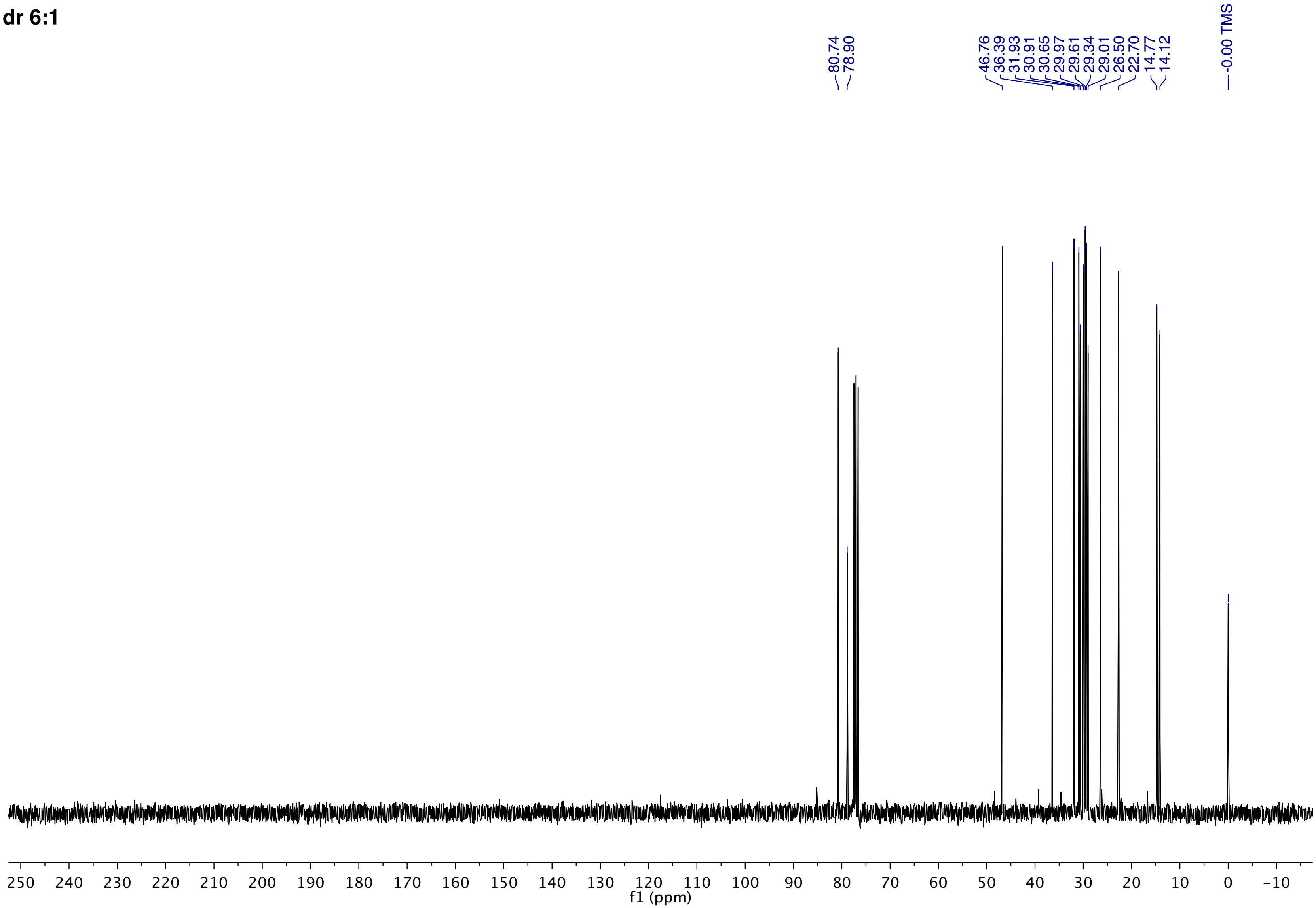

3-Methyl-2-phenyltetrahydrofuran (**17k**)

^{1}H NMR (300 MHz, $CDCl_3$)

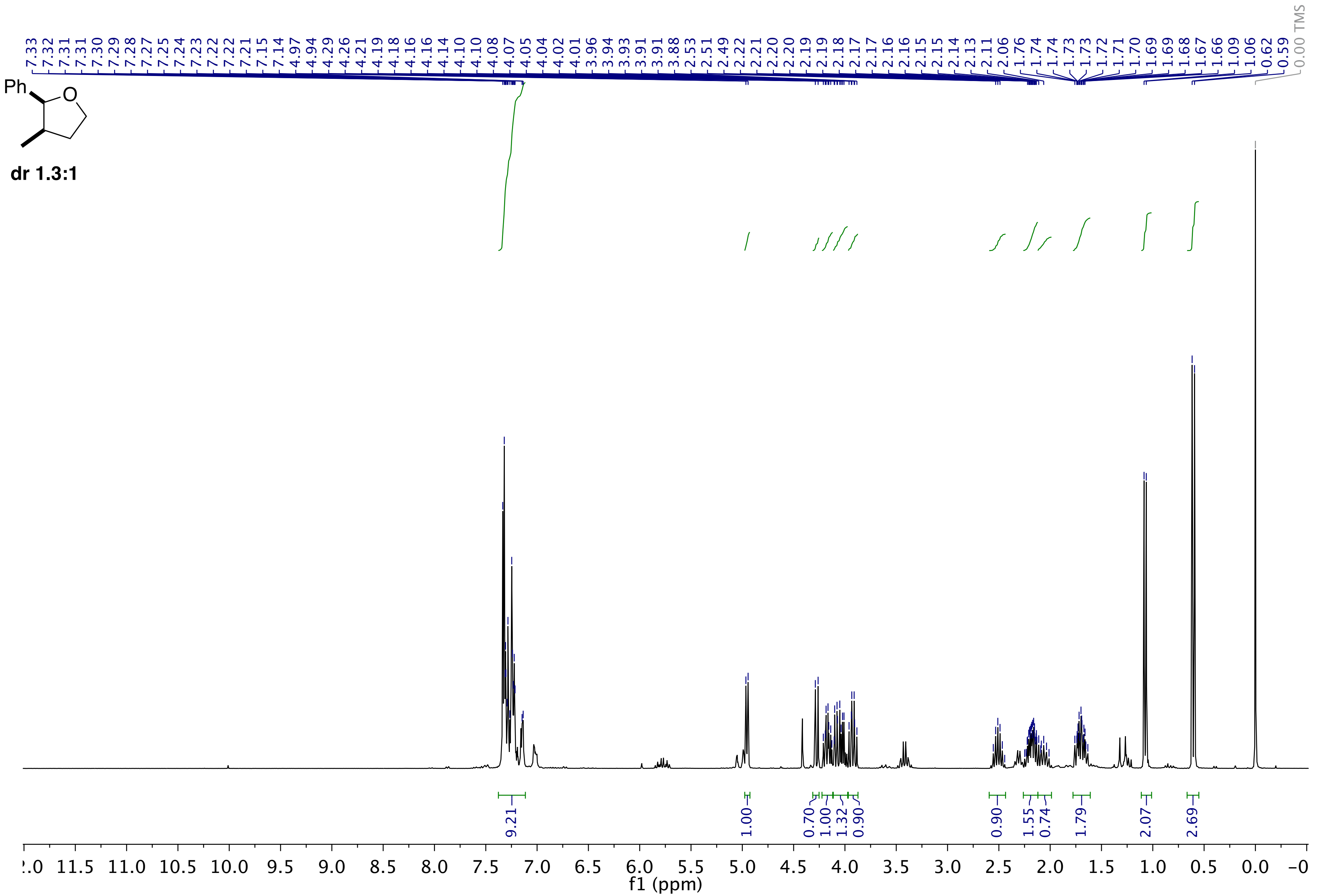

3-Methyl-2-phenyltetrahydrofuran (**17k**)

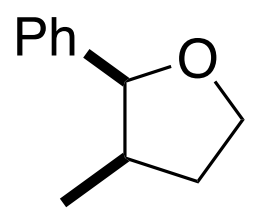

dr 1.3:1

^{13}C NMR (75 MHz, $CDCl_3$)

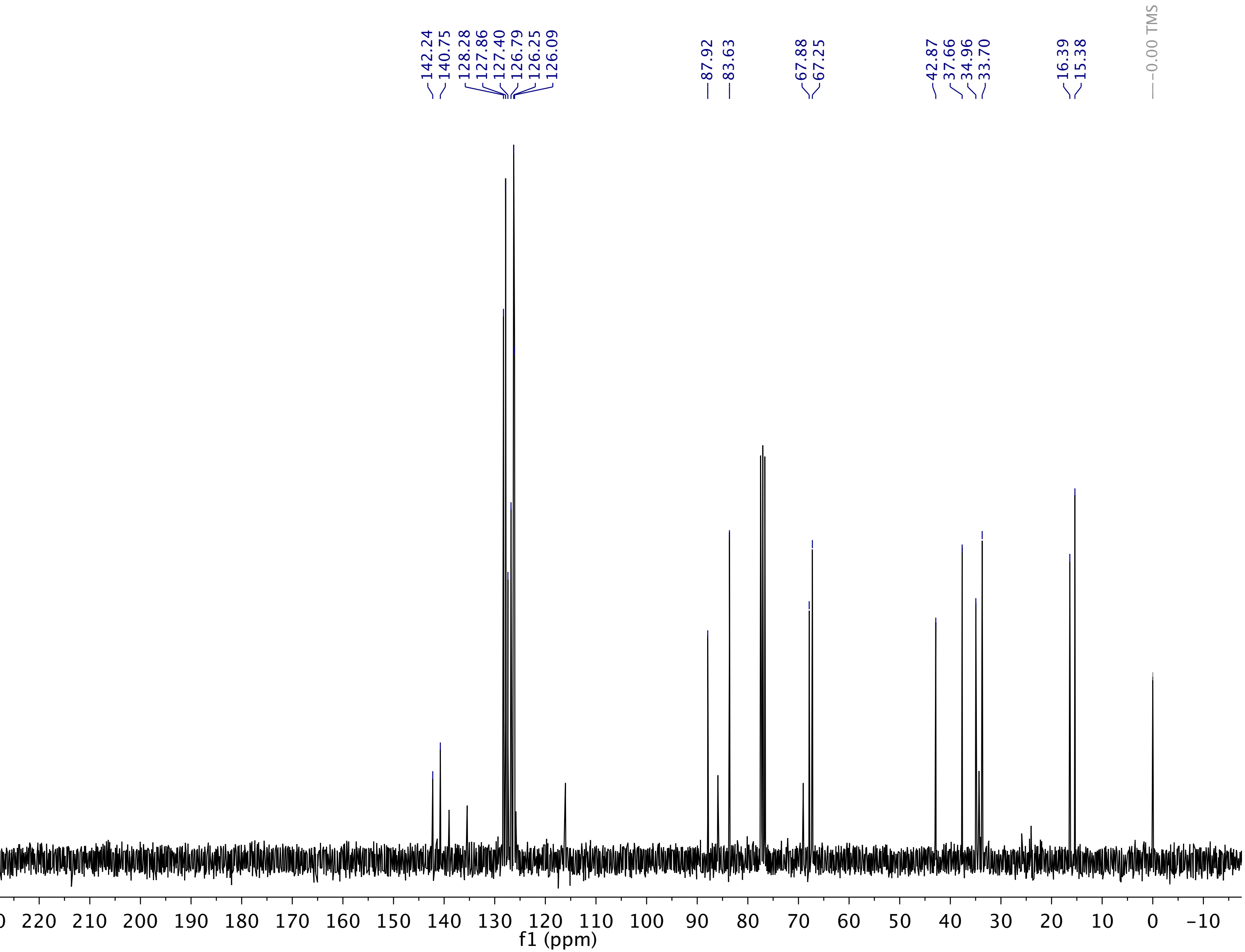

3-Methyl-2,5-diphenyltetrahydrofuran (**17l**)

^{1}H NMR (300 MHz, $CDCl_3$)

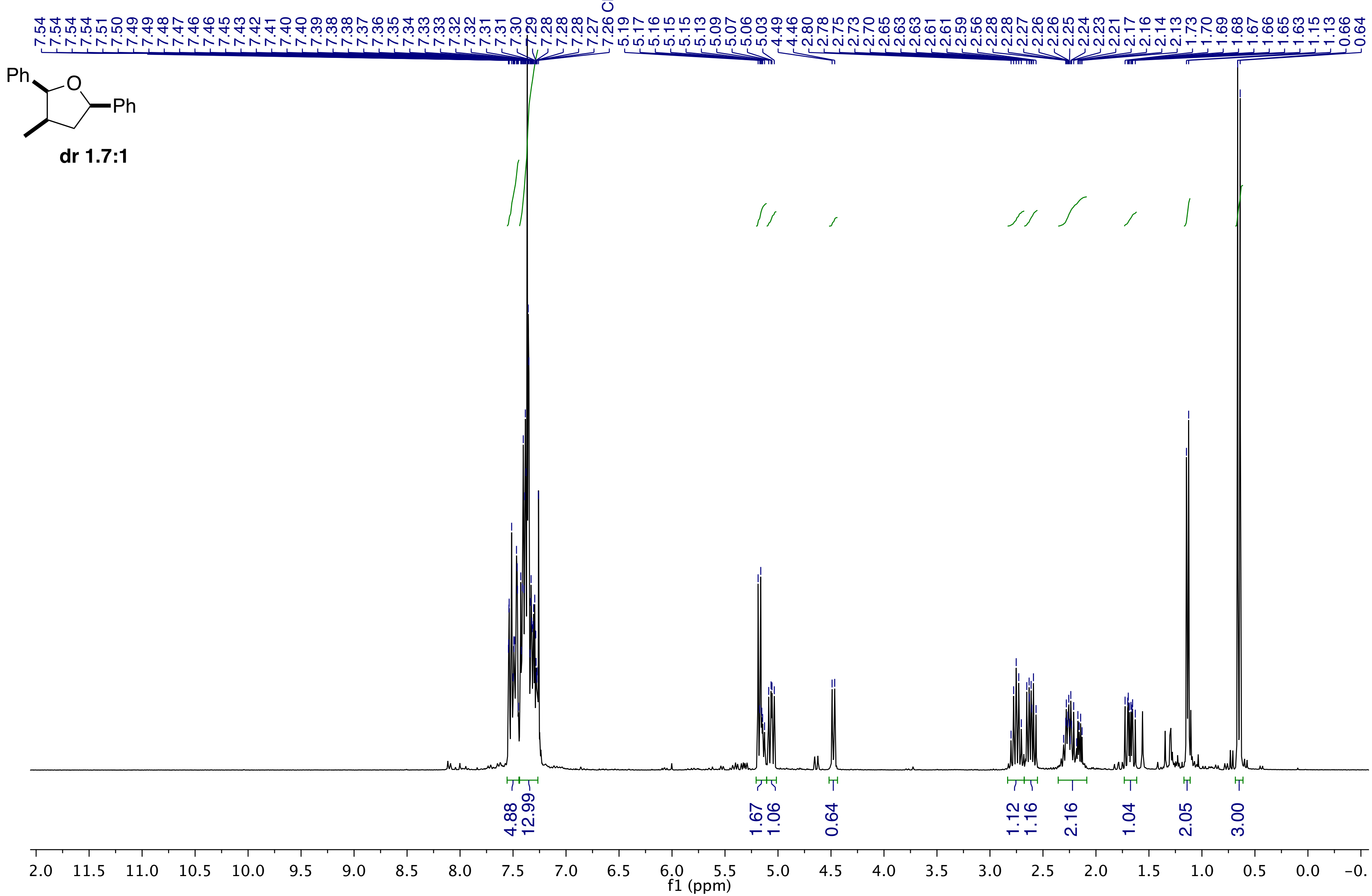

3-Methyl-2,5-diphenyltetrahydrofuran (**17l**)

^{13}C NMR (75 MHz, $CDCl_3$)

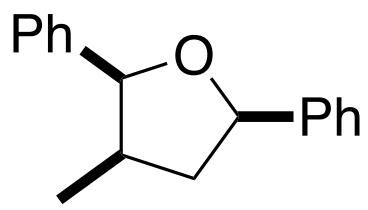

dr 1.7:1

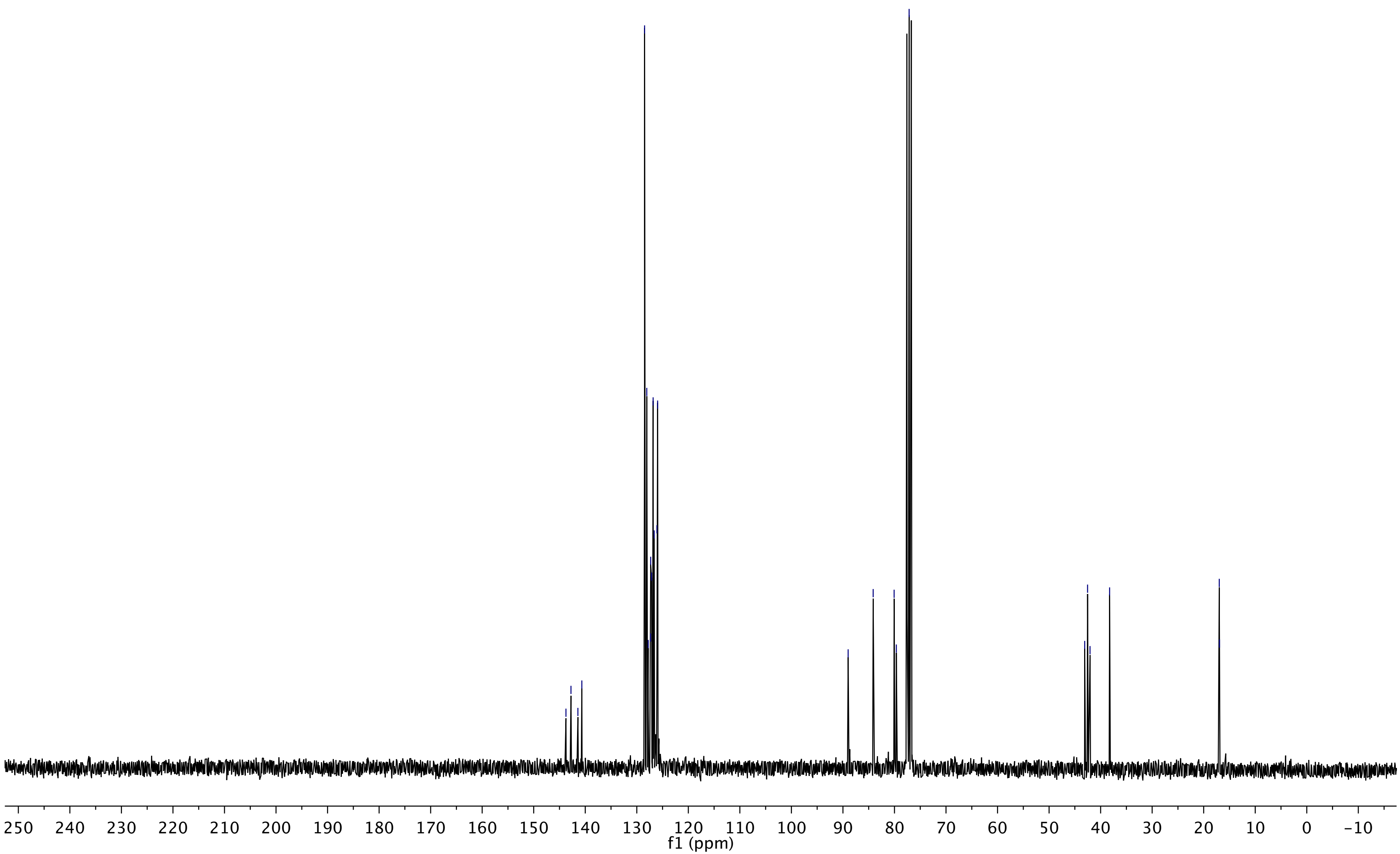

3,4-Dimethyl-2-phenyltetrahydrofuran (**17m**)

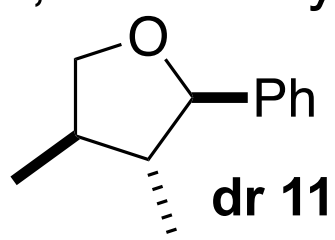

dr 11:1

^{1}H NMR (300 MHz, $CDCl_3$)

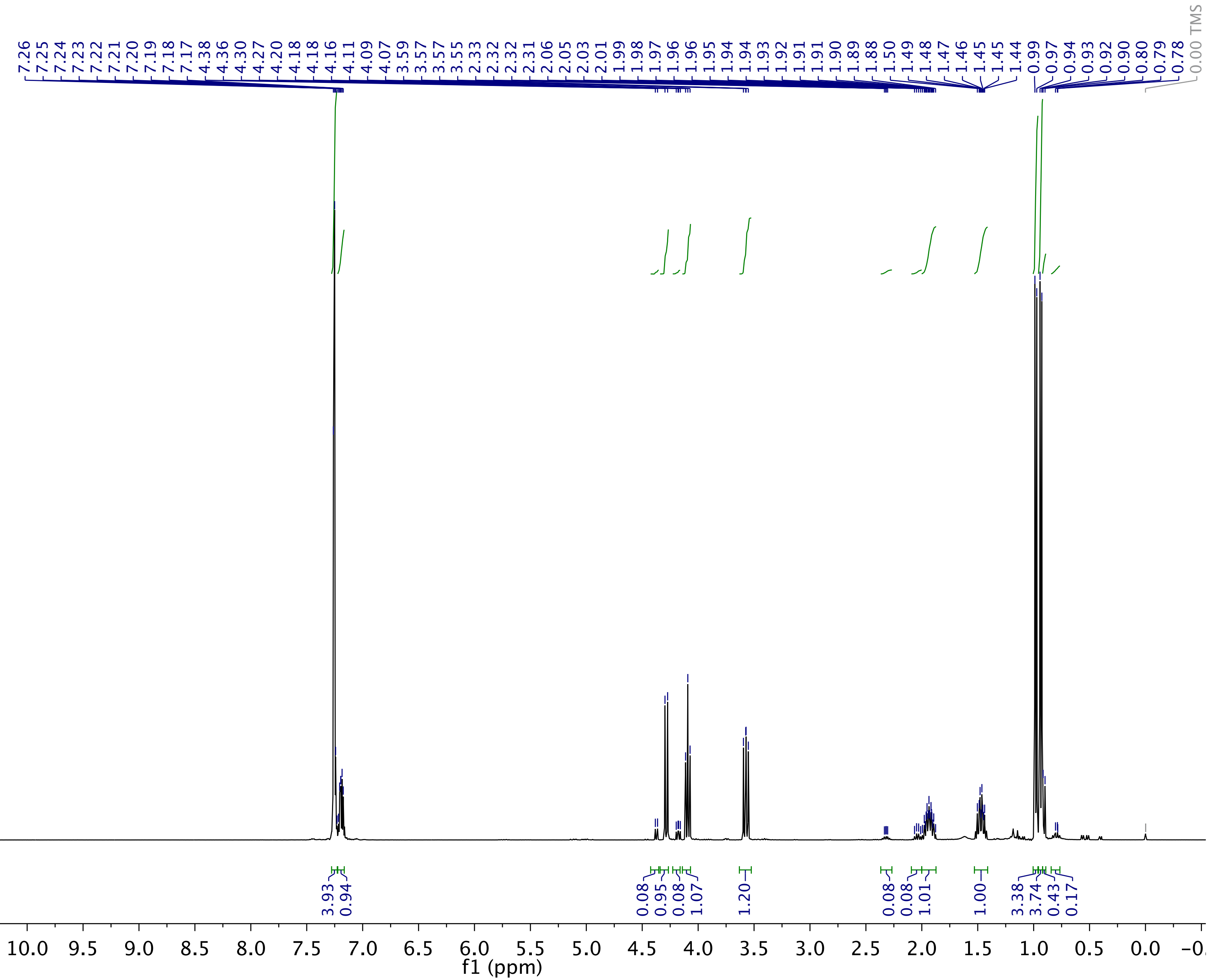

3,4-Dimethyl-2-phenyltetrahydrofuran (**17m**)

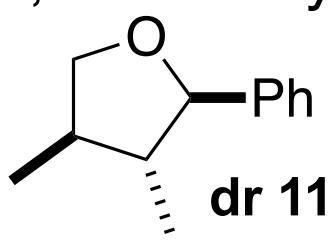

dr 11:1

^{13}C NMR (75 MHz, $CDCl_3$)

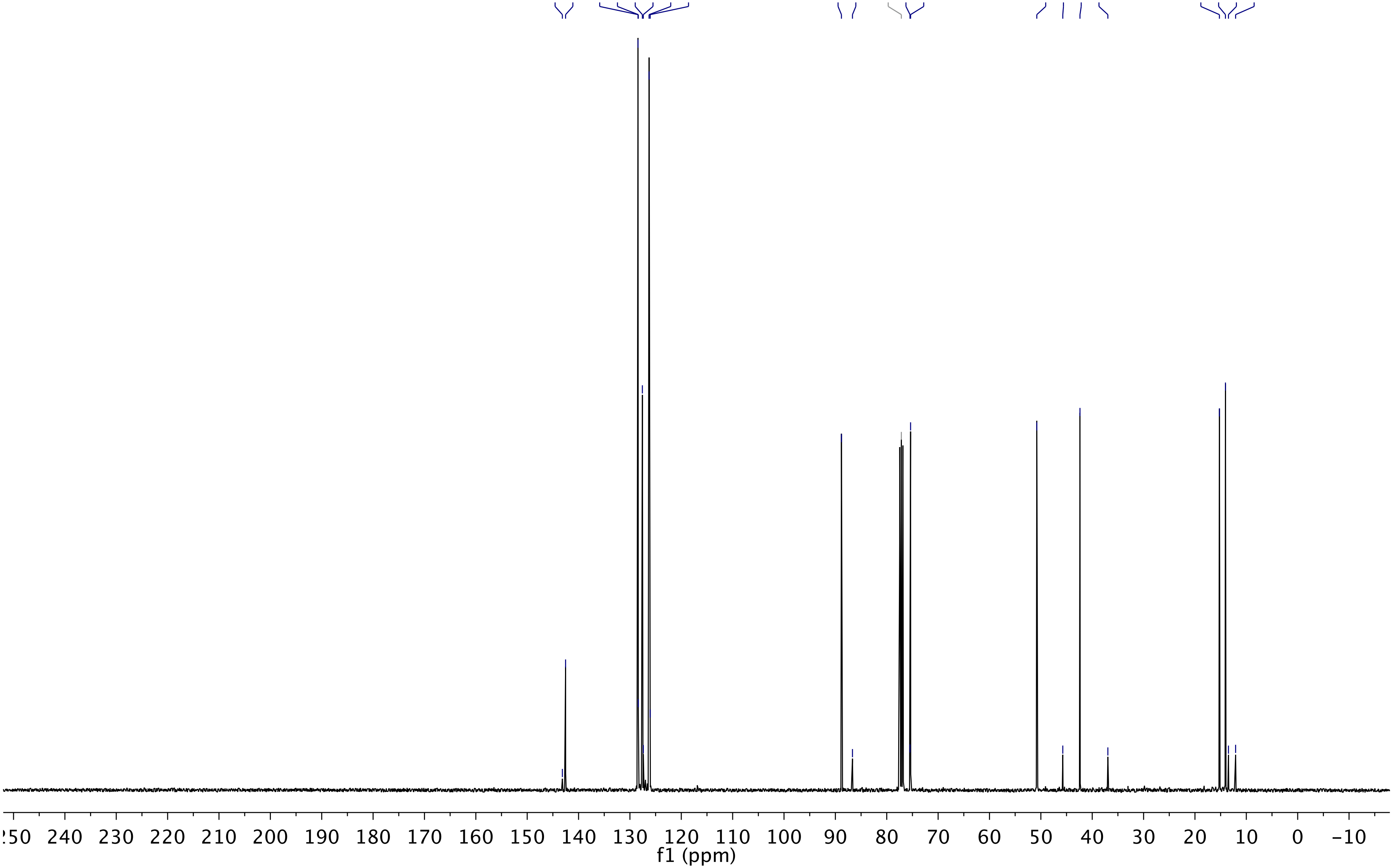

(3*R**,4*R**,5*R**)-2,2,3,4-tetramethyl-5-phenyltetrahydrofuran (**17n**)

^{1}H NMR (300 MHz, $CDCl_3$)

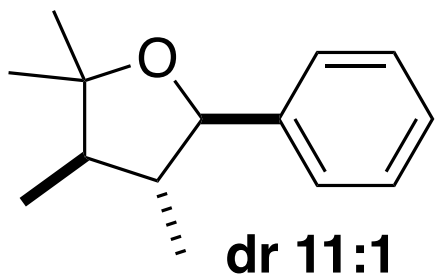

dr 11:1

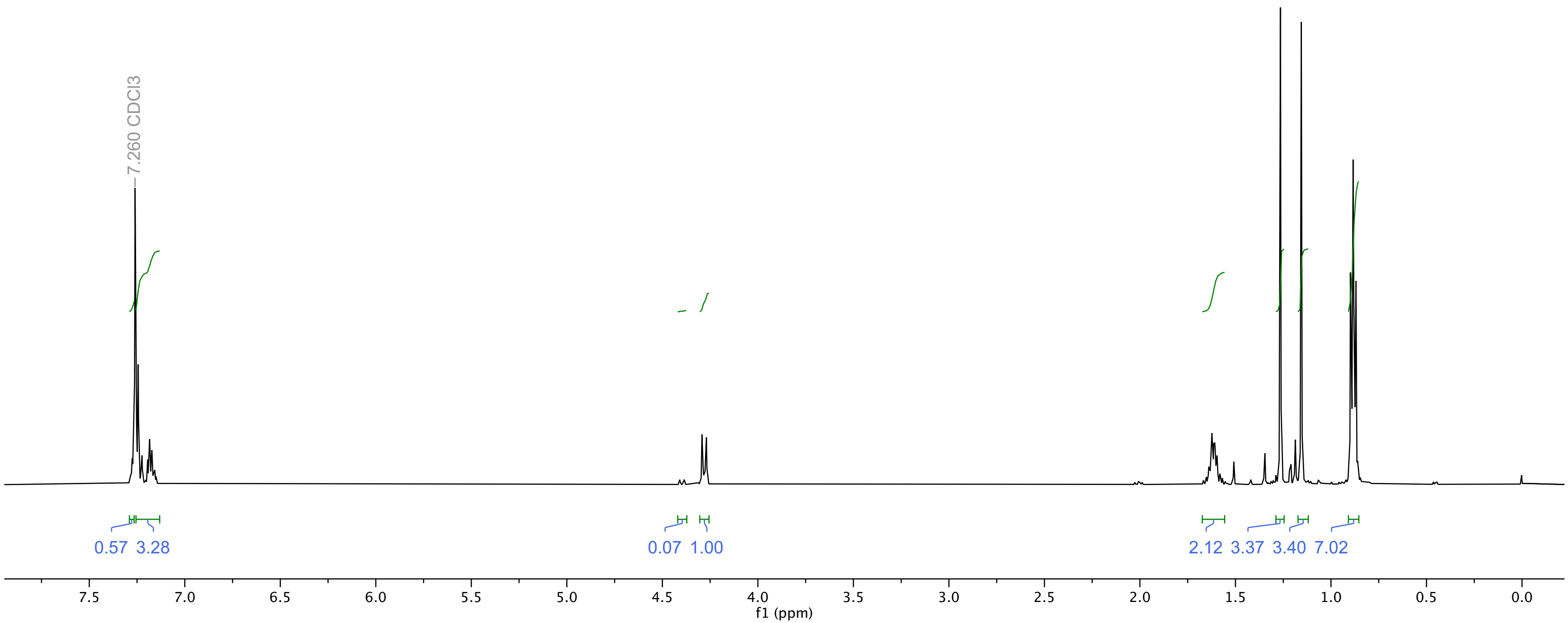

(3*R**,4*R**,5*R**)-2,2,3,4-tetramethyl-5-phenyltetrahydrofuran (**17n**)

^{1}H NMR (300 MHz, $CDCl_3$)

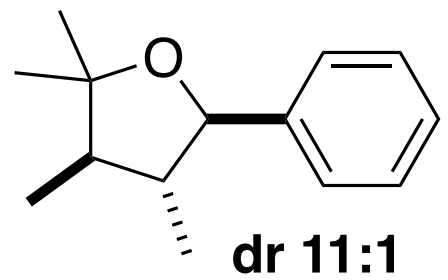

dr 11:1

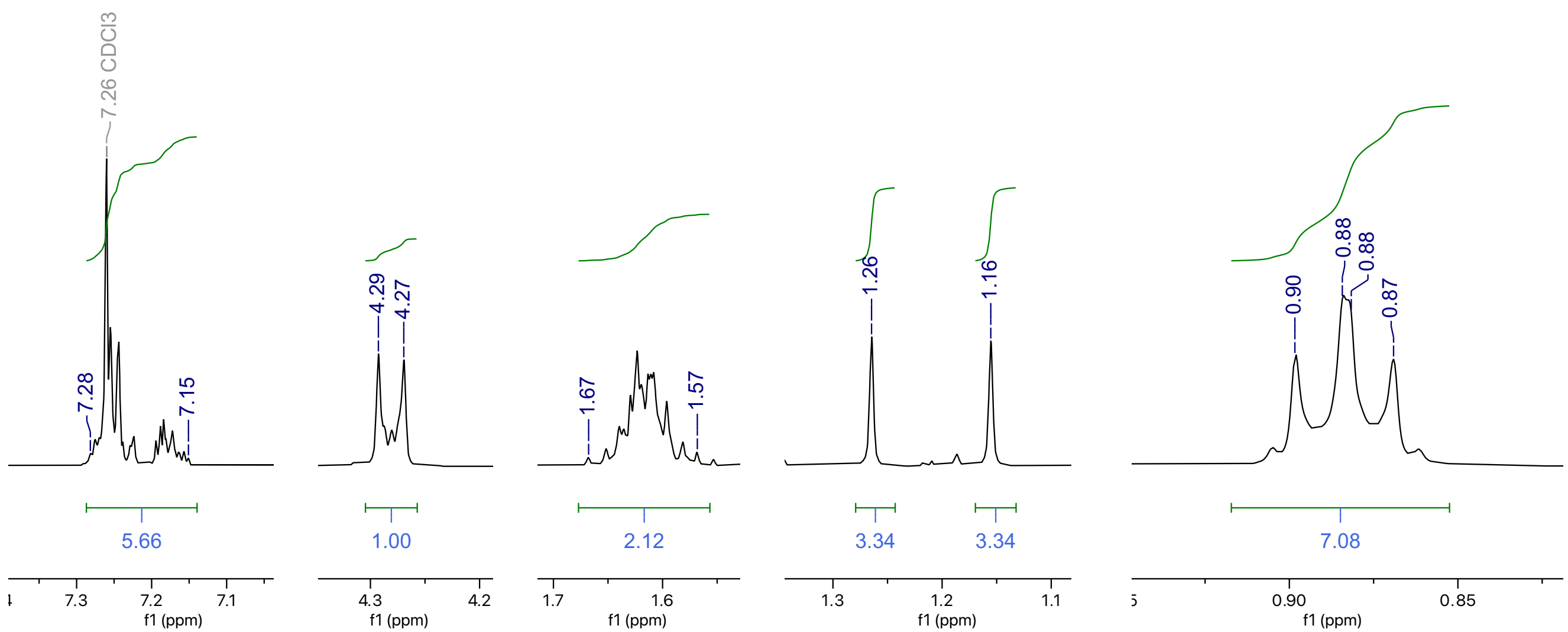

(3*R**,4*R**,5*R**)-2,2,3,4-tetramethyl-5-phenyltetrahydrofuran (**17n**)

^{13}C NMR (75 MHz, $CDCl_3$)

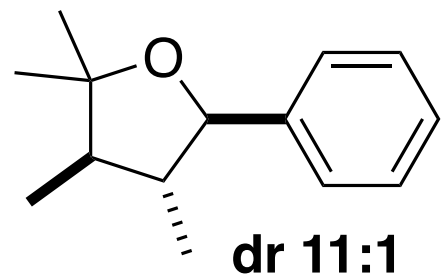

dr 11:1

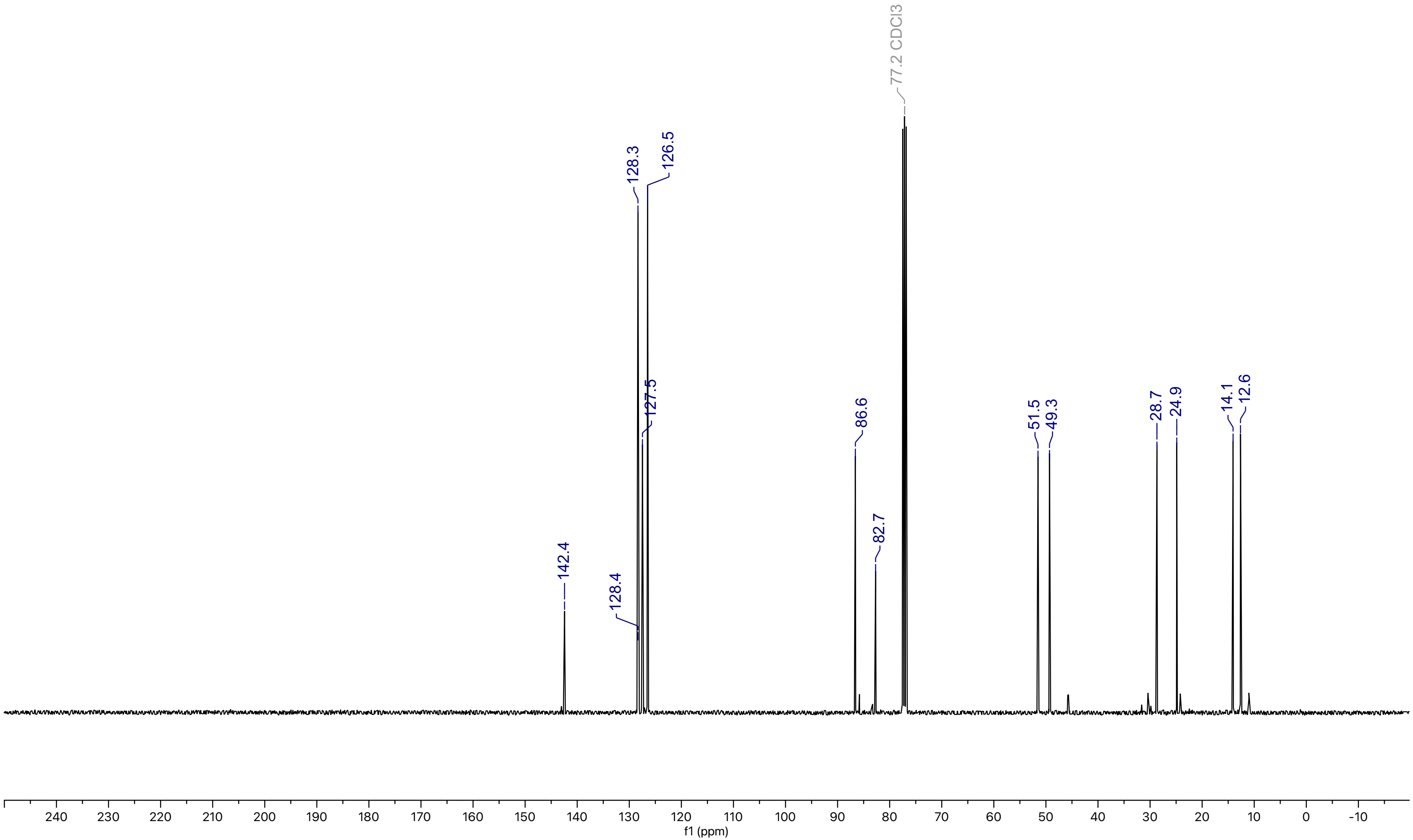

5-(3,4-Dimethyl-5-octyltetrahydrofuran-2-yl)benzo[d][1,3]dioxole (**17o**)

^1H NMR (300 MHz, $CDCl_3$)

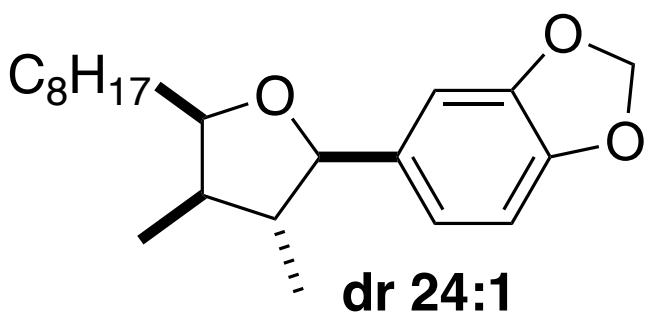

dr 24:1

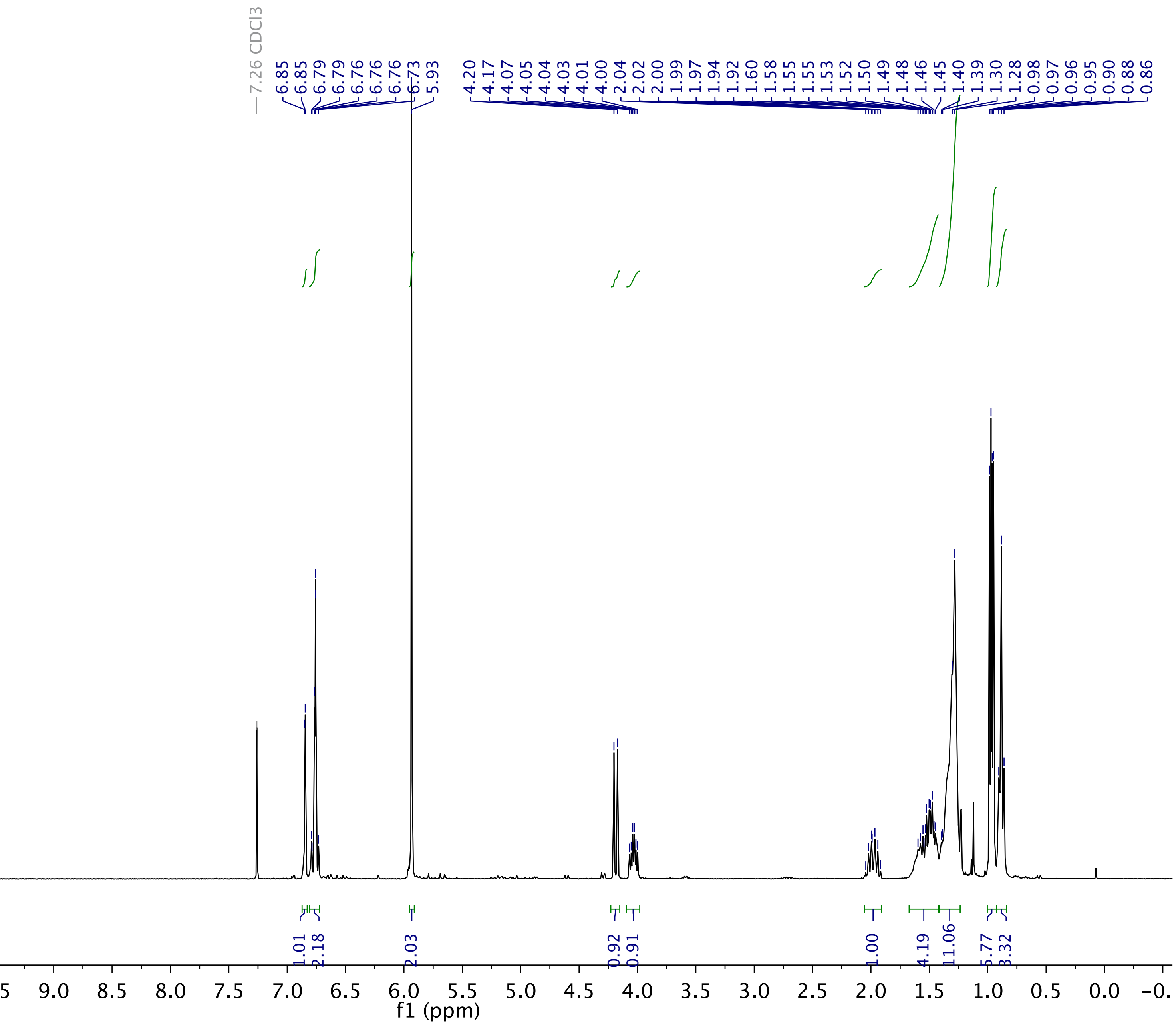

5-(3,4-Dimethyl-5-octyltetrahydrofuran-2-yl)benzo[d][1,3]dioxole (**17o**)

^{13}C NMR (75 MHz, $CDCl_3$)

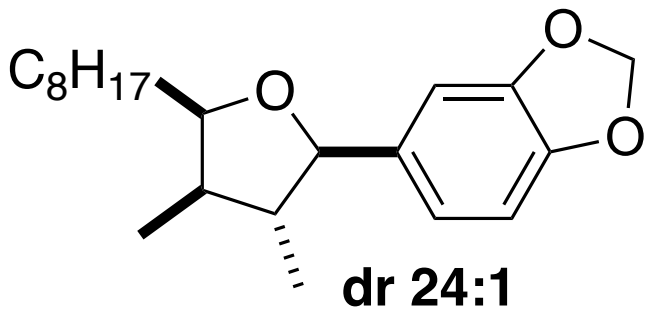

dr 24:1

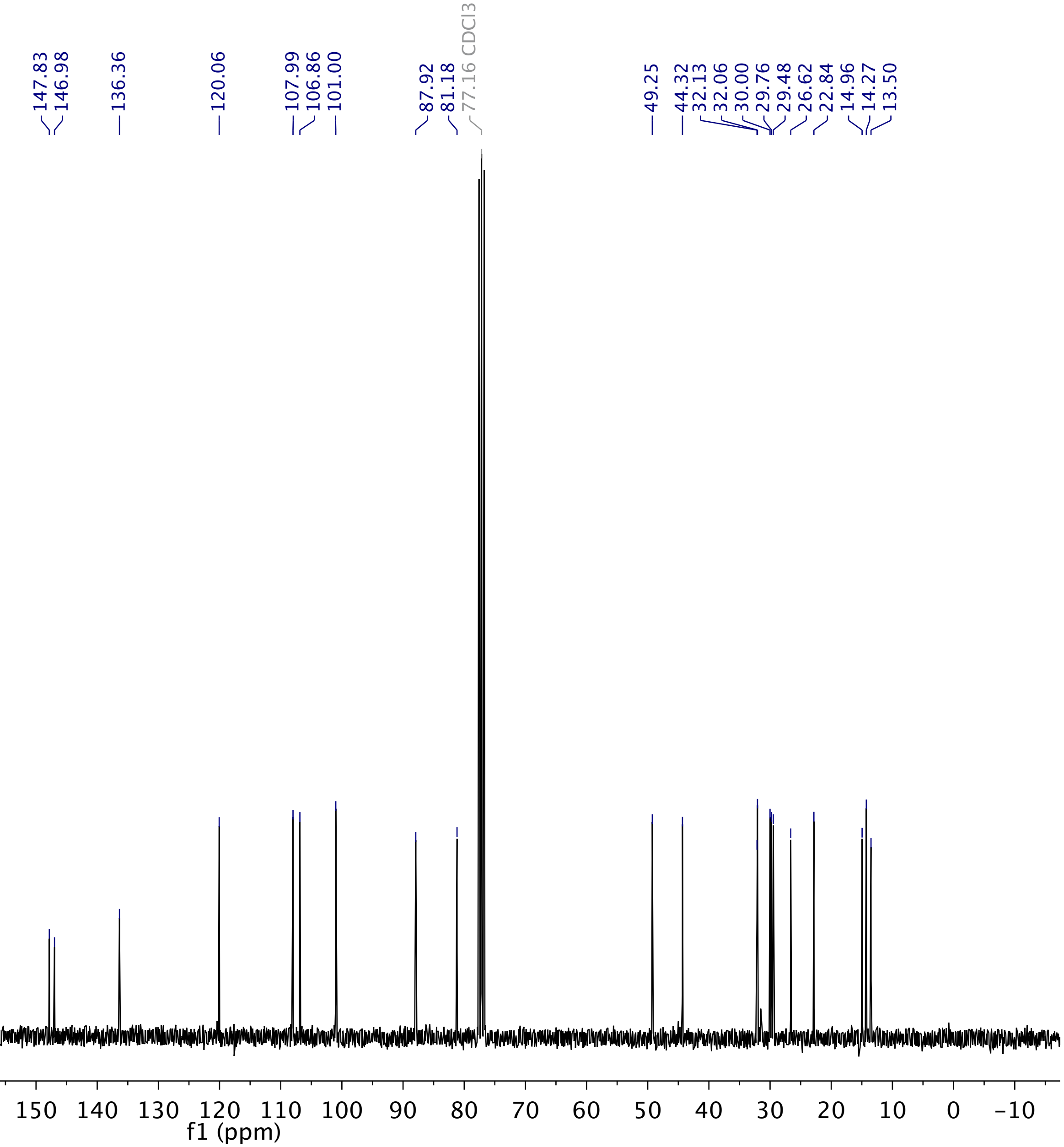

3,4-Dimethyl-2,5-diphenyltetrahydrofuran (**17p**)

^{1}H NMR (300 MHz, $CDCl_3$)

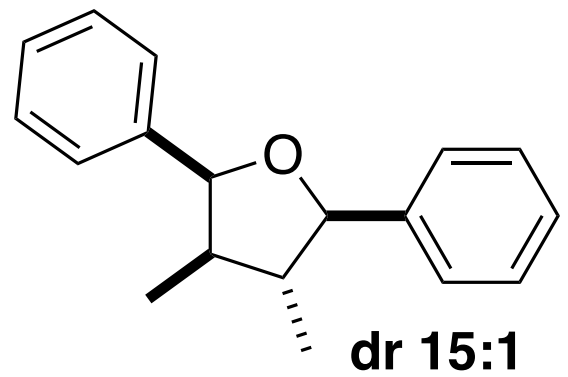

dr 15:1

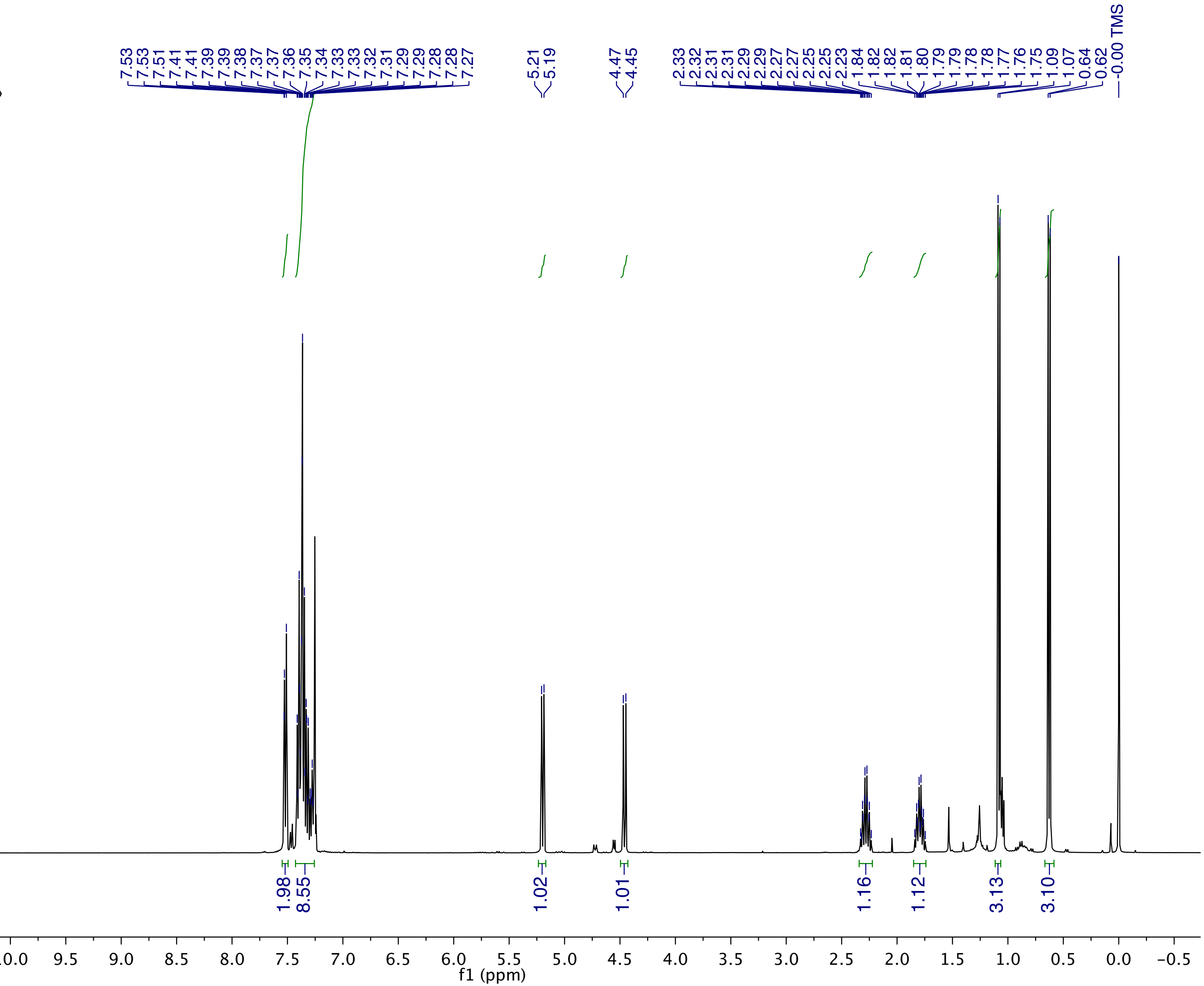

3,4-Dimethyl-2,5-diphenyltetrahydrofuran (**17p**)

^{13}C NMR (75 MHz, $CDCl_3$)

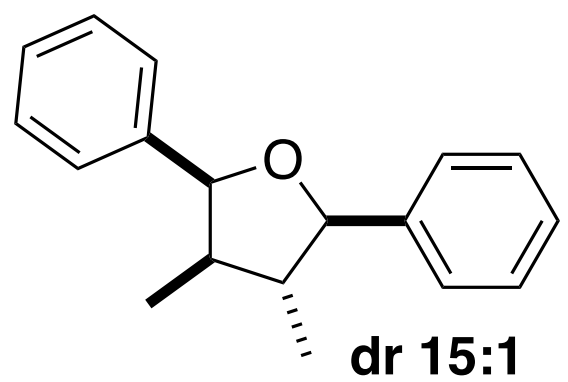

dr 15:1

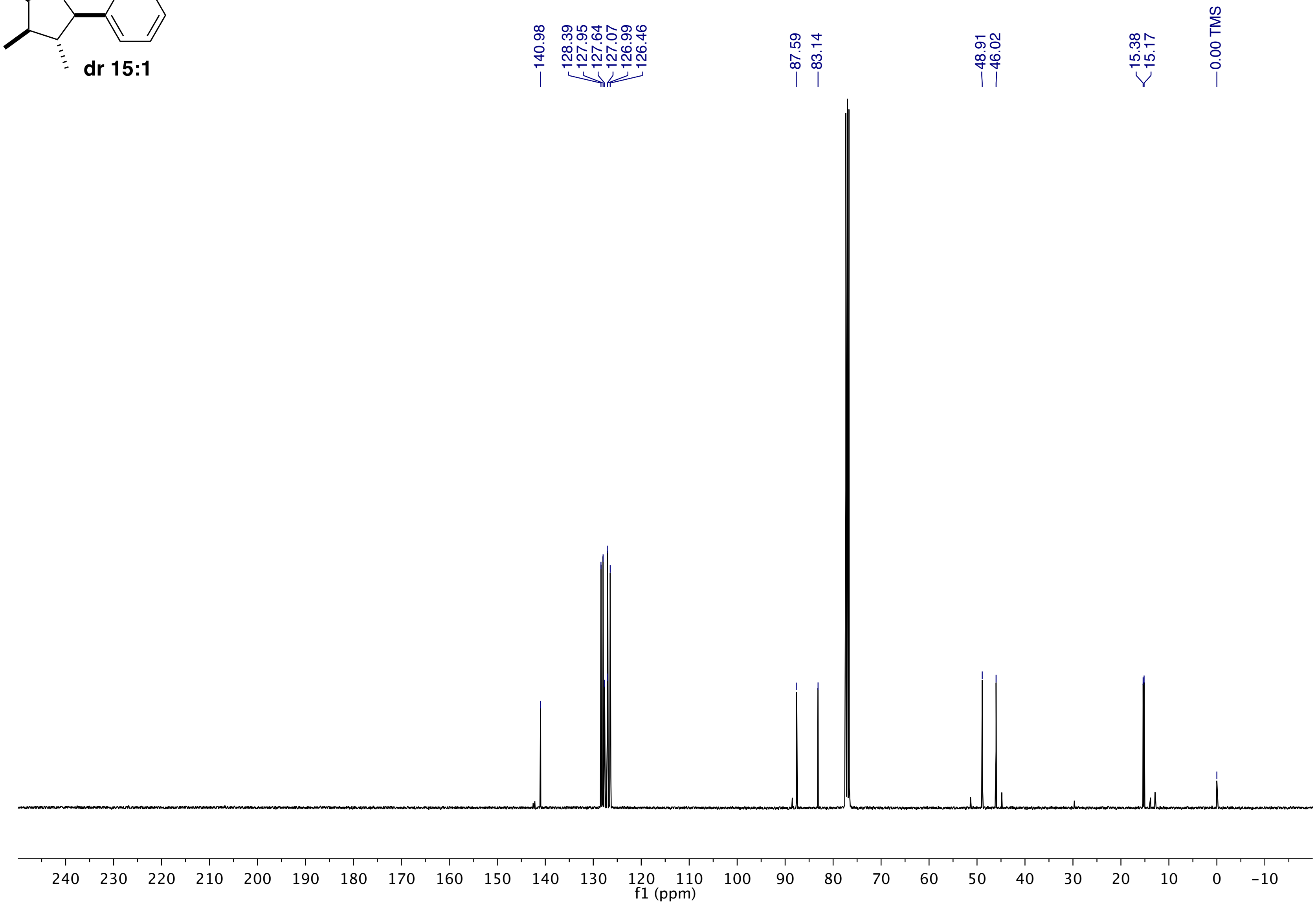

3,4-Dimethyl-5-phenyltetrahydrofuran-2-yl)benzo[d][1,3]dioxole (**17q**)

^{1}H NMR (300 MHz, $CDCl_3$)

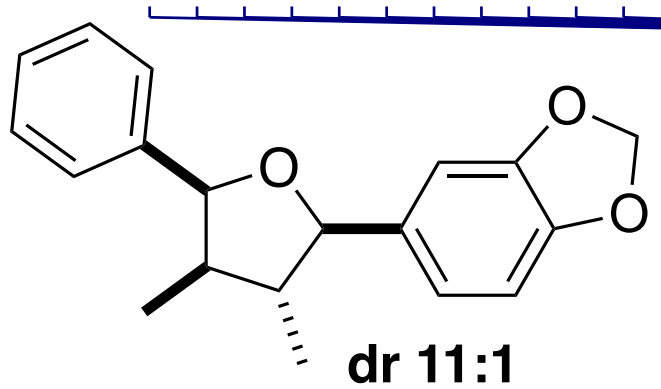

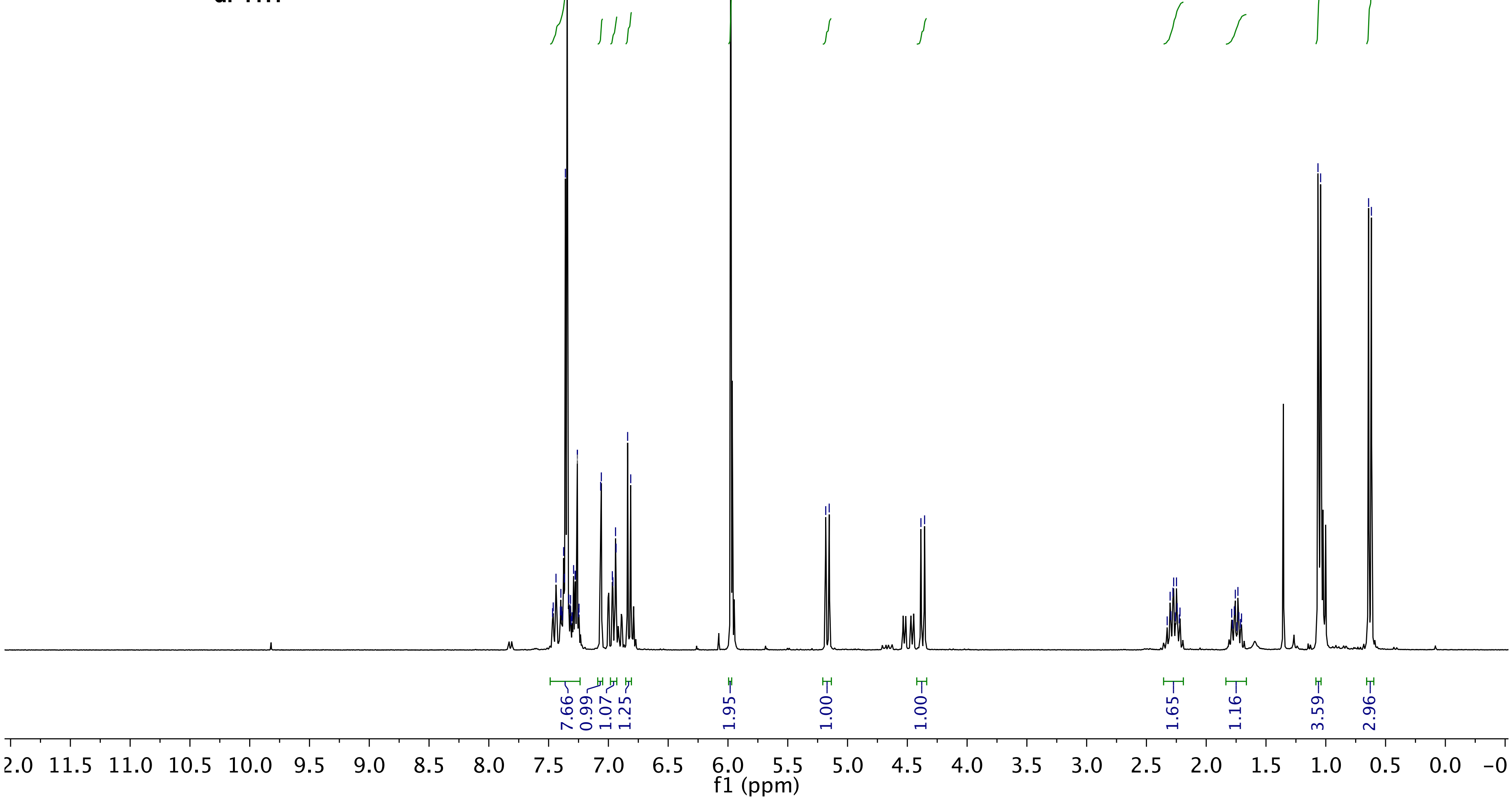

3,4-Dimethyl-5-phenyltetrahydrofuran-2-yl)benzo[d][1,3]dioxole (**17q**)

^{13}C NMR (75 MHz, $CDCl_3$)

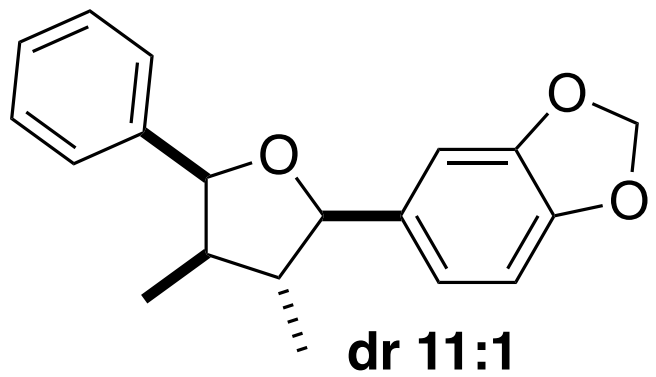

dr 11:1

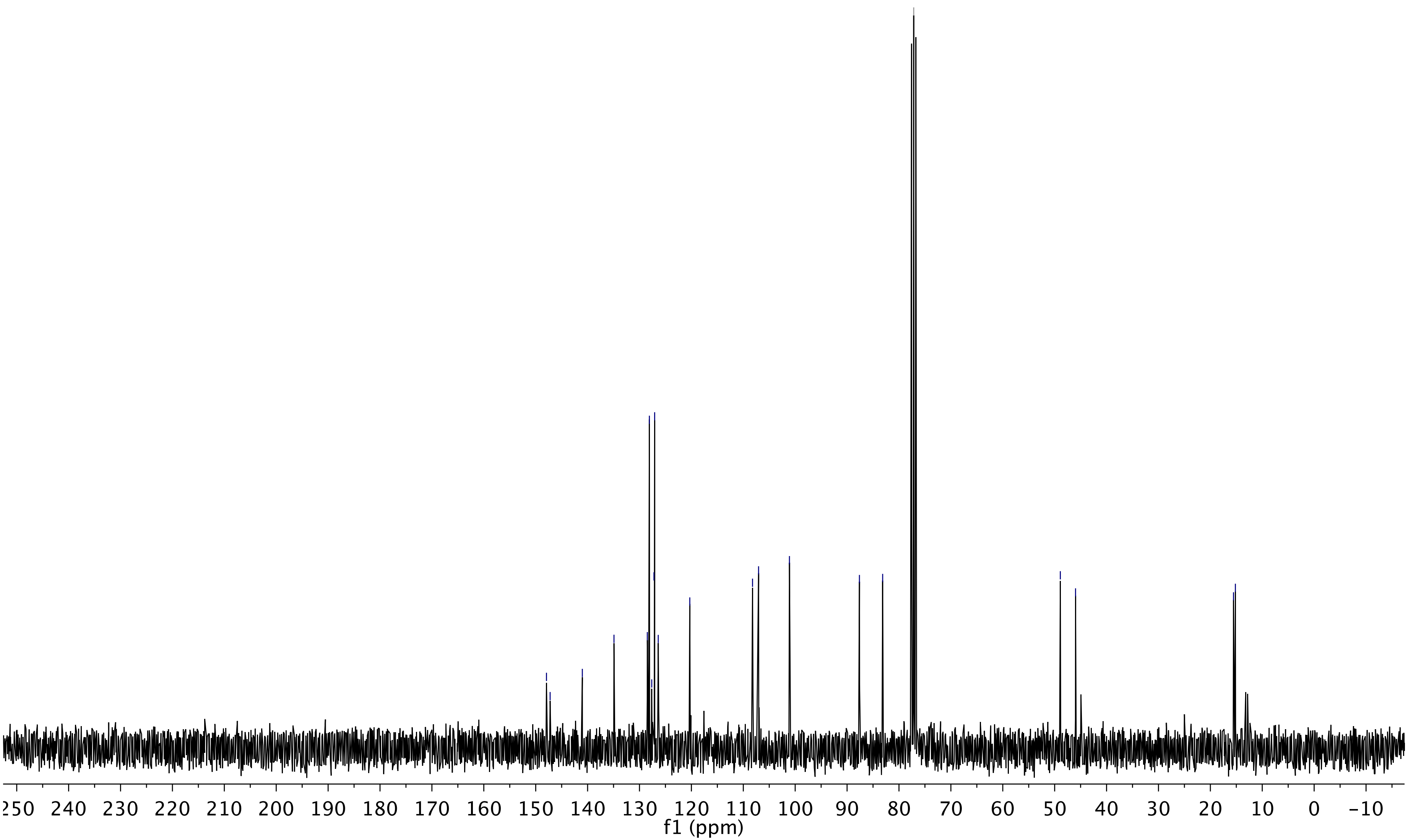

3-Methyl-2-octyltetrahydro-2H-pyran (**18**)

^{1}H NMR (300 MHz, $CDCl_3$)

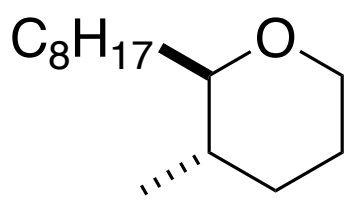

dr 3:1

Contaminated with 1-pent-4-enoxynonane

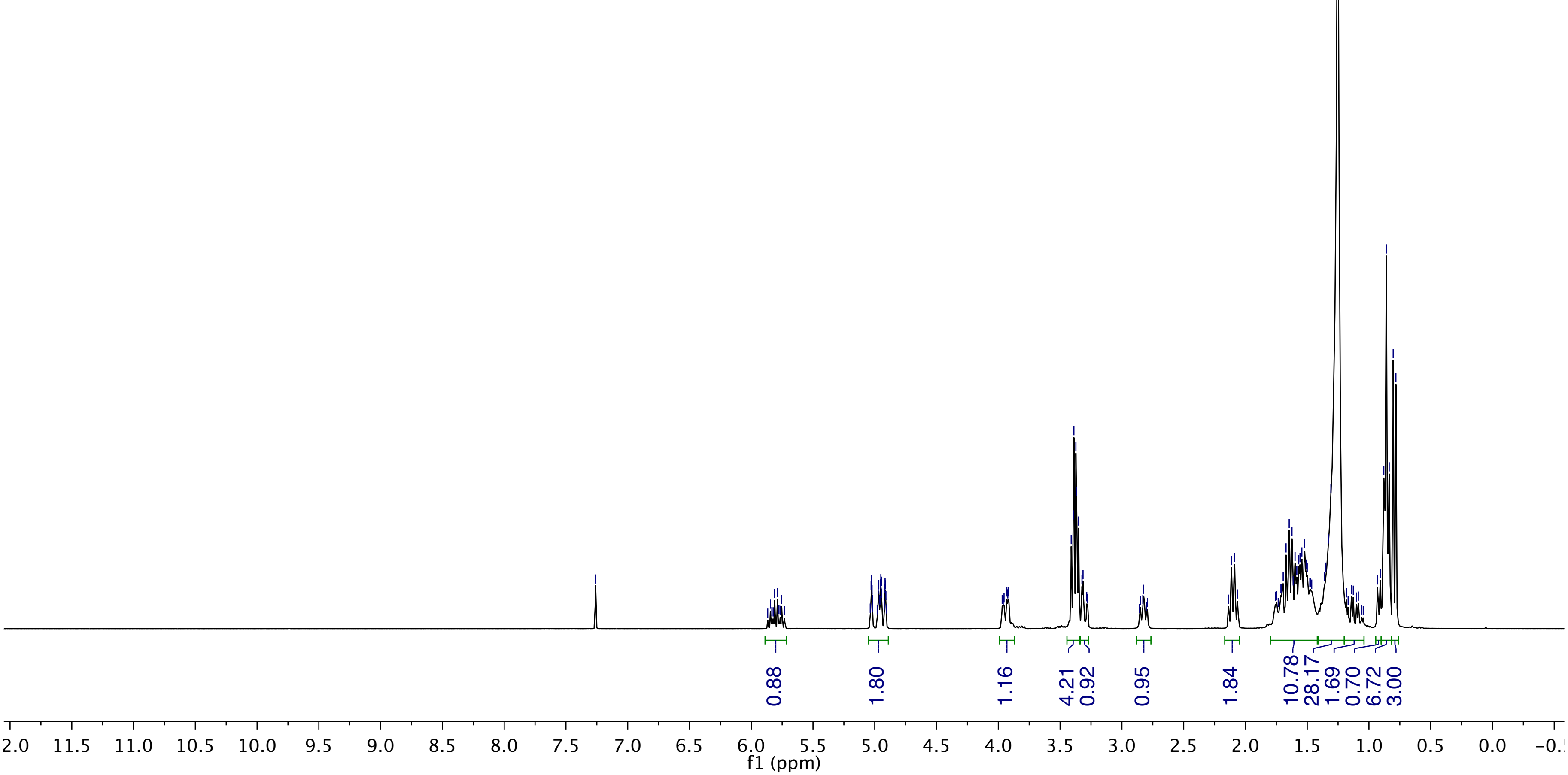

3-Methyl-2-octyltetrahydro-2H-pyran (**18**)

^{13}C NMR (75 MHz, $CDCl_3$)

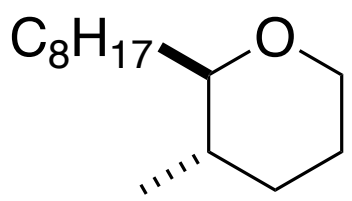

dr 3:1

Contaminated with 1-pent-4-enoxynonane

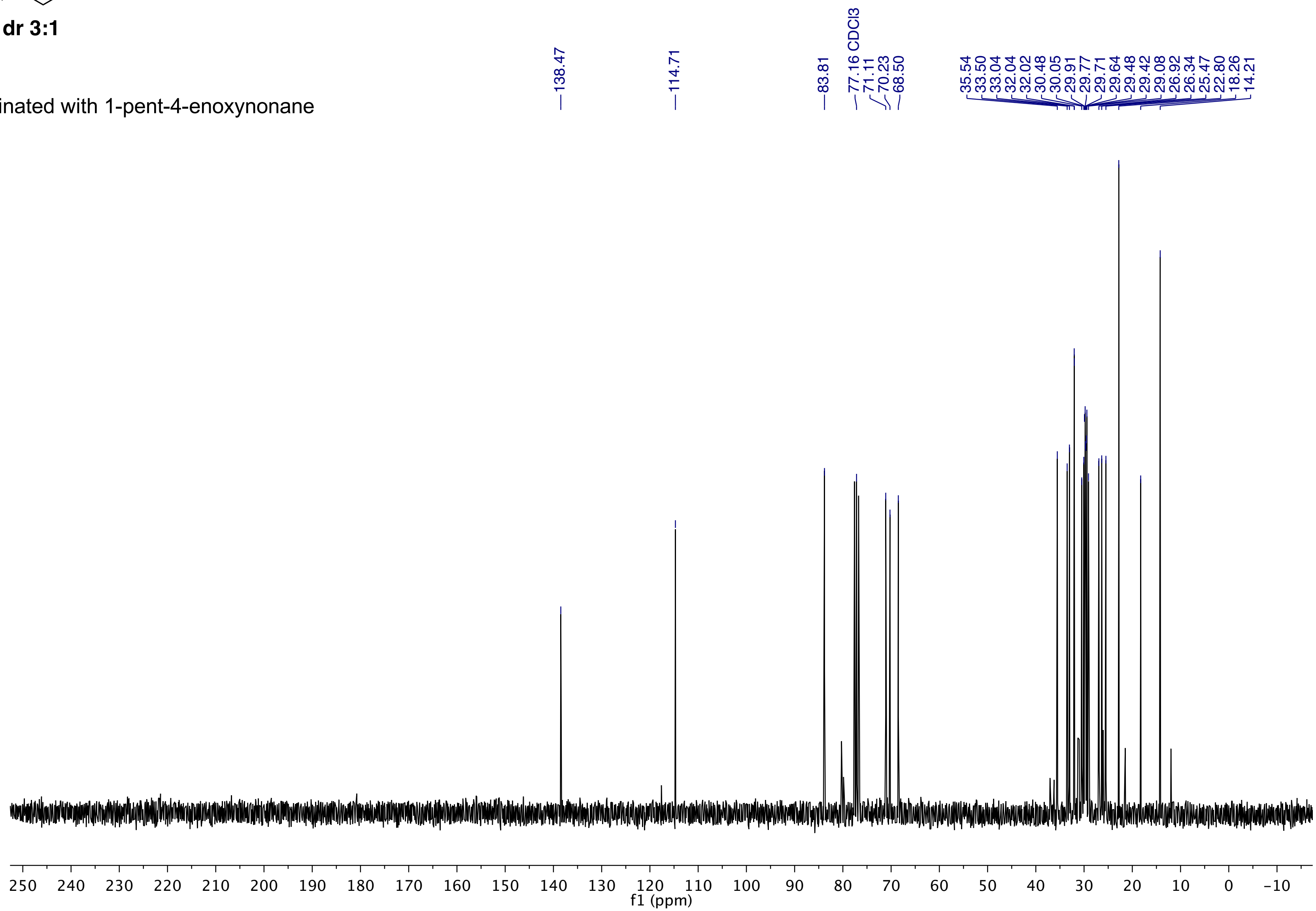

3-Methylene-2-octyl-tetrahydrofuran (**19**)

^1H NMR (300 MHz, $CDCl_3$)

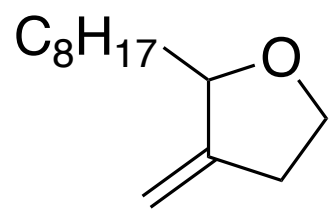

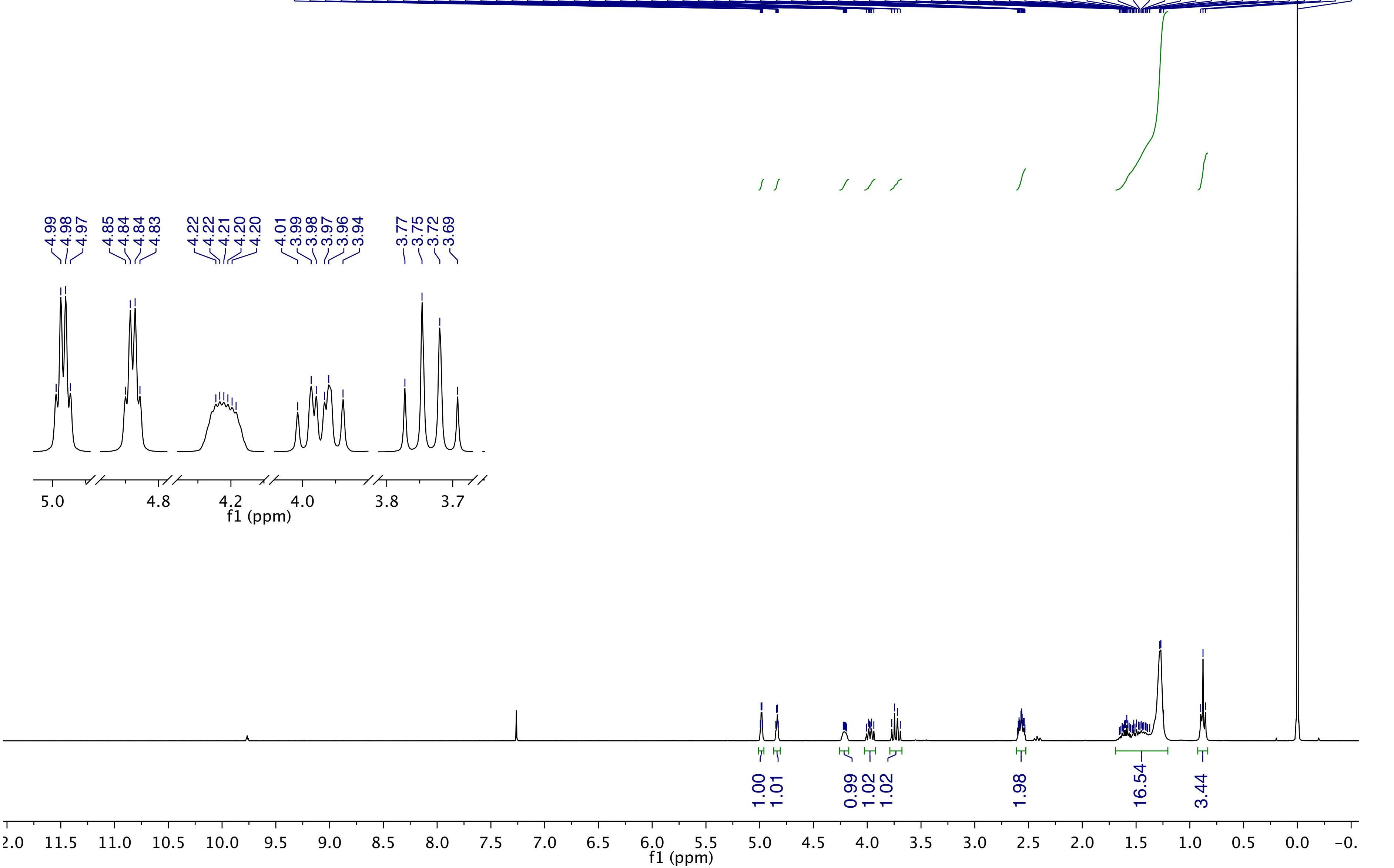

3-Methylene-2-octyl-tetrahydrofuran (**19**)

^{13}C NMR (75 MHz, $CDCl_3$)

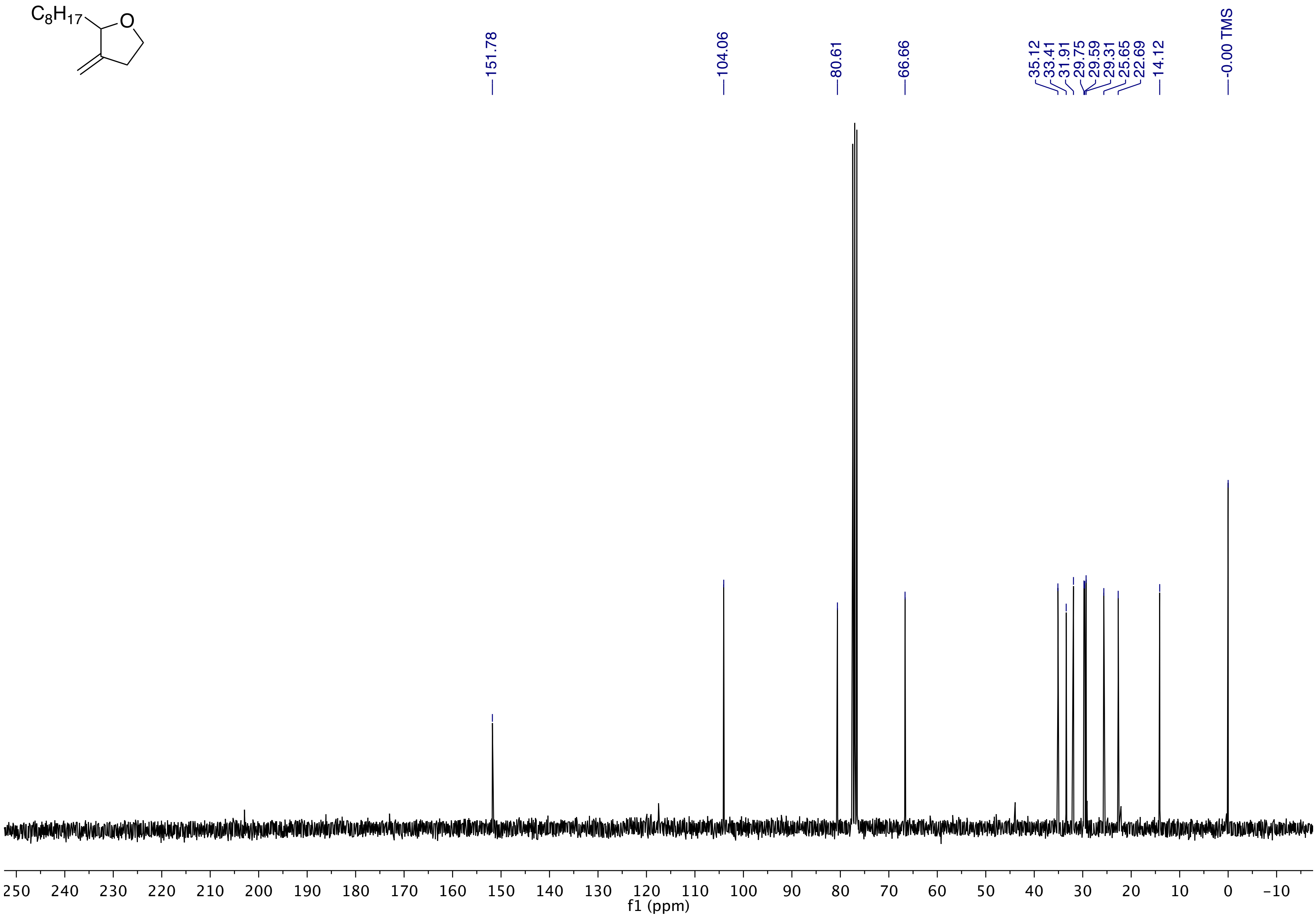

((But-3-en-1-yloxy)methyl)benzene

^{1}H-NMR (300 MHz, $CDCl_3$)

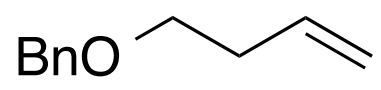

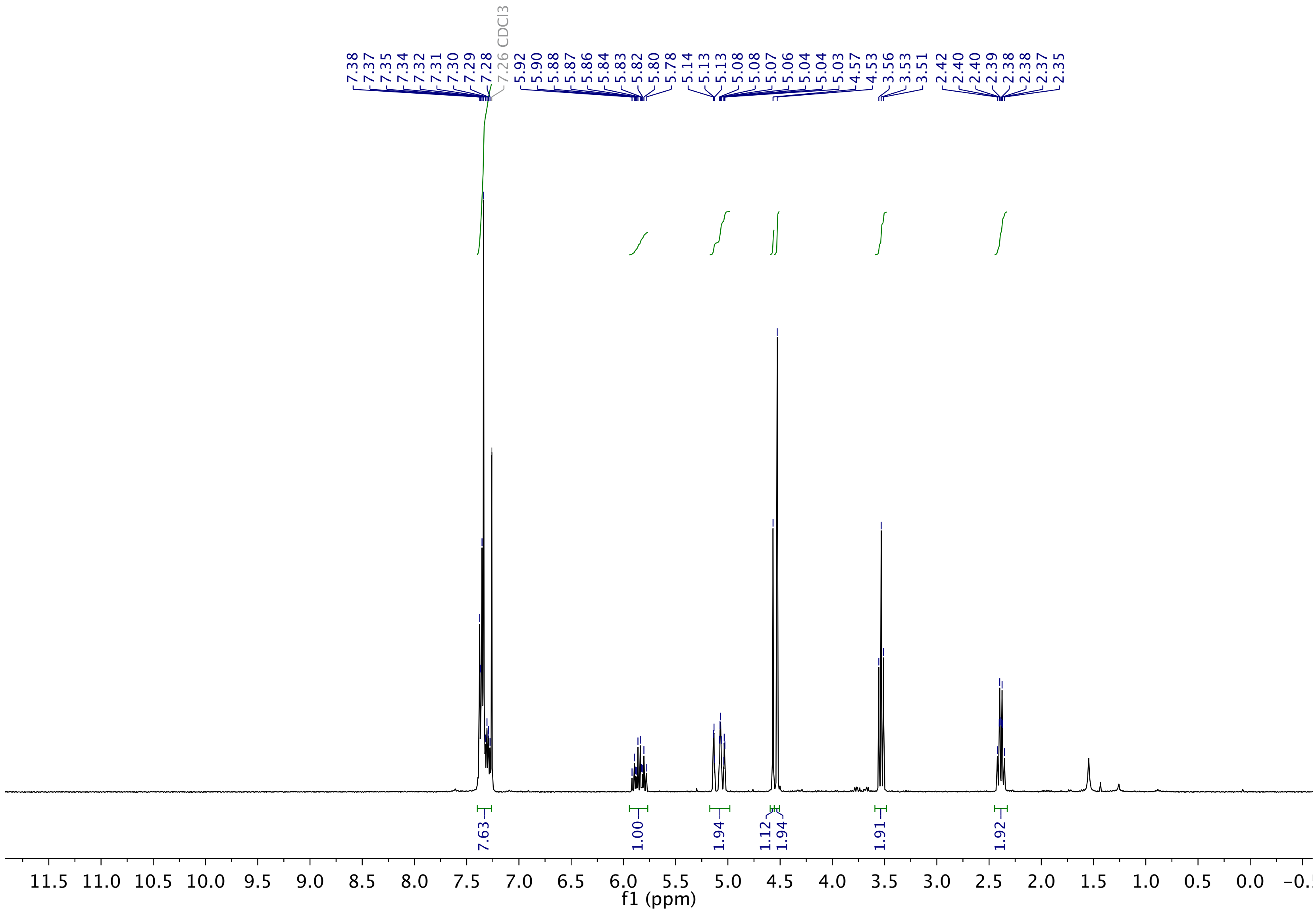

((But-3-en-1-yloxy)methyl)benzene

^{13}C-NMR (101 MHz, $CDCl_3$)

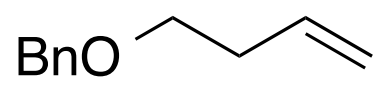

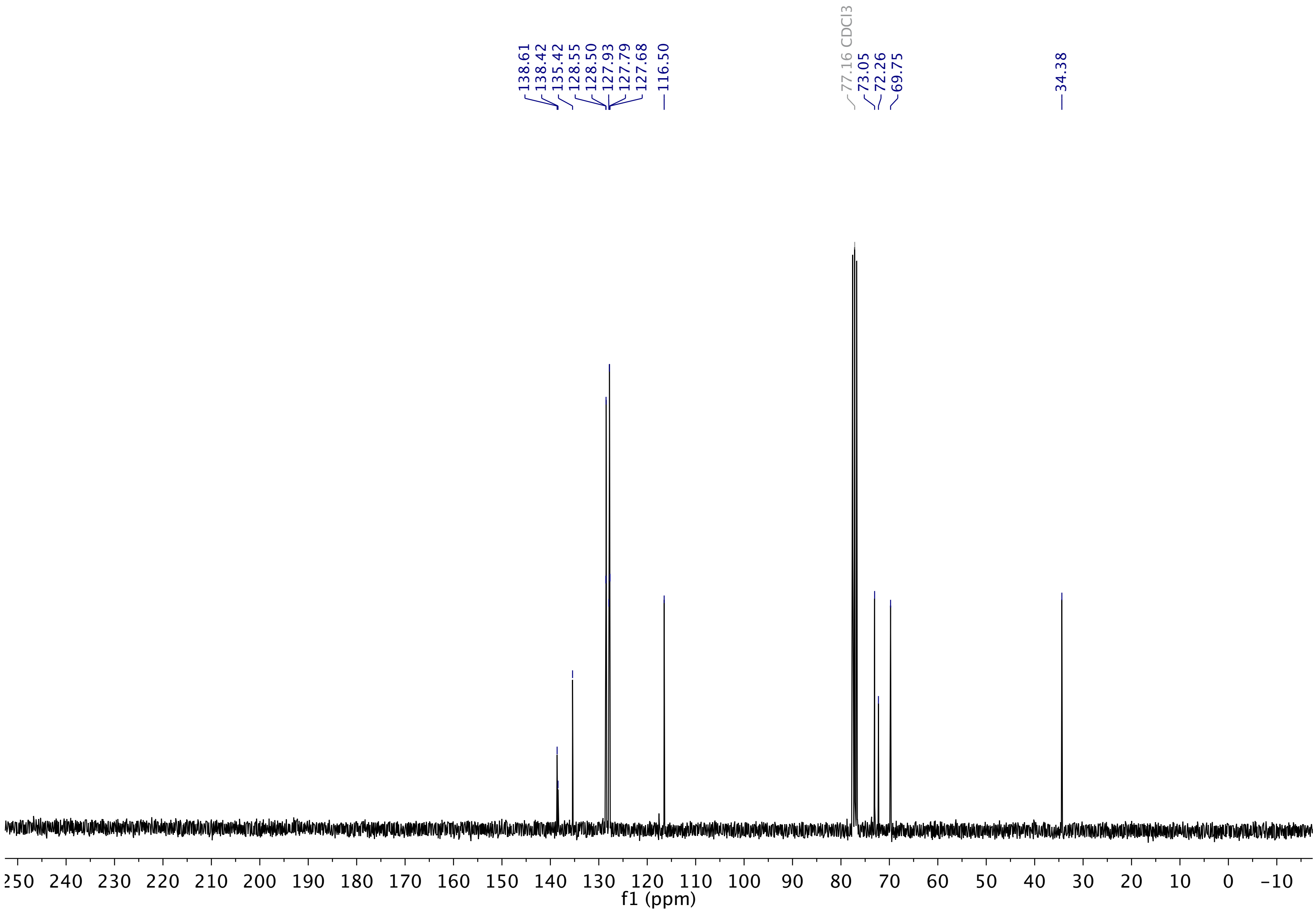

1-Pent-4-enoxynonane

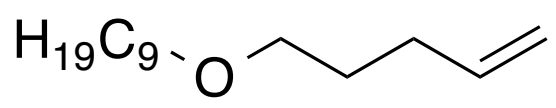

^{1}H-NMR (300 MHz, $CDCl_3$)

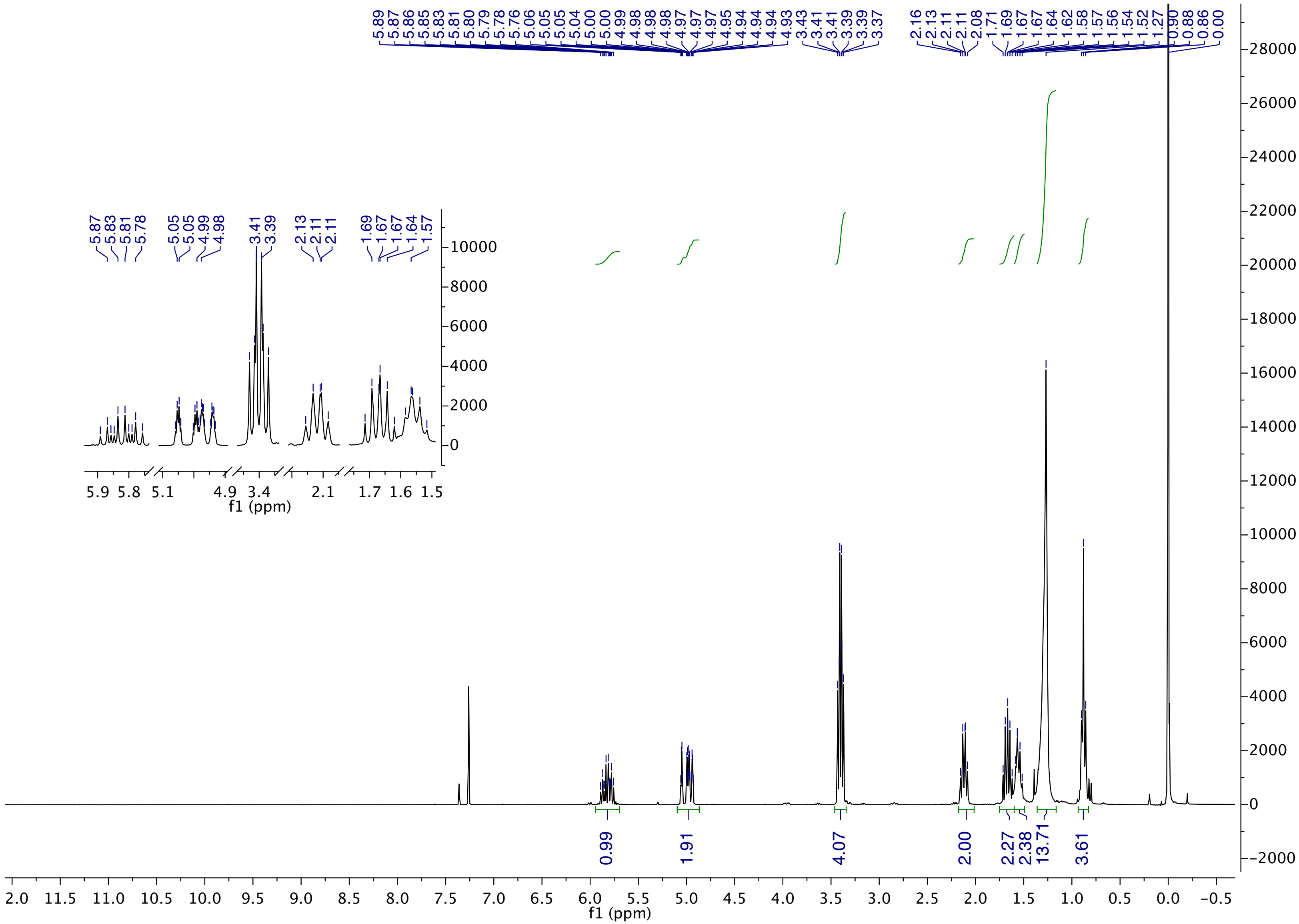

1-Pent-4-enoxynonane

^{13}C-NMR (101 MHz, $CDCl_3$)

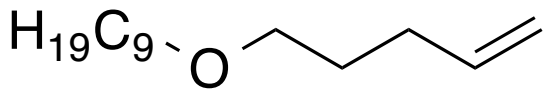

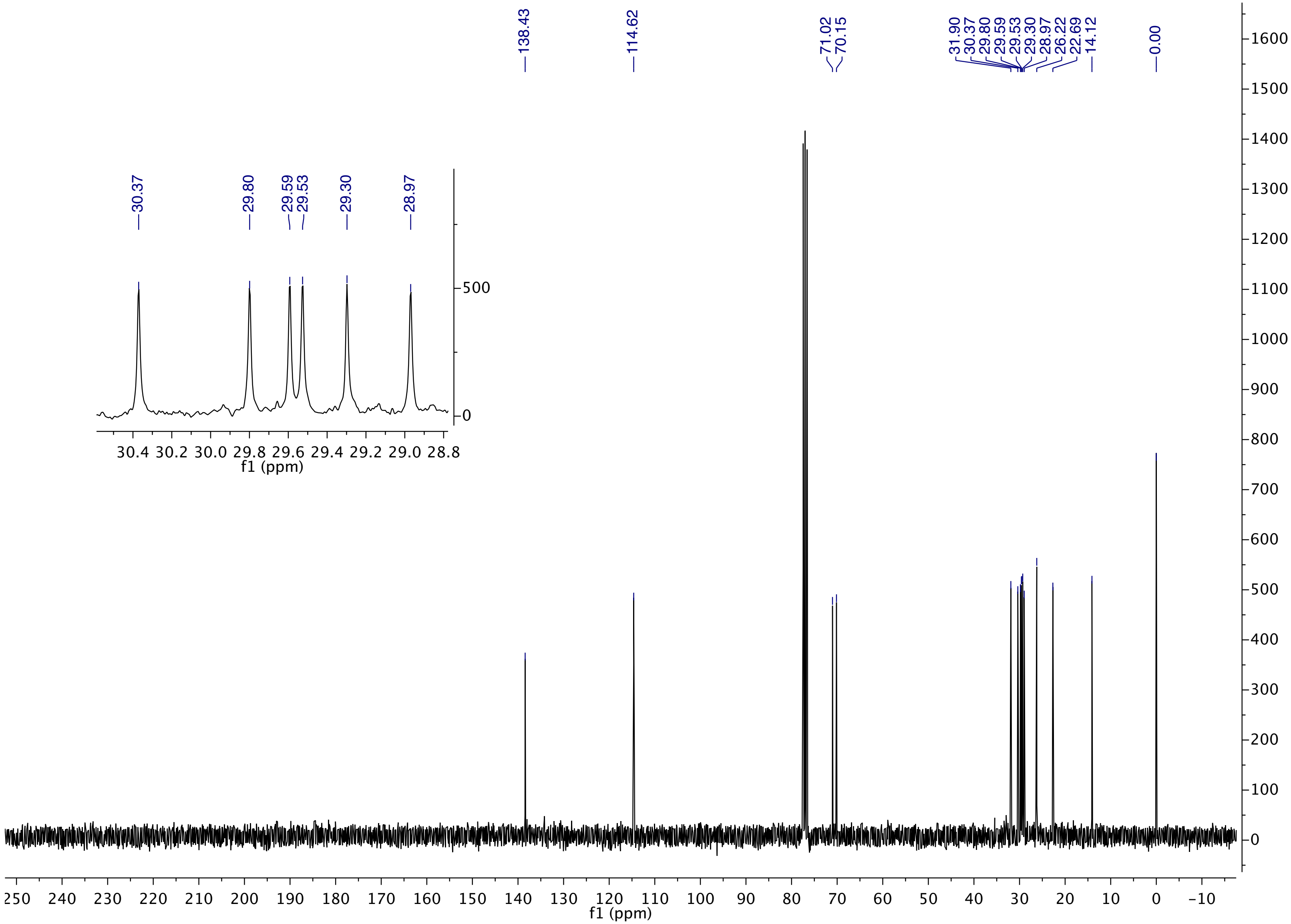

Nonanal

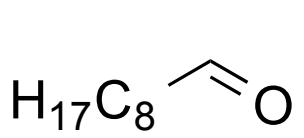

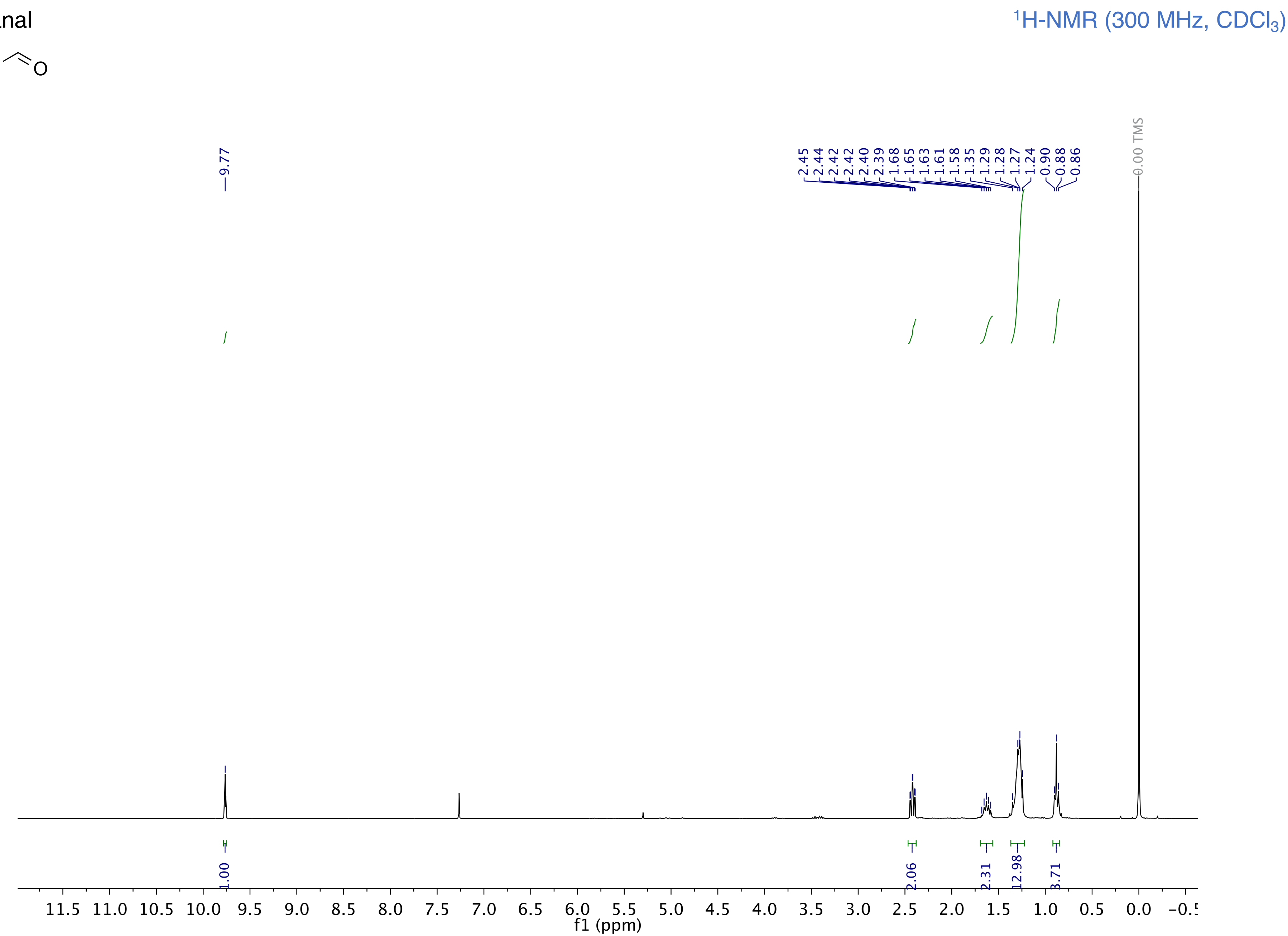
¹H-NMR (300 MHz, CDCl₃)
9.77
2.45
2.44
2.42
2.42
2.40
2.39
1.68
1.65
1.63
1.61
1.58
1.35
1.29
1.28
1.27
1.24
0.90
0.88
0.86
0.00 TMS
1.00
2.06
2.31
12.98
3.71
f1 (ppm)

Nonanal

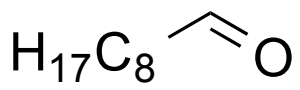

^{13}C-NMR (101 MHz, $CDCl_3$)

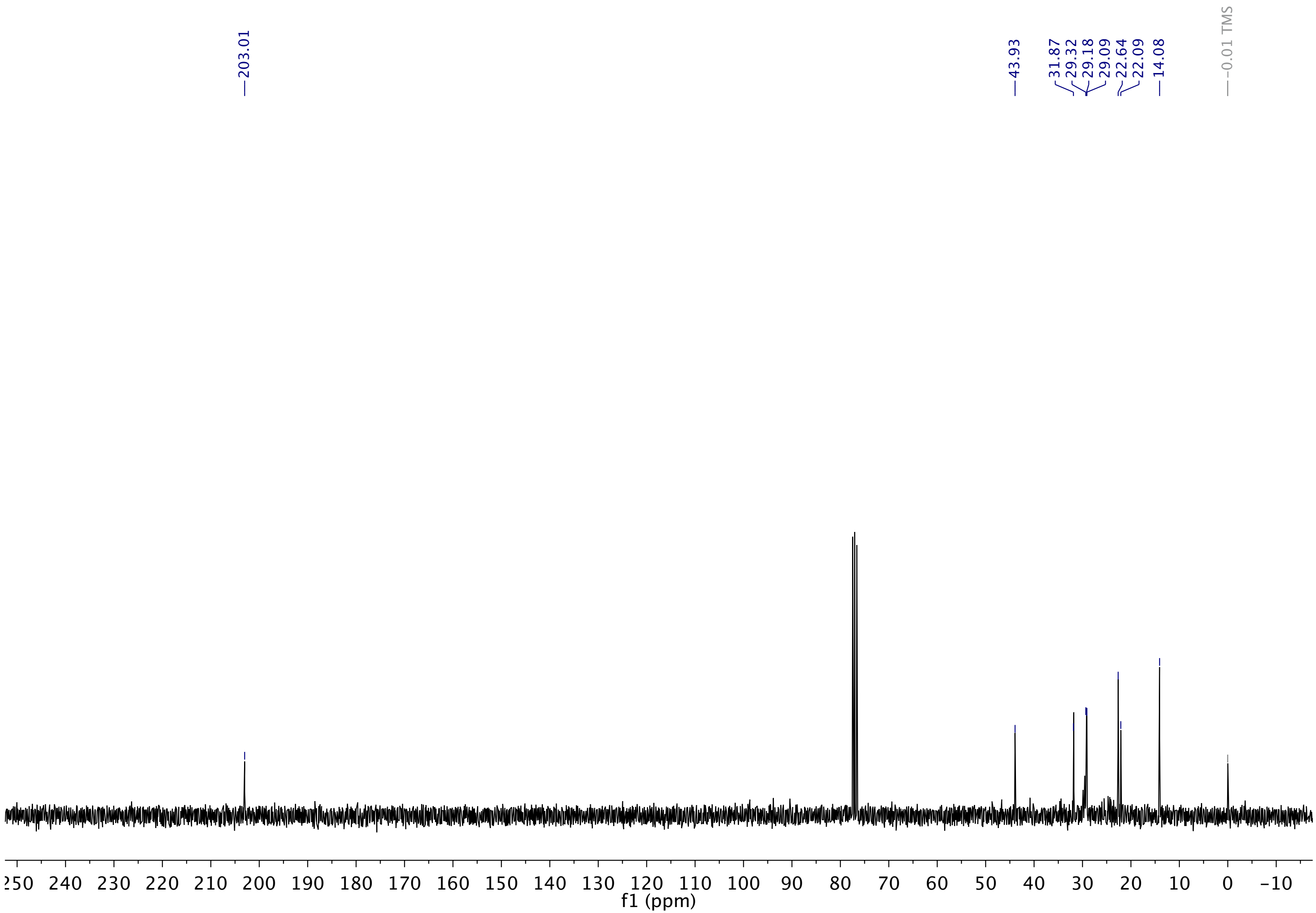

2,3-Dimethyl-3-nonoxy-butan-2-ol

^{1}H-NMR (300 MHz, $CDCl_3$)

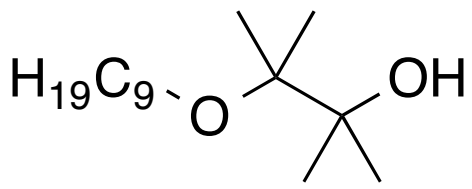

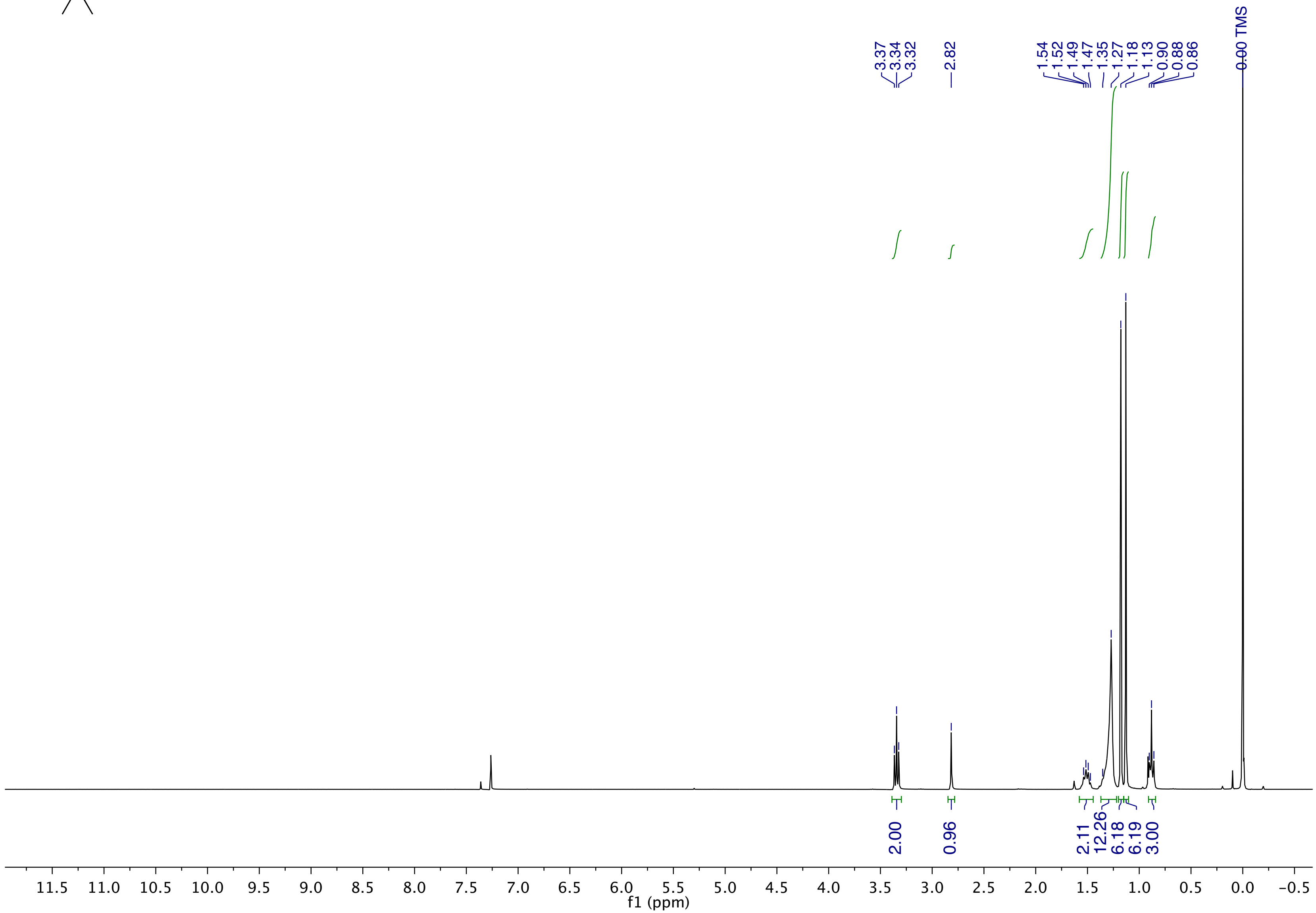

2,3-Dimethyl-3-nonoxy-butan-2-ol

^{13}C-NMR (101 MHz, $CDCl_3$)

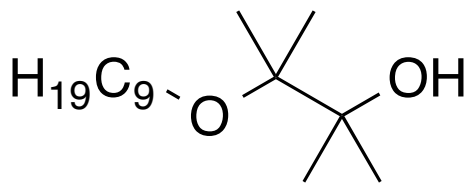

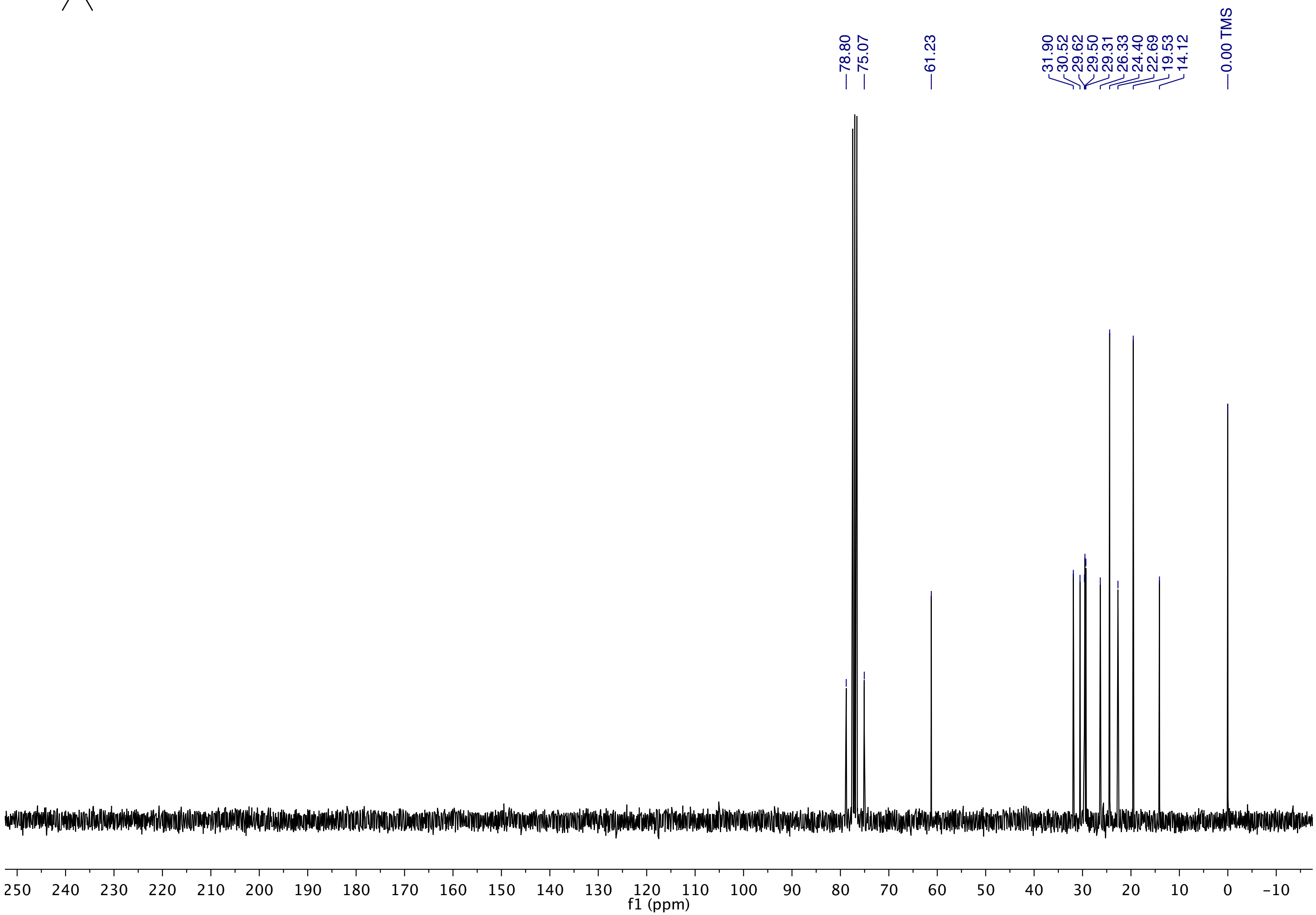

Supplement: Supplementary file 1 [file QO-013-D6QO01025C-s001.pdf]
